# Cysteine Thioaldehydes: Photolytic Generation, Reactivity, and Biological Implications

**DOI:** 10.1002/anie.202526017

**Published:** 2026-08-17

**Authors:** Ardra Karthika, Matthew Murray, Guillermo Palop, Lee‐Ann J. Keane, Patrick McArdle, Nicholas D. J. Yates, Christopher D. Spicer, Alison Parkin, Martin A. Fascione, Eddie L. Myers

**Affiliations:** ^1^ School of Biological and Chemical Sciences University of Galway Galway Ireland; ^2^ Department of Chemistry University of York York North Yorkshire UK

**Keywords:** enethiol, formylglycine, isothiazolone, oxazoles, photochemistry, thiazole

## Abstract

Cysteine thioaldehydes are putative intermediates in sulfatase‐maturing enzyme catalysis and in the biosynthesis of penicillin, coenzyme A and families of ribosomally synthesized and posttranslationally modified peptides (RiPPs). Norrish type‐II photolysis of phenacylsulfides gives cysteine thioaldehydes but can be inefficient in aqueous solution owing to competing β‐scission. We show that irradiation of phenacylsulfides bearing non‐conjugating electron‐withdrawing substituents leads to efficient Norrish type‐II fragmentation. In aqueous buffer, photolysis of cysteine phenacylsulfides leads to high conversion into cysteine enethiolate and isothiazolone, which can be interconverted with an oxidant (Ellman's reagent) or reductant (phosphine). Cysteine enethiolates are relatively stable in aqueous buffer at neutral‐to‐high pH but undergo cyclocondensation to cysteine thiazole at low pH. Cysteine thioaldehydes undergo appreciable levels of hydrolysis to give formylglycine in the absence of buffer and for longer peptides that adopt enabling secondary structure. Nucleophilic (n → π*) participation of the proximal backbone carbonyl group on cysteine thioaldehydes can lead to peptide oxazoles. These results suggest viable oxidation‐before‐cyclocondensation biosynthetic pathways for peptide azoles and that the hydrolysis of cysteine thioaldehydes into formylglycine, as proposed for sulfatase activation, is not trivial and relies on a peptide environment that promotes the desired hydration at the expense of other similarly efficient thiocarbonyl‐quenching processes.

## Graphical abstract

Photolysis of cysteine phenacylsulfides bearing non‐conjugating electron‐withdrawing substituents leads to high conversions into cysteine thioaldehydes through a Norrish type‐II pathway. This methodology enabled the study of the aqueous reactivity of these important biosynthetic intermediates, which, depending on peptide sequence, pH and buffer concentration, can transform into azoles, enethiols, isothiazolones, formylglycine, and 1,2,4‐trithiolanes.

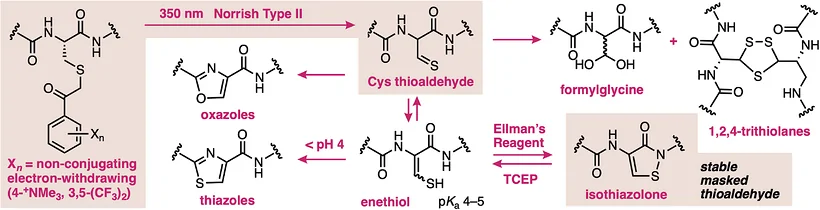

## 1 Introduction

Cysteine thioaldehydes are putative intermediates in the biosynthesis of the penicillin/cephalosporin family of antibiotics (Figure 1A) [1, 2, 3, 4], the biosynthesis of families of ribosomally synthesized and post‐translationally modified peptides (RiPPs), particularly those that contain aminovinyl cysteine bridges (Figure 1B) [5, 6, 7, 8, 9, 10, 11, 12, 13, 14, 15, 16], the biosynthetic transformation of pantothenate (vitamin B5) into co‐enzyme A (Figure 1C) [17, 18, 19, 20], and the activation of type‐I sulfatases (Figure 1D) [21, 22, 23, 24, 25, 26, 27, 28, 29, 30, 31, 32, 33, 34, 35]. In aerobic organisms, the latter process is mediated by the formylglycine‐generating enzyme (FGE), a Cu(I) oxidase that is believed to transform a cysteine thiol into a cysteine thioaldehyde, which subsequently undergoes hydrolysis into a formylglycine residue (fgly) [21, 22, 23, 24, 25, 26, 27, 28, 29]. The same transformation is apparent in anaerobic organisms but mediated by an S‐adenosyl‐L‐methionine (SAM)‐dependent reductase (e.g., AtsB, Figure 1D) [30, 31, 32, 33, 34, 35]. In sulfatase catalysis, the fgly residue, which exists as the hydrate (*gem*‐diol), acts as a nucleophile to accept sulfate from the sulfated biomolecule [36]. Mutations in human FGE, which is encoded by the SUMF1 gene, can lead to multiple sulfatase deficiency (MSD), an ultra‐rare but fatal disorder [37]. Recently, in vivo and in vitro enzymatic transformation of cysteine residues into fgly residues within engineered proteins is currently being used to introduce the aldehyde functional group for subsequent bioconjugation applications [38, 39, 40, 41, 42, 43, 44].

**FIGURE 1:**
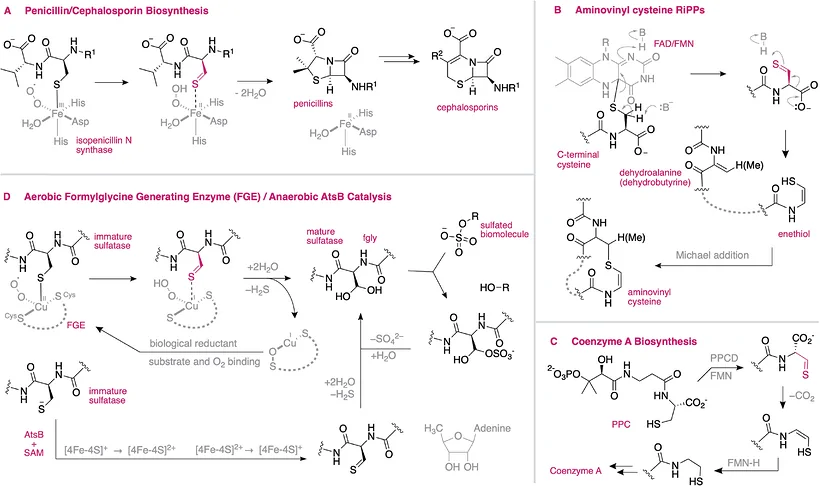
Cysteine thioaldehydes in biosynthesis. Abbreviations: Asp, aspartic acid; Cys, cysteine; FAD, flavin adenosine dinucleotide; fgly, formylglycine; FMN, flavin mononucleotide; His, histidine; PPC, 4′‐phospho‐N‐pantothenoylcysteine; PPCD, phosphopantothenoylcysteine decarboxylase; SAM, S‐adenosyl methionine.

Understanding of the nature, reactivity and synthetic potential of thioaldehydes is underdeveloped owing to their instability, which includes a high propensity to undergo polymerisation [45, 46, 47]. However, the generation of thioaldehydes through Norrish type‐II photolysis of phenacylsulfides has been highly enabling (Figure 2A) [48, 49, 50, 51, 52, 53, 54, 55, 56, 57, 58]. Evidence for the intermediacy of cysteine thioaldehydes in biosynthesis includes the characterization of shunt products derived from enzyme‐catalyzed transformation of non‐native substrates [59, 60, 61, 62, 63]. Although, Norrish type‐II photolysis of phenacylsulfides has been used to generate the thioaldehydes proposed in penicillin/cephalosporin biosynthesis, further transformation into the β‐lactam—thiazolidine bicycle was not observed (Figure 2B) [64]. In the late 1970s, Clark and Lowe showed that a phenacylsulfide derivative (2,4‐dimethoxy substitution) of papain, a cysteine protease, underwent photolysis to the cysteine thioaldehyde, the presence of which was inferred through the detection of labeled serine, derived from presumed spontaneous hydrolysis of the thioaldehyde to give formylglycine (fgly), which underwent reduction upon treatment with ^3^H‐labeled sodium borohydride (Figure 2C) [65, 66]. They showed that Norrish type‐II fragmentation competes with β‐scission, which regenerated the native cysteine residue and was the origin of the low efficiency of the method; however, multiple rounds of cysteine alkylation–photolysis did lead to high levels of conversion.

**FIGURE 2:**
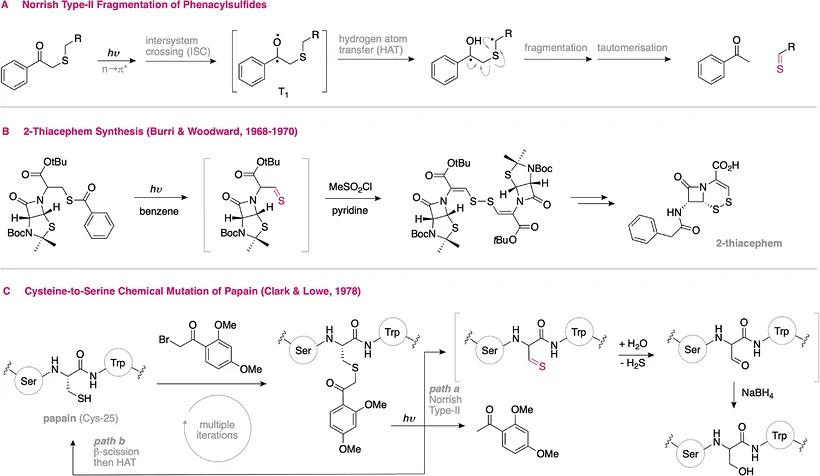
Norrish type‐II photolysis of phenacylsulfides. Abbreviations: Boc, *t*‐butoxycarbonyl; Cys, cysteine; Ser, serine; Trp, tryptophan.

Herein, we document our investigation into the reactivity of cysteine thioaldehydes, leading to key insights into their involvement in biosynthetic transformations. This investigation was enabled by success in optimizing the Norrish type‐II photolysis reaction of phenacylsulfide derivatives of cysteine in aqueous media.

## 2 Results and Discussion

### 2.1 Optimization of Norrish Type‐II Photolysis

Simple cysteine derivative **1** (Figure 3A) was S‐alkylated with phenacylbromides **2a**–**l** in either aqueous buffer (0.1 m phosphate‐buffered H_2_O, pH 9/DMF, 9:1) or in *N*,*N*‐diisopropylethylamine (DIPEA)/CH_2_Cl_2_. Purified phenacylsulfides **3a**–**l**, dissolved at 0.1 m in phosphate‐buffered D_2_O (0.1 m, pD 7.8)/CD_3_OD (1:1), were illuminated with 350 nm light until complete conversion was observed by ^1^H NMR spectroscopy (1–2 h, Figure 3A). 2,4‐Dimethoxy phenacylsulfide **3a**, analogous to Lowe's S‐alkylated derivative of papain (Figure 2C), was almost completely converted into corresponding cysteine disulfide **4** (95%, Figure 3B). Disulfide **4** presumably originates from homolytic fragmentation at the C_α_–S bond with subsequent dimerization of the thiyl radical [67]. The concomitantly formed enol radical either abstracts a hydrogen/deuterium atom from methanol, to give 2,4‐dimethoxyacetophenone (**7a**), which is observed as a by‐product, or undergoes dimerization to 1,4‐diketone derivative **8a–f**, which is observed in D_2_O/acetonitrile (Figure 3C).

**FIGURE 3:**
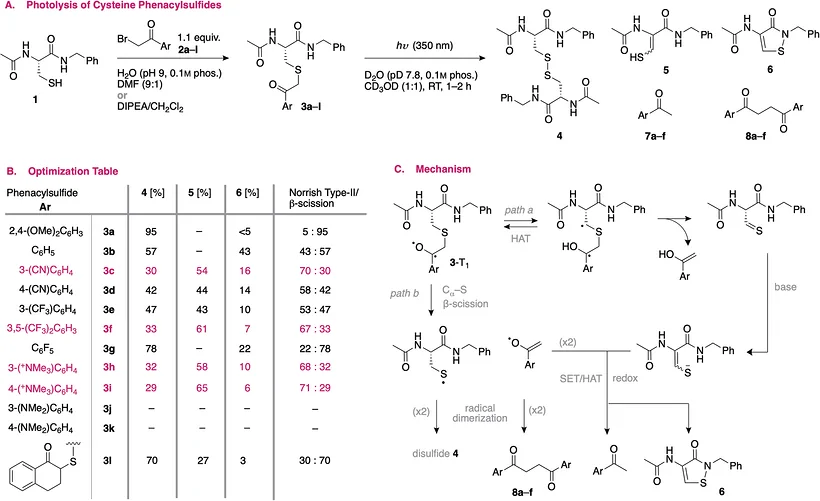
Optimization of Norrish type‐II photolysis of cysteine phenacylsulfides. The conversion values provided in the optimization table were obtained through analysis of the ^1^H NMR spectra of reaction mixtures. Substrates **3j** and **3k** were unreactive under the photochemical conditions.

Unsubstituted phenacylsulfide **3b** was partially converted into disulfide **4** (57%). The remainder was converted into cysteine isothiazolone **6** (43%), which was characterized by ^1^H/^13^C NMR spectroscopy and x‐ray crystallography [68]. Isothiazolone **6** presumably forms by enolization–oxidation of the thioaldehyde, which is generated through the desired Norrish type II photolysis (Figure 3C). Cysteine isothiazolones have been documented in the literature in the context of synthetic work on the penicillins [69, 70, 71]. Although not yet found in Nature, the corresponding reduced heterocycles (N‐sulfenyl amides) are biosynthetic intermediates in the recycling of cysteine sulfenic acids [72, 73, 74]. Synthetic isothiazolones have demonstrated biocidal activity and the ability to inhibit cysteine‐based proteases [75, 76, 77]. Biological activity originates from inhibitory covalent adducts formed through ring‐opening attack of the active‐site cysteine thiolate on the isothiazolone sulfur atom.

Wagner showed that suitably positioned non‐conjugating electron‐withdrawing substituents on the aryl ring stabilize the n‐to‐π* triplet state relative to the π‐to‐π* triplet state, the former having higher spin density on the carbonyl oxygen atom and thus more disposed to undergo the desired intramolecular hydrogen atom transfer [78]. In accordance with this precedent, illumination of a solution of phenacylsulfide **3c** (*m*‐cyano) led to much higher levels of Norrish Type‐II reactivity (∼70%), manifesting in the production of isothiazolone **6** (16%) and its precursor enethiolate **5** (54%; *E*/*Z* 1:7), which forms through C_α_–H deprotonation of the putative thioaldehyde. The now dominant enethiolate **5** at the expense of isothiazolone **6** is a consequence of the diminished β‐scission pathway and the associated lower levels of oxidizing enol radicals. We investigated other phenacylsulfides, but the β‐scission pathway could not be suppressed further. In summary, 3‐cyano (**3c**), 3,5‐bis(trifluoromethyl) (**3f**), 3‐(*N*,*N*,*N*‐trimethylammonium) (**3h**) and 4‐(*N*,*N*,*N*‐trimethylammonium) (**3i**) derivatives exhibited the highest levels (∼70%) of Norrish type‐II reactivity (Figure 3b), the latter derived from water‐soluble reagents and thus attractive for biological applications. Interestingly, electron‐rich *N*,*N*‐dimethylamine derivatives **3j** and **3k**, which were expected to react via the β‐scission pathway, were unreactive, perhaps owing to efficient excited‐state quenching by N → O charge‐transfer [79]. We also investigated tetralone derivative **3l**, anticipating that the more rigid structure would lead to higher proportions of conformations where the β‐carbon atom is near the carbonyl oxygen atom, thus engendering high levels of Norrish type II reactivity [80, 81]. However, the level of Norrish type II reactivity exhibited by phenacylsulfide **3l** was less than that of phenacylsulfide **3b** (43% vs. 30%, Figure 3b). Interestingly, there appeared to be significant levels of racemization as indicated by disulfide **4** being isolated as a ∼1:1 mixture of diastereomers, but with no H → D exchange at the C_α_ position. To probe this process further, photolysis was carried out in the presence of dithiothreitol (DTT) to quench tetralone radical, as generated through β‐scission and a potential mediator of racemization. However, upon subsequent treatment with 5,5´‐dithio‐bis‐(2‐nitrobenzoic acid) (DTNB, Ellman's reagent)**—**to oxidize thiol **1** to disulfide **4—**the latter was still observed as a mixture of diastereomers. We tentatively suggest that racemization is occurring through reversible C → O 1,6‐hydrogen atom transfer (1,6‐HAT) of excited state **3l**‐1,2‐T_1_ to give the 1,5‐diradical (**3l**‐1,5‐T_1_) at a rate faster than H/D exchange at the oxygen atom of the carbinol radical [82]. We considered other racemization processes involving the desired 1,4‐diradical (**3l**‐1,4‐T_1_), such as reversible β‐fragmentation to amidyl radical and enesulfide [83], amide/imidate rearrangement [84, 85, 86, 87, 88], 1,2‐acyl transfer, and 1,2‐HAT (exchanging the vicinal H_α_ and H_β_ atoms), but the associated rate‐determining reaction barriers, as estimated by density functional theory (DFT) calculation [89, 90, 91, 92, 93, 94, 95, 96, 97, 98, 99, 100], were too high (> 30 kcal/mol, see the Supporting Information). We subsequently confirmed low levels of racemization in the photolysis of phenacylsulfides **3g** and **3i**.

We investigated the effect of organic co‐solvent, which was used to ensure full solubility [101]. For phenacylsulfide **3b**, which exhibited 43% Norrish Type‐II reactivity in CD_3_OD/D_2_O (1:1) solvent, higher or similar levels were apparent in d_6_‐DMSO/D_2_O (3:2, 58%) and in CD_3_CN/D_2_O (1:1, 41%), respectively. Interestingly, for d_6_‐DMSO/D_2_O, the presence of a diastereomeric mixture of disulfide **4** (d.r., 1:1) with no C_α_‐deuteration suggested the operation of the parasitic 1,6‐HAT‐mediated racemization process described above.

### 2.2 Interconversion of Cysteine Enethiol and Isothiazolone

Treatment of isothiazolone **6**, which was stable at high and low pH values, with tris(2‐carboxyethyl)phosphine (TCEP), effected quantitative reduction to enethiolate **5** (Figure 4). Interestingly, enethiolate **5** was generated as an isomeric mixture (85:15) in favor of the minor isomer that was detected after phenacylsulfide photolysis. This result allows us to tentatively assign the species with the more upfield methine chemical shift as the (*E*)‐enethiolate, which would likely undergo more facile oxidation into the isothiazolone during photolysis, thus allowing the mixture to become enriched in (*Z*)‐enethiolate (see below). These data also suggest that enethiolates under these conditions are configurationally stable or undergo *E*/*Z* interconversion very slowly. That the *E*/*Z* ratio was not 100% upon TCEP‐based reduction of the isothiazolone might indicate that the intermediate S‐phosphonium species is more susceptible to isomerization, which occurs to some extent before hydrolysis of the P–S bond.

^1^H NMR spectroscopic monitoring of a buffered solution of enethiolate **5** at pD 10.0 over 3 days led to no change in the spectrum. However, when left exposed to air, slow oxidation to isothiazolone **6** was apparent. The use of DTNB effected rapid and selective oxidation of (*E*)‐enethiolate **5** into isothiazolone **6**; the minor isomer, (*Z*)‐enethiolate **5** undergoes conversion into the mixed (*Z*)‐enethiol/arylthiol‐derived disulfide **11** (Figure 4). The use of KI/I_2_, led to mixtures of isothiazolone **6** and isothiazolone S‐oxide **9**. Treatment of enethiolate **5** in phosphate‐buffered D_2_O (pD 7.8)/CD_3_OD (1:1) with MeI gave methyl–vinyl sulfide **10**.

**FIGURE 4:**
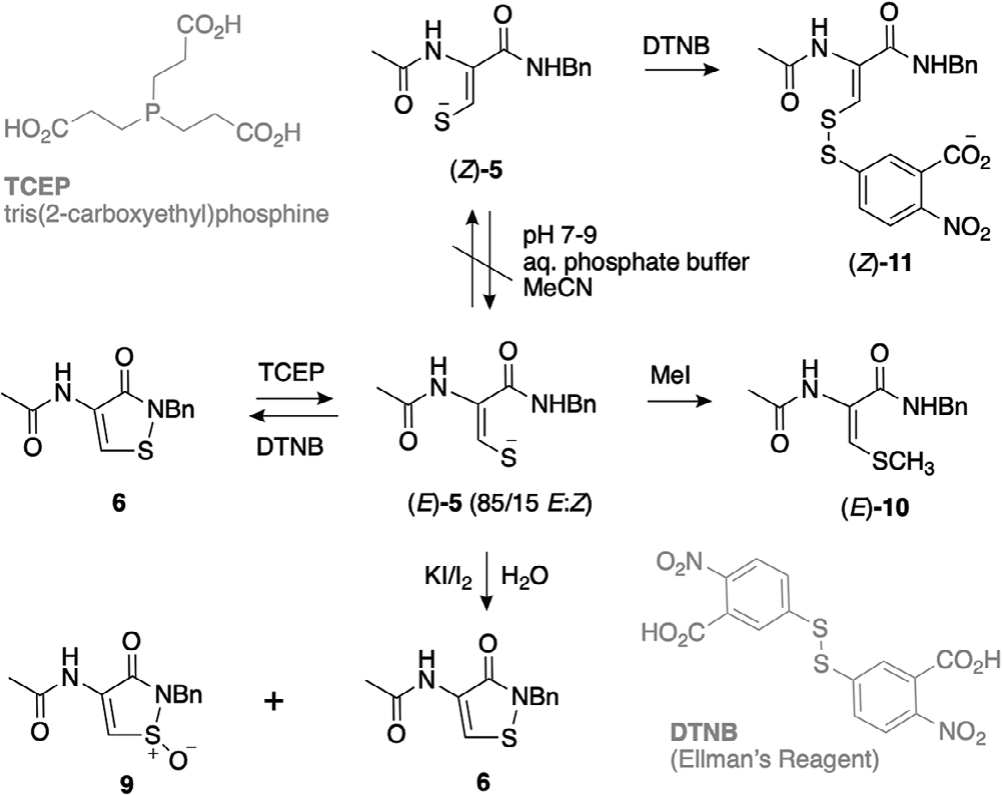
Hydrolytic and redox reactivity of cysteine isothiazolones and enethiolates. All reactions were carried out in phosphate‐buffered H_2_O/MeCN mixtures.

### 2.3 Photolysis in Non‐Buffered Aqueous Solvent

To slow thioaldehyde deprotonation/enolization, we conducted the photolysis reaction of phenacylsulfide **3f** in non‐buffered aqueous media (H_2_O/CD_3_CN), adjusted to pH 7.4 by using aq. NaOH; during photolysis, the pH dropped to 3.8. The NMR spectra of the reaction mixture were complex but included methine signals consistent with the hydrated formylglycine **14** (^1^H: 5.18 ppm, ^3^ *J* = 4.7 Hz; ^13^C: 90 ppm) [63, 102]. The photolysis reaction in D_2_O/CD_3_CN gave a mixture showing the same signal at 5.16 ppm but as a singlet, suggesting that formylglycine derivatives undergo rapid H → D exchange at C_α_. The assignment was confirmed through preparation of formylglycine **14** from known diethylacetal **12** (Figure 5A) [103]. The effort also revealed that formylglycine **14** undergoes slow deacetylation at low pH, perhaps via oxazoline **17**. Raising the pH to 7 initiated self‐condensation of ammonium **15** and then air oxidation to pyrazine **16**. In addition to formylglycine **14**, enethiolate **5**, isothiazolone **6** (trace amounts) and disulfide **4**, the photolysis mixture in non‐buffered media also contained a species assigned as cysteine 1,2,4‐trithiolane **19**, which was observed as mixture of three diastereomers. This species is likely generated from efficient trapping of the cysteine thioaldehyde by dihydrogen sulfide, which would be released through concomitant hydrolysis of the thioaldehyde to give formylglycine **14**, followed by addition of the resulting *gem*‐dithiol to a second equivalent of thioaldehyde and then ring‐closing oxidation. It is known that 1,2,4‐trithiolanes can be generated from reactions of the corresponding thiocarbonyl compounds [104], but, to the best of our knowledge, cysteine 1,2,4‐trithiolanes have not been described in the literature.

**FIGURE 5:**
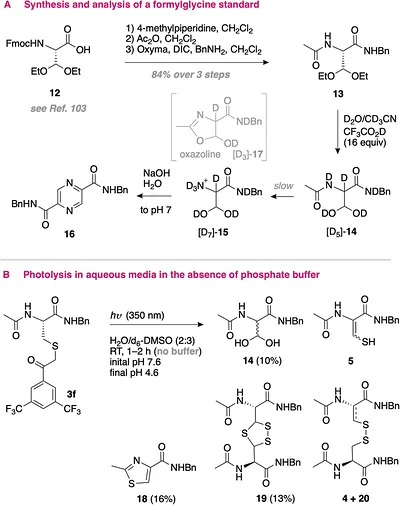
Synthesis of a formylglycine standard and photolysis reactions in the absence of phosphate buffer. Conversion values, where measurable, are shown in parentheses.

Photolysis of phenacylsulfide **3f** in non‐buffered H_2_O/d_6_‐DMSO solvent (2:3, initial pH: 7.6, final pH: 4.6) gave a spectrum more amenable for quantification (Figure 5B). In addition to the species observed in H_2_O/CD_3_CN solvent—conversion values measured immediately after photolysis: formylglycine **14** (10%); 1,2,4‐trithiolanes **19** (13%); isothiazolone **6** (trace), enethiol **5** and disulfides **4**/**20** (approximately 61%)—cysteine thiazole **18** (16%) was also detected and presumed to be generated by cyclocondensation of enethiol **5** under conditions of low pH (see below). Racemization of cysteine derivatives, as evident in the photolysis of phenacylsulfide **3b** in buffered H_2_O/d_6_‐DMSO (see Section 2.1), resulted in a more complex set of signals in the 5.30–5.00 region of the ^1^H NMR spectrum, owing to access to the full complement of 1,2,4‐trithiolane diastereomers. Interestingly, the composition of this mixture evolved over several days: analysis of the mixture 11 days post‐photolysis showed a marked increase in levels of thiazole, formylglycine and 1,2,4‐trithiolanes species, with a concomitant decrease in the levels of enethiol species—conversion values measured 11 days after photolysis: formylglycine **14** (15%); thiazole **18** (29%); 1,2,4‐trithiolanes **19** (29%); isothiazolone **6** (< 1%); enethiol **5** and disulfide derivatives **4**/**20** (approximately 26%).

The above studies (and other studies, see the Supporting Information) suggest that at low‐to‐neutral pH values, cysteine enethiol(ate) is in equilibrium with the putative thioaldehyde, thus allowing repartitioning into formylglycine, dithioacetal species, which can ultimately lead to 1,2,4‐trithiolanes, and enethiol(ate), the latter also susceptible to conversion into cysteine isothiazolones under oxidizing conditions and cyclocondensation into cysteine thiazoles under acidic conditions. These conclusions were further strengthened by monitoring the fate of enethiol(ate) **5**—generated through TCEP‐mediated reduction of purified isothiazolone **6** in aqueous solution—which evolved over several days into the same selection of products (Figure 6). Interestingly, in the presence of excess TCEP, cysteine thioaldehyde is trapped as the stable α‐thiophosphonium species (**5•TCEP**) as it emerges through C‐protonation/enolization of the enethiol(ate) [105]. In D_2_O/MeCN (1:1; [**5**] = 10 mM), TCEP trapping of cysteine thioaldehyde was so rapid that it was independent of the concentration of TCEP (10–30 mM), thus allowing a first‐order rate constant to be measured for the conversion of the in situ formed *E*‐enethiolate into putative cysteine thioaldehyde (∼2.5 × 10^−5^ s^−1^). The highly efficient TCEP trapping was confirmed through DFT calculation [89, 90, 91, 92, 93, 94, 95, 96, 97, 98, 99, 100], which revealed a very low barrier (∼2.5 kcal/mol calculated for PMe_3_ trapping). In H_2_O/MeCN (1:1), and with similar concentrations of TCEP, the rate of formation of the α‐thiophosphonium **5•TCEP** was faster (> 5 times at 30 mM TCEP), but the rate of trapping was dependent on the concentration of TCEP (10–30 mM), reflecting isotope effects associated with both C_α_‐protonation/deuteronation of enethiolate and the partitioning of cysteine thioaldehyde forwards and irreversibly to α‐thiophosphonium **5•TCEP** or backwards to enethiolate **5**; the latter process involves the deprotonation/dedeuteronation of C_α_–H/C_α_–D moiety, with dedeuteronation of C_α_–D being sufficiently slow to allow complete partitioning toward TCEP trapping in D_2_O‐based solvent. Notably, these mixtures appear to be highly sensitive to low levels of O_2_, as indicated by the generation of isothiazolone **6** and 1,2,4‐trithiolanes **19**. In the absence of excess TCEP, that is, when only enough TCEP was added to reduce isothiazolone **6** into enethiolate **5**, the rate of formation of formylglycine in H_2_O/D_2_O/MeCN (0.25:0.25:0.50) was measured (∼2.7 × 10^−6^ mM s^−1^). Under these conditions, the kinetic partitioning of cysteine thioaldehyde is ∼200:1 (as a lower limit) in favor of returning to enethiolate. Notably, the triacetate moiety of TCEP is acting as a general base in the deprotonation/dedeuteronation of the C_α_–H/C_α_–D moiety of cysteine thioaldehyde (DFT‐calculated reaction barrier: 5–8 kcal/mol); in our experiments, citric acid (a tricarboxylic acid, like TCEP) was used as an additive to maintain acetate concentration across all kinetics experiments. The steps involved in cysteine thioaldehyde hydrolysis were investigated by DFT calculation [89, 90, 91, 92, 93, 94, 95, 96, 97, 98, 99, 100], which revealed a barrier of ∼13–15 kcal/mol for the addition of water to the thiocarbonyl group, leading to the zwitterionic hemi‐thioacetal; participation of either the N‐terminal or C‐terminal backbone carbonyl group, through directing and activating the water molecule for addition emerged from the calculations. The mechanism of subsequent expulsion of the sulfur moiety to generate formylglycine was most likely via the conjugate base of the hemi‐thioacetal (DFT‐calculated p*K*_a_ value of cysteine thioacetal: ∼6.8), involving initial water‐mediated S → O proton transfer (reaction barrier: 13.6 kcal/mol) followed by rapid elimination of hydrogen thiolate (reaction barrier: ∼1.7 kcal/mol; see the Supporting Information). These results show that cysteine isothiazolones can be viewed as stable masked cysteine thioaldehydes, which can be unmasked under reducing conditions.

**FIGURE 6:**
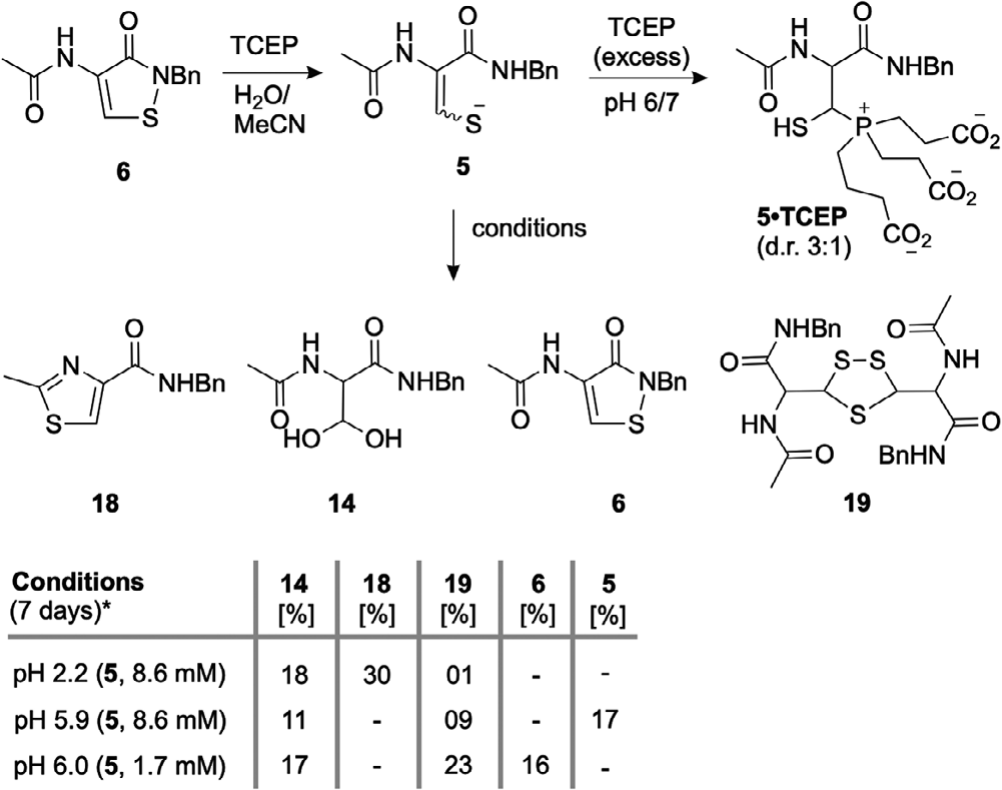
Isothiazolones as stable masked derivatives of cysteine thioaldehydes. Table shows conversion [%] into products, determined by integration of ^1^H NMR spectra. *Precipitate comprising 1,2,4‐trithiolane **19** as the major species was sometimes observed.

The above knowledge facilitates the design of conditions for maximizing conversion of cysteine thioaldehydes into the product of interest. For example, we found that TCEP‐mediated reduction of purified isothiazolone in buffered aqueous solution (MeCN/H_2_O, 1:1) with subsequent lowering of the pH value to ∼3.3 allowed cysteine thiazole to be isolated in moderate yield on a preparative scale (42% yield). Backbone thiazoles are common motifs both in non‐ribosomal peptides (NRP) and RiPPs, which are often highly bioactive [106, 107, 108]. Investigations into the biosynthetic origins of such thiazolyl peptide (or thiopeptide) motifs almost invariably produced firm evidence in support of a mechanism involving initial ATP‐driven cyclocondensation of the cysteine residue to give the intermediate thiazoline, which can be detected and isolated, followed by flavin mononucleotide (FMN)‐dependent oxidation to the thiazole [109, 110, 111]. Our results show that the alternative synthetic pathway—oxidation followed by cyclocondensation—is feasible and that future confirmation of such a biosynthetic mechanism is probable. Indeed, it has recently been disclosed that analysis of the biosynthetic machinery linked with a novel family of microbially‐produced peptide thiazoles, which were isolated from coral in the Pacific, revealed that the thiazole moiety was not produced through the standard mechanism, but instead through the action of a glucose–methanol–choline (GMC) oxidase on a cysteine residue [112]; GMC oxidases have been shown to be capable of effecting the transformation of a thiol into a thioaldehyde [113]. Finally, our results demonstrate an alternative synthetic route to such motifs, for which there are already several good strategies [114, 115, 116, 117, 118, 119, 120, 121, 122, 123, 124].

### 2.4 Investigations Into Tripeptide Derivatives

Having established a general understanding of the reactivity of simple cysteine thioaldehydes, we investigate these species as part of longer peptides to determine the effect of flanking residues and secondary structure (Figure 7). We began with tripeptide substrate AcNH‐Ala‐Cys‐Gly‐CONH_2_ (**21**). As anticipated, the 2,4‐dimethoxy phenacylsulfide derivative (**22a**) underwent predominant β‐scission, giving disulfide **23** (96% conversion). 3,5‐Bis(trifluoromethyl) derivative **22b** transformed through both β‐scission (38%) and Norrish‐Type II (62%) processes, the latter manifesting in the detection of isothiazolone (**24**; 16%) and enethiol (**25**; 46%, 85:15 dr) derivatives. 4‐Trimethylammonium derivative **22c** underwent more β‐scission (62%) at the expense of Norrish Type II fragmentation (32%). Interestingly, as well as the disulfide (**23**; 56%), isothiazolone (**24**; 19%) and enethiol (**25**; 13%) species, mixed thiol/enethiol disulfide **26** was also detected (12%). That 3,5‐bis(trifluoromethyl) derivative **22b** gives almost 24% more conversion through the Norrish Type II process than 4‐trimethylammonium derivative **22c—**chromophores that engendered similar levels of Norrish Type II reactivity for simple cysteine derivatives—highlights the importance of conformer distribution, which is difficult to predict and control.

**FIGURE 7:**
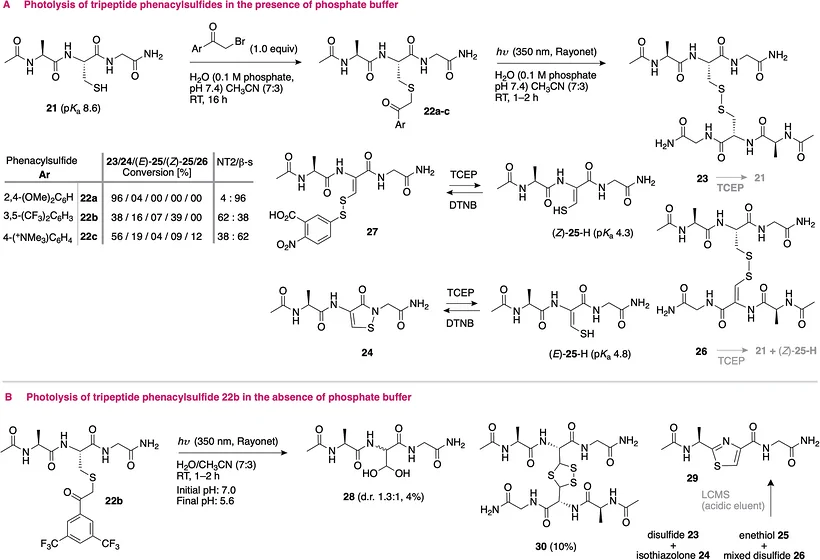
Synthesis and photolysis reactions of phenacylsulfide derivatives of cysteine‐containing tripeptide **21**. Abbreviations: β‐s, β‐scission; DTNB (Ellman's reagent), 5,5‐dithiobis‐(2‐nitrobenzoic acid); NT2, Norrish Type II; TCEP, tri(carboxyethyl)phosphine.

Treatment of aliquots of the photolysis mixture derived from phenacylsulfide **22b** with TCEP and DTNB facilitated assignment of the (*E*)‐ and (*Z*)‐enethiolate species (**25**) with high confidence and was fully consistent with the results obtained with the simple cysteine derivatives. These assignments, and the high water solubility of these derivatives, facilitated the measurement of the p*K*_a_ values of the respective enethiols (**25**.H) by using ^1^H NMR titration experiments. The p*K*_a_ values of (*E*)‐ and (*Z*)‐enethiols **25**.H were measured as 4.8 and 4.3, respectively; the p*K*_a_ value of parent cysteine thiol **21** was measured as 8.6.

Finally, photolysis of 3,5‐bis(trifluoromethyl) phenacylsulfide **22b** in aqueous media but in the absence of phosphate buffer species (pH adjusted to pH 7.0) gave formylglycine **28** (d.r. 1.3:1) and 1,2,4‐trithiolanes **30**. Furthermore, enethiols **25** transformed into thiazole **29** during LC‐MS analysis owing to the acidic nature of the eluent.

### 2.5 Investigations on FGE Consensus‐Sequence Peptides of Sulfatases

Our investigation turned to understanding the apparent high efficiency by which putative cysteine thioaldehydes within sulfatases undergo hydrolysis as part of FGE/AtsB catalysis [21, 22, 23, 24, 25, 26, 27, 28, 29, 30, 31, 32, 33, 34, 35]. Therefore, we investigated the photolytic generation of cysteine thioaldehydes from phenacylsulfide derivatives **32** of ALCTPSRGSLFTGRG (**31**), which aligns with the relevant region of the AtsG arylsulfatase from *M. tuberculosis* and contains the CXPXR consensus sequence found in Type 1 sulfatases (Figures 8 and 9) [125]. HPLC analysis (with collection of fractions for subsequent high‐resolution ESI MS analysis)—carried out within 30 min after photolysis—showed the presence of disulfide **32_DS**, isothiazolone **32_IT**, mixed thiol–enethiol disulfide **32_teDS**, thiazole **32_TZ**, oxazole **32_OZ**, and formylglycine **32_gdFG**/**32_FG** (table entry 1, Figure 8). Under the conditions of LC analysis (acidic H_2_O/CH_3_CN), formylglycine *gem*‐diol **32_gdFG**, which exists as a pair of diastereomers, undergoes partial dehydration to formyl‐glycine species **32_FG**. Indeed, NMR analysis of mono‐ and tripeptide derivatives in aqueous CH_3_CN or DMSO, as described above, shows small amounts of the unhydrated formylglycine species coexisting with the *gem*‐diol derivative, as indicated by a singlet in the 9–10 ppm region of the ^1^H NMR spectrum. Two fragment peptides derived from an apparent oxidative cleavage reaction at the cysteine position were also detected: the Ala–Leu dipeptide as the C‐terminal primary amide derivative (**32_PA**) and the TPSRGSLFTGRG dodecapeptide as the N‐terminal oxamate derivative (**32_OX**). Additional HRMS(ESI) analysis (direct injection of crude mixture; no LC; neutral conditions) confirmed that thiazole **32_TZ** and oxazole **32_OZ** are derived from enethiol **32_ET** and cyclic O,S acetal **32_TO**, which undergo cyclocondensation and elimination of H_2_S, respectively, under the acidic conditions of HPLC analysis. Furthermore, treatment of the mixture with NaIO_4_ led to quantitative oxidative fragmentation of formylglycine species (**32_gdFG/FG**) into dipeptide (**32_PA**) and dodecapeptide oxamate (**32_OX**) fragments, suggesting that the origin of these fragments, as detected in the original photolysis mixture, is partial oxidative fragmentation of formylglycine **32_gdFG/FG** by oxidizing enol radicals, which are produced by β‐scission of phenacylsulfide **32**. This oxidative cleavage reaction is reminiscent of the known RuO_4_‐ or CrO_3_‐mediated transformation of serine and threonine‐containing peptides [126, 127]. In accordance with the method previously described by Yates and Fascione et al. [128], we generated an authentic sample of formylglycine **32_FG** through solid‐phase peptide synthesis of the pentadecapeptide incorporating a 4‐hydroxythreonine residue in place of cysteine; NaIO_4_‐mediated oxidative cleavage of the side‐chain vicinal‐diol of the purified peptide gave the formylglycine species (**32_gdFG**/**FG**), which matched the material generated through photolysis in all respects. Furthermore, the NaIO_4_ treatment led to some oxidative fragmentation of the peptide to give dipeptide **32_PA** and dodecapeptide oxamate **32_OX**.

**FIGURE 8:**
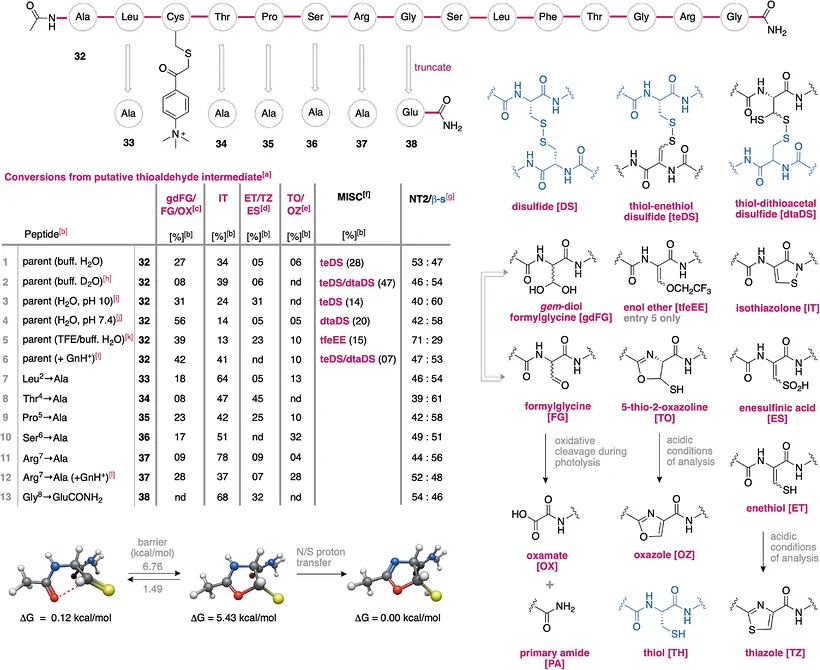
Photolysis reactions of phenacylsulfide derivatives of pentadecapeptides corresponding to the relevant region of the AtsG aryl‐sulfatase from M. tuberculosis. ^a^General conditions unless otherwise indicated: 350 nm, aq. buffer (H_2_O, 0.1 M phosphate, pH 7.4), RT, 2 h. ^b^Relative conversions from putative thioaldehyde, the levels of which are estimated based on summing overall conversions (from phenacylsulfide) of all products obtained through a Norrish Type II process only. ^c^Combined conversions into *gem*‐diol formylglycine (gdFG) and formylglycine (FG), the latter prevalent in the presence of acidic MeCN/H_2_O eluent of HPLC analysis; includes oxamate derivative (OX) formed through oxidative cleavage of FG/gdFG under the conditions of photolysis. ^d^Conversion into thiazole (TZ), which is formed during HPLC analysis by acid‐catalysed cyclocondensation of enethiol (ET); enesulfinic acid (ES) is sometimes detected. ^e^Conversion into oxazole (OZ), which is formed during HPLC analysis by acid‐catalysed elimination of H_2_S from 5‐thio‐2‐oxazoline (TO). ^f^Miscellaneous species, including mixed disulfides involving the parent thiol and either enethiol (etDS) or dithioacetal (daDS); in the case of TFE solvent mixtures, the TFE enol ether (tfeEE) is detected. ^g^Distribution of products according to photolytic pathway (Norrish type II versus β‐scission). ^h^350 nm, aq. buffer (D_2_O, 0.1 M phosphate, pD 7.4), RT, 2 h. ^i^350 nm, H_2_O (pH 10→9.3), RT, 2 h. ^j^350 nm, H_2_O (pH 7.4 → 6.4), RT, 2 h. ^k^350 nm, aq. buffer (H_2_O, 0.1 M phosphate, pD 7.4)/trifluoroethanol (1:1), RT, 2 h. ^l^350 nm, aq. buffer (H_2_O, 0.1 M phosphate, 3.0 M guanidinium chloride, pD 7.4) RT, 2 h.

**FIGURE 9:**
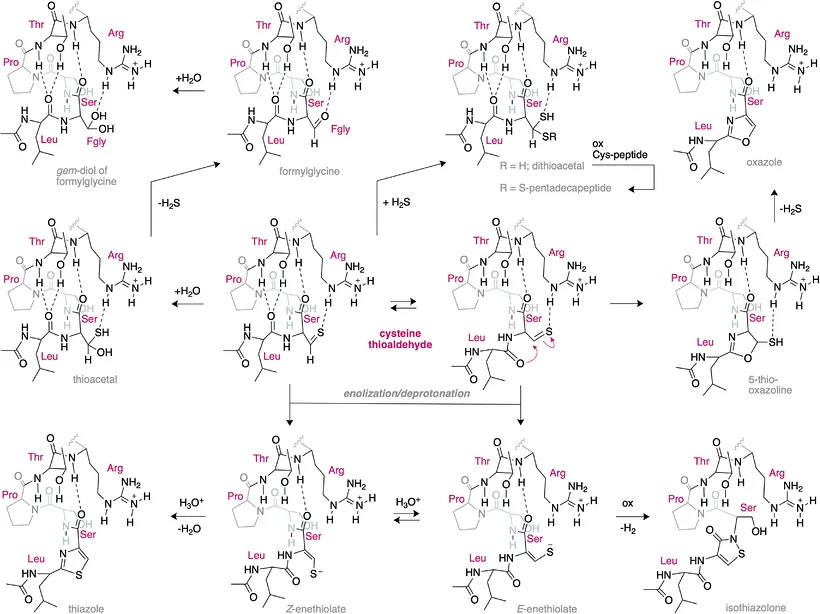
Reactivity of cysteine thioaldehydes within peptides corresponding to the FGE consensus sequence of sulfatases. Note, the peptides are depicted with aspects of α‐helical conformation, as observed in x‐ray crystal structures of sulfatases; however, alternative secondary structure (e.g., β‐sheet and PPII) may be preferable for some transformations.

The relatively high conversion into formylglycine **32_gdFG**/**FG** in aqueous buffer—conditions that engendered rapid enolization of the putative cysteine thioaldehyde of simple mono and tripeptides—suggests that either residues proximal to cysteine, secondary structures adopted by such sequences, or both aspects engender efficient hydrolysis of the putative cysteine thioaldehyde. The detection of oxazole **32_OZ** (detected by LC‐HRMS/ESI analysis), which is derived from 5‐thio‐2‐oxazoline (**32_TO**; detected through direct‐injection‐mode HRMS‐ESI analysis) shows that the thioaldehyde moiety is partially trapped by irreversible nucleophilic participation of the proximal backbone carbonyl group. DFT calculations [89, 90, 91, 92, 93, 94, 95, 96, 97, 98, 99, 100], including Natural Bond Orbital (NBO) analysis [129], shows that some minor conformers of a simple cysteine thioaldehyde model exhibit a relatively strong donor–acceptor n → π* interaction (*E*_d‐a_ = 1.4–6.5 kcal/mol) involving the N‐terminal backbone carbonyl oxygen atom as the donor and the thiocarbonyl group as the acceptor [130]. The barrier for the formation of either the *trans*‐configured or *cis*‐configured zwitterionic oxazolidine from these conformers was calculated as being very low, suggesting rapid cyclisation (5.9–6.8 kcal/mol, see Figure 8, lower left, and the Supporting Information).

Conducting the photolysis reaction of phenacylsulfide **32** in buffered D_2_O revealed differential solvent isotope effects (cf. entries 1 and 2, Figure 8) that engendered increased partitioning into products derived from deprotonation/tautomerization. NMR analysis with integration of assignable peaks gave product distributions that were in‐line with those calculated from HPLC spectra, thus validating the latter for quantitative analysis. When the photolysis reaction of phenacylsulfide **32** was carried out in non‐buffered H_2_O (pH 7.4; pH measured as 6.4 after photolysis; entry 4, Figure 8) a significant increase in the relative amount of thioaldehyde hydrolysis: approximately 56% based on HPLC quantification of the formylglycine species (**32_gdFG**/**FG**) and oxamate dodecapeptide fragment (**32_OX**). Conducting the reaction under the same conditions, but in D_2_O, facilitated ^1^H NMR analysis (see Supporting Information), which showed the acetal methine signals of the diastereomeric *gem*‐diol derivatives of formylglycine (**32_gdFG**) and the thiol/dithioacetal disulfide (**32_dtaDS**).

Owing to the less‐well‐defined conformation of pentadecapeptide **31** compared to that of similar sequences as domains within sulfatases, where they adopt well‐defined α‐helical secondary structures (for example, see PDB files: 7STT [131], 6J66 [132], 4CXU [133], 1HDH [134]) the photolysis was carried out in H_2_O (0.1 M phosphate, pH 7.4)/trifluoroethanol (TFE) 1:1 (table entry 5, Figure 8); the use of TFE solvent is known to stabilize the α‐helical conformation of peptides [135, 136, 137], and this effect was confirmed for pentadecapeptide **31** through circular dichroism (CD) studies (see Supporting Information). Although photolysis in this solvent mixture promoted oxidative processes, there was a significant increase in the apparent conversion of the putative cysteine thioaldehyde into formylglycine **32_gdFG**/**FG** (cf. table entries 1 and 5, Figure 8; conversions of 27% and 39%, respectively).

### 2.6 Alanine‐Scan Investigation of FGE Consensus‐Sequence Peptides of Sulfatases

The effect of the flanking amino‐acid residues was determined through an alanine scan: the N‐terminal leucine and C‐terminal threonine, proline, serine and arginine residues were sequentially replaced with an alanine residue and aqueous buffered solutions of the resulting phenacylsulfides **33**–**37** were photolyzed and analyzed. Product species like those detected for parent phenacylsulfide **32** were also detected, but the relative levels of these species were dependent on the mutation position (Figure 8, table entries 7–12).

Mutation of the threonine residue and the arginine residue had the most deleterious effect on thioaldehyde hydrolysis with conversion into the relevant products being reduced to a third of the value observed for the parent phenacylsulfide (cf. table entries 1, 7 and 11, Figure 8). Because the relative positions of the cysteine and arginine side‐chains would place them in close proximity on the same face of an α‐helix, as observed in x‐ray crystallographic structures of sulfatases (Figure 9) [131, 132, 133, 134], we surmised that thioaldehyde–guanidinium hydrogen‐bonding interactions might be promoting thioaldehyde hydrolysis. To probe this hypothesis, the photolysis reaction of the arginine → alanine mutant (**37**) was also carried out in aqueous phosphate buffer (0.1 M, pH 7.4) with 3 M guanidinium chloride (table entry 12, Figure 8), with the expectation that the guanidinium ion might act as an intermolecular surrogate of the pendant guanidinium moiety of the arginine residue in the parent pentadecapeptide. Under these conditions, and in support of the hypothesis, products derived from thioaldehyde hydrolysis had recovered to levels measured for the parent peptide (cf. table entries 1, 11 and 12, Figure 8; 26%, 9% and 28% conversion from the thioaldehyde, respectively). Interestingly, photolysis of parent phenacylsulfide **32**, but in the presence of 3 M guanidinium chloride, gave even higher levels of thioaldehyde hydrolysis (cf. table entries 1 and 6, Figure 8; 26% and 43% conversion from the thioaldehyde, respectively). Significantly higher levels of oxazole, and thus n → π* participation of the backbone carbonyl group, were detected for the serine → alanine mutant (**36**, table entry 10, Figure 8) and, in the presence of guanidinium chloride, the arginine → alanine mutant (**37**, table entry 12, Figure 8). The presence of high concentrations of guanidinium disrupts peptide/protein tertiary structure [138]. The effect on secondary structure is more difficult to predict with conflicting effects on α‐helical and β‐sheet structure being reported [139, 140]. We attempted circular dichroism and IR analysis of the parent pentadecapeptide thiol **31** in the absence and presence of 3 M guanidinium chloride; however, the presence of the salt obscured the detection of the circular dichroism signal and deconvolution analysis of the amide‐I IR band (1600–1700 cm^−1^) could not distinguish random coil from α‐helical character (see the Supporting Information). However, the IR analysis confirmed that guanidinium chloride was modulating secondary structure, the data suggesting a doubling of the β‐sheet character (17% → 41%). Molecular dynamics simulations of the parent sequence containing the cysteine thioaldehyde and that of the corresponding arginine → alanine mutant provided some contrasting insight: [141, 142, 143, 144, 145, 146] (1) Both peptides were intrinsically disordered, but many residues, specifically the cysteine thioaldehyde residue, exhibited a strong preference for secondary structure located on top left quadrant of the Ramachandran plot, namely, the fully extended structure (ϕ ∼ −160**°**, ψ ∼ +160**°**; ∼12%), the polyproline type‐II (PPII) structure (ϕ ∼ −75**°**, ψ ∼ +150**°**; ∼11%), and β‐like structure (ϕ ∼ −120**°**, ψ ∼ +130**°**; ∼5%). (2) α‐Helical structure was not prevalent in simulations (ϕ ∼ −57**°**, ψ ∼ −47**°**; < 2%), but the pre‐helix region exhibited relatively significant populations (ϕ ∼ −75**°**, ψ ∼ −20**°**; 9%). (3) Neither of the two arginine residues sampled low‐energy stable contacts with the thiocarbonyl group. (4) Both peptides moderately populated conformations that placed either the N‐terminal (leucine) or C‐terminal (cysteine thioaldehyde) backbone carbonyl oxygen atom in close proximity with the thiocarbonyl group that would engender either an n → π* star interaction [130], thus priming the system for the generation of oxazole, or to assist in the delivery of a water molecule for the generation of formylglycine. Additional DFT studies on a cysteine thioaldehyde model showed that free guanidinium, through interaction with the thiocarbonyl sulfur atom, can lower the barrier of hydration of the thiocarbonyl group by ca. 2 kcal/mol (see the Supporting Information).

Finally, photolysis of octapeptide **38** (table entry 13, Figure 8), corresponding to truncation of parent pentadecapeptide **32** after the glycine residue, only gave products derived from deprotonation of the putative thioaldehyde (isothiazolone **38_IT** and thiazole **38_TZ**) and β‐scission (disulfide **38_DS**).

The above analysis shows that the fate of a cysteine thioaldehyde moiety, which involve relatively low barriers for C_α_‐deprotonation, hydration toward formylglycine, and cyclisation toward oxazolidine, is highly dependent on secondary structure and that the conformation in which cysteine thioaldehyde is generated is likely decisive. Interestingly, fully extended, PPII and β‐sheet type environments, in accordance with molecular dynamics simulations, being prevalent for derivatives of pentadecapeptide **31**, and, in accordance with IR analysis, increasing in prevalence in the presence of guanidinium chloride, exhibit ψ dihedrals that place the C_α_–H bond in close to antiparallel alignment with the adjacent carbonyl group (H–C_α_–C═O dihedral > 140°), thus suppressing enolization/deprotonation. Furthermore, while PPII structure avoids placing the N‐terminal carbonyl oxygen in proximity with the thiocarbonyl carbon atom, thus suppressing cyclisation toward oxazolidine, it does place the C‐terminal carbonyl oxygen atom in proximity to direct a water molecule for hydration of the thiocarbonyl group. Moreover, β‐sheet‐type structures exhibit ϕ dihedrals (−135°) that better facilitate cyclisation toward oxazolidine (or assisted hydration), consistent with the observed increase in conversion into oxazole for photolysis mixtures containing 3 M guanidinium chloride, which, in accordance with IR analysis, leads to increased β‐sheet‐type character. DFT‐based conformational analysis of a simple cysteine thioaldehyde derivative reveals that the top three conformers exhibit ϕ and ψ angles within or close to the ranges associated with pre‐helix, PPII and fully‐extended/β‐sheet‐like secondary structure, respectively. Interestingly, these studies show that small‐molecule cysteine thioaldehydes prefer the pre‐helix type structure (Conformer 1, Figure 10), which in calculations could undergo facile thiocarbonyl hydration (barrier 13–15 kcal/mol), but also primed for deprotonation/enolization to the enethiol(ate), the latter process showing very low barriers in the presence of basic species (acetate, 5–8 kcal/mol). Therefore, the relatively high prevalence for these types of structures in small‐molecule systems and relatively low prevalence in derivatives of pentadecapeptide **31**, might explain the significant levels of formylglycine in photolysis reactions involving the latter, even in the presence of phosphate buffer.

**FIGURE 10:**
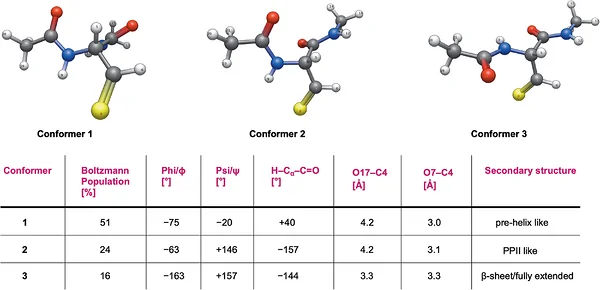
DFT‐calculated structures of a simple cysteine derivative (N‐acetylated, C‐terminal methylamide). O17 = oxygen atom of N‐terminal acetate; O7 = oxygen atom of C‐terminal methylamide; C4 = thiocarbonyl carbon atom.

### 2.7 Implications for FGE/anSME Catalysis, Sulfatase Stability, and the Biosynthetic Origin of Azole‐Containing RiPPs

The activation of formylglycine‐generating enzyme (FGE) and cysteine‐based anaerobic sulfatase‐maturing enzymes (anSME; e.g., AtsB and anSMEcpe) likely proceed via a cysteine thioaldehyde intermediate, which then undergoes hydrolysis (Figure 1D) [21, 22, 23, 24, 25, 26, 27, 28, 29, 30, 31, 32, 33, 34, 35]. In FGE catalysis, the cysteine thiolate of the sulfatase substrate binds to a Cu(I) center; subsequent binding of a molecule of O_2_ leads to a four‐coordinate Cu(II)–O–O· species, which intramolecularly abstracts a hydrogen atom from the β‐methylene of the substrate cysteine to give a Cu(I)‐bound thioaldehyde (Figure 1D) [21, 22, 23, 24, 25, 26, 27, 28, 29]. Clarity on the details of the remaining steps in the catalytic cycle are beginning to emerge. The thioaldehyde—either bound to the Cu center, unbound but still within the active site, or outside of the active site—could undergo hydrolysis through the attack of a water molecule. Recent computational studies suggest that hydrolysis could be initiated through nucleophilic attack of the Cu‐bound peroxide ligand [28]. A more recent experimental study favors a pathway where the peroxide ligand instead engages with one of the FGE thiolate ligands to form a Cu(I)–sulfenate species followed by hydrolysis of the substrate thioaldehyde and reduction of the Cu(I)–sulfenate species [29]. For both mechanisms, a two‐electron reduction of the copper complex is required to complete the cycle. Although the biological reductant is unknown, in vitro catalysis is achieved by using dithiothreitol (DTT) [147, 148, 149, 150].

In anaerobic anSME catalysis, the bound substrate cysteine thiol is in close proximity to two [4Fe‐4S] clusters and a bound molecule of S‐adenosyl methionine (SAM) [35]. It has been suggested that the thiol is deprotonated by a proximal aspartic acid residue, which is crucial for catalysis [35]. The SAM‐derived adenosyl radical abstracts a hydrogen atom from the β‐methylene of the substrate cysteine thiolate. The resulting substrate radical anion then undergoes oxidation to the cysteine thioaldehyde through transfer of a single electron to a proximal [4Fe‐4S] cluster. Presumably, the thioaldehyde then undergoes hydrolysis (Figure 11B).

**FIGURE 11:**
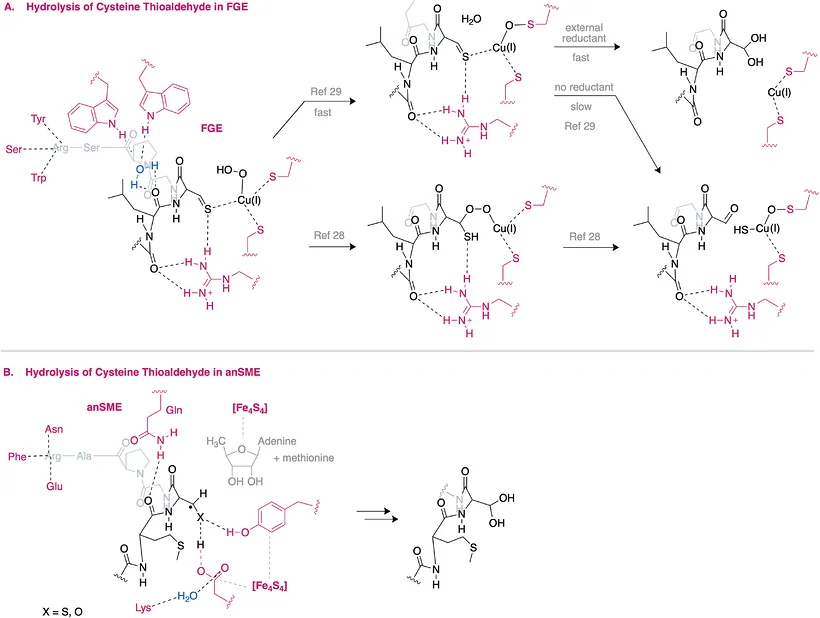
Hydrolysis of cysteine thioaldehydes within the active sites of (A) FGE and (B) anSME. The representations of the active sites of FGE with bound putative cysteine thioaldehyde containing substrates are based on recent crystallographic data of FGE and anSME with bound cysteine‐containing peptide substrate.

We examined crystallographic data of substrate‐bound FGE—from Homo sapiens (PDB 2AIJ) [151] and *Thermomonospora curvata* (PDB 6S07) [26, 27]—and substrate‐bound anSME (AtsB)—from *Clostridium perfringens* (PDB 4K38) [35]—through the lens of our newly established understanding of cysteine thioaldehyde reactivity. Our studies show that cysteine thioaldehydes can undergo hydrolysis into formylglycine with first‐order rate constants (DFT‐calculated reaction barrier 13–15 kcal mol ≈64–1900 s^−1^) that greatly exceed the *k*_cat_ values associated with FGE enzymes (0.023 s^−1^) [23, 26], but with low‐to‐moderate efficiency owing to competing transformations. Therefore, if enzyme‐free cysteine thioaldehydes are intermediates in FGE catalysis, hydrolysis is not rate‐limiting. However, enzymatic control of thioaldehyde hydrolysis is deemed likely so as to avoid other transformations. Indeed Cu‐OOH mediated hydrolysis in the case of FGE catalysis is consistent with this assessment (Figure 11A) [28]. Solomon and Bertozzi's study is consistent with a mechanism involving hydrolysis of the Cu‐bound thioaldehyde through attack of a water molecule [29]. However, this step, which was only observed in the absence of an external reducing agent (DTT), was found to be relatively slow (*k* = 0.003 s^−1^), leading the authors to suggest that it was not relevant to the catalytic cycle of FGE. Therefore, the action of the external reducing agent precedes or is coupled to thioaldehyde hydrolysis.

Our studies suggest that an interaction between a cysteine thioaldehyde and the guanidinium moiety of an arginine has the potential to promote hydrolysis of the thioaldehyde. Sulfatases adopt α‐helical secondary structure in the region where the guanidinium group engages in a stabilizing hydrogen‐binding interaction with the formylglycine residue [131, 132, 133, 134]. However, crystallographic data shows that the cysteine‐containing precursor adopts a different conformation within the FGE and anSME binding sites during catalysis [26, 27, 35]. The sulfatase arginine residue is not available to engage with the putative thioaldehyde; instead, the residue engages strongly with the FGE/anSME pocket, serving as an important anchor (Figure 11A,B). However, there is an FGE‐based arginine residue engaged in a hydrogen‐bonding interaction with the Cu‐bound substrate thiolate, and thus available to promote hydrolysis of an ensuing thioaldehyde (Figure 11A) [26, 27]. In the anSME complex, there is anSME‐based tyrosine and aspartic acid residues engaging with the substrate thiol/thiolate [35]; these residues could promote hydrolysis of the thioaldehyde through hydrogen‐bonding (Figure 11B).

Our studies of a serine → alanine mutant (Figure 9, table entry 10), showed significant amounts of peptide oxazole derived from participation of the backbone with the putative thioaldehyde. When bound to the active site of FGE and anSME, the potentially problematic carbonyl group is preoccupied in a hydrogen‐bonding interaction within the active site that holds it in an orientation that would not support backbone participation on the thioaldehyde (PDB 6S07) [26, 27]. Interestingly, in the FGE complex, the C_α_–H bond of the bound cysteine residue forms a very small dihedral angle with the backbone carbonyl group, a conformation that minimizes C_α_–H acidity and potentially suppresses enolization of the putative thioaldehyde (PDB 6S07) [26, 27].

Backbone participation might be relevant for formylglycine‐containing sulfatases, albeit perhaps as a weak n→π* interaction rather than a fully established O–C bond [130]. In crystallographic studies of sulfatases, the serine (threonine in some sulfatases) hydroxyl group engages in a hydrogen‐bonding interaction with the oxygen atom of the N‐terminal backbone carbonyl group proximal to the formylglycine residue, presumably reinforcing the α‐helical secondary structure and preventing backbone participation on the unhydrated formyl group during sulfatase catalysis (Figure 10, see PDB 7STT [131], 4CXU [133], 1HDH [134]). Such additional stabilization of the α‐helical secondary structure seems necessary as the formylglycine residue is located at one end of the α‐helix close to where it gives way to a random coil structure.

Finally, the intramolecular trapping of the cysteine thioaldehyde by the proximal N‐terminal backbone carbonyl group, appears to have biosynthetic relevance. The research group of Bandarian demonstrated that SbtM, a bacterial radical SAM enzyme, could transform a cysteine residue within a 53‐residue peptide into a 5‐thio‐oxazole moiety [13]. They propose a mechanism involving radical‐SAM‐mediated oxidation of the cysteine thiol to the cysteine thioaldehyde, which undergoes intramolecular trapping to generate the 5‐thio‐oxazoline, and then radical‐SAM mediated oxidation of the sulfur‐bearing methine (Figure 12). Other research groups have since found novel families of RiPPs that contain the same 5‐thio‐oxazole moiety, which appears to be involved in copper homeostasis [14, 15]. Peptide azoles are normally generated biosynthetically through ATP‐driven cyclocondensation to give the azoline, which then undergo flavin‐mediated oxidation to the azole [109, 110, 111]. Our studies suggest that investigations of azole peptides that appear to be generated in the absence of the standard azole biosynthetic enzymes should include consideration of an alternative biosynthetic sequence involving initial cysteine thiol oxidation followed by cyclocondensation.

**FIGURE 12:**
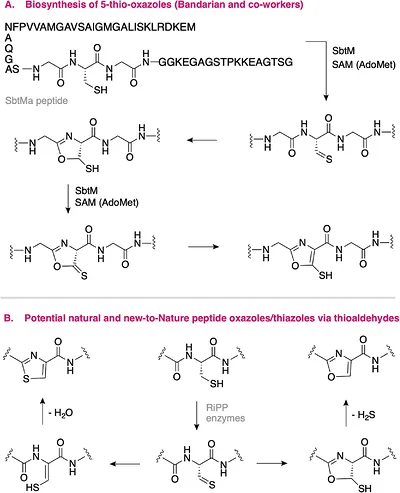
Cysteine thioaldehydes as alternative intermediates toward natural and new‐to‐Nature peptide thiazoles and oxazoles.

## 3 Conclusion

We have used NMR spectroscopy, LC‐MS and computation to investigate the reactivity of cysteine thioaldehydes, as generated through the photolysis of the corresponding phenacylsulfides in aqueous media. The results allow us to make the following conclusions:

- The excited state of phenacylsulfides undergo competing Norrish type‐II and β‐scission processes, the former being favored for substrates bearing non‐conjugating electron‐withdrawing substituents (3,5‐bis(trifluoromethyl)‐ and 4‐trimethylammonium) on the aryl ring.
- Racemization of the cysteine residue can be observed during photolysis. The mechanism of racemization appears to involve reversible 1,6‐HAT involving the excited‐state arylketone carbonyl group and the C_α_–H moiety.
- For simple cysteine derivatives, intermediate thioaldehydes undergo efficient deprotonation/enolization to give a mixture of *E*‐ and *Z*‐configured enethiols (p*K*_a_ 4.3–4.8). At low pH, these enethiols undergo cyclocondensation to give cysteine thiazoles.
- For photolytic reactions with significant levels of β‐scission, which leads to the formation of oxidizing enol radicals, enethiolates either undergoes oxidation to the cysteine isothiazolone or can undergo oxidative dimerization with other thiyl radicals.
- *E*‐configured cysteine enethiolates and cysteine isothiazolones can be interconverted by using either an oxidant (Ellman's reagent) or a reductant (TCEP).
- Depending on sequence and the concentration of buffer, intermediate cysteine thioaldehydes can undergo either nucleophilic attack of a water molecule or be trapped intramolecularly by the proximal N‐terminal backbone carbonyl group. For hydrolysis to formylglycine, DFT studies suggest that the competent species is the conjugate base of the hemi‐thioacetal (p*K*_a_ (calc) 6.8), which undergoes rapid elimination of hydrogen sulfide anion after a slower O → S proton transfer.
- Cysteine thioaldehydes are rapidly trapped by dihydrogen sulfide to give dithioacetal species, which, under oxidizing conditions, can either undergo homo‐ or heterodimerization to give 1,2,4‐trithiolanes and mixed disulfides.
- Cysteine isothiazolones can be viewed as stable isolable masked thioaldehydes: reduction in the presence of TCEP leads to enethiolates, which, at close‐to‐neutral pH, can establish an equilibrium with the corresponding thioaldehyde and avoid cyclocondensation into thiazoles. In the presence of excess TCEP, extremely rapid trapping of cysteine thioaldehyde to give the stable α‐thio‐phosphonium adduct dominates.
- The fate of cysteine thioaldehydes within peptides is highly dependent on secondary structure. Residues or buffer species that can stabilize the ensuing thiolate anion during addition reactions of the thiocarbonyl group (the guanidinium moiety of arginine) or that support a secondary structure that preoccupies the proximal N‐terminal carbonyl group are likely to be critical.
- Formylglycine residues appear to be susceptible to oxidative cleavage, giving C‐terminal primary amide and N‐terminal oxamate peptide fragments.
- The facile generation of peptide thiazoles and oxazoles via cysteine thioaldehydes under biologically relevant conditions suggests that such a pathway might be followed in the biosynthesis of some peptide natural products.

## Conflicts of Interest

The authors declare no conflicts of interest.

## Supporting information




**Supporting File**: The authors have cited additional references within the Supporting Information [152, 153, 154, 155, 156, 157, 158, 159, 160, 161].

## Data Availability

The data that support the findings of this study are available in the supplementary material of this article.
