# Supplementary material for: Cysteine Thioaldehydes: Photolytic Generation, Reactivity, and Biological Implications
Source: Angew Chem Int Ed Engl. 2026 Aug 17;65(41):e26017. doi: 10.1002/anie.202526017 (PMC13638132; doi:10.1002/anie.202526017)
Supplement: Supplementary file 1 [file ANIE-65-e26017-s001.zip › anie74148-sup-0001-SuppMat_Sections A_O.pdf]

Supporting Information

Cysteine Thioaldehydes: Photolytic Generation, Reactivity and Biological Implications

Ardra Karthika,† Matthew Murray,† Guillermo Palop,† Lee-Ann Keane,† Patrick McArdle,† Nicholas D. J. Yates,‡ Christopher D. Spicer,‡ Alison Parkin,‡ Martin Fascione,‡ and Eddie L. Myers*†

† School of Biological and Chemical Sciences, University of Galway, University Road, Galway H91 TK33, Ireland

‡ Department of Chemistry, University of York, Heslington, York YO10 5DD, United Kingdom.

Correspondence: Eddie.Myers@universityofgalway.ie

Table of Contents

**A.**	General experimental	3
**B.**	Synthesis of cysteine derivative	7
**C.**	Synthesis of phenacylbromide compounds	10
**D.**	Synthesis of cysteine-containing peptides and phenacylsulfide derivatives	16
**E.**	Photolysis of simple cysteine phenacylsulfide derivatives	28
**F**.	Investigation of simple cysteine isothiazolone and enethiol derivatives	42
**G**.	Synthesis and hydrolysis of formylglycine diethylacetal derivative	50
**H**.	Photolysis of simple cysteine phenacylsulfides in non-buffered aqueous media.	54
**I**.	Photolysis of tripeptide phenacylsulfide derivatives	153
**J**.	Investigation of tripeptide isothiazolone and enethiol derivatives	170
**K**.	Photolysis of pentadecapeptide phenacylsulfide derivatives	193
**L**.	X-ray diffraction data of simple isothiazolone derivative	305
**M**.	NMR Spectra	306
**N**.	Kinetics studies	432
**O**.	IR studies	443

A. General Experimental

Chemicals:

The following starting materials, reagents and solvents were purchased from the indicated supplier and generally used as supplied. Procedures for known and novel compounds have been provided in the sections below and the associated literature has been cited.

Starting Materials and Reagents	Supplier
(2-Bromo-1-(2, 4-dimethoxyphenyl)-ethanone<br>2-bromo-1-(3-(trifluoromethyl)phenyl) ethan-1-one<br>2-bromo-1-(bis-3,5-(trifluoromethyl)phenyl)ethan-1-one<br>3-(2-bromoacetyl) benzonitrile<br>3,4-dihydronaphthalen-1(*2H*)-one<br>3-dimethylaminoacetophenone<br>4-(2-bromoacetyl) benzonitrile<br>4-ethynyl-acetophenone<br>4-hydroxy-2-bromoacetophenone<br>4-methylpiperidine<br>Acetic anhydride<br>Bromine<br>Copper bromide<br>Diethylphosphite<br>Methyl iodide<br>Methyl trifluoromethanesulfonate<br>Sodium iodide<br>Trifluoroacetic acid (TFA)<br>2,2,2-Trifluoroethanol (TFE)	Sigma Aldrich/Merck
2-bromoacetophenone<br>4-dimethylacetophenone<br>5,5′-dithiobis(2-nitrobenzoic acid) (DTNB)<br>Benzyl amine<br>Benzyl bromide<br>Disodium hydrogen phosphate<br>Guanidinium hydrochloride<br>Magnesium sulfate<br>2,3,4,5,6-pentafluroacetophenone<br>N,N-diisopropylethylamine (DIEA)<br>O-benzyl hydroxylamine hydrochloride<br>Sodium carbonate<br>Triethylsilane (TES)<br>Trimethylamine	Fluorochem Ltd
Iodine<br>N,N-diisopropylcarbodiimide (DIC)<br>Ethyl cyanohydroxyiminoacetate (Oxyma)<br>*S*-(triphenylmethyl) cysteine<br>Triisopropylsilane (TIS)<br>Tris(2-carboxyethyl)phosphine hydrochloride (TCEP)	TCI chemicals

30% hydrogen peroxide<br>Hydrochloric acid (36%)<br>Potassium iodide<br>Sodium hydroxide<br>Sulfuric acid (99.9%)	Fisher Scientific Ltd
Sodium periodate ($NaIO_4$)	BDH
Chloroform-d (> 99.80% D)<br>Methanol-d_4 (> 99.80% D)<br>Deuterium oxide (> 99.90% D)<br>Acetonitrile-d_3 (> 99.80% D)<br>Dimethylsulfoxide-d_6 (> 99.80% D)<br>Acetone-d_6 (> 99.80% D)	Deutero GmbH
Fmoc-protected amino acid building blocks<br>Piperidine<br>N-methyl morphine<br>Fmoc-rink amide AM resin (0.64 mmol/g)	Iris Biotech GmbH

**Solvents**	**Supplier**
Tetrahydrofuran (THF)<br>Petroleum ether<br>Methanol<br>Water (HPLC grade)<br>Acetonitrile (HPLC grade)<br>Cyclohexane<br>Ethyl acetate<br>N,N-dimethylformamide (DMF, reagent grade)	Fisher Scientific Ltd
N,N-dimethylformamide (DMF, peptide synthesis grade)	Iris Biotech GmbH
Dichloromethane (DCM)<br>Diethyl ether (DEE)<br>*tert*-butyl methyl ether (TBME)	Sigma Aldrich/Merck

**Materials**	**Supplier**
Reactors for peptide synthesis, 5 mL	Carl Roth GmbH

Synthesis:
Photolysis reactions were performed on an analytical scale by using solutions (10.8 mM for simple cysteine phenacylsulfide derivatives; 1.7 mM for pentadecapeptide phenacylsulfide derivatives) within a quartz NMR tube and placed within a Rayonet photochemical reactor (RPR-100, The Southern New England Ultraviolet Co.) with 350 nm ultraviolet lamps. For preparative-scale reactions of simple cysteine phenacylsulfide derivatives (6.4 mM), Pyrex tubes were used as vessels. Solutions were degassed with N_2 gas followed by sealing before illumination. The operation of the incorporated fan within the RPR-100 unit kept reaction mixtures below 35 °C during photolysis.

Automated solid phase peptide synthesis (SPPS) was performed on a CEM Multipep 1 instrument and peptides were grown on a Rink amide resin (0.64 mmol/g, Iris Biotech) on a scale of ca. 100 μmol. Standard N^{α}-Fmoc-protected amino acids (Iris Biotech) were used. Fmoc deprotection was achieved by using piperidine (20% in DMF) and amide bond coupling was achieved by using Oxyma/DIC. Reagent-grade DMF was used for washing steps and, peptide-grade DMF was used for Fmoc deprotection and coupling reactions. Peptides were N-acetylated after addition and Fmoc deprotection of the last amino acid building block (acetic anhydride, *N*-methylmorpholine, DMF (peptide grade)). Peptide cleavage was effected with the following cocktail of chemicals: (TFA/TIS/H_2O, 95:2.5:2.5, 5 mL). The filtrate was concentrated over a stream of air and crude peptide was precipitated with *tert*-butyl methyl ether (TBME) and isolated through centrifugation/decanting cycles. More detailed protocols are provided in the relevant sections below.

Purification:
Flash column chromatography was carried out by using silica gel (Sigma Aldrich, technical grade, particle size 40–63 µm). Semi-preparative HPLC was performed on an Agilent 1260 Infinity HPLC system with an Agilent 1200 series auto sampler. A Macherey Nagel NUCLEODUR C18 HTec column (10 x 250 mm, 5 μm packing diameter) at 24 °C with a flow rate of 2.5 mL/min was used and the absorbance were measured at 214 nm. The eluent used was a gradient composed of mixtures of the following components: A = 0.1 % v/v solution of TFA in water; B = 0.1 % v/v solution of TFA in acetonitrile. Collected fractions were frozen and lyophilised.

Analysis
Thin layer chromatography (TLC) was carried out on Merck TLC Silica gel 60 F_{254} plates by using a UV lamp (λ = 254 nm) for visualization.

Proton (^{1}H) and carbon (^{13}C) NMR spectra and associated homonuclear and heteronuclear 2D experiments were recorded on the following instruments: (a) JEOL NMR ECZ-S 400 with Autotune™ Royal™ 5mm RT Probe (400 MHz and 100.6 MHz); (b) Agilent NMR with OneNMR Probe (500 MHz and 125.7 MHz); Agilent NMR with $^{1}H(^{13}C/^{15}N)$ 5 mm 13C Enhanced Cold Probe (600 MHz and 150.9 MHz). ^{19}F NMR spectra were recorded on an Agilent NMR with OneNMR Probe (470 MHz). ^{31}P NMR spectra were recorded on an Agilent NMR with OneNMR Probe (200 MHz). NMR assignments are supported by 2-D ^{1}H–^{1}H and ^{1}H–^{13}C correlation experiments. NMR data is reported as follows: chemical shift in ppm, integration, multiplicity (s = singlet, d = doublet, dd = doublet of doublet, t = triplet, q = quartet, sep = septet, m = multiplet, bs = broad singlet, app.t = apparent triplet, app.q = apparent quartet, ABq = AB quartet) and coupling constants in Hertz (Hz).

Analytical HPLC was performed on an Agilent 1260 Infinity HPLC system with an Agilent 1260 Infinity auto sampler. An Agilent Zorbax RX-C18 column (4.6 x 250 mm, 5 µm packing diameter) at 24 °C with a flow rate of 1 mL/min was used and the absorbance were measured at 214 nm. The eluent used was a gradient composed of mixtures of the following components: A = 0.1 % v/v solution of TFA in water; B = 0.1 % v/v solution of TFA in acetonitrile.

High-resolution mass spectra were recorded on an Agilent 6530 Accurate-Mass Q-TOF LC/MS system. An Acquity UPLC® BEH C18 column (2.1 x 150 mm, 1.7 µm packing diameter) was used with a flow rate of 0.3 mL/min. The solvents employed were as follows: A = 0.1 % v/v solution of formic acid in water. B = 0.1 % v/v solution of formic acid in acetonitrile.

Infrared spectra were recorded using a Perkin-Elmer Spec 1 with ATR attached. FT-IR data are reported in wavenumber(cm^{-1}) within the range 500–4000 cm^{-1}.

Far ultraviolet (UV) CD spectra of peptides (50 µL, 64 µM) were recorded on a Chirascan VX Circular Dichroism spectrometer. Spectra were recorded in the range 190–250 nm by using a 0.1 mm path length cuvette. The distributions of secondary structural elements were subsequently obtained by uploading the raw data for processing on the BeStSel (Beta Structure Selection) webserver: https://bestsel.elte.hu/index.php.

pH Meter measurements were recorded by using a Mettler Toledo FiveEasy Plus pH meter FP20 equipped with an LE-422 sensor for small volumes. pD values were obtained by adding 0.4 units to the recorded pH-meter reading value.

Single-crystal x-ray structure analysis (for compound **6**) was obtained by collecting data on a XCalibur S Single Crystal X-Ray diffractometer.

B. Synthesis of cysteine derivative

N-acetyl-*S*-(triphenylmethyl) cysteine (1a)

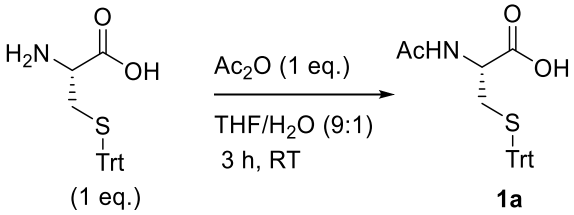

S-(triphenylmethyl) cysteine (6.00 g, 16 mmol) was suspended in THF/H_2O 9:1 (160 mL) at room temperature. Acetic anhydride (1.9 mL, 16 mmol) was added to the suspension over 15 minutes to give an amber solution. Upon completion, as determined by TLC analysis, the mixture was concentrated *in vacuum* at 40 ^{o}C. *N*-acetyl-*S*-(triphenylmethyl) cysteine **1a** was obtained as a white solid and used in the next step without further purification. Spectroscopic analysis was in agreement with the literature.[152]

TLC - R_f 0.68 (EtOAc/MeOH/H_2O, 4:1:1).

^{1}H NMR (DMSO-d_6, 500 MHz): δ 7.92 (d, J = 8.0 Hz, 1H), 7.19–7.31 (m, 15H), 4.12 (m, 1H), 2.34–2.43 (m, 2H), 1.81 (s, 3H) ppm.

^{13}C NMR (DMSO-d_6, 125 MHz): δ 172.5, 169.2, 144.9, 129.5, 128.4, 127.1, 66.1, 52.4, 34.6, 23.0 ppm.

N^2-acetyl-*N*-benzyl-*S*-(triphenylmethyl) cysteinamide (1b)

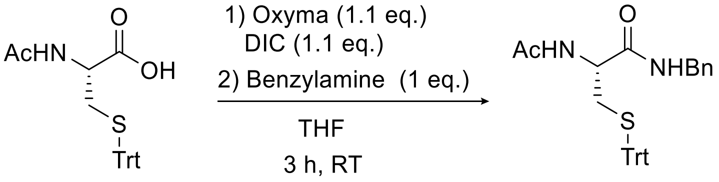

1a (1 eq.) **1b**

Crude *N*-acetyl-*S*-(triphenylmethyl) cysteine **1a** (ca. 16 mmol) was dissolved in THF (190 mL) and ethyl 2-cyano-2-(hydroxyimino)acetate (2.58 g, 18 mmol) and *N*,*N′*-diisopropylcarbodiimide (2.84 mL, 18 mmol) were added at room temperature. Then, benzylamine (1.8 mL, 16 mmol) was added giving a yellow solution. After completion of the reaction, the mixture was concentrated *in vacuum* and the resulting yellow residue was dissolved in Et_2O (50 mL) and was let to stand. Petroleum ether (250 mL) was added to promote precipitation of N^2-acetyl-*N*-benzyl-*S*-(triphenylmethyl) cysteinamide **1b** as a white solid together with the by-product, *N*,*N′*-diisopropylurea. The mixture was used in the next step without additional purification.

TLC - R_f 0.55 (EtOAc/MeOH/H_2O, 5:2:1).

^1H NMR (Chloroform-*d*, 500 MHz): δ 7.38–7.46 (m, 6H), 7.18–7.34 (m, 14H), 6.30 (app. t, *J* = 5.8 Hz, NH), 5.87 (d, *J* = 7.6 Hz, NH), 4.39 (dd, *J* = 14.9, 5.8 Hz, 1H), 4.33 (dd, *J* = 14.9, 5.8 Hz, 1H), 4.13 (app. q, *J* = 6.8 Hz, 1H), 2.78 (dd, *J* = 13.1, 6.8 Hz, 1H), 2.56 (dd, *J* = 13.1, 6.0 Hz, 1H), 1.89 (s, 3H) ppm.

^{13}C NMR (Chloroform-*d*, 125 MHz): δ 170.1, 169.9, 144.4, 137.6, 129.5, 128.6, 128.1, 127.7, 127.5, 126.9, 67.2, 52.3, 43.6, 33.4, 23.1 ppm.

HRMS (ESI) *m/z* $(M+H)^+$, $C_{31}H_{31}N_2O_2S$ calcd. 495.2106, observed 495.2120.

N^2-acetyl-*N*-benzylcysteinamide (1)

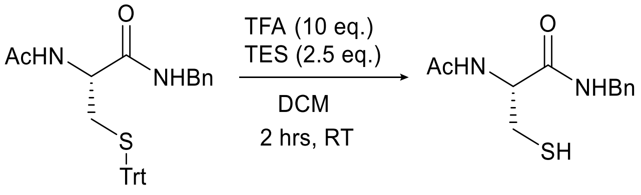

1b (1 eq.) **1** (80% yield)

To a solution of crude N^2-acetyl-*N*-benzyl-*S*-(triphenylmethyl) cysteinamide **1b** (ca. 16 mmol) in DCM (60 mL) was added trifluoroacetic acid (12.2 mL, 160 mmol) and triethylsilane (6.40 mL, 40 mmol). The reaction mixture was then allowed to stir for 1 hour at RT. The residue was purified by flash column chromatography by using cyclohexane/ethyl acetate (5:1) as eluent to give N^2-acetyl-*N*-benzylcysteinamide **1** (2.7 g, 67 % yield) as a white solid (m.p. 180–181 ºC).

TLC - R_f 0.36 (EtOAc/cyclohexane, 5:1).

^{1}H NMR (Chloroform-*d*, 500 MHz): δ 7.09–7.42 (m, 5H), 6.83 (br. s, NH), 6.57 (d, *J* = 7.3 Hz, NH), 4.62 (app. q, *J* = 7.3 Hz, 1H), 4.48 (dd, *J* = 14.7, 5.8 Hz, 1H), 4.43 (dd, *J* = 14.7, 5.8 Hz, 1H), 3.05 (ddd, *J* = 14.0, 7.7, 4.4 Hz, 1H), 2.76 (ddd, *J* = 14.0, 10.1, 7.3 Hz, 1H), 2.03 (s, 3H) ppm.

^{13}C NMR (Chloroform-*d*, 125 MHz): δ 170.4, 169.7, 137.6, 128.7, 127.7, 127.6, 54.3, 43.7, 26.7, 23.1 ppm.

HRMS (ESI) *m/z* $(M+H)^+$, $C_{12}H_{17}N_2O_2S$ calcd. 253.1005, observed 253.1011;

IR ν_{max} (neat, cm^{-1}) 3282, 3086, 2930, 1736, 1630, 1541, 1535, 1452, 1369, 1242, 1225.

C. Synthesis of phenacylbromide compounds

2-bromo-1-(perfluorophenyl) ethan-1-one (2g)

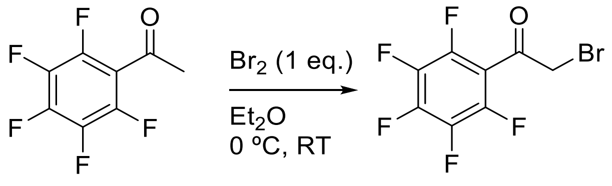

This phenacylbromide was prepared by following a protocol by Chapman et al[2]. Br_2 (0.18 mL, 3.51 mmol) was added slowly to a stirred solution of 2,3,4,5,6-pentafluoroacetophenone (0.50 mL, 3.51 mmol) in Et_2O (14 mL) at 0 ºC. The mixture was left to stir overnight at room temperature. The reaction mixture was washed with water and aq. Na_2CO_3 (10%). The organic layer was dried over $MgSO_4$ and evaporated *in vacuum*. The residue was purified by flash column chromatography by using cyclohexane/DCM (9:1) as eluent to give 2-bromo-1-(perfluorophenyl) ethan-1-one **2g** (560 mg, 55% yield) as a white solid. Spectroscopic analysis was in agreement with the literature.[153]

^{1}H NMR (Chloroform-*d*, 500 MHz): δ 4.31 ppm (s, 2H).

^{19}F NMR (Chloroform-*d*, 470 MHz): δ –159.33 (m, 2F), –146.92 (tt, J = 20.8, 4.8 Hz, 1F), –139.63 ppm (m, 2F).

3-(2-bromoacetyl)-*N*,*N*,*N*-trimethylbenzenaminium trifluoromethylsulfonate (2h)

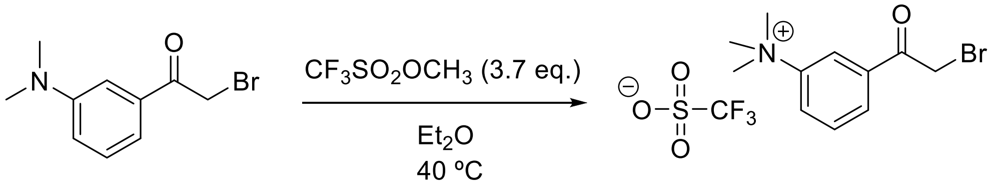

2j (1 eq.) **2h** (85% yield)

Methyl trifluoromethanesulfonate (0.16 mL, 1.48 mmol) was added to a stirred solution of 2-bromo-1-(3-(dimethylamino) phenyl) ethan-1-one **2j** (100 mg, 0.40 mmol) in Et_2O (12 mL) at 40 ºC. Over 2 hours, a brown oil separated and solidified. The solid was suspended in water and centrifuged. The supernatant was collected and lyophilised to afford 3-(2-bromoacetyl)-*N*,*N*,*N*-trimethylbenzenaminium trifluoromethylsulfonate **2h** (124 mg, 85% yield) as a white powder (m.p. 172–173 ºC).

TLC - R_f 0.00 EtOAc/cyclohexane (8:2).

^{1}H NMR (D_2O, 500 MHz): δ 8.42 (dd, J = 2.6, 1.2 Hz 1H), 8.25 (d, J = 7.8 Hz, 1H), 8.17 (dd, J = 8.5, 2.6 Hz, 1H), 7.85 (app. t, J = 7.8 Hz, 1H), 4.89 (s, 2H), 3.72 ppm (s, 9H).

^{13}C NMR (D_2O, 126 MHz): δ 193.4, 158.6, 135.5, 131.3, 131.0, 125.1, 119.9, 56.9, 32.8 ppm.

^{19}F NMR (D_2O, 470 MHz): δ –78.88 ppm (3F, s).

IR ν_{max} (neat, cm^{-1}) 1703, 1591, 1492, 1384, 1256, 1158, 1028, 953, 881, 808, 755, 685, 633.

4-(2-bromoacetyl)-*N*,*N*,*N*-trimethylbenzenaminium trifluoromethylsulfonate (2i)

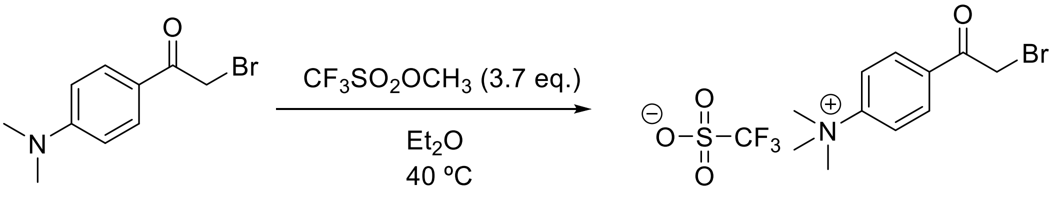

2k (1 eq.) **2i** (77% yield)

Methyl trifluoromethanesulfonate (0.81 mL, 7.4 mmol) was added to a stirred solution of 2-bromo-1-(4-(dimethylamino)phenyl) ethan-1-one **2k** (500 mg, 2.0 mmol) in Et_2O (20 mL) at 40 ºC. Over 2 hours, a brown oil separated and solidified. The solid was suspended in water and centrifuged. The supernatant was collected and lyophilised to afford 4-(2-bromoacetyl)-*N*,*N*,*N*-trimethylbenzenaminium trifluoromethylsulfonate **2i** (570 mg, 77% yield) as a white powder (m.p. 95–96 ºC).

TLC- R_f 0.00 EtOAc/cyclohexane (8:2).

^{1}H NMR (D_2O, 500 MHz): δ 8.26 (m, 2H), 8.03 (m, 2H), 4.87 (s, 2H), 3.71 ppm (s, 9H).

^{13}C NMR (D_2O, 126 MHz): δ 193.6, 150.2, 135.2, 130.9, 120.6, 56.8, 32.9 ppm.

^{19}F NMR (D_2O, 470 MHz): δ –78.88 ppm (s, 3F).

IR ν_{max} (neat, cm^{-1}) 3047, 1680, 1604, 1449, 1252, 1144, 1024, 940, 850, 757, 703, 633.

2-bromo-1-(3-(dimethylamino) phenyl)ethan-1-one (2j)

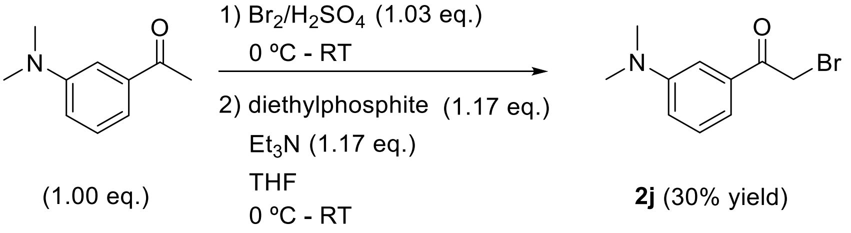

This phenacylbromide was prepared by following a protocol by Diwu et al[3]. 3-dimethylaminoacetophenone (1.00 g, 6.12 mmol) was dissolved in cold H_2SO_4 (6 mL) with the help of a sonicator. Then Br_2 (0.32 mL, 6.31 mmol) was slowly added to the stirred solution at 0 °C. The mixture was then allowed to stir at room temperature overnight. The reaction mixture was poured over ice. The aqueous mixture was washed with several portions of DCM, which were combined, dried over $MgSO_4$, and concentrated *in vacuum* to give crude 2,2-dibromo-3'-dimethylaminoacetophenone (1.85 g) as an orange solid. Et_3N (1.00 mL, 7.2 mmol) and diethylphosphite (0.93 mL, 7.2 mmol) was added to a stirred solution of the solid in THF (21 mL) at 0 °C. The mixture was allowed to stir overnight at room temperature. The mixture was concentrated *in vacuum*. Purification of the residue by flash column chromatography by using cyclohexane/EtOAc (8:1) as eluent gave 2-bromo-1-(3-(dimethylamino) phenyl) ethan-1-one **2j** (415 mg, 30% yield) as a yellow solid. Spectroscopic analysis was in agreement with the literature.[154]

^{1}H NMR (Chloroform-*d*, 500 MHz): δ 7.30–7.35 (m, 2H), 7.25–7.28 (m, 1H), 6.95 (ddd, J = 8.2, 2.8, 1.0 Hz, 1H), 4.46 (s, 2H), 3.01 ppm (s, 6H).

^{13}C NMR (Chloroform-*d*, 126 MHz): δ 191.9, 150.7, 134.7, 129.3, 117.7, 116.9, 111.7, 40.4, 31.4 ppm.

2-bromo-1-(4-(dimethylamino) phenyl) ethan-1-one (2k)

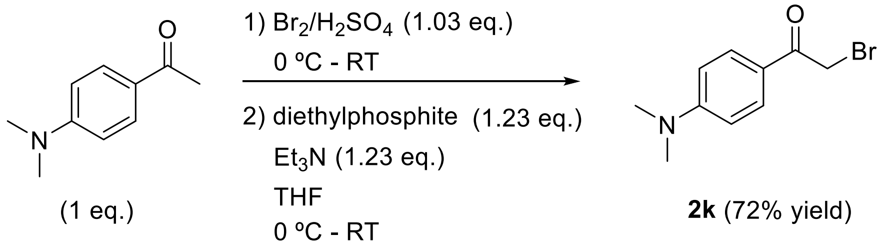

This phenacylbromide was prepared by following a protocol by Diwu et al[3].
4-dimethylaminoacetophenone (2.00 g, 12.3 mmol) was dissolved in cold H_2SO_4 (12 mL) with the help of a sonicator. Br_2 (0.65 mL, 12.7 mmol) was slowly added to the stirred solution at 0 ºC, and was allowed to stir at room temperature overnight. The mixture was poured over ice and the green precipitate, 2,2-dibromo-4'-dimethylaminoacetophenone (3.92 g), was collected. Et_3N (2.13 mL, 15.25 mmol) and diethylphosphite (1.97 mL, 15.25 mmol) was added to a stirred solution of the solid in THF (44 mL) at 0 ºC. The mixture was allowed to stir overnight at room temperature. The mixture was poured over ice. The resulting green precipitate was washed with water and collected by filtration to afford 2-bromo-1-(4-(dimethylamino) phenyl) ethan-1-one **2k** (2.13 g, 72% yield). Spectroscopic analysis was in agreement with the literature.[154]

^{1}H NMR (Chloroform-*d*, 500 MHz): δ 7.89 (d, J = 9.1 Hz, 2H), 6.66 (d, J = 9.1 Hz, 2H), 4.36 (s, 2H), 3.08 ppm (s, 6H).

2-bromo-3,4-dihydronapthalen-1(*2H*)-one (2l)

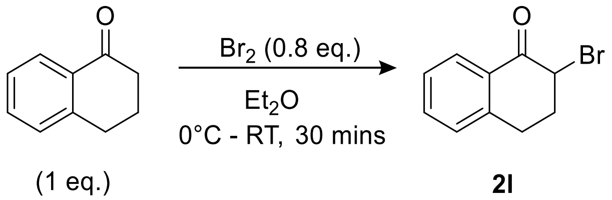

Br_2 (0.8 g, 5.47 mmol) was added to a stirred solution of 3,4-dihydronaphthalen-1(*2H*)-one (0.9 g, 6.84 mmol) in Et_2O (3.7 mL) at 0 °C. The resulting mixture was then allowed to stir for 15 mins, warmed to room temperature, and stirred for a further 15 mins. Water (10 mL) was added to the reaction mixture and the organic layer was separated, washed with two 10 mL portions of sat. aq. $Na_2S_2O_3$ and 10 mL of water. The organic layer was then dried over $MgSO_4$ and concentrated *in vacuum* to yield an inseparable mixture of the target bromide and starting material in an 8:2 ratio favouring the bromide (981 mg, 86% purity). The crude product was used without further purification. Spectroscopic data was in agreement with the literature.[155,156]

TLC - R_f 0.83 EtOAc/cyclohexane (1:1).

^{1}H NMR (Chloroform-*d* , 500 MHz) δ 8.09 (dd, J = 7.3, 1.3 Hz, 1H), 7.52 (app. td, J = 7.5, 1.3 Hz, 1H), 7.35 (app. t, J = 7.3 Hz, 1H), 7.28 (d, J = 7.5 Hz, 1H), 4.73 (app. t, J = 4.3 Hz, 1H), 3.31 (ddd, J = 17.1, 10.1, 4.3 Hz, 1H), 2.92 (dt, J = 17.1, 4.3 Hz, 1H), 2.57–2.41 ppm (m, 2H).

D. Synthesis of cysteine-containing peptides and phenacylsulfide derivatives

N²**-acetyl-*N*-benzyl-*S*-[2-(2, 4-dimethoxyphenyl)-2-oxoethyl] cysteinamide (3a)**

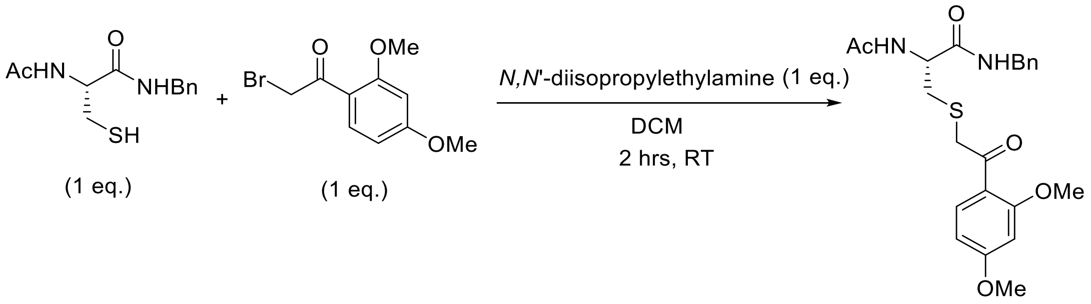

3a (75% yield)

(2-Bromo-1-(2,4-dimethoxyphenyl)-ethanone, (103 mg, 0.40 mmol) and *N,N*-diisopropylethylamine (0.07 mL, 0.40 mmol) were added to a stirred solution of N^2-acetyl-*N*-benzylcysteinamide **1** (100 mg, 0.40 mmol) in DCM (20 mL) at room temperature. The reaction mixture was then stirred at room temperature for 2 hrs. The mixture was concentrated *in vacuum*. The residue was purified by silica gel flash column chromatography by using EtOAc/cyclohexane (8:2) as eluent to give N^2-acetyl-*N*-benzyl-*S*-[2-(2,4-dimethoxyphenyl)-2-oxoethyl] cysteinamide **3a** (128 mg, 75% yield) as a white solid (m.p. 210–211 ºC).

TLC - R_f 0.11 EtOAc/pet ether (8:2).

^{1}H NMR (Chloroform-*d*, 500 MHz): δ 7.86 (d, *J* = 8.8 Hz, 1H), 7.49 (t, *J* = 5.0 Hz, NH), 7.24–7.32 (m, 5H), 7.05 (d, *J* = 7.0 Hz, NH), 6.53 (dd, *J* = 8.8, 2.3 Hz, 1H), 6.44 (d, *J* = 2.3 Hz, 1H), 4.58 (app. q, *J* = 7.1 Hz, 1H), 4.49 (dd, *J* = 15.1, 5.0 Hz, 1H), 4.45 (dd, *J* = 15.1, 5.0 Hz, 1H), 4.16–3.98 (m, 2H), 3.89 (s, 3H), 3.85 (s, 3H), 3.07 (dd, *J* = 13.9, 4.8 Hz, 1H), 2.74 (dd, *J* = 13.9, 7.1 Hz, 1H), 2.01 ppm (s, 3H).

^{13}C NMR (Chloroform-*d*, 125 MHz): δ 195.3, 170.4, 170.2, 165.3, 161.2, 137.9, 133.5, 128.6, 127.5, 127.3, 118.8, 105.8, 98.3, 55.7, 55.6, 52.7, 44.0, 43.6, 35.2, 23.1 ppm.

HRMS (ESI) *m/z* $(M+H)^+$, $C_{22}H_{25}N_2O_5S$ calcd. 431.1641, observed 431.1634.

IR ν_{max} (neat, cm^{-1}) 3267, 3067, 2920, 1735, 1663, 1630, 1602, 1544, 1498, 1455, 1415, 1368, 1291, 1213, 1160, 1128, 1025.

N^2-acetyl-*N*-benzyl-*S*-(2-oxo-2-phenylethyl) cysteinamide (3b)

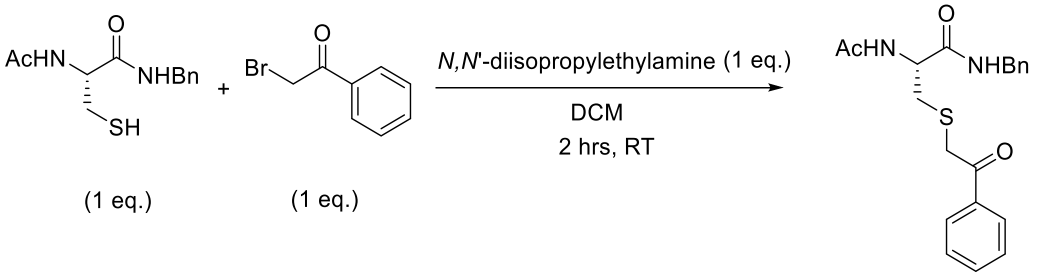

3b (86% yield)

2-bromoacetophenone (290 mg, 1.19 mmol) and *N*,*N*-diisopropylethylamine (0.21 mL, 1.19 mmol) were added to a stirred solution of N^2-acetyl-*N*-benzylcysteinamide **1** (300 mg, 1.19 mmol) in DCM (60 mL). The reaction mixture was stirred at room temperature for 2 hrs. The mixture was concentrated *in vacuum*. The residue was purified by silica gel flash column chromatography by using EtOAc/cyclohexane (8:2) as eluent to give N^2-acetyl-*N*-benzyl-*S*-(2-oxo-2-phenylethyl) cysteinamide **3b** (380 mg, 86% yield) as a white solid (m.p. 148–149 ºC).

TLC - R_f 0.26 EtOAc/pet ether (8:2).

^{1}H NMR (Chloroform-*d*, 500 MHz) δ 7.95 (d, *J* = 7.4 Hz, 2H), 7.60 (t, *J* = 7.4 Hz, 1H), 7.47 (t, *J* = 7.4 Hz, 2H), 7.36 (br. t, *J* = 5.5 Hz, NH), 7.22–7.34 (m, 5H), 6.89 (d, *J* = 7.1 Hz, NH), 4.60 (app. q, *J* = 7.1 Hz, 1H), 4.48 (dd, *J* = 15.0, 5.0 Hz, 1H), 4.46 (dd, *J* = 15.0, 5.0 Hz, 1H), 4.03–4.15 (m, 2H), 3.06 (dd, *J* = 14.2, 5.5 Hz, 1H), 2.77 (dd, *J* = 14.2, 7.1 Hz, 1H), 2.02 ppm (s, 3H).

^{13}C NMR (Chloroform-*d*, 125 MHz): δ 196.9, 170.4, 170.0, 137.7, 135.2, 133.9, 128.8, 128.6, 128.6, 127.6, 127.4, 52.3, 43.7, 39.0, 35.3, 23.1 ppm.

HRMS (ESI) *m/z* $(M+H)^+$, $C_{20}H_{23}N_2O_3S$ calcd. 371.1429, observed 371.1415.

IR ν_{max} (neat, cm^{-1}) 3269, 3064, 2927, 1739, 1675, 1630, 1543, 1445, 1368, 1277, 1235, 1015.

(*R*)-2-acetamido-N-benzyl-3-((2-(3-cyanophenyl)-2-oxoethyl) thio) propanamide (3c)

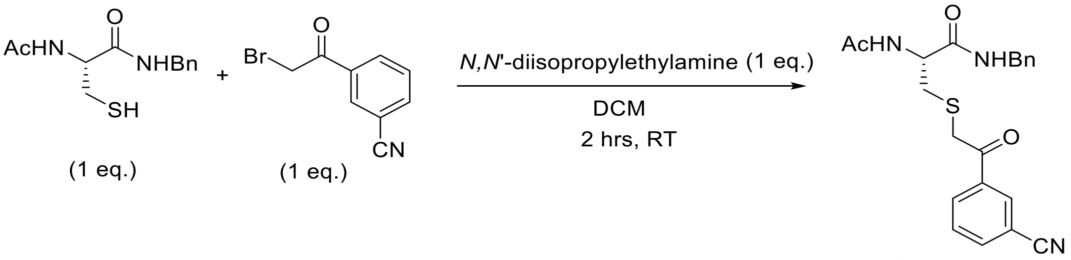

3c (86% yield)

3-(2-bromoacetyl) benzonitrile, (265 mg, 1.19 mmol) and *N*,*N*-diisopropylethylamine (0.21 mL, 1.19 mmol) was added to a stirred solution of N^2-acetyl-*N*-benzylcysteinamide **1** (300 mg, 1.19 mmol) in DCM (60 mL). The reaction mixture was stirred at room temperature for 2 hrs. The mixture was concentrated *in vacuum*. The residue was purified by silica gel flash column chromatography by using EtOAc/cyclohexane (8:2) as eluent to give N^2-acetyl-*N*-benzyl-*S*-(2-oxo-2-phenylethyl) cysteinamide **3c** (92 mg, 60%) as a white solid (m.p. 113–115 ºC).

TLC - R_f 0.12 (80:20 EtOAc/pet. ether).

^{1}H NMR (Chloroform-*d*, 500 MHz) δ 8.19 (s, 1H), 8.12 (d, *J* = 7.8 Hz, 1H), 7.83 (d, *J* = 7.8 Hz, 1H), 7.61 (app. t, *J* = 7.8 Hz, 1H), 7.28–7.34 (m, 6H), 6.78 (d, *J* = 7.5 Hz, NH), 4.61 (app. q, *J* = 7.1 Hz, 1H), 4.44 (dd, *J* = 14.8, 6 Hz, 1H), 4.39 (dd, *J* = 14.8, 6 Hz, 1H), 4.04 (m, 2H), 2.99 (dd, *J* = 14.2, 5.7 Hz, 1H), 2.80 (dd, *J* = 14.2, 7.1 Hz, 1H), 2.01 ppm (s, 3H).

^{13}C NMR (Chloroform-*d*, 126 MHz) δ 193.7, 170.5, 170.0, 137.7, 136.6, 136.2, 132.7, 132.5, 130.0, 128.9, 127.7, 127.7, 117.8, 113.6, 52.2, 43.8, 38.6, 35.3, 23.3 ppm.

HRMS (ESI) *m/z* (*M*−H), $C_{21}H_{20}N_3O_3S$ calcd. 394.1225, observed 394.1221.

(*R*)-2-acetamido-N-benzyl-3-((2-(4-cyanophenyl)-2-oxoethyl) thio) propanamide (3d)

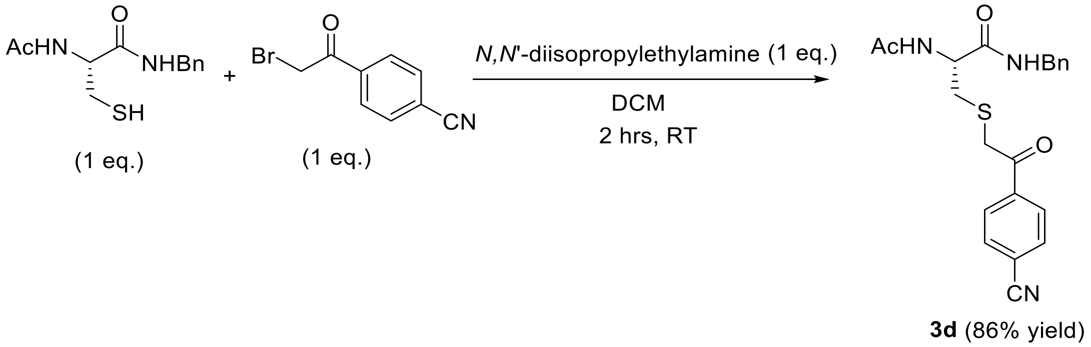

4-(2-bromoacetyl) benzonitrile, (265 mg, 1.19 mmol) and *N*,*N*-diisopropylethylamine (0.21 mL, 1.19 mmol) were added to a stirred solution of N^2-acetyl-*N*-benzylcysteinamide **1** (300 mg, 1.19 mmol) in DCM (60 mL). The reaction mixture was stirred at room temperature for 2 hrs. After completion, the mixture was concentrated *in vacuum*. The residue was purified by silica gel flash column chromatography by using EtOAc/cyclohexane (8:2) as eluent to give **3d** (74 mg, 47%) as a white solid (m.p. 150–151 °C).

TLC - R_f 0.18 (80:20 EtOAc/pet. ether).

^{1}H NMR (Chloroform-*d*, 500 MHz,) δ 8.03 (d, J = 8.1 Hz, 2H), 7.77 (d, J = 8.1 Hz, 2H), 7.20 –7.40 (m, 5H), 7.14 (t, J = 5.8 Hz, NH), 6.70 (d, J = 7.3 Hz, NH), 4.57 (app. q, J = 7.3 Hz, 1H), 4.48 (dd, J = 15.1, 5.8 Hz, 1H), 4.44 (dd, J = 15.1, 5.8 Hz, 1H), 4.08 (ABq, $\Delta\delta_{AB}$ = 0.03, J_{AB} =15 Hz, 2H), 3.01 (dd, J = 14.3, 5.3 Hz, 1H), 2.78 (dd, J = 14.3, 7.3 Hz, 1H), 2.03 ppm (s, 3H).

^{13}C NMR (Chloroform-*d*, 126 MHz) δ 195.6, 170.5, 169.9, 138.5, 137.7, 132.8, 129.2, 128.9, 127.7, 127.7, 117.9, 117.2, 52.3, 43.9, 39.1, 35.4, 23.3 ppm.

HRMS (ESI) *m/z* (*M*+H)$^+$, $C_{21}H_{22}N_3O_3S$ calcd. 396.1382, observed 396.1401.

(*R*)-2-acetamido-N-benzyl-3-((2-oxo-2-(3-(trifluoromethyl) phenyl) ethyl) thio) propanamide (3e)

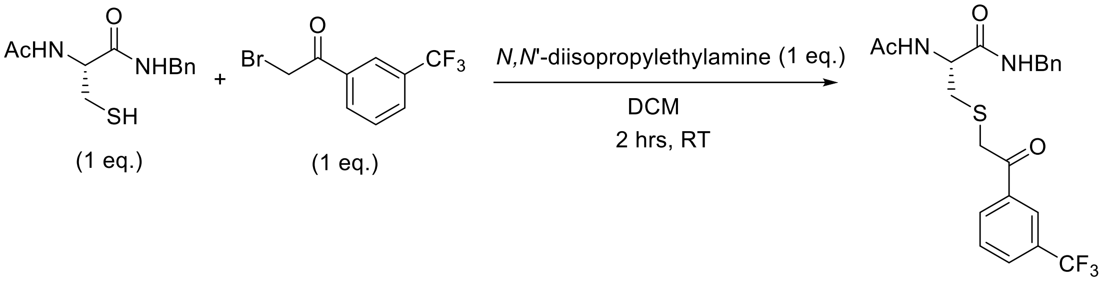

3e (86% yield)

2-bromo-1-(3-(trifluoromethyl)phenyl) ethan-1-one (315 mg, 1.19 mmol) and *N*,*N*-diisopropylethylamine (0.21 mL, 1.19 mmol) were added to a stirred solution of N^2-acetyl-*N*-benzylcysteinamide **1** (300 mg, 1.19 mmol) in DCM (60 mL). The reaction mixture was stirred at room temperature for 2 hrs. The reaction mixture was concentrated *in vacuum*. The residue was purified by silica gel flash column chromatography by using EtOAc/cyclohexane (8:2) as eluent to give **3e** (0.10 g, 60%) as a white solid (m.p. 128–129 °C).

TLC - R_f 0.36 (80:20, EtOAc/pet. ether).

^{1}H NMR (Chloroform-*d*, 500 MHz) δ 8.18 (s, 1H), 8.09 (d, *J* = 7.8 Hz, 1H), 7.82 (d, *J* = 7.8 Hz, 1H), 7.59 (m, 2H), 7.26 (m, 5H), 7.07 (d, *J* = 7.6 Hz, NH), 4.74 (app. q, *J* = 6.9 Hz, 1H), 4.43 (dd, *J* =15.0, 5.8 Hz, 1H), 4.37 (dd, *J* = 15.0, 5.8 Hz, 1H), 4.00 (m, 2H), 2.97 (dd, *J* = 14.1, 6.1 Hz, 1H), 2.85 (dd, *J* = 14.1, 6.9 Hz, 1H), 1.94 ppm (s, 3H).

^{13}C NMR (Chloroform-*d*, 126 MHz) δ 194.1, 170.7, 170.3, 137.8, 135.8, 131.9, 131.4 (q, *J* = 32.5 Hz), 130.1 (q, *J* = 3.7 Hz), 129.6, 128.7, 127.7, 127.5, 125.5 (q, *J* = 3.7 Hz), 123.61 (q, *J* = 271.6 Hz), 52.2, 43.7, 38.3, 35.1, 23.0 ppm.

HRMS (ESI) *m/z* $(M+Na)^+$, $C_{21}H_{21}N_2O_3F_3S$ calcd. 461.1123, observed 461.1132.

(*R*)-2-acetamido-N-benzyl-3-((2-(3,5-bis(trifluoromethyl)phenyl)-2-oxoethyl)thio) propanamide (3f)

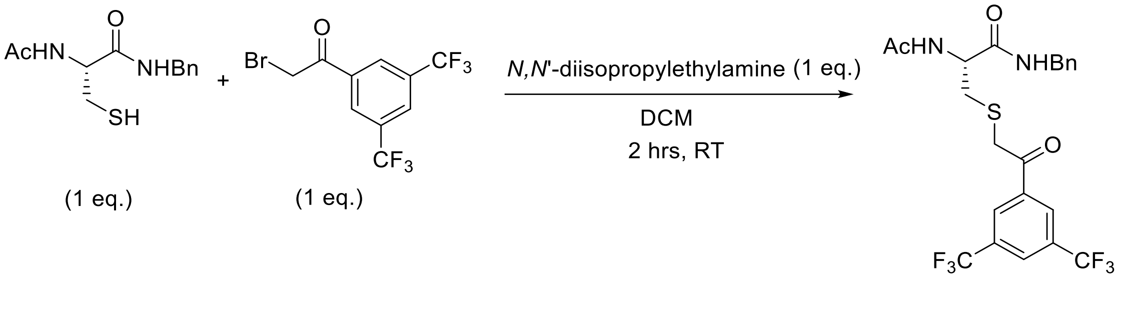

3f (86% yield)

2-bromo-1-(bis-3,5-(trifluoromethyl)phenyl)ethan-1-one (602.27 mg, 1.19 mmol) and *N,N*-diisopropylethylamine (0.21 mL, 1.19 mmol) were added to a stirred solution of N^2-acetyl-*N*-benzylcysteinamide **1** (300 mg, 1.19 mmol) in DCM (60 mL). The reaction mixture was stirred at room temperature for 2 hrs. The mixture was concentrated *in vacuum*. The residue was purified by silica gel flash column chromatography by using EtOAc/cyclohexane (8:2) as eluent to give **3f** (0.125 g, 62%) as a white solid (m.p. 146–147 °C).

TLC - R_f 0.41 (80:20 EtOAc/pet. ether).

^{1}H NMR (Chloroform-*d*, 500 MHz) δ 8.36 (s, 2H), 8.08 (s, 1H), 7.46 (t, *J* = 5.8 Hz, NH), 7.32–7.20 (m, 5H), 6.93 (d, *J* = 7.9 Hz, NH), 4.75 (app. q, *J* = 6.8 Hz, 1H), 4.43 (dd, *J* =14.9, 5.8 Hz, 1H), 4.37 (dd, *J* = 14.9, 5.8 Hz, 1H), 4.03 (m, 2H), 2.96 (dd, *J* = 14.1, 6.0 Hz, 1H), 2.86 (dd, *J* = 14.1, 6.8 Hz, 1H), 1.95 ppm (s, 3H),

^{13}C NMR (Chloroform-*d*, 126 MHz) δ 192.5, 170.7, 170.2, 137.7, 136.9, 132.5 (q, *J* = 33.8 Hz), 128.8, 128.7, 127.7, 127.6, 126.8 (p, *J* = 3.7 Hz), 122.8 (q, *J* = 271.5 Hz), 52.1, 43.9, 38.1, 35.1, 23.1 ppm.

HRMS (ESI) *m*/*z* (*M*+H)$^+$, $C_{22}H_{21}N_2O_3F_6S$ calcd. 507.1177, observed 507.1198.

(*R*)-2-acetamido-*N*-benzyl-3-((2-oxo-2-(perfluorophenyl)ethyl)thio) propanamide (3g)

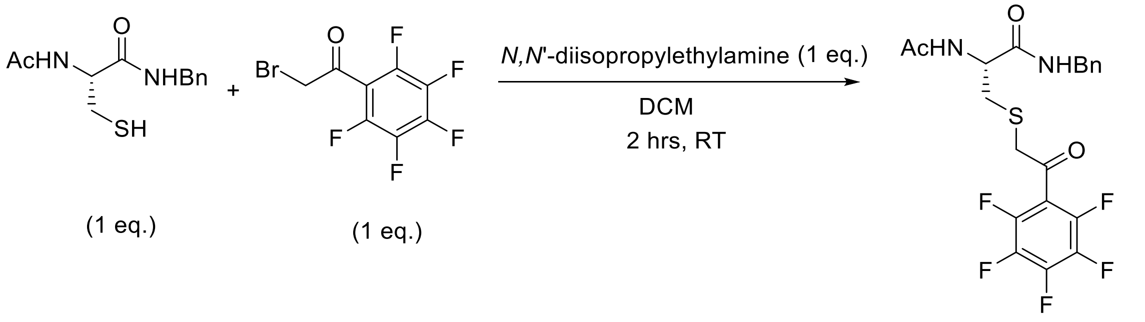

2-bromo-1-(perfluorophenyl)ethan-1-one (171 mg, 0.59 mmol) and *N*,*N*-diisopropylethylamine (0.10 mL, 0.59 mmol) were added to a stirred solution of N^2-acetyl-*N*-benzylcysteinamide **1** (150 mg, 0.59 mmol) in DCM (30 mL) at room temperature. After 2 h, the mixture was concentrated *in vacuum*. The residue was purified by silica gel flash column chromatography by using EtOAc/cyclohexane (8:1) as eluent to give (*R*)-2-acetamido-*N*-benzyl-3-((2-oxo-2-(perfluorophenyl)ethyl)thio) propanamide **3g** (245 mg, 90% yield) as a white/pale yellow solid (m.p. 134–135 °C).

TLC - R_f 0.60 EtOAc/cyclohexane (8:1).

^{1}H NMR (Chloroform-*d*, 500 MHz): δ 7.21–7.36 ppm (m, 5H), 7.01 (t, *J* = 5.7 Hz, NH), 6.61 (d, *J* = 7.8 Hz, NH), 4.61 (app. q, *J* = 6.8 Hz, 1H), 4.44 (dd, *J* = 15.1, 5.7 Hz, 1H), 4.40 (dd, *J* = 15.1, 5.7 Hz, 1H), 3.80 (ABq, $\Delta\delta_{AB}$ = 0.04, J_{AB} = 15 Hz, 2H), 2.91 (dd, *J* = 14.1, 5.9 Hz, 1H), 2.80 (dd, *J* = 14.1, 6.8 Hz, 1H), 1.98 ppm (s, 3H).

^{13}C NMR (Chloroform-*d*, 126 MHz): δ 188.7, 170.5, 169.9, 137.6, 128.9, 127.8, 127.7, 52.1, 43.9, 43.5, 34.8, 23.2 ppm (observed signals).

^{19}F NMR (Chloroform-*d*, 470 MHz): δ –159.5 (m, 2F), –147.74 (m, 1F), –139.80 pm (m, 2F).

HRMS (ESI) *m/z* $(M+H)^+$, $C_{20}H_{18}F_5N_2O_3S$ calcd. 461.0958, observed 461.0975.

IR ν_{max} (neat, cm^{-1}) 3293, 3031, 2938, 1696, 1630, 1494, 1398, 1323, 1237, 1146, 984, 876, 750, 696, 602.

(*R*)-3-(2-((2-acetamido-3-(benzylamino)-3-oxopropyl)thio)acetyl)-*N,N,N*-trimethylbenzenaminium trifluoromethylsulfonate (3h)

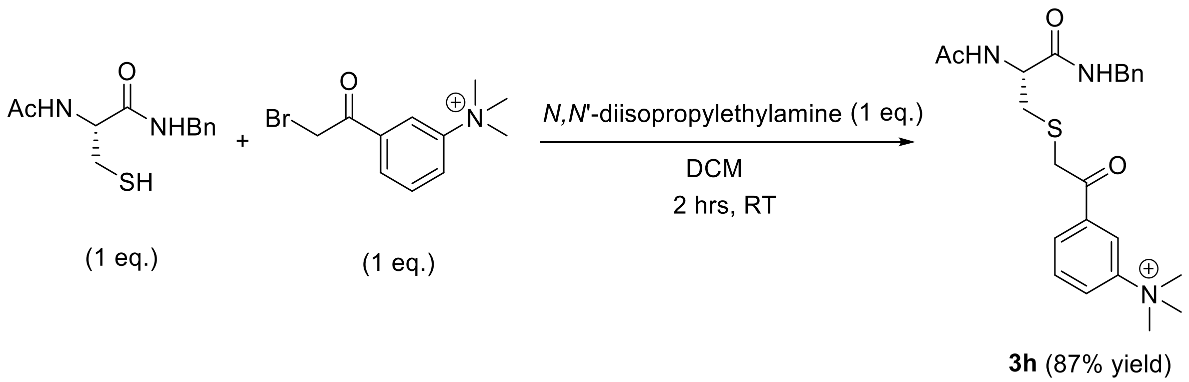

3-(2-bromoacetyl)-*N,N,N*-trimethylbenzenaminium trifluoromethylsulfonate (100 mg, 0.27 mmol) and *N,N*-diisopropylethylamine (0.05 mL, 0.27 mmol) were added to a stirred solution of N^2-acetyl-*N*-benzylcysteinamide **1** (68 mg, 0.27 mmol) in DCM (13 mL) at room temperature. After 4 hours, the white precipitate was separated from the solvent to give (*R*)-3-(2-((2-acetamido-3-(benzylamino)-3-oxopropyl)thio)acetyl)-*N,N,N*-trimethylbenzenaminium trifluoromethylsulfonate **3h** (136 mg, 87% yield), which was used without further purification (m.p. 126–127 ºC).

TLC R_f 0.00 EtOAc/cyclohexane (8:1).

^{1}H NMR (DMSO-d_6, 500 MHz) δ 8.59 (t, J = 6.0 Hz, NH), 8.43 (dd, J = 2.8, 1.4 Hz, 1H), 8.26 (dd, J = 8.3, 2.8 Hz, 1H), 8.22 (d, J = 8.3, Hz, 1H),), 8.17 (d, J = 7.9 Hz, NH), 7.81 (app. t, J = 8.1 Hz, 1H), 7.34 – 7.19 (m, 5H), 4.52 (app. q, J = 6.9 Hz, 1H), 4.30 (dd, J = 15.4, 6.0 Hz, 1H), 4.25 (dd, J = 15.4, 6.0 Hz, 1H), 4.19 (ABq, $\Delta\delta_{AB}$ = 0.03, J_{AB} = 15 Hz, 2H), 3.67 (s, 9H), 2.89 (dd, J = 13.4, 5.9 Hz, 1H), 2.68 (dd, J = 13.4, 8.5 Hz, 1H), 1.86 ppm (s, 3H).

^{13}C NMR (DMSO-d_6, 126 MHz) δ 194.1, 170.6, 169.9, 148.1, 139.6, 137.2, 131.1, 130.7, 128.7, 127.5, 127.2, 125.7, 120.8, 56.9, 52.4, 42.6, 37.9, 34.1, 23.2 ppm (observed signals).

^{19}F NMR (DMSO-d_6, 470 MHz): δ –77.76 ppm (s, 3F).

HRMS (ESI) *m/z* $(M)^+$, $C_{23}H_{30}N_3O_3S$ calcd. 428.2002, observed 428.2019.

IR ν_{max} (neat, cm^{-1}) 3470, 3218, 3064, 2920, 1651, 1555, 1491, 1374, 1302, 1205, 1126, 1029, 958, 879, 808, 682.

(*R*)-4-(2-((2-acetamido-3-(benzylamino)-3-oxopropyl)thio)acetyl)-*N*,*N*,*N*-trimethylbenzenaminium trifluoromethylsulfonate (3i)

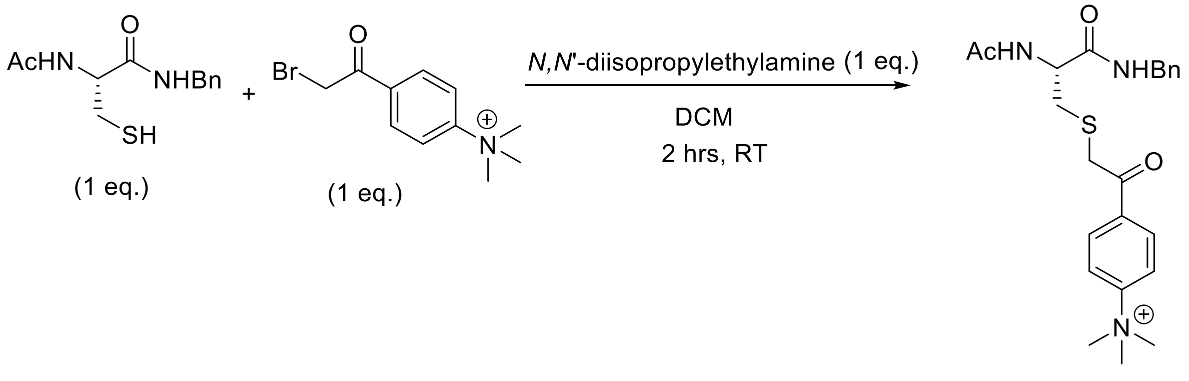

3i (80% yield)

4-(2-bromoacetyl)-*N*,*N*,*N*-trimethylbenzenaminium trifluoromethylsulfonate (217 mg, 0.59 mmol) and *N*,*N*-diisopropylethylamine (0.10 mL, 0.59 mmol) were added to a stirred solution of N^2-acetyl-*N*-benzylcysteinamide **1** (150 mg, 0.59 mmol) in DCM (30 mL) at room temperature. The mixture was allowed to stir overnight. (*R*)-4-(2-((2-acetamido-3-(benzylamino)-3-oxopropyl)thio)acetyl)-*N*,*N*,*N*-trimethylbenzenaminium trifluoromethylsulfonate **3i** (272 mg, 80% yield) separated from the reaction mixture as a yellow oil. The oil was collected and used without further purification.

TLC - R_f 0.00 EtOAc/cyclohexane (8:2).

^{1}H NMR (DMSO-d_6, 500 MHz): δ 8.58 (t, J = 6.1 Hz, NH), 8.21 (d, J = 8.4 Hz, NH), 8.18 (d, J = 9.1 Hz, 2H), 8.13 (d, J = 9.1 Hz, 2H), 7.33–7.20 (m, 5H), 4.51 (app. q, J = 6.8 Hz, 1H), 4.30 (dd, J = 15.5, 6.1 Hz, 1H), 4.25 (dd, J = 15.5, 6.1 Hz, 1H), 4.14 (ABq, $\Delta\delta_{AB}$ = 0.04, J_{AB} = 15 Hz, 2H), 3.64 (s, 9H), 2.86 (dd, J = 13.4, 6.0 Hz, 1H), 2.67 (dd, J = 13.4, 8.3 Hz, 1H), 1.86 ppm (s, 3H).

^{13}C NMR (DMSO-d_6, 126 MHz): δ 193.5, 170.1, 169.4, 150.3, 139.1, 136.2, 130.2, 128.2, 127.1, 126.7, 121.2, 56.4, 51.9, 42.1, 37.5, 33.5, 22.6 ppm (observed signals).

^{19}F NMR (DMSO-d_6, 470 MHz): δ –77.77 ppm (3F, s).

HRMS (ESI) *m/z* $(M)^+$, $C_{23}H_{30}N_3O_3S$ calcd. 428.2002, observed 428.2032.

IR ν_{max} (neat, cm^{-1}) 3295, 3053, 2930, 1650, 1531, 1418, 1254, 1159, 1029, 940, 847, 732, 637.

(*R*)-2-acetamido-*N*-benzyl-3-((2-(3-(dimethylamino)phenyl)-2-oxoethyl)thio) propanamide (3j)

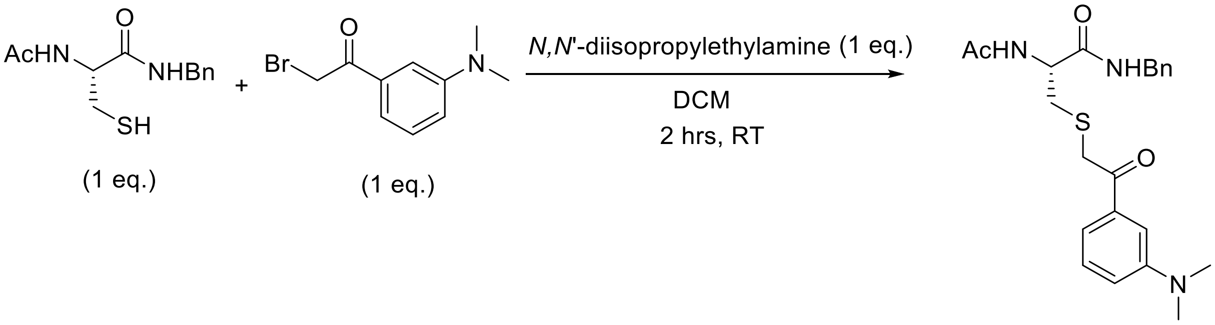

3j (80% yield)

2-bromo-1-(3-(dimethylamino) phenyl) ethan-1-one (143 mg, 0.59 mmol) and *N*,*N*-diisopropylethylamine (0.10 mL, 0.59 mmol) were added to a stirred solution of N^2-acetyl-*N*-benzylcysteinamide **1** (150 mg, 0.59 mmol) in DCM (30 mL) at room temperature. The reaction mixture was stirred for 2 hours. The solvent was then removed *in vacuum*. The residue was purified by silica gel flash column chromatography by using EtOAc/cyclohexane (8:1) as eluent to give (*R*)-2-acetamido-*N*-benzyl-3-((2-(3-(dimethylamino)phenyl)-2-oxoethyl)thio)propanamide **3j** (186 mg, 80% yield) as a yellow solid (m.p. 95–96 °C).

TLC - R_f 0.67 EtOAc/cyclohexane (8:1).

^{1}H NMR (Chloroform-*d*, 500 MHz) δ 7.41 (t, J = 5.9 Hz, NH), 7.33–7.27 (m, 5H), 7.27–7.21 (m, 3H), 6.96 (d, J = 7.2 Hz, NH), 6.93 (dd, J = 8.2, 2.2 Hz, 1H), 4.61 (app. q, J = 6.2 Hz, 1H), 4.47 (dd, J = 15.1, 5.9 Hz, 1H), 4.43 (dd, J = 15.1, 5.9 Hz, 1H), 4.05 (ABq, $\Delta\delta_{AB}$ = 0.03, J_{AB} = 15 Hz, 2H), 3.07 (dd, J = 14.2, 5.6 Hz, 1H), 2.99 (s, 6H), 2.77 (dd, J = 14.2, 7.1 Hz, 1H), 2.02 ppm (s, 3H).

^{13}C NMR (Chloroform-*d*, 126 MHz) δ 196.9, 170.5, 170.2, 150.8, 137.9, 136.2, 129.5, 128.8, 127.7, 127.5, 117.8, 116.9, 111.6, 52.6, 43.8, 40.6, 39.2, 35.4, 23.3 ppm.

HRMS (ESI) *m/z* $(M\text{+H})^+$, $C_{22}H_{28}N_3O_5S$ calcd. 414.1851, observed 414.1862.

IR ν_{max} (neat, cm^{-1}) 3275, 3065, 2920, 2808, 1737, 1633, 1536, 1434, 1358, 1234, 1043, 988, 856, 743, 694.

(*R*)-2-acetamido-*N*-benzyl-3-((2-(4-(dimethylamino)phenyl)-2-oxoethyl)thio) propanamide (3k)

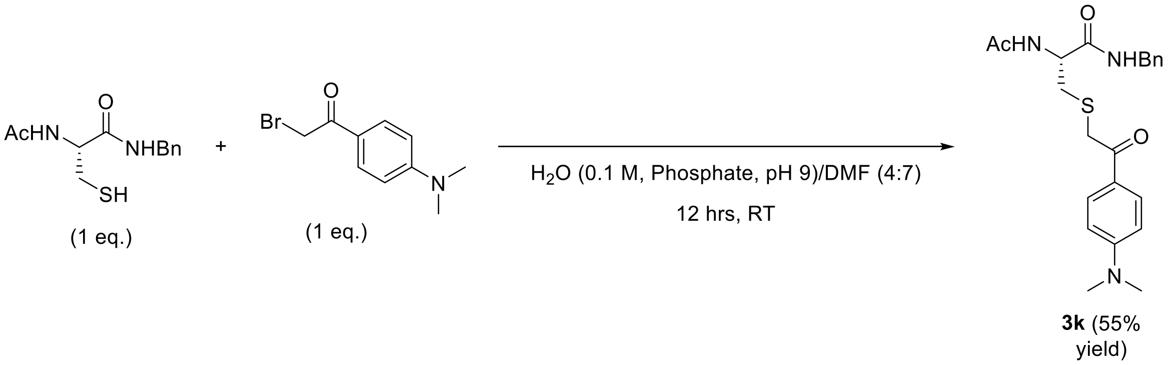

2-bromo-1-(4-(dimethylamino)phenyl)ethan-1-one (53 mg, 0.22 mmol) was added to a stirred solution of N^2-acetyl-*N*-benzylcysteinamide **1** (50 mg, 0.198 mmol) in DMF (1.75 mL) and phosphate buffer (0.1 M, 1 mL, pH 9) at room temperature. The reaction mixture was then allowed to stir overnight. The resulting precipitate was collected and washed with EtOAc and cold water to give (*R*)-2-acetamido-*N*-benzyl-3-((2-(4-(dimethylamino)phenyl)-2-oxoethyl)thio)propanamide **3k** (45 mg, 55% yield) as a white solid, which was used without further purification (m.p. 132–133 °C).

TLC - R_f 0.10 EtOAc/cyclohexane (8:2).

^{1}H NMR (DMSO-d_6, 500 MHz) δ 8.56 (t, J = 6.0 Hz, NH), 8.18 (d, J = 8.3 Hz, NH), 7.80 (d, J = 8.7 Hz, 2H), 7.35–7.15 (m, 5H), 6.71 (d, J = 8.7 Hz, 2H), 4.50 (app. q, J = 8.2 Hz, 1H), 4.28 (dd, J = 16.0, 6.0 Hz, 1H), 4.24 (dd, J = 16.0, 6.0 Hz, 1H), 3.92 (ABq, $\Delta\delta_{AB}$ = 0.03, J_{AB} = 15 Hz, 2H), 3.02 (s, 6H), 2.88 (dd, J = 13.5, 5.9 Hz, 1H), 2.70 (dd, J = 13.5, 8.3 Hz, 1H), 1.86 ppm (s, 3H).

^{13}C NMR (DMSO-d_6, 126 MHz): δ 192.9, 170.7, 169.7, 153.9, 139.6, 131.1, 128.7, 127.5, 127.2, 123.1, 111.1, 52.6, 42.6, 40.1, 37.5, 34.3, 23.0 ppm

HRMS (ESI) *m/z* $(M+H)^+$, $C_{22}H_{28}N_3O_3S$ calcd. 414.1851, observed 414.1857.

IR ν_{max} (neat, cm^{-1}) 3290, 3097, 3051, 2898, 2819, 2654, 1604, 1530, 1429, 1370, 1279, 1185, 1006, 815, 715.

(2*R*)-2-acetamido-N-benzyl-3-((1-oxo-1,2,3,4-tetrahydronaphthalen-2-yl)thio)propanamide (3l)

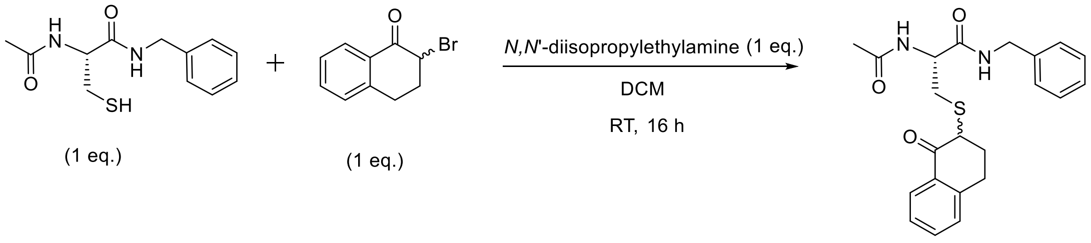

3l

Crude 2-bromo-3,4-dihydronathalen-1(2*H*)-one (166 mg, 0.743 mmol, contains 14% non-brominated compound) and *N,N*-diisopropylethylamine (0.103 mL, 0.594 mmol) were added to a stirred solution of N^2-acetyl-*N*-benzylcysteinamide **1** (150 mg, 0.594 mmol) in DCM (30 mL) at room temperature. The reaction mixture was allowed to stir overnight. The solvent was then removed *in vacuum*. The residue was purified by silica gel flash column chromatography by using EtOAc/cyclohexane (8:1) as eluent to give (*R*)-2-amido-*N*-benzyl-3-((1-oxo-1,2,3,4-tetrahydronaphthalen-2-yl)thio)propanamide **3l** (190 mg, 81%, d.r. = 6:4) as a pale brown solid.

TLC - R_f 0.4 EtOAc/cyclohexane (8:1).

NMR Data for major isomer:
^{1}H NMR (Chloroform-*d*, 500 MHz): δ 8.00 (d, *J* = 7.8 Hz, 1H), 7.53–7.47 (m, 1H, overlapped with minor isomer), 7.39 (t, *J* = 5.0 Hz, NH), 7.36–7.22 (m, 7H), 6.69 (d, *J* = 7.5 Hz, NH), 4.74 (app. q, *J* = 6.7 Hz, 1H), 4.54–4.47 (m, 2H), 3.91 (dd, *J* = 7.2, 4.4 Hz, 1H), 3.22–3.10 (m, 1H), 3.00–2.89 (m, 2H), 2.88–2.81 (m, 1H), 2.59–2.47 (m, 1H), 2.24 (dtd, *J* = 13.8, 6.8, 4.6 Hz, 1H), 2.04 ppm (s, 3H).

^{13}C NMR (Chloroform-*d*, 126 MHz): δ 195.4, 170.5, 170.3, 143.8, 137.9, 134.2, 128.9, 128.8, 128.4, 127.7, 127.5, 127.1, 52.2, 49.8, 43.8, 35.0, 29.7, 27.3, 23.3 ppm.

NMR Data for minor isomer:
^{1}H NMR (Chloroform-*d*, 500 MHz): δ 8.06 (d, *J* = 7.8 Hz, 1H), 7.53–7.46 (m, 1H, overlapped with major isomer), 7.36–7.17 (m, 9H, incl. NH) 4.61 (app. q, *J* = 6 Hz, 1H), 4.49–4.40 (m, 2H), 3.70 (dd, *J* = 6.7, 4.3 Hz, 1H), 3.25 (dd, *J* = 14.4, 5.8 Hz, 1H), 3.22–3.10 (m, 1H, overlapped with major isomer), 3.00–2.89 (m, 1H, overlapped with major isomer), 2.88–2.81 (m, 1H), 2.52–2.47 (m, 1H, overlapped with major isomer), 2.36 (m, 1H), 2.04 ppm (s, 3H).

^{13}C NMR (Chloroform-*d*, 126 MHz): δ 195.2, 170.8, 170.3, 143.6, 138.0, 131.1, 129.0, 128.8, 128.4, 127.8, 127.5, 127.1, 53.9, 49.8, 43.8, 32.6, 29.3, 26.9, 23.3 ppm.

HRMS (ESI) *m/z* $(M+H)^+$, $C_{22}H_{24}N_2O_3S$ calcd. 397.1580, observed 397.160

E. Photolysis of simple cysteine phenacylsulfide derivatives (3a–l) in buffered aqueous solvent

General procedure

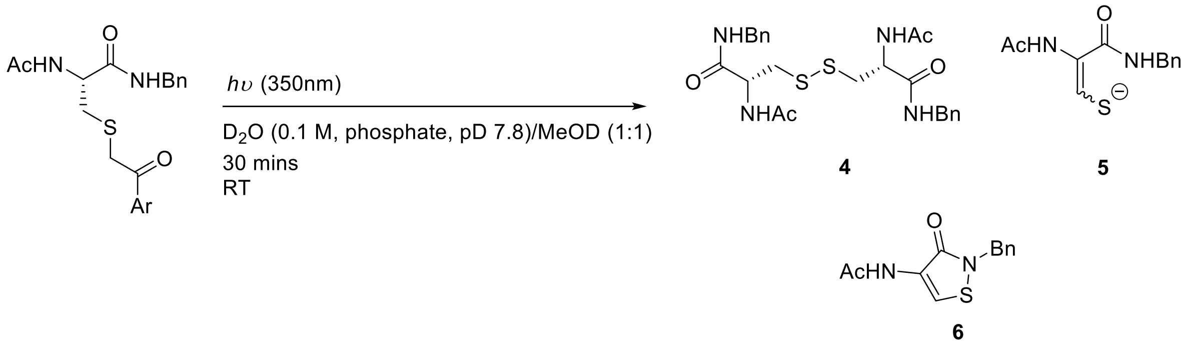

Phenacylsulfide **3a–l** (5.4 µmol) was dissolved in 250 µL of CD_3OD. To this solution 250 µL of D_2O phosphate buffer (0.1 M, pD 7.8) was added. The resulting mixture was then sparged with N_2 for 15 mins and transferred to an NMR tube, which was flushed with N_2 gas prior to sealing. The sealed NMR tube was then photolysed under UV light at 350 nm in a Rayonet photoreactor for 30 minutes. The resulting reaction mixtures were analysed by NMR spectroscopy. Relative conversions (Table E1) were determined through integration of relevant signals (see Figure E1 below for an example of a spectrum).

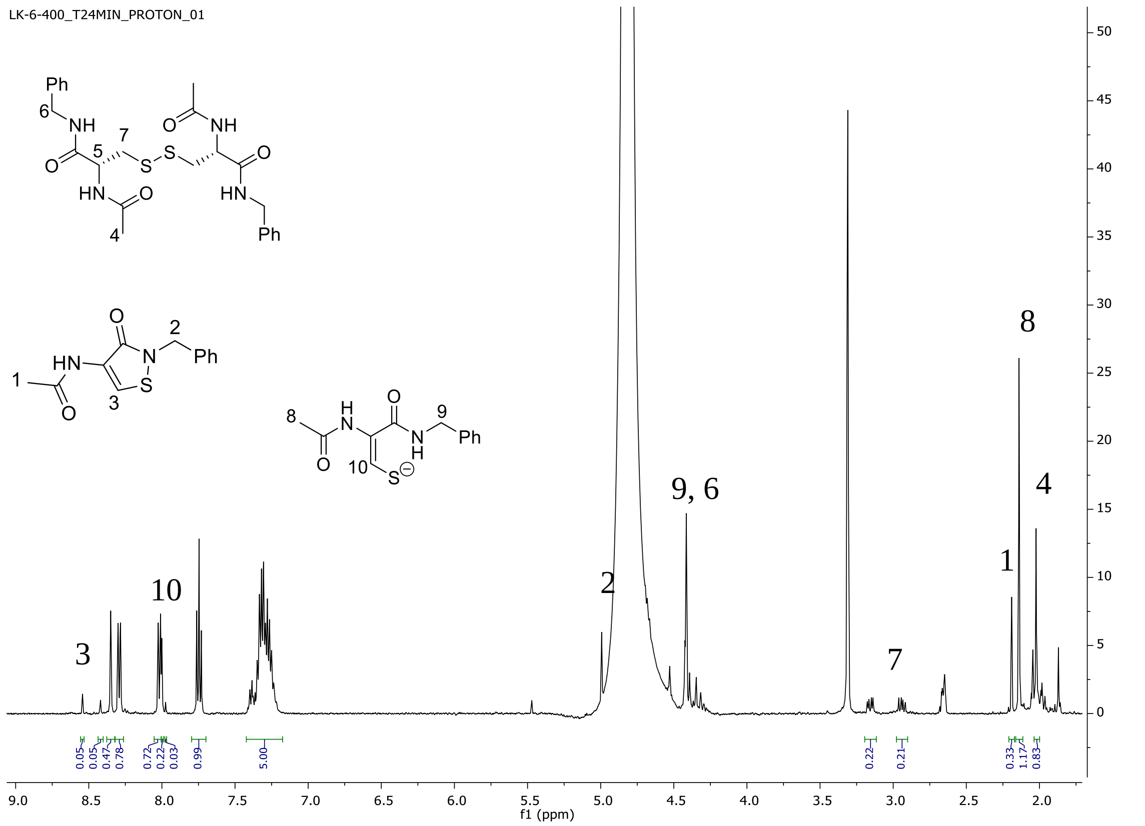

*Figure E1: ^{1}H NMR (500 MHz) spectrum of the reaction mixture derived from the photolysis of phenacylsulfide **3c** in phosphate-buffered D_2O/CD_3OD. Characteristic signals of each reaction product are highlighted. Integration of each signal relative to the aromatic multiplet at 7.5–7.0 ppm allows for conversion values to be determined. Note: The methine signal of disulfide is obscured by the H_2O solvent peak at 4.99 ppm.*

Table E1: Conversion values from photolysis of cysteine phenacylsulfides (3a–l).

Ar		**4** [%]	**5** [%]	**6** [%]
2,4-$(OMe)_2C_6H_3$	**3a**	95	–	<5
C_6H_5	**3b**	57	–	43
3-$(CN)C_6H_4$	**3c**	30	54	16
4-$(CN)C_6H_4$	**3d**	42	44	14
3-$(CF_3)C_6H_4$	**3e**	47	43	10
3,5-$(CF_3)_2C_6H_3$	**3f**	33	61	7
C_6F_5	**3g**	78	–	22
3-$(^{+}NMe_3)C_6H_4$	**3h**	32	58	10
4-$(^{+}NMe_3)C_6H_4$	**3i**	29	65	6
3-$(NMe_2)C_6H_4$	**3j**	–	–	–
4-$(NMe_2)C_6H_4$	**3k**	–	–	–
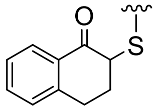	**3l**	70	27	3

Interestingly, in the case of compound **3l**, disulfide **4** was observed with a diastereomeric ratio of 1:1 (Figure E2 and E3). See below for further investigations.

Photolysis of compound 3l

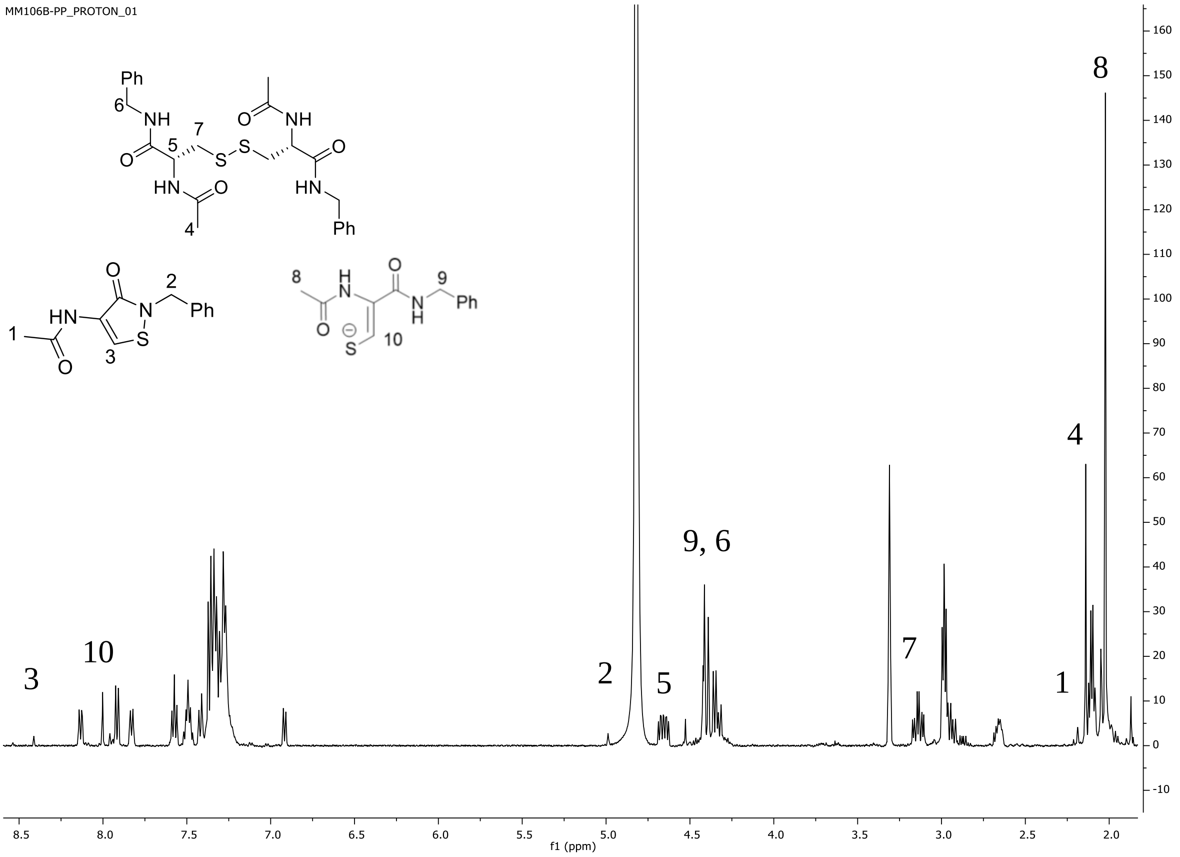

Figure E2: ¹H NMR (500 MHz) spectrum of the reaction mixture following photolysis of ***3l*** *in D_2O (0.1 M phosphate, pD 7.8):MeOD (1:1). Characteristic signals of each of the reaction products are highlighted.*

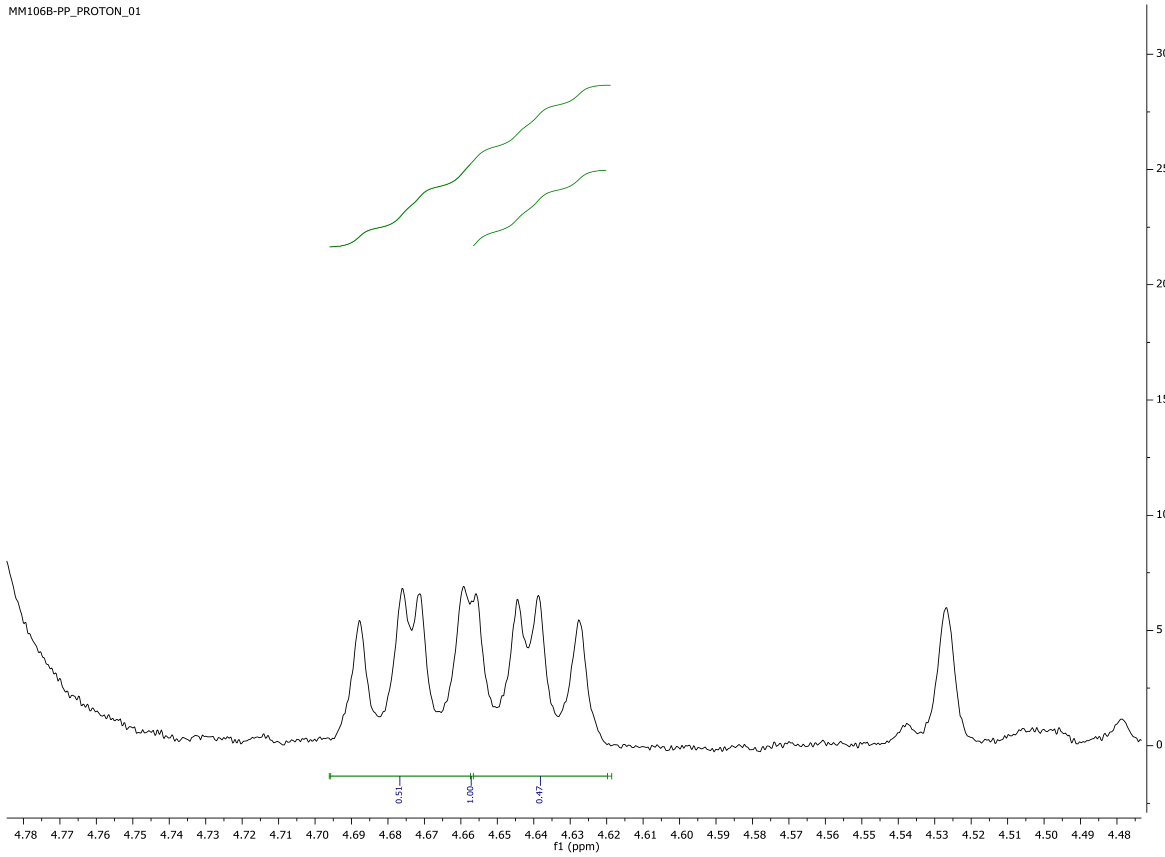

*Figure E3: Zoomed in region of the ^{1}H NMR spectrum of the reaction mixture derived from the photolysis of **3l** in D_2O (0.1 M phosphate, pD 7.8):MeOD (1:1). Highlighted is the signal corresponding to the α proton of the disulfide species noting a 1:1 mixture of diastereomers.*

Photolysis of compound 3l in the presence of DTT followed by DTNB treatment

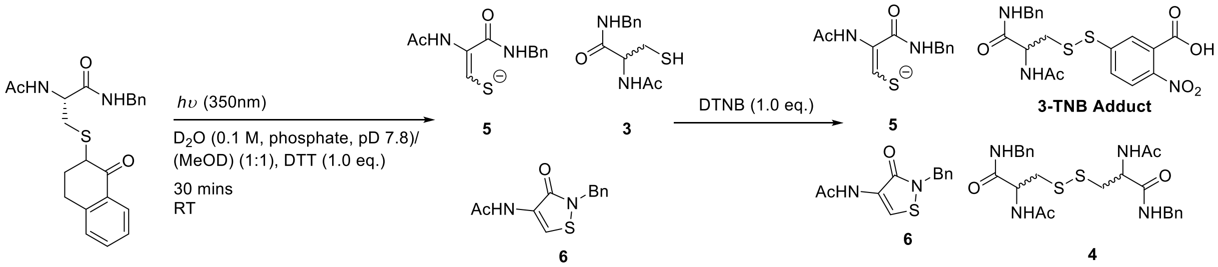

Phenacylsulfide **3l** (10.1 µmol) was dissolved in 500 µL of CD_3OD. To this solution 433 µL of D_2O phosphate buffer (0.1 M, pD 7.8) was added. This was followed by addition of DTT (10.1 µmol, 1.0 eq) from a preneutralised stock in D_2O phosphate buffer (20 mg/mL). The resulting mixture was then sparged with N_2 for 15 mins and transferred to an NMR tube, which was flushed with N_2 gas prior to sealing. The sealed NMR tube was then photolysed under UV light at 350 nm in a Rayonet photoreactor for 30 minutes. The resulting reaction mixture was analysed by NMR spectroscopy. Following initial characterisation, DTNB (10.1 µmol, 1.0 eq) was added from a stock in CD_3OD (40 mg/mL) and the reaction mixture was characterised by NMR spectroscopy to determine if addition of DTT during photolysis prevented racemization of disulfide formed.

Table E2: Conversion values for the photolysis of compound ***3l*** *under standard conditions, in the presence of DTT and following DTNB oxidation.*

	Conversion (%)				
Conditions	Disulfide (**4**)	Enethiol (**5**)	Isothiazolone (**6**)	Thiol **(3)**	TNB–Thiol **(3)** adduct
Standard Conditions (*Taken form Table 1)*	70 (dr = 1:1)	27	3	0	0
In the presence to DTT	0	9	4	87	0
Following DTNB Oxidation	70 (dr = 1:1)	8	5	0	17

Photolysis of compound 3l in the presence of DTT

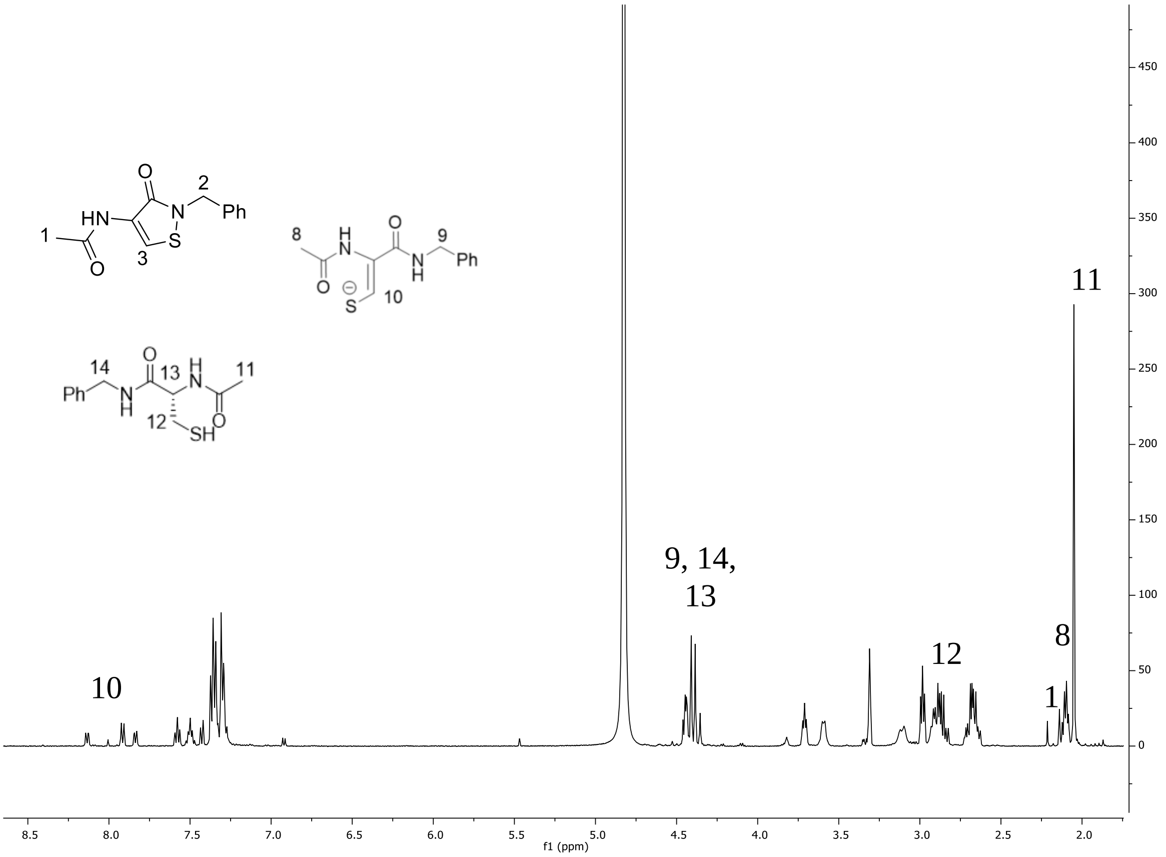

*Figure E5: ^{1}H NMR (500 MHz) spectrum of the reaction mixture following photolysis of **3l** in D_2O (0.1 M phosphate, pD 7.8):MeOD (1:1) in the presence of 1 equivalent of DTT. Characteristic signals of each of the reaction products are highlighted.*

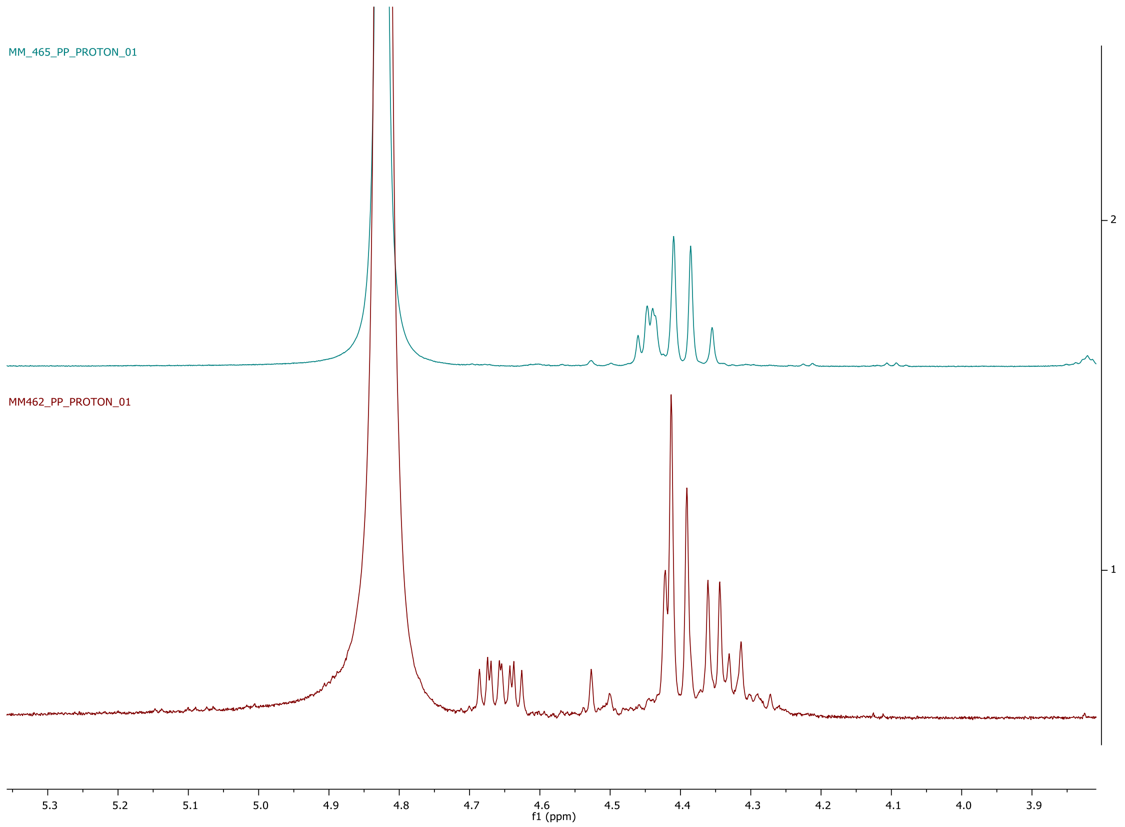

Figure E6: Zoomed in region of the stacked ^{1}H NMR spectrum of the reaction mixture derived from the photolysis of ***3l*** *in D_2O (0.1 M phosphate, pD 7.8):MeOD (1:1) in the absence of DTT (red) and the presence of DTT (Blue). Highlighted is the signal corresponding to the α proton of the disulfide species at 4.65 ppm. Note: in the presence of DTT, the corresponding thiol* ***1*** *is generated in the reaction. The α proton of the thiol resonates at 4.44 ppm (obscured by benzyl methylene signal).*

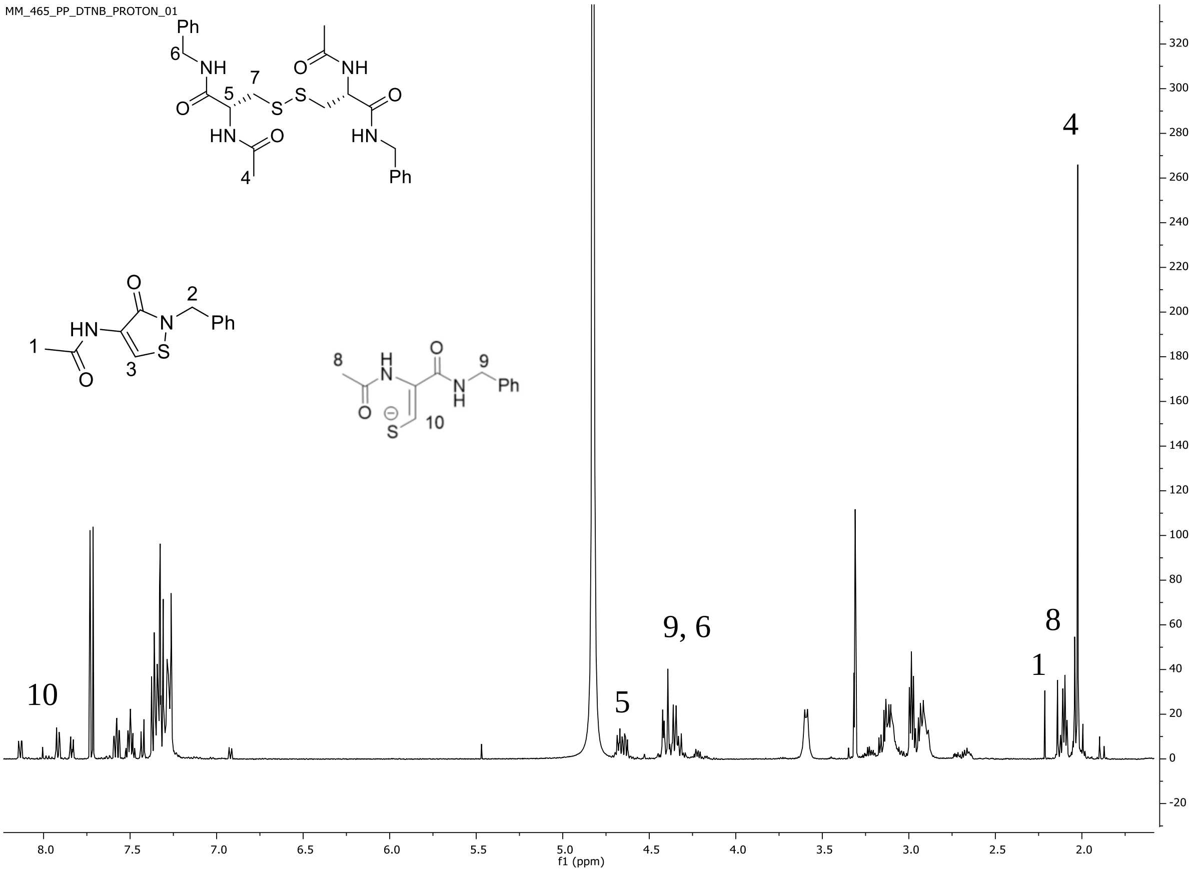

*Figure E7: ^{1}H NMR spectrum of the reaction mixture following photolysis of **3l** in D_2O (0.1 M phosphate, pD 7.8):MeOD (1:1) in the presence of 1 equivalent of DTT, following treatment with 1 equivalent of DTNB. Characteristic signals of each of the reaction products are highlighted. Note the oxidation of the thiol present to the corresponding disulfide.*

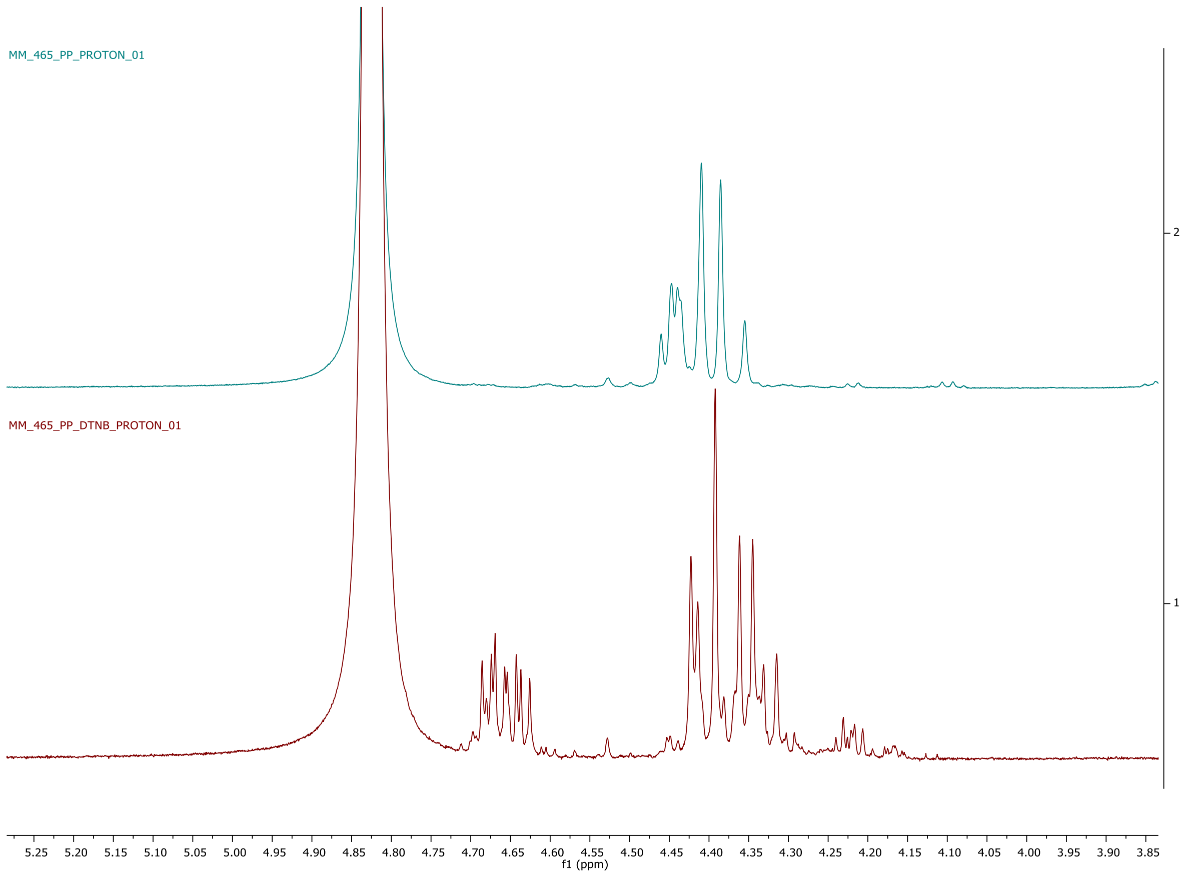

Figure E8: Zoomed in region of the stacked ^{1}H NMR spectrum of the reaction mixture derived from the photolysis of ***3l*** *in D_2O (0.1 M phosphate, pD 7.8):MeOD (1:1) in the presence of DTT, prior to DTNB treatment (blue) and following DTNB treatment (red). Highlighted is the signal corresponding to the α methine proton of the disulfide species in addition to the thiol–TNB adduct at 4.65 ppm.*

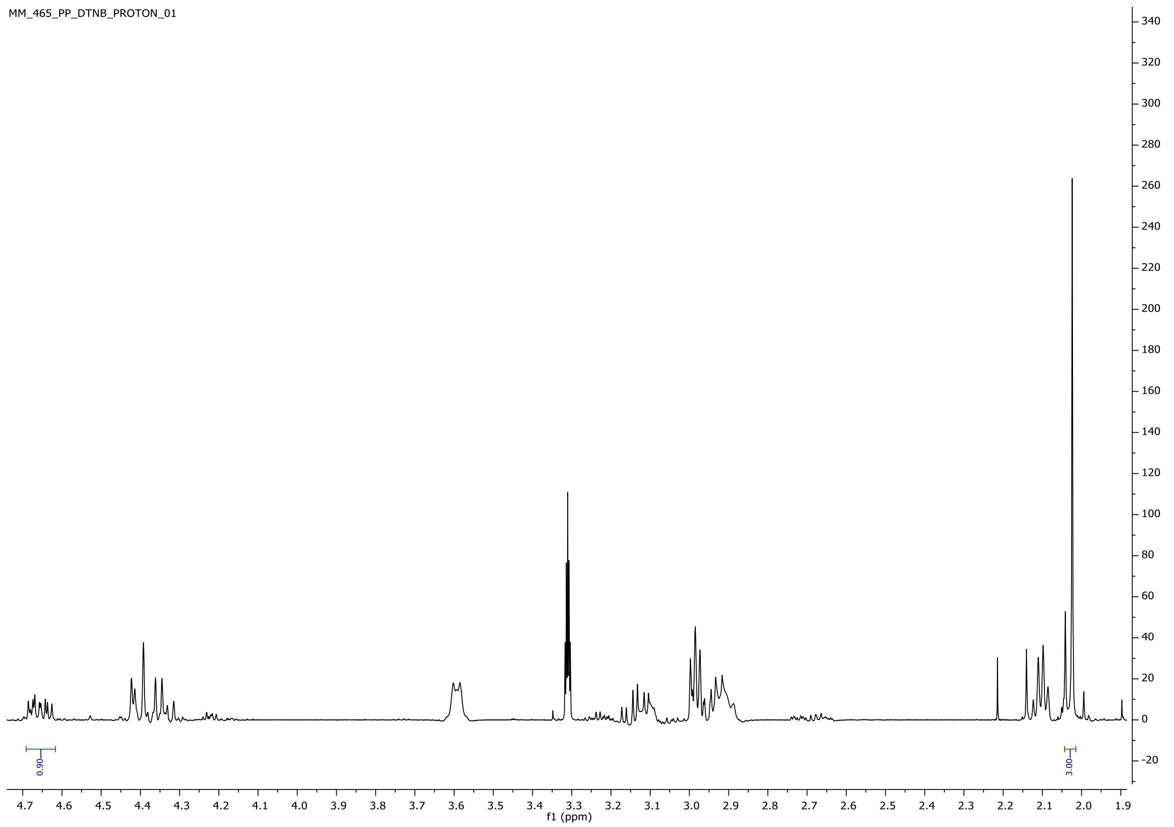

Figure E9: Zoomed in region of the ^{1}H NMR spectrum of the reaction mixture derived from the photolysis of ***3l*** *in D_2O (0.1 M phosphate, pD 7.8):MeOD (1:1). Shown is the integration of the α proton of the diastereomeric mixture of disulfide at 4.66 ppm relative to the acetyl methyl signal of the species at 2.02 ppm exhibiting a 1:3 ratio as expected. No deuterium incorporation is observed at the α centre.*

Photolysis of compound 3b by using different organic co-solvents

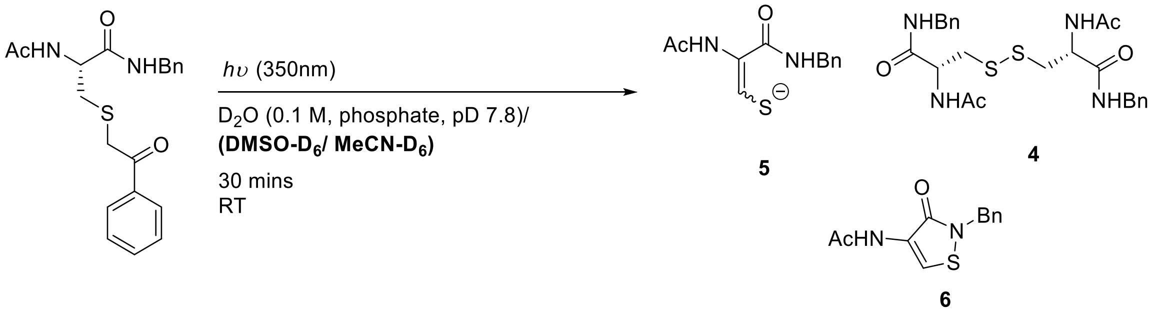

Phenacylsulfide **3b** (5.4 µmol) was photolysed following the general procedure above but using different organic co-solvents. The conversion value of each reaction product was determined by ^{1}H NMR and pre-saturation ^{1}H NMR spectroscopy.

*Table E3: Conversion values for the photolysis of compound **3b** by using different organic co-solvents*

	Conversion (%)		
Solvent	Disulfide (**4**)	Enethiol (**5**)	Isothiazolone (**6**)
D_2O/DMSO-D_6 (2:3)	42 (dr = 2:1)	44	14
D_2O/MeCN-D_3 (1:1)	59	24	17
D_2O/MeOD-D_4 (1:1) *(Taken from Table 1)*	57	-	43

Photolysis of compound 3b with DMSO-D6 co-solvent

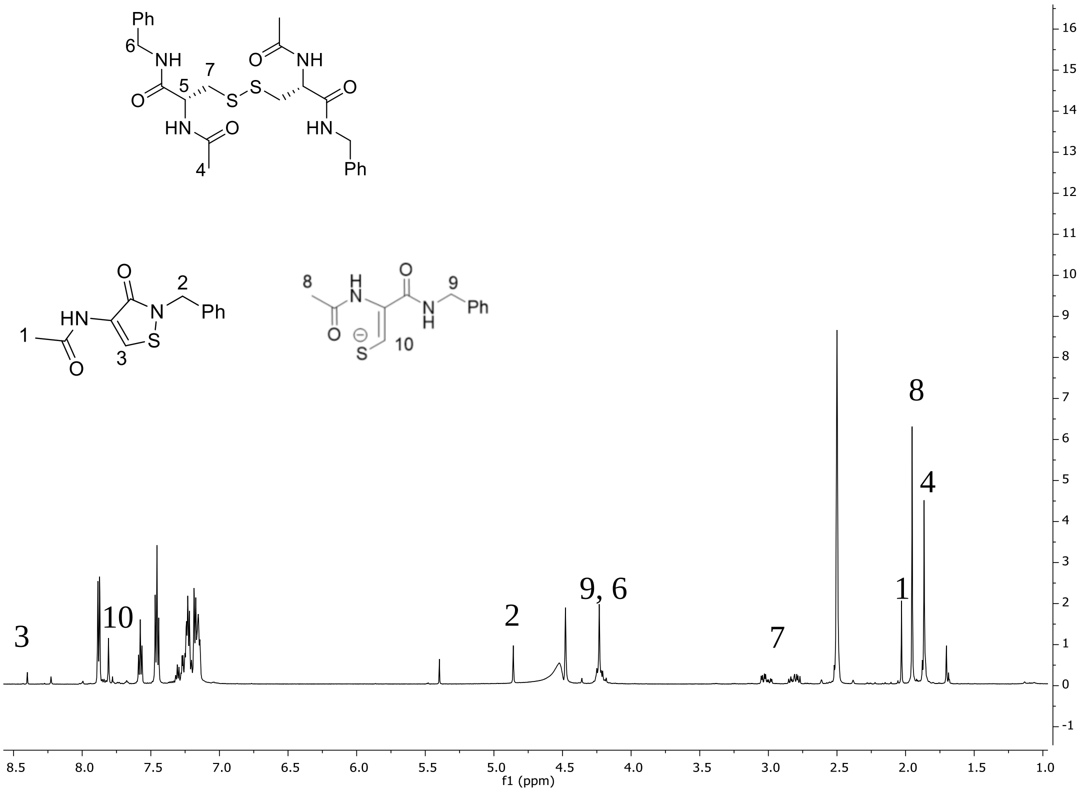

*Figure E10: Presaturated ^{1}H NMR (600 MHz) spectrum of the reaction mixture derived from the photolysis of compound **3b** in D_2O (0.1 M phosphate, pD 7.8): DMSO-D_6 (2:3). Characteristic signals of each of the reaction products are highlighted.*

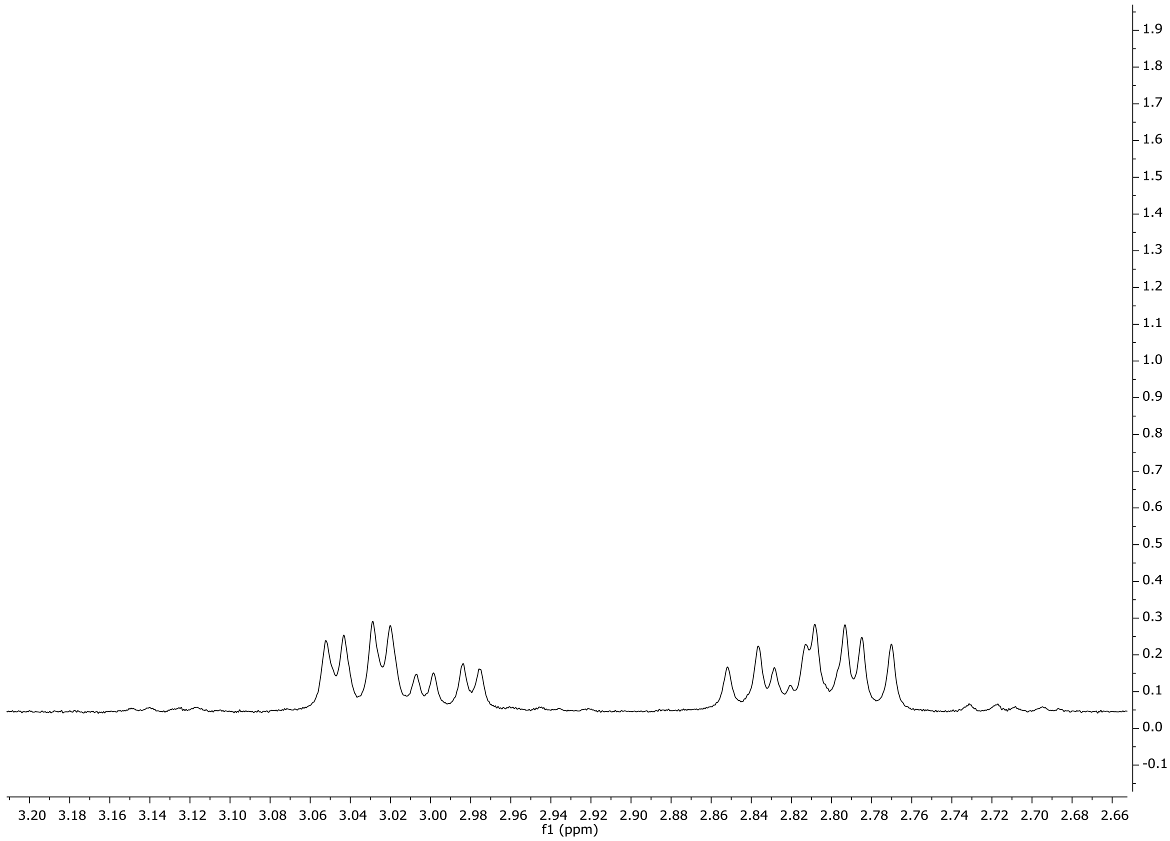

Figure E11: Zoomed in presaturated ^{1}H NMR (600 MHz) spectrum of the reaction mixture derived from the photolysis of compound ***3b*** *in D_2O (0.1 M phosphate, pD 7.8): DMSO-D_6 (2:3) showing the doublets of doublets corresponding to the β protons of diastereomeric disulfide (d.r. ca. 2:1)*

F. Investigation of simple cysteine isothiazolone, enethiol and disulfide derivatives

Synthesis of disulfide (4)

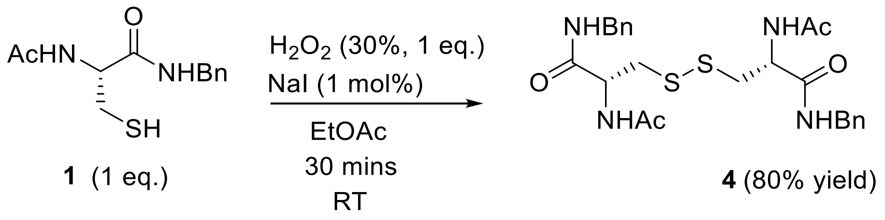

Sodium iodide (0.6 mg, 0.0039 mmol) and 30% H_2O_2 (40 µL, 0.396 mmol) were added to a stirred solution of N^2-acetyl-*N*-benzylcysteinamide **1** (0.100 g, 0.396 mmol) in EtOAc (0.45 mL). The reaction mixture was stirred at room temperature for 30 min. The reaction mixture was diluted with DCM (10 mL) and washed with sat. aq. Na_2CO_3 (2 × 10 mL). The organic extracts were dried with $MgSO_4$. The solvent was then removed *in vacuum* and the resulting residue was purified by silica gel flash column chromatography (80:20 EtOAc/pet ether) to give **4** (0.159 g, 80% yield) as a white solid (m.p. 230– 233 ºC).[157]

TLC - R_f 0.12 (80:20 EtOAc/pet ether).

^{1}H NMR (Chloroform-*d*, 500 MHz): δ 8.54 (t, *J* = 5.8 Hz, NH), 7.36–7.30 (m, 7H), 7.30–7.26 (m, 3H), 6.47 (d, *J* = 8.9 Hz, NH), 5.34–5.39 (ddd, *J* = 10.6, 8.9, 4 Hz, 2H), 4.47 (dd, *J* = 14.7, 5.8 Hz, 2H), 4.41 (dd, *J* = 14.7, 5.8 Hz, 2H), 3.01 (dd, *J* = 14.8, 4 Hz, 2H), 2.89 (dd, *J* = 14.8, 10.6 Hz, 2H), 1.95 ppm (s, 6H).

^{13}C NMR (Chloroform-*d*, 125 MHz): δ 170.3, 170.0, 137.9, 128.6, 127.9, 127.5, 53.4, 46.6, 43.7, 23.3 ppm.

HRMS (ESI) *m/z* $(M\text{+H})^+$, $C_{24}H_{31}N_4O_4S_2$ calcd. 503.1787, observed 503.1790; $(M\text{+Na})^+$, $C_{24}H_{31}N_4O_4S_2Na$ calcd. 525.1606, observed 525.1612.

Isolation of 1,4-bis(2,4-dimethoxyphenyl)butane-1,4-dione (8a) from photolysis reaction mixture of 3a

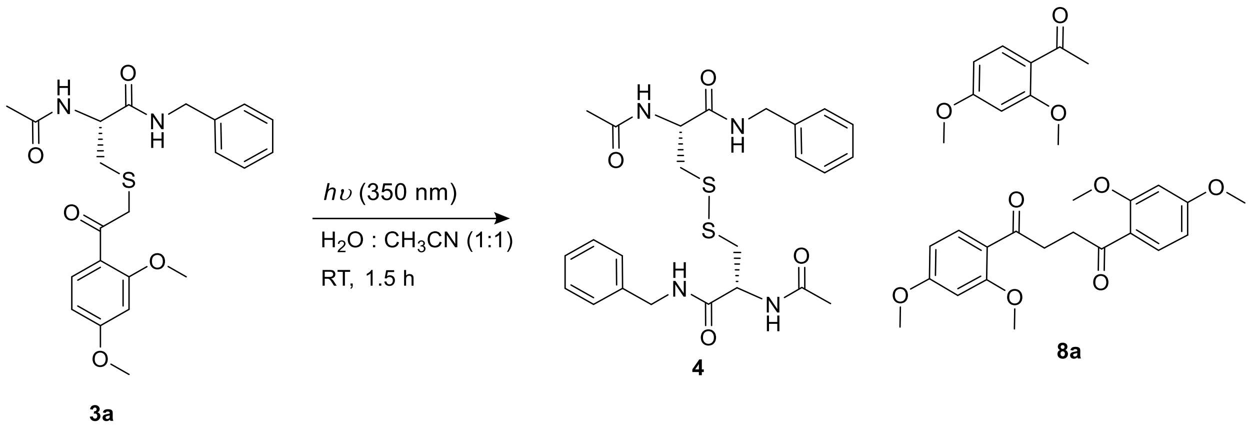

2-bromo-1-(2,4-dimethoxyphenyl)ethan-1-one **3a** (100 mg, 232 µmol) was dissolved in H_2O/CH_3CN (1:1, 42 mL) in Pyrex round-bottomed flask. The reaction mixture was then sparged with N_2 for 30 minutes. The mixture was photolysed at 350 nm for 90 minutes within a Rayonet photoreactor. The mixture was diluted with EtOAc, separated and the organics dried over $MgSO_4$ and concentrated *in vacuum*. The residue was purified by silica gel flash column chromatography to give 1,4-bis(2,4-dimethoxyphenyl)butane 1,4-dione **8a** (5.4 mg, 13% yield) as an off-white/ yellow solid. Spectroscopic data in accordance to literature.[158]

TLC: R_f 0.54 EtOAc/cyclohexane, (1:1).

^{1}H NMR Chloroform-*d*, (400 MHz): δ 7.85 (d, J = 8.7 Hz, 2H), 6.52 (dd, J = 8.7, 2.2 Hz, 2H), 6.46 (d, J = 2.2 Hz, 2H), 3.89 (s, 6H), 3.85 (s, 6H), 3.35 ppm (s, 4H).

HRMS (ESI) *m/z* (M+H)$^+$, $C_{20}H_{22}O_6$ calcd. 359.149, observed 359.148.

Isolation and characterisation of N-(2-benzyl-3-oxo-2,3-dihydroisothiazol-4-yl)acetamide (6)

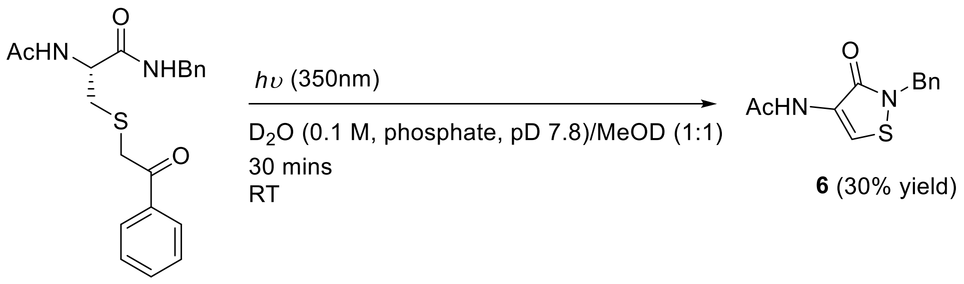

3b

N^2-acetyl-*N*-benzyl-*S*-(2-oxo-2-phenylethyl) cysteinamide **3b** (0.150 g, 0.39 mmol) was dissolved in 1:1 MeOH/D_2O (0.1 M phosphate, pD 7.8) (62 mL). The solution was degassed with N_2 for 30 minutes and irradiated with UV light (350 nm) within a Rayonet photoreactor for 2 hours. The reaction mixture was extracted into DCM (3 x 10 mL) and the combined organic layers were dried over $MgSO_4$ and then concentrated *in vacuum*. The residue was purified by flash column chromatography (80:20 EtOAc/cyclohexane) to give *N*-(2-benzyl-3-oxo-2, 3-dihydroisothiazol-4-yl) acetamide **6** (30 mg, 30 % yield) as a white solid (m.p. 216–218 °C).

TLC - R_f 0.50 EtOAc/Pet ether (8:2).

^{1}H NMR (Methanol-d_4/D_2O, 1:1, 500 MHz): δ 8.54 (s, 1H), 7.38–7.42 (m, 3H), 7.31–7.33 (m, 2H), 5.00 (s, 2H), 2.19 ppm (s, 3H).

^{1}H NMR (Chloroform-*d*, 500 MHz): δ 8.42 (s, 1H), 7.87 (br. s, NH), 7.27–7.35 (m, 5H), 4.97 (s, 2H), 2.19 ppm (s, 3H).

^{13}C NMR (Chloroform-*d*, 125 MHz): δ 168.3, 162.7, 135.4, 128.9, 128.5, 128.3, 120.5, 118.4, 48.1, 23.6 ppm.

HRMS (ESI) *m/z* $(M{+}H)^+$, $C_{12}H_{13}N_2O_2S$ calcd. 249.0698, observed 249.0654.

IR ν_{max} (neat, cm^{-1}) 3238, 3192, 3123, 3034, 2919, 2850, 1728, 1684, 1621, 1581, 1537, 1495, 1374, 1349, 1239, 1149, 1031, 1006.

X-ray data: see Section L

Reduction of isothiazolone (6) to give enethiolate (5) with subsequent *S*-methylation.

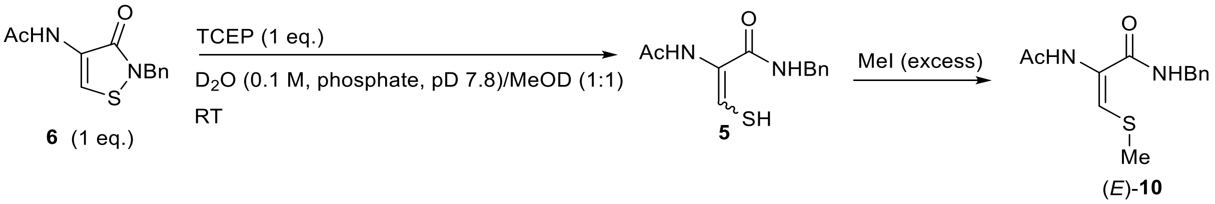

N-(2-benzyl-3-oxo-2,3-dihydro-1,2-thizol-4-yl)acetamide **6** (1 mg, 0.004 mmol) was dissolved in 1:1 MeOD/D_2O (0.1 M phosphate, pD 7.8) (0.25 mL). Then, a solution of TCEP (0.064 M) in phosphate buffer (63 µL, 0.004 mmol) was added to successfully generate enethiolate **5** as detected by NMR spectroscopy. An excess of methyl iodide (3 µL, 0.028 mmol) was added to afford the methyl vinyl sulfide **10**.

NMR data of *E*-enethiolate (5):

^{1}H NMR (Methanol-d_4/D_2O, 1:1, 500 MHz): δ 7.90 (s, 1H), 7.39- 7.30 (m, 4H), 7.26 (m, 1H), 4.49 (s, 2H), 2.01 ppm (s, 3H).

^{13}C NMR (Methanol-d_4/D_2O, 1:1, 125 MHz): δ 178.8, 168.3, 153.7, 139.5, 129.5, 128.3, 128.1, 123.8, 43.9, 22.9 ppm.

NMR data of *Z*-enethiolate (5):

^{1}H NMR (Methanol-d_4/D_2O, 1:1, 500 MHz): δ 7.98 (s, 1H), 7.39- 7.30 (m, 4H), 7.26 (m, 1H), 4.51 (s, 2H), 2.15 ppm (s, 3H).

^{13}C NMR (Methanol-d_4/D_2O, 1:1, 125 MHz): δ 178.8, 168.3, 158.2, 139.5, 129.5, 128.4, 128.1, 123.8, 43.9, 22.9 ppm.

NMR data of *E*-methyl vinyl sulfide (10):

^{1}H NMR (Methanol-d_4/D_2O, 1:1, 500 MHz): δ 7.23–7.36 ppm (m, 5H), 6.97 (s, 1H), 4.43 (s, 2H), 2.35 (s, 3H), 2.09 ppm (s, 3H).

^{13}C NMR (Methanol-d_4/D_2O, 1:1, 125 MHz): δ 174.81, 166.91, 145.0, 139.6, 124.90, 129.4, 128.1, 128.0, 43.7, 22.9, 19.4 ppm.

HRMS (ESI) *m/z* $(M+H)^+$, $C_{13}H_{17}N_2O_2S$ calcd. 265.1004, observed 265.1011.

Stability of enethiolate at pD 10

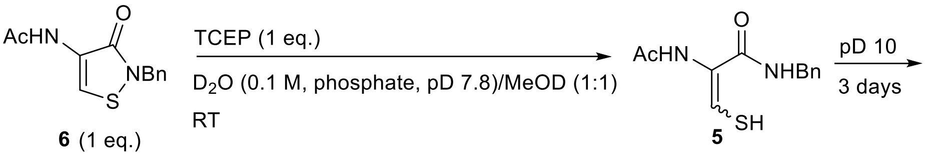

N-(2-benzyl-3-oxo-2,3-dihydro-1,2-thizol-4-yl)acetamide **6** (1 mg, 0.004 mmol) was dissolved in 1:1 MeOD/D_2O (0.1 M phosphate, pD 7.8) (0.25 mL). Then, a solution of TCEP (0.064 M) in phosphate buffer (63 µL, 0.004 mmol) was added to successfully generate enethiolate **5** as detected by ^{1}H NMR spectroscopy. The pD of this solution was then adjusted to 10 followed by analysing the evolution of enethiolate over a duration of 3 days. ^{1}H NMR spectroscopy of the reaction mixture showed that the enethiolate is stable at pD 10.

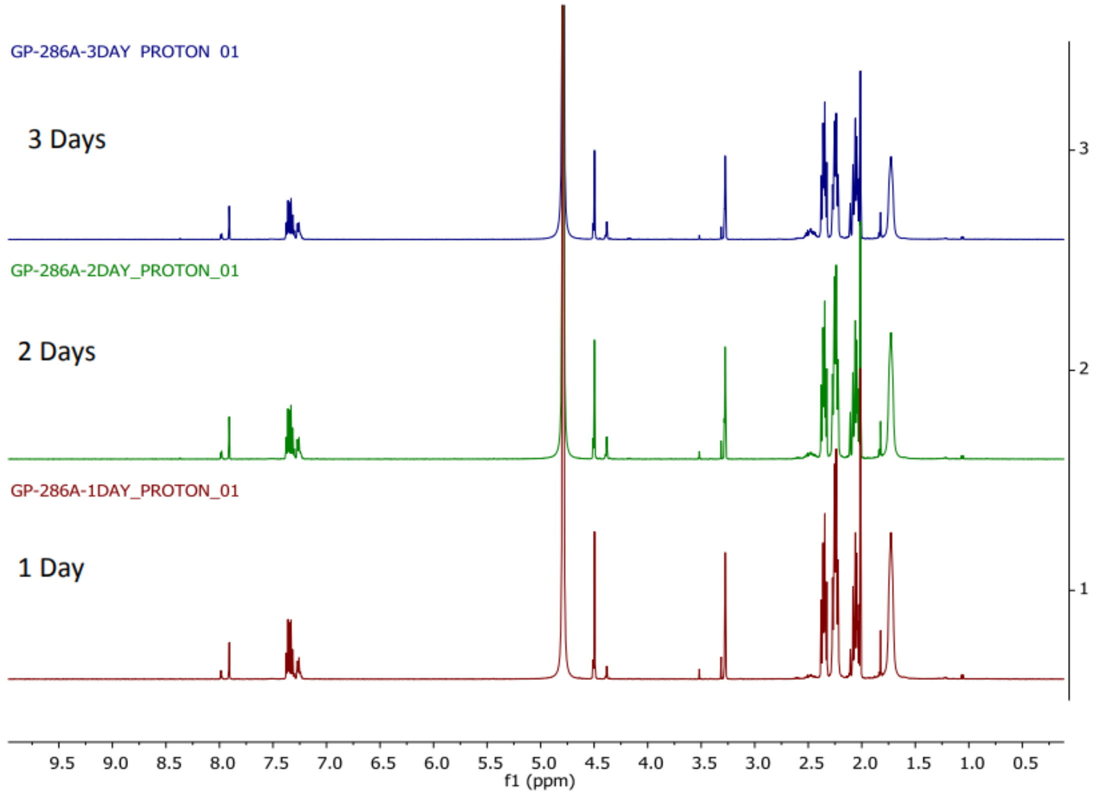

*Figure F1: ^{1}H NMR spectrum of enethiolate **5** in MeOD:D_2O (0.1 M phosphate, pD 10) (1:1) over a duration of 3 days showing the stability of enethiolate **5** at higher pD values.*

Oxidation of enethiolate (5) with DTNB to give isothiazolone (6)

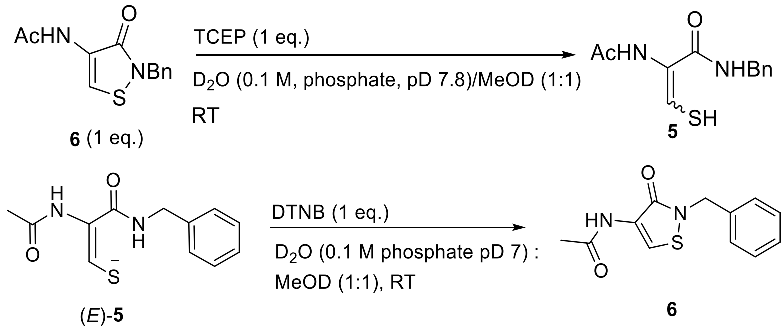

N-(2-benzyl-3-oxo-2,3-dihydro-1,2-thizol-4-yl)acetamide **6** (1 mg, 0.004 mmol) was dissolved in 1:1 MeOD/D_2O (0.1 M phosphate, pD 7.8, 0.5 mL). Then, a solution of TCEP (0.064 M) in phosphate buffer (63 µL, 0.004 mmol) was added to generate enethiolate **5**. DTNB (100 µL, 0.004 mmol) was added to solution. The (*E*) isomer of enethiolate **5**, regenerated isothiazolone **6**.

^{1}H NMR (Methanol-d_4/D_2O, 1:1, 500 MHz): δ 8.54 (s, 1H), 7.27–7.43 (m, 5H), 5.00 (s, 2H), 2.19 ppm (s, 3H).

Oxidation of enethiolate (5) with KI/I_2 to give isothiazolone (6) and isothiazolone S-oxide

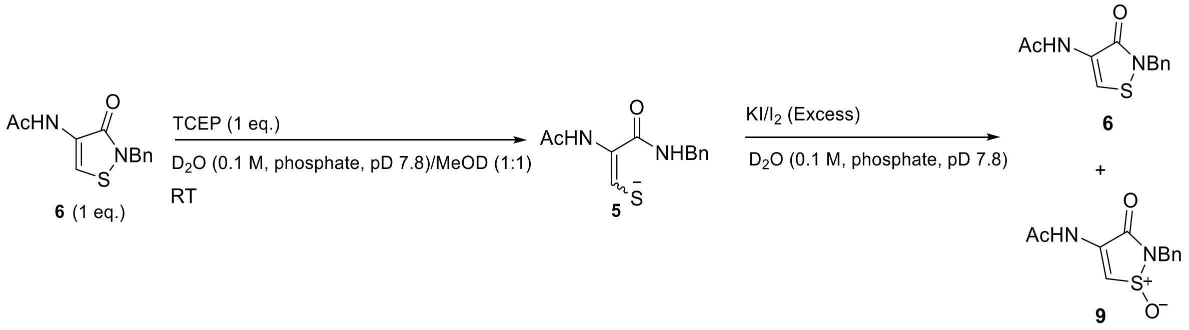

N-(2-benzyl-3-oxo-2,3-dihydro-1,2-thizol-4-yl) acetamide **6** (2 mg, 0.008 mmol) was dissolved in a solution of D_2O (0.1 M phosphate, pD 7.8)/MeOD (1:1). Then, a pre-neutralised solution of TCEP in D_2O (0.064 M, 125 µL, 0.008 mmol) was added. The reaction mixture was analysed by ^{1}H NMR spectroscopy to confirm complete reduction of the starting material to the corresponding enethiolate **5**. To this reaction mixture, 20 µL of a solution of KI/I_2 (241 µmol/50 µmol) in 0.7 mL D_2O (0.1 M, phosphate pD 7.8) was added forming a mixture of isothiazolone **6** and chiral isothiazolone S-oxide **9** with a ratio of 1.9:1.

NMR data for isothiazolone S-oxide

^{1}H NMR (Methanol-d_4/D_2O, 1:1, 500 MHz): δ 7.74 (s, 1H), 7.29–7.42 (m, 5H), 5.06 (d, J = 15.5 Hz, 1H), 4.76 (d, J =15.5 Hz, 1H), 2.25 ppm (s, 3H).

Isolation and characterisation of *N*-benzyl-2-methylthiazole-4-carboxamide (18)

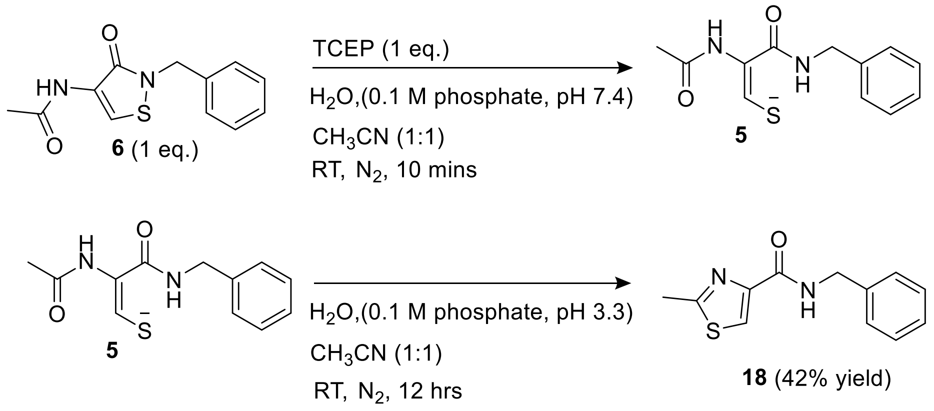

N-(2-benzyl-3-oxo-2,3-dihydro-1,2-thizol-4-yl) acetamide **6** (25 mg, 0.102 mol) was dissolved in 1:1 CH_3CN/H_2O (0.1M phosphate, pH 7.4) (5 mL) under N_2. To this mixture, TCEP (98 µL, 1 eq.) was added from a pre-neutralised stock (1M). The reaction mixture was allowed to stir for 1 hour to ensure complete reduction of the isothiazolone, as confirmed by TLC. The pH of the sample was then adjusted to pH 3.3 through addition of 2 M aq. HCl solution (0.15 mL). The reaction mixture was then allowed to stir overnight under N_2. The solvent was removed *in vacuum*. The residue was purified by silica gel flash column chromatography by using EtOAc/cyclohexane (4:1) as an eluent to give *N*-benzyl-2-methylthiazole-4-carboxamide (**18**) as a white solid (10 mg, 42 % yield).

TLC - R_f 0.3 EtOAc/cyclohexane (4:1).

^{1}H NMR (Chloroform-*d*, 600 MHz): δ 7.98 (s, 1H), 7.64 (br. s, NH), 7.40–7.27 (m, 5H), 4.63 (d, J = 6.1 Hz, 2H), 2.68 ppm (s, 3H).

^{13}C NMR (Chloroform-*d*, 151 MHz): δ 166.2, 161.2, 149.7, 138.3, 128.9, 128.1, 127.6, 123.3, 43.5, 19.2 ppm.

HRMS (ESI) *m/z* (M+H)$^+$, $C_{12}H_{12}N_2OS$ calculated: 233.074, observed: 233.076

G. Synthesis and hydrolysis of a formylglycine diethylacetal derivative

Synthesis of (*S*)-2-acetamido-3,3-diethoxypropanoic acid

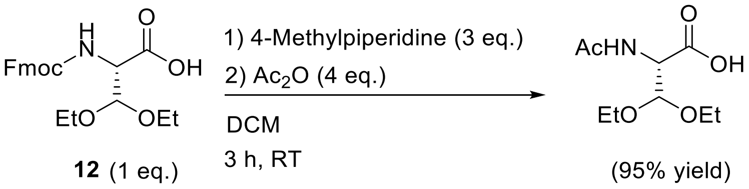

(*S*)-2-((((9H-fluoren-9-yl)methoxy)carbonyl)amino)-3,3-diethoxypropanoic acid **12** (600 mg, 1.5 mmol), which was prepared according to the protocol of Bertozzi [103], was dissolved in DCM (2 mL) with 4-methylpiperidine (0.53 mL, 4.5 mmol). After 20 min, the solvent was removed *in vacuum*. The residue was resuspended in DCM (2 mL) and acetic anhydride (0.57 mL, 6 mmol) was added. After completion of the reaction, as determined by TLC, the mixture was concentrated *in vacuum*. The residue was purified by silica gel flash column chromatography by using EtOAc as eluent to give (*S*)-2-acetamido-3,3-diethoxypropanoic acid as a white solid (312 mg, 95% yield).

TLC - R_f 0.40 EtOAc/MeOH (5:2).

^{1}H NMR (Chloroform-*d*, 500 MHz): δ 6.41 (d, J = 8.1 Hz, NH), 4.85 (d, J = 2.7 Hz, 1H), 4.80 (dd, J = 8.1, 2.7 Hz, 1H), 3.81 (dq, J = 9.4, 7.1 Hz, 1H), 3.67 (dq, J = 9.4, 7.0 Hz, 1H), 3.56–3.63 (m, 2H), 2.08 (s, 3H), 1.21 ppm (t, J = 7.0 Hz, 3H), 1.20 ppm (t, J = 7.0 Hz, 3H).

^{13}C NMR (Chloroform-*d*, 125 MHz): δ 175.9, 171.4, 100.9, 64.5, 64.1, 54.8, 22.9, 15.0, 14.8 ppm.

Synthesis of (*S*)-2-acetamido-*N*-benzyl-3,3-diethoxypropanamide (13)

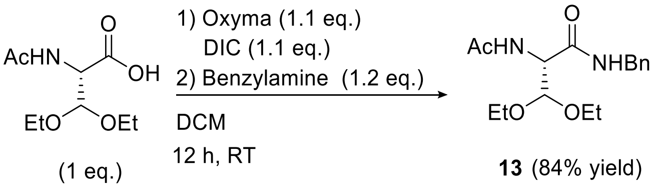

Ethyl 2-cyano-2-(hydroxyimino)acetate (234 mg, 1.65 mmol) and *N,N′*-diisopropylcarbodiimide (0.26 mL, 1.65 mmol) were added to a stirred solution of (*S*)-2-acetamido-3,3-diethoxypropanoic acid (328 mg, 1.5 mmol) in DCM (20 mL). Benzyl amine (0.2 mL, 1.8 mmol) was then added to the stirred mixture. After completion of the reaction, as determined by TLC analysis, the solvent was removed *in vacuum.* The residue was purified by silica gel flash column chromatography by using EtOAc/cyclohexane (8:2) as eluent to give amide **13** (390 mg, 84% yield) as a white solid (m.p. 145–147 °C).

TLC - R_f 0.41 EtOAc/MeOH (5:2).

^{1}H NMR (Chloroform-*d*, 500 MHz): δ 7.23–7.35 (m, 5H), 6.99 (t, J = 4.7 Hz, NH), 6.70 (d, J = 6.5 Hz, NH), 4.80 (d, J = 2.8 Hz, 1H), 4.60–4.57 (dd, J = 6.5, 2.9 Hz, 1H), 4.57 (dd, J = 15.5, 4.7 Hz, 1H), 4.43 (dd, J = 15.5, 4.7 Hz, 1H), 3.81–3.68 (m, 2H), 3.64 (dq, J = 9.5, 7.0 Hz, 1H), 3.53 (dq, J = 9.5, 7.0 Hz, 1H), 2.08 (s, 3H), 1.20 ppm (t, J = 7.0 Hz, 3H), 1.17 ppm (t, J = 7.0 Hz, 3H). .

^{13}C NMR (Chloroform-*d*, 125 MHz): δ 171.1, 168.1, 137.8, 128.5, 127.4, 127.3, 101.7, 65.1, 64.3, 55.83, 43.5, 23.1, 15.2, 15.1 ppm.

HRMS (ESI) *m/z* (2*M*+Na)$^+$, $C_{32}H_{48}N_4NaO_8$ calcd. 639.3364, observed 639.3365.

IR ν_{max} (neat, cm^{-1}) 3283, 3101, 2977, 2929, 1728, 1635, 1548, 1430, 1371, 1299, 1066, 846, 697.

2-acetamido-N-benzyl-3,3-dihydroxypropanamide (15)

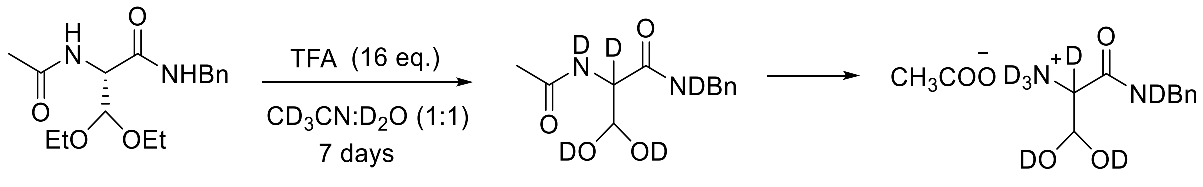

13 (1 eq.) **14** **15**

(*S*)-2-acetamido-*N*-benzyl-3,3-diethoxypropanamide **13** (5 mg, 0.015 mmol) was suspended in D_2O (0.25 mL): CD_3CN (0.25mL) and TFA (20 µL, 0.24 mmol) was added. The reaction was then monitored over a course of 7 days via ^{1}H NMR. ^{1}H NMR analysis of the reaction mixture showed the formation of 2-acetamido-N-benzyl-3,3-dihydroxypropanamide **14** (32 % conversion (from NMR spectra)) and the appearance of a new species 1-(benzylamino)-3,3-dihydroxy-1-oxopropan-2-aminiumacetate **15** (61 % conversion (from NMR spectra)) at the expense of 2-acetamido-*N*-benzyl-3,3-dihydroxypropanamide **14**.

^{1}H NMR (CD_3CN-D_3: D_2O (1:1), 500 MHz, **14**): δ 7.18–7.34 (m, 5H), 5.19 (s, 1H), 4.27–4.44 (m, 2H), 1.98 (s, 3H) ppm.

^{13}C NMR (CD_3CN-D_3: D_2O (1:1), 125 MHz, **14**): δ 171.7, 168.5, 139.0, 129.5, 128.3, 128.1, 89.8, 64.5, 42.4, 22.8 ppm.

^{1}H NMR (CD_3CN-D_3: D_2O (1:1), 500 MHz, **15**): δ 7.18–7.34 (m, 5H), 5.27 (s, 1H), 4.27–4.44 (m, 2H) ppm.

^{13}C NMR (CD_3CN-D_3: D_2O (1:1), 125 MHz, **15**): δ 169.4, 139.7, 129.6, 128.2, 128.1, 88.13, 58.1, 42.9 ppm.

***N*[2],*N*[5]-dibenzylpyrazine-2,5-dicarboxamide (16)**

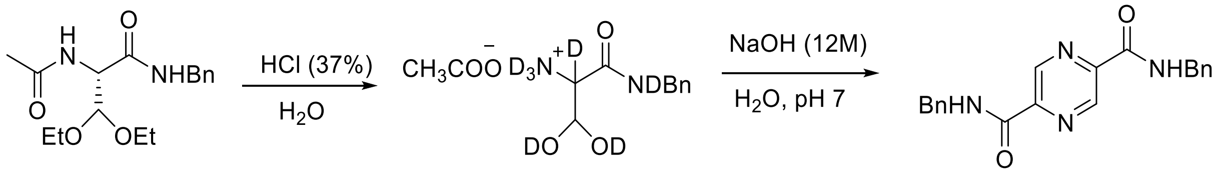

(*S*)-2-acetamido-*N*-benzyl-3,3-diethoxypropanamide **13** (43 mg, 0.14 mmol) was suspended in H_2O (1.6 mL). Concentrated HCl (0.4 mL, 37%) was then added to the stirred solution. The suspension was allowed to stir for 5 days to form 1-(benzylamino)-3,3-dihydroxy-1-oxopropan-2-aminium 2,2,2-trifluoroacetate **15**. The pH of the solution was then adjusted to pH 7 with aq. NaOH (12 M). The resulting yellow precipitate was purified by silica gel flash column chromatography by using EtOAc/cyclohexane (8:2) as eluent to give *N*[2],*N*[5]-dibenzylpyrazine-2,5-dicarboxamide **16** (22 mg, 90% yield) as a white solid (m.p. >250 ºC).

TLC- R_f 0.5 EtOAc/cyclohexane (4:1).

^{1}H NMR (Chloroform-*d*, 500 MHz): δ 9.33 (s, 2H), 8.16 (t, J = 4.7 Hz, NH), 7.39–7.34 (m, 8H), 7.34–7.29 (m, 2H), 4.69 (d, J = 6.1 Hz, 4H) ppm.

^{13}C NMR (Chloroform-*d*, 125 MHz): δ 162.2, 146.2, 142.2, 137.4, 128.8, 127.9, 127.8, 43.6 ppm.

HRMS (ESI) *m/z* $(M+H)^+$, $C_{20}H_{18}N_4O_2$ calcd. 347.1502, observed 347.1513.

IR ν_{max} (neat, cm^{-1}) 3366, 3035, 2923, 2853, 1729, 1653, 1531, 1362, 1276, 1169, 1042, 800, 750, 695.

H. Photolysis of simple cysteine phenacylsulfides in non-buffered aqueous solvent

Photolysis of phenacylsulfide 3f in non-buffered H_2O/MeCN-D_3 solvent

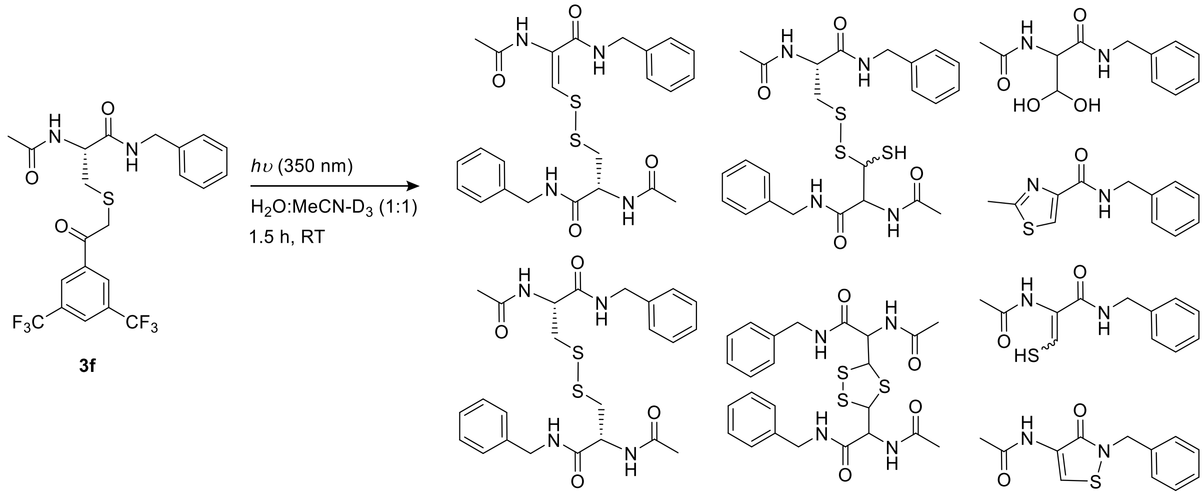

Compound **3f** (5 mg, 9.87 µmol) was dissolved in H_2O/MeCN-D_3 (1:1, 0.8 mL). The pH of this solution was then adjusted to pH 7.4 through addition of NaOH (0.5 M, H_2O). The mixture was then sparged with N_2 for 15 minutes, transferred to an NMR tube and back-flushed with N_2 prior to sealing. The resulting solution was then illuminated at 350 nm in a Rayonet photoreactor for 1.5 hrs. The pH of the reaction mixture was then measured after completion of the photolysis and was found to have dropped to pH 3.8. The resulting reaction mixture was then analysed by pre-saturated ^{1}H NMR and ^{13}C NMR spectroscopy, LC-HRMS (ESI positive mode) and direct-injection HRMS (ESI positive mode).

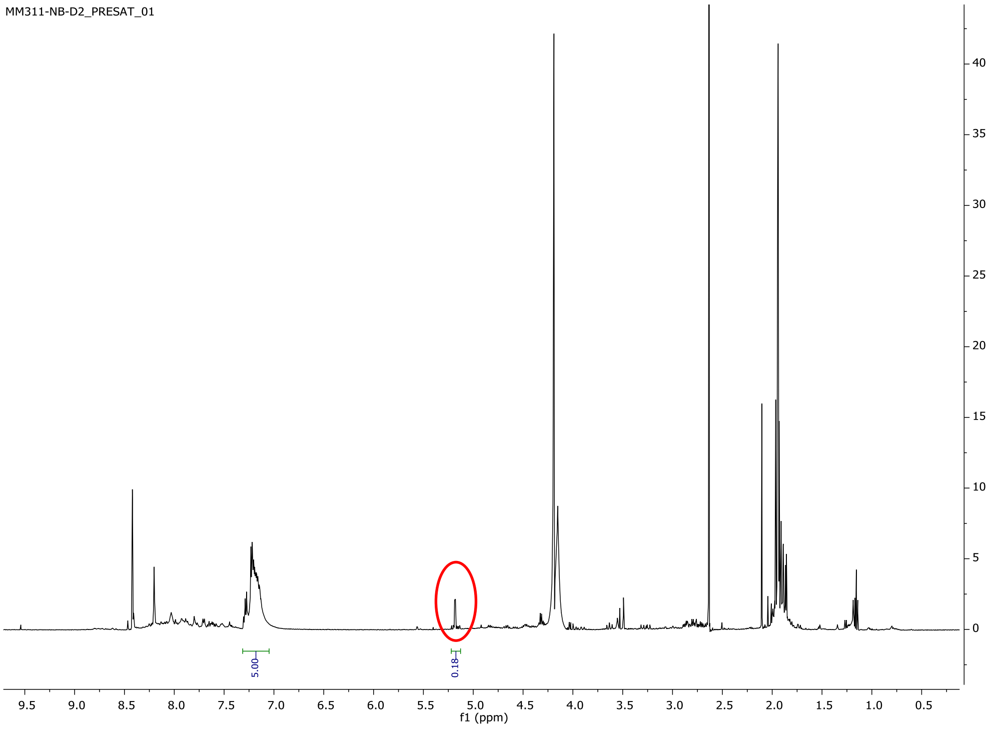

*Figure H1: Presaturated ^{1}H NMR spectrum of the reaction mixture derived from the photolysis of phenacylsulfide **3f** in H_2O/CD_3CN (1:1), highlighting the β-methine region, 5.12 – 5.23 ppm.*

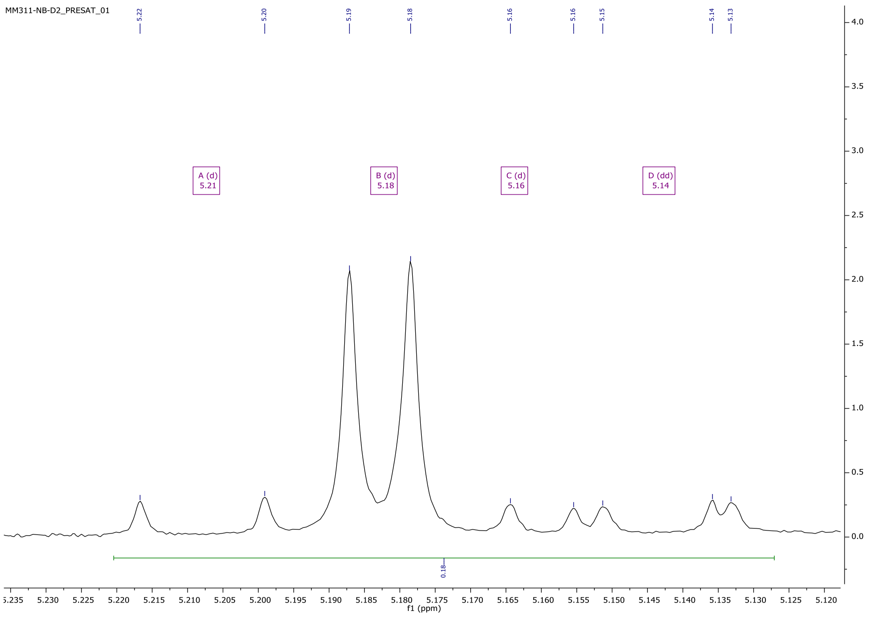

*Figure H2: Zoomed-in image of the β-methine region, 5.13–5.23 ppm, of the presaturated ^{1}H NMR spectrum of the reaction mixture derived from the photolysis of phenacylsulfide **3f** in H_2O/CD_3CN (1:1). The doublet at 5.18 ppm (J = 4.7 Hz) is assigned to the β-methine hydrogen of the gem-diol form of the formylglycine derivative (12% conversion from phenacylsulfide). The large doublet is flanked on either side by a collection of four doublets (J = 8.8, ca. 7.1, 9.8, 9.1 Hz), assigned to the β-methine hydrogens of three diastereomeric 1,2,4-trithiolane species (6% conversion from phenacylsulfide). Note: Region labelled as Doublet D is two overlapping doublets.*

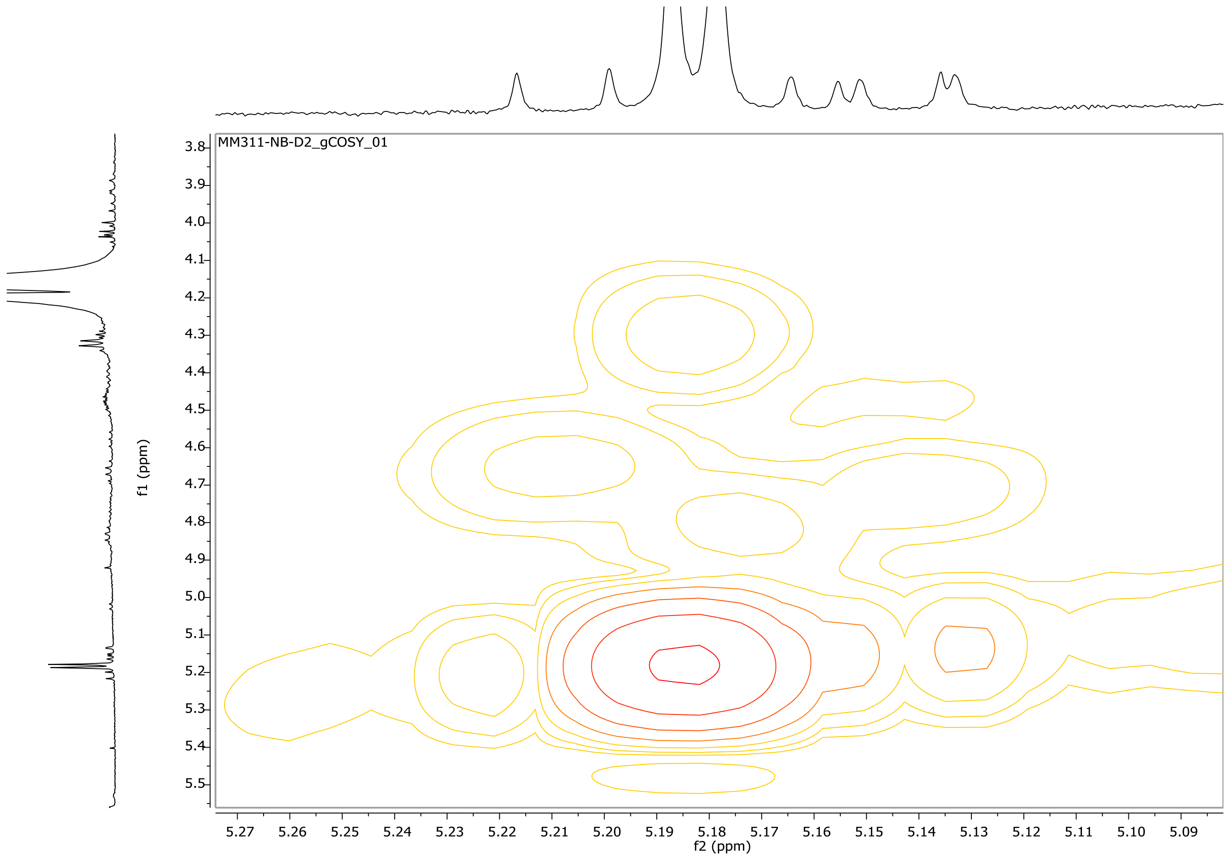

*Figure H3: Zoomed-in region of COSY spectrum of the reaction mixture derived from the photolysis of phenacylsulfide **3f** in H_2O/CD_3CN (1:1) showing the ^3J coupling between the β-methine and α-methine hydrogen nuclei of gem-diol and 1,2,4-trithiolane species.*

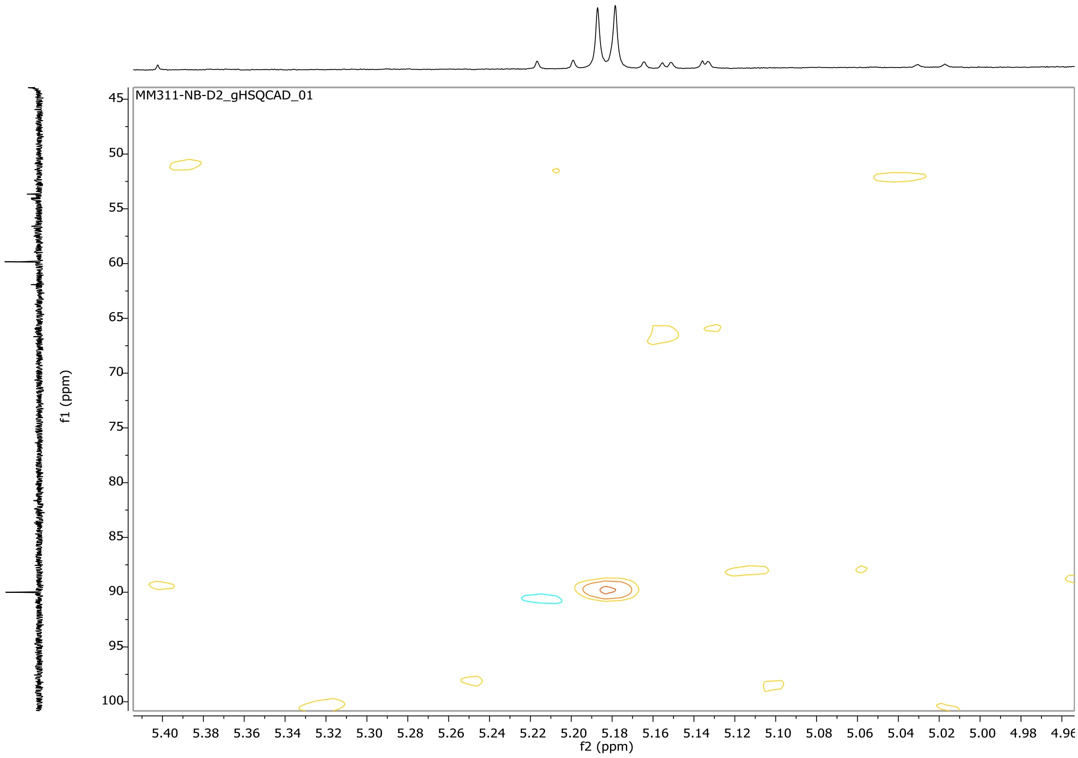

Figure H4: Zoomed-in region of HSQC spectrum of the reaction mixture derived from the photolysis of phenacylsulfide ***3f*** *in H_2O/CD_3CN (1:1) showing a correlation between the β-methine at 5.18 ppm to the ^{13}C nucleus at 90 ppm, consistent with assigned gem-diol species. Weaker correlations between the β-methine hydrogen nuclei of assigned 1,2,4-trithiolanes and ^{13}C nuclei at around 65 ppm is apparent. These correlations are clearer in other spectra presented below.*

HRMS analysis of the reaction mixture derived from the photolysis of phenacylsulfide 3f in H_2O/CD_3CN (1:1)

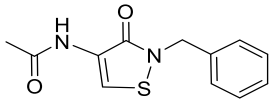

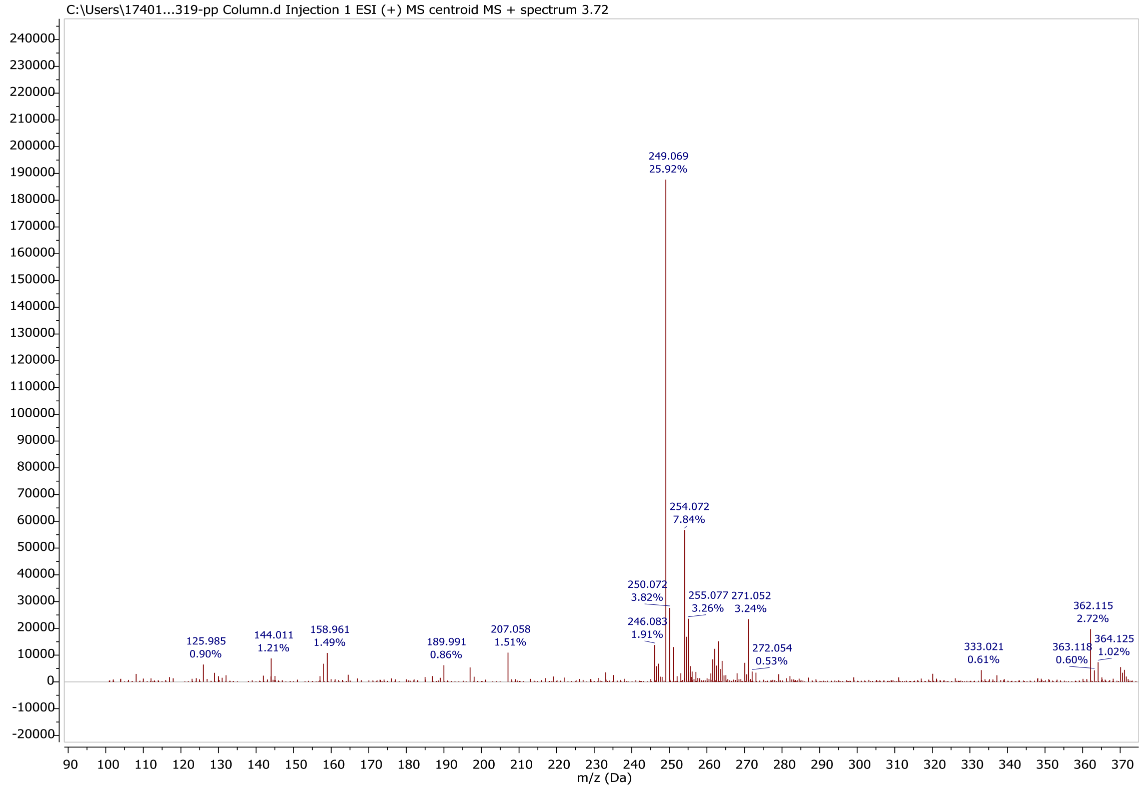

Figure H5: HRMS (ESI positive mode) m/z peaks of isothiazolone showing the M+H adduct (Calculated: 249.069, Observed 249.069).

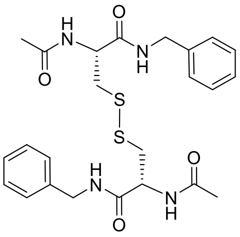

Exact Mass: 502.1708

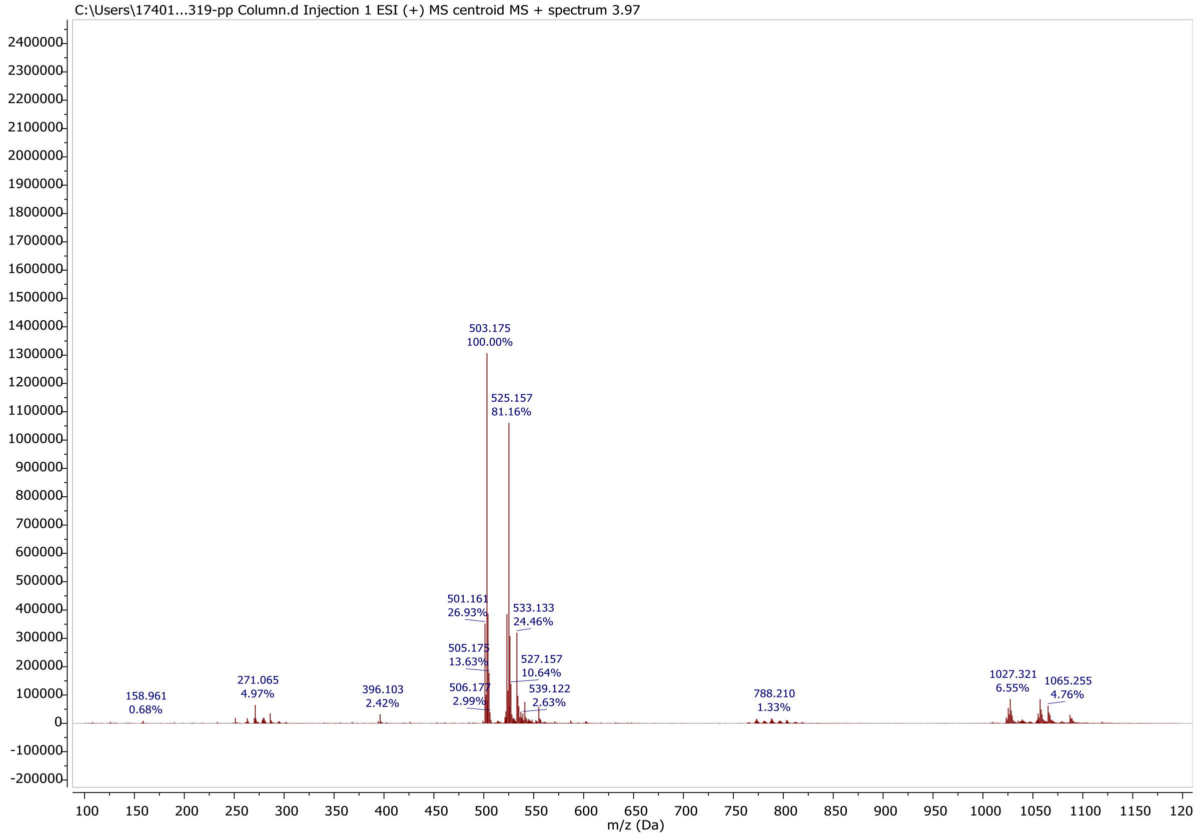

Figure H6: HRMS (ESI positive mode) of the disulfide showing the M+H (Calculated: 503.178, Observed 503.175) and M+Na (Calculated: 525.160, Observed: 525.157) adducts

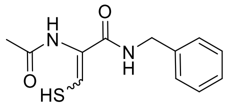

Exact Mass: 250.0776

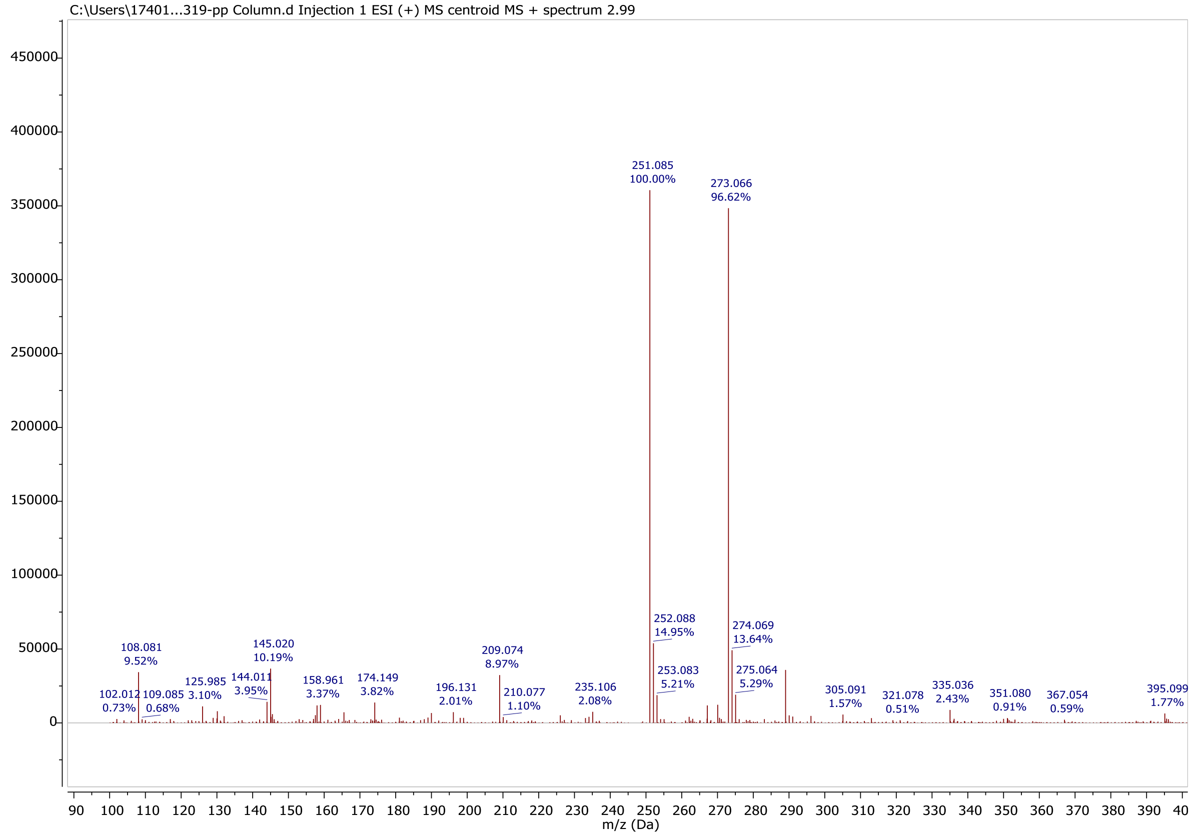

Figure H7: HRMS (ESI positive mode) of enethiol showing the M+H (Calculated: 251.085, Observed 251.085) and M+Na (Calculated: 273.067, Observed: 273.066) adducts.

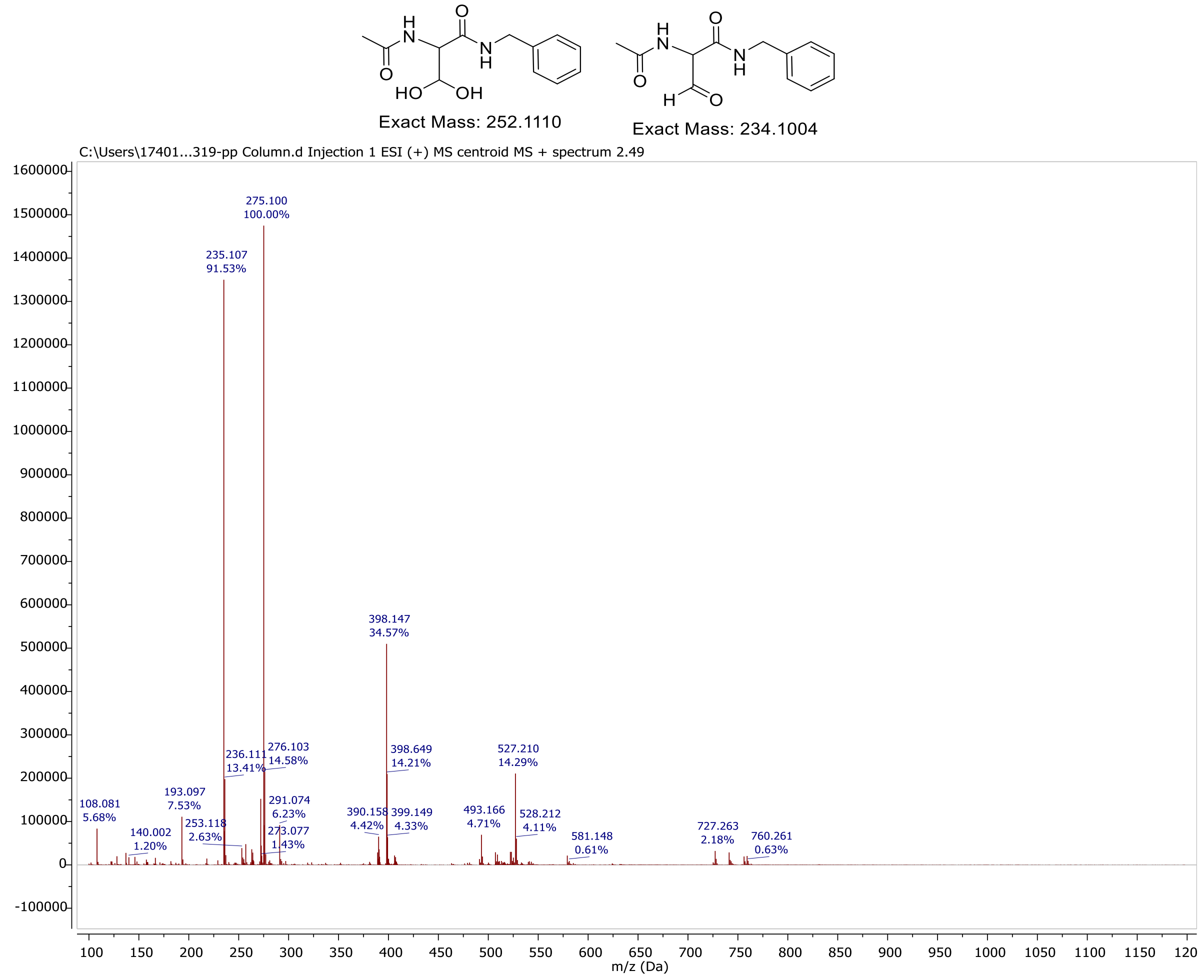

Figure H8: HRMS (ESI positive mode) of non-hydrated aldehyde showing M+H (Calculated: 235.108, Observed 235.107). M+Na of gem-diol is also observed (Calculated: 275.100, Observed 275.100)

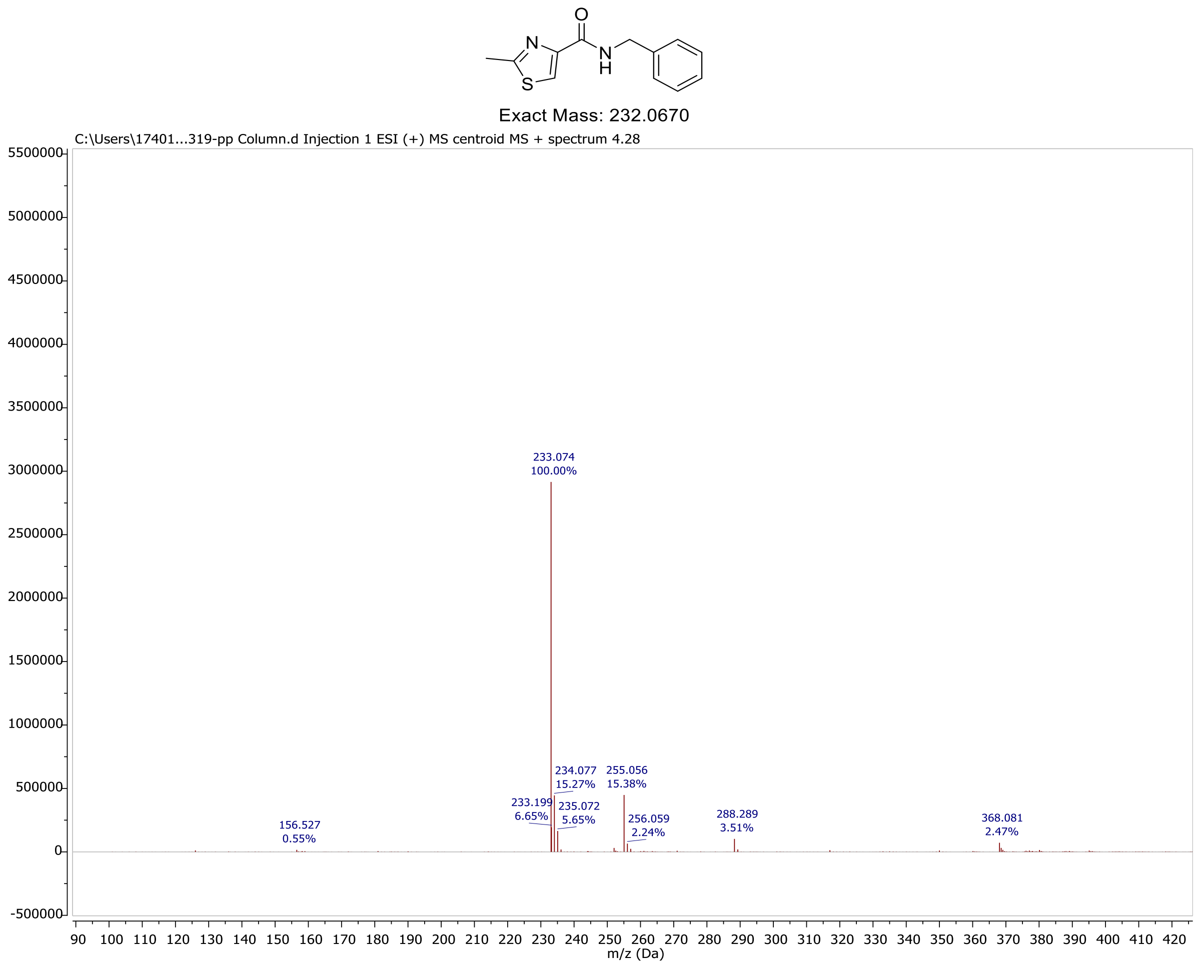

Figure H9: HRMS (ESI positive mode) of the thiazole showing the M+H (Calculated: 233.074, Observed 233.074) and M+Na (Calculated: 255.056, Observed: 255.056) adducts

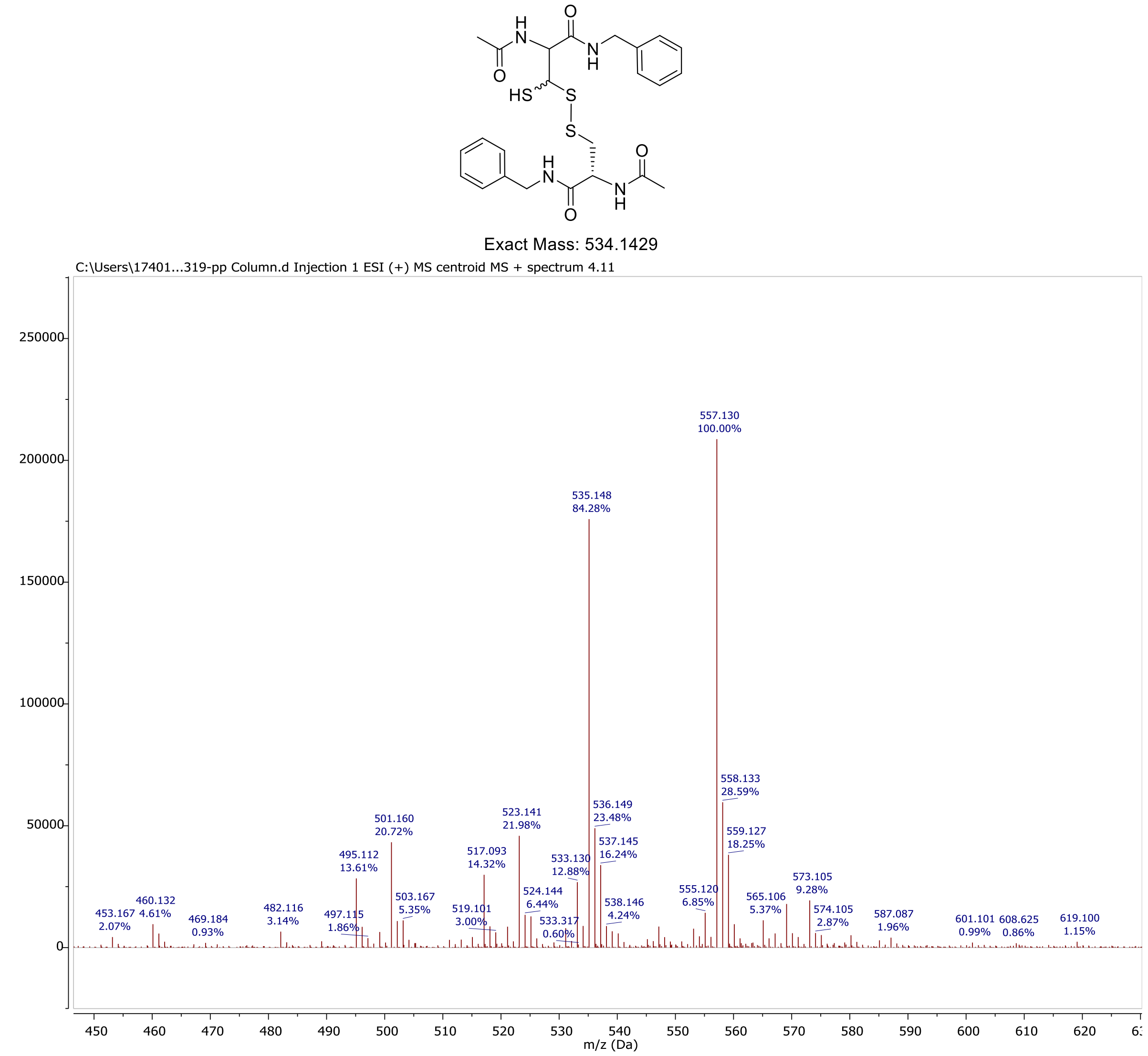

Figure H10: HRMS (ESI positive mode) of the dithioacetal/thiol disulfide showing the M+H (Calculated: 535.150, Observed 535.148) and M+Na (Calculated: 557.132, Observed 557.130) adducts.

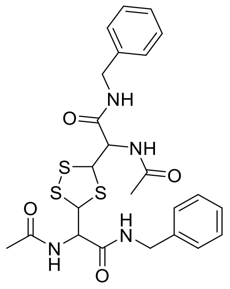

Exact Mass: 532.1273

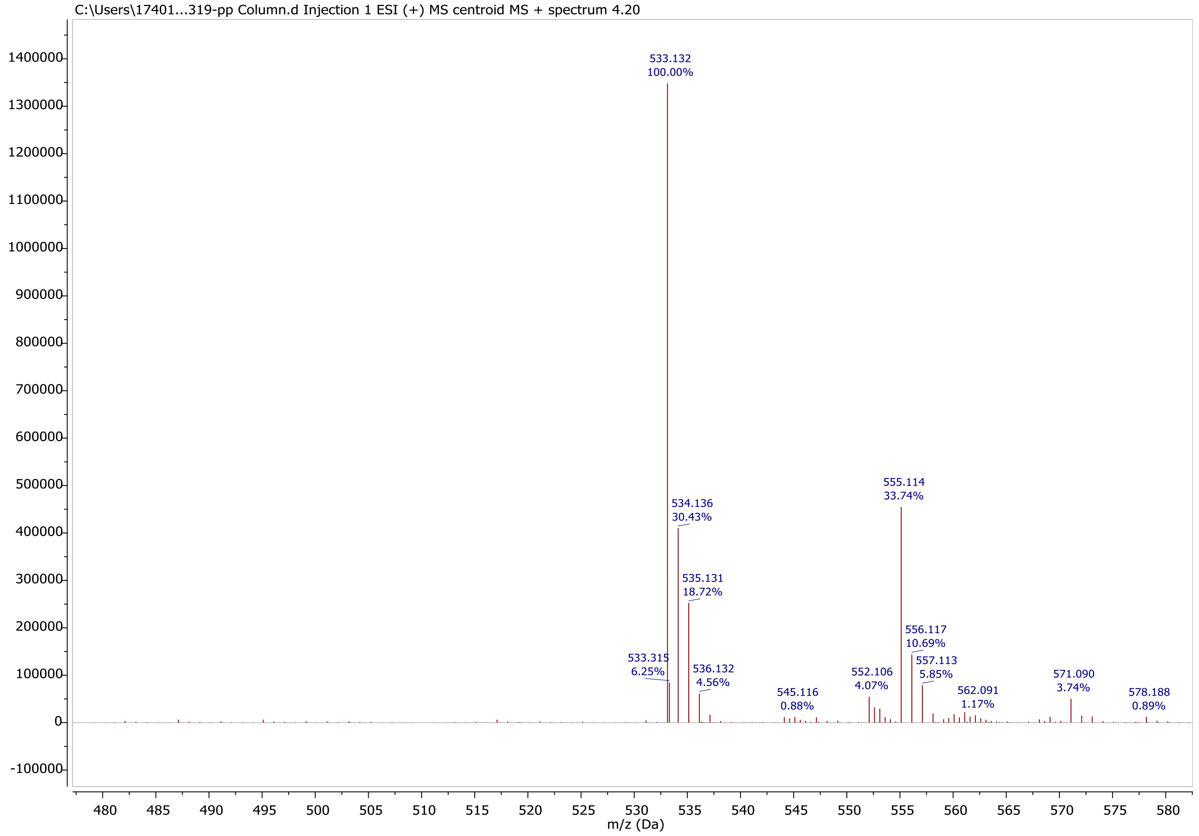

Figure H11: HRMS (ESI positive mode) of 1,2,4-trithiolane M+H (Calculated: 533.135, Observed 533.132) and M+Na (Calculated: 555.117, Observed: 555.114) adducts

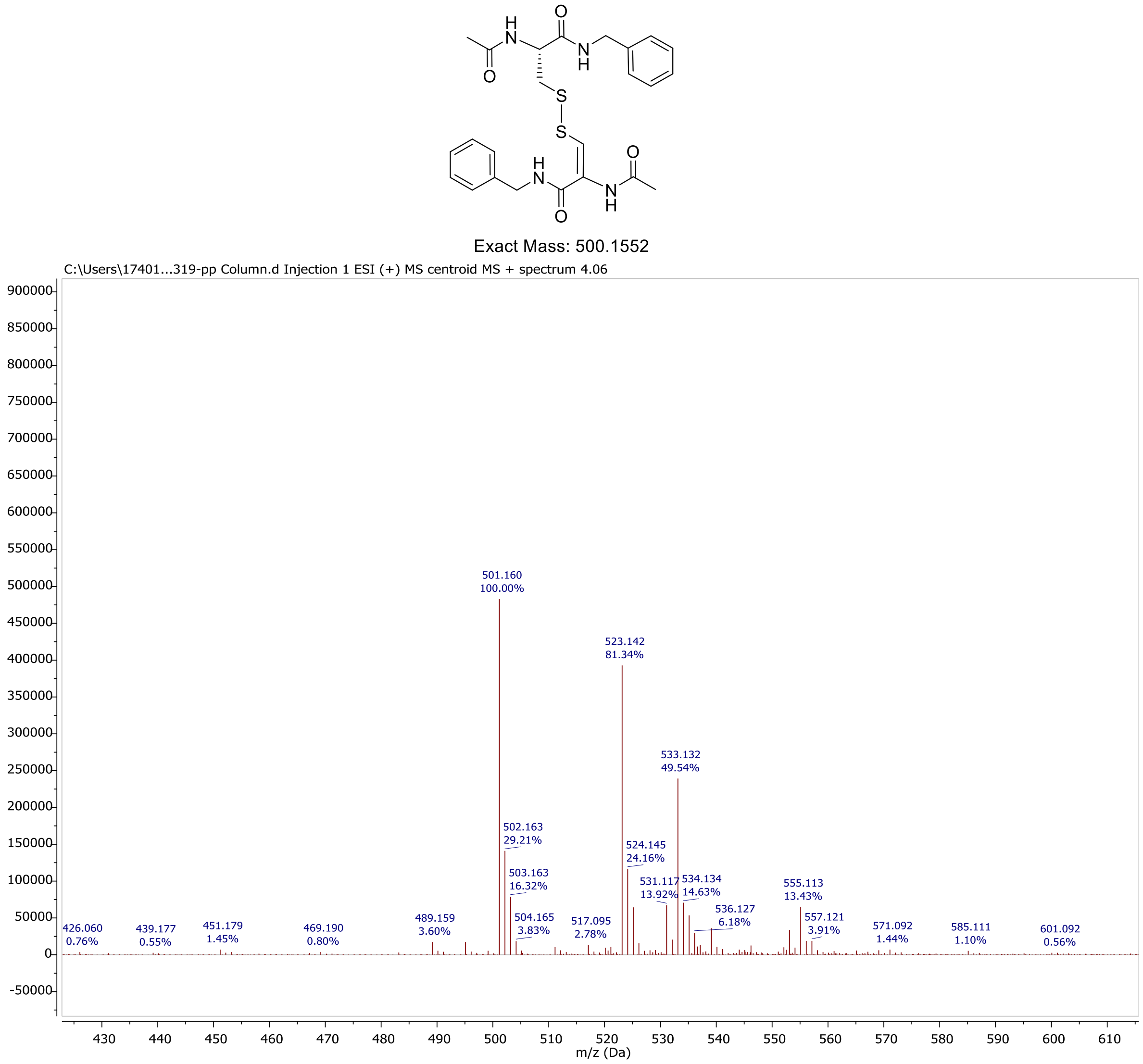

Figure H12: HRMS (ESI positive mode) of the enethiol/thiol disulfide showing the M+H (Calculated: 501.162, Observed 501.160) and M+Na (Calculated: 523.144, Observed: 523.142) adducts

Photolysis of phenacylsulfide 3f in non-buffered D_2O/MeCN-D_3 solvent

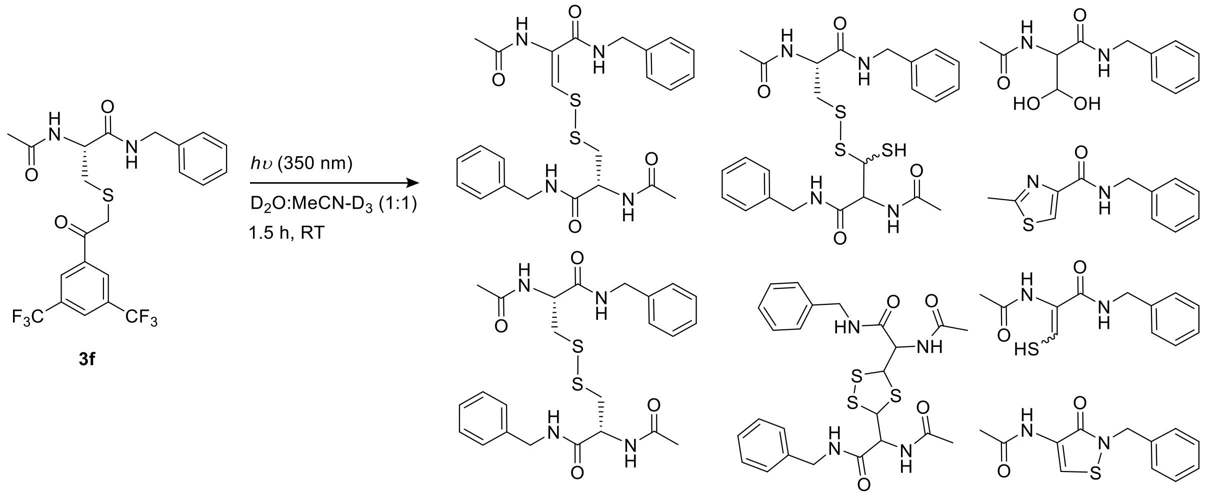

Compound **3f** (5 mg, 9.87 µmol) was dissolved D_2O/MeCN-D_3 (1:1, 0.8 mL). The pH of this solution was then adjusted to pD 7.5 through addition of a NaOH stock solution (0.5 M, D_2O). The mixture was then sparged with N_2 for 15 minutes, transferred to an NMR tube and then back-flushed with N_2 prior to sealing. The resulting solution was then illuminated at 350 nm in a Rayonet photoreactor for 1.5 hrs. The pD of the reaction mixture was then measured after completion of the photolysis and was found to have dropped to pD 4.2. MeCN-D_3 (0.15 mL) was added to the reaction mixture to ensure a homogeneous solution prior to NMR analysis.

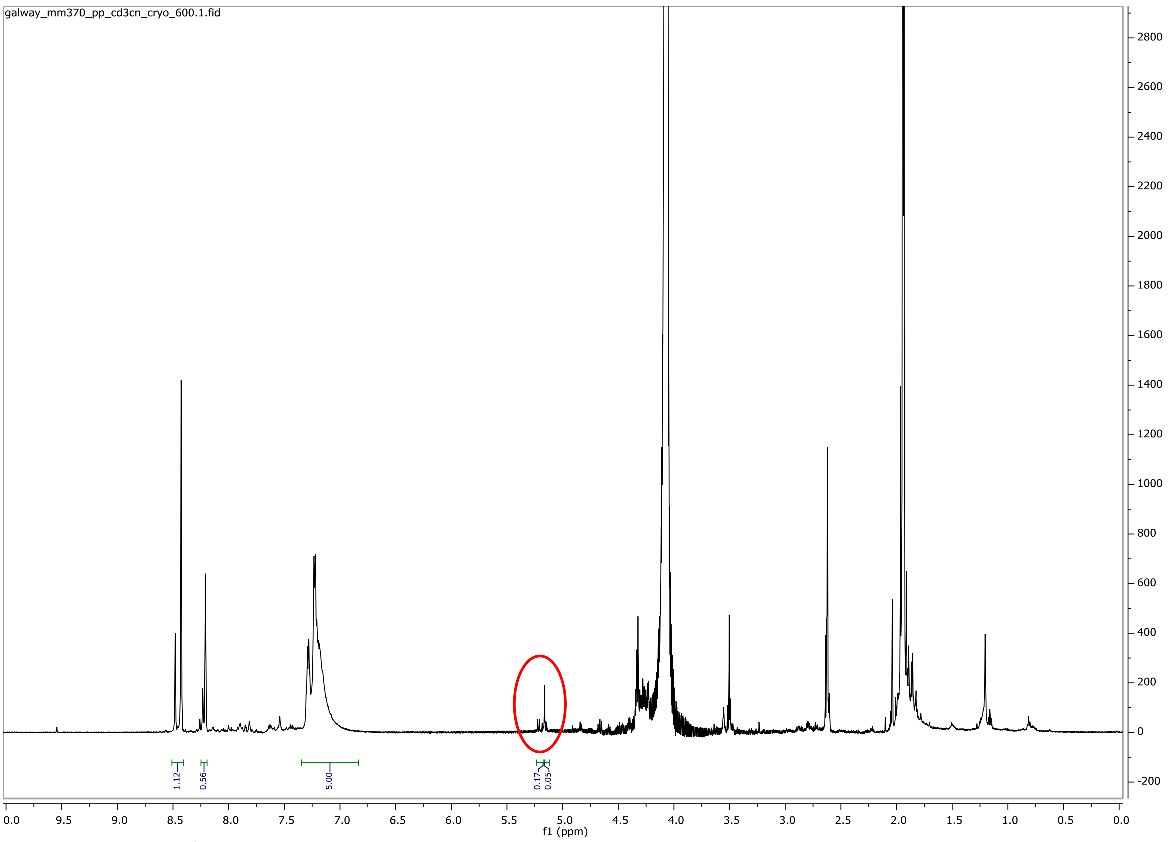

*Figure H13: ^1H NMR spectrum of the reaction mixture derived from the photolysis of phenacylsulfide **3f** in D_2O/CD_3CN (1:1), highlighting the β-methine region, 5.12 – 5.23 ppm*

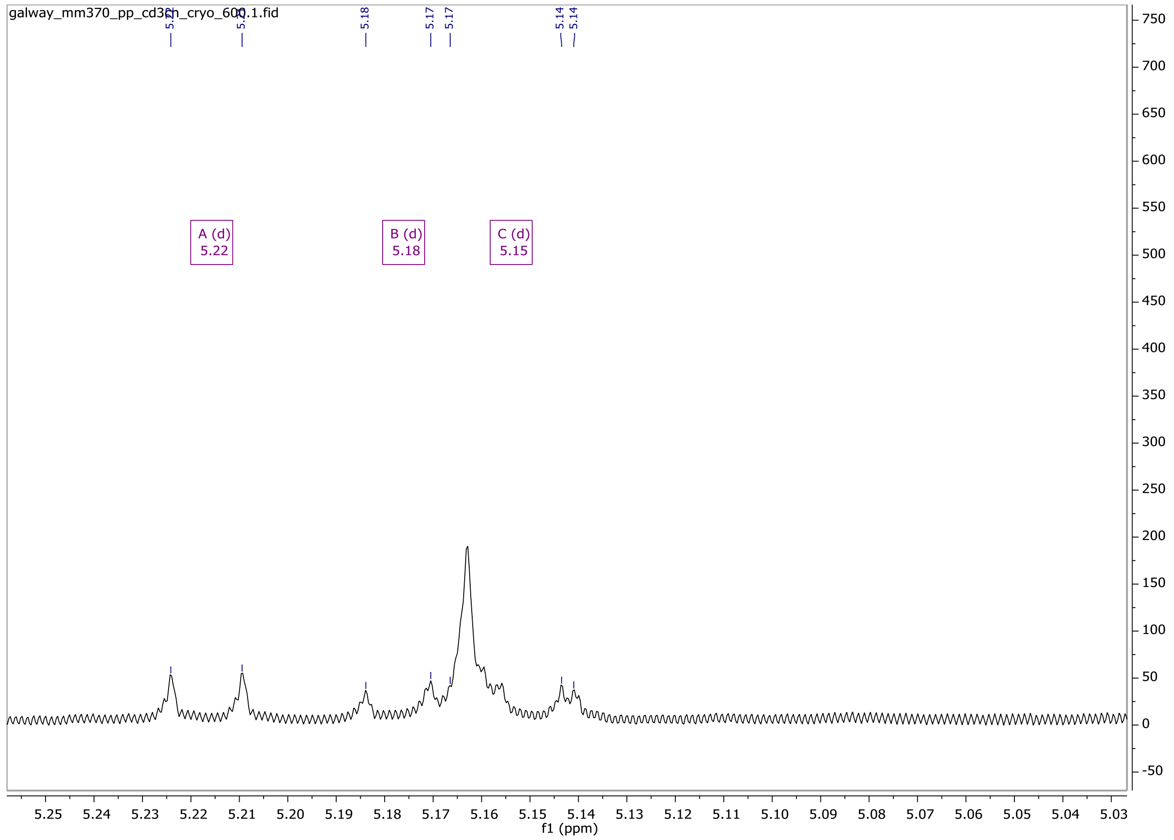

Figure H14: Zoomed-in image of the β-methine region, 5.13–5.23 ppm, of the ^{1}H NMR spectrum of the reaction mixture derived from the photolysis of phenacylsulfide ***3f*** *in D_2O/CD_3CN (1:1). The singlet at 5.16 ppm is assigned to the β-methine hydrogen of the gem-diol form of the formylglycine derivative (5% conversion from phenacylsulfide); H/D exchange at the α position reduces the doublet (as observed in H_2O) to a singlet. The singlet is flanked on either side by a collection of four doublets (J = 8.8, ca. 8.0, ca. 9.6, ca. 9.5 Hz), assigned to the β-methine hydrogens of three diastereomeric 1,2,4-trithiolane species (12% conversion from phenacylsulfide).*

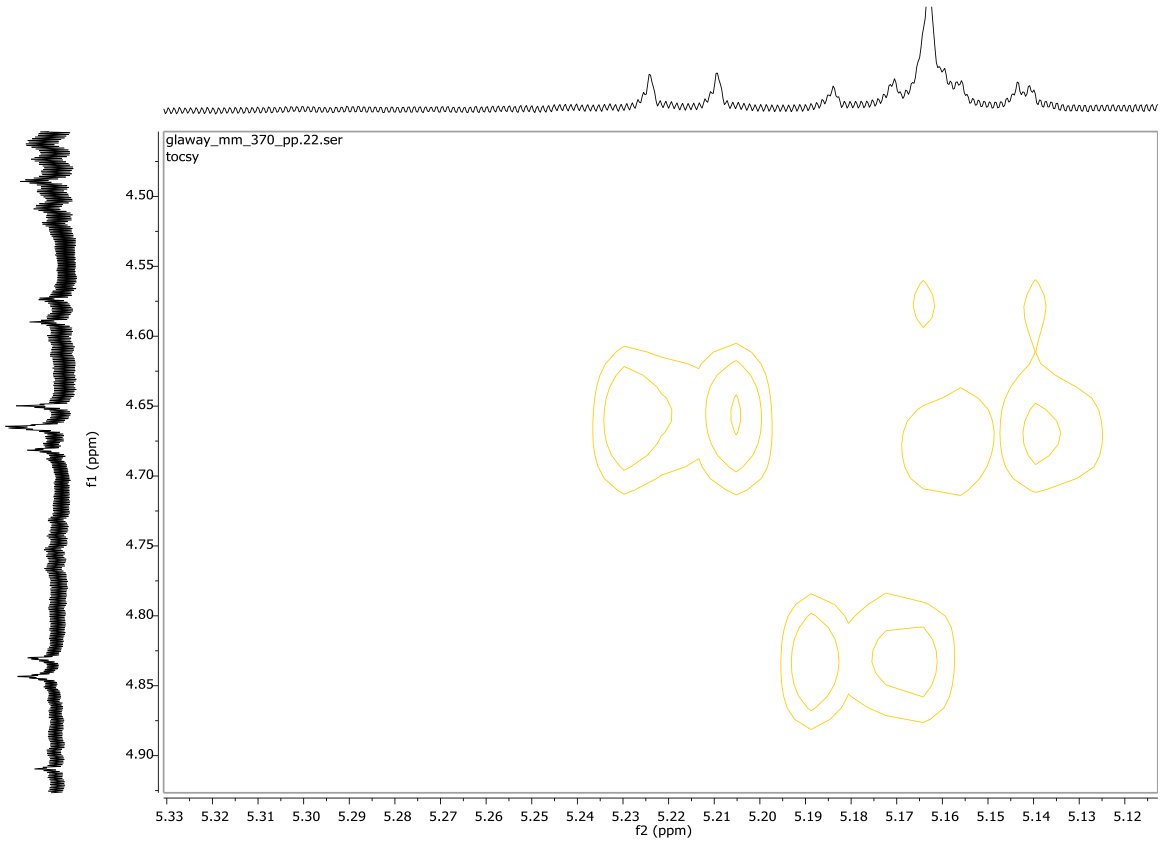

*Figure H15: Zoomed-in region of COSY spectrum of the reaction mixture derived from the photolysis of phenacylsulfide **3f** in D_2O/CD_3CN (1:1) showing the ^3J coupling between the β-methine and α-methine hydrogen nuclei of 1,2,4-trithiolane species.*

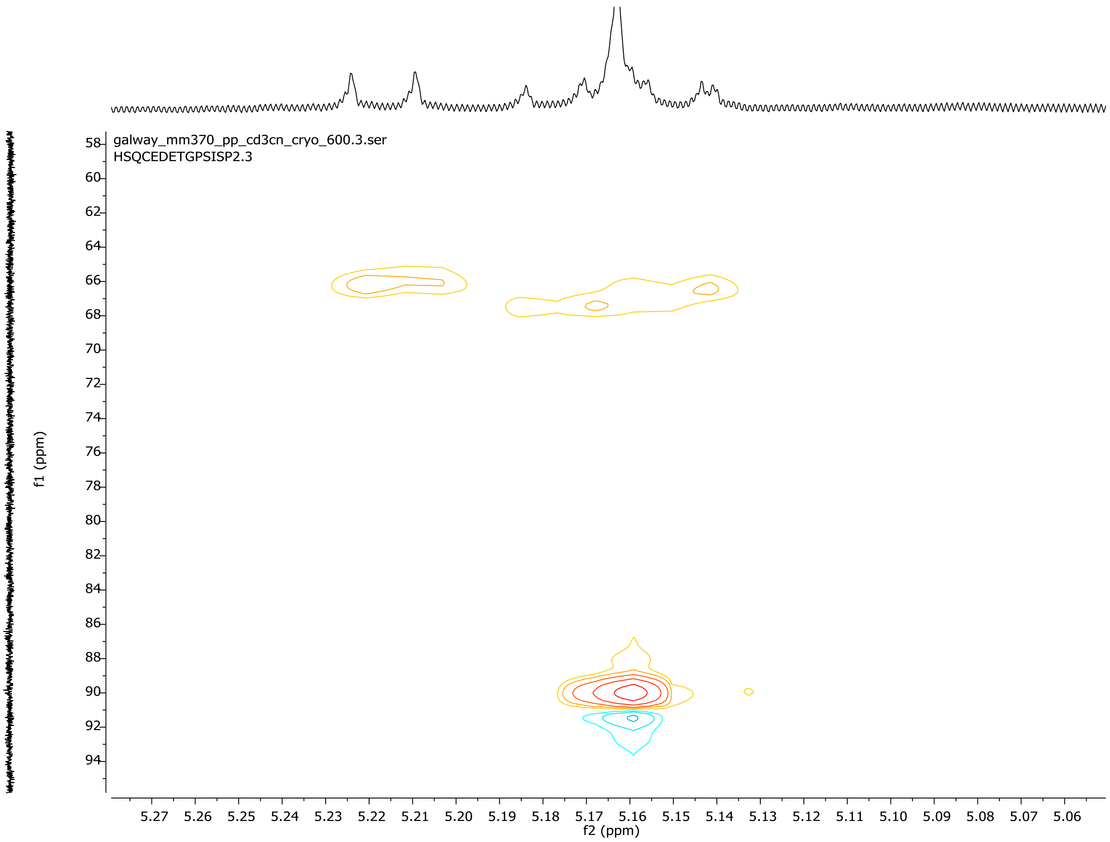

Figure H16: Zoomed-in region of HSQC spectrum of the reaction mixture derived from the photolysis of phenacylsulfide ***3f*** *in D_2O/CD_3CN (1:1) showing a correlation between the β-methine at 5.17 ppm to the ^{13}C nucleus at 90 ppm, consistent with assigned gem-diol species, and correlations between the β-methine hydrogen nuclei of assigned 1,2,4-trithiolanes and ^{13}C nuclei in the region 66–68 ppm is apparent.*

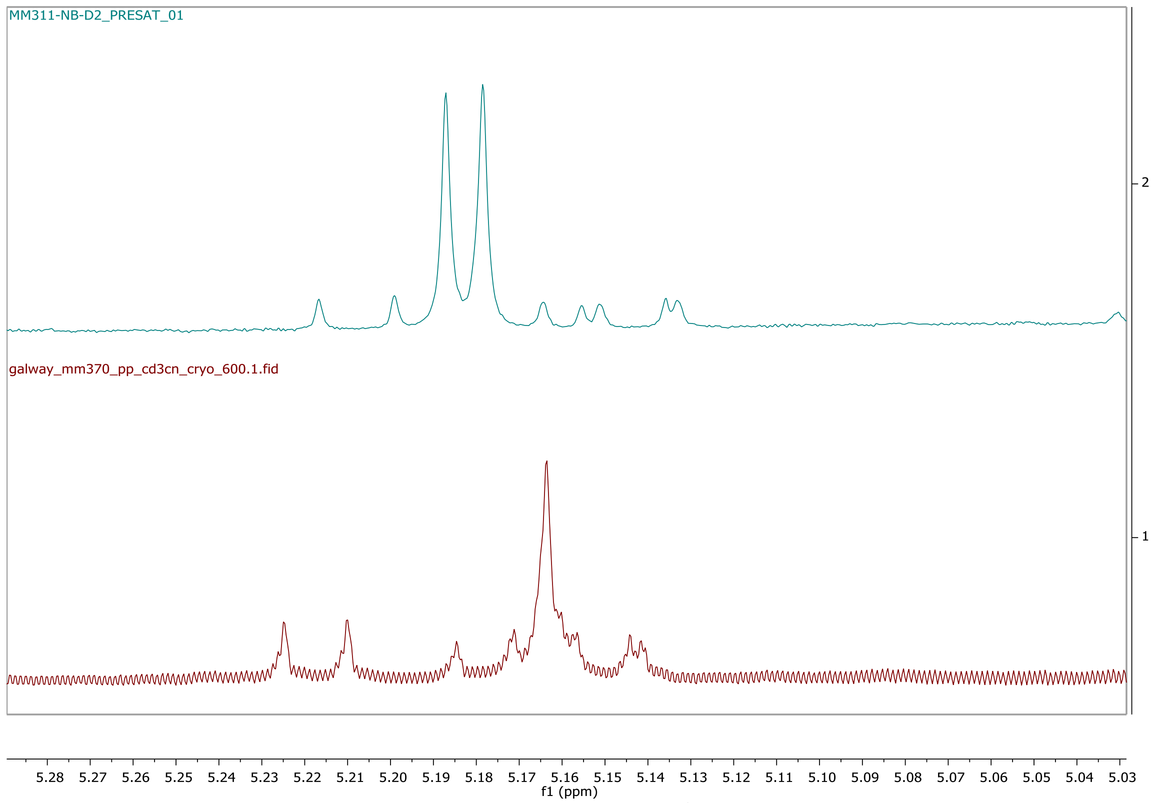

*Figure H17: Comparison of methine region in the ^{1}H NMR spectra of the reaction mixture derived from the photolysis of phenacylsulfide **3f** in H_2O/CD_3CN (top) D_2O/CD_3CN (bottom) Signals are shifted owing to slightly different conditions.*

Evolution of the composition of aq. DMSO-D6 photolysis mixtures derived from the photolysis of phenacylsulfide 3f under various conditions (pH/pD value and the presence of buffer)

Photolysis of phenacylsulfide 3f in H_2O/DMSO–d_6 (2:3)

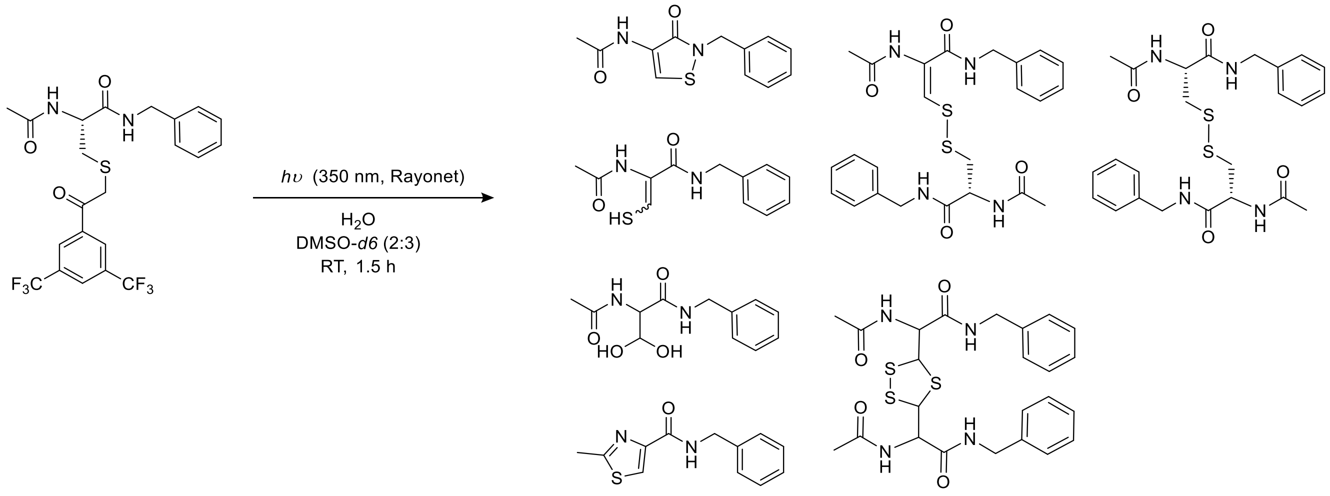

Compound **3f** (60 mg, 118.4 µmol) was dissolved in H_2O/DMSO-d_6 (2:3, 9.6 mL). The pH of this solution was then adjusted to pH 7.6 through addition of 0.5 M NaOH in H_2O. The mixture was then sparged with N_2 for 15 minutes, transferred to a NMR tube and then back-flushed with N_2 prior to sealing. The resulting solution was then illuminated at 350 nm in Rayonet photoreactor for 1.5 hrs. The pH of the solution was measured and after 11 days and was found to have dropped to pH 4.6.

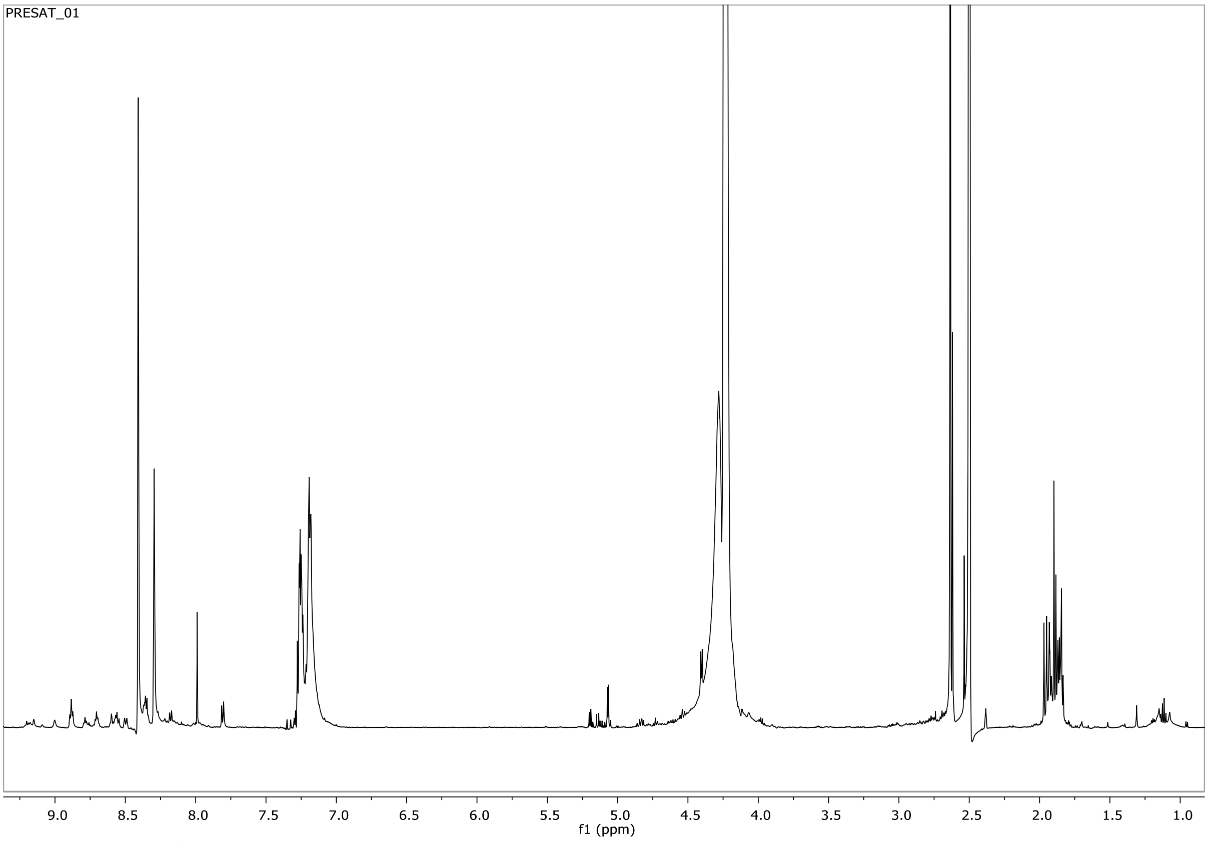

*Figure H18: ^{1}H NMR spectrum of the mixture derived from the photolysis of phenacylsulfide **3f** in H_2O/DMSO-d_6 (2:3) at day 0.*

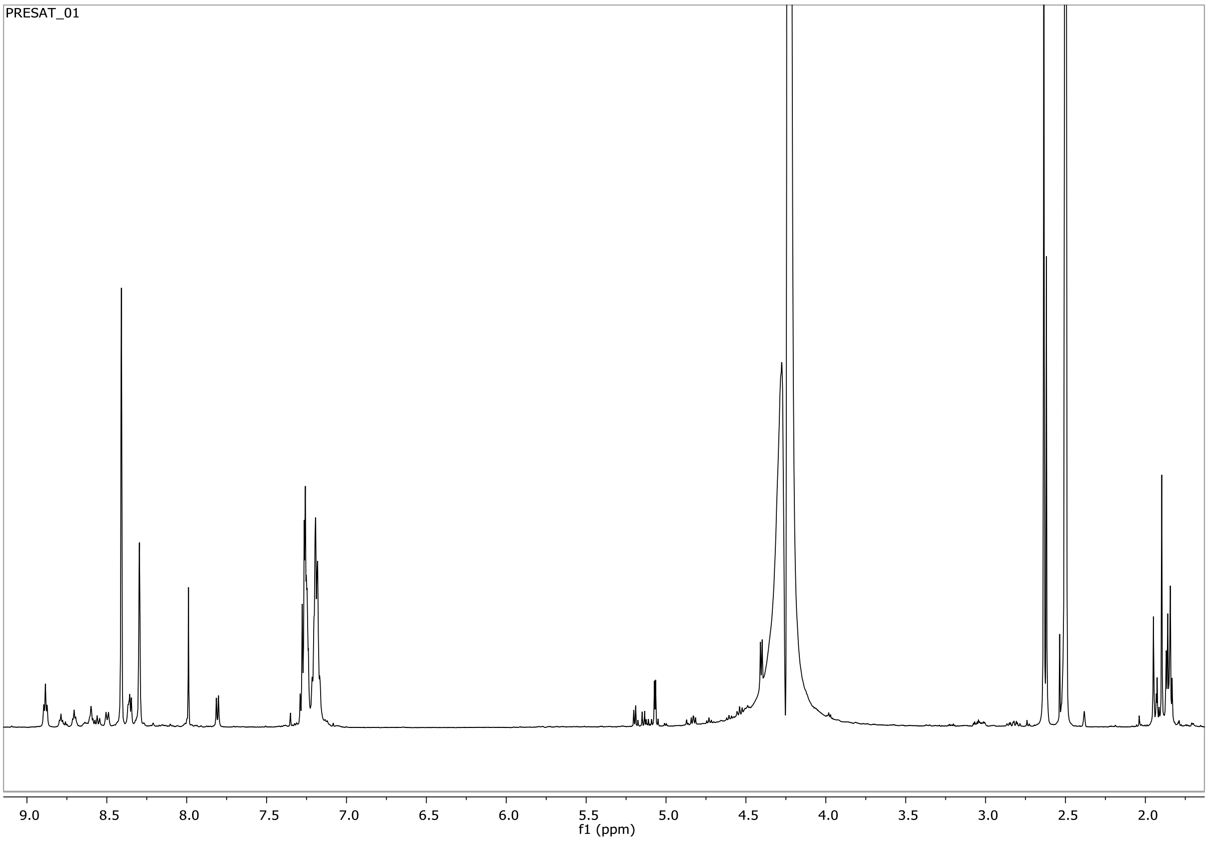

*Figure H19: ^{1}H NMR spectrum of the mixture derived from the photolysis of phenacylsulfide **3f** in H_2O/DMSO-D_6 (2:3) at day 11.*

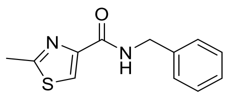

Exact Mass: 232.0670

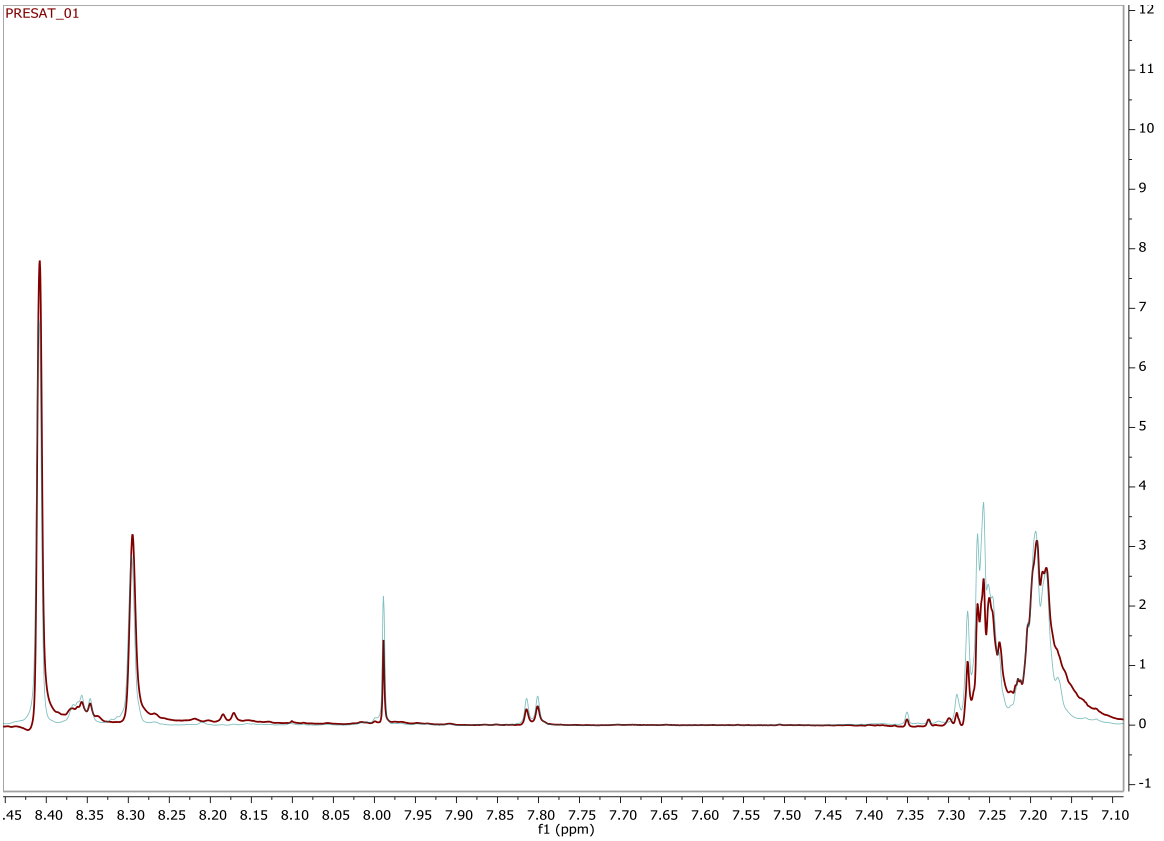

*Figure H20: Overlapped ^{1}H NMR spectra of the mixture derived from the photolysis of phenacylsulfide **3f** in H_2O/DMSO-D_6 (2:3) as analysed over 11 days. The methine signals of the thiazole at 7.99 ppm increases from day 0 (red) to day 11 (green).*

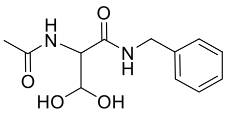

Exact Mass: 252.1110

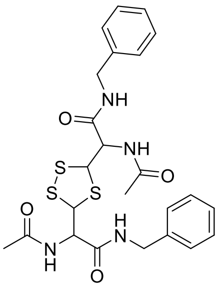

Exact Mass: 532.1273

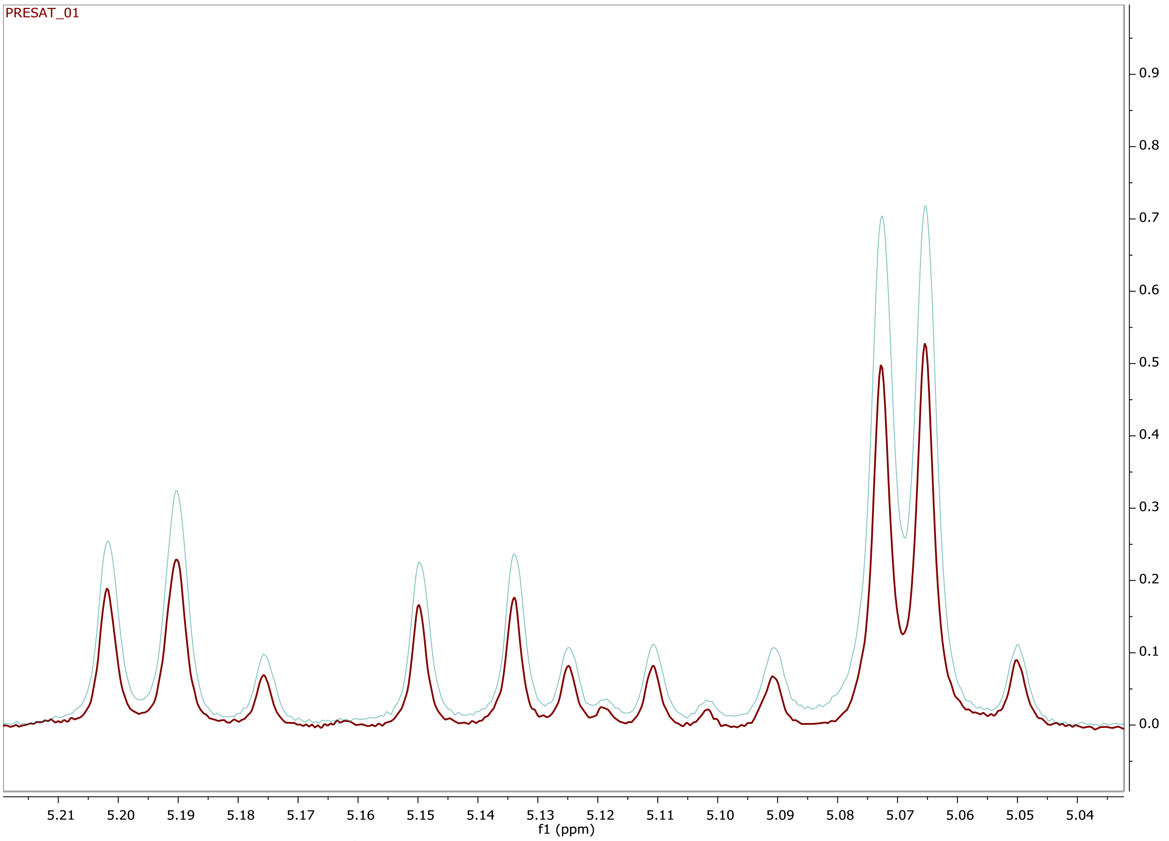

*Figure H21: Overlapped ^{1}H NMR spectra of the mixture derived from the photolysis of phenacylsulfide **3f** in H_2O/DMSO-D_6 (2:3) as analysed over 11 days. Zoomed in region shows signals associated with the methine signal of the trithiolanes and geminal diol from 5.04–5.21 ppm. The doublet at 5.06–5.08 correspond to the methine signal of geminal diol. The trithiolanes and geminal diol increases from day 0 (red) to day 11 (green). Note the increased number of doublets for the 1,2,4-trithiolane species relative to the spectrum of the mixture in aqueous acetonitrile. The use of DMSO-D_6 solvent appears to promote racemisation at the alpha position.*

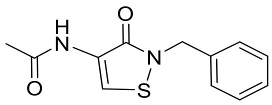

Exact Mass: 248.0619

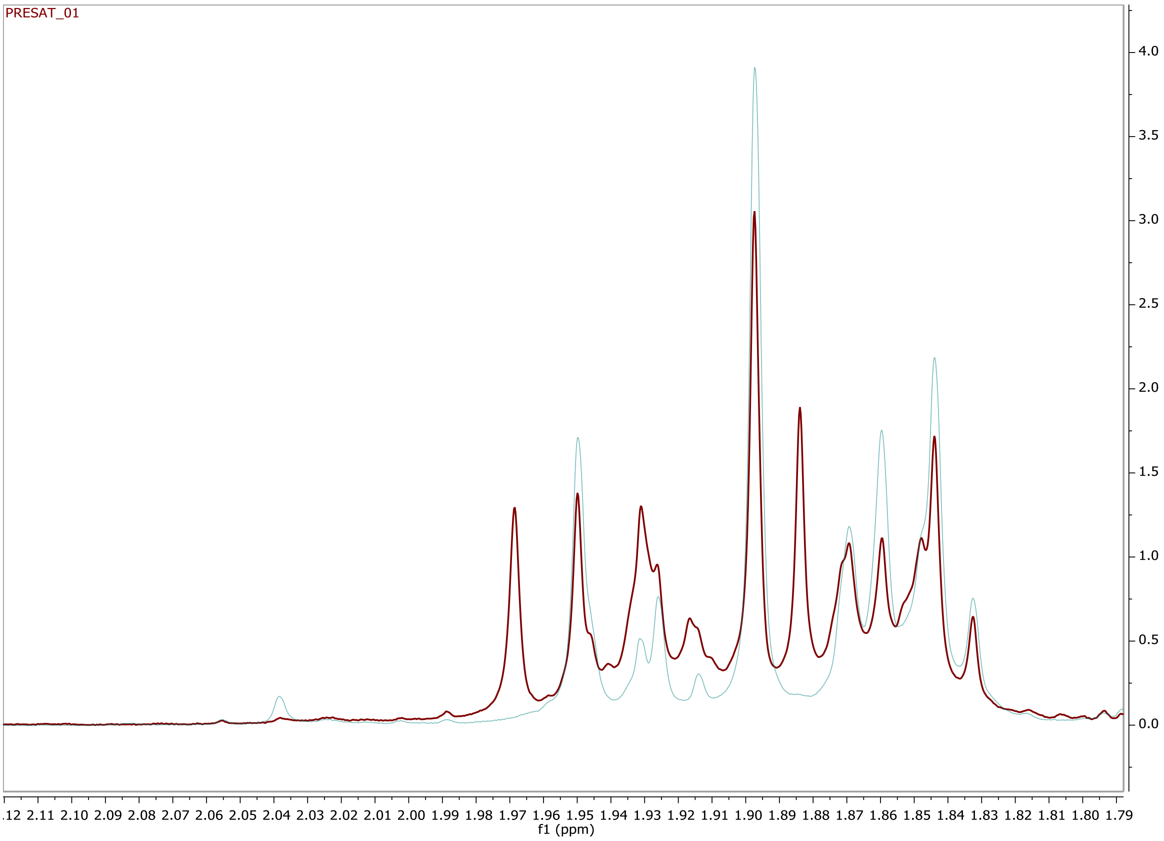

Figure H22: Overlapped ^{1}H NMR spectra of the mixture derived from the photolysis of phenacylsulfide ***3f*** *in H_2O/DMSO-D_6 (2:3) as analysed over 11 days. The methyl signal of the isothiazolone at 2.04 ppm increases from day 0 (red) to day 11 (green).*

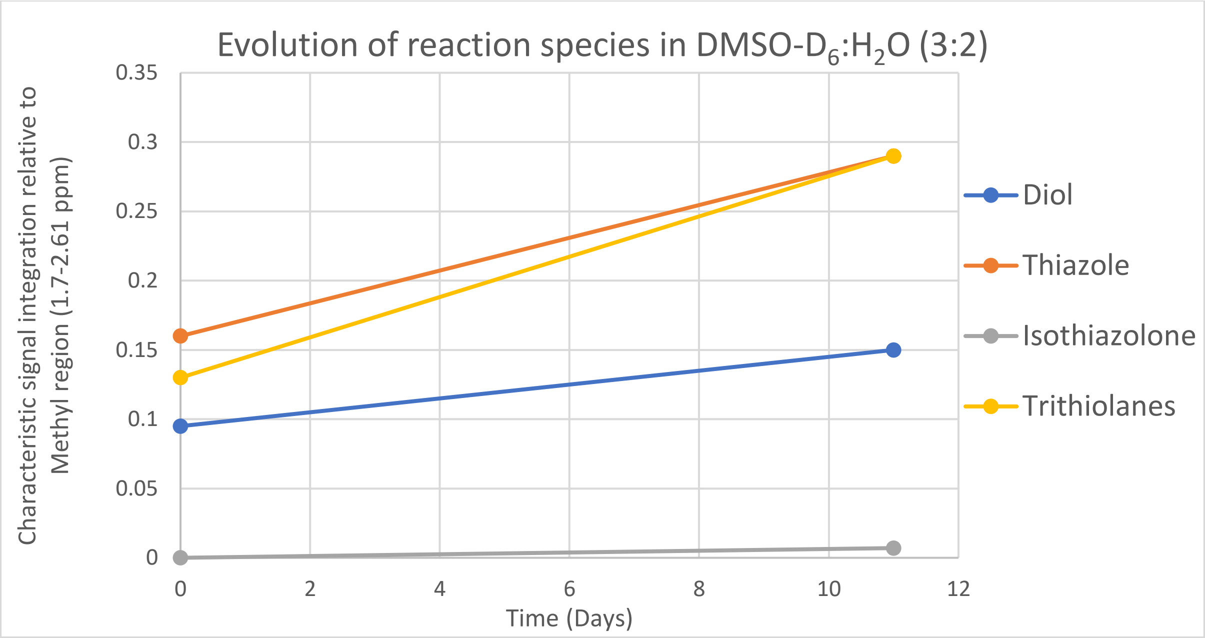

Time (Days)	Diol (5.06 ppm)	1,2,4-Trithiolanes (5.21-5.04 ppm)	Isothiazolone (4.87 ppm)	Thiazole (2.61 ppm)
T0	0.095	0.13	0	0.16
T11	0.15	0.29	0.007	0.29

Figure H23: The evolution of the reaction mixture derived from the photolysis of phenacylsulfide ***3f*** *in H_2O/DMSO-D_6 (2:3) over 11 days. The integration of a methine CH signal for the diol, and trithiolanes are calculated relative to the methyl region from 1.7-2.61 ppm. The integration of the methyl CH_3 of the isothiazolone and the methyl CH_3 of the thiazole are also calculated relative to the same region. Table of corresponding data shown below graph. Note: integral values for both the methyl CH_3 of the isothiazolone and the methyl CH_3 of the thiazole are divided by 3, to provide the value of a single proton for each species.*

Photolysis of phenacylsulfide 3f in D_2O/DMSO–d_6 (2:3) and evolution of photolysis mixture under different conditions.

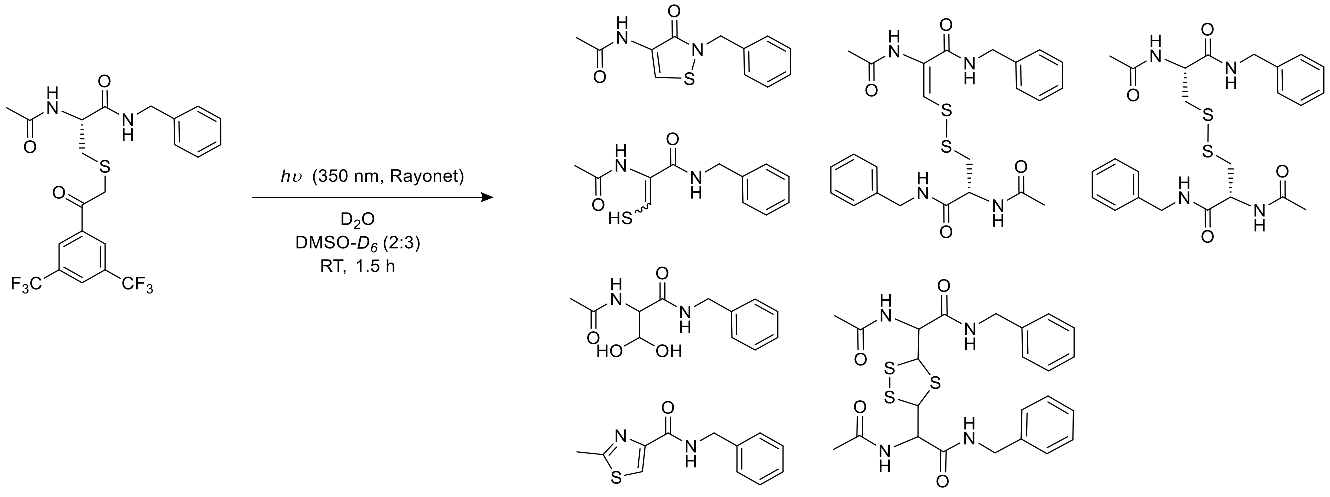

Compound **3f** (60 mg, 118.4 µmol) was dissolved in D_2O/DMSO-D_6 (2:3, 9.6 mL). The pD of this solution was then adjusted to pD 8 through addition of 0.5 M NaOH in D_2O. The mixture was then sparged with N_2 for 15 minutes, transferred to a NMR tube and then back-flushed with N_2 prior to sealing. The resulting solution was then illuminated at 350 nm in Rayonet photoreactor for 1.5 hrs. After photolysis, the pD of the solution was measured and was found to have dropped to pD 3.80. The reaction mixture was then divided into 8 equal aliquots (1 mL) and treated as follows:

Aliquot (A): Addition of D_2O/DMSO-D_6 (2:3) – adjusted to pH 4.0
Aliquot (B): Addition of H_2O/DMSO-D_6 (2:3) – adjusted to pH 3.6
Aliquot (C): Addition of D_2O (0.1M phosphate)/DMSO-D_6 (2:3) – adjusted to pH 4.0
Aliquot (D): Addition of H_2O (0.1M phosphate)/DMSO-D_6 (2:3) – adjusted to pH 3.6
Aliquot (E): Addition of D_2O/DMSO-D_6 (2:3) – adjusted to pH 8.0
Aliquot (F): Addition of H_2O/DMSO-D_6 (2:3) – adjusted to pH 8.0
Aliquot (G): Addition of D_2O (0.1M phosphate)/DMSO-D_6 (2:3) – adjusted to pH 8.0
Aliquot (H): Addition of H_2O (0.1M phosphate)/DMSO-D_6 (2:3) – adjusted to pH 8.0

The resulting reaction mixtures were then analysed at intervals over several days by using ^{1}H NMR spectroscopy and HRMS (ESI positive mode).

Analysis of spectra at low pH (aq. DMSO-D$_6$) – Aliquots A–D

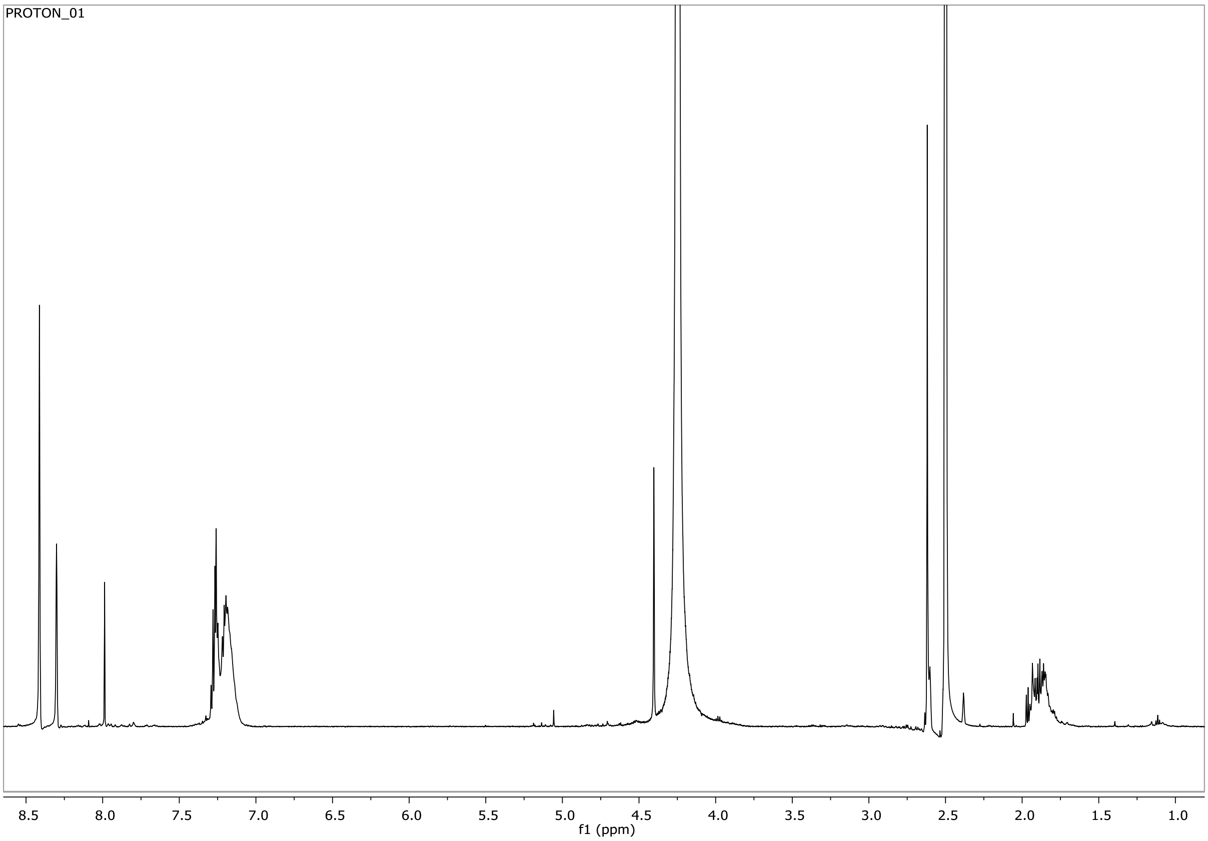

*Figure H24: ^{1}H NMR spectrum of the mixture derived from the photolysis of phenacylsulfide **3f** in D_2O/DMSO-D_6 (2:3) followed by addition of D_2O/DMSO-D_6 (2:3) with the pD adjusted to 4.0 (Aliquot A).*

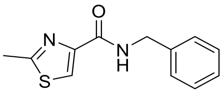

Exact Mass: 232.0670

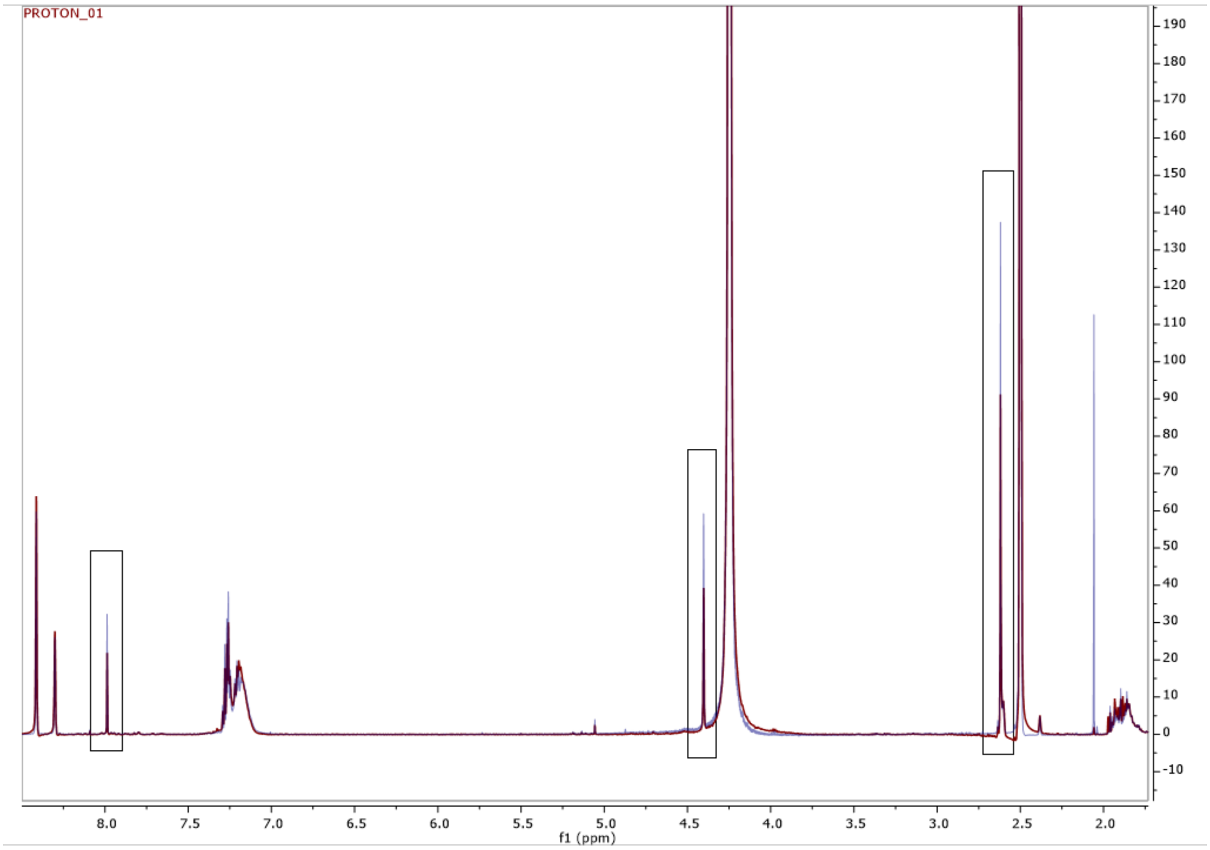

Figure H25: Overlapped ^{1}H NMR spectra of the mixture derived from the photolysis of phenacylsulfide ***3f*** *in D_2O/DMSO-D_6 (2:3) followed by addition of D_2O/DMSO-D_6 (2:3) with the pD adjusted to 4.0 (Aliquot A) as analysed over 9 days. Highlighted are the signals associated with the methyl, methylene and methine moieties of the thiazole product at 2.62, 4.40 and 7.99 ppm respectively, from day 0 (red) to day 9 (blue) as shown in the spectra.*

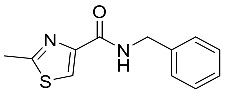

Exact Mass: 232.0670

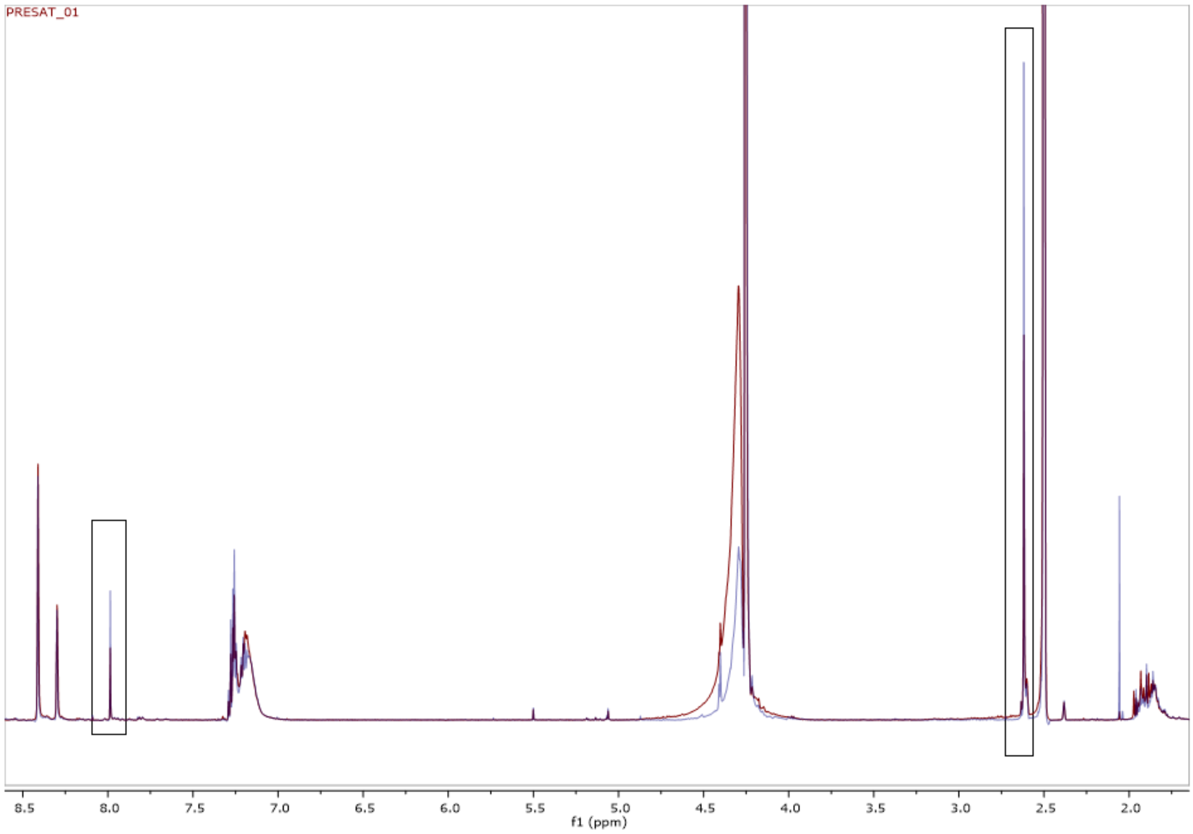

*Figure H26: Overlapped ^{1}H NMR spectra of the mixture derived from the photolysis of phenacylsulfide **3f** in D_2O/DMSO-D_6 (2:3) followed by addition of H_2O/DMSO-D_6 (2:3) with the pH meter reading adjusted to 3.6 (Aliquot B) as analysed over 9 days. Highlighted are the signals associated with the methyl and methine moieties of the thiazole product at 2.62 and 7.99 ppm respectively, from day 0 (red) to day 9 (blue) as shown in the spectra.*

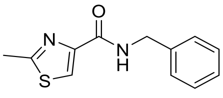

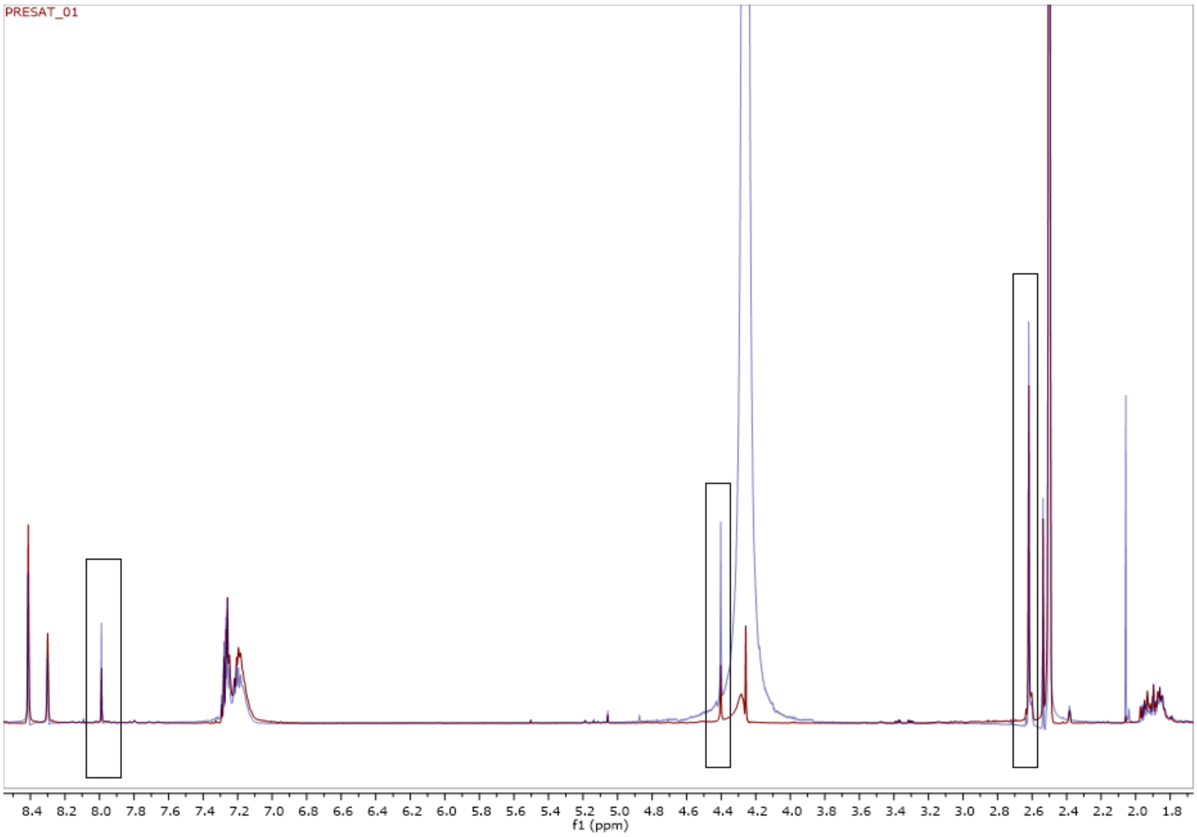

Figure H27: ^{1}H NMR spectra of the mixture derived from the photolysis of phenacylsulfide ***3f*** *in D_2O/DMSO-D_6 (2:3) followed by addition of D_2O (0.1 M phosphate)/DMSO-D_6 (2:3) with the pD adjusted to 4.0 (Aliquot C) as analysed over 9 days. Highlighted are the signals associated with the methyl, methylene and methine moieties of the thiazole product at 2.62, 4.40 and 7.99 ppm, respectively, from day 0 (red) to day 9 (blue) as shown in the spectra.*

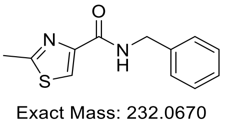

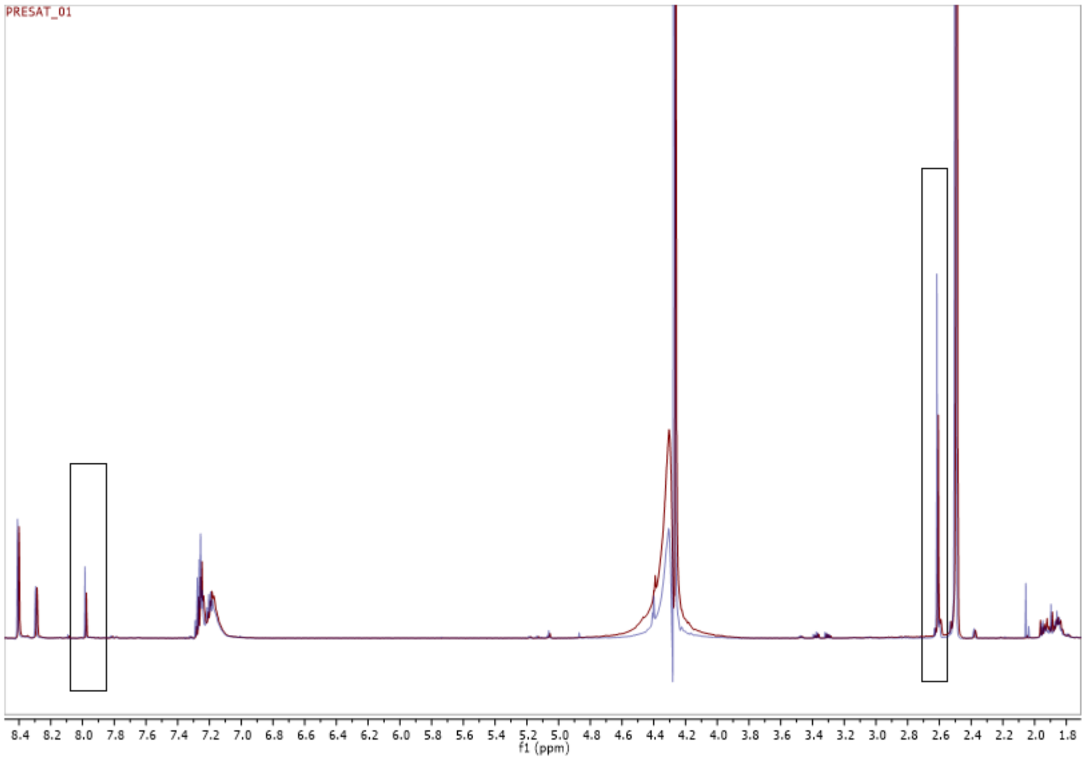

Figure H28: ^{1}H NMR spectra of the mixture derived from the photolysis of phenacylsulfide ***3f*** *in D_2O/DMSO-D_6 (2:3) followed by addition of H_2O (0.1 M phosphate)/DMSO-D_6 (2:3) with the pH meter reading adjusted to 3.6 (Aliquot D) as analysed over 9 days. Highlighted are the signals associated with the methyl and methine moieties of the thiazole product at 2.62 ppm and 7.99 ppm, respectively, from day 0 (red) to day 9 (blue) as shown in the spectra.*

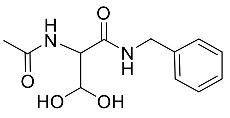

Exact Mass: 252.1110

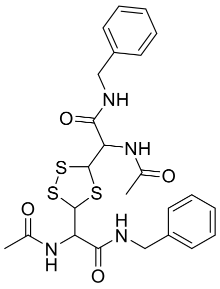

Exact Mass: 532.1273

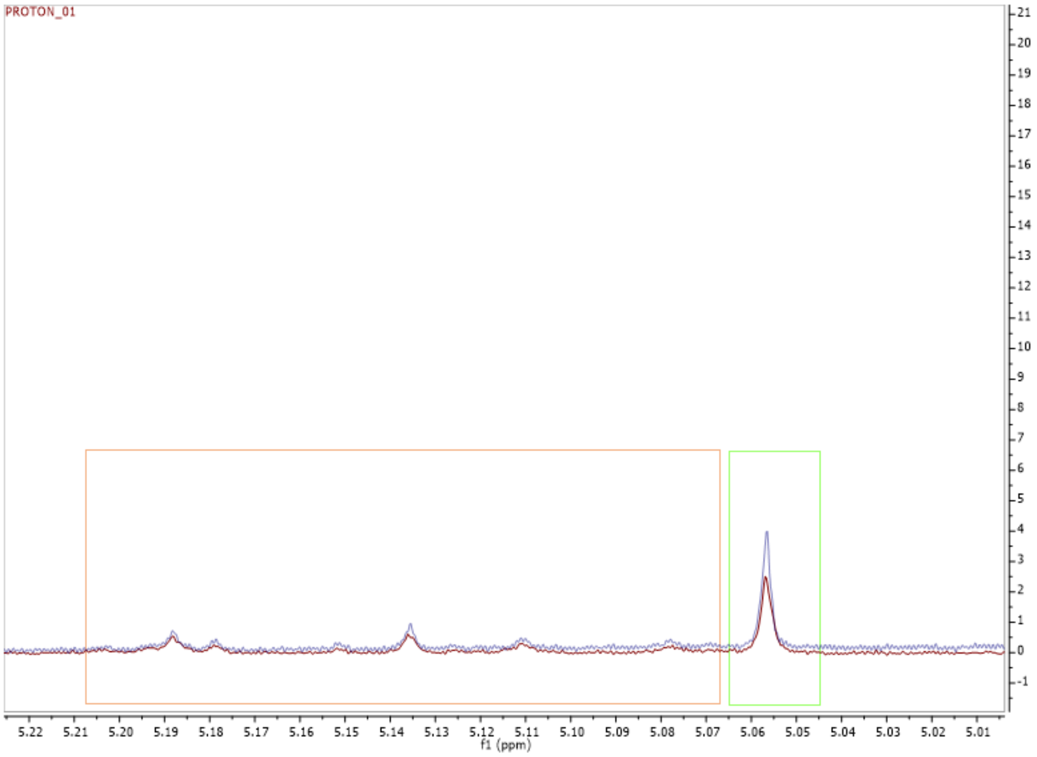

Figure H29: Overlapped and zoomed-in image of β-methine region of ^{1}H NMR spectra of the mixture derived from the photolysis of phenacylsulfide ***3f*** *in D_2O/DMSO-D_6 (2:3) followed by addition of D_2O/DMSO-D_6 (2:3) with the pD adjusted to 4.0 (Aliquot A) as analysed from day 0 (red) to day 9 (blue). Highlighted are the β-methine hydrogen nuclei of the gem-diol (green box) and 1,2,4-trithiolane (orange box) species. Note the lack of coupling owing to incorporation of deuterium into the α centre of both types of species.*

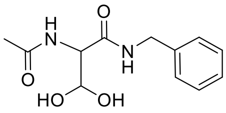

Exact Mass: 252.1110

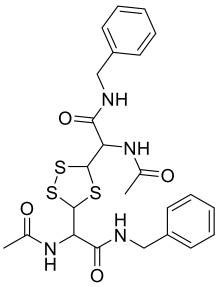

Exact Mass: 532.1273

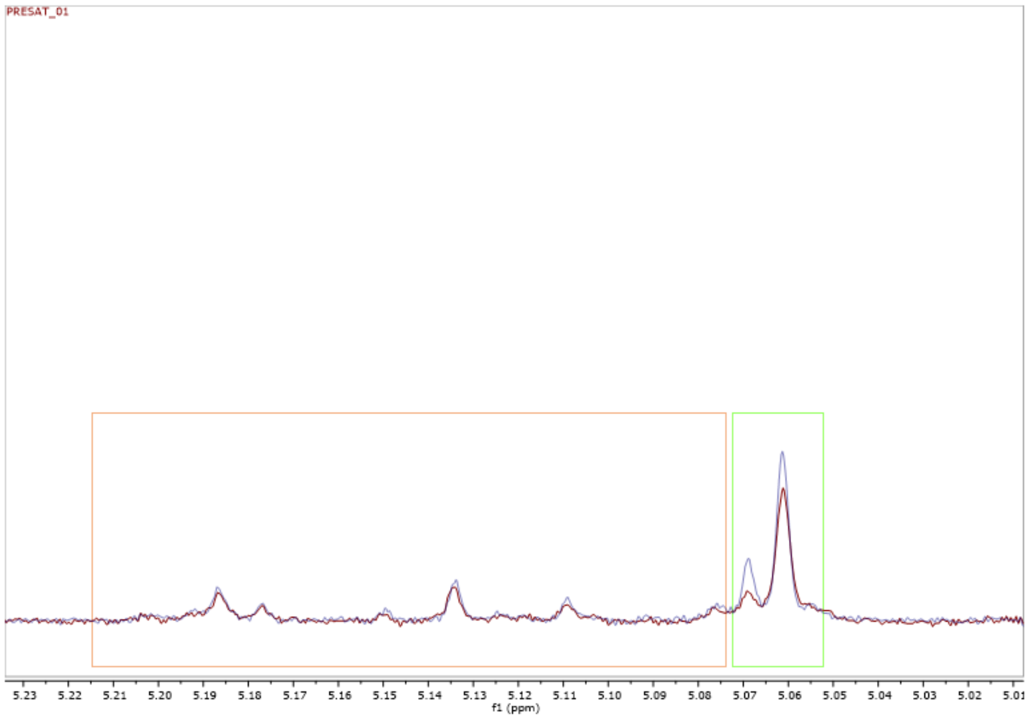

Figure H30: Overlapped and zoomed-in image of β-methine region of [1]*H NMR spectra of the mixture derived from the photolysis of phenacylsulfide* ***3f*** *in D_2O/DMSO-D_6 (2:3) followed by addition of H_2O/DMSO-D_6 (2:3) with the pH meter reading adjusted to 3.6 (Aliquot B) as analysed from day 0 (red) to day 9 (blue). Highlighted are the β methine hydrogen nuclei of the gem-diol(green box) and 1,2,4-trithiolane (orange box) species. The emerging peak at 5.07 ppm is assigned as part of a doublet associated with the gem-diol species where hydrogen has been incorporated into the α position.*

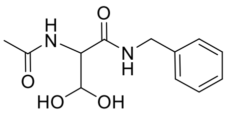

Exact Mass: 252.1110

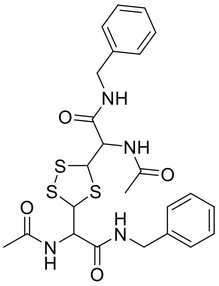

Exact Mass: 532.1273

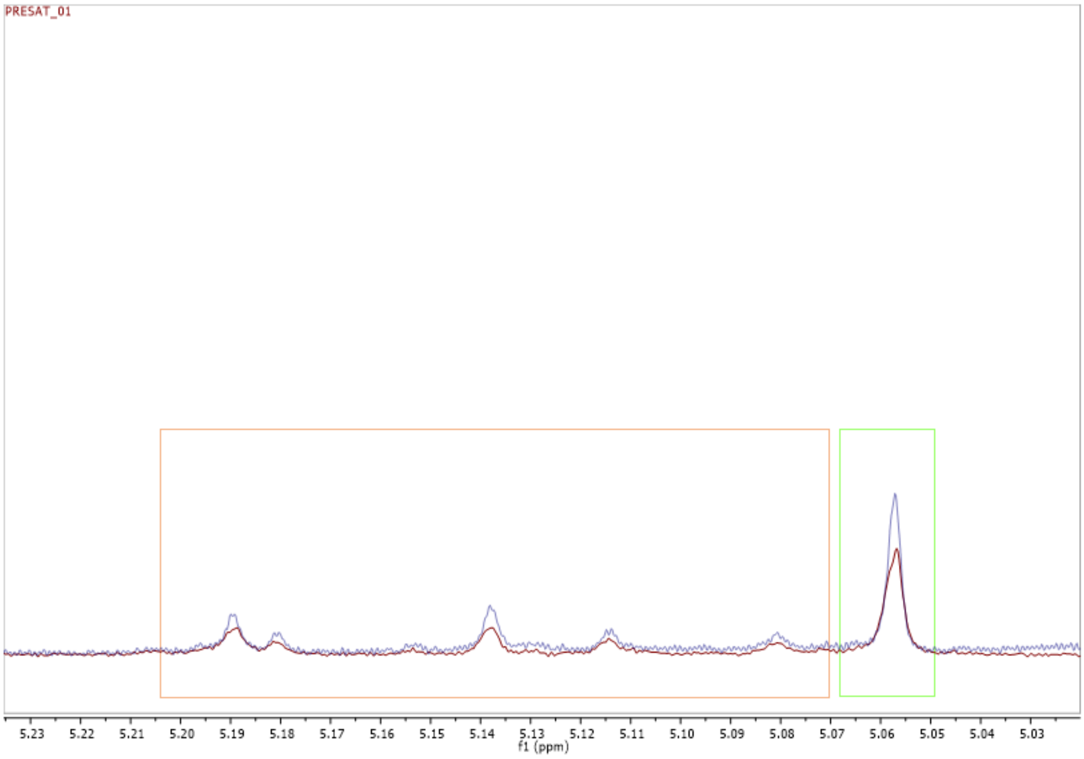

Figure H31: Overlapped and zoomed-in image of β-methine region of ^{1}H NMR spectra of the mixture derived from the photolysis of phenacylsulfide ***3f*** *in D_2O/DMSO-D_6 (2:3) followed by addition of D_2O (0.1 M phosphate)/DMSO-D_6 (2:3) with the pH meter reading adjusted to 3.6 (Aliquot C) as analysed from day 0 (red) to day 9 (blue). Highlighted are the β-methine hydrogen nuclei of the gem-diol (green box) and 1,2,4-trithiolane (orange box) species. Note the lack of coupling owing to incorporation of deuterium into the α centre of both types of species.*

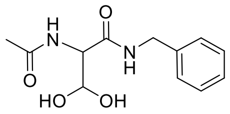

Exact Mass: 252.1110

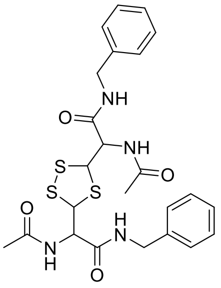

Exact Mass: 532.1273

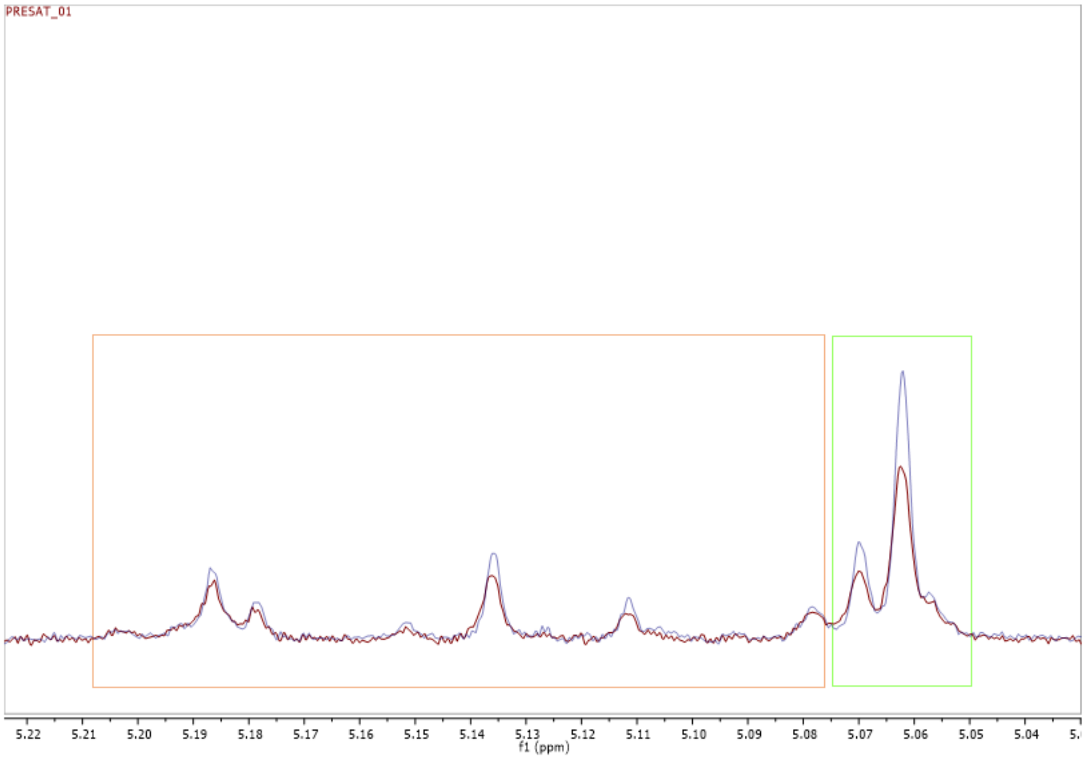

*Figure H32: Overlapped and zoomed-in image of β-methine region of ¹H NMR spectra of the mixture derived from the photolysis of phenacylsulfide **3f** in D_2O/DMSO-D_6 (2:3) followed by addition of H_2O (0.1 M phosphate)/DMSO-D_6 (2:3) with the pH meter reading adjusted to 3.6 (Aliquot D) as analysed from day 0 (red) to day 9 (blue). Highlighted are the β-methine hydrogen nuclei of the gem-diol (green box) and 1,2,4-trithiolane (orange box) species. The emerging peak at 5.07 ppm is assigned as part of a doublet associated with the gem-diol species where hydrogen has been incorporated into the α-position.*

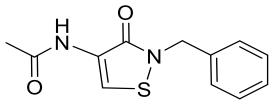

Exact Mass: 248.0619

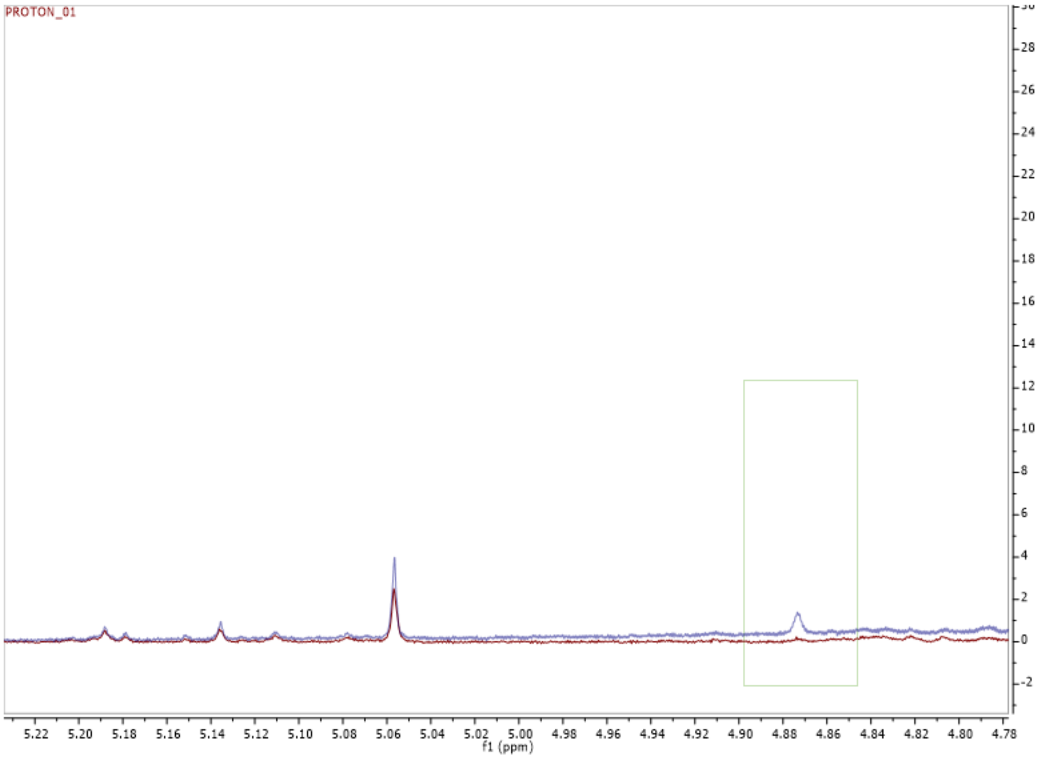

*Figure H33: Overlapped and zoomed-in image of ^{1}H NMR spectra of the mixture derived from the photolysis of phenacylsulfide **3f** in D_2O/DMSO-D_6 (2:3) followed by addition of D_2O /DMSO-D_6 (2:3) with the pD adjusted to 4 (Aliquot A) as analysed from day 0 (red) to day 9 (blue). Highlighted is the methylene signal associated with the isothiazolone (green box).*

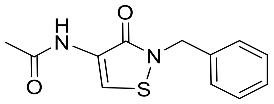

Exact Mass: 248.0619

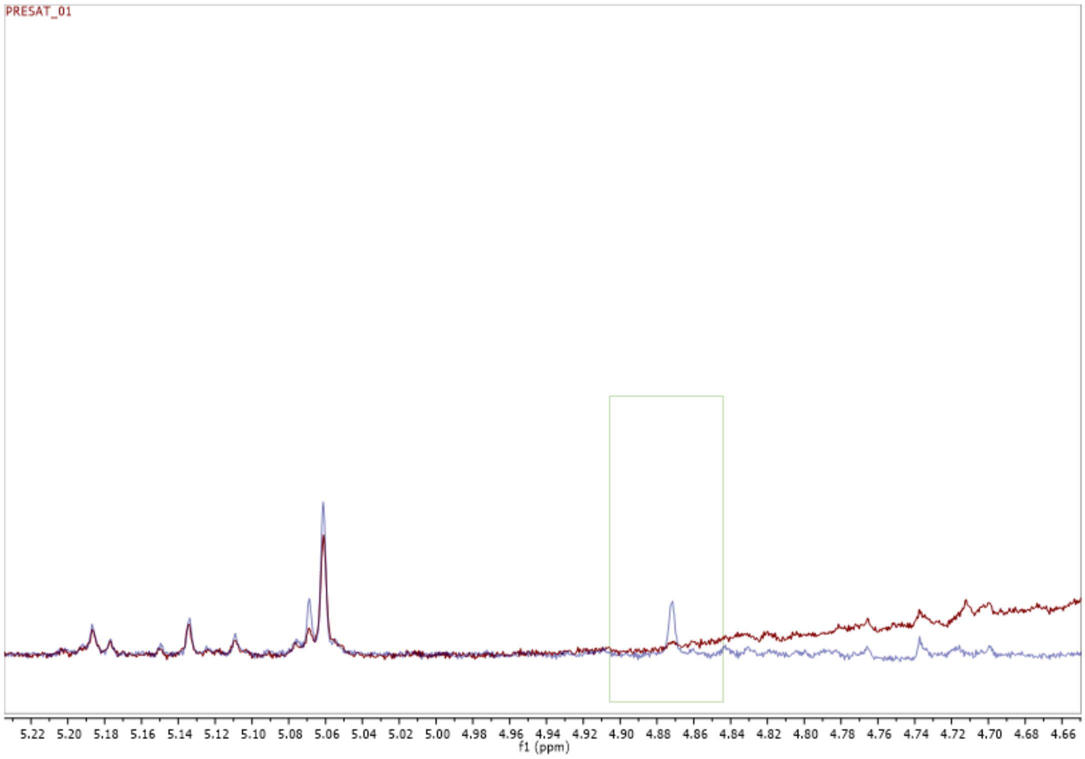

Figure H34: Overlapped and zoomed-in image of ^{1}H NMR spectra of the mixture derived from the photolysis of phenacylsulfide ***3f*** *in D_2O/DMSO-D_6 (2:3) followed by addition of H_2O /DMSO-D_6 (2:3) with the pH meter reading adjusted to 3.6 (Aliquot B) as analysed from day 0 (red) to day 9 (blue). Highlighted is the methylene signal associated with the isothiazolone (green box).*

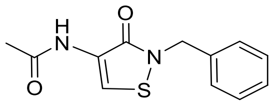

Exact Mass: 248.0619

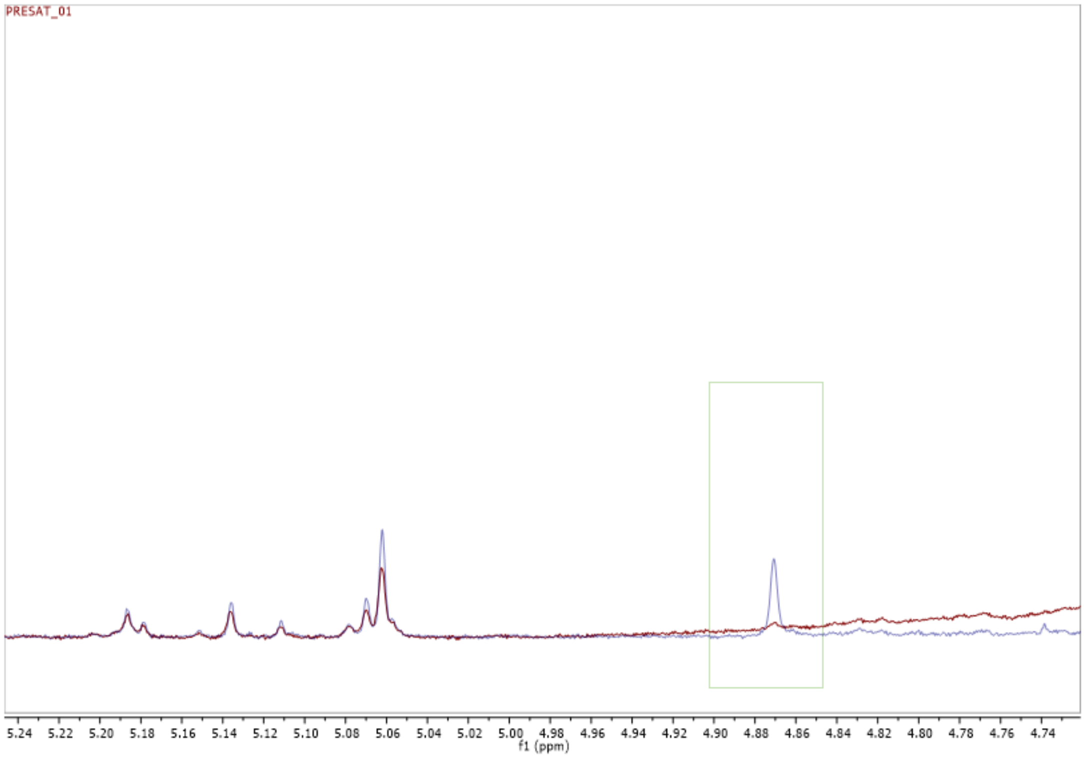

*Figure H35: Overlapped and zoomed-in image of ^{1}H NMR spectra of the mixture derived from the photolysis of phenacylsulfide **3f** in D_2O/DMSO-D_6 (2:3) followed by addition of H_2O (0.1 M phosphate)/DMSO-D_6 (2:3) with the pH meter reading adjusted to 3.6 (Aliquot D) as analysed from day 0 (red) to day 9 (blue). Highlighted is the methylene signal associated with the isothiazolone (green box).*

Analysis of spectra at high pH (aq. DMSO) – Aliquots E–H

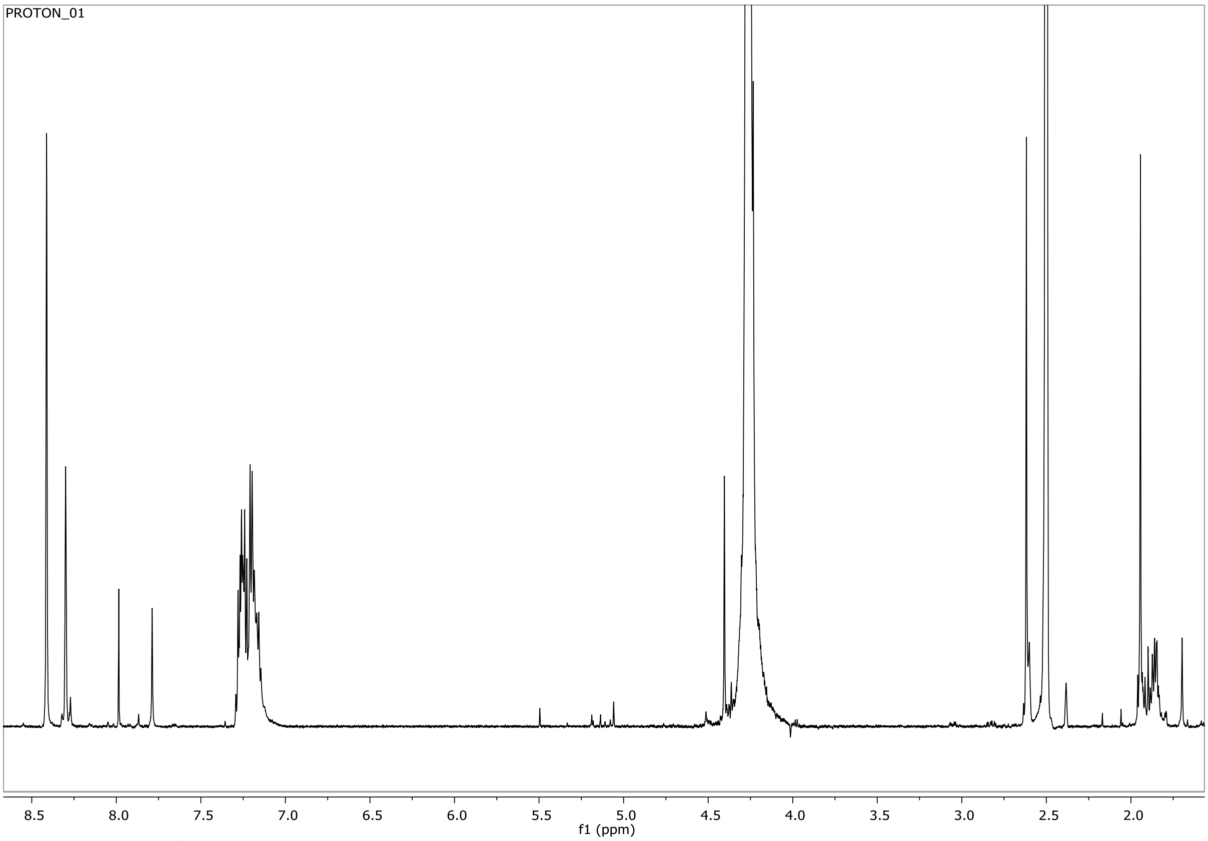

*Figure H36: ^{1}H NMR spectrum of the mixture derived from the photolysis of phenacylsulfide **3f** in D_2O/DMSO-D_6 (2:3) followed by addition of D_2O/DMSO-D_6 (2:3) with the pD adjusted to 8.0 (Aliquot E).*

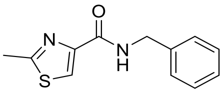

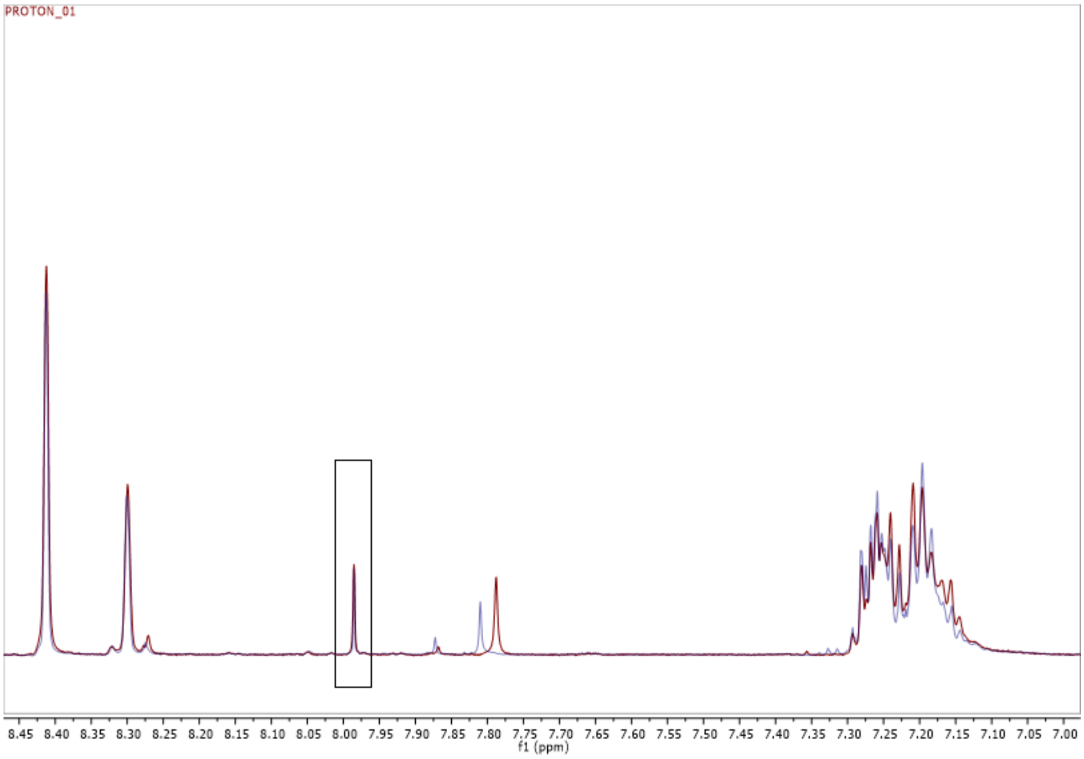

*Figure H37: Overlapped and zoomed-in image of ¹H NMR spectra of the mixture derived from the photolysis of phenacylsulfide **3f** in D_2O/DMSO-D_6 (2:3) followed by addition of D_2O/DMSO-D_6 (2:3) with the pD adjusted to 8 (Aliquot E) as analysed from day 0 (red) to day 9 (blue). Highlighted is the signal associated with the methine moiety of the thiazole product at 7.99 ppm (black box).*

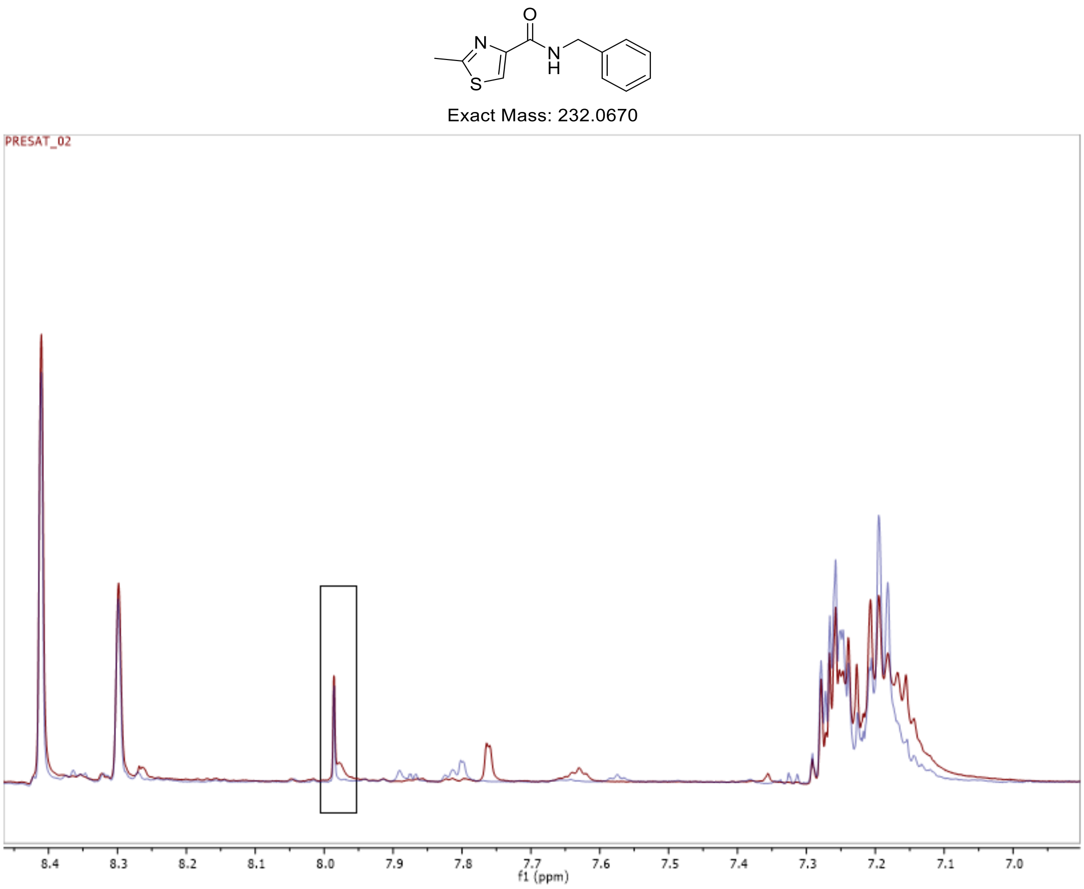

*Figure H38: Overlapped and zoomed-in image of ^{1}H NMR spectra of the mixture derived from the photolysis of phenacylsulfide **3f** in D_2O/DMSO-D_6 (2:3) followed by addition of H_2O/DMSO-D_6 (2:3) with the pH meter reading adjusted to 7.6 (Aliquot F) as analysed from day 0 (red) to day 9 (blue). Highlighted is the signal associated with the methine moiety of the thiazole product at 7.99 ppm (black box).*

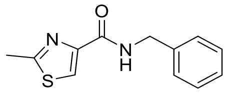

Exact Mass: 232.0670

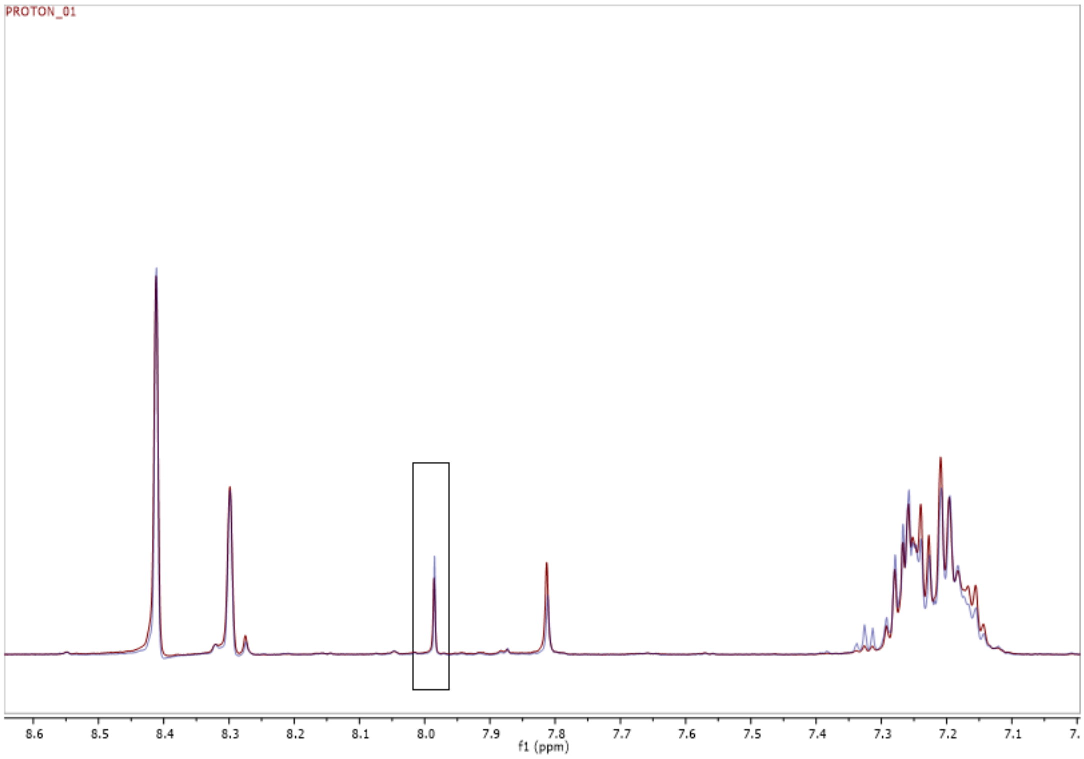

Figure H39: Overlapped and zoomed-in image of ^{1}H NMR spectra of the mixture derived from the photolysis of phenacylsulfide ***3f*** *in D_2O/DMSO-D_6 (2:3) followed by addition of buffered D_2O/DMSO-D_6 (2:3) with the pD adjusted to 8 (Aliquot G) as analysed from day 0 (red) to day 9 (blue). Highlighted is the signal associated with the methine moiety of the thiazole product at 7.99 ppm (black box).*

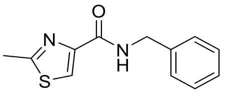

Exact Mass: 232.0670

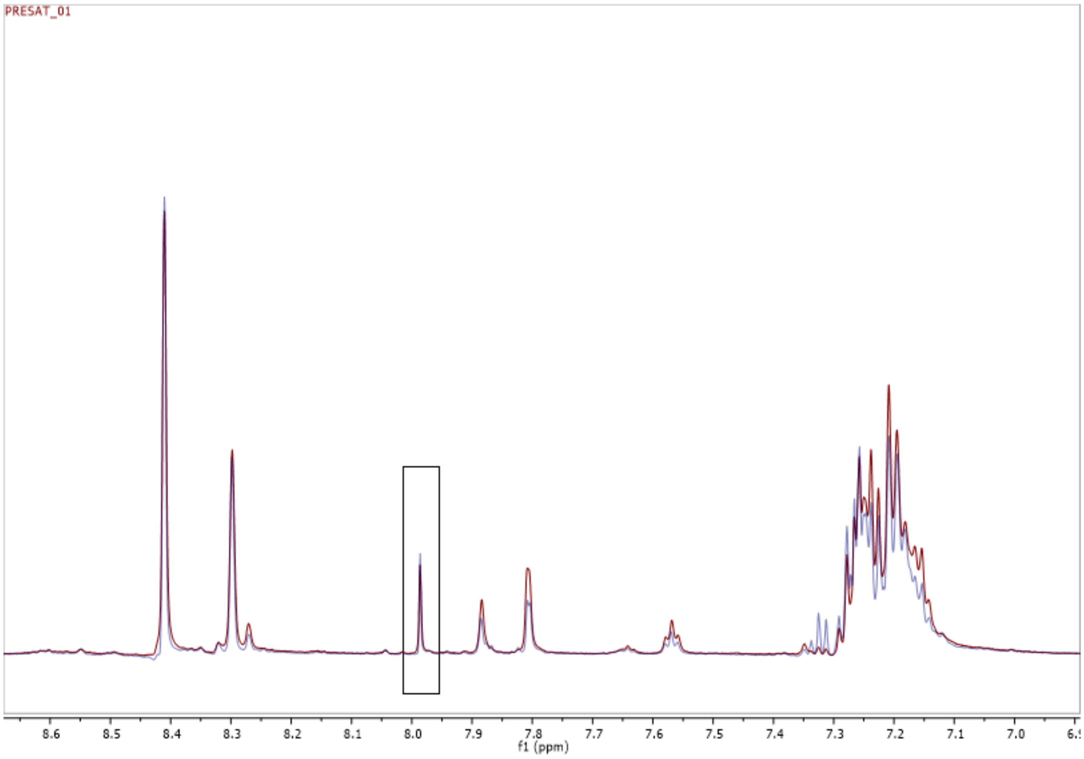

Figure H40: Overlapped and zoomed-in image of ^{1}*H NMR spectra of the mixture derived from the photolysis of phenacylsulfide* ***3f*** *in* D_2O*/DMSO-*D_6 *(2:3) followed by addition of buffered* H_2O*/DMSO-*D_6 *(2:3) with the pH meter reading adjusted to 7.6 (Aliquot H) as analysed from day 0 (red) to day 9 (blue). Highlighted is the signal associated with the methine moiety of the thiazole product at 7.99 ppm (black box).*

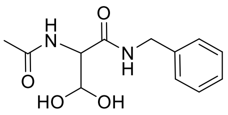

Exact Mass: 252.1110

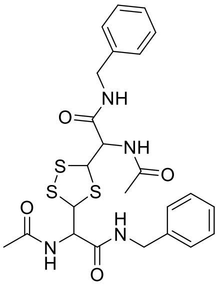

Exact Mass: 532.1273

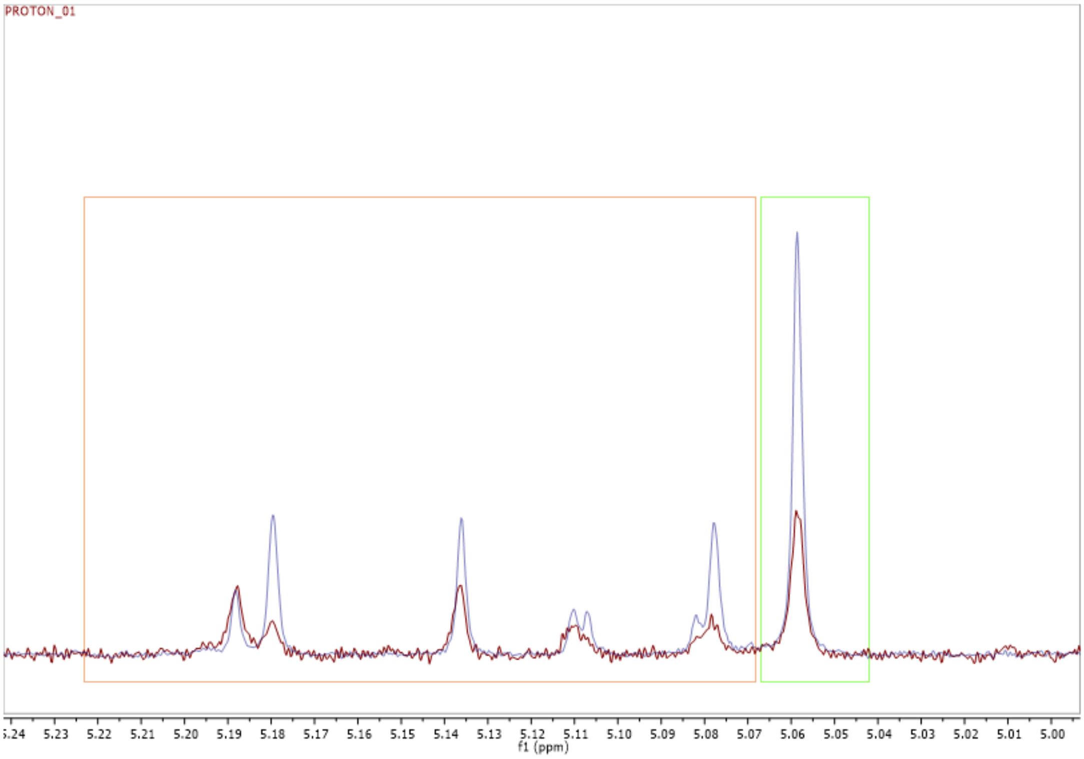

*Figure H41: Overlapped and zoomed-in image of β-methine region of ¹H NMR spectra of the mixture derived from the photolysis of phenacylsulfide **3f** in D_2O/DMSO-D_6 (2:3) followed by addition of D_2O/DMSO-D_6 (2:3) with the pD adjusted to 8.0 (Aliquot E) as analysed from day 0 (red) to day 9 (blue). Highlighted are the β-methine hydrogen nuclei of the gem-diol (green box) and 1,2,4-trithiolane (orange box) species. Note the lack of coupling owing to incorporation of deuterium into the α centre of both types of species.*

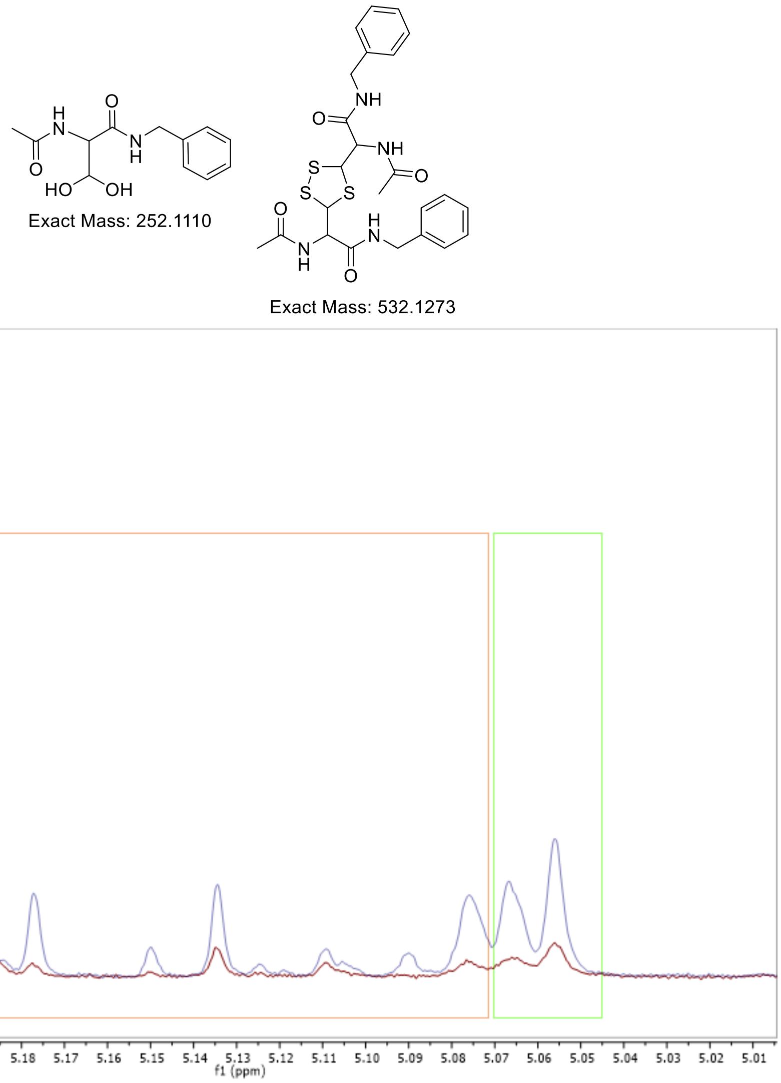

*Figure H42: Overlapped and zoomed-in image of β-methine region of ^{1}H NMR spectra of the mixture derived from the photolysis of phenacylsulfide **3f** in D_2O/DMSO-D_6 (2:3) followed by addition of H_2O/DMSO-D_6 (2:3) with the pH meter reading adjusted to 7.6 (Aliquot F) as analysed from day 0 (red) to day 9 (blue). Highlighted are the β-methine hydrogen nuclei of the gem-diol (green box) and 1,2,4-trithiolane (orange box) species. Compare with all-D_2O sample (Figure 20) and note the more complicated region, which we can be characterised as the generation of species with a-hydrogen nuclei, thus leading to the appearance of additional doublets.*

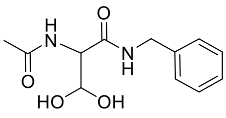

Exact Mass: 252.1110

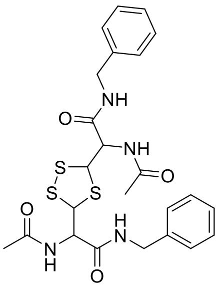

Exact Mass: 532.1273

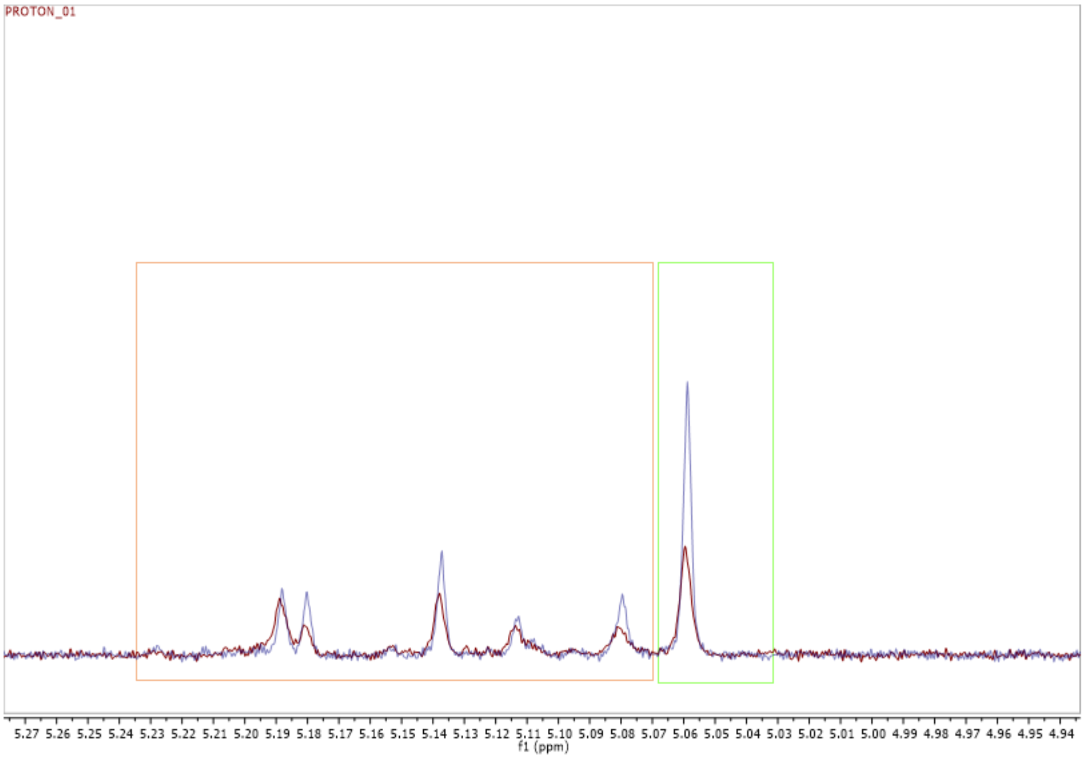

Figure H43: Overlapped and zoomed-in image of β-methine region of ^{1}H NMR spectra of the mixture derived from the photolysis of phenacylsulfide ***3f*** *in D_2O/DMSO-D_6 (2:3) followed by addition of D_2O (0.1 M phosphate)/DMSO-D_6 (2:3) with the pD adjusted to 8.0 (Aliquot G) as analysed from day 0 (red) to day 9 (blue). Highlighted are the β-methine hydrogen nuclei of the gem-diol (green box) and 1,2,4-trithiolane (orange box) species.*

Note the lack of coupling owing to incorporation of deuterium into the α centre of both types of species.

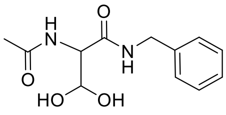

Exact Mass: 252.1110

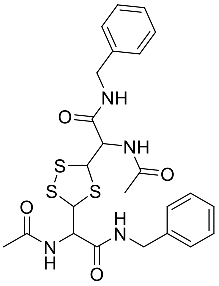

Exact Mass: 532.1273

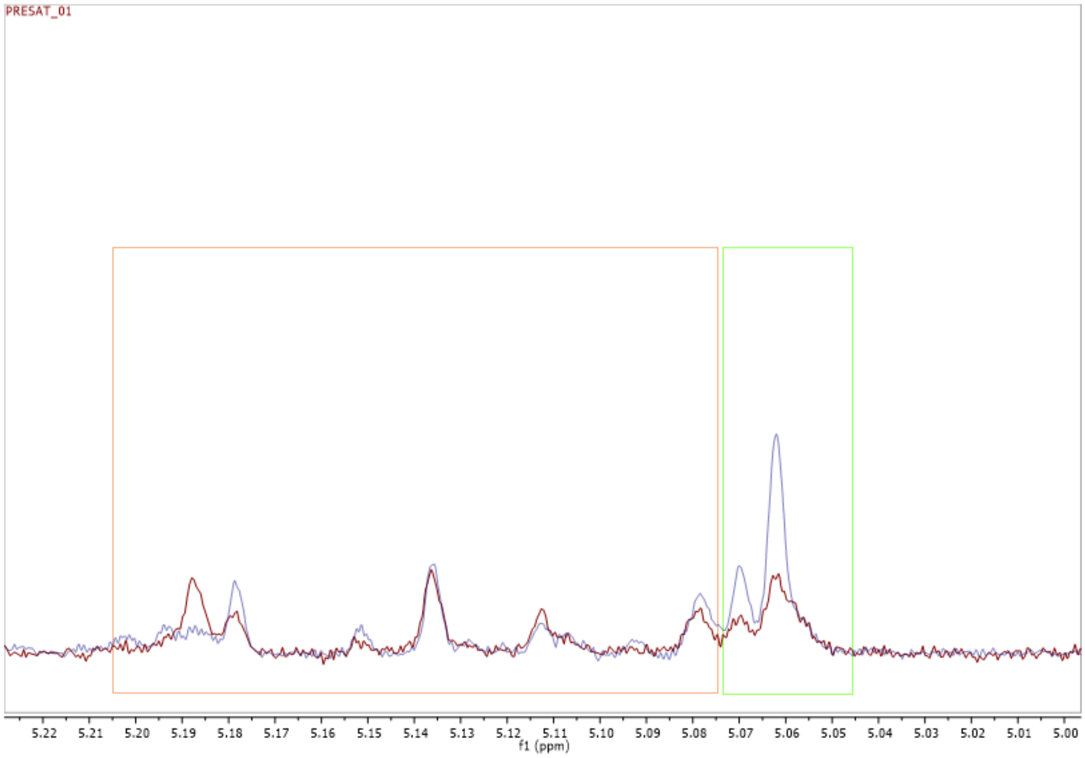

*Figure H44: Overlapped and zoomed-in image of β-methine region of ^{1}H NMR spectra of the mixture derived from the photolysis of phenacylsulfide **3f** in D_2O/DMSO-D_6 (2:3) followed by addition of H_2O (0.1M phosphate)/DMSO-D_6 (2:3) with the pH meter reading adjusted to 7.6 (Aliquot H) as analysed from day 0 (red) to day 9 (blue). Highlighted are the β-methine hydrogen nuclei of the gem-diol (green box) and 1,2,4-trithiolane (orange box) species. Compare with all-D_2O sample (Figure H22) and note the more complicated region, which we can be characterised as the generation of species with a-hydrogen nuclei, thus leading to the appearance of doublets.*

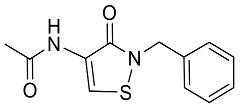

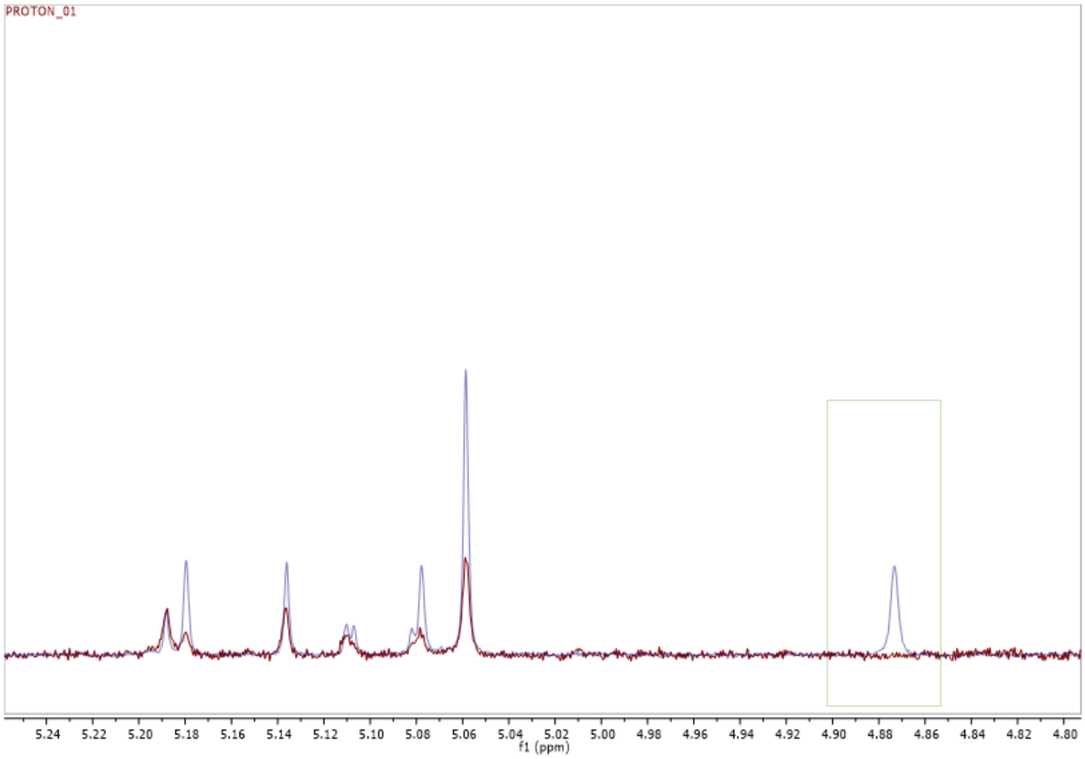

*Figure H45: Overlapped and zoomed-in image of ^{1}H NMR spectra of the mixture derived from the photolysis of phenacylsulfide **3f** in D_2O/DMSO-D_6 (2:3) followed by addition of D_2O/DMSO-D_6 (2:3) with the pD adjusted to 8.0 (Aliquot E) as analysed from day 0 (red) to day 9 (blue). Highlighted is the methylene signal associated with the isothiazolone (green box).*

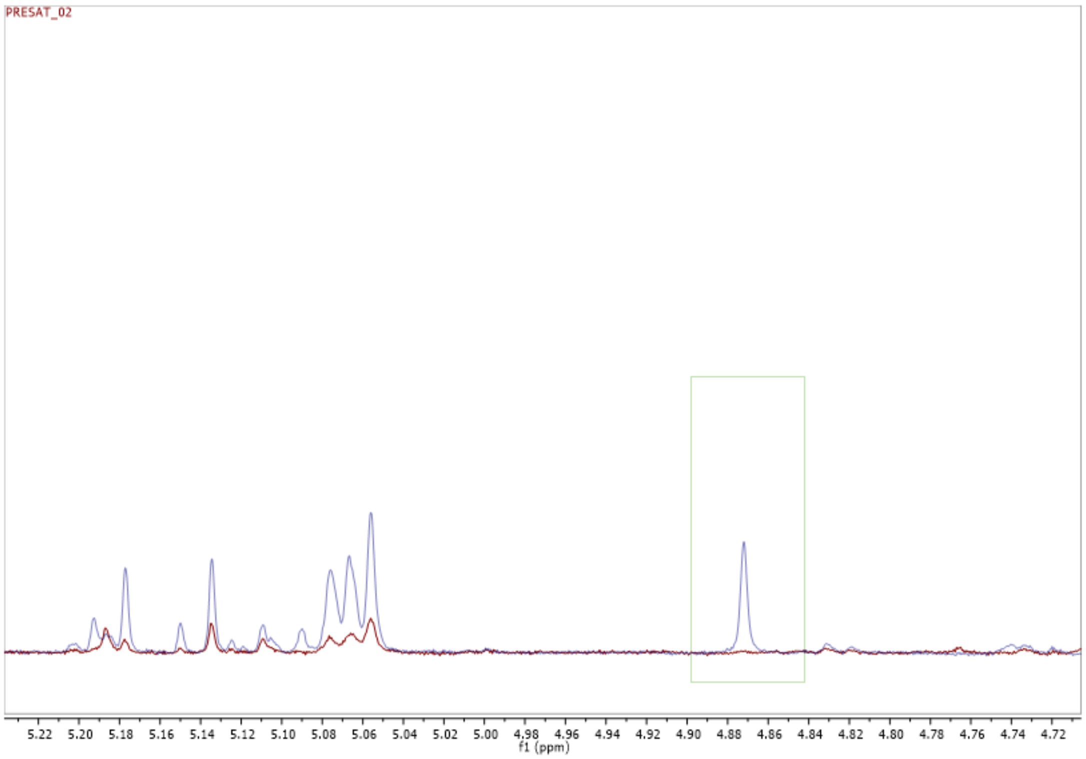

*Figure H46: Overlapped and zoomed-in image of ^{1}H NMR spectra of the mixture derived from the photolysis of phenacylsulfide **3f** in D_2O/DMSO-D_6 (2:3) followed by addition of H_2O/DMSO-D_6 (2:3) with the pH meter reading adjusted to 7.6 (Aliquot F) as analysed from day 0 (red) to day 9 (blue). Highlighted is the methylene signal associated with the isothiazolone (green box).*

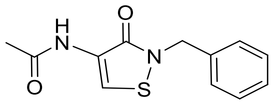

Exact Mass: 248.0619

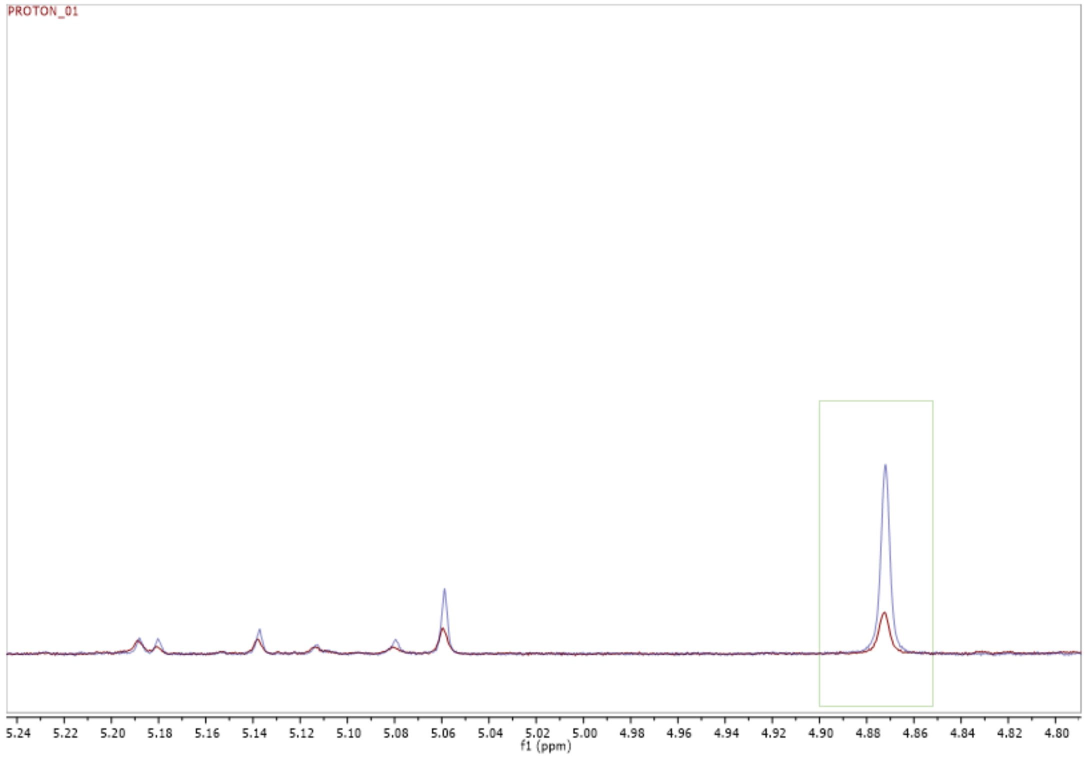

Figure H47: Overlapped and zoomed-in image of ^{1}H NMR spectra of the mixture derived from the photolysis of phenacylsulfide ***3f*** *in D_2O/DMSO-D_6 (2:3) followed by addition of D_2O (0.1 M phosphate)/DMSO-D_6 (2:3) with the pD adjusted to 8.0 (Aliquot G) as analysed from day 0 (red) to day 9 (blue). Highlighted is the methylene signal associated with the isothiazolone (green box).*

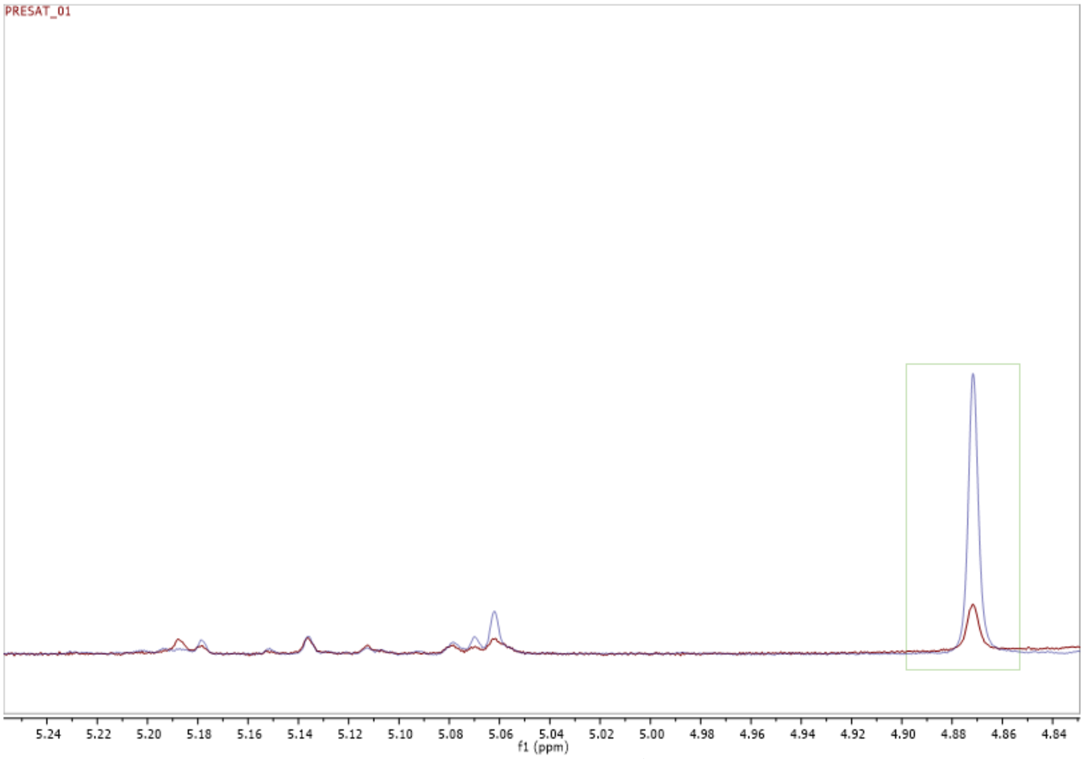

Figure H48: Overlapped and zoomed-in image of the ^{1}H NMR spectra of the mixture derived from the photolysis of phenacylsulfide ***3f*** *in D_2O/DMSO-D_6 (2:3) followed by addition of H_2O (0.1 M phosphate)/DMSO-D_6 (2:3) with the pH meter reading adjusted to 7.6 (Aliquot H) as analysed from day 0 (red) to day 9 (blue). Highlighted is the methylene signal associated with the isothiazolone (green box).*

Evolution of reaction species in each aliquot based on integration of characteristic signals relative to the methyl region from 2.61-1.71 ppm

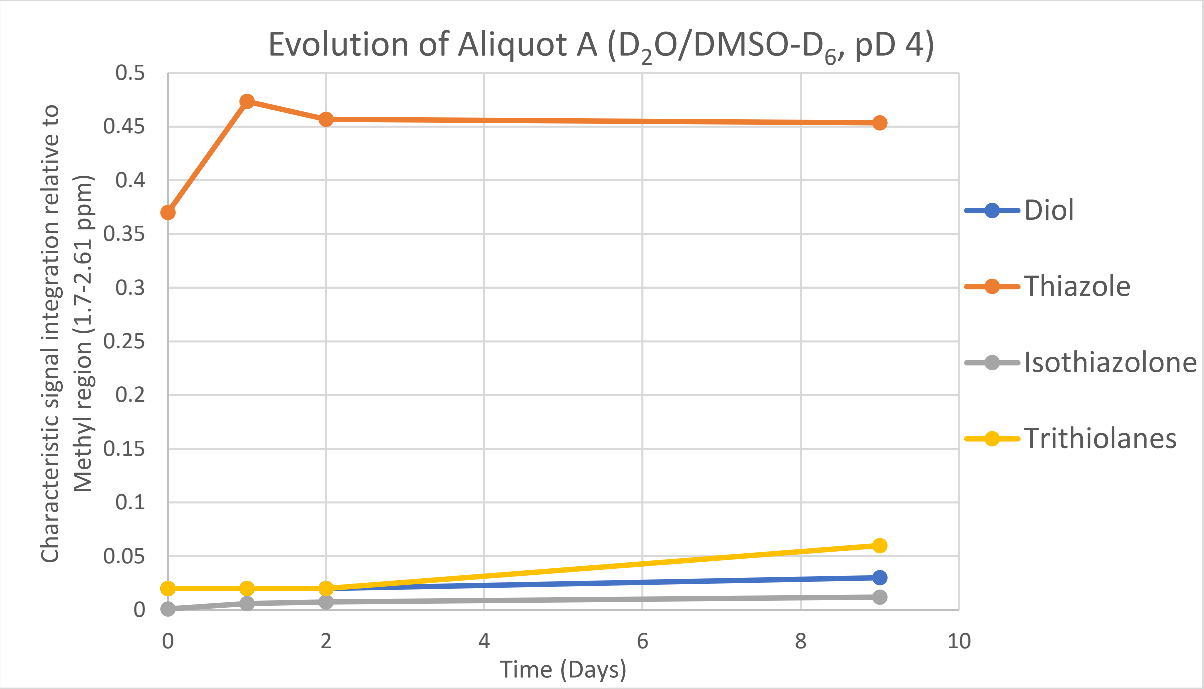

Time (Days)	Ketone (8.41 ppm)	Ketone (8.3 ppm)	Diol (5.06 ppm)	1,2,4-Trithiolanes (5.21–5.04 ppm)	Isothiazolone (4.87 ppm)	Thiazole (2.61 ppm)
T0	1	0.51	0.02	0.02	0.001	0.37
T1	1.03	0.53	0.02	0.02	0.006	0.47
T2	1	0.51	0.02	0.02	0.0075	0.46
T9	0.89	0.46	0.03	0.06	0.012	0.45

Figure H49: The evolution of the reaction mixture derived from the photolysis of phenacylsulfide ***3f*** *in D_2O/DMSO-D_6 (2:3) followed by addition of D_2O/DMSO-D_6 (2:3) with the pD adjusted to 4.0 (Aliquot A) as analysed over 9 days. The integration of a methine CH signal for the diol and trithiolanes are calculated relative to the methyl region from 1.7–2.6 ppm. The integration of the methylene CH_2 of the isothiazolone and the methyl CH_3 of the thiazole are also calculated relative to the same region. Table of corresponding data shown below graph. Note: integral values for both the methylene CH_2 of the isothiazolone and the methyl CH_3 of the thiazole are divided by 2 and 3 respectively, to provide the value of a single proton for each species.*

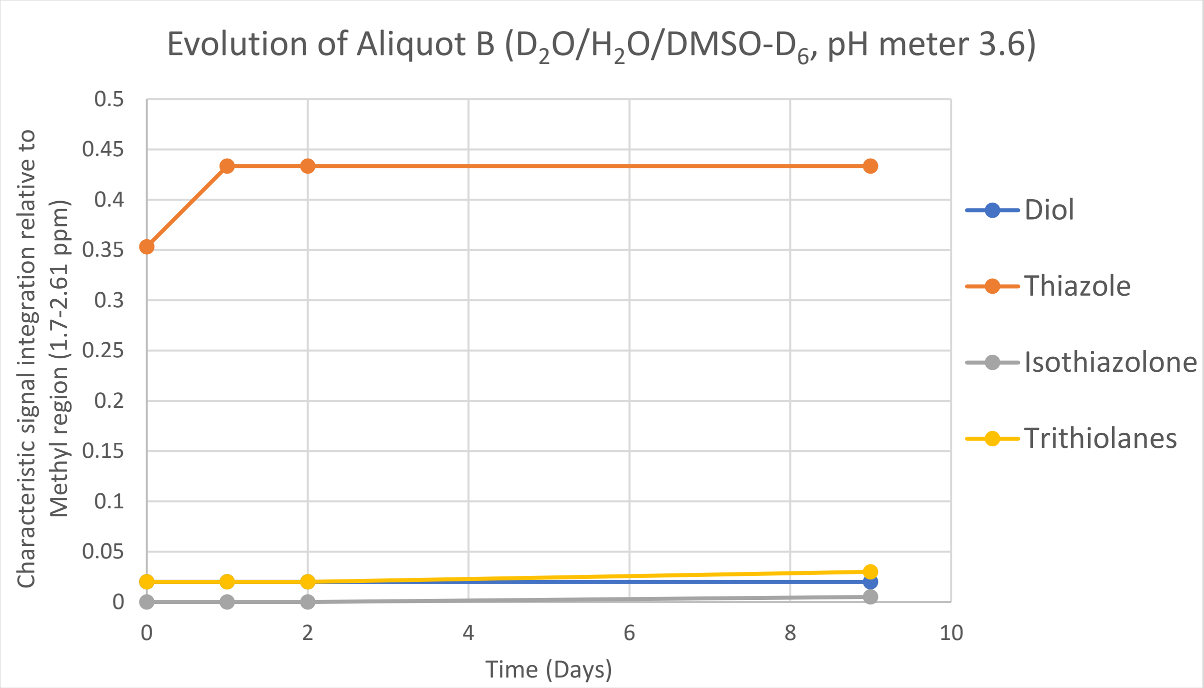

Time (Days)	Ketone (8.41 ppm)	Ketone (8.3 ppm)	Diol (5.06 ppm	1,2,4-Trithiolanes (5.21–5.04 ppm)	Isothiazolone (4.87 ppm)	Thiazole (2.61 ppm)
T0	0.98	0.51	0.02	0.02	0	0.35
T1	0.93	0.50	0.02	0.02	0	0.43
T2	0.89	0.48	0.02	0.02	0	0.43
T9	0.82	0.44	0.02	0.03	0.005	0.43

*Figure H50: The evolution of the reaction mixture derived from the photolysis of phenacylsulfide **3f** in D_2O/DMSO-D_6 (2:3) followed by addition of H_2O/DMSO-D_6 (2:3) with the pH meter reading adjusted to 3.6 (Aliquot B) as analysed over 9 days The integration of a methine CH signal for the diol and trithiolanes are calculated relative to the methyl region from 1.7–2.6 ppm. The integration of the methylene CH_2 of the isothiazolone and the methyl CH_3 of the thiazole are also calculated relative to the same region. Table of corresponding data shown below graph. Note: integral values for both the methylene CH_2 of the isothiazolone and the methyl CH_3 of the thiazole are divided by 2 and 3 respectively, to provide the value of a single proton for each species.*

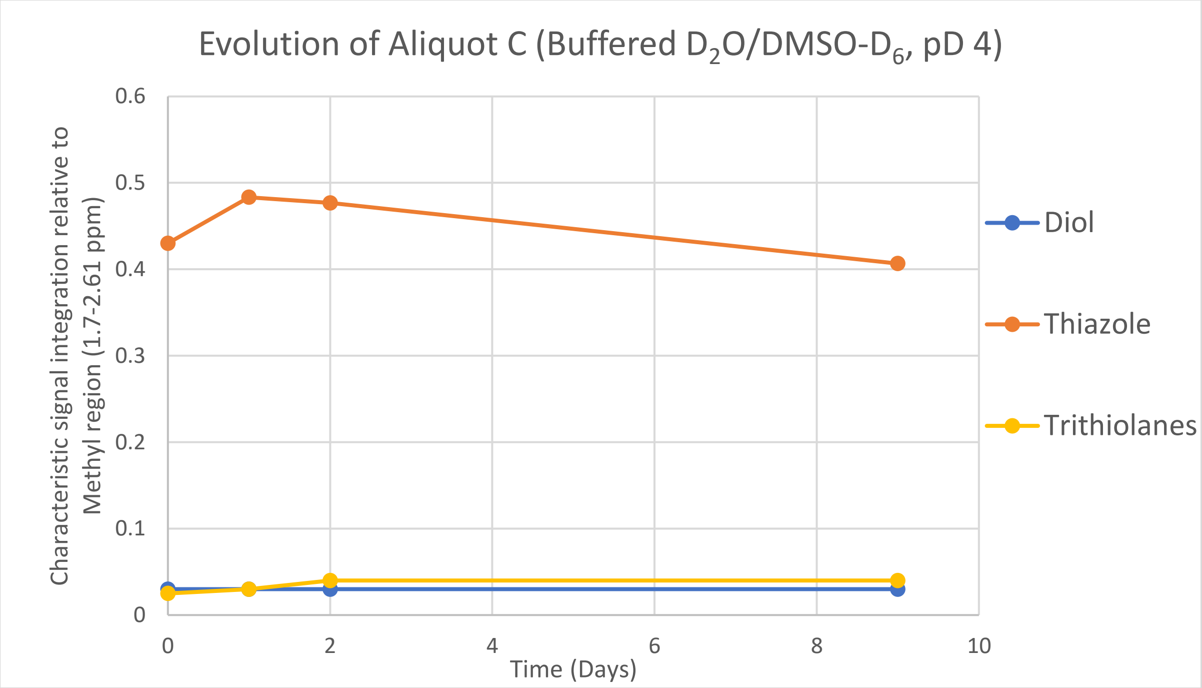

Time (Days)	Ketone (8.41 ppm)	Ketone (8.3 ppm)	Diol (5.06 ppm	1,2,4-Trithiolanes (5.21–5.04 ppm)	Isothiazolone (4.87 ppm)	Thiazole (2.61 ppm)
T0	0.92	0.47	0.03	0.03	0	0.43
T1	0.86	0.45	0.03	0.03	0	0.48
T2	0.87	0.44	0.03	0.03	0	0.49
T9	0.8	0.38	0.03	0.03	0	0.41

Figure H51: The evolution of the reaction mixture derived from the photolysis of phenacylsulfide ***3f*** *in D_2O/DMSO-D_6 (2:3) followed by addition of D_2O (0.1 M phosphate)/DMSO-D_6 (2:3) with the pD adjusted to 4.0 (Aliquot C) as analysed over 9 days. The integration of a methine CH signal for the diol, and trithiolanes are calculated relative to the methyl region from 1.7–2.6 ppm. The integration of the methylene CH_2 of the isothiazolone and the methyl CH_3 of the thiazole are also calculated relative to the same region. Table of corresponding data shown below graph. Note: integral values for both the methylene CH_2 of the isothiazolone and the methyl CH_3 of the thiazole are divided by 2 and 3 respectively, to provide the value of a single proton for each species.*

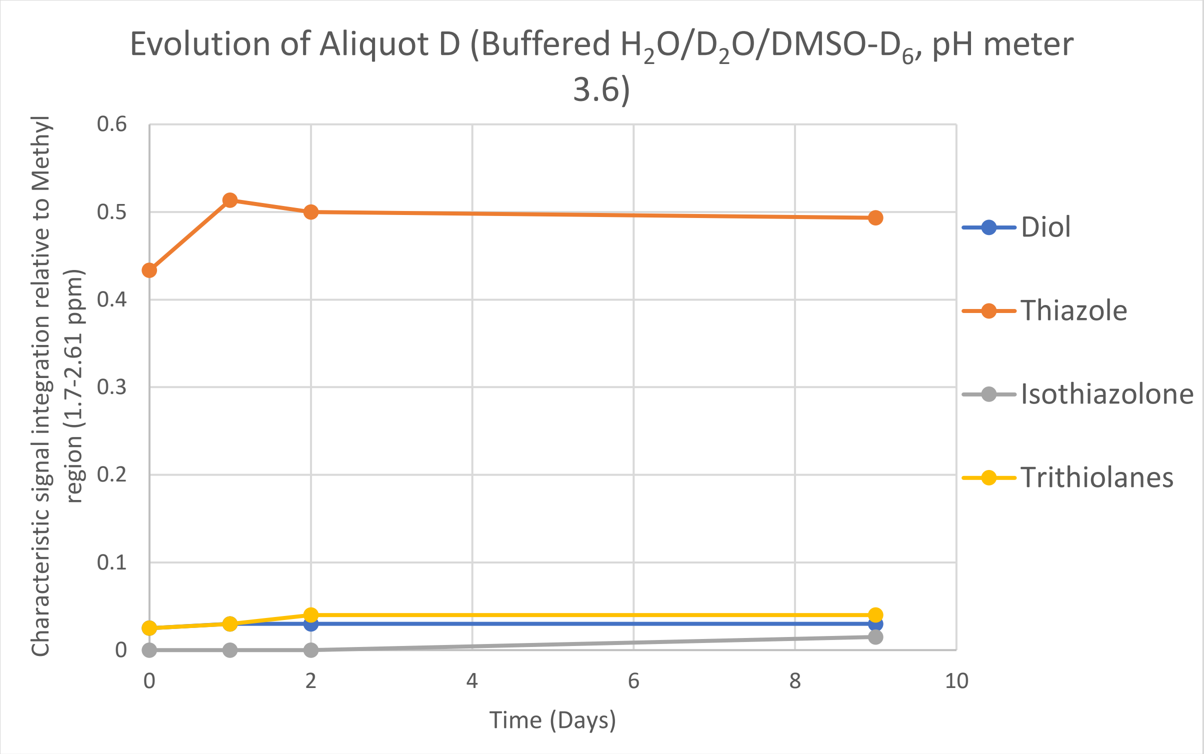

Time (Days)	Ketone (8.41 ppm)	Ketone (8.3 ppm)	Diol (5.06 ppm)	1,2,4-Trithiolanes (5.21–5.04 ppm)	Isothiazolone (4.87 ppm)	Thiazole (2.61 ppm)
T0	0.79	0.42	0.025	0.025	0	0.43
T1	0.83	0.45	0.03	0.03	0	0.51
T2	0.85	0.46	0.03	0.04	0	0.5
T9	0.79	0.43	0.03	0.04	0.015	0.49

*Figure H52: The evolution of the reaction mixture derived from the photolysis of phenacylsulfide **3f** in D_2O/DMSO-D_6 (2:3) followed by addition of H_2O (0.1 M phosphate)/DMSO-D_6 (2:3) with the pH meter reading adjusted to 3.6 (Aliquot D) as analysed over 9 days. The integration of a methine CH signal for the diol, and trithiolanes are calculated relative to the methyl region from 1.7–2.6 ppm. The integration of the methylene CH_2 of the isothiazolone and the methyl CH_3 of the thiazole are also calculated relative to the same region. Table of corresponding data shown below graph. Note: integral values for both the methylene CH_2 of the isothiazolone and the methyl CH_3 of the thiazole are divided by 2 and 3 respectively, to provide the value of a single proton for each species.*

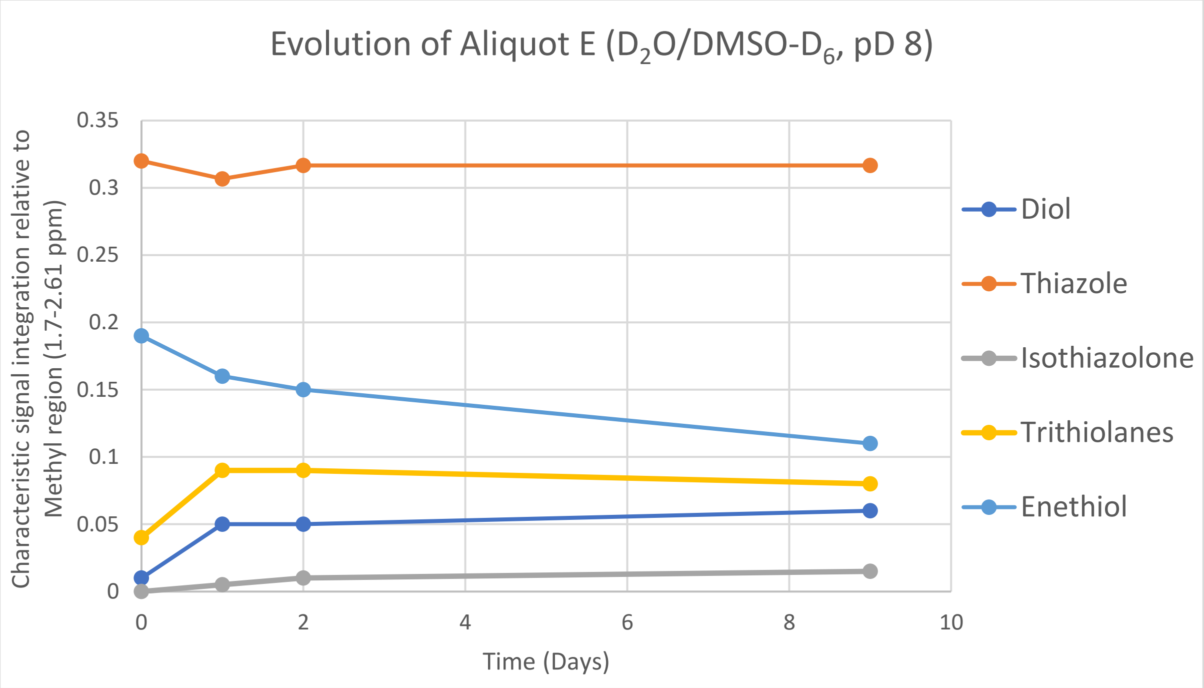

Time (Days)	Ketone (8.41 ppm)	Ketone (8.3 ppm)	Enethiol (7.8 ppm)	Diol (5.06 ppm)	1,2,4-Trithiolanes (5.21–5.04 ppm)	Isothiazolone (4.87 ppm)	Thiazole (2.61 ppm)
T0	1.18	0.63	0.19	0.01	0.04	0	0.32
T1	1.11	0.55	0.16	0.05	0.09	0.005	0.31
T2	1.02	0.52	0.15	0.05	0.09	0.01	0.32
T9	0.96	0.5	0.11	0.06	0.08	0.015	0.32

*Figure H53: The evolution of the reaction mixture derived from the photolysis of phenacylsulfide **3f** in D_2O/DMSO-D_6 (2:3) followed by addition of D_2O/DMSO-D_6 (2:3) with the pD adjusted to 8.0 (Aliquot E) as analysed over 9 days. The integration of a methine CH signal for the diol, and trithiolanes are calculated relative to the methyl region from 1.7–2.6 ppm. The integration of the methylene CH_2 of the isothiazolone and the methyl CH_3 of the thiazole are also calculated relative to the same region. Table of corresponding data shown below graph. Note: integral values for both the methylene CH_2 of the isothiazolone and the methyl CH_3 of the thiazole are divided by 2 and 3 respectively, to provide the value of a single proton for each species.*

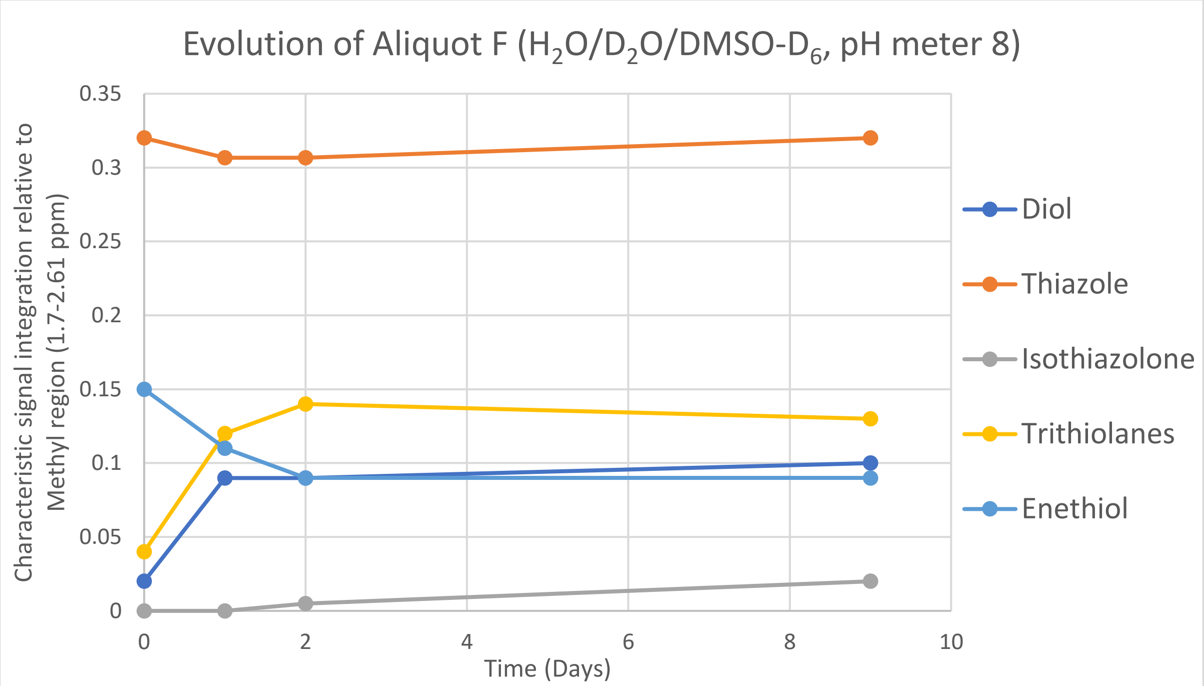

Time (Days)	Ketone (8.41 ppm)	Ketone (8.3 ppm)	Enethiol (7.8 ppm)	Diol (5.06 ppm)	1,2,4-Trithiolanes (5.21–5.04 ppm)	Isothiazolone (4.87 ppm)	Thiazole (2.61 ppm)
T0	1.02	0.56	0.15	0.02	0.04	0	0.32
T1	0.93	0.49	0.11	0.09	0.12	0	0.31
T2	0.91	0.53	0.09	0.09	0.14	0.005	0.31
T9	0.85	0.49	0.09	0.1	0.13	0.02	0.32

Figure H54: The evolution of the reaction mixture derived from the photolysis of phenacylsulfide ***3f*** *in D_2O/DMSO-D_6 (2:3) followed by addition of H_2O/DMSO-D_6 (2:3) with the pH meter reading adjusted to 7.6 (Aliquot F) as analysed over 9 days. The integration of a methine CH signal for the diol, and trithiolanes are calculated relative to the methyl region from 1.7–2.6 ppm. The integration of the methylene CH_2 of the isothiazolone and the methyl CH_3 of the thiazole are also calculated relative to the same region. Table of corresponding data shown below graph. Note: integral values for both the methylene CH_2 of the isothiazolone and the methyl CH_3 of the thiazole are divided by 2 and 3 respectively, to provide the value of a single proton for each species.*

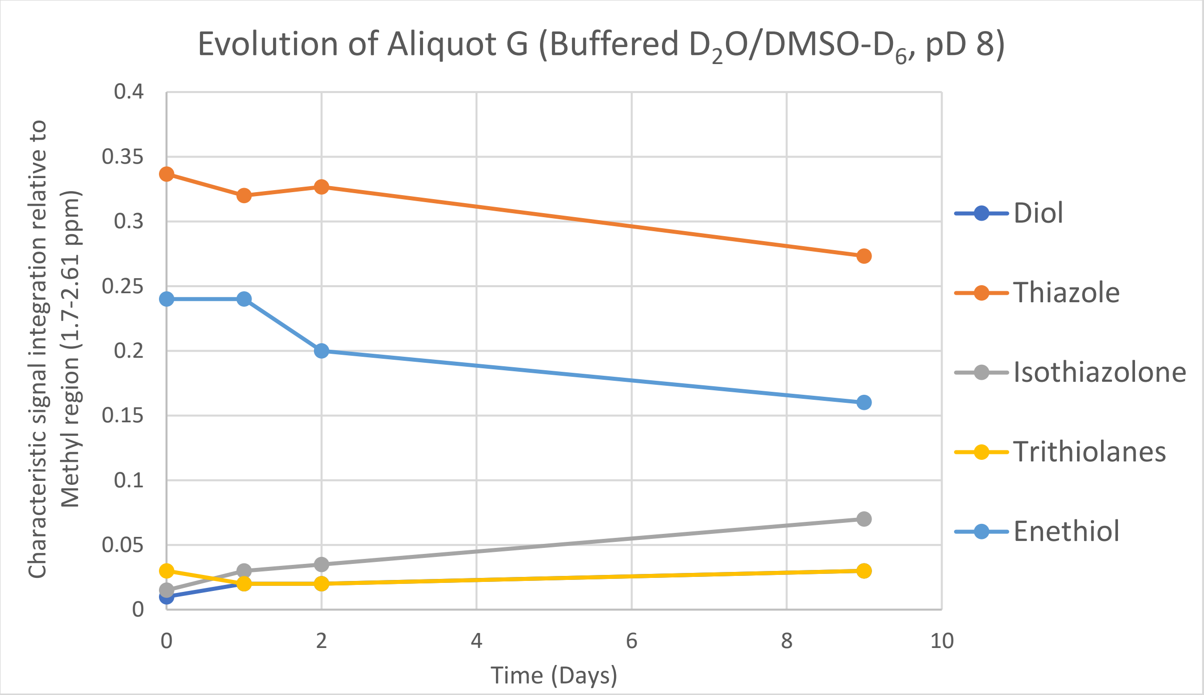

Time (Days)	Ketone (8.41 ppm)	Ketone (8.3 ppm)	Enethiol (7.8 ppm)	Diol (5.06 ppm)	1,2,4-Trithiolanes (5.21–5.04 ppm)	Isothiazolone (4.87 ppm)	Thiazole (2.61 ppm)
T0	1.14	0.54	0.24	0.01	0.04	0.015	0.34
T1	1.13	0.58	0.24	0.02	0.03	0.03	0.32
T2	1.06	0.52	0.2	0.02	0.02	0.035	0.33
T9	1.07	0.53	0.16	0.03	0.04	0.07	0.27

Figure H55: The evolution of the reaction mixture derived from the photolysis of phenacylsulfide ***3f*** *in D_2O/DMSO-D_6 (2:3) followed by addition of D_2O (0.1 M phosphate)/DMSO-D_6 (2:3) with the pD adjusted to 8.0 (Aliquot G) as analysed over 9 days The integration of a methine CH signal for the diol, and trithiolanes are calculated relative to the methyl region from 1.7–2.6 ppm. The integration of the methylene CH_2 of the isothiazolone and the methyl CH_3 of the thiazole are also calculated relative to the same region. Table of corresponding data shown below graph. Note: integral values for both the methylene CH_2 of the isothiazolone and the methyl CH_3 of the thiazole are divided by 2 and 3 respectively, to provide the value of a single proton for each species.*

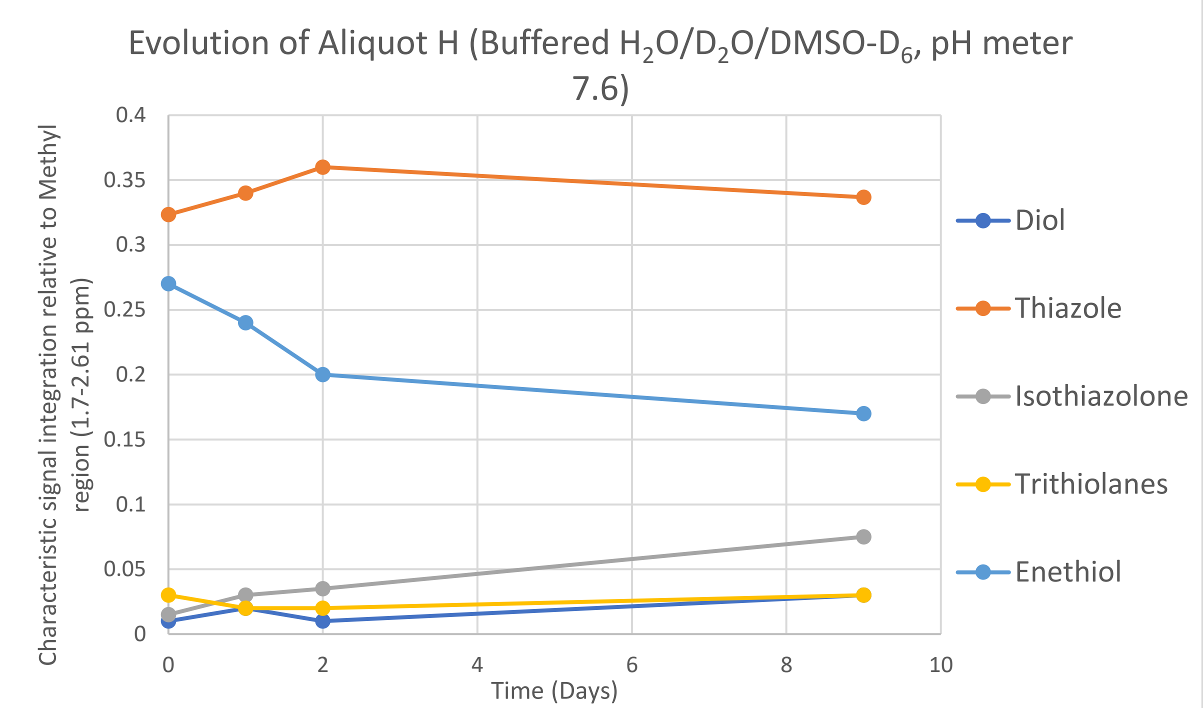

Time (Days)	Ketone (8.41 ppm)	Ketone (8.3 ppm)	Enethiol (7.8 ppm)	Diol (5.06 ppm)	1,2,4-Trithiolanes (5.21–5.04 ppm)	Isothiazolone (4.87 ppm)	Thiazole (2.61 ppm)
T0	0.94	0.51	0.27	0.01	0.03	0.015	0.32
T1	0.92	0.49	0.24	0.02	0.02	0.03	0.34
T2	0.83	0.44	0.2	0.01	0.02	0.035	0.36
T9	0.8	0.44	0.17	0.03	0.03	0.075	0.34

*Figure H56: The evolution of the reaction mixture derived from the photolysis of phenacylsulfide **3f** in D_2O/DMSO-D_6 (2:3) followed by addition of H_2O (0.1 M phosphate)/DMSO-D_6 (2:3) with the pH meter reading adjusted to 7.6 (Aliquot H) as analysed over 9 days The integration of a methine CH signal for the diol and trithiolanes are calculated relative to the methyl region from 1.7–2.6 ppm. The integration of the methylene CH_2 of the isothiazolone and the methyl CH_3 of the thiazole are also calculated relative to the same region. Table of corresponding data shown below graph. Note: integral values for both the methylene CH_2 of the isothiazolone and the methyl CH_3 of the thiazole are divided by 2 and 3 respectively, to provide the value of a single proton for each species.*

Evolution of photolysis products of *3f* in D_2O:DMSO-D_6 (2:3) at pD 2.6, 6.6 and 8.4

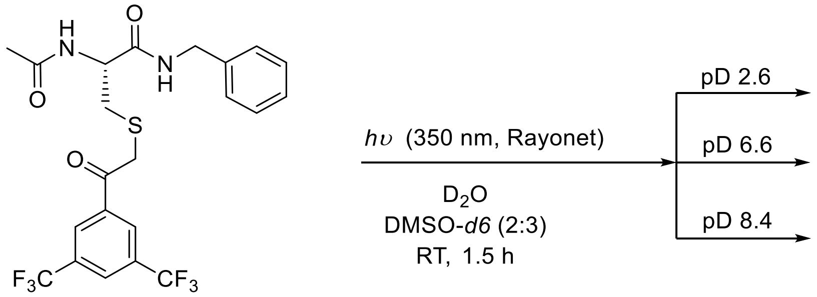

Compound **3f** (60 mg, 118.4 µmol) was dissolved in D_2O/DMSO-D_6 (2:3, 9.6 mL). The pD of this solution was then adjusted to pD 8 through addition of 0.5 M NaOH in D_2O. The mixture was then sparged with N_2 for 15 minutes, transferred to a NMR tube and then back-flushed with N_2 prior to sealing. The resulting solution was then illuminated at 350 nm in Rayonet photoreactor for 1.5 hrs. After photolysis, the pD of the solution was measured and was found to have dropped to pD 4.3. The reaction mixture was then divided into 3 equal aliquots (1 mL) and treated as follows:

Aliquot (A): adjusted to pD 2.6
Aliquot (B): adjusted to pD 6.6
Aliquot (C): adjusted to pD 8.4

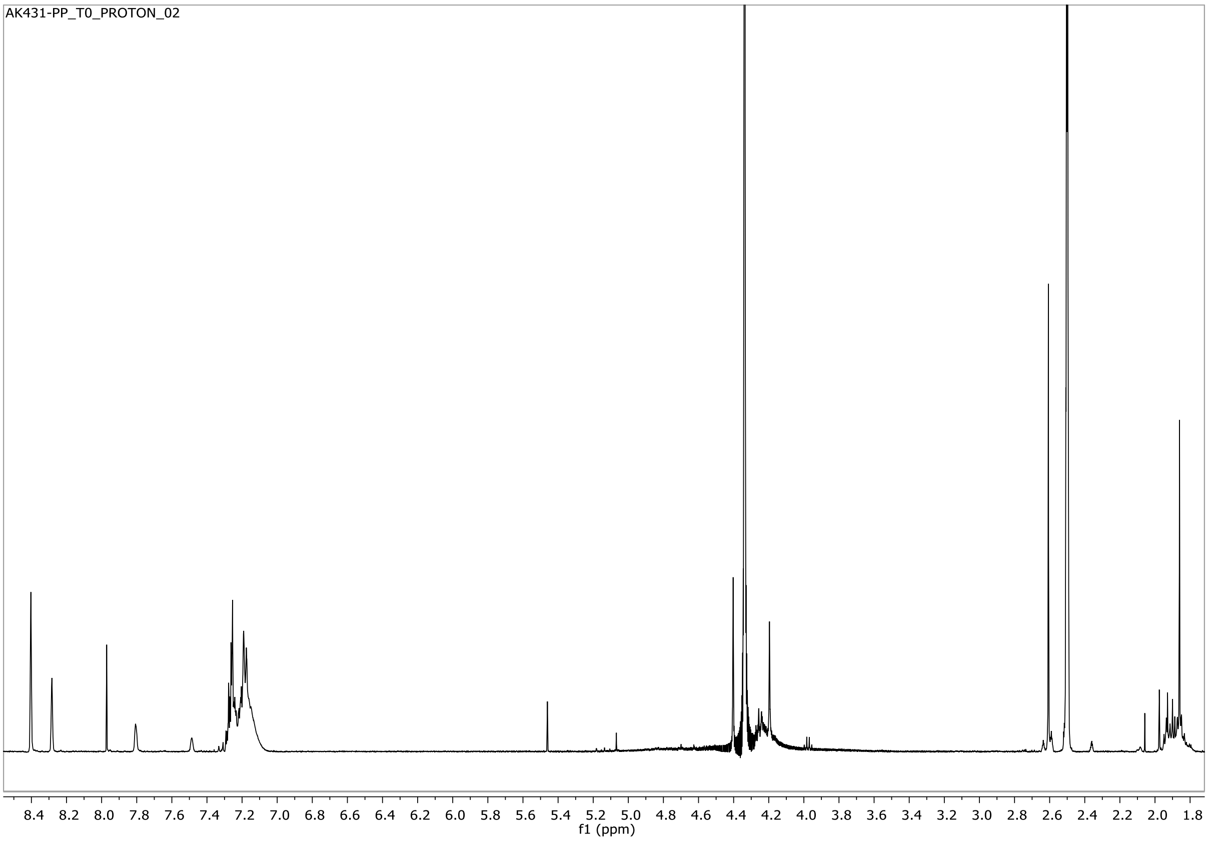

*Figure H57: ^{1}H NMR spectrum of the mixture derived from the photolysis of phenacylsulfide **3f** in D_2O/DMSO-D_6 (2:3) at pD 4.3*

Analysis of spectra at pD 2.6 (aq. DMSO-D$_6$) – Aliquot A

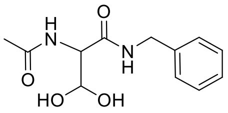

Exact Mass: 252.1110

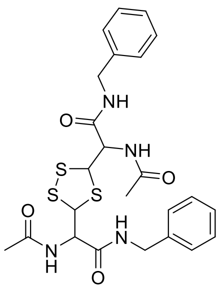

Exact Mass: 532.1273

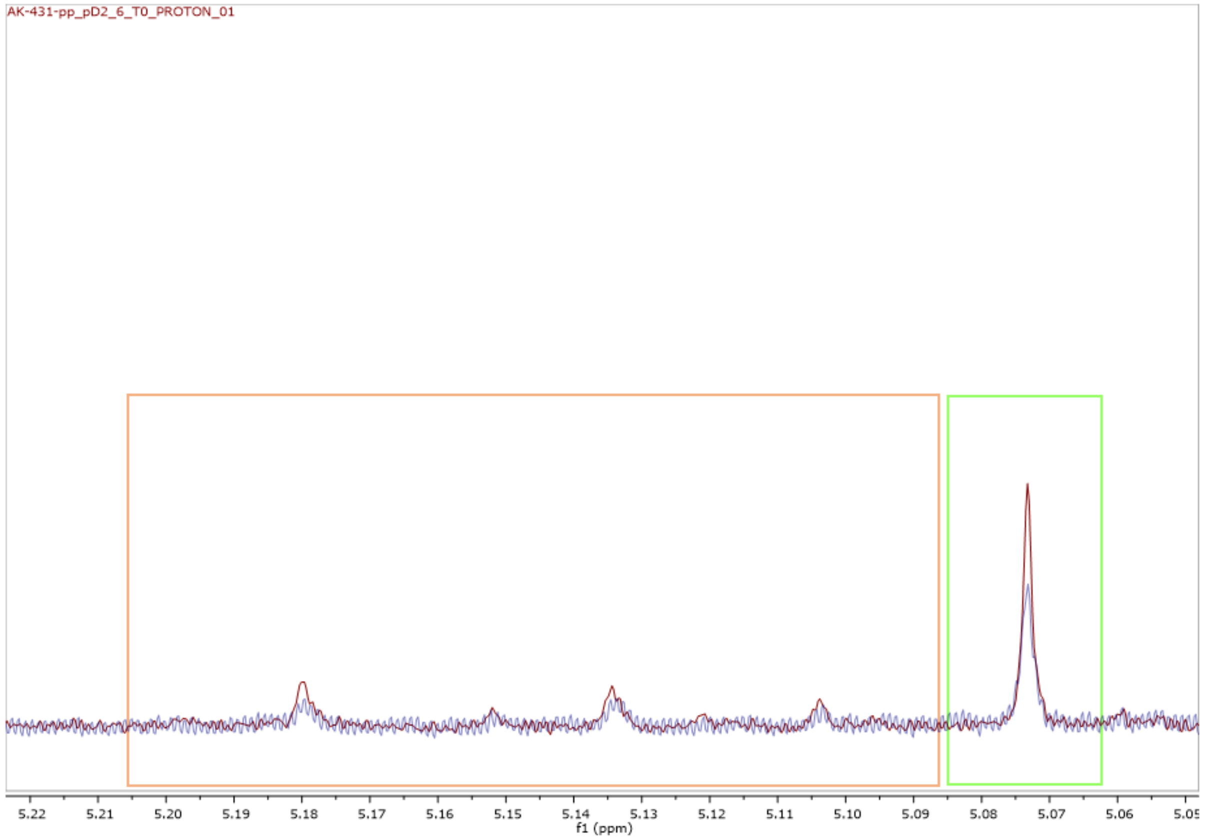

Figure H58: Overlapped and zoomed-in image of β-methine region of ^{1}H NMR spectra of the mixture derived from the photolysis of phenacylsulfide ***3f*** *in D_2O/DMSO-D$_6$ (2:3) with the pD adjusted to 2.6 (Aliquot A) as analysed from day 0 (red) to day 5 (blue). Highlighted are the β-methine hydrogen nuclei of the gem-diol (green box) and 1,2,4-trithiolane (orange box) species.*

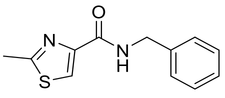

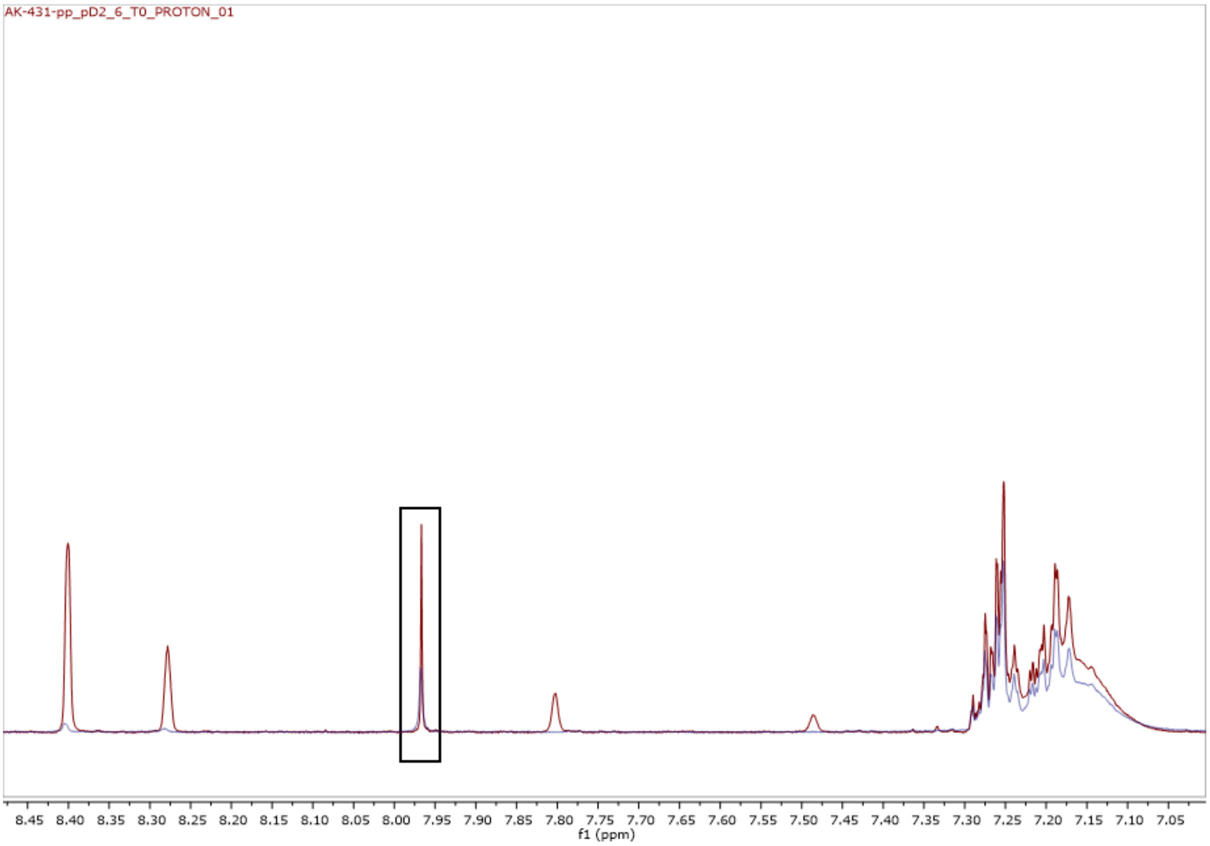

Figure H59: Overlapped and zoomed-in image of ^{1}H NMR spectra of the mixture derived from the photolysis of phenacylsulfide ***3f*** *in D_2O/DMSO-D_6 (2:3) with the pD adjusted to 2.6 (Aliquot A) as analysed from day 0 (red) to day 5 (blue). Highlighted are the signals associated with the methine moiety of the thiazole product at 7.99 ppm (black box).Apart from the initial increase in the yield of thiazole after adjusting pD to 2.6, thiazole does not increase in amount on keeping the reaction mixture over a duration of 5 days. Note, the ketone is volatile and appears to evaporate within the flask which is pressurised under a balloon of N_2 gas, which was replaced at intervals.*

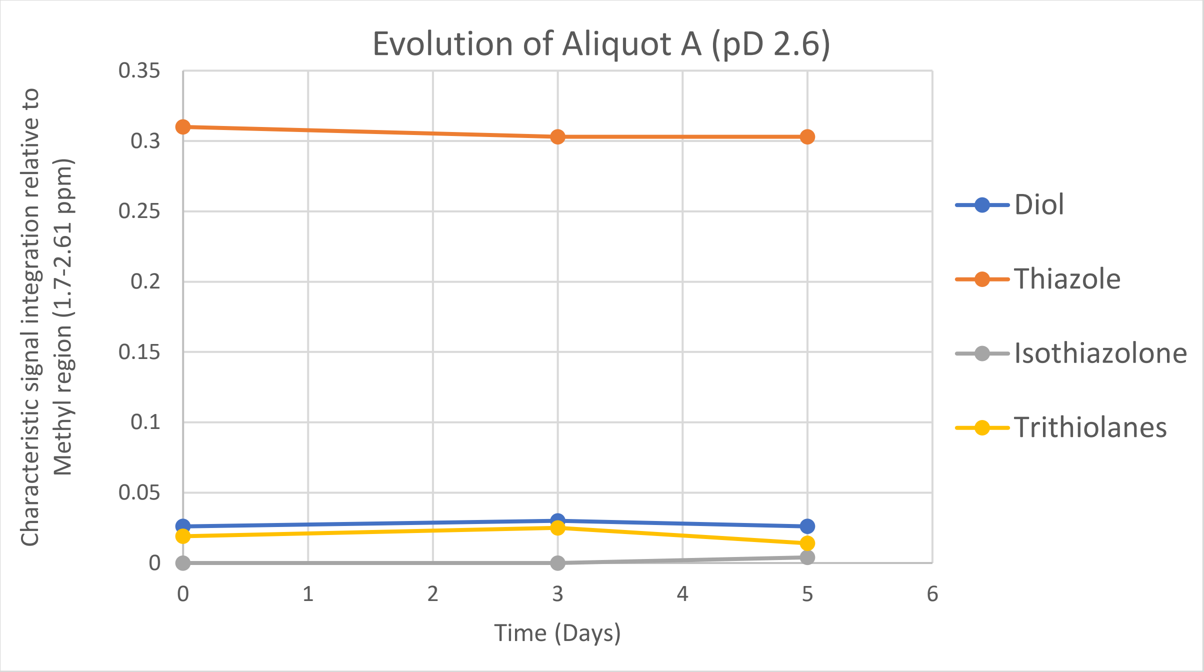

Time (Days)	Diol (5.06 ppm	1,2,4-Trithiolanes (5.21–5.04 ppm)	Isothiazolone (4.87 ppm)	Thiazole (2.61 ppm)
T0 (pD 4.3)	0.021	0.022	0	0.24
T0 (pD 2.6)	0.026	0.019	0	0.31
T3 (pD 2.6)	0.030	0.025	0	0.303
T5 (pD 2.6)	0.026	0.014	0.004	0.303

Figure H60: The integration of a methine CH signal for the diol, and trithiolanes are calculated relative to the methyl region from 1.7–2.6 ppm. The integration of the methyl CH_3 of the thiazole are also calculated relative to the same region. Table of corresponding data shown below graph. Note: integral values for the methyl CH_3 of the thiazole is divided by 3 to provide the value of a single proton for each species.

Analysis of spectra at pD 6.6 (aq. DMSO-D_6) – Aliquot B

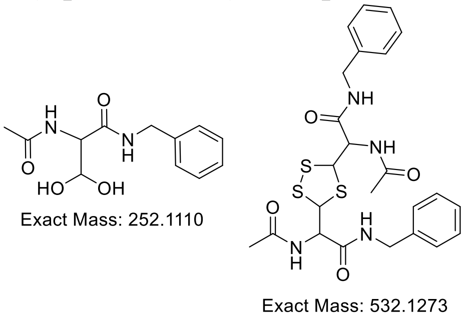

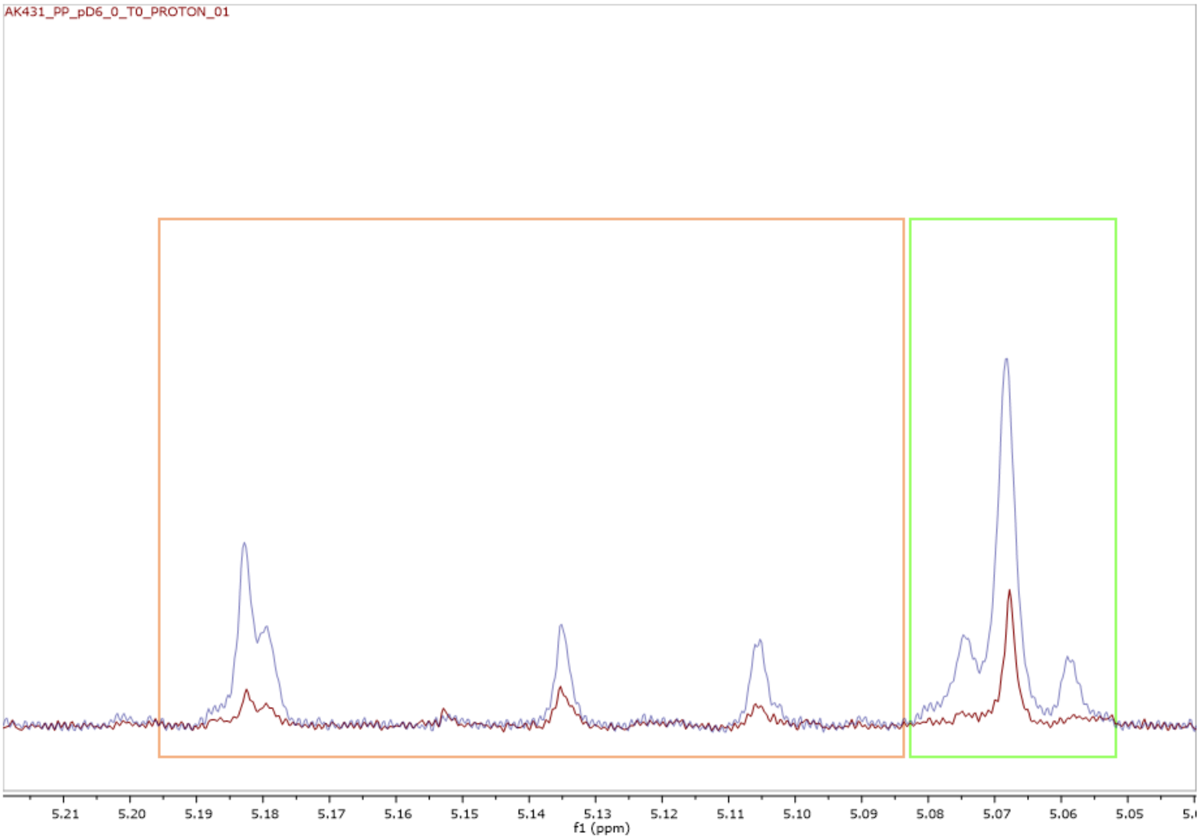

Figure H61: Overlapped and zoomed-in image of β-methine region of ^{1}H NMR spectra of the mixture derived from the photolysis of phenacylsulfide ***3f*** *in D_2O/DMSO-D_6 (2:3) with the pD adjusted to 6.6 (Aliquot B) as analysed from day 0 (red) to day 5 (blue). Highlighted are the β-methine hydrogen nuclei of the gem-diol (green box) and 1,2,4-trithiolane (orange box) species.*

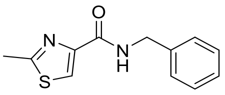

Exact Mass: 232.0670

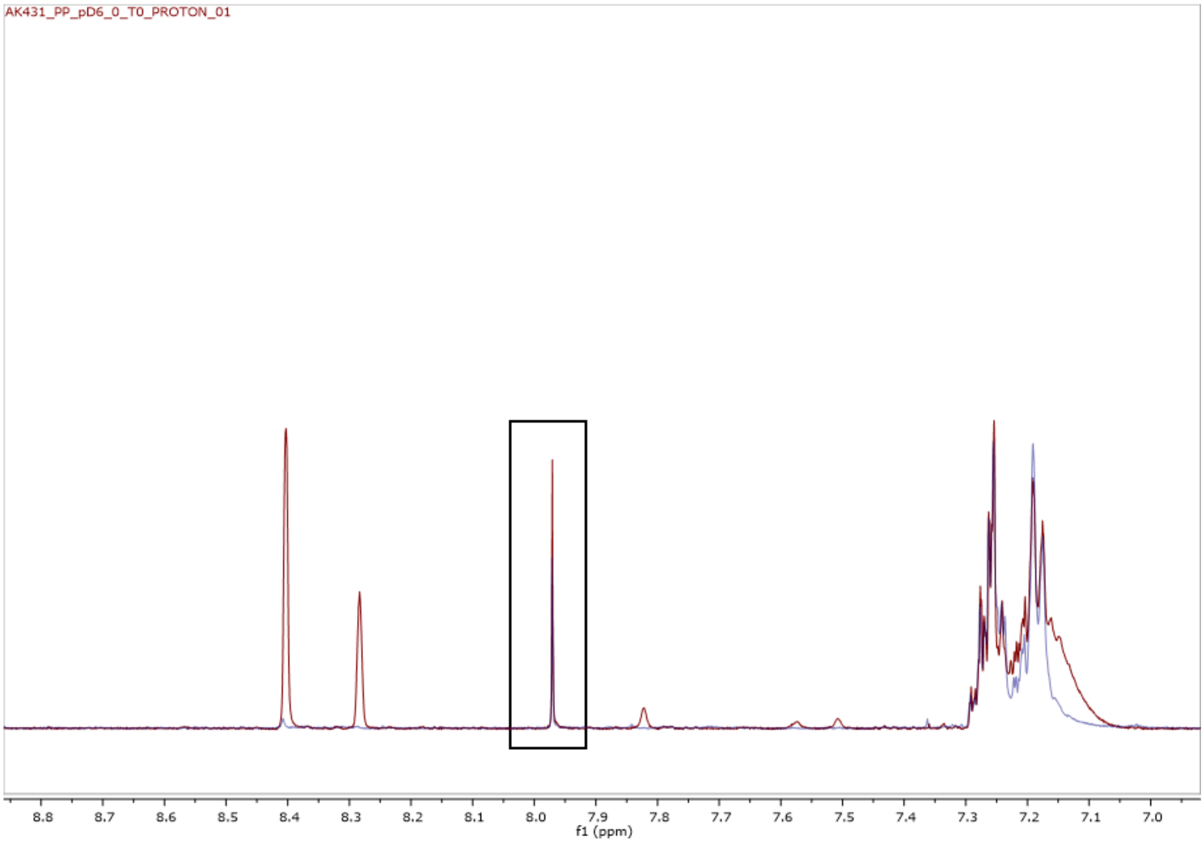

Figure H62: Overlapped and zoomed-in image of ^{1}H NMR spectra of the mixture derived from the photolysis of phenacylsulfide ***3f*** *in D_2O/DMSO-D_6 (2:3) with the pD adjusted to 6.6 (Aliquot B) as analysed from day 0 (red) to day 5 (blue). Highlighted are the signals associated with the methine moiety of the thiazole product at 7.99 ppm (black box).*

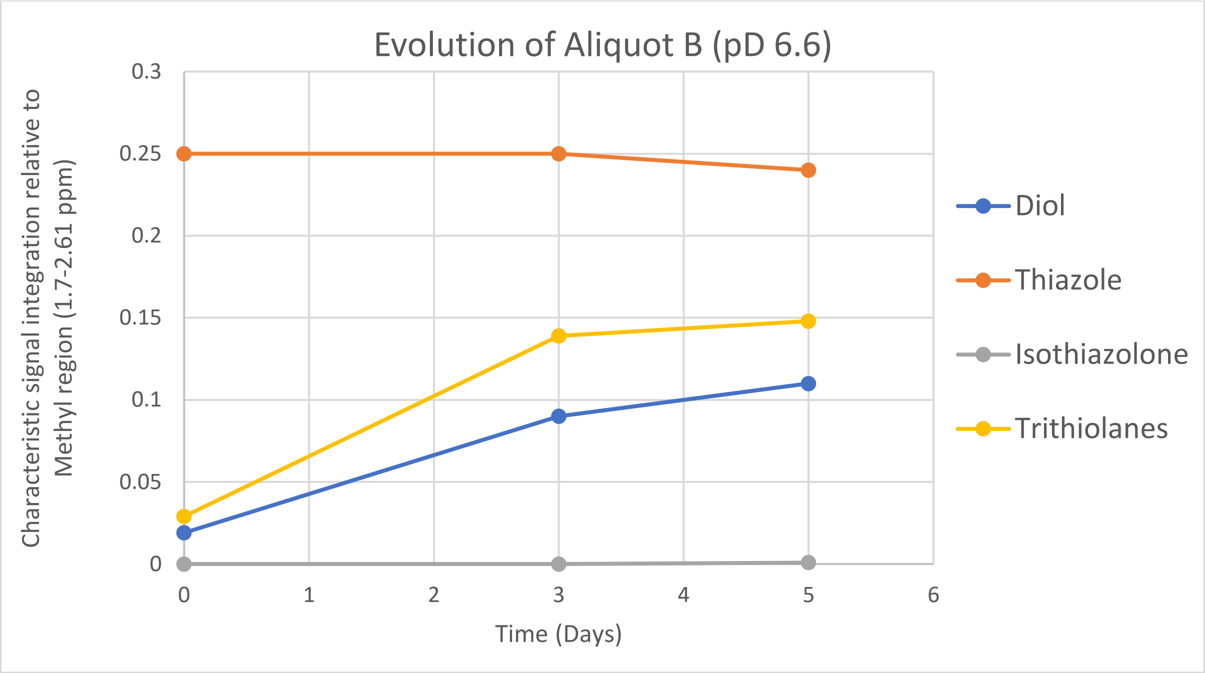

Time (Days)	Diol (5.06 ppm)	1,2,4-Trithiolanes (5.21–5.04 ppm)	Isothiazolone (4.87 ppm)	Thiazole (2.61 ppm)
T0 (pD 4.3)	0.021	0.022	0	0.24
T0 (pD 6.6)	0.019	0.029	0	0.25
T3 (pD 6.6)	0.09	0.139	0	0.25
T5 (pD 6.6)	0.11	0.148	0.008	0.24

Figure H63: The integration of a methine CH signal for the diol, and trithiolanes are calculated relative to the methyl region from 1.7–2.6 ppm. The integration of the methyl CH_3 of the thiazole are also calculated relative to the same region. Table of corresponding data shown below graph. Note: integral values for the methyl CH_3 of the thiazole is divided by 3 to provide the value of a single proton for each species.

Analysis of spectra at pD 8.6 (aq. DMSO-D_6) – Aliquot C

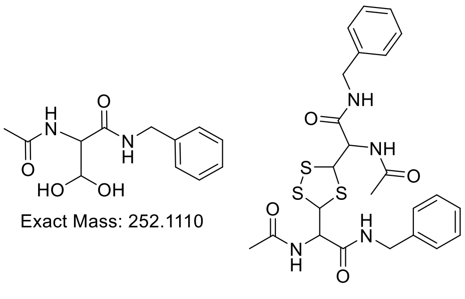

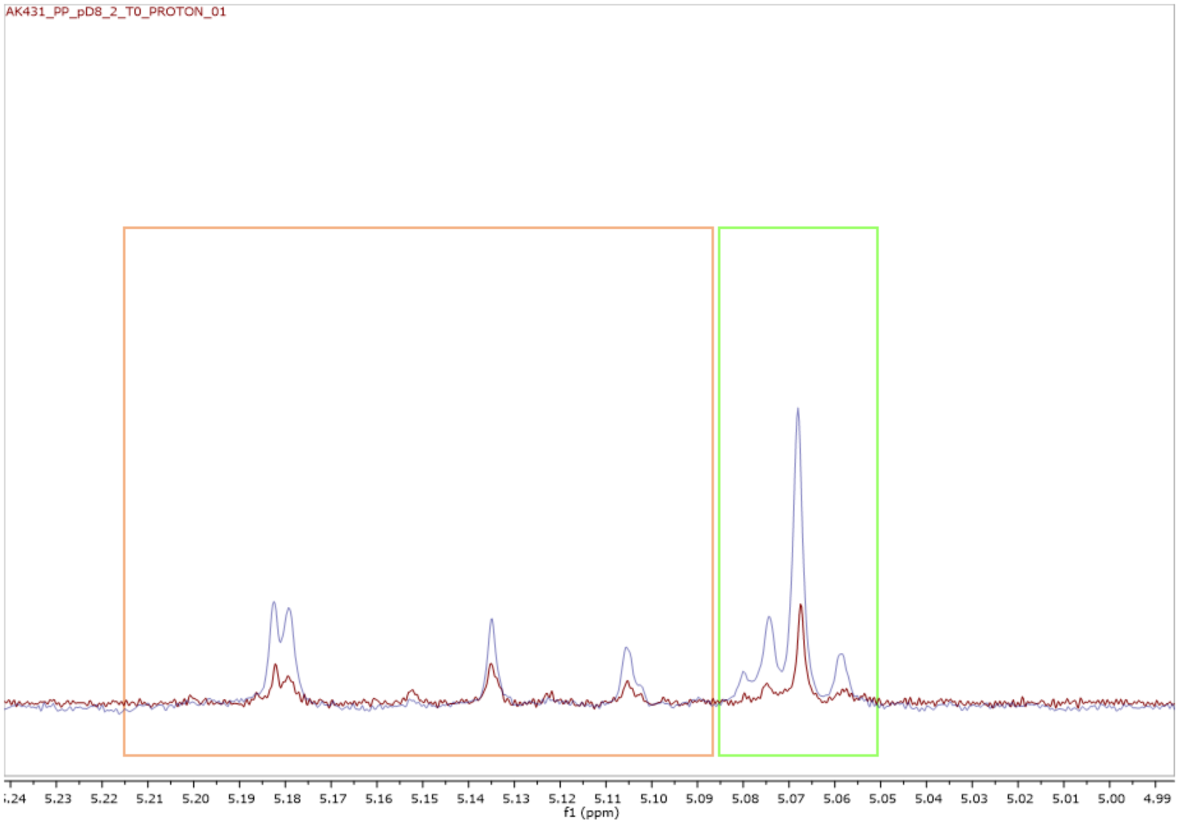

Figure H64: Overlapped and zoomed-in image of β-methine region of ^{1}H NMR spectra of the mixture derived from the photolysis of phenacylsulfide ***3f*** *in D_2O/DMSO-D_6 (2:3) with the pD adjusted to 8.4 (Aliquot C) as analysed from day 0 (red) to day 5 (blue). Highlighted are the β-methine hydrogen nuclei of the gem-diol (green box) and 1,2,4-trithiolane (orange box) species.*

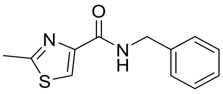

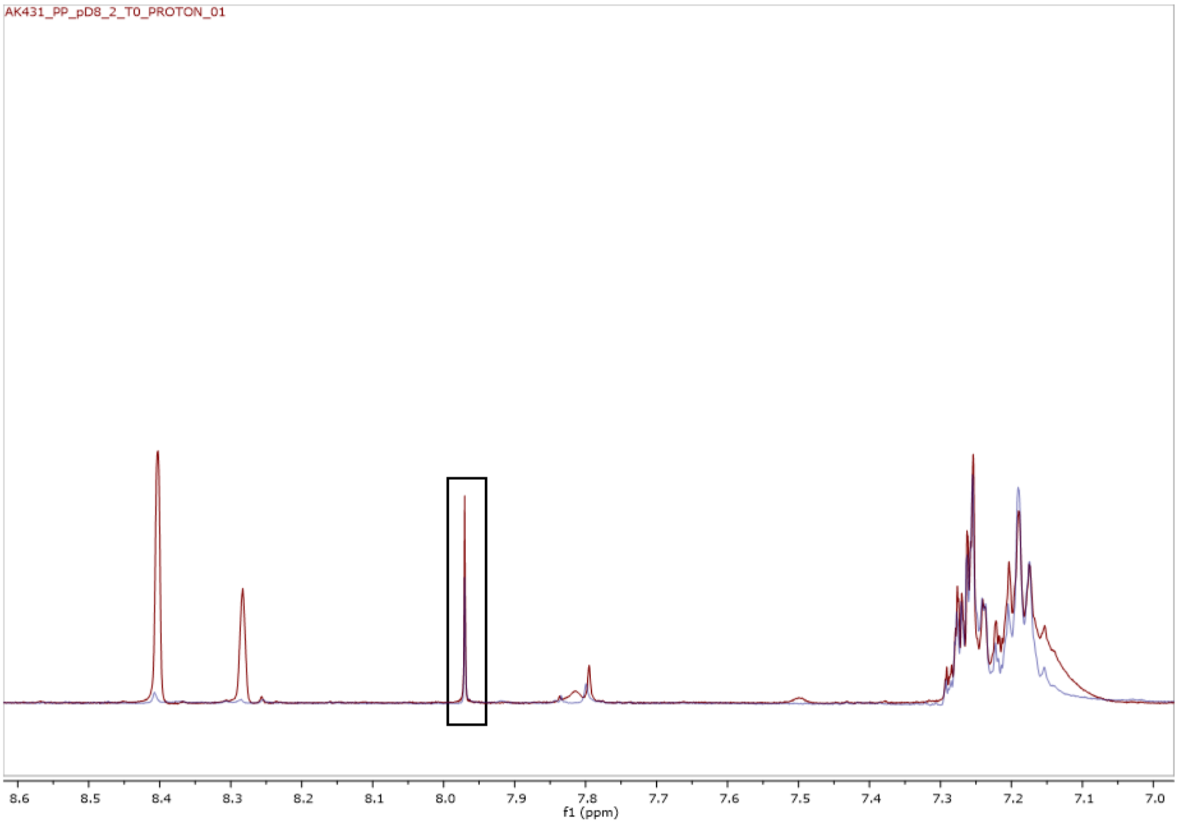

*Figure H65: Overlapped and zoomed-in image of ^{1}H NMR spectra of the mixture derived from the photolysis of phenacylsulfide **3f** in D_2O/DMSO-D_6 (2:3) with the pD adjusted to 8.4 (Aliquot C) as analysed from day 0 (red) to day 5 (blue). Highlighted are the signals associated with the methine moiety of the thiazole product at 7.99 ppm (black box).*

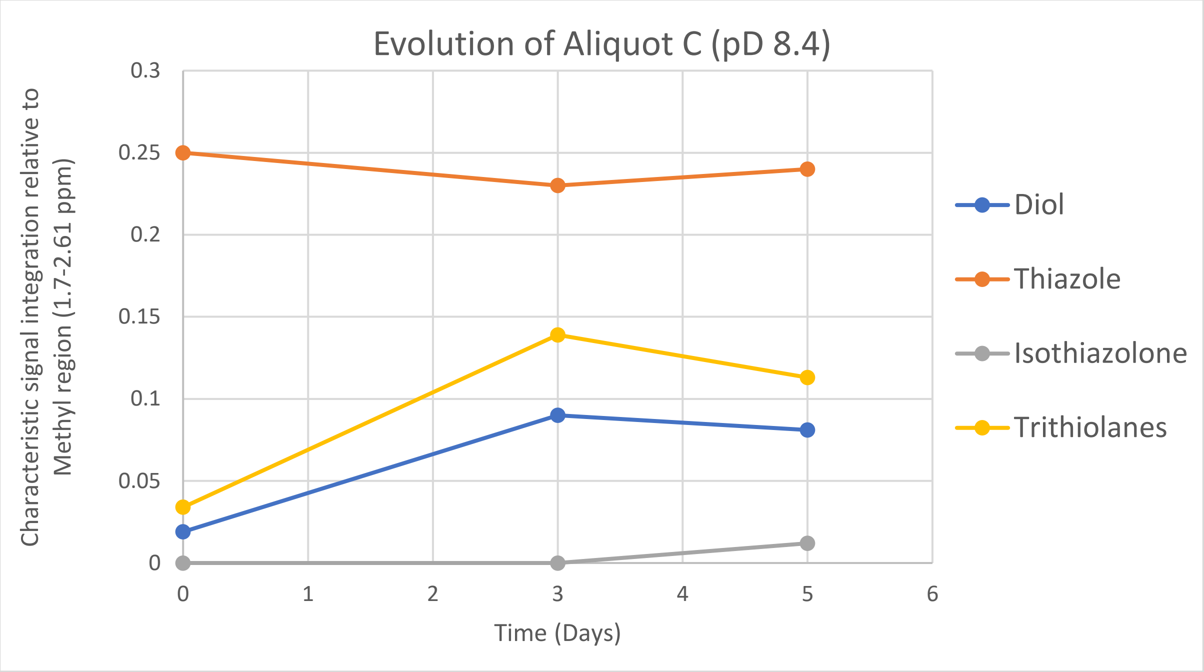

Time (Days)	Diol (5.06 ppm)	1,2,4-Trithiolanes (5.21–5.04 ppm)	Isothiazolone (4.87 ppm)	Thiazole (2.61 ppm)
T0 (pD 4.3)	0.021	0.022	0	0.24
T0 (pD 8.4)	0.019	0.034	0	0.25
T3 (pD 8.4)	0.09	0.139	0	0.23
T5 (pD 8.4)	0.081	0.113	0.012	0.24

Figure H66: The integration of a methine CH signal for the diol, and trithiolanes are calculated relative to the methyl region from 1.7–2.6 ppm. The integration of the methyl CH_3 of the thiazole are also calculated relative to the same region. Table of corresponding data shown below graph. Note: integral values for the methyl CH_3 of the thiazole is divided by 3 to provide the value of a single proton for each species.

Investigation of isothiazolone (6) as a masked cysteine thioaldehyde: Reduction in the absence of buffer in the presence of excess TCEP.

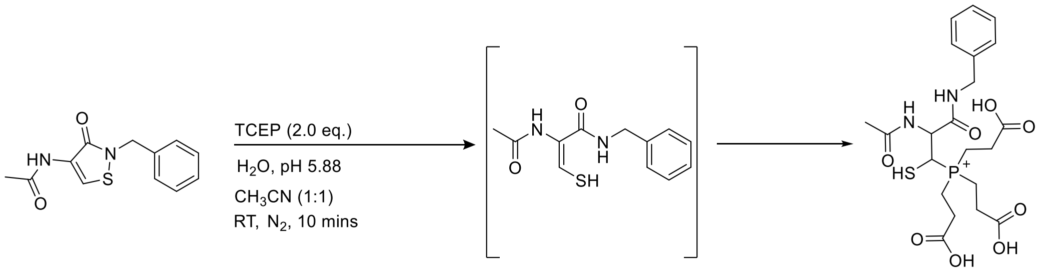

N-(2-benzyl-3-oxo-2,3-dihydro-1,2-thizol-4-yl) acetamide **6** (2.5 mg, 0.01 mmol) was dissolved in H_2O/CD_3CN (1:1, 2 mL). A H_2O solution of TCEP (1M, pH 7.4, 0.02 mmol) was added. The resulting reaction mixture was allowed to evolve for 10 mins and was then analysed by ^1H NMR spectroscopy, analysis was then repeated at the 1 hour and 1 day time points to confirm product conversion. The pH of the sample was then recorded to be pH 5.88. The reaction mixture was analysed by ^1H NMR spectroscopy over 1 day.

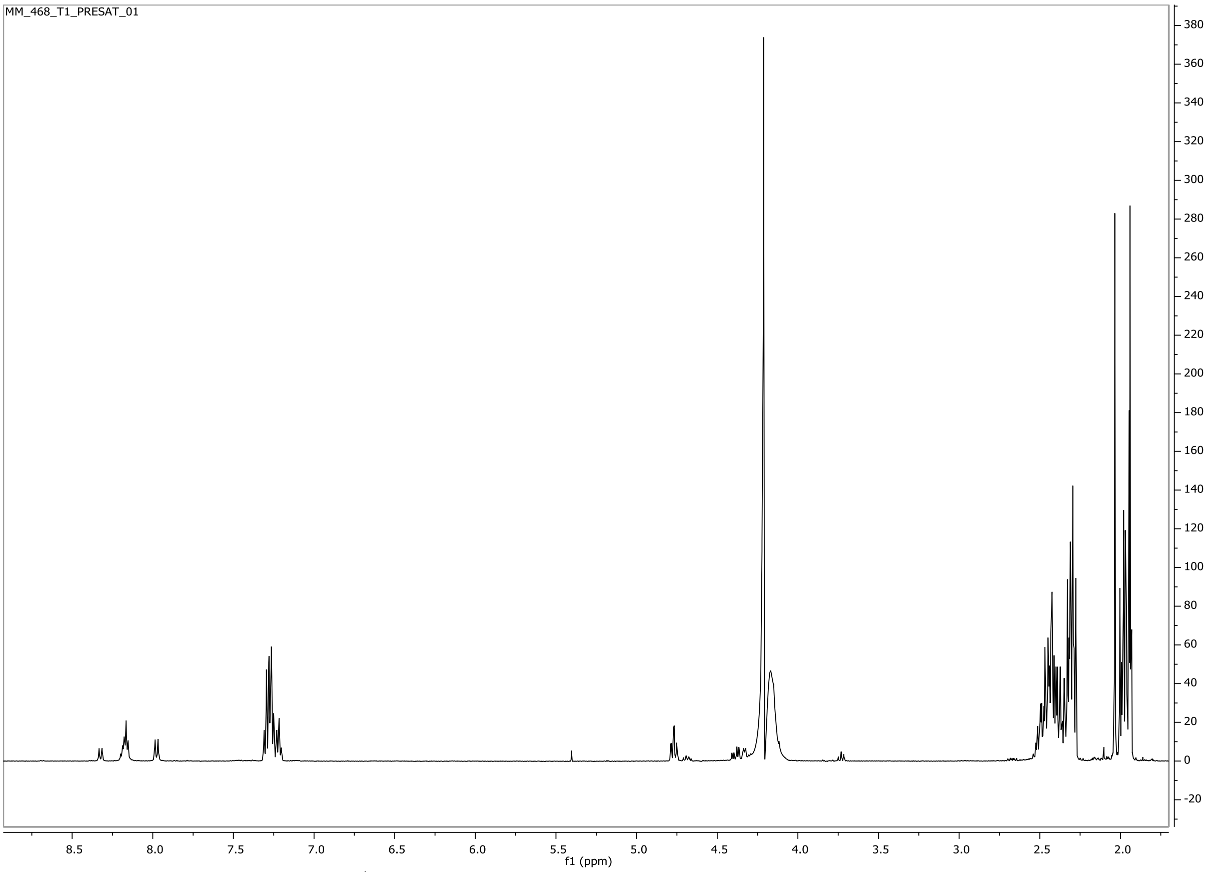

*Figure H67: Presaturation ^1H NMR spectrum (500 MHz) of the reaction mixture derived from the TCEP reduction of isothiazolone (**6**) in H_2O/CD_3CN (pH 5.88) in the presence of excess TCEP after 1 day of reaction.*

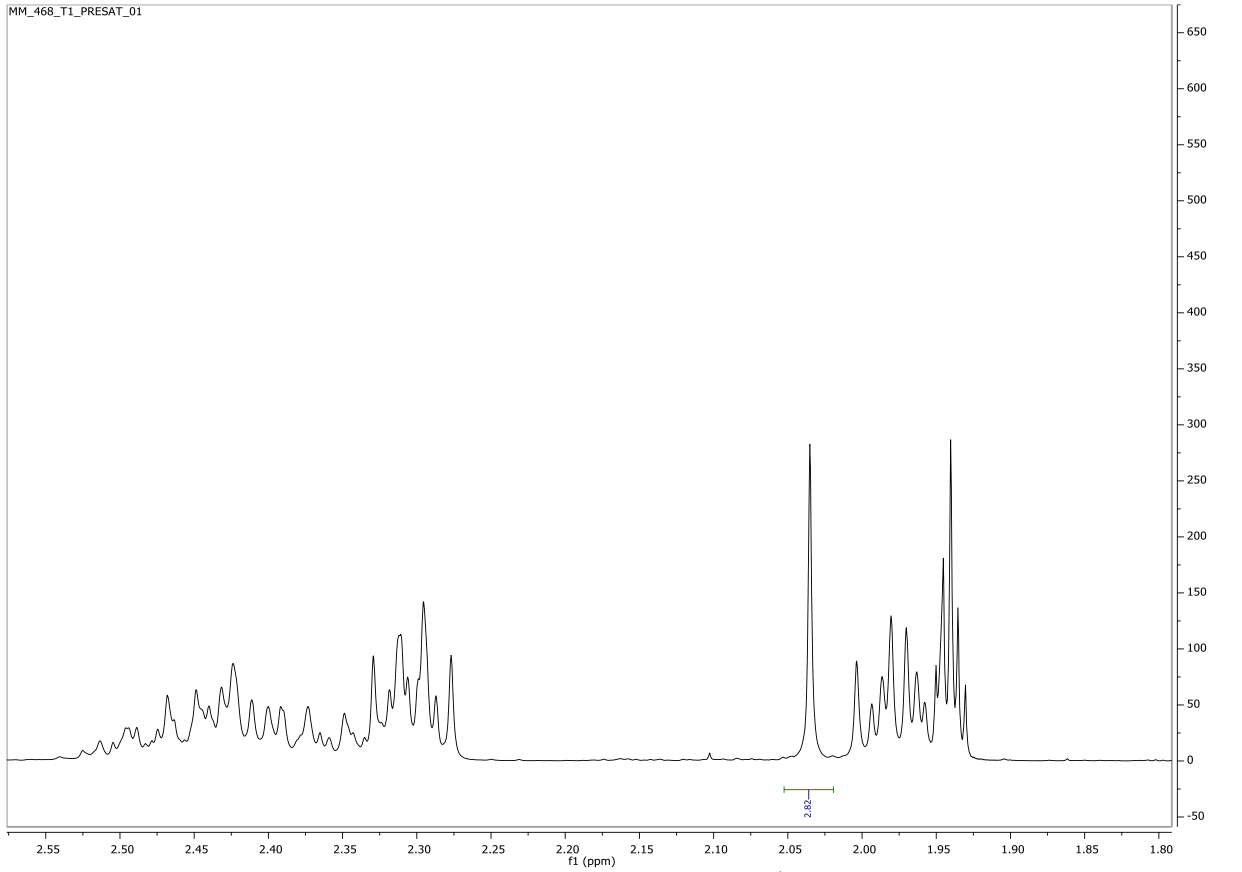

*Figure H68: Zoomed-in image of the upfield region of the ^{1}H NMR spectrum of the reaction mixture derived from the TCEP reduction of isothiazolone (**6**) in H_2O/CD_3CN (pH 5.88) in the presence of excess TCEP after 1 day. Highlighted are signals corresponding to the acetyl group of the phosphonium adduct. Based on the integration of the aromatic region the adduct has an approximate conversion of 94%*

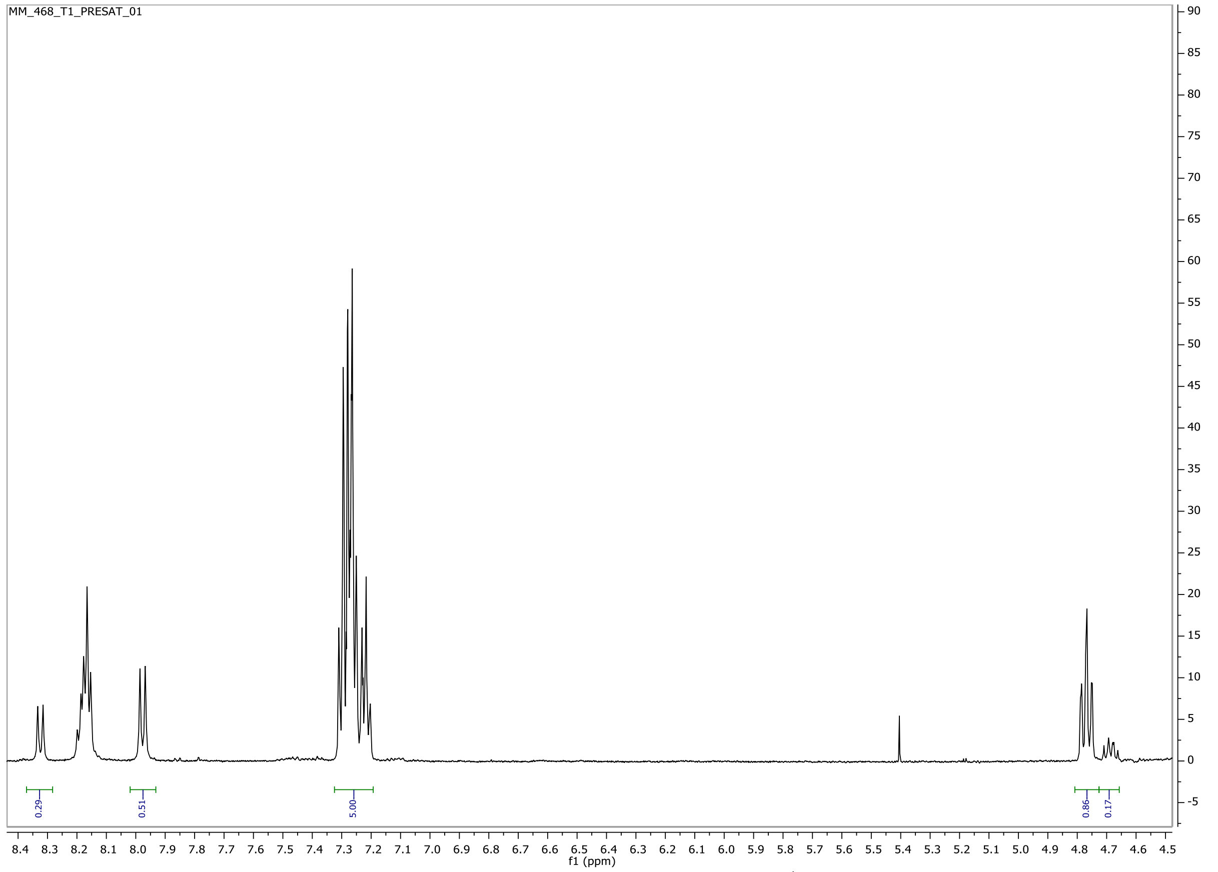

*Figure H69: Zoomed-in image of the downfield region of the ^{1}H NMR spectrum of the reaction mixture derived from the TCEP reduction of isothiazolone (**6**) in H_2O/CD_3CN (pH 5.88) in the presence of excess TCEP after 1 day of reaction. Highlighted are signals corresponding to the α-methine of the cysteine moiety showing both isomers (4.77, 4.69 ppm) of the phosphonium adduct. In addition, the signals corresponding to the NH adjacent to the α-centre of both isomers are highlighted at 7.98 ppm for the major isomer and 8.32 ppm for the minor isomer. Note: the isomers are present in an approximate ratio of 3:1 based on the integration of the phorphorus signals in the ^{31}P NMR spectrum (see Figure H74).*

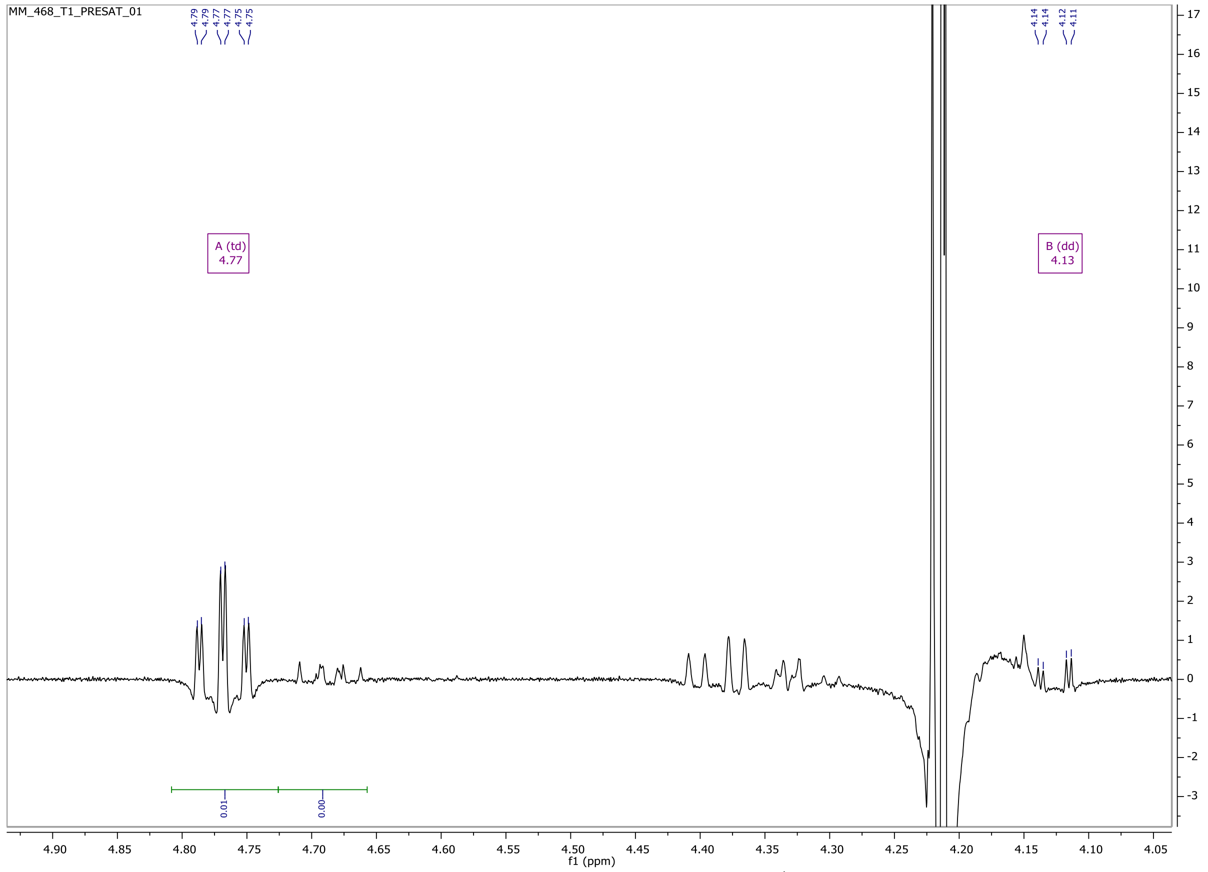

*Figure H70: Zoomed-in image of the methine region of the ^{1}H NMR spectrum of the reaction mixture derived from the TCEP reduction of isothiazolone (**6**) in H_2O/CD_3CN (pH 5.88) in the presence of excess TCEP after 1 day. Apodization of the spectrum was altered to a sine bell of 0° allowing for the visualization of the signal corresponding to the β methine of the major isomer of the phosphonium species at 4.13 ppm. This signal is observed as a doublet of doublet showing $^{3}J_{H\alpha\text{-}H\beta}$ =1.6 Hz and $^{2}J_{H\beta\text{-}P}$ =10.8 Hz. The signal corresponding to the α methine of the species appears as an apparent triplet of doublets at 4.77 ppm showing a $^{3}J_{H\alpha\text{-}H\beta}$ =1.6 Hz, $^{3}J_{NH\text{-}H\alpha}$ =9.0 Hz and $^{3}J_{H\alpha\text{-}P}$ ~9.0 Hz*

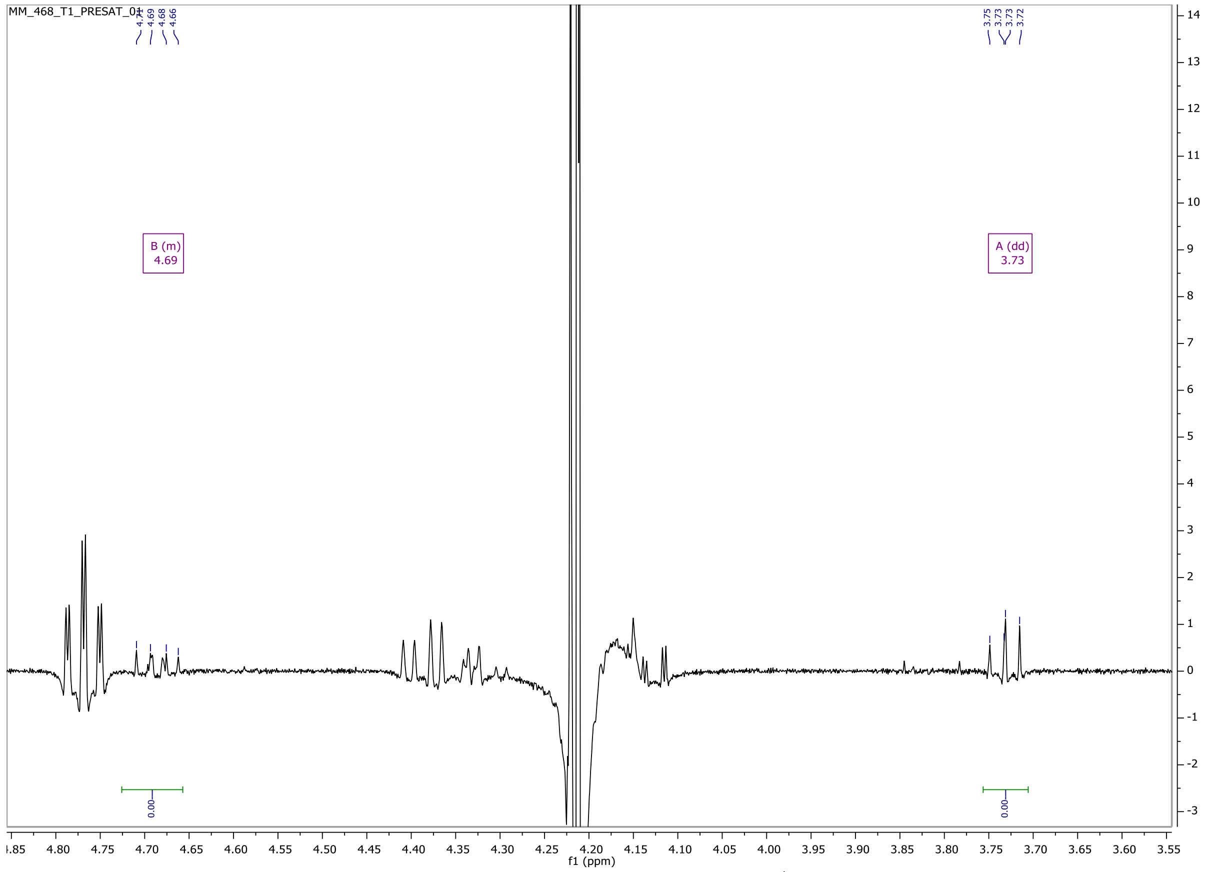

*Figure H71: Zoomed-in image of the methine region of the ^{1}H NMR spectrum of the reaction mixture derived from the TCEP reduction of isothiazolone (**6**) in H_2O/CD_3CN (pH 5.88) in the presence of excess TCEP after 1 day of reaction. Apodization of the spectrum was altered to a sine bell of 0° allowing for the observation of the doublet of doublet corresponding to the β methine of the minor isomer of the phosphonium species at 3.73 ppm. This signal is observed as a doublet of doublet showing $^{3}J_{H\alpha\text{-}H\beta}$ =8.0 Hz and $^{2}J_{H\beta\text{-}P}$ =8.8 Hz. The signal corresponding to the α methine of the species appears as a doublet of doublet of doublets at 4.69 ppm showing $^{3}J_{H\alpha\text{-}H\beta}$ =8.0 Hz, $^{3}J_{NH\text{-}H\alpha}$ =9.0 Hz and $^{3}J_{H\alpha\text{-}P}$ ~6.4 Hz*

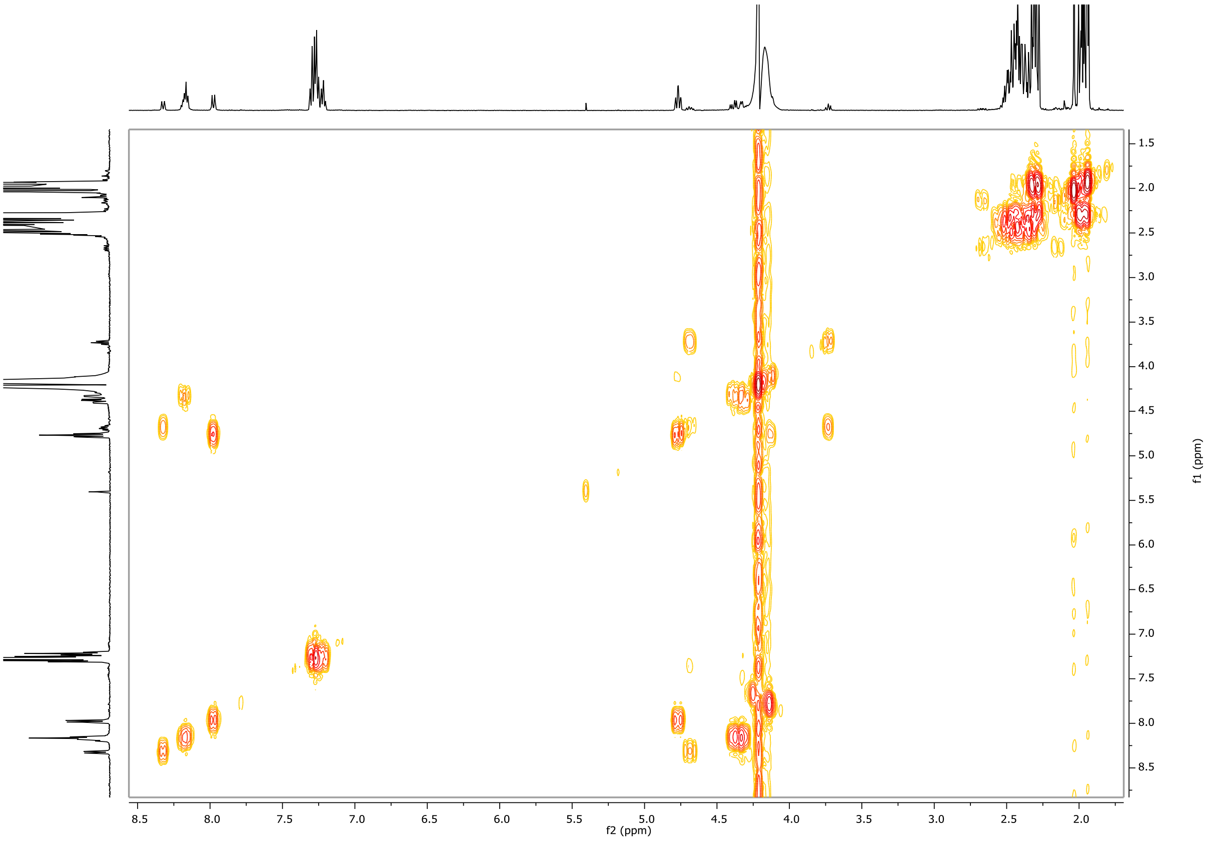

*Figure H72: COSY NMR spectrum of the reaction mixture derived from the TCEP reduction of isothiazolone (**6**) in H_2O/CD_3CN (pH 5.88) in the presence of excess TCEP after 1 day of reaction.*

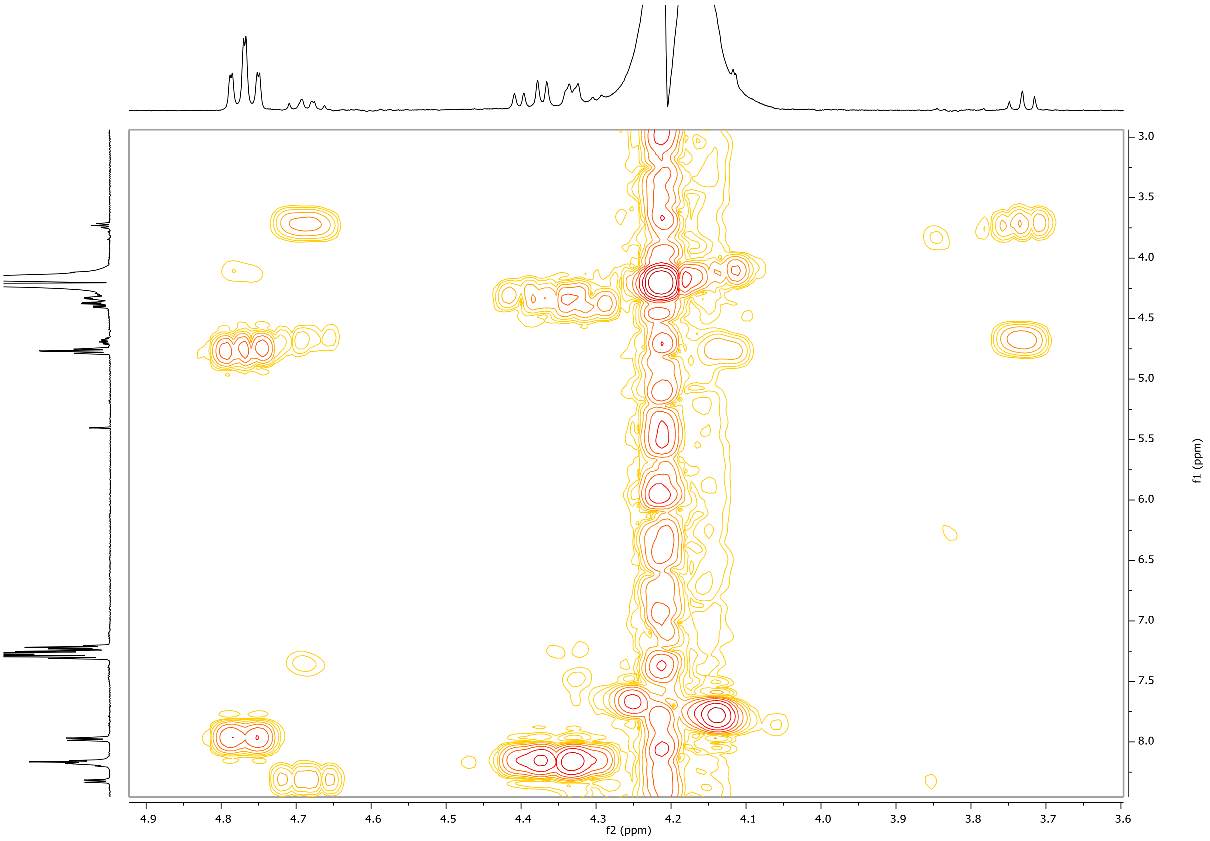

*Figure H73: Zoomed in COSY NMR spectrum of the reaction mixture derived from the TCEP reduction of isothiazolone (**6**) in H_2O/CD_3CN (pH 5.88) in the presence of excess TCEP following 1 day zoomed in on the methine region of the phosphonium species isomers. Note the correlation of the α methine of the major isomer at 4.77 ppm with the NH at 7.98 ppm and the β methine at 4.14 ppm (obscured by HDO signal). Also, note the correlation of the α methine of the minor isomer at 4.69 ppm with the NH at 8.32 ppm and the β methine at 3.73 ppm.*

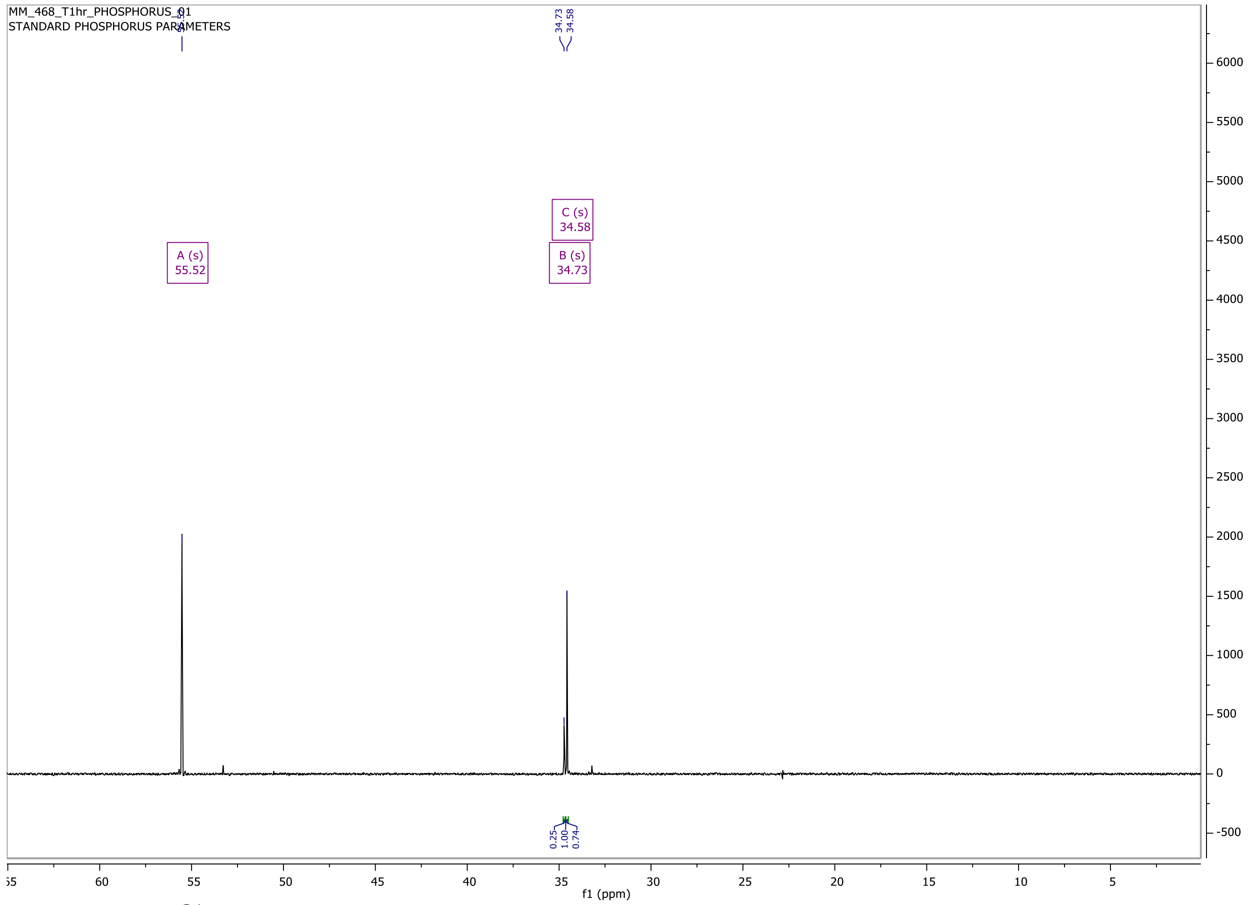

*Figure H74: ^{31}P NMR spectrum (200 MHz) of the reaction mixture derived from the TCEP reduction of isothiazolone (**6**) in H_2O/CD_3CN (pH 5.88) in the presence of excess TCEP following 4 days. Highlighted are the signals corresponding to oxidised TCEP (55.52 ppm) and both isomers of the phosphonium species; minor (34.73 ppm) and major (34.58 ppm).*

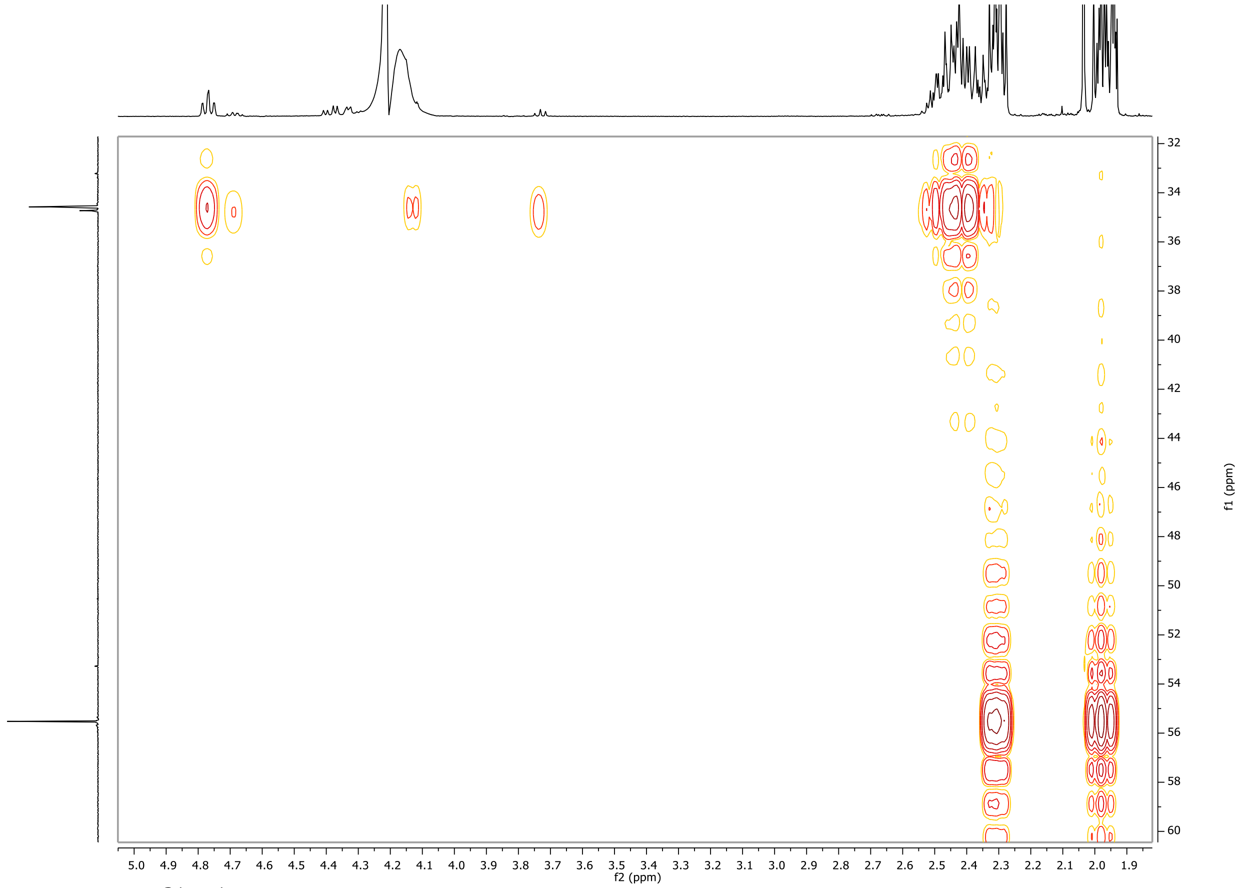

*Figure H75: ^{31}P-^{1}H HMBC NMR spectrum of the reaction mixture derived from the TCEP reduction of isothiazolone (**6**) in H_2O/CD_3CN (pH 5.88) in the presence of excess TCEP following 1 day of reaction. Highlighted are the signals corresponding to oxidised TCEP; correlation of the ^{1}H signals at 2.31 and 1.98 ppm with the ^{31}P signal at 55.52 ppm. The correlations of both species of the phosphonium adduct are also highlighted with the α methine of the major isomer (4.77 ppm) correlating to the ^{31}P signal at 34.58 ppm and the α methine of the minor isomer (4.69 ppm) correlating to the ^{31}P signal at 34.73 ppm. The HMBC also shows the correlation of the β methine of the major isomer (4.13 ppm, obscured by HDO signal) to the ^{31}P signal of the major isomer (34.58 ppm) and the β methine of the minor isomer at (3.73 ppm) with the ^{31}P signal of the minor isomer (34.73 ppm).*

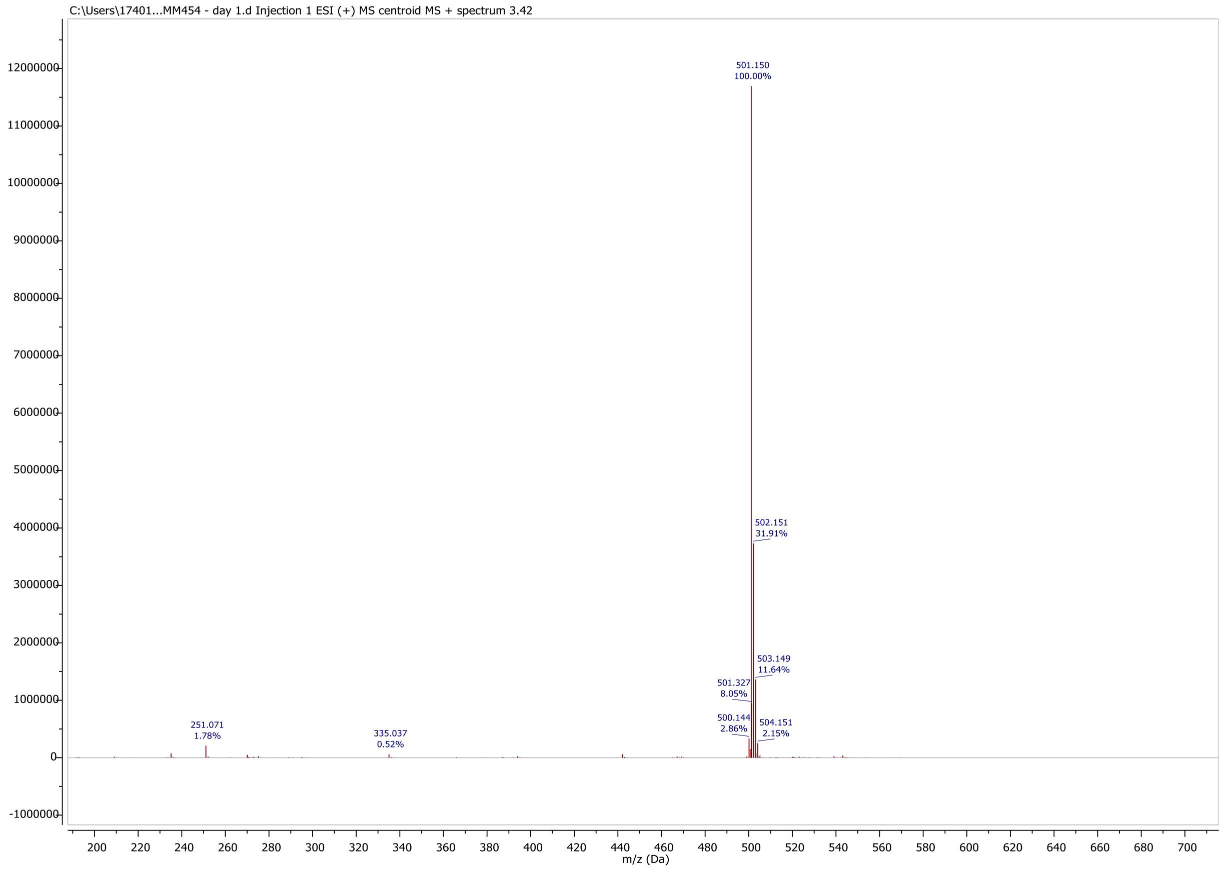

Figure H76: HRMS (ESI) spectrum of the phosphonium adduct showing the m/z $(M)^{+}$ (calcd. 501.145, observed 501.150) adduct.

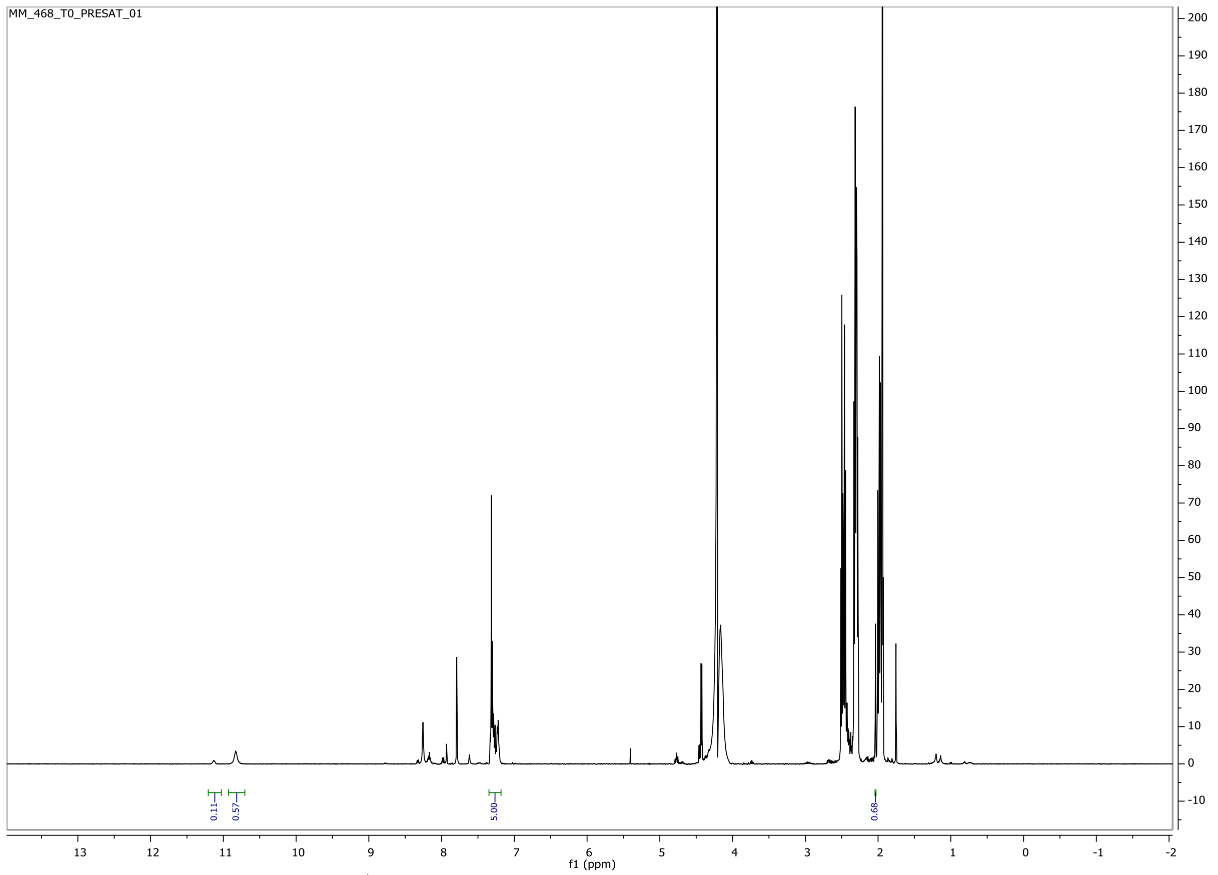

*Figure H77: Presaturation ^{1}H NMR spectrum (500 MHz) of the reaction mixture derived from the TCEP reduction of isothiazolone (**6**) in H_2O/CD_3CN (pH 5.88) in the presence of excess TCEP following 10 minutes, exhibiting an initial conversion percentage of 23%.*

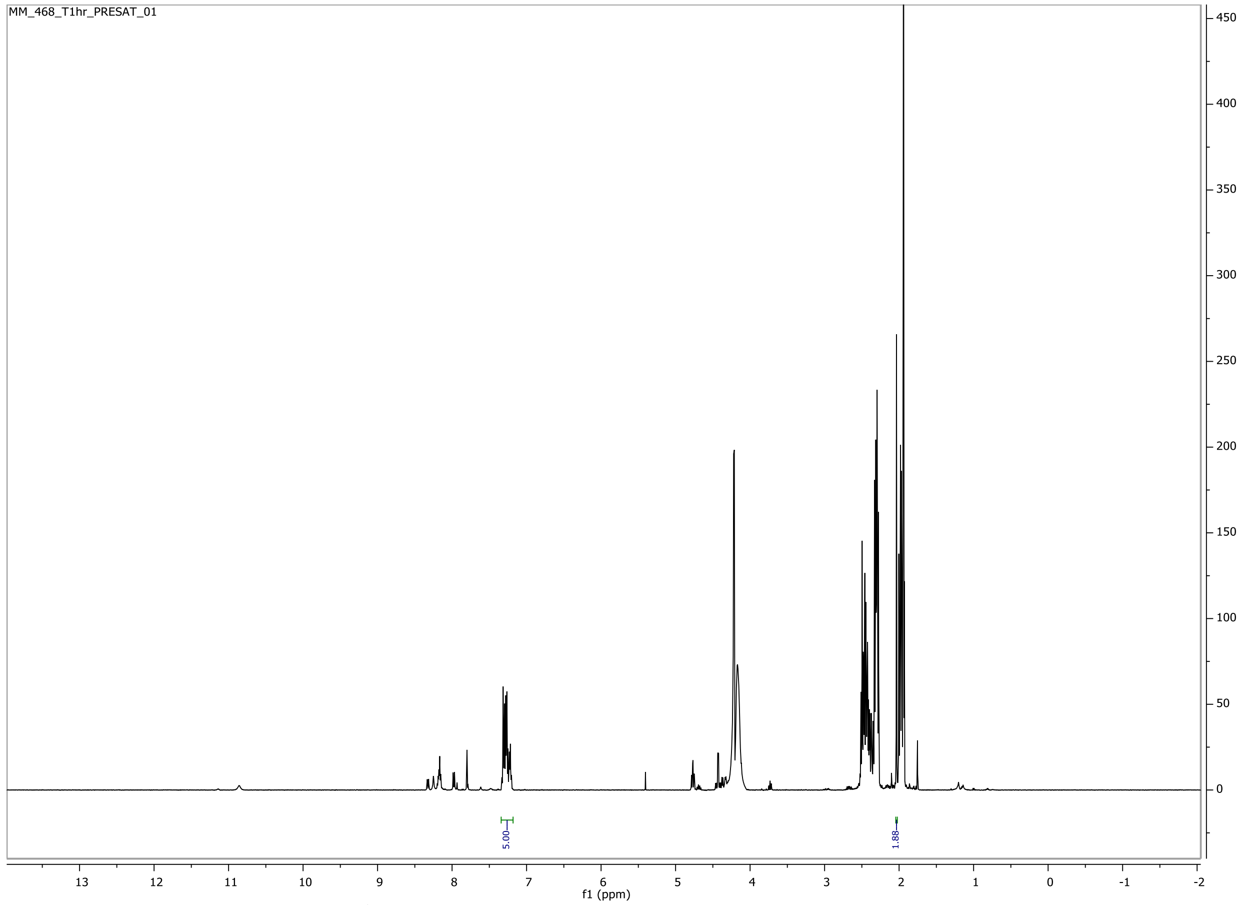

*Figure H78: Presaturation ^{1}H NMR spectrum (500 MHz) of the reaction mixture derived from the TCEP reduction of isothiazolone (**6**) in H_2O/CD_3CN (pH 5.88) in the presence of excess TCEP following 1 hour, exhibiting a conversion percentage of 63%.*

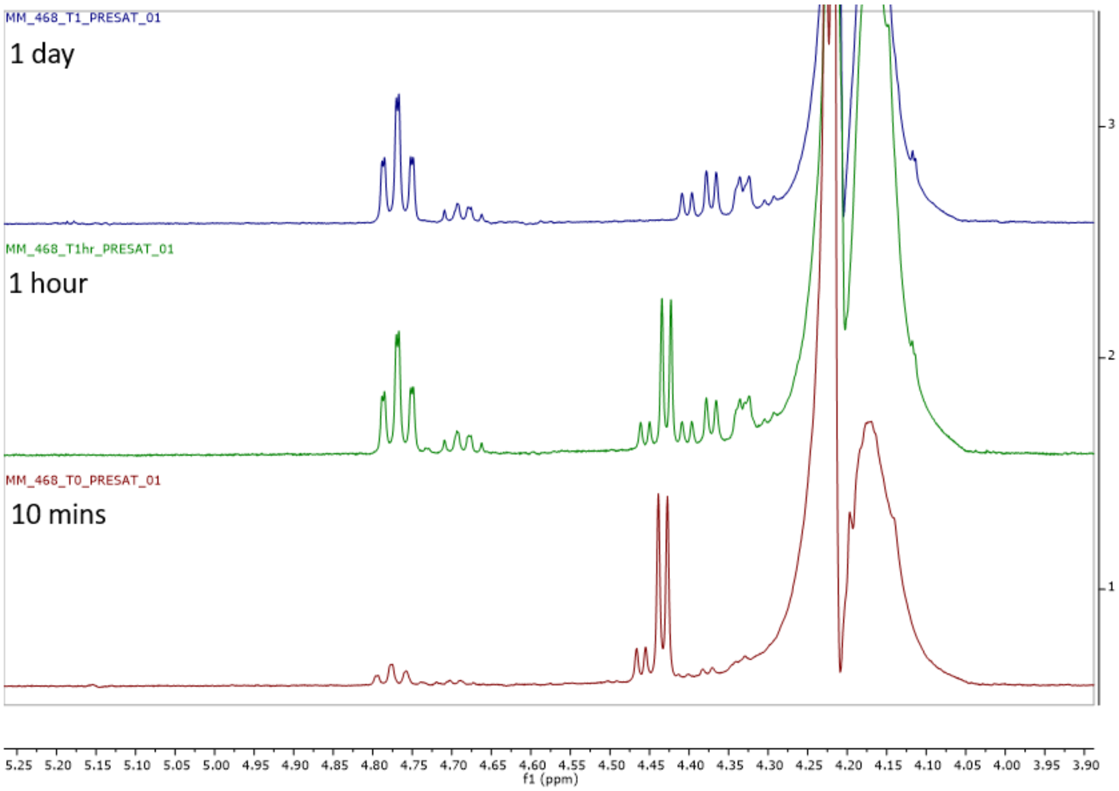

Figure H79: Stacked and zoomed in image of the ^{1}H *NMR spectrum (500 MHz) of the reaction mixture derived from the TCEP reduction of isothiazolone (**6**) in* H_2O/CD_3CN *(pH 5.88) in the presence of excess TCEP following 10 mins, 1 hour and 1 day. The methine signals corresponding to the proton of the* α *centre of the both isomers of the phosphonium adduct appears at 4.66 ppm, 4.77 ppm. The benzyl methylene signals of both isomers of enethiolate appears at 4.43 ppm, 4.45 ppm. The benzyl methylene signals of the phosphonium adduct appears as a doublet of doublet at 4.39 ppm, 4.31 ppm. Note: the significant reduction of the intensity of the signals corresponding to the enethiolate over time.*

Investigation of the mechanism of formation for the phosphonium adduct

To determine whether the phosphonium adduct could form by 1,4-addition of the phosphine to the enethiol, the corresponding benzyl-vinyl sulfide was prepared and subsequently treated with TCEP. Over the course of 2 days, no phosphonium adduct was detected, providing evidence that the phosphonium adduct was likely generated through 1,2-addition of TCEP to the transient cysteine thioaldehyde.

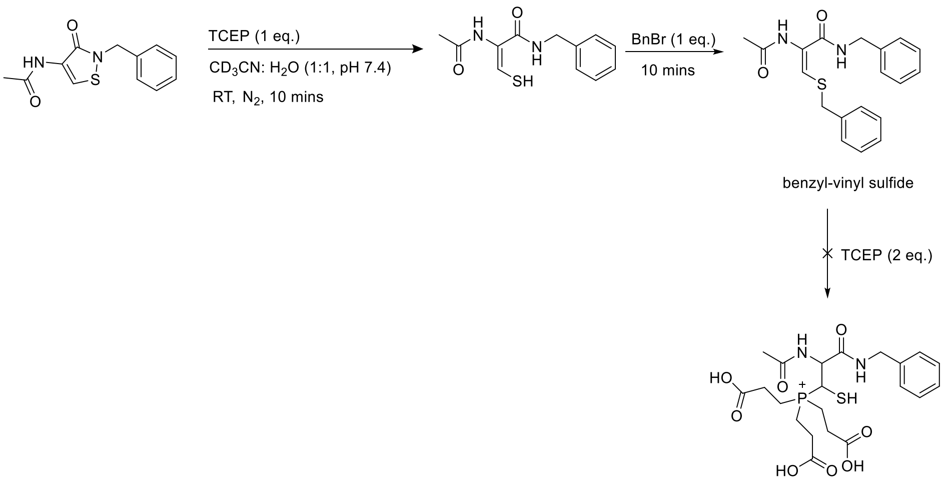

N-(2-benzyl-3-oxo-2,3-dihydro-1,2-thizol-4-yl) acetamide **6** (2.5 mg, 0.01 mmol) was dissolved in H_2O/CD_3CN (1:1, 1 mL). A H_2O solution of TCEP (0.04 M, pH 7.4, 0.01 mmol) preneuatralised with 2M NaOH solution was added. The resulting reaction mixture was allowed to evolve for 10 mins and was then analysed by ^1H NMR spectroscopy. A solution of benzyl bromide (0.1 M, 0.01 mmol) in CD_3CN was added followed by analysis through ^1H NMR spectroscopy. The benzyl-vinyl sulfide formed was then treated with a H_2O solution of TCEP (0.04 M, 0.02 mmol) adjusted to pH 7.4 with 2M NaOH. The reaction mixture was allowed to evolve for 10 mins and was then analysed by ^1H NMR spectroscopy. No signals corresponding to phosphonium adduct were detected. Over the course of two days, benzyl vinyl sulfide precipitated from the reaction mixture. The resulting solid was collected by centrifugation, redissolved in CD_3CN, and subsequently analyzed by NMR spectroscopy.

NMR characterisation of (*E*)-2-acetamido-N-benzyl-3-(benzylthio)acrylamide:

^1H NMR (CD_3CN, 500 MHz): δ 8.56 (s, NH), 7.63 (t, J = 5.3 Hz, NH),
7.41– 7.12 (m, 10H), 6.86 (s, 1H), 4.29 (d, J = 6.3 Hz, 2H), 3.86 (s, 2H), 1.94 ppm (s, 1H).

^{13}C NMR (CD_3CN, 151 MHz): δ 171.4, 164.6, 139.5, 138.7, 137.2, 129.8, 129.6, 129.3, 128.2, 128.2, 127.8, 122.6, 43.6, 40.4, 23.4 ppm.

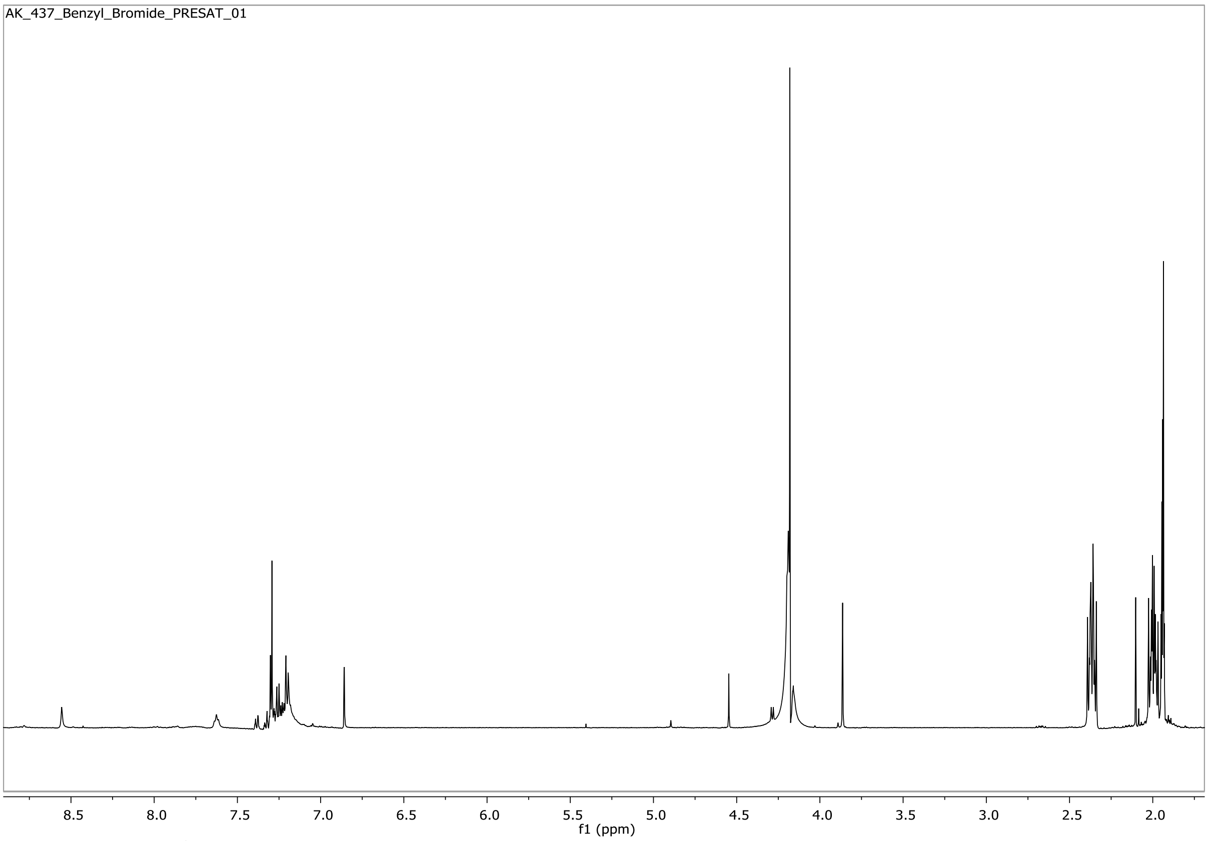

*Figure H80: ^{1}H NMR spectrum (500 MHz) of the reaction mixture derived from the TCEP reduction of isothiazolone (**6**) in H_2O/CD_3CN followed by addition of benzyl bromide. ^{1}H NMR spectrum of the reaction mixture shows complete conversion of enethiolate into benzyl-vinyl sulfide after 10 mins.*

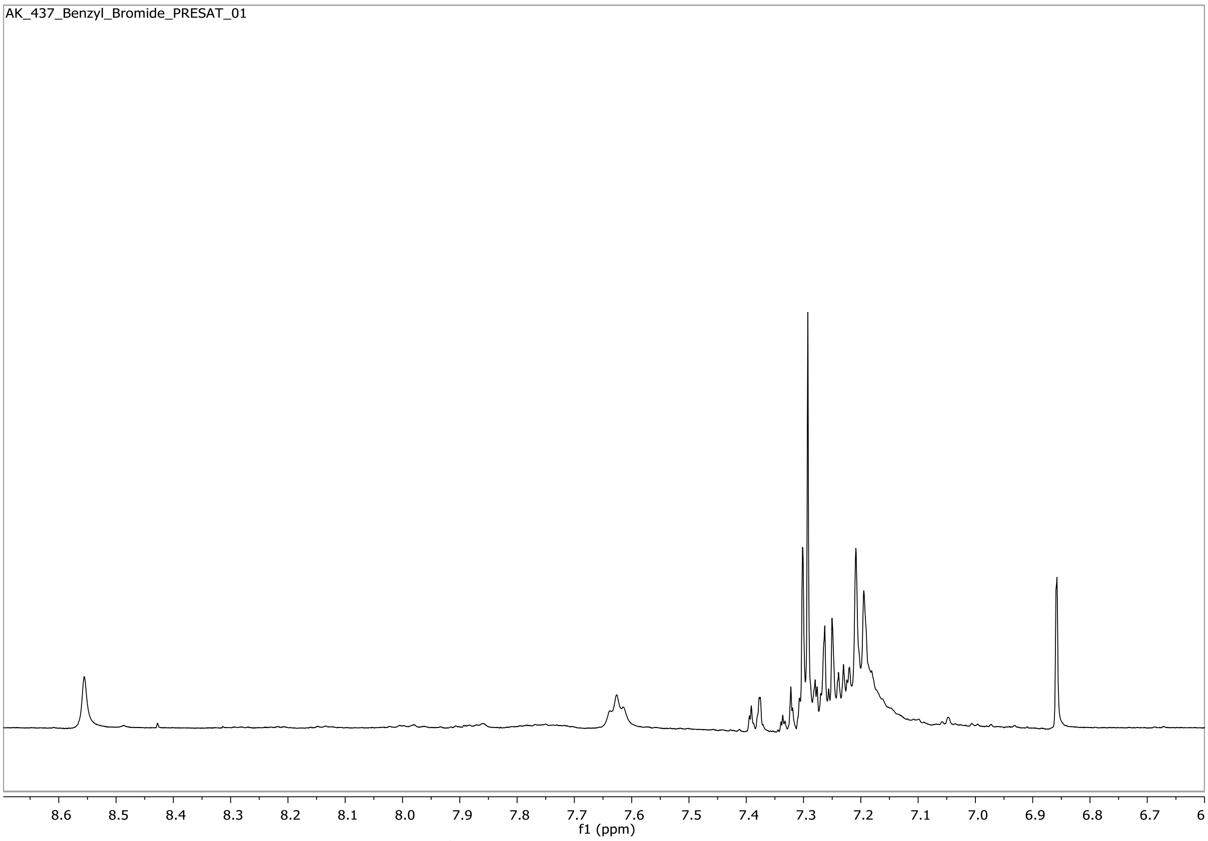

*Figure H81: Zoomed in image of ^{1}H NMR spectrum of the reaction mixture derived from the TCEP reduction of isothiazolone (**6**) in H_2O/CD_3CN followed by addition of benzyl bromide. The methine signal of benzyl vinyl sulfide appears at 6.85 ppm. Singlet at 8.56 ppm and triplet at 7.63 ppm corresponds to the amine protons of benzyl-vinyl sulfide.*

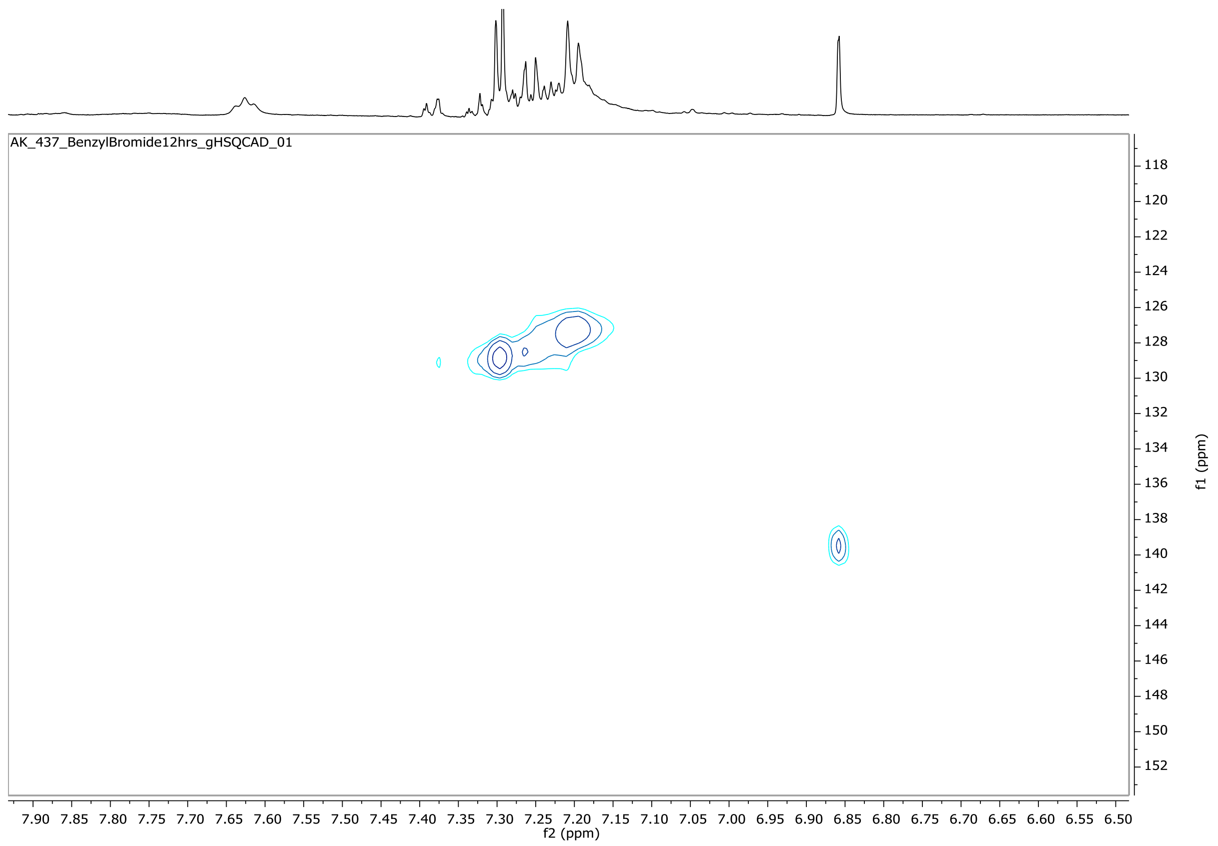

*Figure H82: Zoomed in image of HSQC spectrum of the reaction mixture derived from the TCEP reduction of isothiazolone (**6**) in H_2O/CD_3CN followed by addition of benzyl bromide. The methine signal of benzyl vinyl sulfide at 6.85 ppm shows a correlation to the ^{13}C signal at 139 ppm.*

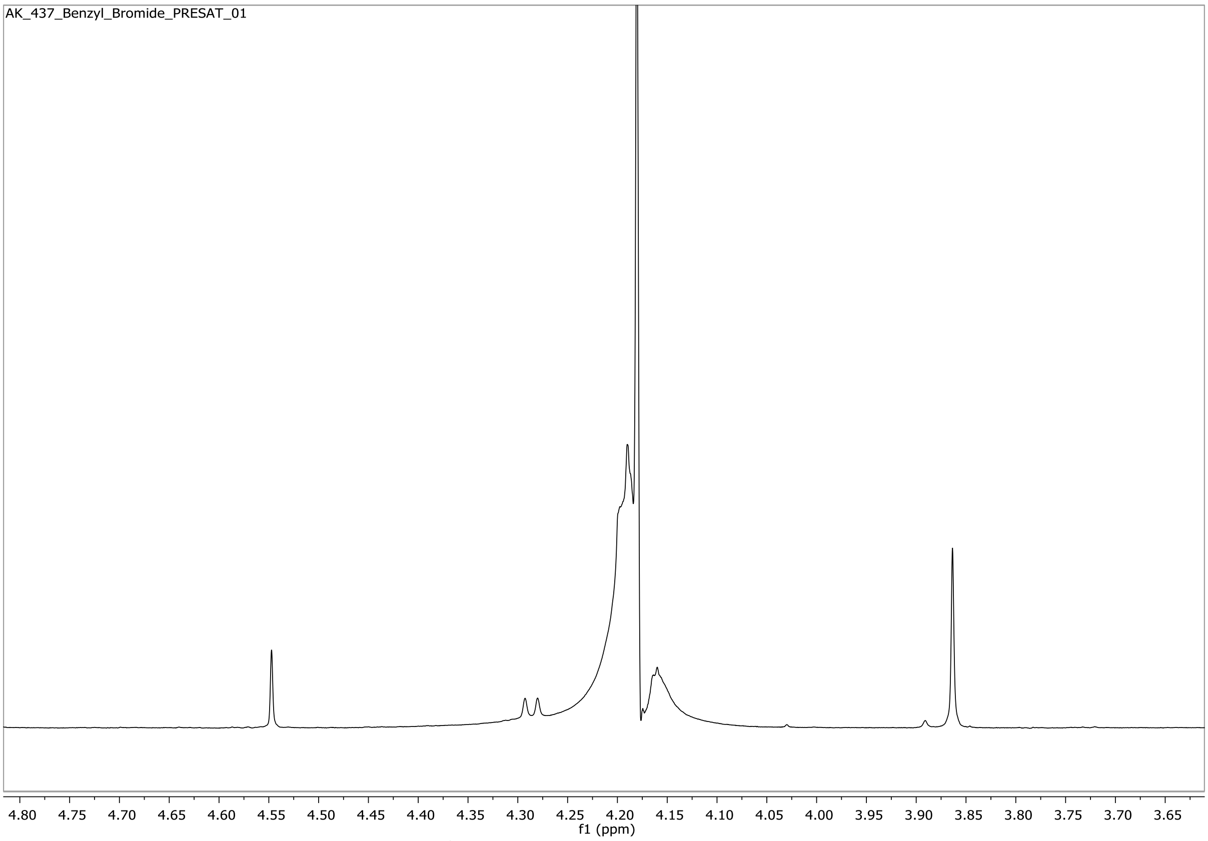

*Figure H83: Zoomed in image of ^{1}H NMR spectrum of the reaction mixture derived from the TCEP reduction of isothiazolone (**6**) in H_2O/CD_3CN followed by addition of benzyl bromide. The singlet at 3.86 ppm corresponds to the methylene protons of the S-benzyl group of the benzyl vinyl sulfide. The doublet at 4.30 ppm corresponds to the methylene protons of the N-benzyl group of the benzyl vinyl sulfide. The singlet at 4.55 ppm is the methylene protons of excess benzyl bromide present in the reaction mixture.*

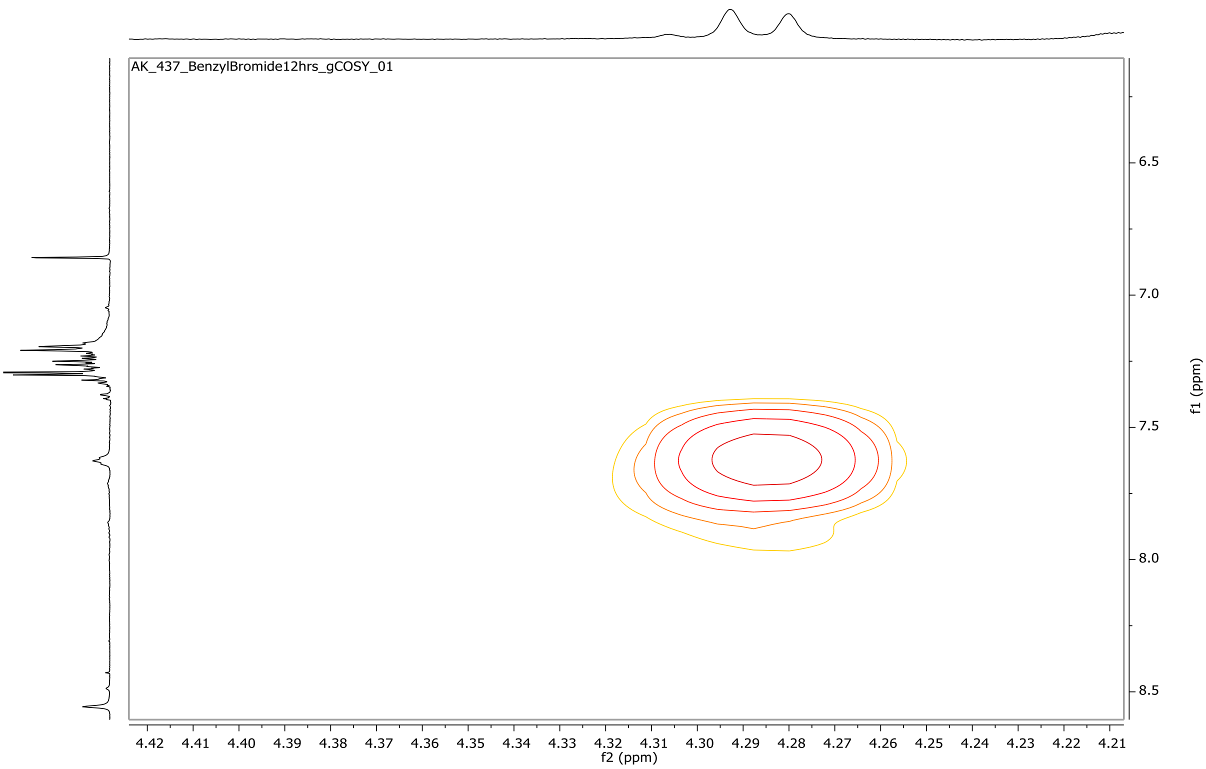

*Figure H84: Zoomed in image of the COSY spectrum of the reaction mixture derived from the TCEP reduction of isothiazolone (**6**) in H_2O/CD_3CN followed by addition of benzyl bromide. The doublet at 4.28 ppm corresponds to the methylene protons of the N-benzyl group of the benzyl-vinyl sulfide which shows a correlation to the amine proton signal appearing as a triplet at 7.63 ppm.*

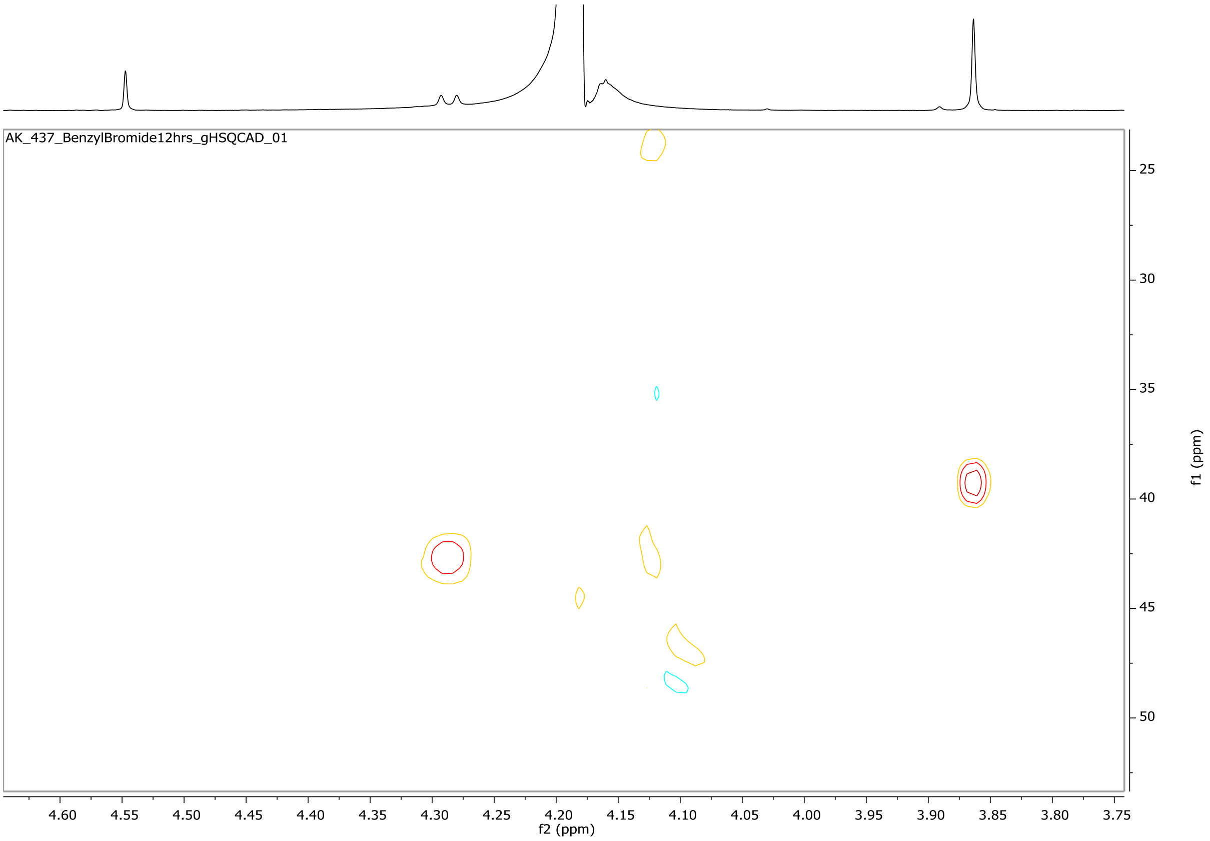

*Figure H85: Zoomed in image of the HSQC spectrum of the reaction mixture derived from the TCEP reduction of isothiazolone (**6**) in H_2O/CD_3CN followed by addition of benzyl bromide. The doublet corresponding to the methylene protons of the N-benzyl group of the benzyl-vinyl sulfide at 4.28 ppm shows a correlation to a ^{13}C signal at 42.8 ppm. The singlet corresponding to the methylene protons of the S-benzyl group of the benzyl vinyl sulfide shows a correlation to a ^{13}C signal at 39.3 ppm.*

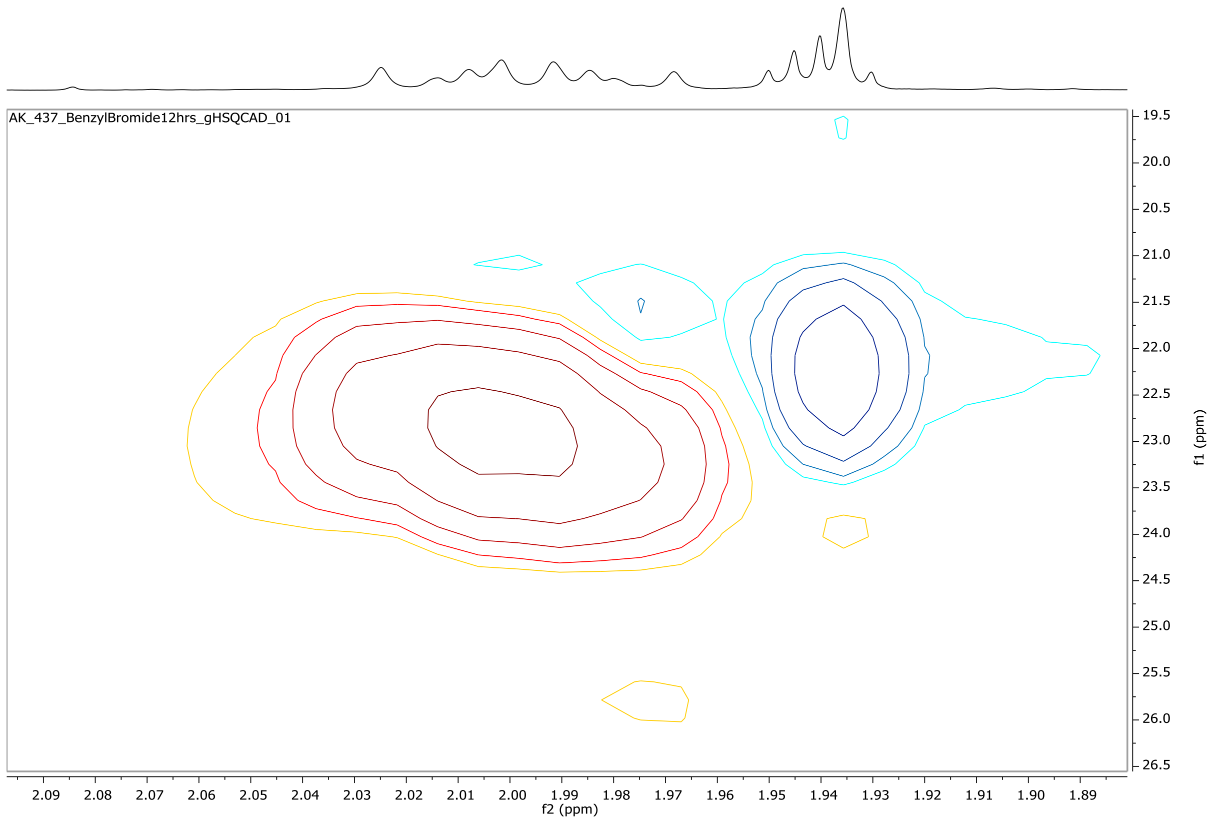

*Figure H86: Zoomed in image of the HSQC spectrum of the reaction mixture derived from the TCEP reduction of isothiazolone (**6**) in H_2O/CD_3CN followed by addition of benzyl bromide. The singlet corresponding to the methyl protons of the N-acetyl group of the benzyl-vinyl sulfide is overlapping with the residual acetonitrile solvent peak. The methyl protons of the N-acetyl group of the benzyl-vinyl sulfide at 1.94 ppm shows a correlation to a ^{13}C signal at 22.1 ppm.*

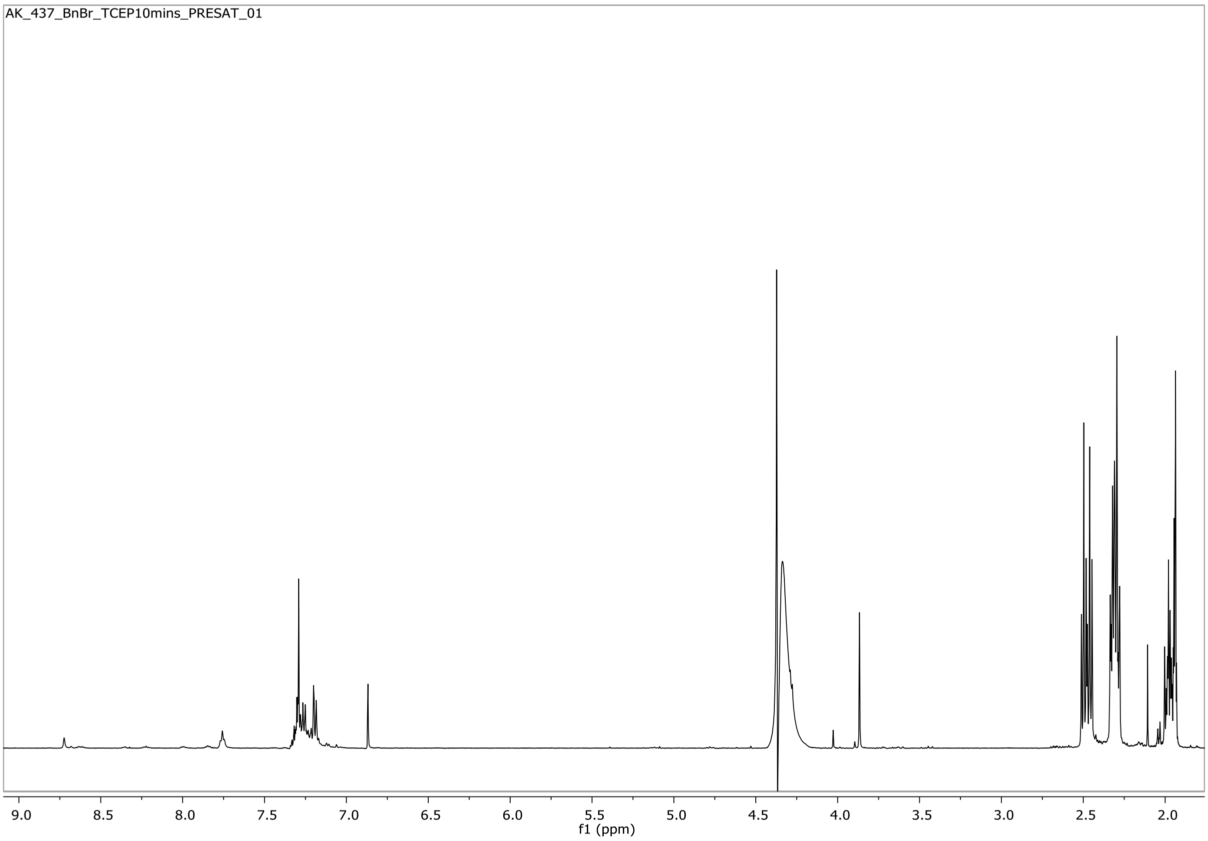

*Figure H87: ^{1}H NMR spectrum of the reaction mixture derived from the TCEP reduction of isothiazolone (**6**) in H_2O/CD_3CN followed by addition of benzyl bromide and TCEP. The ^{1}H NMR signals corresponding to benzyl-vinyl sulfide remains unchanged even after the addition of 2 equivalents of TCEP. This indicates that TCEP does not undergo 1,4-addition to the enethiol/enesulfide species under the reaction conditions. The methylene protons of TCEP appears as two multiplets, one at 2.26-2.35 ppm and the other at 2.44-2.52 ppm. Note: the multiplet of TCEP appearing at 2.26-2.35 ppm overlaps with the multiplet of oxidised TCEP appearing in the same region.*

Investigation into isothiazolone (6) as masked a cysteine thioaldehyde: Reduction at pH 5.9 in H_2O in the absence of buffer.

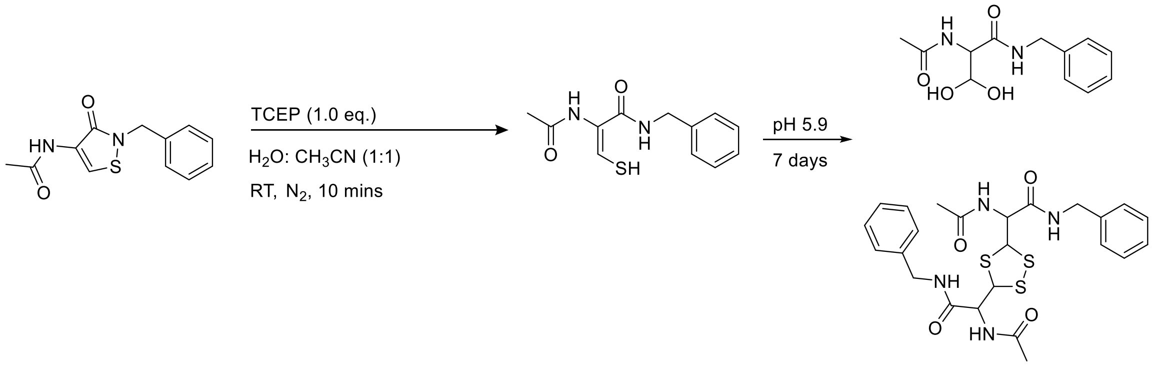

N-(2-benzyl-3-oxo-2,3-dihydro-1,2-thizol-4-yl) acetamide **6** (4.3 mg, 0.0173 mmol) was dissolved in H_2O/CD_3CN (1:1, 2 mL). A H_2O solution of TCEP (1M, pH 7.4, 0.0173 mmol) was added. The resulting reaction mixture was allowed to evolve for 10 mins and was then analysed by ^1H NMR spectroscopy. The pH of the reaction mixture was then measured to be 5.9. The reaction mixture was analysed by ^1H NMR spectroscopy over 7 days.

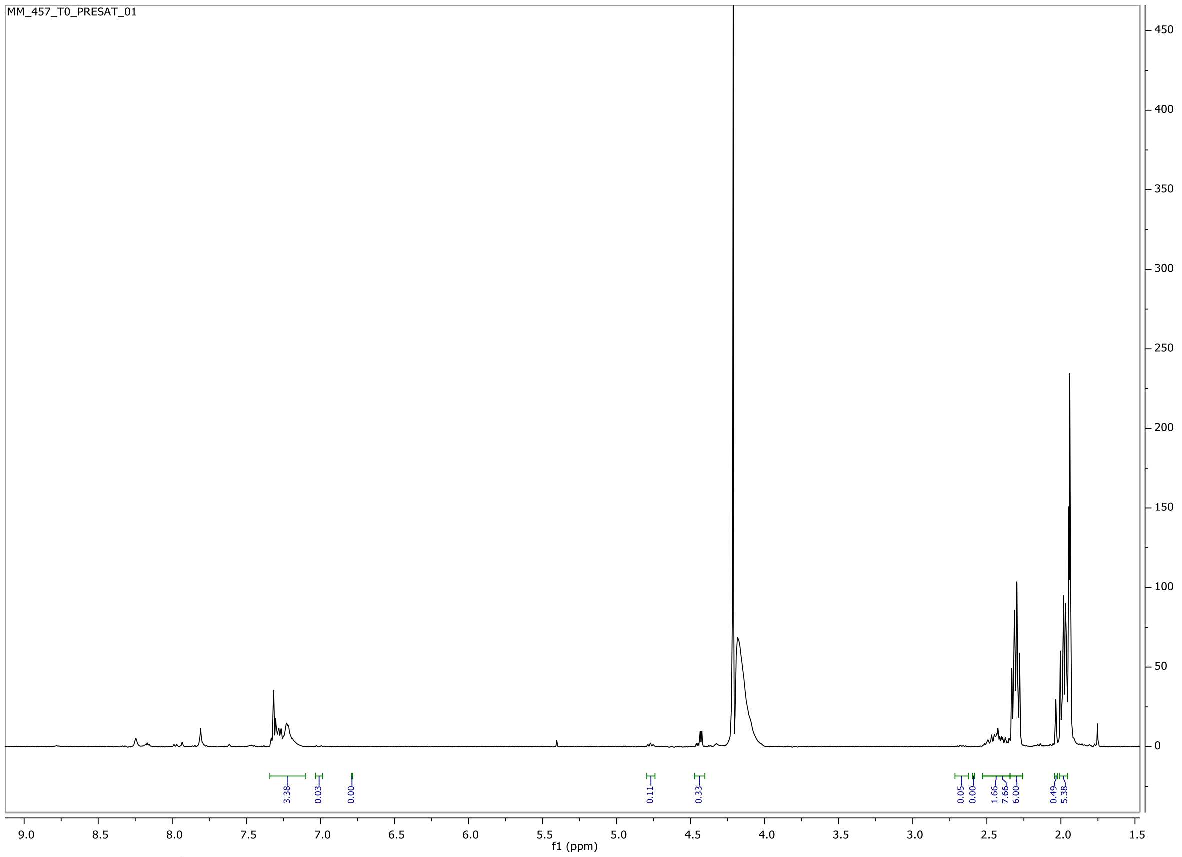

*Figure H88: ^1H NMR spectrum (500 MHz) of the reaction mixture derived from the TCEP reduction of isothiazolone (**6**) in H_2O/CH_3CN (pH 5.9).*

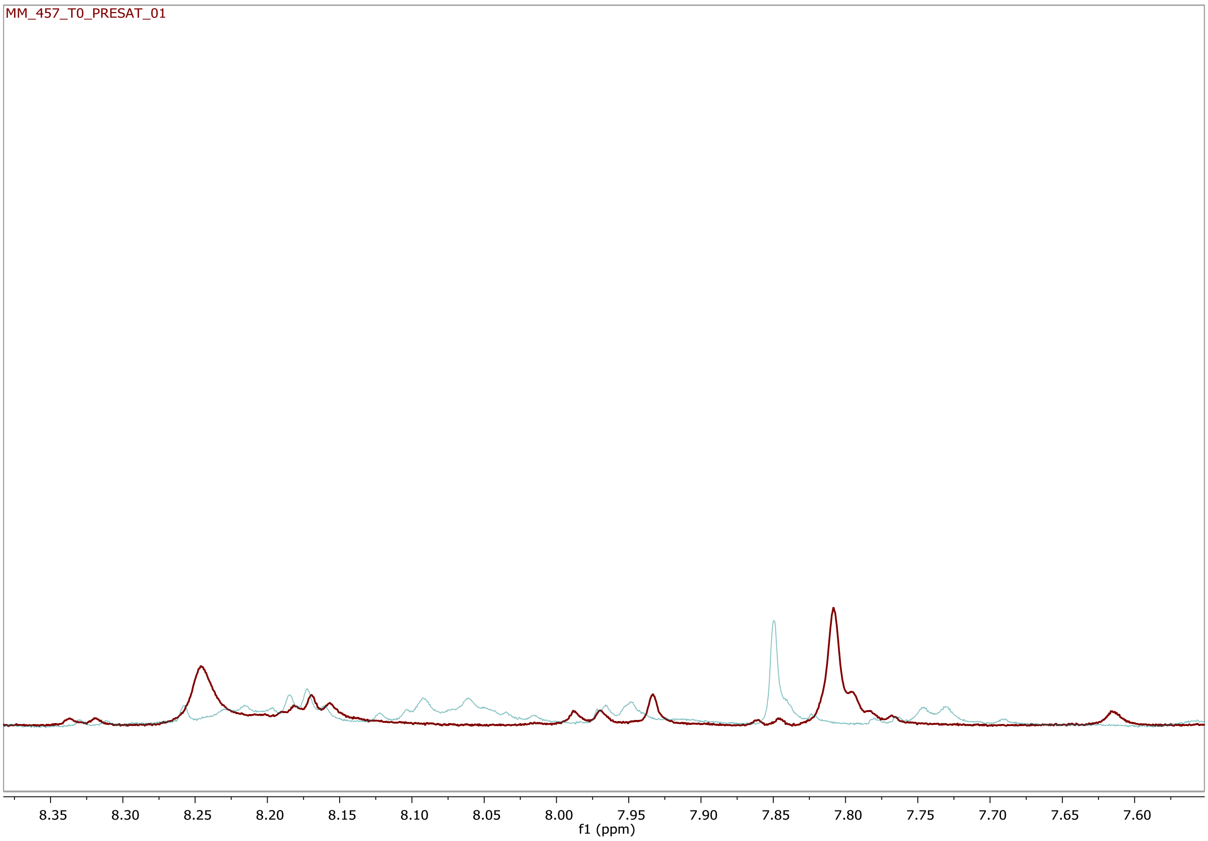

*Figure H89: Superimposed and zoomed-in image of the downfield region of the ^{1}H NMR spectrum of the reaction mixture derived from the TCEP reduction of isothiazolone (**6**) in H_2O/CD_3CN (pH 5.9) on day 0 (red) and day 7 (green). The methine signal of enethiol appearing at 7.80 ppm on day 0 (pH 5.9) moves to 7.85 ppm by day 7 (pH 6.4) due to change in the pH of the reaction mixture.*

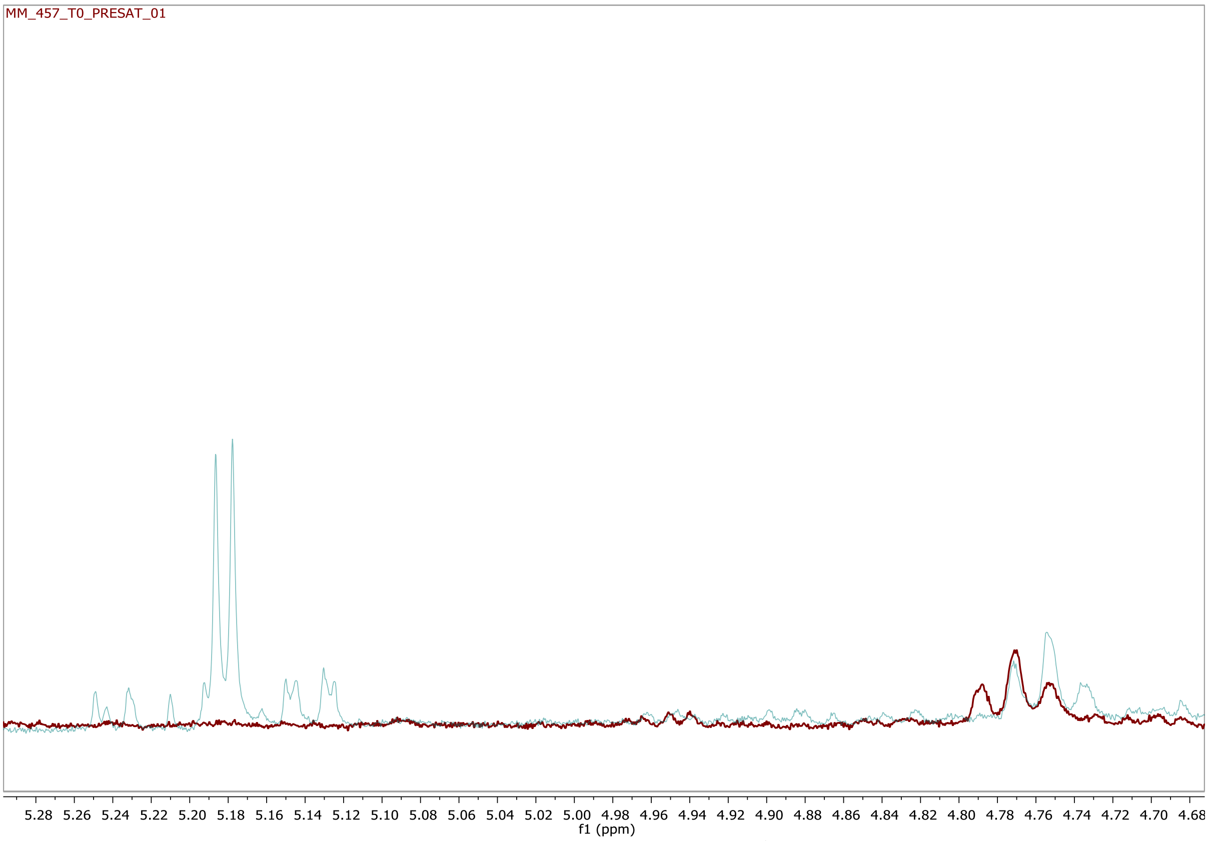

*Figure H90: Superimposed and zoomed-in image of the ^{1}H NMR spectrum of the reaction mixture derived from the TCEP reduction of isothiazolone (**6**) in H_2O/CD_3CN (adjusted to pH 5.9) on day 0 (red) and day 7 (green). The methine signals of the gem-diol (5.18 ppm) and diastereromeric 1,2,4-trithiolanes (5.26–5.12 ppm), are shown to form over the course of 7 days. The signal of the phosphonium adduct appearing at 4.78 ppm on day 0 (pH 5.9) moves to 4.75 ppm by day 7 (pH 6.4) due to change in the pH of the reaction mixture.*

*Table H1: Relative amount (%) of species in reaction mixture derived from the TCEP reduction of isothiazolone (**6**) in H_2O (pH 5.9) /CD_3CN at Day 0 and at Day 7.*

	**Enethiol**	**Isothiazolone**	**Thiazole**	**1,2,4-Trithiolanes (5.24–5.12 ppm)**	***Gem*-diol (5.17 ppm)**
Day 0	35	0	0	0	0
Day 7	17	0	0	9	11

Investigation into isothiazolone (6) as a masked cysteine thioaldehyde: Reduction at pH 6 in H_2O in the absence of buffer under dilute conditions.

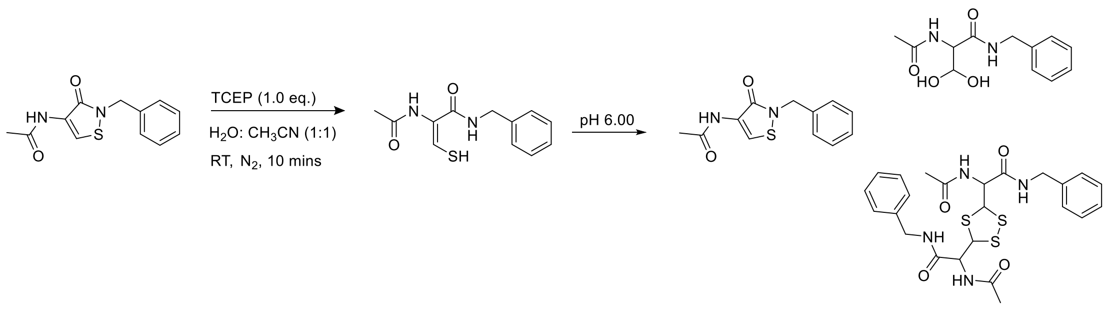

N-(2-benzyl-3-oxo-2,3-dihydro-1,2-thizol-4-yl) acetamide **6** (4.3 mg, 0.0173 mmol) was dissolved in 0.1 M phosphate H_2O/CD_3CN (1:1, 2 mL). A H_2O solution of TCEP (1M, pH 7.4, 0.0173 mmol) was added. The resulting reaction mixture was allowed to evolve for 10 mins and was then analysed by ^1H NMR spectroscopy. The pH of the sample was then measured to be 6.0. The solution was then diluted with a 1:1 solution of $H_2O:CD_3CN$ to a final concentration of 1.7 mM. The reaction mixture was stored under N_2 and analysed by ^1H NMR spectroscopy over 7 days.

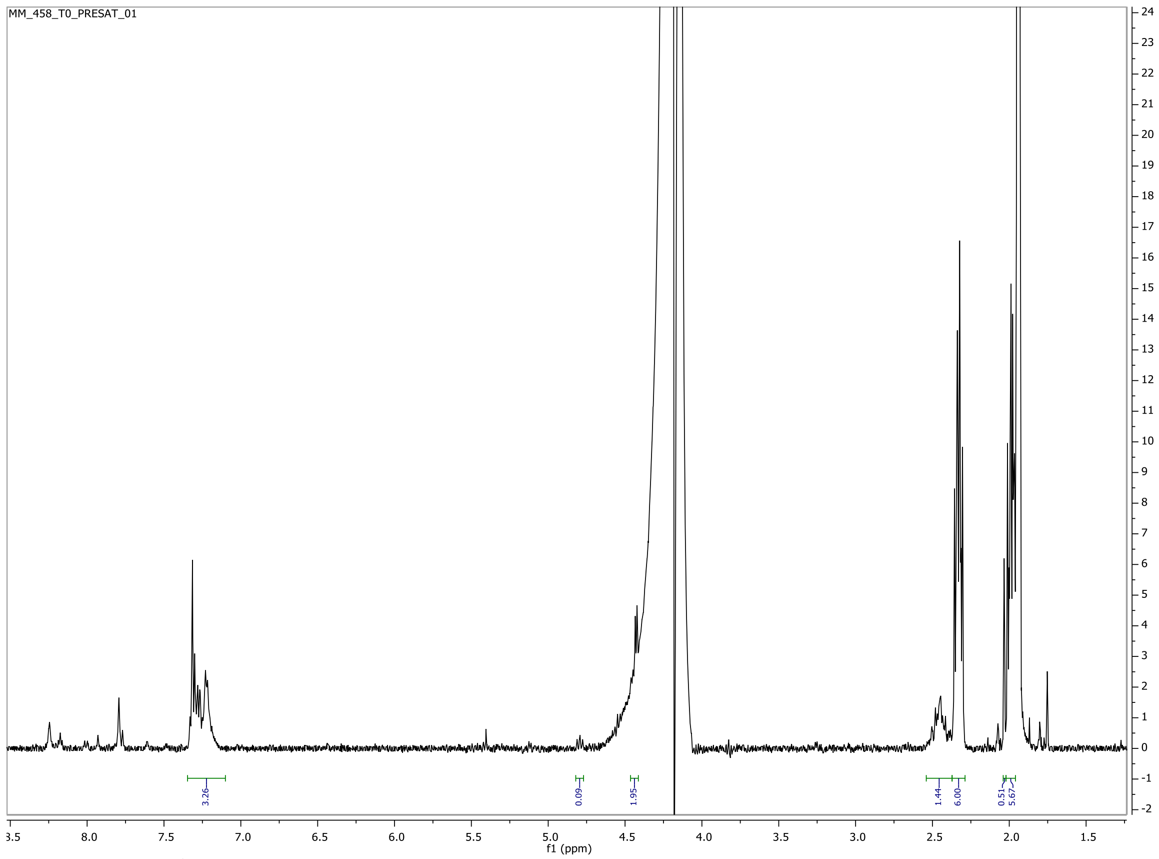

*Figure H91: ^1H NMR spectrum of the reaction mixture derived from the TCEP reduction of isothiazolone (**6**) in H_2O/CH_3CN (pH 6.0).*

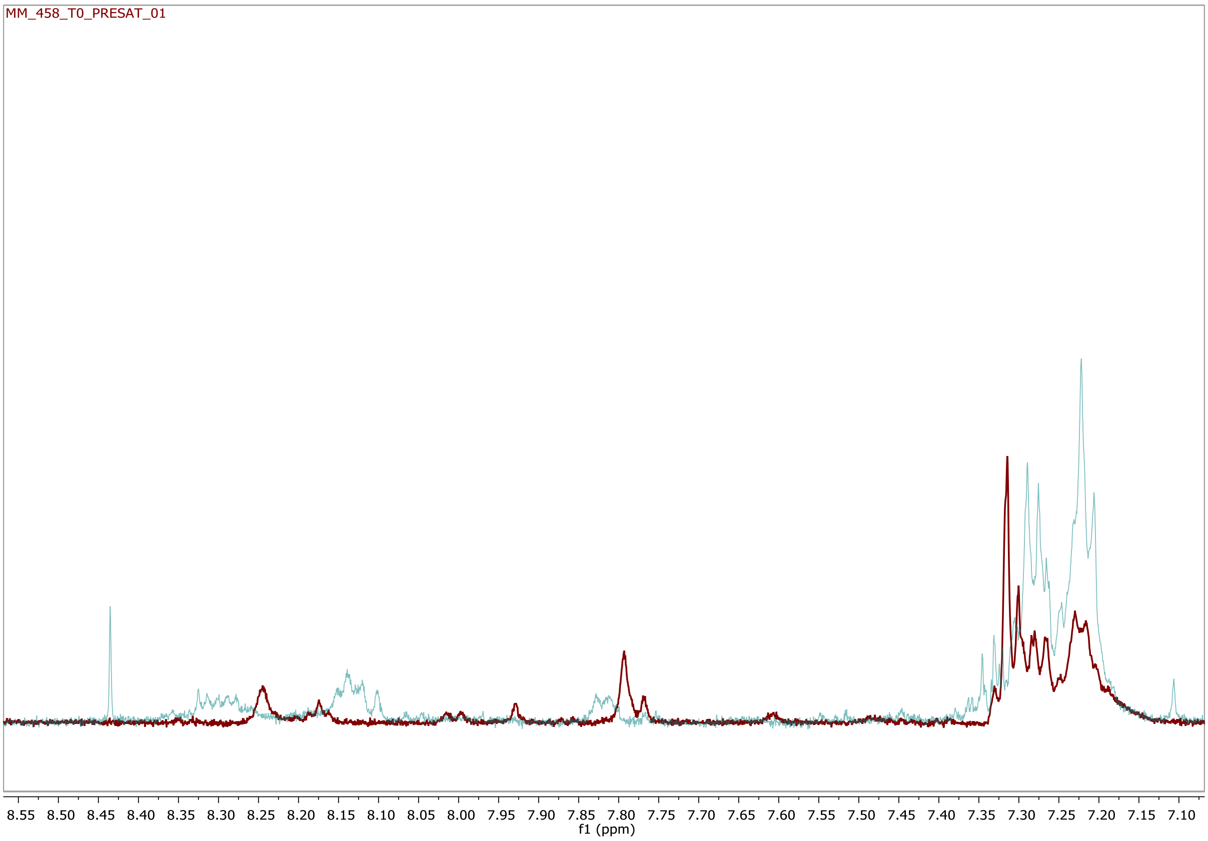

*Figure H92: Superimposed and zoomed-in image of the downfield region of the ^{1}H NMR spectrum of the reaction mixture derived from the TCEP reduction of isothiazolone (**6**) in H_2O/CD_3CN (pH 6) on day 0 (red) and day 7 (green). The methine signal of enethiol appears at 7.80 ppm on day 0 (pH 6.0) and reduces significantly by day 7.*

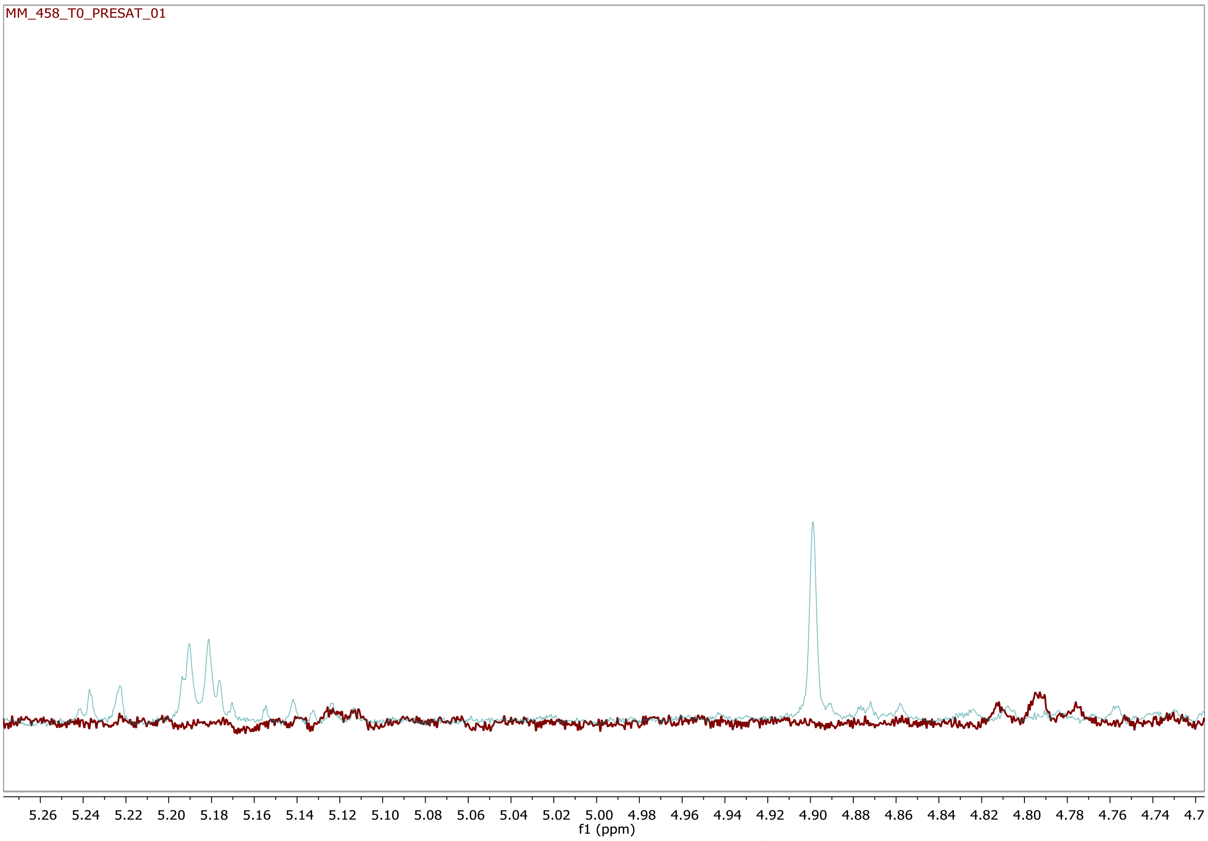

*Figure H93: Superimposed and zoomed-in image of the ^{1}H NMR spectrum of the reaction mixture derived from the TCEP reduction of isothiazolone (**6**) in H_2O/CD_3CN (pH 6.0) on day 0 (red) and day 7 (green). The methine signals of the gem-diol (5.18 ppm) and diastereromeric 1,2,4-trithiolanes (5.26–5.12 ppm), are shown to form over the course of 7 days. The methylene signal of isothiazolone appears at 4.90 ppm on day 7. The signal of the phosphonium adduct (4.79 ppm) decreases in intensity over the course of 7 days.*

*Table H2: Relative amount (%) of species in reaction mixture derived from the TCEP reduction of isothiazolone (**6**) in H_2O (pH 6.0) /CD_3CN at Day 0 and at Day 7.*

	**Enethiol (7.81 ppm)**	**Isothiazolone (2.08 ppm)**	**Thiazole**	**1,2,4-Trithiolanes (5.24–5.12 ppm)**	***Gem*-diol (5.17 ppm)**
Day 0	27	0	0	0	0
Day 7	0	16	0	23	17

Investigation into isothiazolone (6) as a masked cysteine thioaldehyde: Reduction at pH 2.2 in H_2O in the absence of buffer.

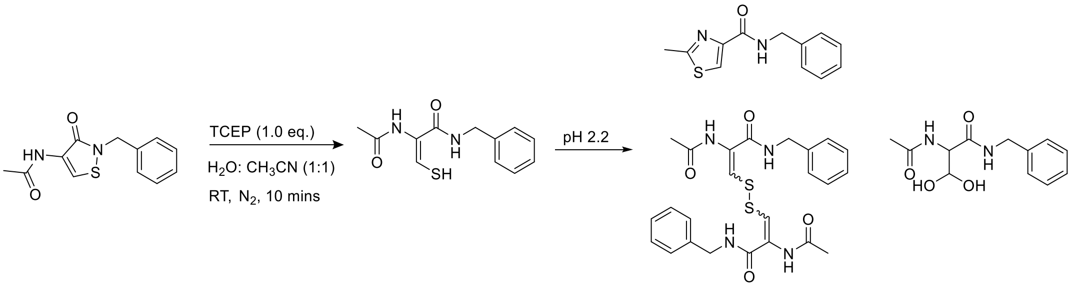

N-(2-benzyl-3-oxo-2,3-dihydro-1,2-thizol-4-yl) acetamide **6** (4.3 mg, 0.0173 mmol) was dissolved in H_2O/CD_3CN (1:1, 2 mL). A H_2O solution of TCEP (1M, pH 7.4, 0.0173 mmol) was added. The resulting reaction mixture was allowed to evolve for 10 mins and was then analysed by ^1H NMR spectroscopy. The pH of the sample was then adjusted to pH 2.2 through addition of 2 M HCl in H_2O. The reaction mixture was stored under N_2 analysed by ^1H NMR spectroscopy over 3 days.

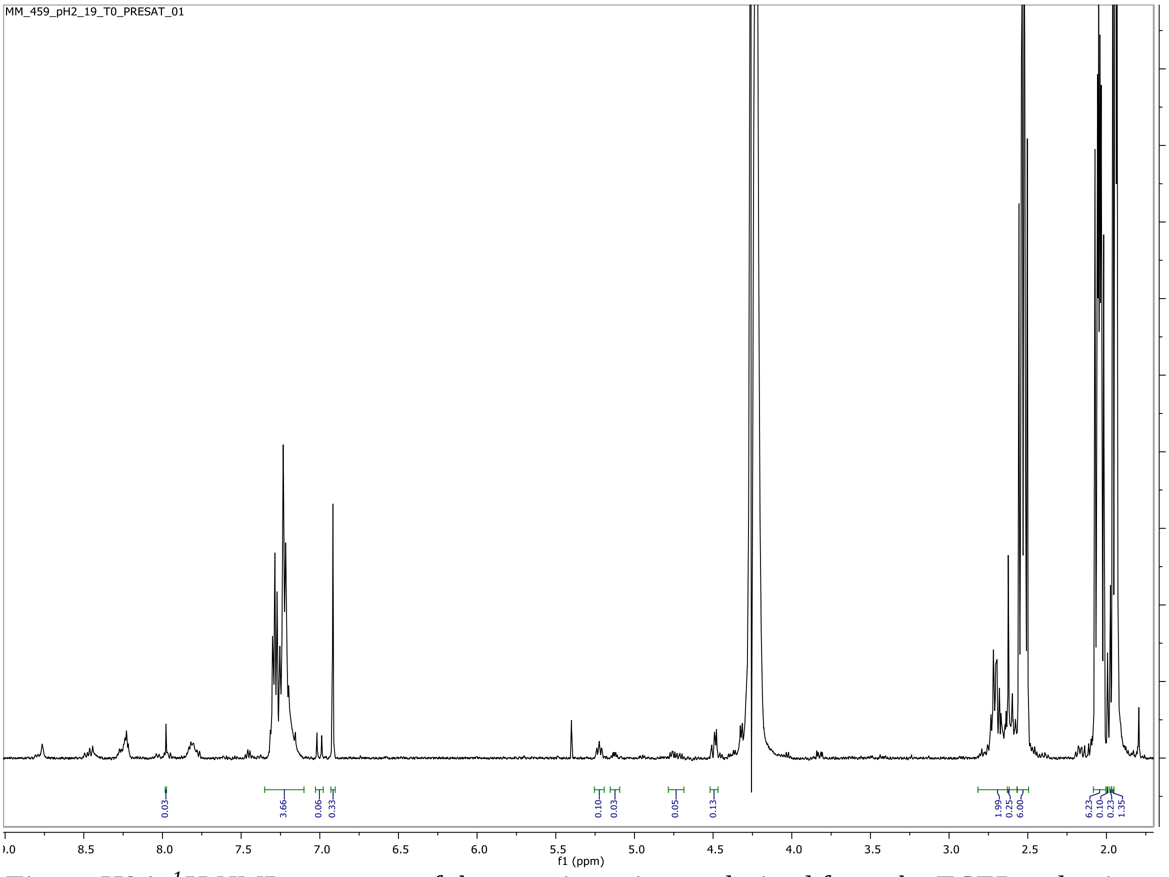

*Figure H94: ^1H NMR spectrum of the reaction mixture derived from the TCEP reduction of isothiazolone (**6**) in H_2O/CH_3CN (adjusted to pH 2.2).*

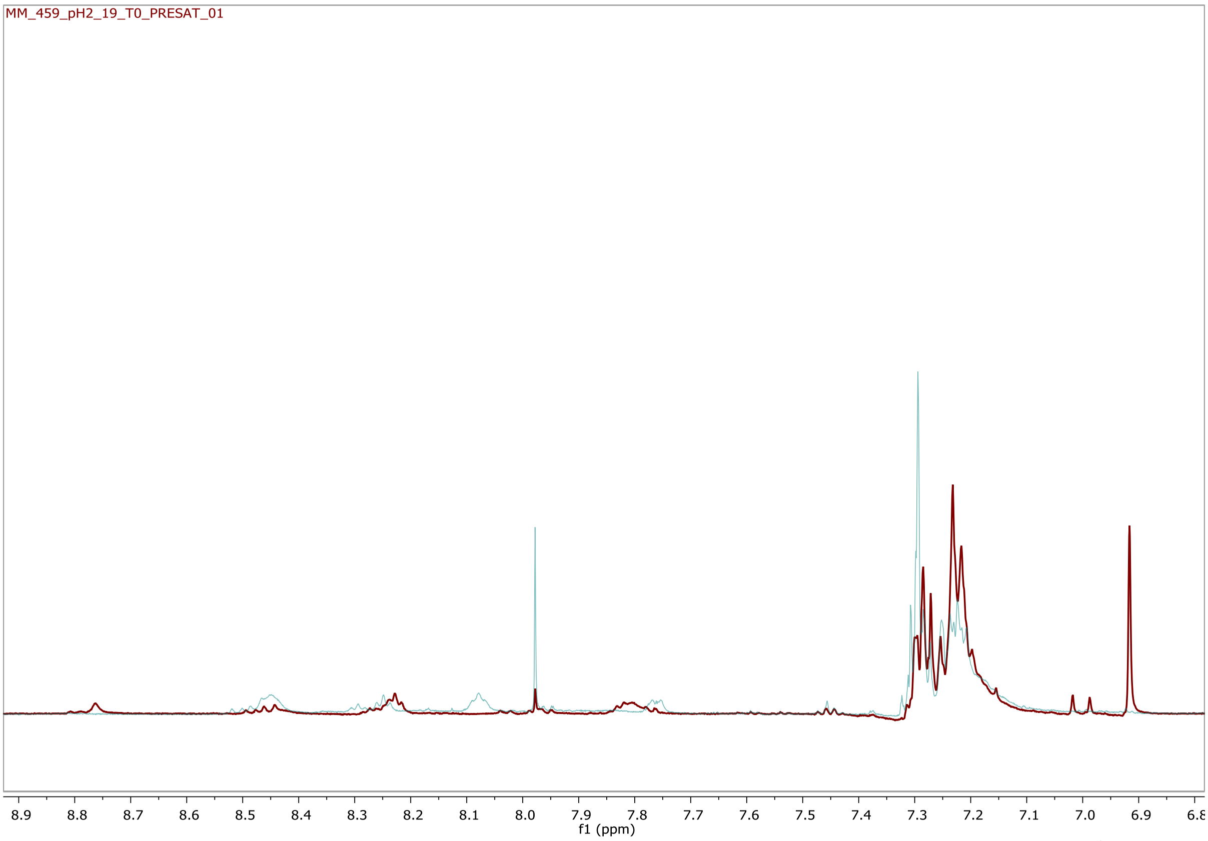

*Figure H95: Superimposed and zoomed-in image of the downfield region of the ^{1}H NMR spectrum of the reaction mixture derived from the TCEP reduction of isothiazolone (**6**) in H_2O/CD_3CN (adjusted to pH 2.2) on day 0 (red) and day 7 (green). The methine signal of enethiol appears at 6.9 ppm on day 0 and reduces significantly by day 7.*

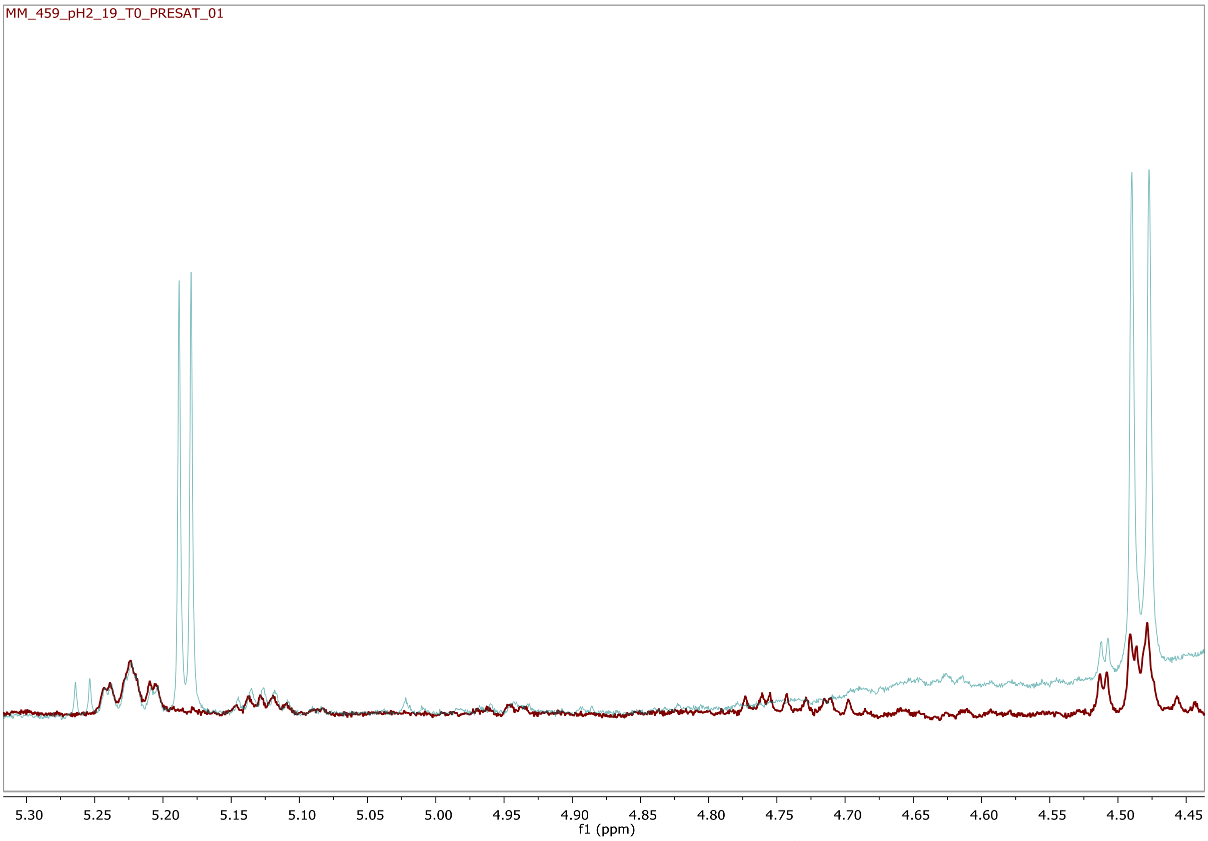

*Figure H96: Superimposed and zoomed-in image of the of the ^{1}H NMR spectrum of the reaction mixture derived from the TCEP reduction of isothiazolone (**6**) in H_2O/CD_3CN (adjusted to pH 2.2) on day 0 (red) and day 7 (green). The methine signal of the gem-diol (5.17 ppm) appears at day 7. The methine signals of the diastereromeric 1,2,4-trithiolanes (5.26 ppm) appears at day 7.The methine signal of the phosphonium adduct (5.22 ppm) shows no change over time. The benzyl methylene signals of both enethiol isomers (4.46-4.44 ppm) overlaps with the benzyl methylene signals of thiazole (4.46 ppm) at day 0. The benzyl methylene signals of enethiol isomers decreases in intensity by day 7 and the benzyl methylene signals of thiazole increases in intensity by day 7.*

*Table H3: Relative amount (%) of species in reaction mixture derived from the TCEP reduction of isothiazolone (**6**) in H_2O (pH 2.2) /CD_3CN at Day 0 and at Day 7.*

	**Enethiol (1.96 ppm)**	**Isothiazolone (2.08 ppm)**	**Thiazole (2.62 ppm)**	**1,2,4-Trithiolanes (5.24–5.12 ppm)**	***Gem*-diol (5.17 ppm)**
Day 0	33	0	20	0	0
Day 7	0	0	30	1	18

I. Synthesis and photolysis of tripeptide phenacylsulfide derivatives

General procedure for automated solid-phase peptide synthesis

(i) **Materials and instrumentation**: Automated solid-phase peptide synthesis was carried out on a CEM Multipep 1 instrument. Protected amino acid derivatives were purchased from (Iris Biotech GmbH). Diisopropylcarbodimide (DIC), ethyl cyanohydroxyiminoacetate (Oxyma) and triisopropylsilane (TIS) were purchased from TCI chemicals. Acetic anhydride (Ac_2O), 4-methyl morpholine, trifluoroacetic acid (TFA) were purchased from Sigma Aldrich. Piperidine was purchased from Iris biotech GmbH. Washing steps were carried by using DMF (reagent grade, Fisher Scientific). All other steps (coupling, deprotection) were carried by using peptide-grade DMF (Iris Biotech GmbH). Dichloromethane (DCM), diethylether (DEE) and tert-butylmethylether (TBME) were purchased from Sigma Aldrich. All peptides were prepared as the terminally *N*-acetylated derivatives and were grown on a Rink-amide resin (0.67 mmol/g loading, Iris Biotech GmbH) to give the C-terminal primary amides. Peptides were generally prepared on a 100 μmol scale and all steps carried out at room temperature.

(ii) **Swelling of the resin**: The Fmoc-protected Rink-amide resin was weighed and placed in a disposable 5-mL reaction column with fritted disk (purchased from Carl Roth). DMF (5 mL) was added and the resin was allowed to swell for 30 minutes. The solvent was drained.

(iii) **Fmoc deprotection**: Piperidine in DMF (20 %, 5 mL) was added to the resin and the mixture was agitated for 5 min. The liquid phase was drained and the deprotection cycle was repeated for 10 min. The resin was finally washed with DMF (5 mL, x 5).

(iv) **Coupling**: Fmoc-protected amino acid (5 eq.) was dissolved in DMF (4 mL) followed by activation of the carboxylic acid through the addition of DIC (5 eq.) and Oxyma (5 eq.) and allowing the mixture to agitate for 5 minutes. The solution of activated amino acid was added to the resin and the mixture was agitated for 60 min. The liquid phase was drained and the resin washed with DMF (5 mL, x 5). Typically, the above coupling procedure was repeated (double coupling) to ensure complete reaction.

(v) **Capping**: Capping solution (acetic anhydride/*N*-methylmorpholine/DMF,(5:6:89), 5 mL) was added to the resin and the mixture was agitated for 5 minutes. The liquid phase was drained and the resin was washed with DMF (5 mL, x 5). Capping is typically carried out after coupling of each amino acid and after Fmoc deprotection of the last amino acid residue. *Note: the capping solution should be replaced every 12 hrs with freshly prepared material.*

(vi) **Cleavage from resin**: Before cleavage, the resin was washed with DMF (5 mL, x 5), DCM (5 mL, x 5), and DEE (5 mL, x 5) and allowed to dry. The cleavage cocktail (TFA/TIS/H_2O, 95:2.5:2.5, 5 mL) was added to the resin and the mixture was allowed to sit for 2 hrs. The liquid phase was then drained, collected in a 50 mL centrifuge tube and concentrated under a stream of air. Cold *tert*-butylmethylether (25 mL) was added to the residue resulting in precipitation. The

product was then isolated through centrifugation. The supernatant was decanted and the remaining precipitate was resuspended in *tert*-butyl methyl ether, centrifuged and decanted. The resulting pellet was dissolved in HPLC-grade water. The solution was then lyophilized to yield the crude peptide as a white powder.

(vii) **Analysis and Purification**: The crude peptide was analysed by analytical HPLC (reverse-phase C18 column), LC-HRMS (ESI) and sometimes NMR spectroscopy. When necessary, crude peptides were purified by using semi-preparative HPLC. Analytical HPLC was performed on an Agilent 1260 Infinity HPLC system with an Agilent 1260 Infinity auto sampler. An Agilent Zorbax RX-C18 column (4.6 x 250 mm, 5 μm packing diameter) at 24 $^{\circ}$C with a flow rate of 1 mL/min was used and the absorbance were measured at 214 nm. The eluent used was a gradient composed of mixtures of the following components: A = 0.1 % v/v solution of TFA in water; B = 0.1 % v/v solution of TFA in acetonitrile. Semi-preparative HPLC was performed on an Agilent 1260 Infinity HPLC system with an Agilent 1200 series auto sampler. A Macherey Nagel NUCLEODUR C18 HTec column (10 x 250 mm, 5 μm packing diameter) at 24 $^{\circ}$C with a flow rate of 2.5 mL/min was used and the absorbance were measured at 214 nm Mobile phase consisted of a linear gradient of water and acetonitrile (MeCN) each with 0.1% TFA. Detector wavelength was set to 214 nm
Note: *HPLC conditions were tailored to each substrate see in more detail below each HPLC trace.*

Solid phase peptide synthesis of Ac-Ala-Cys-Gly-NH_2 (21)

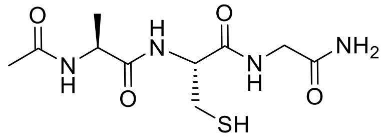

21

Ac-Ala-Cys-Gly-NH_2 was prepared according to the above general protocol on a 0.1 mmol scale by using the Fmoc—rink amide resin (0.67 mmol/g) (154 mg, 0.103 mmol). The crude peptide (28 mg, ca. 0.096 mmol) was deemed sufficiently pure for further manipulations.

^{1}H NMR (D_2O, 500 MHz): δ 4.49 (app. t, J = 6.4 Hz, 1H), 4.30 (q, J = 7.2 Hz, 1H), 3.95 (d, J = 17.2 Hz, 1H), 3.90 (d, J = 17.2 Hz, 1H), 3.00–2.90 (m, 2H), 2.02 (s, 3H), 1.38 ppm (d, J = 7.2 Hz, 3H).

HRMS (ESI) *m/z* (M+H)$^+$, $C_{10}H_{18}N_4O_4S$ calcd. 291.112, observed 291.119

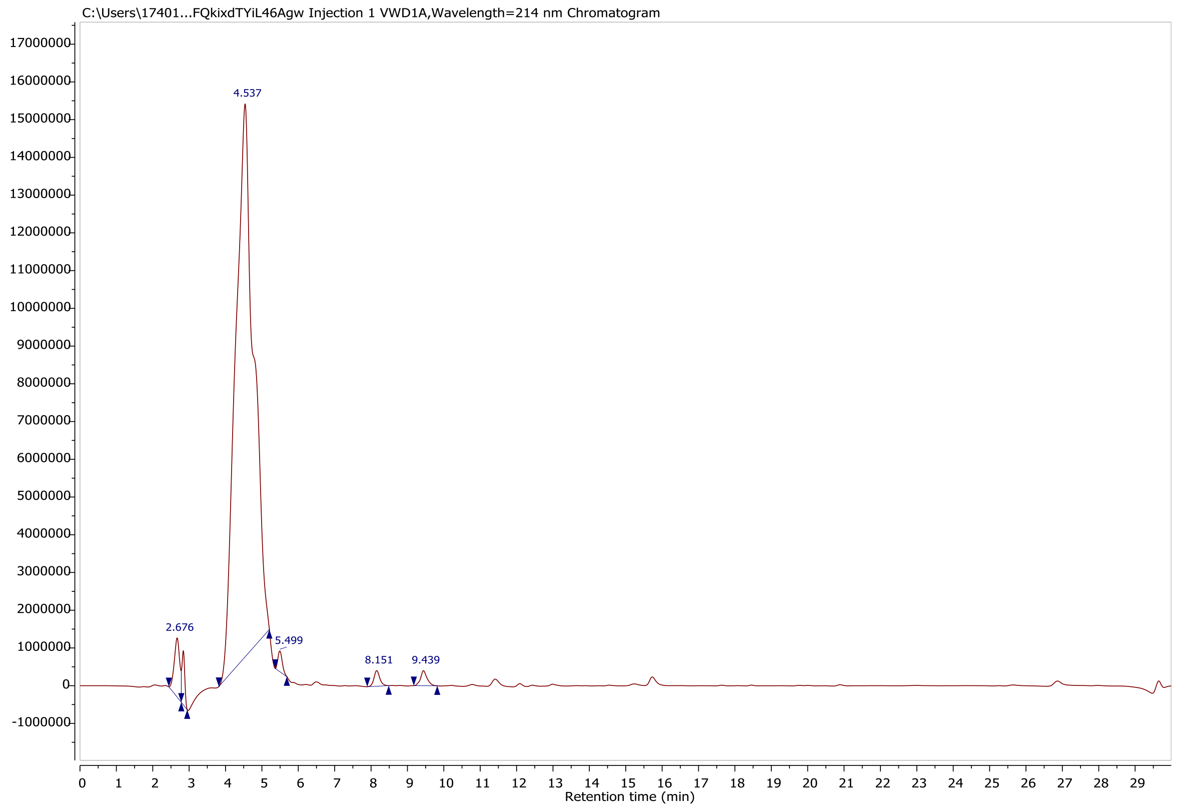

*Figure I1: Analytical HPLC trace of compound **21**. Mobile phase consisted of a linear gradient of water and acetonitrile (MeCN) each with 0.1% TFA. MeCN concentration followed a linear gradient from 5% to 80% over 15 mins. For the next 5 mins MeCN concentration was dropped to 5% and this ratio was held for 10 mins, providing a total run time of 30 mins. Detected at 214 nm.*

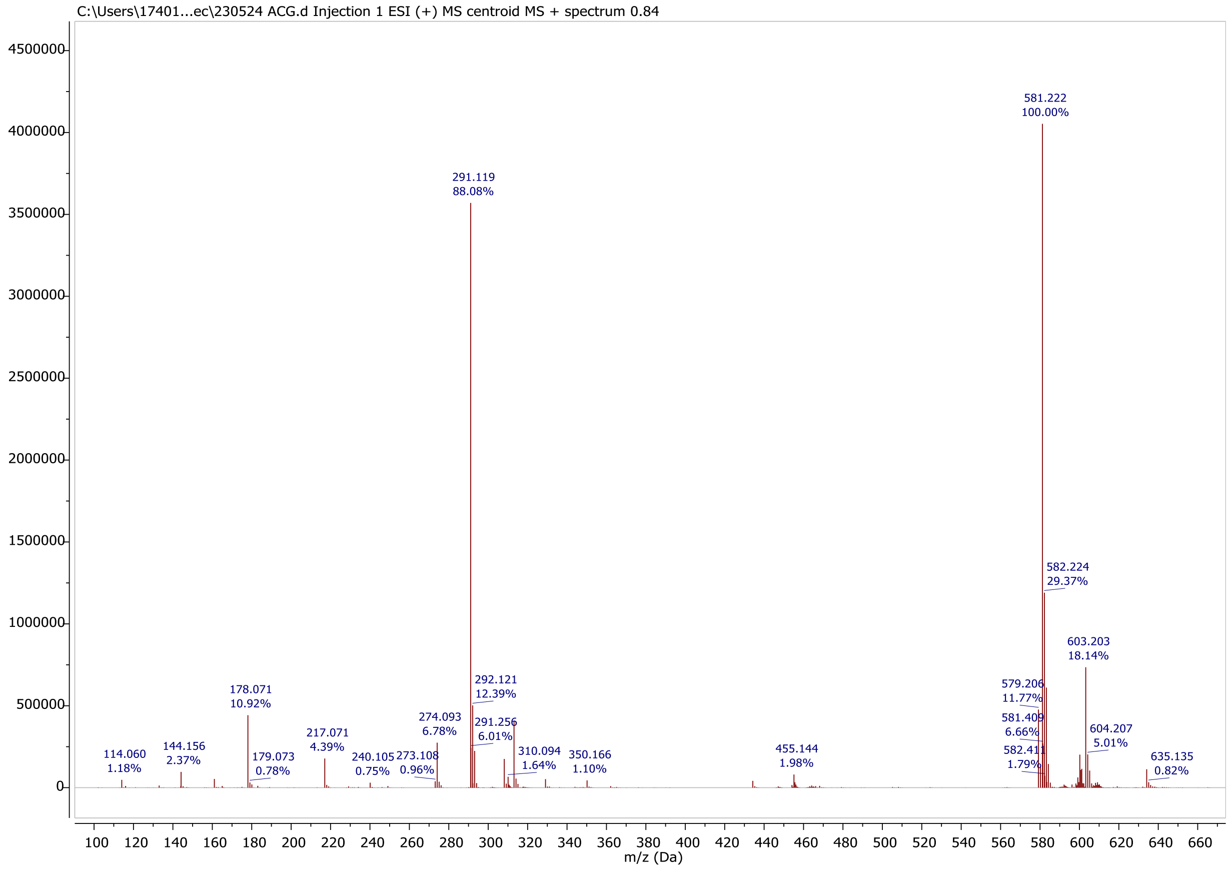

*Figure I2: HRMS (ESI) spectrum of compound **21** showing the m/z $(M+H)^+$ (calcd. 291.112, observed 291.119) and $(2M+H)^+$ (calcd. 581.217, observed. 581.222) adducts*

Alkylation of Ac-AlaCysGly-NH_2 (21) with 2-bromo-1-(2,4-dimethoxyphenyl)ethan-1-one (22a)

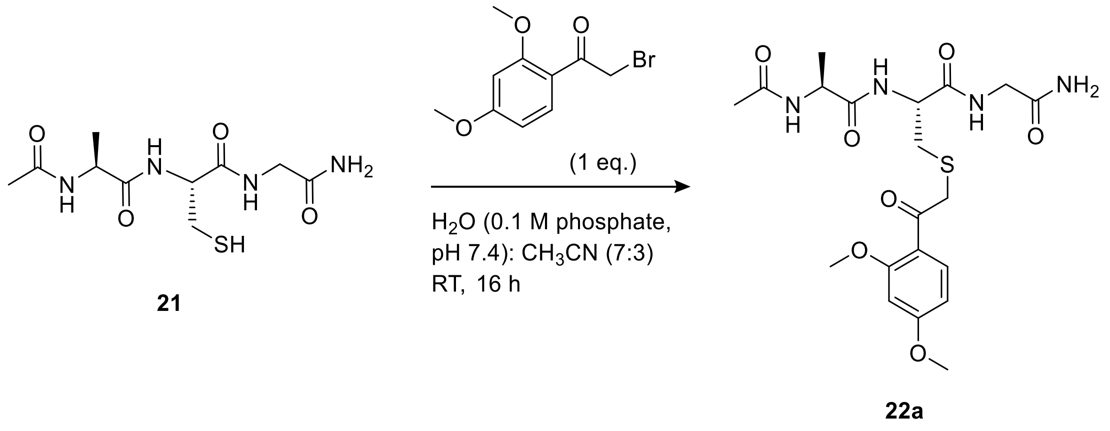

Ac-AlaCysGly-NH_2 (**21**) (10 mg, 34.5 µmol) was dissolved in 700 µL of aq. phosphate buffer (0.1 M, pH 7.4). A solution of 2-bromo-1-(2,4-dimethoxyphenyl)ethan-1-one (8.6 mg, 34.5 µmol) in 300 µL CH_3CN was added dropwise to the aqueous peptide solution. The reaction mixture was allowed to stir overnight and monitored by analytical HPLC. Following completion of the reaction the phenacylsulfide was purified by flash chromatography using C18 silica with H_2O/CH_3CN (7:3) as an eluent. The purified fraction was then frozen and lyophilized to give **22a** as a pale brown solid (9 mg, 56% yield).

HRMS (ESI) *m/z* (M+H)$^+$, $C_{20}H_{28}N_4O_7S$ calcd 469.176, observed 469.176

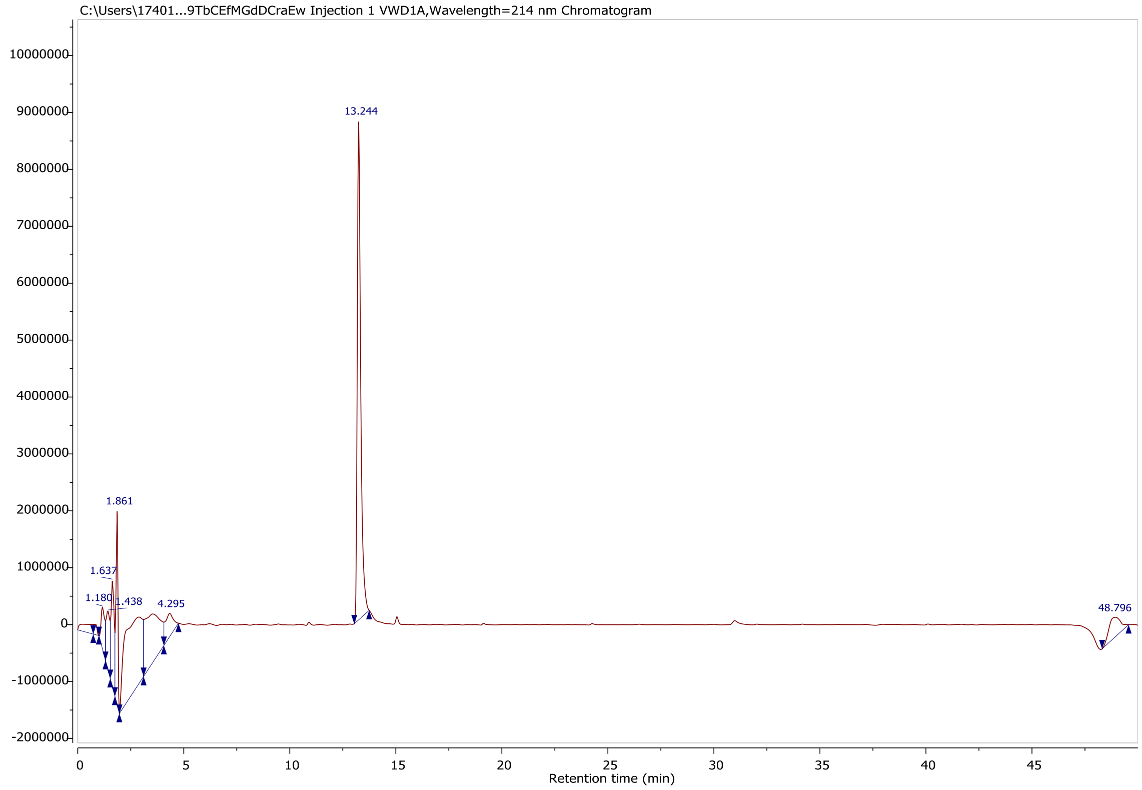

*Figure I3: Analytical HPLC trace of compound **22a**. Mobile phase consisted of a linear gradient of water and acetonitrile (MeCN) each with 0.1% TFA. MeCN concentration followed a linear gradient from 5% to 80% over 35 mins. Following this for the next 10 mins, MeCN concentration was increased to 95% and this was held for 5 mins. Finally, the solvent ratio was restored to 5% MeCN and held for 5 mins. Final run time was 50 mins.*

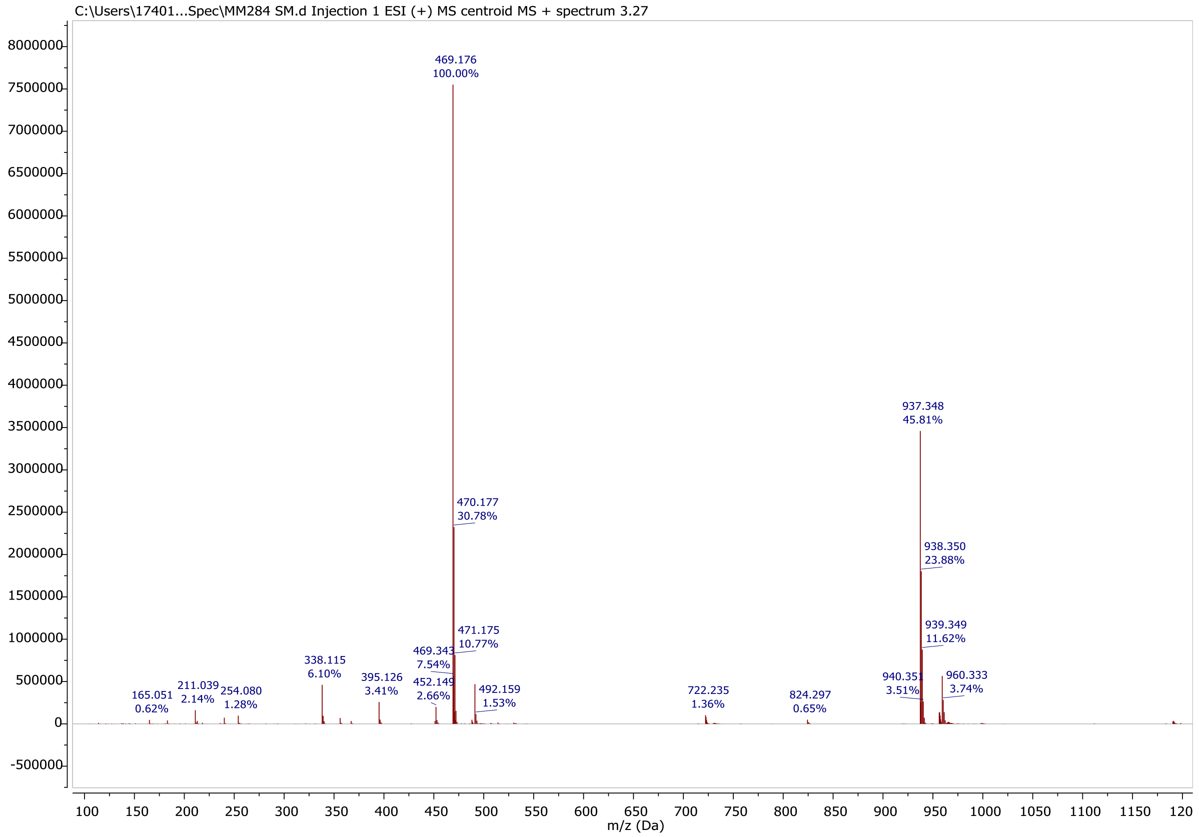

*Figure I4: HRMS (ESI) spectrum of compound **22a** showing the m/z $(M+H)^+$ (calcd. 469.176, observed 469.176) and $(2M+H)^+$ (calcd. 937.343, observed. 937.348) adducts*

Alkylation of Ac-AlaCysGly-NH2 (21) with 1-(3,5-bis(trifluoromethyl)phenyl)-2-bromoethan-1-one (22b).

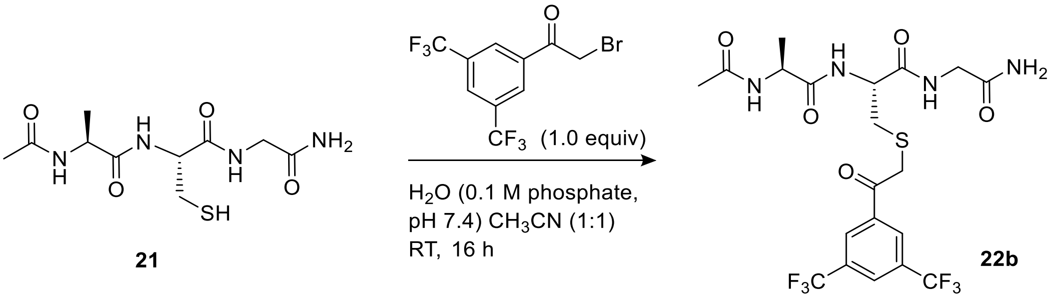

21

22b

Ac-AlaCysGly-NH2 (**21**) (20 mg, 68.9 µmol) was dissolved in 2.5 mL of aq. phosphate buffer (0.1 M, pH 7.4). 1-(3,5-bis (trifluoromethyl)phenyl)-2-bromoethan-1-one (23 mg, 68.9 µmol) in 2.5 mL MeCN was added to the stirred aqueous solution dropwise. The reaction mixture was then allowed to stir overnight. Following completion of the reaction, as determined by HPLC analysis, the product was purified by flash chromatography by using C18 silica with H_2O/ MeCN (7:3) as eluent. The fractions containing product were frozen and lyophilized to give **22b** as a pale green solid (24.4 mg, 65% yield), which was analysed by ^{1}H NMR spectroscopy, HRMS and HPLC.

^{1}H NMR (D_2O/MeCN-D_3, 7:3, 600 MHz): δ 8.46 (s, 2H), 8.23 (s, 1H), 4.42 (dd, J = 8.8, 5.3 Hz, 1H), 4.17 (q, J = 7.2 Hz, 1H), 3.81 (d, J = 17.2 Hz, 1H), 3.71 (d, J = 17.2 Hz, 1H), 2.97 (dd, J = 14.0, 5.3 Hz, 1H), 2.82 (dd, J = 14.0, 8.8 Hz, 1H), 1.90 (s, 3H), 1.24 ppm (d, J = 7.2 Hz, 3H). *Note: the methylene between the carbonyl group and the sulfur atom rapidly incorporated deuterium from solvent.*

HRMS (ESI) *m/z* (*M*+H)$^+$, $C_{20}H_{22}F_6N_4O_5S$ calcd. 545.129, observed 545.130

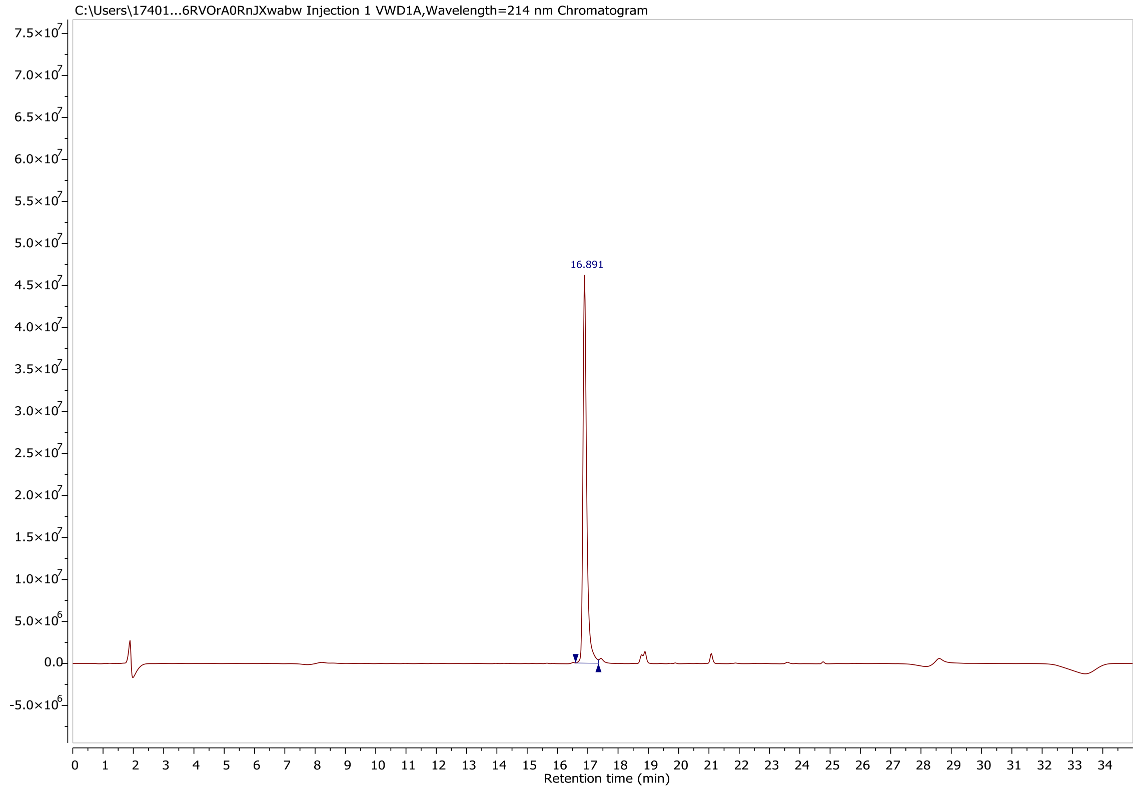

*Figure I5: Analytical HPLC trace of **22b**. Mobile phase consisted of a linear gradient of H_2O and acetonitrile (MeCN) each with 0.1% TFA. MeCN concentration followed a linear gradient from 5% to 95% over 25 mins. For the next 5 mins MeCN concentration was dropped to 5%. This ratio was held for 5 mins, providing a total run time of 35 mins. Detected at 214 nm.*

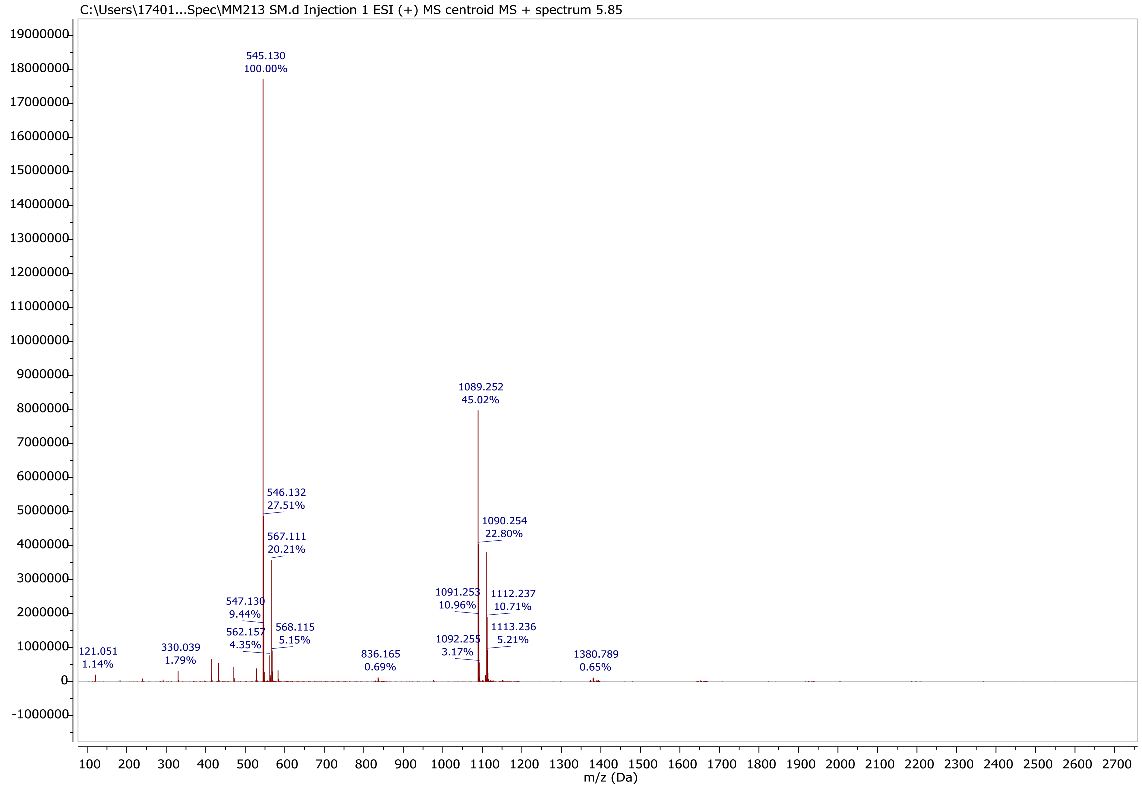

*Figure I6: HRMS (ESI) spectrum of compound **22b** showing the m/z $(M+H)^+$ (calcd. 545.128, observed 545.130) and $(2M+H)^+$ (calcd. 1089.250, observed. 1089.252) adducts.*

Alkylation of Ac-AlaCysGly-NH_2 (21) with 4-(2-bromoacetyl)-*N,N,N*-trimethylbenzenaminium (22c)

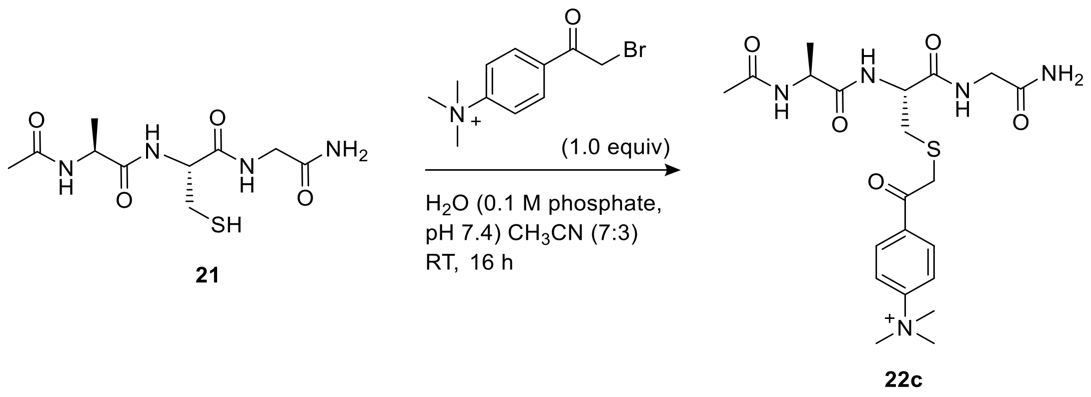

Ac-AlaCysGly-NH_2 **(21)** (4.3 mg, 14.9 µmol) was dissolved in 500 µL of an aq. phosphate buffer (0.1 M, pH 7.4)/MeCN, (7:3) solution. A solution of 4-(2-bromoacetyl)-*N,N,N*-trimethylbezenaminium (6.05 mg, 14.9 µmol) in 500 µL of the same solvent mixture was added. The reaction mixture was then allowed to stir overnight. The product, **22c** (6.95 mg, >95% yield by HPLC analysis) was used without any further purification.

HRMS (ESI) *m/z* $(M)^+$, $C_{21}H_{32}N_5O_5S$ calcd 466.212, observed 466.212

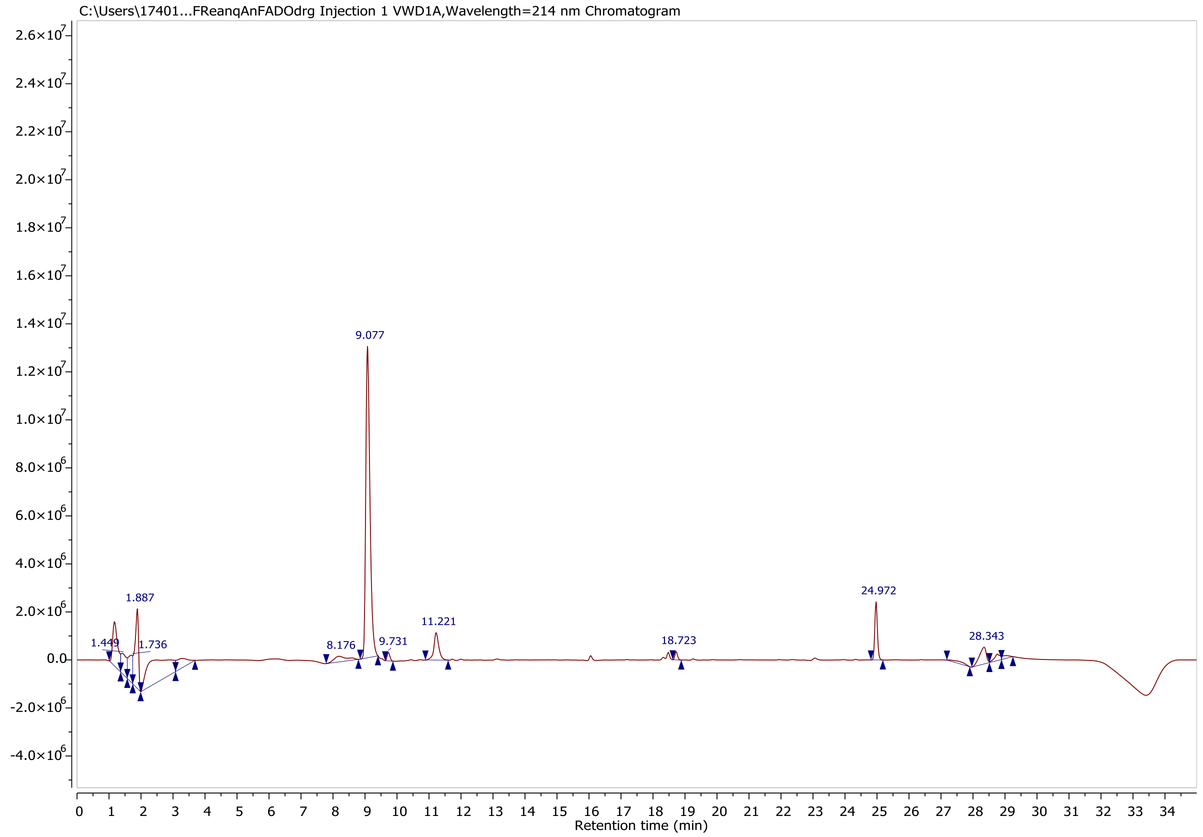

*Figure I7: Analytical HPLC trace of **22c**. Mobile phase consisted of a linear gradient of H_2O and acetonitrile (MeCN) each with 0.1% TFA. MeCN concentration followed a linear gradient from 5% to 95% over 25 mins following this for the next 5 mins MeCN dropped to 5% and this ratio was held for 5 mins, providing a total run time of 35 mins. Detected at 214 nm.*

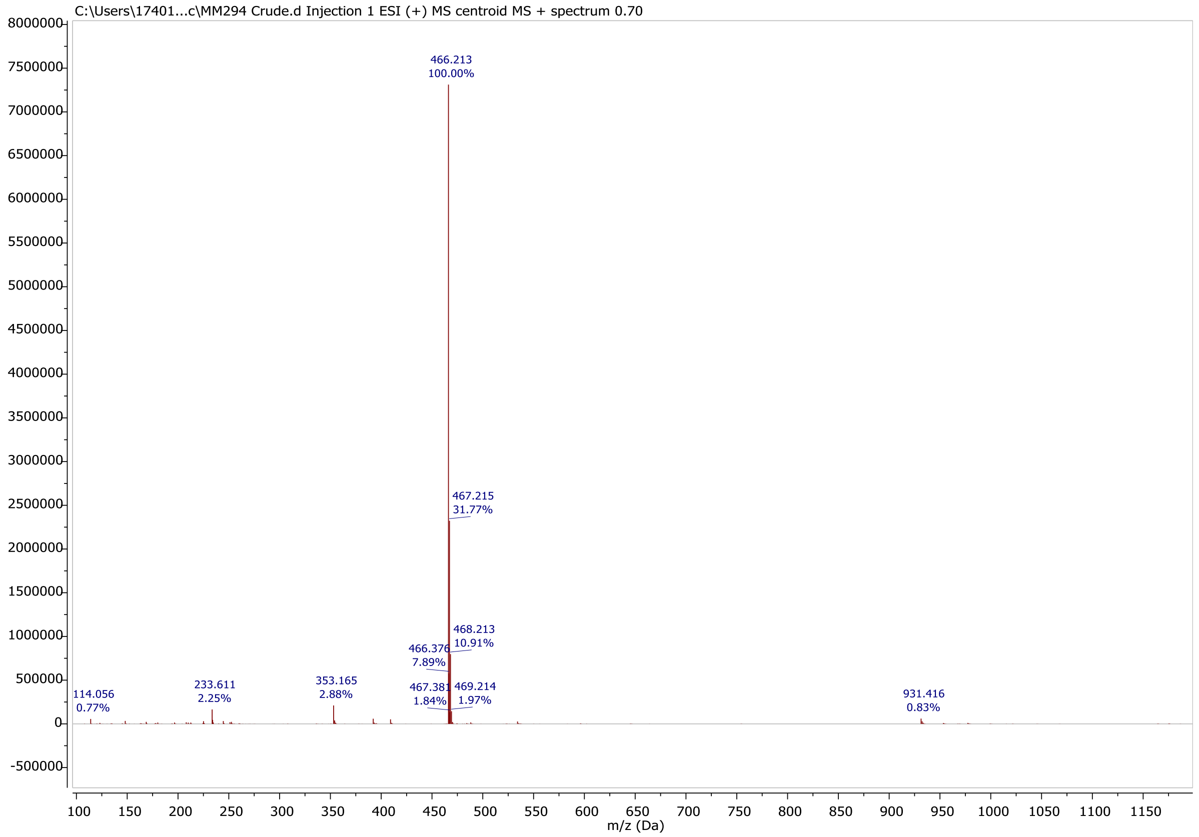

*Figure I8: HRMS (ESI) spectrum of compound **22c** showing the m/z (M)$^{+}$ (calcd. 466.212, observed 466.213) adduct.*

Photolysis of tripeptide based phenacylsulfides

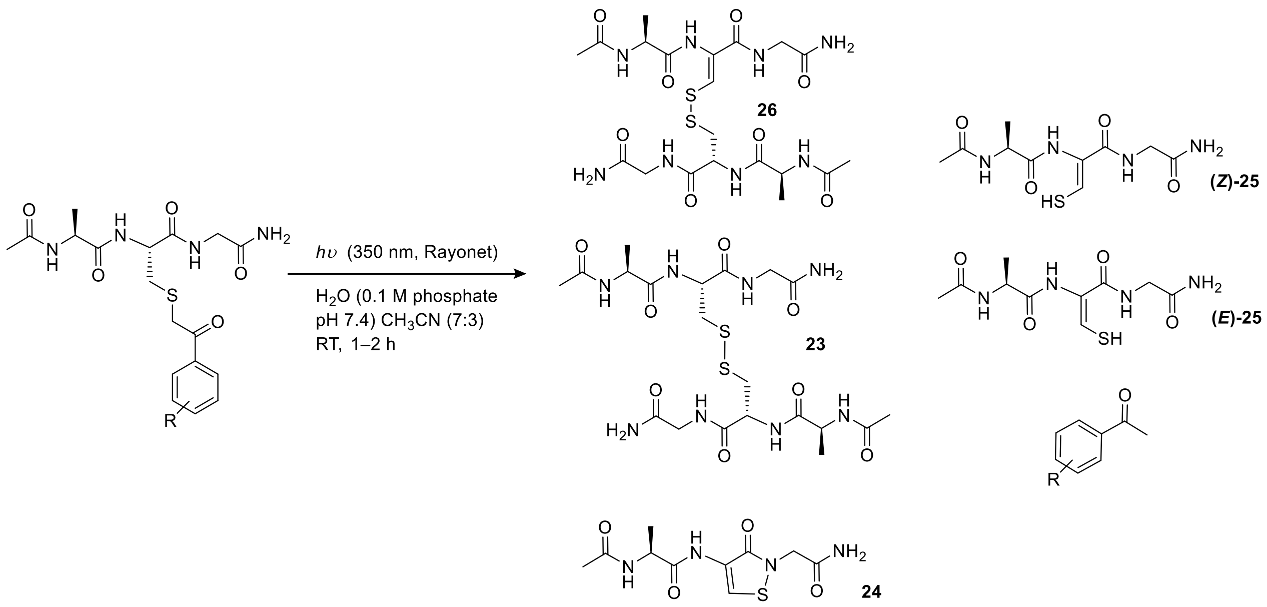

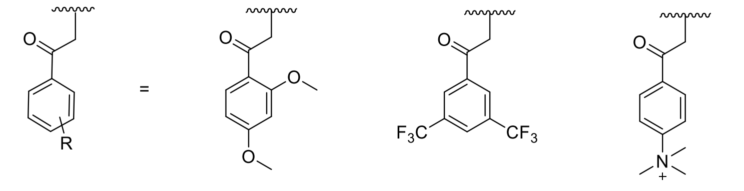

22a **22b** **22c**

Tripeptide phenacylsulfide (14.9 µmol) was dissolved in a solution of H_2O (0.1 M, phosphate pH 7.4)/MeCN (7:3, 1 mL). This solution was then sparged with N_2 for 15 minutes and transferred to an NMR tube flushed with N_2 gas prior to sealing. The sealed NMR tube was then photolysed under UV light at 350 nm in a Rayonet photoreactor for 90 minutes. Completion of the reaction was confirmed by HPLC and HRMS analysis. Once the reaction was complete, the sample was frozen and lyophilized. The lyophilized reaction mixture was then dissolved in 500 µL of D_2O and characterised by NMR spectroscopy.

Note: In the case of compound ***22c****, photolysis only progressed to 94% completion*

Product distributions obtained from the photolysis of tripeptide phenacylsulfide derivatives

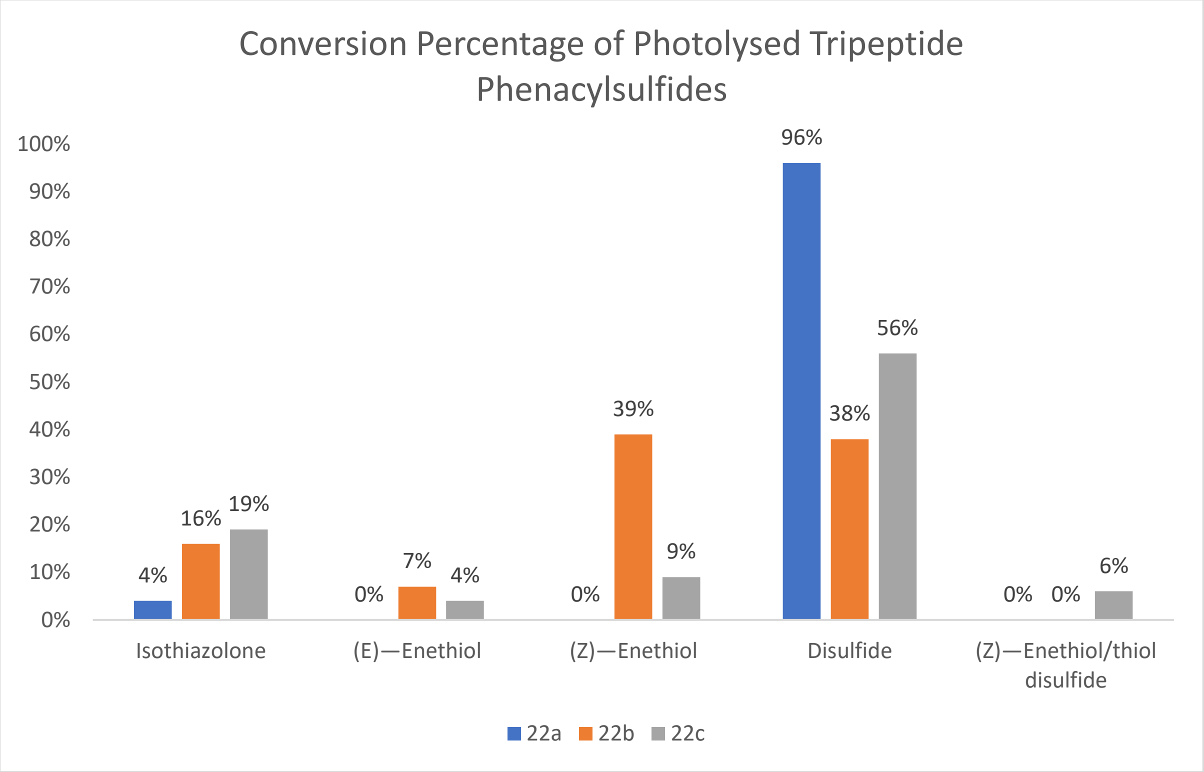

Figure I9: Conversion percentages for products in mixture derived from the photolysis of tripeptide phenacylsulfide derivatives.

Photolysis of tripeptide phenacylsulfide 22b in the presence of 3 M guanidinium

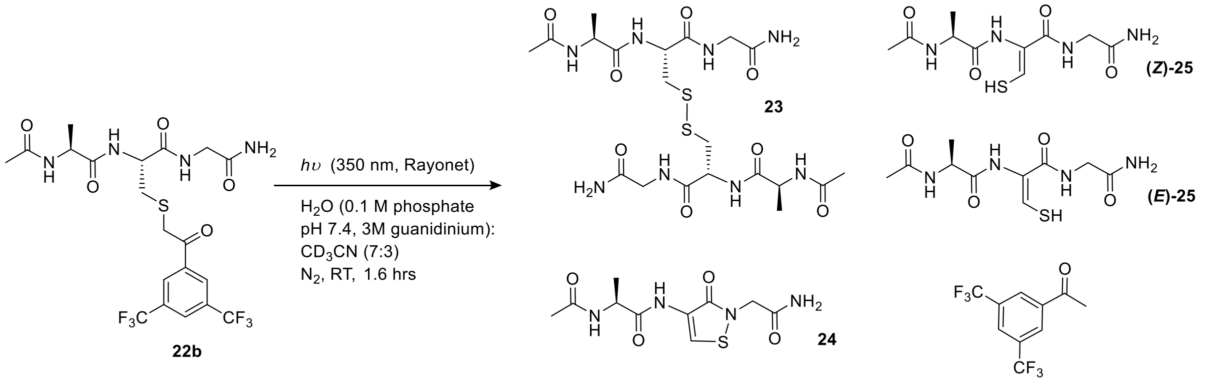

Tripeptide phenacylsulfide **22b** (7.45 µmol) was dissolved in a solution of H_2O (0.1 M, phosphate pH 7.4, 3M guanidinium)/CD_3CN (7:3, 0.5 mL). This solution was then sparged with N_2 for 15 minutes and transferred to an NMR tube flushed with N_2 gas prior to sealing. The sealed NMR tube was then photolysed under UV light at 350 nm in a Rayonet photoreactor for 100 minutes. Once the reaction was complete, the sample was characterised by presaturation ^{1}H NMR (400 MHz) spectroscopy.

*Table I2: Conversion percentage of each product observed in the photolysis mixture derived from the photolysis of phenacylsulfide **22b** in a solution of H_2O (0.1 M, phosphate pH 7.4, 3M guanidinium)/CD_3CN (7:3, 0.5 mL).*

	Disulfide (**23**)	(*Z*)-enethiol ((***Z***)-**25**)	(*E*)-enethiol ((***E***)-**25**)	Isothiazolone (**24**)	SM (**22b**)
Conversion (%)	32	37	9	16	6

Characterisation of Ac-AlaCysGly-NH_2 photolysis products from crude reaction mixtures

Characterisation of Ac-AlaCysGly-NH_2 disulfide (23)

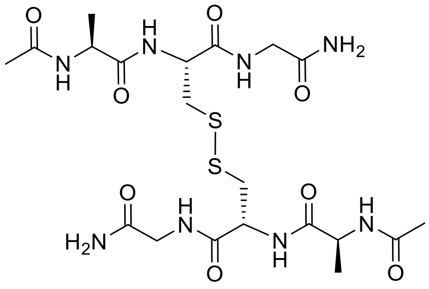

Characterised from photolysis mixture derived from compound 22a

^{1}H NMR (600 MHz, D_2O): δ 4.70 (dd, J = 9.1, 5.1 Hz, 2H), 4.30 (q, J = 7.2 Hz, 2H), 3.96 (d, J = 17.2 Hz, 2H), 3.89 (d, J = 17.2 Hz, 2H), 3.29 (dd, J = 14.3, 5.1 Hz, 2H), 3.02 (dd, J = 14.3, 9.1 Hz, 2H), 2.02 (s, 6H), 1.38 (d, J = 7.2 Hz, 6H) ppm.

^{13}C NMR (151 MHz, D_2O): δ 175.7, 174.2, 173.8, 172.5, 52.6, 49.8, 42.1, 37.9, 21.5, 16.3 ppm.

HRMS (ESI) *m/z* (M+H)$^+$, $C_{20}H_{34}N_8O_8S_2$ calcd. 579.201, observed 579.203

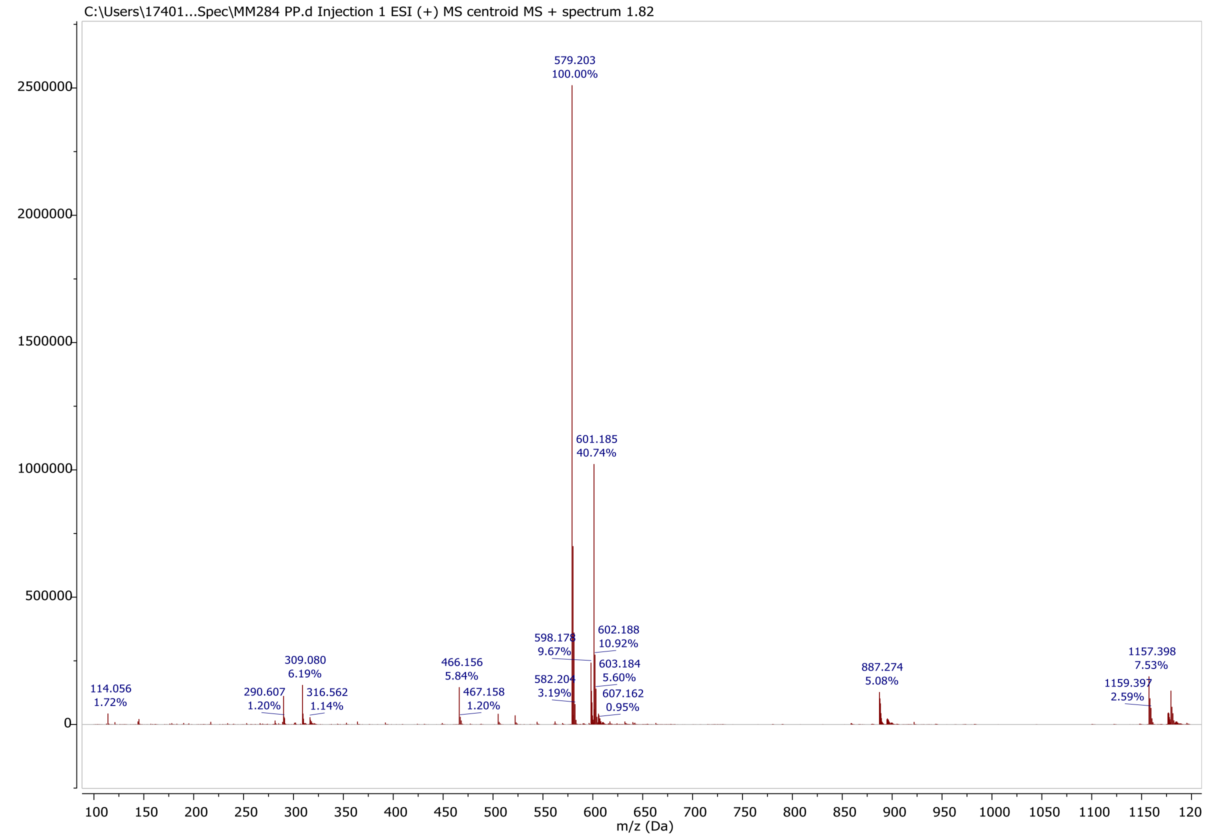

*Figure I10: HRMS (ESI) spectrum of compound **23** showing the m/z (M+H)$^+$ (calcd. 579.201, observed 579.203) adduct.*

Characterisation of Ac-AlaCysGly-NH2 isothiazolone (24)

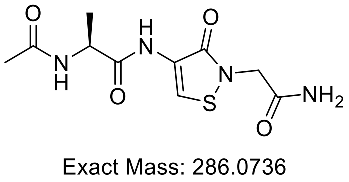

Characterised from photolysis mixture derived from compound 22c

^{1}H NMR (600 MHz, D_2O): δ 8.64 (s, 1H), 4.59 (s, 2H), 4.45 (q, J = 7.3 Hz, 1H), 2.04 (s, 3H), 1.43 (d, J = 7.3 Hz, 3H) ppm.

^{13}C NMR (151 MHz, D_2O): δ 174.2, 173.8, 171.3, 164.2, 127.2, 49.8, 46.1, 21.5, 16.4 ppm. (α carbon of isothiazolone not observed)

HRMS (ESI) *m/z* (M+H)$^+$, $C_{10}H_{14}N_4O_4S$ calcd 287.081, observed 287.082

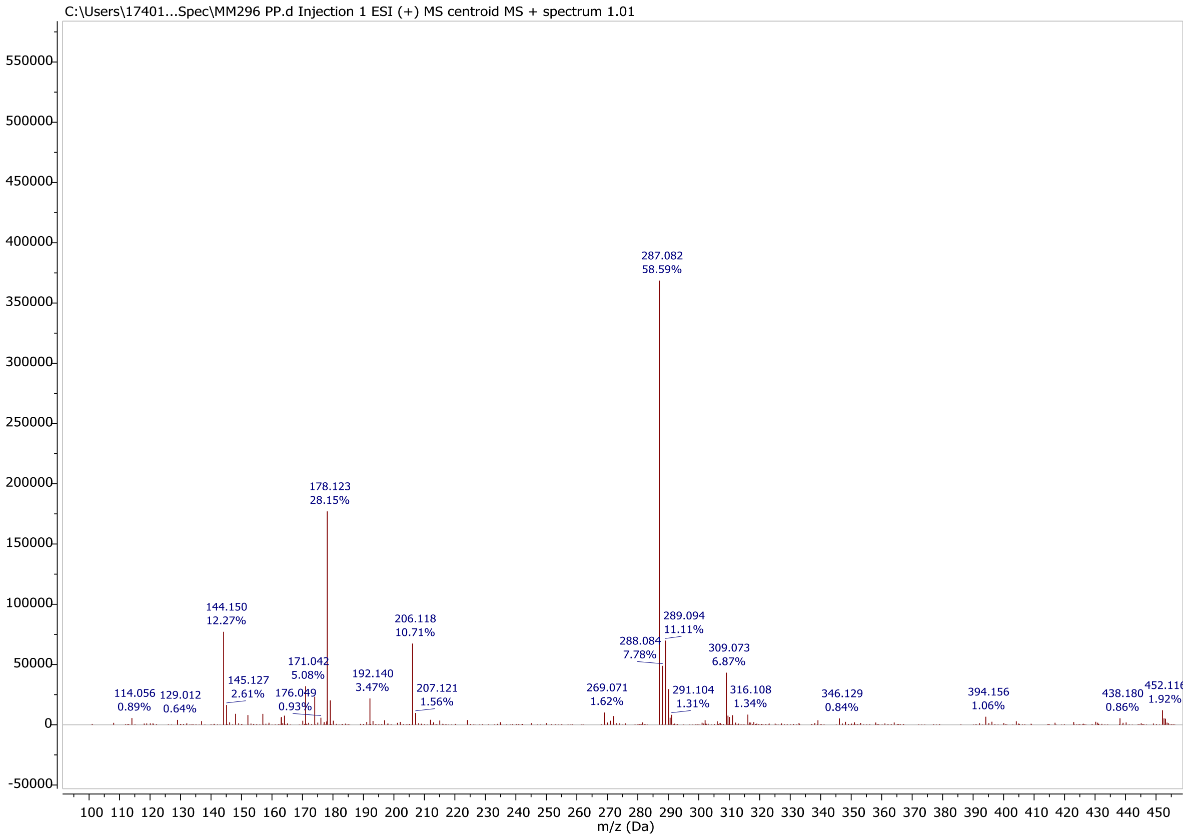

*Figure I11: HRMS (ESI) spectrum of compound **24** showing the m/z (M+H)$^+$ (calcd. 287.081, observed 287.082) adduct.*

Characterisation of Ac-AlaCysGly-NH2 (Z)-enethiol (25)

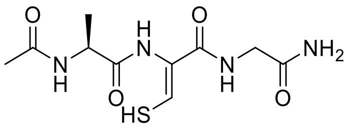

Characterised from photolysis mixture derived from compound 22b

^{1}H NMR (600 MHz, D_2O): δ 8.21 (s, 1H), 4.39 (q, J = 7.2 Hz, 1H), 3.93 (d, J = 17.3 Hz, 1H), 3.87 (d, J = 17.3 Hz, 1H), 2.05 (s, 3H), 1.50 (d, J = 7.2 Hz, 3H) ppm.

^{13}C NMR (151 MHz, D_2O): δ 175.2, 175.1, 174.2, 166.8, 153.7, 123.5, 50.3, 42.1, 21.5, 16.3 ppm.

HRMS (ESI) m/z $(M+H)^+$, $C_{10}H_{16}N_4O_4S$ calcd 289.096, observed 289.097

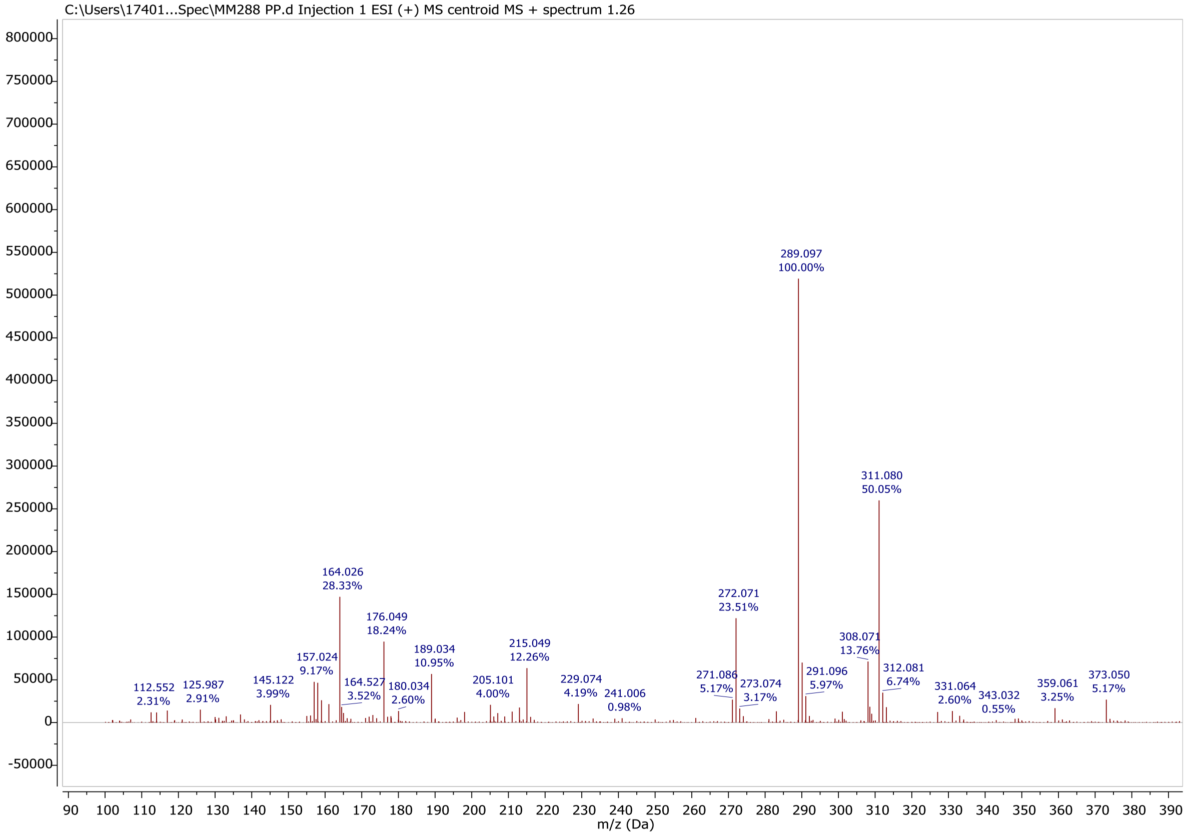

*Figure I12: HRMS (ESI) spectrum of compound **25** showing the m/z $(M+H)^+$ (calcd. 289.096, observed 289.097) adduct.*

J. Investigation of tripeptide isothiazolone and enethiol derivatives

pK_a studies of tripeptide-based (*E*)-enethiol and (*Z*)-enethiol (25)

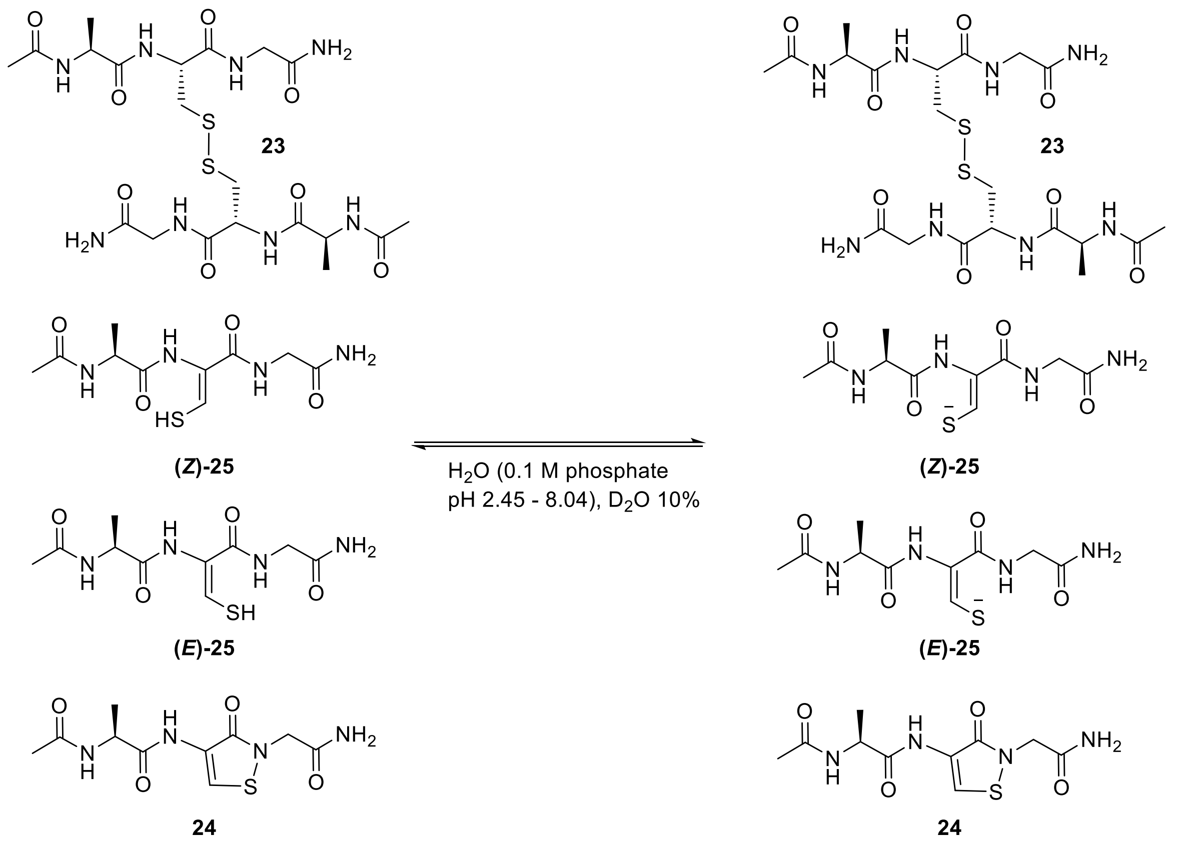

Compound **22b** (4.3 mg, 7.9 µmol) was dissolved in a solution of H_2O (0.1 M, phosphate pH 7.4)/MeCN, 7:3, 2 mL). The reaction mixture was then sparged with N_2 for 15 minutes. The mixture was then transferred to an NMR tube, which was flushed with N_2 and sealed. The mixture was illuminated at 350 nm in a Rayonet photoreactor for 100 minutes. The resulting reaction mixture was then frozen and lyophilized. Following lyophilisation, the residue was resuspended in 1.26 mL of HPLC-grade water with 0.14 mL D_2O. The solution was then divided into two, the first sample adjusted to pH 8.04 with aq. NaOH (2 M) and the second adjusted to pH 2.45 with aq. HCl (2 M). Both samples were then analysed by presaturation ^{1}H NMR spectroscopy. The basic solution was added in varying portions to the acidic solution so that the pH change was between 0.1 and 0.5, with smaller changes being preferable close to the pK_a value. After each addition, the sample was analysed by presaturation ^{1}H NMR spectroscopy. Using the isothiazolone methine signal (green) at 8.65 ppm as a reference, the change in the position of the signal corresponding to the methine protons attached to the β-carbon of the enethiol/enethiolate relative to pH value was noted. The resulting changes the chemical shift (Δppm) were plotted against sample pH to produce a logarithmic curve, with the inflection indicating the pK_a value.

*Table I1: Change in Δppm (between β methine protons of isothiazolone (**24**) and (Z)-enethiol/enethiolate (**25**) with pH*

**pH**	**Δppm (β methine protons of isothiazolone (24) and (Z)-enethiol/enethiolate (25))**
8.04	0.45
7.4	0.45
5.92	0.47
5.65	0.48
5.55	0.49
5.44	0.49
5.31	0.51
5.01	0.55
4.84	0.57
4.40	0.68
3.71	0.87
3.35	0.93
2.45	0.97

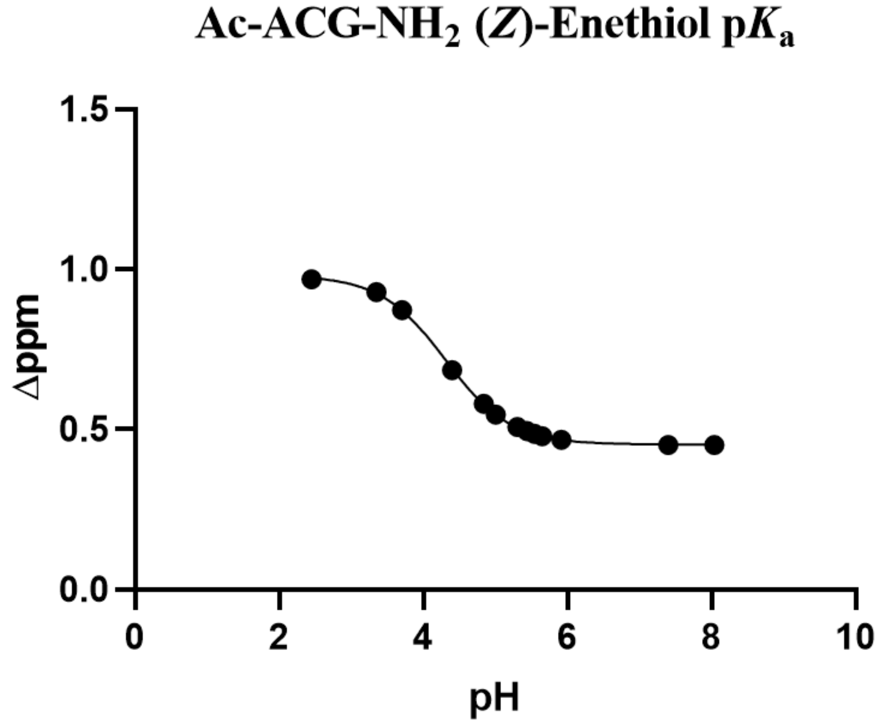

Figure I13: Plot of Δppm against pH for (Z)-enethiol/enethiolate ***(25)****.*

R^2 = 0.9998
Calculated pK_a = *4.3*

*Table I2: Change in Δppm (between β methine protons of isothiazolone **(24)** and (E)-enethiol/enethiolate **(25)** with pH*

pH	Δppm (β methine protons of isothiazolone (24) and (*E*)-enethiol/enethiolate (25))
8.04	0.69
7.4	0.69
5.92	0.71
5.65	0.72
5.55	0.73
5.44	0.74
5.31	0.76
5.01	0.81
4.84	0.87
4.40	1.01
3.71	1.08
3.35	1.08
2.45	1.09

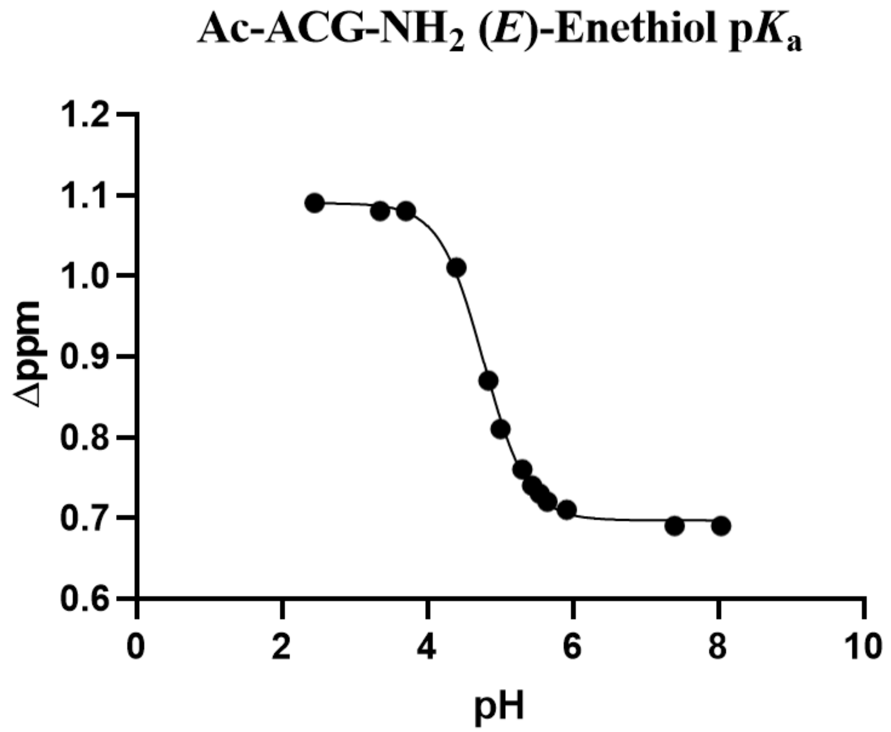

*Figure I14: Plot of Δppm against pH for (E)-enethiol/enethiolate **(25)**.*

R^2 = 0.9986
Calculated pK_a = 4.8

pK_a studies of Ac-AlaCysGly-NH_2 (21)

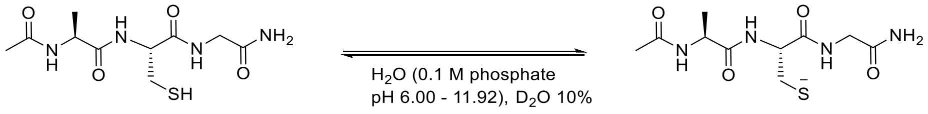

21 **21**

Ac-AlaCysGly-NH_2 **(21)** (2.35 mg, 8.1 µmol) was dissolved in 700 µL of phosphate buffer (0.1 M, pH 6, 10% D_2O) and a second solution of peptide (2.35 mg, 8.1 µmol) in 700 µL of aq. phosphate buffer (0.1 M, pH 11.92, 10% D_2O) was also prepared. Both samples were then analysed via presaturation ^1H NMR. Both solutions were then titrated against each other, the pH of the sample was then determined and presaturation ^1H NMR was taken after each change in pH. Using an amine signal at 7.75 ppm as a reference, the change in the position of the signal corresponding to the diastereotopic protons of the β carbon of the cysteine residue were determined. The resulting change the chemical shift (Δppm) was plotted against sample pH to produce a logarithmic pH curve allowing for pK_a determination.

Table I3: Change in Δppm (between β methylene protons of cysteine and amine signal at 7.75 ppm) with pH

**pH**	**Δppm (β methylene protons of cysteine and amine signal at 7.75 ppm)**
11.92	5.05
11.5	5.05
10.89	5.05
10.19	5.05
9.64	5.04
9.12	5.03
8.69	5.01
6.00	4.96

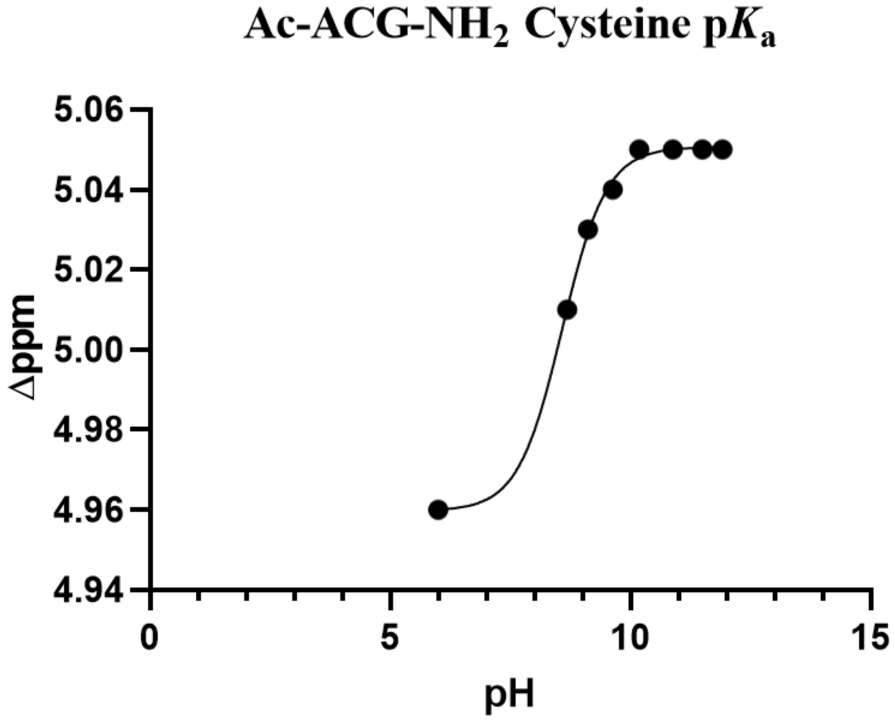

Figure I15: Plot of Δppm against pH for unmodified Ac-AlaCysGly-NH$_2$ ***(21)***

R^2 = 0.9983
Calculated pK_a = 8.6

Studies into the interconversion of Ac-AlaCysGly-NH_2 enethiol and isothiazolone derivatives

Treatment of photolysis mixture derived from phenacylsulfide 22b with tris(2-carboxyethyl)phosphine (TCEP)

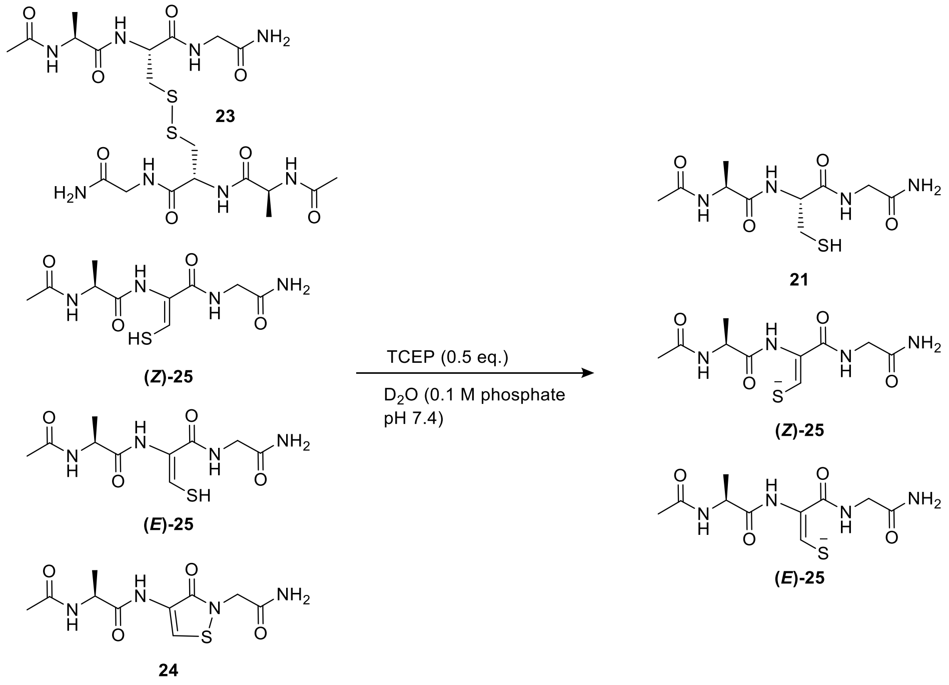

Compound **22b** (2.15 mg, 3.95 µmol) was dissolved in a solution of H_2O (0.1 M, phosphate pH 7.4/MeCN, 7:3, 1 mL). The reaction mixture was then sparged with N_2 for 15 minutes and transferred to an NMR tube, which was flushed with N_2 before sealing. The mixture was illuminated at 350 nm in a Rayonet photoreactor for 100 minutes. The resulting reaction mixture was then lyophilized and the resulting residue dissolved in D_2O (0.7 mL). The resulting reaction mixture was then analysed by ^1H NMR spectroscopy. TCEP (0.1 M in H_2O, pH 7.2, 1.975 µmol) was then added to the reaction mixture. The sample was reanalysed by ^1H NMR spectroscopy and changes in the relative level of each species was noted; see Table 8 below for data.

*Table I4: Distribution of cysteine derivatives before and after TCEP treatment. Values provided are conversion % from original phenacylsulfide (**22b**)*

Species	Relative conversion before TCEP treatment	Relative conversion after TCEP treatment
(*Z*)-enethiol(ate)	39%	41%
Isothiazolone	16%	0%
(*E*)-enethiol(ate)	7%	21%
Disulfide	38%	0%
Parent peptide thiol	0%	38%

Treatment of photolysis mixture derived from phenacylsulfide 3b with 5,5-dithiobis-(2-nitrobenzoic acid) (DTNB)

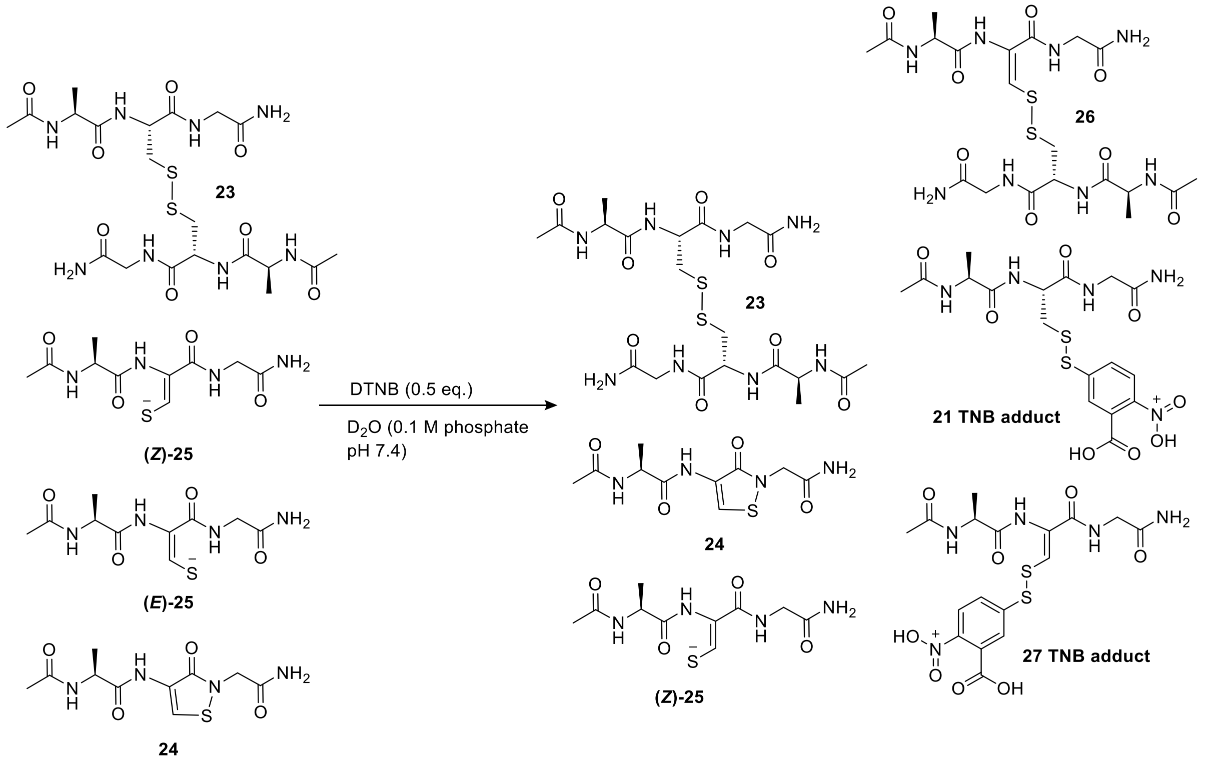

Compound **22b** (2.15 mg, 3.95 µmol) was dissolved in a solution of H_2O (0.1 M phosphate pH 7.4/MeCN, 7:3, 1 mL). The reaction mixture was then sparged with N_2 for 15 minutes and transferred to an NMR tube, which was flushed with N_2 before sealing. The mixture was illuminated at 350 nm in a Rayonet photoreactor for 100 minutes. The resulting reaction mixture was then lyophilized. Following lyophilisation the residue was dissolved in D_2O (0.7 mL) and analysed by ^1H NMR spectroscopy. DTNB (Ellman's reagent) was added to the reaction (1.975 µmol, 0.1 M, H_2O). The mixture was reanalysed by ^1H NMR spectroscopy and changes in relative level of each species was noted; see Table 9 below for data.

*Table I5: Distribution of cysteine derivatives before and after DTNB treatment. Values provided are conversion % from original phenacylsulfide (**22b**)*

Species	Relative conversion before DTNB treatment	Relative conversion after DTNB treatment
(*Z*)-enethiol(ate)	39%	35%
Isothiazolone	16%	21%
(*E*)-enethiol(ate)	7%	0%
Disulfide	38%	7%
Thiol—TNB disulfide	0%	29%
(*Z*)-enethiol—TNB disulfide	0%	1%
(*E*)-enethiol—TNB disulfide	0%	0%
(*Z*)-enethiol—thiol disulfide	0%	7%

Photolysis of compound 22b in the absence of phosphate buffer

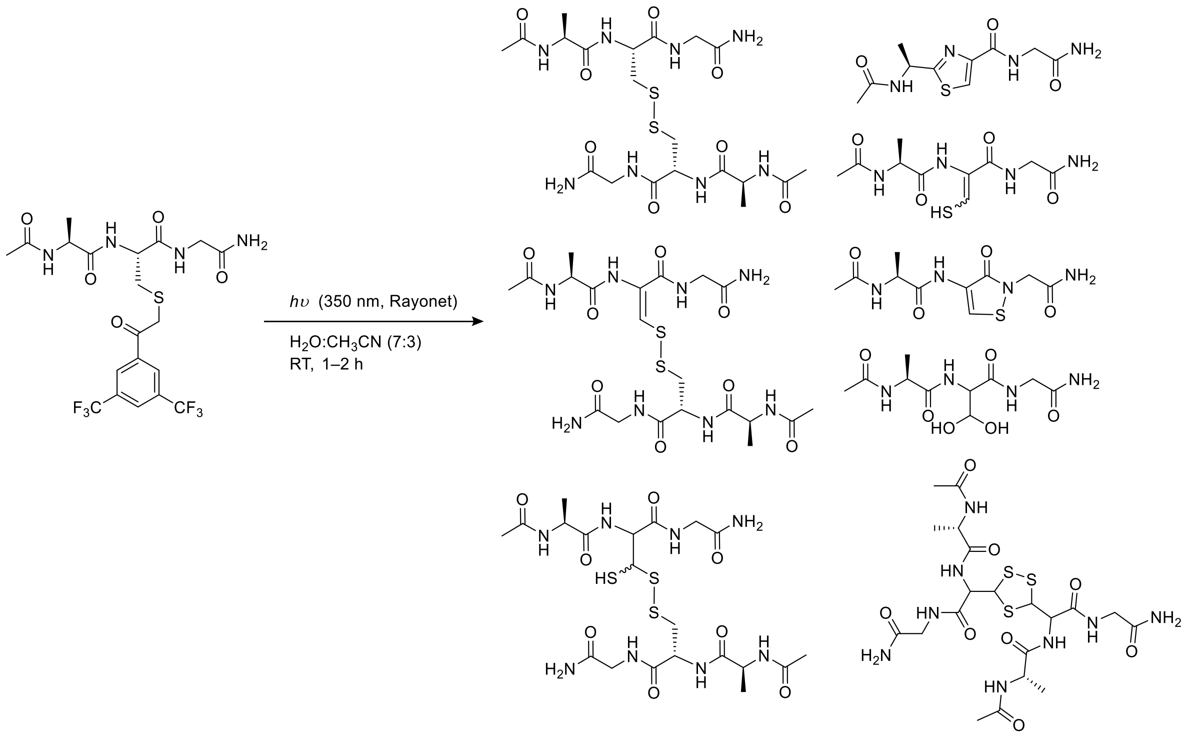

Compound **22b** (8.11 mg, 14.9 µmol) was dissolved in a solvent mixture of H_2O/MeCN-D_3 (7:3, 1 mL). The pH of this solution was then adjusted to pH 7.0 with a stock solution of NaOH (0.5 M). The sample was then sparged with N_2 for 15 minutes, and transferred to a clean NMR tube and back flushed with N_2 prior to sealing. The resulting solution was then subjected to photolysis at 350 nm for 100 minutes. The pH of the reaction mixture was then measured once more (drop to pH 5.6). The resulting reaction mixture was then filtered and fully characterised via presat NMR in and LCMS. Sample was also analysed via direct injection HRMS in order to observe potentially acid labile species.

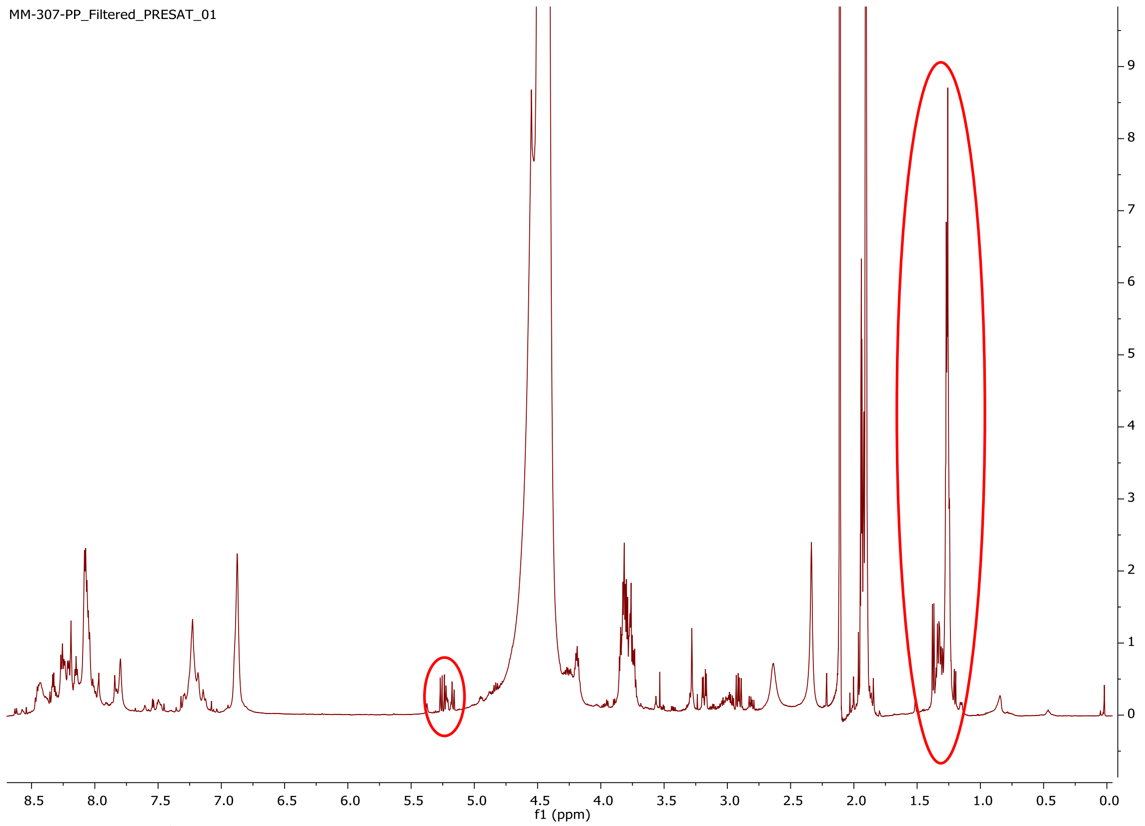

Figure I16: ^{1}H Presat NMR spectrum of compound ***22b*** *following photolysis in the absence of phosphate buffer, highlighting the complexity of the spectrum relative to the buffered reaction mixture in addition to key regions of interest in the sample: the methyl region of the alanine residue and the downfield methine region in the range 5.15–5.30 ppm.*

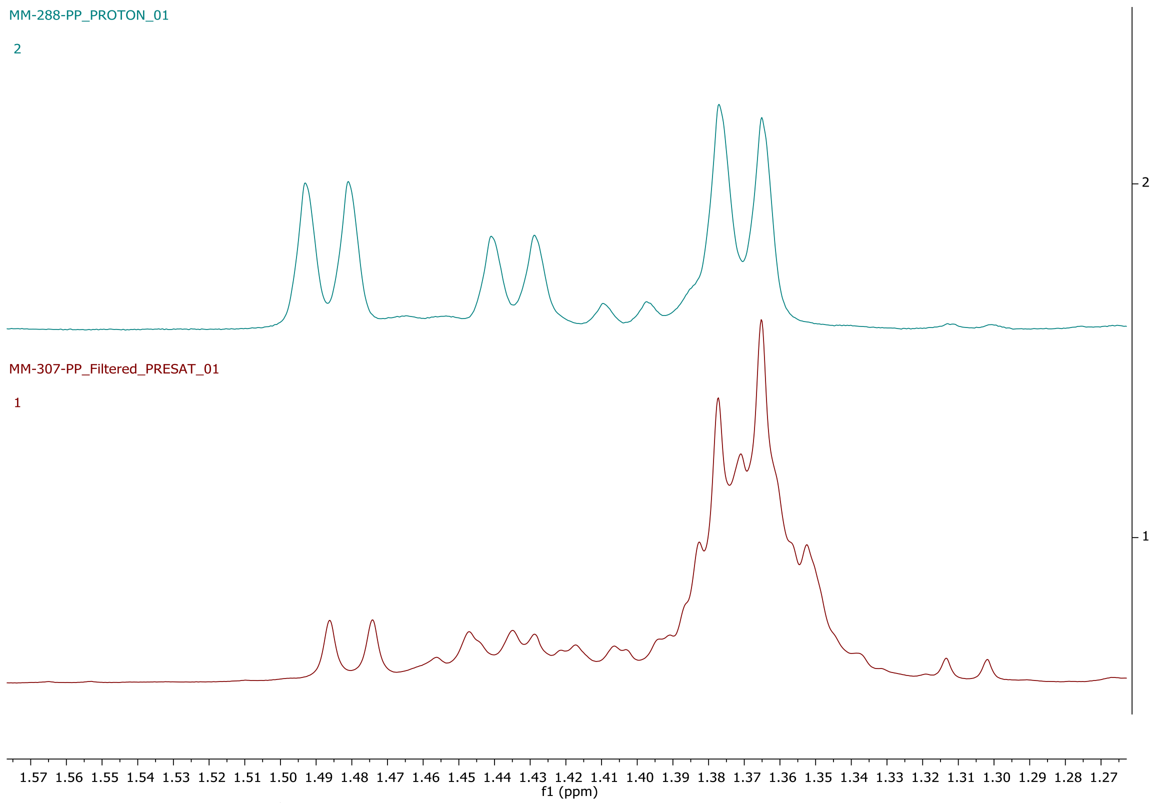

Figure I17: Stacked ^{1}H NMR spectra of the mixture derived from the photolysis of compound ***22b*** *in the presence of phosphate buffer (blue) and the absence of phosphate buffer (red). Focusing on the alanine region, highlights the complexity of the reaction mixture in the absence of buffer. Four products are clearly formed in the buffered reaction; in the absence of buffer, multiple additional new products are observed.*

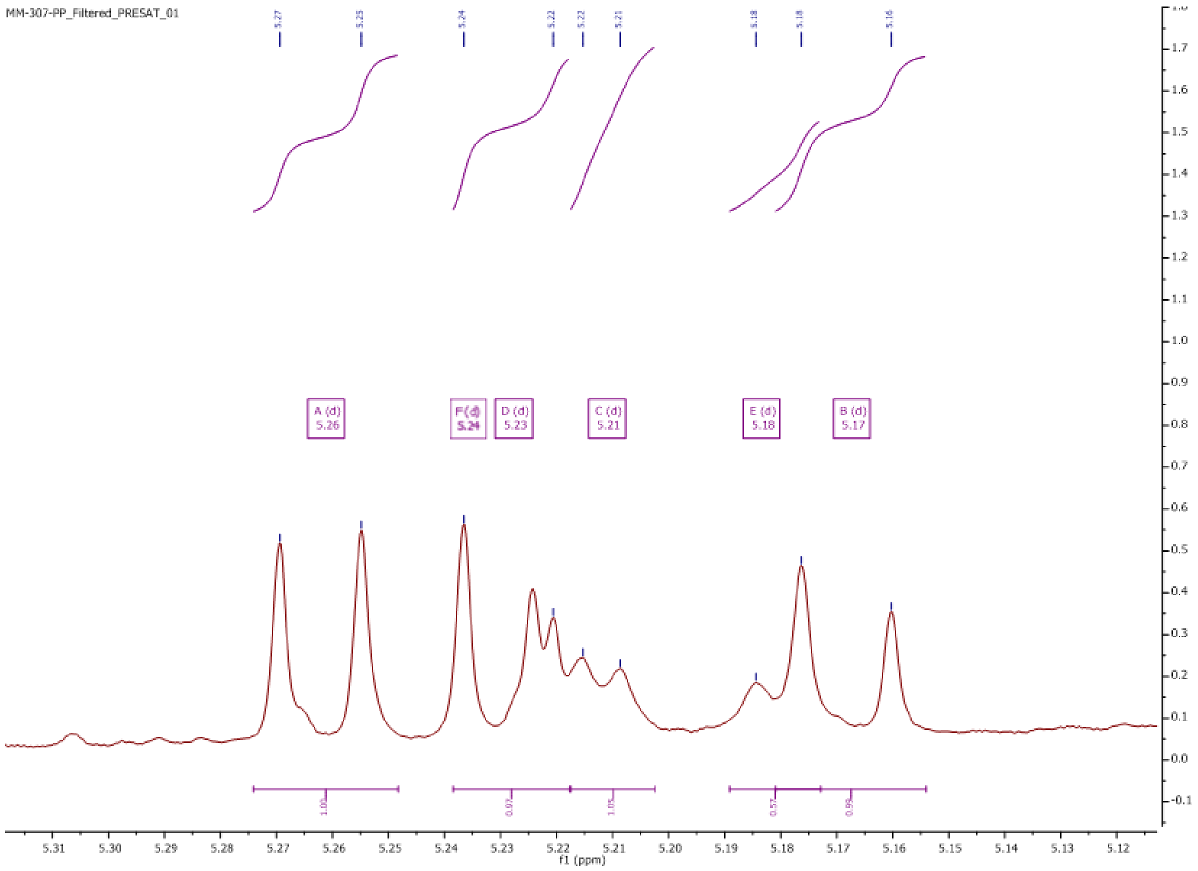

Figure I18: Methine region of the ^{1}H presat NMR spectrum of the mixture derived from the photolysis of compound ***22b*** *in the absence of phosphate buffer. Key doublets are highlighted. Doublets A and B are assigned to the β proton of a trithiolane moiety showing J values of 8.7 and 9.6 Hz, respectively. Doublets C and E are assigned to the β proton of a pair of gem-diol isomers with coupling constants of 4.0 and 4.7 Hz, respectively. Doublet D is assigned to o an additional trithiolane species with a large coupling constant of 9.6 Hz indicative of a molecule with free rotation. (Note. Doublets assigned based on COSY and HSQC correlations.) An additional doublet F is also observed overlapping with doublet D and C with a coupling constant value of 7.4 Hz.*

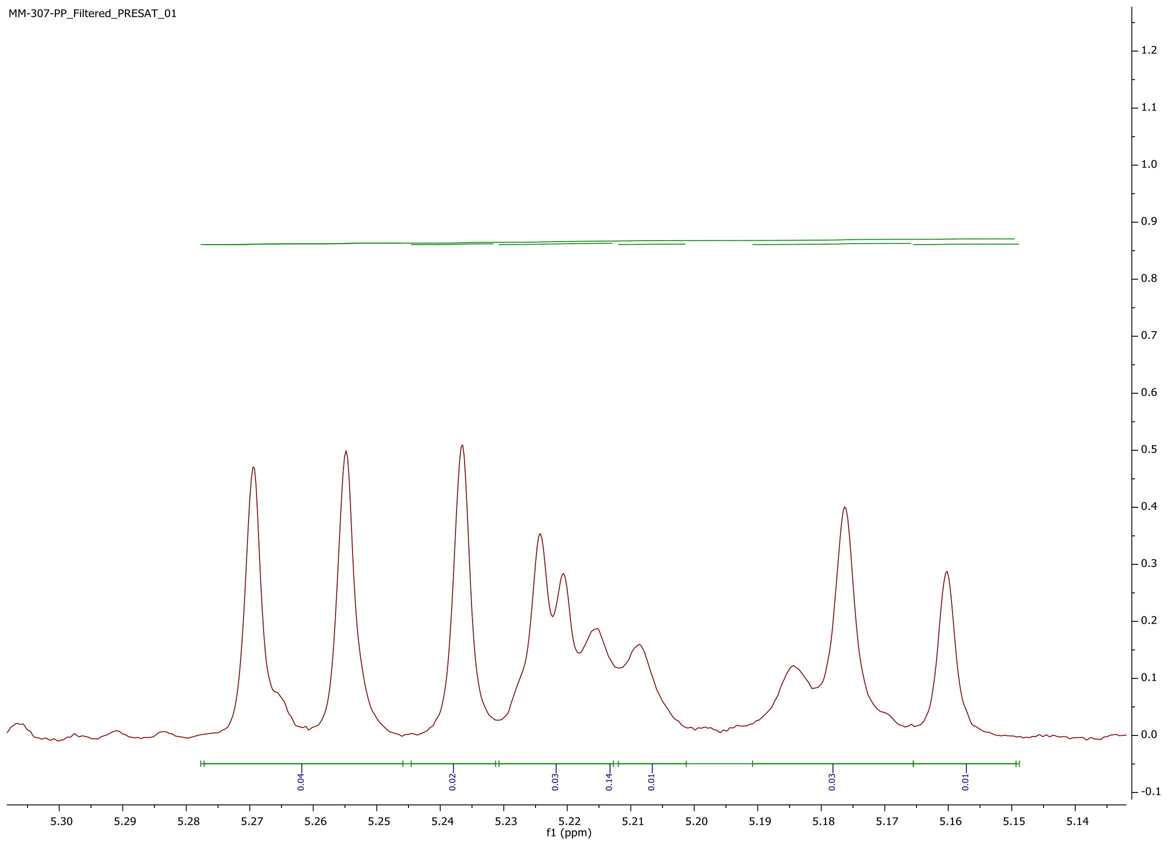

Figure I19: Integrating the methyl signal of the alanine residue outlined above allows for determination of the relative yields of some of the reaction products. The peaks in the range 5.15– 5.30 ppm account for 14% of the reaction products. Both isomers of the diol species account for 4% and trithiolanes account for 10%. The yields of the remaining reaction products could not be determined due to the complexity of the reaction mixture.

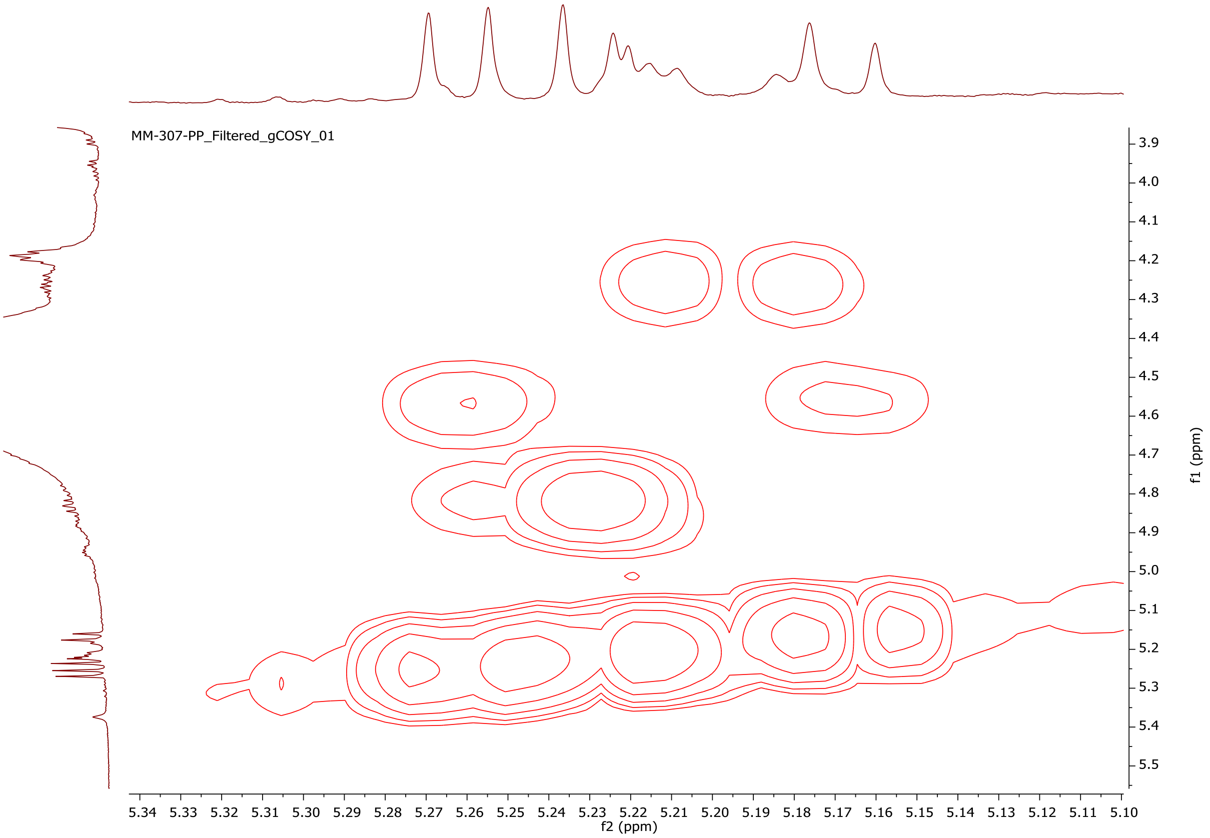

Figure I20: COSY spectrum showing correlations between the α and β protons of each species.

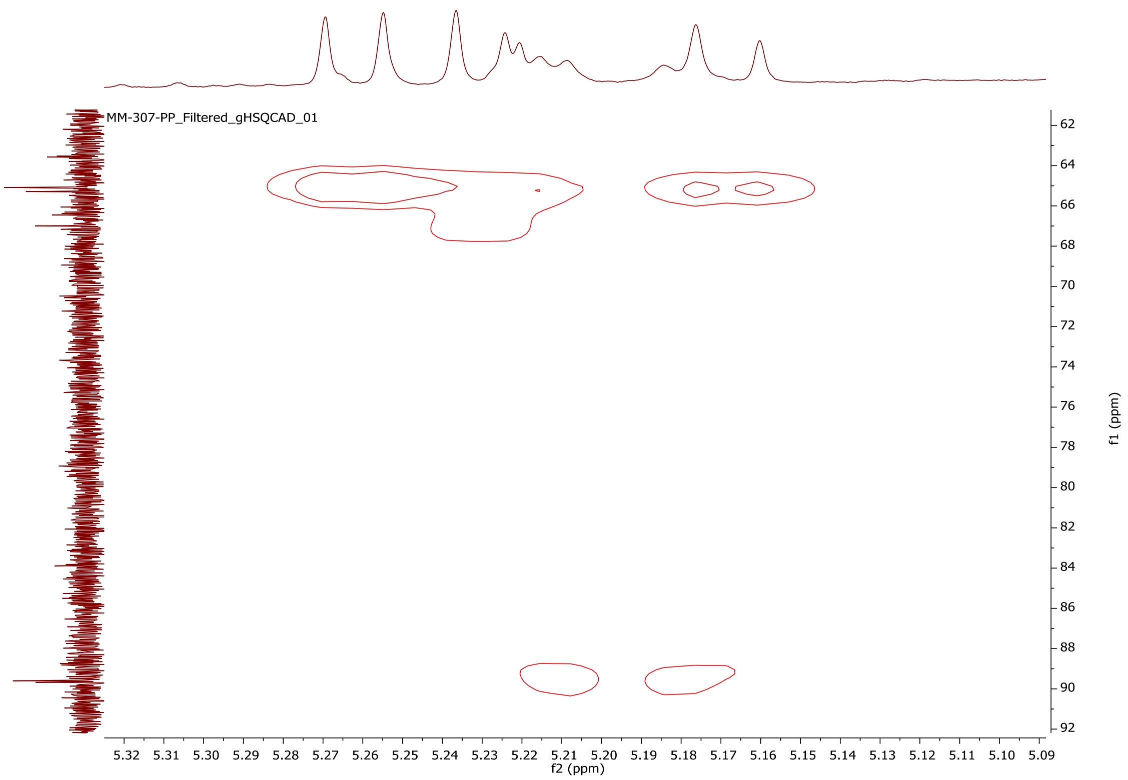

Figure I21: HSQC spectrum of the photolysis for compound ***22b*** *in the absence of buffer. Highlighted are the correlations of each of the methine doublets with the attached carbon. The gem-diol methine protons correlate with carbon signals at ca. 89.6 ppm. The trithiolane methine protons correlate with carbon signals at a more uplfield location (ca. 65–67 ppm).*

HRMS analysis of the reaction mixture derived from the photolysis of phenacylsulfide 22b in H2O/CD3CN (7:3)

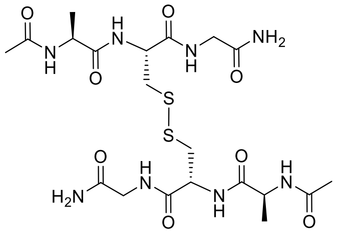

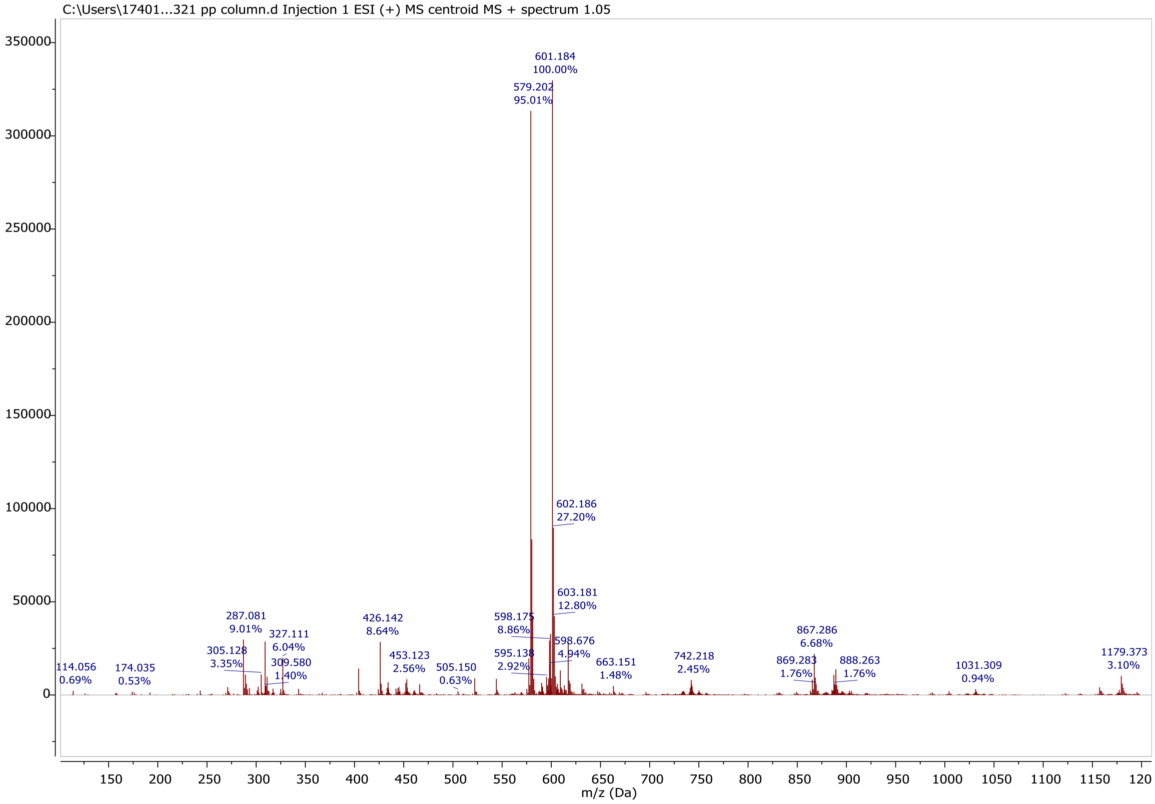

Figure I22: HRMS of AC-ACG-NH$_2$ disulfide showing the M+H (Expected: 579.201, Observed: 579.202) M+Na (Expected: 601.183, Observed: 601.184)

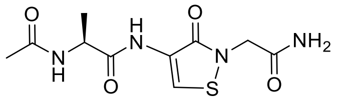

Exact Mass: 286.0736

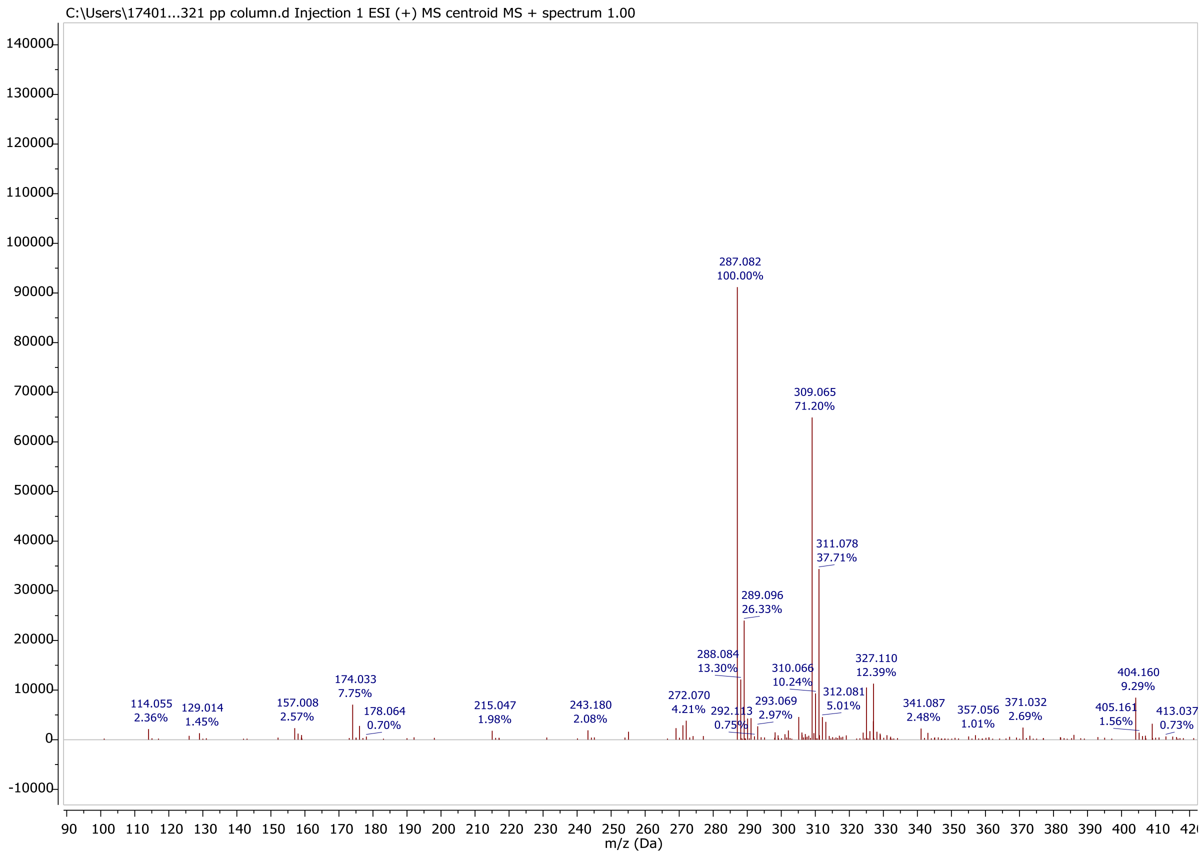

Figure I23: HRMS of AC-ACG-NH$_2$ isothiazolone showing the M+H (Expected: 287.081, Observed: 287.082) M+Na (Expected: 309.063, Observed: 309.065)

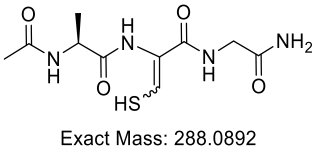

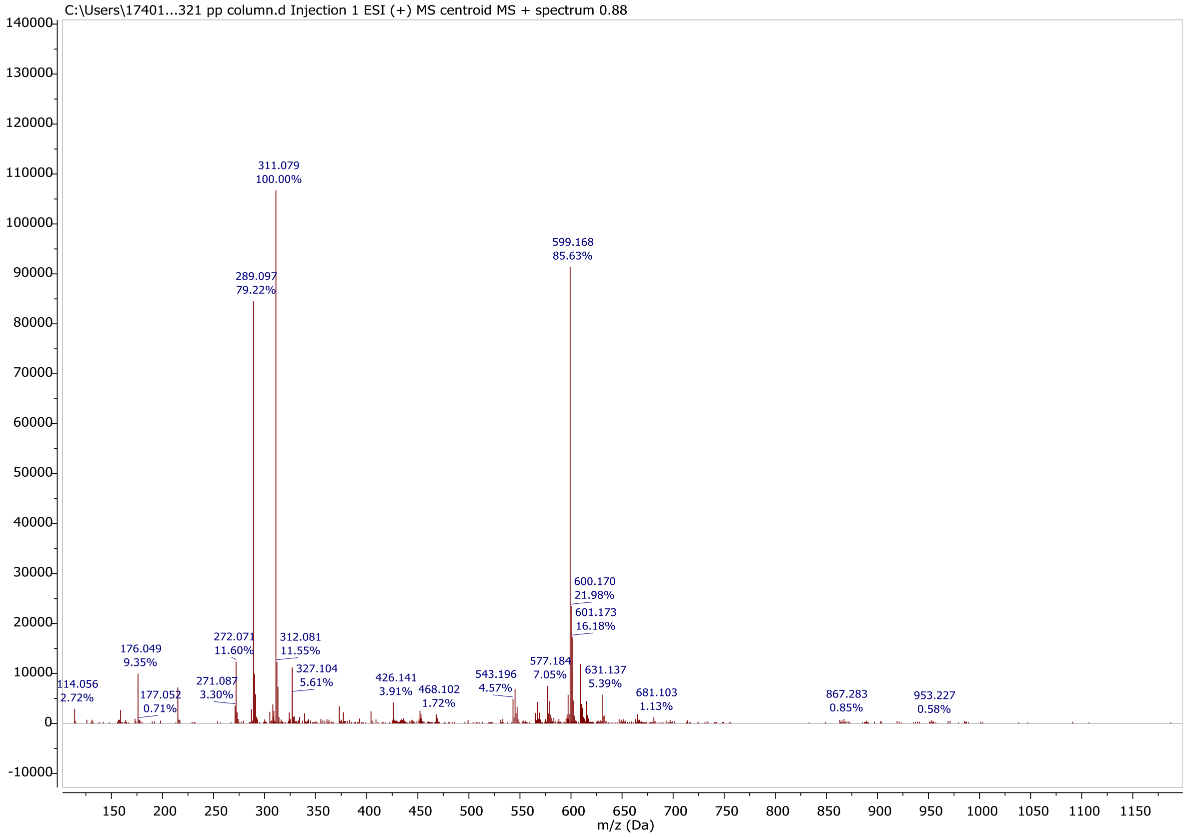

Figure I24: HRMS of Ac-ACG-NH$_2$ enethiol showing the M+H (Expected: 289.096, Observed: 289.097) M+Na (Expected: 311.079, Observed: 311.079) 2M+Na (Expected 599.167, Observed 599.168)

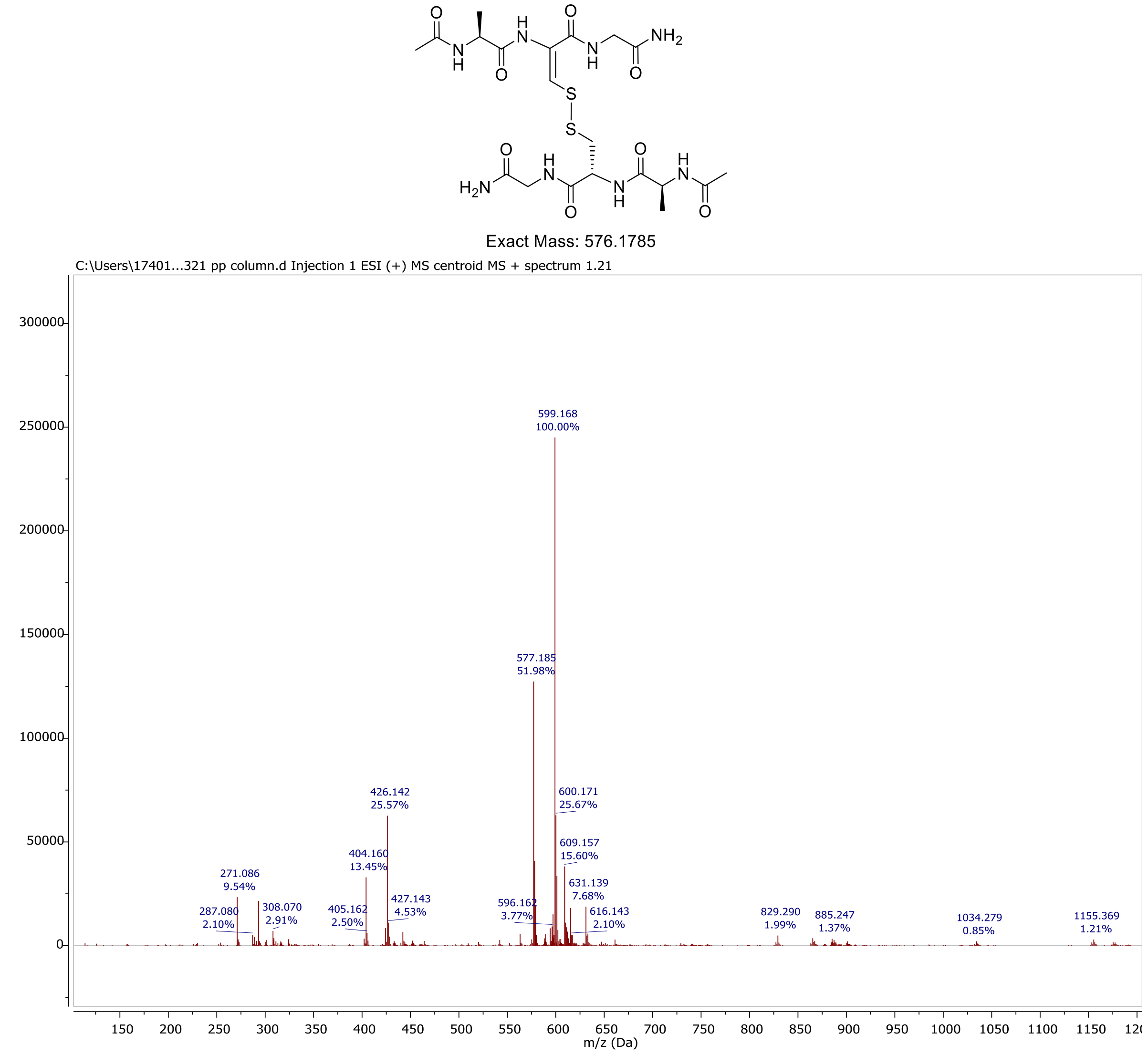

Figure I25: HRMS of Ac-ACG-NH$_2$ enethiol/thiol disulfide showing the M+H (Expected: 577.186, Observed: 577.185) M+Na (Expected: 599.168, Observed: 599.168)

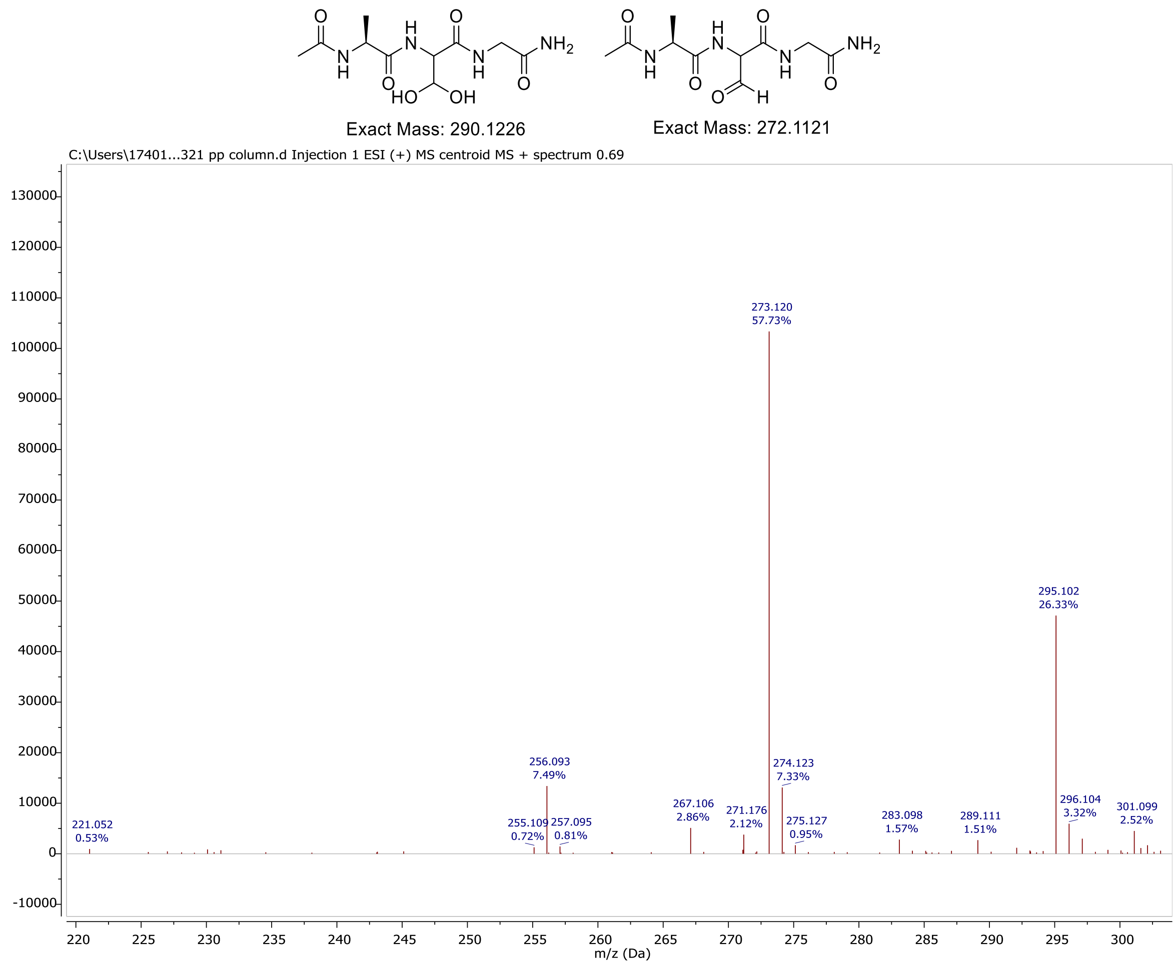

Figure I26: HRMS of Ac-ACG-NH$_2$ formylglycine species showing the M+H (Expected: 273.119, Observed: 273.120) M+Na (Expected: 295.101, Observed: 295.102)

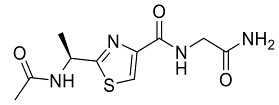

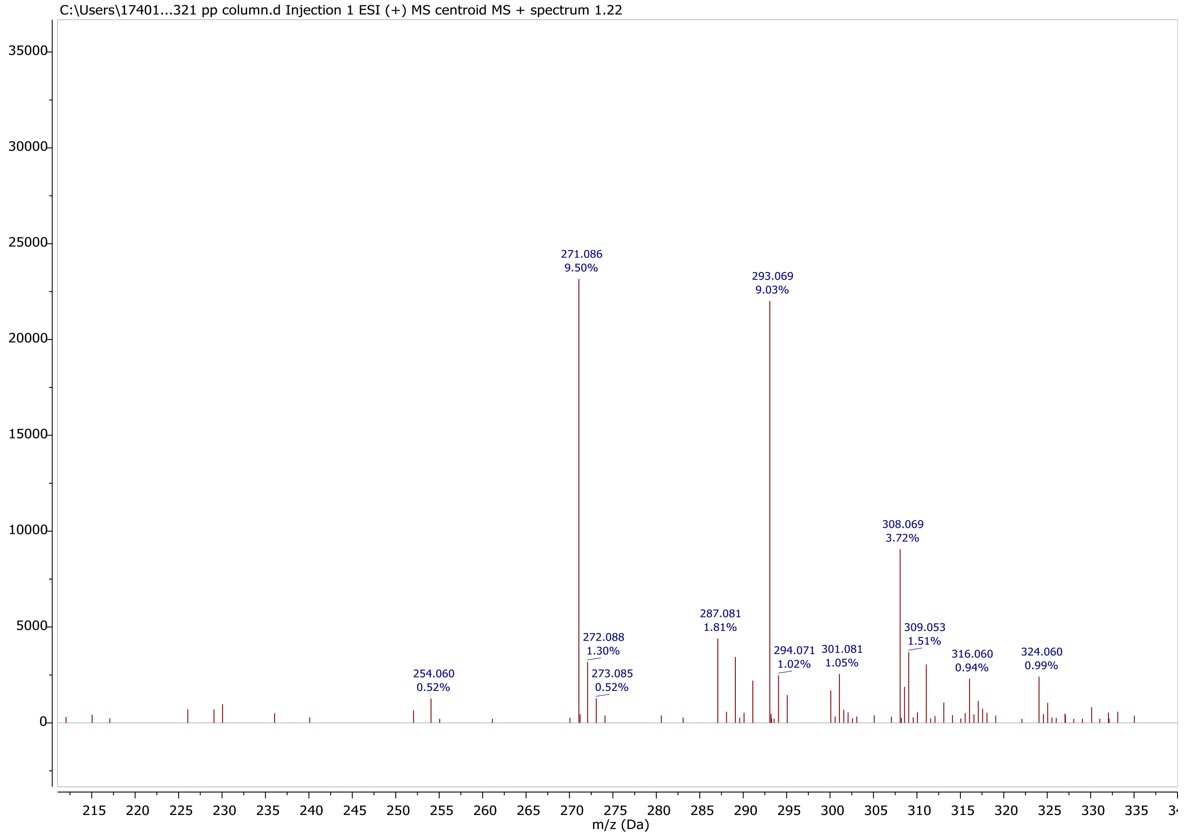

Figure I27: HRMS of Ac-ACG-NH$_2$ thiazole showing the M+H (Expected: 271.086, Observed: 271.086) M+Na (Expected: 293.06, Observed: 295.102)

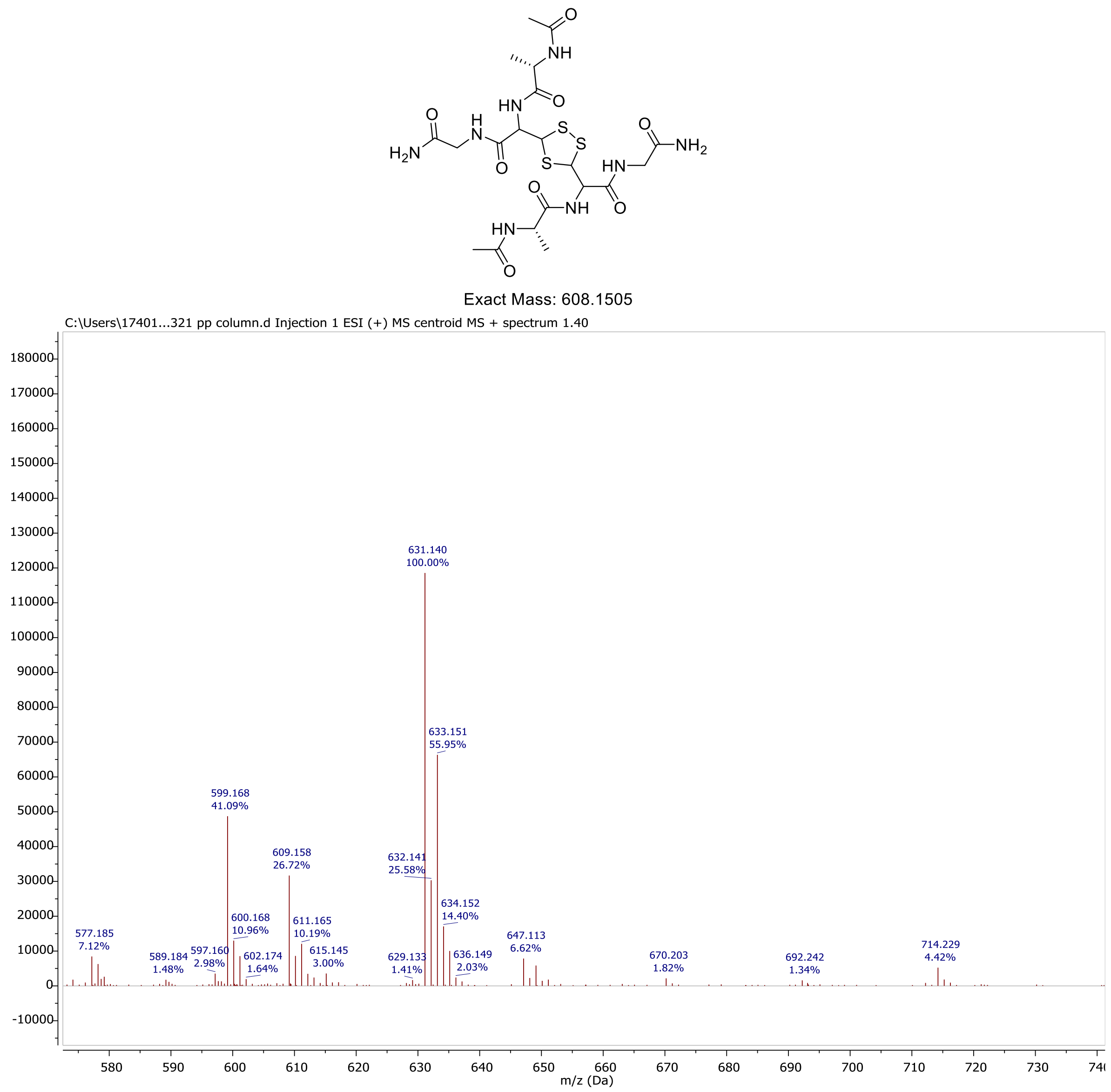

Figure I28: HRMS of the Ac-ACG-NH$_2$ 1,2,4-trithiolane showing M+H (Expected: 609.158, Observed: 609.158) & M+Na (Expected 631.140, Observed: 631.140)

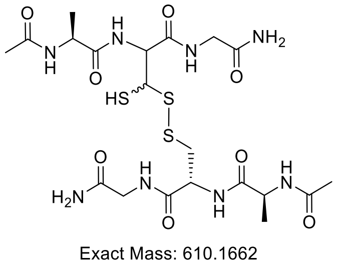

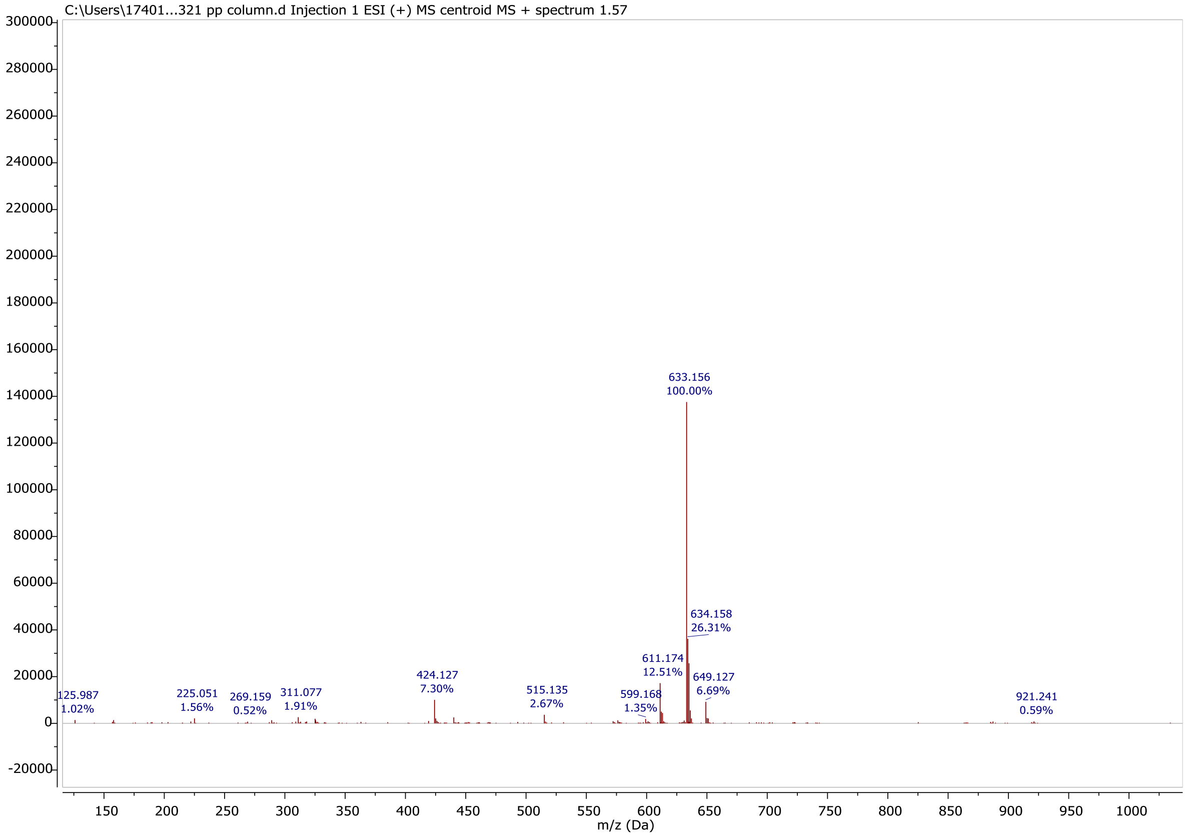

Figure I29: HRMS of Ac-ACG-NH_2 S,S-acetal/thiol adduct showing the M+H (Expected: 611.173, Observed: 611.174) & M+Na (Expected 633.155, Observed: 633.156)

K. Photolysis of pentadecapeptide phenacylsulfide derivatives

Solid phase peptide synthesis of pentadecapeptides

Peptides were synthesised on 0.1 mmol scale following the general procedure outlined in section I. The crude mixtures were analysed by analytical HPLC and HRMS (ESI). Analytical HPLC was performed on an Agilent 1260 infinity HPLC system with an Agilent 1260 infinity auto sampler. An Agilent Zorbax RX-C18 column (4.6 x 250 mm, 5 μm packing diameter) at 24 ^{o}C with a flow rate of 1 mL/min was used and the absorbance values were measured at 214 nm. The eluent used was a gradient composed of mixtures of the following components: A = 0.1 % v/v solution of TFA in water; B = 0.1 % v/v solution of TFA in acetonitrile.

Table K1: Analytical HPLC method used for characterisation of peptide thiol ***31a–31f***

time (min)	A (%)	B (%)
0	95	5
5	80	20
7	80	20
25	63	37
30	0	100
35	100	0

The crude peptides were subjected to *S*-alkylation without further purification.

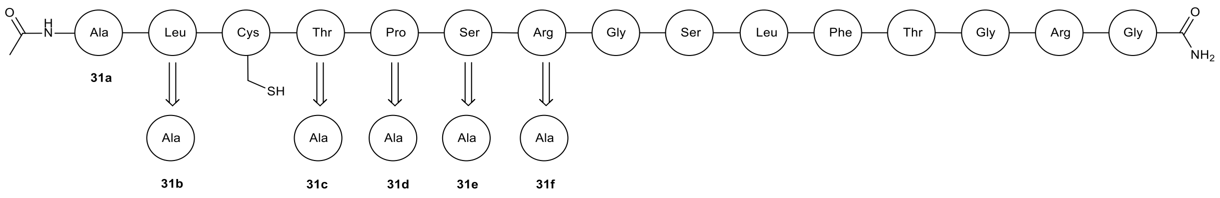

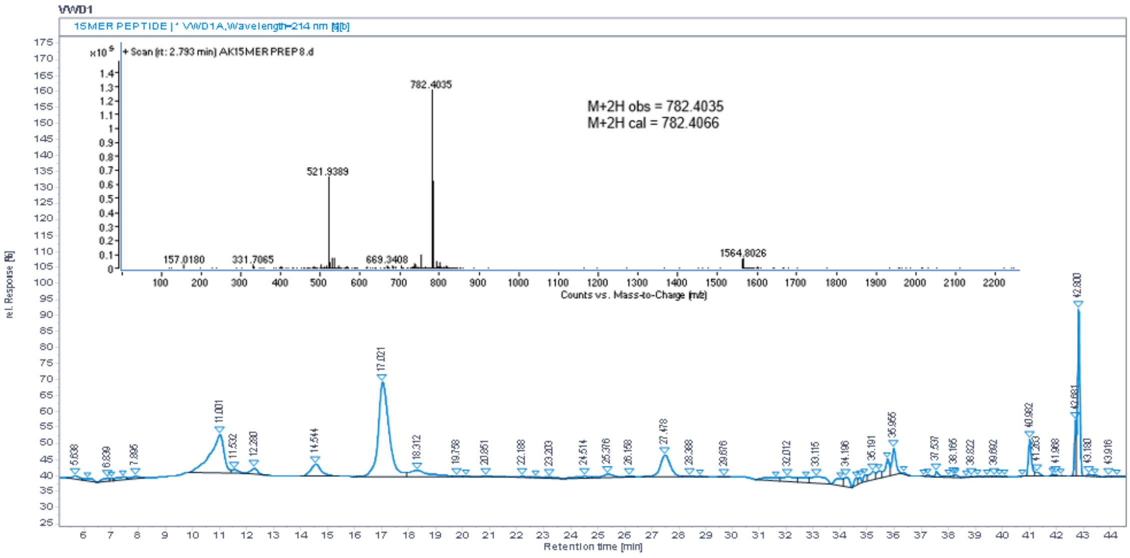

Figure K1: Analytical HPLC spectrum of crude pentadecapeptide ***31a*** *(RT: 17.021 min). HRMS (ESI positive mode) m/z peaks (observed and calculated) are presented in the inset.*

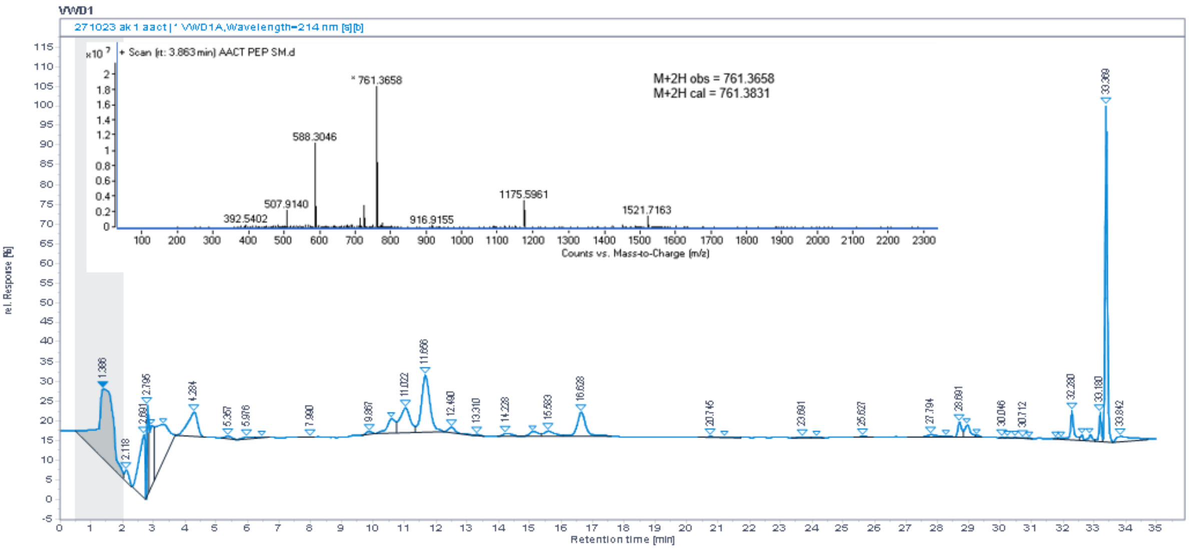

*Figure K2: Analytical HPLC spectrum of crude pentadecapeptide **31b** (RT: 16.628 min). HRMS (ESI positive mode) m/z peaks (observed and calculated) are presented in the inset.*

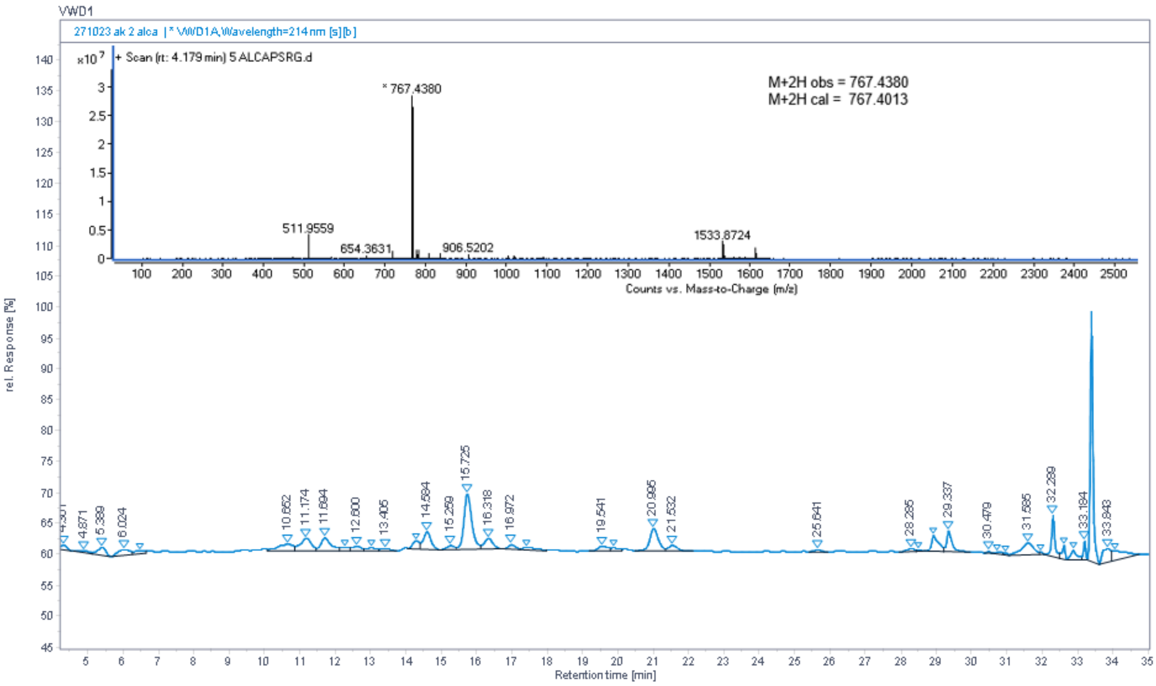

*Figure K3: Analytical HPLC spectrum of crude pentadecapeptide **31c** (RT: 15.725 min). HRMS (ESI positive mode) m/z peaks (observed and calculated) are presented in the inset.*

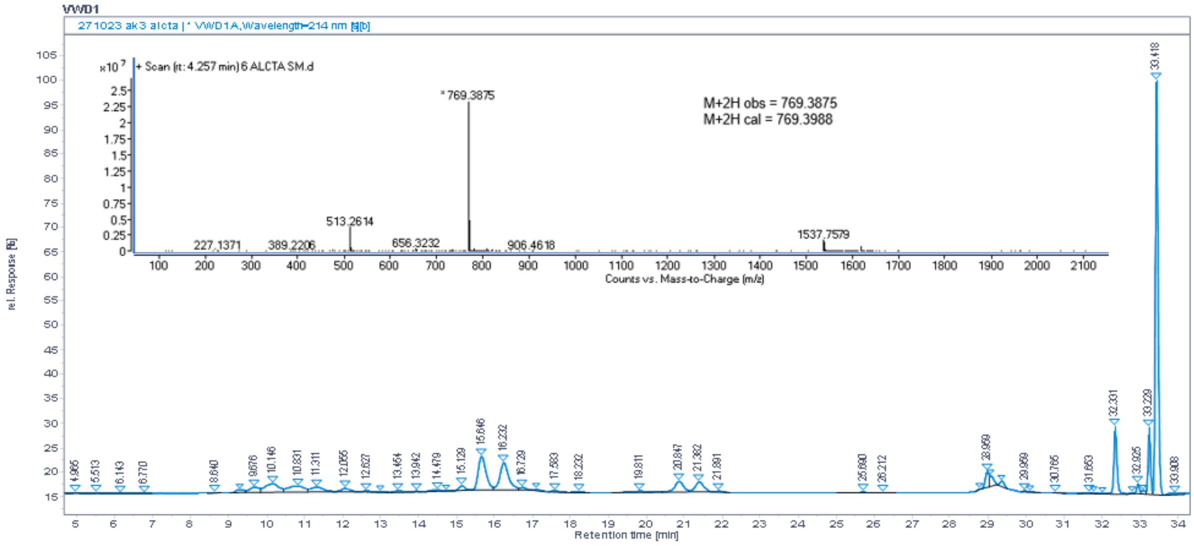

Figure K4: Analytical HPLC spectrum of crude pentadecapeptide ***31d*** *(RT: 15.565 min). HRMS (ESI positive mode) m/z peaks (observed and calculated) are presented in the inset.*

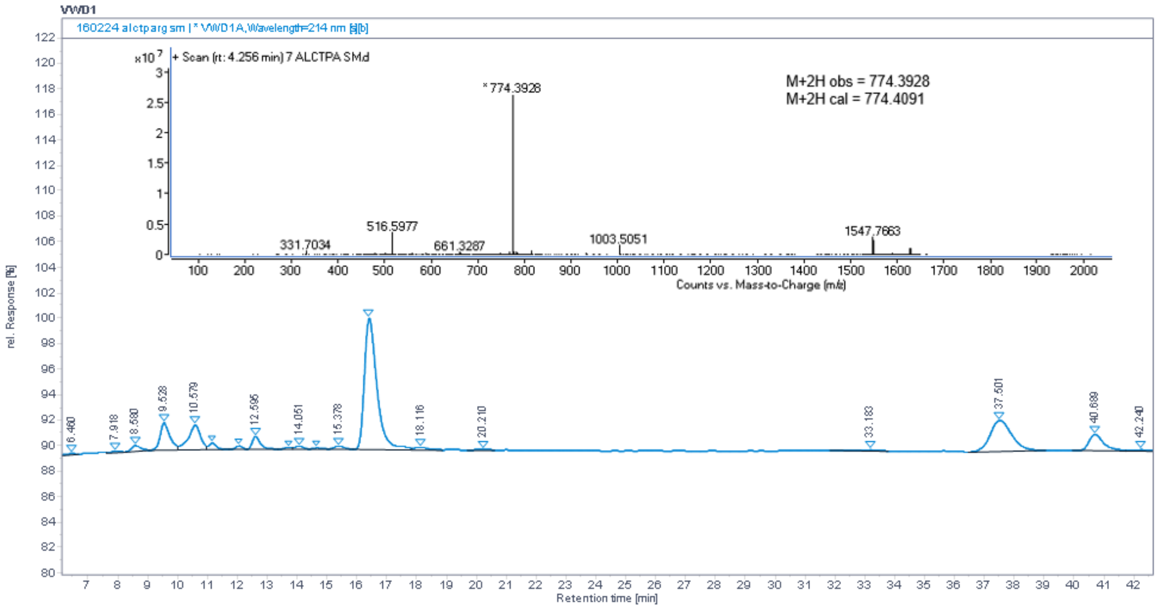

Figure K5: Analytical HPLC spectrum of crude pentadecapeptide ***31e*** *(RT: 16.553 min). HRMS (ESI positive mode) m/z peaks (observed and calculated) are presented in the inset.*

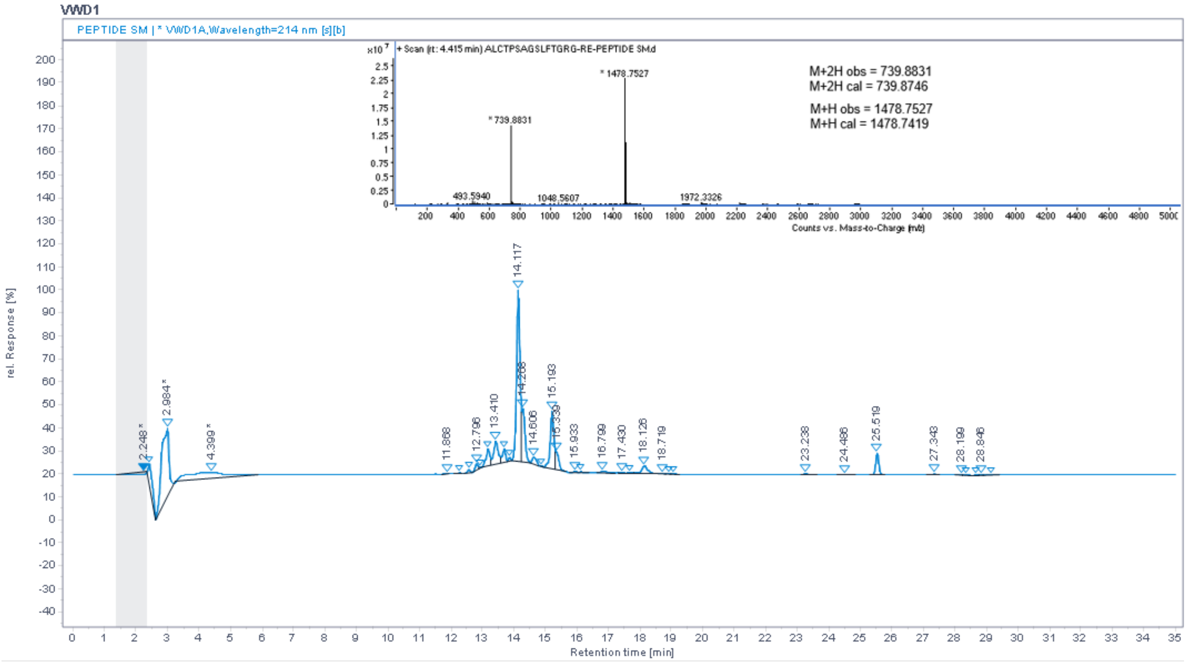

Figure K6: Analytical HPLC spectrum of crude pentadecapeptide ***31f*** *(RT: 14.117 min). HRMS (ESI positive mode) m/z peaks (observed and calculated) are presented in the inset.*

Table K2: Purity of crude peptide thiol ***31a–f***, as determined *by analytical HPLC analysis after SPPS.*

**Peptide**	**Purity**
ALCTPSRGSLFTGRG ***31a***	46 %
AACTPSRGSLFTGRG ***31b***	15 %
ALCAPSRGSLFTGRG ***31c***	25 %
ALCTASRGSLFTGRG ***31d***	31 %
ALCTPARGSLFTGRG ***31e***	41 %
ALCTPSAGSLFTGRG ***31f***	49 %

Synthesis of pentadecapeptide phenacylsulfide derivatives.

General procedure

Crude peptide thiol (**31a**–**f**, 0.03 mmol, 1 eq.) was dissolved in H_2O (0.1 M phosphate, pH 7.4, 1 mL). *p*-Trimethylammonium phenacylbromide (0.03 mmol, 1 eq.) was then added as a solution in H_2O (0.1 M phosphate, pH 7.4, 0.5 mL). The reaction mixture (1.5 mL) was stirred overnight under N_2. The target phenacylsulfide was purified by using semi-preparative HPLC. Semi-preparative HPLC was performed on an Agilent 1260 Infinity HPLC system with an Agilent 1200 series auto sampler. A Macherey Nagel NUCLEODUR C18 HTec column (10 x 250 mm, 5 μm packing diameter) at 24 °C with a flow rate of 2.5 mL/min was used and the absorbance were measured at 214 nm. The eluent used was a gradient composed of mixtures of the following components: A = 0.1 % v/v solution of TFA in water; B = 0.1 % v/v solution of TFA in acetonitrile. Collected fractions were frozen and lyophilised.

Table K3: Analytical HPLC method used for characterisation of phenacylsulfides ***32*****–*****37***

**time (min)**	**A (%)**	**B (%)**
0	95	5
5	80	20
7	80	20
25	63	37
30	0	100
35	100	0

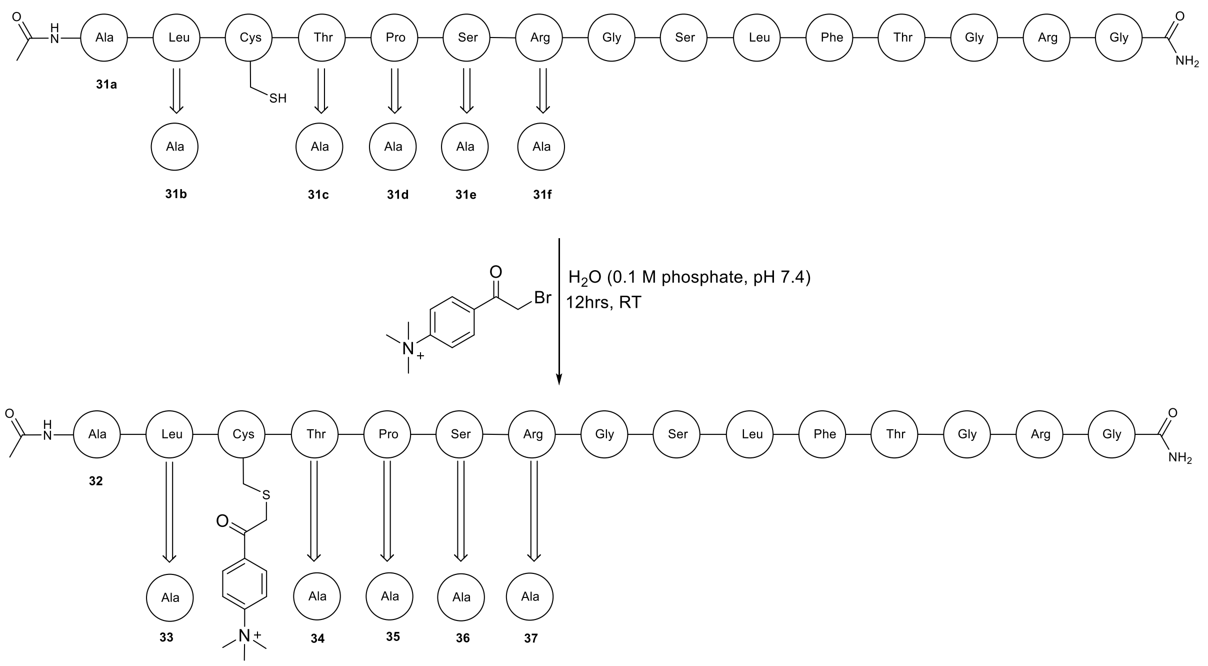

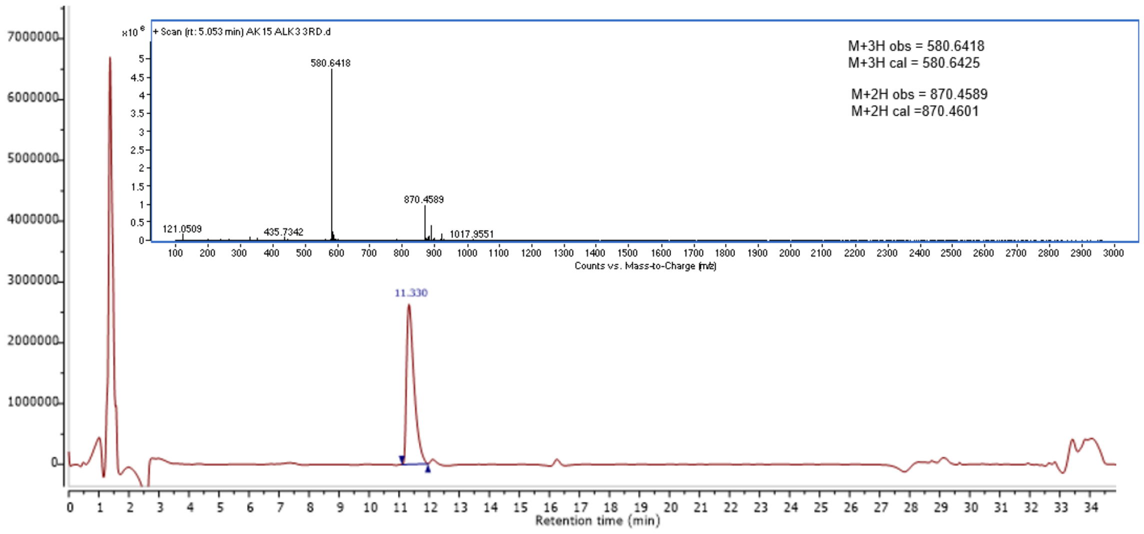

Figure K7: Analytical HPLC spectrum of purified phenacylsulfide ***32*** *(RT: 11.330 min). HRMS (ESI positive mode) m/z peaks (observed and calculated) are presented in the inset.*

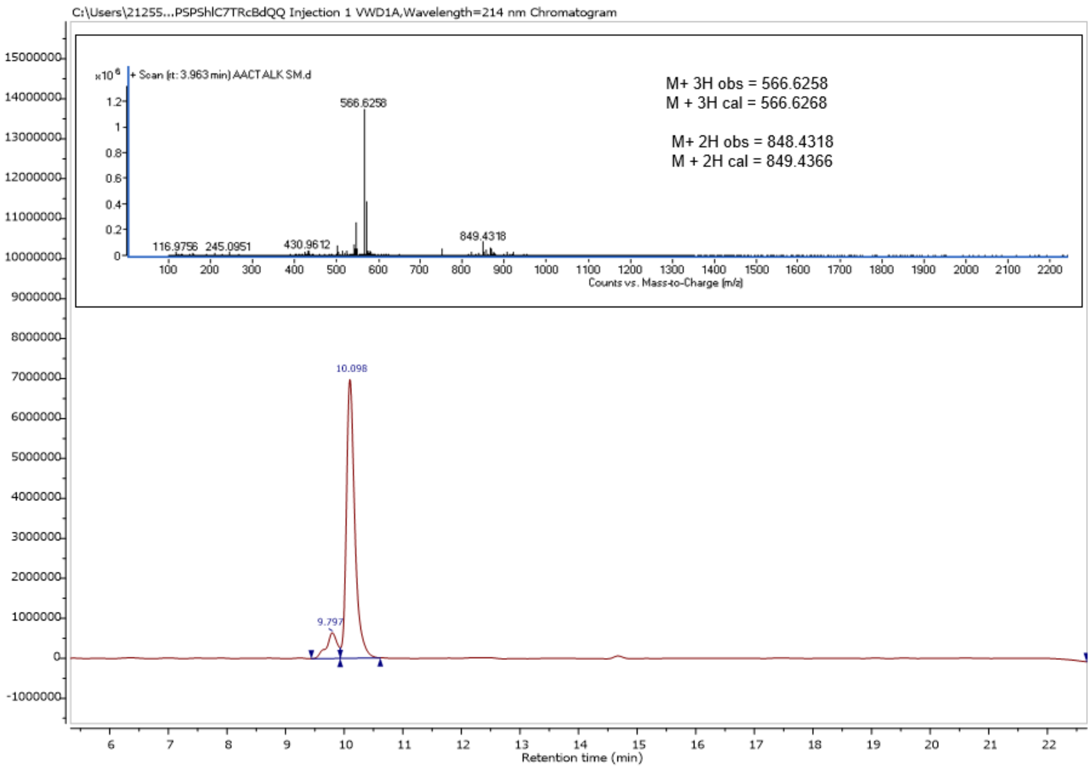

*Figure K8: Analytical HPLC spectrum of purified phenacylsulfide **33** (RT: 10.098 min). HRMS (ESI positive mode) m/z peaks (observed and calculated) are presented in the inset.*

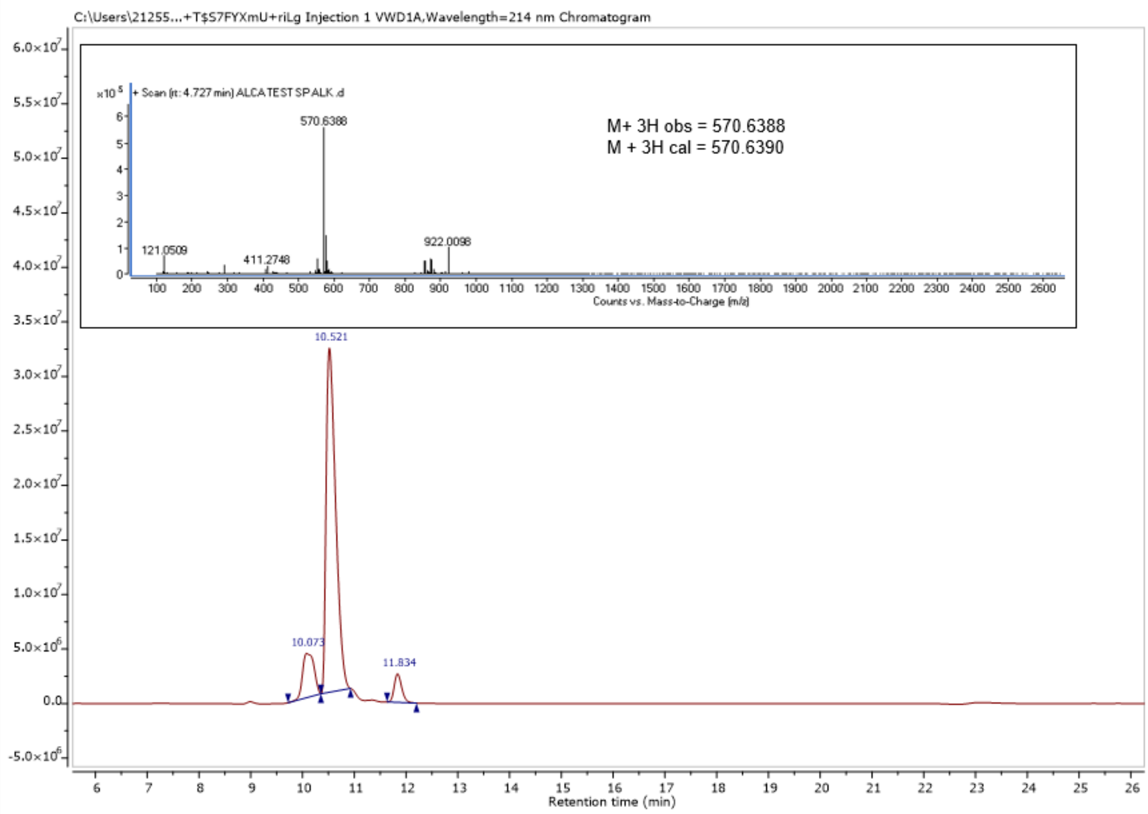

*Figure K9: Analytical HPLC spectrum of purified phenacylsulfide **34** (RT: 10.521 min). HRMS (ESI positive mode) m/z peaks (observed and calculated) are presented in the inset.*

.

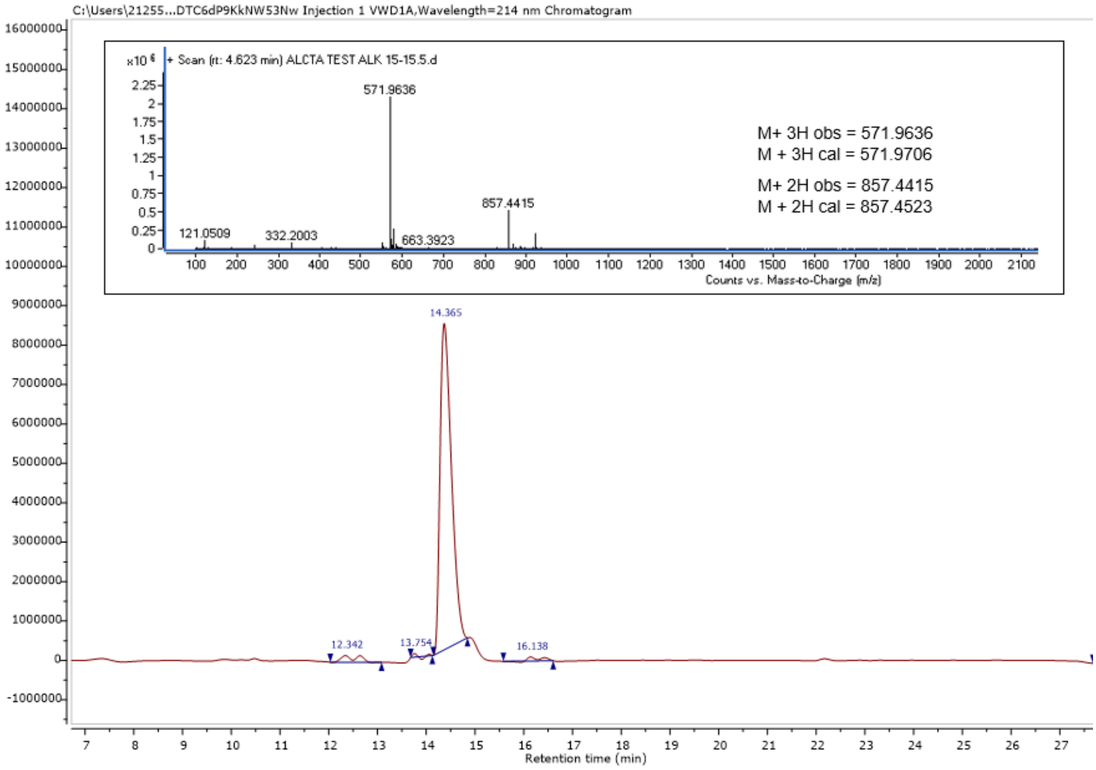

*Figure K10: Analytical HPLC spectrum of purified phenacylsulfide **35** (RT: 14.365 min). HRMS (ESI positive mode) m/z peaks (observed and calculated) are presented in the inset.*

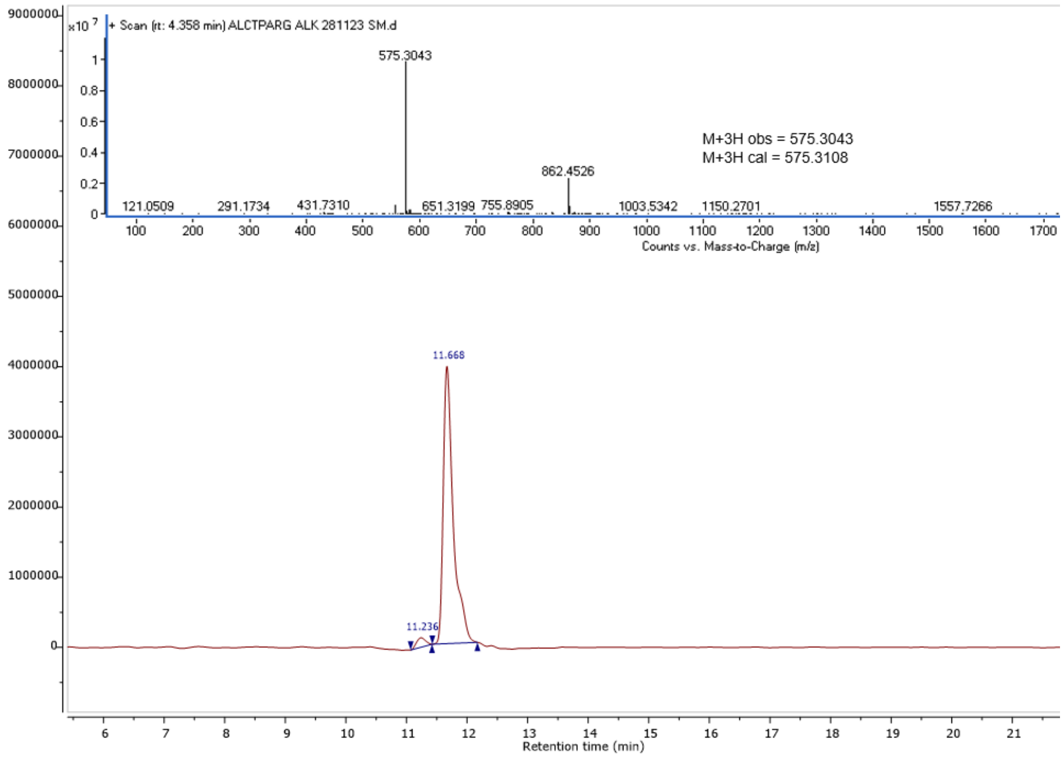

*Figure K11: Analytical HPLC spectrum of purified phenacylsulfide **36** (RT: 11.668 min). HRMS (ESI positive mode) m/z peaks (observed and calculated) are presented in the inset.*

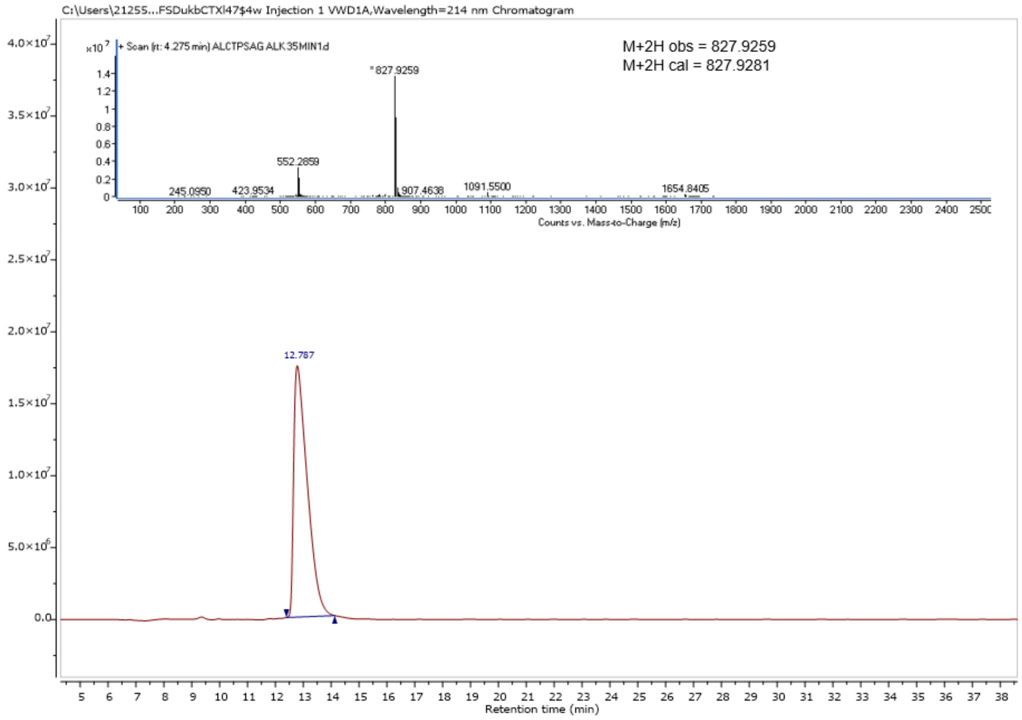

*Figure K12: Analytical HPLC spectrum of purified phenacylsulfide **37** (RT: 12.787 min). HRMS (ESI positive mode) m/z peaks (observed and calculated) are presented in the inset.*

*Table K4: Yield of isolated peptide phenacylsulfides **32–37** after solid-phase peptide synthesis, alkylation and semi-preparative HPLC purification.*

peptide	yield
ALCTPSRGSLFTGRG ***32***	6 %
AACTPSRGSLFTGRG ***33***	5 %
ALCAPSRGSLFTGRG ***34***	7 %
ALCTASRGSLFTGRG ***35***	6 %
ALCTPARGSLFTGRG ***36***	5 %
ALCTPSAGSLFTGRG ***37***	5 %

Products formed in the photolysis of pentadecapeptide phenacylsulfides

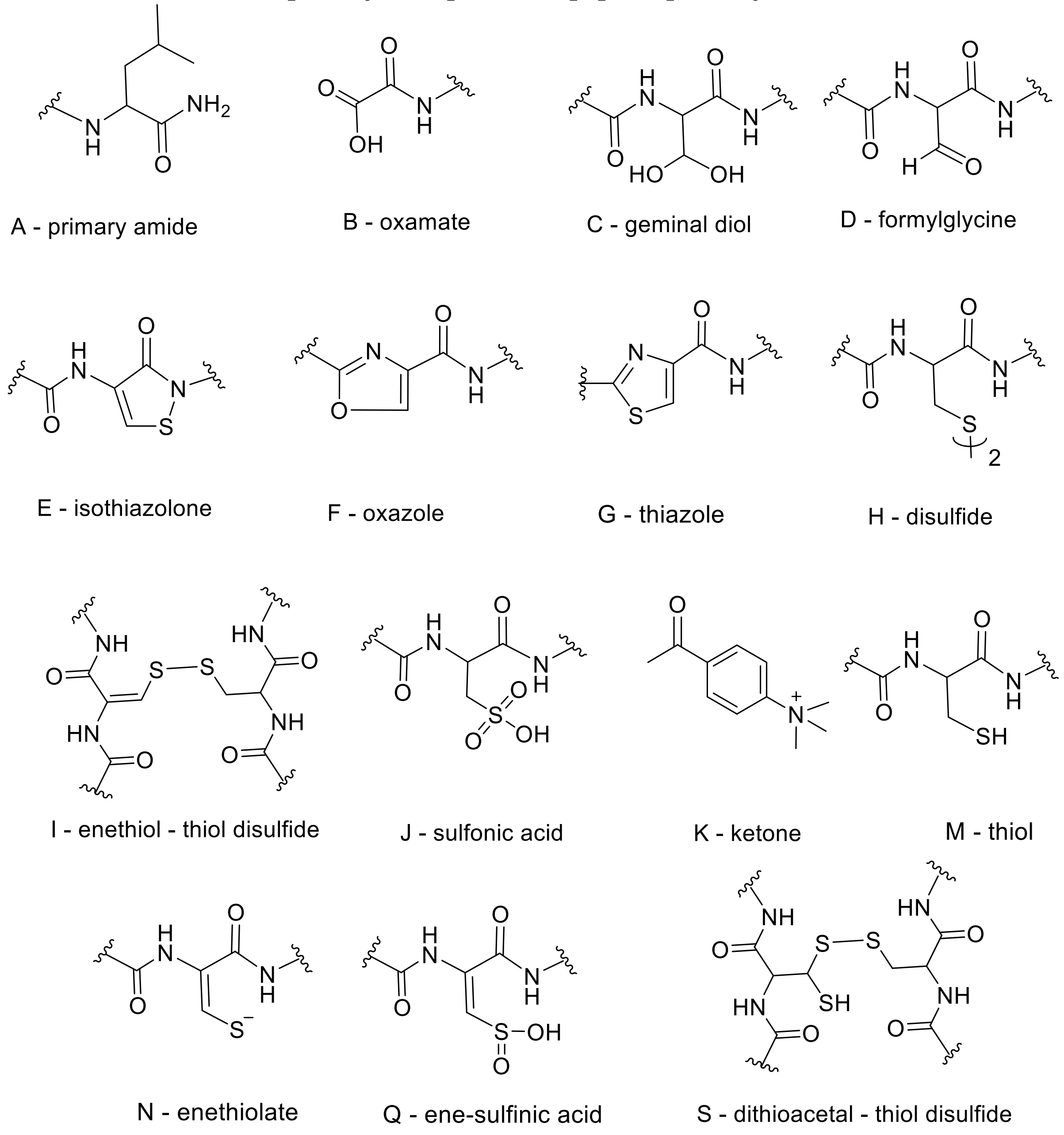

Figure K13: Products formed from the photolysis of phenacylsulfides ***32–37****.*

Photolysis of phenacylsulfide (32) and investigation of products.

A solution of phenacylsulfide ***32*** (0.0017 mmol) in H_2O (0.1 M phosphate, pH 7.4, 1 mL, 1.7 mM solution) was degassed by passing N_2 gas through the solution in a glass vial sealed with a rubber stopper for 20 mins. The solution was then transferred to an NMR tube and illuminated at 350 nm in a Rayonet photo reactor for 2 hrs at room temperature. The reaction mixture (50 μL) upon complete conversion of starting material was analysed by analytical HPLC by using Agilent Zorbax RX-C18 column (4.6 x 250 mm, 5 μm packing diameter) at 24 °C with a flow rate of 1 mL/min. The HPLC method was optimised to achieve good separation between product peaks, before collection of each peak in the HPLC spectrum for HRMS analysis. Relative integration of HPLC peaks was used to approximate conversion values for each product. The collected samples from the HPLC system were then injected into the mass spectrometer passing through an Acquity UPLC® BEH C18 column (2.1 x 150 mm, 1.7 μm packing diameter) with a flow rate of 0.3 mL/min. The solvents employed were as follows: A = 0.1 % v/v solution of formic acid in water; B = 0.1 % v/v solution of formic acid in acetonitrile. Mass-to-charge (*m/z*) ratio of the products were identified from the mass spectrum (observed *m/z* value) and compared with the calculated *m/z* value to ensure that the difference between the values do not differ more than 0.003.

*Table K5: Analytical HPLC method used for characterisation of photolysis products (phenacylsulfide **32**, H_2O, 0.1 M phosphate, pH 7.4)*

time (min)	A (%)	B (%)
0	95	5
5	80	20
7	80	20
25	63	37
30	0	100
35	100	0

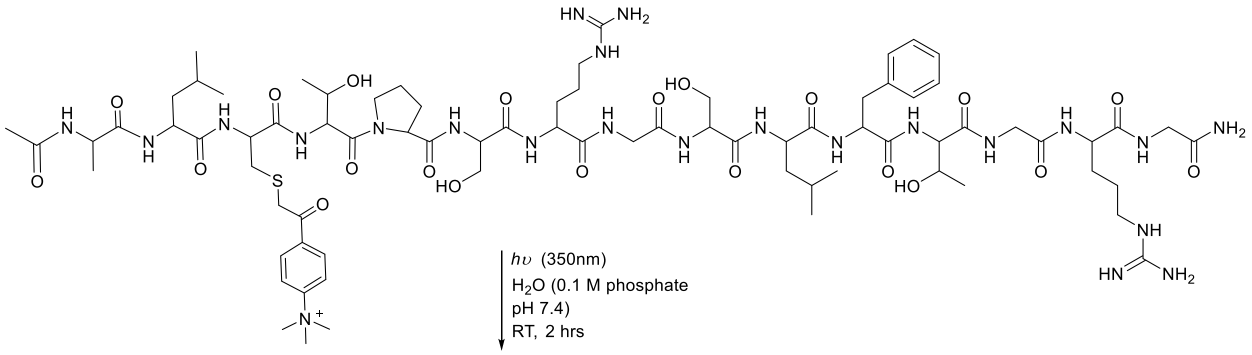

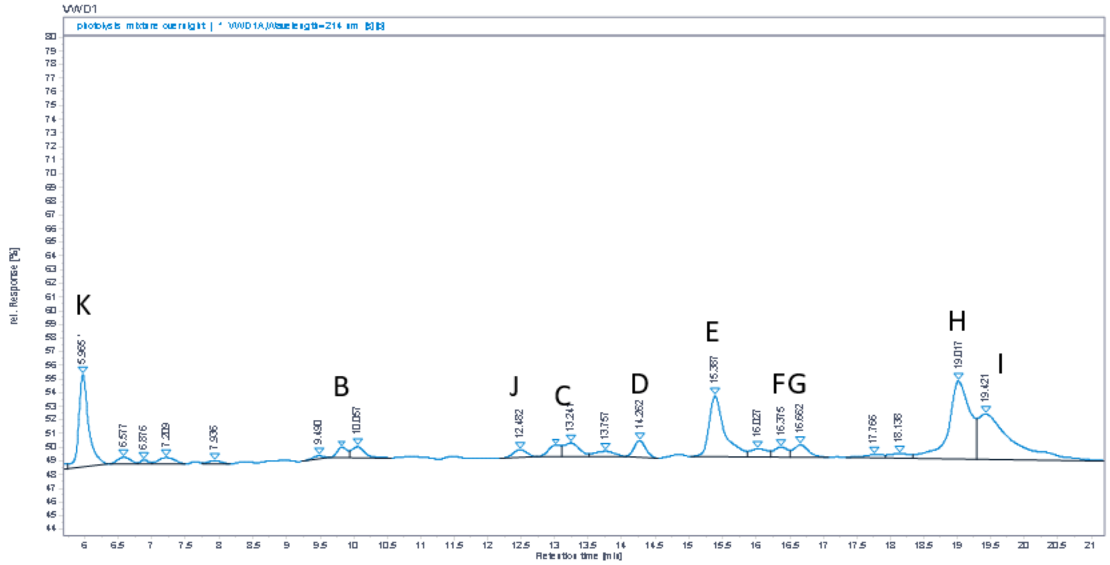

Figure K14: Analytical HPLC spectrum of photolysis reaction mixture. K= ketone, B=oxamate, J=sulfonic acid, C=geminal diol, D=formylglycine, E=isothiazolone, F=oxazole, G= thiazole (formed through cyclocondensation of enethiol in acidic HPLC eluent), H=disulfide, I=enethiol-thiol disulfide.

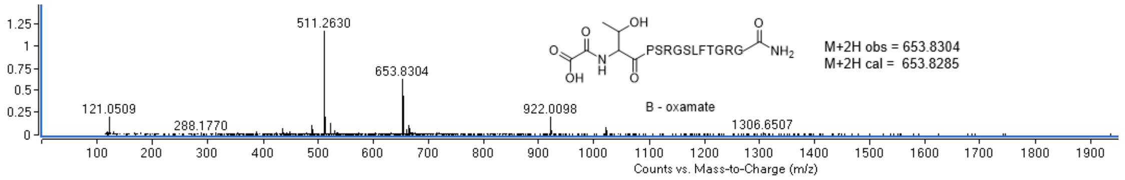

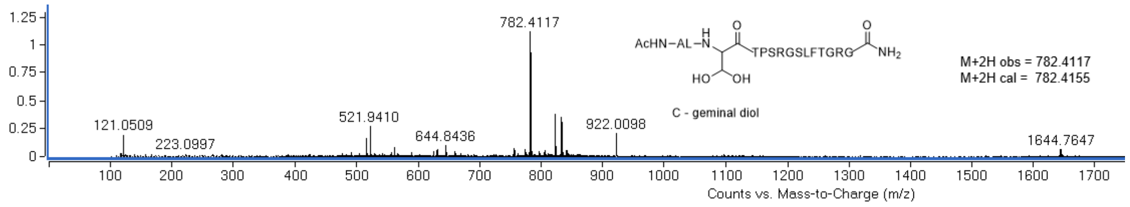

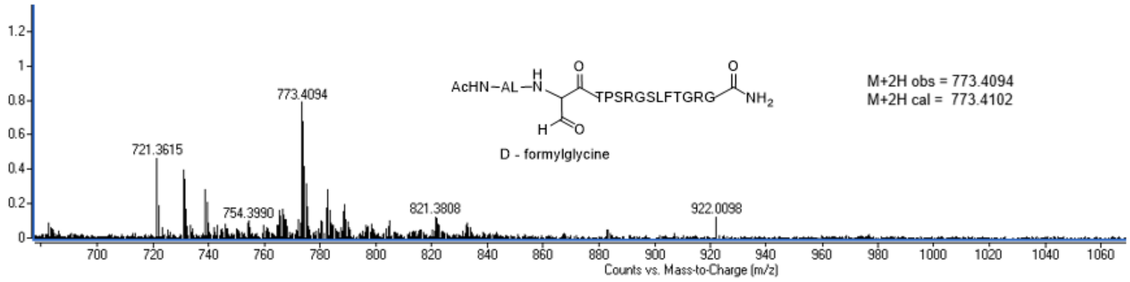
773.4094
721.3615
754.3990
821.3808
922.0098
AcHN–AL–N(H)–…–TPSRGSLFTGRG–NH2
D - formylglycine
M+2H obs = 773.4094
M+2H cal = 773.4102
Counts vs. Mass-to-Charge (m/z)

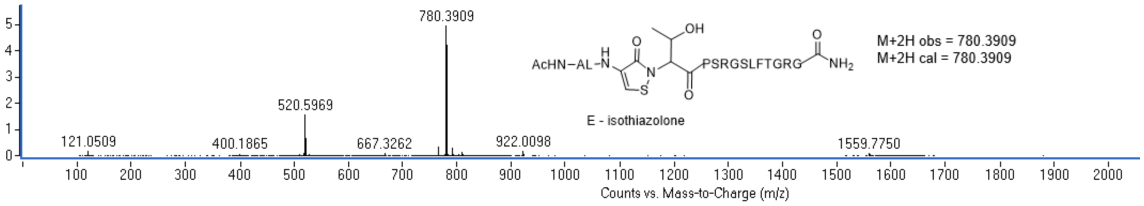
780.3909
520.5969
121.0509
400.1865
667.3262
922.0098
1559.7750
AcHN–AL–N(H)
PSRGSLFTGRG
NH2
E - isothiazolone
M+2H obs = 780.3909
M+2H cal = 780.3909
Counts vs. Mass-to-Charge (m/z)

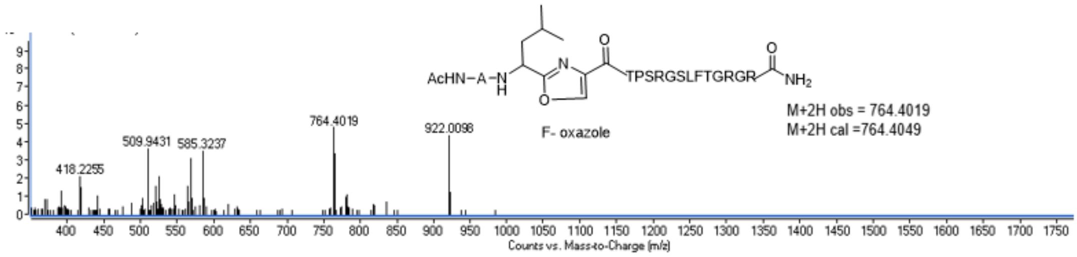
764.4019
922.0098
509.9431
585.3237
418.2255
AcHN–A–N(H)
TPSRGSLFTGRGR
NH2
F- oxazole
M+2H obs = 764.4019
M+2H cal =764.4049
Counts vs. Mass-to-Charge (m/z)

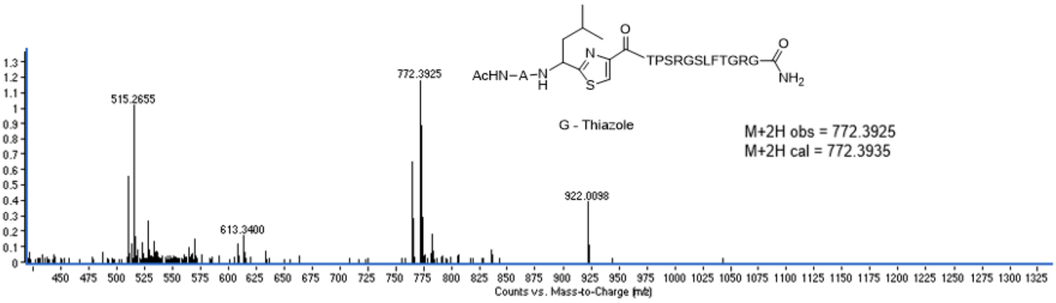
772.3925
515.2655
613.3400
922.0098
AcHN–A–N(H)
TPSRGSLFTGRG
NH2
G - Thiazole
M+2H obs = 772.3925
M+2H cal = 772.3935
Counts vs. Mass-to-Charge (m/z)

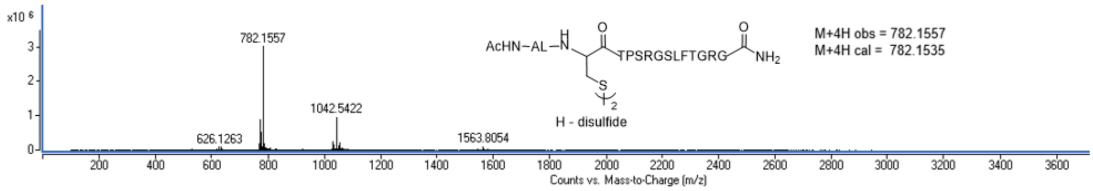
x10 6
782.1557
1042.5422
626.1263
1563.8054
AcHN–AL–N(H)
TPSRGSLFTGRG
NH2
H - disulfide
M+4H obs = 782.1557
M+4H cal = 782.1535
Counts vs. Mass-to-Charge (m/z)

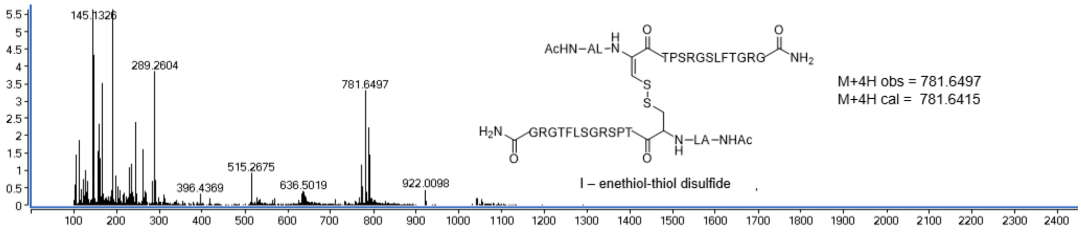
145.1326
289.2604
781.6497
515.2675
396.4369
636.5019
922.0098
AcHN–AL–N(H)
TPSRGSLFTGRG
NH2
H2N
GRGTFLSGRSPT
N(H)–LA–NHAc
I – enethiol-thiol disulfide
M+4H obs = 781.6497
M+4H cal = 781.6415

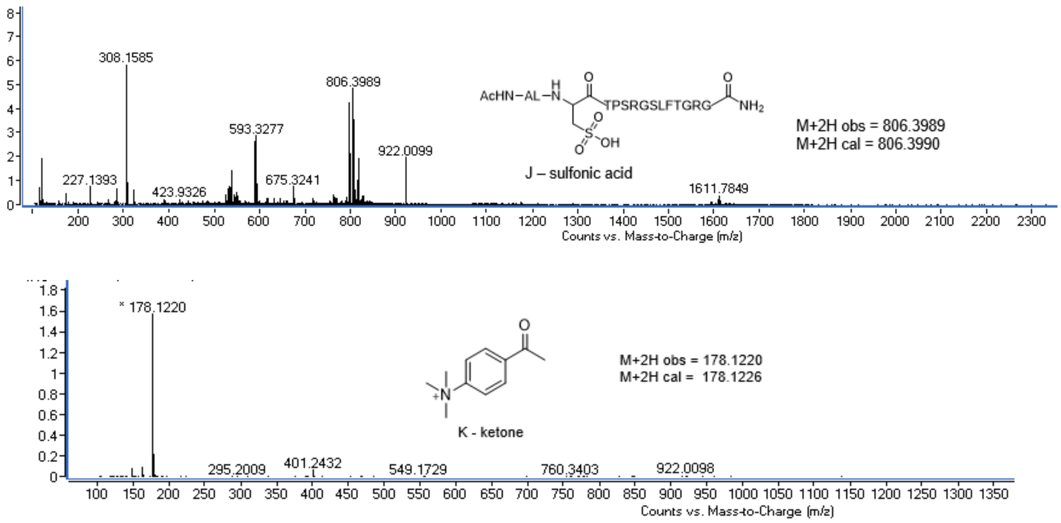

*Figure K15: HRMS (ESI positive mode) spectra of products derived from the photolysis of phenacylsulfide **32** (H_2O, 0.1 M phosphate, pH 7.4). Observed and calculated m/z peaks are provided.*

*Table K6: Approximate conversion values (%) of photolysis products (phenacylsulfide **32**, H_2O, 0.1 M phosphate, pH 7.4) based on integration of HPLC peaks (detection at 214 nm). Approximate conversions from phenacylsulfide (Norrish type-II and β-scission processes, column 2) and from putative thioaldehyde (Norrish type-II processes only, column 3).*

**Product**	**% Area (Conversion from phenacylsulfide)**	**% Area (Conversion from putative thioaldehyde)**
oxamate	2%	4%
geminal diol	8%	16%
formylglycine	4%	7%
isothiazolone	18%	34%
thiazole	3%	5%
oxazole	3%	6%
enethiol-thiol disulfide	29%	28%
disulfide	31%	
sulfonic acid	2%	

Synthesis of benzyl-vinyl sulfide

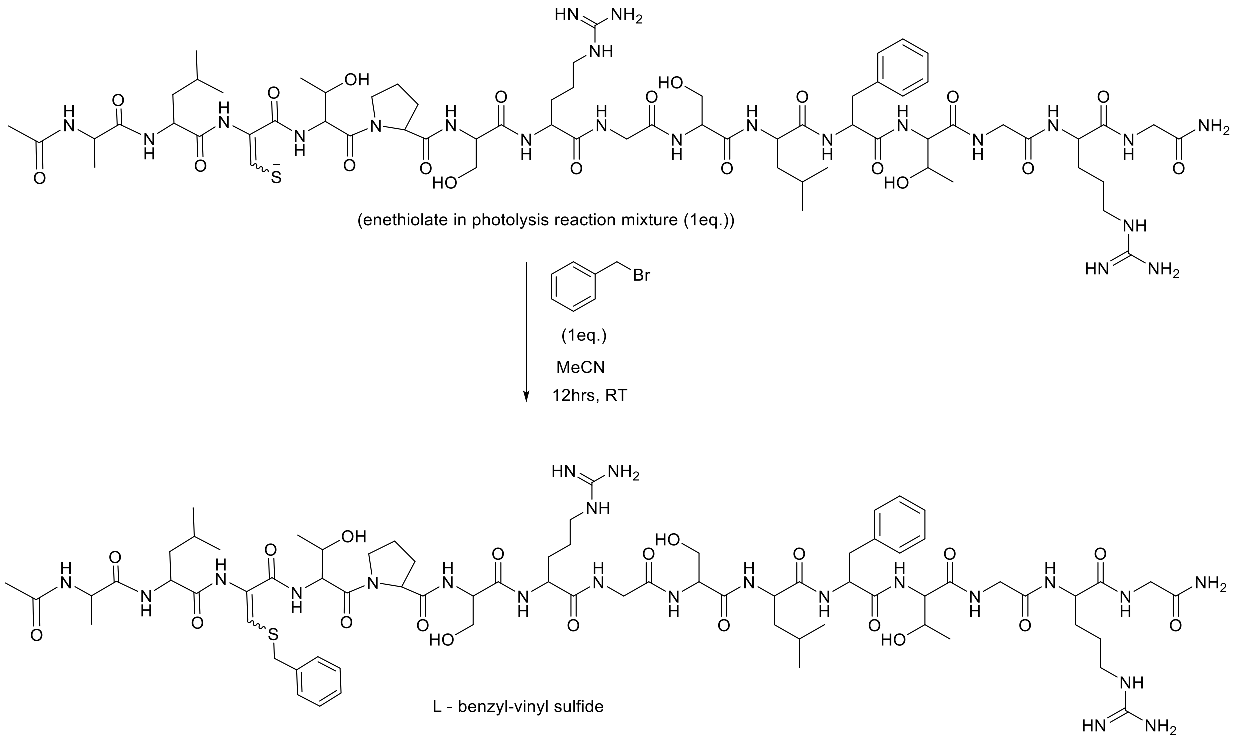

Benzyl bromide in acetonitrile (2 µL, 0.3 % v/v) was added to the photolysis reaction mixture derived from phenacylsulfide ***32*** (300 µL). The reaction mixture was allowed to evolve overnight under N_2 and was then analysed by analytical HPLC and HRMS (ESI positive mode).

*Table K7: Analytical HPLC method used for characterisation of photolysis products (phenacylsulfide **32**, H_2O, 0.1 M phosphate, pH 7.4) after addition of benzyl bromide.*

**time (min)**	**A (%)**	**B (%)**
0	95	5
5	80	20
7	80	20
25	63	37
30	0	100
35	100	0

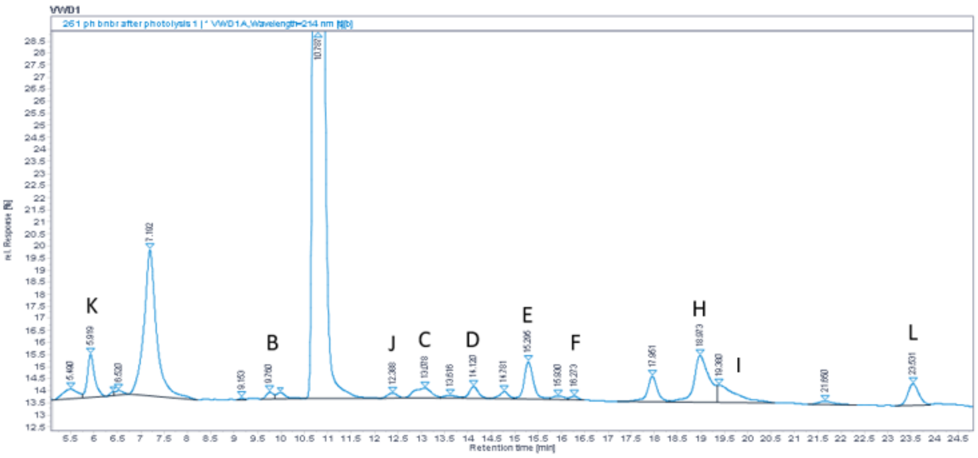

Figure K16: Analytical HPLC spectrum of photolysis reaction mixture after the addition of benzyl bromide. K=ketone, B=oxamate, J=sulfonic acid, C=geminal diol, D=formylglycine, E=isothiazolone, F=oxazole, H=disulfide, I=enethiolate-thiol disulfide, L = benzyl-vinyl sulfide. Peaks at 7.19 min and 17.95 min are impurities present in starting material.

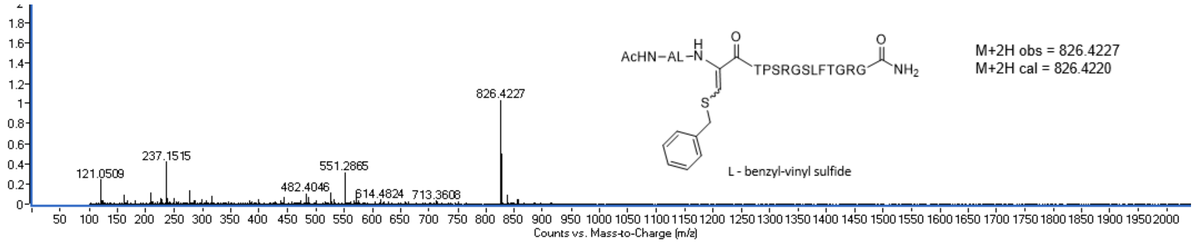

Figure K17: HRMS (ESI positive mode) spectrum of benzyl-vinyl sulfide. Observed and calculated m/z peaks are provided.

Reduction with TCEP

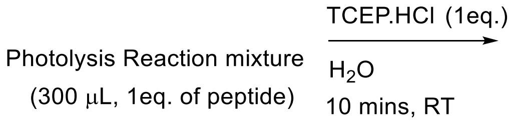

TCEP.HCl (12.8 mg, 0.05 mmol) was dissolved in HPLC-grade water (1 mL) and the pH of the solution was adjusted to pH 7.4 through dropwise addition of aqueous NaOH solution (6 mg, 0.15 mmol, 0.1 mL). The neutralised solution of TCEP (10 µL) was added to the photolysis reaction mixture (300 µL). After 10 mins, the mixture was analysed by analytical HPLC and HRMS (ESI positive mode).

*Table K8: Analytical HPLC method used for characterisation of photolysis products (phenacylsulfide **32**, H_2O, 0.1 M phosphate, pH 7.4) after TCEP addition.*

time (min)	A (%)	B (%)
0	95	5
5	80	20
7	80	20
25	63	37
30	0	100
35	100	0

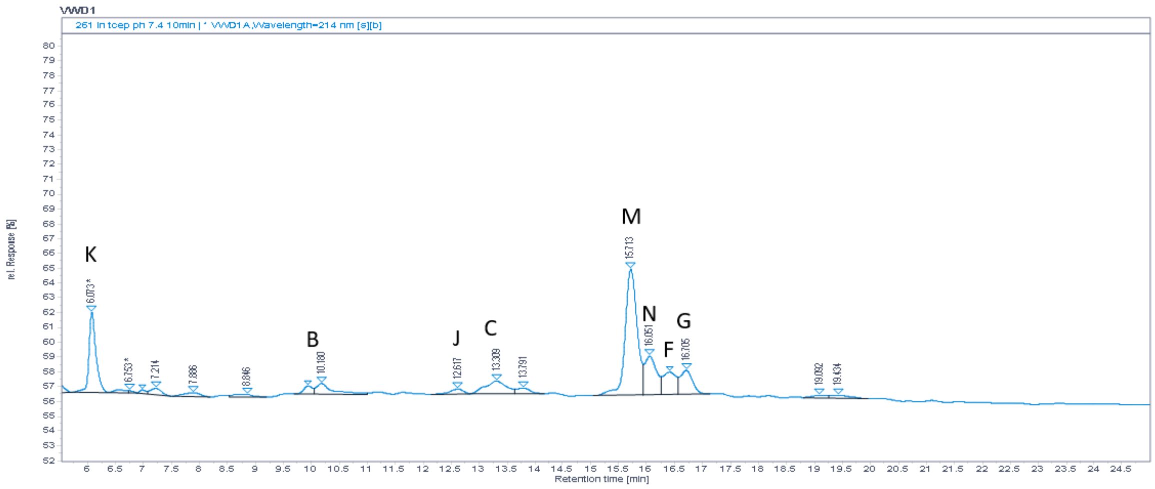

Figure K18: Analytical HPLC spectrum of photolysis reaction mixture following addition of TCEP. K= ketone, B=oxamate, J=sulfonic acid, C=geminal diol, F=oxazole, G=thiazole (formed through cyclocondensation of enethiol in acidic HPLC eluent), M=thiol, N=enethiolate.

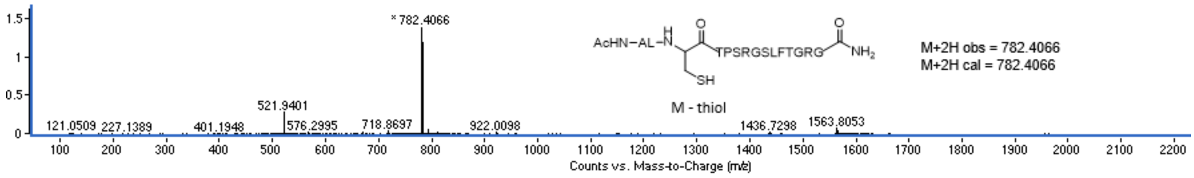

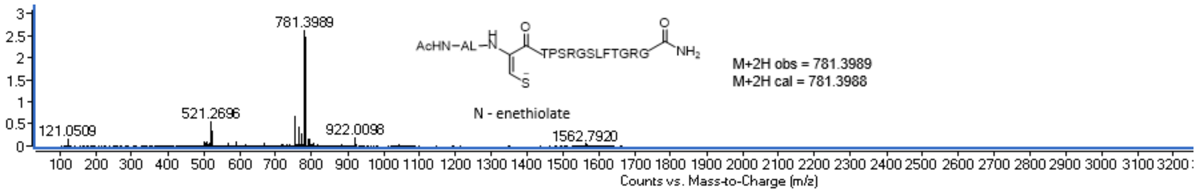

Figure K19: HRMS (ESI positive mode) spectrum after addition of TCEP to the photolysis reaction mixture. Observed and calculated m/z peaks are provided.

Treatment of photolysis reaction mixture with $NH_2OBn.HCl$

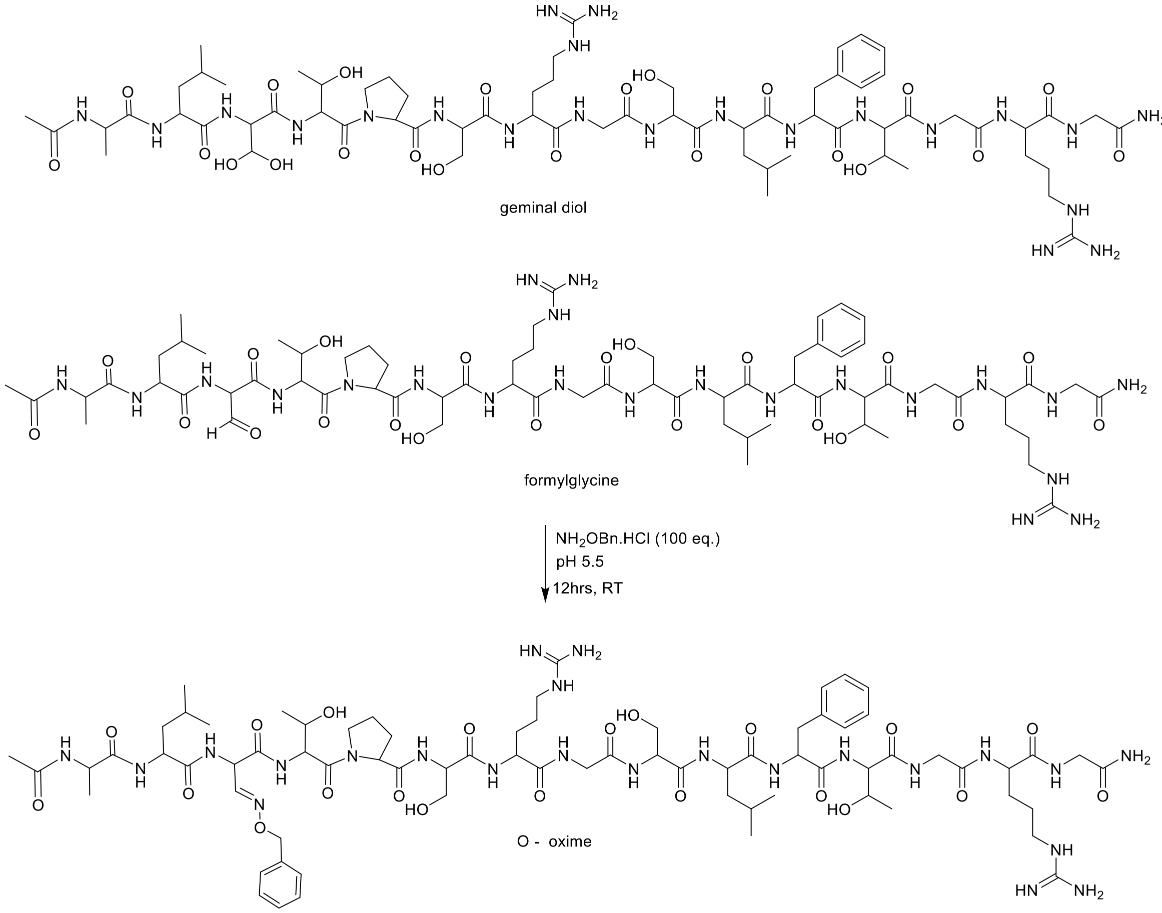

$NH_2OBn.HCl$ (2.7 mg, 100 eq.) was added to the photolysis reaction mixture (1 eq., adjusted to pH 5.5, 100 µL) and the reaction mixture was allowed to evolve overnight at room temperature. The reaction mixture was analysed by analytical HPLC and HRMS (ESI positive mode).

*Table K9: Analytical HPLC method used for characterisation of photolysis products (phenacylsulfide **32**, H_2O, 0.1 M phosphate, pH 7.4) after addition of $NH_2OBn.HCl$.*

**time (min)**	**A (%)**	**B (%)**
0	95	5
5	80	20
7	80	20
25	63	37
30	0	100
35	100	0

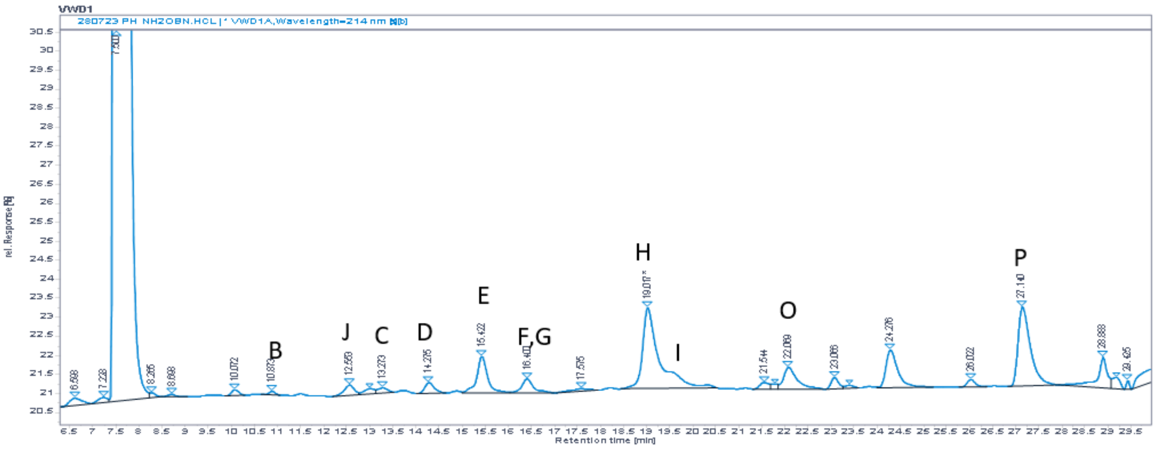

Figure K20: Analytical HPLC spectrum of photolysis reaction after treatment with $NH_2OBn.HCl$. B=oxamate, J=sulfonic acid, C=geminal diol, D=formylglycine, E=isothiazolone, F=oxazole, G=thiazole (formed through cyclocondensation of enethiol in acidic HPLC eluent), H=disulfide, I=enethiol-thiol disulfide, O =oxime, P=oxime conjugate of ketone. Peak at 24.27 min is impurity present in the $NH_2OBn.HCl$ solution.

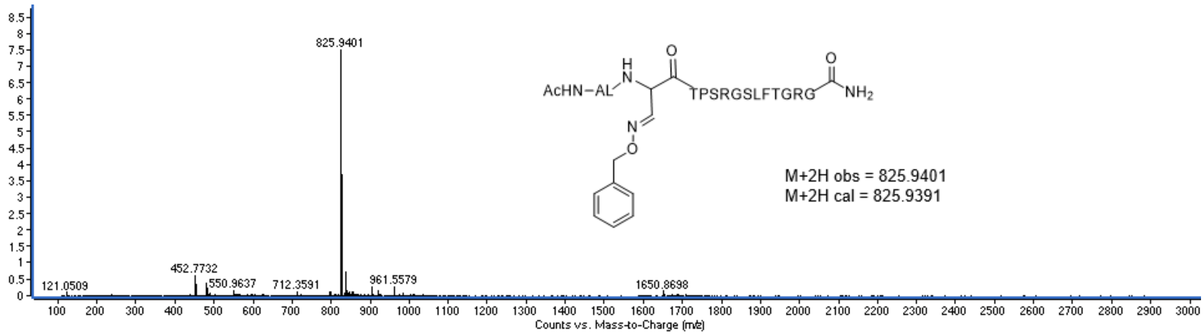

Figure K21: HRMS (ESI positive mode) spectrum of photolysis reaction mixture after treatment with $NH_2OBn.HCl$. Observed and calculated HRMS m/z peaks are provided.

Photolysis of phenacylsulfide *32* in buffered D_2O (0.1 M phosphate, pD 7.8)

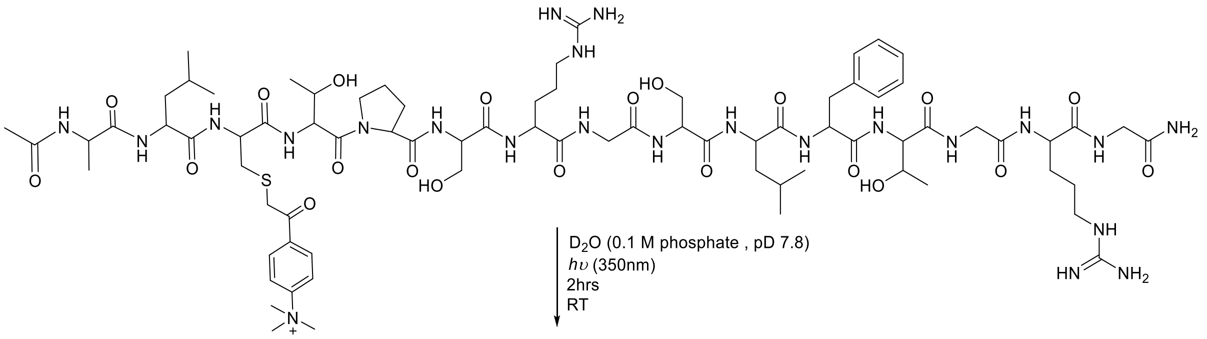

A solution of phenacylsulfide **32** (3 mg, 0.0017 mmol) in D_2O (0.1 M phosphate, pD 7.8, 300 µL, 5.7 mM) was degassed by passing N_2 gas through the solution for 20 mins. The solution was then illuminated at 350 nm in a Rayonet photoreactor for 2 hrs at room temperature. The photolysis reaction mixture was analysed by analytical HPLC, HRMS (ESI positive mode) and NMR spectroscopy .

*Table K10: Analytical HPLC method used for characterisation of photolysis products (phenacylsulfide **32**, D_2O, 0.1 M phosphate, pD 7.8)*

time (min)	A (%)	B (%)
0	95	5
5	80	20
7	80	20
25	63	37
30	0	100
35	100	0

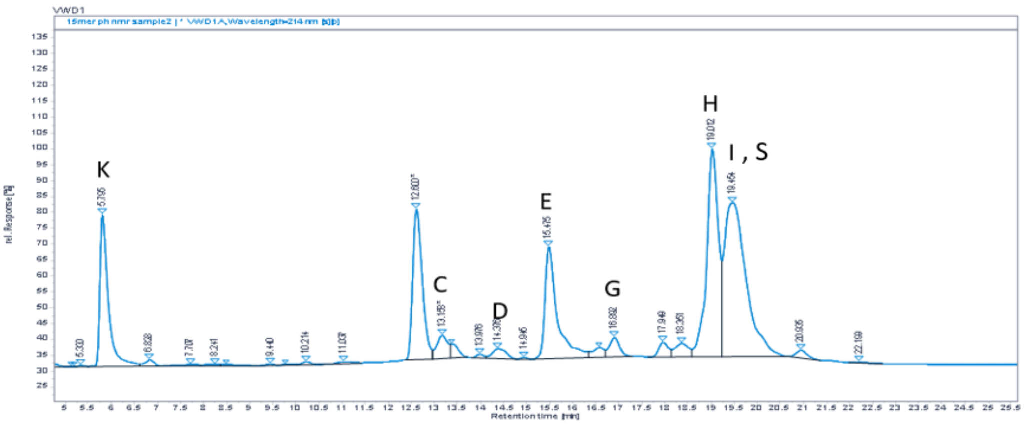

*Figure K22: Analytical HPLC spectrum of reaction mixture derived from photolysis of phenacylsulfide **32** in D_2O (0.1 M phosphate, pD 7.8). K=ketone, C=geminal diol, D=formylglycine, E=isothiazolone, G=thiazole (formed through cyclocondensation of enethiol in acidic HPLC eluent), H=disulfide, I=enethiol–thiol disulfide, S=dithioacetal–thiol disulfide. Peak at 12.60 min is an impurity present in starting material.*

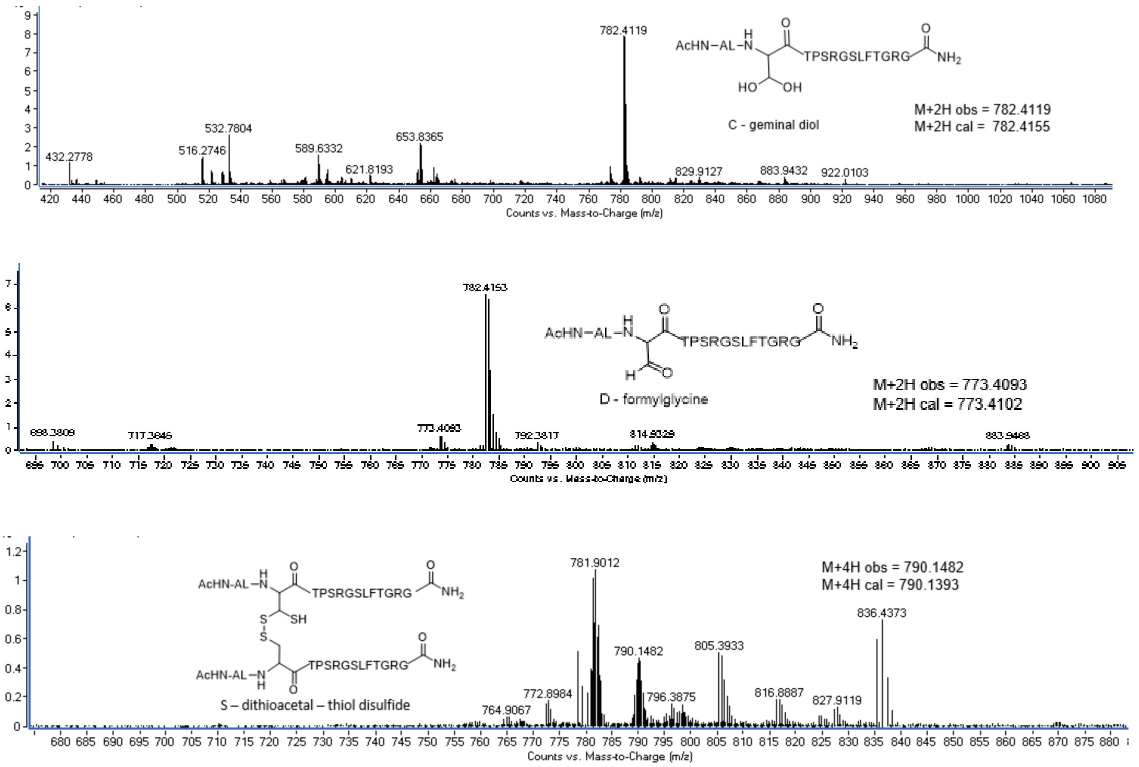

Figure K23: HRMS (ESI positive mode) spectrum of photolysis reaction mixture in buffered D_2O (0.1 M phosphate, pD 7.8) showing the m/z peaks of C-geminal diol, D-formylglycine and S-dithioacetal-thiol disulfide. Observed and calculated HRMS m/z peaks are provided.

Table K11: Approximate conversion values (%) of photolysis products (phenacylsulfide ***32****, D_2O, 0.1 M phosphate, pD 7.8) based on integration of HPLC peaks (detection at 214 nm). Approximate conversions from phenacylsulfide (Norrish type-II and β-scission processes, column 2) and from putative thioaldehyde (Norrish type-II processes only, column 3).*

**Product**	**% Area (Conversion from phenacylsulfide)**	**% Area (Conversion from putative thioaldehyde)**
geminal diol	2%	4%
formylglycine	2%	4%
isothiazolone	18%	39%
thiazole	2%	6%
enethiolate-thiol disulfide , dithioacetal-thiol disulfide	44%	47%
disulfide	32%	

^{1}H NMR analysis of phenacylsulfide *32*

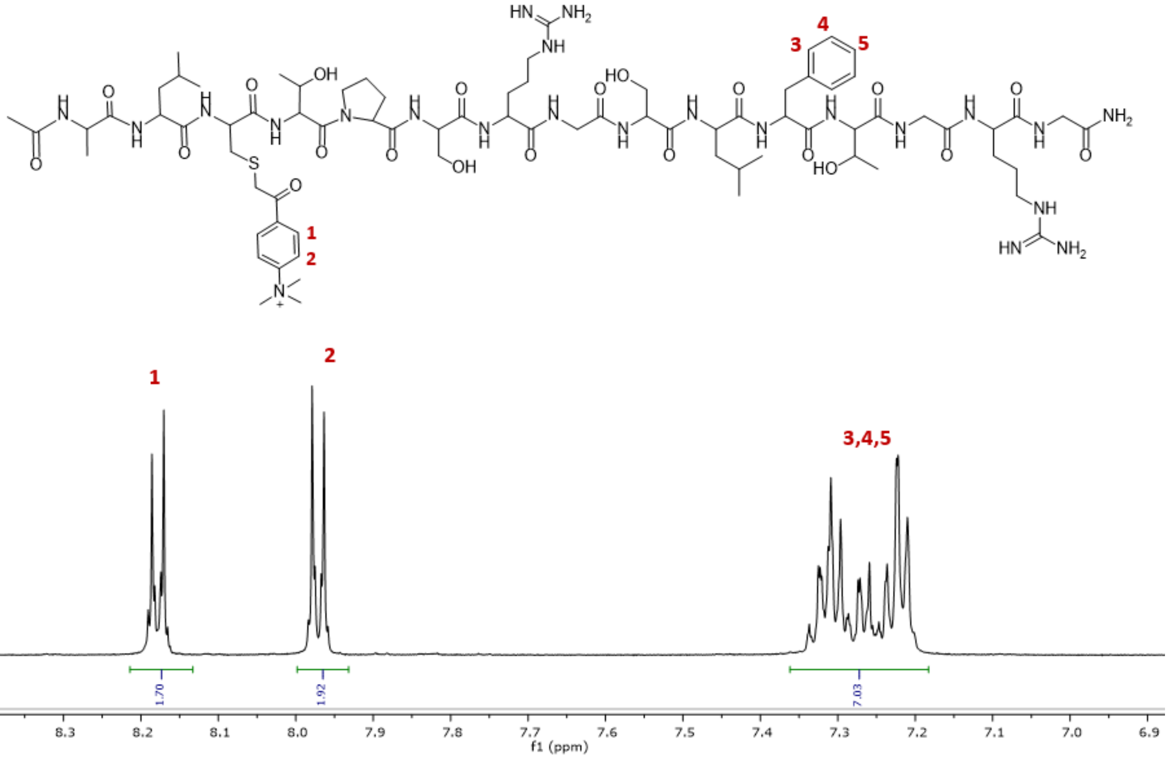

Figure K24: Zoomed in region of ^{1}H NMR spectrum of peptide phenacylsulfide ***32*** *in D_2O (0.1 M phosphate, pD 7.8) showing the characteristic peaks of the compound.*

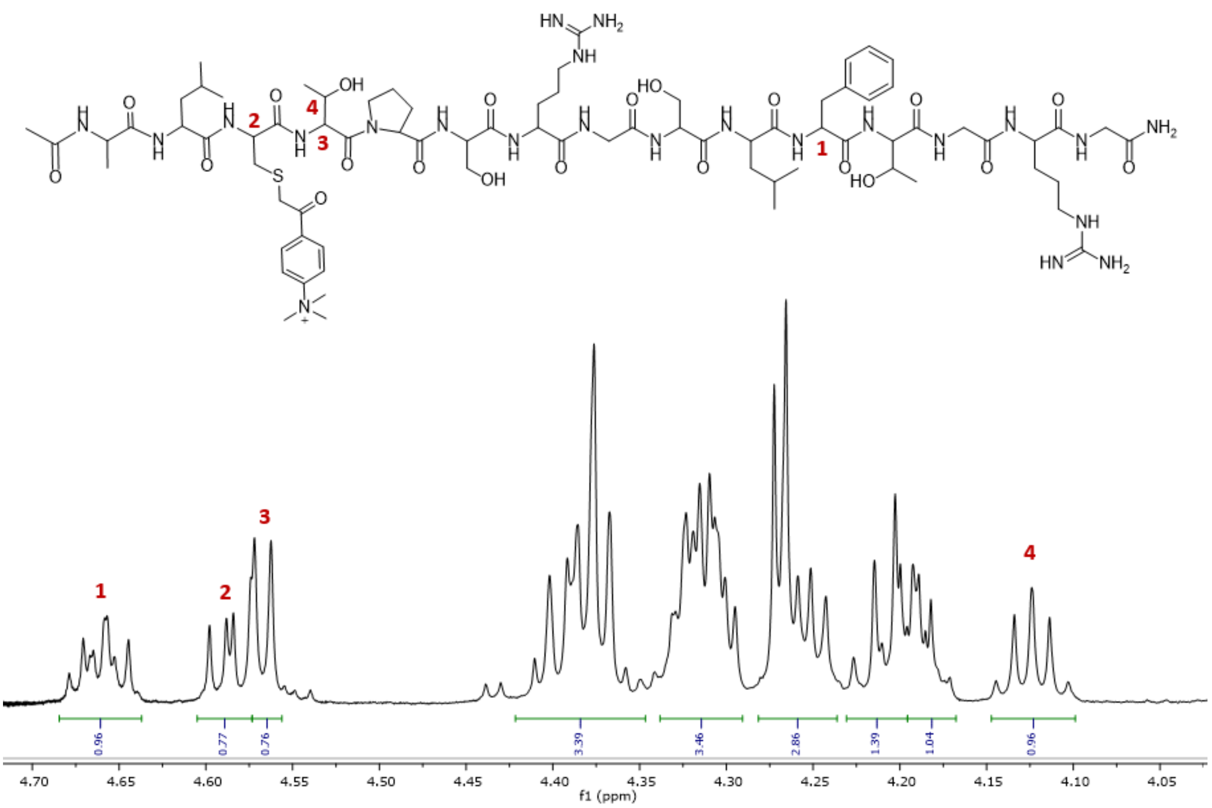

Figure K25: Zoomed in region of ^{1}H NMR spectrum of peptide phenacylsulfide ***32*** *in D_2O (0.1 M phosphate, pD 7.8) showing the characteristic peaks of the compound.*

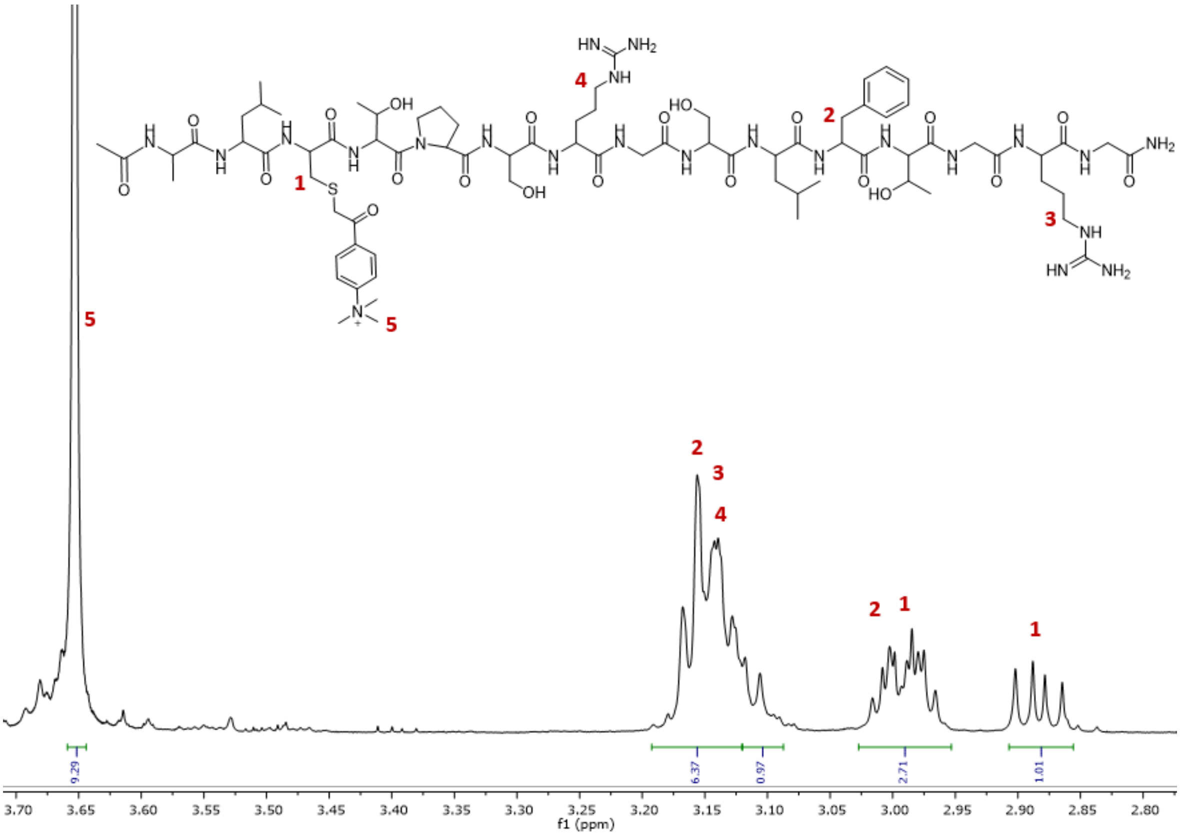

*Figure K26: Zoomed in region of ^{1}H NMR spectrum of peptide phenacylsulfide **32** in D_2O (0.1 M phosphate, pD 7.8) showing the characteristic peaks of the compound.*

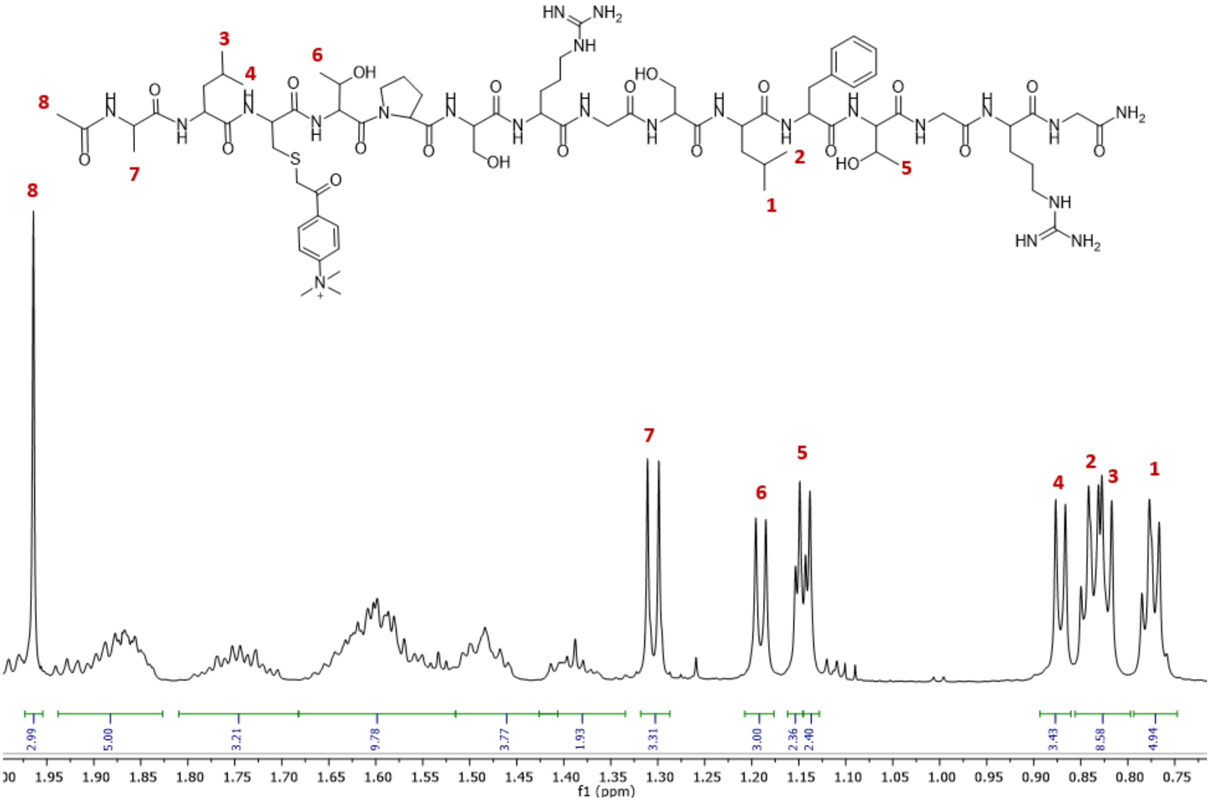

Figure K27: Zoomed in region of ^{1}H NMR spectrum of peptide phenacylsulfide ***32*** *in D_2O (0.1 M phosphate, pD 7.8) showing the characteristic peaks of the compound.*

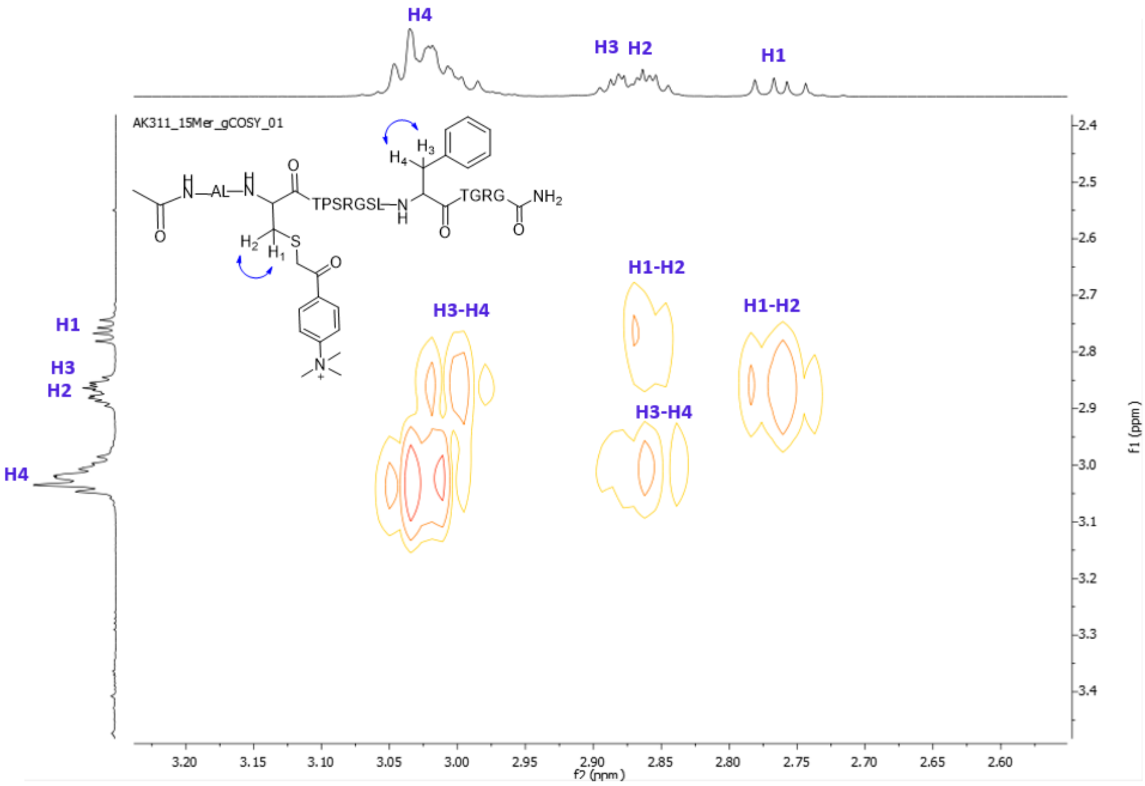

*Figure K28: Figure 26: Zoomed in region of COSY NMR spectrum of peptide phenacylsulfide **32** in D_2O (0.1 M phosphate, pD 7.8) showing the key H-H correlation between the diastereotopic methylene protons H_1, H_2 appearing as a doublet of doublet within the range 2.72–2.77, 2.83–2.87 ppm respectively. Note: The diastereotopic methylene protons H_3, H_4 is appearing as a doublet of doublet within the range 2.83–2.87,2.96–3.06 ppm respectively.*

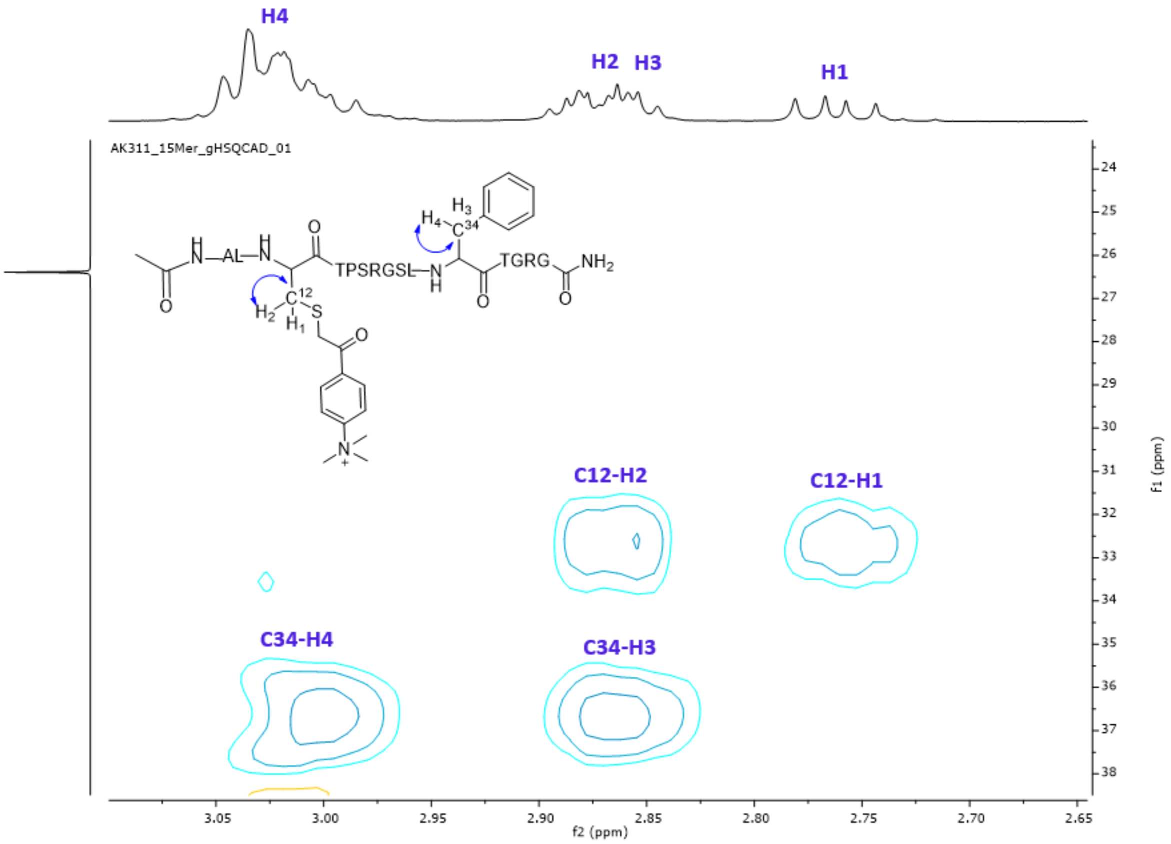

Figure K29: Zoomed in region of HSQC spectrum of peptide phenacylsulfide ***32*** *in D_2O (0.1 M phosphate, pD 7.8) showing the key C-H correlation between carbon C_{12} at 32.5 ppm to the methylene protons H_1, H_2 at 2.72–2.77, 2.83–2.87 ppm and C-H correlation between carbon C_{34} at 37 ppm to the methylene protons H_3,H_4 at 2.83–2.87,2.96–3.06 ppm respectively.*

^{1}H NMR analysis of reaction mixture derived from the photolysis of phenacylsulfide *32*

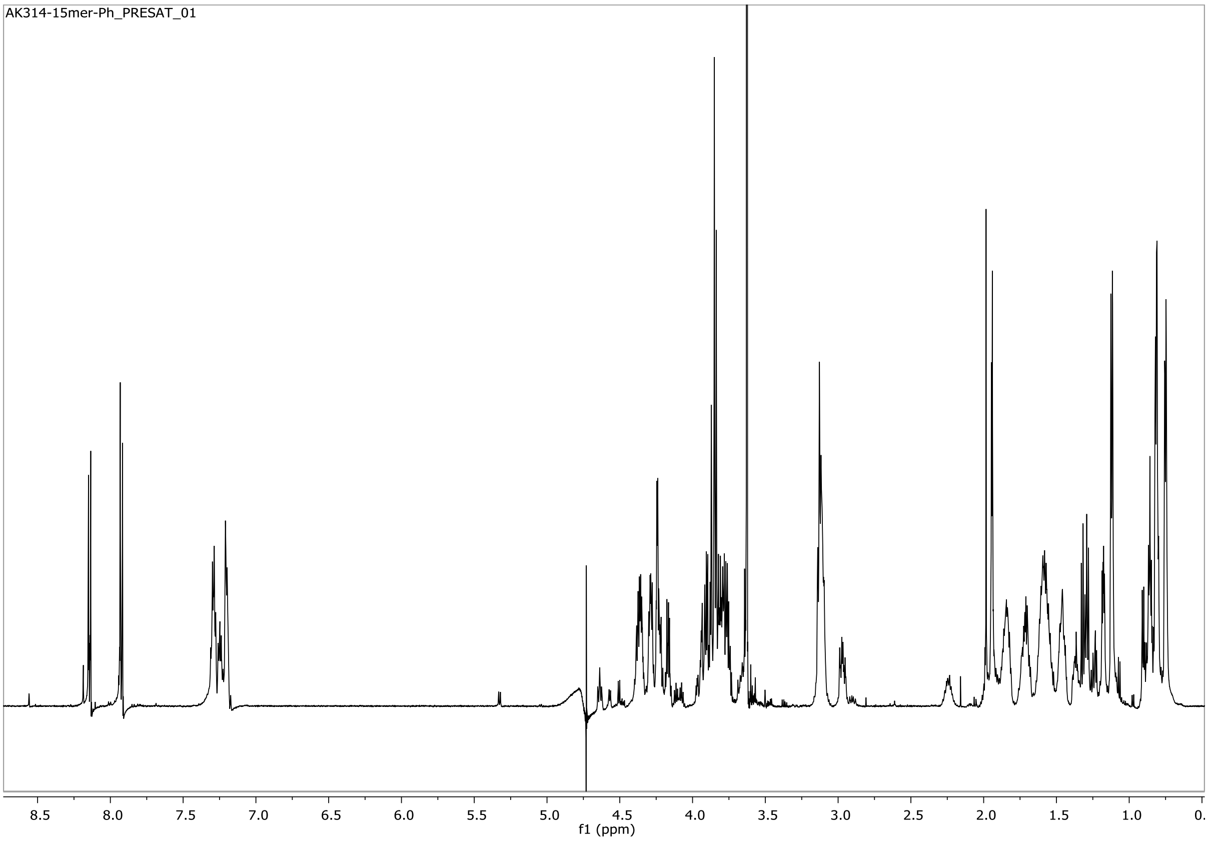

*Figure K30: ^{1}H NMR spectrum of the photolysis reaction mixture of phenacylsulfide **32** in D_2O (0.1 M phosphate, pD 7.8)*

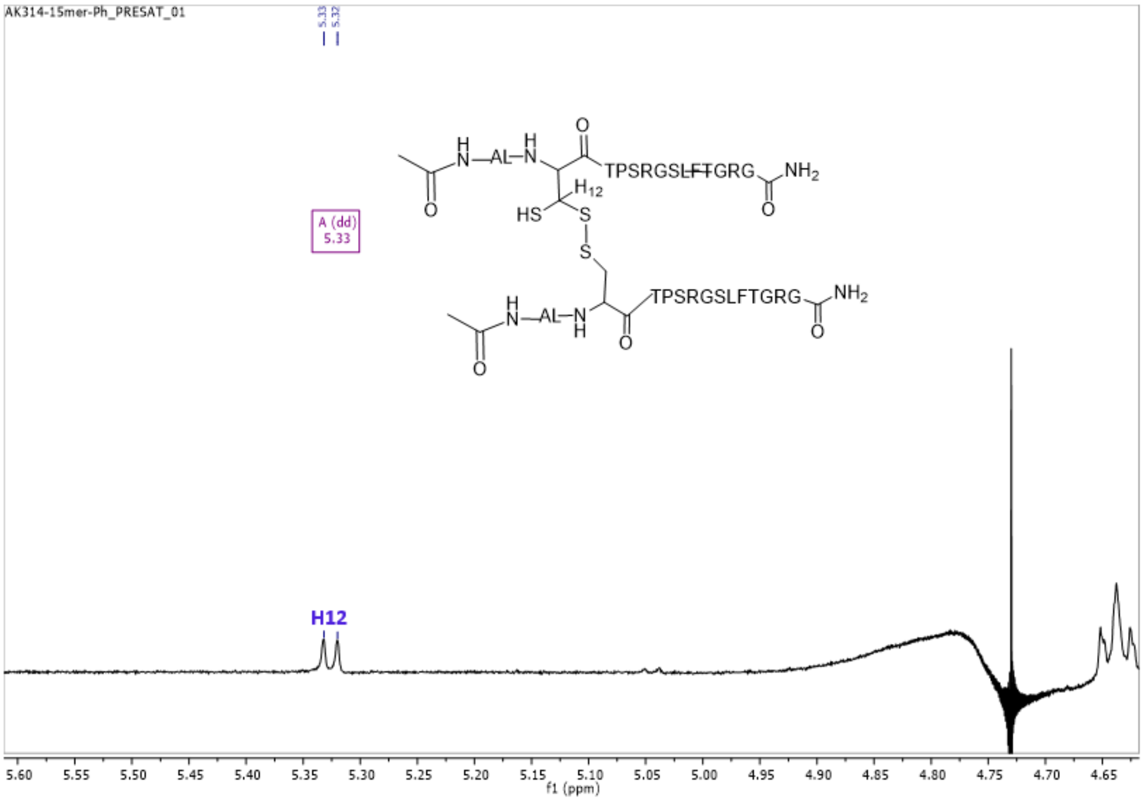

Figure K31: Zoomed in region of ^{1}H NMR spectrum of photolysis reaction mixture of phenacylsulfide ***32*** *in D_2O (0.1 M phosphate, pD 7.8) showing a doublet for the β proton H_{12} of dithioacetal-thiol disulfide at 5.33 ppm with a J value = 7.6 Hz.*

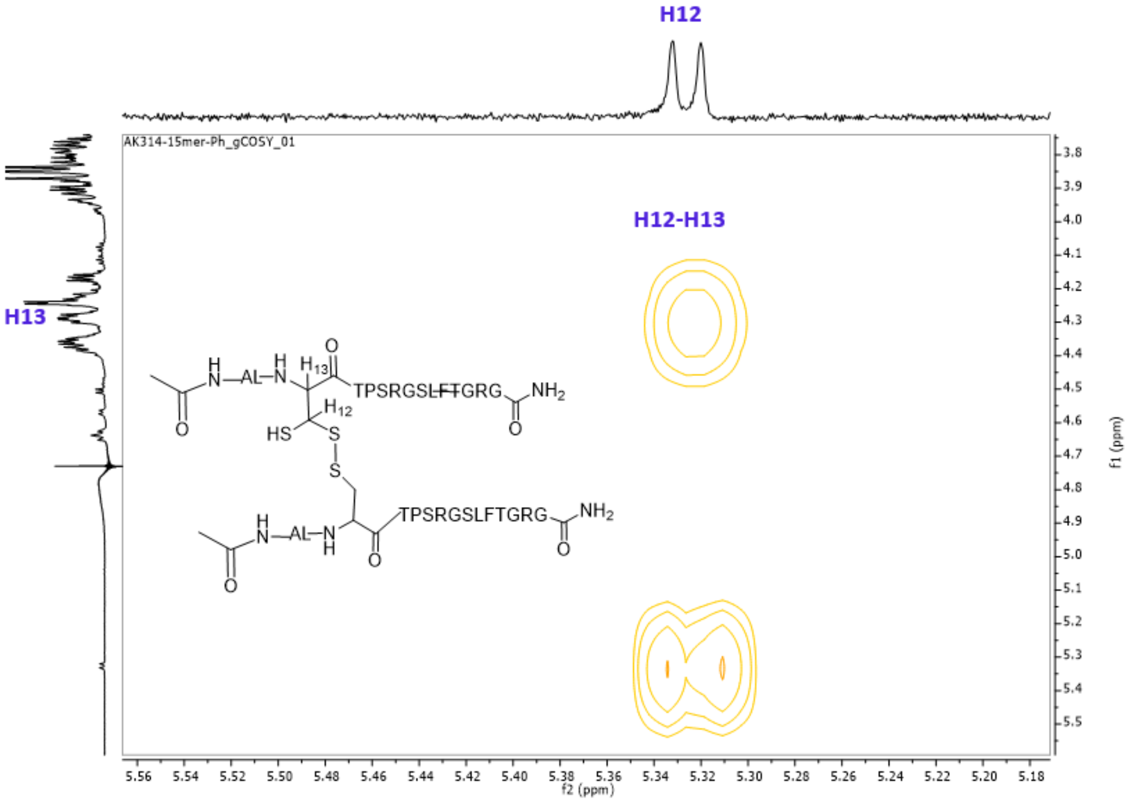

*Figure K32: Zoomed in region of COSY spectrum of photolysis reaction mixture of phenacylsulfide **32** in D_2O (0.1 M phosphate, pD 7.8) showing a H-H correlation between β proton H_{12} at 5.33 ppm and α proton H_{13} at 4.29 ppm of dithioacetal-thiol disulfide.*

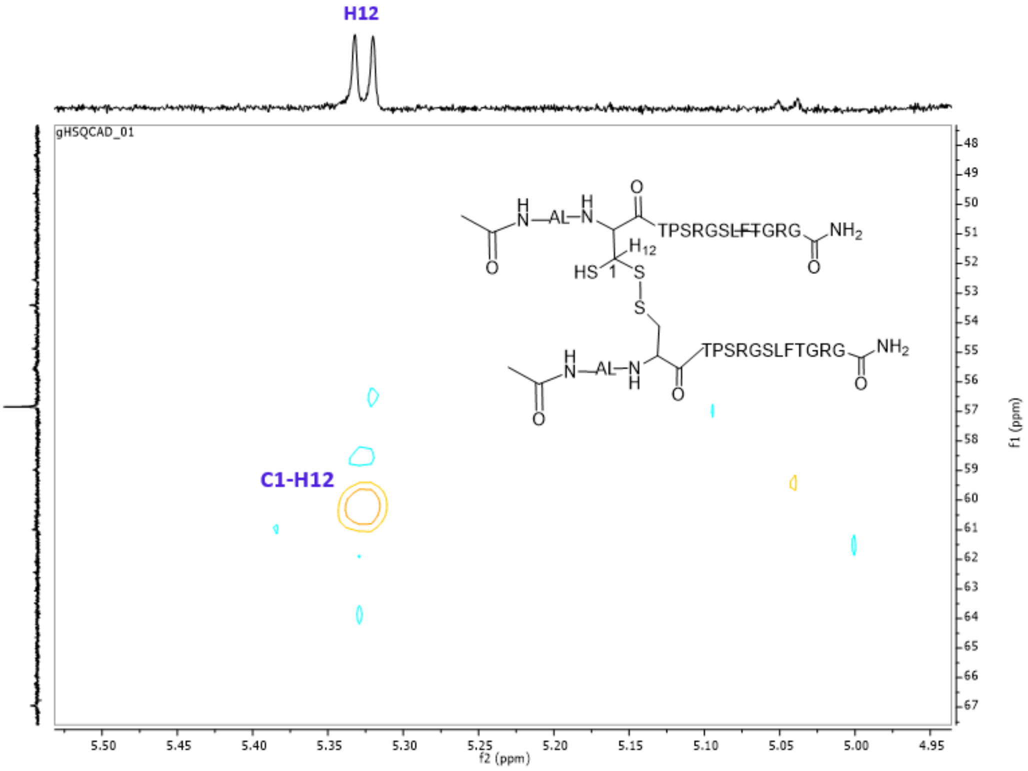

Figure K33: Zoomed in HSQC spectrum of photolysis reaction mixture of phenacylsulfide ***32*** *in D_2O (0.1 M phosphate, pD 7.8) showing a correlation between β proton H_{12} at 5.33 ppm and β carbon C_1 at 60 ppm of dithioacetal-thiol disulfide.*

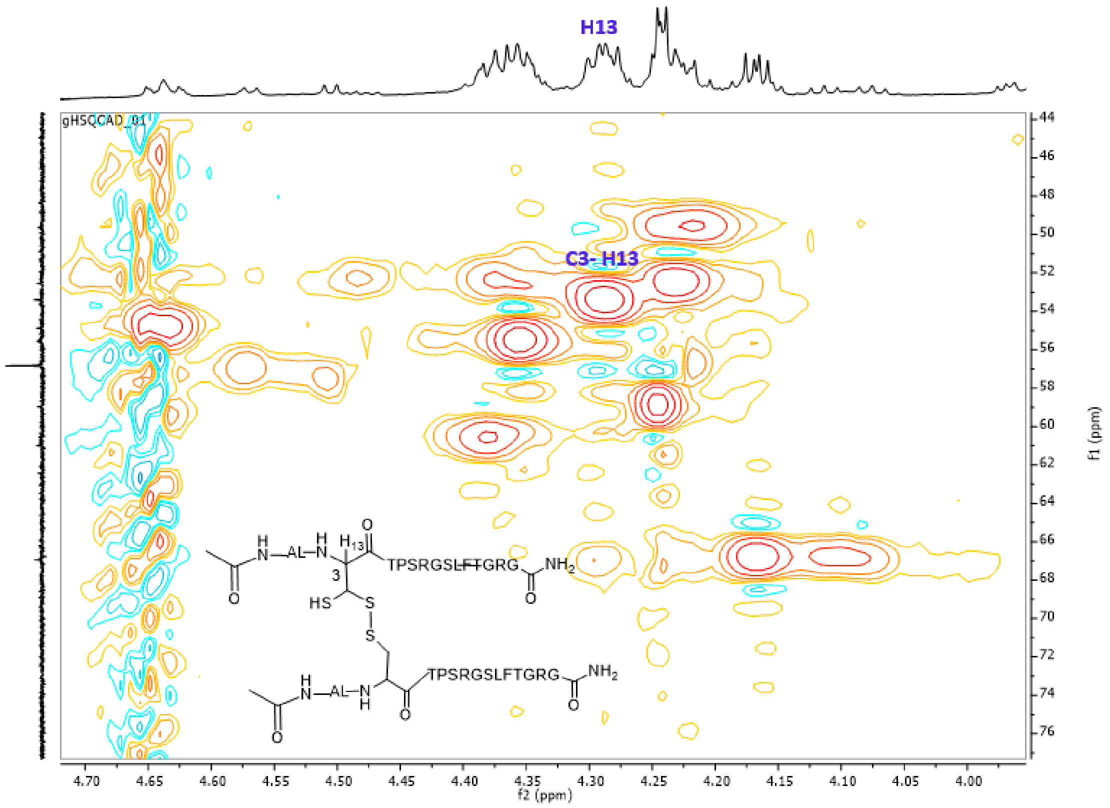

*Figure K34: Zoomed in region of HSQC spectrum of photolysis reaction mixture of phenacylsulfide **32** in D_2O (0.1 M phosphate, pD 7.8) showing a correlation between α proton H_{13} at 4.28 ppm and α carbon C_3 at 53 ppm of dithioacetal-thiol disulfide.*

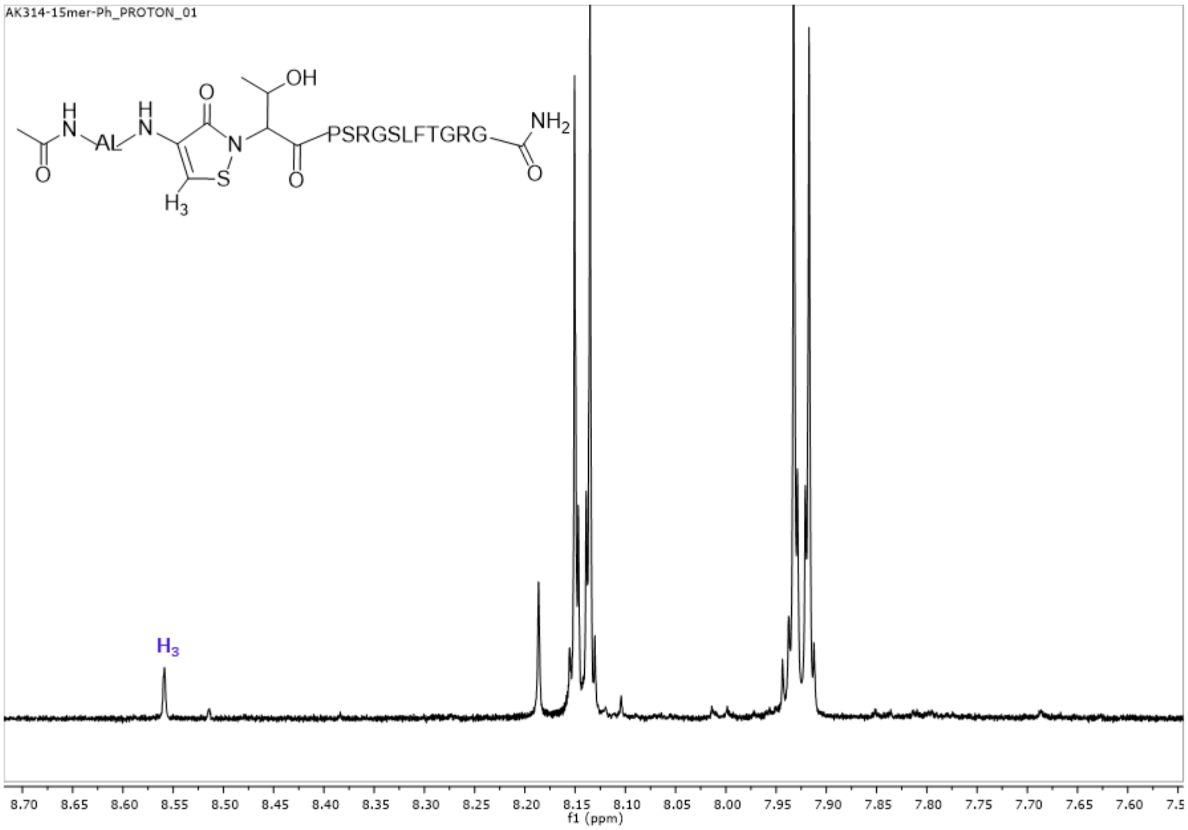

Figure K35: Zoomed in region of ^{1}*H NMR spectrum of photolysis reaction mixture of phenacylsulfide* ***32*** *in* D_2O *(0.1 M phosphate, pD 7.8) showing a singlet for the methine proton* H_3 *of isothiazolone at 8.55 ppm.*

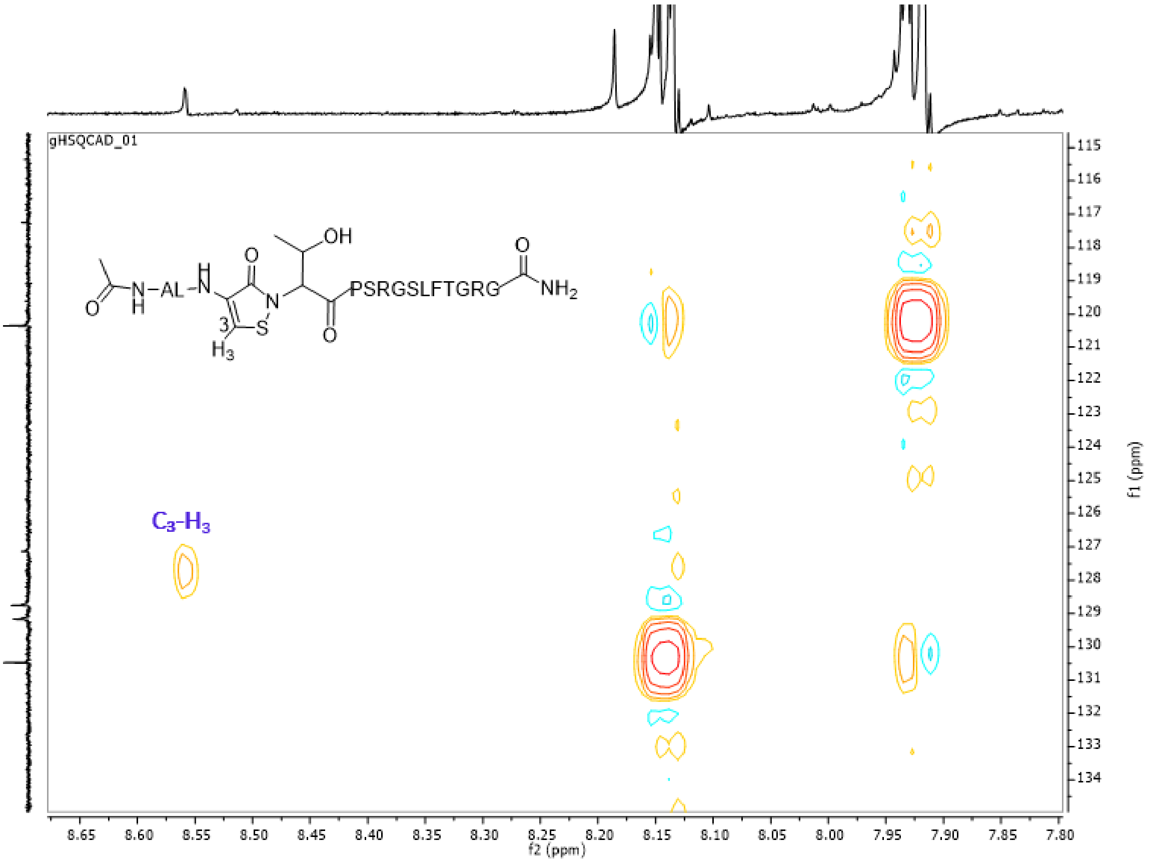

*Figure K36: Zoomed in region of HSQC spectrum of photolysis reaction mixture of phenacylsulfide **32** in D_2O (0.1 M phosphate, pD 7.8) showing the C-H correlation between the methine proton H_3 of the isothiazolone at 8.55 ppm and β carbon C_3 at 127.5 ppm.*

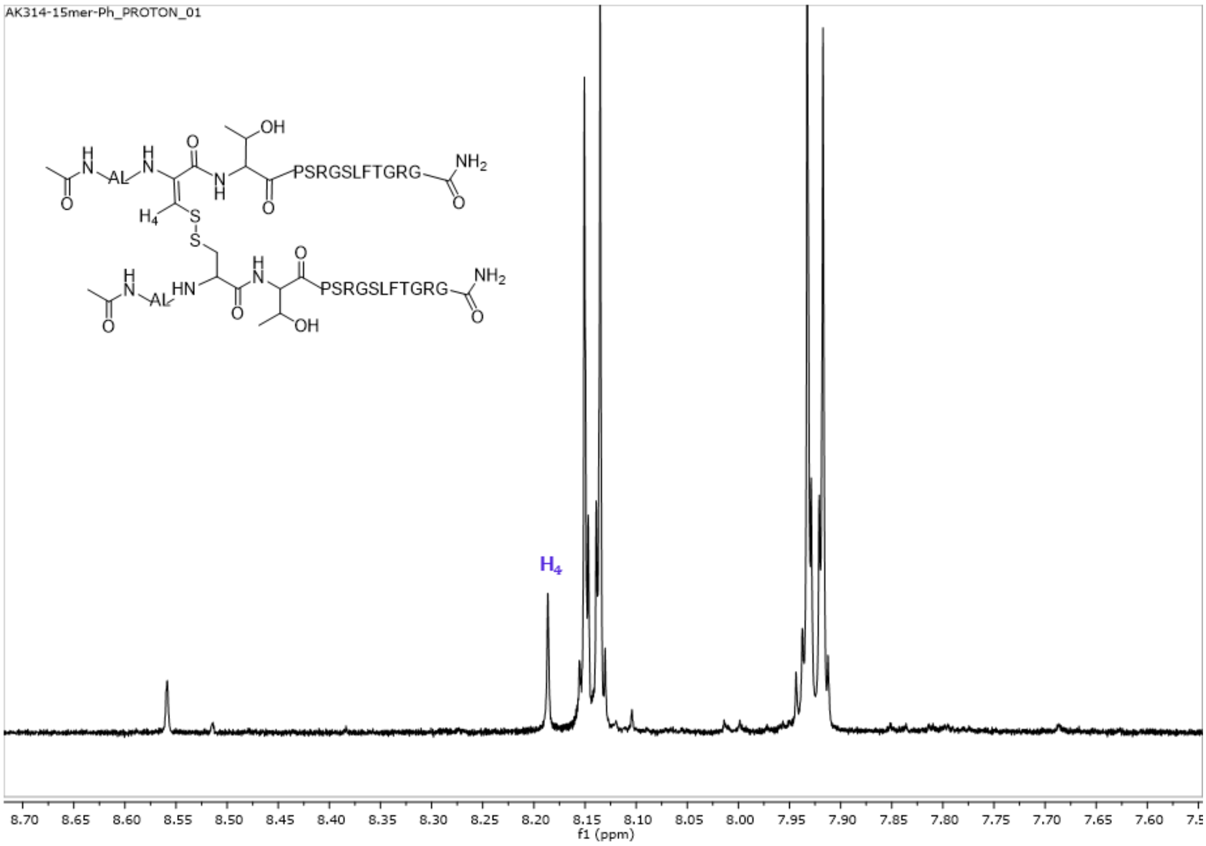

*Figure K37: Zoomed in region of ^{1}H NMR spectrum of photolysis reaction mixture of phenacylsulfide **32** in D_2O (0.1 M phosphate, pD 7.8) showing a singlet for the methine proton H_4 of enethiolate–thiol disulfide at 8.18 ppm.*

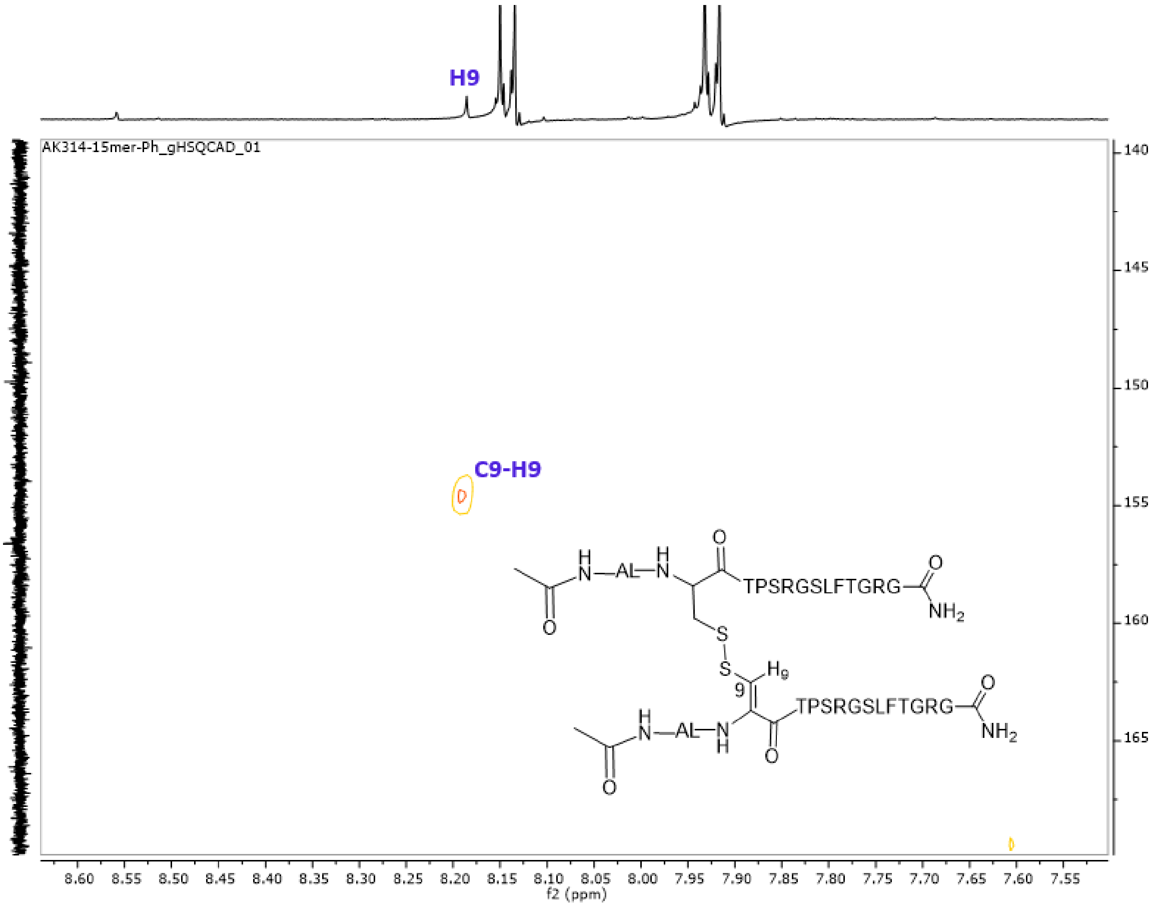

*Figure K38: Zoomed in region of HSQC spectrum of photolysis reaction mixture of phenacylsulfide **32** in D_2O (0.1 M phosphate, pD 7.8) showing the C-H correlation of the enethiol–thiol disulfide. The methine proton H_9 of enethiol–thiol disulfide at 8.18 ppm correlates to the β carbon C_9 at 153 ppm.*

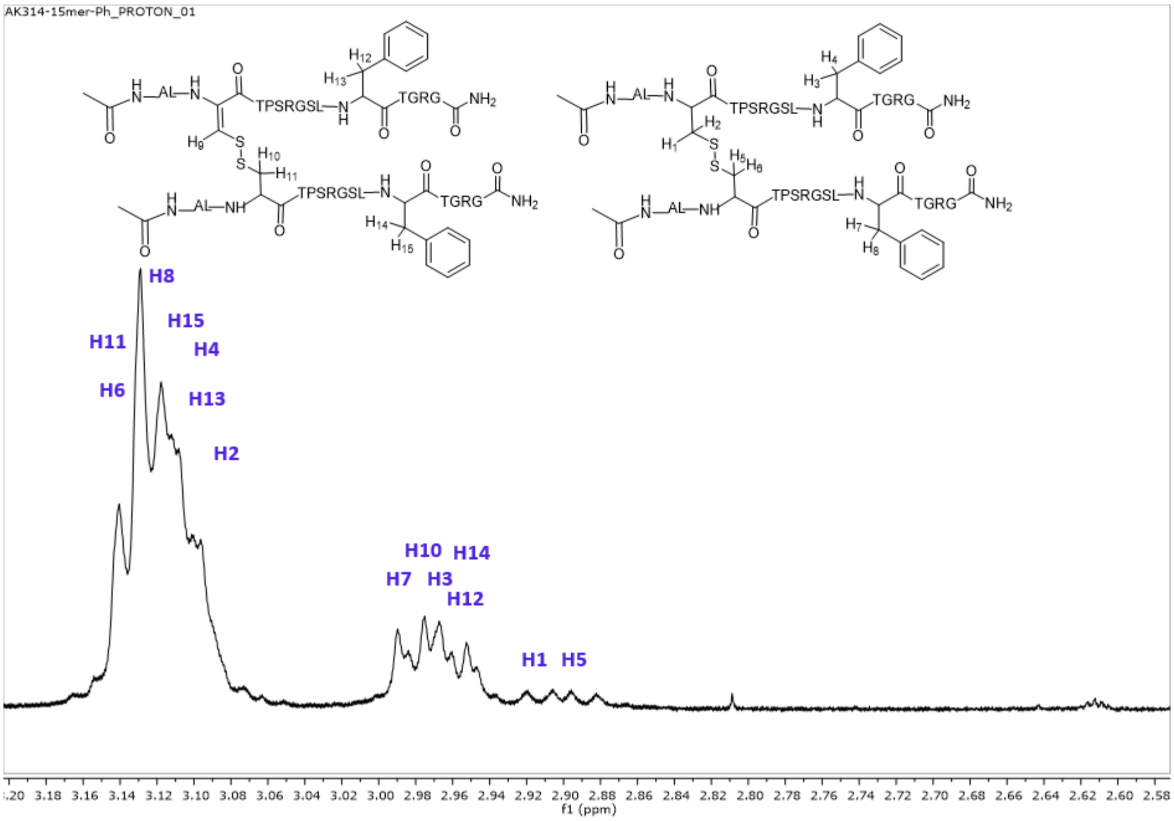

*Figure K39: Zoomed in region of ^{1}H NMR spectrum of photolysis reaction mixture of phenacylsulfide **32** in D_2O (0.1 M phosphate, pD 7.8) showing a doublet of doublet for the diastereotopic methylene protons of disulfide and enethiol–thiol disulfide. Note: One set of diastereotopic protons H_1, H_5 of disulfide appears as a doublet of doublet from 2.87 to 2.92 ppm. The second set of diastereotopic protons H_2, H_6 appears in the range 3.14–3.08 ppm. The γ methylene hydrogens of arginine residue is also present in the range 3.08–3.14 ppm. The diastereotopic protons H_{10}, H_{11} of enethiol-thiol disulfide are obscured by the benzyl hydrogens of phenylalanine from 2.94–3.00 ppm and γ methylene hydrogens of arginine from 3.08–3.14 ppm.*

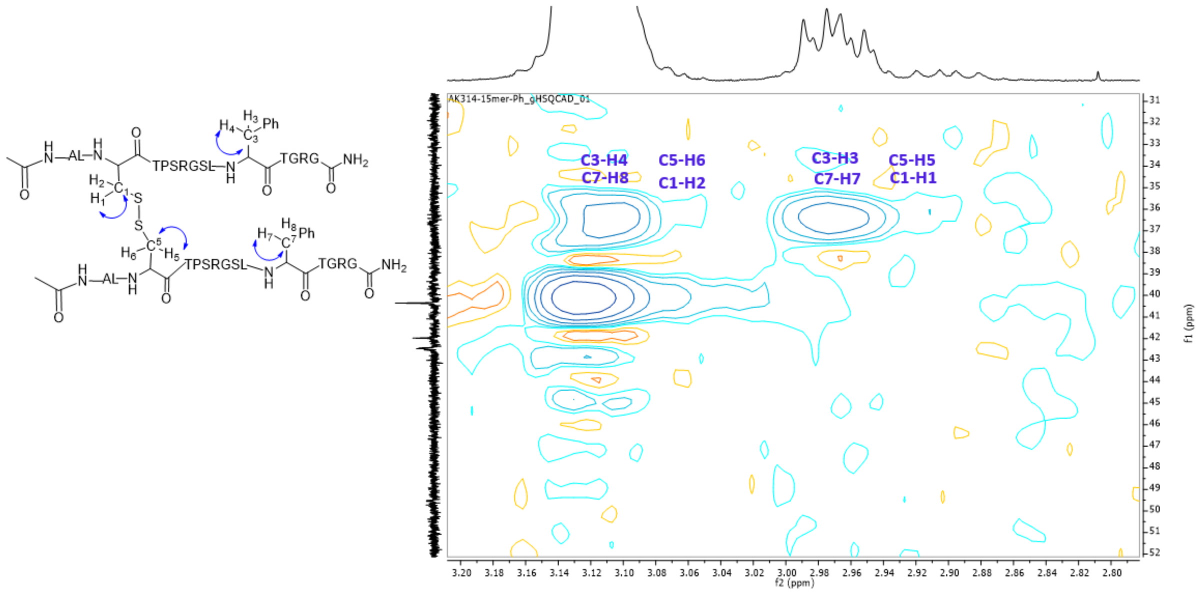

*Figure K40: Zoomed in region of HSQC spectrum of photolysis reaction mixture of phenacylsulfide **32** in D_2O (0.1 M phosphate, pD 7.8) showing the C-H correlations of disulfide. Diastereotopic methylene protons H_1, H_2, and H_5, H_6 of disulfide correlates to the carbons C_1 and C_5 at 36 ppm respectively.*

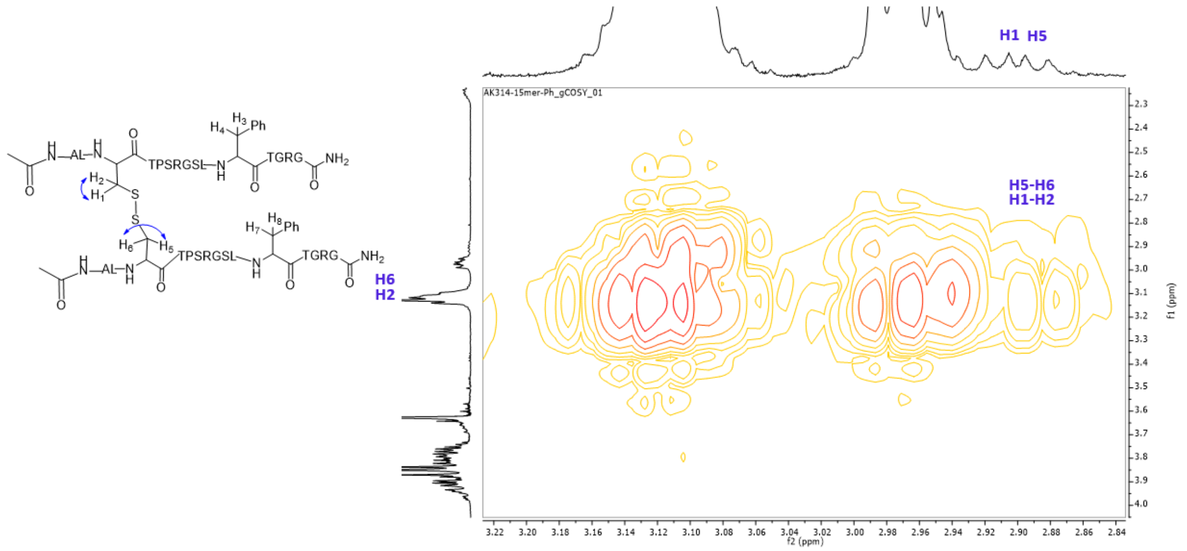

*Figure K41: Zoomed in region of COSY spectrum of phenacylsulfide **32** in D_2O (0.1 M phosphate, pD 7.8) showing the H-H correlations of disulfide. Diastereotopic methylene protons H_1, H_5 appearing within the range 2.87 to 2.92 ppm shows correlation to H_2, H_6 appearing within the range 3.14–3.08 ppm.*

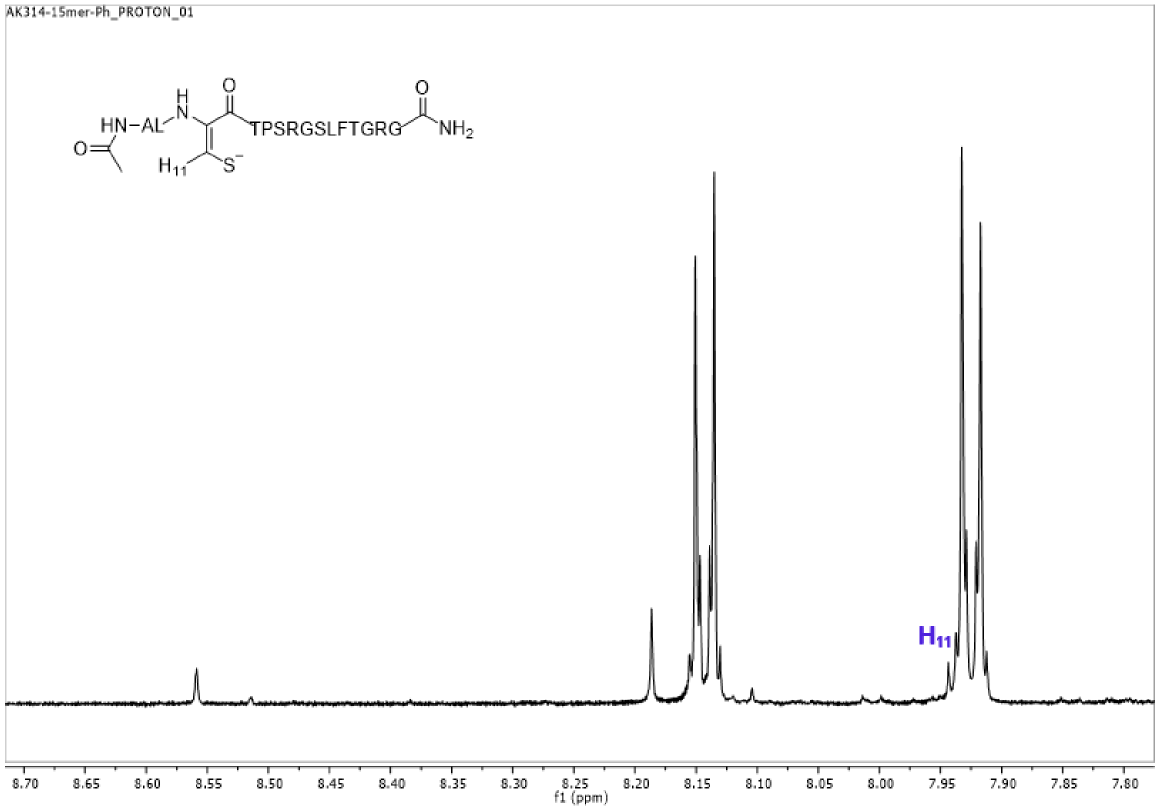

*Figure K42: Zoomed in region of ^{1}H NMR spectrum of photolysis reaction mixture of phenacylsulfide **32** in D_2O (0.1 M phosphate, pD 7.8) showing a singlet for the methine proton H_{11} of (E)-enethiolate.*

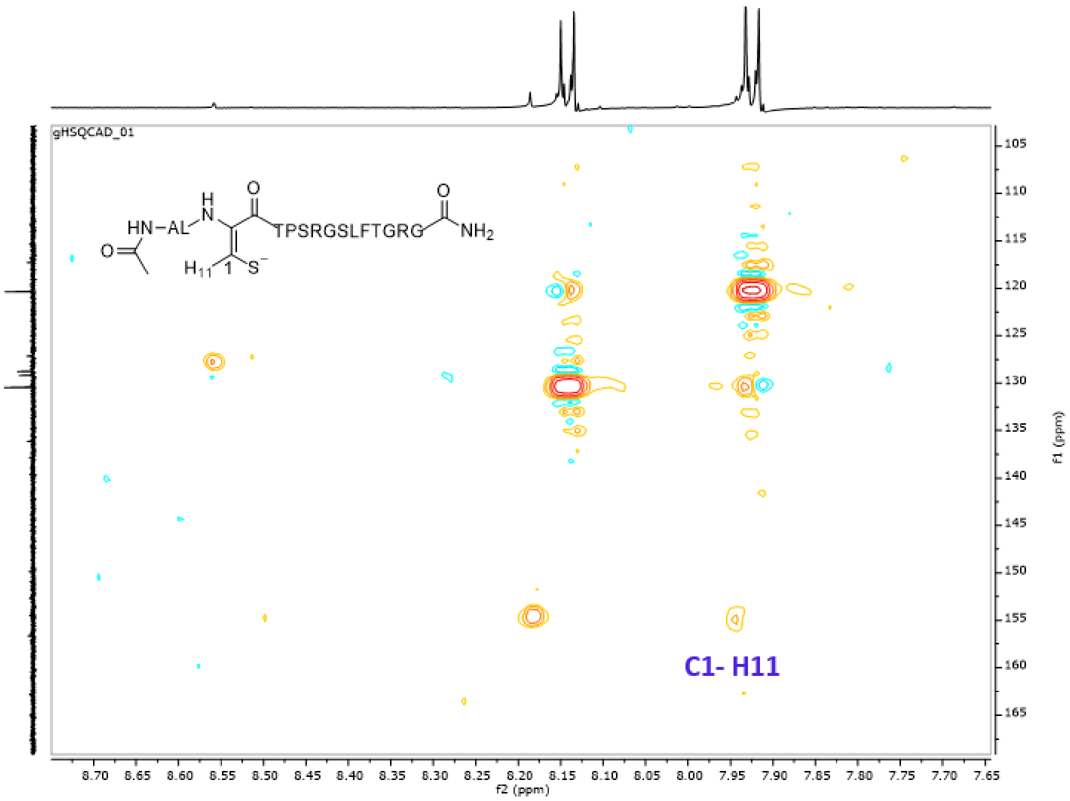

*Figure K43: Zoomed in region of HSQC spectrum of photolysis reaction mixture phenacylsulfide **32** in D_2O (0.1 M phosphate, pD 7.8) showing C-H correlations between the methine proton H_{11} at 8.18 ppm and β carbon C_1 at 153 ppm of (E)-enethiolate.*

*Table K12: Conversion values (%) of photolysis products (phenacylsulfide **32**, D_2O, 0.1 M phosphate, pD 7.8) based on the integration of methyl protons of threonine residue in the NMR spectra. Approximate conversions from phenacylsulfide (Norrish type-II and β-scission processes, column 2) and from putative thioaldehyde (Norrish type-II processes only, column 3). Note: the integration of a methine CH signal for the dithioacetal–thiol disulfide, enethiol-thiol disulfide, isothiazolone and (E)-enethiolate are calculated relative to the methyl protons of the threonine residue of respective molecules appearing in the region 1.14–1.27 ppm.*

**product**	**% Area (Conversion from phenacylsulfide)**	**% Area (Conversion from putative thioaldehyde)**
isothiazolone	14%	29%
(*E*)-enethiolate	9%	19%
enethiolate-thiol disulfide	31%	33%
dithioacetal-thiol disulfide	18%	19%
disulfide	28%	

Stability of geminal diol/formylglycine

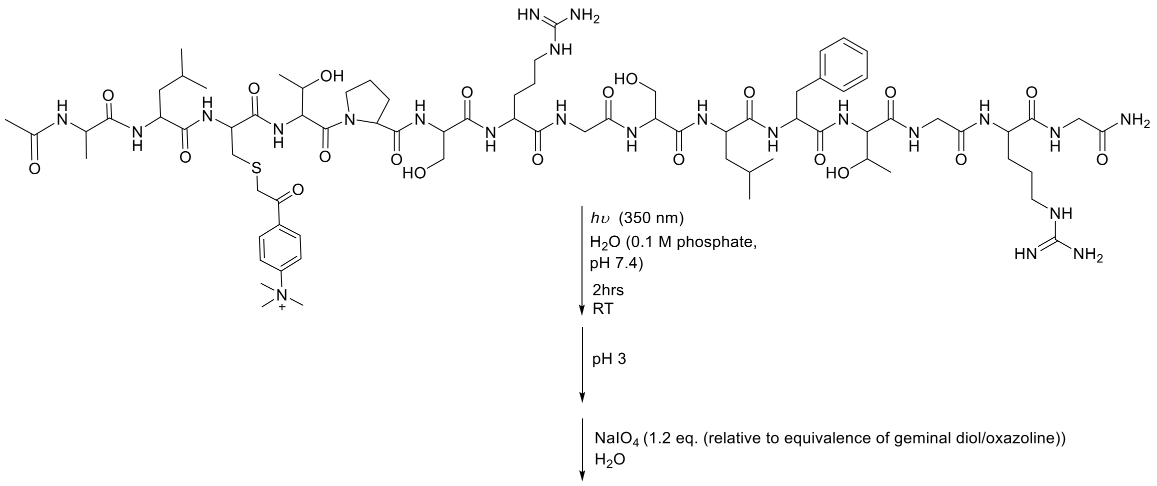

A solution of phenacylsulfide ***32*** (1.5 mg, 0.00085 mmol) in H_2O (0.1 M phosphate buffer, pH 7.4, 200 µL, 4.3 mM solution) was degassed with N_2 for 20 mins. The solution was illuminated at 350 nm in a Rayonet photoreactor for 2 hours. Aqueous HCl (0.5 M, 100 µL) was added dropwise to the photolysis reaction mixture to reduce the pH of the solution to 3. The reaction mixture was analysed by LC-HRMS (ESI positive mode) and showed that the *gem*-diol was stable. $NaIO_4$ (1.2 eq.) was added to this reaction mixture. LC-HRMS (ESI positive mode) analysis after 2 days confirmed oxidative cleavage of the *gem*-diol/formylglycine into an oxamate and a primary amide.

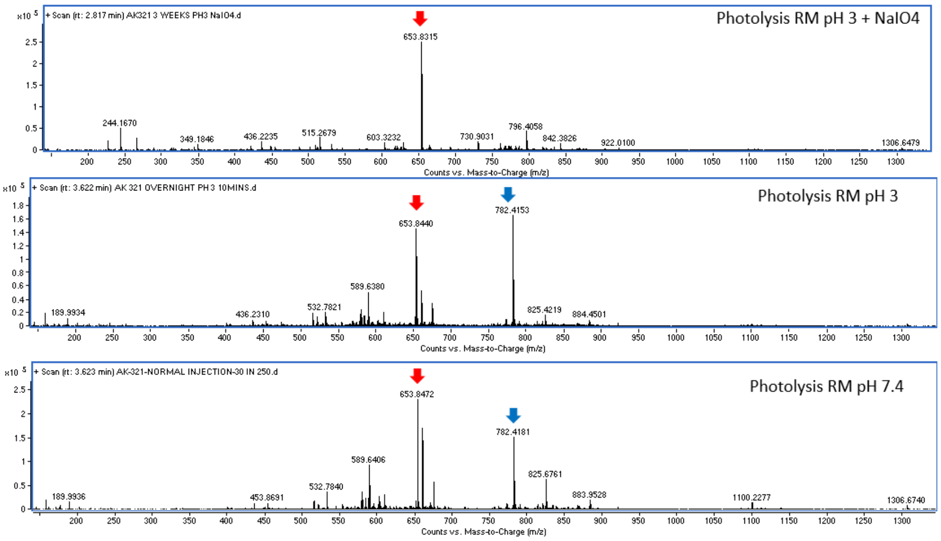

*Figure K44: HRMS (ESI positive mode) spectra of photolysis reaction mixture derived from phenacylsulfide **32** showing the evolution of geminal diol with changes to the reaction conditions. The spectra show m/z ion peaks for (a) oxamate, (M/2+H), calcd.653.8285, observed 653.8472; (b) geminal diol, (M/2+H), calcd.782.4155, observed 782.4181. (Note: The intensity of peaks are changing owing to dilution through the addition of 0.5M HCl and $NaIO_4$).*

Photolysis in the presence of guanidinium hydrochloride

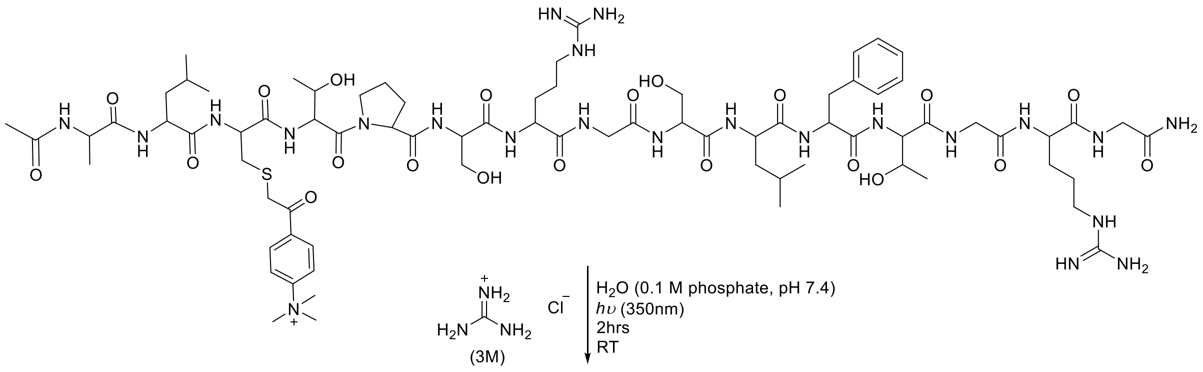

Guanidinium hydrochloride (287 mg, 3 mmol) was added to a solution of phenacylsulfide ***32*** (3 mg, 0.0017 mmol) in H_2O (0.1 M phosphate, pH 7.4, 1 mL, 1.7 mM solution). The solution was then degassed with N_2 for 20 mins and illumination at 350 nm in a Rayonet photoreactor for 2 hours. The reaction mixture was analysed by analytical HPLC and HRMS (ESI positive mode).

*Table K13: Analytical HPLC method used for characterisation of photolysis products (phenacylsulfide **32**, H_2O, 0.1 M phosphate, pH 7.4) in the presence of guanidinium hydrochloride.*

time (min)	A (%)	B (%)
0	95	5
5	80	20
7	80	20
25	63	37
30	0	100
35	100	0

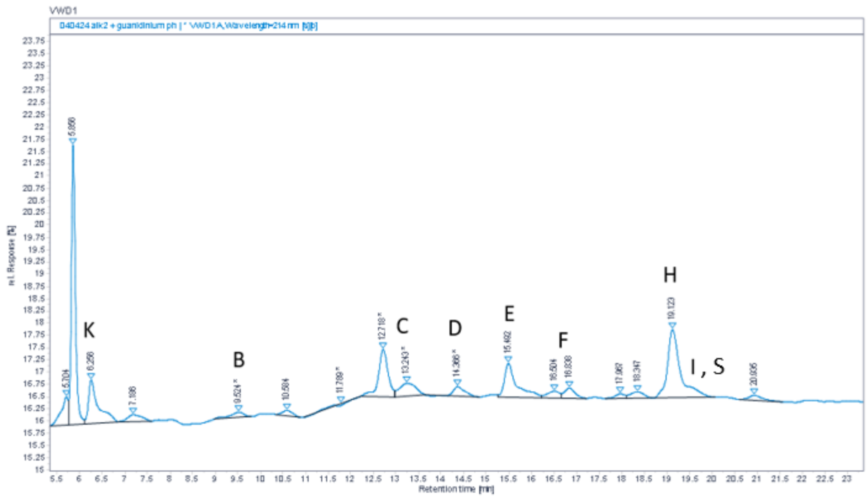

*Figure K45: HPLC spectrum of reaction mixture derived from the photolysis of phenacylsulfide **32** (H_2O, 0.1 M phosphate, pH 7.4) in the presence of guanidinium hydrochloride (3M). K=ketone, B=oxamate, C=geminal diol, D=formylglycine, E=isothiazolone, F=oxazole, H=disulfide, I=enethiol–thiol disulfide, S=dithioacetal-thiol disulfide. Peak at 12.7 min is an impurity present in starting material.*

Table K14: Approximate conversion values (%) of photolysis products (phenacylsulfide ***32****, H_2O, 0.1 M phosphate, 3M Gn.HCl, pH 7.4) based on integration of HPLC peaks (detection at 214 nm). Approximate conversions from phenacylsulfide (Norrish type-II and β-scission processes, column 2) and from putative thioaldehyde (Norrish type-II processes only, column 3).*

**products**	**% Area (Conversion from phenacylsulfide)**	**% Area (Conversion from putative thioaldehyde)**
oxamate	4%	9%
geminal diol	11%	23%
formylglycine	5%	10%
isothiazolone	20%	41%
oxazole	4 %	10%
enethiol – thiol disulfide, dithioacetal – thiol disulfide	7%	7%
disulfide	49 %	

Effect of TFE co-solvent on photolysis product distribution

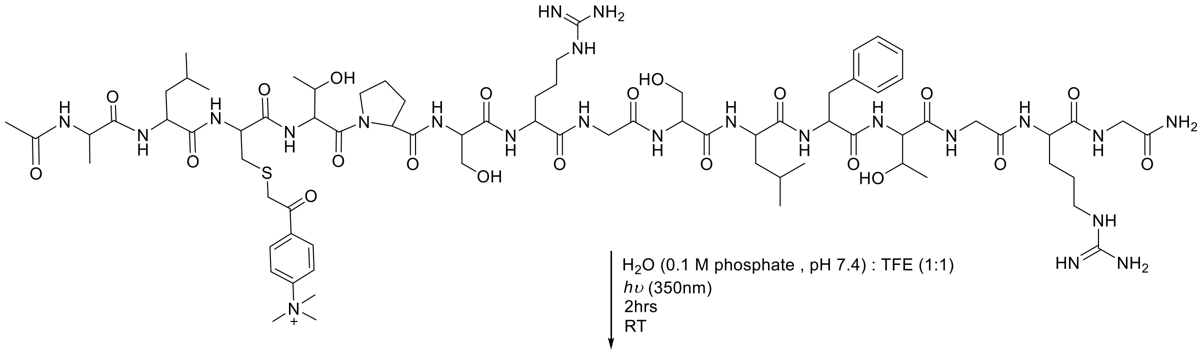

A solution of phenacylsulfide ***32*** (3 mg, 0.0017 mmol) in H_2O (0.1 M phosphate, pH 7.4)/TFE, (1:1, 1.5 mL) was degassed with N_2 for 20 mins. The solution was illuminated at 350 nm in a Rayonet photoreactor for 2 hours. The reaction mixture was analysed by analytical HPLC and HRMS (ESI positive mode).

*Table K15: Analytical HPLC method used for characterisation of photolysis products (phenacylsulfide **32**, H_2O (0.1 M phosphate, pH 7.4)/TFE (1:1)*

**time (min)**	**A (%)**	**B (%)**
0	95	5
5	80	20
7	80	20
25	63	37
30	0	100
35	100	0

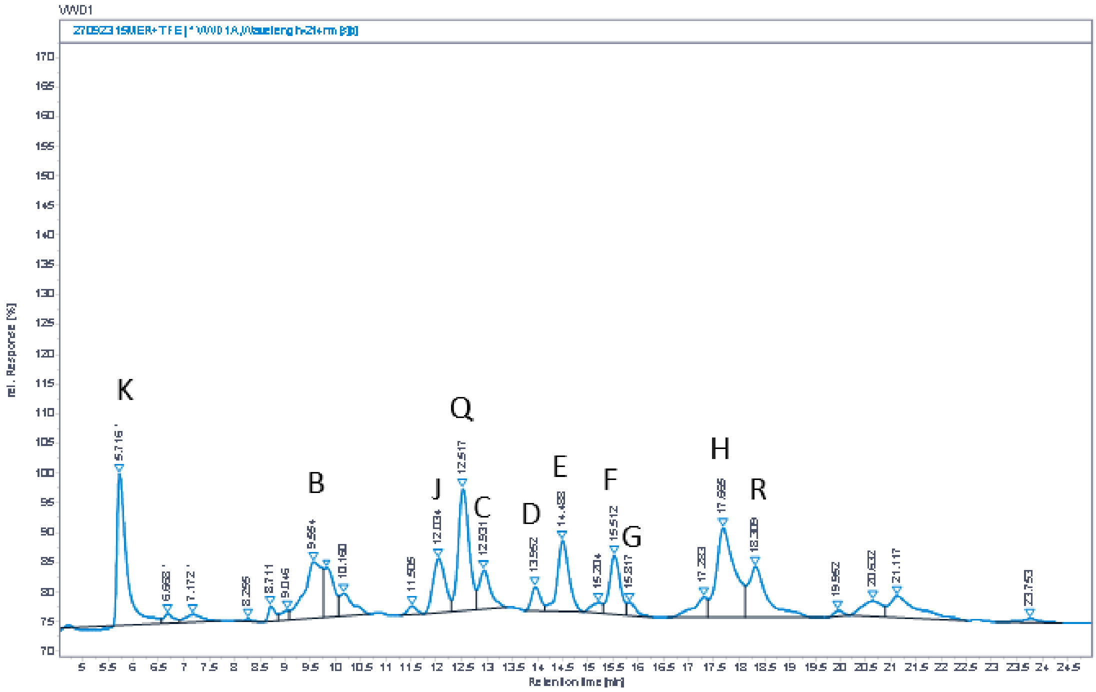

Figure K46: Analytical HPLC spectrum of reaction mixture derived from the photolysis of phenacylsulfide ***32*** *in H_2O (0.1M phosphate, pH 7.4)/TFE solvent (1:1). K=ketone, B=oxamate, J=sulphonic acid, Q=ene-sulfinic acid, C=geminal diol, D=formylglycine, E=isothiazolone, F=oxazole, G=thiazole, H=disulfide, R=trifluoroethyl enol ether.*

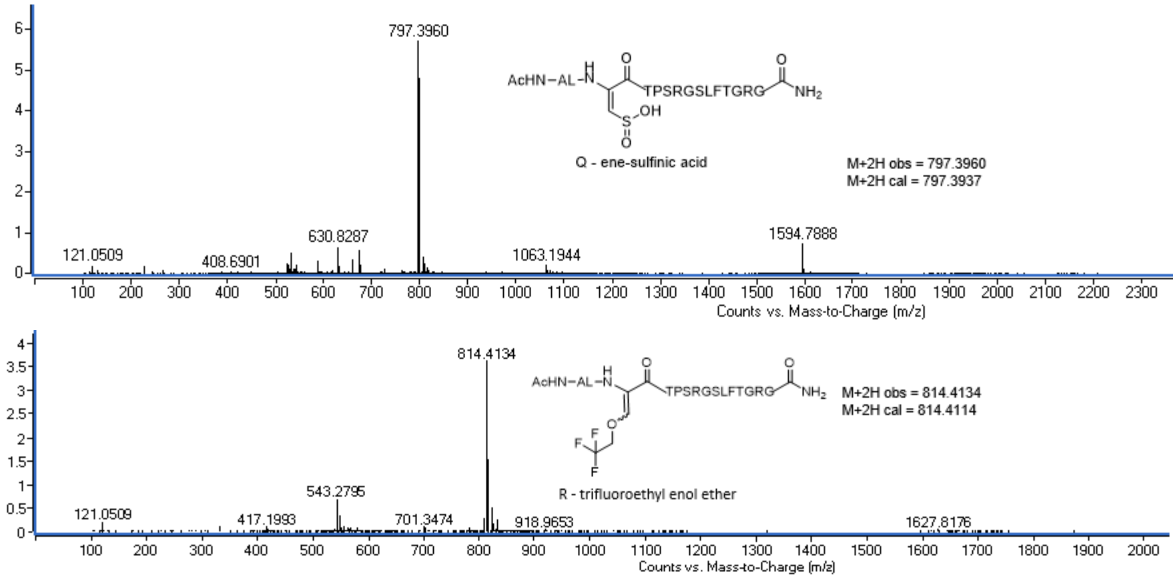

Figure K47: HRMS (ESI positive mode) spectra of reaction mixture derived from the photolysis of phenacylsulfide ***32*** *in H_2O (0.1M phosphate, pH 7.4)/TFE solvent (1:1). Observed and calculated ion peak values for each products are provided.*

*Table K16: Approximate conversion values (%) of photolysis products (phenacylsulfide **32**, H_2O (0.1M phosphate, pH 7.4)/TFE solvent, 1:1) based on integration of HPLC peaks (detection at 214 nm). Approximate conversions from phenacylsulfide (Norrish type-II and β-scission processes, column 2) and from putative thioaldehyde (Norrish type-II processes only, column 3).*

**product**	**% Area (Conversion from phenacylsulfide)**	**% Area (Conversion from putative thioaldehyde)**
oxamate	20%	29%
ene-sulfinic acid	15%	21%
geminal diol	5%	7%
formylglycine	2%	3%
isothiazolone	10%	13%
thiazole	1%	2%
oxazole	7%	10%
disulfide	22%	
sulfonic acid	7%	
trifluoroethyl enol ether	11%	15%

Reaction in non-buffered H_2O (initial pH 10)

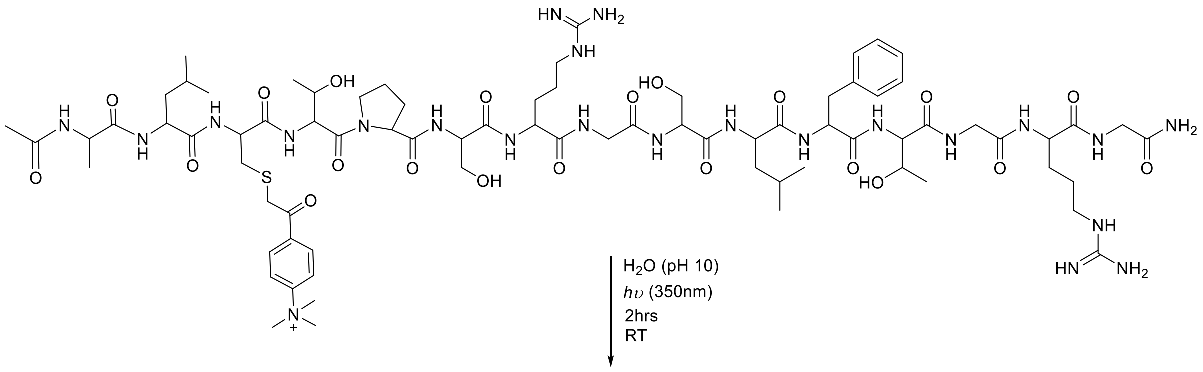

A solution of phenacylsulfide ***32*** (3 mg, 0.0017 mmol) in H_2O (1 mL, 1.7 mM solution, pH 3) was adjusted to pH 10 by using 0.25 M NaOH solution. The solution was degassed by passing N_2 gas through the solution for 20 mins. The solution was then illuminated at 350 nm in a Rayonet photoreactor for 2 hrs at room temperature. The pH of the reaction mixture after photolysis was measured to be 9.6. The reaction mixture was analysed by analytical HPLC and HRMS (ESI positive mode).

*Table K17: Analytical HPLC method used for characterisation of photolysis products (phenacylsulfide **32**, H_2O, initial pH 10)*

**time (min)**	**A (%)**	**B (%)**
0	95	5
5	80	20
7	80	20
25	63	37
30	0	100
35	100	0

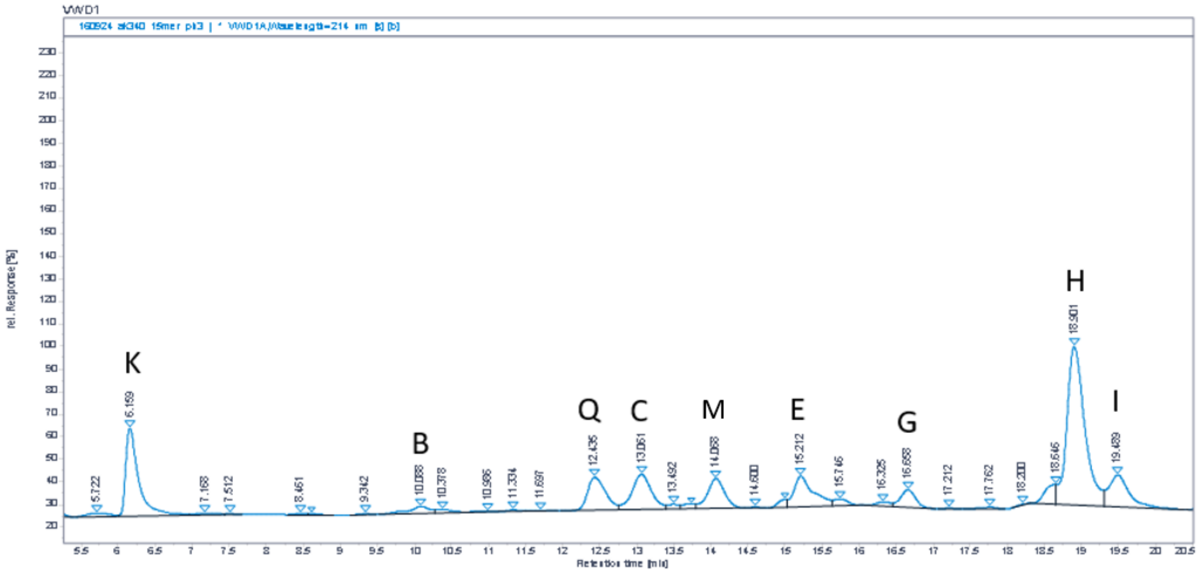

Figure K48: Analytical HPLC spectrum of reaction mixture derived from the photolysis of phenacylsulfide ***32*** *in H_2O (initial pH 10, final pH 9.6). K=ketone, B=oxamate, Q=ene-sulfinic acid, C=geminal diol, M = thiol, E=isothiazolone, G=thiazole, H=disulfide, I = enethiol-thiol disulfide.*

Table K18: Approximate conversion values (%) of photolysis products (phenacylsulfide ***32****, H_2O, initial pH 10, final pH 9.6) based on integration of HPLC peaks (detection at 214 nm). Approximate conversions from phenacylsulfide (Norrish type-II and β-scission processes, column 2) and from putative thioaldehyde (Norrish type-II processes only, column 3).*

**products**	**% Area (Conversion from phenacylsulfide)**	**% Area (Conversion from putative thioaldehyde)**
oxamate	2%	6%
ene-sulfinic acid	9%	21%
geminal diol	10%	25%
thiol	7%	
isothiazolone	10%	24%
thiazole	4%	10%
enethiolate-thiol disulfide	11%	14%
disulfide	47 %	

Reaction in non-buffered H_2O (initial pH 7.4)

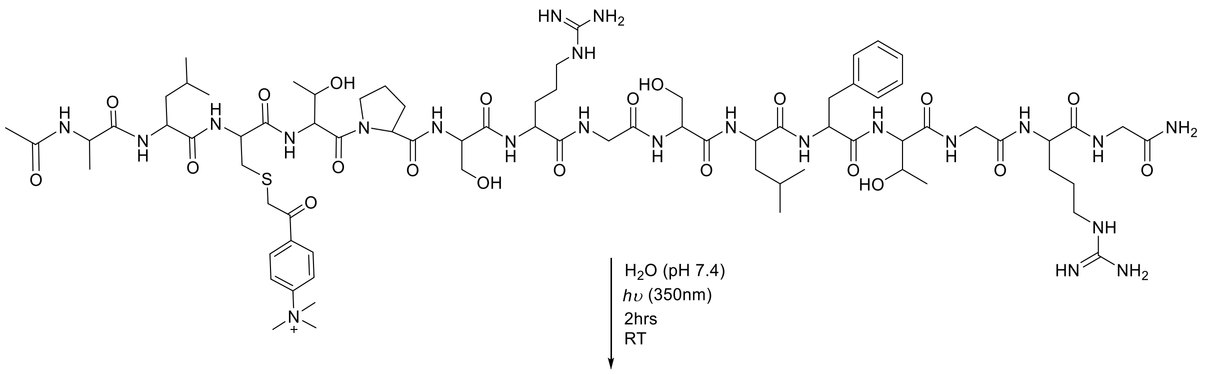

A solution of phenacylsulfide ***32*** (3 mg, 0.0017 mmol) in H_2O (1 mL, 1.7 mM solution, pH 3) was adjusted to pH 7.4 by using 0.25 M NaOH solution. The solution was degassed by passing N_2 gas through the solution for 20 mins. The solution was then illuminated at 350 nm in a Rayonet photoreactor for 2 hrs at room temperature. The pH was the reaction mixture after photolysis was measured to be 6.4. The reaction mixture was analysed by analytical HPLC and HRMS (ESI positive mode).

*Table K19: Analytical HPLC method used for characterisation of photolysis products (phenacylsulfide **32**, H_2O, initial pH 7.4)*

time (min)	A (%)	B (%)
0	95	5
5	80	20
7	80	20
25	63	37
30	0	100
35	100	0

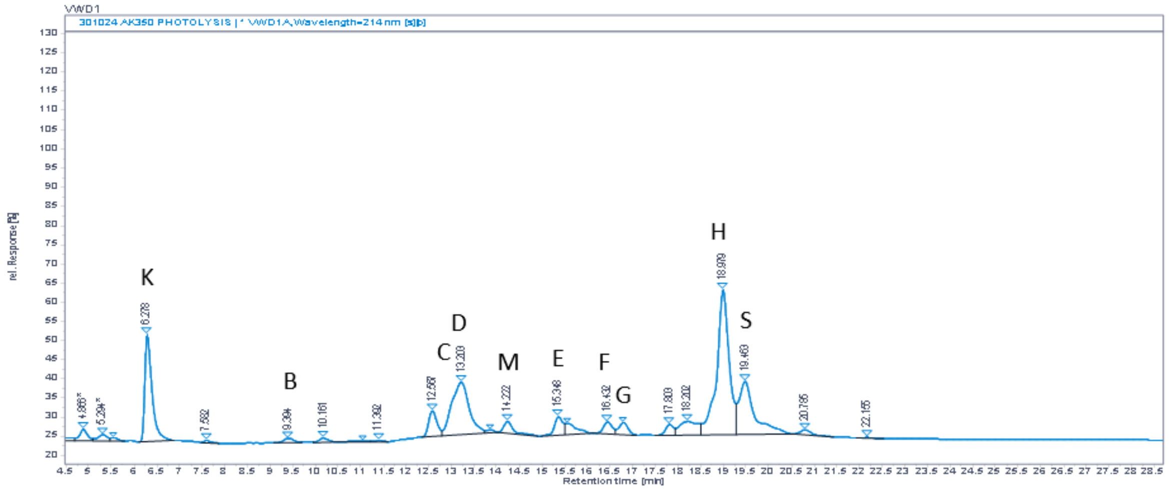

*Figure K49: Analytical HPLC spectrum of reaction mixture derived from the photolysis of phenacylsulfide **32** in H_2O (initial pH 7.4, final pH 6.4). K=ketone, B=oxamate, C=geminal diol, D=formylglycine, M=thiol, E=isothiazolone, F=oxazole, G=thiazole, H=disulfide, S=dithioacetal-thiol disulfide. Peak at 12.5 min is an impurity present in the starting material.*

Table K20: Approximate conversion values (%) of photolysis products (phenacylsulfide ***32****, H_2O, initial pH 7.4, final pH 6.4) based on integration of HPLC peaks (detection at 214 nm). Approximate conversions from phenacylsulfide (Norrish type-II and β-scission processes, column 2) and from putative thioaldehyde (Norrish type-II processes only, column 3).*

**products**	**% Area (Conversion from phenacylsulfide)**	**% Area (Conversion from putative thioaldehyde)**
oxamate	2%	4%
geminal diol	5%	13%
formylglycine	17%	39%
thiol	4%	
isothiazolone	6%	14%
oxazole	2%	5%
thiazole	2%	5%
disulfide	45%	
dithioacetal-thiol disulfide	17%	20%

Treatment of non-buffered photolysis mixture with DTNB

The reaction mixture derived from the photolysis of phenacylsulfide ***32*** in non-buffered H_2O (initial pH 7.4, 0.5 mL, 0.00085 mmol) was treated with DTNB (0.00068 mmol, 0.8 eq.) The reaction mixture was allowed to evolve overnight. The reaction mixture was analysed by analytical HPLC and HRMS (ESI positive mode).

*Table K21: Analytical HPLC method used for characterisation of photolysis products (phenacylsulfide **32**, H_2O, initial pH 7.4) after DTNB addition.*

time (min)	A (%)	B (%)
0	95	5
5	80	20
7	80	20
25	63	37
30	0	100
35	100	0

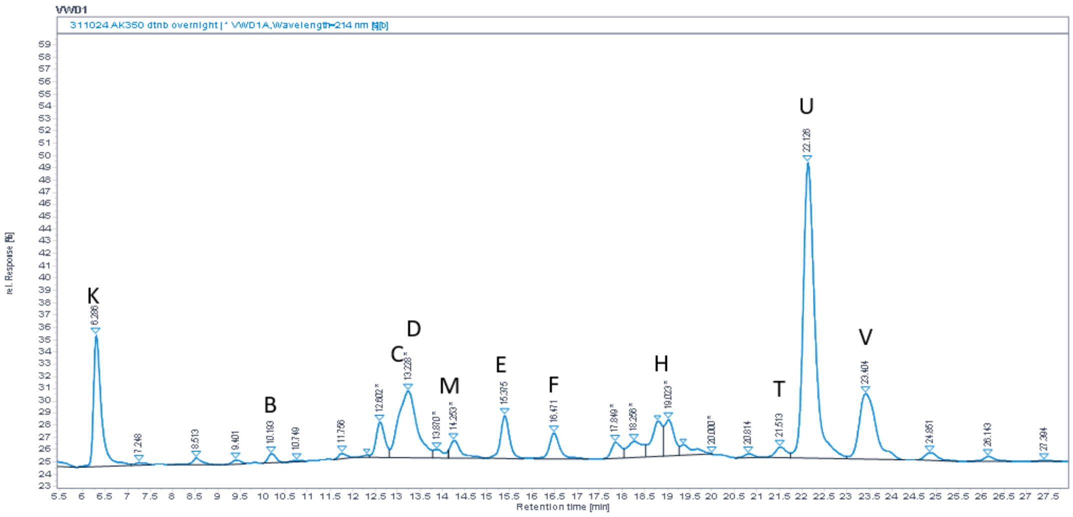

*Figure K50: Analytical HPLC spectrum of reaction mixture derived from the photolysis of phenacylsulfide **32** in H_2O (initial pH 7.4, final pH 6.4) followed by treatment with DTNB. K=ketone, B=oxamate, C=geminal diol, D=formylglycine, M=thiol, E=isothiazolone, F=oxazole, H=disulfide, T=enethiol-dtnb disulfide, U=thiol-dtnb disulfide, V=dithioacetal-dtnb disulfide. Peak at 12.60 min is an impurity in the starting material.*

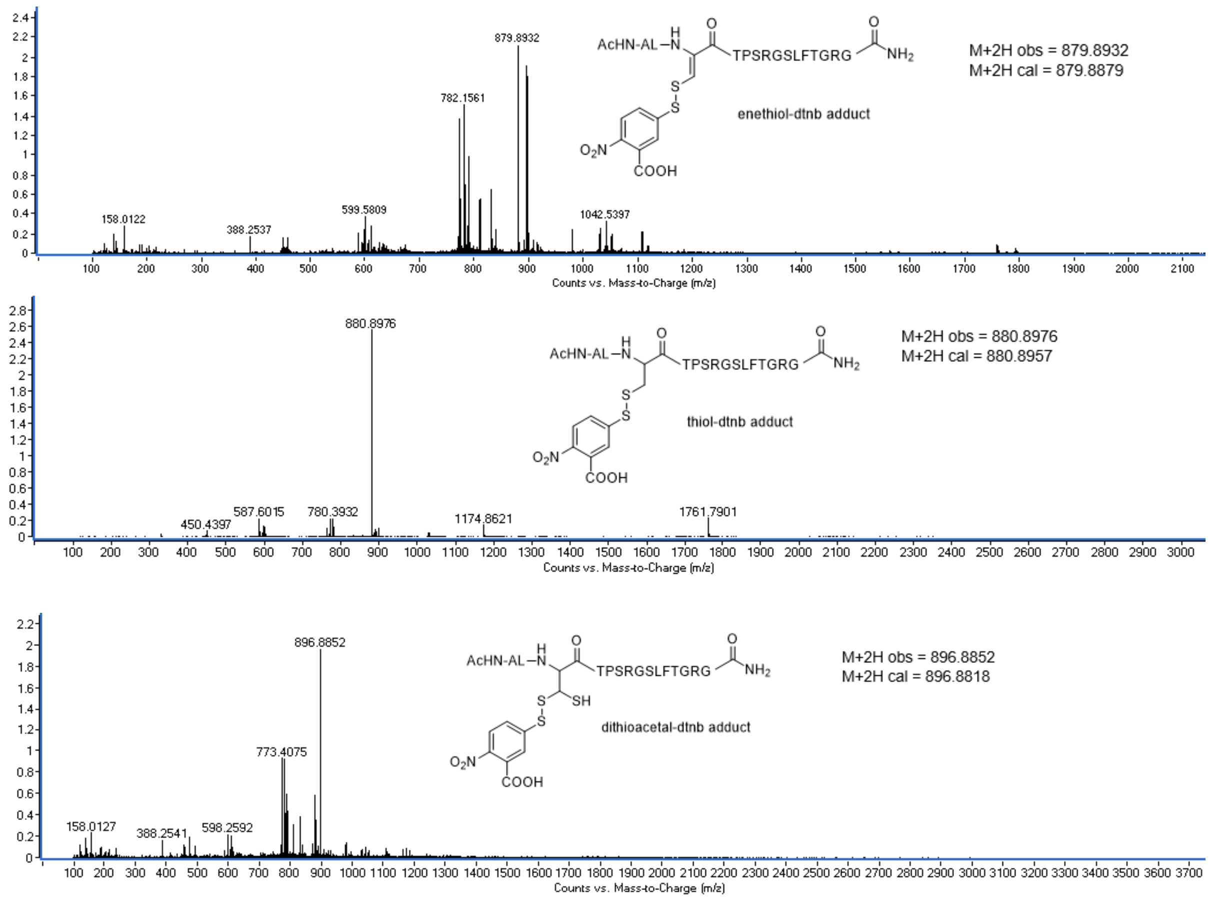

Figure K51: HRMS (ESI positive mode) spectra of reaction mixture derived from the photolysis of phenacylsulfide ***32*** *in H_2O (initial pH 7.4, final pH 6.4) followed by treatment with DTNB. Observed and calculated ion peak values for each products are provided.*

Reaction in non-buffered D_2O (initial pD 7.8)

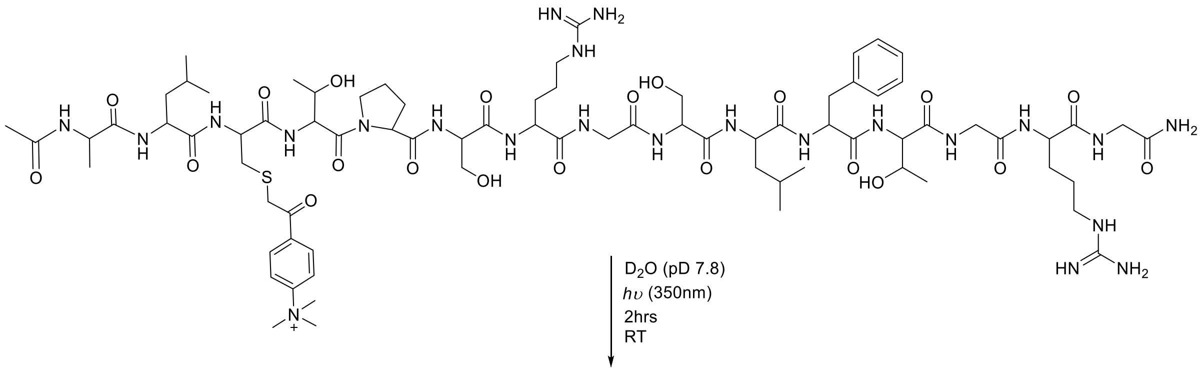

A solution of phenacylsulfide ***32*** (3 mg, 0.0017 mmol) in D_2O (1 mL, 1.7 mM solution, pD 3.4) was adjusted to pD 7.8 by using 0.25 M NaOD solution. The solution was degassed by passing N_2 gas through the solution for 20 mins. The solution was then illuminated at 350 nm in a Rayonet photoreactor for 2 hrs at room temperature. The pD of the reaction mixture after photolysis was measured to be 6.8. The reaction mixture was analysed by ^{1}H NMR spectroscopy.

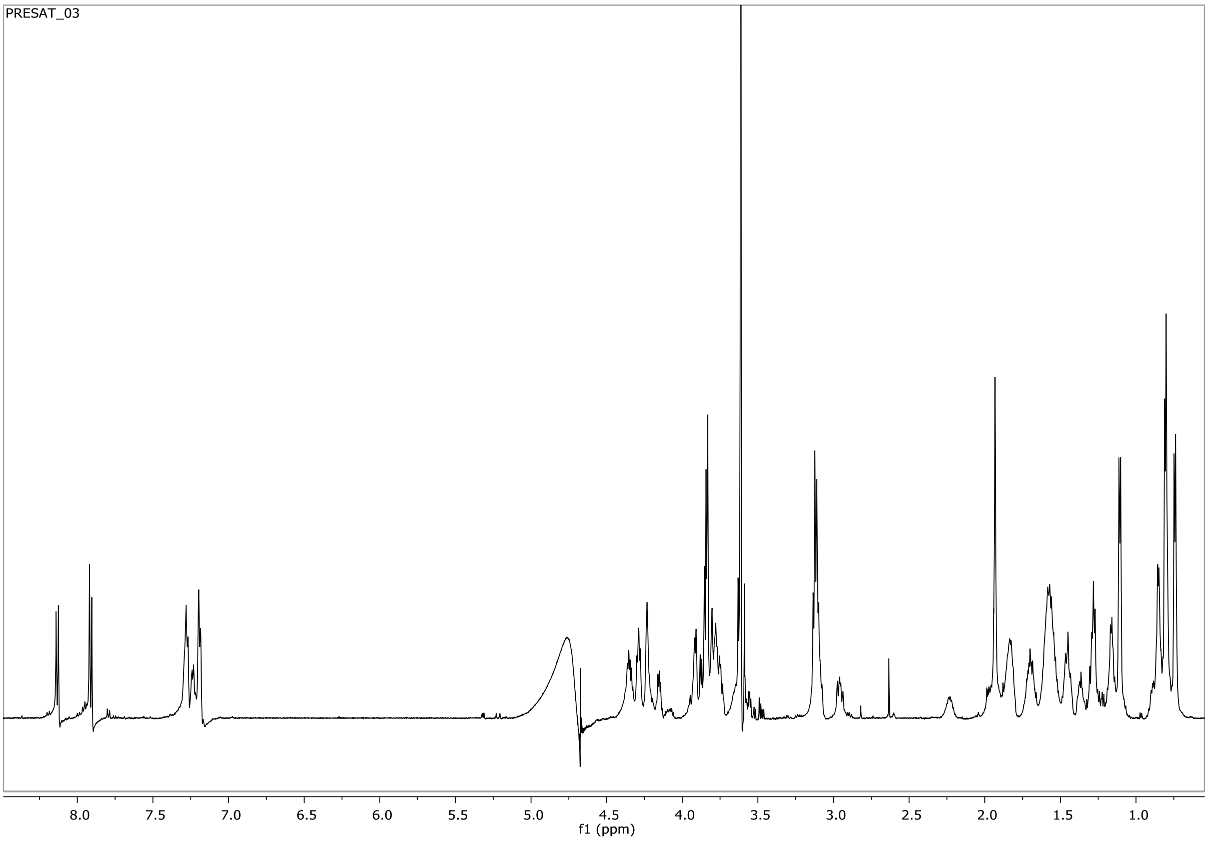

Figure K52: ^{1}H NMR spectrum of photolysis reaction mixture of phenacylsulfide ***32*** *in D_2O, pD 7.8*

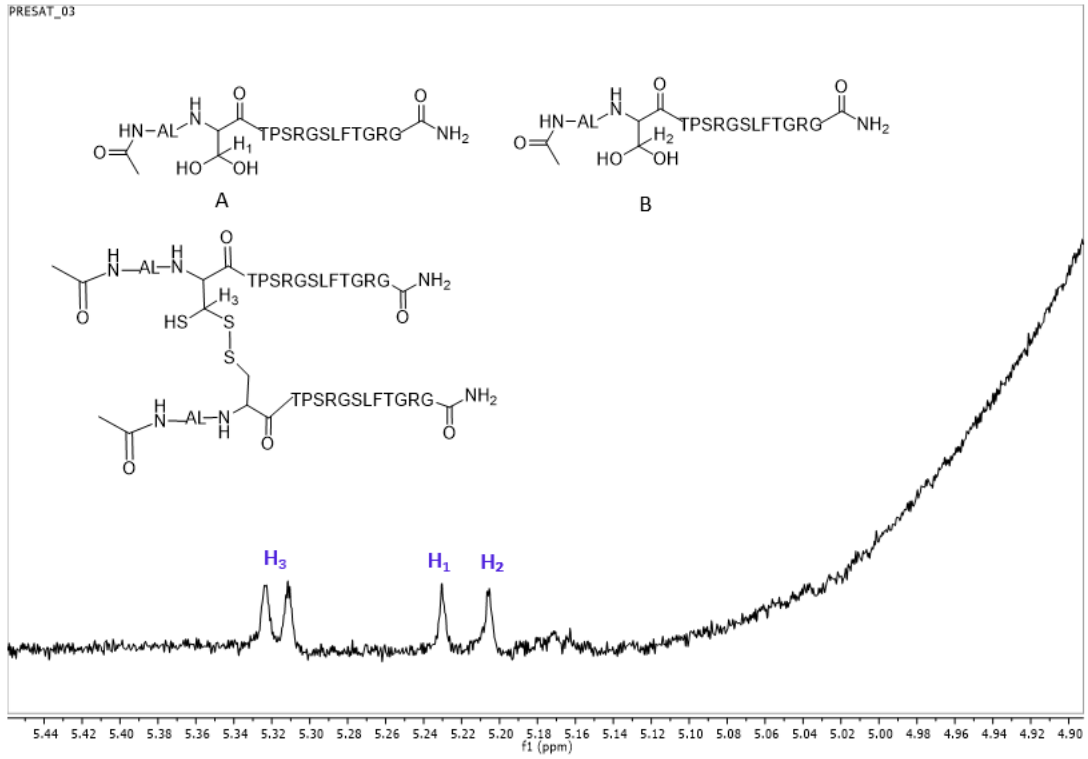

*Figure K53: ^{1}H NMR spectrum of photolysis reaction mixture of phenacylsulfide **32** in D_2O, pD 7.8 showing the methine protons of two diastereomers of geminal diol (A and B) and dithioacetal–thiol disulfide at 5.20, 5.22 and 5.32 ppm respectively. The doublet at 5.32 ppm has a coupling constant value of 7.4 Hz, which is consistent with the coupling constant values obtained for the doublet corresponding to the methine proton of dithioacetal–thiol disulfide present in photolysis reaction mixture of phenacylsulfide **32** in buffered D_2O. Note: The methine protons of geminal diol H_1, H_2 are appearing as singlets due to the incorporation of deuterium into the α centre.*

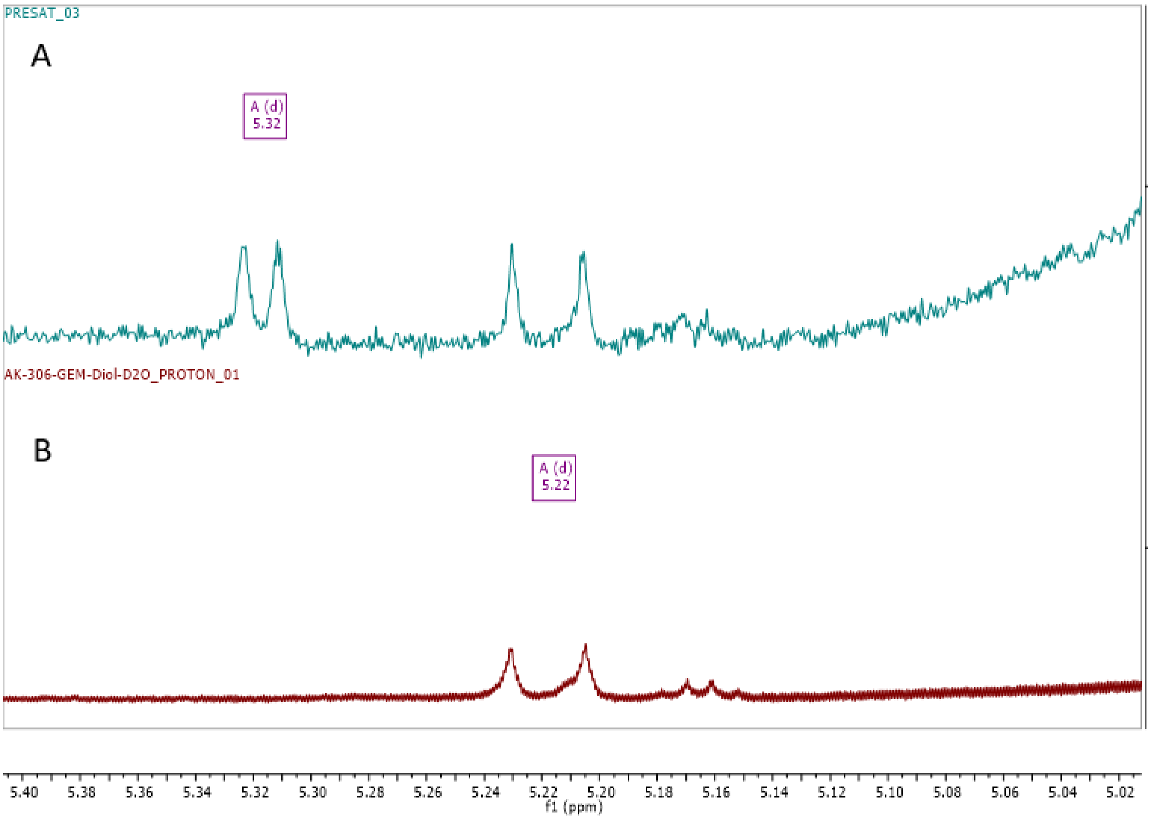

Figure K54: Stacked ^{1}H NMR spectrum of (A) photolysis reaction mixture of phenacylsulfide ***32*** *in D_2O, pD 7.8 showing the methine protons of two diastereomers of geminal diol and dithioacetal-thiol disulfide at 5.20, 5.22 and 5.32 ppm respectively. (B) Oxidative cleavage reaction mixture of hydroxythreonine-containing pentadecapeptide showing the β methine protons of diastereomers of geminal diol at 5.20 and 5.22 ppm. The singlets at 5.20, 5.22 ppm corresponding to the methine signals of diastereomers of geminal diol present in the oxidative cleavage reaction mixture of hydroxythreonine-containing pentadecapeptide is consistent with the singlets for methine signals of diastereomers of geminal diol present in the photolysis reaction mixture of phenacylsulfide* ***32*** *in D_2O, pD 7.8.*

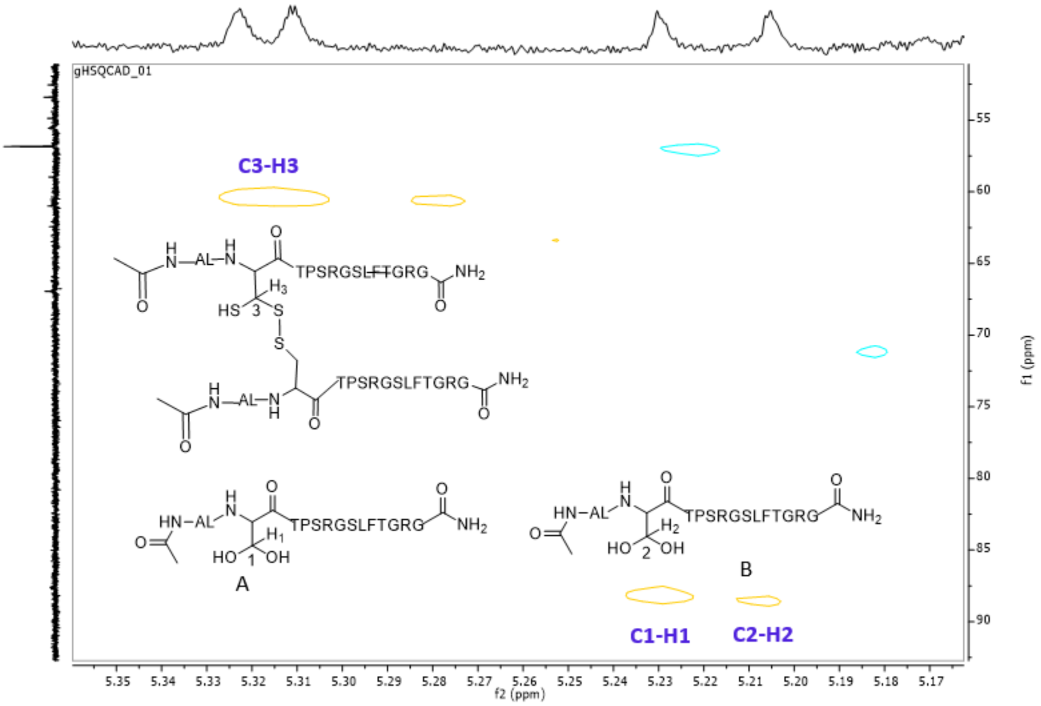

Figure K55: Zoomed in region of HSQC spectrum of photolysis reaction mixture of phenacylsulfide ***32*** *in D_2O, pD 7.8. The spectra shows the key C-H correlation between a methine proton at 5.22 ppm and a carbon at 89.07 ppm, a methine proton at 5.20 ppm and a carbon at 88.93 ppm indicating the C-H correlations of the diastereomers of the geminal diol (A and B) consistent with the standard oxidative cleavage reaction mixture of hydroxythreonine-containing pentadecapeptide. Doublet at 5.33 correlates to a carbon at 60 ppm which is consistent with the C-H correlations of dithioacetal–thiol disulfide observed in the photolysis reaction mixture of phenacylsulfide* ***27*** *in D_2O (0.1 M phosphate, pD 7.8).*

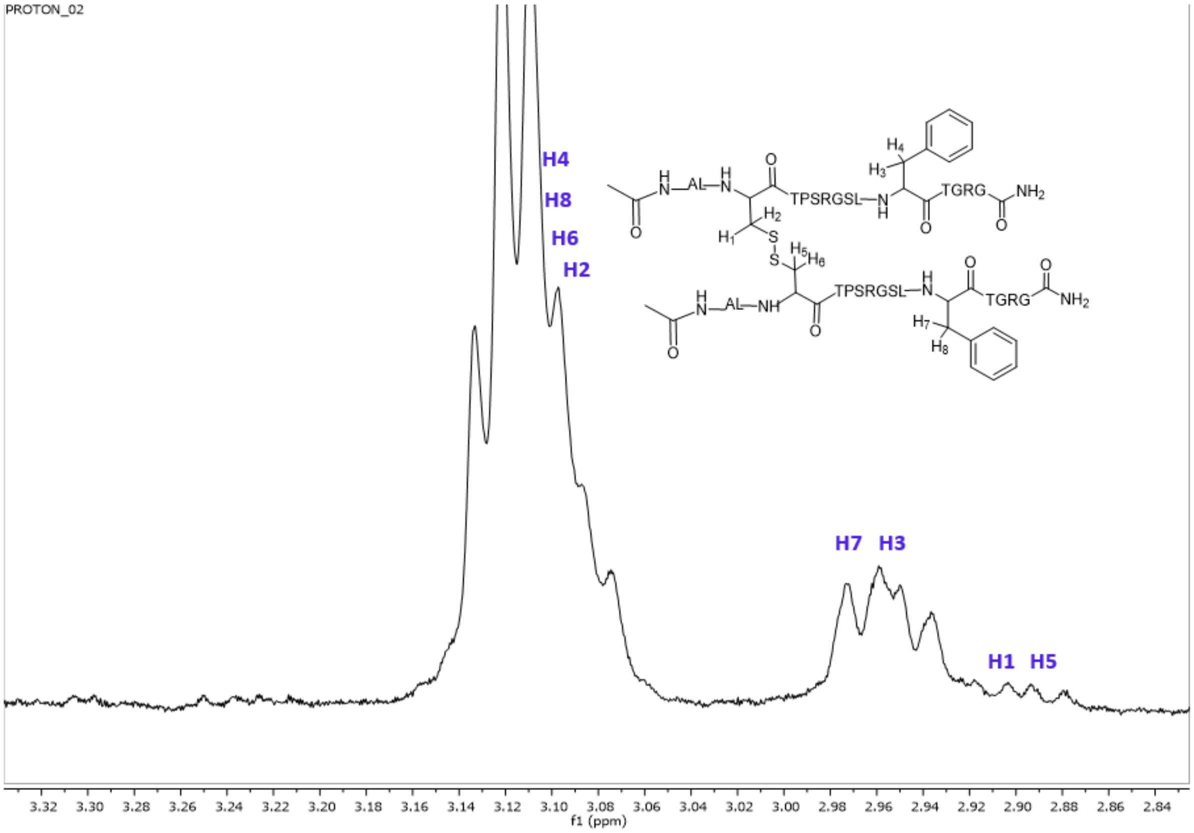

*Figure K56: ^{1}H NMR spectrum of photolysis reaction mixture of phenacylsulfide **32** in D_2O pD 7.8 showing the doublet of doublets for the diastereotopic methylene protons of disulfide. Note: One set of diastereotopic protons H_1, H_5 of disulfide appears as a doublet of doublet in the region 2.87 to 2.92 ppm. The second set of signals for the diastereotopic protons H_2, H_6 is hidden under the γ methylene signals of arginine from 3.14–3.08 ppm.*

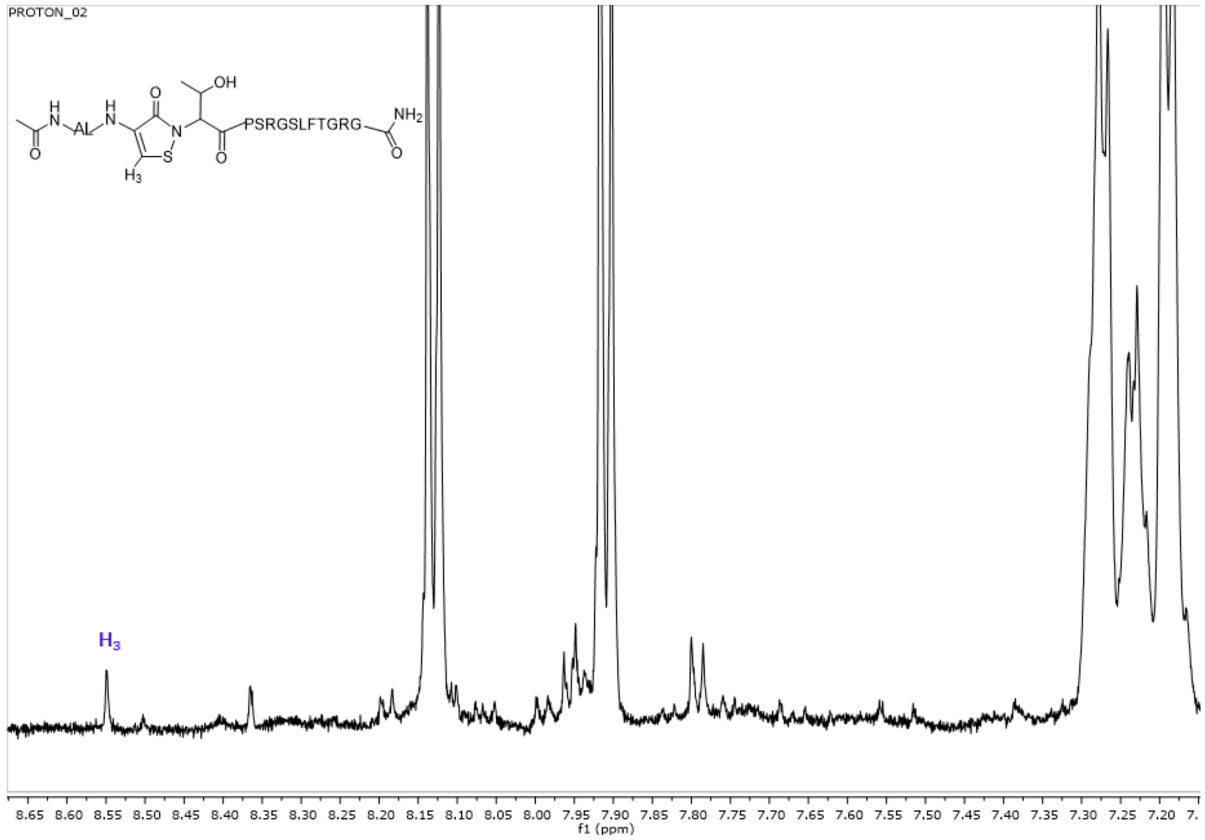

*Figure K57: Zoomed in region of ^{1}H NMR spectrum of photolysis reaction mixture of phenacylsulfide **32** in D_2O, pD 7.8 showing a singlet for the H_3 proton of isothiazolone at 8.55 ppm.*

*Table K22: Conversion values (%) of photolysis products (phenacylsulfide **32**, D_2O, pD 7.8) based on the integration of methyl protons of threonine residue in the NMR spectra. Approximate conversions from phenacylsulfide (Norrish type-II and β-scission processes, column 2) and from putative thioaldehyde (Norrish type-II processes only, column 3).*
Note: The integration of a methine CH signal for the dithioacetal–thiol disulfide, geminal diol, isothiazolone and disulfide are calculated relative to the methyl protons of the threonine residue of respective molecules appearing in the region 1.14–1.27 ppm.

**Product**	**Conversion percentage from NMR**
geminal diol	27 %
isothiazolone	11%
dithioacetal-thiol disulfide	27 %
disulfide	35%

Preparation of formylglycine standard

Preparation of hydroxythreonine-containing pentadecapeptide

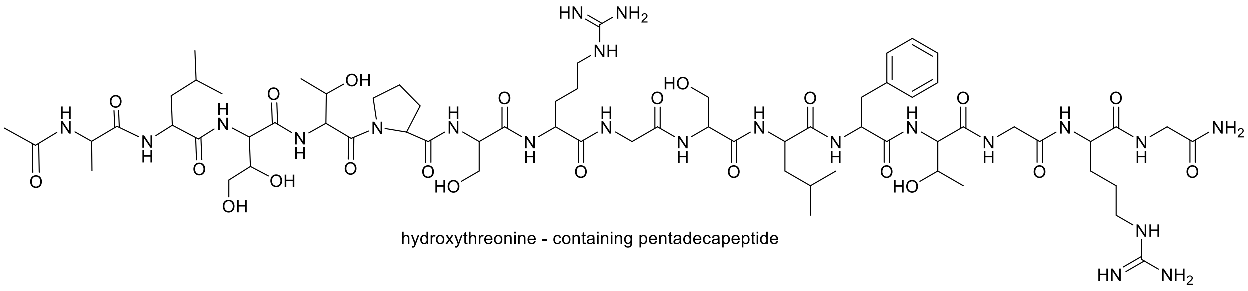

hydroxythreonine - containing pentadecapeptide

The Fmoc- and dimethylacetonide-protected hydroxythreonine building block and hydroxythreonine-containing pentadecapeptide was prepared according to the procedure described by Yates et al[128]. The pentadecapeptide was purified by semi-preparative HPLC. The semi-preparative HPLC was performed on Machery Nagel C18 HTec column 5 µm packing at 24 °C with a flow rate of 2.5 mL/min. The solvents employed were A = 0.1 % v/v solution of TFA in water. B = 0.1 % v/v solution of TFA in acetonitrile.

Table K23: Analytical HPLC method used for characterisation of hydroxythreonine-containing pentadecapeptide

**time (min)**	**A (%)**	**B (%)**
0	95	5
5	80	20
7	80	20
25	63	37
30	0	100
35	100	0

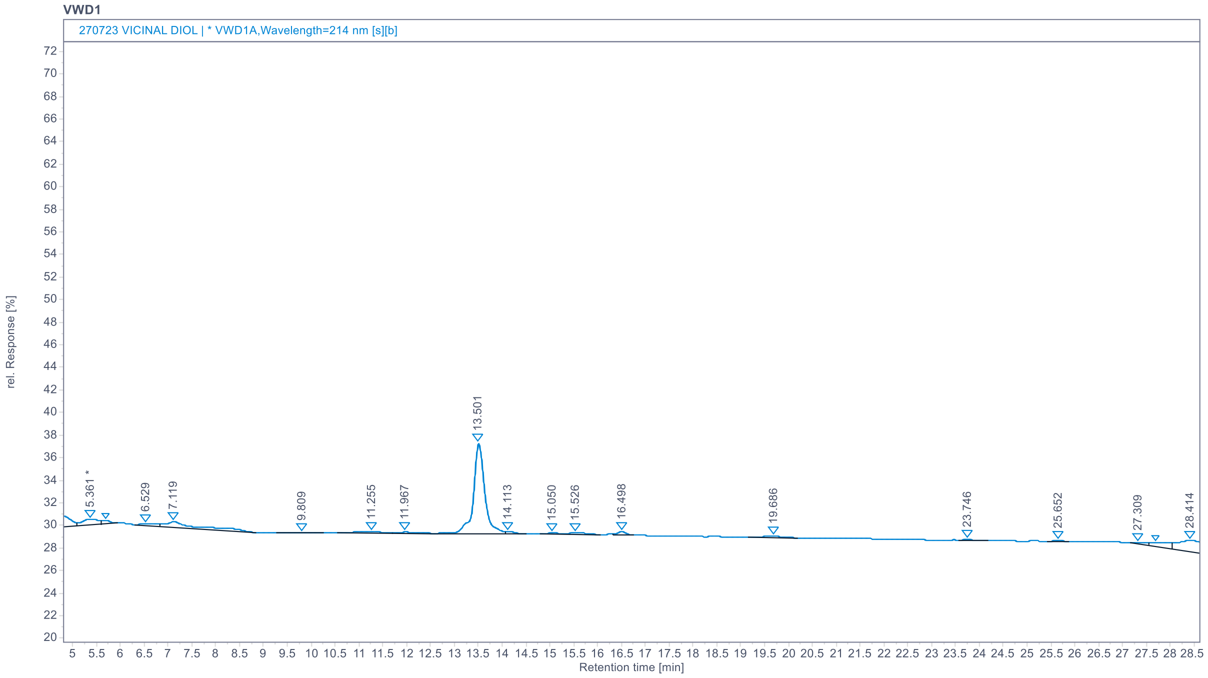

Figure K58: Analytical HPLC spectrum of hydroxythreonine-containing pentadecapeptide (RT = 13.50 min). Peak at RT = 16.49 min is an impurity.

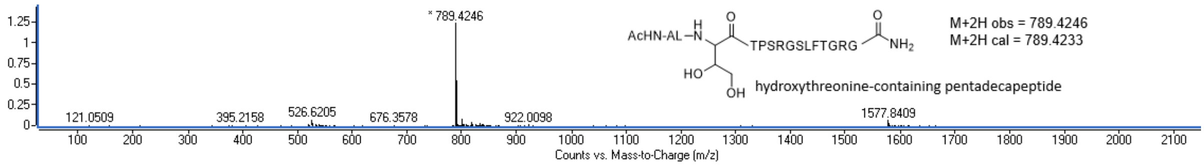

Figure K59: HRMS (ESI positive mode) spectrum of hydroxythreonine-containing pentadecapeptide . Observed and calculated m/z peaks are provided.

Oxidative cleavage of hydroxythreonine-containing pentadecapeptide in H_2O

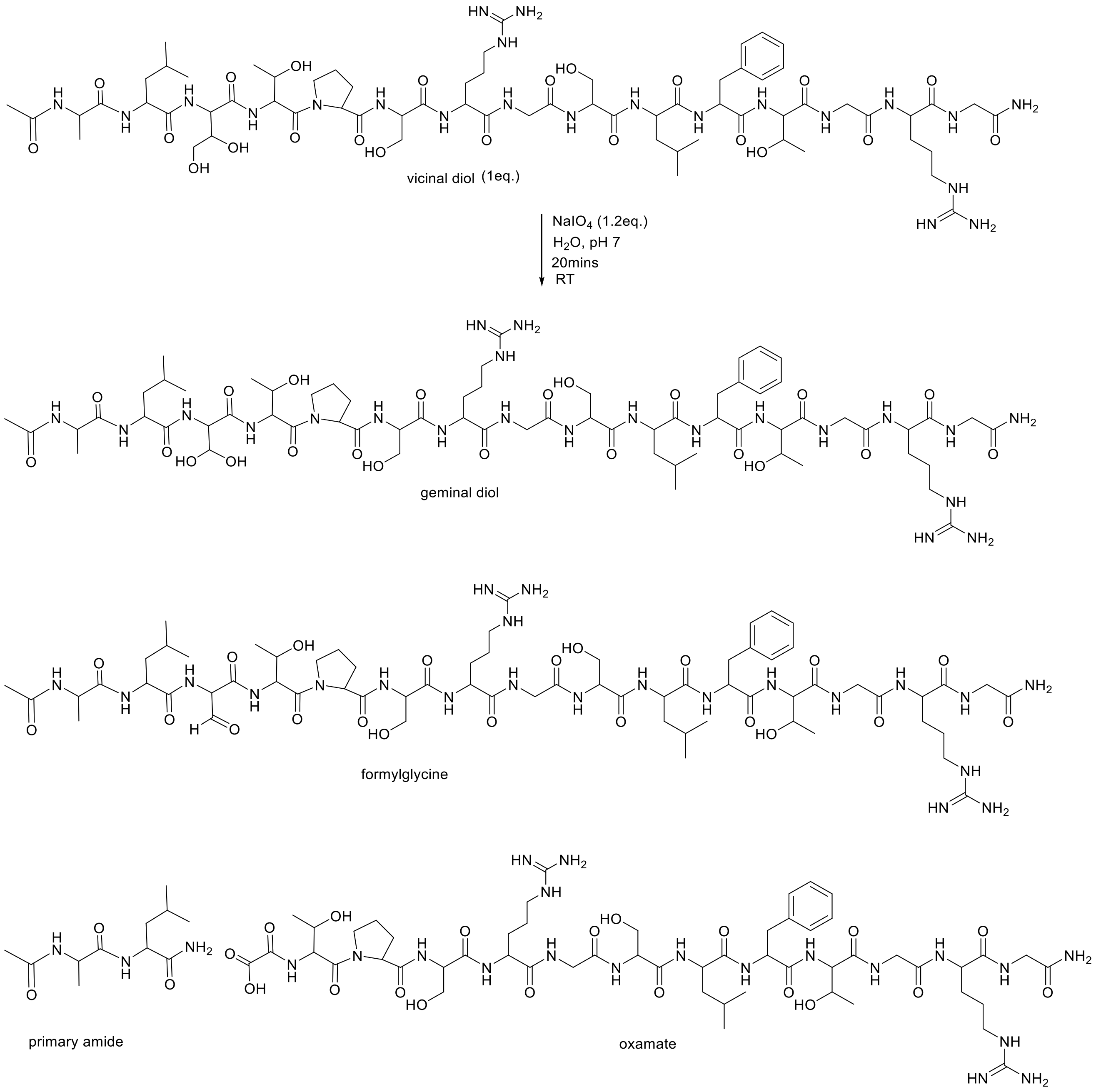

An aqueous solution of $NaIO_4$ (1.2 eq., 76 µL, 50 mM) was added in 5 aliquots over 5 min to a solution of hydroxythreonine-containing pentadecapeptide (1 eq., 1.5 mL, 2.0 mM) in HPLC-grade water. The reaction mixture, as monitored by analytical HPLC, showed complete conversion of starting material into the *gem*-diol formylglycine derivative within 20 minutes at room temperature. In the presence of excess oxidising agent, the *gem*-diol, undergoes oxidative cleavage to give the primary amide and oxamate.

Table K24: Analytical HPLC method used for characterisation of hydroxythreonine-containing pentadecapeptide oxidative cleavage mixture

time (min)	A (%)	B (%)
0	95	5
5	80	20
7	80	20
25	63	37
30	0	100
35	100	0

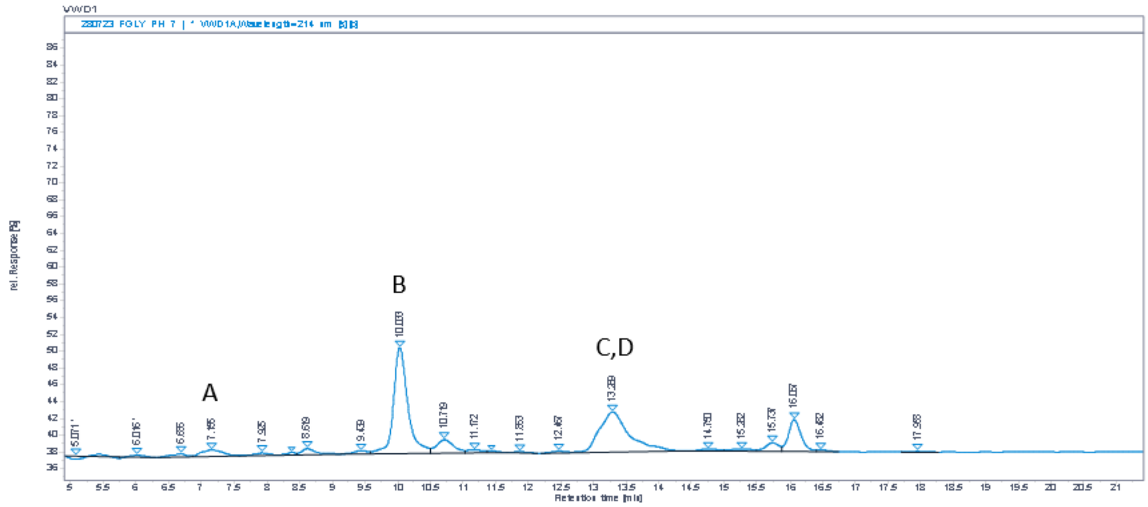

Figure K60: Analytical HPLC spectrum of reaction mixture derived from the treatment of hydroxythreonine-containing pentadecapeptide with $NaIO_4$. A= primary amide, B=oxamate, C=geminal diol, D =formylglycine. Peak at 16.05 min is an impurity present in starting material.

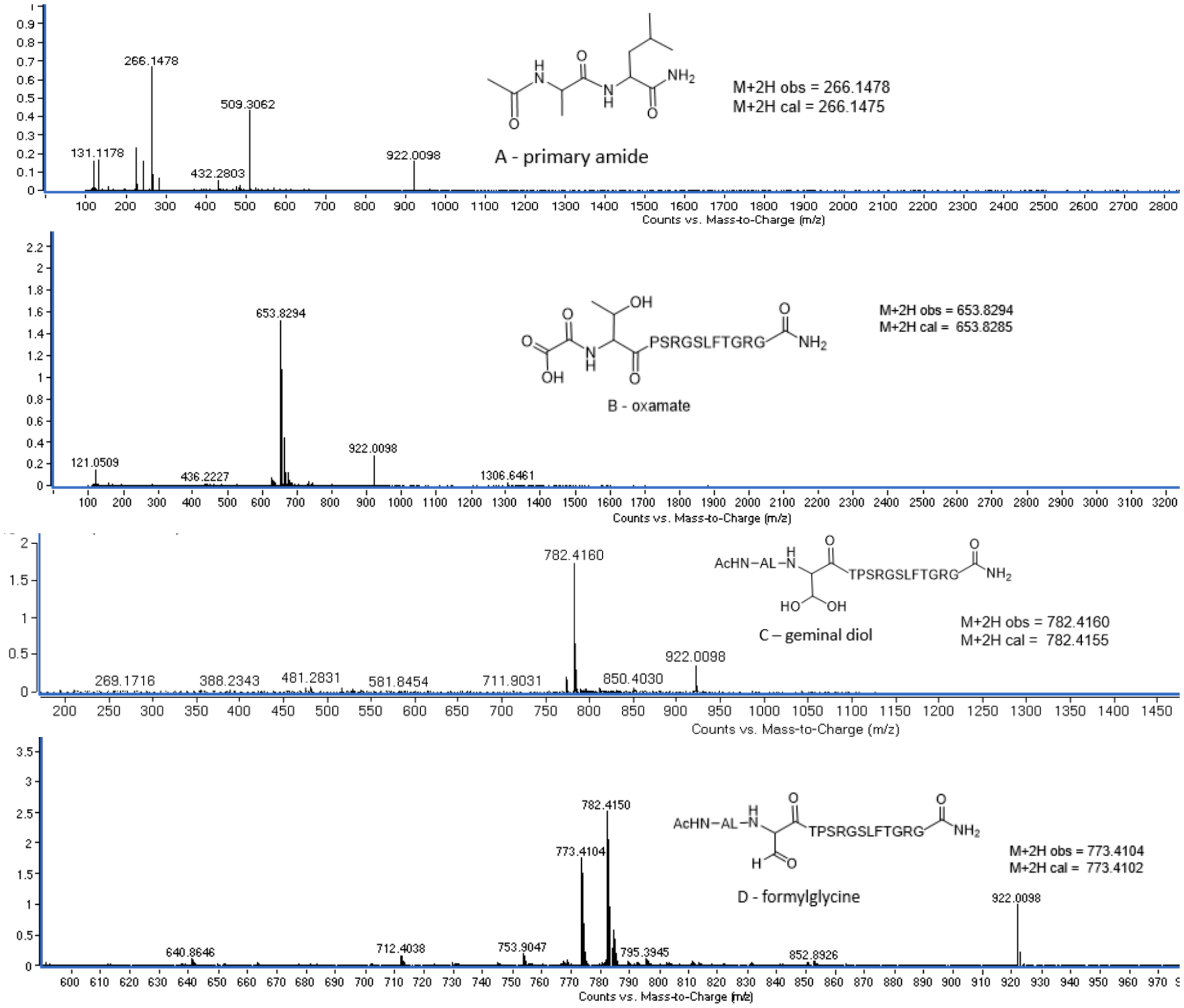

Figure K61: HRMS (ESI positive mode) spectrum of reaction mixture derived from the treatment of hydroxythreonine-containing pentadecapeptide with $NaIO_4$. Observed and calculated HRMS (ESI positive mode) m/z peaks are given.

Table K25: Approximate conversion values (%) of oxidative cleavage products of hydroxythreonine-containing pentadecapeptide based on integration of HPLC peaks (detection at 214 nm).

**Product**	**Conversion percentage from HPLC**
Oxamate	58 %
Geminal diol/formylglycine	42 %

Oxidative cleavage of hydroxythreonine-containing pentadecapeptide in D_2O

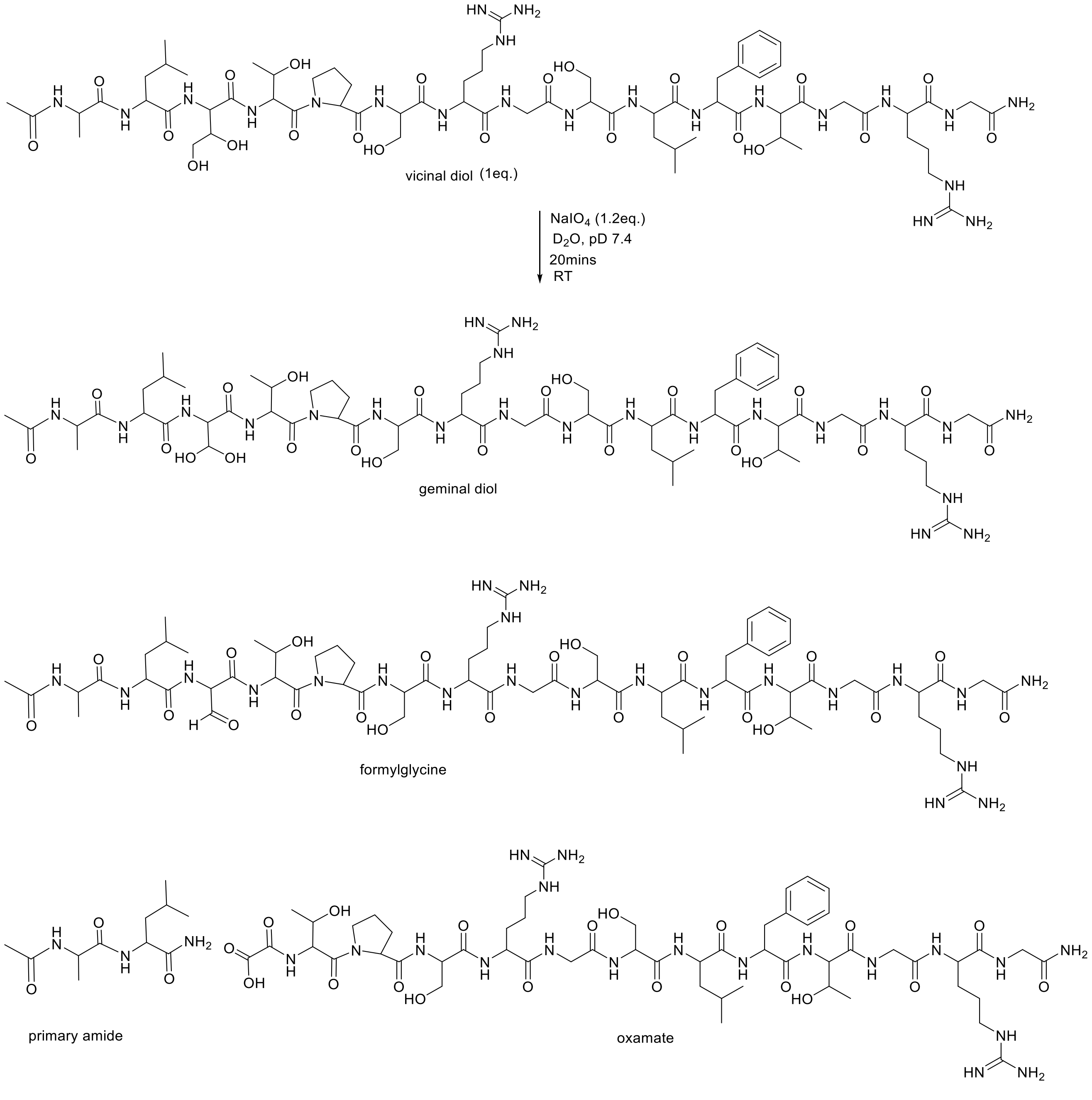

An aqueous solution of $NaIO_4$ (1.2 eq., 76 µL, 50 mM) was added in 5 aliquots over 5 min to a solution of hydroxythreonine-containing pentadecapeptide (1 eq., 1.5 mL, 2.0 mM) in D_2O. The reaction mixture was analysed by ^1H NMR spectroscopy.

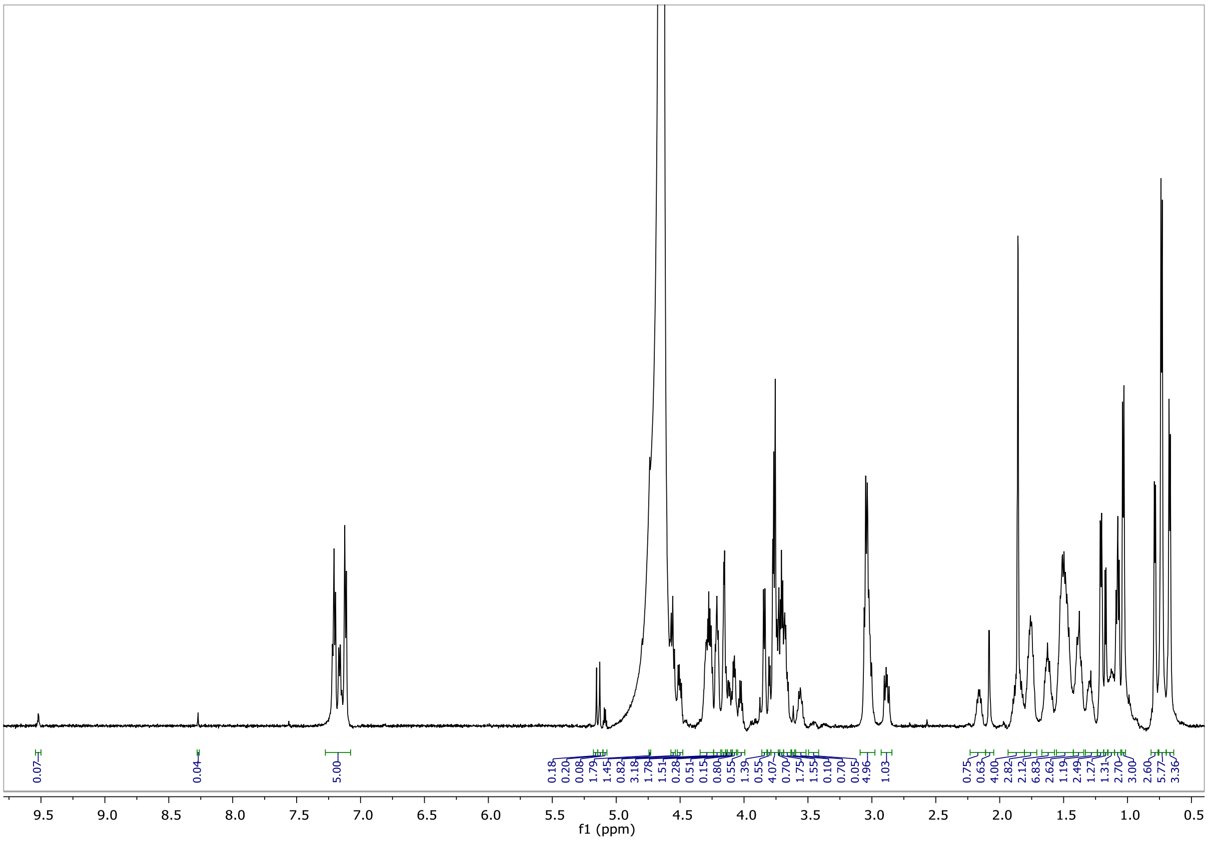

Figure K62: ^{1}H NMR spectrum of oxidative cleavage reaction mixture of hydroxythreonine-containing pentadecapeptide in D_2O, pD 7.4.

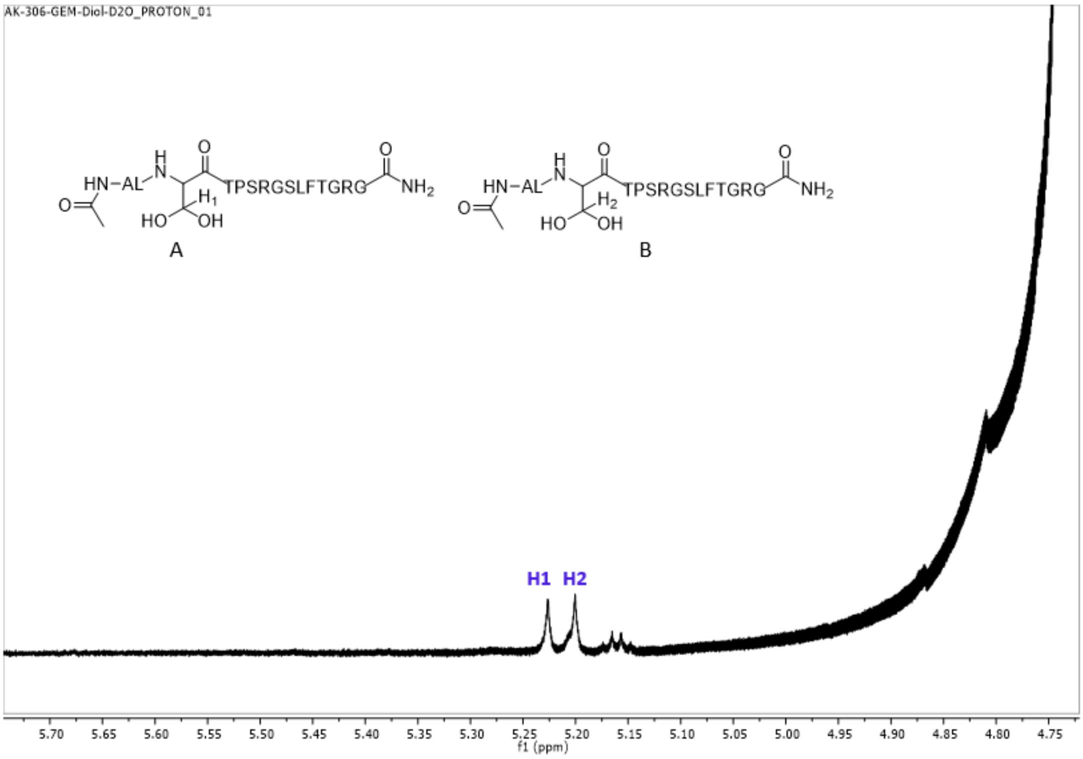

Figure K63: Zoomed in region of ^{1}H NMR spectrum of oxidative cleavage reaction mixture of hydroxythreonine-containing pentadecapeptide showing the β proton H_1 and H_2 of diastereomers of geminal diol at 5.20 and 5.23 ppm respectively accounting for 47% of the oxidative cleavage reaction mixture.

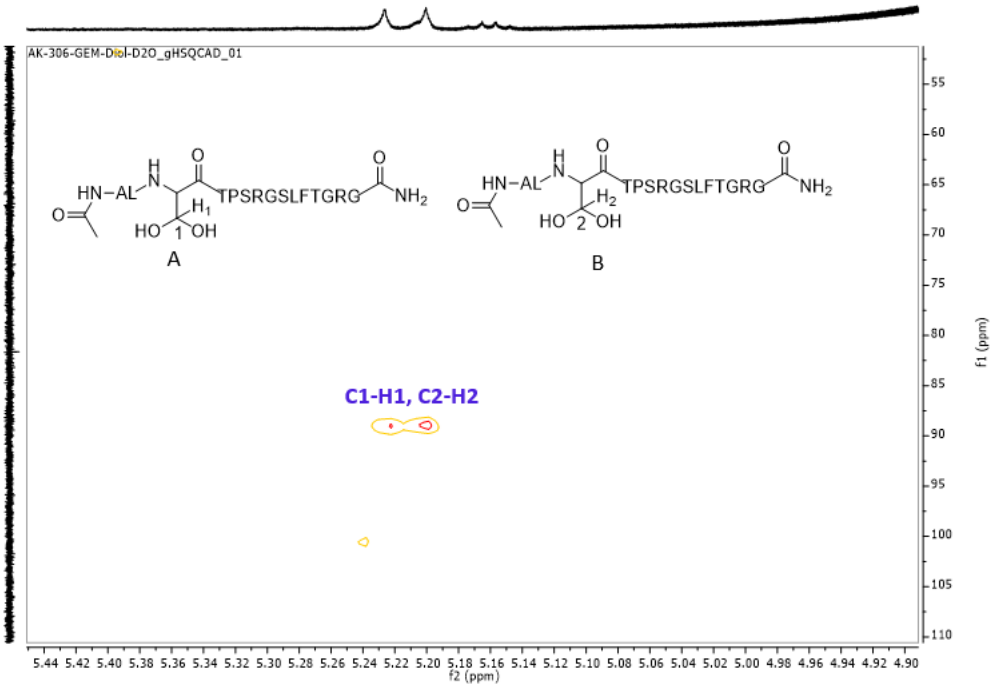

Figure K64: Zoomed in region of HSQC spectrum of oxidative cleavage reaction mixture of hydroxythreonine-containing pentadecapeptide showing the key C-H correlations of diastereomers of germinal diol A and B. Correlations are assigned for a proton H_2 at 5.22 ppm and a carbon C_2 at 89.07 ppm of diastereomer B, a proton H_1 at 5.20 ppm and a carbon C_1 at 88.93 ppm of diastereomer A respectively.

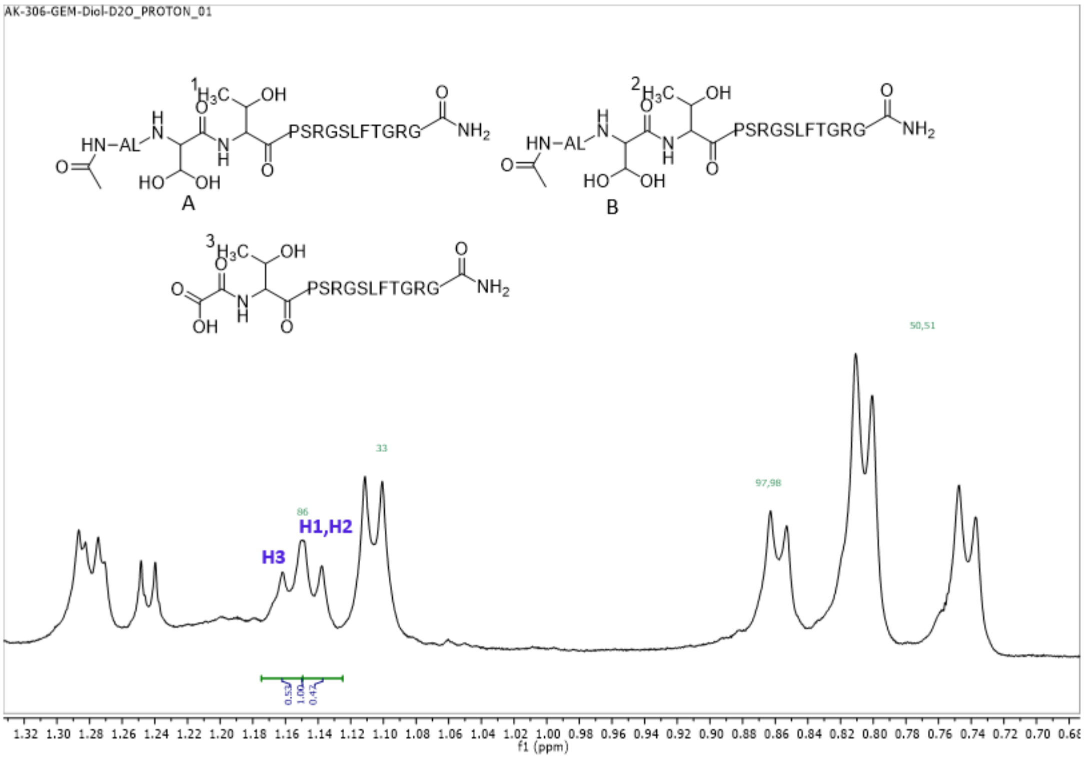

Figure K65: Zoomed in region of ^{1}H NMR spectrum of oxidative cleavage reaction mixture of hydroxythreonine-containing pentadecapeptide showing the methyl protons of threonine residue of two diastereomers (A and B) of geminal diol and oxamate at 1.12–1.15 ppm and 1.15–1.17 ppm respectively. Note: the doublets corresponding to the methyl peaks are merged in the spectra. From NMR spectra, oxamate accounts for 53% of the oxidative cleavage reaction mixture.

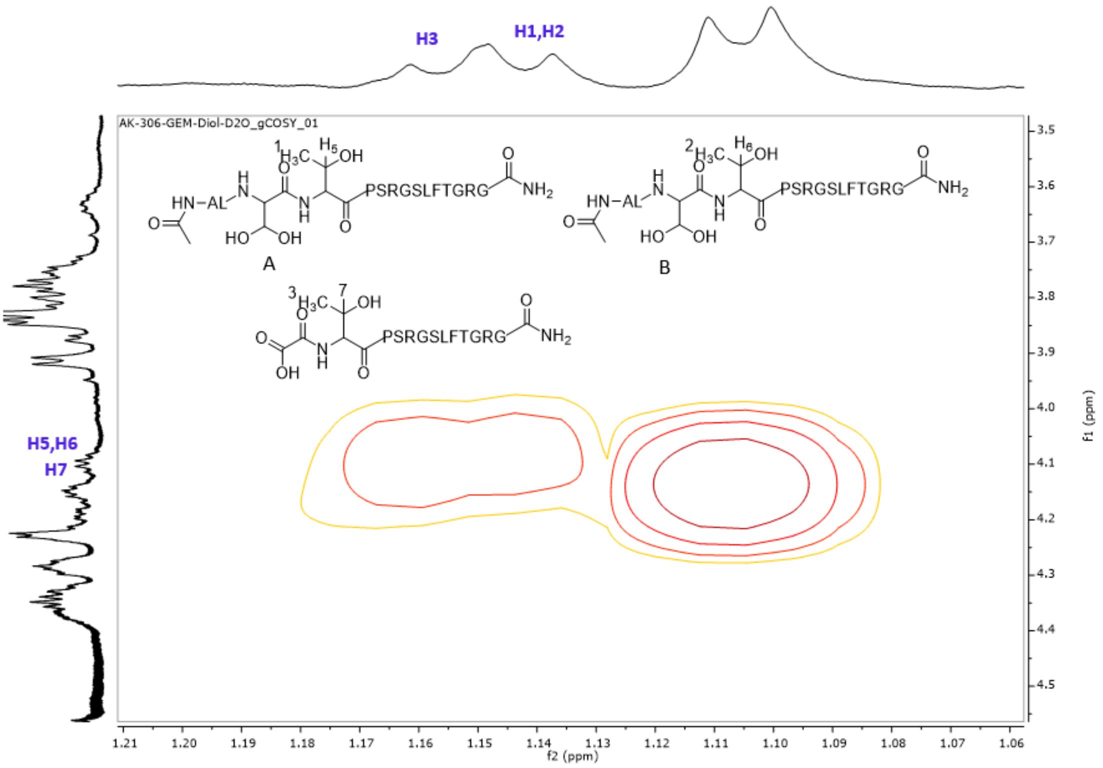

Figure K66: Zoomed in region of COSY spectrum of oxidative cleavage reaction mixture of hydroxythreonine-containing pentadecapeptide showing the H-H correlations. The methyl protons (H_1, H_2) of threonine residue of two diastereomers of geminal diol (A and B) appearing in the range 1.12–1.15 ppm shows a correlation to the β methine protons (H_5, H_6) of the threonine residue appearing in the range 3.99–4.05 ppm. The methyl proton (H_3) of threonine residue of oxamate appearing in the range 1.15–1.17 ppm shows a correlation to the β methine proton (H_7) of the threonine residue appearing in the range 3.99–4.05 ppm. Note: the multiplet corresponding to the β methine protons of threonine residue of two diastereomers of geminal diol (A and B) and oxamate are merged in the spectra.

Condensation reaction of geminal diol/formylglycine with $NH_2OBn.HCl$

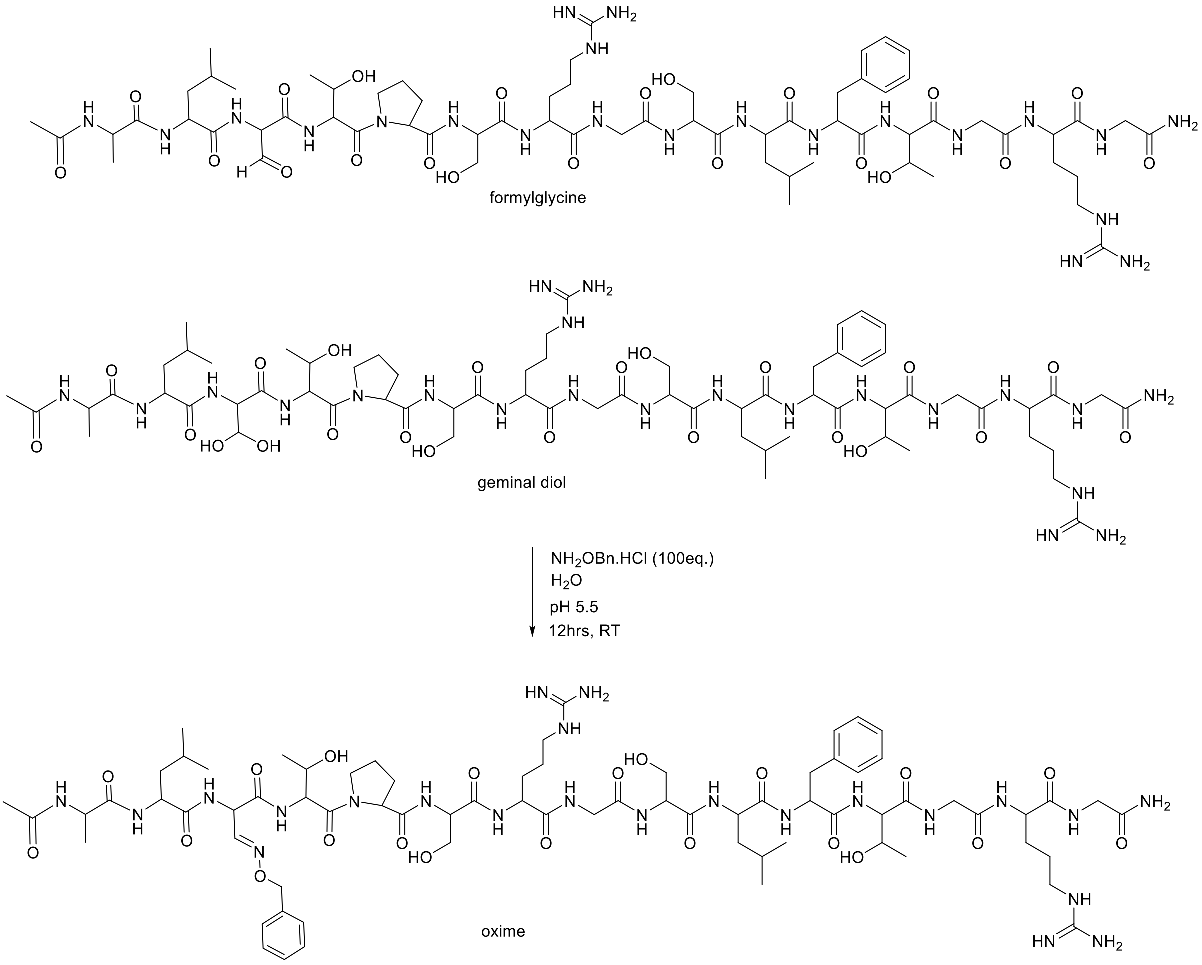

$NH_2OBn.HCl$ (3.2 mg, 0.02 mmol, 100 eq.) was added to the reaction mixture derived from the treatment of hydroxythreonine-containing pentadecapeptide with $NaIO_4$ (100 µL, pH 5.5, 1eq.) The reaction mixture was allowed to evolve overnight at room temperature. The reaction mixture was analysed by analytical HPLC and HRMS (ESI positive mode).

Table K26: Analytical HPLC method used for characterisation of condensation reaction mixture of geminal diol/formylglycine with $NH_2OBn.HCl$

time (min)	A (%)	B (%)
0	95	5
5	80	20
7	80	20
25	63	37
30	0	100
35	100	0

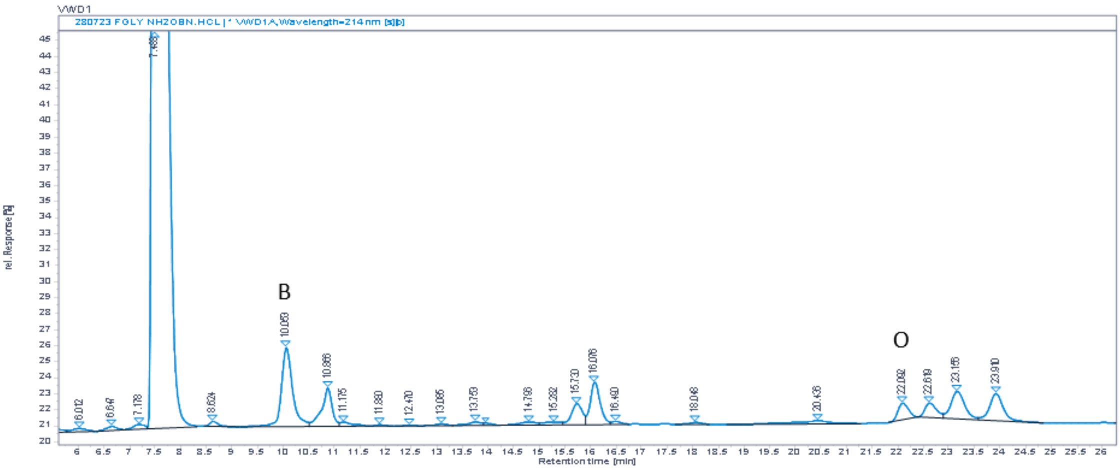

Figure K67: Analytical HPLC spectrum of reaction mixture derived from the treatment of hydroxythreonine-containing pentadecapeptide with $NaIO_4$ followed by addition of $NH_2OBn.HCl$. B=oxamate, O=oxime. The peaks at 10.80, 15.73, 16.07 and 23.15, 23.91 are impurities present in the starting material.

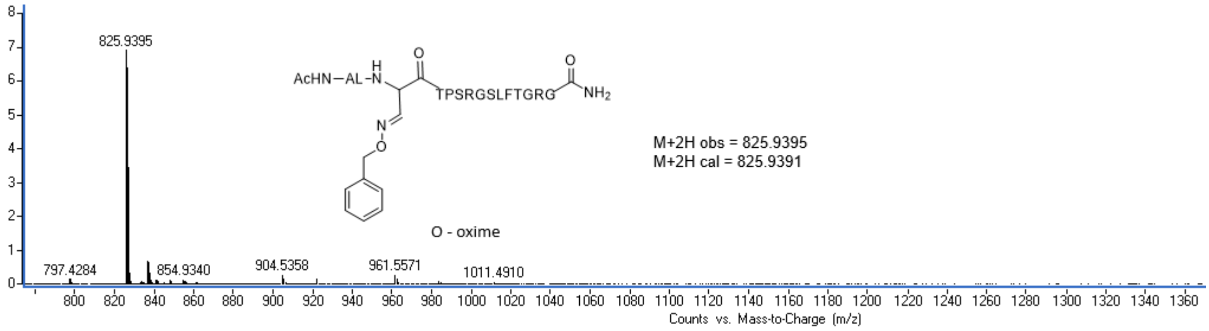

Figure K68: HRMS (ESI positive mode) spectrum of reaction mixture derived from the treatment of hydroxythreonine-containing pentadecapeptide with $NaIO_4$ followed by addition of $NH_2OBn.HCl$. Observed and calculated HRMS m/z peaks are given.

Photolysis of phenacylsulfide (33)

See general procedure, pg: 203

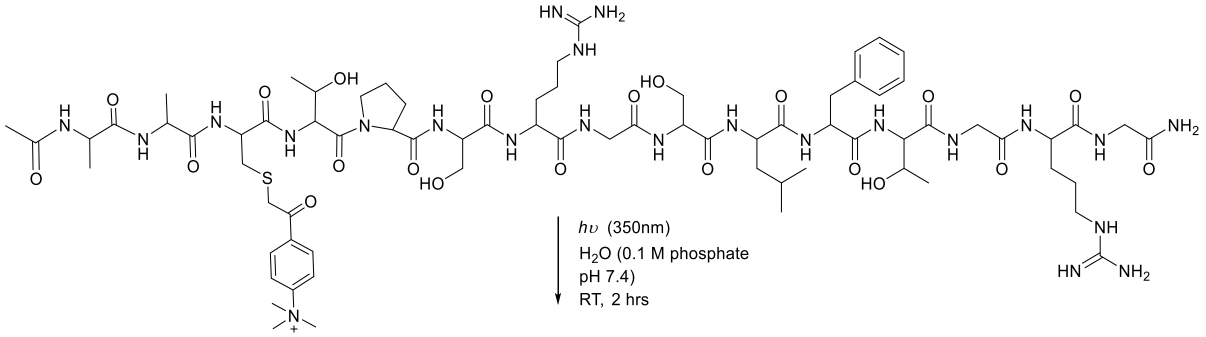

*Table K27: Analytical HPLC method used for characterisation of photolysis products (phenacylsulfide **33**, H_2O, 0.1 M phosphate, pH 7.4)*

time (min)	A (%)	B (%)
0	95	5
10	70	30
20	64	36
25	0	100
30	100	100
35	100	0

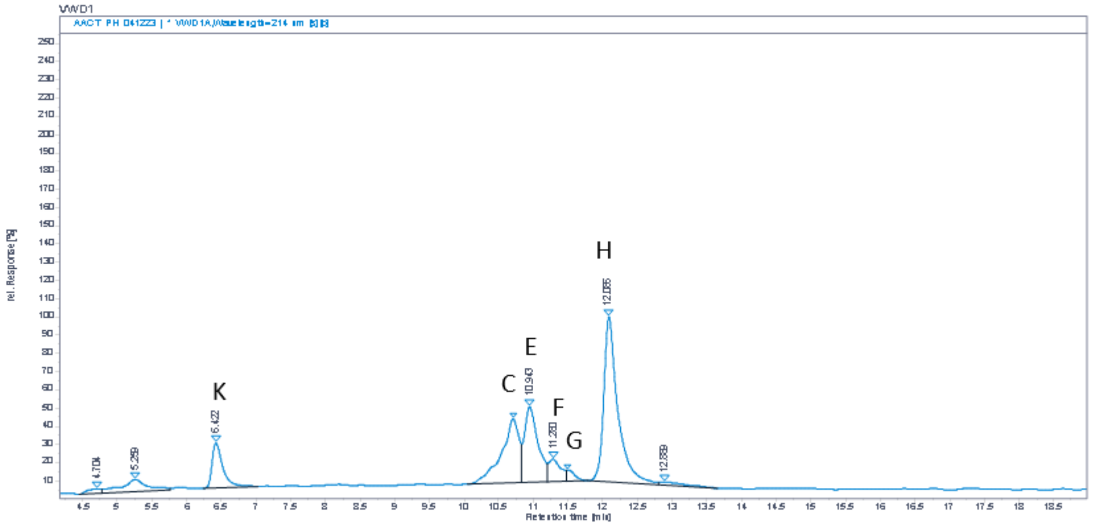

Figure K69: Analytical HPLC spectrum of photolysis reaction mixture where K=ketone, C= geminal diol +impurity present in starting material, E=isothiazolone, F=oxazole, G = thiazole (formed through cyclocondensation of enethiol in acidic HPLC eluent), H= disulfide.

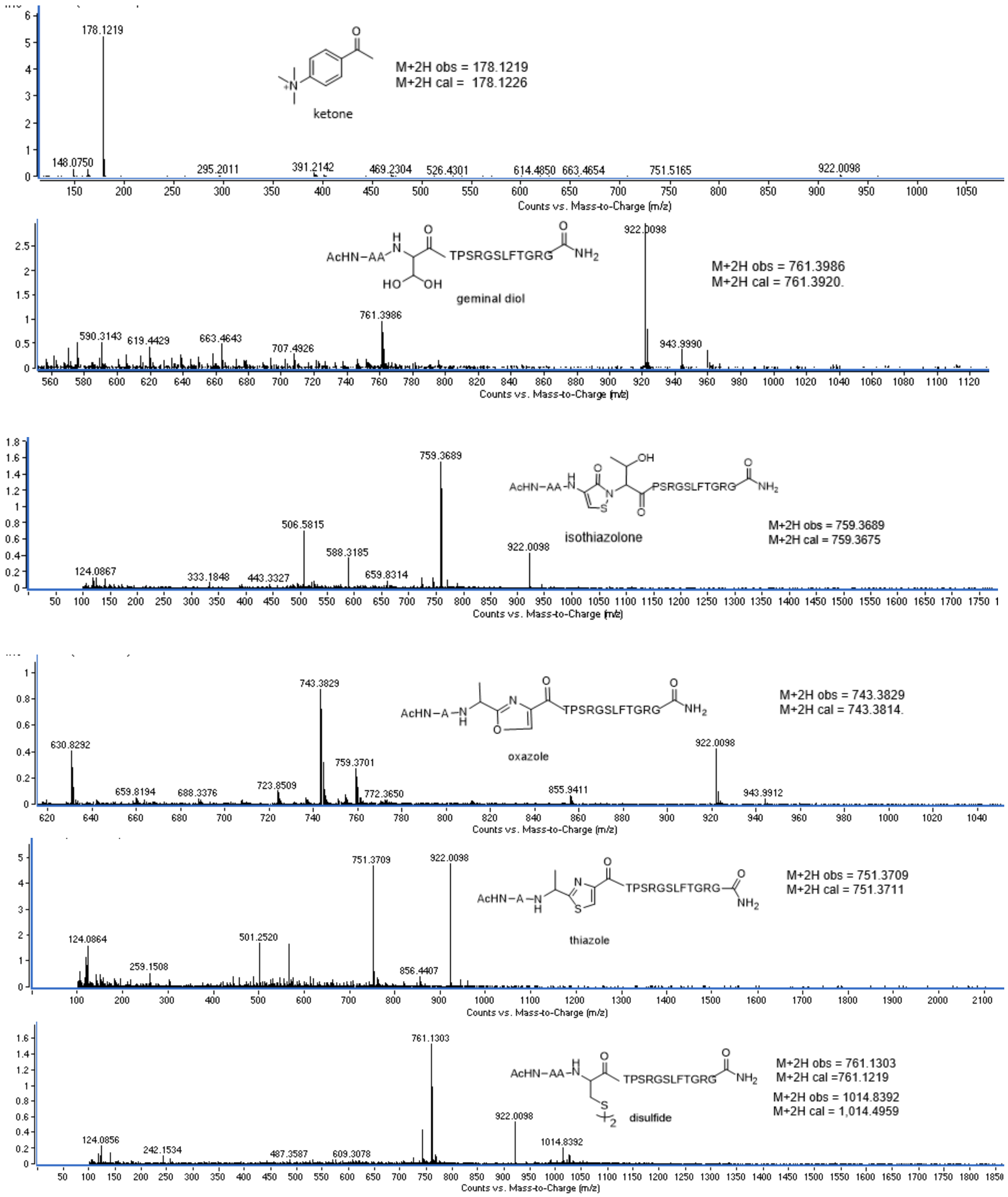

*Figure K70: HRMS (ESI positive mode) spectra of products derived from the photolysis of phenacylsulfide **33** (H_2O, 0.1 M phosphate, pH 7.4). Observed and calculated m/z peaks are provided.*

*Table K28: Approximate conversion values (%) of photolysis products (phenacylsulfide **33**, H_2O, 0.1 M phosphate, pH 7.4) based on integration of HPLC peaks (detection at 214 nm). Approximate conversions from phenacylsulfide (Norrish type-II and β-scission processes, column 2) and from putative thioaldehyde (Norrish type-II processes only, column 3).*

**Product**	**% Area (Conversion from phenacylsulfide)**	**% Area (Conversion from putative thioaldehyde)**
geminal diol	8%	18%
isothiazolone	30%	64%
oxazole	6%	13%
thiazole	2%	5%
disulfide	54%	

Photolysis of peptide phenacylsulfide (34)

See general procedure pg: 203

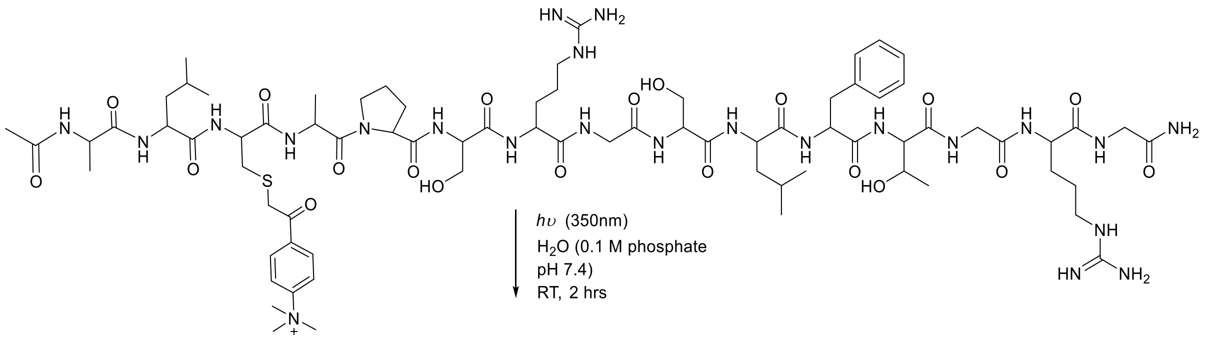

*Table K29: Analytical HPLC method used for characterisation of photolysis products (phenacylsulfide **34**, H_2O, 0.1 M phosphate, pH 7.4)*

time (min)	A (%)	B (%)
0	95	5
5	80	20
7	80	20
25	63	37
30	0	100
35	100	0

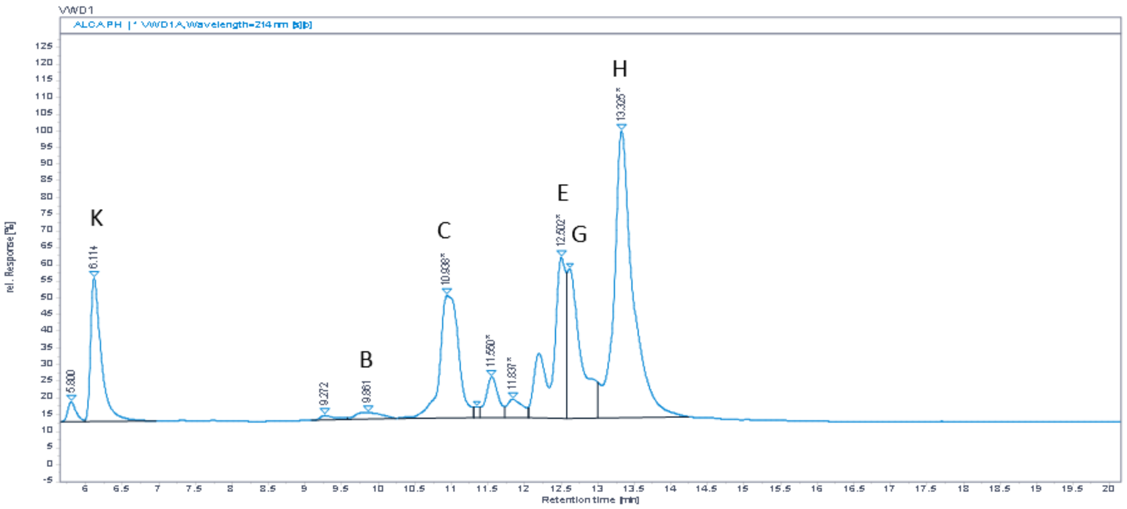

Figure K71: Analytical HPLC spectrum of photolysis reaction mixture where K=ketone, B=oxamate, C= geminal diol +impurity present in starting material, E=isothiazolone, G = thiazole (formed through cyclocondensation of enethiol in acidic HPLC eluent), H= disulfide. Peak at rt= 11.55, rt = 11.84 and 12.32 are impurities present in starting material.

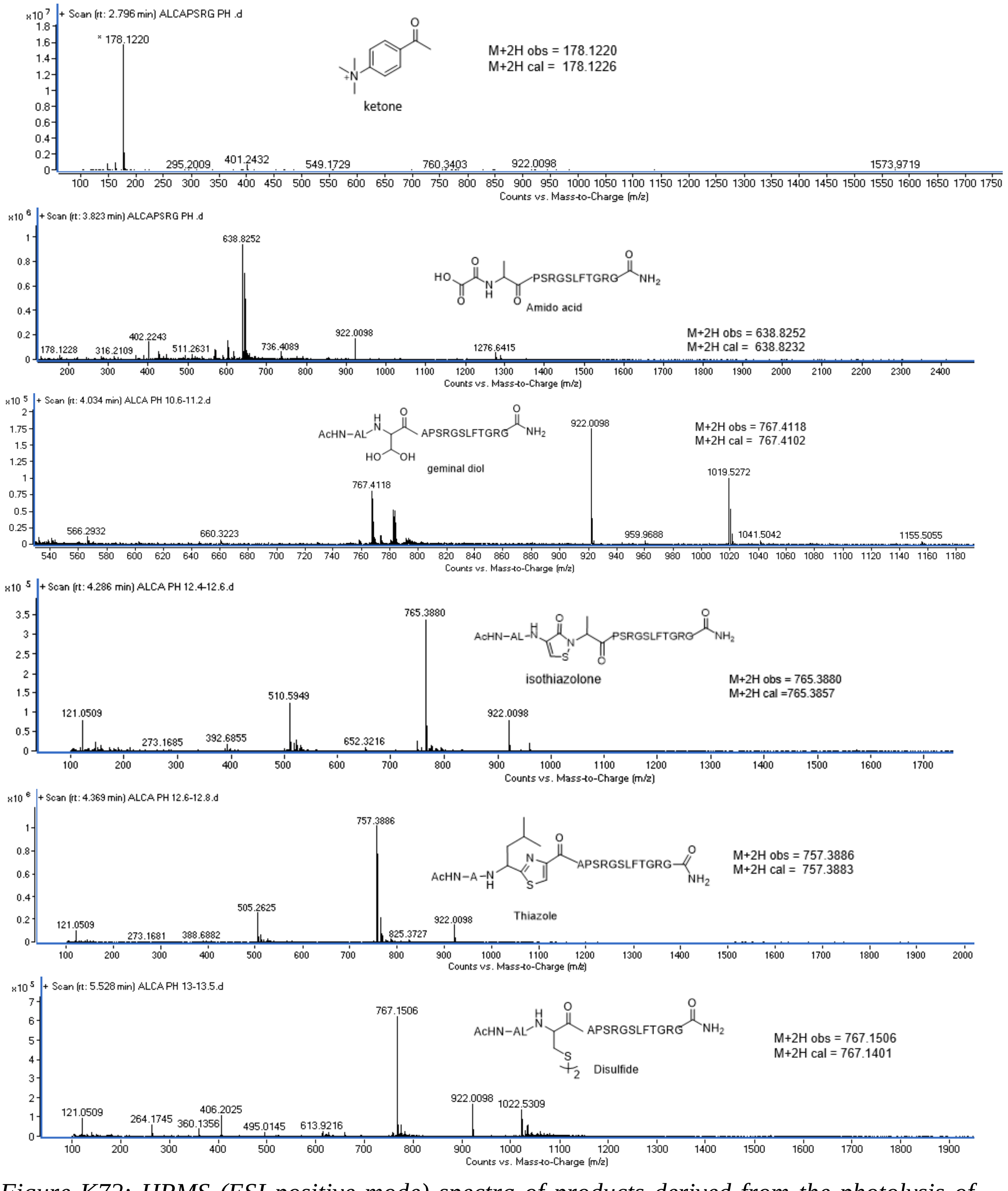

*Figure K72: HRMS (ESI positive mode) spectra of products derived from the photolysis of phenacylsulfide **34** (H_2O, 0.1 M phosphate, pH 7.4). Observed and calculated m/z peaks are provided.*

*Table K30: Approximate conversion values (%) of photolysis products (phenacylsulfide **34**, H_2O, 0.1 M phosphate, pH 7.4) based on integration of HPLC peaks (detection at 214 nm). Approximate conversions from phenacylsulfide (Norrish type-II and β-scission processes, column 2) and from putative thioaldehyde (Norrish type-II processes only, column 3).*

**product**	**% Area (Conversion from phenacylsulfide)**	**% Area (Conversion from putative thioaldehyde)**
oxamate	2%	5%
geminal diol	1%	3%
isothiazolone	18%	47%
thiazole	17%	45%
disulfide	62%	

Photolysis of peptide phenacylsulfide (35)

See general procedure pg: 203

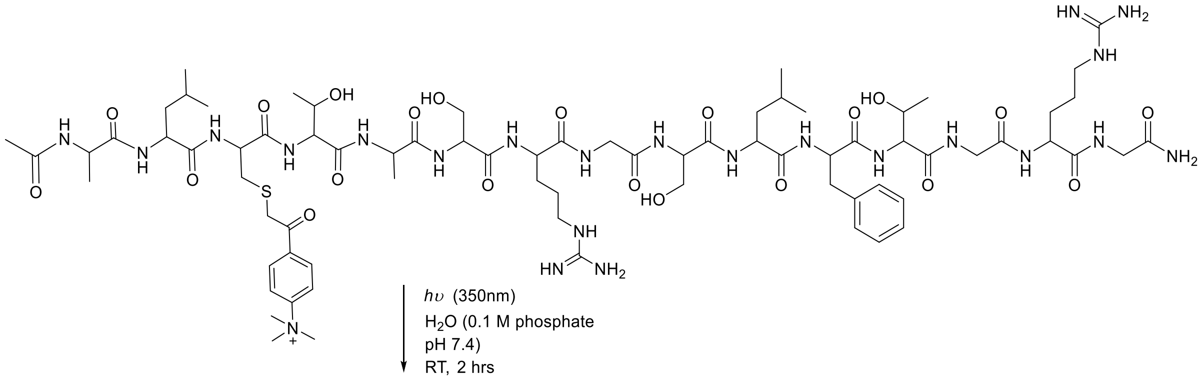

*Table K31: Analytical HPLC method used for characterisation of photolysis products (phenacylsulfide **35**, H_2O, 0.1 M phosphate, pH 7.4)*

time (min)	A (%)	B (%)
0	95	5
7	80	20
25	63	37
30	0	100
35	100	0
40	100	0

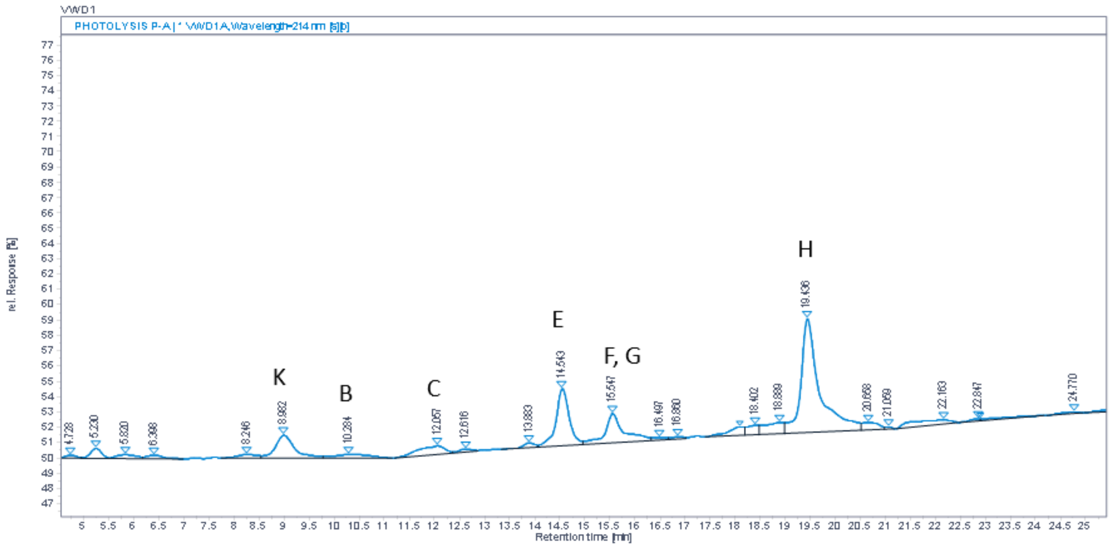

Figure K73: Analytical HPLC spectrum of photolysis reaction mixture where K=ketone, B=oxamate, C= geminal diol, E=isothiazolone, F=oxazole, G = thiazole (formed through cyclocondensation of enethiol in acidic HPLC eluent), H= disulfide.

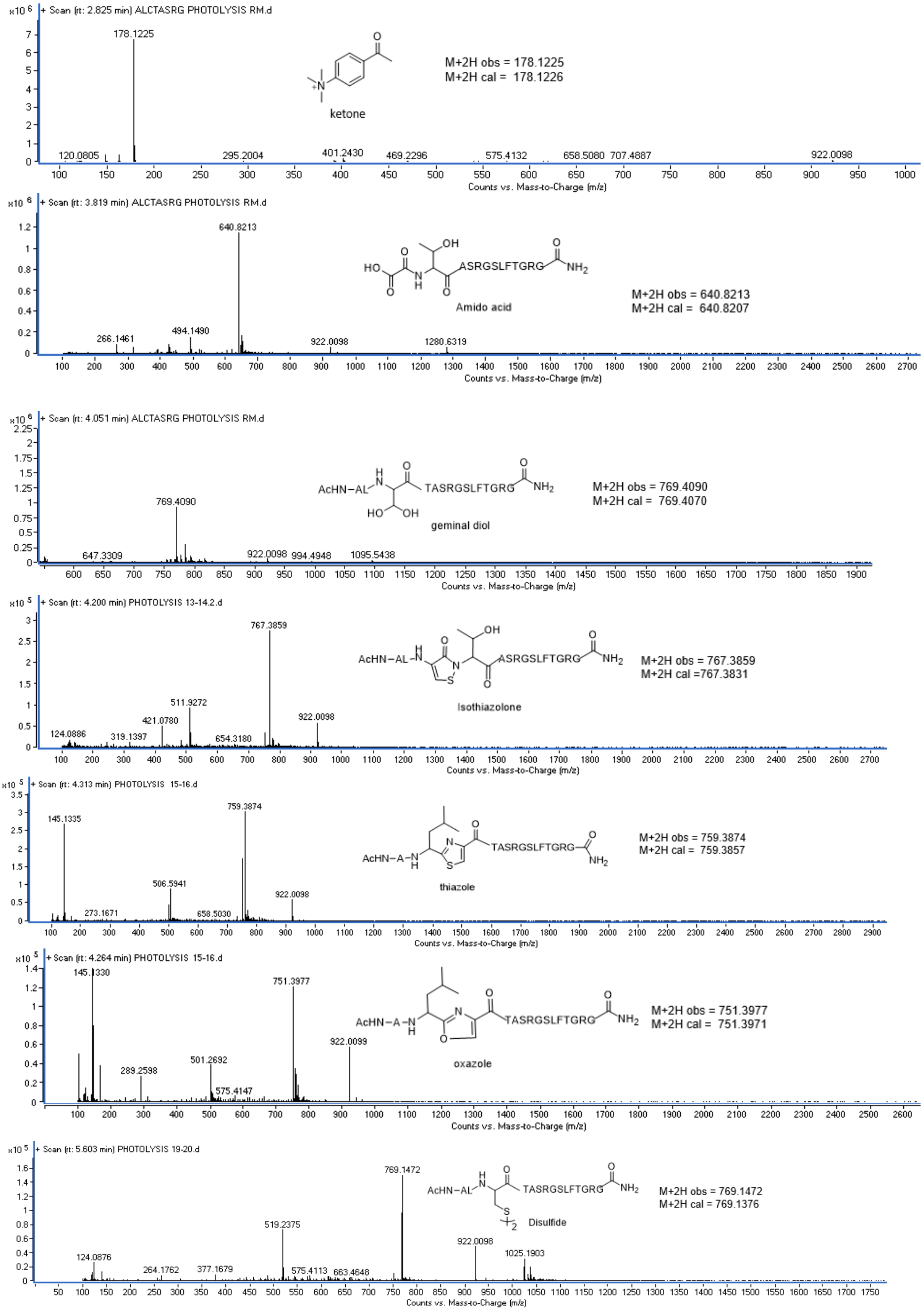

*Figure K74: HRMS (ESI positive mode) spectra of products derived from the photolysis of phenacylsulfide **35** (H_2O, 0.1 M phosphate, pH 7.4). Observed and calculated m/z peaks are provided.*

*Table K32: Approximate conversion values (%) of photolysis products (phenacylsulfide **35**, H_2O, 0.1 M phosphate, pH 7.4) based on integration of HPLC peaks (detection at 214 nm). Approximate conversions from phenacylsulfide (Norrish type-II and β-scission processes, column 2) and from putative thioaldehyde (Norrish type-II processes only, column 3).*

**product**	**% Area (Conversion from phenacylsulfide)**	**% Area (Conversion from putative thioaldehyde)**
oxamate	3%	8%
geminal diol	6%	15%
isothiazolone	18%	42%
thiazole	11%	25%
oxazole	4%	10%
disulfide	58%	

Synthesis of benzyl-vinyl sulfide

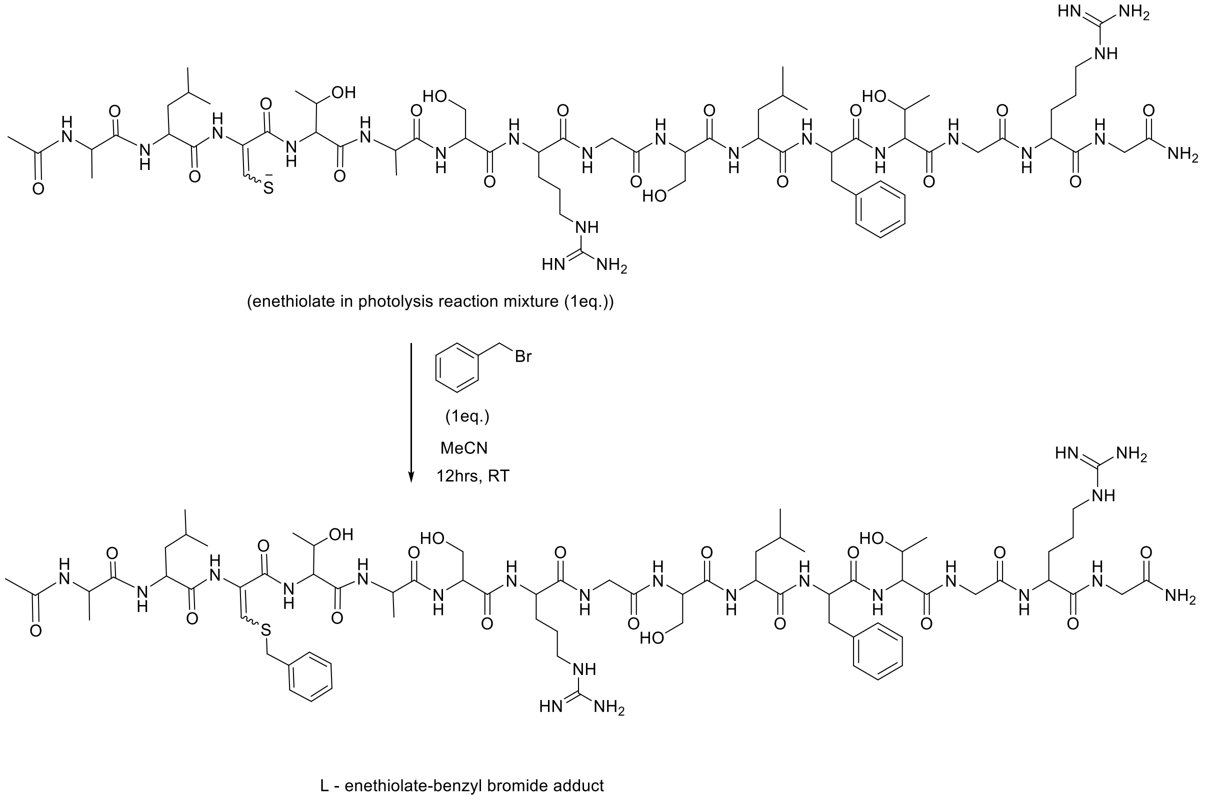

Benzyl bromide in acetonitrile (2 µL, 0.3 % v/v) was added to the photolysis reaction mixture derived from phenacylsulfide **35** (300 µL). The reaction mixture was allowed to evolve overnight under N_2 and was then analysed by analytical HPLC and HRMS (ESI positive mode). Approximate conversion percentages of each compounds based on integration of HPLC peaks *(detection at 214 nm)* were then calculated to determine the percentage conversion of oxazole in the photolysis reaction mixture.

*Table K33: Analytical HPLC method used for characterisation of photolysis products (phenacylsulfide **35**, H_2O, 0.1 M phosphate, pH 7.4)*

time (min)	A (%)	B (%)
0	95	5
7	80	20
25	63	37
30	0	100
35	100	0
40	100	0

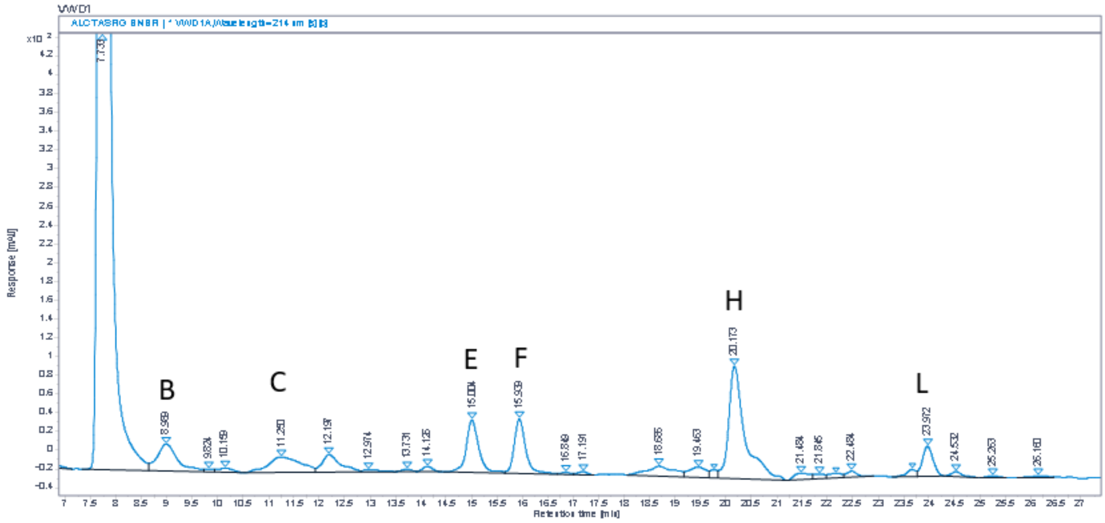

Figure K75: Analytical HPLC spectrum of photolysis reaction mixture after the addition of benzyl bromide. B=oxamate, C=geminal diol, E=isothiazolone, F=oxazole, H=disulfide, L = benzyl-vinyl sulfide. Note: the peak at 15.93 consist of F = oxazole and an impurity present in the starting material benzyl bromide. The area for this impurity is subtracted from the total area under the peak with retention time 15.93 to get the area corresponding to the oxazole, which was then used to find the approximate conversion percentage of oxazole.

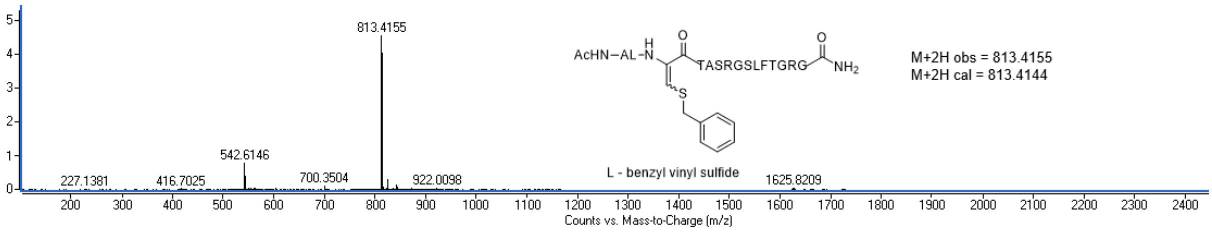

Figure K76: HRMS (ESI positive mode) spectrum of benzyl-vinyl sulfide. Observed and calculated m/z peaks are provided.

*Table K34: Approximate conversion values (%) of photolysis products (phenacylsulfide **35**, H_2O, 0.1 M phosphate, pH 7.4) after addition of benzyl bromide based on integration of HPLC peaks (detection at 214 nm). Approximate conversions from phenacylsulfide (Norrish type-II and β-scission processes, column 2) and from putative thioaldehyde (Norrish type-II processes only, column 3).*

**product**	**% Area (Conversion from phenacylsulfide)**	**% Area (Conversion from putative thioaldehyde)**
oxamate	3%	7%
geminal diol	10%	22%
isothiazolone	17%	38%
oxazole	4%	10%
disulfide	56%	
benzyl vinyl sulfide	10%	23%

Photolysis of peptide phenacylsulfide (36)

See general procedure pg: 203

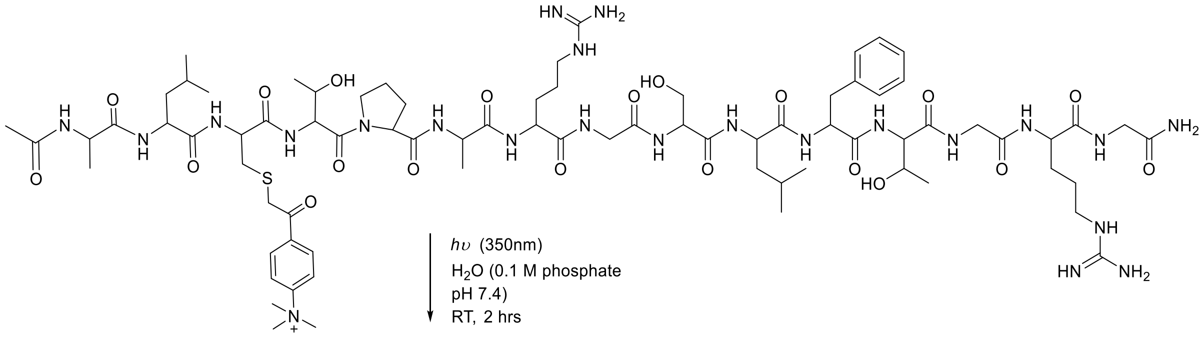

*Table K35: Analytical HPLC method used for characterisation of photolysis products (phenacylsulfide **36**, H_2O, 0.1 M phosphate, pH 7.4)*

time (min)	A (%)	B (%)
0	95	5
10	70	30
20	64	36
25	0	100
30	100	0
35	100	0

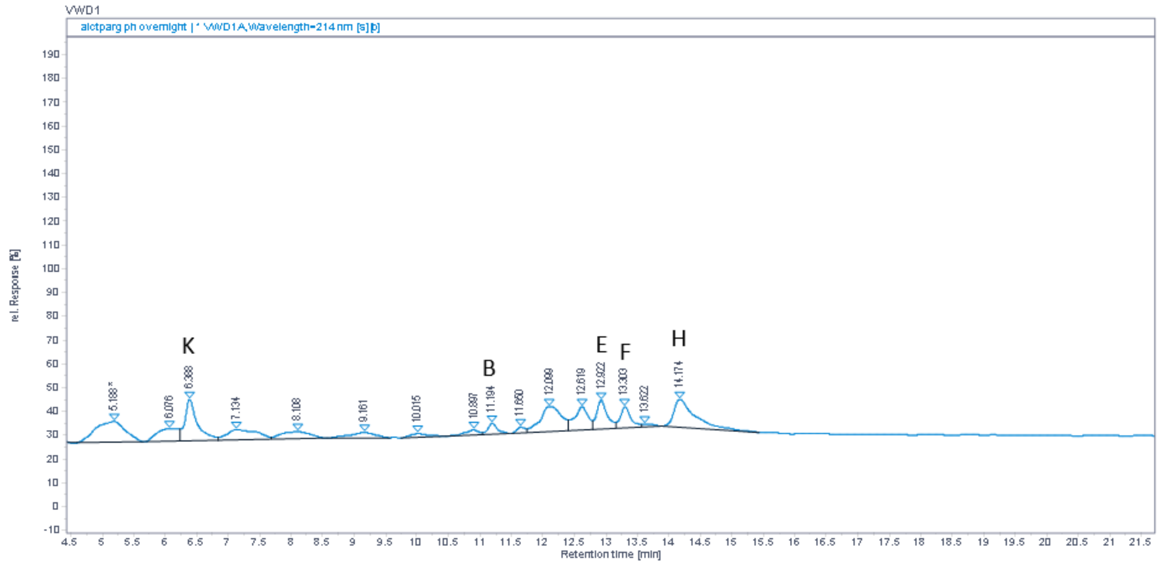

Figure K77: Analytical HPLC spectrum of photolysis reaction mixture where K=ketone, B=oxamate, E=isothiazolone, F=oxazole, H= disulfide. Peak at 12.09, 12.61 are impurities present in the starting material.

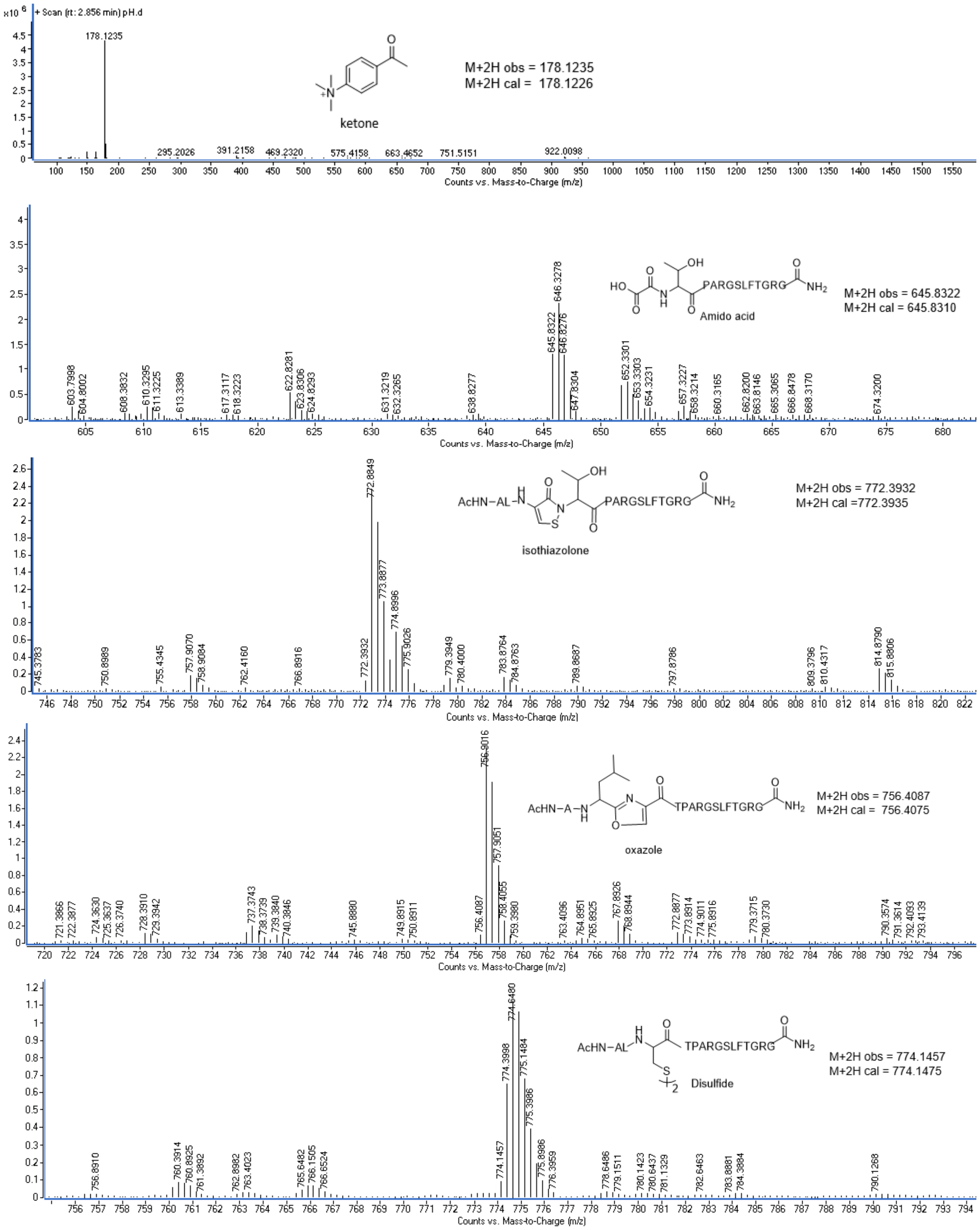

Figure K78: HRMS (ESI positive mode) spectra of products derived from the photolysis of phenacylsulfide ***36*** *(H_2O, 0.1 M phosphate, pH 7.4). Observed and calculated m/z peaks are provided.*

Table K36: Approximate conversion values (%) of photolysis products (phenacylsulfide ***36****, H_2O, 0.1 M phosphate, pH 7.4) based on integration of HPLC peaks (detection at 214 nm). Approximate conversions from phenacylsulfide (Norrish type-II and β-scission processes, column 2) and from putative thioaldehyde (Norrish type-II processes only, column 3).*

**product**	**% Area (Conversion from phenacylsulfide)**	**% Area (Conversion from putative thioaldehyde)**
oxamate	9%	17%
isothiazolone	24%	51%
oxazole	16%	32%
disulfide	51%	

Photolysis of peptide phenacylsulfide (37)
See general procedure pg: 203

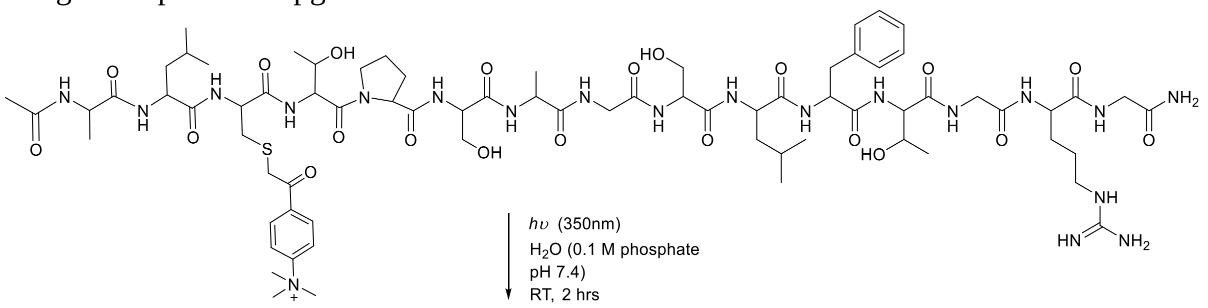

*Table K37: Analytical HPLC method used for characterisation of photolysis products (phenacylsulfide **37**, H_2O, 0.1 M phosphate, pH 7.4)*

time (min)	A (%)	B (%)
0	95	5
5	80	20
7	76	24
35	73	27
40	70	30
45	75	25

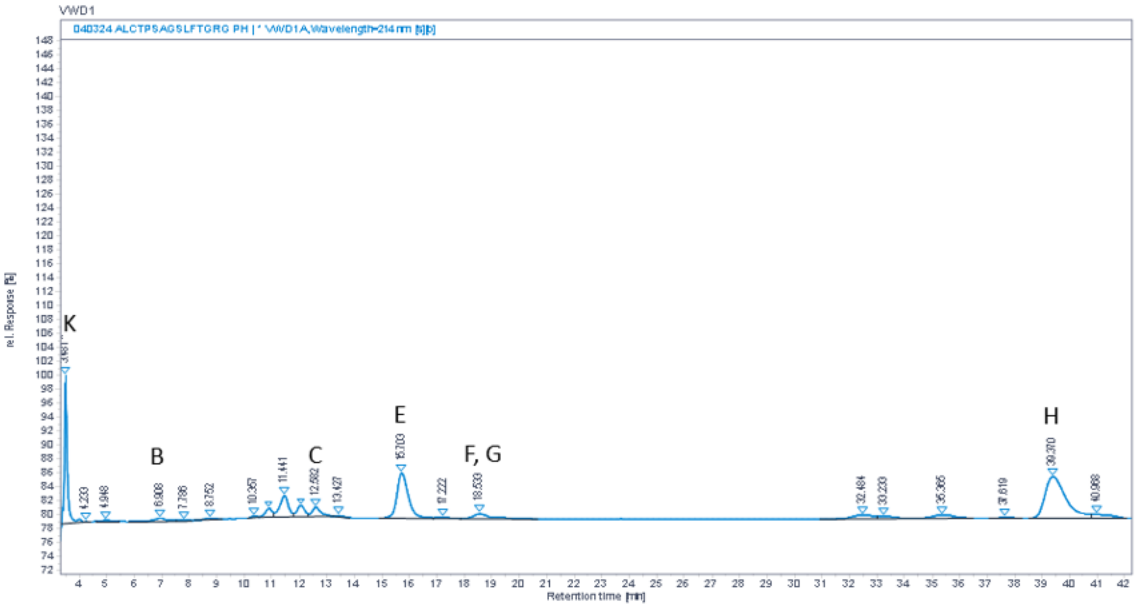

Figure K79: Analytical HPLC spectrum of photolysis reaction mixture where K=ketone, B=oxamate, C= geminal diol, E=isothiazolone, F=oxazole, G = thiazole (formed through cyclocondensation of enethiol in acidic HPLC eluent), H= disulfide. Peak from 10.35–12.23 are impurities present in starting material.

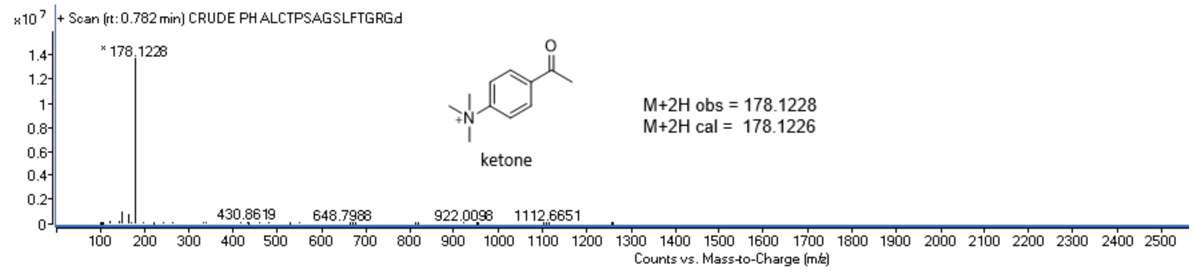
+ Scan (rt: 0.782 min) CRUDE PH ALCTPSAGSLFTGRG.d
* 178.1228
430.8619
648.7988
922.0098
1112.6651
ketone
M+2H obs = 178.1228
M+2H cal = 178.1226
Counts vs. Mass-to-Charge (m/z)

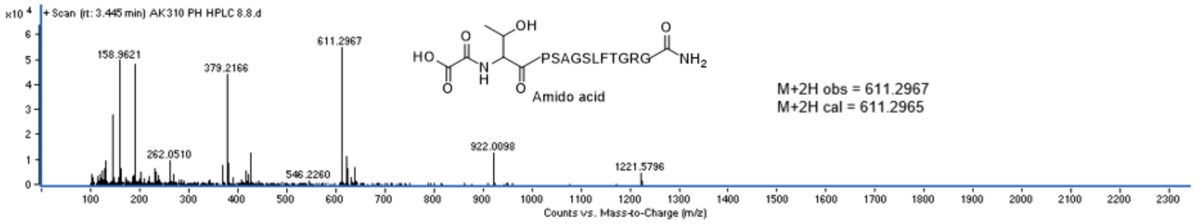
+ Scan (rt: 3.445 min) AK310 PH HPLC 8.8.d
158.9621
379.2166
611.2967
262.0510
546.2260
922.0098
1221.5796
Amido acid
M+2H obs = 611.2967
M+2H cal = 611.2965
Counts vs. Mass-to-Charge (m/z)

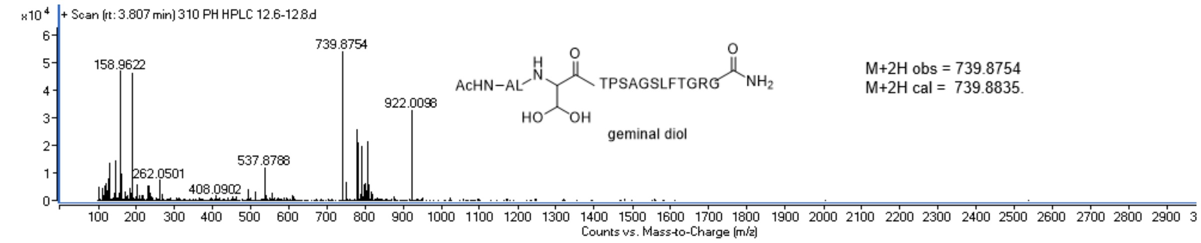
+ Scan (rt: 3.807 min) 310 PH HPLC 12.6-12.8.d
158.9622
739.8754
922.0098
537.8788
262.0501
408.0902
geminal diol
M+2H obs = 739.8754
M+2H cal = 739.8835.
Counts vs. Mass-to-Charge (m/z)

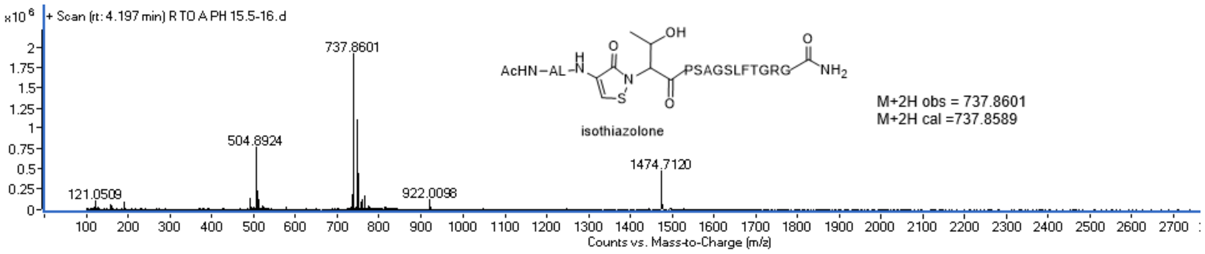
+ Scan (rt: 4.197 min) R TO A PH 15.5-16.d
737.8601
504.8924
1474.7120
121.0509
922.0098
isothiazolone
M+2H obs = 737.8601
M+2H cal =737.8589
Counts vs. Mass-to-Charge (m/z)

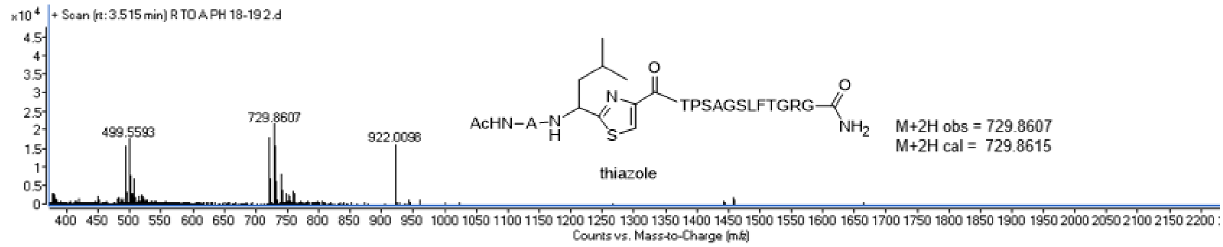
+ Scan (rt: 3.515 min) R TO A PH 18-19.2.d
499.5593
729.8607
922.0098
thiazole
M+2H obs = 729.8607
M+2H cal = 729.8615
Counts vs. Mass-to-Charge (m/z)

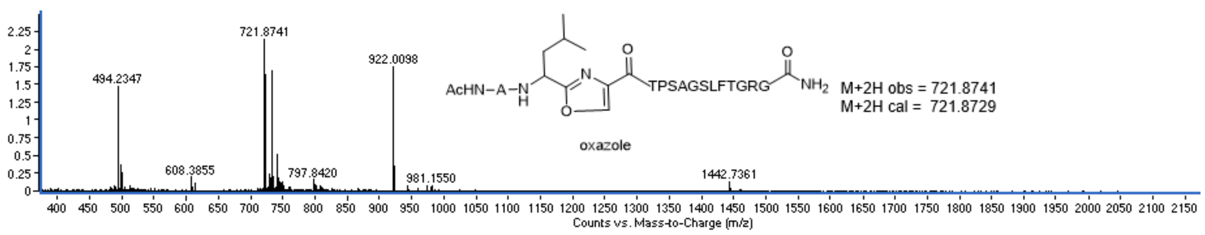
721.8741
922.0098
494.2347
608.3855
797.8420
981.1550
1442.7361
oxazole
M+2H obs = 721.8741
M+2H cal = 721.8729
Counts vs. Mass-to-Charge (m/z)

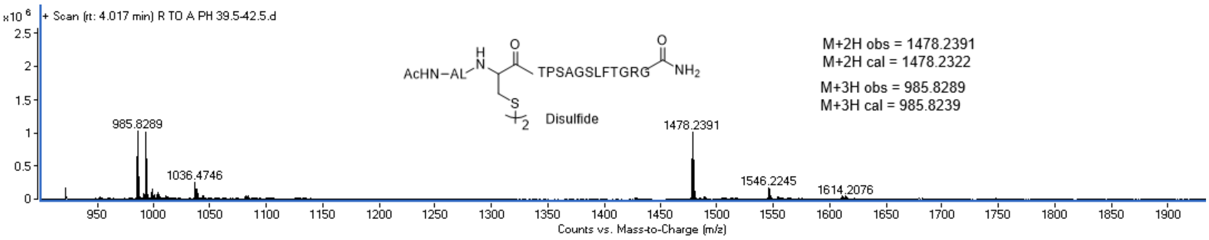

*Figure K80: HRMS (ESI positive mode) spectra of products derived from the photolysis of phenacylsulfide **37** (H_2O, 0.1 M phosphate, pH 7.4). Observed and calculated m/z peaks are provided.*

*Table K38: Approximate conversion values (%) of photolysis products (phenacylsulfide **37**, H_2O, 0.1 M phosphate, pH 7.4) based on integration of HPLC peaks (detection at 214 nm). Approximate conversions from phenacylsulfide (Norrish type-II and β-scission processes, column 2) and from putative thioaldehyde (Norrish type-II processes only, column 3).*

**product**	**% Area (Conversion from phenacylsulfide)**	**% Area (Conversion from putative thioaldehyde)**
oxamate	1%	3%
geminal diol	3%	6%
isothiazolone	33%	78%
thiazole	4%	9%
oxazole	2%	4%
disulfide	57%	

Photolysis in the presence of guanidinium hydrochloride

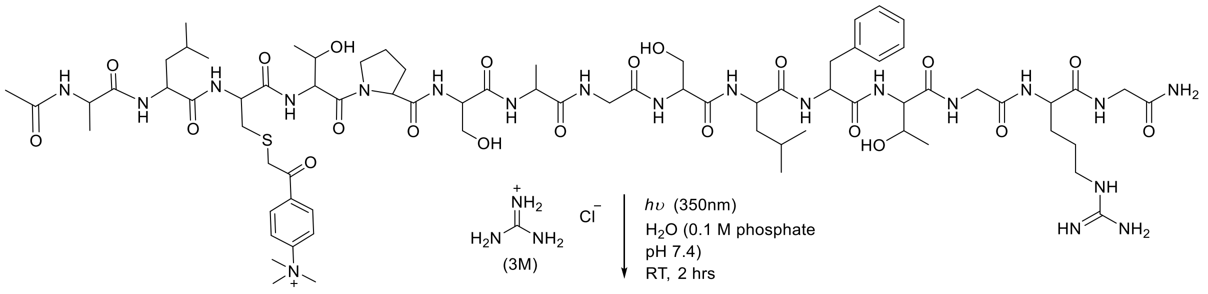

Guanidinium hydrochloride (287 mg, 3 mmol) was added to a solution of phenacylsulfide ***37*** (3 mg, 0.0017 mmol) in H_2O (0.1 M phosphate, pH 7.4, 1 mL, 2 mM solution). The solution was then degassed with N_2 for 20 mins and illumination at 350 nm in a Rayonet photoreactor for 2 hours. The reaction mixture was analysed by analytical HPLC and HRMS (ESI positive mode).

*Table K39: Analytical HPLC method used for characterisation of photolysis products (phenacylsulfide **37**, H_2O, 0.1 M phosphate, 3M Gn.HCl, pH 7.4)*

time (min)	A (%)	B (%)
0	95	5
5	80	20
7	76	24
35	73	27
40	70	30
45	75	25

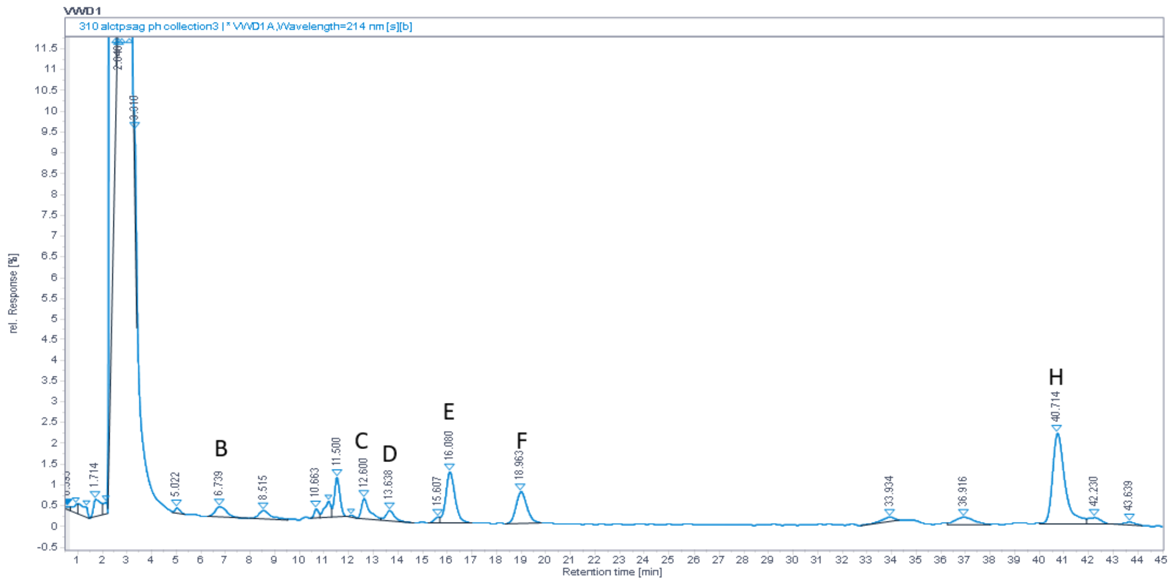

Figure K81: Analytical HPLC spectrum of photolysis reaction mixture where B=oxamate, C= geminal diol, D = formylglycine, E=isothiazolone, F=oxazole, H= disulfide.

*Table K40: Approximate conversion values (%) of photolysis products (phenacylsulfide **37**, H_2O, 0.1 M phosphate, 3M Gn.HCl, pH 7.4) based on integration of HPLC peaks (detection at 214 nm). Approximate conversions from phenacylsulfide (Norrish type-II and β-scission processes, column 2) and from putative thioaldehyde (Norrish type-II processes only, column 3).*

**product**	**% Area (Conversion from phenacylsulfide)**	**% Area (Conversion from putative thioaldehyde)**
oxamate	3%	7%
geminal diol	7%	13%
formylglycine	5%	8%
isothiazolone	19%	37%
thiazole	4%	7%
oxazole	14%	28%
disulfide	48%	

Stability of thio-oxazoline [TO]

The stability of thio oxazoline formed in the reaction mixture was investigated by analysing the evolution of oxazole present in the reaction mixture over a duration of 14 days. It was observed that the thio oxazoline formed in the reaction was stable and does not undergo ring opening to the thioaldehyde, since the yield of oxazole remained the same after 14 days. The oxazole formed in the reaction mixture was then isolated from the analytical hplc followed by freezedrying the purified sample. The sample was then redissolved in D_2O and analysed by ^1H NMR.

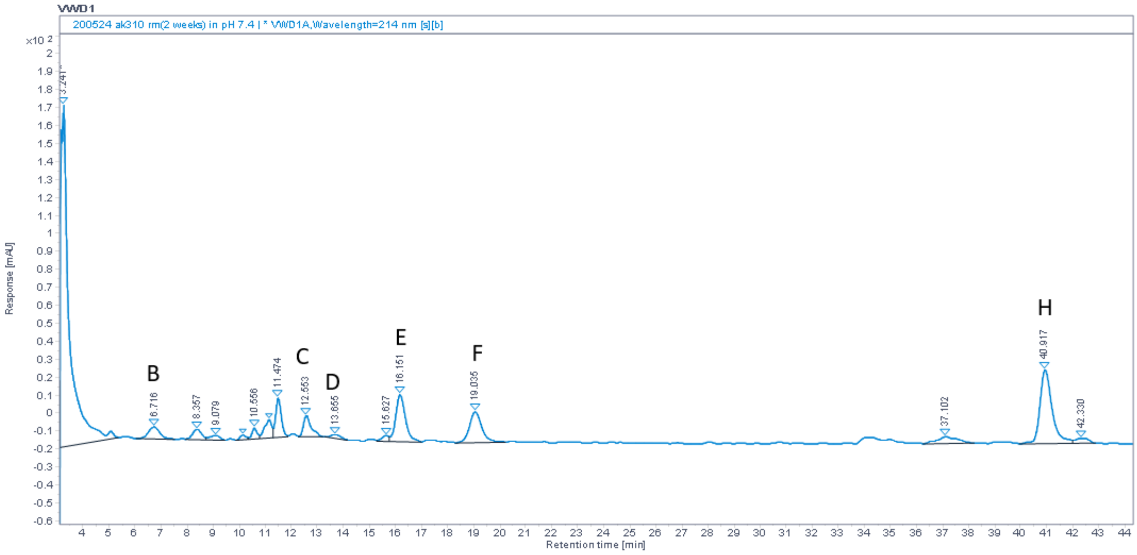

Figure K82: Analytical HPLC spectrum of photolysis reaction mixture where B=oxamate, C= geminal diol, D = formylglycine, E=isothiazolone, F=oxazole, H= disulfide.

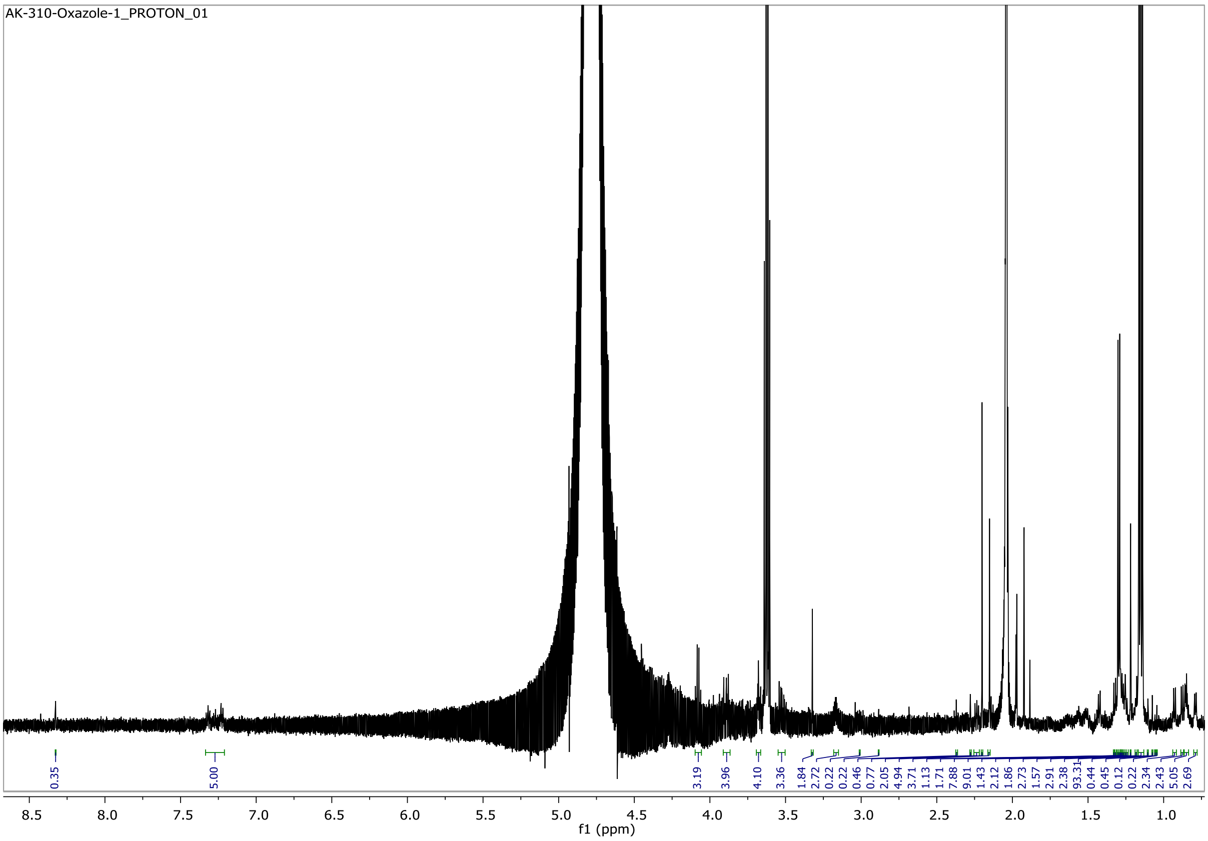

Figure K83: ^{1}H NMR spectrum of oxazole in D_2O

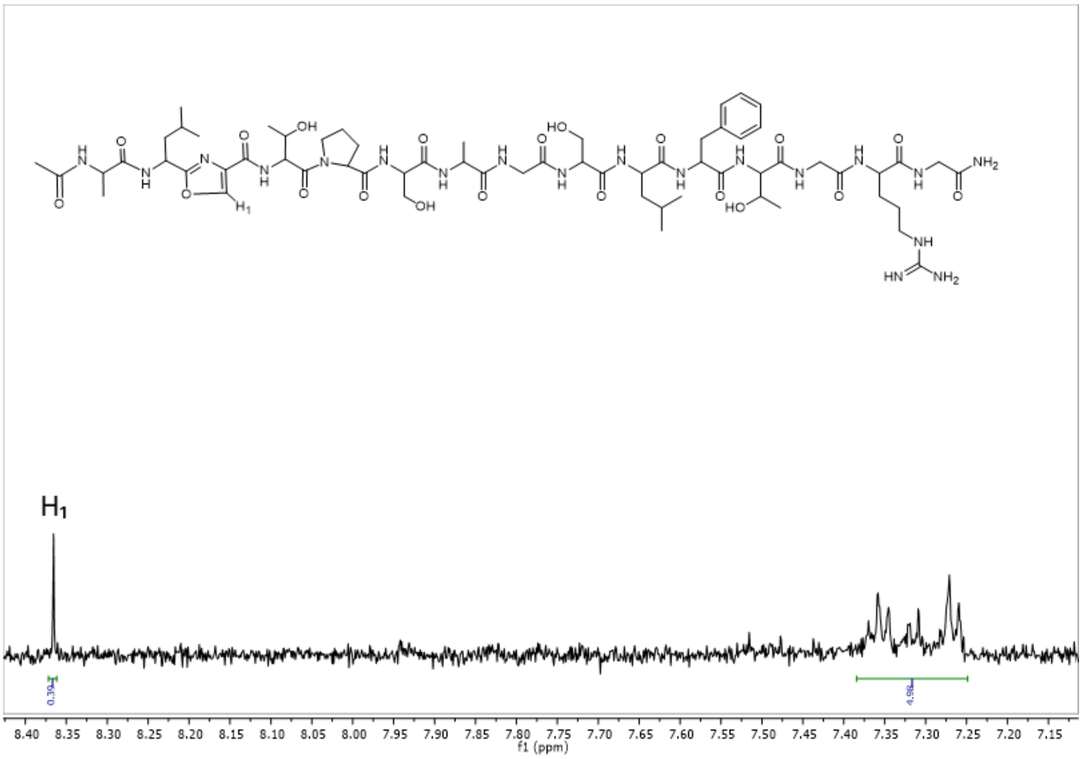

Figure K84: Zoomed in region of ^1H NMR spectrum of oxazole in D_2O showing the characteristic peaks. The methine proton of oxazole is appearing at 8.33 ppm. The region from 7.25–7.36 ppm contains the aromatic C-H protons of phenyl alanine residue.

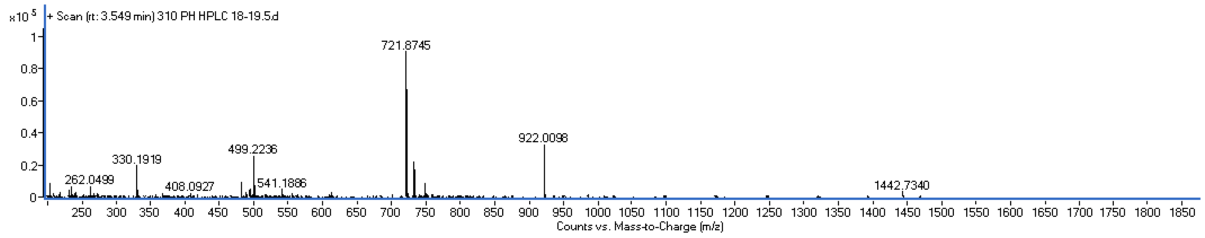

Figure K85: HRMS (ESI) spectrum of oxazole showing the m/z $(M/2+H)^+$ (calcd. 721.8729, observed 721.8745) adduct.

Synthesis of octapeptide (38a)

Peptide was synthesised through SPPS general procedure outlined in section I.

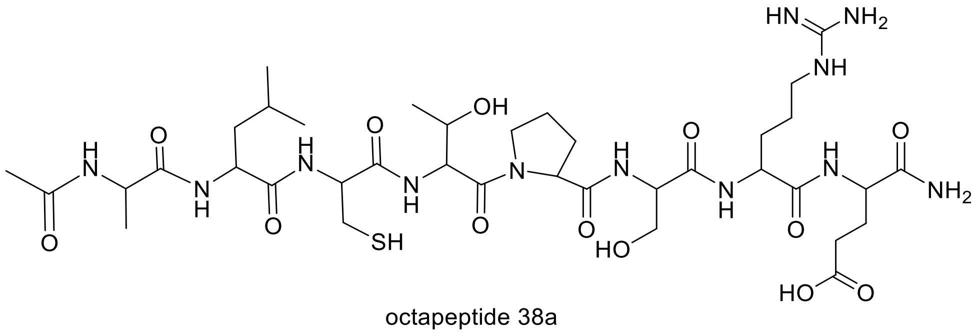

octapeptide 38a

*Table K41: Analytical HPLC method used for characterisation of octapeptide **38a***

time (min)	A (%)	B (%)
0	95	5
15	80	20
20	80	20
35	5	95
40	95	5

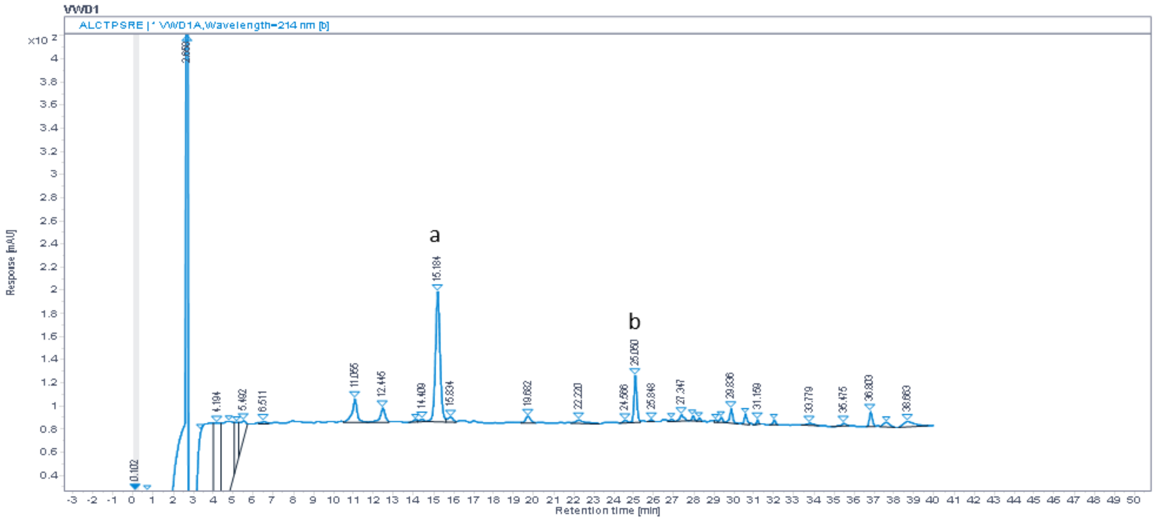

*Figure K86: Analytical HPLC spectrum of peptide thiol **38a**, where a = octapeptide **38a**, b= tert butyl protected cysteine derivative of octapeptide **38a**.*

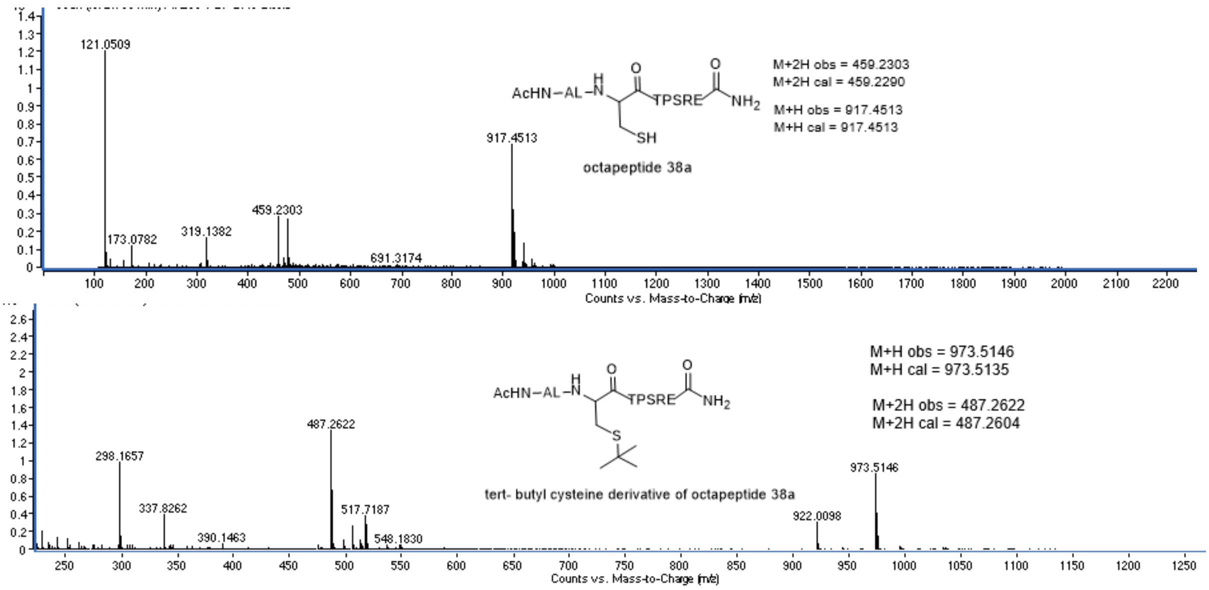

*Figure K87: HRMS (ESI positive mode) spectra of a=octapeptide **38a** and b= tert butyl protected cysteine derivative of octapeptide **38a**. Observed and calculated m/z peaks are provided.*

Synthesis and photolysis of phenacylsulfide (38)

Octapeptide ***38a*** (5 mg, 0.005 mmol) was dissolved in H_2O (0.1 M phosphate, pH 7.4, 0.5 mL, 5 mM) followed by addition of a solution of *p*-trimethylammonium phenacyl bromide (2 mg, 0.005 mmol) in H_2O (0.1 M phosphate, pH 7.4, 0.5 mL). The reaction mixture was stirred overnight. The target phenacylsulfide ***38*** was analysed through HPLC-MS. The alkylated peptide reaction mixture was degassed by passing N_2 gas through the solution for 20 mins. The solution was then illuminated in a Rayonet photo reactor with a light of wavelength 350 nm for 2 hrs at room temperature. Products formed in the reaction mixture was analysed by HPLC-MS**.**

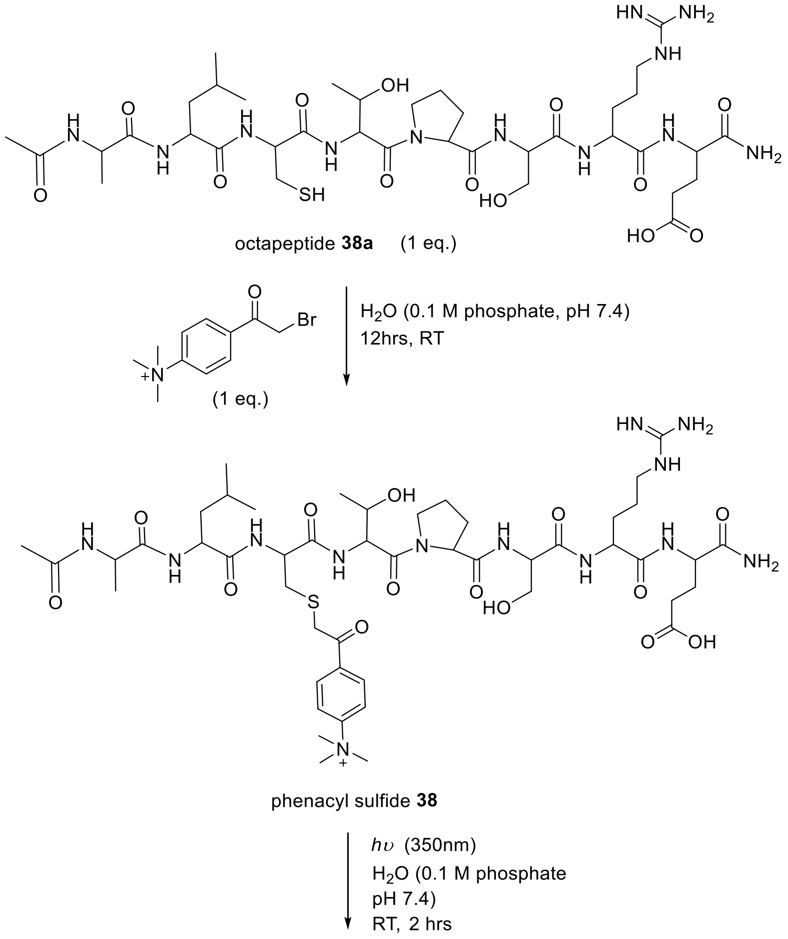

*Table K42: Analytical HPLC method used for characterisation of phenacylsulfide **38***

**time (min)**	**A (%)**	**B (%)**
0	95	5
15	80	20
20	80	20
35	5	95
40	95	5

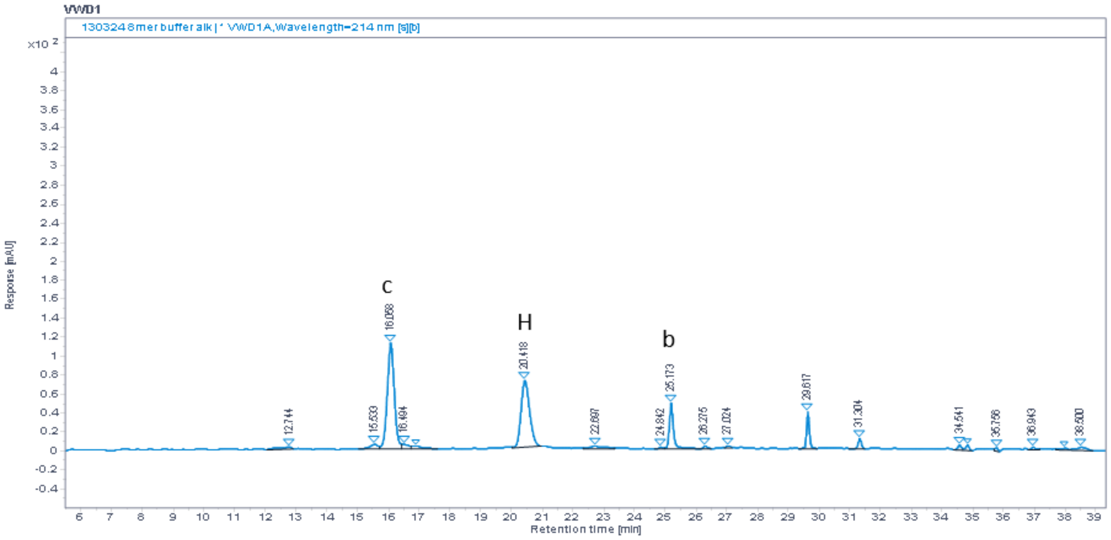

*Figure K88: Analytical HPLC spectrum of peptide alkylation of octapeptide **38a** in H_2O (0.1 M phosphate, pH 7.4, 0.5 mL, 5 mM) where c = phenacylsulfide **38**, H = disulfide, b = tert butyl protected cysteine derivative of the octapeptide **38a**.*

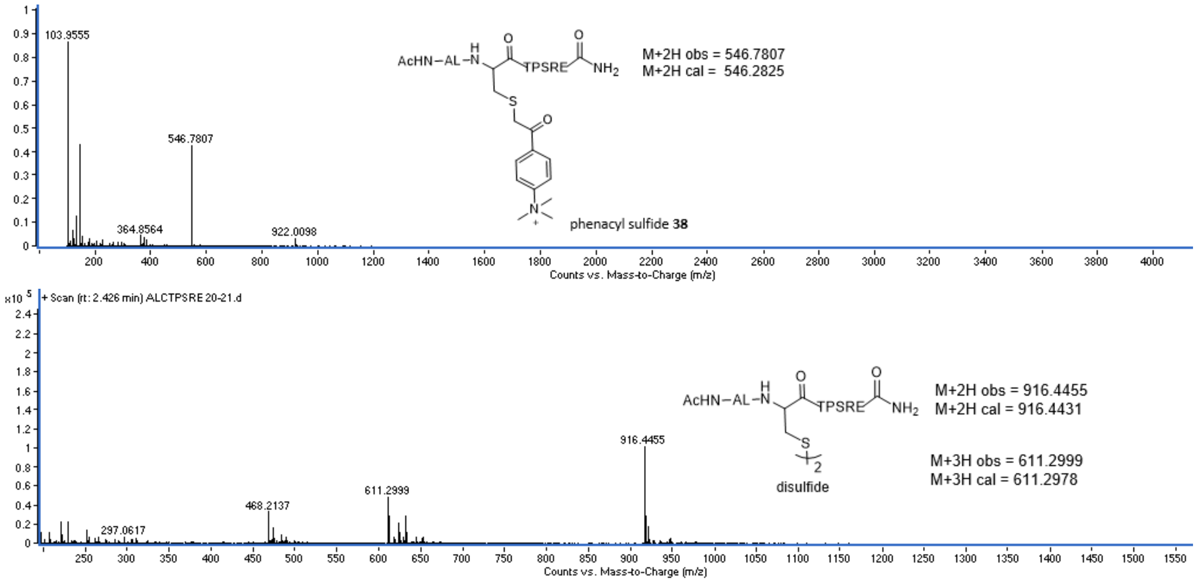

Figure K89: HRMS (ESI positive mode) spectra of peptide alkylation. Observed and calculated m/z peaks are provided.

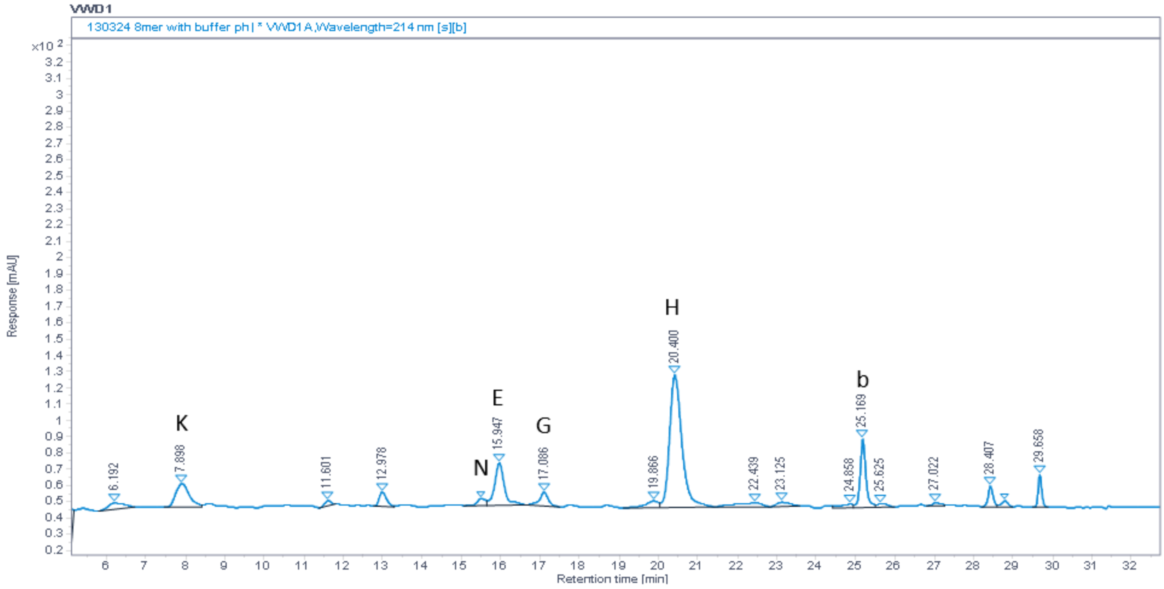

Figure K90: Analytical HPLC spectrum of photolysis reaction mixture of phenacylsulfide ***38*** *in H_2O (0.1 M phosphate, pH 7.4, 0.5 mL, 5 mM), where K=ketone, N=enethiolate, E=isothiazolone, G=thiazole (formed through cyclocondensation of enethiol in acidic HPLC eluent), H=disulfide, b= tert butyl protected cysteine derivative of octapeptide* ***38a****.*

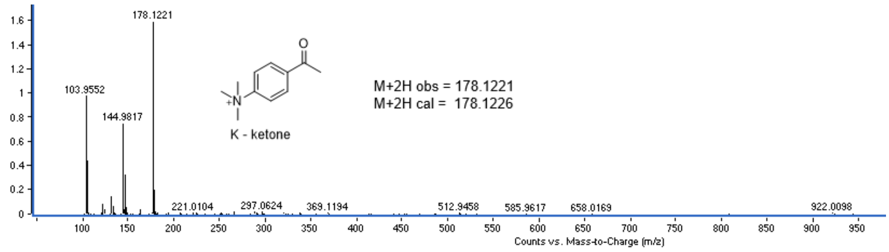

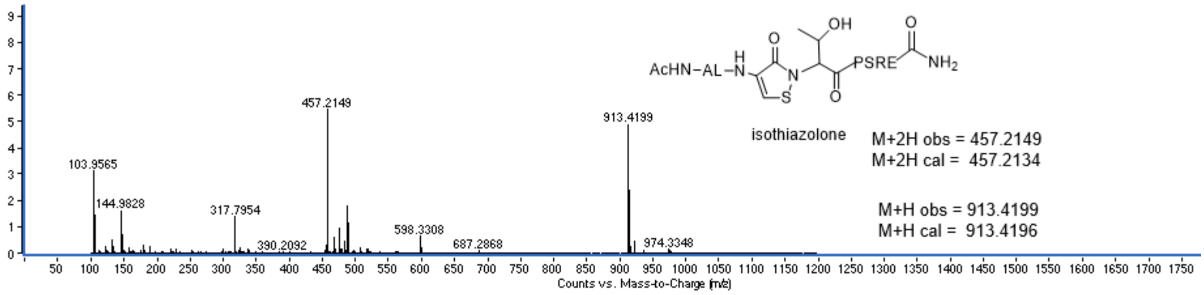

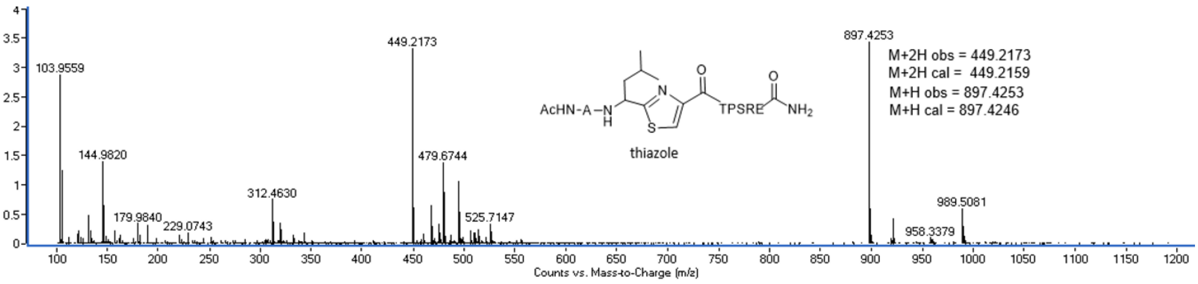

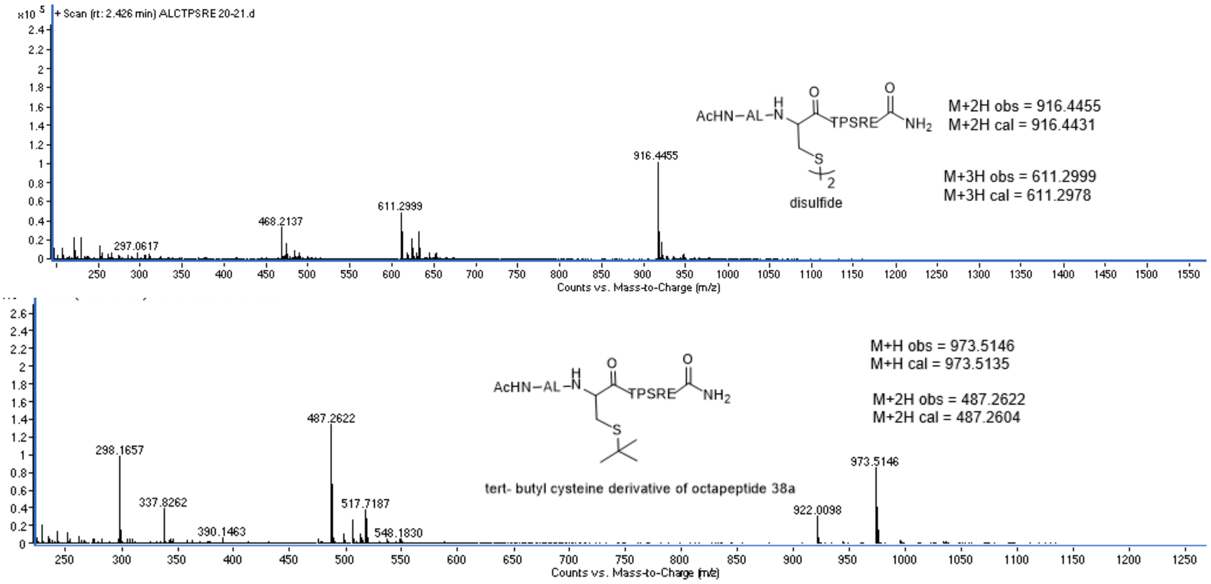

*Figure K91: HRMS (ESI positive mode) spectra of photolysis reaction mixture of phenacylsulfide **38** in H_2O (0.1 M phosphate, pH 7.4, 0.5 mL, 5 mM). Observed and calculated m/z peaks are provided.*

*Table K43: Approximate conversion values (%) of photolysis products (phenacylsulfide **38**, H_2O, 0.1 M phosphate, pH 7.4) based on integration of HPLC peaks (detection at 214 nm). Approximate conversions from phenacylsulfide (Norrish type-II and β-scission processes, column 2) and from putative thioaldehyde (Norrish type-II processes only, column 3).*

**product**	**% Area (Conversion from phenacylsulfide)**	**% Area (Conversion from putative thioaldehyde)**
enethiolate	6%	11%
isothiazolone	37%	68%
thiazole	11%	21%
disulfide	46%	

Reaction in non-buffered H_2O (pH 9 initial)

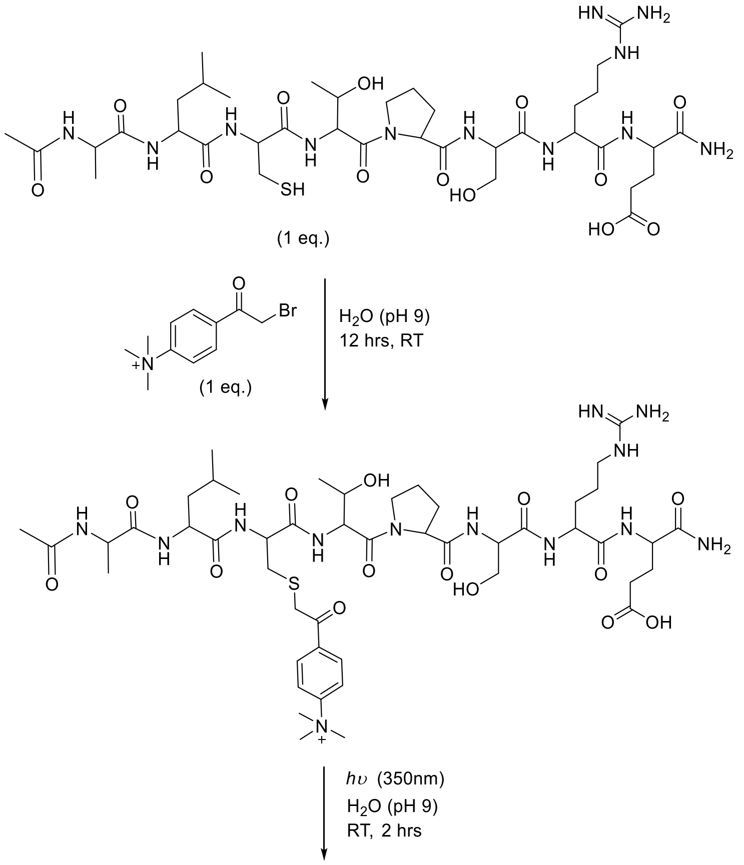

Octapeptide ***38a*** (5 mg, 0.005 mmol) was dissolved in H_2O (0.8 mL, 6 mM) followed by addition of a solution of *p*-trimethylammonium phenacyl bromide (2 mg, 1 eq.) in H_2O (0.2 mL). pH of the solution was adjusted to 9 using 1M NaOH solution. The alkylation reaction mixture was kept overnight and analysed through HPLC-MS. On completion of alkylation, the reaction mixture was degassed by passing N_2 gas for 20 mins. The solution was then illuminated in a Rayonet photo reactor with a light of wavelength 350 nm for 2 hrs at room temperature. The pH of the reaction mixture after photolysis was measured to be 7.8. Products formed in the reaction mixture was analysed by HPLC-MS.

*Table K44: Analytical HPLC method used for characterisation of phenacylsulfide **38** and photolysis products (phenacylsulfide **38**, H_2O, pH initial 9, pH final 7.8)*

time (min)	A (%)	B (%)
0	95	5
15	80	20
20	80	20
35	5	95
40	95	5

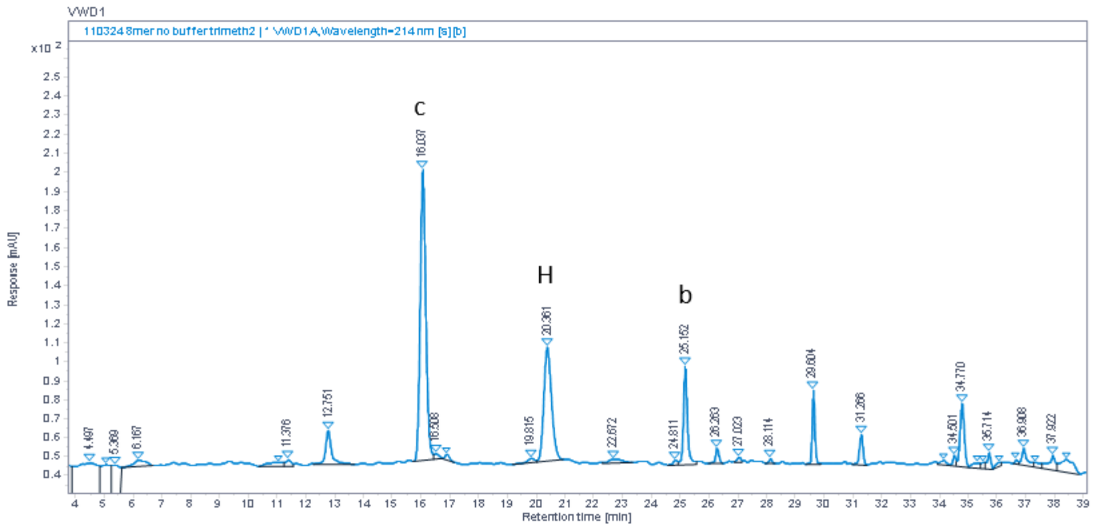

*Figure K92: Analytical HPLC spectrum of peptide alkylation of octapeptide **38a** in H_2O (0.8 mL, 6 mM), where c = phenacylsulfide **38**, H = disulfide, b = tert butyl protected cysteine derivative of octapeptides **38a**.*

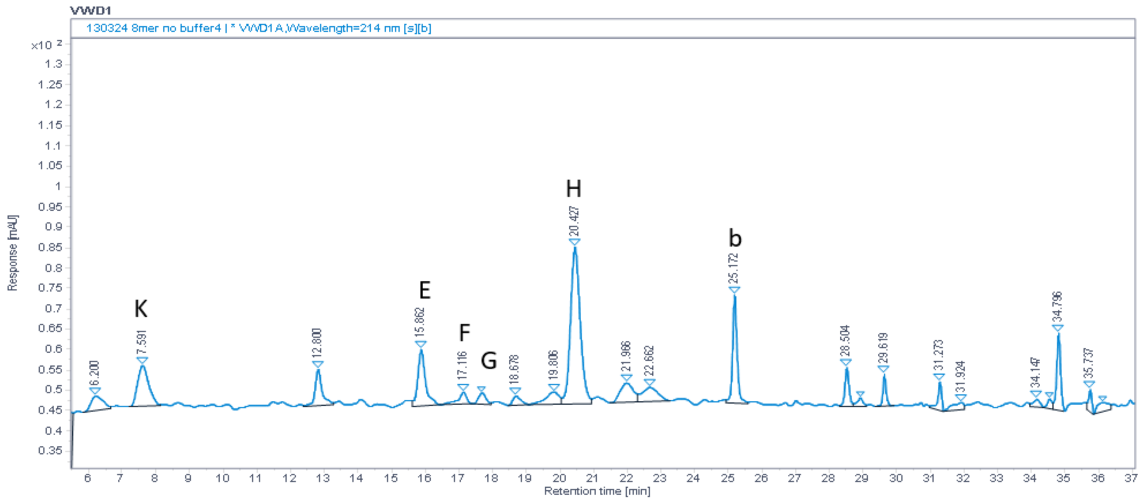

*Figure K93: Analytical HPLC spectrum of photolysis reaction mixture of phenacylsulfide **38** in H_2O (0.8 mL, 6 mM, pH initial 9, pH final 7.8), where K=ketone, E=isothiazolone, F=oxazole, G=thiazole (formed through cyclocondensation of enethiol in acidic HPLC eluent), H=disulfide, b= tert butyl protected cysteine derivative of octapeptide **38a**.*

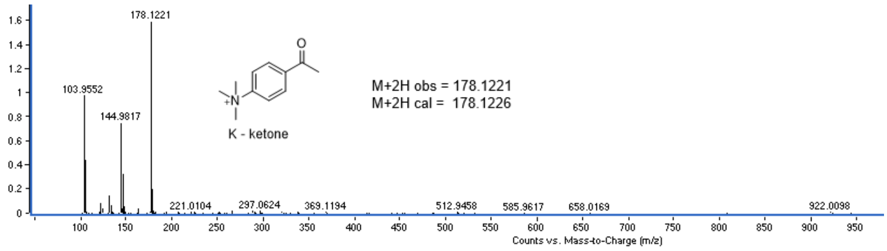
178.1221
103.9552
144.9817
221.0104
297.0624
369.1194
512.9458
585.9617
658.0169
922.0098
K - ketone
M+2H obs = 178.1221
M+2H cal = 178.1226
Counts vs. Mass-to-Charge (m/z)

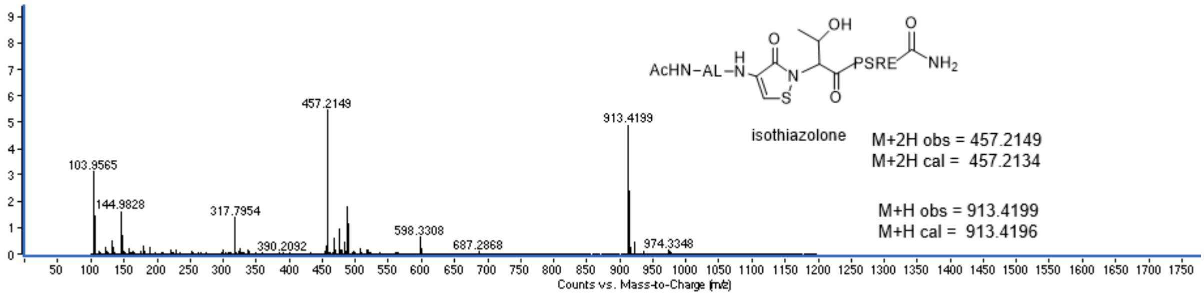
AcHN–AL–N
isothiazolone
PSRE
NH2
457.2149
913.4199
103.9565
144.9828
317.7954
390.2092
598.3308
687.2868
974.3348
M+2H obs = 457.2149
M+2H cal = 457.2134
M+H obs = 913.4199
M+H cal = 913.4196
Counts vs. Mass-to-Charge (m/z)

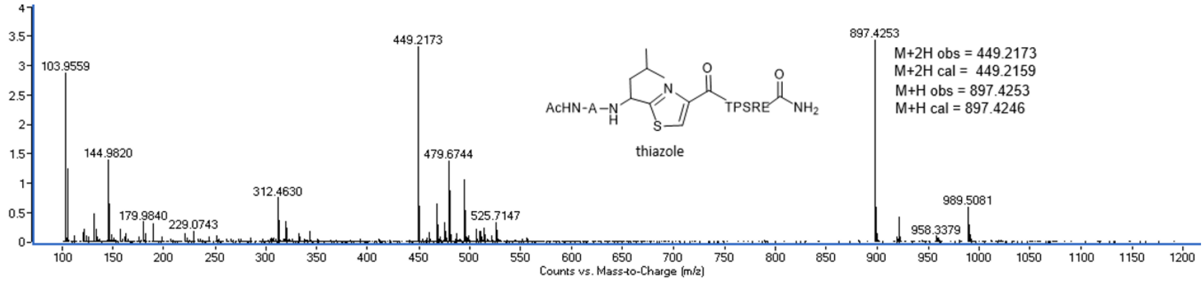
449.2173
897.4253
103.9559
144.9820
179.9840
229.0743
312.4630
479.6744
525.7147
958.3379
989.5081
AcHN–A–N
TPSRE
NH2
thiazole
M+2H obs = 449.2173
M+2H cal = 449.2159
M+H obs = 897.4253
M+H cal = 897.4246
Counts vs. Mass-to-Charge (m/z)

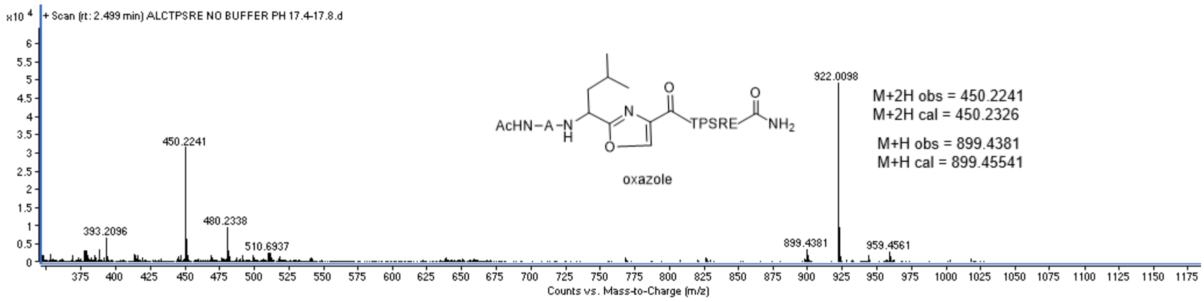
x10 4
+ Scan (rt: 2.499 min) ALCTPSRE NO BUFFER PH 17.4-17.8.d
922.0098
450.2241
393.2096
480.2338
510.6937
899.4381
959.4561
AcHN–A–N
TPSRE
NH2
oxazole
M+2H obs = 450.2241
M+2H cal = 450.2326
M+H obs = 899.4381
M+H cal = 899.45541
Counts vs. Mass-to-Charge (m/z)

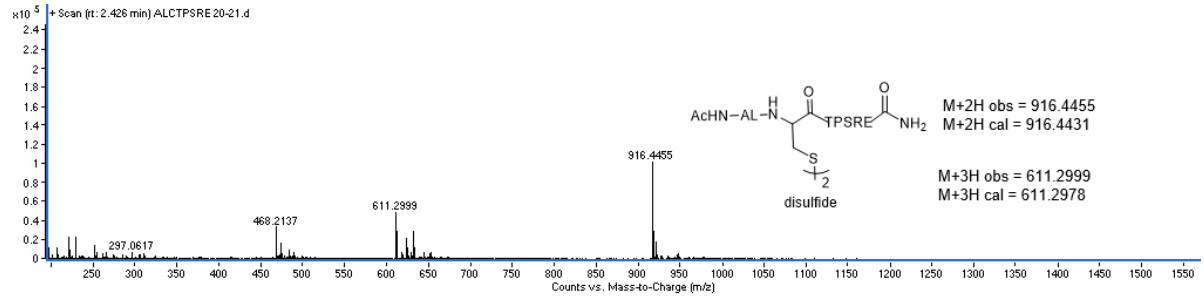
x10 5
+ Scan (rt: 2.426 min) ALCTPSRE 20-21.d
916.4455
611.2999
468.2137
297.0617
AcHN–AL–N
TPSRE
NH2
disulfide
M+2H obs = 916.4455
M+2H cal = 916.4431
M+3H obs = 611.2999
M+3H cal = 611.2978
Counts vs. Mass-to-Charge (m/z)

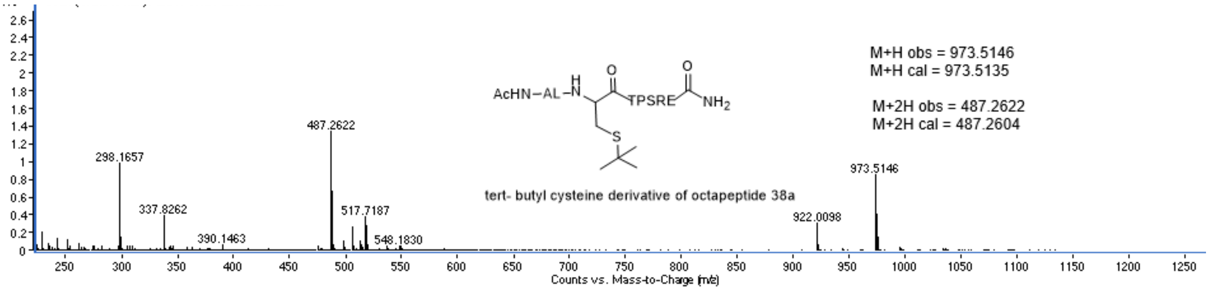

*Figure K94: HRMS (ESI positive mode) spectra of photolysis reaction mixture of phenacylsulfide **38** in H_2O (pH initial 9, pH final 7.8). Observed and calculated m/z peaks are provided.*

*Table K45: Approximate conversion values (%) of photolysis products (phenacylsulfide **38**, H_2O, pH initial 9, pH final 7.8) based on integration of HPLC peaks (detection at 214 nm). Approximate conversions from phenacylsulfide (Norrish type-II and β-scission processes, column 2) and from putative thioaldehyde (Norrish type-II processes only, column 3).*

**product**	**% Area (Conversion from phenacylsulfide)**	**% Area (Conversion from putative thioaldehyde)**
isothiazolone	37%	71%
thiazole	9%	18%
oxazole	6%	11%
disulfide	48%	

Effect of TFE on photolysis reaction

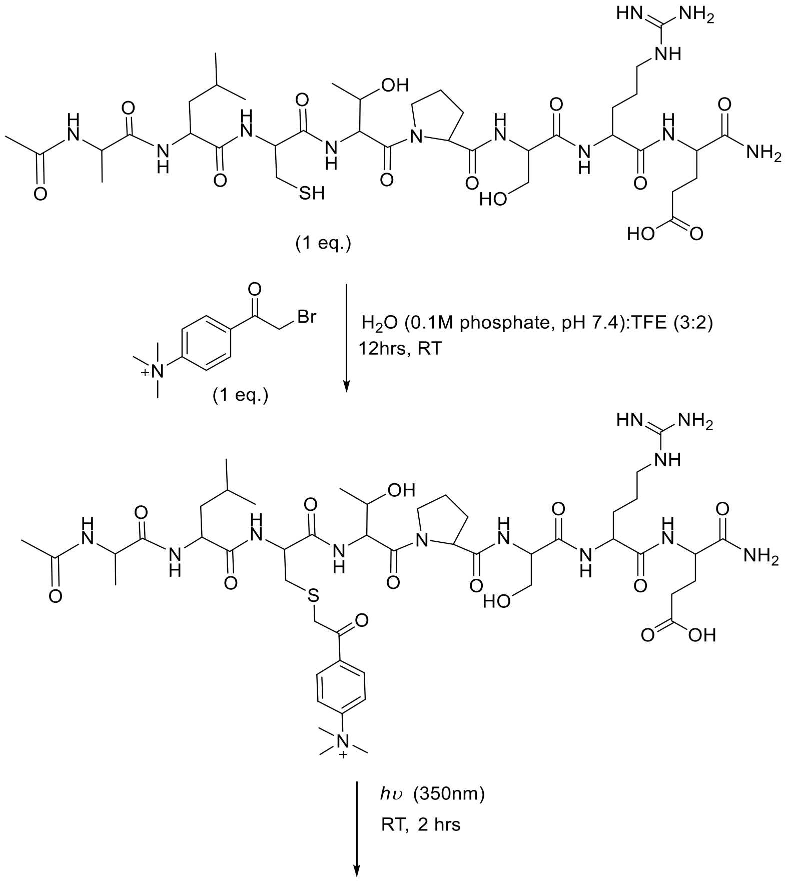

Octapeptide ***38a*** (5 mg, 0.005 mmol) was dissolved in 3:2 H_2O (0.1M phosphate, pH 7.4, 0.4 mL): tfe (0.4 mL) followed by addition of a solution of *p*-trimethylammonium phenacyl bromide (2 mg, 0.005 mmol) in H_2O (0.1 M phosphate, pH 7.4, 0.2 mL).The reaction mixture was kept overnight and analysed through HPLC-MS. On completion of alkylation, the reaction mixture was degassed by passing N_2 gas for 20 mins. The solution was then illuminated with a light of wavelength 350 nm in a Rayonet photo reactor for 2 hrs at room temperature. Products formed in the reaction mixture was analysed by HPLC-MS.

*Table K46: Analytical HPLC method used for characterisation of phenacylsulfide **38** and of photolysis products (phenacylsulfide **38**, 3:2 H_2O (0.1M phosphate, pH 7.4): TFE)*

time (min)	A (%)	B (%)
0	95	5
15	80	20
20	80	20
35	5	95
40	95	5

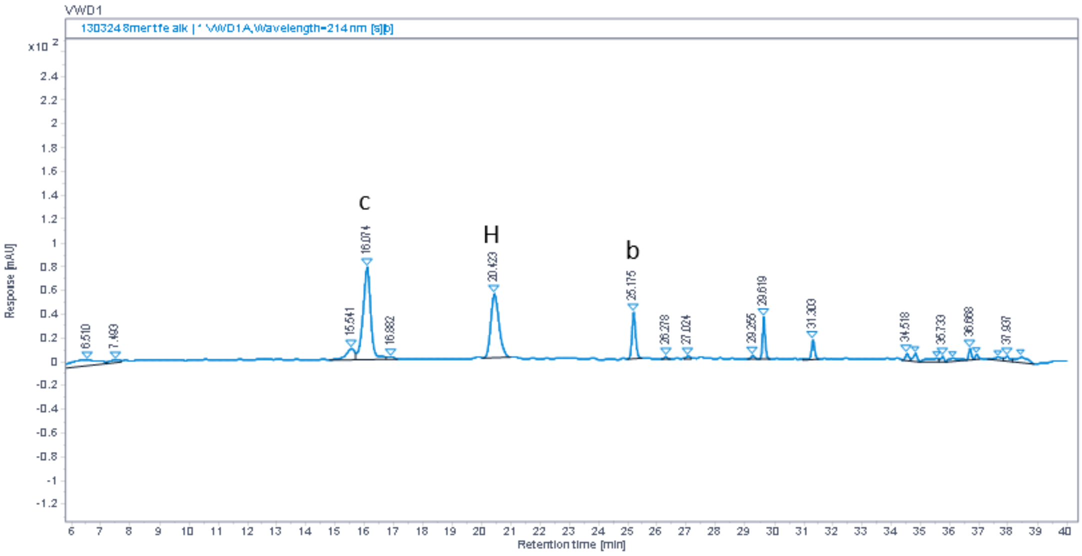

*Figure K95: Analytical HPLC spectrum of peptide alkylation of octapeptide **38a** in 3:2 H_2O (0.1M phosphate, pH 7.4): tfe mixture where c = phenacylsulfide **38**, H = disulfide, b = tert butyl protected cysteine derivative of octapeptide **38a**.*

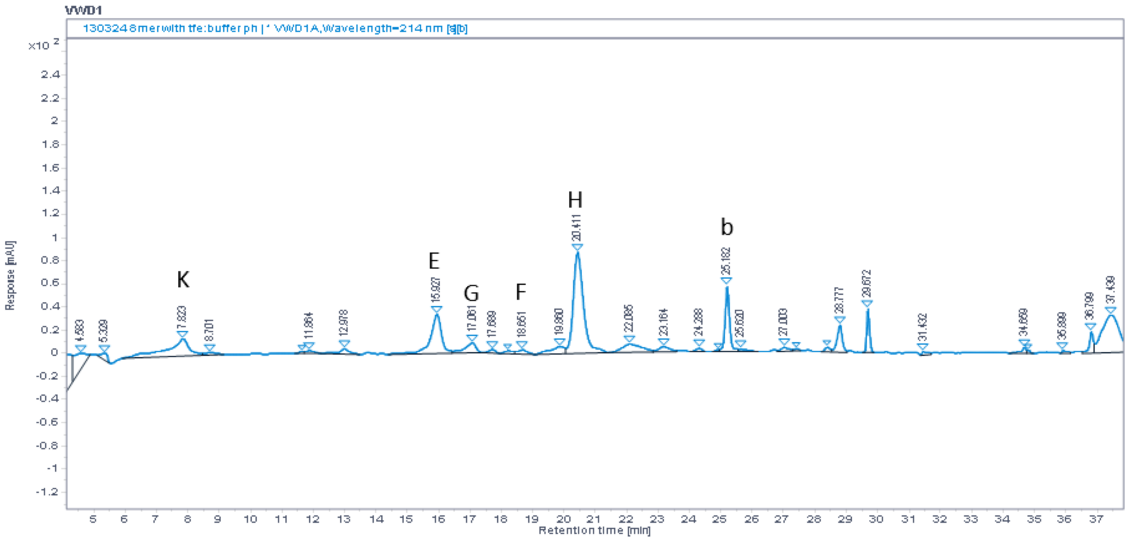

*Figure K96: Analytical HPLC spectrum of photolysis reaction of phenacylsulfide **38** in 3:2 H_2O (0.1M phosphate, pH 7.4): tfe mixture where K= ketone, E=isothiazolone, G =thiazole (formed through cyclocondensation of enethiol in acidic HPLC eluent), F=oxazole, H=disulfide, b= tert butyl protected cysteine derivative of octapeptide **38a.***

*Table K47: Approximate conversion values (%) of photolysis products (phenacylsulfide **38**, 3:2 H_2O (0.1M phosphate, pH 7.4): tfe) based on integration of HPLC peaks (detection at 214 nm). Approximate conversions from phenacylsulfide (Norrish type-II and β-scission processes, column 2) and from putative thioaldehyde (Norrish type-II processes only, column 3).*

**product**	**% Area (Conversion from phenacylsulfide)**	**% Area (Conversion from putative thioaldehyde)**
isothiazolone	62%	75%
thiazole	16%	19%
oxazole	5%	6%
disulfide	17%	

Secondary structure of peptides

To determine the effect of secondary structure on the reaction, CD spectroscopy was performed on peptide thiol ***31a*** and peptide thiol ***38a***.

Far ultraviolet (UV) CD spectra of peptides (50 µL, 64 µM) was recorded on Chirascan VX Circular Dichroism spectrometer. Samples were prepared in (a) H_2O (0.1 M phosphate, pH 7.4 and (b) H_2O (0.1 M phosphate, pH 7.4: TFE (1:1). Temperature was maintained at 20 ºC. Spectra were recorded in the 190–250 nm range by using a 0.1 mm path length cuvette. The distribution of secondary structures were obtained by uploading the data for processing on the Bestsel webserver: https://bestsel.elte.hu/index.php.

A

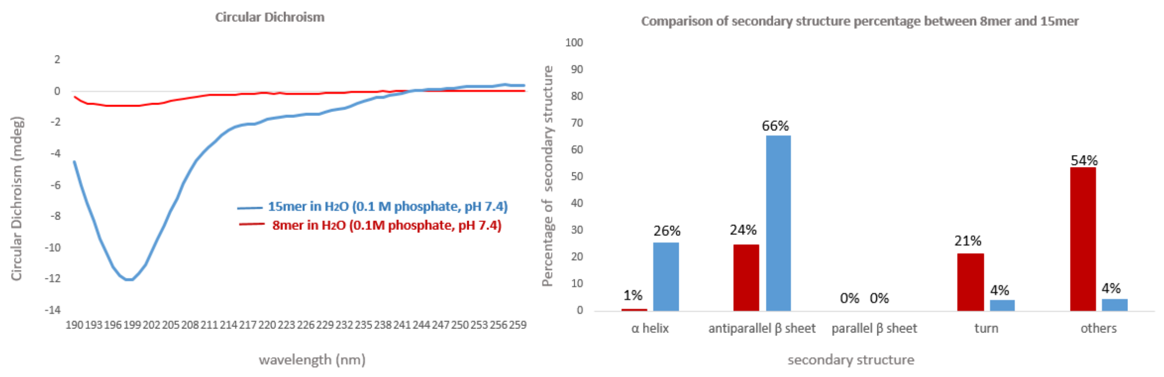

B

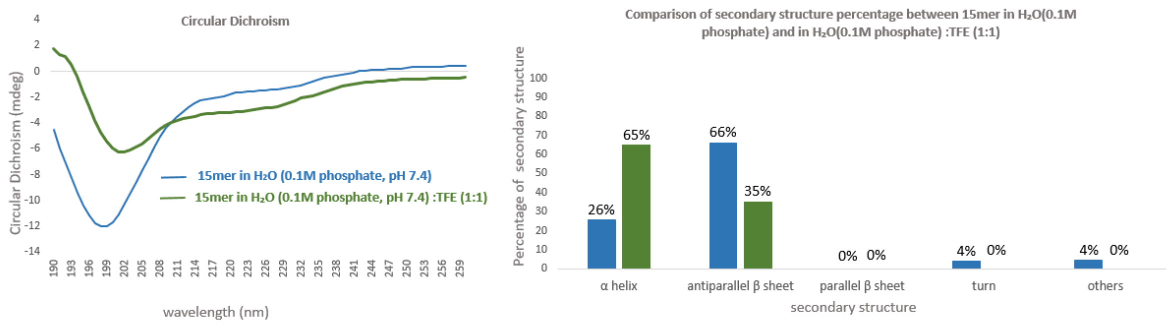

*Figure K97: CD spectra of pentadecapeptide thiol **31a** and octapeptide thiol **38a**. **A**: CD spectra of pentadecapeptide thiol 31a in H_2O (0.1M phosphate, pH 7.4) marked in blue, octapeptide thiol **38a** in H_2O (0.1M phosphate, pH 7.4) marked in red. **B**: CD spectra of pentadecapeptide thiol **31a** in H_2O (0.1M phosphate, pH 7.4) marked in blue, pentadecapeptide thiol **38a** in H_2O (0.1M phosphate, pH 7.4): TFE (1:1) marked in green. On addition of TFE, α-helical character of peptide thiol **31a** increased from 26% to 65% indicating the influence of TFE in inducing a more ordered α-helical character to the secondary structure of peptide.*

L. X-ray diffraction data of simple isothiazolone derivative 6

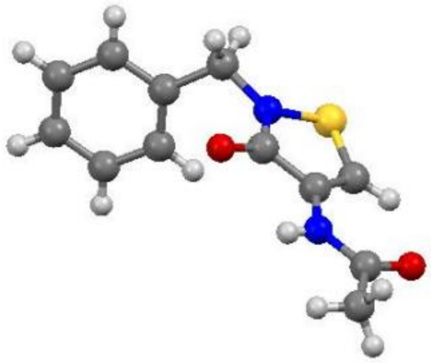

*Figure L1: X-ray crystal structure for simple isothiazolone derivative **6**, crystallized from DCM/petroleum ether.*

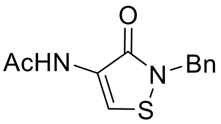	
Empirical formula	$C_{12}H_{12}N_2O_2S$
Formula weight	248.30
Temperature	299.0(1) K
Wavelength	0.71073 Å
Crystal system	Monoclinic
Space group	$P2_1/n$
Unit cell dimensions	a = 10.9773(7) Å<br>b = 5.8082(4)<br>c = 18.7529(15) Å
Volume	1192.80(15) $Å^3$
Z	4
Density (calculated)	1.383 Mg/m^3
Absorption coefficient	0.262 mm^{-1}
F(000)	520
Crystal size	0.50 x 0.40 x 0.20 mm^3
Theta range for data collection	3.647 to 29.150°.
Index ranges	$-13 \leq h \leq 14$, $-7 \leq k \leq 7$, $-24 \leq l \leq 25$
Reflections collected	10229
Independent reflections	2899 [R(int) = 0.0269]
Completeness to theta = 25.242°	99.9 %
Absorption correction	Semi-empirical from equivalents
Max. and min. transmission	1.00000 and 0.70368
Refinement method	Full-matrix least-squares on F^2
Data / restraints / parameters	2899 / 121 / 159
Goodness-of-fit on F^2	1.030
Final R indices [I>2sigma(I)]	R1 = 0.0475, wR2 = 0.1123
R indices (all data)	R1 = 0.0666, wR2 = 0.1243
Extinction coefficient	n/a
Largest diff. peak and hole	0.197 and -0.361 $e.Å^{-3}$
Empirical formula	$C_{12}H_{12}N_2O_2S$

M. NMR spectra

Synthesis of cysteine derivative

Preparation of *N*-acetyl-*S*-(triphenylmethyl)cysteine (compound 1a)

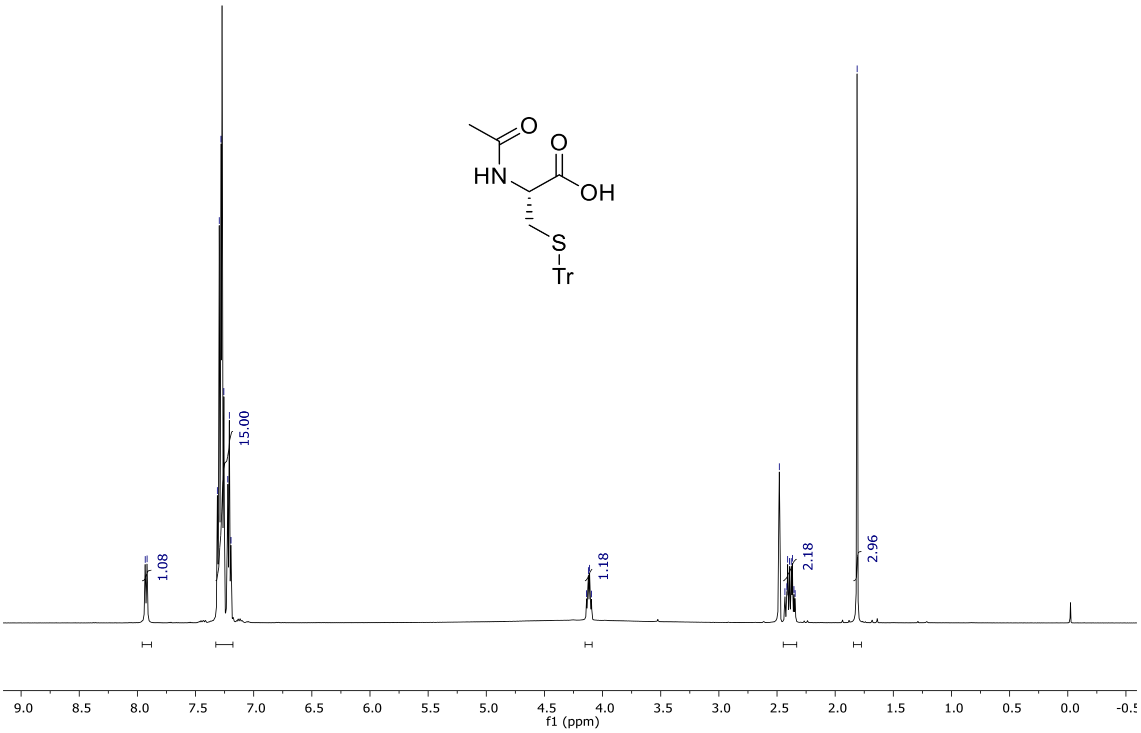

*Figure M1: ^{1}H NMR (500 MHz) spectrum of compound **1a** in DMSO-d_6 following purification*

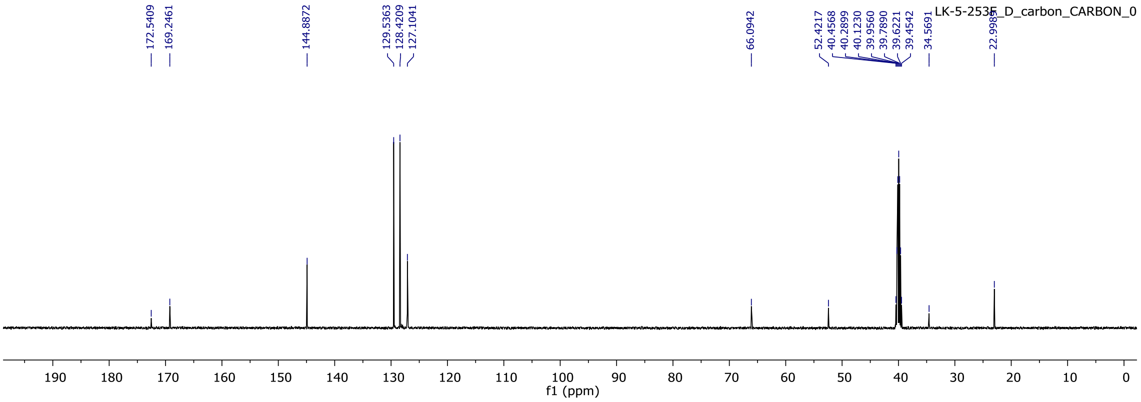

*Figure M2: ^{13}C NMR (125 MHz) spectrum of compound **1a** in DMSO-d_6 following purification*

Preparation of N^2-acetyl-*N*-benzyl-*S*-(triphenylmethyl)cysteinamide (compound 1b)

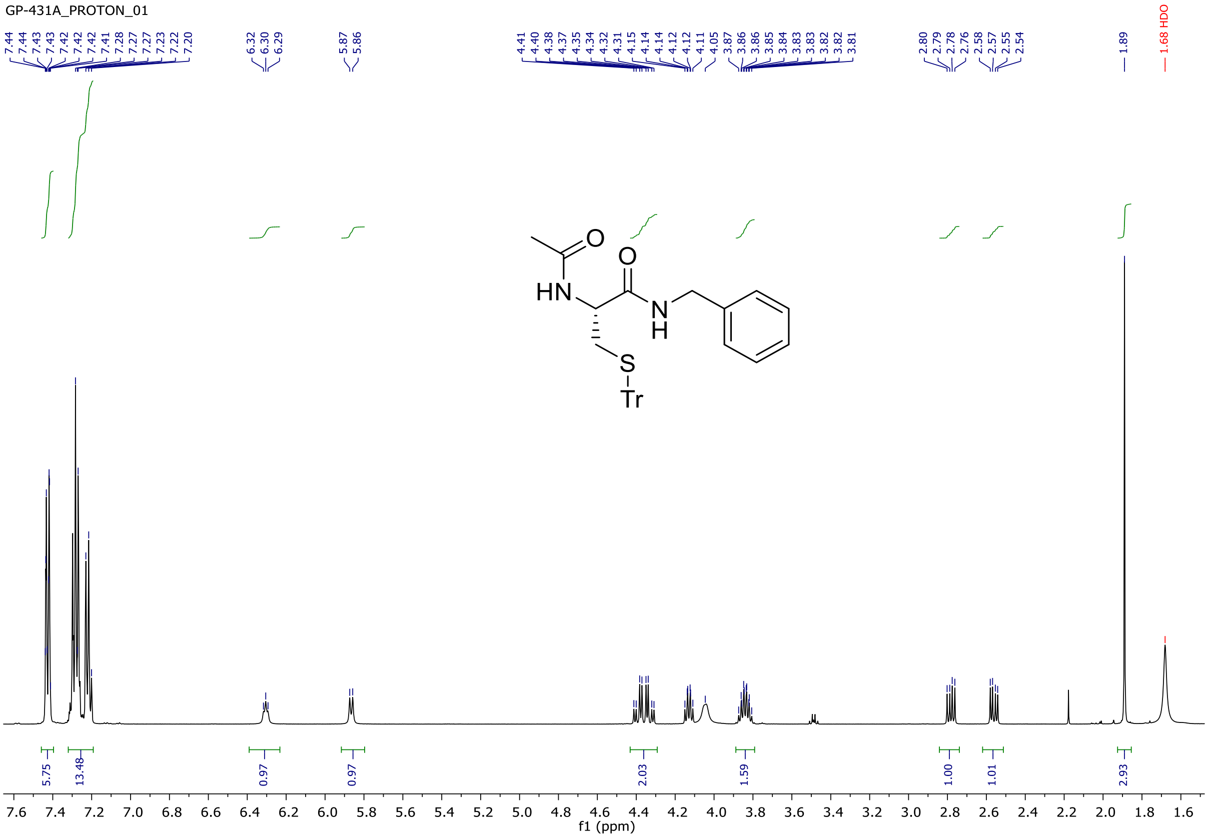

*Figure M3: ^{1}H NMR (500 MHz) spectrum of compound **1b** in $CDCl_3$ following purification*

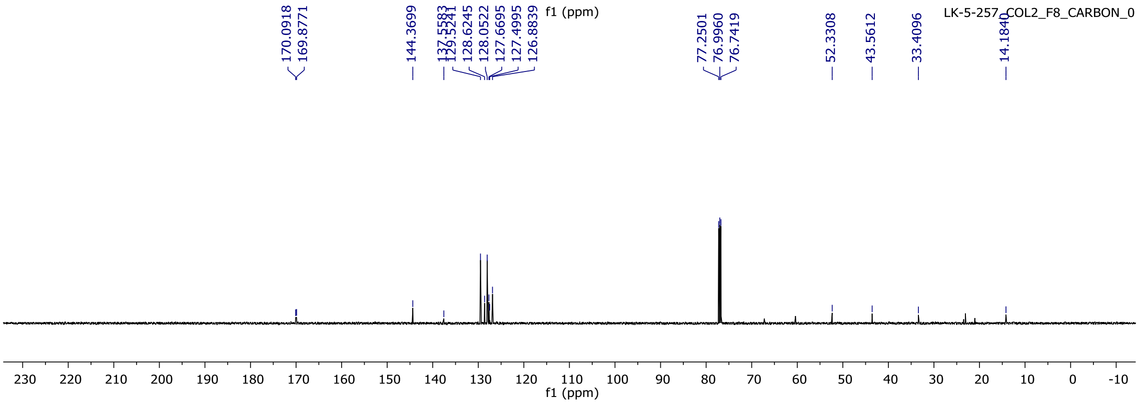

*Figure M4: ^{13}C NMR (125 MHz) spectrum of compound **1b** in $CDCl_3$ following purification*

Preparation of N^2-acetyl-*N*-benzylcysteinamide (compound 1)

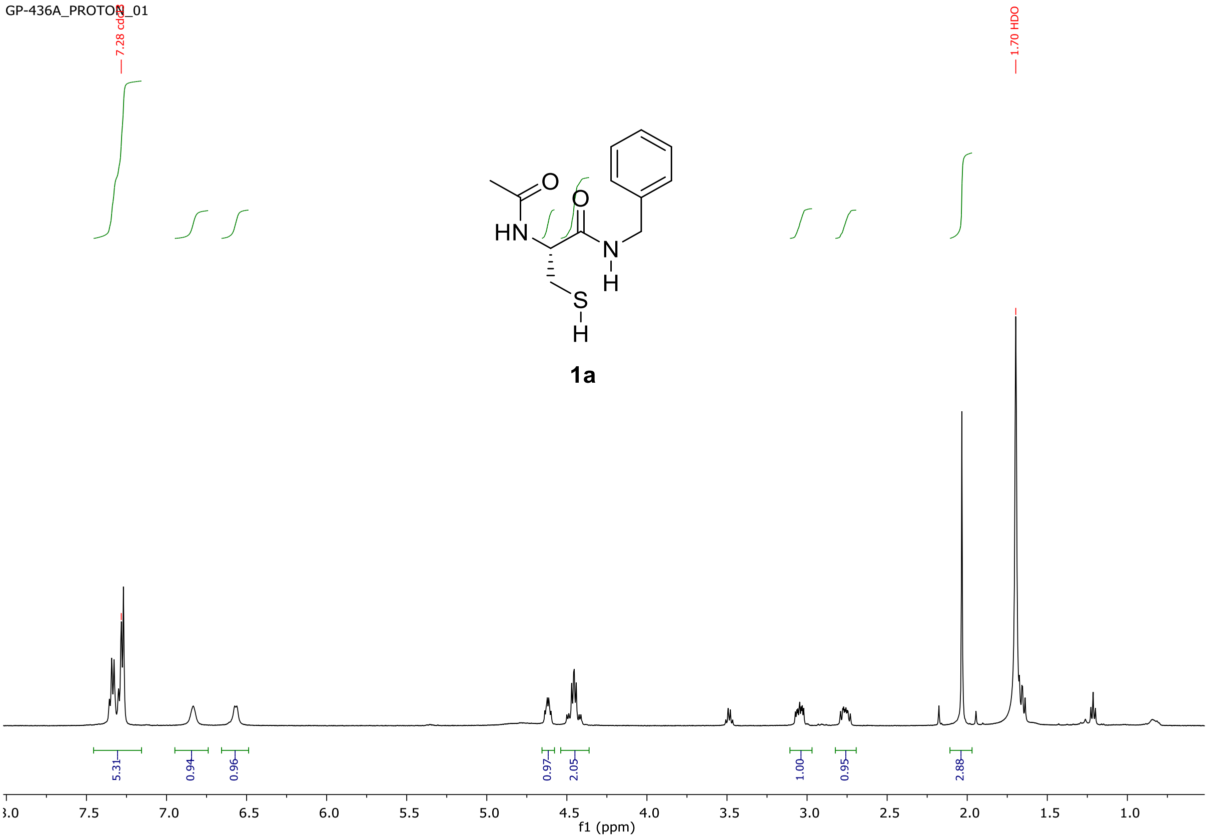

*Figure M5: ^{1}H NMR (500 MHz) spectrum of compound **1** in $CDCl_3$ following purification*

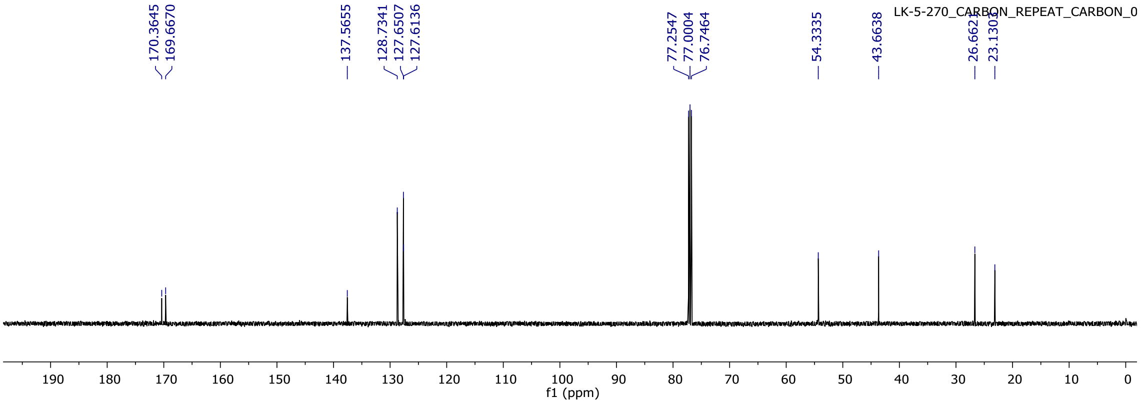

*Figure M6: ^{13}C NMR (125 MHz) spectrum of compound **1** in $CDCl_3$ following purification*

Synthesis of phenacylbromide compounds

Synthesis of **compound 2g**

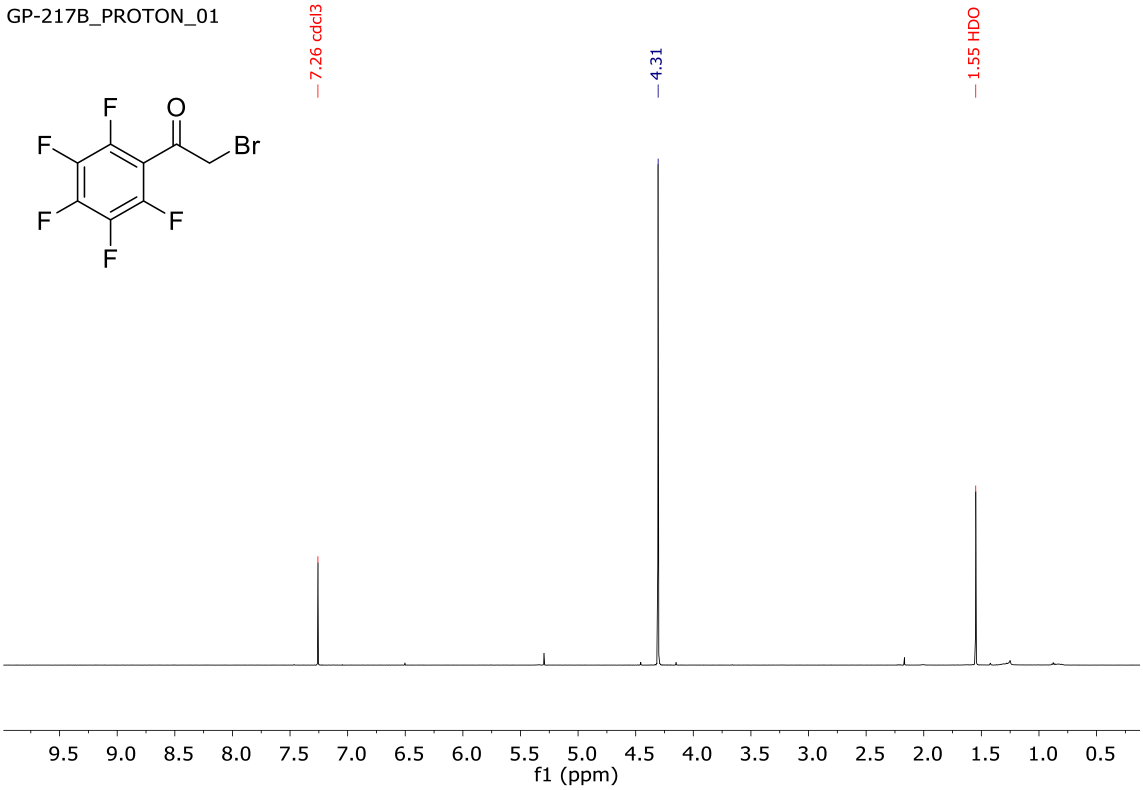

*Figure M7: ^{1}H NMR (500 MHz) spectrum of compound **2g** in $CDCl_3$ following purification*

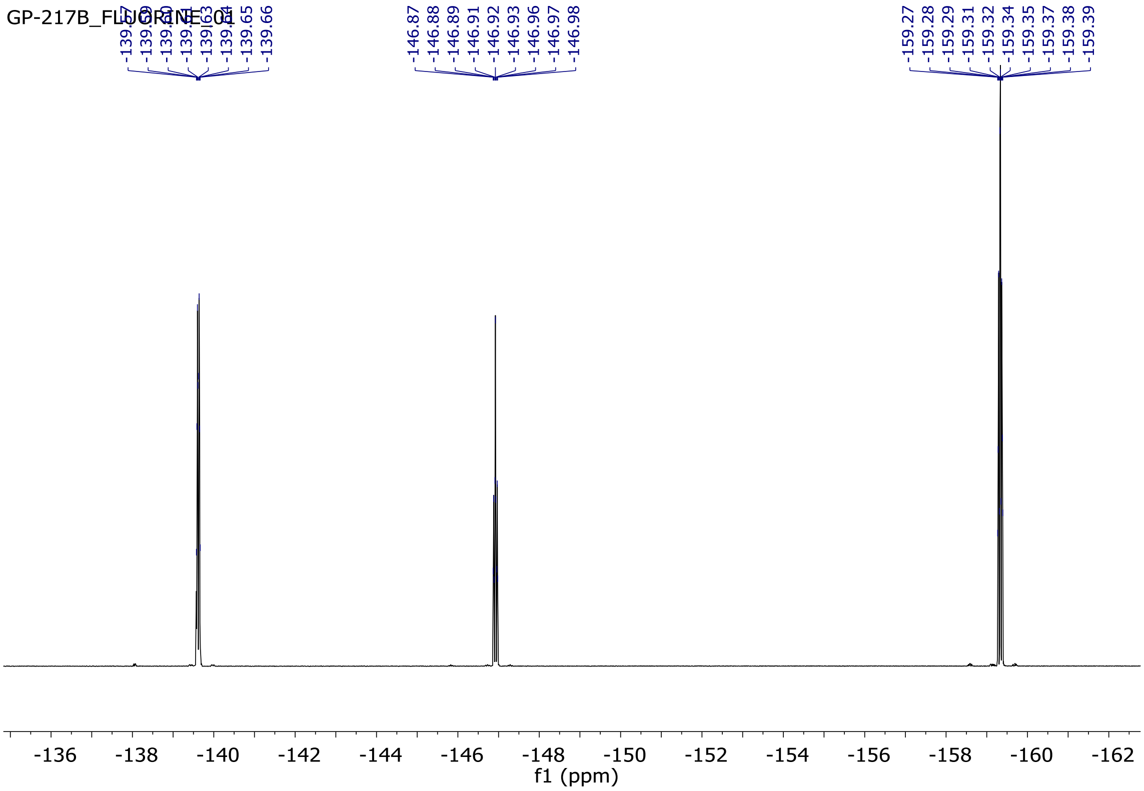

*Figure M8: ^{19}F NMR (470 MHz) spectrum of compound **2g** in $CDCl_6$ following purification*

Synthesis of **compound 2h**

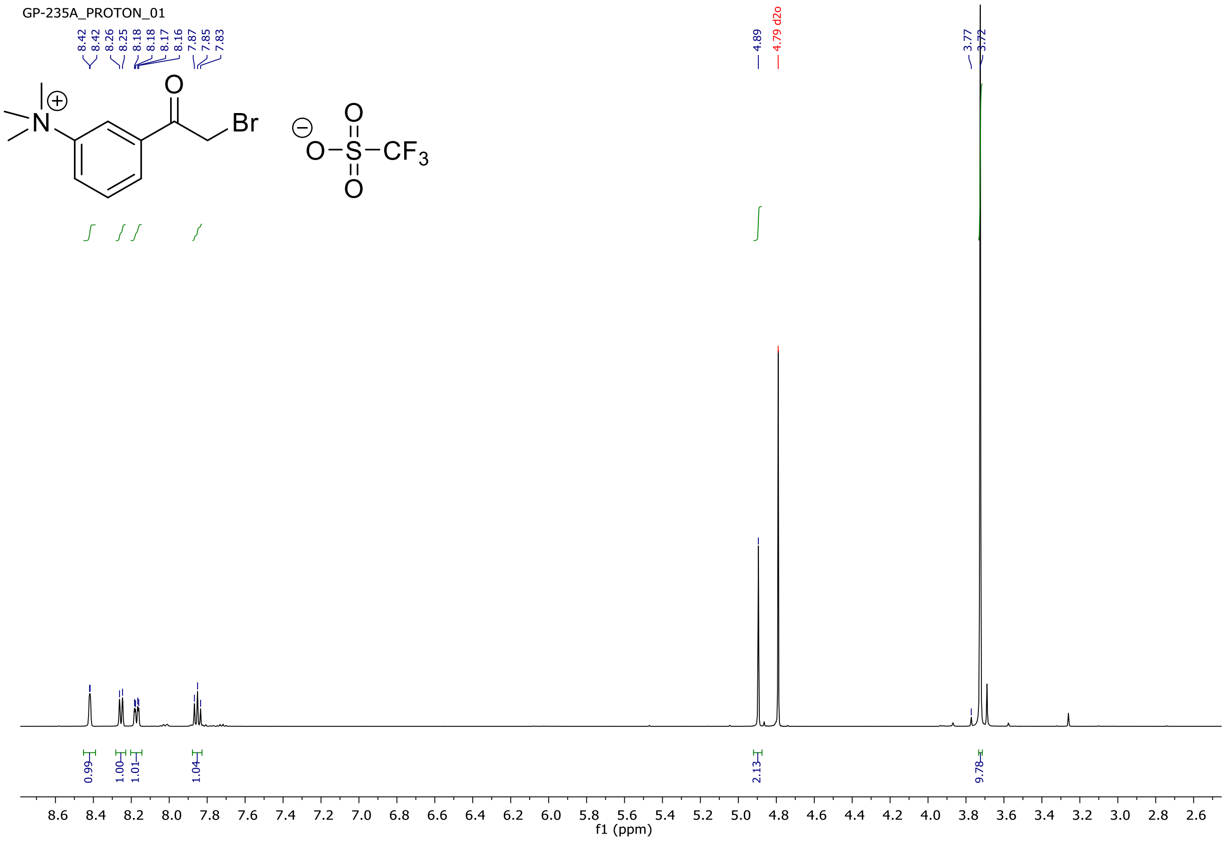

*Figure M9: ^{1}H NMR (500 MHz) spectrum of compound **2h** in D_2O following purification*

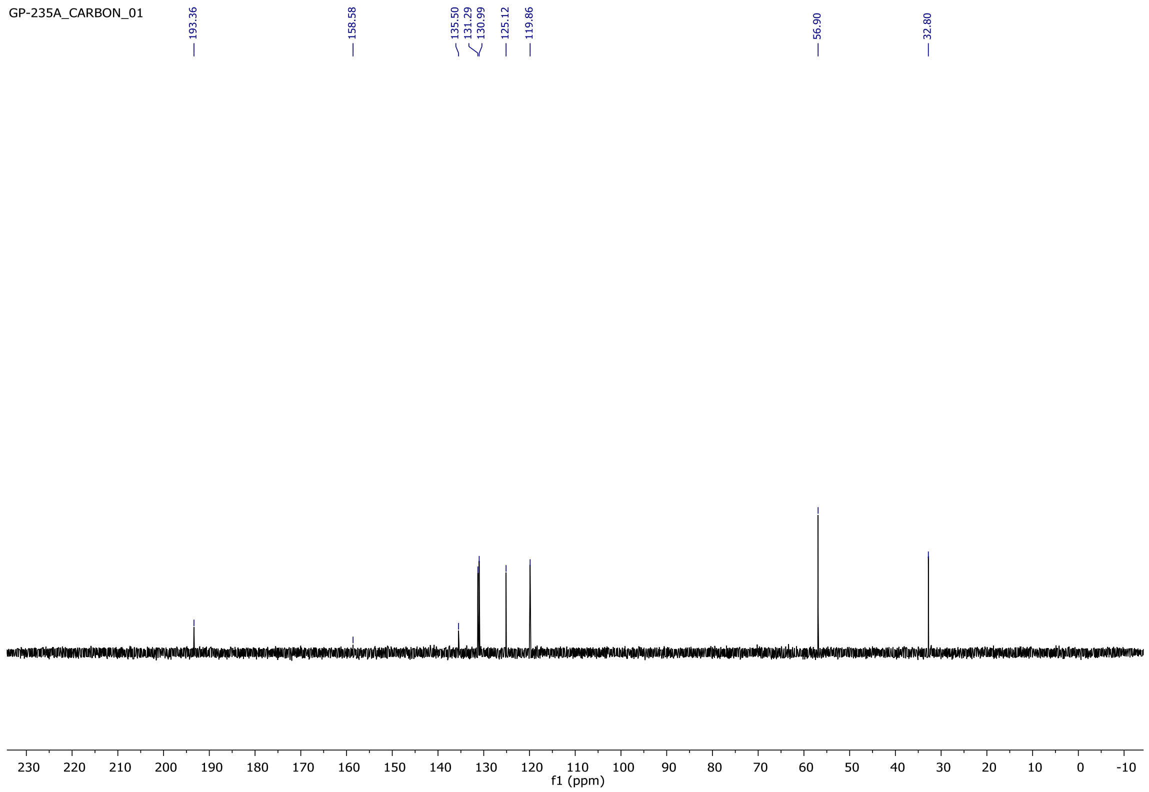

*Figure M10: ^{13}C NMR (126 MHz) spectrum of compound **2h** in D_2O following purification*

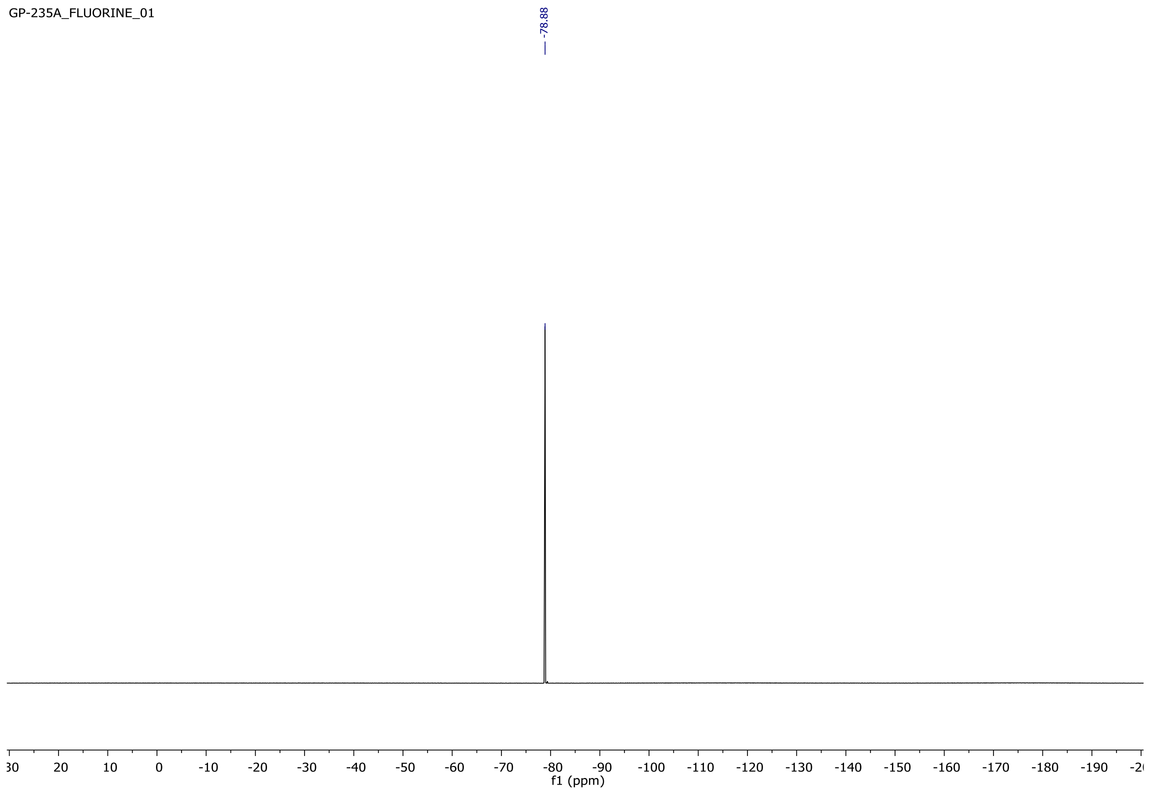

*Figure M11: ^{19}F NMR (470 MHz) spectrum of compound **2h** in D_2O following purification*

Synthesis of **compound 2i**

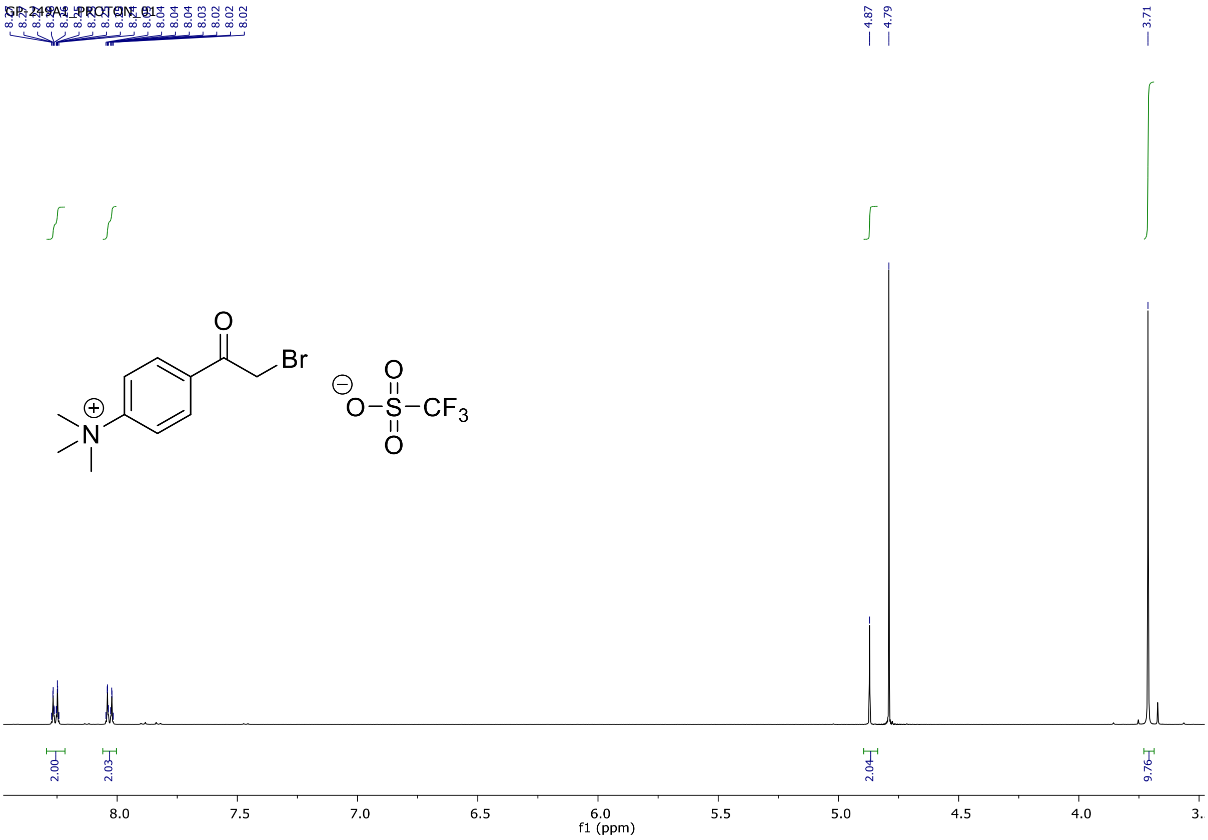

*Figure M12: ^{1}H NMR (500 MHz) spectrum of compound **2i** in D_2O following purification*

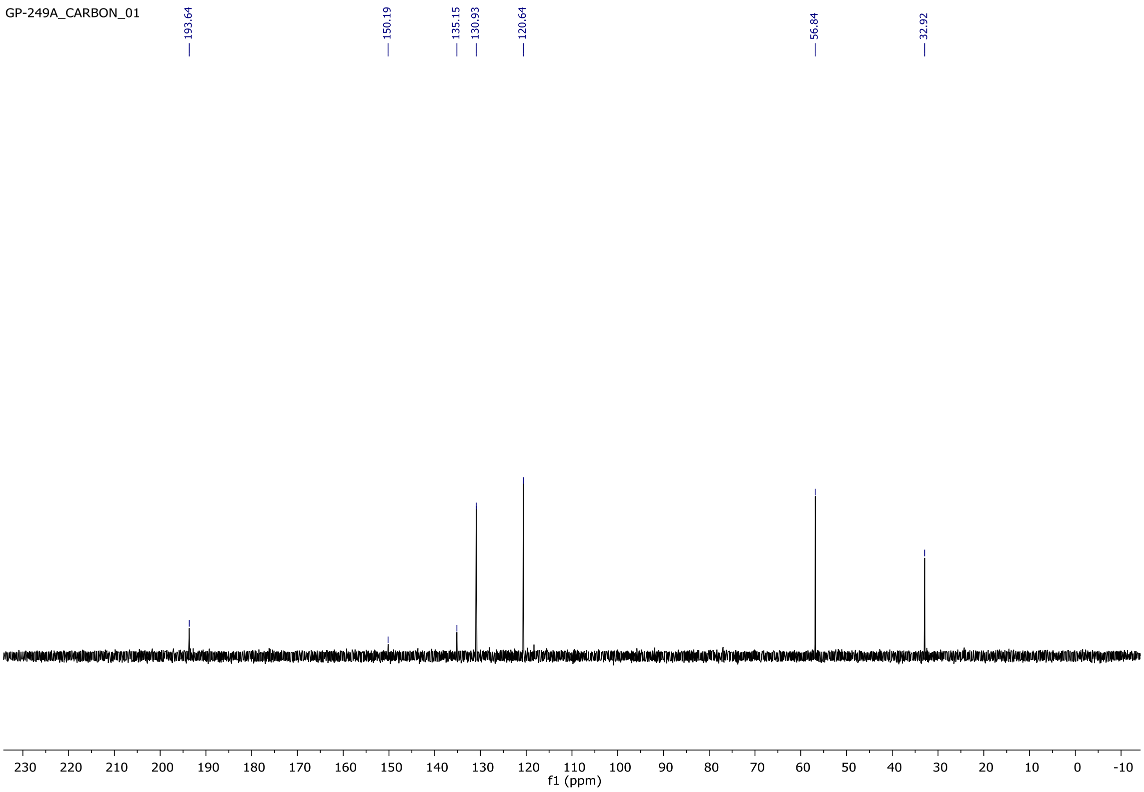

*Figure M13: ^{13}C NMR (126 MHz) spectrum of compound **2i** in D_2O following purification*

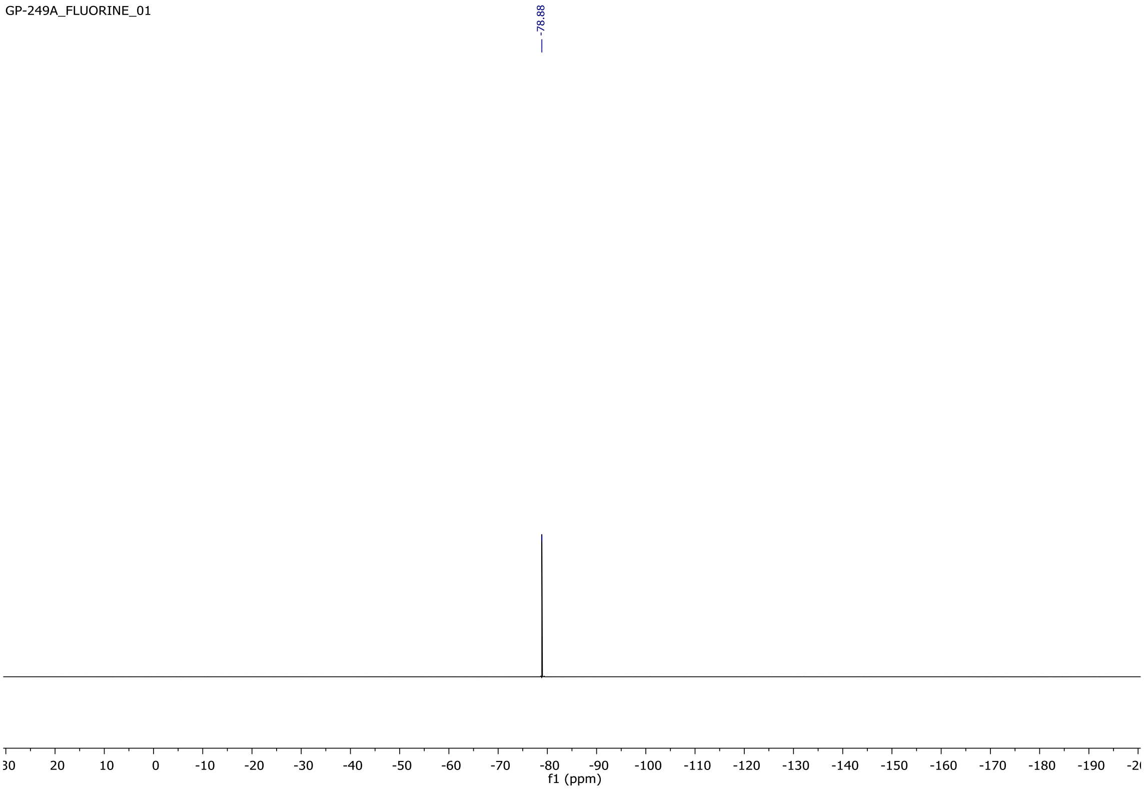

*Figure M14: ^{19}F NMR (470 MHz) spectrum of compound **2i** in D_2O following purification*

Synthesis of **compound 2j**

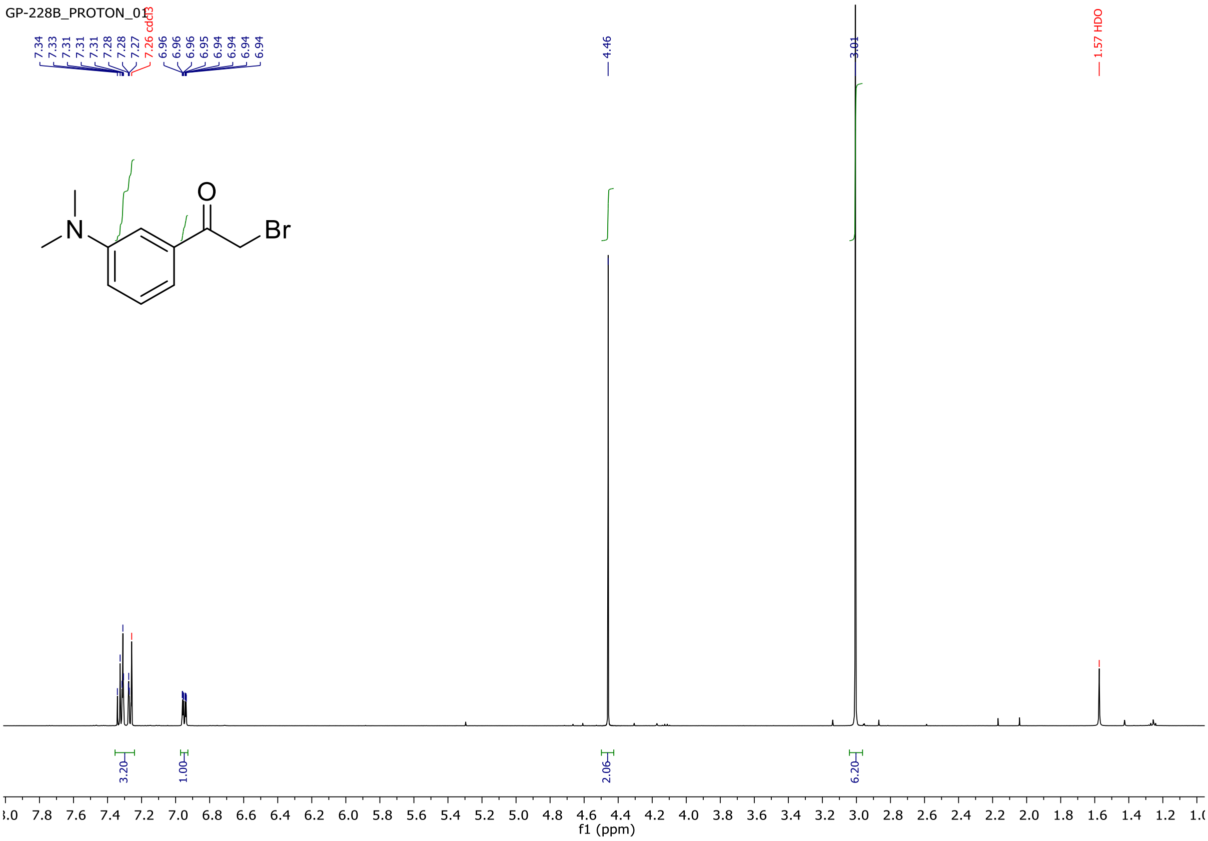

*Figure M15:^{1}H NMR (500 MHz) spectrum of compound **2j** in $CDCl_3$ following purification*

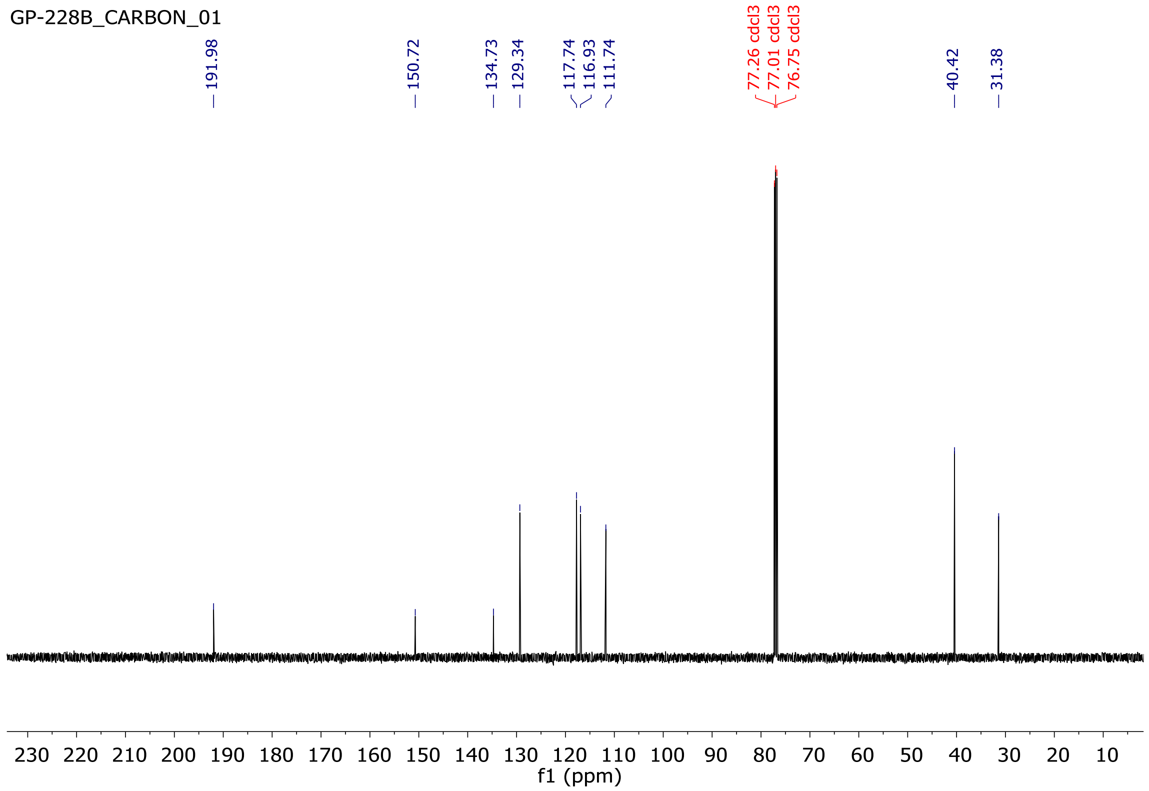

Figure M16:^{13}*C NMR (126 MHz) spectrum of compound* ***2j*** *in* $CDCl_3$ *following purification*

Synthesis of **compound 2k**

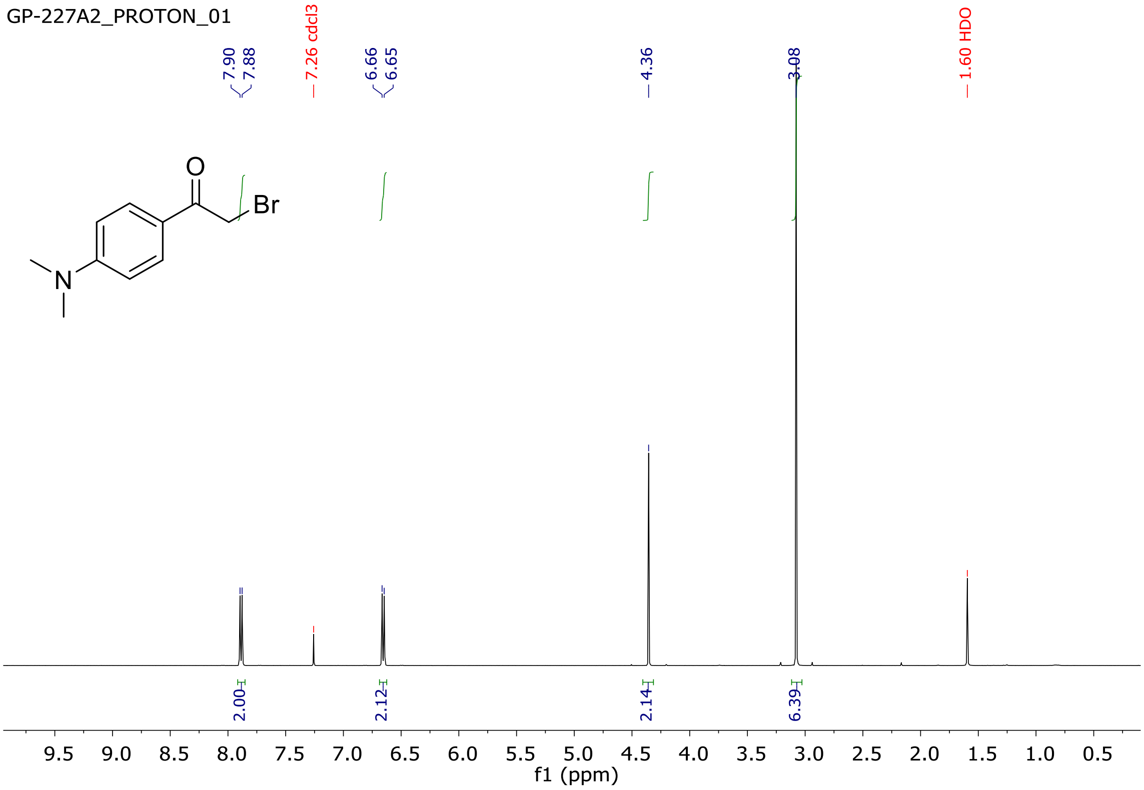

*Figure M17: ^{1}H NMR (500 MHz) spectrum of compound **2k** in $CDCl_3$ following purification*

Synthesis of **compound 2l**

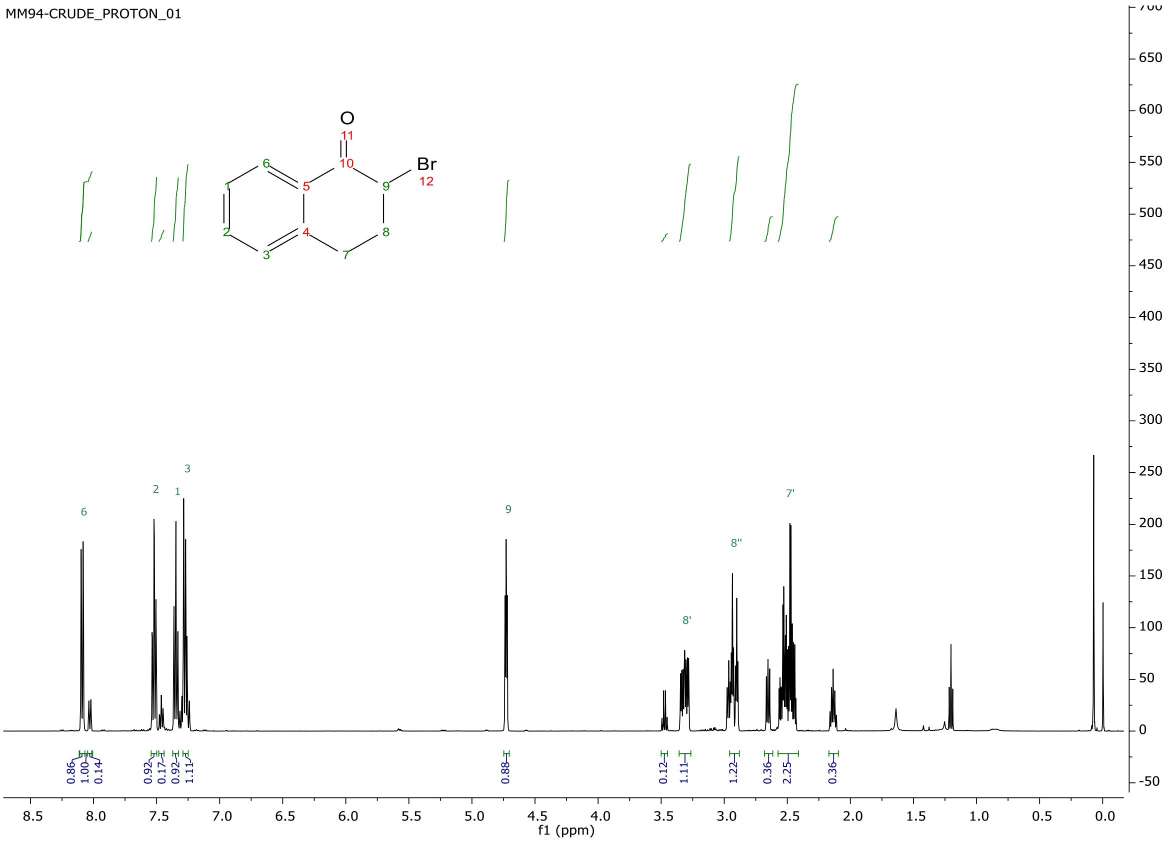

*Figure M18: ^{1}H NMR spectrum ($CDCl_3$) of 2-bromo-3,4-dihydonapthalen-1(*2H*)-one from crude reaction mixture; unassigned signals correspond to unreacted 3,4-dihydronaphthalen-1(*2H*)-one.*

Synthesis of cysteine containing peptides and phenacylsulfide derivatives

Preparation of compound 3a

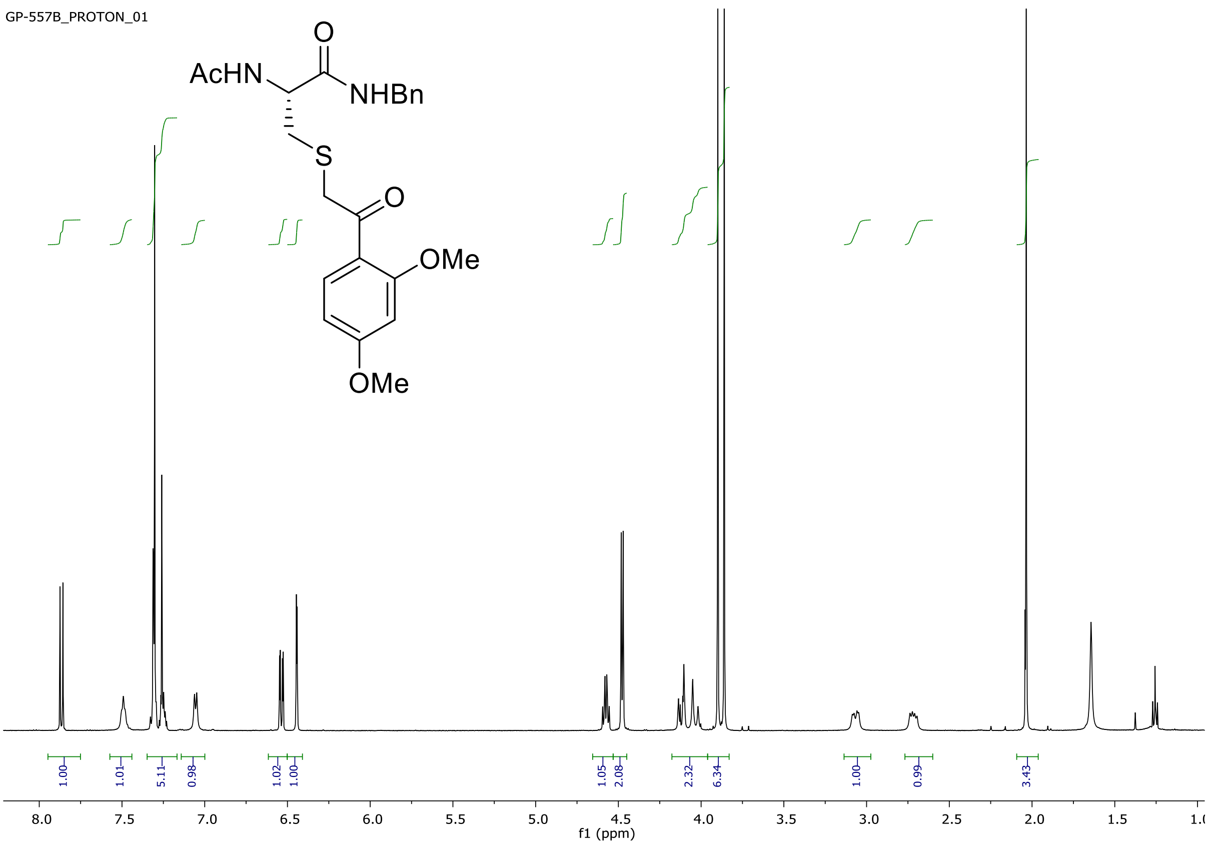

*Figure M19: ^{1}H NMR (500 MHz) spectrum of compound **3a** in $CDCl_3$ following purification*

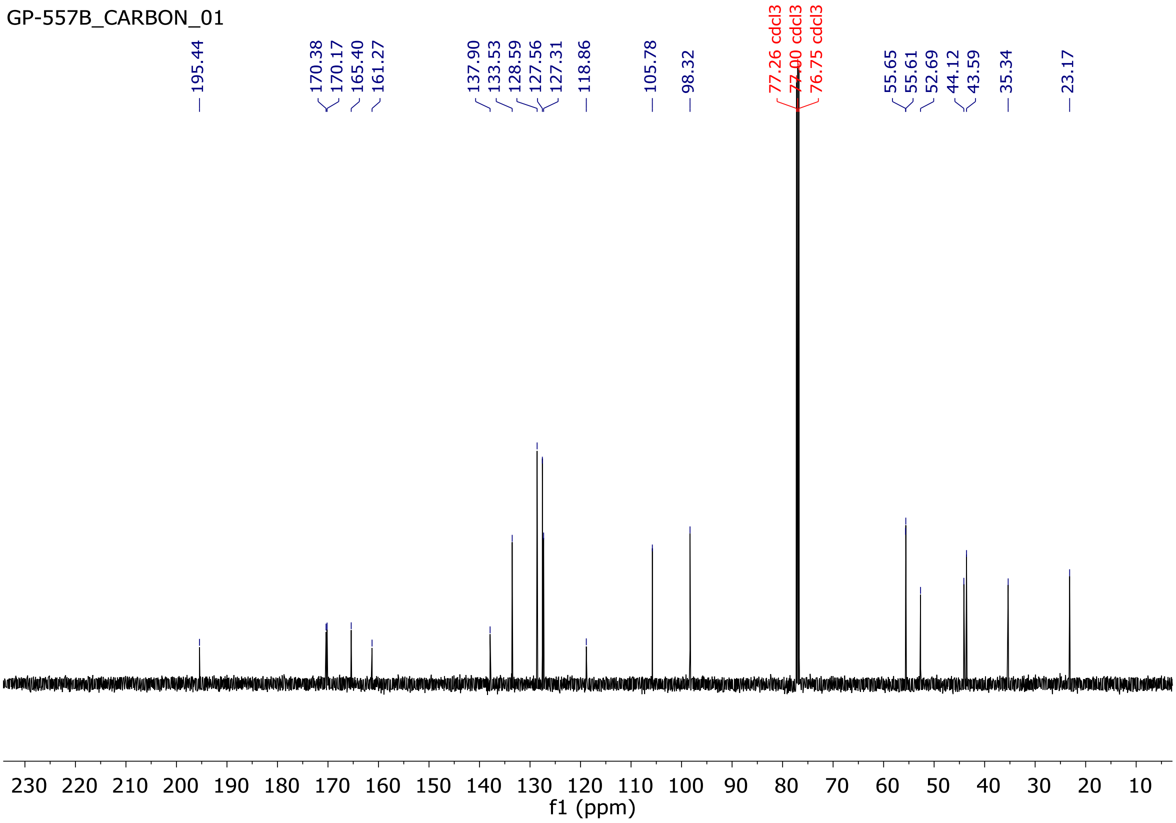

*Figure M20: ^{13}C NMR (125 MHz) spectrum of compound **3a** in $CDCl_3$ following purification*

Preparation of compound 3b

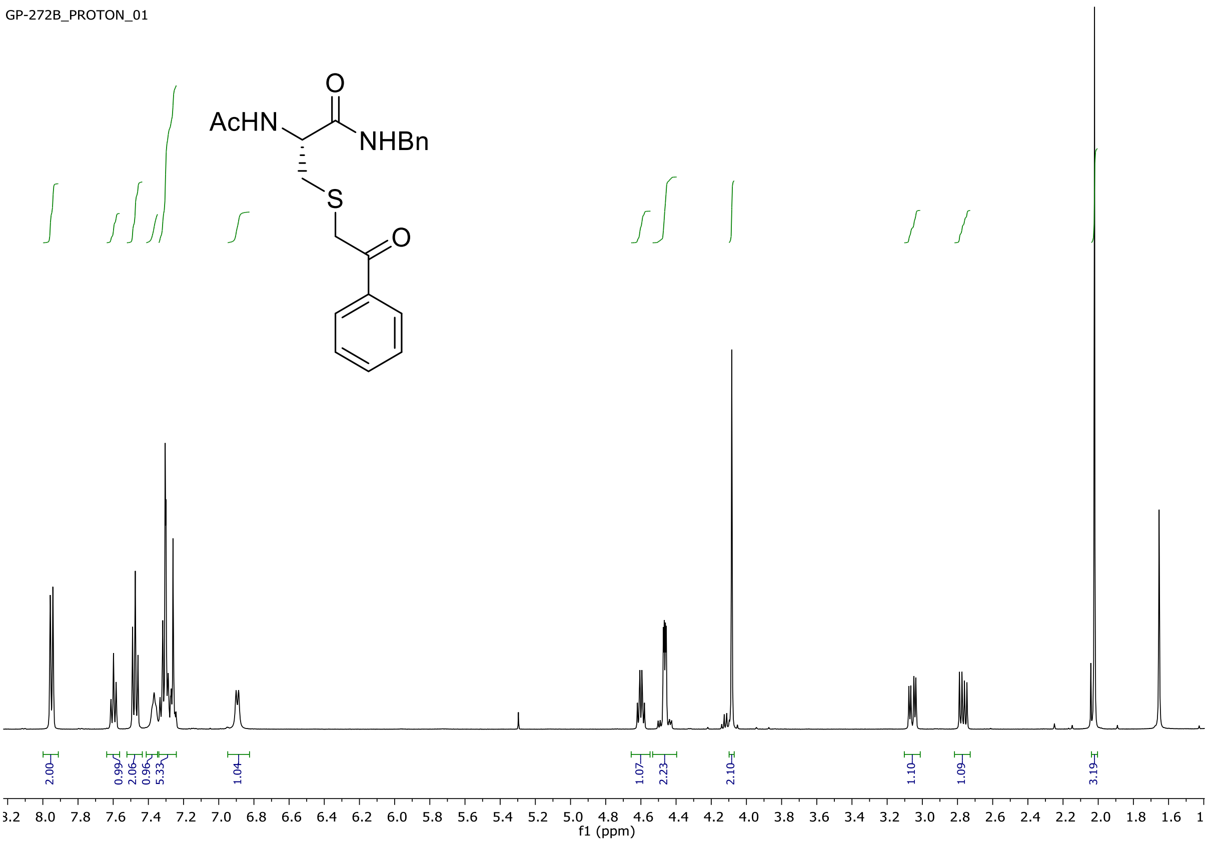

*Figure M21: ^{1}H NMR (500 MHz) spectrum of compound **3b** in $CDCl_3$ following purification*

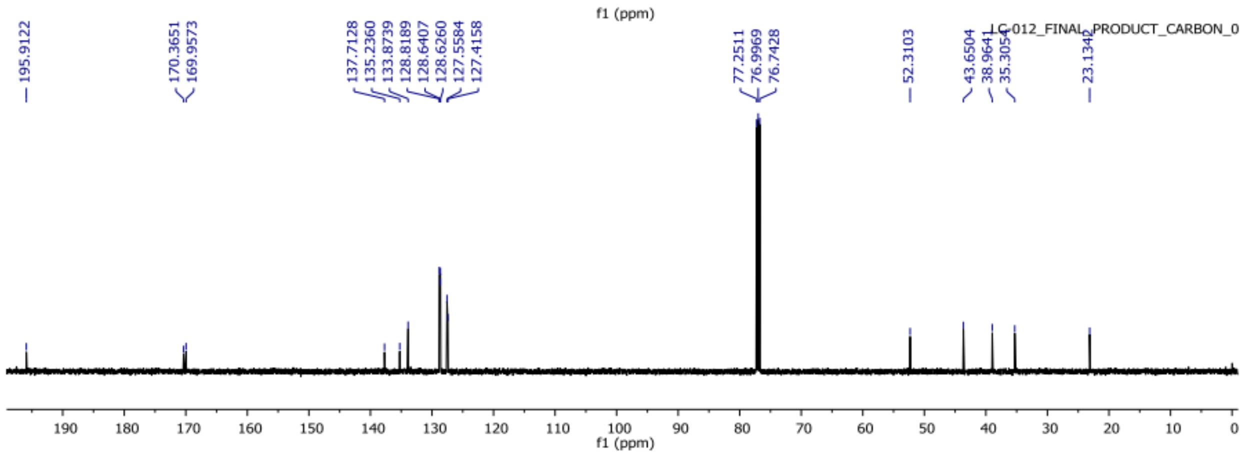

*Figure M22: ^{13}C NMR (125 MHz) spectrum of compound **3b** in $CDCl_3$ following purification*

Preparation of compound 3c

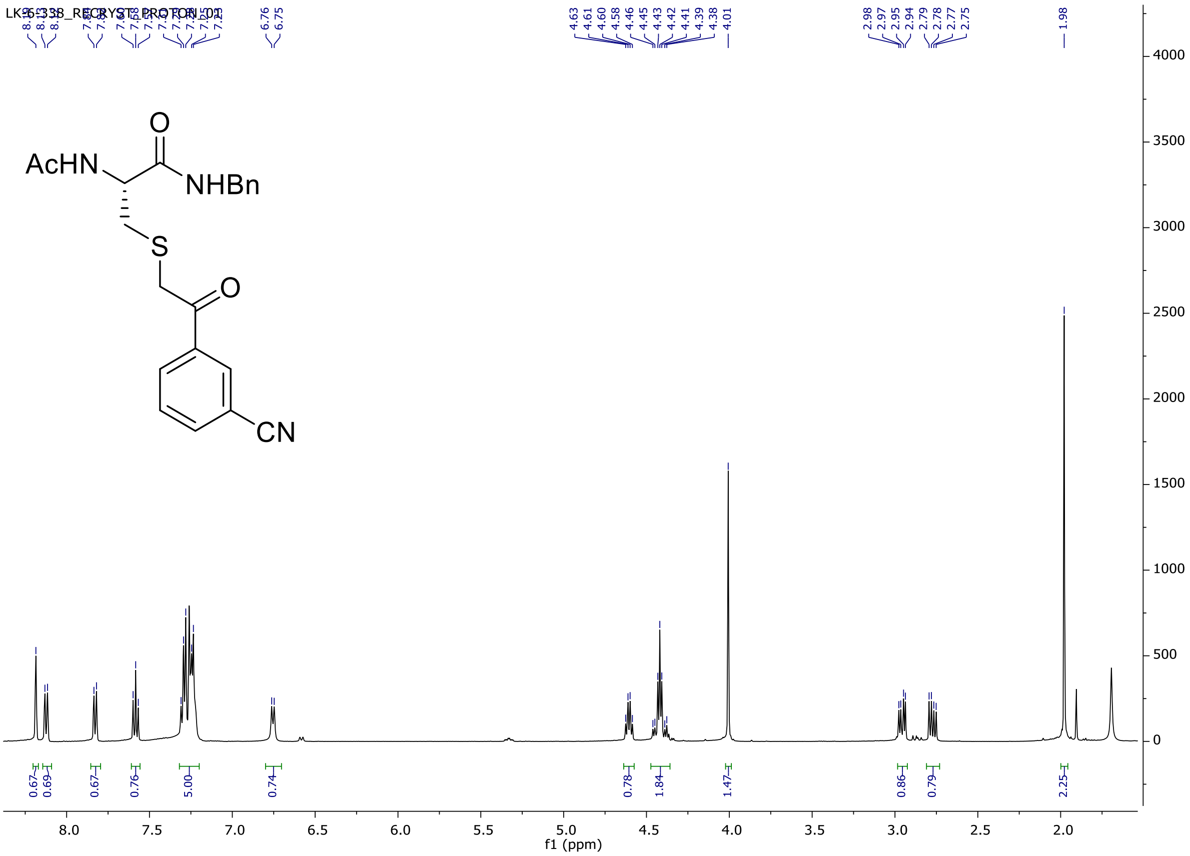

*Figure M23: ^{1}H NMR (500 MHz) spectrum of compound **3c** in $CDCl_3$ following purification*

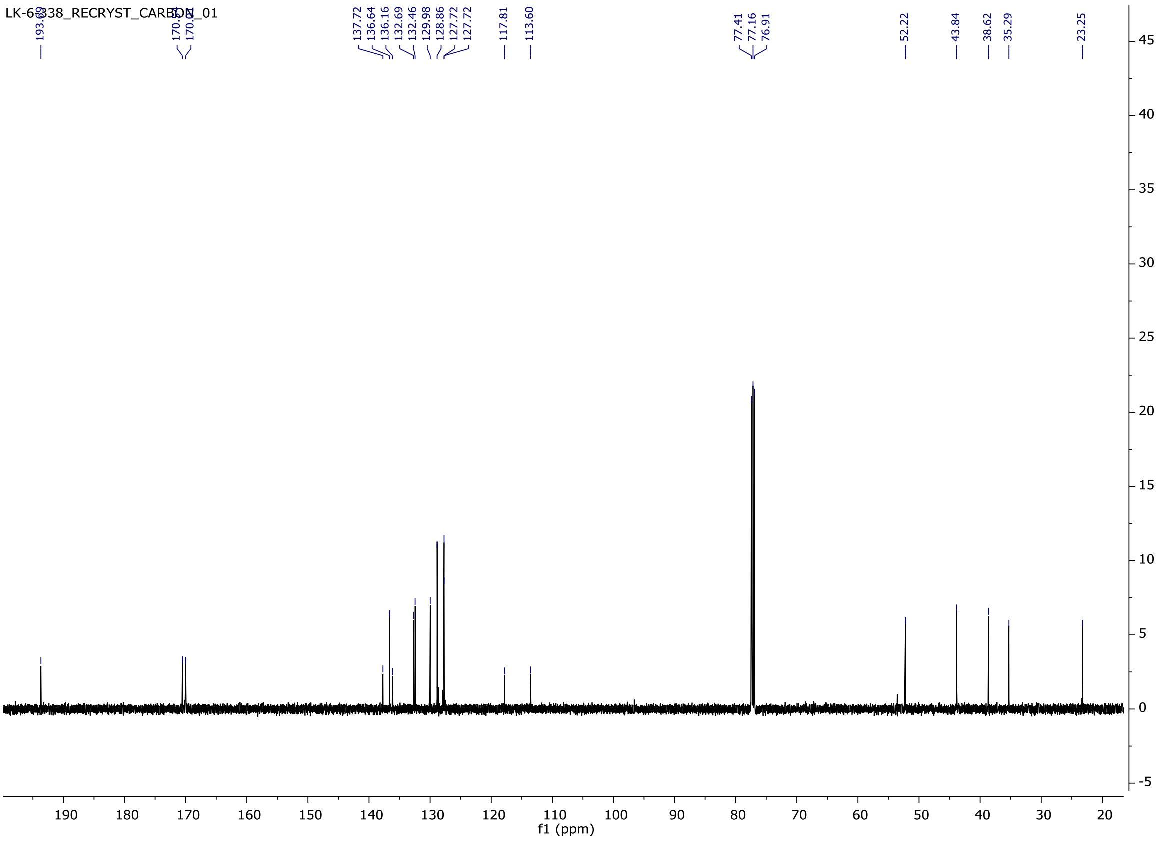

*Figure M24: ^{13}C NMR (125 MHz) spectrum of compound **3c** in $CDCl_3$ following purification*

Preparation of compound 3d

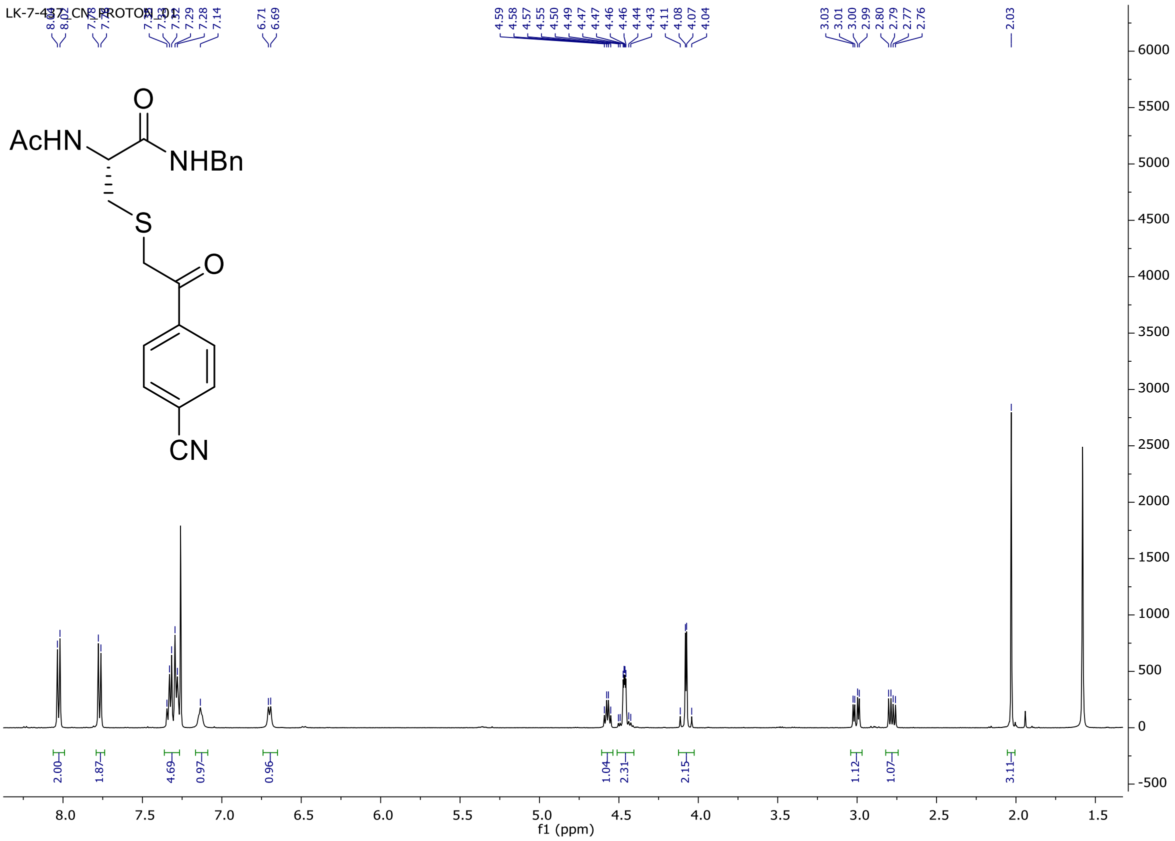

*Figure M25: ^{1}H NMR (500 MHz) spectrum of compound **3d** in $CDCl_3$ following purification*

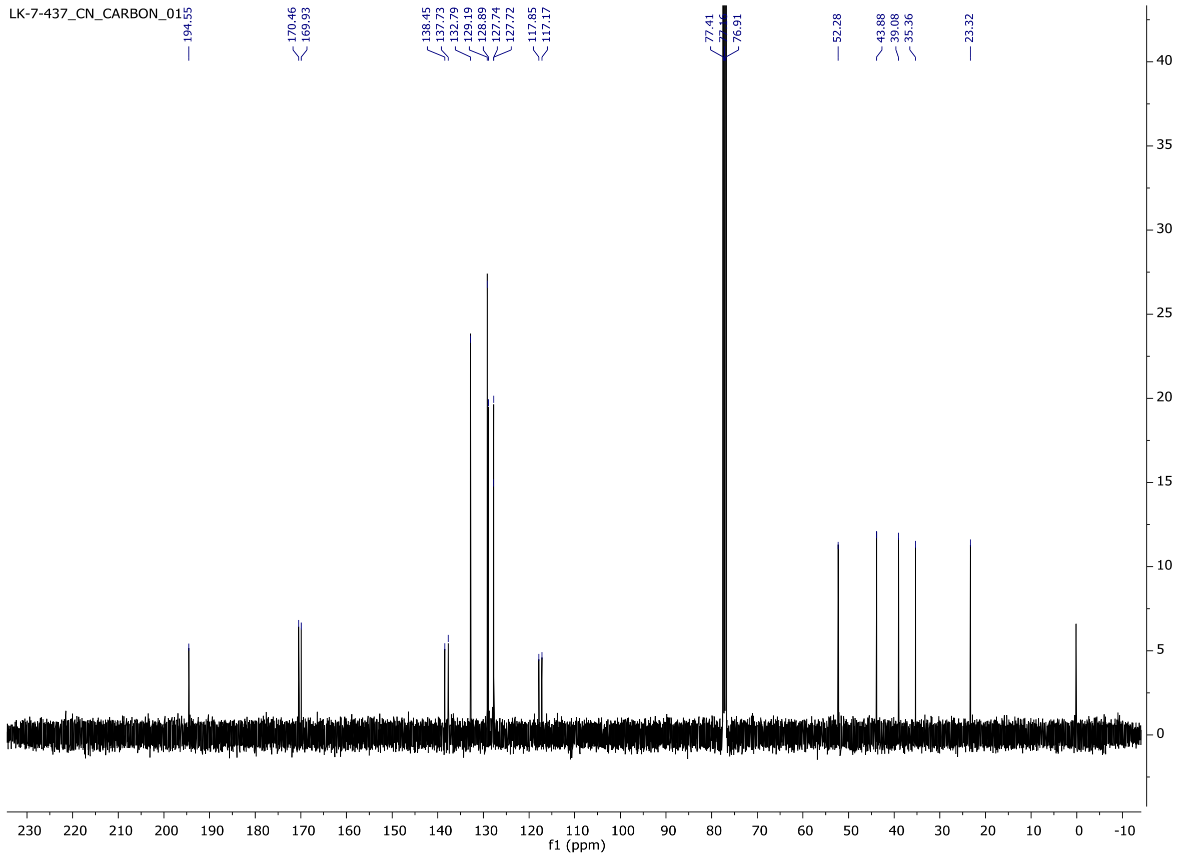

*Figure M26: ^{13}C NMR (125 MHz) spectrum of compound **3d** in $CDCl_3$ following purification*

Preparation of compound 3e

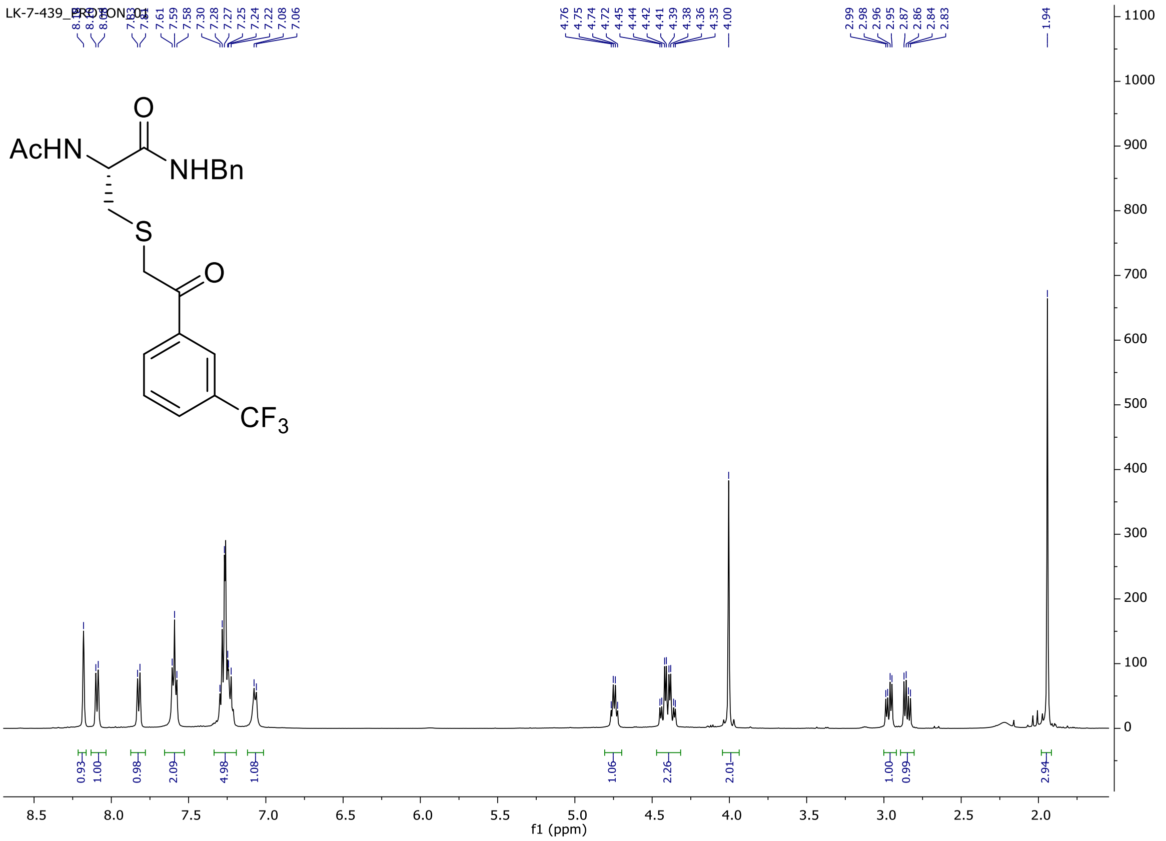

*Figure M27: ^{1}H NMR (500 MHz) spectrum of compound **3e** in $CDCl_3$ following purification*

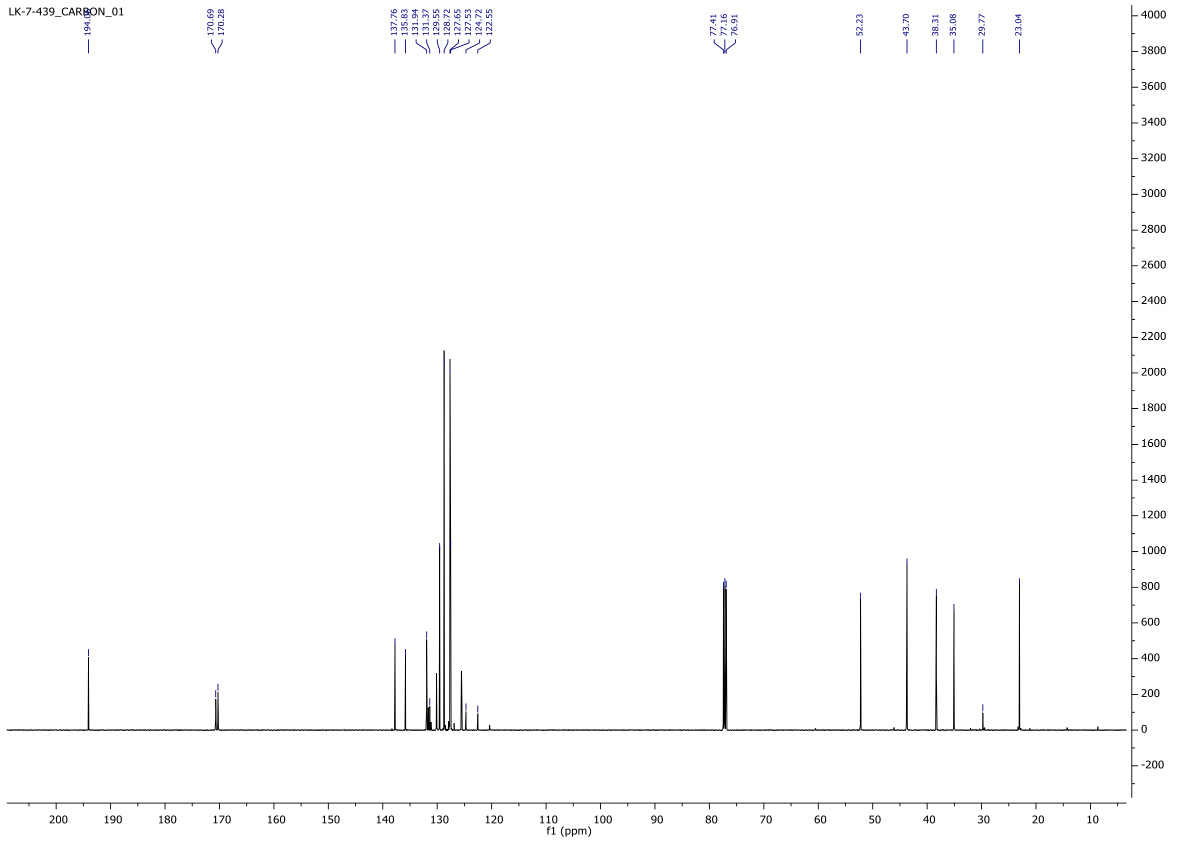

*Figure M28: ^{13}C NMR (125 MHz) spectrum of compound **3e** in $CDCl_3$ following purification*

Preparation of compound 3f

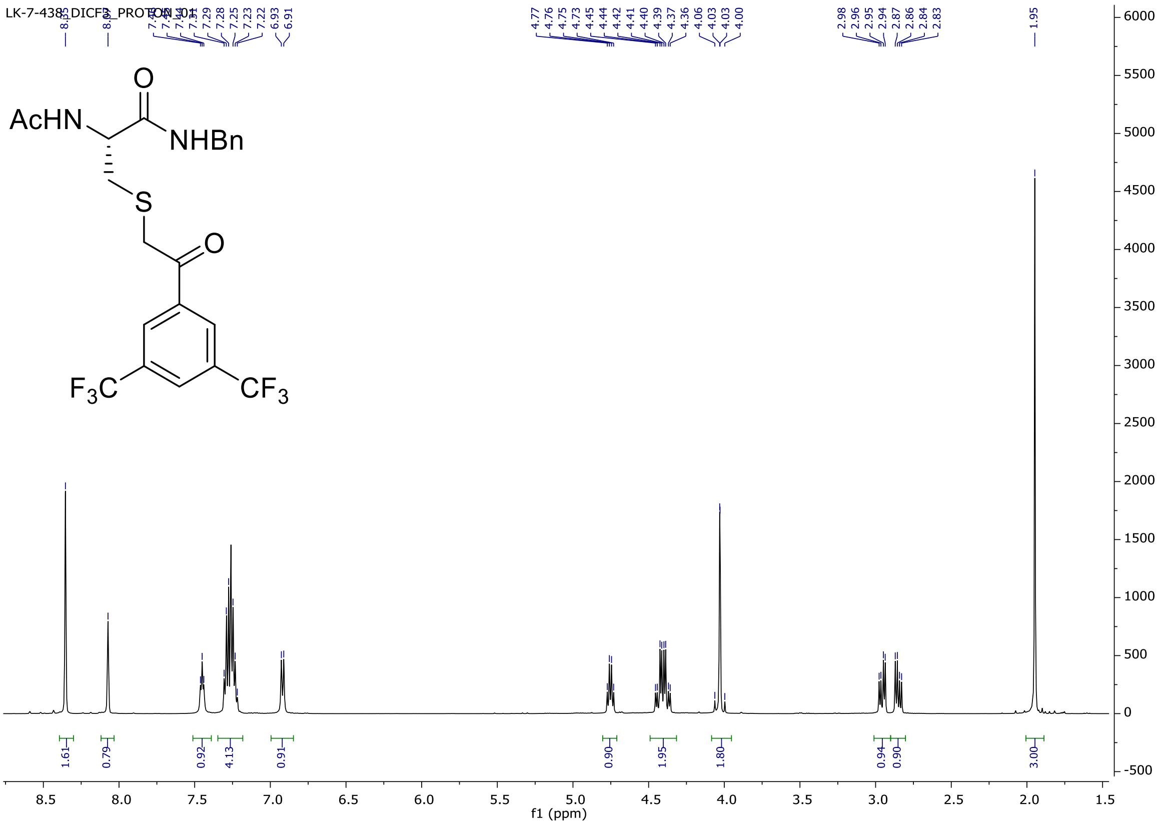

*Figure M29: ¹H NMR (500 MHz) spectrum of compound **3f** in $CDCl_3$ following purification*

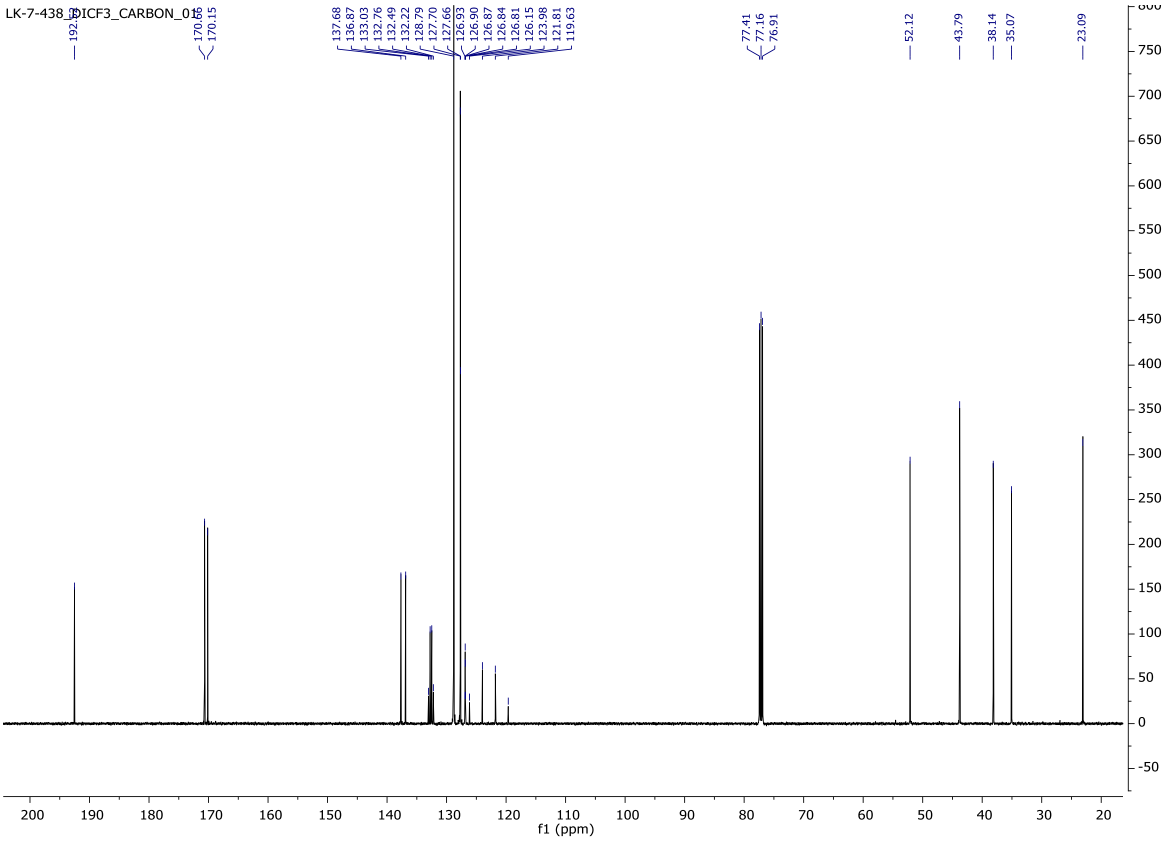

*Figure M20: ^{13}C NMR (125 MHz) spectrum of compound **3f** in $CDCl_3$ following purification*

Preparation of compound 3g

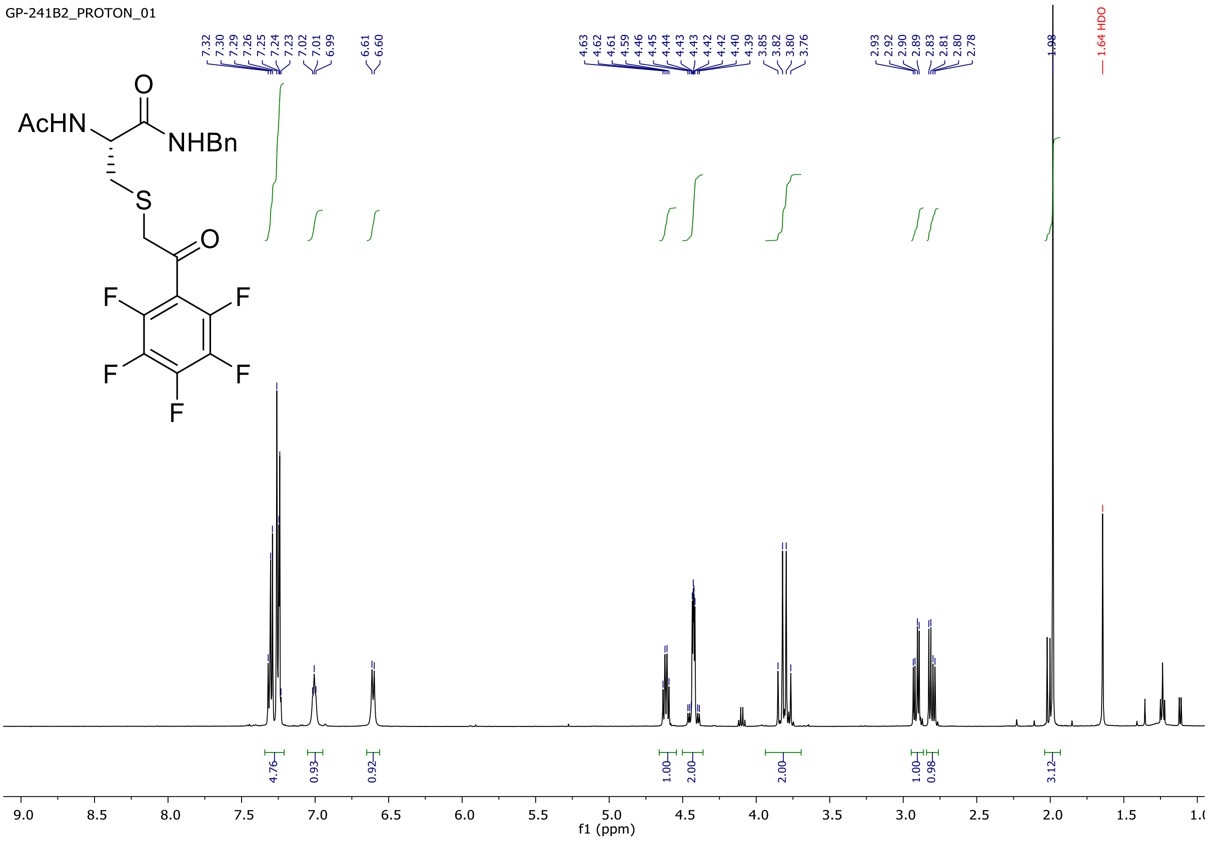

*Figure M31: ^{1}H NMR (500 MHz) spectrum of compound **3g** in $CDCl_3$ following purification*

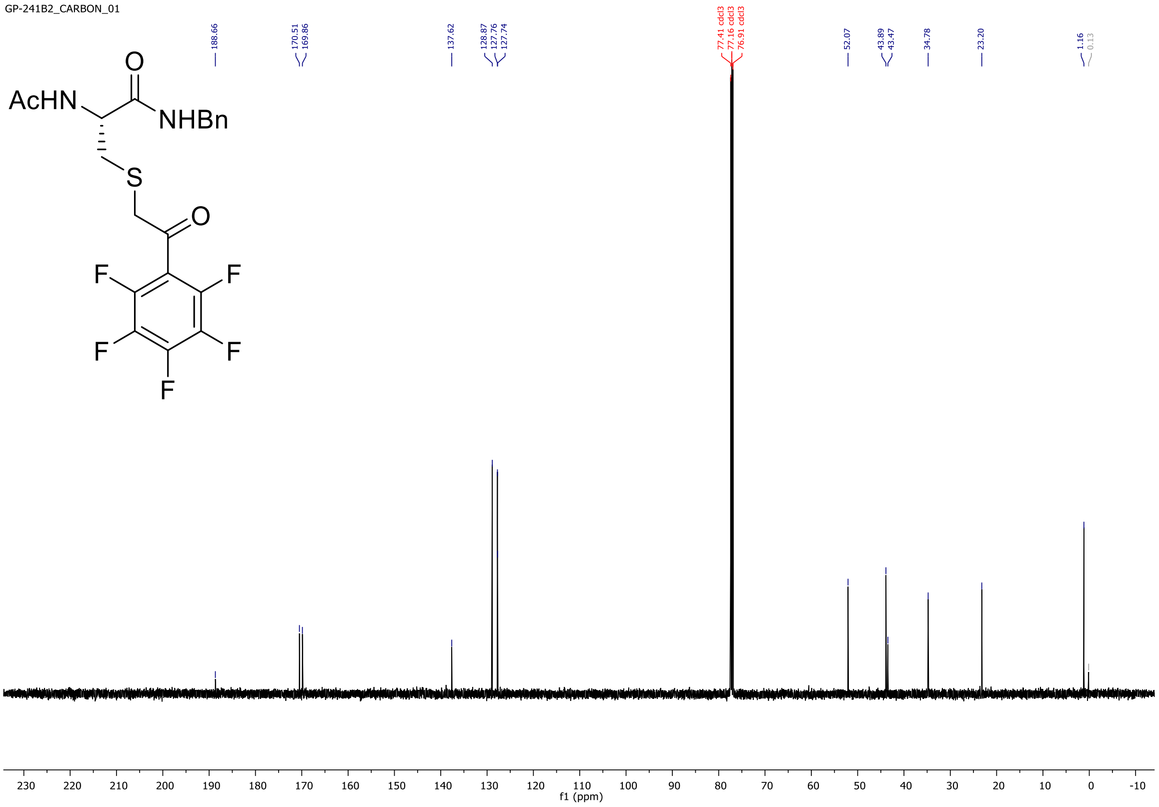

*Figure M32: ^{13}C NMR (125 MHz) spectrum of compound **3g** in CDCl3 following purification*

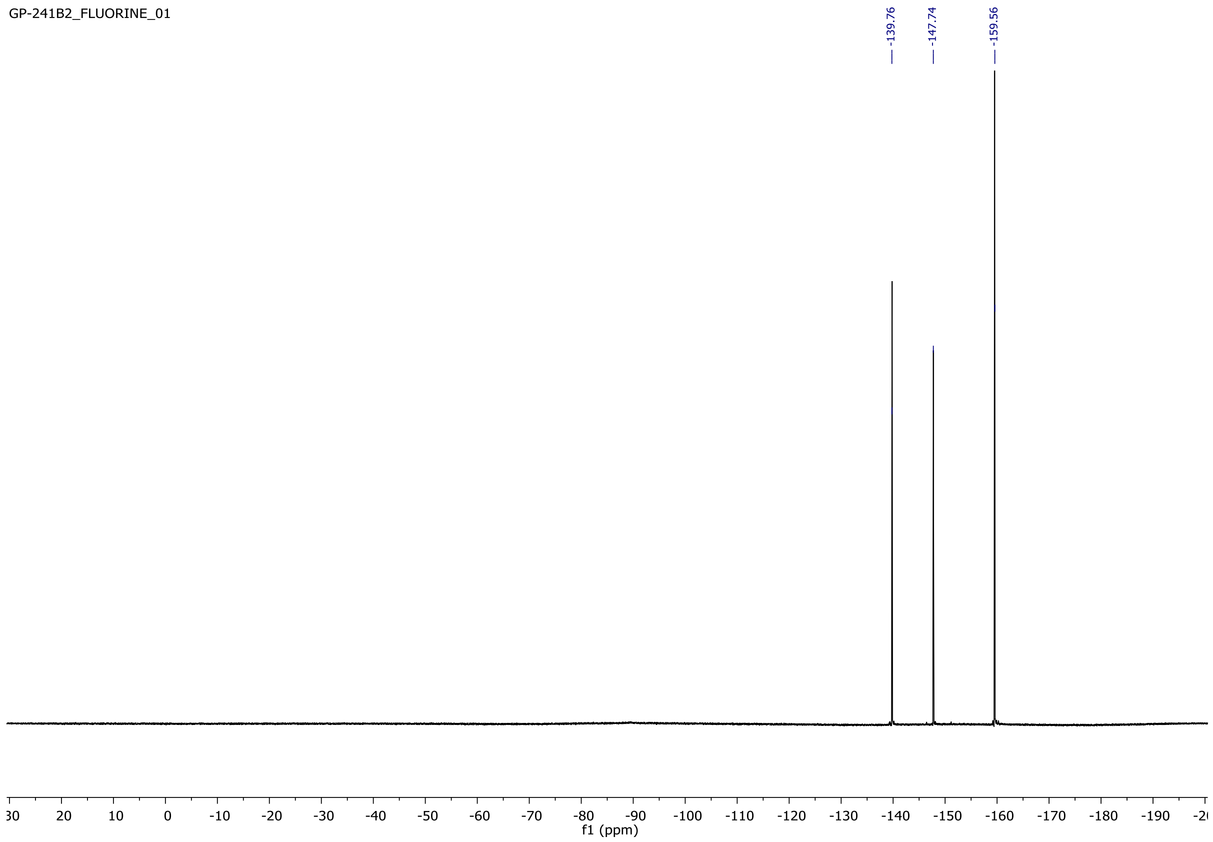

*Figure M33: ^{19}F NMR (470 MHz) spectrum of compound **3g** in $CDCl_3$ following purification*

Preparation of compound 3h

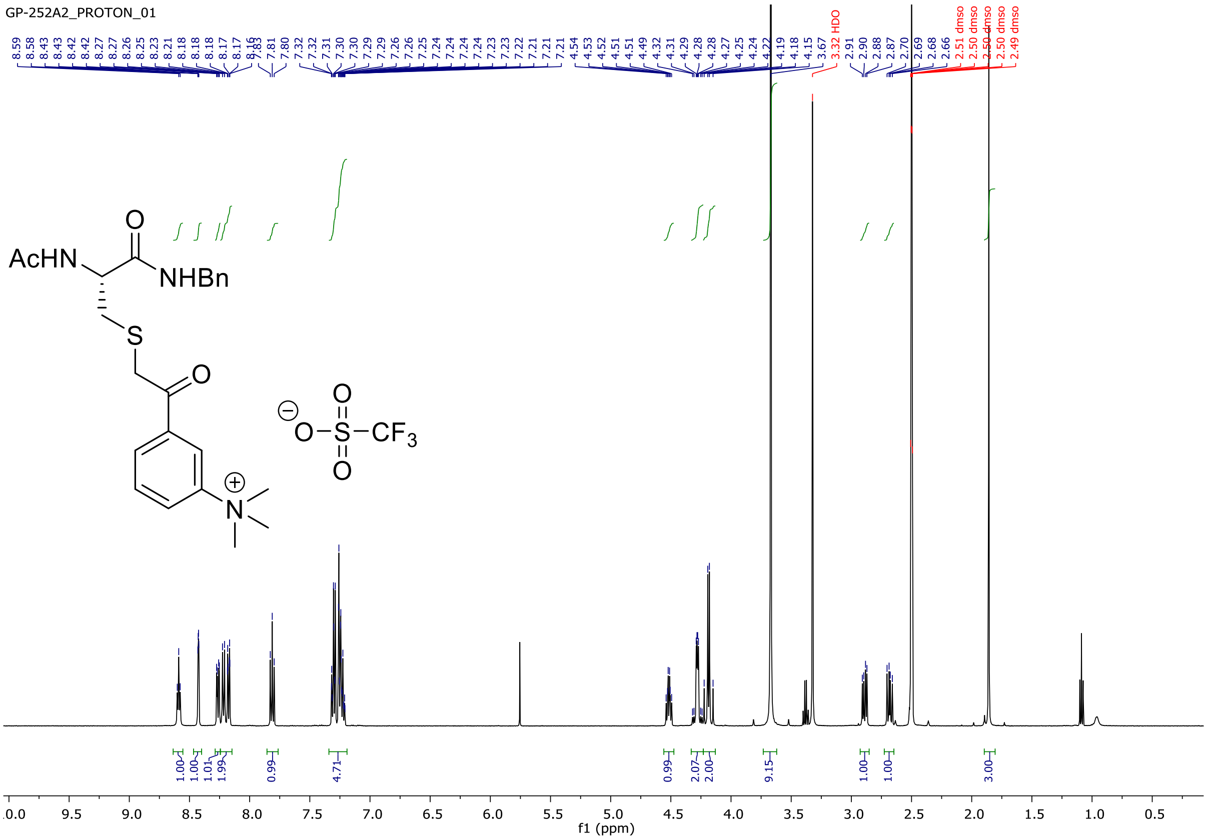

*Figure M34: ^{1}H NMR (500 MHz) spectrum of compound **3h** in DMSO-d_6 following purification*

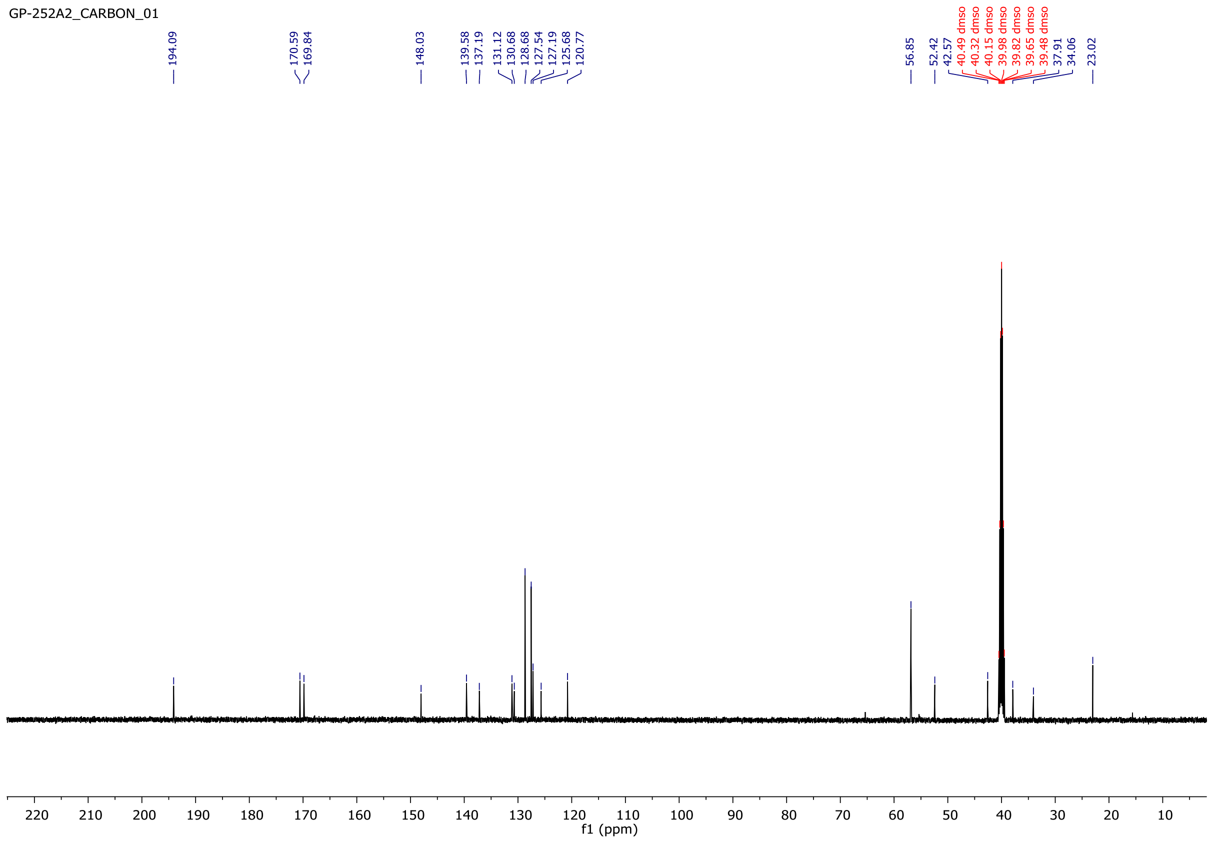

*Figure M35: ^{13}C NMR (125 MHz) spectrum of compound **3h** in DMSO-d_6 following purification*

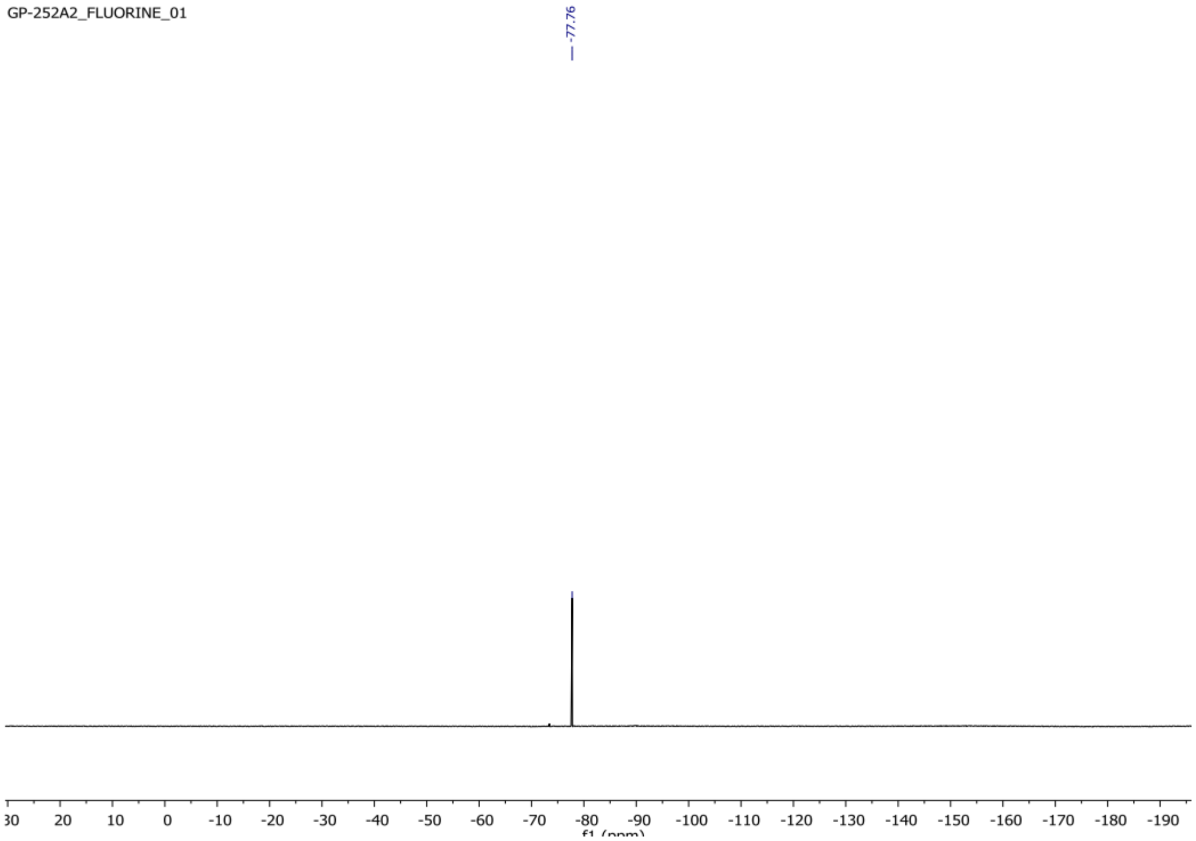

Figure M36: ^{19}F NMR (470 MHz) spectrum of compound ***3h*** *in DMSO-d_6 following purification*

Preparation of compound 3i

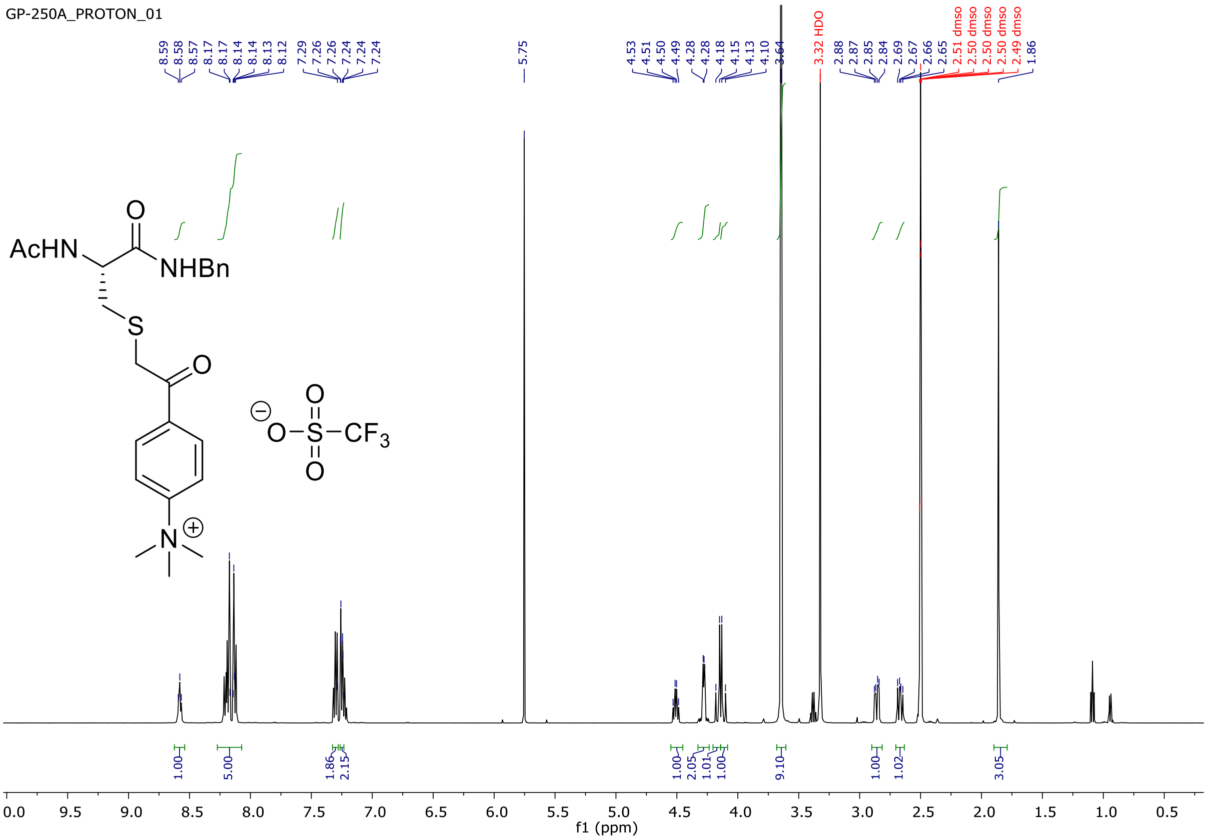

Figure M37: ^{1}H NMR (500 MHz) spectrum of compound **3i** in DMSO-d_6 following purification

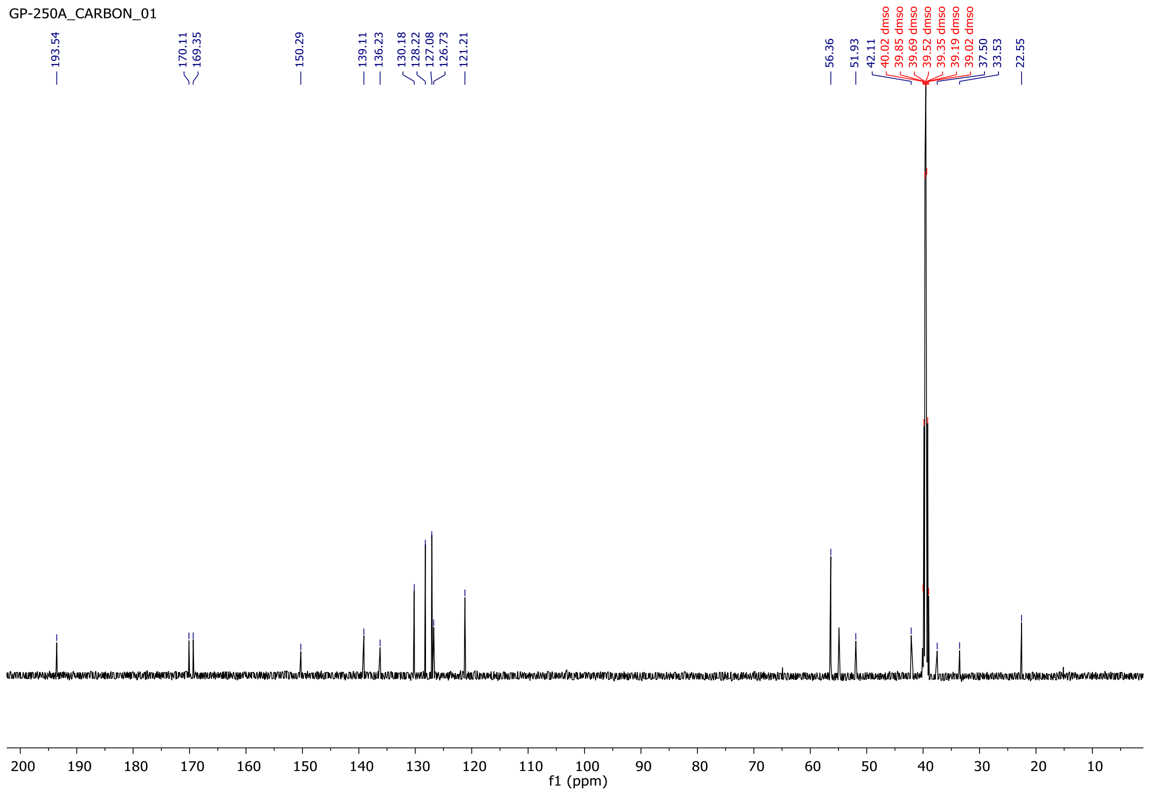

*Figure M38: ^{13}C NMR (125 MHz) spectrum of compound **3i** in DMSO-d_6 following purification*

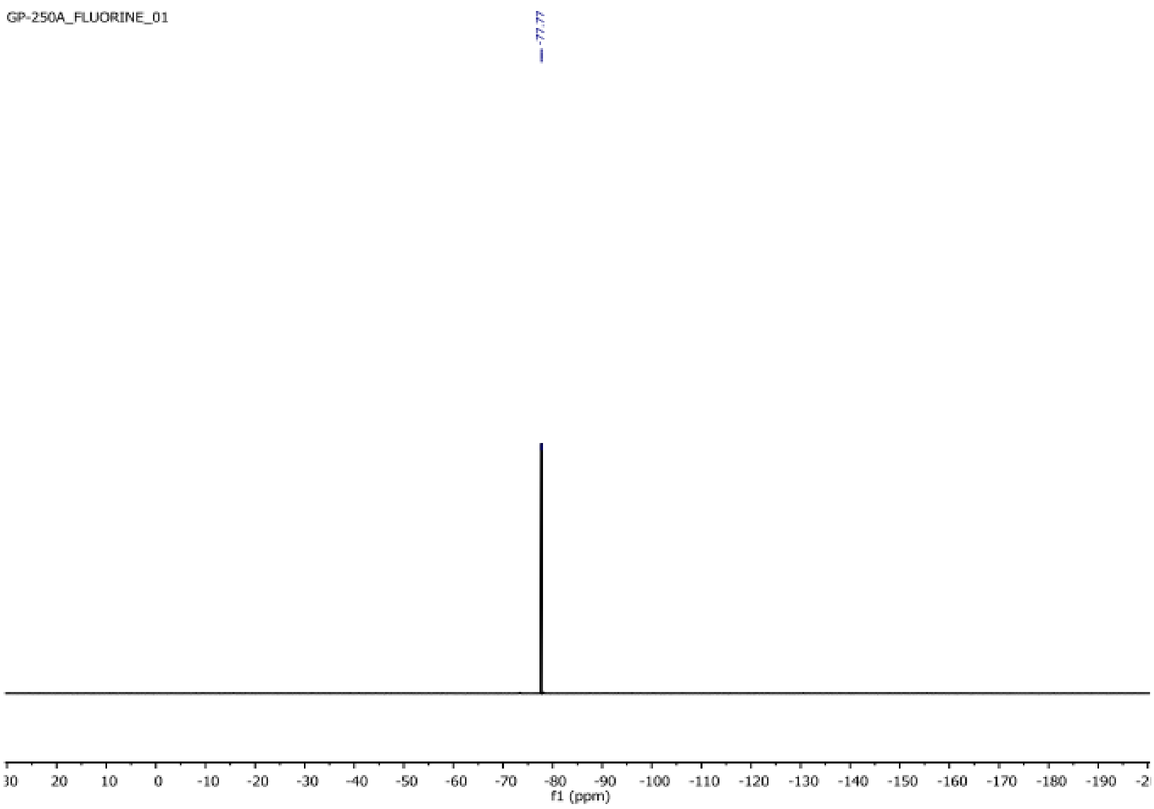

*Figure M39: ^{19}F NMR (470 MHz) spectrum of compound **3i** in DMSO-d_6 following purification*

Preparation of compound 3j

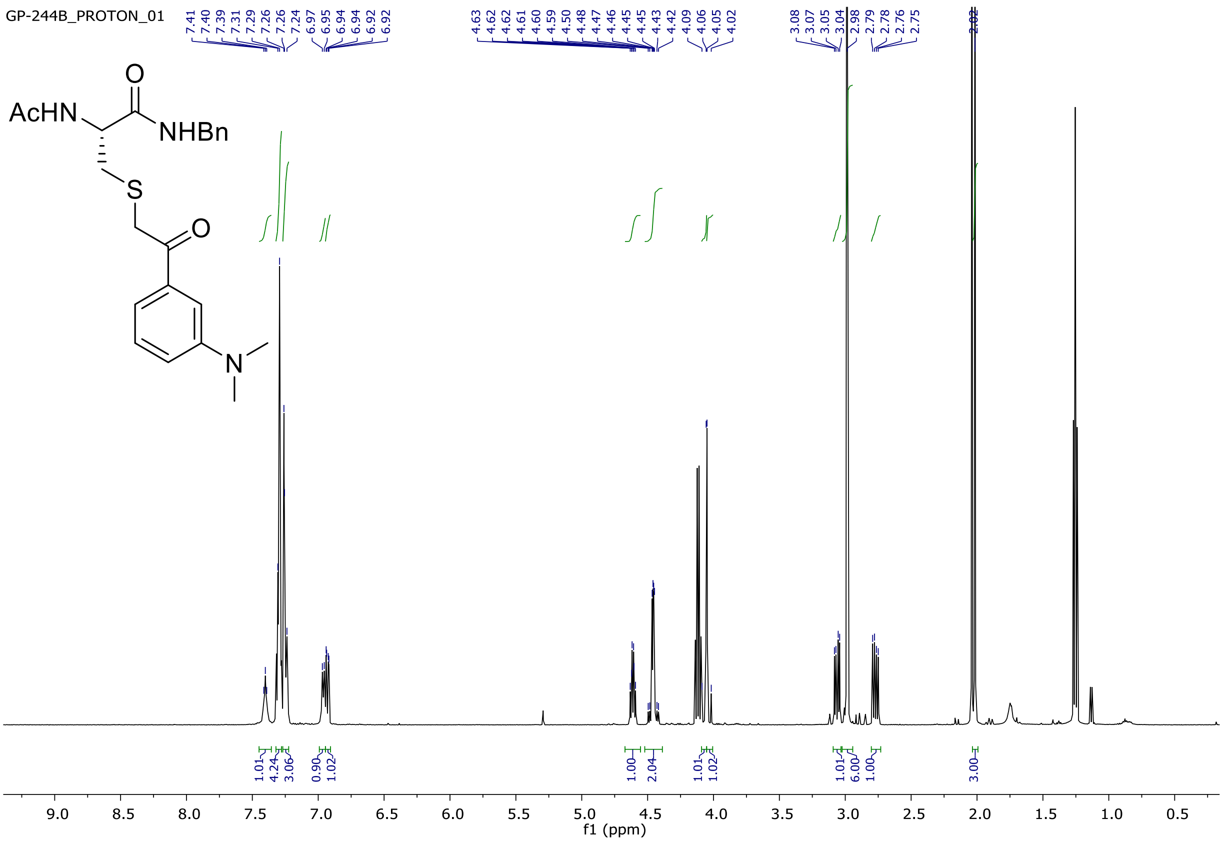

*Figure M40: ^{1}H NMR (500 MHz) spectrum of compound **3j** in $CDCl_3$ following purification*

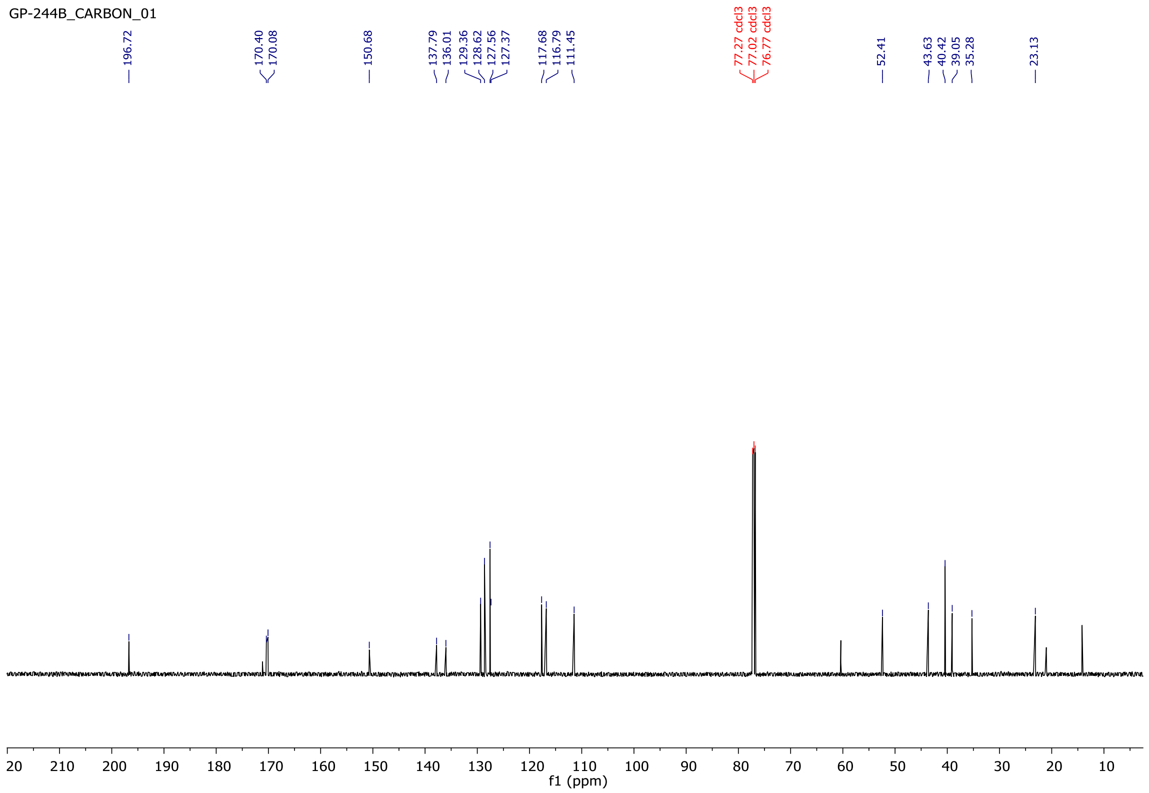

*Figure M41: ^{13}C NMR (125 MHz) spectrum of compound **3j** in $CDCl_3$ following purification*

Preparation of compound 3k

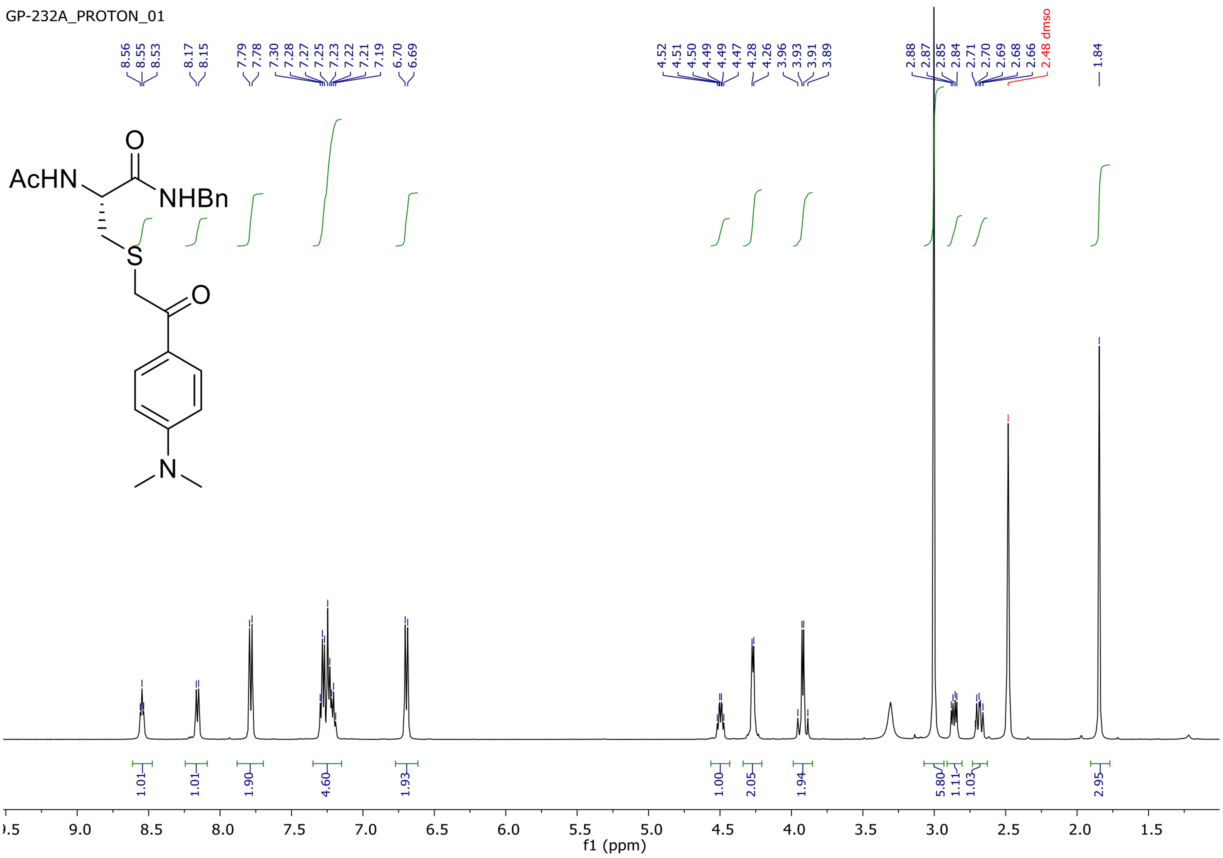

*Figure M42: ^{1}H NMR (500 MHz) spectrum of compound **3k** in DMSO-d_6 following purification*

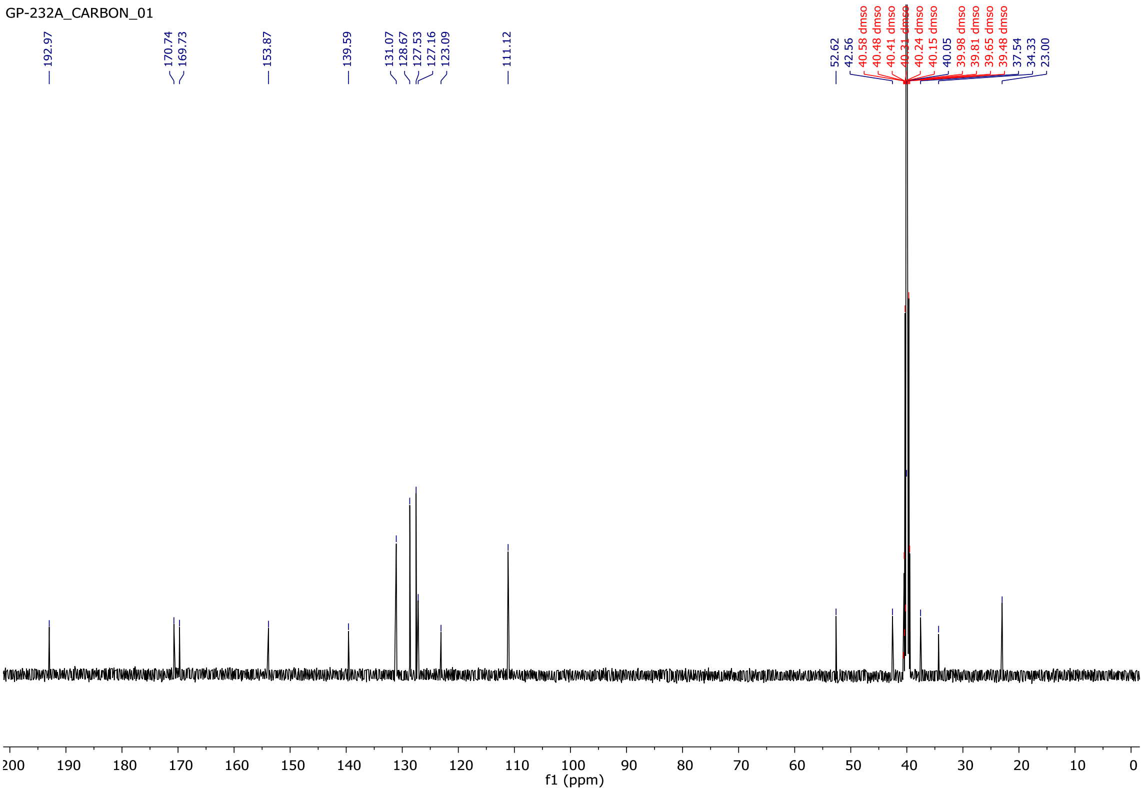

*Figure M43: ^{13}C NMR (125 MHz) spectrum of compound **3k** in DMSO-d_6 following purification*

Preparation of compound 3l

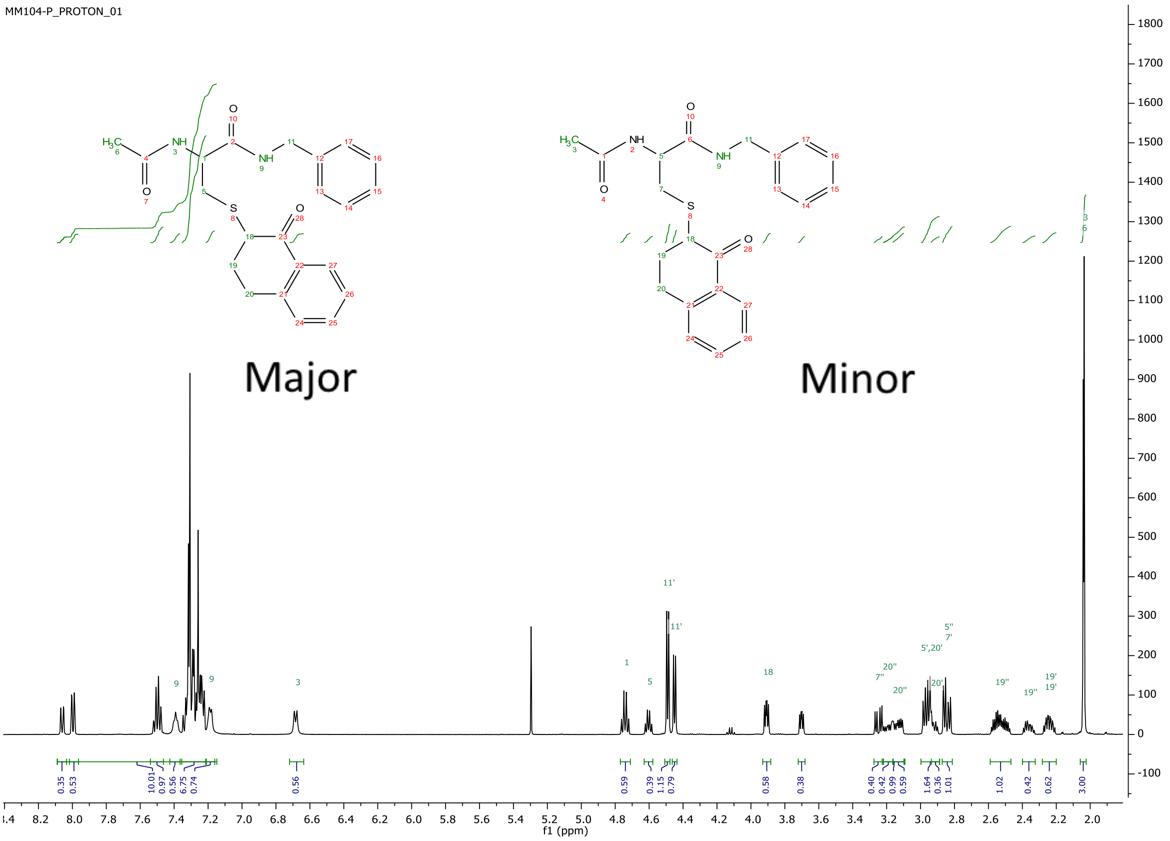

*Figure M44: ^{1}H NMR (500 MHz) spectrum of compound **3l** in $CDCl_3$ following purification*

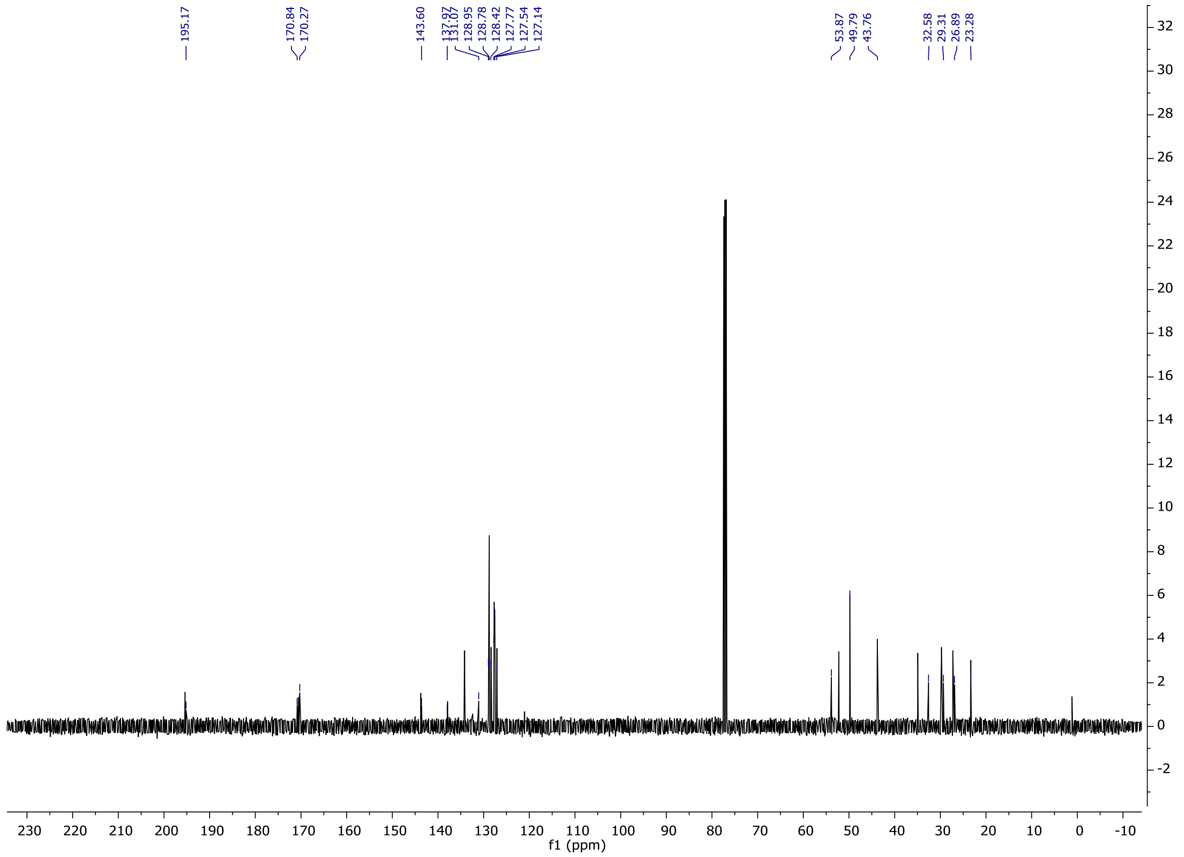

*Figure M45: ^{13}C NMR (125 MHz) spectrum of compound **3l** in $CDCl_3$ following purification*

Photolysis of simple cysteine phenacylsulfide derivatives

Photolysis of compound 3a

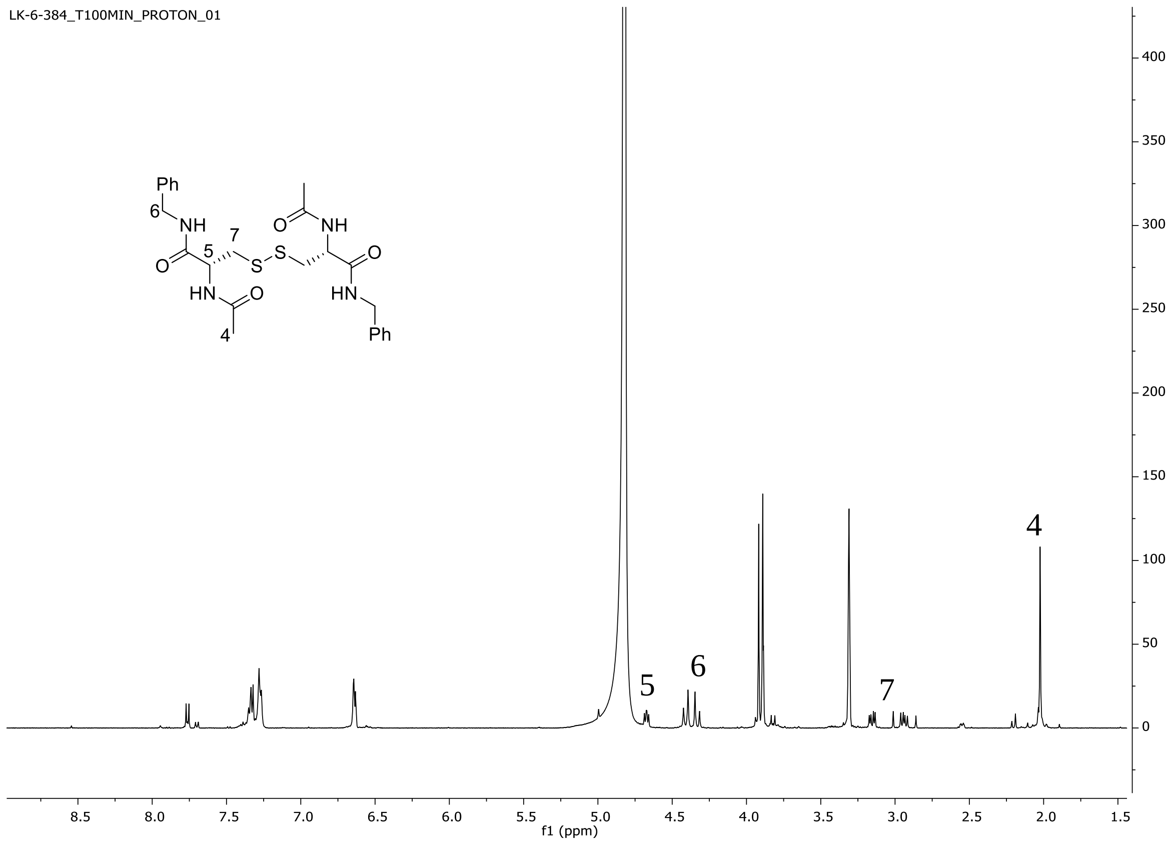

*Figure M46: ^{1}H NMR (500 MHz) spectrum of the reaction mixture following photolysis of compound **3a** in D_2O (0.1 M phosphate, pD 7.8):MeOD (1:1). Characteristic signals of each of the reaction products are highlighted.*

Photolysis of compound 3b

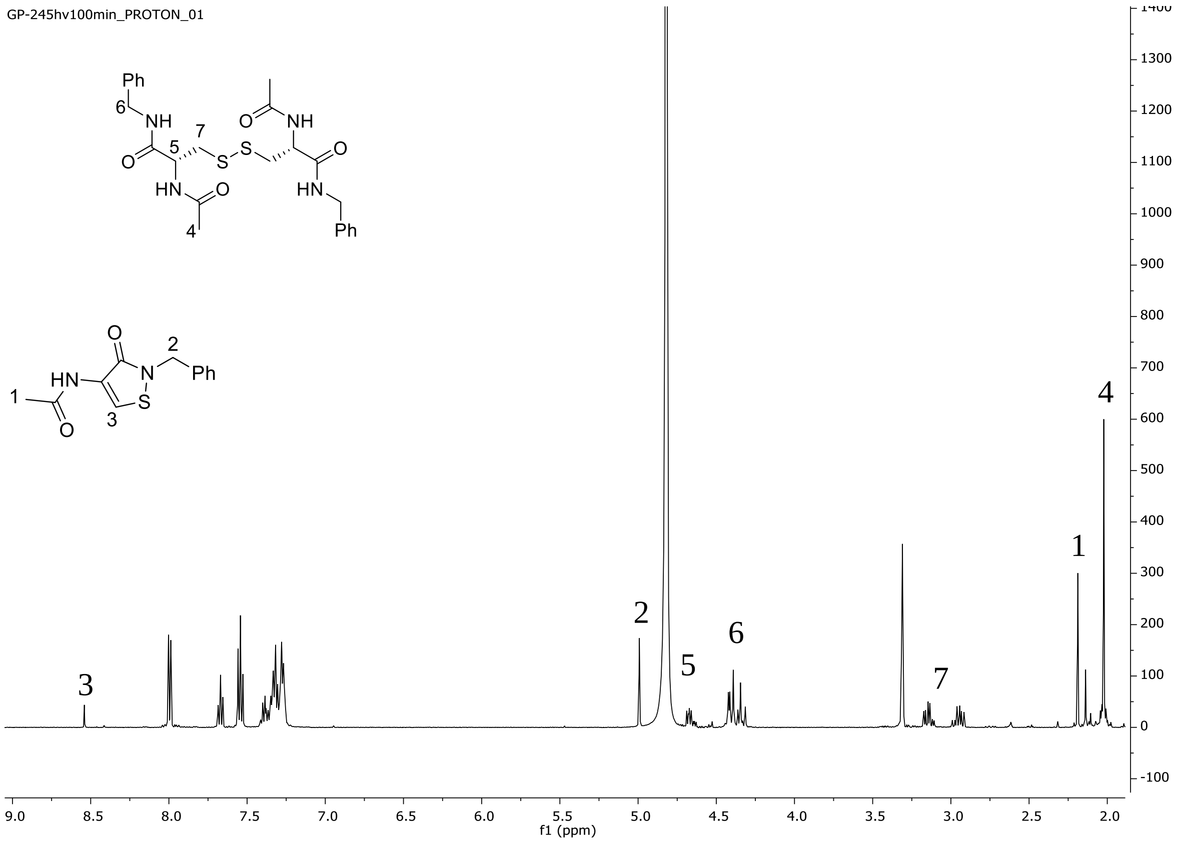

Figure M47: ^{1}H NMR (500 MHz) spectrum of the reaction mixture derived from the photolysis of compound ***3b*** *in D_2O (0.1 M phosphate, pD 7.8):MeOD (1:1). Characteristic signals of each of the reaction products are highlighted. Note: disulfide shows evidence of racemization as described in section E, with a diastereomeric ratio of ~3:1*

Photolysis of compound 3b with MeCN-D$_3$ co-solvent

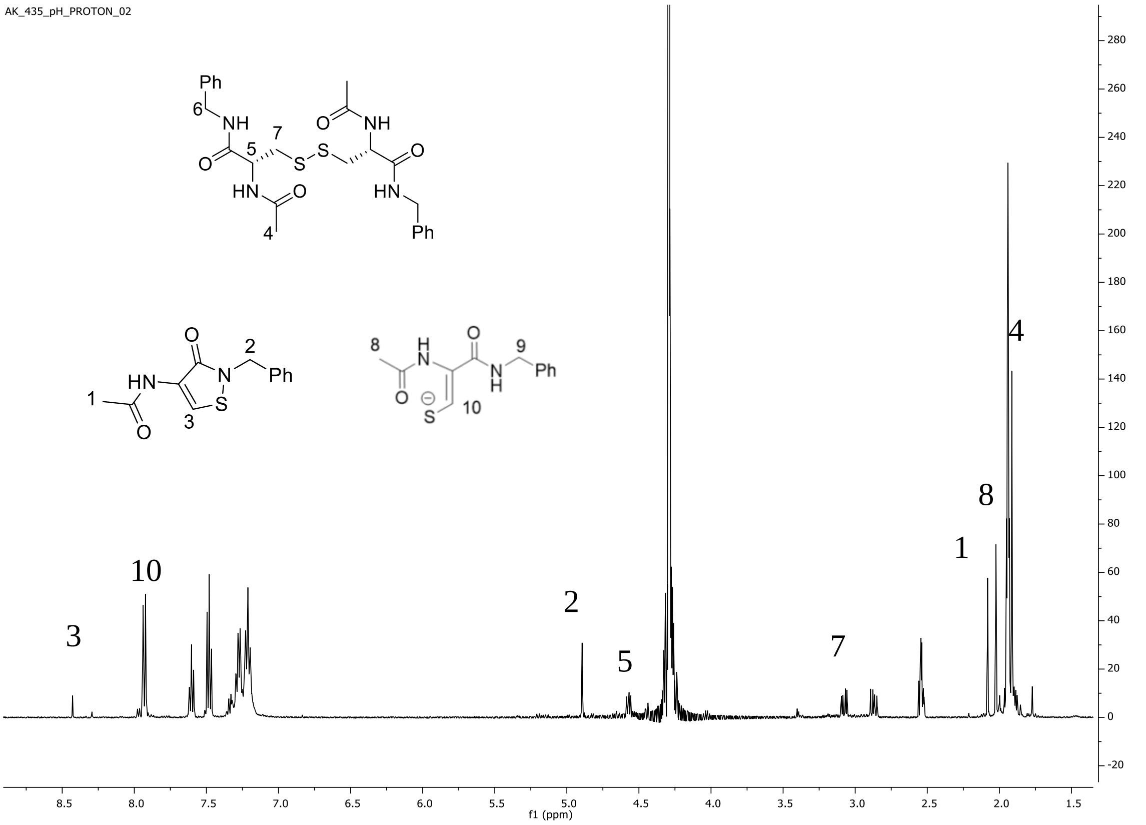

*Figure M48:^{1}H NMR (600 MHz) spectrum of the reaction mixture derived from the photolysis of compound **3b** in D_2O (0.1 M phosphate, pD 7.8): MeCN-D_3 (1:1). Characteristic signals of each of the reaction products are highlighted. Note: the signal for the methylene protons (6) of disulfide are obscured by the HDO solvent signal at 4.99 ppm. The signal for methine proton (10) of enethiol is obscured by the aromatics signals.*

Photolysis of compound 3c

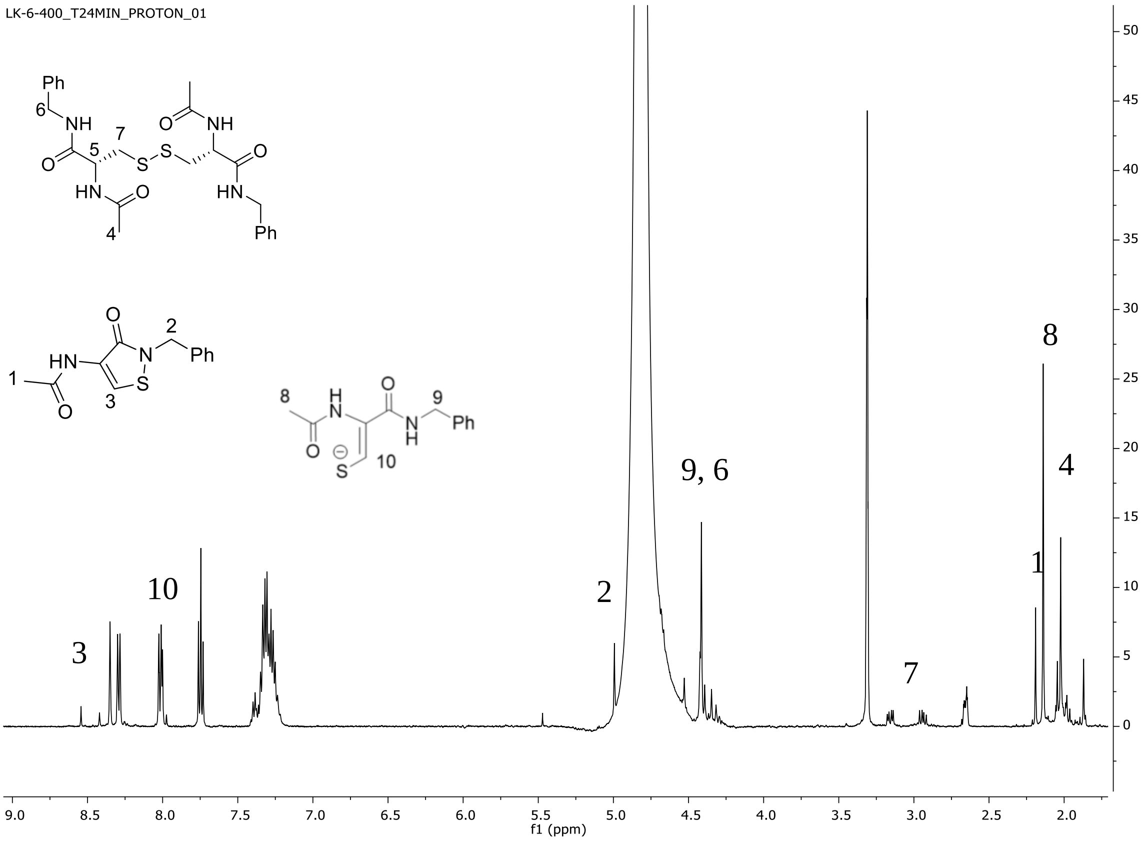

*Figure M49: ^{1}H NMR (500 MHz) spectrum of the reaction mixture following photolysis of compound **3c** in D_2O (0.1 M phosphate, pD 7.8):MeOD (1:1). Characteristic signals of each of the reaction products are highlighted. Note: the signal for methine proton (5) of the disulfide is obscured by the HDO solvent signal at 4.99 ppm.*

Photolysis of compound 3d

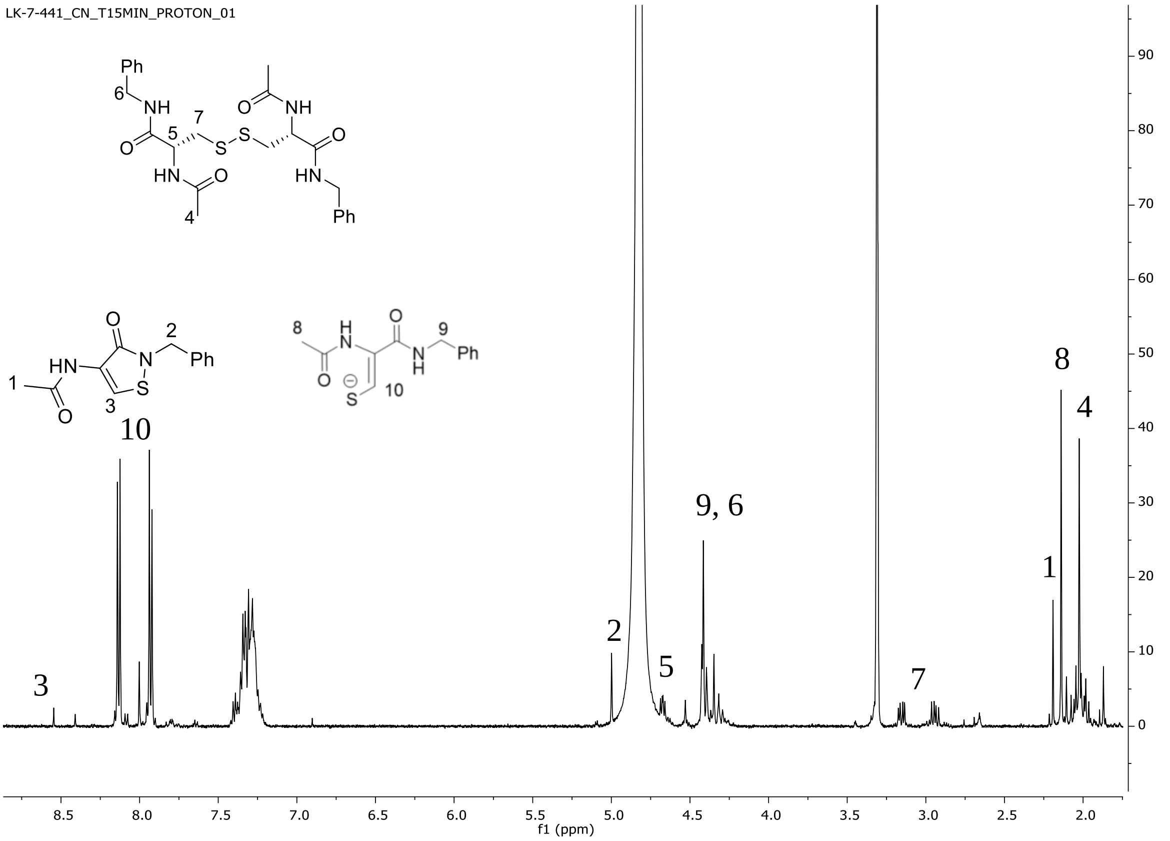

*Figure M50: ^{1}H NMR (500 MHz) spectrum of the reaction mixture following photolysis of compound **3d** in D_2O (0.1 M phosphate, pD 7.8):MeOD (1:1). Characteristic signals of each of the reaction products are highlighted.*

Photolysis of compound 3e

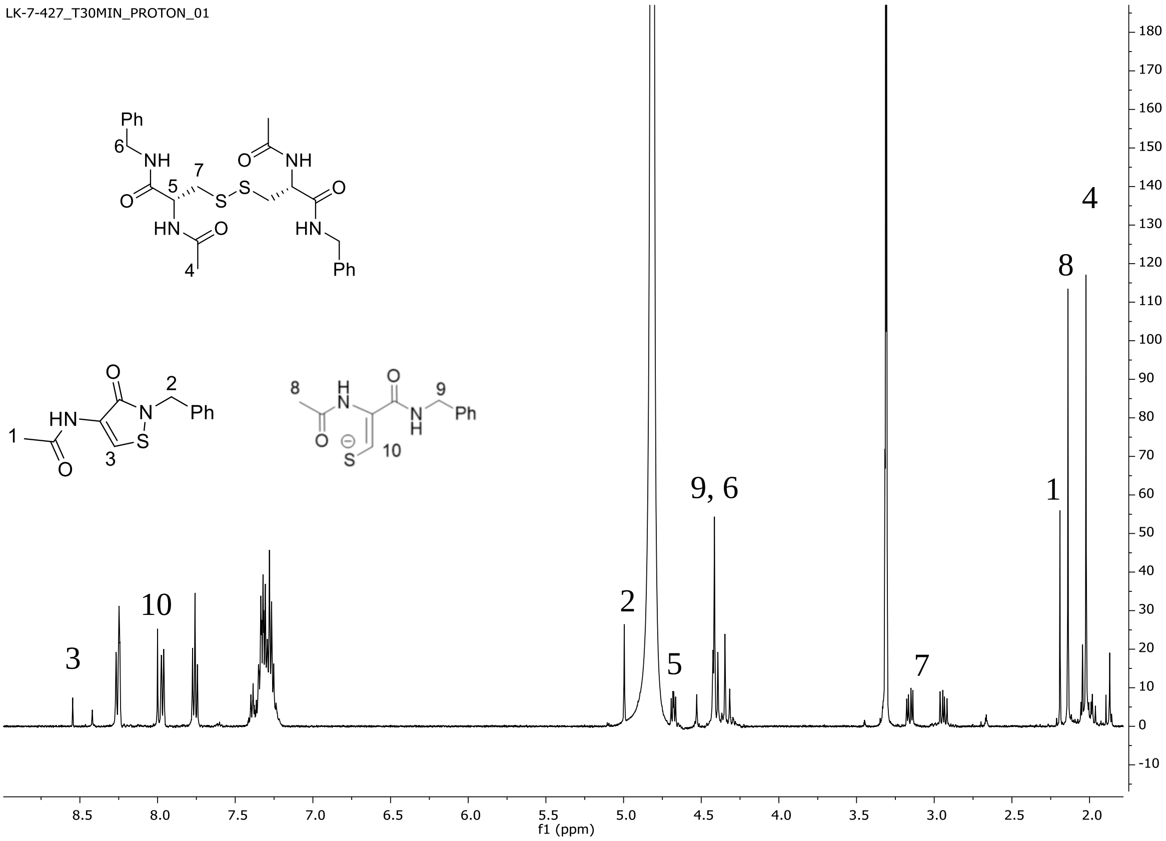

*Figure M51: ^{1}H NMR (500 MHz) spectrum of the reaction mixture following photolysis of compound **3e** in D_2O (0.1 M phosphate, pD 7.8):MeOD (1:1). Characteristic signals of each of the reaction products are highlighted.*

Photolysis of compound 3f

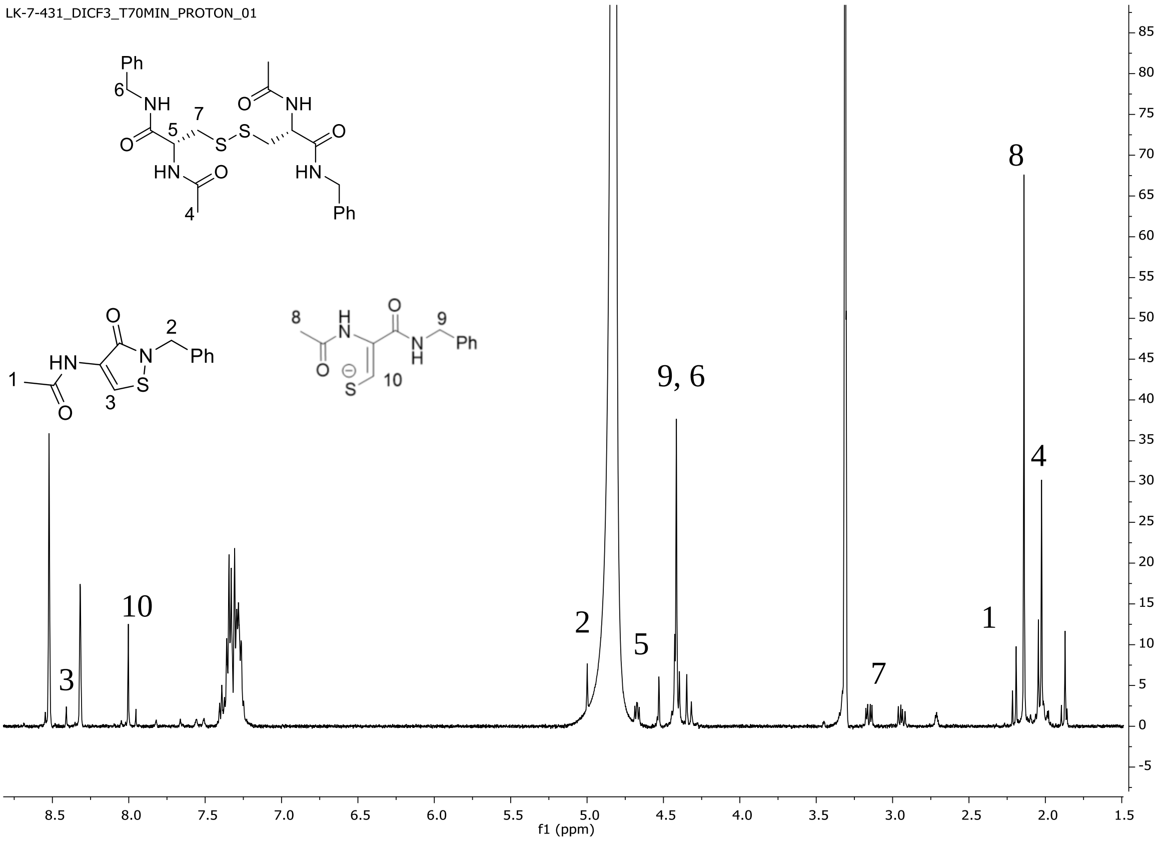

*Figure M52: ^{1}H NMR (500 MHz) spectrum of the reaction mixture following photolysis of compound **3f** in D_2O (0.1 M phosphate, pD 7.8):MeOD (1:1). Characteristic signals of each of the reaction products are highlighted.*

Photolysis of compound 3g

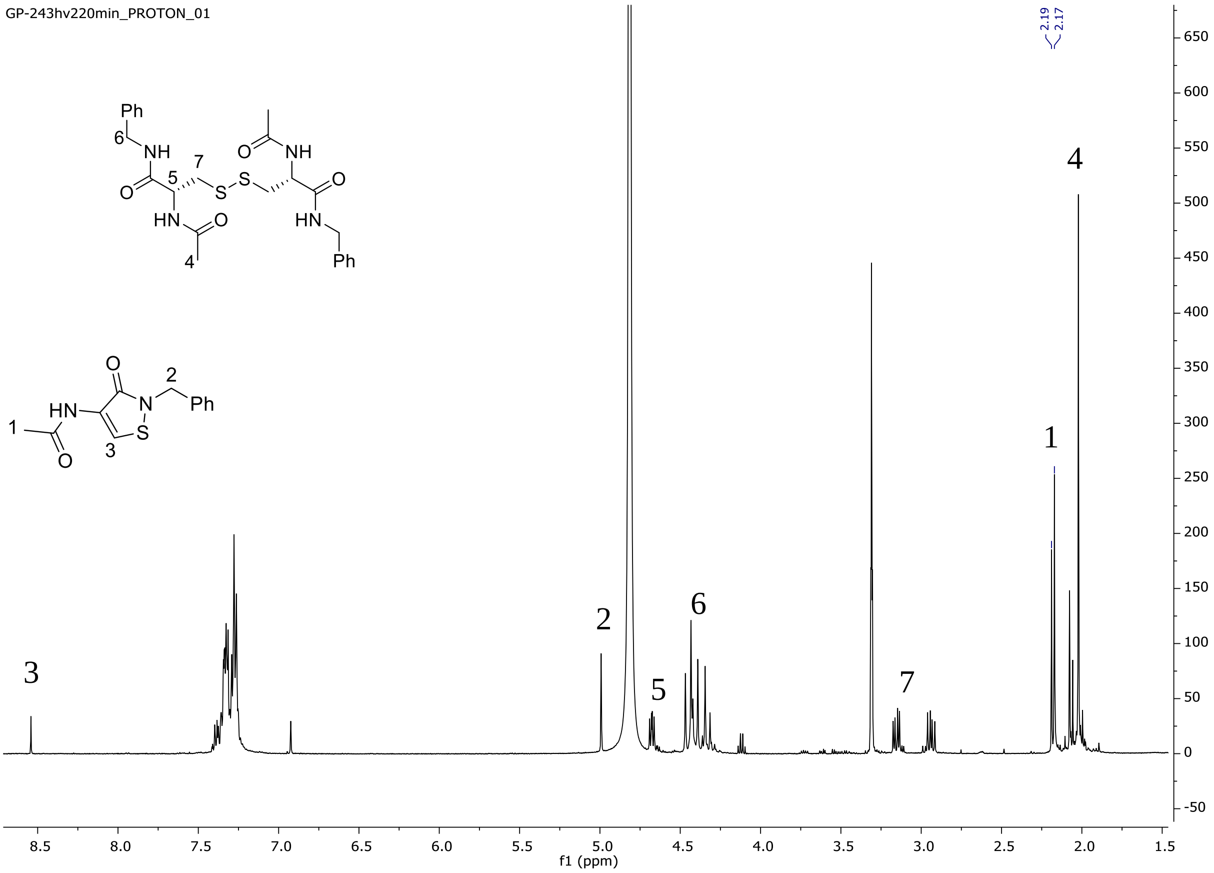

*Figure M53: ^{1}H NMR (500 MHz) spectrum of the reaction mixture following photolysis of compound **3g** in D_2O (0.1 M phosphate, pD 7.8):MeOD (1:1). Characteristic signals of each of the reaction products are highlighted. Note: disulfide shows evidence of racemization as described in section E, with a diastereomeric ratio of ~8:1*

Photolysis of compound 3h

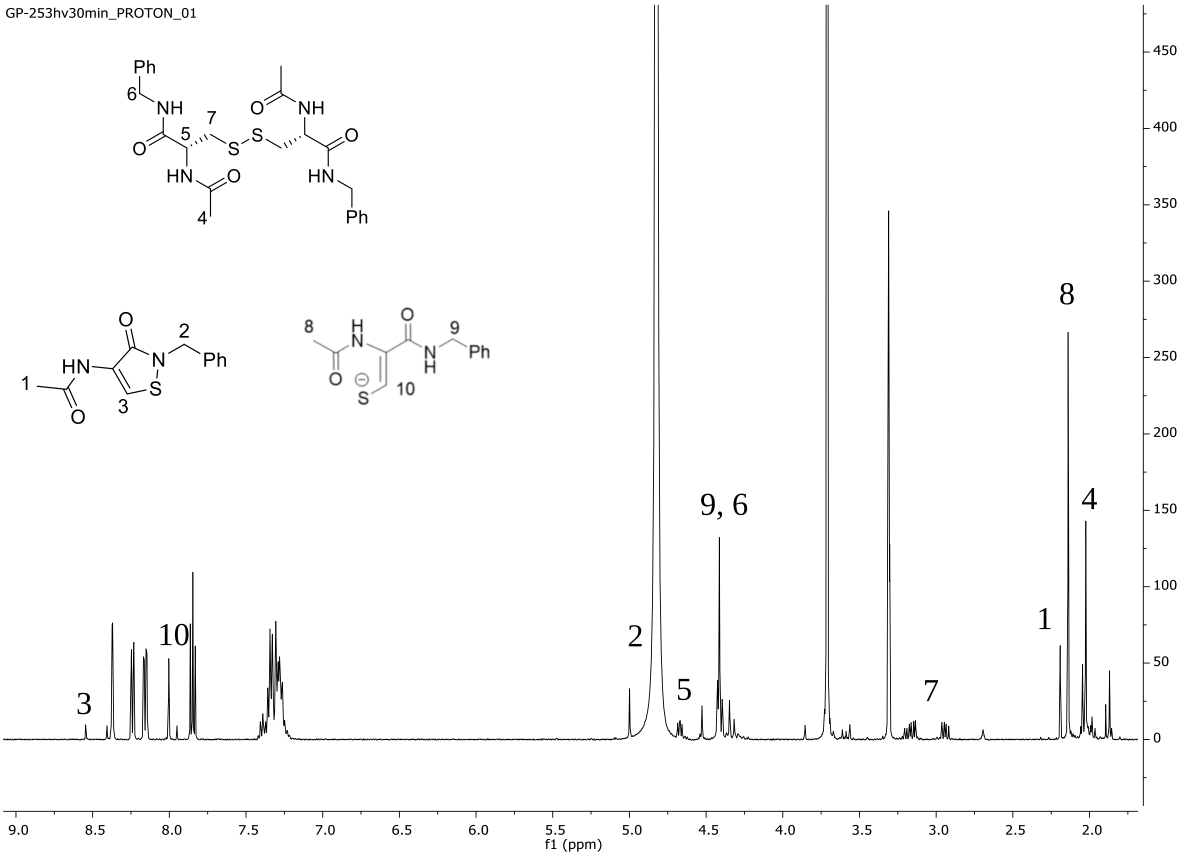

Figure M54: ^{1}H NMR (500 MHz) spectrum of the reaction mixture following photolysis of compound ***3h*** *in D_2O (0.1 M phosphate, pD 7.8):MeOD (1:1). Characteristic signals of each of the reaction products are highlighted. Note: the quartet at 3.20 ppm is an impurity present in the starting material.*

Photolysis of compound 3i

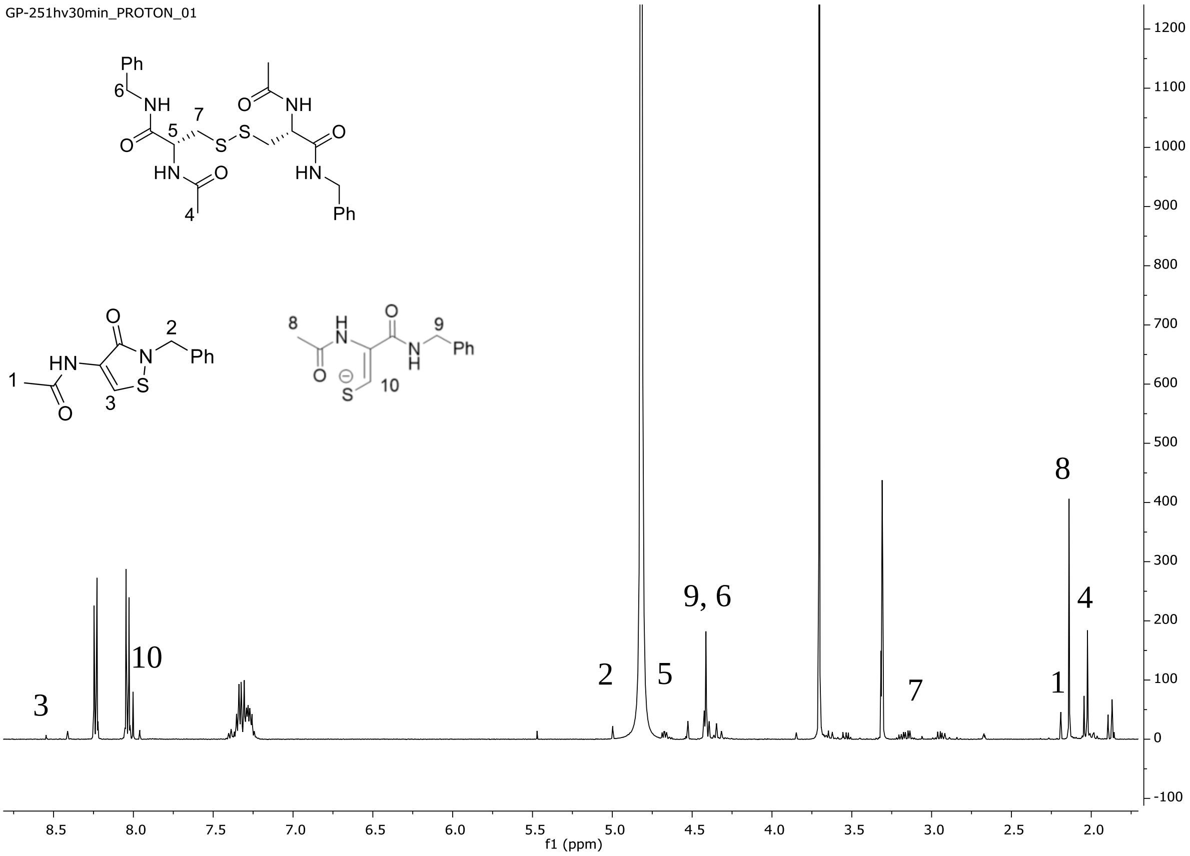

Figure M55: ^{1}H NMR (500 MHz) spectrum of the reaction mixture following photolysis of compound ***3i*** *in D_2O (0.1 M phosphate, pD 7.8):MeOD (1:1). Characteristic signals of each of the reaction products are highlighted. Note: disulfide shows evidence of racemization as described in section E, with a diastereomeric ratio of ~4:1. Note: the quartet at 3.20 ppm is an impurity present in the starting material.*

Investigation of simple cysteine isothiazolone, enethiol and disulfide derivatives

Synthesis of disulfide (4)

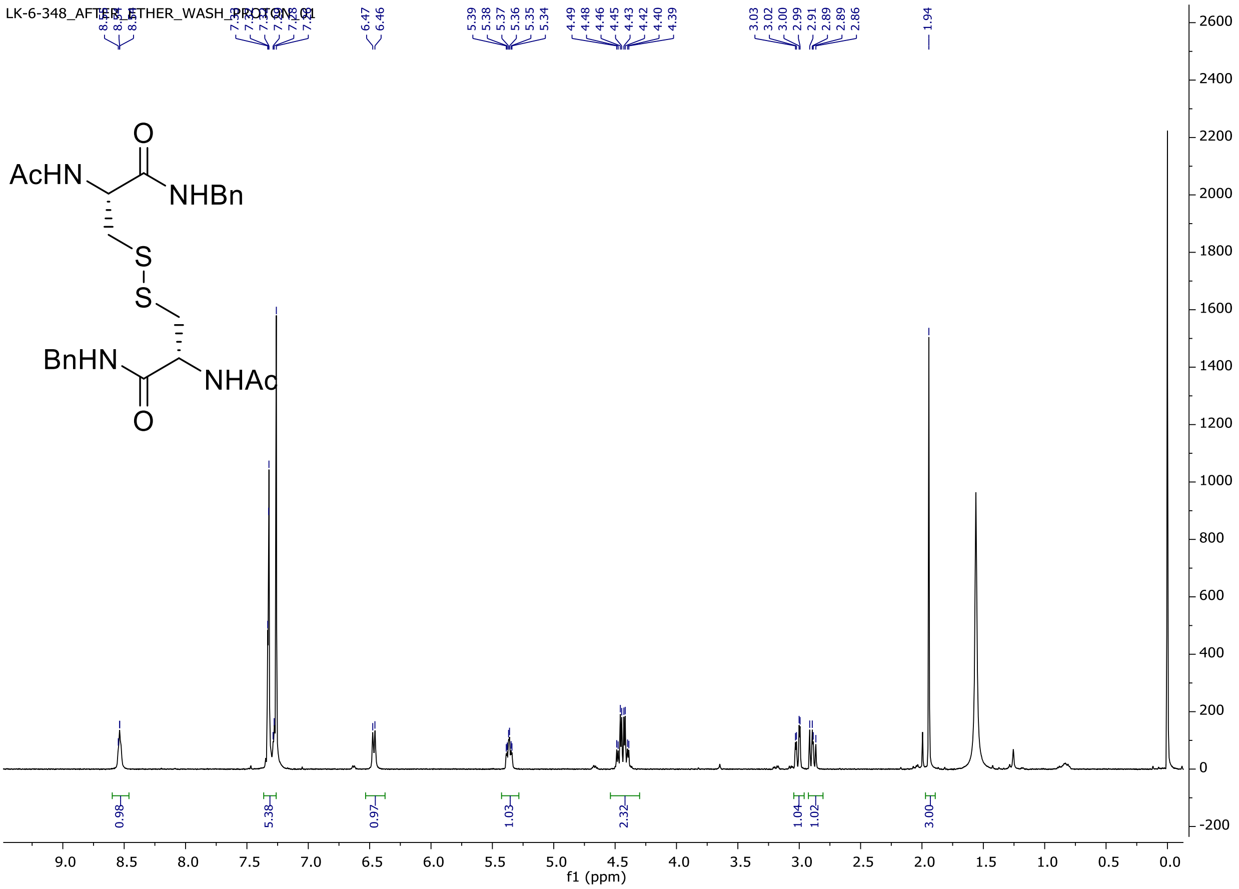

*Figure M56: ^{1}H NMR (500 MHz) spectrum of disulfide **4** in $CDCl_3$ following purification*

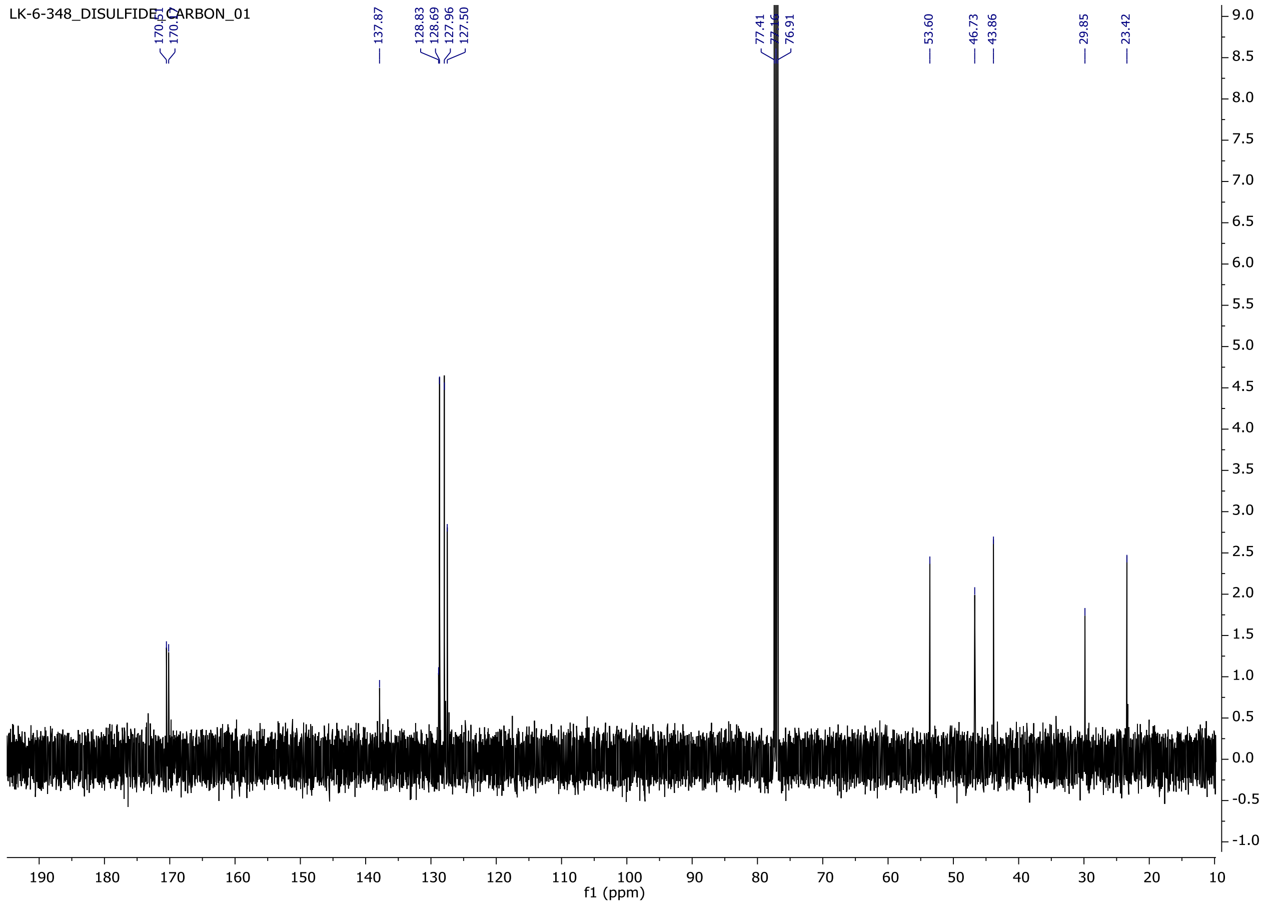

*Figure M57: ^{13}C NMR (125 MHz) spectrum of disulfide **4** in $CDCl_3$ following purification*

N-(2-benzyl-3-oxo-2,3-dihydro-1,2-thizol-4-yl)acetamide (6)

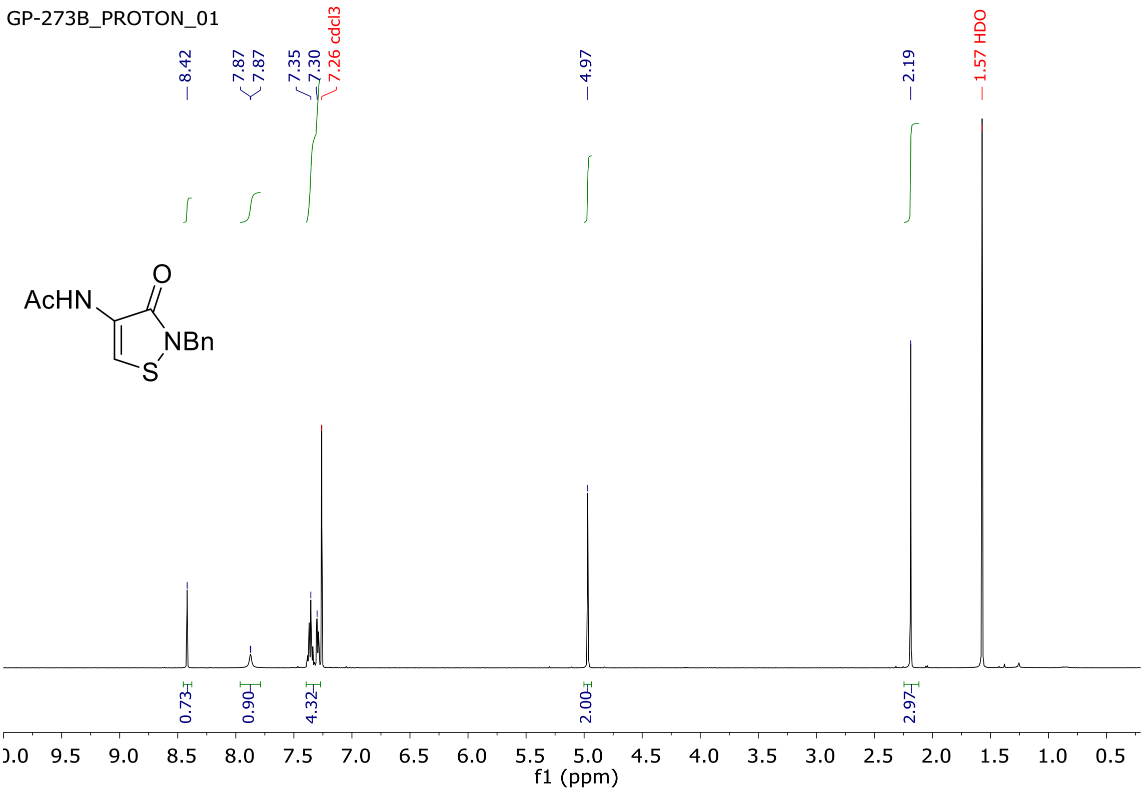

Figure M58: ^{1}H NMR spectrum of N-(2-benzyl-3-oxo-2,3-dihydro-1,2-thizol-4-yl)acetamide ***6*** *in $CDCl_3$*

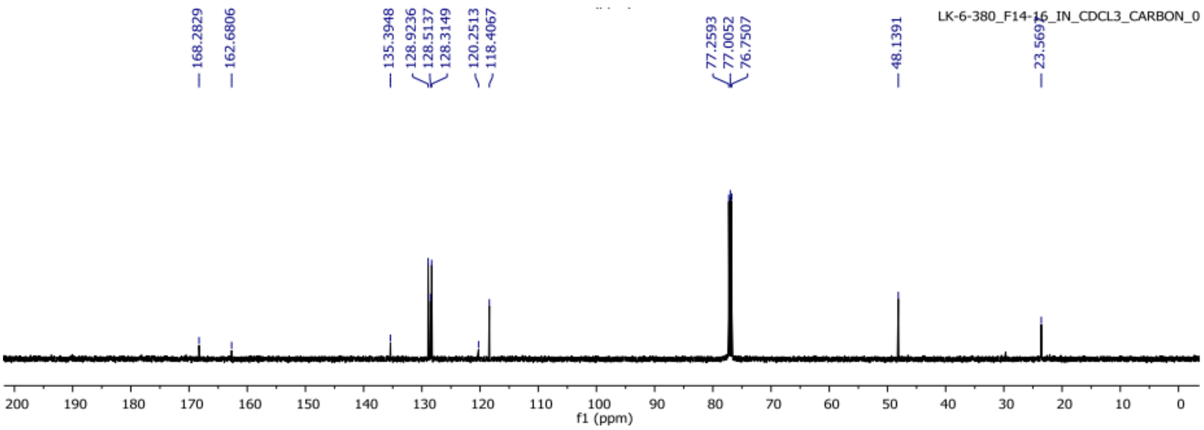

Figure M59: ^{13}C NMR spectrum of N-(2-benzyl-3-oxo-2,3-dihydro-1,2-thizol-4-yl)acetamide ***6*** *in $CDCl_3$*

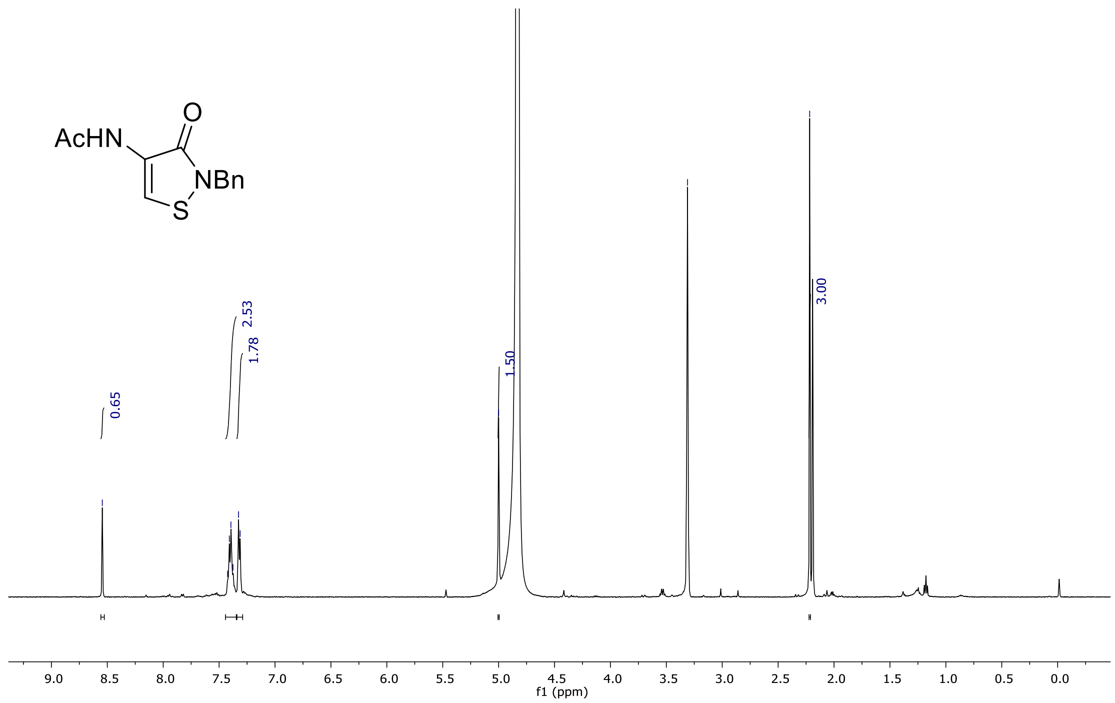

Figure M60: ^{1}H NMR spectrum of N-(2-benzyl-3-oxo-2,3-dihydro-1,2-thizol-4-yl)acetamide ***6*** *in methanol-d$_4$/D$_2$O*

Reduction of isothiazolone (6) to give enethiolate (5) with subsequent *S*-methylation

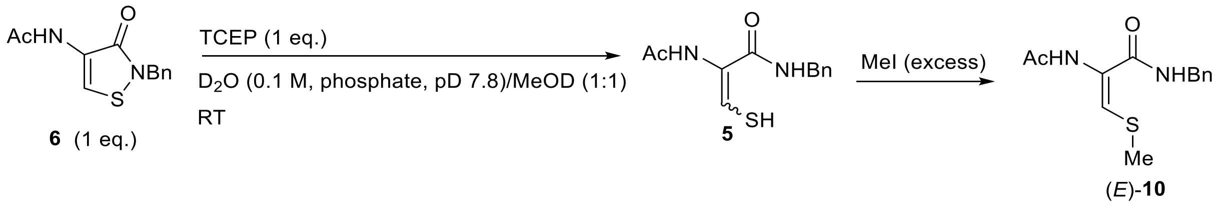

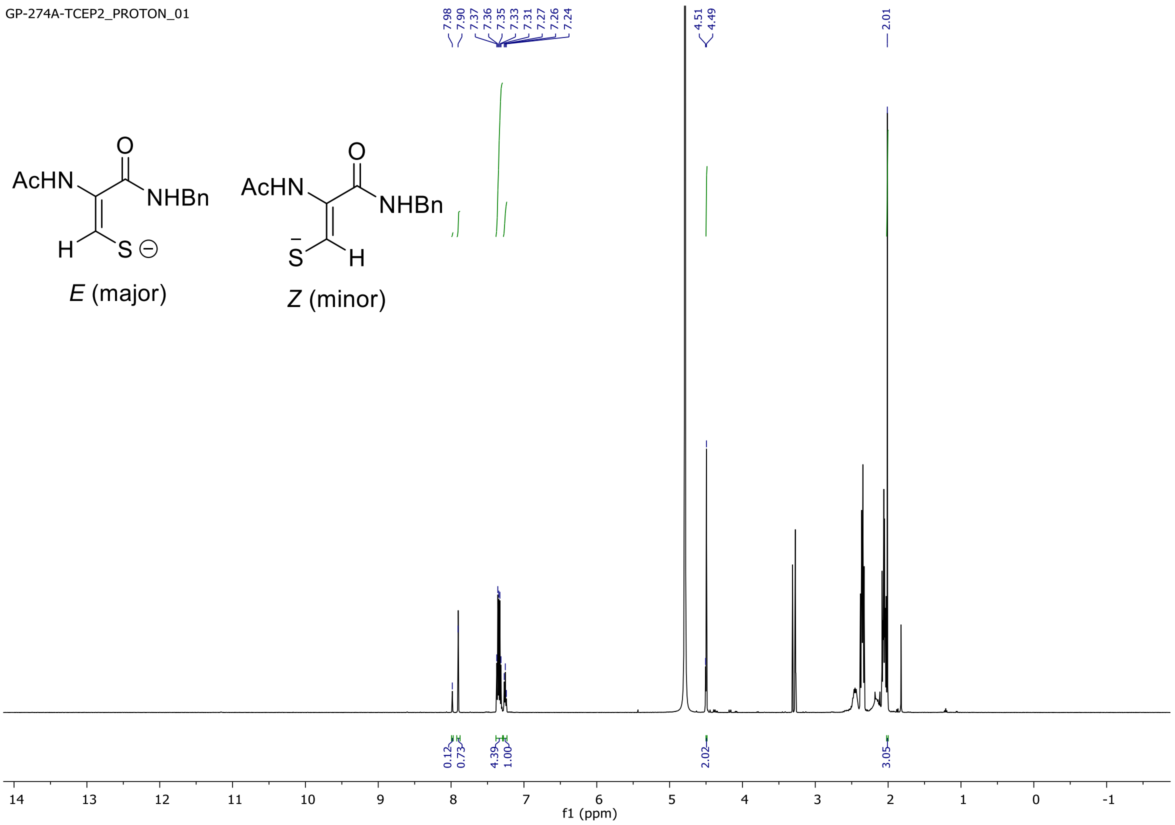

Figure M61: ^{1}H NMR spectrum of 2-acetamido-3-(benzylamino)-3-oxoprop-1-ene-1-thiolate ***5*** *in methanol-d$_4$/D$_2$O in the presence of TCEP oxide.*

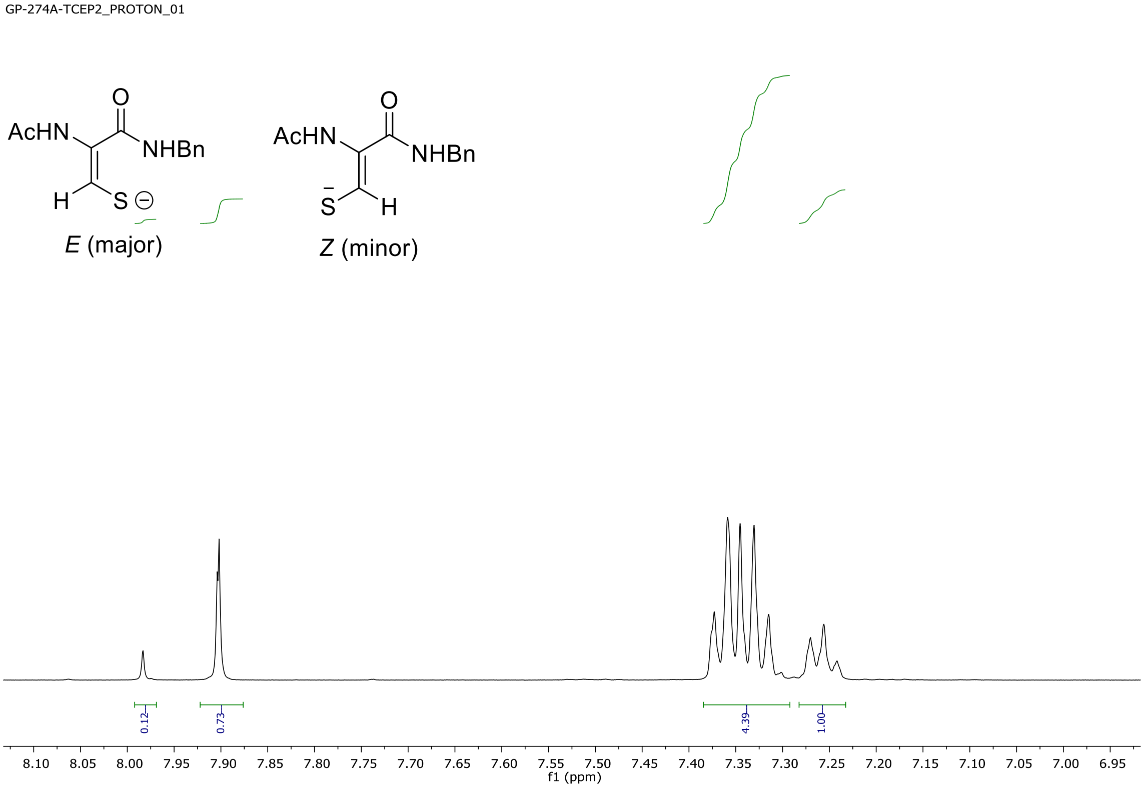

Figure M62: Zoomed in region of ^{1}H NMR spectrum of 2-acetamido-3-(benzylamino)-3-oxoprop-1-ene-1-thiolate **5** *in methanol-d$_4$/D$_2$O in the presence of TCEP oxide. The peak at 7.90 ppm corresponds to the methine proton of E (major) isomer of enethiolate while the peak at 7.99 ppm corresponds to the methine proton of Z (minor) isomer of enethiolate.*

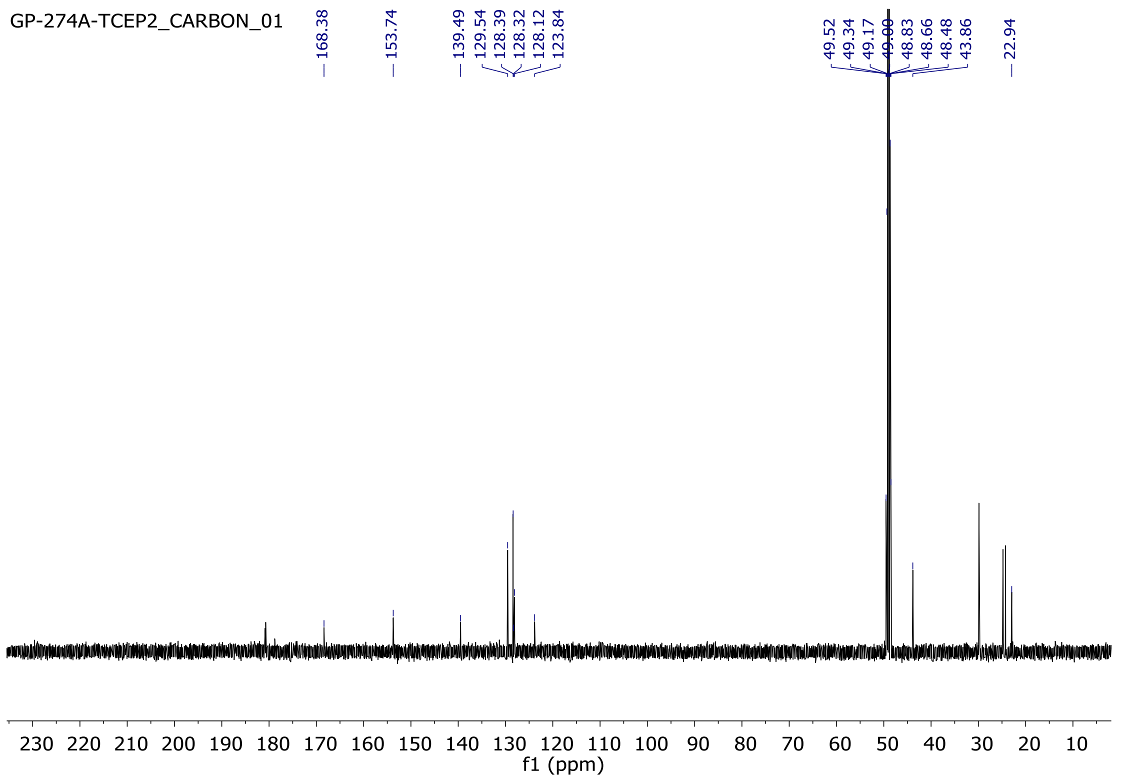

*Figure M63: ^{13}C NMR spectrum of 2-acetamido-3-(benzylamino)-3-oxoprop-1-ene-1-thiolate **5** in methanol-d_4/D_2O in the presence of TCEP oxide.*

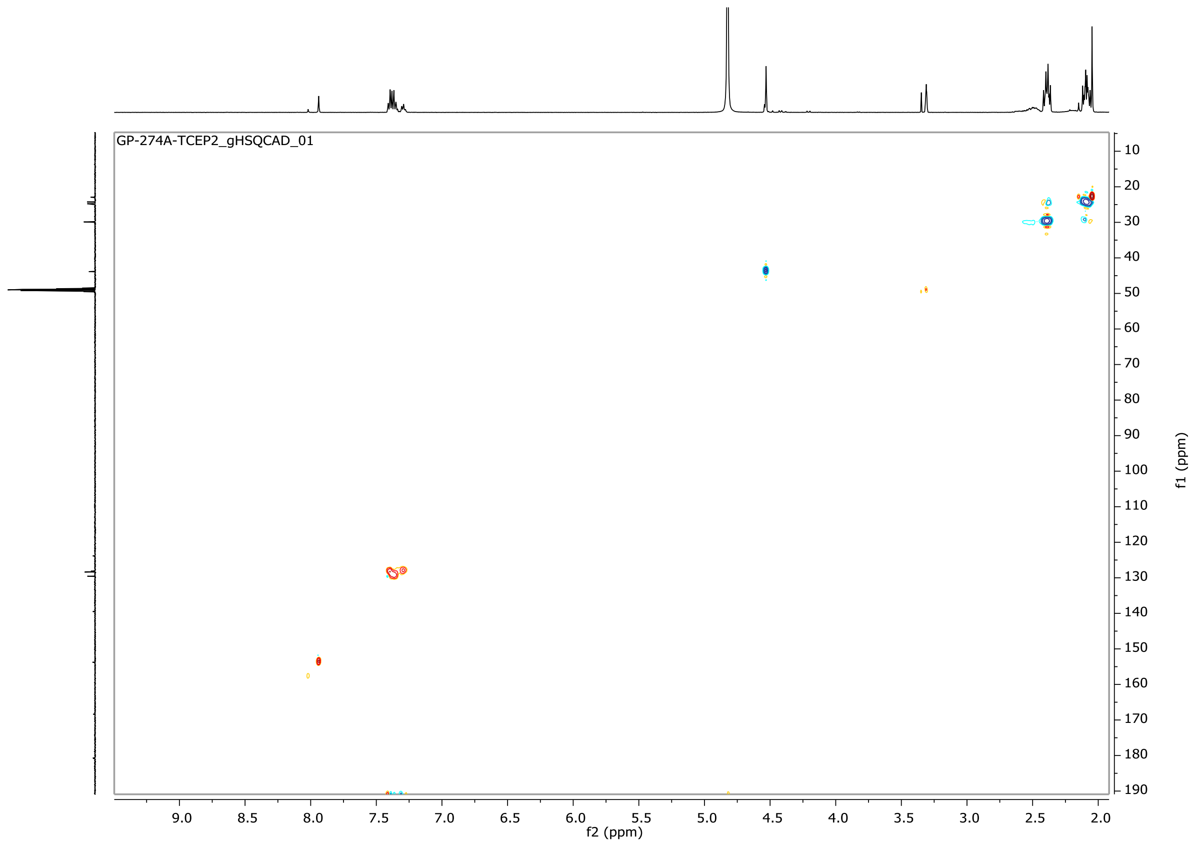

*Figure M64: HSQC NMR spectrum of 2-acetamido-3-(benzylamino)-3-oxoprop-1-ene-1-thiolate **5** in methanol-d_4/D_2O in the presence of TCEP oxide.*

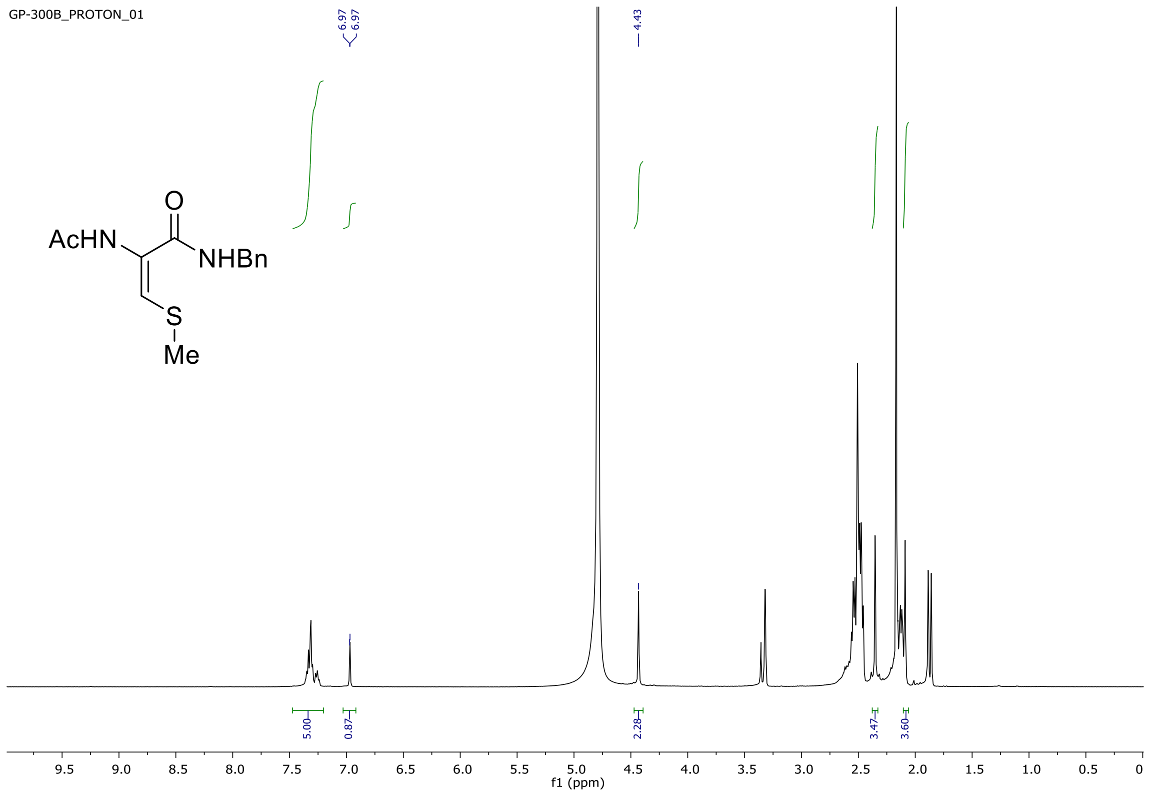

*Figure M65: ^{1}H NMR spectrum of methyl vinyl sulfide (E)-**10** in methanol-d$_4$/D$_2$O in the presence of TCEP oxide and excess methyl iodide.*

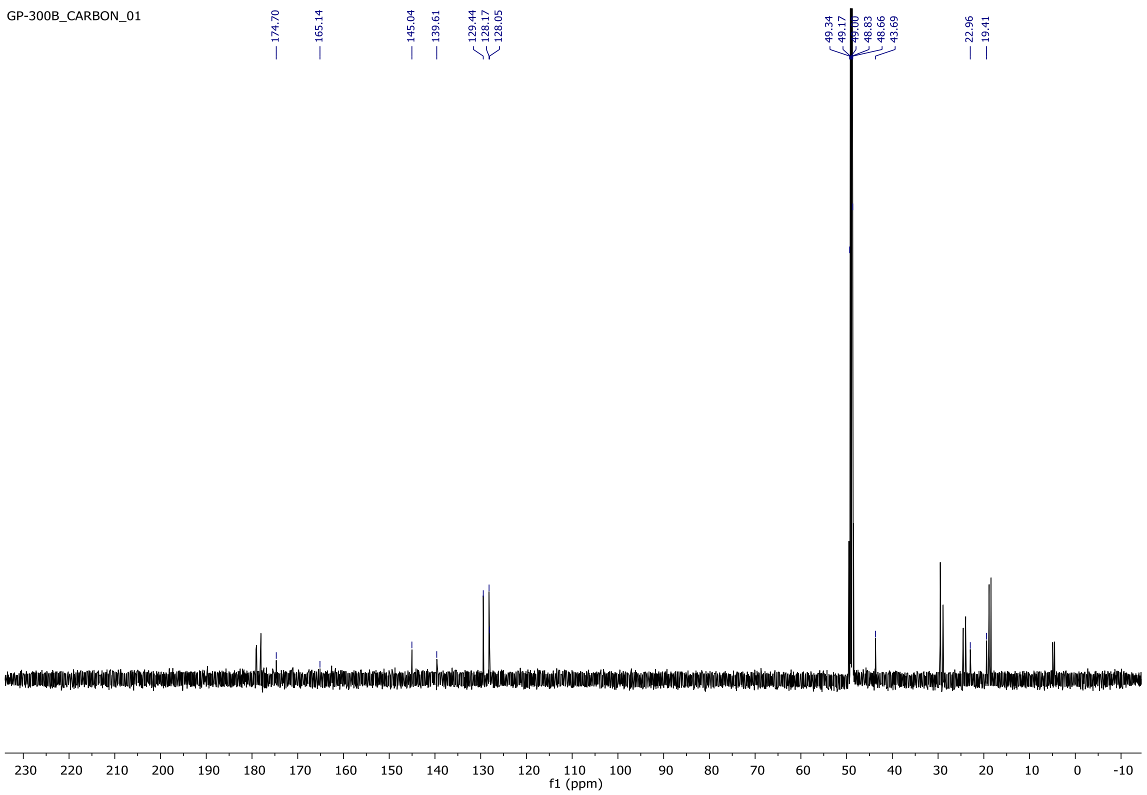

*Figure M66: ^{13}C NMR spectrum of methyl vinyl sulfide (E)-**10** in methanol-d$_4$/D$_2$O in the presence of TCEP oxide and excess methyl iodide.*

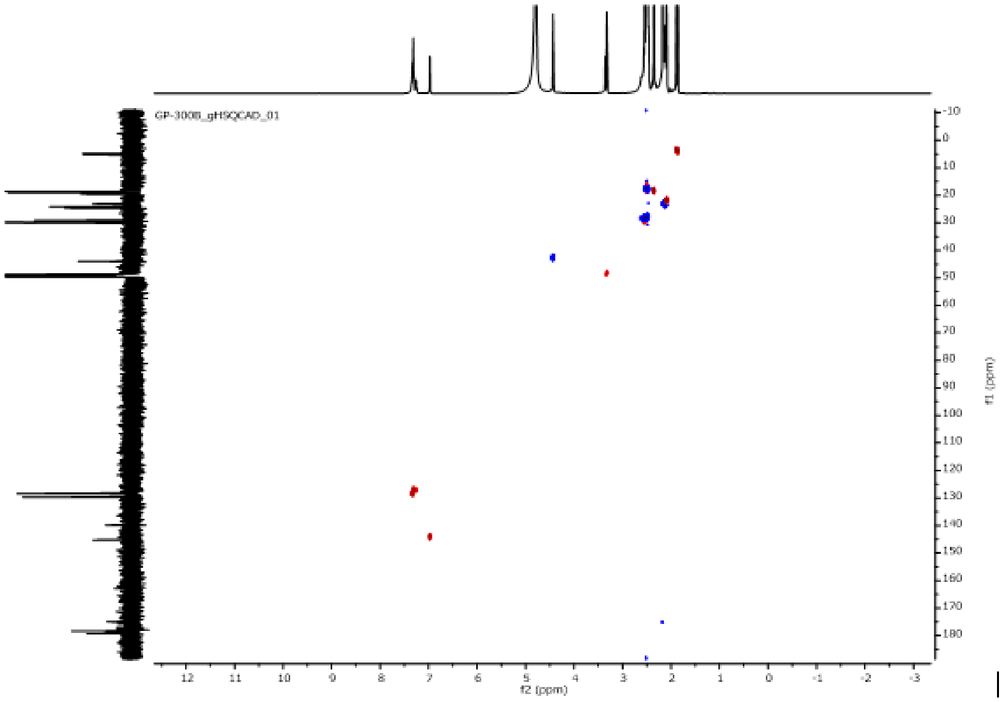

*Figure M67: HSQC NMR spectrum of methyl vinyl sulfide (E)-**10** in methanol-d$_4$/D$_2$O in the presence of TCEP oxide and excess methyl iodide.*

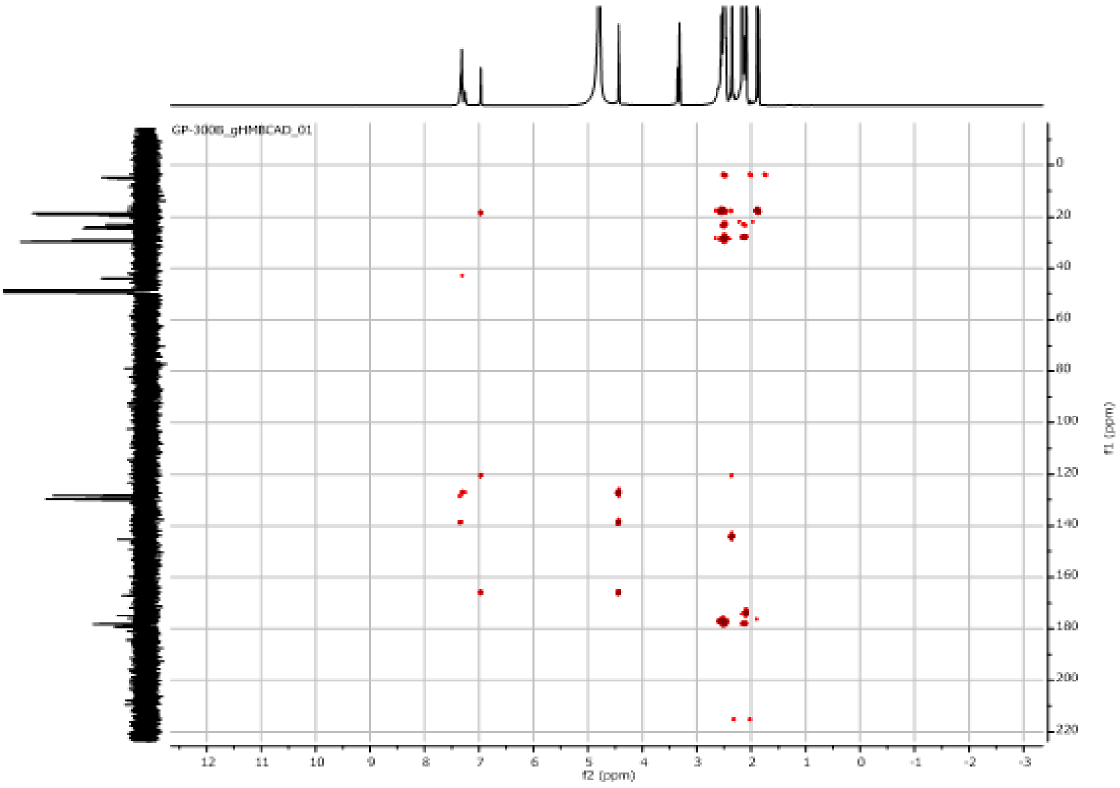

*Figure M68: HMBC NMR spectrum of methyl vinyl sulfide (E)-**10** in methanol-d$_4$/D$_2$O in the presence of TCEP oxide and excess methyl iodide.*

Oxidation of enethiolate (5) with DTNB

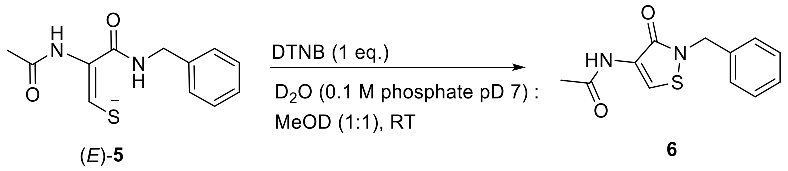

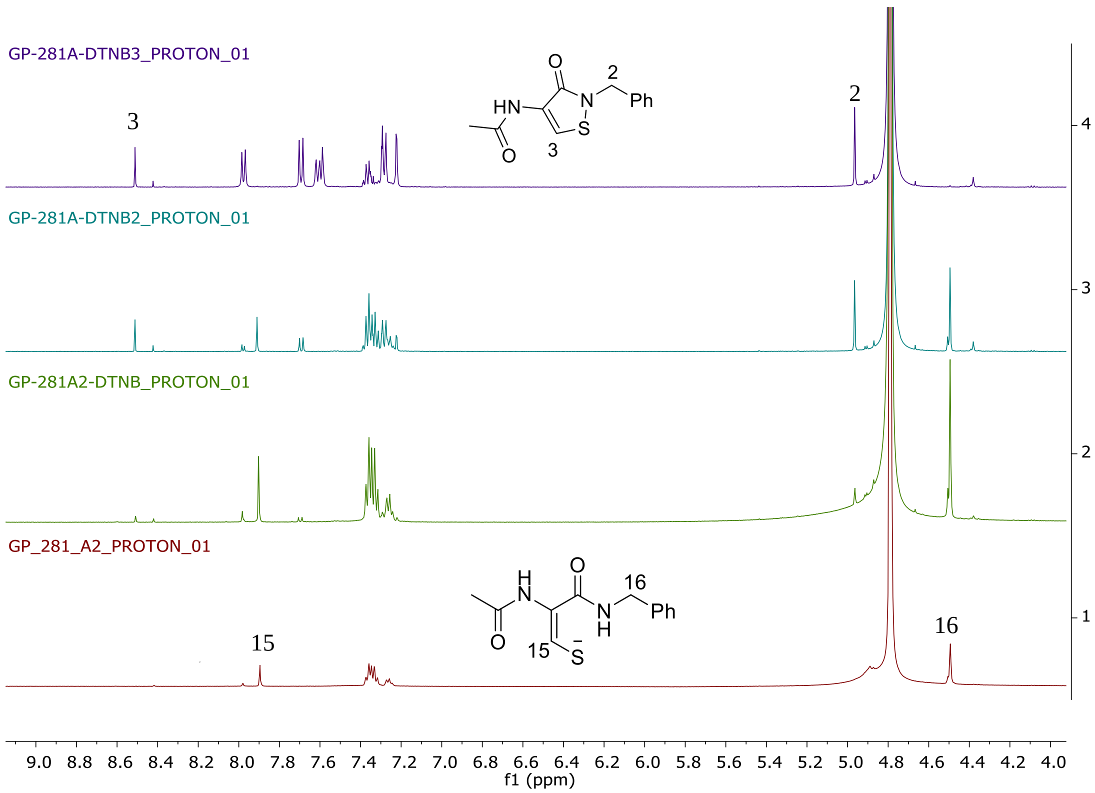

Figure M69: Stacked ^{1}*H NMR-spectrum from reaction mixture derived from reacting enethiolate (E)-**5** with portion wise addition of DTNB to obtain the isothiazolone **6***

Oxidation of enethiolate (5) with KI/I₂ to give isothiazolone (6) and isothiazolone S-oxide (9)

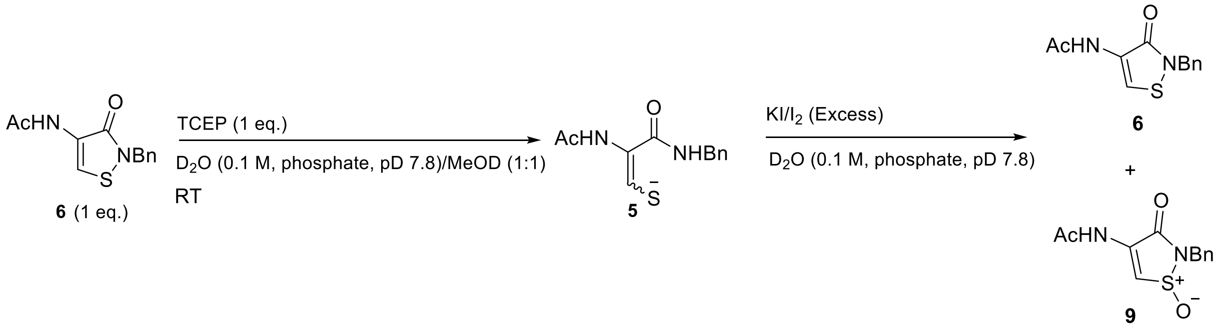

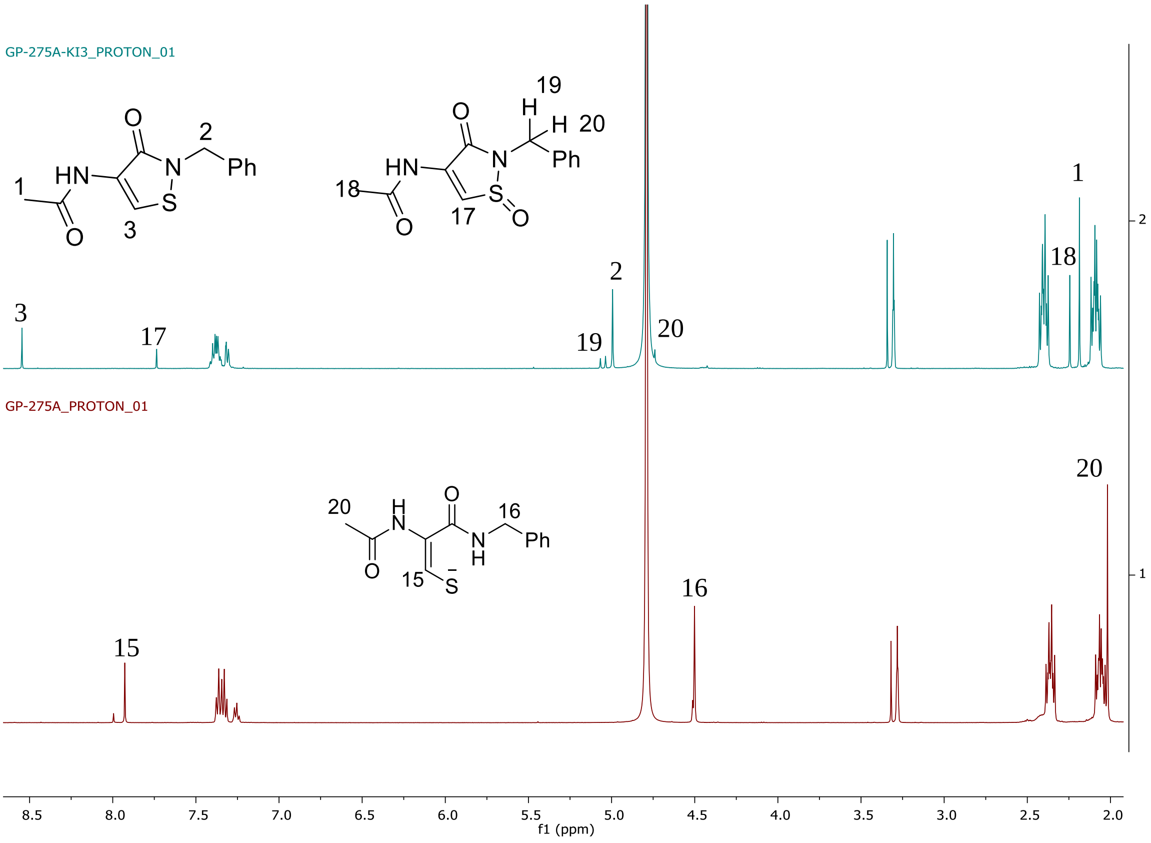

Figure M70:[1]*H NMR spectrum of reaction mixture derived from KI/I$_2$ oxidation of enethiolate* ***5*** *forming isothiazolone* ***6*** *and chiral isothiazolone S-oxide* ***9.***

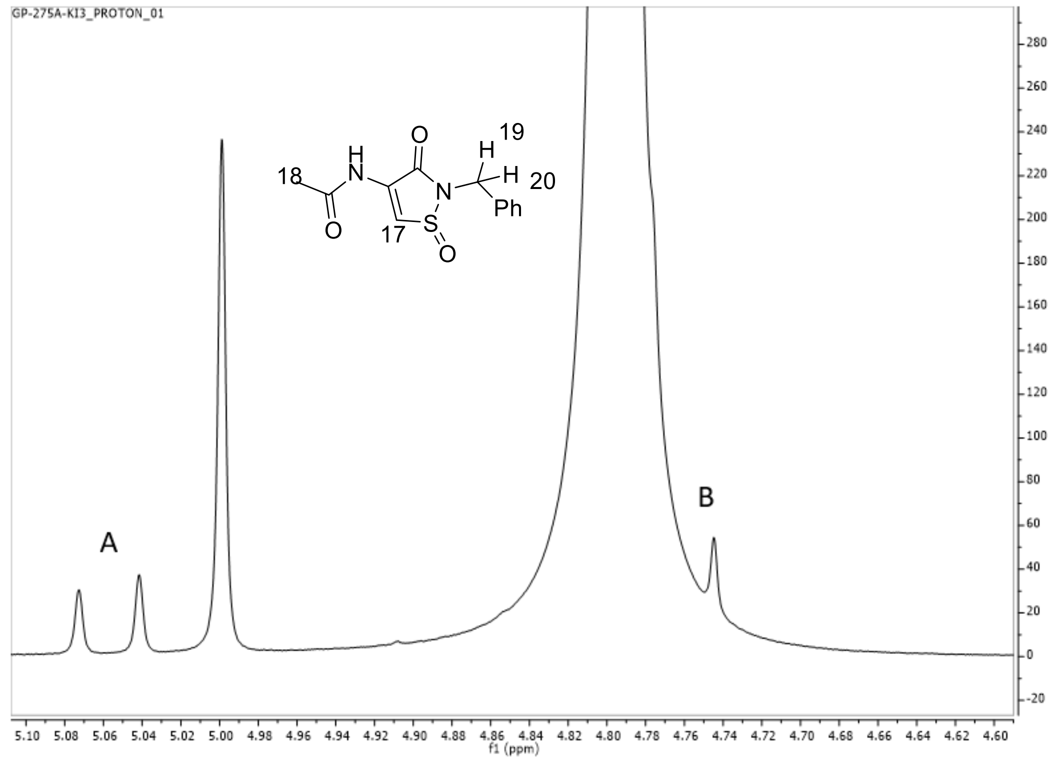

Figure M71: Zoomed in ^{1}H NMR spectrum of reaction mixture derived from KI/I_2 oxidation of enethiolate 5. The diastereotopic methylene protons 19 and 20 of chiral isothiazolone S-oxide 9 appear as two separate doublets, doublet A at 5.06 ppm and doublet B at 4.76 ppm with a coupling constant value of 15.5 Hz. Note: doublet B is partially hidden under the HDO signal.

Isolation and characterisation of *N*-benzyl-2-methylthiazole- 4-carboxamide (18)

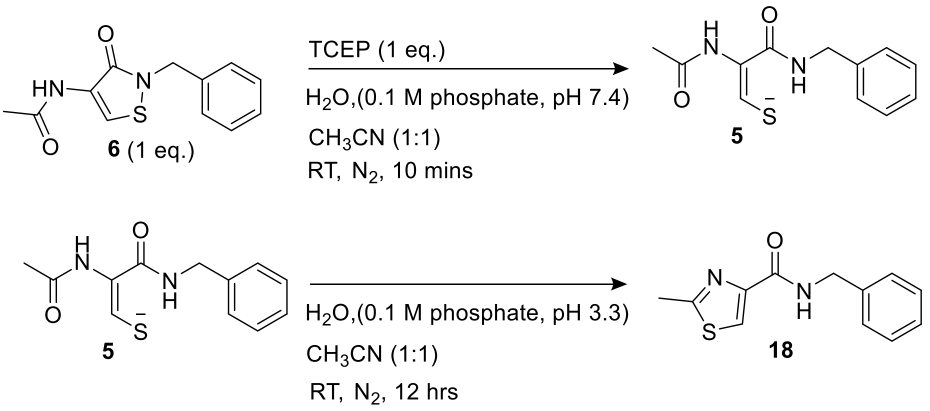

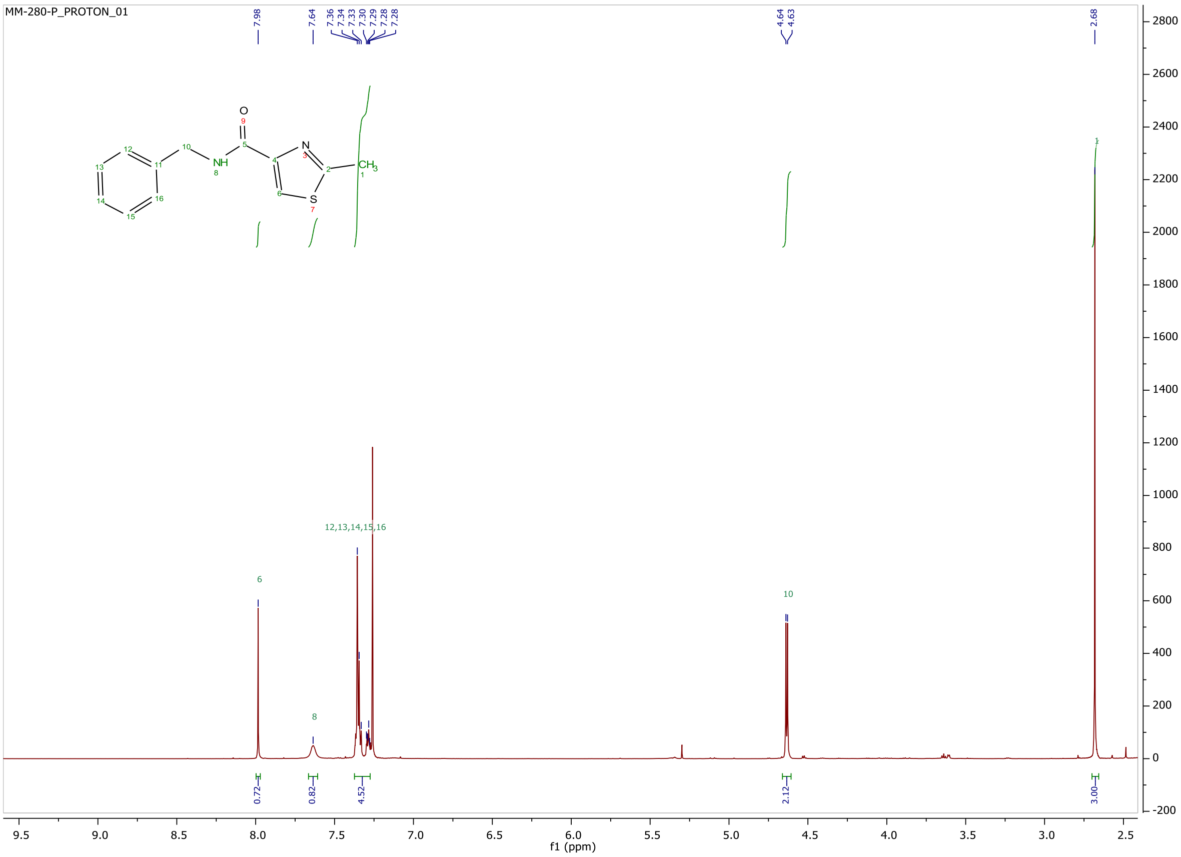

*Figure M72: ^{1}H NMR spectrum of the isolated N-benzyl-2-methylthiazole-4-carbxamide **18** in CDCl3*

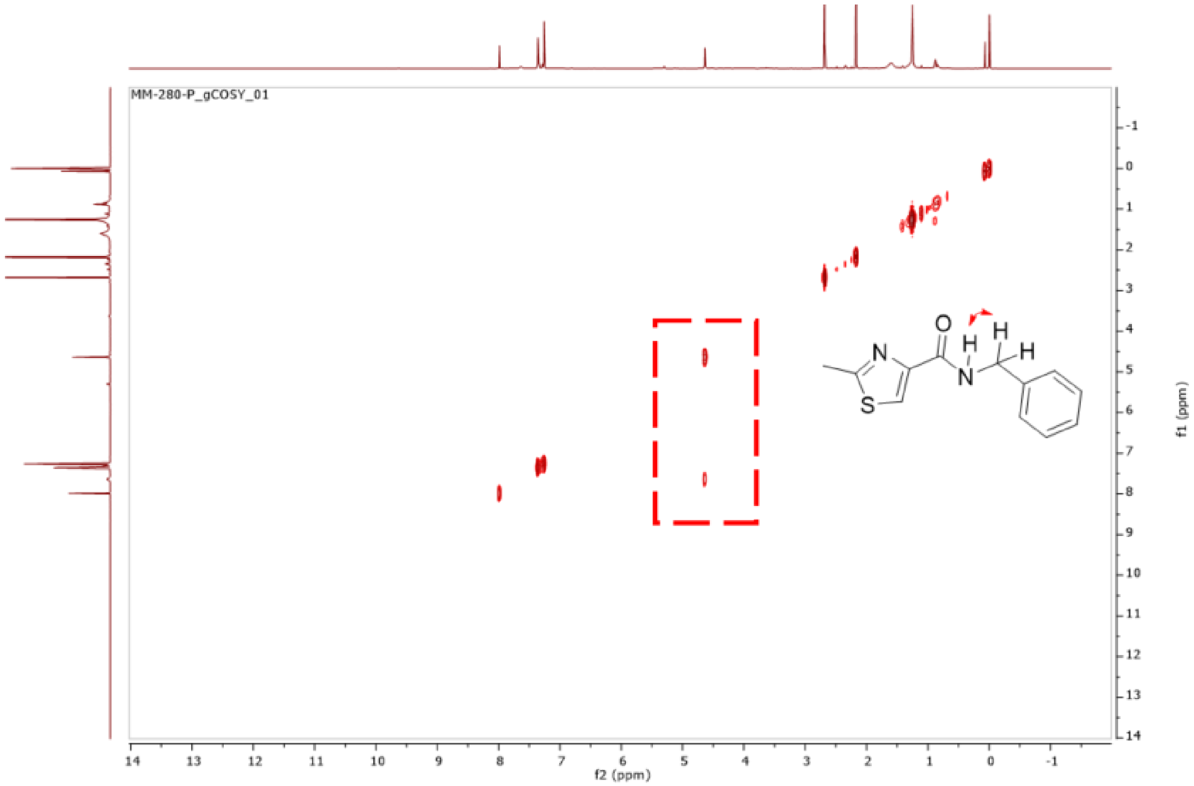

*Figure M73: COSY spectrum of the isolated N-benzyl-2-methylthiazole-4-carbxamide **18** highlighting the H-H correlation of the methylene protons at 4.63 ppm to the broad amine signal at 7.64 ppm.*

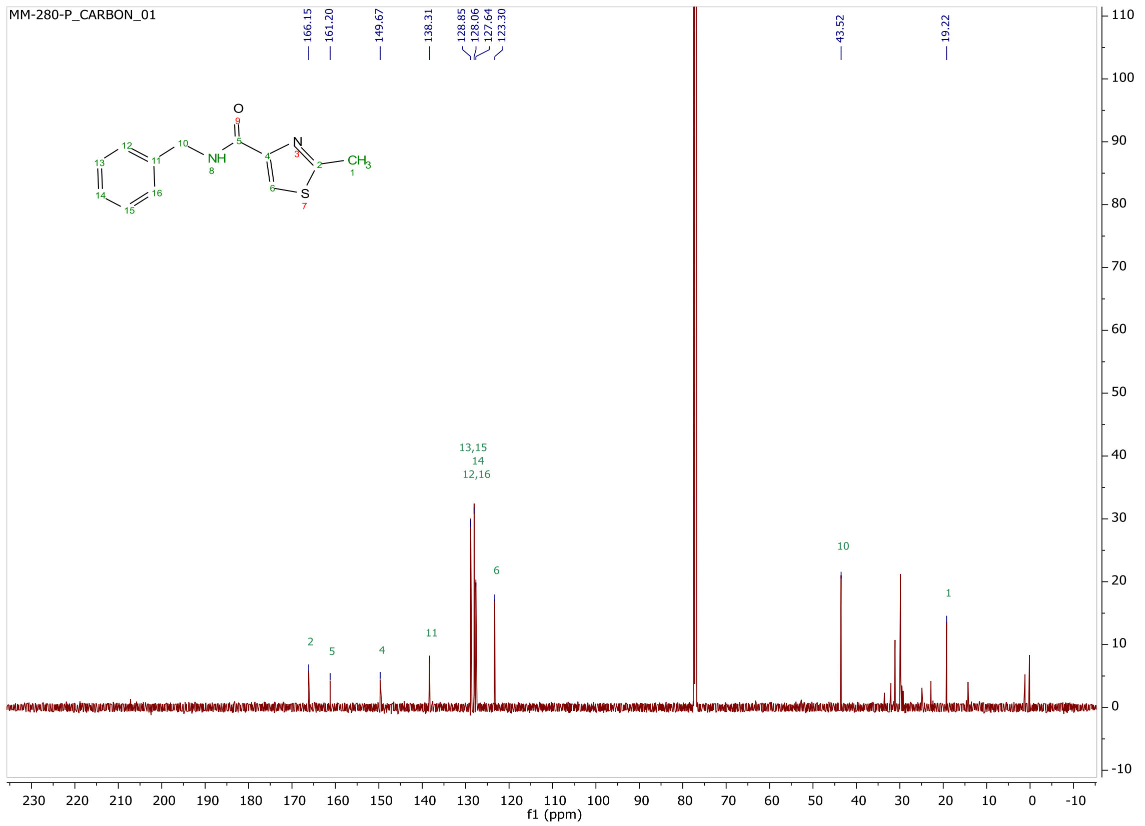

*Figure M74: ^{13}C NMR spectrum of the isolated N-benzyl-2-methylthiazole-4-carbxamide **18.** Additional peaks in the region of 30-10 ppm are accounted for by grease and acetone that are present in the system as impurities.*

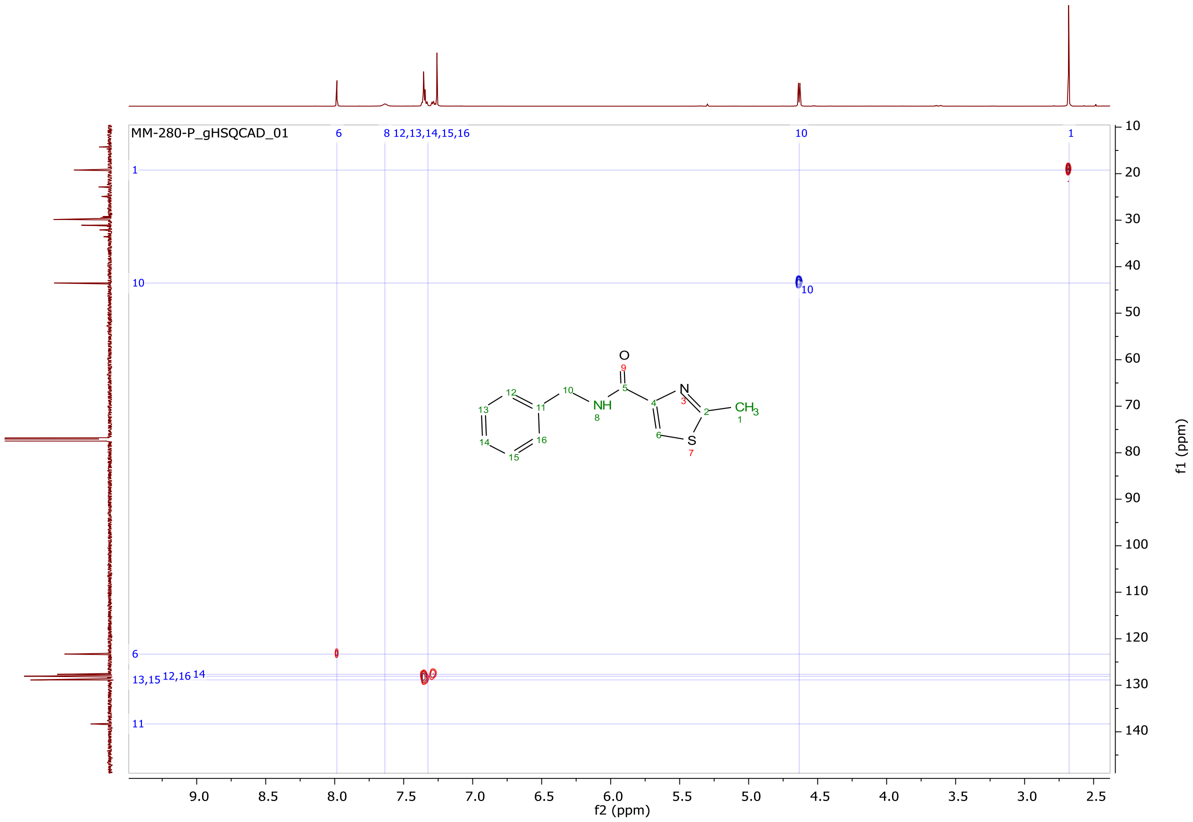

Figure M75: HSQC NMR spectrum of the isolated N-benzyl-2-methylthiazole-4-carbxamide ***18*** *showing the C-H correlations.*

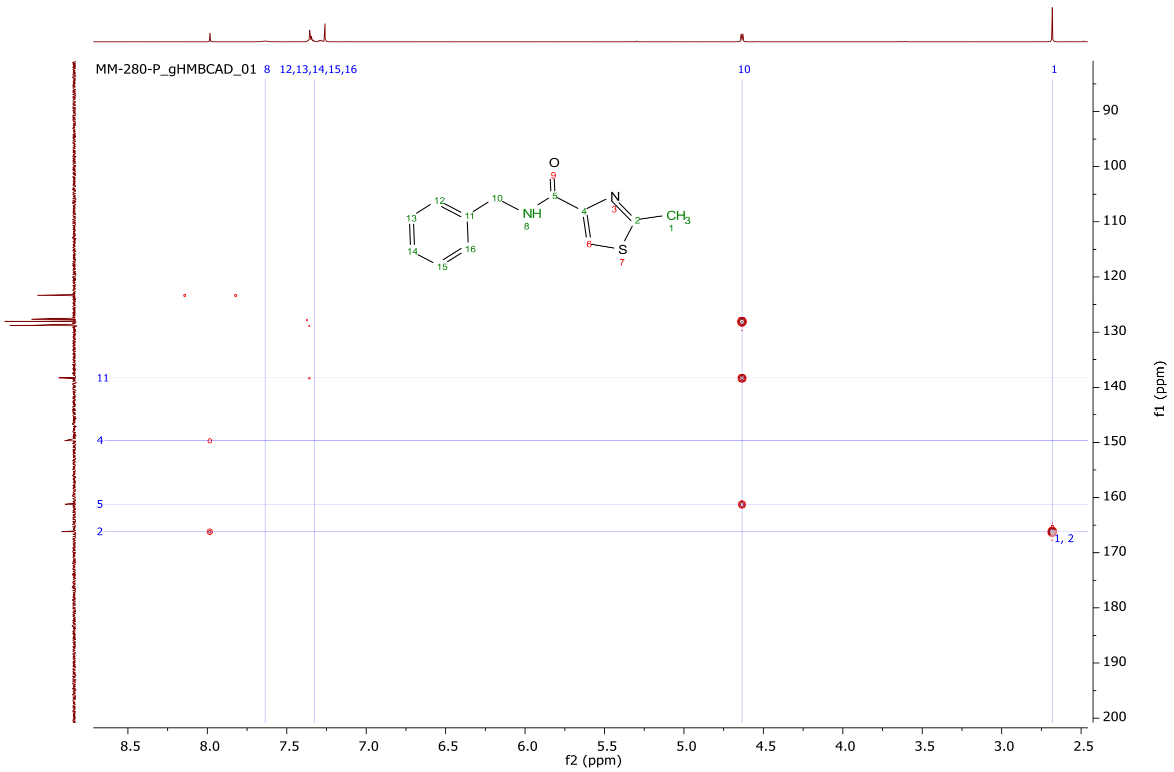

*Figure M76: HMBC NMR spectrum of the isolated N-benzyl-2-methylthiazole-4-carbxamide **18** showing quaternary carbon assignments.*

Synthesis and hydrolysis of a formylglycine diethylacetal derivative

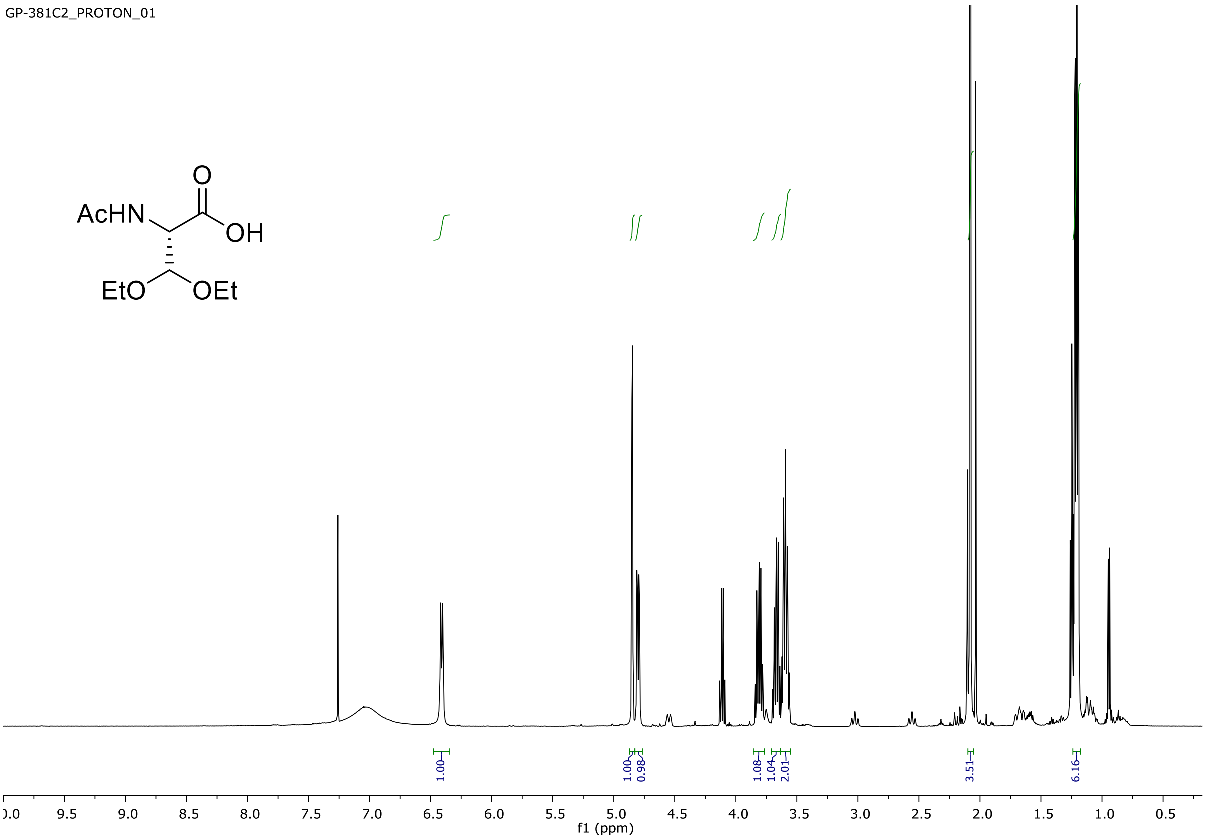

Figure M77: ^{1}H NMR spectrum of (S)-2-((((9H-fluoren-9-yl)methoxy)carbonyl)amino)-3,3-diethoxypropanoic acid ***12*** *in* $CDCl_3$

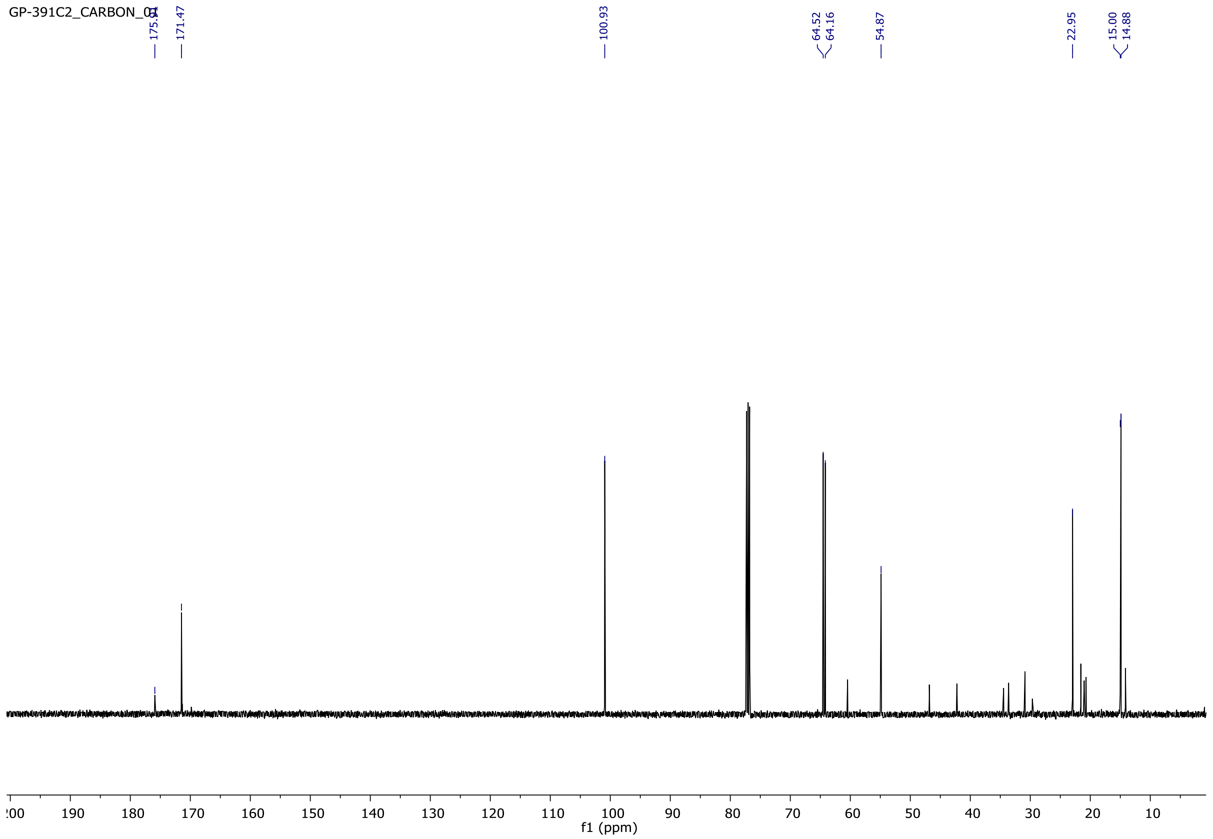

Figure M78: ^{13}C NMR spectrum of (S)-2-((((9H-fluoren-9-yl)methoxy)carbonyl)amino)-3,3-diethoxypropanoic acid ***12*** *in $CDCl_3$*

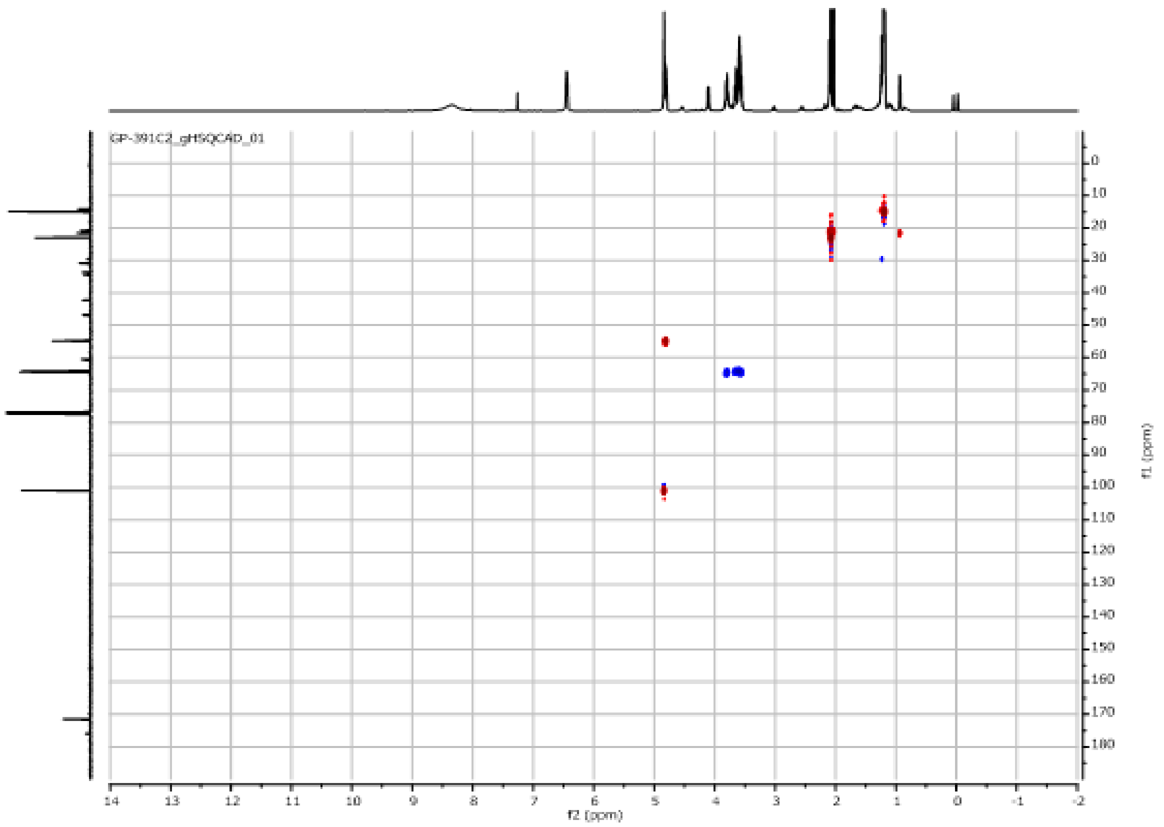

*Figure M79: HSQC NMR spectrum of (S)-2-((((9H-fluoren-9-yl)methoxy)carbonyl)amino)-3,3-diethoxypropanoic acid **12** in $CDCl_3$*

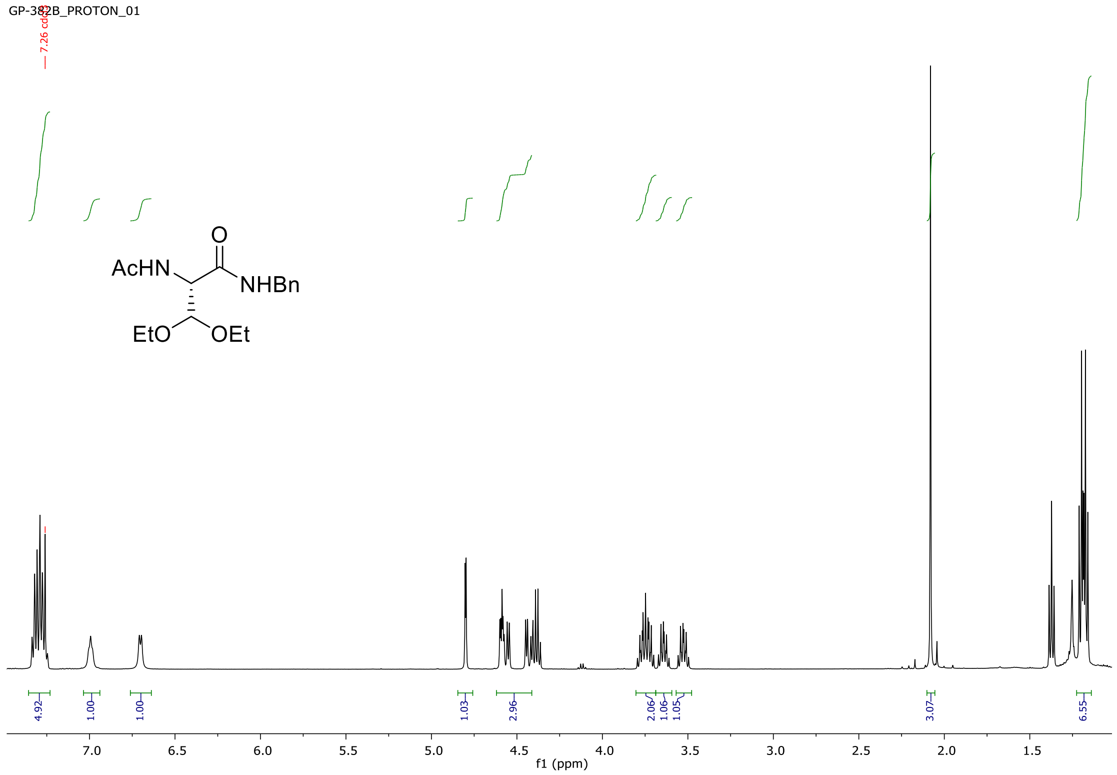

Figure M80: ^{1}H NMR spectrum of (S)-2-acetamido-N-benzyl-3,3-diethoxypropanamide ***13*** *in $CDCl_3$*

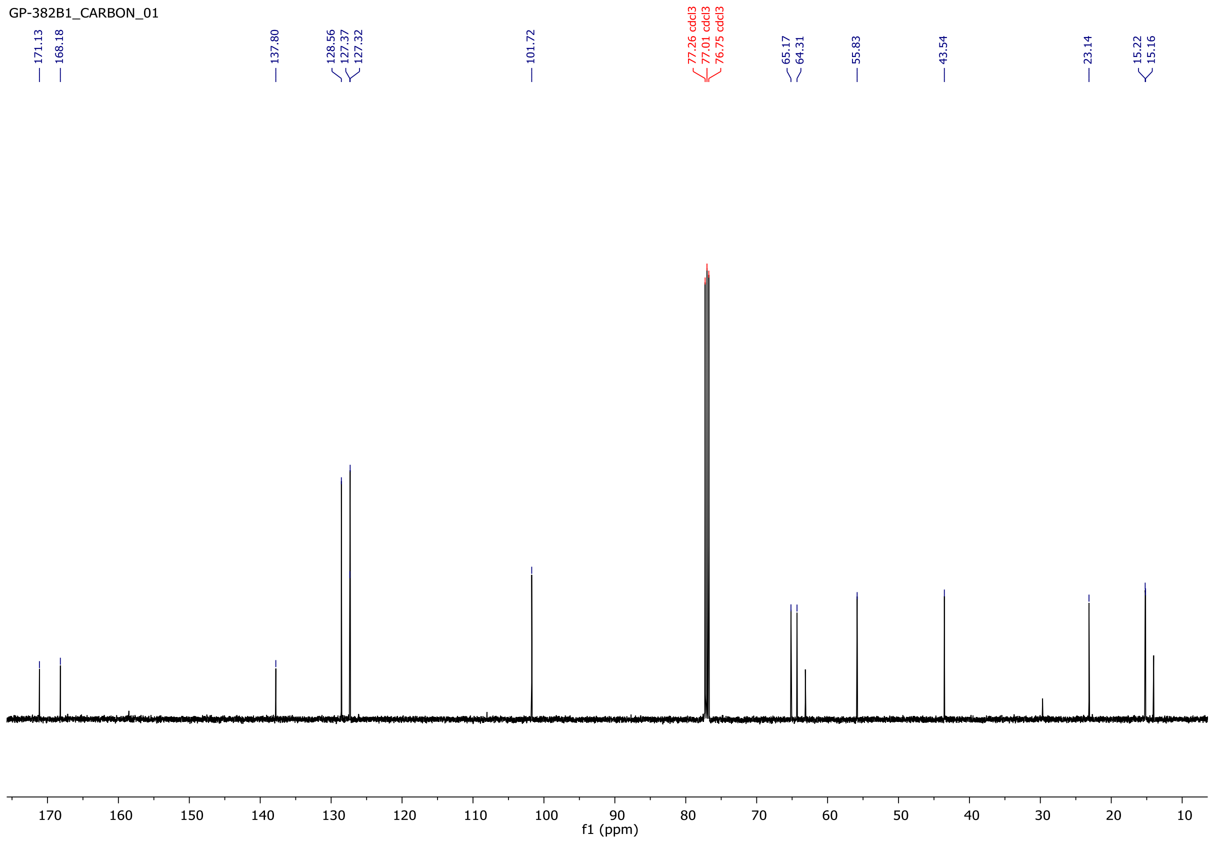

Figure M81: ^{13}C NMR spectrum of (S)-2-acetamido-N-benzyl-3,3-diethoxypropanamide ***13*** *in $CDCl_3$*

2-acetamido-N-benzyl-3,3-dihydroxypropanamide (15)

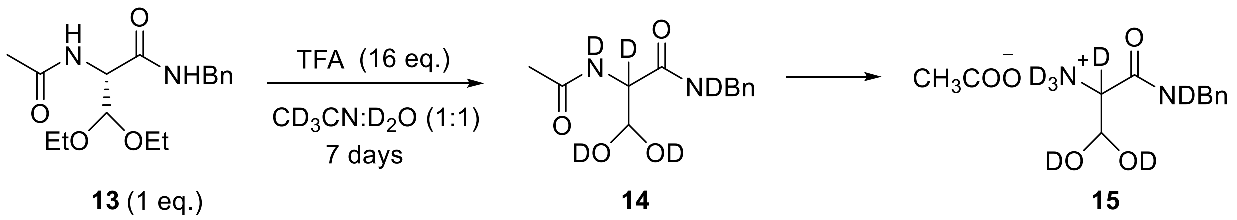

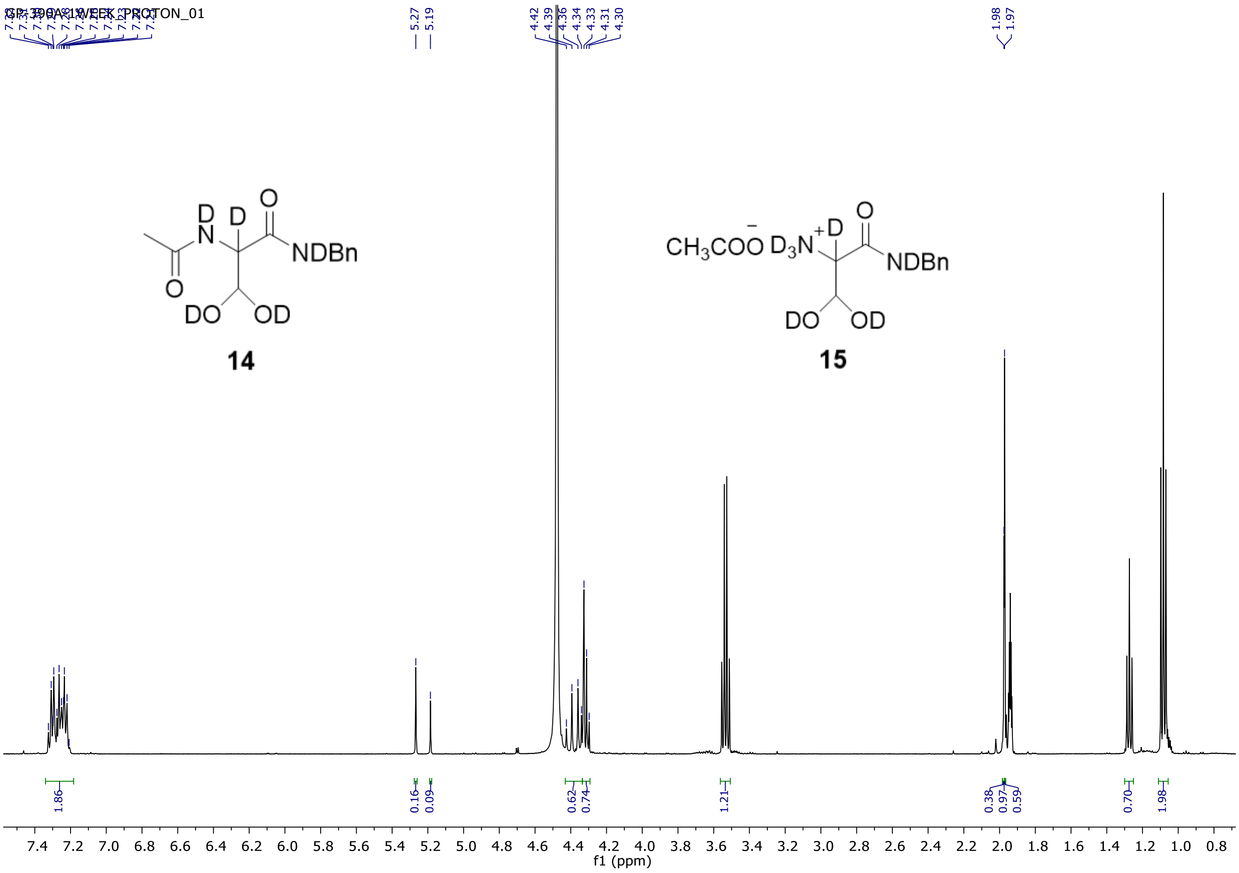

*Figure M82: ^{1}H NMR spectrum of 2-acetamido-N-benzyl-3,3-dihydroxypropanamide **14** and 1-(benzylamino)-3,3-dihydroxy-1-oxopropan-2-aminium acetate **15** in CD_3CN:D_2O (1:1).*

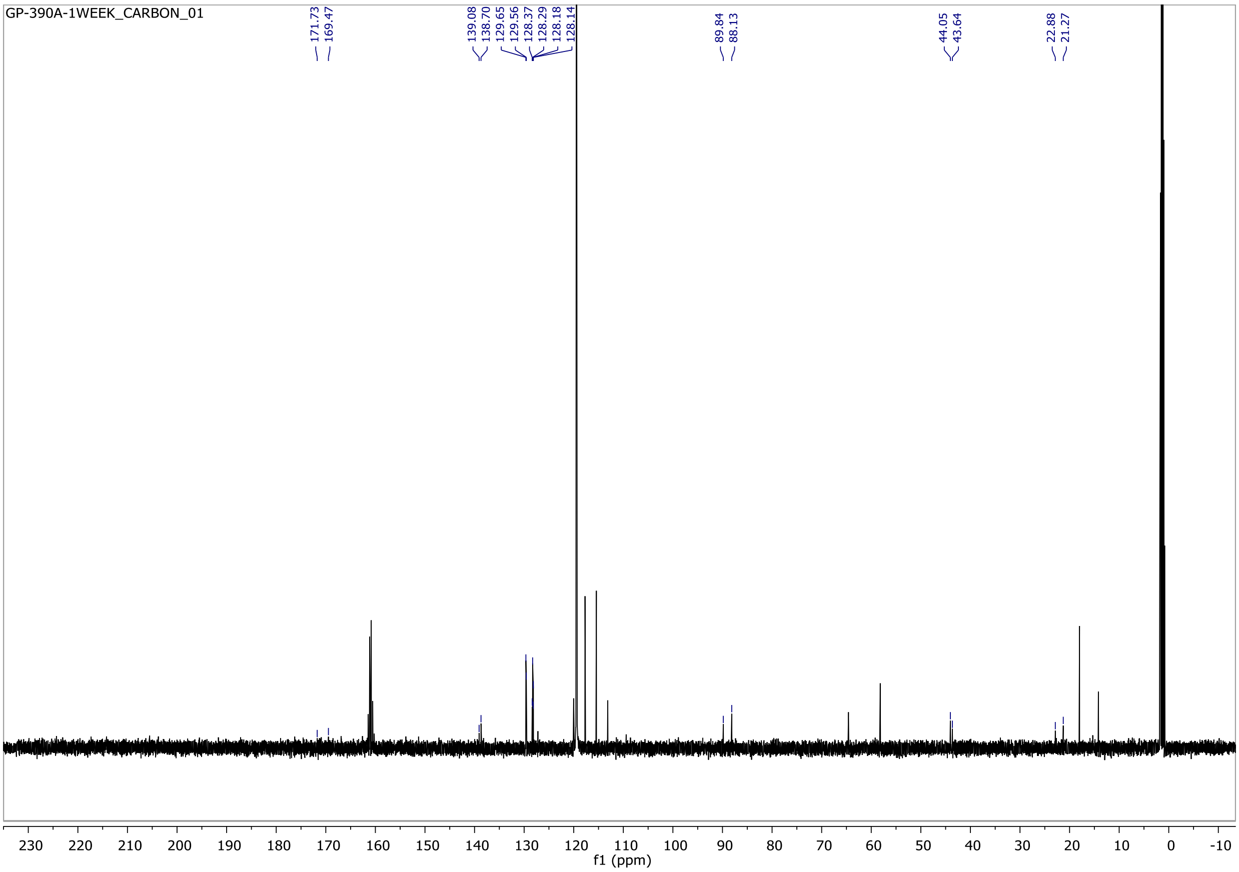

Figure M83: ^{13}C NMR spectrum of 2-acetamido-N-benzyl-3,3-dihydroxypropanamide ***14*** *and 1-(benzylamino)-3,3-dihydroxy-1-oxopropan-2-aminium acetate* ***15*** *in $CD_3CN:D_2O$ (1:1).*

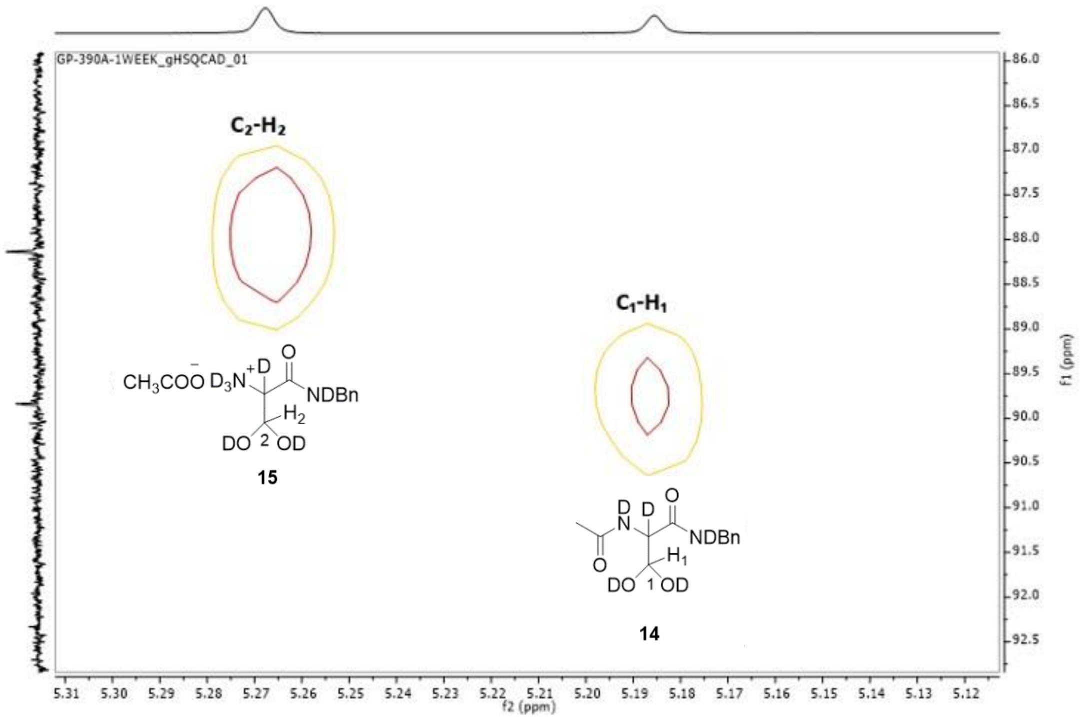

Figure M84: HSQC NMR spectrum of 2-acetamido-N-benzyl-3,3-dihydroxypropanamide 14 and 1-(benzylamino)-3,3-dihydroxy-1-oxopropan-2-aminium acetate 15 in CD_3CN:D_2O (1:1). The HSQC spectrum shows correlation of a proton at 5.18 ppm to a carbon at 89 ppm (C_1-H_1) and a proton at 5.27 ppm to a carbon at 88 ppm (C_2-H_2) indicative of the β C-H of 2-acetamido-N-benzyl-3,3-dihydroxypropanamide 14 and 1-(benzylamino)-3,3-dihydroxy-1-oxopropan-2-aminium acetate 15 respectively.

N^2,N^5-dibenzylpyrazine-2,5-dicarboxamide (16)

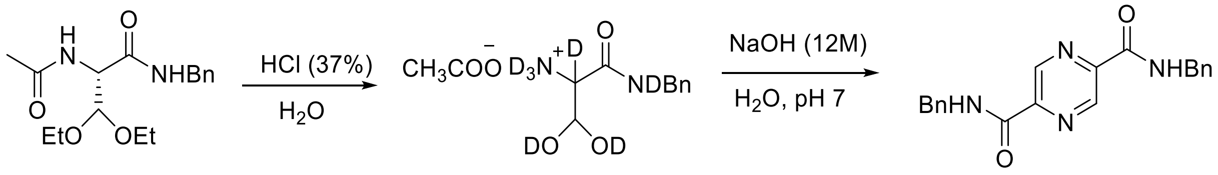

13

15

16

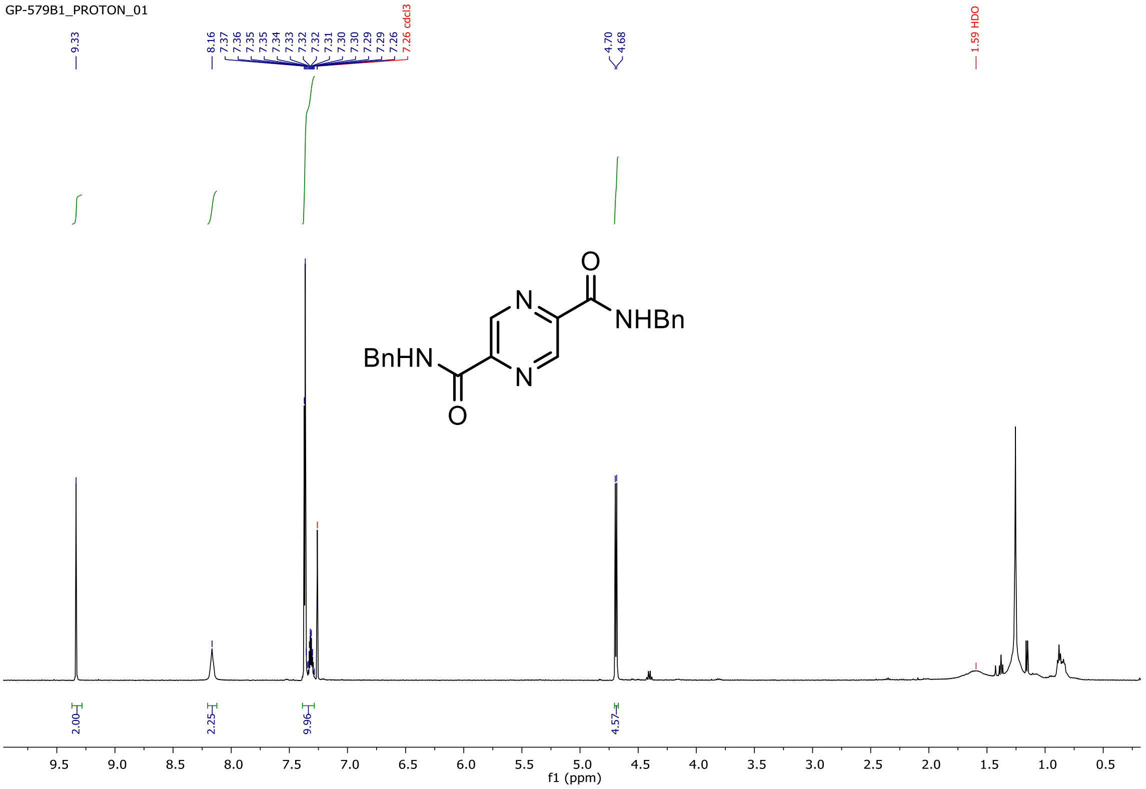

*Figure M85: ^{1}H NMR spectrum of N^2,N^5-dibenzylpyrazine-2,5-dicarboxamide **16** in $CDCl_3$*

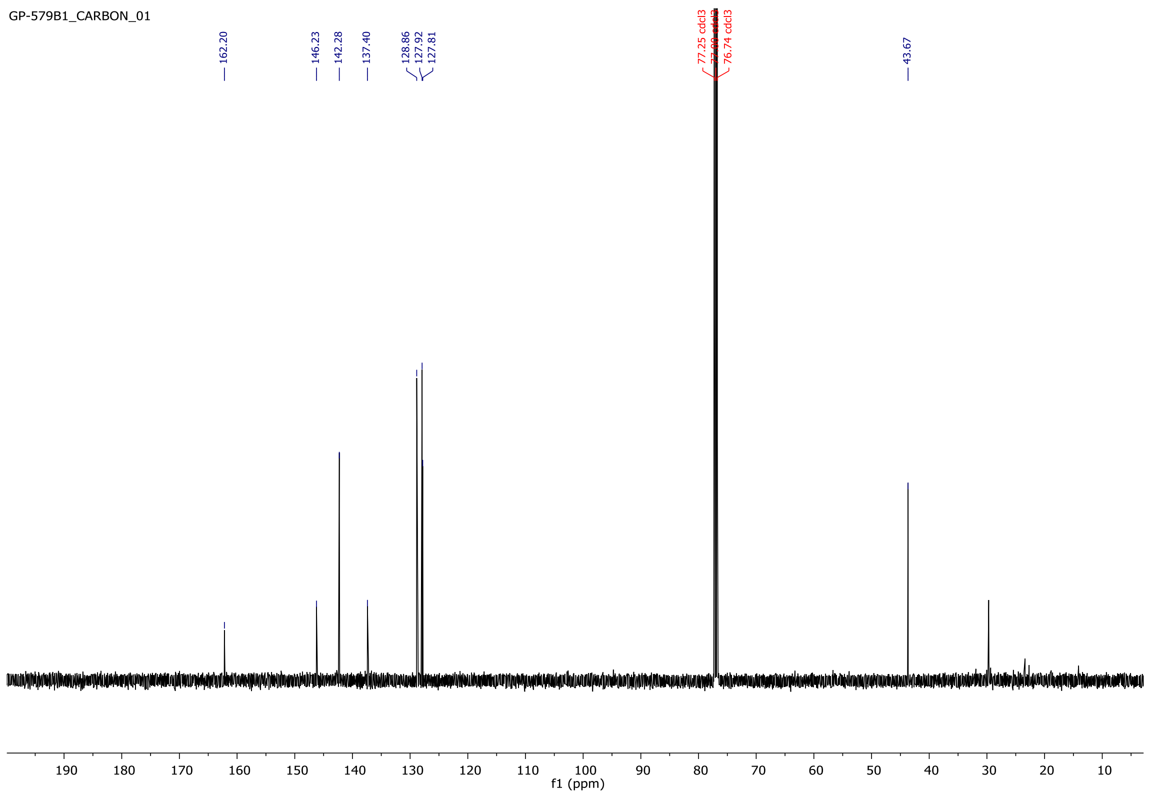

Figure M86: ^{13}C NMR spectrum of N^2,N^5-dibenzylpyrazine-2,5-dicarboxamide ***16*** *in $CDCl_3$*

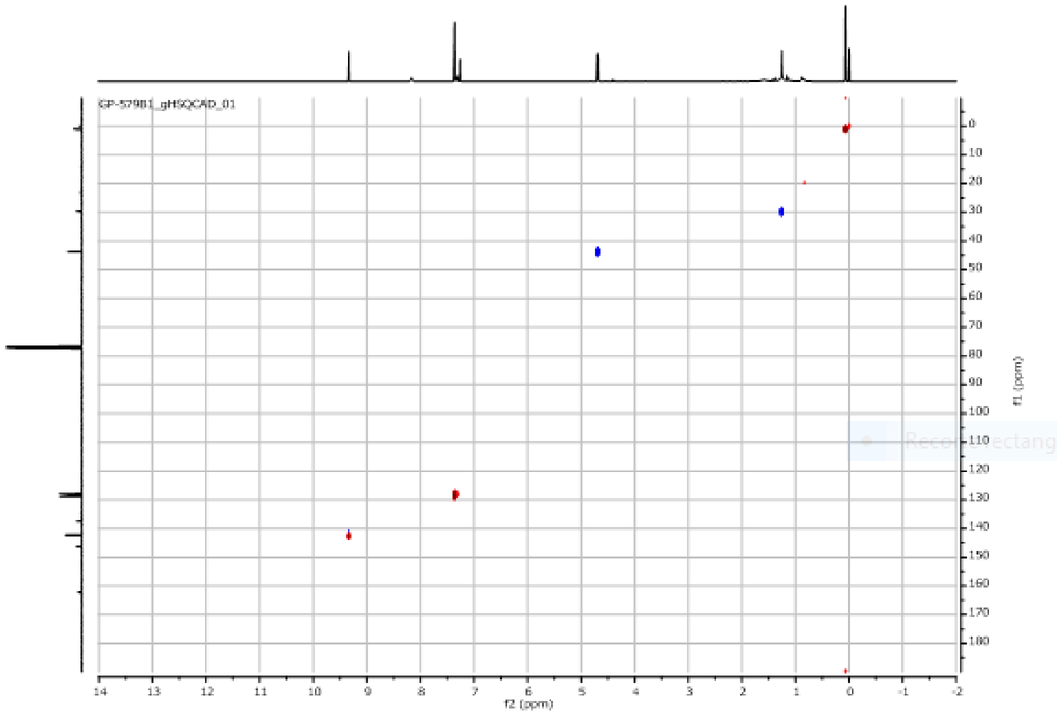

Figure M87: HSQC spectrum of N^2,N^5-dibenzylpyrazine-2,5-dicarboxamide ***16*** *in $CDCl_3$*

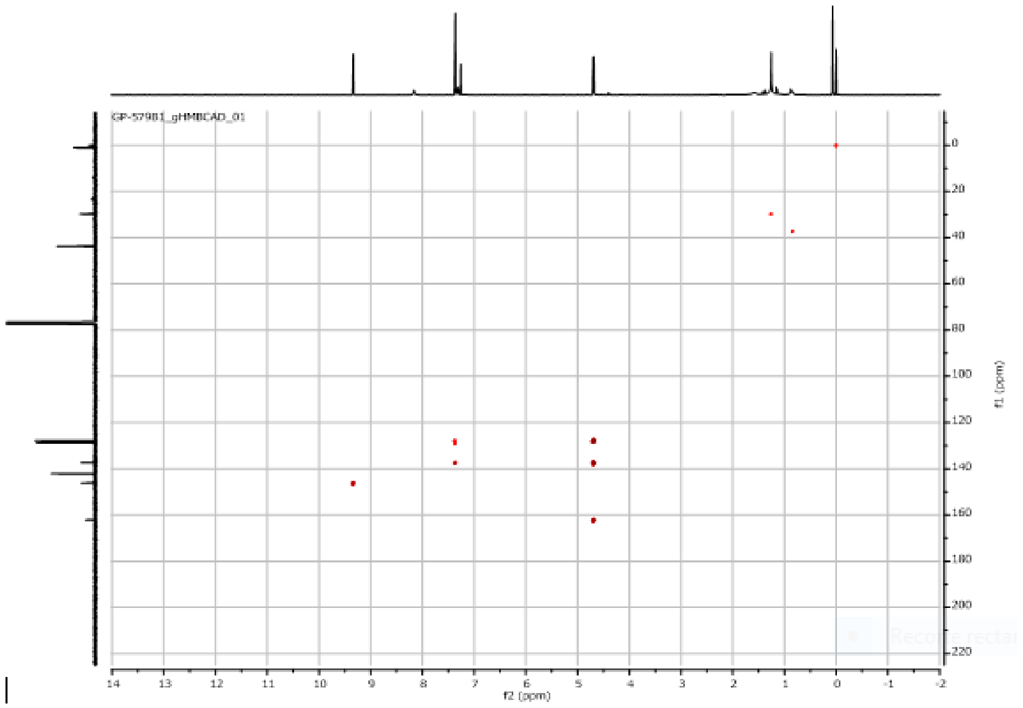

Figure M88: HMBC spectrum of N[2]*,N*[5]*-dibenzylpyrazine-2,5-dicarboxamide* ***16*** *in* $CDCl_3$

1,4-bis(2,4-dimethoxyphenyl)butane-1,4-dione (8a)

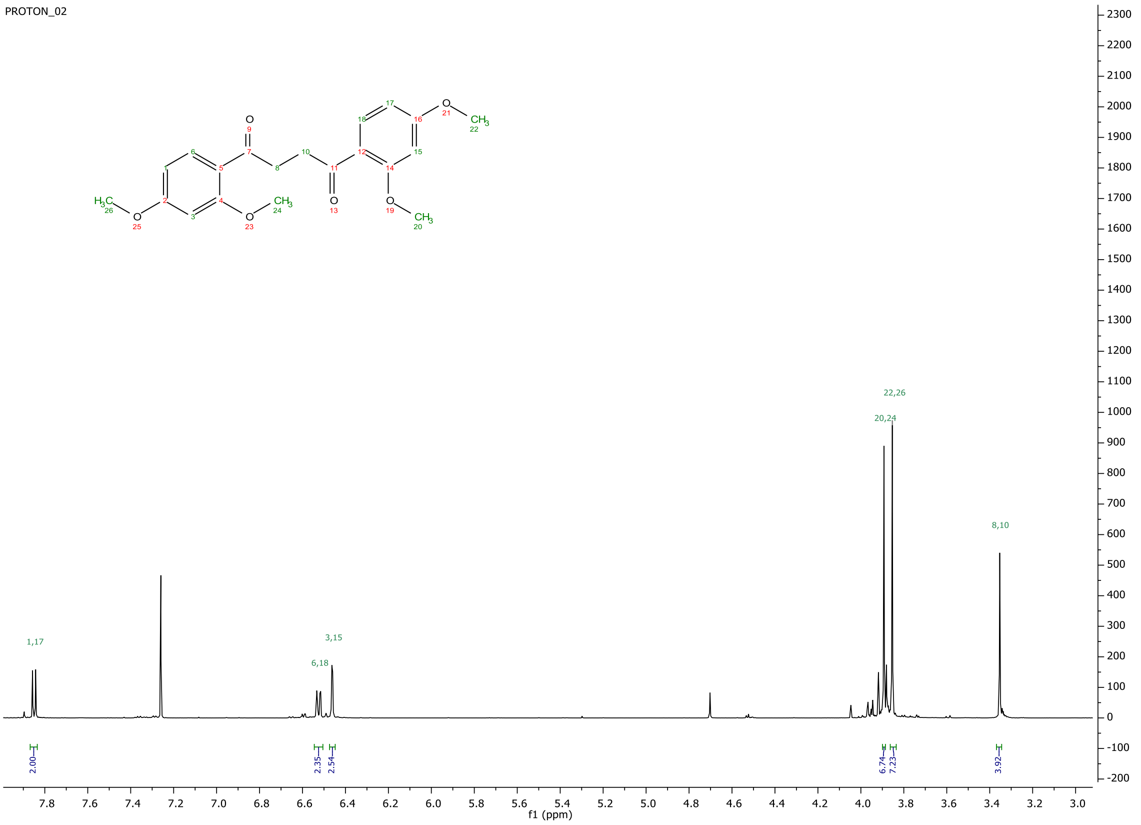

*Figure M89: ^{1}H NMR (500 MHz) spectrum of 1,4-diketone **8a** in $CDCl_3$*

Synthesis of (*E*)-2-acetamido-N-benzyl-3-(benzylthio)acrylamide

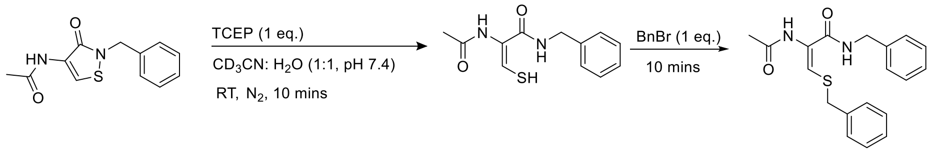

benzyl-vinyl sulfide

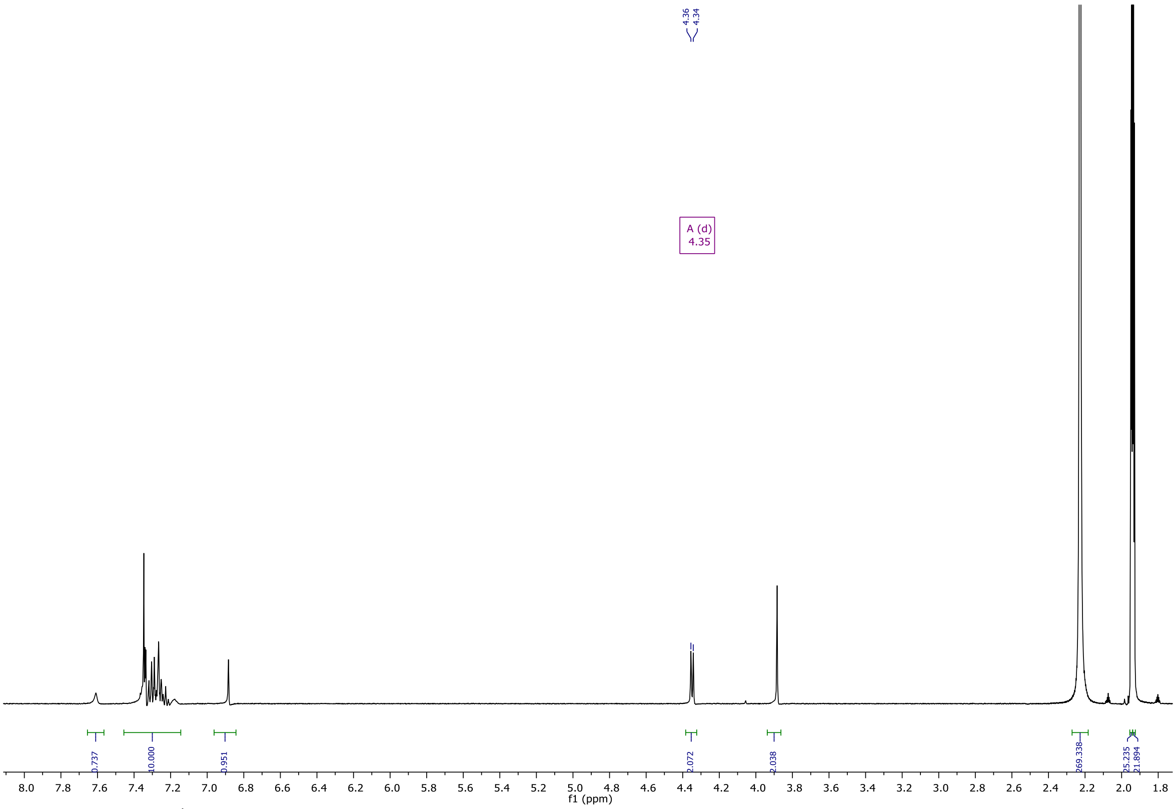

Figure M90: ^{1}H NMR (500 MHz) spectrum of benzyl-vinyl sulfide in CD_3CN following purification.

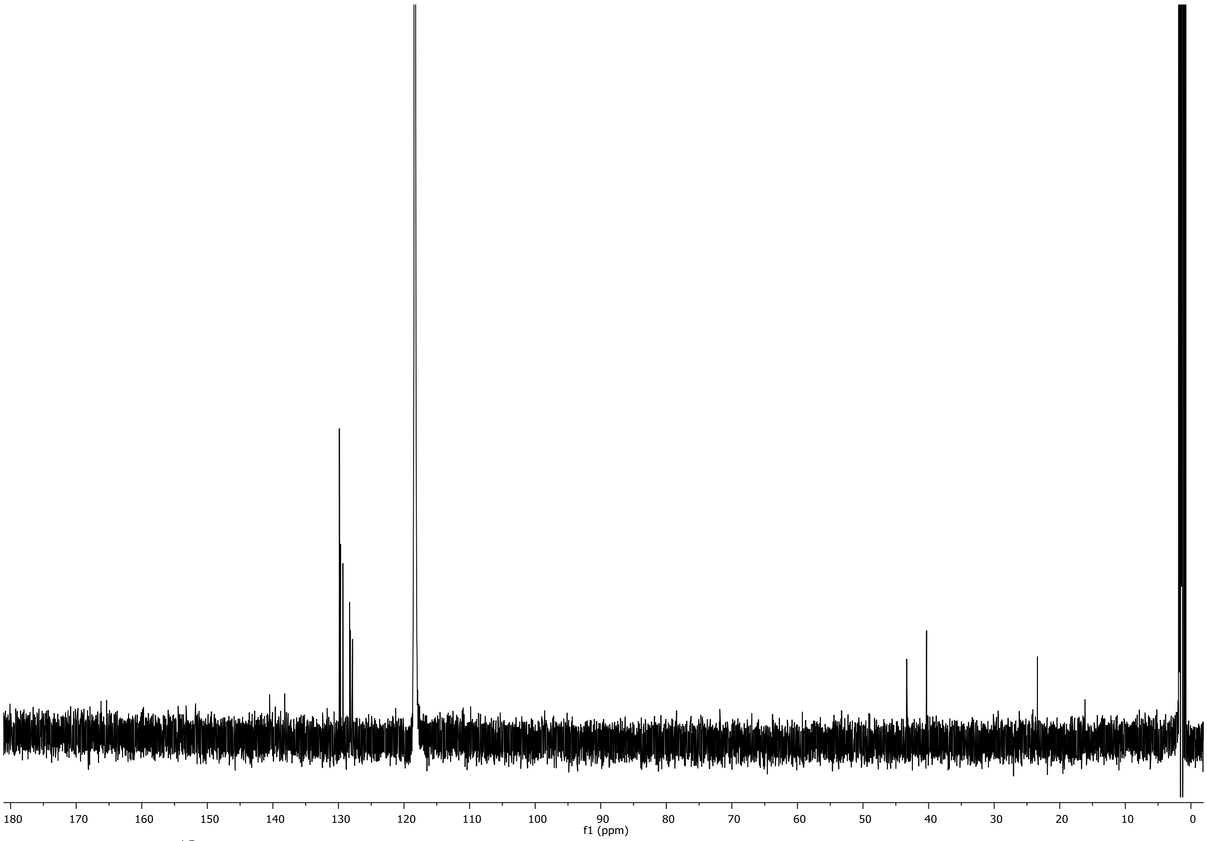

Figure M91: ^{13}C NMR (500 MHz) spectrum of benzyl-vinyl sulfide in CD_3CN following purification.

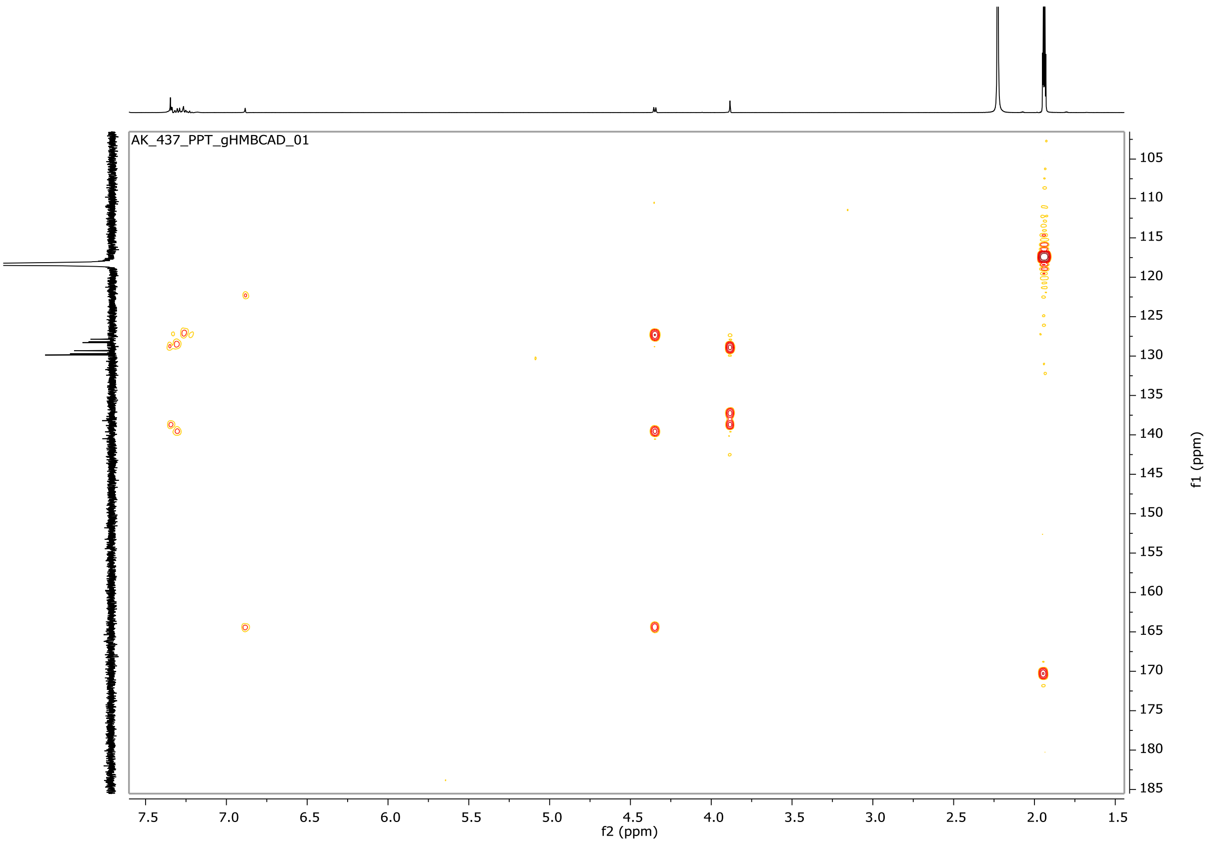

Figure M92: HMBC NMR (500 MHz) spectrum of benzyl-vinyl sulfide in CD_3CN following purification.

I. Photolysis of tripeptide phenacylsulfide derivatives

Synthesis of compound 21

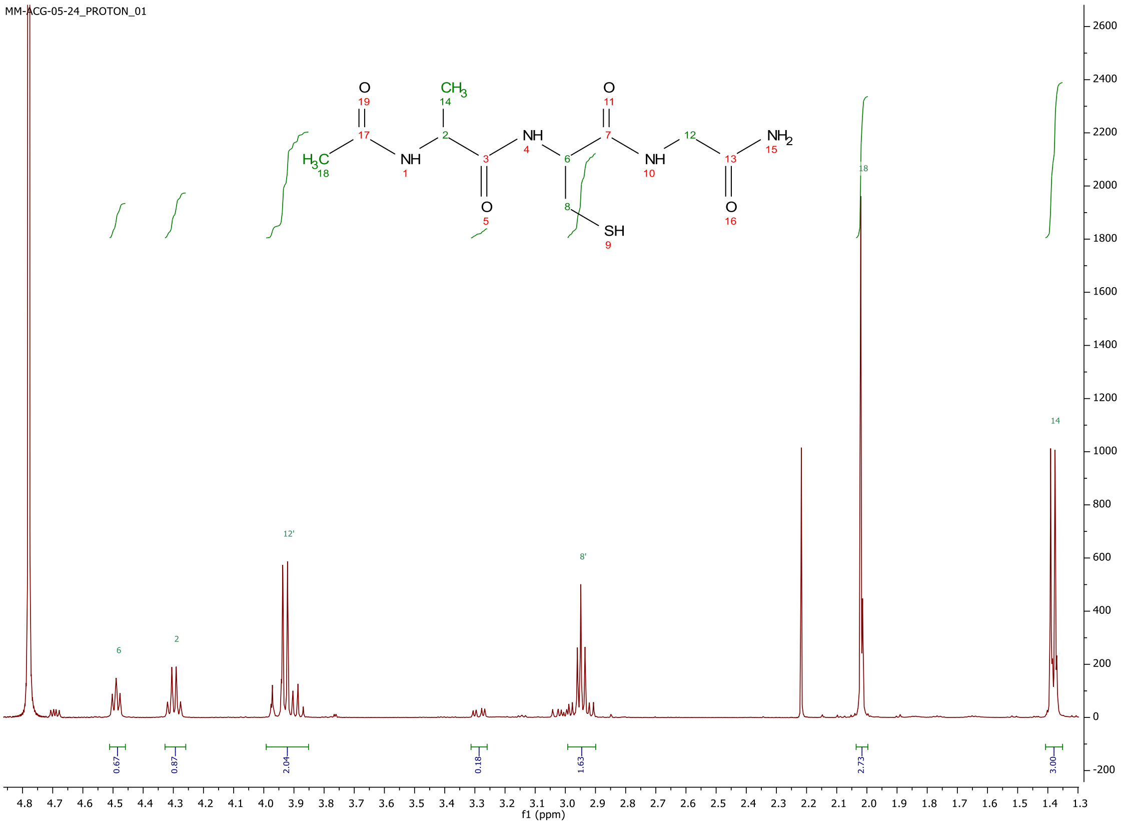

*Figure M93: ^{1}H NMR (500 MHz) spectrum of compound **21** in D_2O. Disulfide (**23**) is present as an impurity(~ 10%).*

Synthesis of compound 22b.

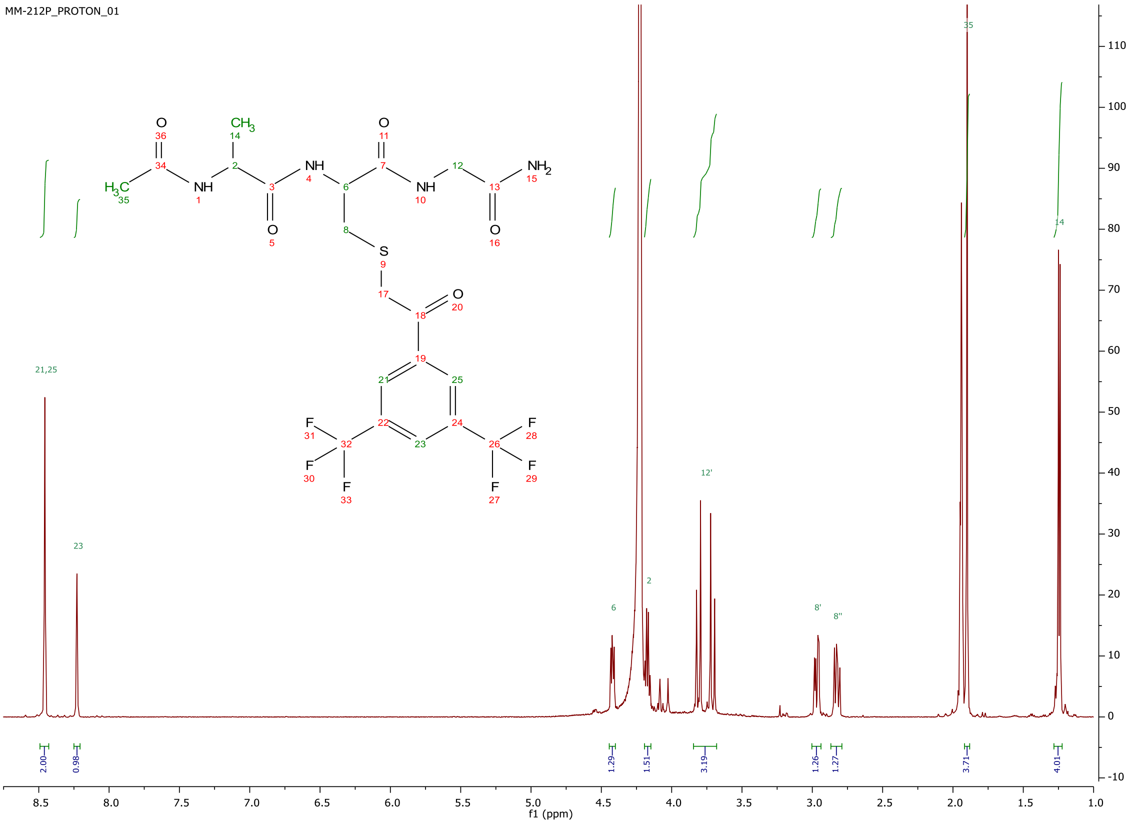

*Figure M94: ^{1}H NMR spectrum (600 MHz) of **22b** following purification in D_2O/MeCN-D_3 (7:3).*

Photolysis of compound 22a.

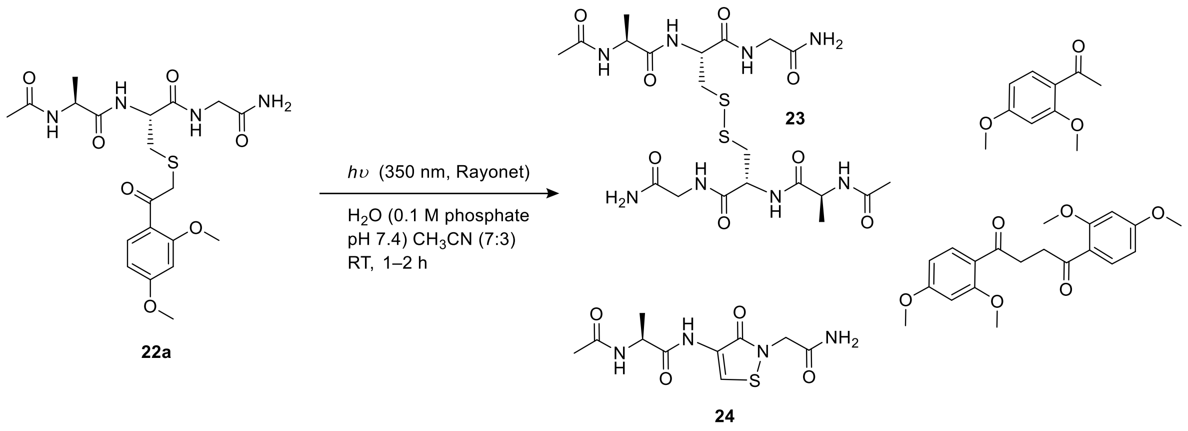

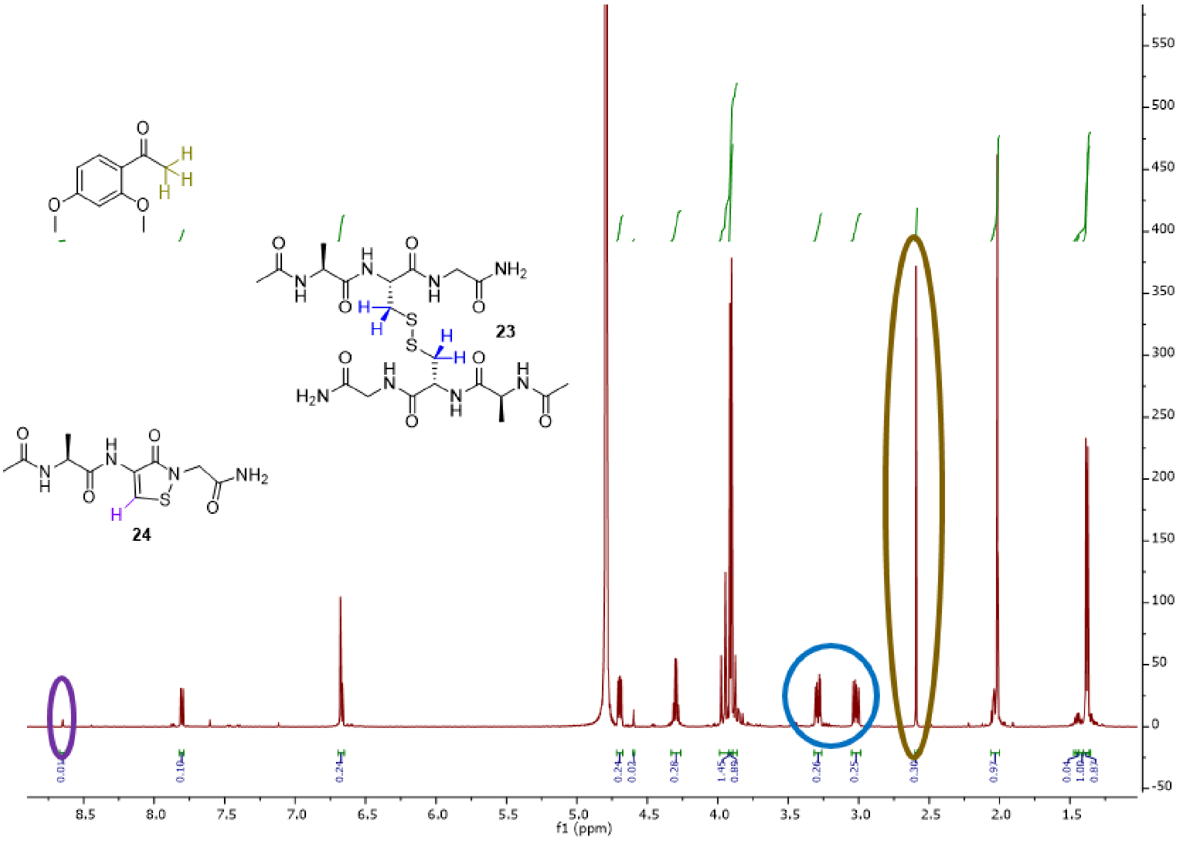

*Figure M95: ^{1}H NMR spectrum (600 MHz) of **22a** following photolysis in D_2O. Characteristic signals of each of the reaction products are highlighted.*

.

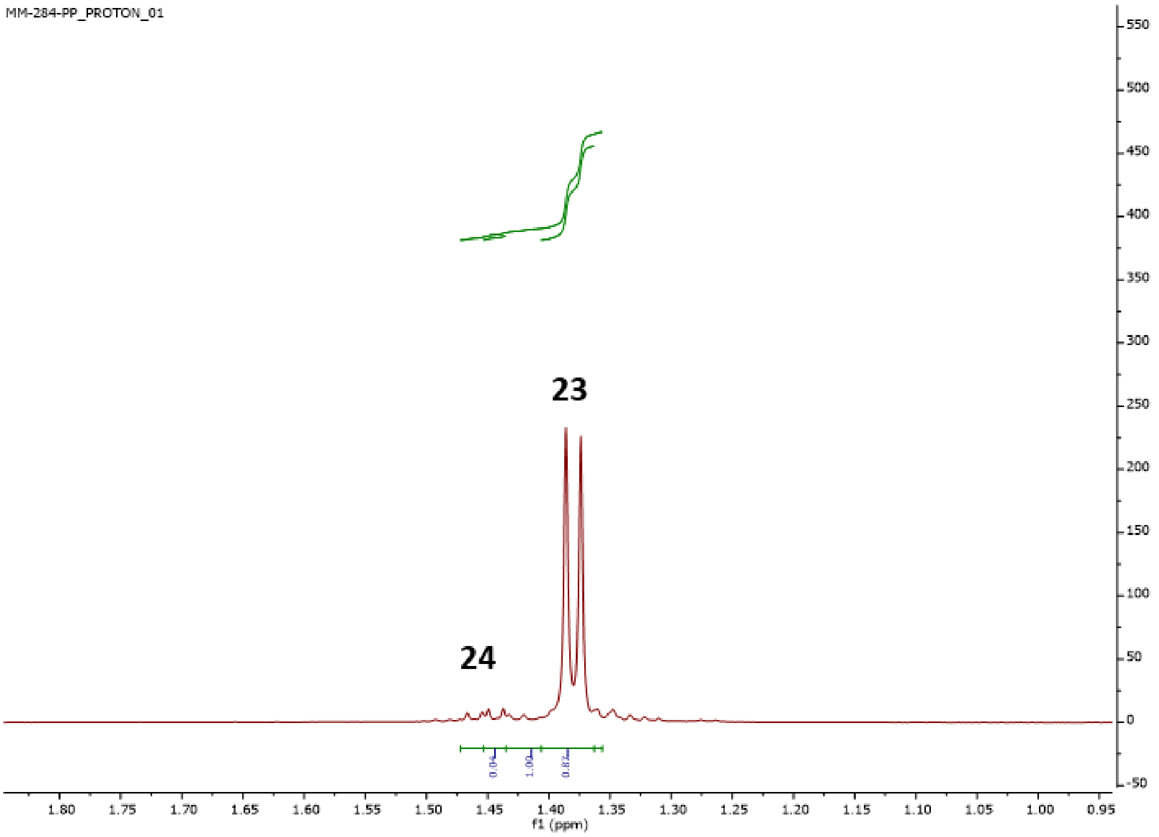

*Figure M96: ^{1}H NMR spectrum (600 MHz) zoomed in the alanine region of the compound **22a** photolysis mixture showing signals corresponding to reaction products, disulfide (**23**) and isothiazolone (**24**) integration of each signal allowing for determination of the conversion percentage of each product.*

Photolysis of compound 22b.

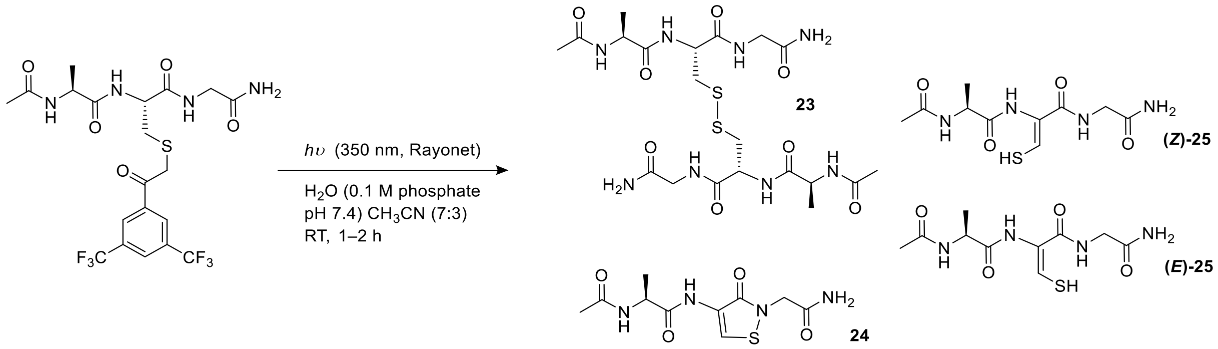

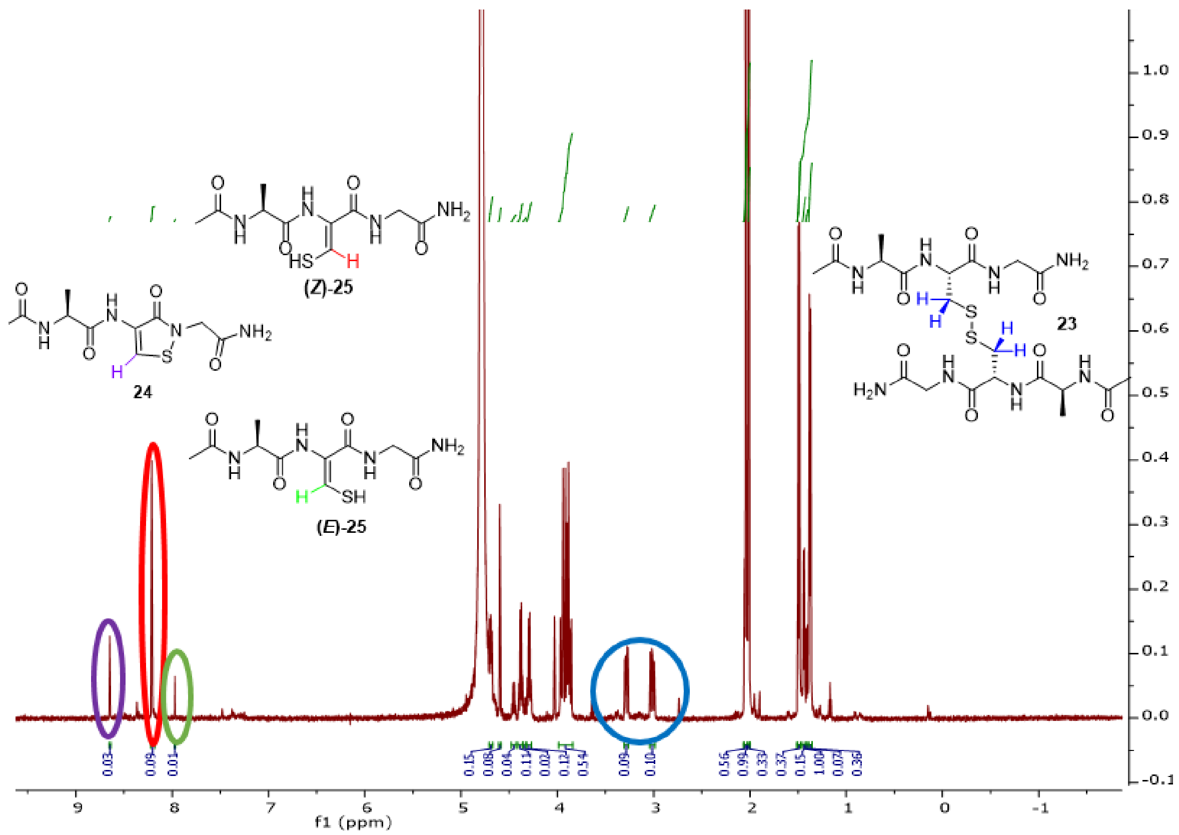

*Figure M97: ^{1}H NMR spectrum (600 MHz) of **22b** following photolysis in D_2O. Characteristic signals of each of the reaction products are highlighted.*

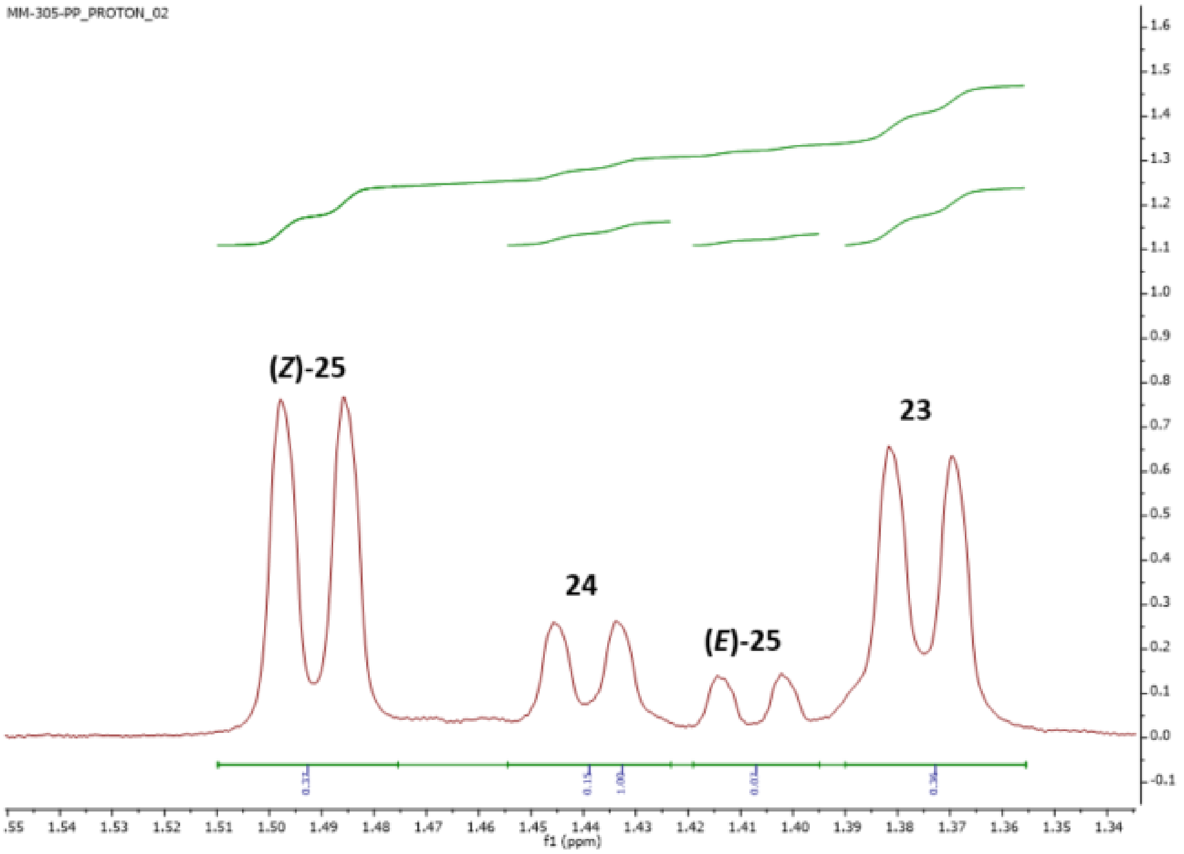

Figure M98: ^{1}H NMR spectrum (600 MHz) zoomed in the alanine region of the compound ***22b*** *photolysis mixture showing signals corresponding to reaction products, disulfide (**23**), isothiazolone (**24**), (E)-enethiol (**(E)-25**) and (Z)-enethiol (**(Z)-25**) integration of each signal allowing for determination of the conversion percentage of each product.*

Photolysis of compound 22c.

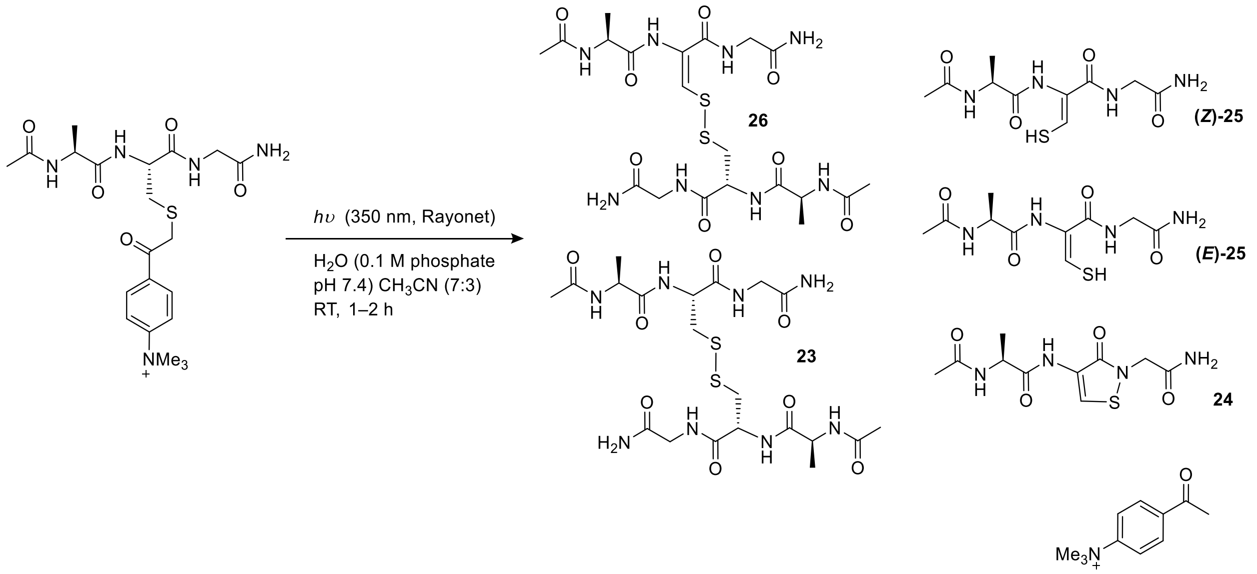

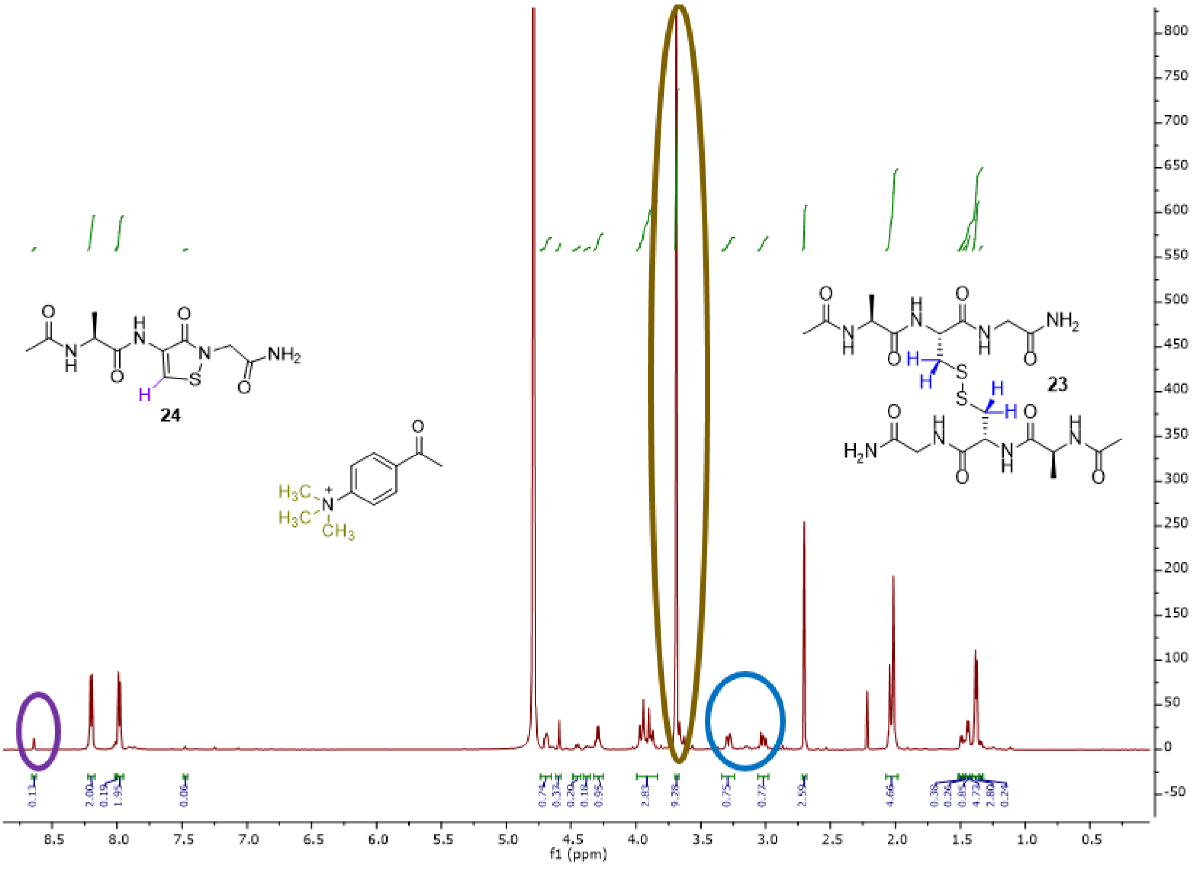

*Figure M99: ^{1}H NMR spectrum (600 MHz) of **22c** following photolysis in D_2O. Characteristic signals of each of the reaction products are highlighted.*

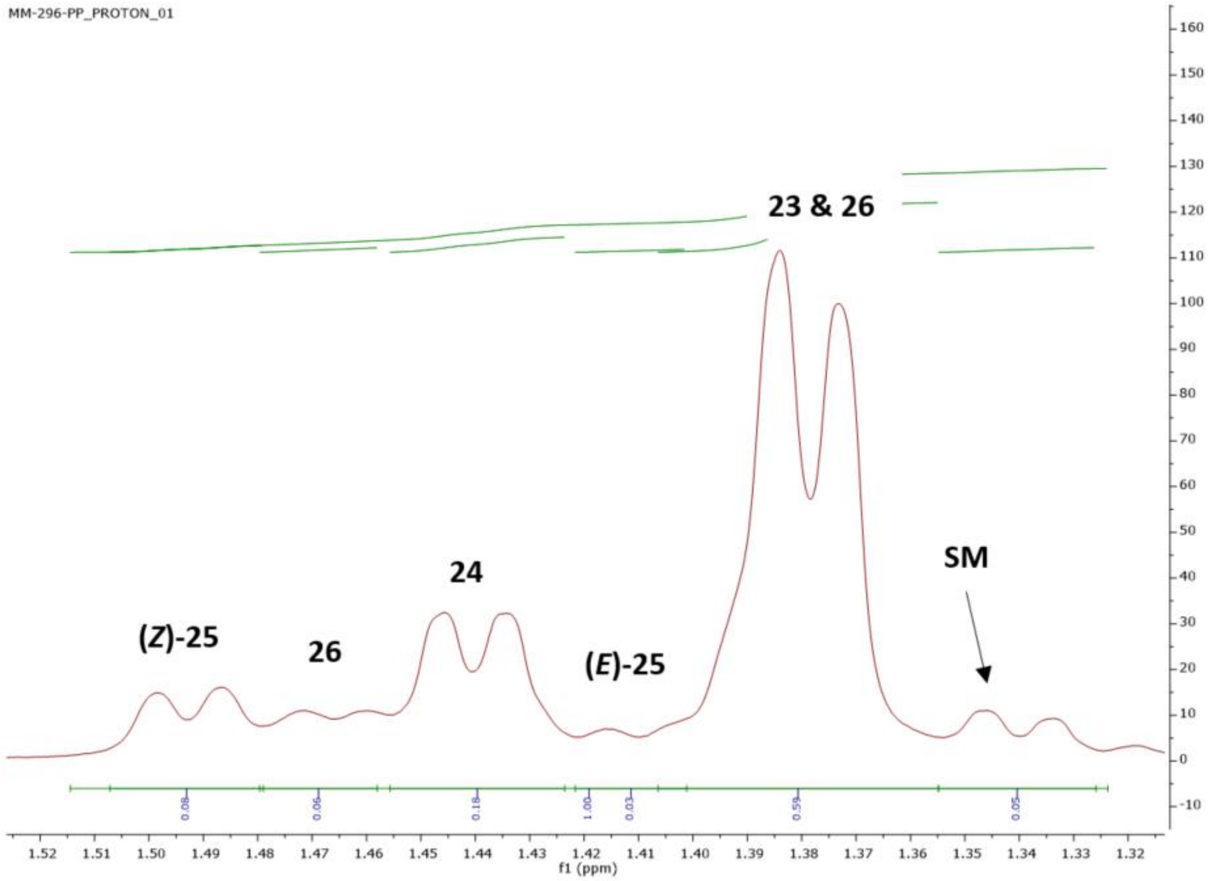

*Figure M100: ^{1}H NMR spectrum (600 MHz) zoomed in the alanine region of the compound **22c** photolysis mixture showing signals corresponding to reaction products, disulfide (**23**), isothiazolone (**24**), (E)-enethiol (**(E)-25**), (Z)-enethiol (**(Z)-25**) and thiol/enethiol disulfide (**26**) integration of each signal allowing for determination of the conversion percentage of each product.*

Photolysis of phenacylsulfide 22b in the presence of 3 M guanidinium

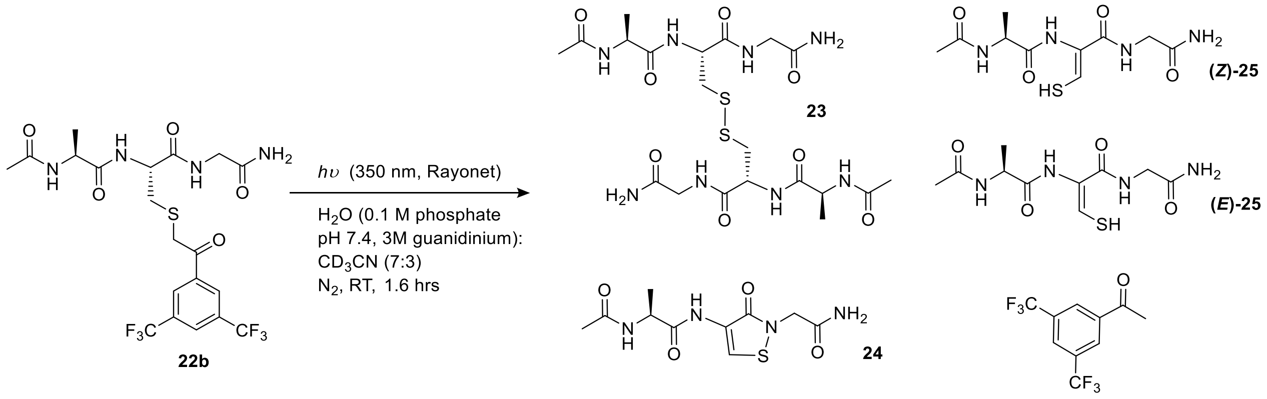

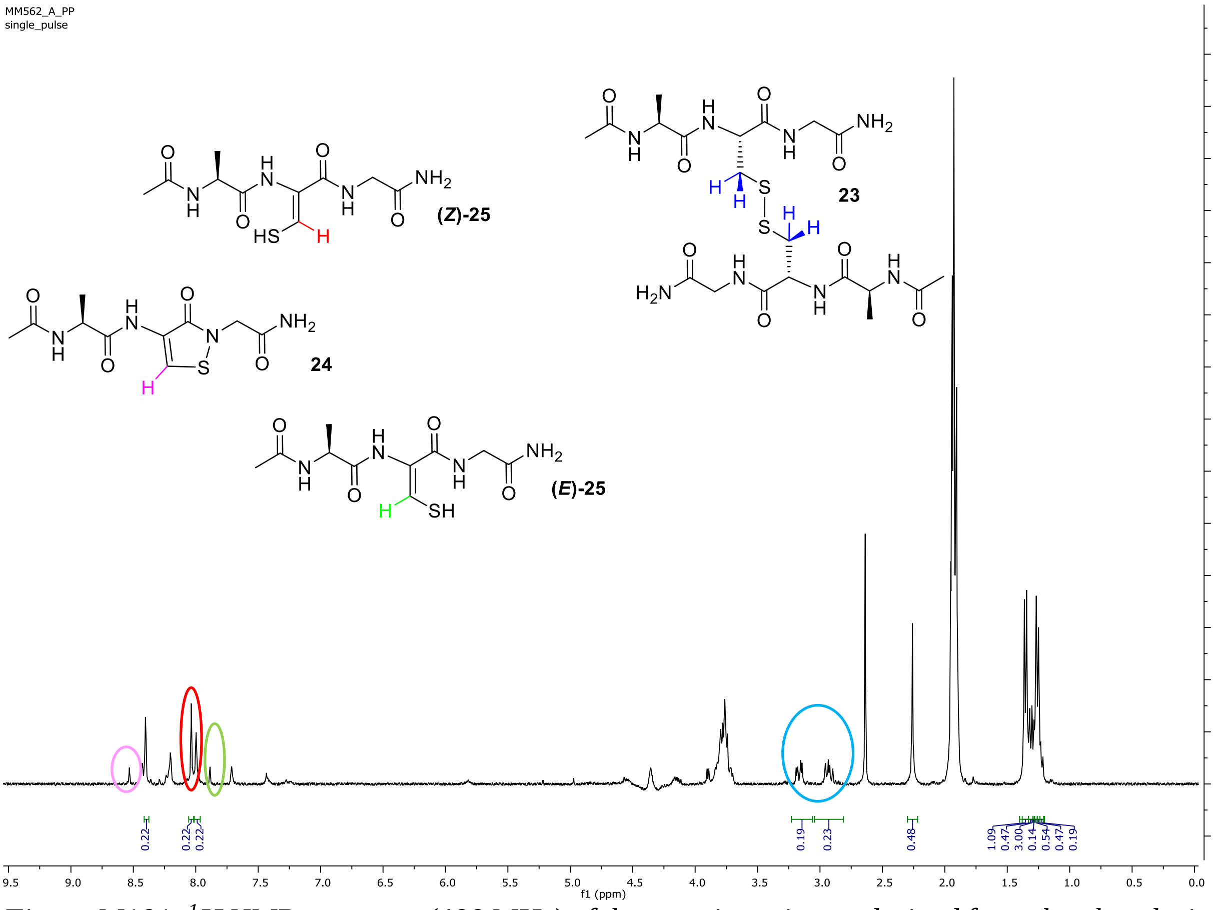

*Figure M101: ^{1}H NMR spectrum (400 MHz) of the reaction mixture derived from the photolysis of **22b** in H_2O (0.1 M, phosphate pH 7.4, 3M guanidinium) /CD_3CN (7:3). Characteristic signals of each of the reaction products are highlighted.*

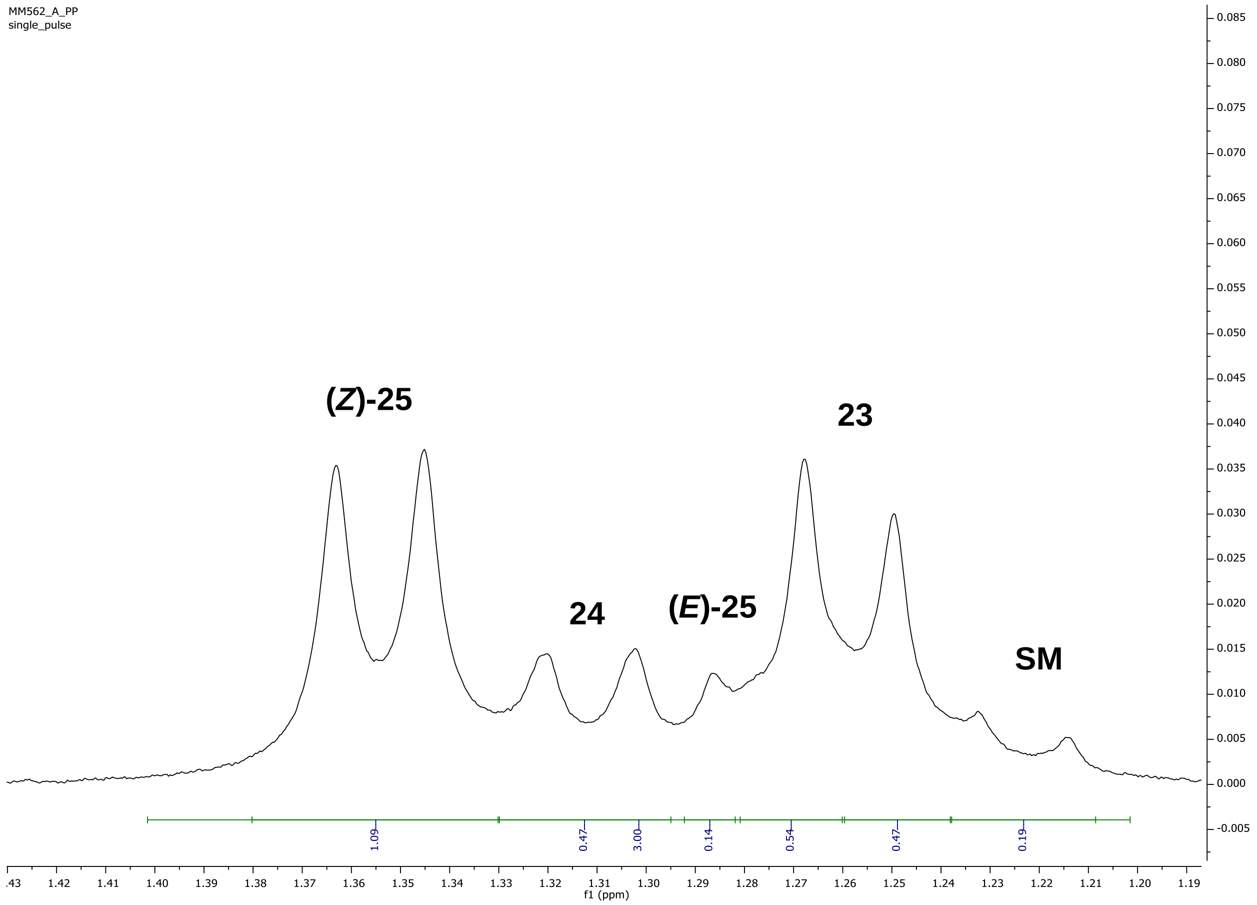

Figure M102: ^{1}H NMR spectrum (400 MHz) of the reaction mixture derived from the photolysis of ***22b*** *in H_2O (0.1 M, phosphate pH 7.4, 3M guanidinium) /CD_3CN (7:3) zoomed in the alanine region. Signals corresponding to reaction products, disulfide (**23**), isothiazolone (**24**), (E)-enethiol (**(E)-25**) and (Z)-enethiol (**(Z)-25**) are highlighted. Integration of each signal allows for determination of the conversion percentage of each product.*

Characterisation of Ac-AlaCysGly-NH2 photolysis products from crude reaction mixtures

Characterisation of AC-AlaCysGly-NH2 disulfide (23)

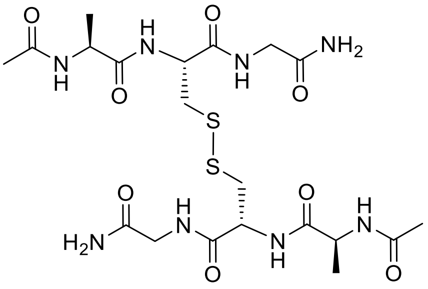

Characterised from photolysis mixture derived from compound 22a

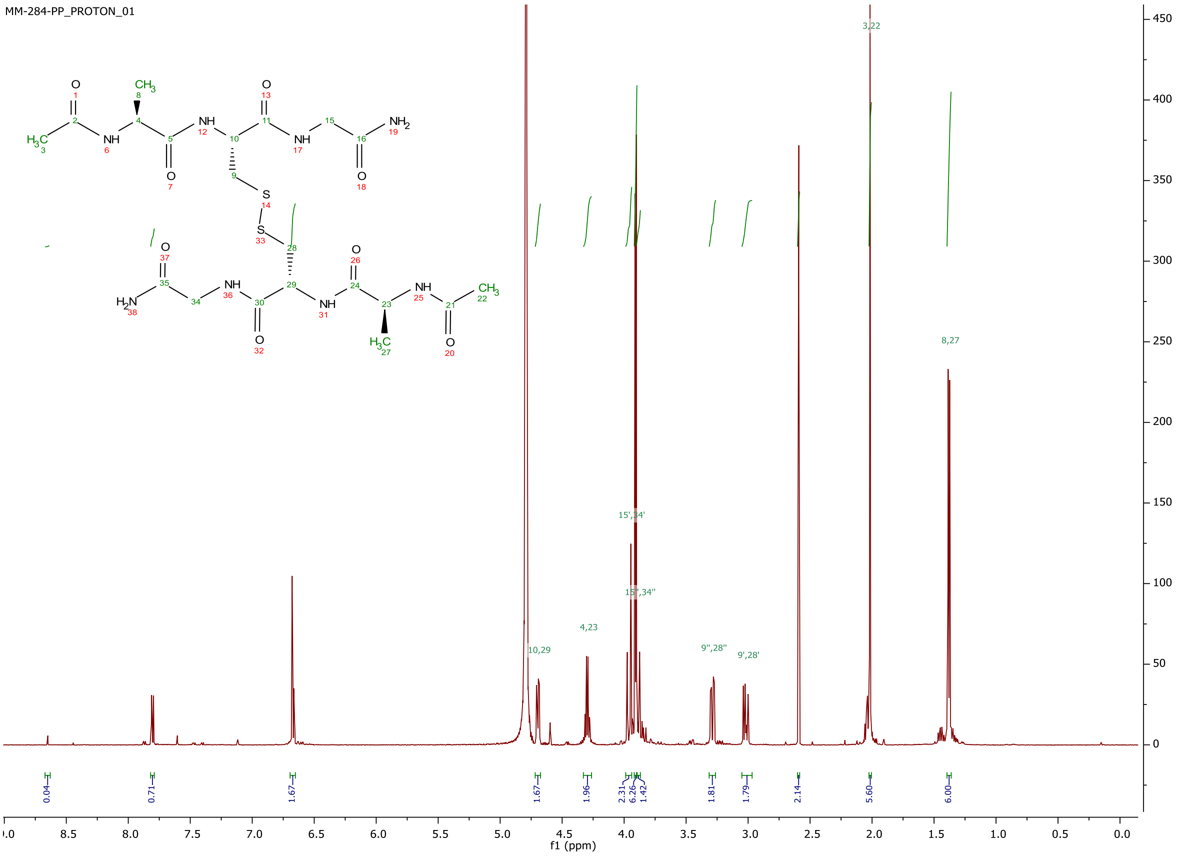

*Figure M103: ^{1}H NMR spectrum (600MHz) of the compound **22a** photolysis mixture in D_2O, highlighting signals corresponding compound **23**. Unassigned signals at 2.59, 3.90, 3.91, 6.66, 6.67 & 7.8 ppm are accounted for by the 1-(2,4-dimethoxyphenyl)ethan-1-one by product of the photolysis reaction.*

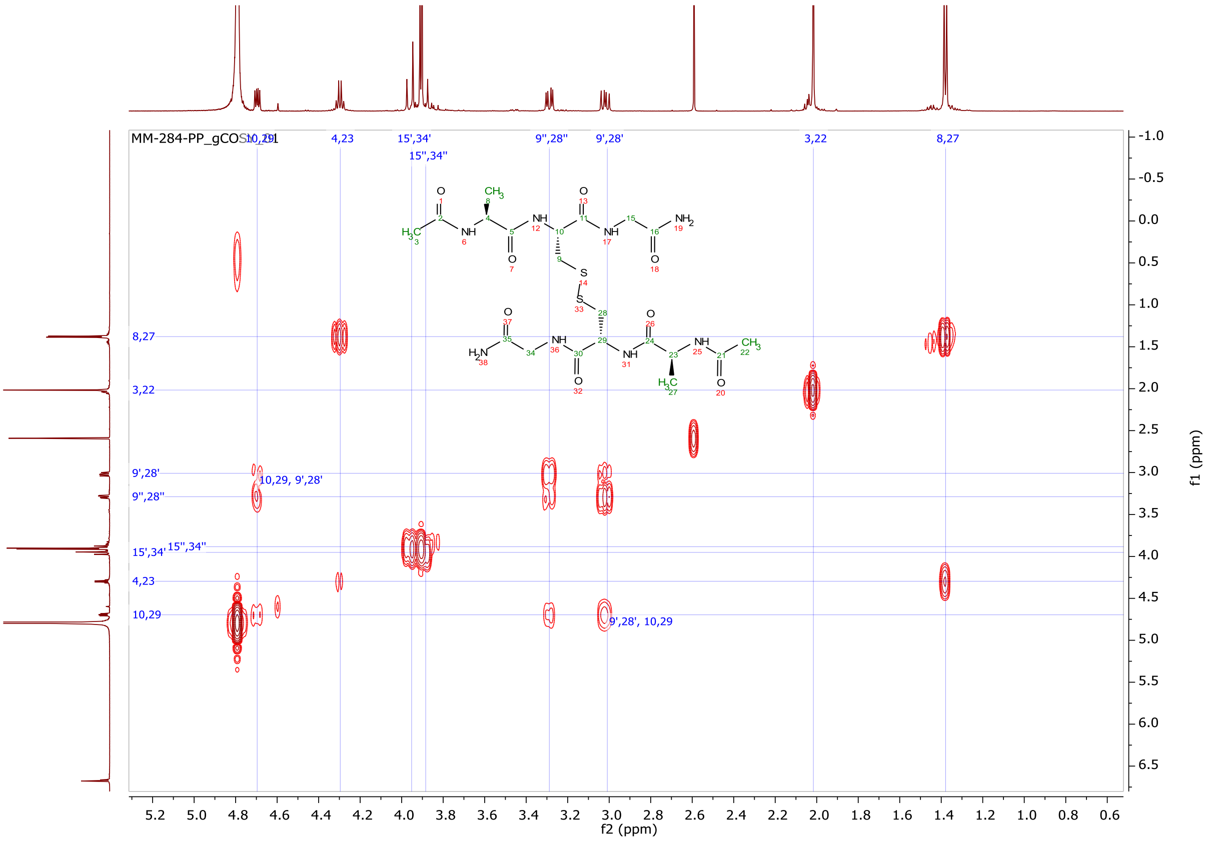

*Figure M104: COSY NMR spectrum of compound **22a** photolysis mixture, highlighting assignments of compound **23**. Unassigned correlations are accounted for by the 1-(2,4-dimethoxyphenyl)ethan-1-one by product of the photolysis reaction.*

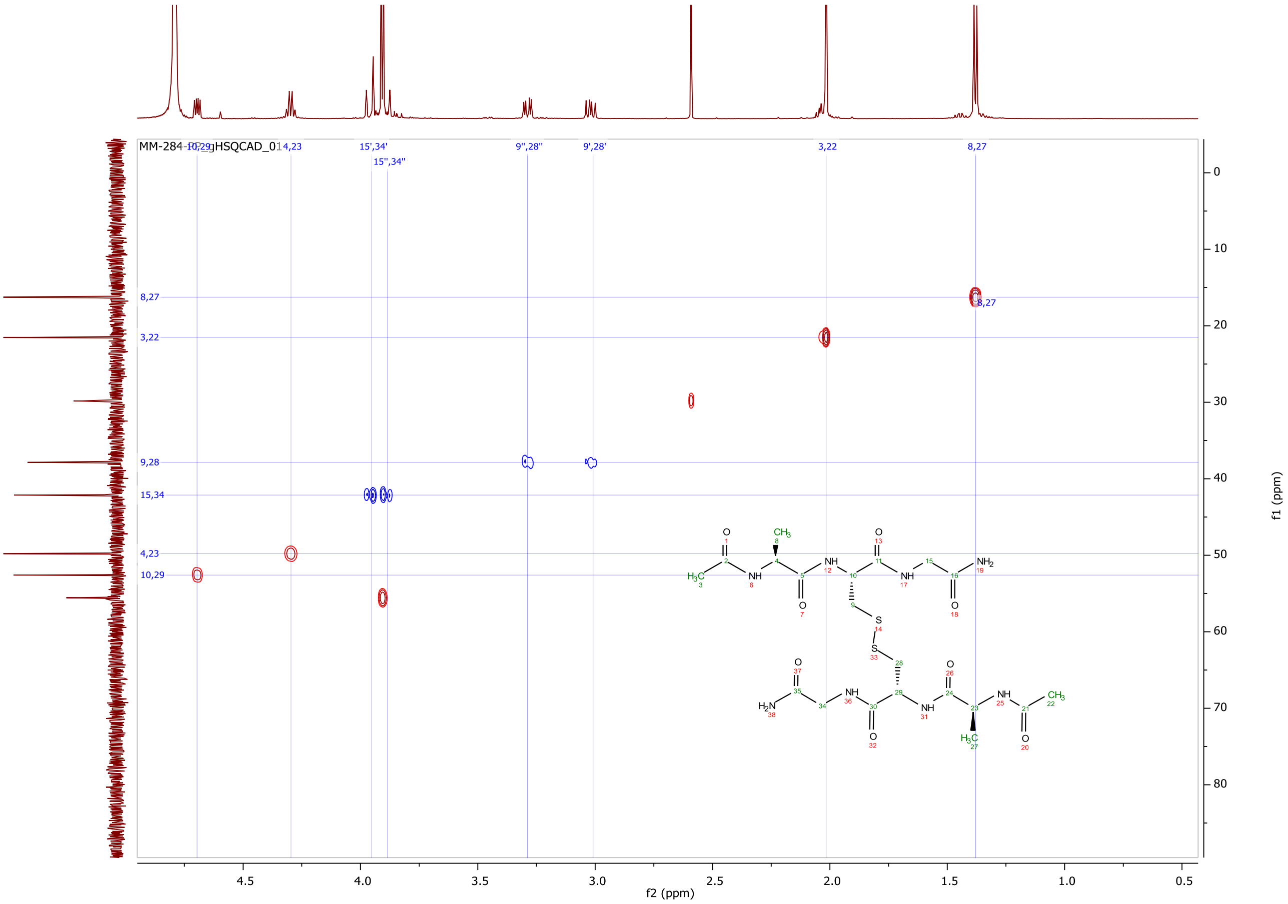

*Figure M105: HSQC NMR spectrum of the compound **22a** photolysis mixture, highlighting assignments of compound **23**. Unassigned correlations are accounted for by the 1-(2,4-dimethoxyphenyl)ethan-1-one by product of the photolysis reaction.*

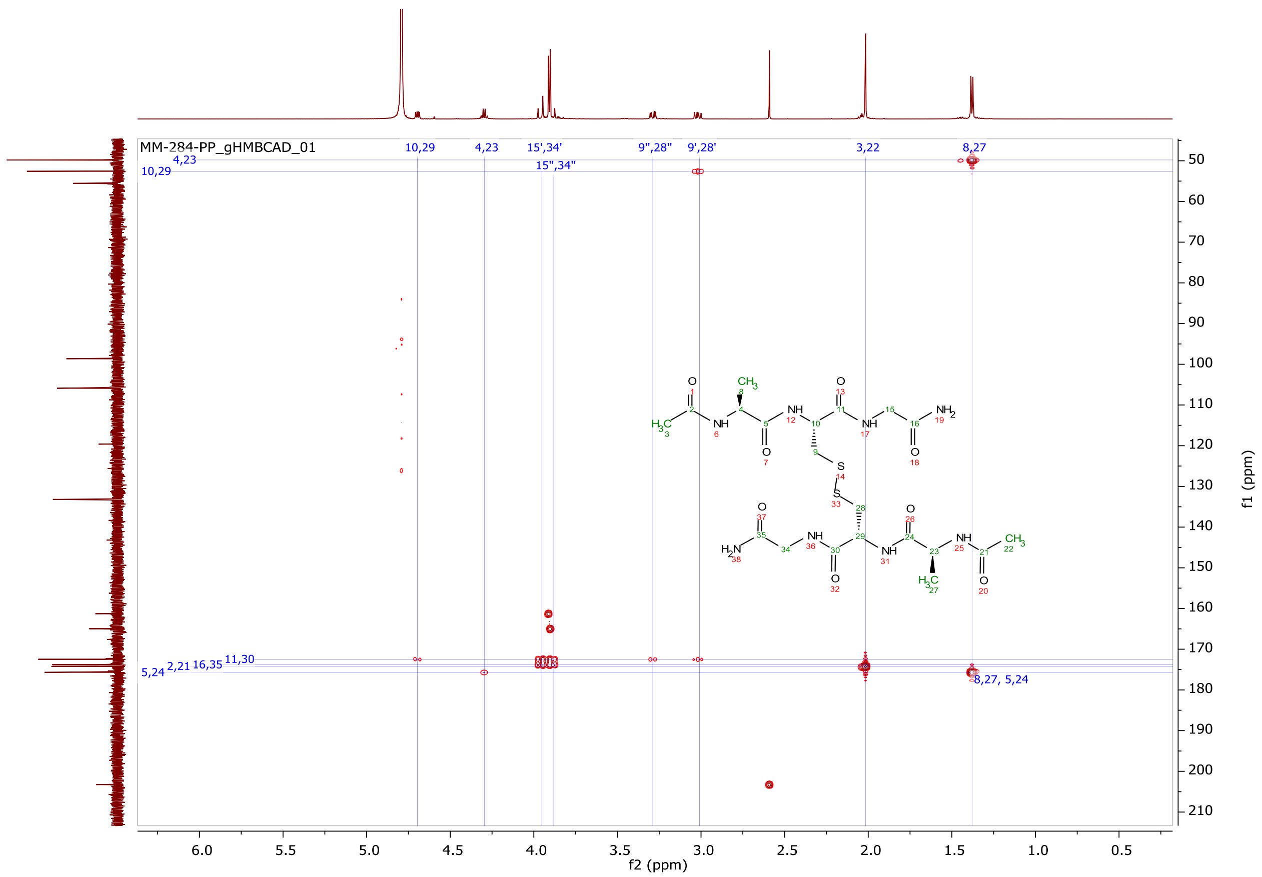

*Figure M106: HMBC spectrum of the compound **22a** photolysis mixture, highlighting the assignments of the quaternary carbons of compound **23**. Unassigned correlations are accounted for by the 1-(2,4-dimethoxyphenyl)ethan-1-one by product of the photolysis reaction.*

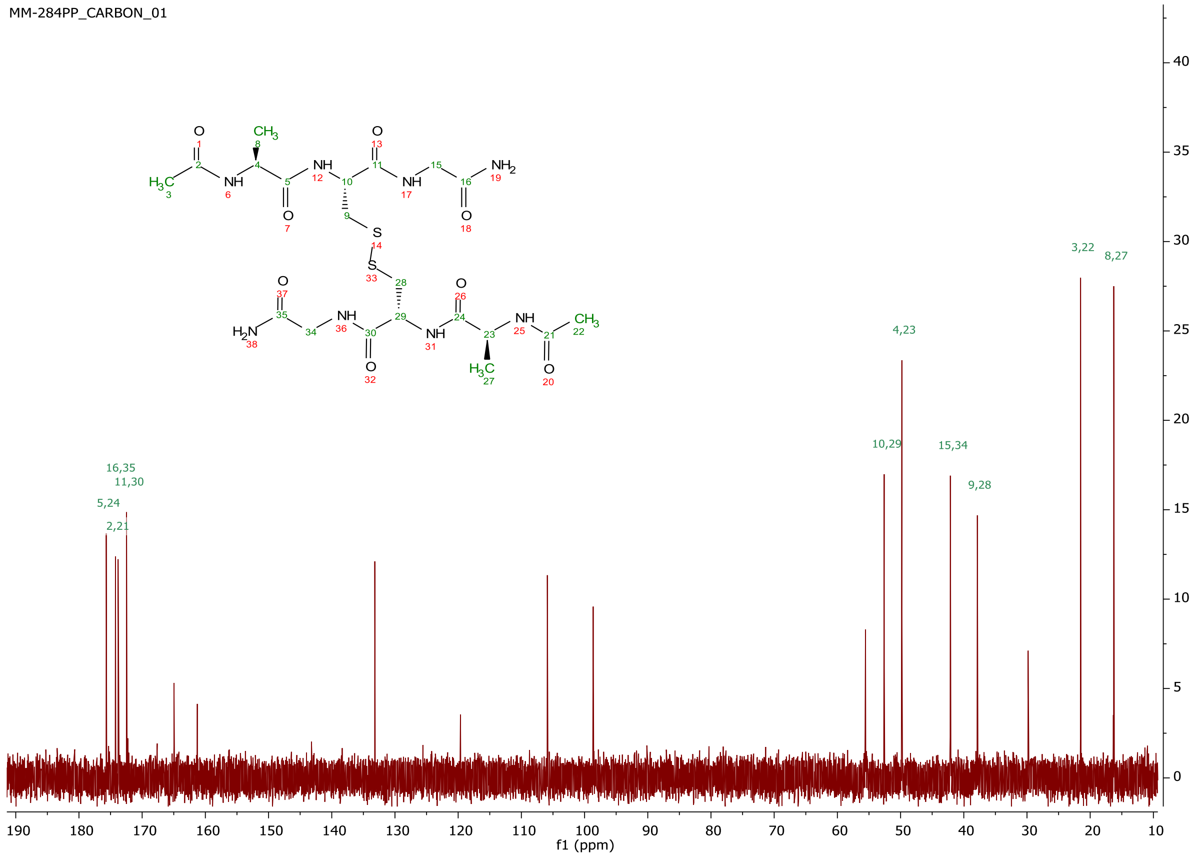

*Figure M107: ^{13}C NMR (151 MHz) spectrum of the compound **22a** photolysis mixture in D_2O, highlighting signals corresponding to compound **23**. Unassigned peaks are accounted for by the 1-(2,4-dimethoxyphenyl)ethan-1-one by product of the photolysis reaction.*

Characterisation of Ac-AlaCysGly-NH_2 isothiazolone (24)

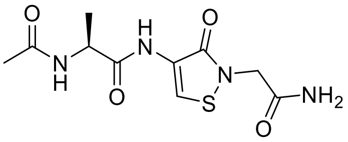

Exact Mass: 286.0736

Characterised from photolysis mixture derived from compound 22c

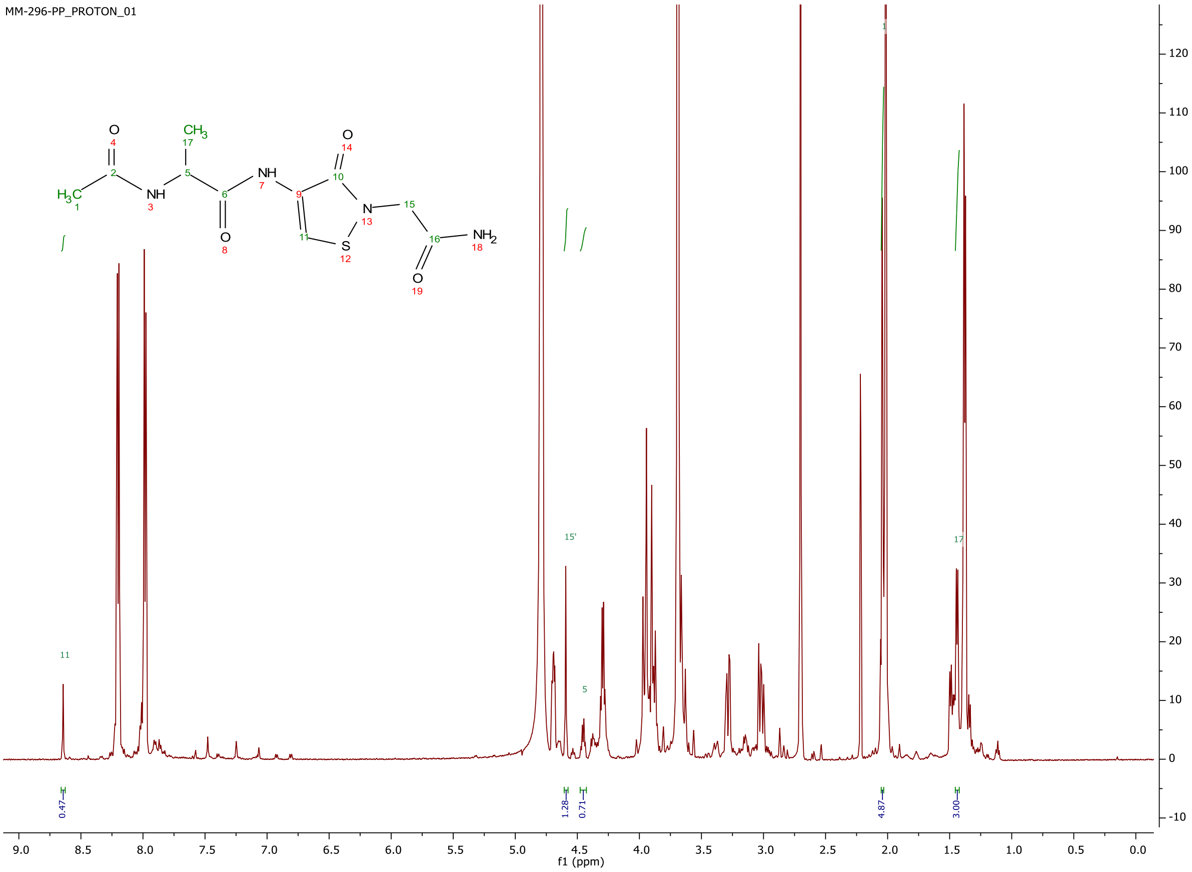

*Figure M108: ^{1}H NMR spectrum (600 MHz) of the compound **22c** photolysis mixture in D_2O, highlighting signals corresponding compound **24**. Unassigned signals are accounted for by the other reaction products,*

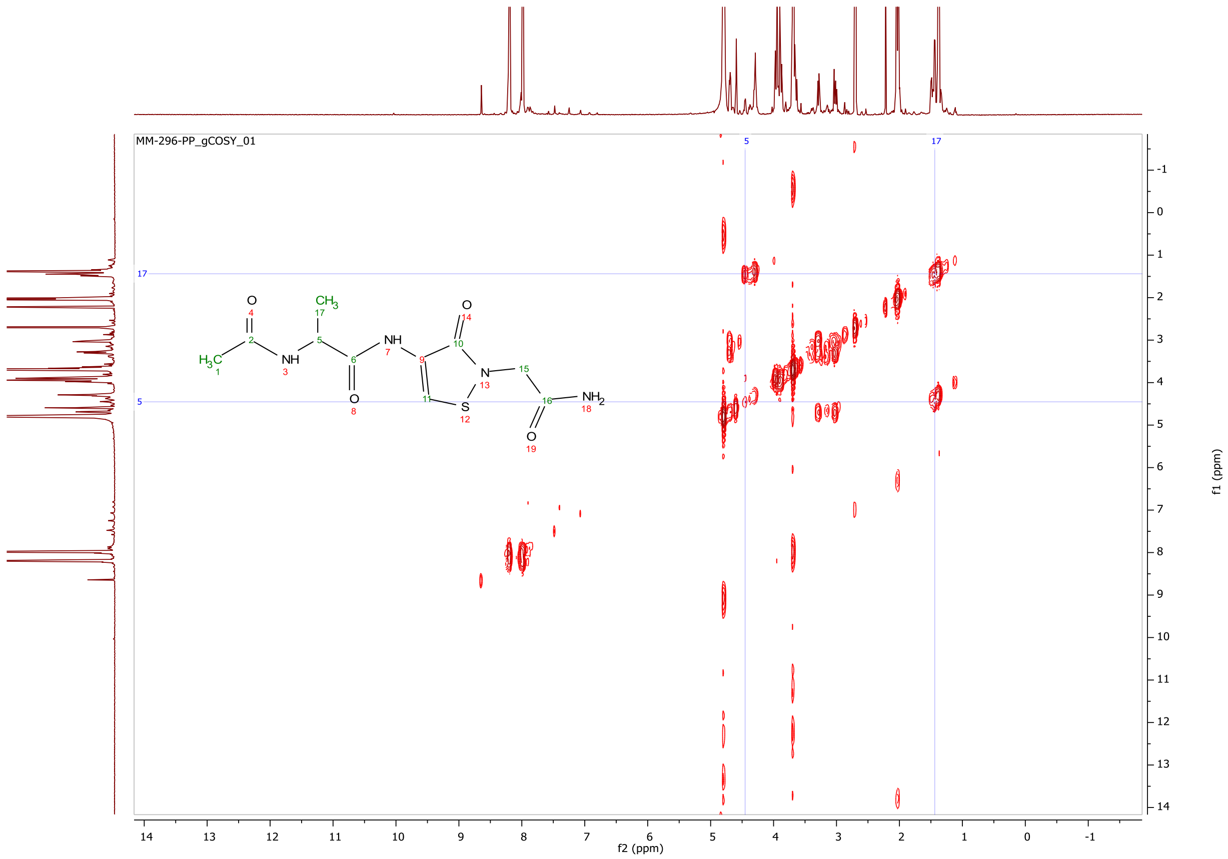

*Figure M109: COSY NMR spectrum of the compound **22c** photolysis mixture highlighting assignments of compound **24**. The correlation between the methyl group of the alanine and the α-proton of the alanine residue is highlighted. Unassigned correlations are accounted for by the other products of the photolysis reaction.*

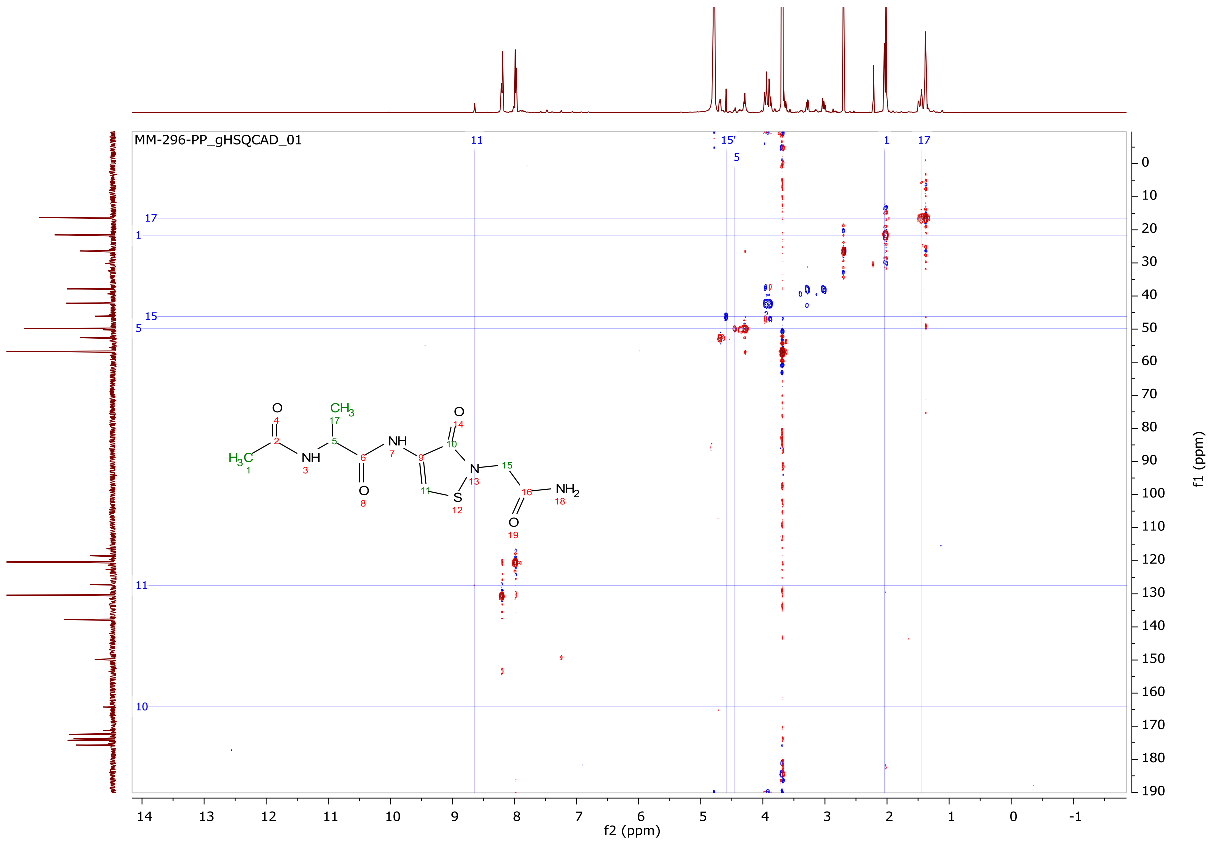

*Figure M110: HSQC NMR spectrum of the compound **22c** photolysis mixture, highlighting assignments of compound **24**. Unassigned correlations are accounted for by the other products of the photolysis reaction*

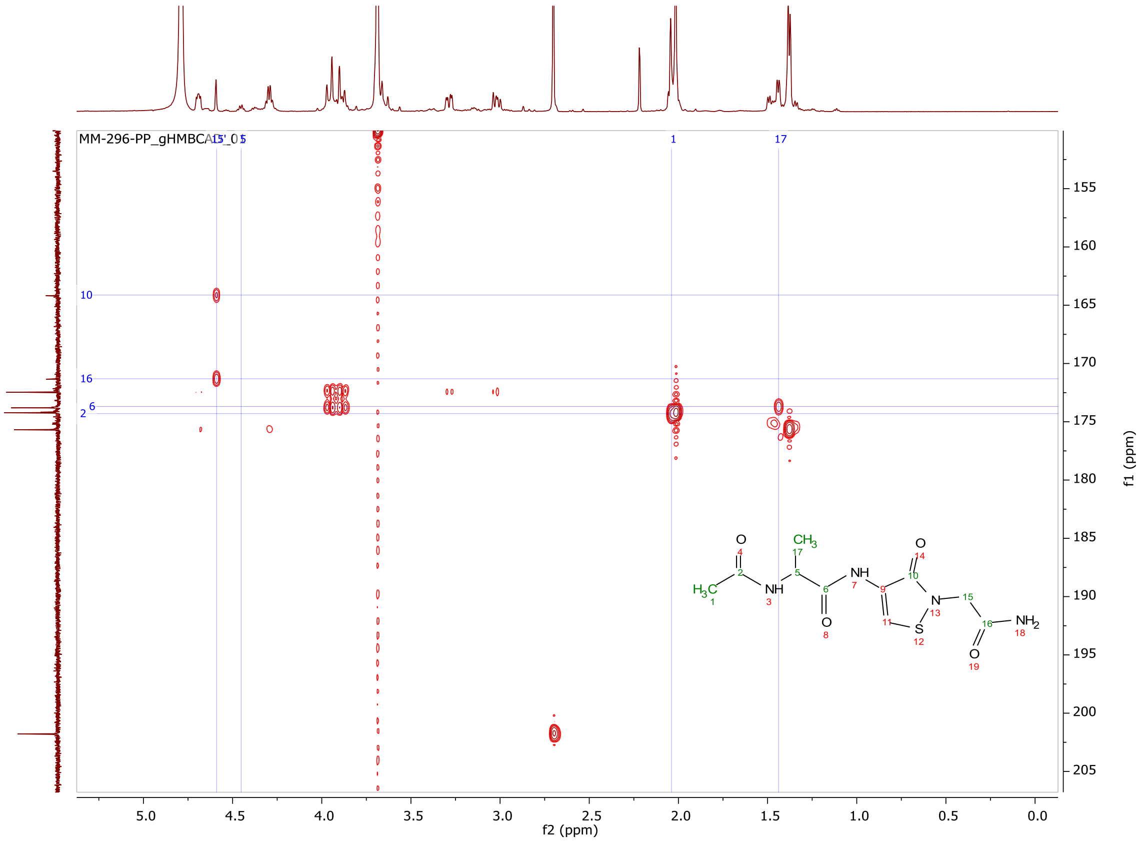

*Figure M111: HMBC NMR spectrum of the compound **22c** photolysis mixture, highlighting assignments of compound **24**. The assignments of the quaternary carbons are highlighted. Note: No correlation was observed for the α carbon of the of the isothiazolone moiety (labelled as 9 in the structure above).*

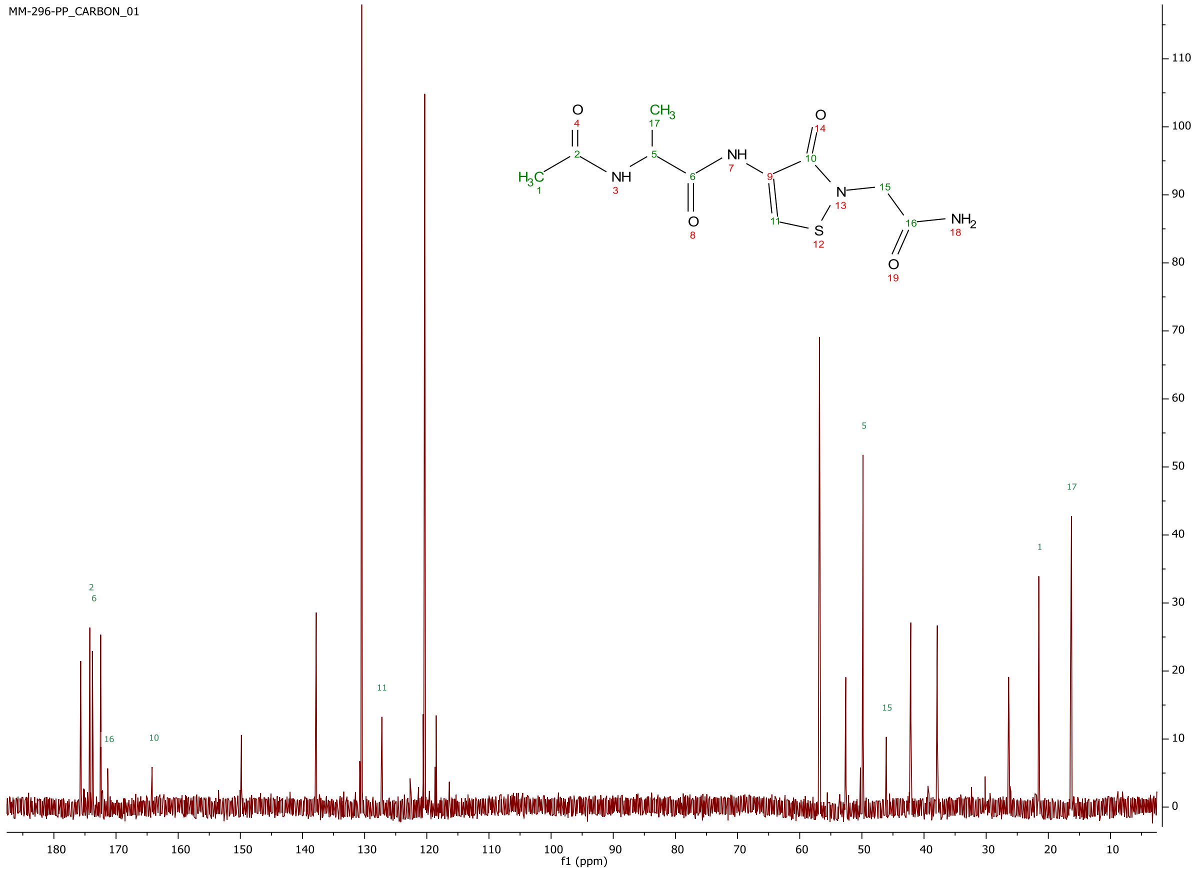

*Figure M112: ^{13}C NMR (151 MHz) spectrum of the compound **22c** photolysis mixture in D_2O, highlighting signals corresponding to compound **24**. Note the α carbon of the isothiazolone moiety (9) could not be assigned.*

Characterisation of Ac-AlaCysGly-NH$_2$ (*Z*)-enethiol (25)

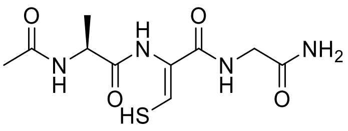

Characterised from photolysis mixture derived from compound 22b

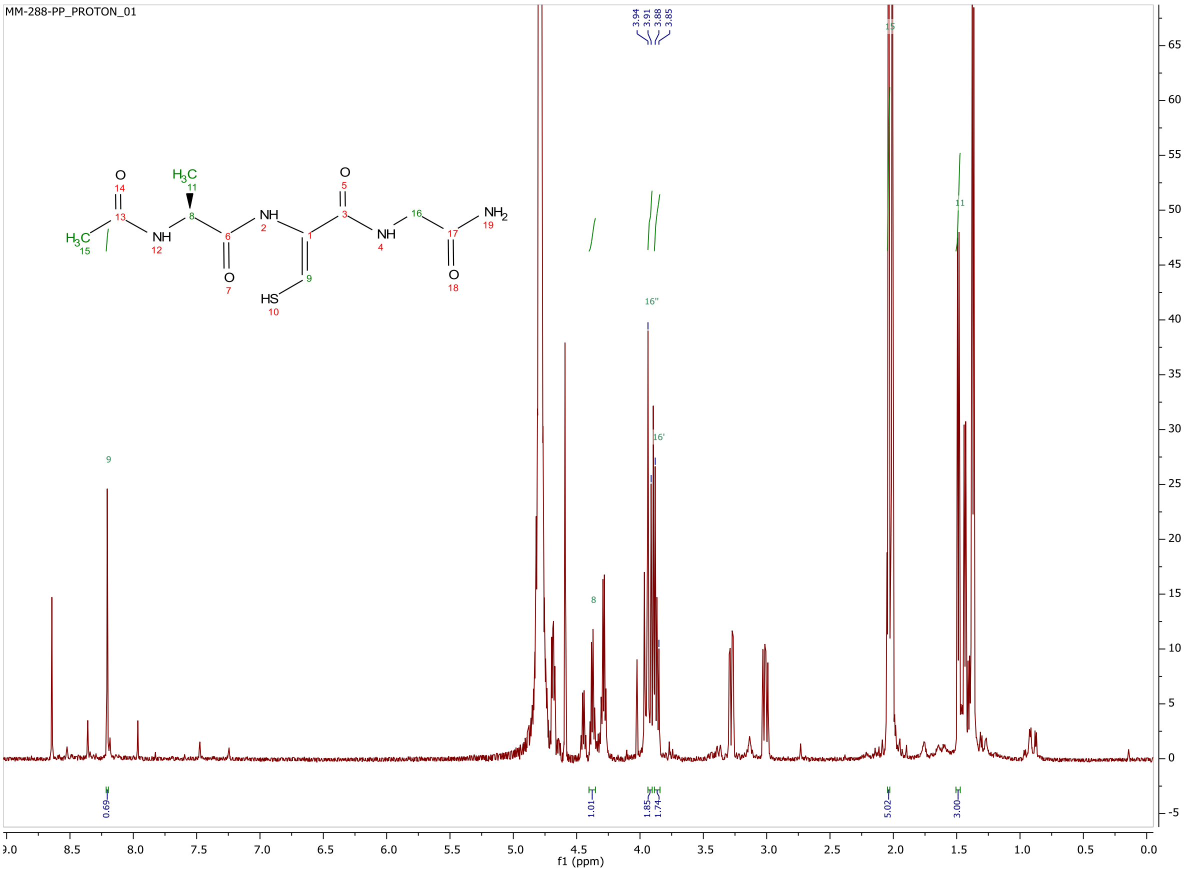

*Figure M113: ^{1}H NMR spectrum (600 MHz) of the compound **22b** photolysis mixture in D_2O, highlighting signals corresponding to compound **(Z)-25**. Unassigned signals are accounted for by the other reaction products.*

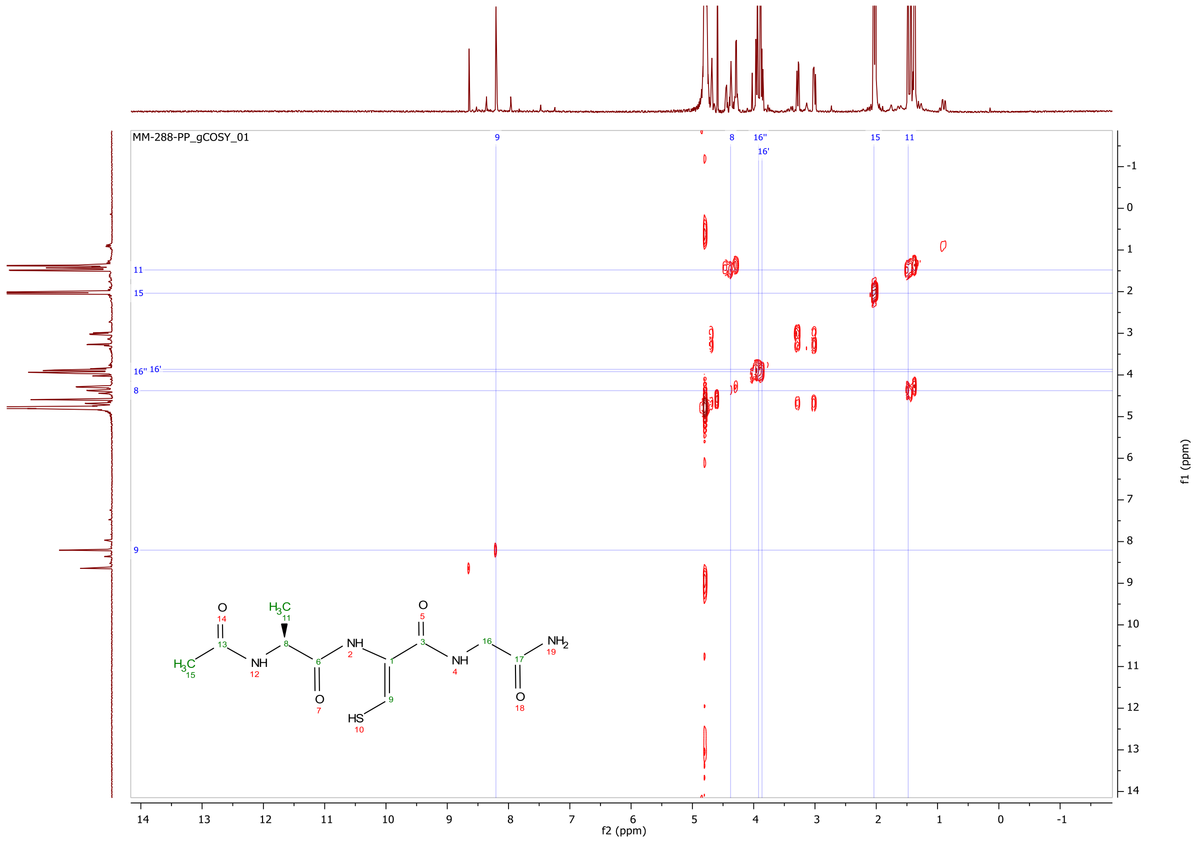

*Figure M114: COSY NMR spectrum of the compound **22b** photolysis mixture highlighting assignments of compound **(Z)-25**. The correlation between the methyl group of the alanine and the α-proton of the alanine residue are highlighted in addition to the germinal coupling of the methylene protons of the glycine residue. Unassigned signals are accounted for by the other reaction products.*

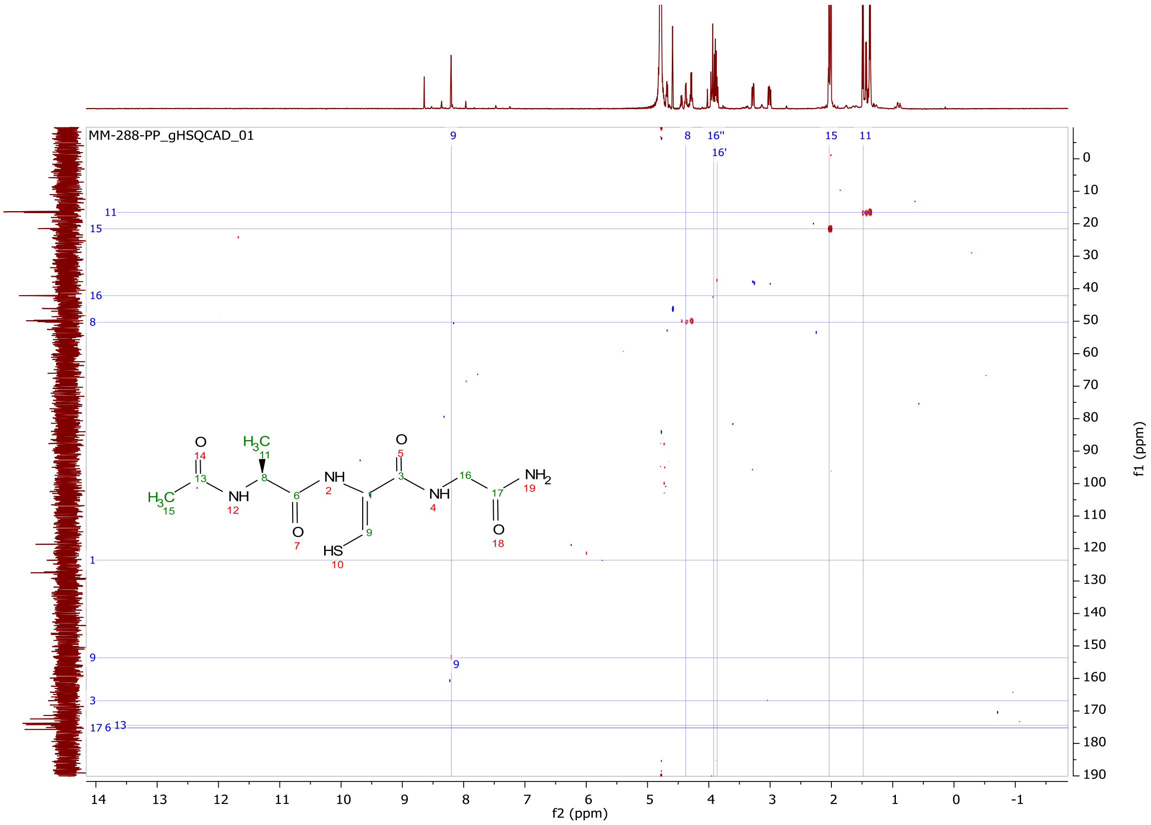

*Figure M115: HSQC NMR spectrum of the compound **22b** photolysis mixture, highlighting assignments of compound **(Z)-25**. Note that while the correlation of the β proton of the enethiol (9) to the β carbon of the moiety is observed, the signal is absent in the ^{13}C spectrum. Unassigned correlations are accounted by the other products of the photolysis reaction.*

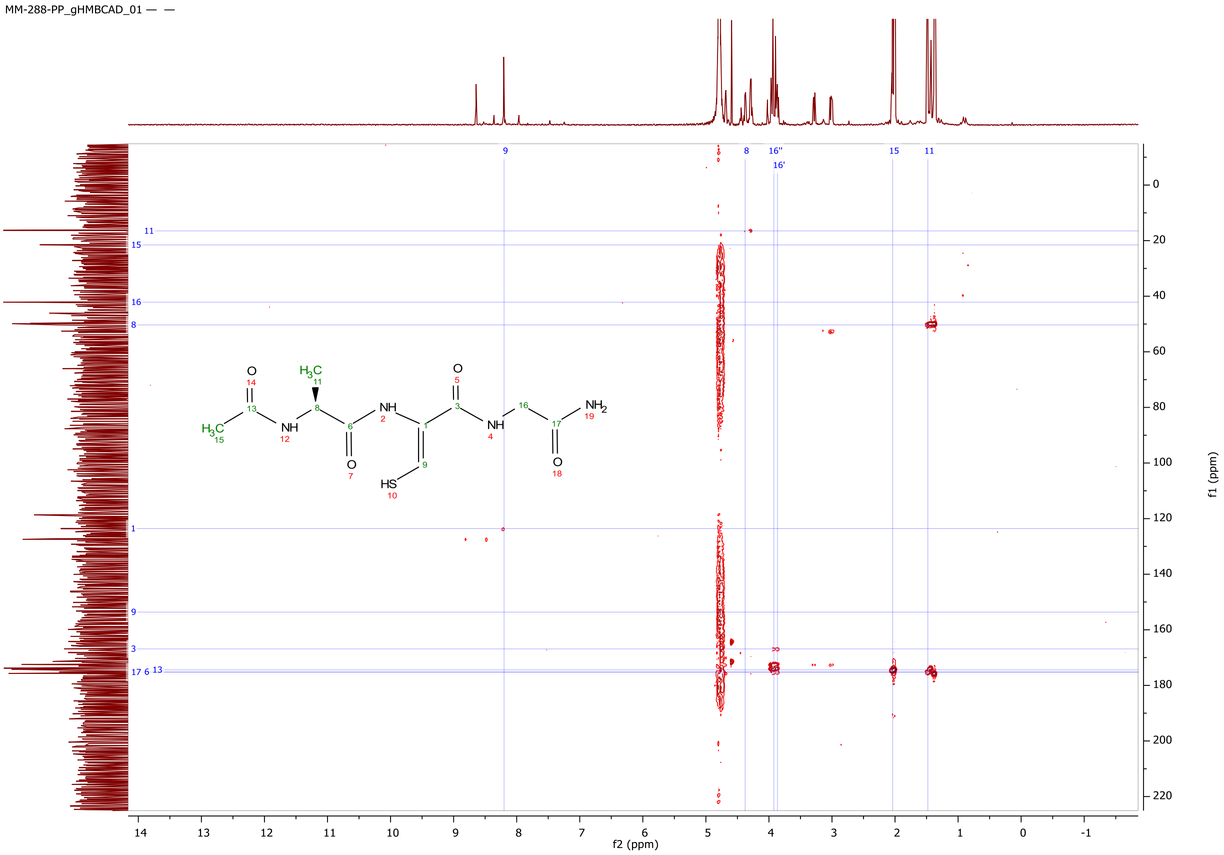

*Figure M116: HMBC NMR spectrum of the compound **22b** photolysis mixture, highlighting assignments of compound **(Z)-25**. The assignments of the quaternary carbons are highlighted.*

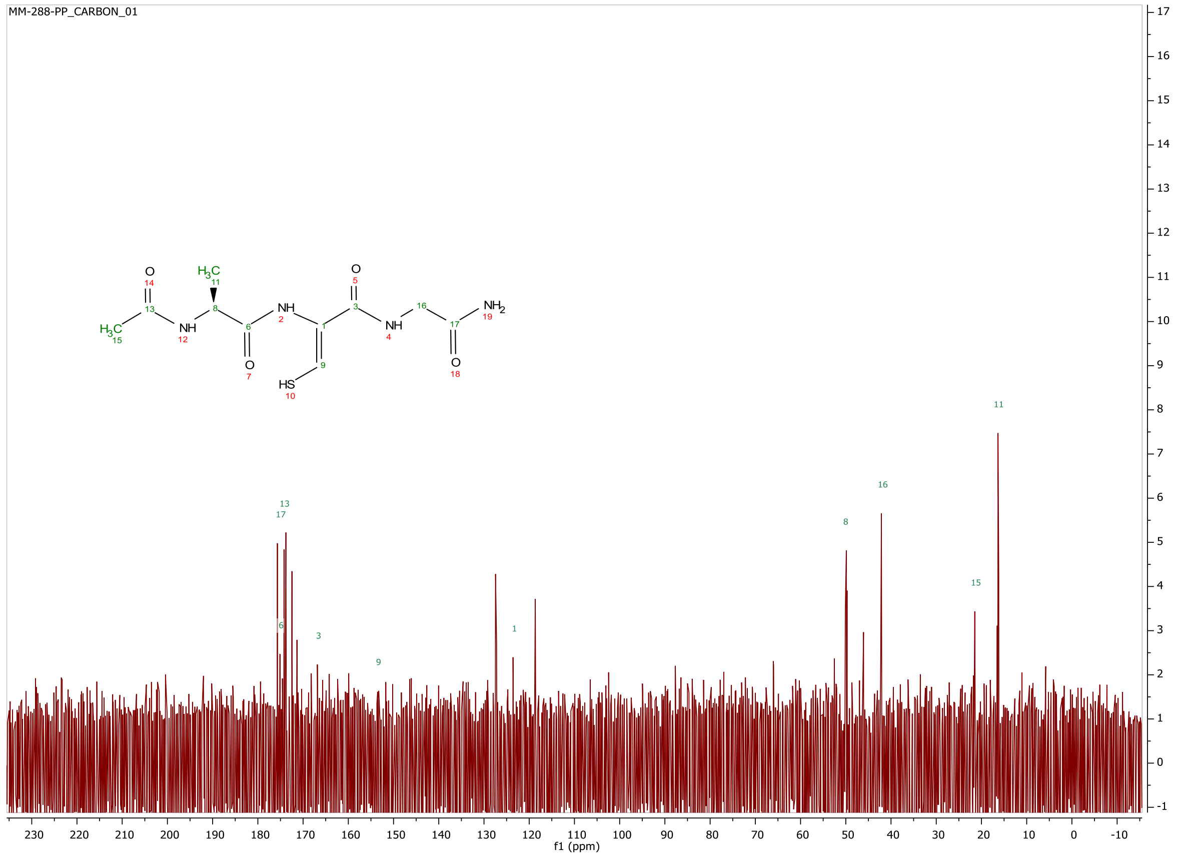

*Figure M117: ^{13}C NMR (151 MHz) spectrum of compound **22b** following photolysis in D_2O, highlighting signals corresponding to compound **(Z)-25**.*

Investigation of tripeptide isothiazolone and enethiol derivatives

pK_a studies of tripeptide based (*E*)-enethiol and (*Z*)-enethiol (25)

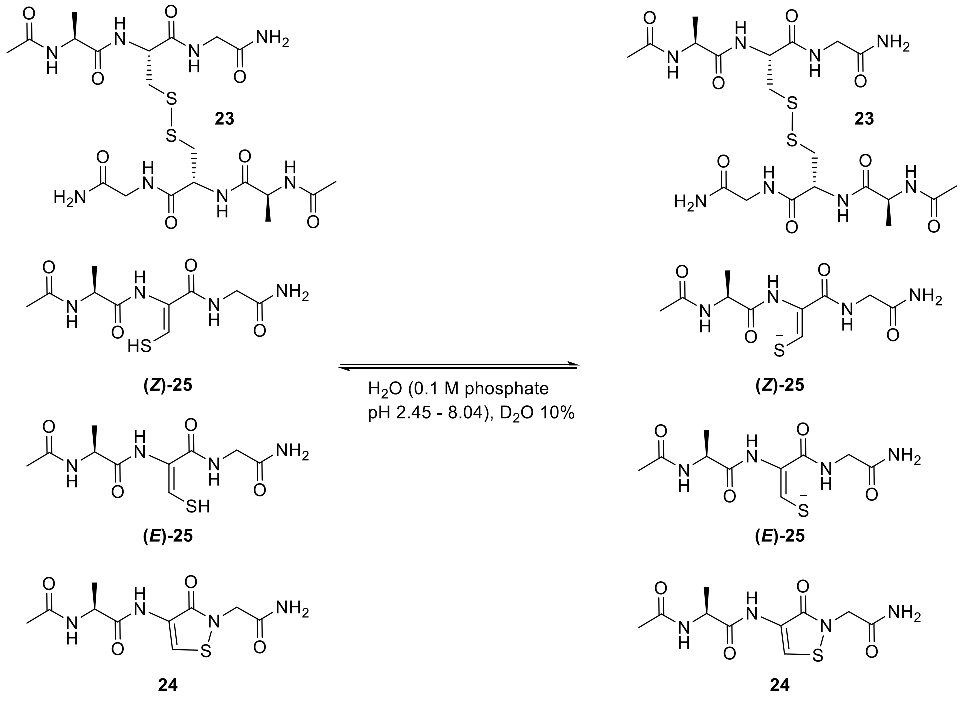

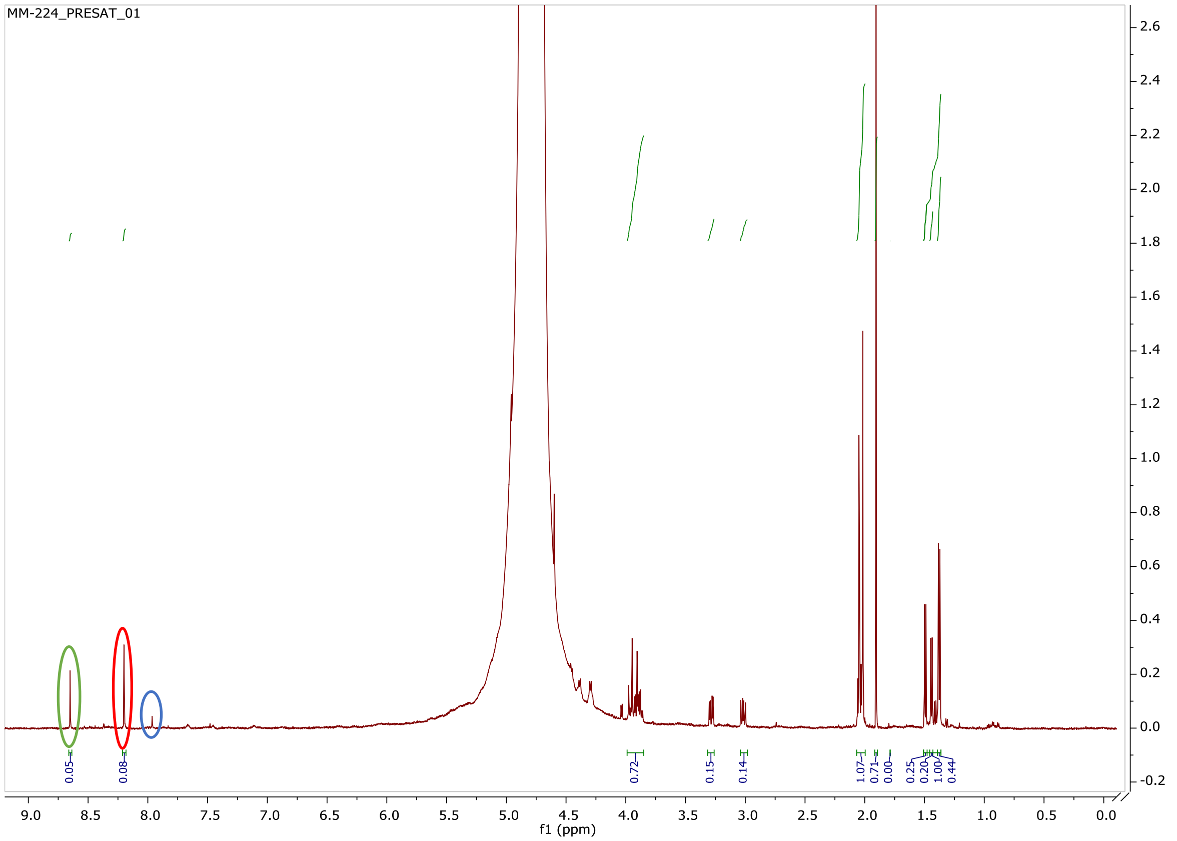

*Figure M118: Presaturation ^{1}H NMR spectrum (600 MHz) of the compound **22b** photolysis mixture in aq. phosphate buffer (0.1M, pH 7.4, 10% D_2O). The methine signals of the isothiazolone (**24**) (green), (Z)-enethiol (**(Z)-25**) (red) and (E)-enethiol (**(E)-25**) (blue) are highlighted.*

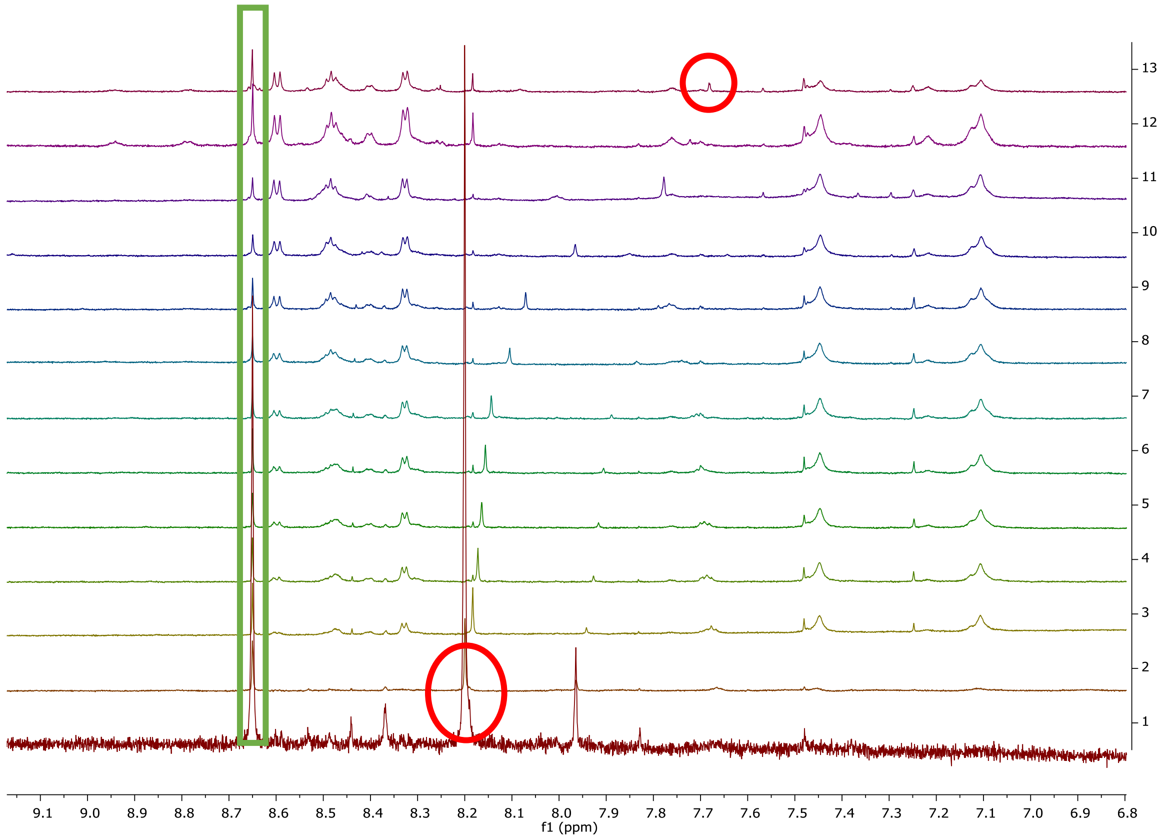

*Figure M119: Stacked presaturation ^{1}H NMR spectra across the pH range 8.04 (spectrum 1) to 2.45 (spectrum 13), highlighting the migration of the methine signal of the (Z)-enethiol (**(Z)-25**) (red) with respect to pH, relative to the reference signal of the methine of the isothiazolone (**24**) (green).*

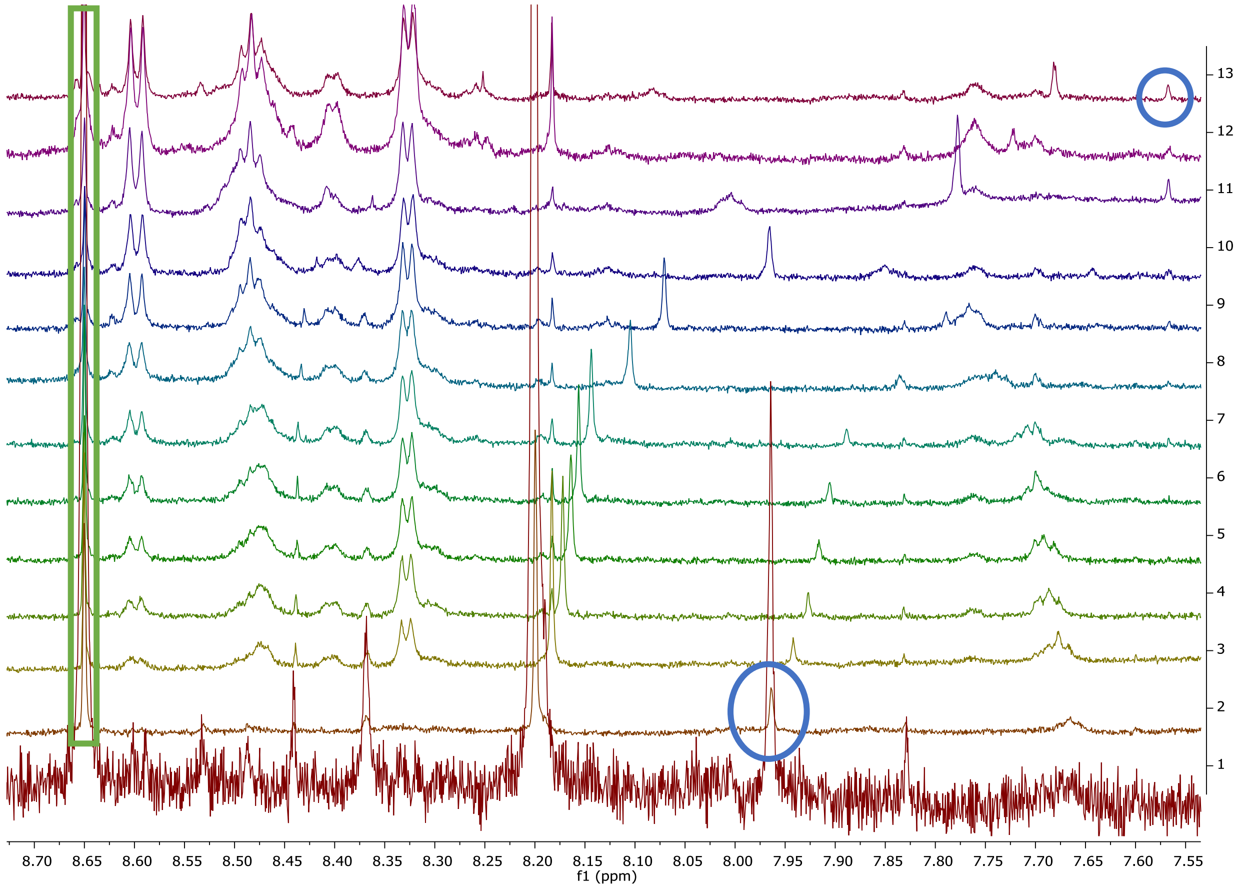

*Figure M120: Stacked presaturation ^{1}H NMR spectra across the pH range 8.04 (spectrum 1) to 2.45 (spectrum 13),highlighting the migration of the methine signal of the (E)-enethiol (**(E)-25**) (blue) with respect to pH, relative to the reference signal of the methine of the isothiazolone (**24**) (green).*

pK_a studies of Ac-AlaCysGly-NH_2 (21)

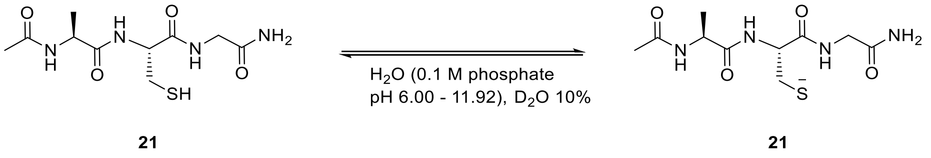

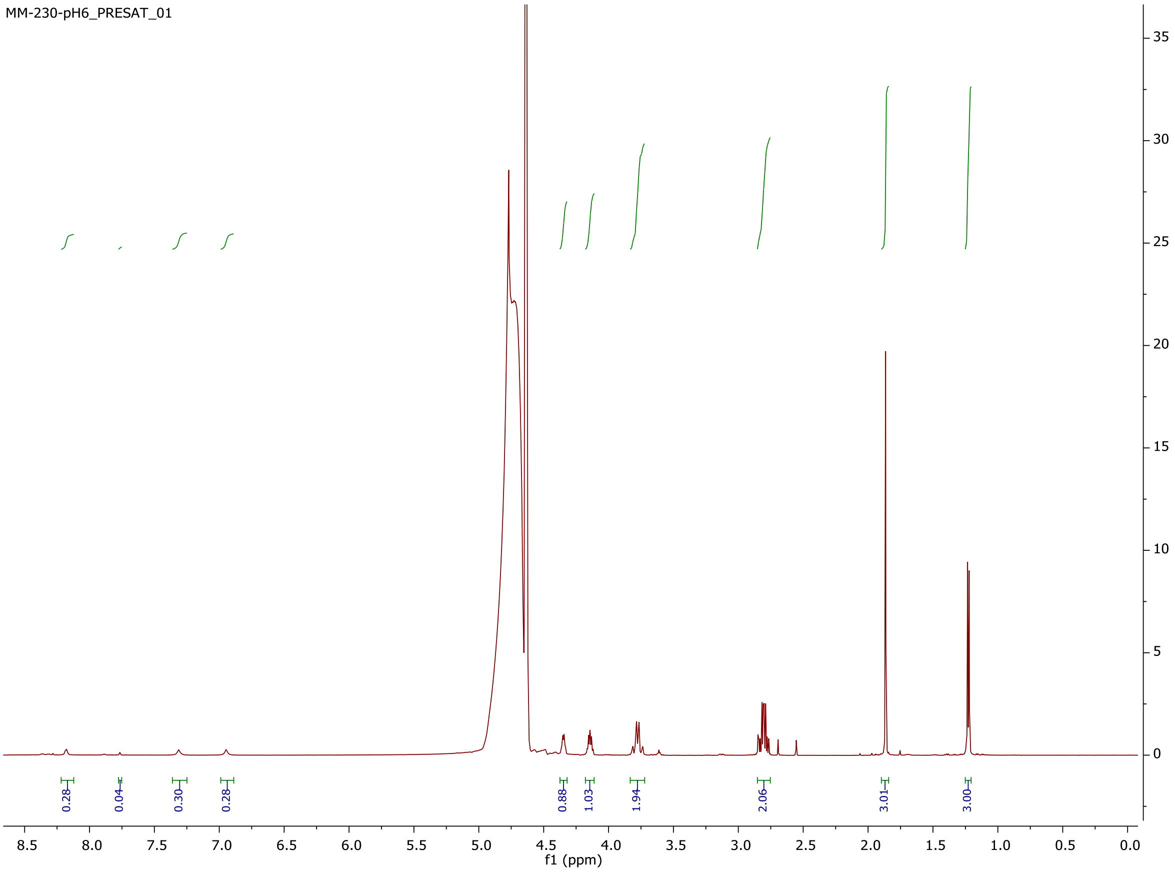

*Figure M121: Presaturation ^{1}H NMR spectrum (600 MHz) of compound **21** in aq. phosphate buffer (0.1 M, pH 6, 10% D_2O)*

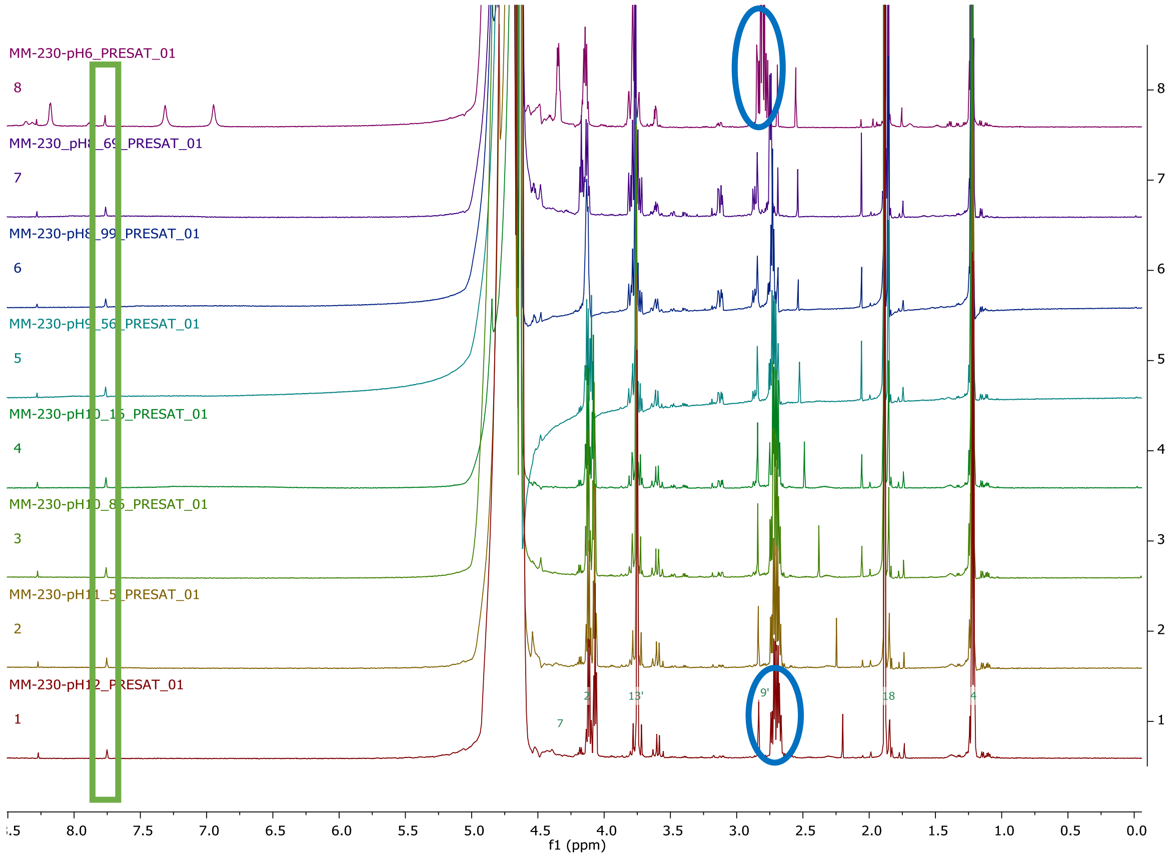

Figure M122: Stacked presaturation ^{1}H NMR spectra across the pH range 11.9 (spectrum 1) to 6.0 (spectrum 8). The diastereotopic signal of the cysteine residue is highlighted in blue and the amine reference highlighted in green.

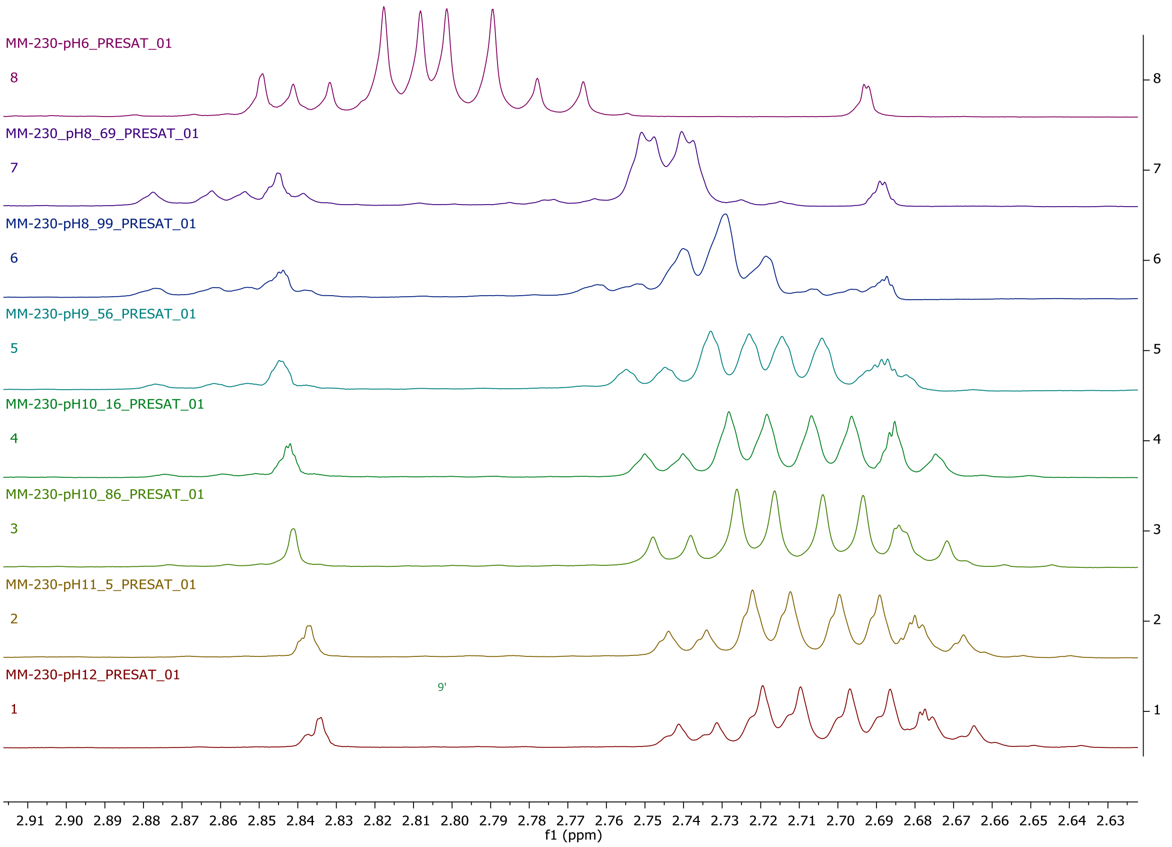

Figure M123: Stacked presaturation ^{1}H NMR spectra across the pH range 11.9 (spectrum 1) to 6.0 (spectrum 6), highlighting the migration of the diastereotopic signal with respect to pH

Studies into the interconversion of Ac-AlaCysGly-NH2 enethiol and isothiazolone derivatives

Treatment of photolysis mixture derived from phenacylsulfide 22b with tris(2-carboxyethyl)-phosphine (TCEP)

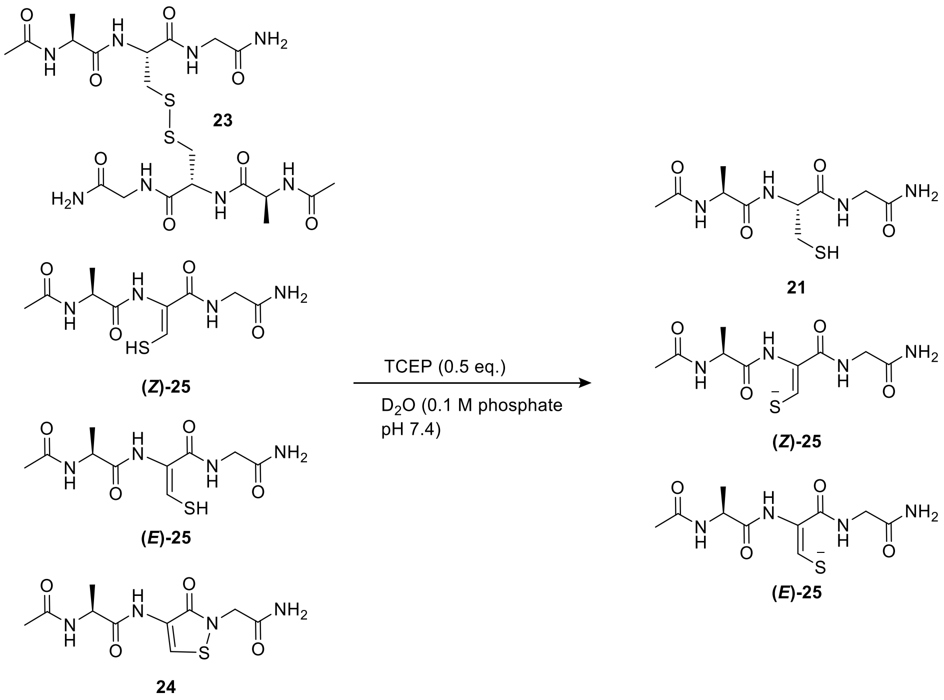

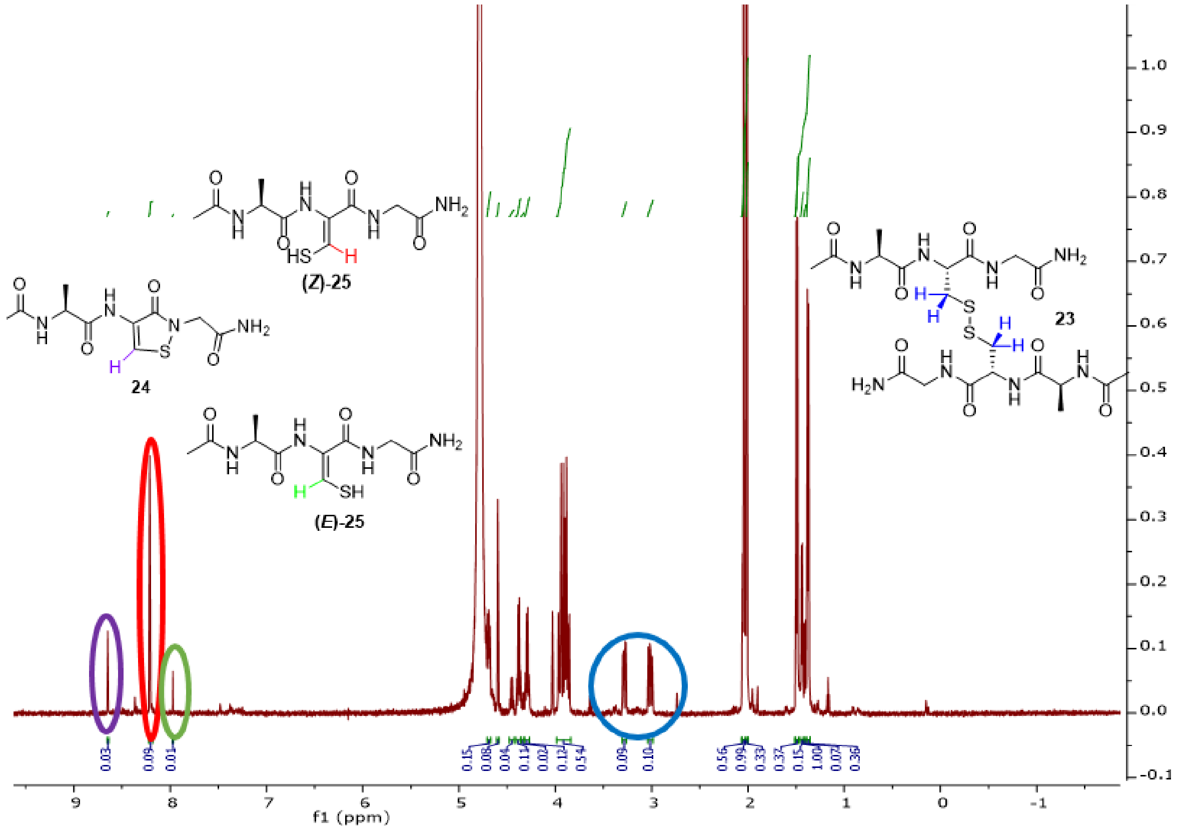

*Figure M124: ^{1}H NMR spectrum (600 MHz) of the compound **22b** photolysis mixture in D_2O. Characteristic signals of each of the reaction products are highlighted.*

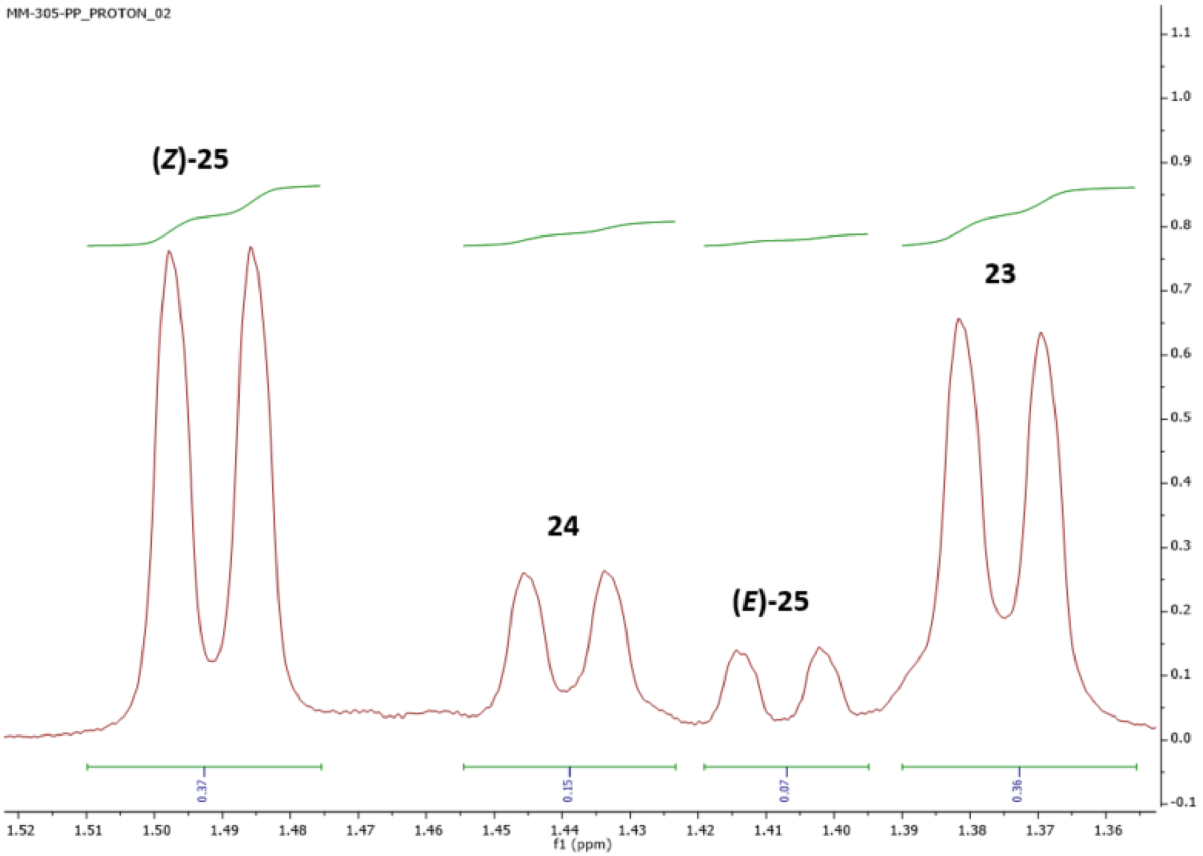

Figure M125: ^{1}H NMR spectrum (600 MHz) zoomed in the alanine region of the compound ***22b*** *photolysis mixture showing signals corresponding to reaction products, disulfide (**23**), 38%, isothiazolone (**24**), 16% , (E)-enethiol (**(E)-25**),7%, and (Z)-enethiol (**(Z)-25**), 39%.*

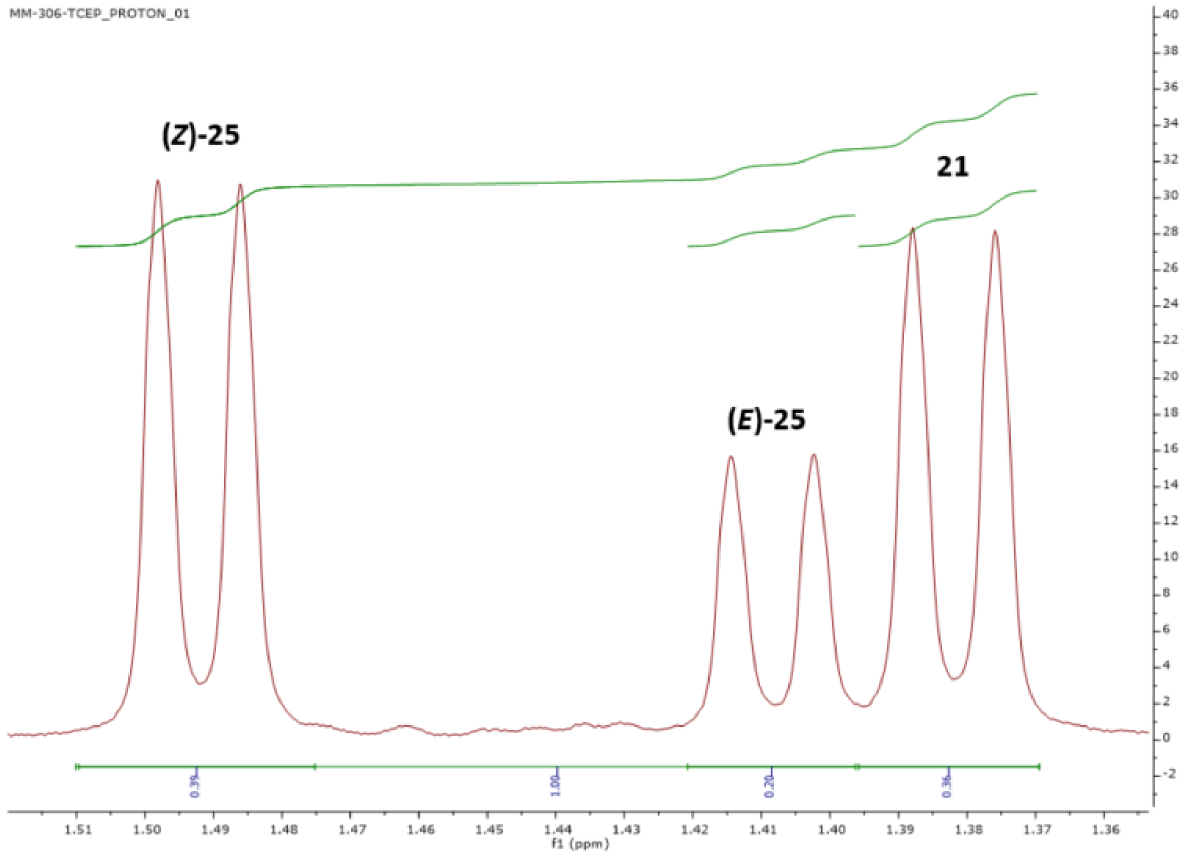

*Figure M126: ^{1}H NMR spectrum (600 MHz) zoomed in the alanine region of the compound **22b** photolysis mixture following TCEP addition showing signals corresponding to reaction products, Parent peptide (**21**), 38%, (E)-enethiol (**(E)-25**),21%, and (Z)-enethiol (**(Z)-25**), 41%.*

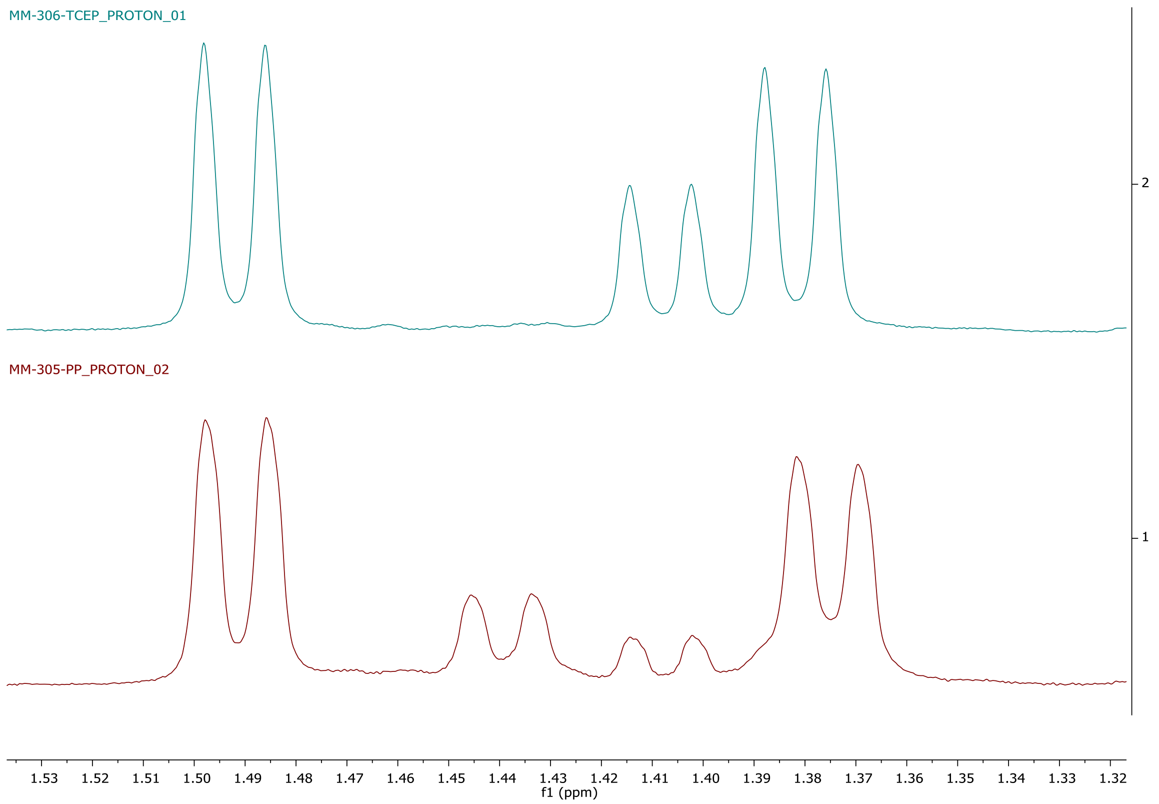

Figure M127: Stacked ^{1}H NMR spectra showing the difference in the alanine signals following treatment of the post photolysis mixture with TCEP (Blue). Highlighting a complete loss of isothiazolone and a corresponding increase in the (E)-enethiol. Proving that reduction of isothiazolone leads selectively to the formation of (E)-enethiol.

Treatment of photolysis mixture derived from the phenacylsulfide 22b with 5,5-dithiobis-(2-nitrobenzoic acid) (DTNB)

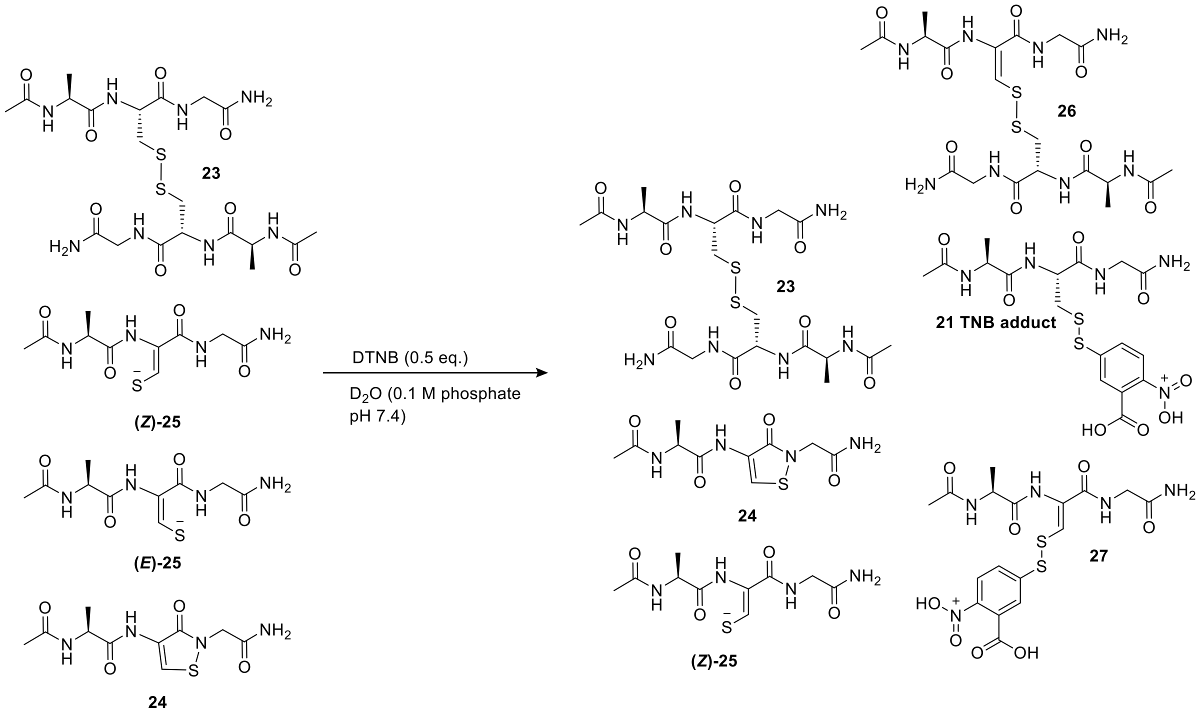

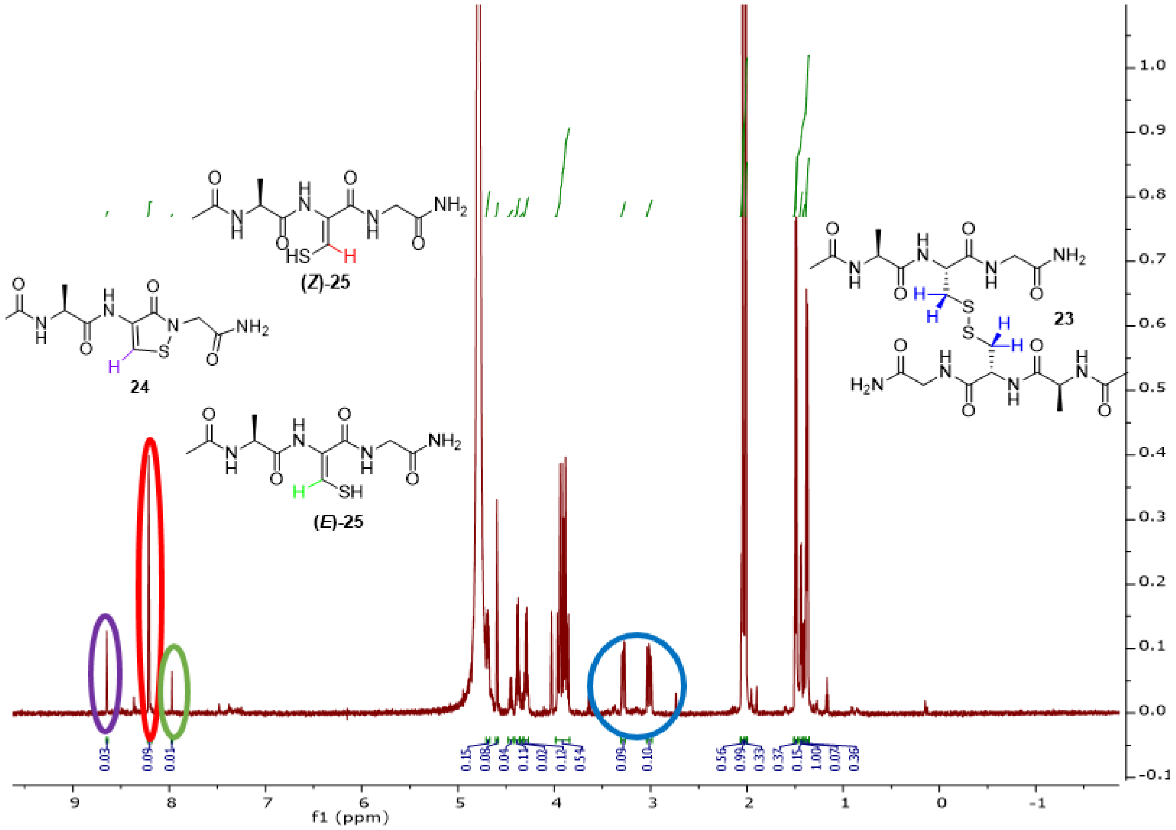

*Figure M128: ^{1}H NMR spectrum (600 MHz) of the compound **22b** photolysis mixture in D_2O. Characteristic signals of each of the reaction products are highlighted.*

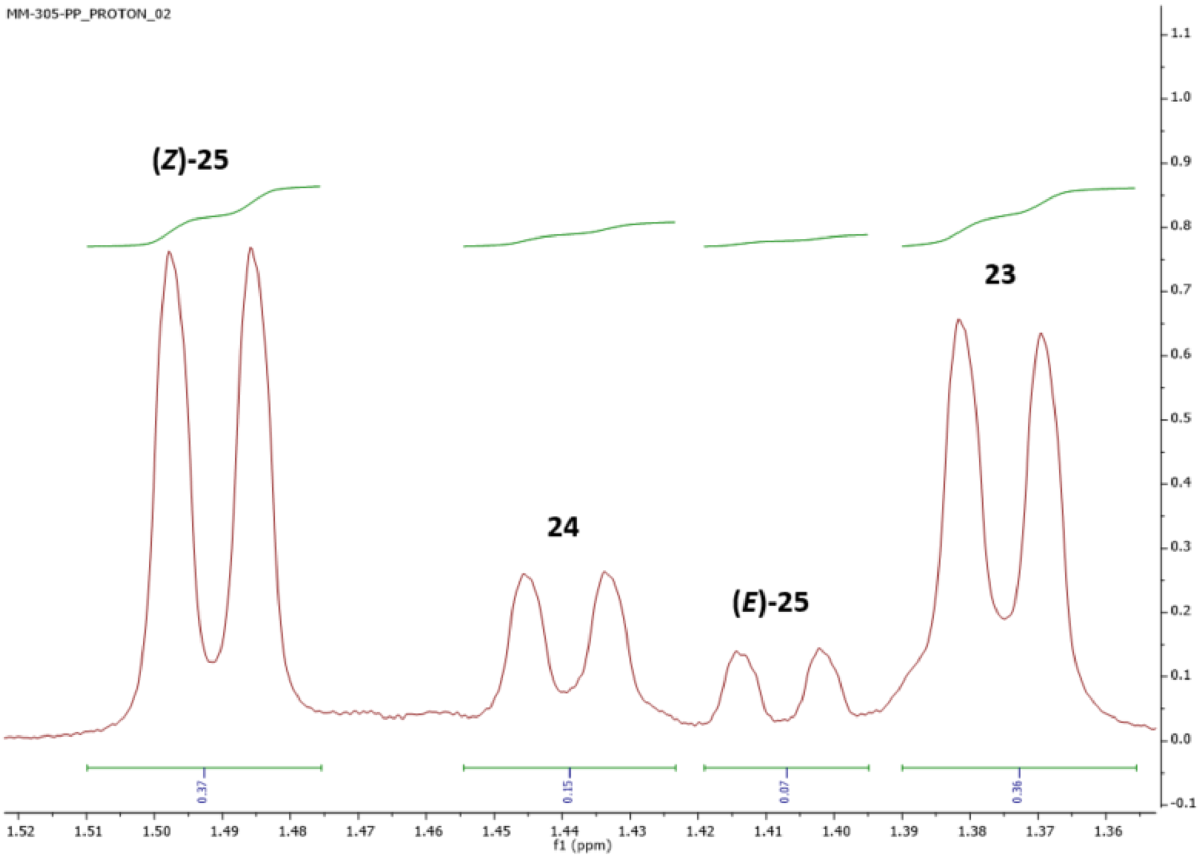

Figure M129: ^{1}H NMR spectrum (600 MHz) zoomed in the alanine region of the compound ***22b*** *photolysis mixture showing signals corresponding to reaction products, disulfide (**23**), 38%, isothiazolone (**24**), 16% , (E)-enethiol (**(E)-25**), 7%, and (Z)-enethiol (**(Z)-25**), 39%.*

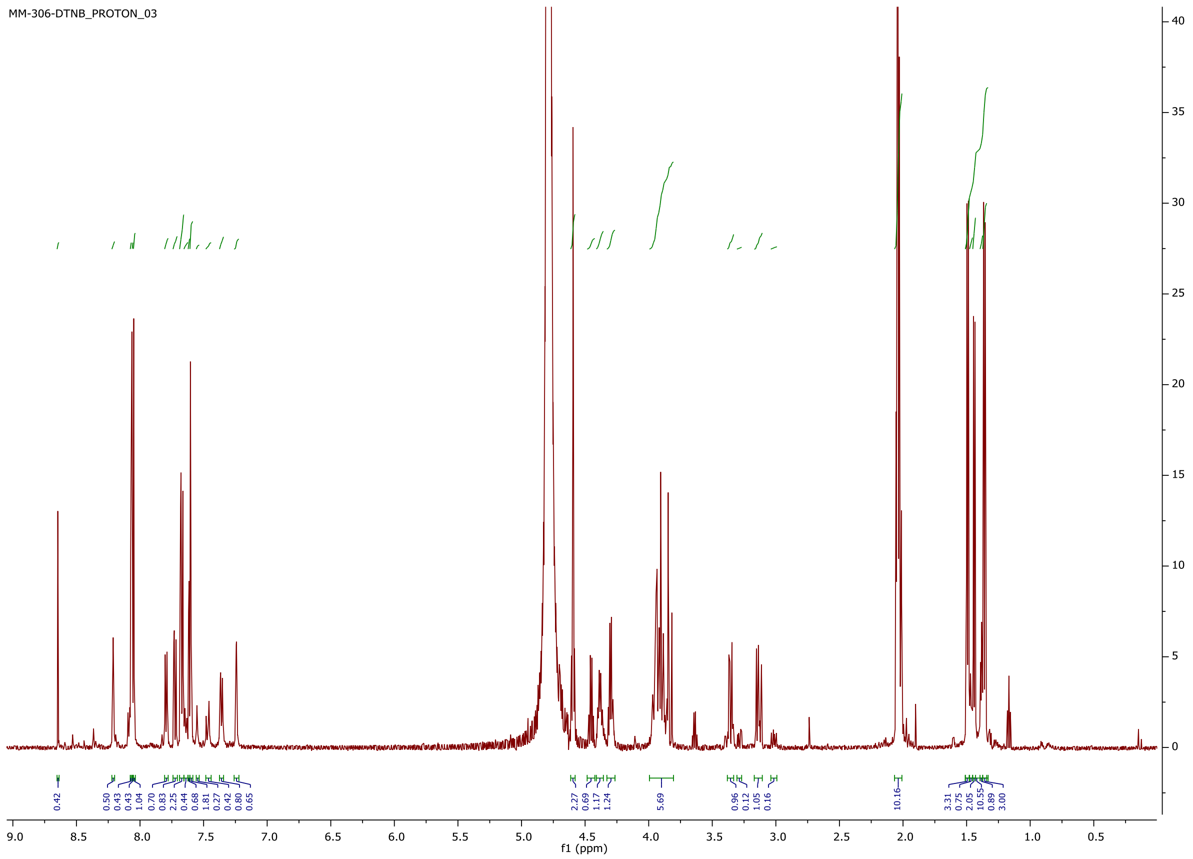

*Figure M130: ^{1}H NMR (600 MHz) spectrum of the **22b** photolysis mixture following DTNB treatment showing formation of mixed disulfide with DTNB and parent peptide in addition to mixed disulfide of (Z)-enethiol and DTNB. Note: There is a complete loss of (E)-enethiol forming isothiazolone. 9% of disulfide still remains.*

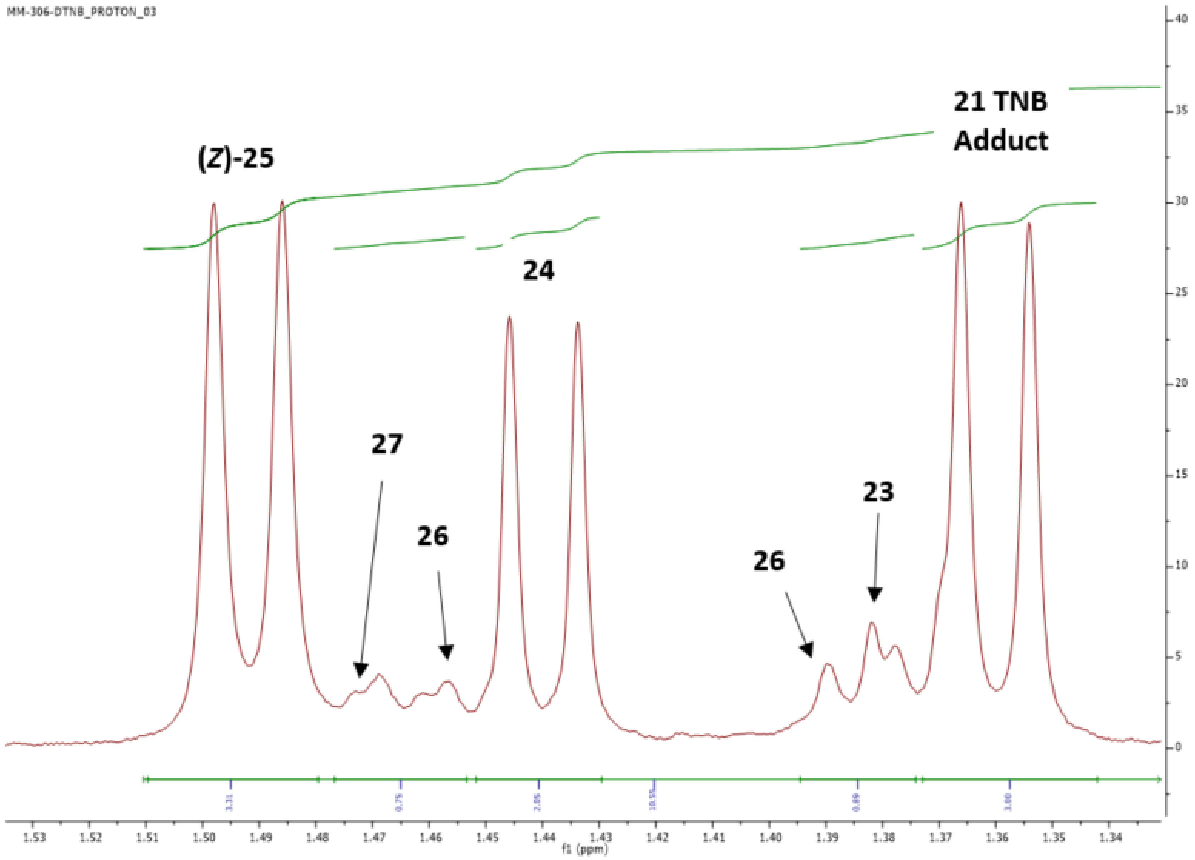

*Figure M131: ^{1}H NMR spectrum (600 MHz) zoomed in the alanine region of the compound **22b** photolysis mixture following DTNB addition showing signals corresponding to reaction products, parent peptide—TNB adduct (**21 TNB Adduct**), 29%, disulfide (**23**), 7%, and (Z)—enethiol (**(Z)-25**), 35%, isothiazolone (**24**), 21%, (Z)-enethiol—TNB adduct (**27**), 1%, thiol—(Z)—enethiol disulfide (**26**), 7%*

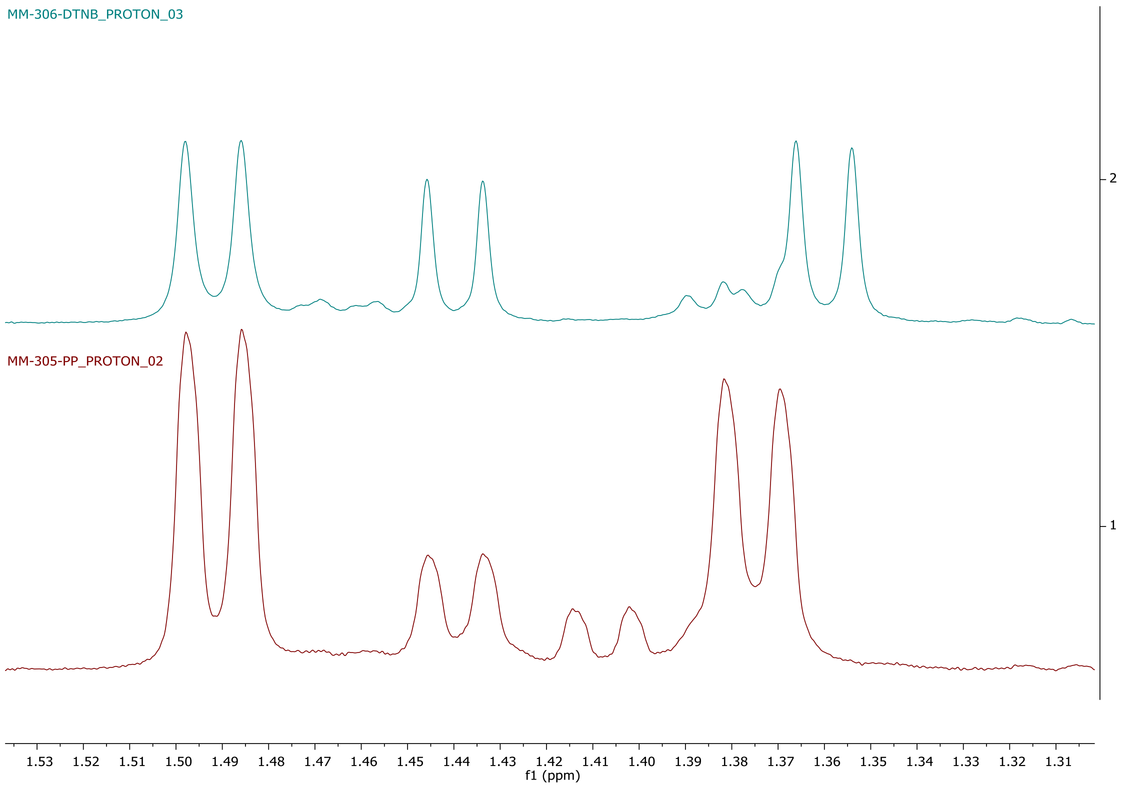

Figure M132: Stacked ^{1}H NMR spectra showing the difference in the alanine signals following treatment of the photolysis mixture with DTNB (Blue). Highlighting a complete loss of (E)-enethiol and a corresponding increase in the isothiazolone signal. Proving that oxidation of (E)-enethiol leads selectively to the formation of isothiazolone.

N. Kinetics studies

Determination of the rate of formation of phosphonium adduct 5·TCEP in aqueous mixtures

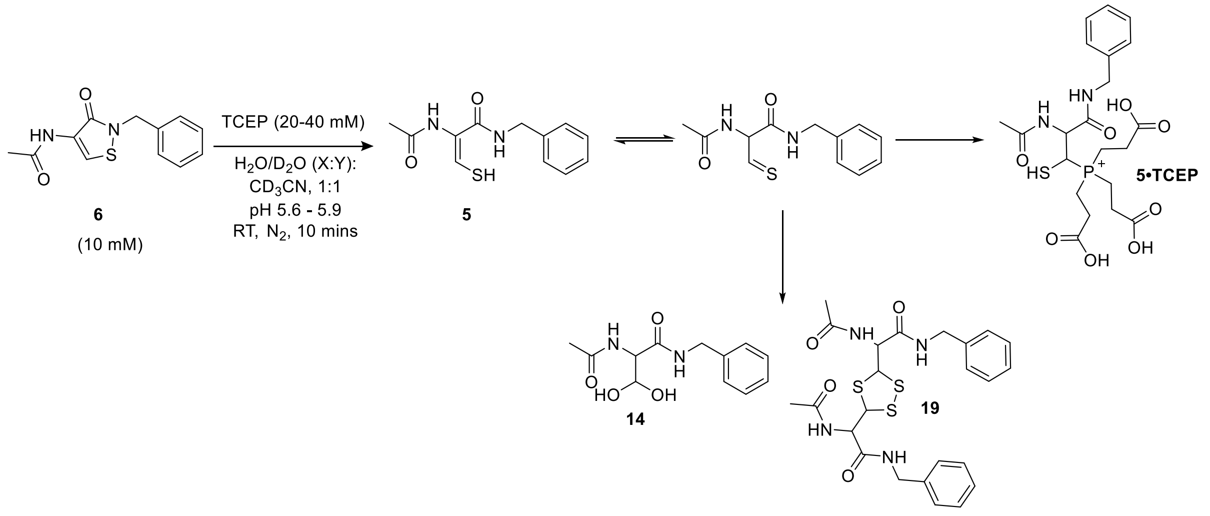

General Procedure

N-(2-benzyl-3-oxo-2,3-dihydro-1,2-thizol-4-yl) acetamide **6** (2.5 mg, 0.01 mmol) was dissolved in degassed CD_3CN (0.503 mL) and H_2O/D_2O (approximately 0.4 mL), under N_2. Following this, a 1M *tert*-butanol stock in H_2O/D_2O (10.1 μL, 0.01 mmol) was added to act as an additional internal standard. A 0.4 M TCEP stock solution in H_2O/D_2O was prepared and brought to pH 5.9 using a 2M NaOH stock solution. Each of the stock solutions were prepared with degassed solvents. The required volume of this TCEP stock solution was added to the reaction mixture bringing the final volume of the mixture to 1.006 mL, resulting in an enethiol **5** concentration of 10 mM. The reaction mixture was allowed to evolve under N_2 for 2 minutes, transferred to an NMR tube and back flushed with N_2 prior to sealing. The evolution of the reaction mixture was analysed by arrayed presaturation ^1H NMR spectroscopy for periods ranging from 1–6 hours. This reaction was repeated with initial TCEP concentrations of 40 mM (30 mM following reduction of isothiazolone **6**) and 20 mM (10 mM following reduction of isothiazolone **6**) varying the fraction of D_2O in the aqueous portion of the reaction mixture.

Note: 20 mM (10 mM following reduction of isothiazolone **6**) TCEP samples were prepared using a TCEP stock solution consisting of the following three components; 0.4 M TCEP, 0.4 M citric acid and 0.4 M NaCl in place of the standard 0.4 M TCEP stock. Citric acid was added to maintain the same acetate concentration as the 40 mM (30 mM following reduction of isothiazolone **6**) TCEP samples, as the acetates of TCEP could act as a general acid/base in the reaction. NaCl was added to maintain an ionic strength of 123 mM, the same ionic strength as the 40 mM (30 mM following reduction of isothiazolone **6**) TCEP samples.

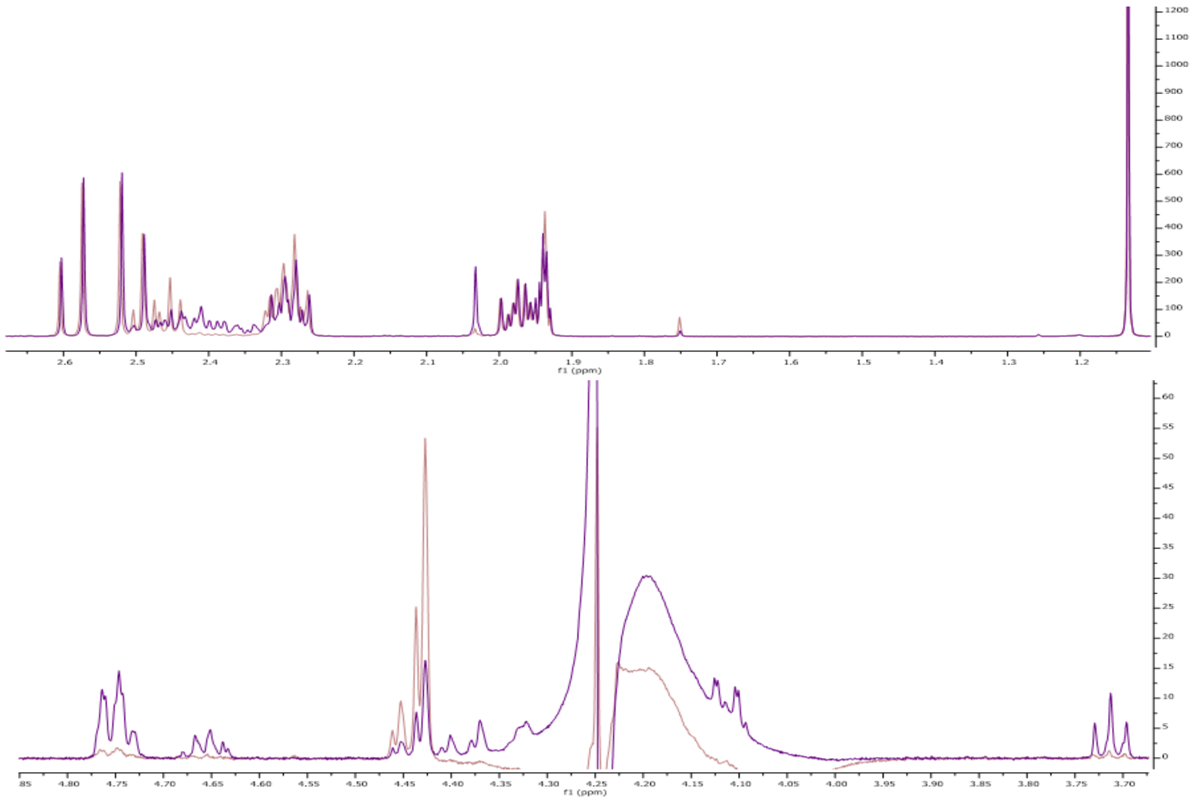

Figure N1: Superimposed presaturated ^{1}H NMR spectra (500 MHz) of the reaction mixture derived from the reduction of isothiazolone ***6*** *in the absence of buffer in a 1:1:2 mixture of $D_2O/H_2O/CD_3CN$ with an initial TCEP concentration of 20 mM (10 mM following reduction of isothiazolone* ***6****), zoomed in to the up field (top) and methine (bottom) region of the spectra. The spectrum of the initial time point at 17 minutes is coloured in red while the blue spectrum represents the final time point at 190 minutes. A marked increase in intensity is observed for the signal corresponding to the acetyl of the phosphonium adduct at 2.04 ppm at the 190 minute timepoint (top). The signals corresponding to the α-methine of the major and minor isomers of the phosphonium adduct appear at 4.75 ppm and 4.65 ppm respectively and show a marked increase over time. A corresponding decrease is observed in the signals corresponding to the benzyl methylene of enethiol* ***5*** *at 4.45 ppm. The β-methine signals of the major and minor isomers of the phosphonium adduct appear at 4.10 and 3.72 ppm, respectively (bottom).*

*Table N1: The time range of analysis, conversion range over the analysis period and initial rates of phosphonium adduct formation for each sample. a) Concentration of TCEP following reduction of isothiazolone **6***

Sample	[TCEP][a] (mM)	Time range of analysis (min)	Total Conversion range (%)	Initial rate of phosphonium adduct formation (mM/s)	Conversion range at initial rate measurement (%)
40 mM TCEP (H_2O)	30	10–69	12.9–49.7	1.11×10^{-3}	12.9–49.7
40 mM TCEP (D_2O)	30	18–189	3.9–24.8	2.15×10^{-4}	5.5–11.8
20 mM TCEP (D_2O)	10	16–157	4–22.8	2.46×10^{-4}	4.5–11.7
20 mM TCEP (D_2O/H_2O, 9:1)	10	26–345	11.6–59.1	3.61×10^{-4}	11.7–29.3
20 mM TCEP (D_2O/H_2O, 3:1)	10	19–332	11.9–59.6	4.24×10^{-4}	12.7–27.2
20 mM TCEP H_2O/D_2O (1:1)	10	17–190	7.5–49.0	6.04×10^{-4}	12.9–29.14
20 mM TCEP (H_2O)	10	10–129	7.4–39.7	6.46×10^{-4}	7.4–30.6

Both the 20 mM TCEP (D_2O) and the 40 mM TCEP (D_2O) samples show near identical rates independent of TCEP concentration. Therefore, it can be concluded that the observed rate is the rate of C-deuteronation of the enethiol **5** to form the putative thioaldehyde.

The derived first-order rate constant (k) for the formation of the putative thioaldehyde is:

$$\text{Rate} = k\,[\text{enethiol}]$$

$$2.46\text{x}10^{-4}\ \text{mM/s} = k\ (10\ \text{mM})$$

$$(2.46\text{x}10^{-4}\ \text{mM/s})/(10\ \text{mM}) = k$$

$$k = \mathbf{2.46 \times 10^{-5}\ s^{-1}}$$

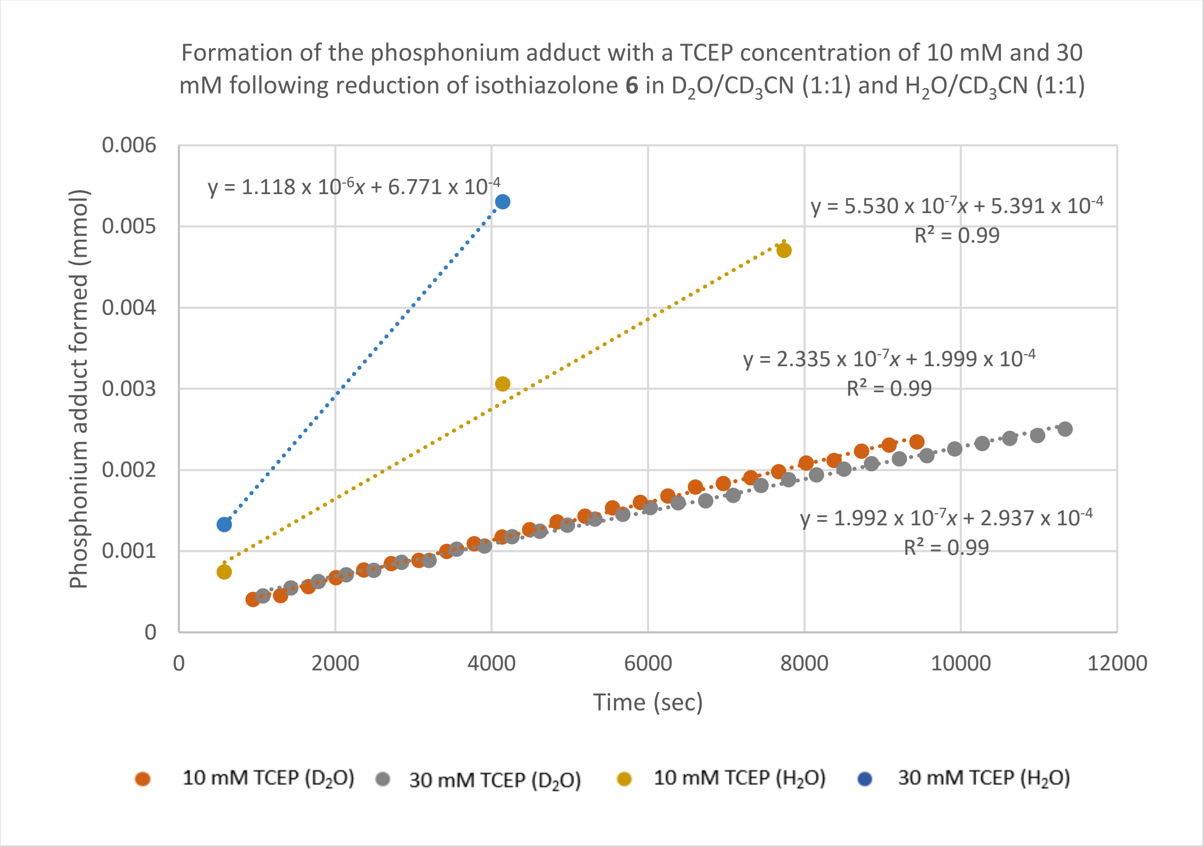

Figure N2: Formation of phosphonium adduct ***5·TCEP****, by trapping thioaldehyde generated via C-protonation/deuteronation of enethiol* ***5****, with varying concentrations of TCEP in either* D_2O/CD_3CN *(1:1) or* H_2O/CD_3CN *(1:1) at approximately pH 5.8. Plotted is the formation of the phosphonium adduct (mmol) over the initial 9–188 minutes of the reaction. Formation was determined by integration of the acetyl signal of the adduct at 2.03 ppm which was normalised to the citric acid methylene signal at 2.59 ppm. In the case of the 30 mM TCEP samples, the TCEP oxide signal at 1.97 ppm was used as a reference.*

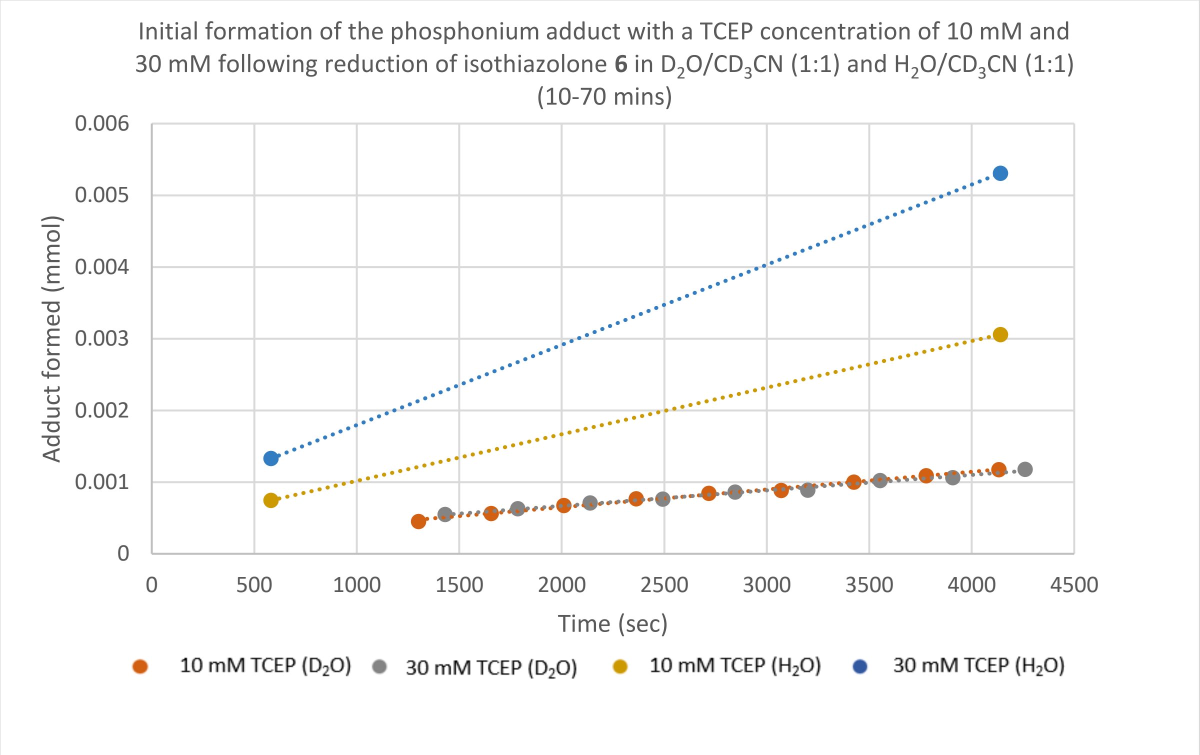

Sample	Equation of trend line
10 mM TCEP (D_2O)	$2.417 \times 10^{-7}x + 1.550 \times 10^{-4}$
10 mM TCEP (H_2O)	$6.502 \times 10^{-7}x + 3.669 \times 10^{-4}$
30 mM TCEP (D_2O)	$2.161 \times 10^{-7}x + 2.362 \times 10^{-4}$
30 mM TCEP (H_2O)	$1.118 \times 10^{-6}x + 6.770 \times 10^{-4}$

*Figure N3: Formation of phosphonium adduct **5·TCEP**, by trapping thioaldehyde generated via C-protonation/deuteronation of enethiol **5**, with varying concentrations of TCEP in either D_2O/CD_3CN (1:1) or H_2O/CD_3CN (1:1) at approximately pH 5.8. Plotted is the formation of the phosphonium adduct (mmol) over the initial 9–69 minutes of the reaction covering a conversion percentage range of 7.0–49.7%. Formation was determined by integration of the acetyl signal of the adduct at 2.03 ppm which was normalised to the citric acid methylene signal at 2.59 ppm. In the case of the 30 mM TCEP samples, the TCEP oxide signal at 1.97 ppm was used as a reference.*

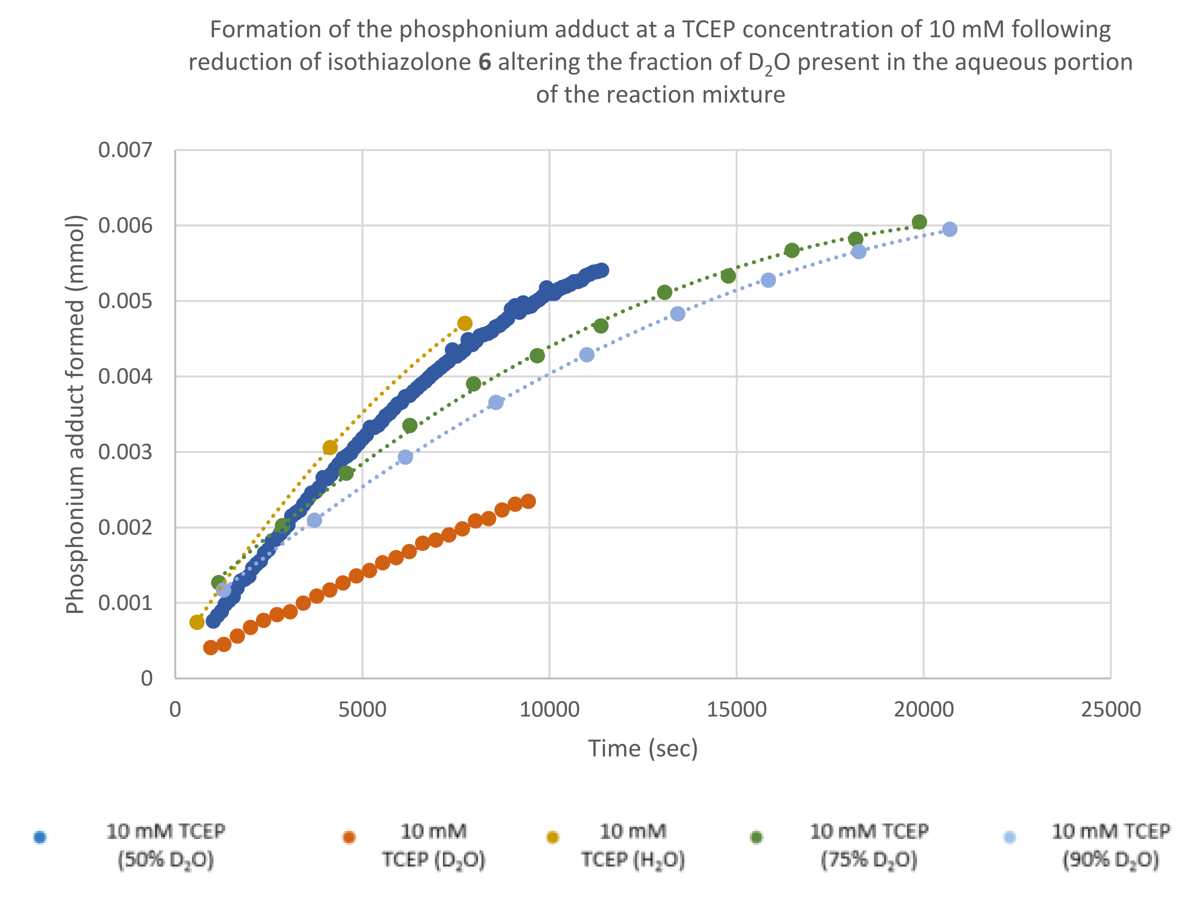

*Figure N4: Formation of phosphonium adduct **5·TCEP**, varying the fraction of D_2O present in the aqueous portion of the reaction mixture at approximately pH 5.9 with a TCEP concentration of 10 mM following reduction of isothiazolone **6**. Plotted is the formation of the phosphonium adduct (mmol) over the analysis period of each reaction. Formation was determined from the integration of the acetyl signal of the adduct at 2.03 ppm which was normalised to the citric acid methylene signal at 2.59 ppm.*

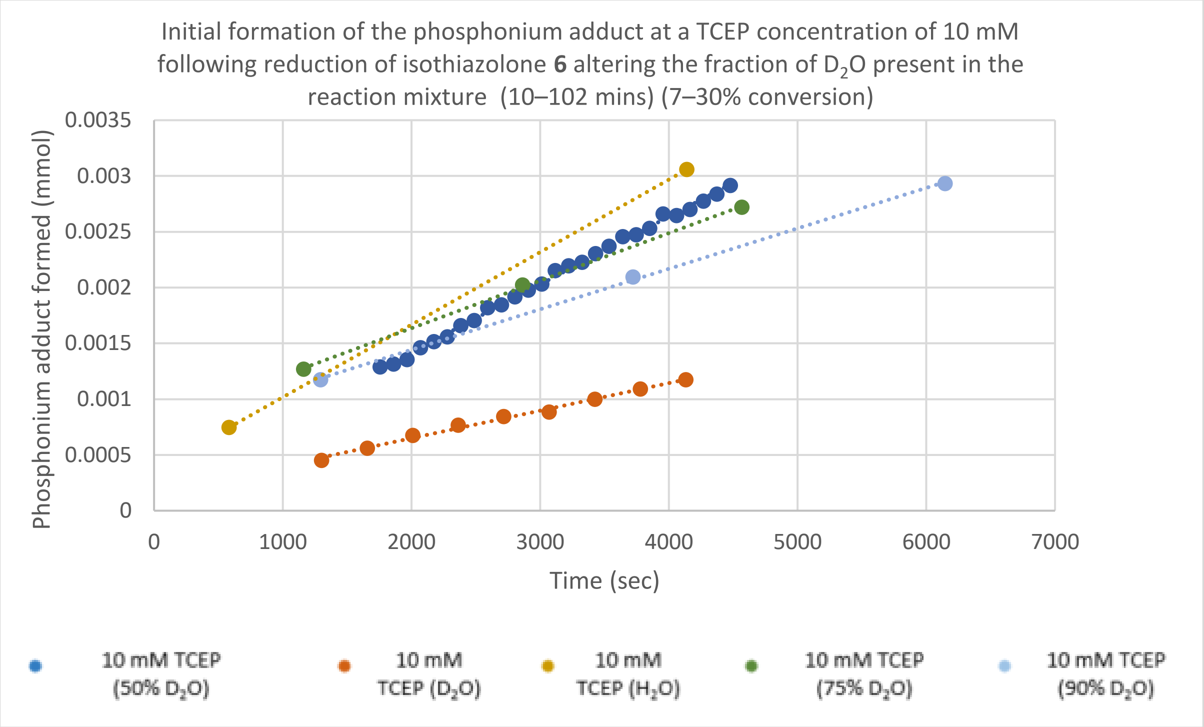

Sample	Equation of trend line
10 mM (D_2O)	$2.417 \times 10^{-7}x + 1.550 \times 10^{-4}$
10 mM (90% D_2O)	$3.627 \times 10^{-7}x + 7.172 \times 10^{-4}$
10 mM (75% D_2O)	$4.262 \times 10^{-7}x + 7.832 \times 10^{-4}$
10 mM (50% D_2O)	$6.076 \times 10^{-7}x + 2.055 \times 10^{-4}$
10 mM (H_2O)	$6.502 \times 10^{-7}x + 3.669 \times 10^{-4}$

Figure N5: Formation of phosphonium adduct ***5·TCEP****, varying the fraction of D_2O present in the aqueous portion of the reaction mixture at approximately pH 5.9 with a TCEP concentration of 10 mM following reduction of isothiazolone* ***6****. Plotted is the formation of the phosphonium adduct (mmol) over the initial 10-102 minutes of the reaction covering a conversion percentage range of 7–30%. Formation was determined by integration of the acetyl signal of the adduct at 2.03 ppm which was normalised to the citric acid methylene signal at 2.59 ppm*

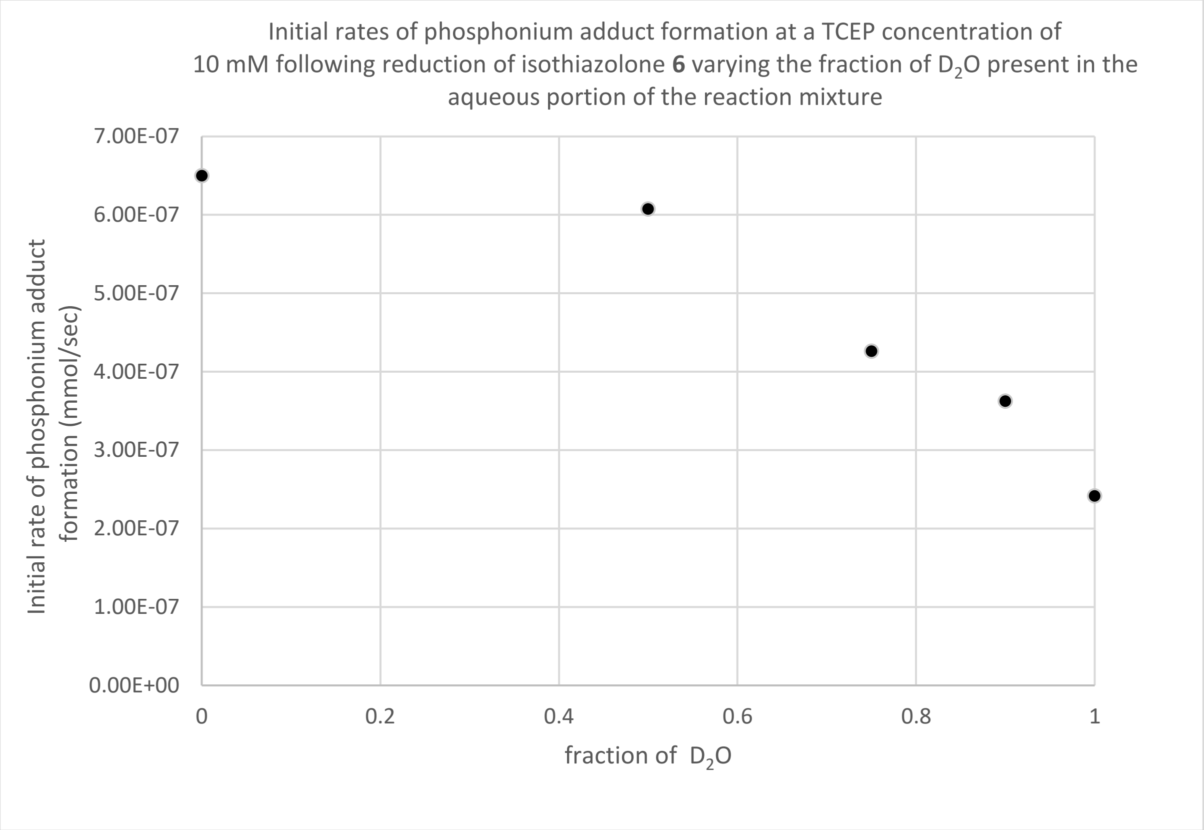

Figure N6: Initial rates of formation of the phosphonium adduct ***5·TCEP*** *with changing D_2O fraction at a TCEP concentration of 10 mM following reduction of isothiazolone* ***6****.*

Determination of the rate of formation of *gem*-diol 14

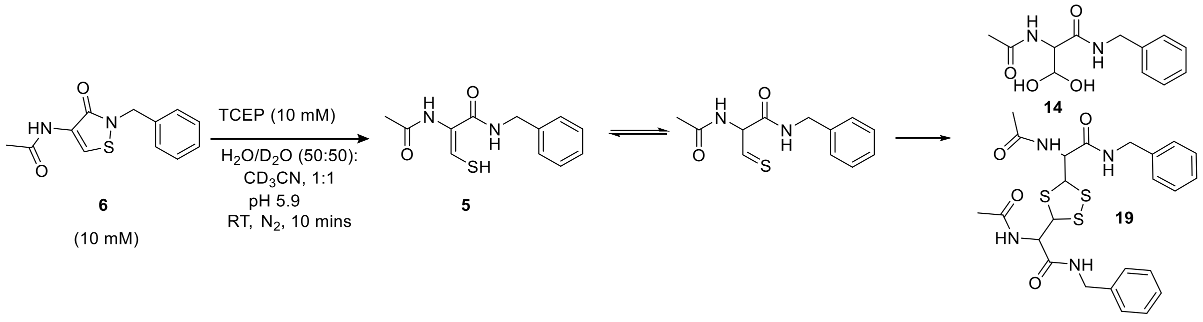

General Procedure

N-(2-benzyl-3-oxo-2,3-dihydro-1,2-thizol-4-yl) acetamide **6** (2.5 mg, 0.01 mmol) was dissolved in degassed CD_3CN (0.503 mL) and H_2O/D_2O (approximately 0.4 mL), under N_2. Following this, 1M *tert*-butanol stock in H_2O/D_2O (10.1 µL, 0.01 mmol) was added to act as an additional internal standard. A TCEP stock solution consisting of the following three components; 0.4 M TCEP, 1.19 M citric acid and 1.19M NaCl in H_2O/D_2O was prepared and brought to pH 5.9 using a 2M NaOH stock solution. Each of the stock solutions were prepared with degassed solvents. Citric acid and NaCl were used as additives in order to maintain the same ionic strength as the prior samples. 25.5 µL of the TCEP/citric acid/NaCl stock solution was then added to the reaction mixture bringing the final volume of the solution to 1.006 mL, resulting in an enethiol **5** concentration of 10 mM. The reaction mixture was then allowed to evolve under N_2 for 2 minutes, transferred to an NMR tube and back flushed with N_2 prior to sealing. The evolution of the reaction mixture was then analysed by means of presaturation ^{1}H NMR spectroscopy periodically.

The total time range of analysis was 22–4320 minutes. Over this period, a conversion percentage range from 0–7% was observed.

The calculated initial rate of diol formation was **2.74 x 10^{-6} mM s^{-1}**

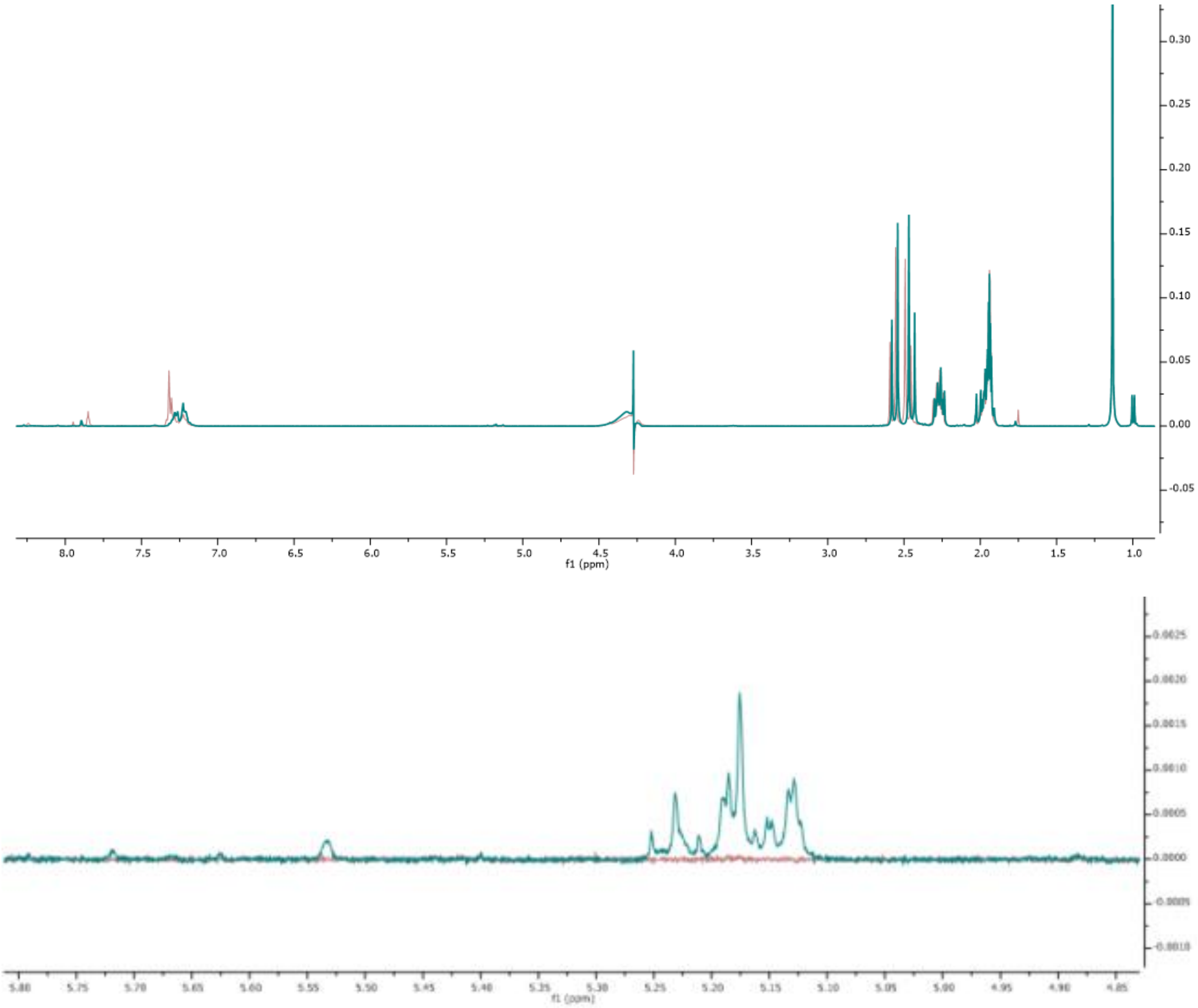

*Figure N7: Superimposed presaturated ^{1}H NMR spectra (400 MHz) of the reaction mixture derived from the reduction of isothiazolone **6** in the absence of buffer with a pH meter reading of 5.9 in a 1:1:2 mixture of $D_2O/H_2O/CD_3CN$. The sample has an initial TCEP concentration of 10 mM (0 mM following reduction of isothiazolone **6**). The spectra shown are zoomed in to the up field (top) and methine (bottom) regions of the spectra. The spectrum of the initial time point at 22 minutes is coloured in red while the blue spectrum represents the final time point at 72 hours. In this case no phosphonium adduct is observed as the TCEP is fully consumed following reduction of the isothiazolone, hydrolysis of the thioaldehyde has occurred to yield gem-diol which is present as a mixture of isotopomers owing to deuterium incorporation at the α-methine of the species. The β-methine of the gem-diol is observed at 5.18 ppm. The remainder of the signals in the region correspond to the β-methine of dithioacetal derivatives.*

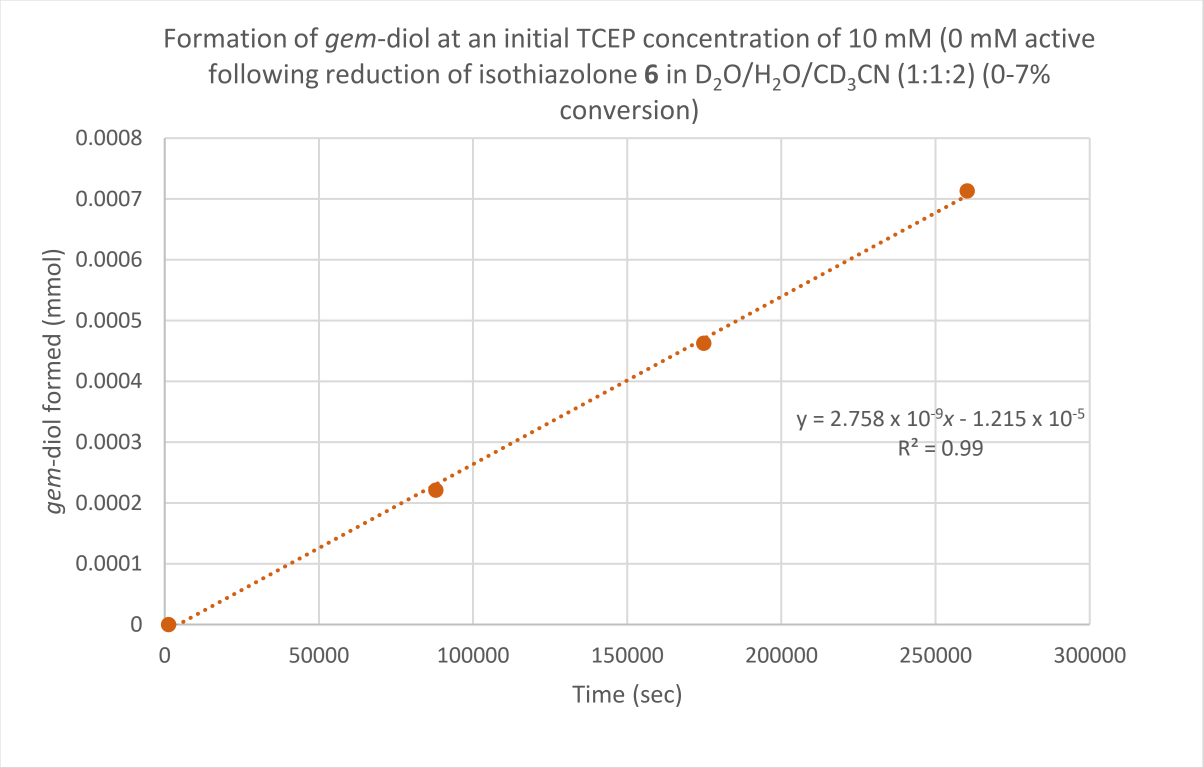

*Figure N8: Formation of the gem-diol (mmol) at approximately pH 5.9 in a 1:1:2 mixture of $D_2O/H_2O/CD_3CN$ with an initial TCEP concentration of 10 mM (0 mM following reduction of isothiazolone **6**). Plotted is the formation of the species over the initial 72 hours of the reaction covering a conversion percentage range of 0–7%. Formation was determined by integration of the β-methine signal of the species at 5.18 ppm, which was normalised to the citric acid methylene signal at 2.59 ppm.*

O. IR spectroscopic studies

FTIR spectra were recorded using a Perkin-Elmer Spectrum 100 FTIR spectrometer with a diamond cell background. 32 scans were collected for each sample from 4000 to 300 cm^{-1} at 2 cm^{-1} resolution.

Data Analysis

All spectral manipulations were performed using OriginPro software. The protein/peptide IR spectrum was background subtracted, reduced to the amide I region (1700 to 1600 cm^{-1}) and baseline corrected. Second derivative spectra were obtained from the baseline–corrected spectra using a third–degree polynomial function with a 20-point Savitsky–Golay smoothing function. Peak positions corresponding to characteristic secondary structural elements in the amide I region were assigned based on literature values[159], and these assignments were used to guide curve fitting of the baseline corrected spectra using a Gaussian model in OriginPro. Secondary structure content was estimated by calculating the percentage area of each fitted band relative to the total deconvoluted amide I band area.

Sample preparation – BSA

Na_2HPO_4 (141.96 mg, 1 mmol) was dissolved in D_2O (10 mL), followed by adjusting the pD of the solution to 7.8 (pH meter reading 7.4) using D_3PO_4 stock solution (0.1 mL con. H_3PO_4 in 0.9 mL D_2O). To ensure complete exchange of residual proton, the solution was freeze-dried and the resulting residue redissolved in D_2O (10 mL). The freeze-drying/redissolution cycle was repeated once more. The final residue was dissolved in D_2O (10 mL), and the pD of the resulting buffer was confirmed to be 7.8 (pH meter reading 7.4).

Guanidinium hydrochloride (0.72 g, 7.5 mmol) was dissolved in D_2O (2.5 mL) and the solution incubated for 24 hrs at rt to allow H/D exchange. The sample was then dried under vacuum, and the H/D exchange cycle (redissolution in D_2O (2.5 mL); incubation (24 hrs, rt); drying under vacuum) was repeated to ensure complete deuteration. The final residue was then dissolved in D_2O (2.5 mL, 0.1 M phosphate, pD 7.8) to make deuterated guanidinium hydrochloride, [D_5-Gu]. DCl (3 M) stock solution[140].

To validate the use of IR spectroscopy in assessing protein secondary structure, native and denatured BSA samples were prepared for comparison. BSA (50 mg) was dissolved in D_2O (1.0 mL, 0.1 M phosphate, pD 7.8) and incubated for 48 hrs at rt to make 5% (w/v) stock solution of native BSA (solution A). Separately, BSA (50 mg) was dissolved in the 3 M [D_5-Gu]. DCl stock solution (1 mL) and incubated for 48 hrs at rt to make 5% (w/v) solution of denatured BSA (solution B). Solution A and B were directly analysed by IR spectroscopy.

Solution A

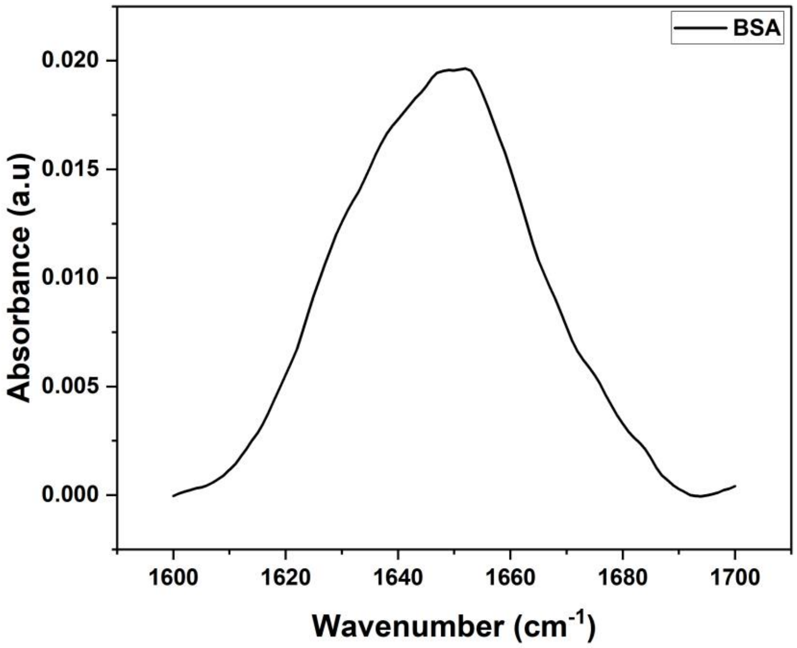

Figure O1: FTIR spectra of native BSA in D_2O (0.1 M phosphate, pD 7.8) with D_2O (0.1 M phosphate, pD 7.8) subtracted and baseline corrected.

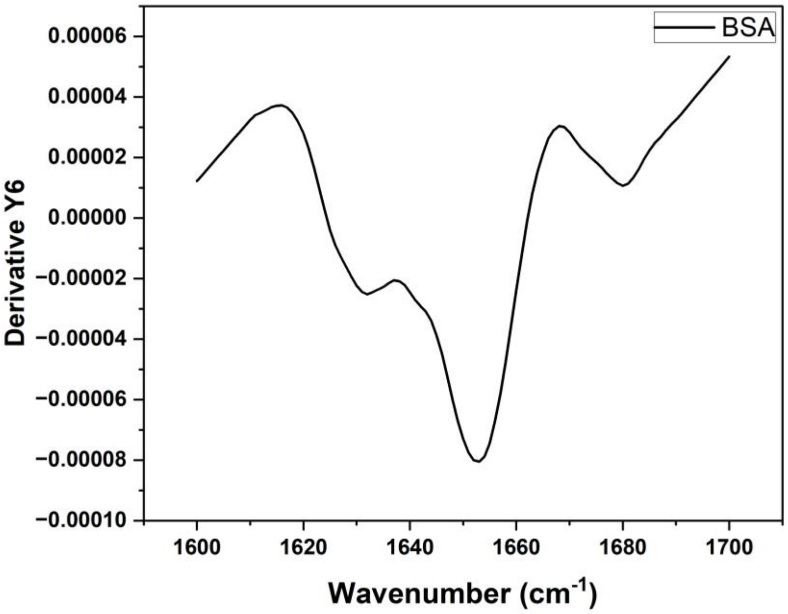

Figure O2: Second derivative amide I spectra of BSA in D_2O (0.1 M phosphate, pD 7.8) showing peaks at 1680, 1654 and 1633 cm^{-1} corresponding to β-turns, α-helix and short-segment chains connecting α-helical segments[159].

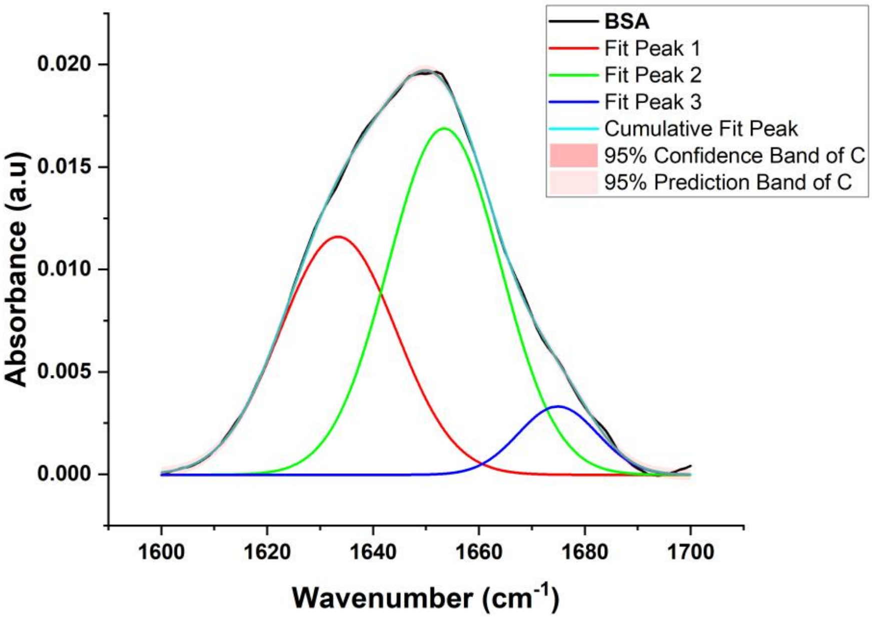

Figure O3: Curve-fit bands of the deconvoluted spectra of BSA in D_2O (0.1 M phosphate, pD 7.8) showing three peaks. Peak 1 represents the short-segment chains connecting α-helical segments, peak 2 represents α-helix and peak 3 represents β-turns.

Solution B

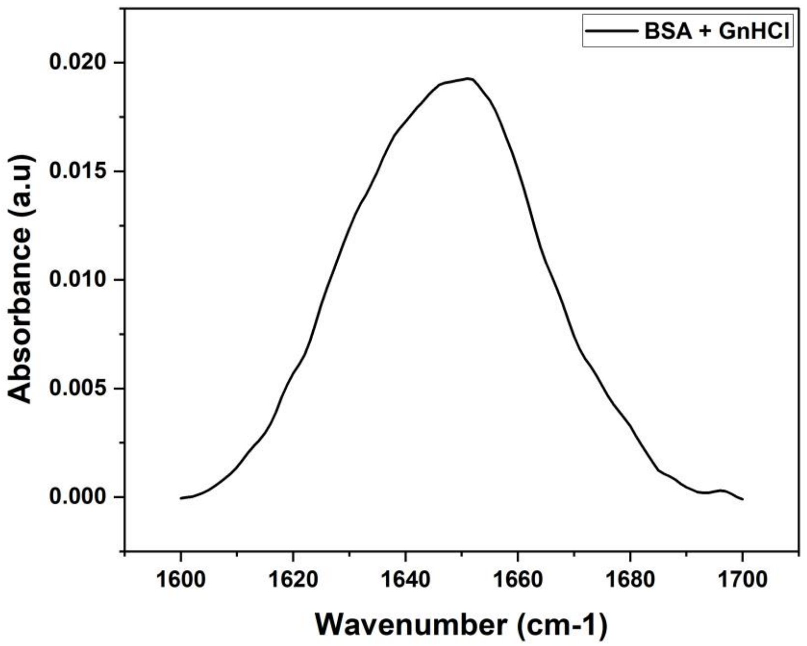

Figure O4: FTIR spectra of BSA in 3 M [D_5-Gu]. DCl with 3 M [D_5-Gu]. DCl subtracted and baseline corrected.

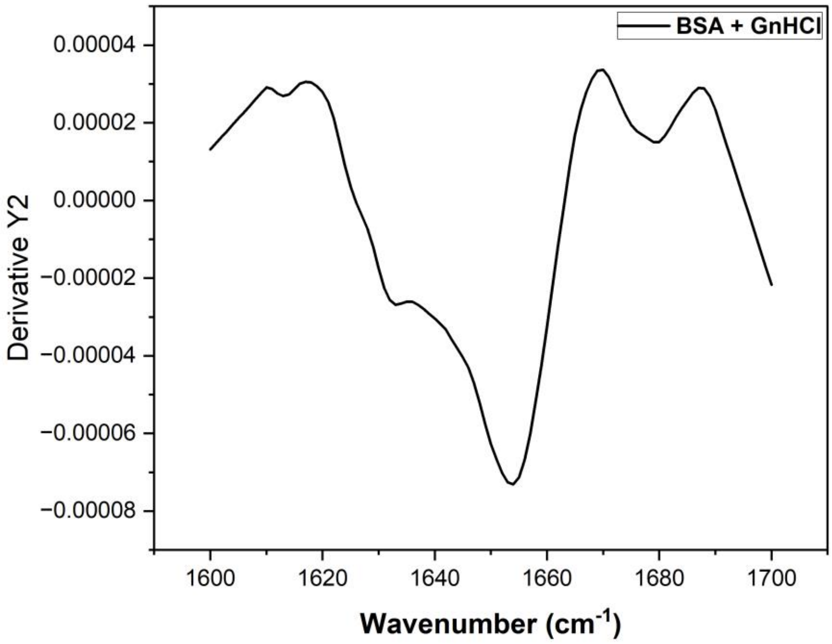

Figure O5: Second derivative amide I spectra of BSA in 3 M [D_5-Gu]. DCl showing peaks at 1680, 1654 and 1633 cm^{-1} corresponding to β-turns, α-helix and short-segment chains connecting α-helical segments[159].

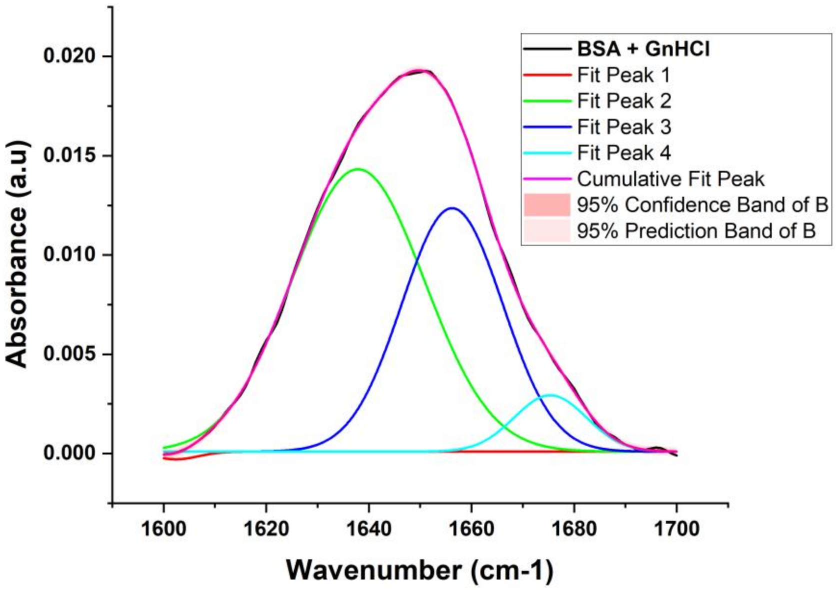

Figure O6: Curve-fit bands of the deconvoluted spectra of BSA in 3 M [D_5-Gu]. DCl showing three peaks. Peak 2 represents the short-segment chains connecting α-helical segments (1633cm^{-1}), peak 3 represents α-helix (1654 cm^{-1}) and peak 4 represents β-turns (1680 cm^{-1}).

Table O1: Comparison of the percentage area of secondary structural elements of BSA from literature[160] to experimentally determined values in D_2O (0.1 M phosphate, pD 7.8) and in 3 M [D_5-Gu]. DCl. Note: the percentage area for each secondary structural element was calculated by dividing the area under the corresponding fitted curve by the total area of all fitted curve bands. [a]The percentage area of secondary structural elements of BSA reported in the literature was determined by CD spectroscopy, wherein the protein was dissolved in H_2O (0.1 M phosphate, pH 7.4).

	**short-segment chains**	**α-helix**	**β-turn**
**BSA (literature)[a]**	23%	67%	10%
**BSA in D_2O (experimental)**	38%	55%	7%
**BSA in 3M [D_5-Gu]. DCl**	56%	37%	6%

The percentage area of secondary structural elements of BSA determined experimentally by IR spectroscopy in D_2O showed notable differences compared to literature values obtained by CD spectroscopy in H_2O, with α-helix content lower (55% vs. 67%) and short segment content higher (38% vs. 23%) in the present study. The inherent difference in technique sensitivity may account for part of the lower α-helix value obtained by IR spectroscopy, when compared to CD spectroscopy. In the presence of 3 M [D_5-Gu]. DCl, the percentage of α-helix decreased further (37% vs. 55%) and short-segment chains connecting α-helical segments increased (56% vs. 38%) indicating that 3 M [D_5-Gu]. DCl affects the secondary structure of BSA.

Sample preparation – pentadecapeptide *31a*

Deuterated TFE, D–TFE was prepared by mixing TFE (2 mL) and D_2O (2 mL) in a centrifuge tube followed by incubating for 24 hrs at rt to allow exchange of the hydroxyl proton of the TFE with D_2O. DCM (2 mL) was added to this solution to extract D–TFE from the aqueous layer, and the resulting DCM layer (bottom layer) collected. The collected solution was dried under vacuum at 35 ^{o}C to remove residual DCM to afford the D–TFE sample. Although partial loss of D–TFE was observed under these drying conditions, the quantity of the D–TFE sample recovered (1 mL) was sufficient for the subsequent use, as only 20 µL of D–TFE was required. Pentadecapeptide ***31a*** (1 mg) was dissolved in D_2O (5 mL) and incubated for 24 hrs at rt to allow complete deuteration, then freeze-dried. This H/D exchange procedure was performed independently for three samples of ***31a***, each subsequently dissolved in 20 µL of a different solvent system, to give 5% (w/v) peptide solutions:

1. D_2O (0.1 M phosphate, pD 7.8) for the native peptide (solution C)

2. 3 M [D_5-Gu]. DCl stock solution for the denatured peptide (solution D)

3. D–TFE for the α-helix inducing condition (solution E)

Solution C, D and E were directly analysed by IR spectroscopy to compare the secondary structural elements of pentadecapeptide ***31a*** under native, denaturing, and α-helix promoting conditions.

Solution C

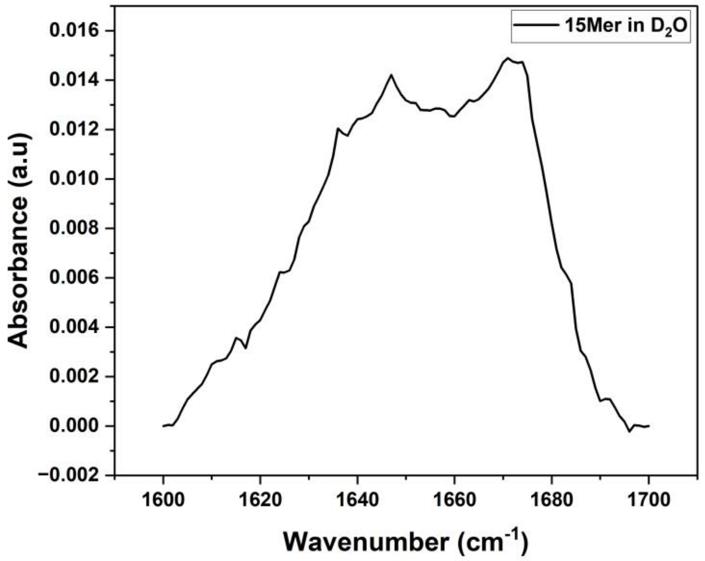

*Figure O7: FTIR spectra of pentadecapeptide **31a** in D_2O (0.1 M phosphate, pD 7.8) with D_2O (0.1 M phosphate, pD 7.8) subtracted and baseline corrected.*

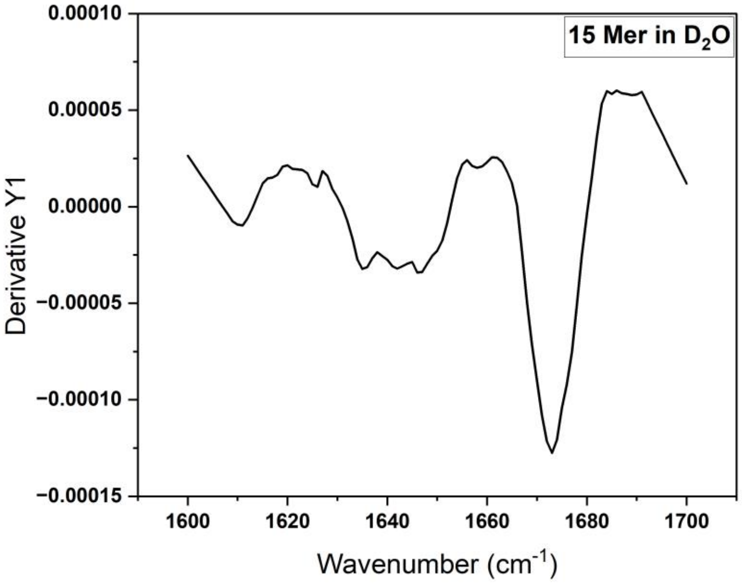

*Figure O8: Second derivative amide I spectra of pentadecapeptide **31a** in D_2O (0.1 M phosphate, pD 7.8) showing peaks at 1648, 1654 and 1673 cm^{-1}, corresponding to random coil, α-helix and β-sheet respectively[140]. Note: the broad overlapping region from 1630 to 1650 cm^{-1} made it difficult to fully resolve the 1648 and 1654 cm^{-1} bands.*

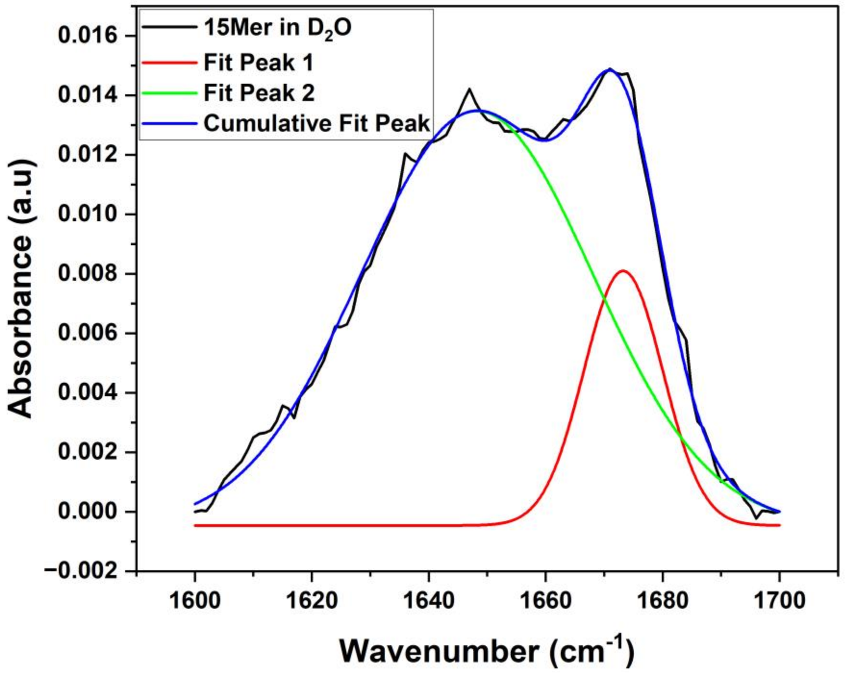

*Figure O9: Curve-fit bands of the deconvoluted spectra of pentadecapeptide **31a** in D_2O (0.1 M phosphate, pD 7.8) showing two resolved Gaussian bands. Peak 1 (1673 cm^{-1}) corresponds to β-sheet, while peak 2 represents an unresolved combination of random coil and α-helix, spanning the 1648–1654 cm^{-1} region identified by second derivative analysis.*

Solution D

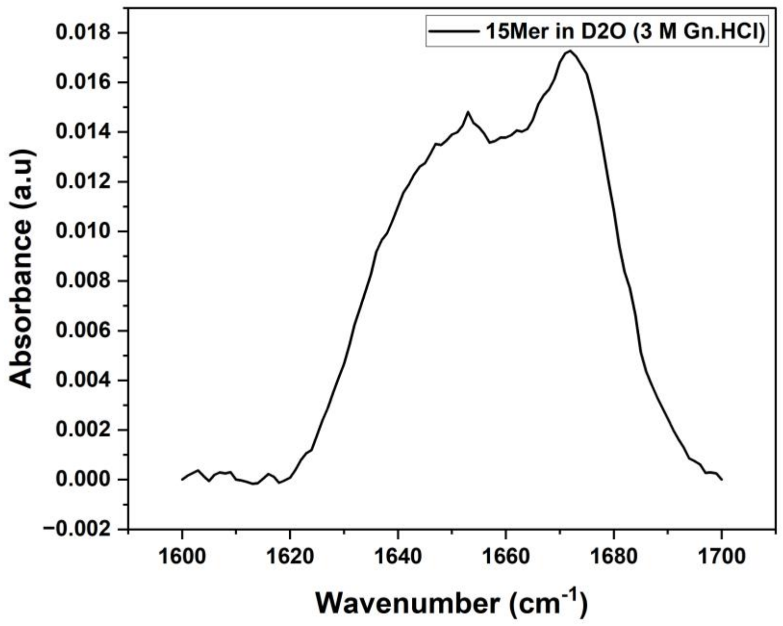

*Figure O10: FTIR spectra of pentadecapeptide **31a** in 3 M [D_5-Gu]. DCl with 3 M [D_5-Gu]. DCl subtracted and baseline corrected.*

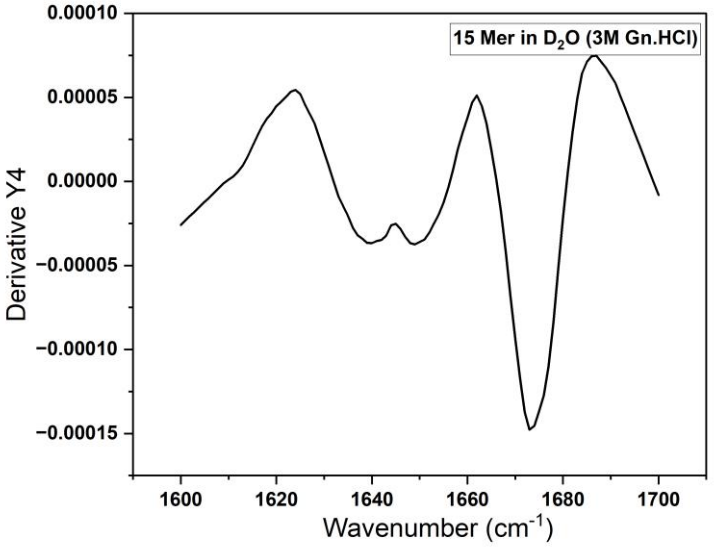

*Figure O11: Second derivative amide I spectra of pentadecapeptide **31a** in 3 M [D_5-Gu]. DCl showing peaks at 1648, 1654 and 1673 cm^{-1}, corresponding to random coil, α-helix and β-sheet respectively[140]. Note: the broad overlapping region from 1630 to 1650 cm^{-1} made it difficult to resolve the 1648 and 1654 cm^{-1} bands.*

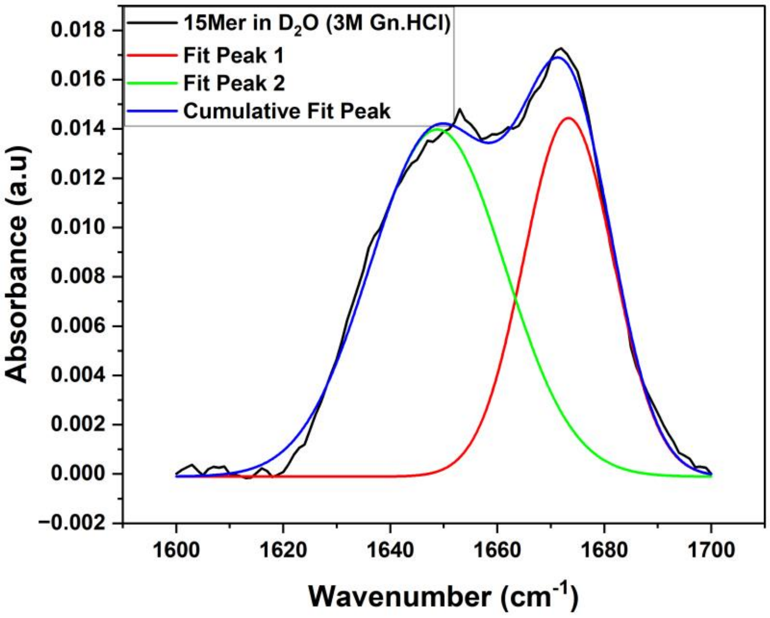

*Figure O12: Curve-fit bands of the deconvoluted spectra of pentadecapeptide **31a** in 3 M [D_5-Gu]. DCl showing two resolved Gaussian bands. Peak 1 (1673 cm^{-1}) corresponds to β-sheet, while peak 2 represents an unresolved combination of random coil and α-helix, spanning the 1648–1654 cm^{-1} region identified by second derivative analysis.*

Solution E

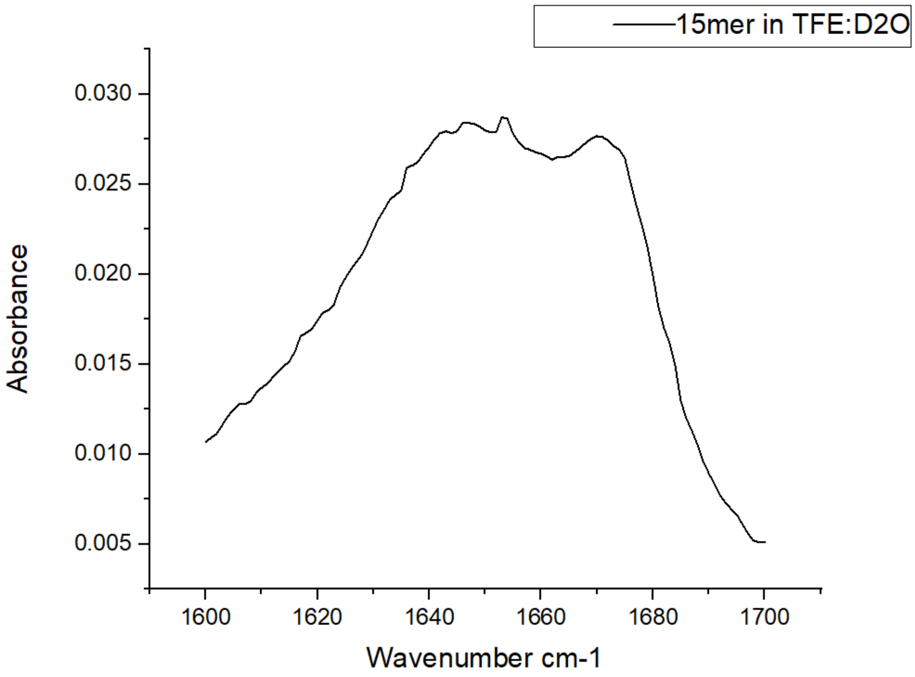

*Figure O13: FTIR spectra of pentadecapeptide **31a** in D–TFE with D–TFE subtracted and baseline corrected.*

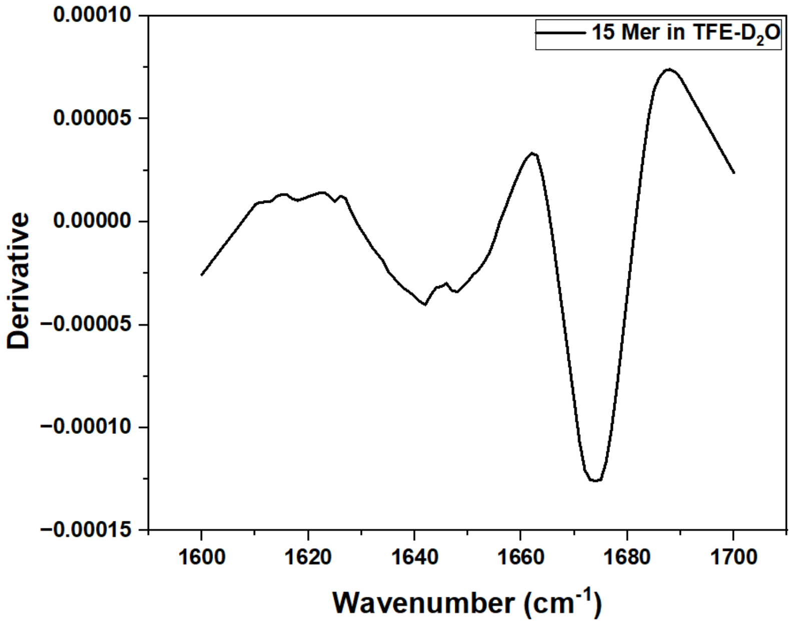

*Figure O14: Second derivative amide I spectra of pentadecapeptide **31a** in D–TFE showing peaks at 1648, 1654 and 1673 cm^{-1}, corresponding to random coil, α-helix and β-sheet respectively[140]. Note: the broad overlapping region from 1630 to 1650 cm^{-1} made it difficult to resolve the 1648 and 1654 cm^{-1}bands.*

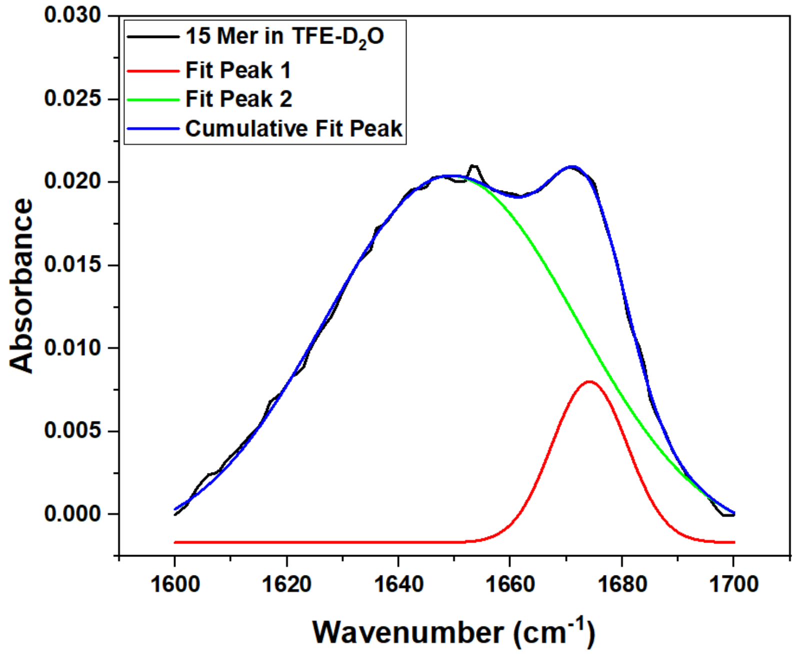

*Figure O15: Curve-fit bands of the deconvoluted spectra of pentadecapeptide **31a** in D–TFE showing two resolved Gaussian bands. Peak 1 (1673 cm^{-1}) corresponds to β-sheet, while peak 2 represents an unresolved combination of random coil and α-helix, spanning the 1648–1654 cm^{-1} region identified by second derivative analysis.*

*Table O2: Comparison of the percentage area of secondary structural elements of pentadecapeptide **31a** in D_2O (0.1 M phosphate, pD 7.8), 3 M [D_5-Gu]. DCl and D–TFE. [b]The percentage area of random coil + α-helix was calculated by dividing the area under the fitted curve spanning the 1648–1654 cm^{-1} region by the total area of all fitted curve bands.*

	**β-sheet (1673 cm-1)**	**random coil + α-helix[b] (1648 cm-1, 1654 cm-1)**
**15Mer in D_2O**	0.14515 (17%)	0.69241 (83%)
**15 Mer in D_2O (3 M [D_5-Gu]. DCl)**	0.30616 (41%)	0.44579 (59%)
**15 Mer in TFE:D_2O (1:1)**	0.1641(11%)	1.249 (89%)

FTIR spectroscopy of pentadecapeptide ***31a*** in D_2O (0.1 M phosphate, pD 7.8) revealed that the peptide has 17% β-sheet and 83% of secondary structure corresponding to α-helix and random coil. In the presence of 3 M [D_5-Gu]. DCl, there was an increase in the amount of β-sheet (41% vs. 17%) and a decrease in the total percentage of α-helix and random coil (59% vs. 83%). Speare and Rush showed that, in the presence of 6.6 M deuterated guanidinium hydrochloride, deuterated cytochrome c showed significant changes in its secondary structure[140]. When compared to the native cytochrome c, the protein solution with 6.6 M [D_5-Gu]. DCl showed an increase in the amount of β-sheet (51% vs. 23%) and turns (13% vs. 18%), while a subsequent decrease was observed in the amount of α-helix (45% vs. 17%) and random coil (19% vs. 15%)[140]. This is consistent with the changes observed in the secondary structure of pentadecapeptide ***31a*** in the presence of [D_5-Gu]. DCl wherein an increase in the amount of β-sheet was observed at the expense of α-helix and random coil.

In the presence of D-TFE, pentadecapeptide ***31a*** showed a decrease in the amount of β-sheet (17% vs. 11%) and an increase in the total percentage of α-helix and random coil (83% vs. 89%). TFE has been reported as a helix stabilising agent and can increase the amount of α-helical secondary structures in proteins implying that the increase in the total percentage of α-helix and random coil is likely arising from an increase in the amount of α-helix in pentadecapeptide ***31a***[161]**.**

References

103. J. Rush, C. R. Bertozzi, An α-Formylglycine Building Block for Fmoc-Based Solid-Phase Peptide Synthesis. *Org. Lett*. **2006**, *8*, 131–134.

128. N. D. J. Yates, M. E. Warnes, R. Breetveld, C. D. Spicer, N. Signoret, M. A. Fascione, Preparation and Application of an Inexpensive α-Formylglycine Building Block Compatible with Fmoc Solid-Phase Peptide Synthesis. *Org. Lett*. **2023**, *25*, 2001–2005.

152. Y. Zheng, C. Duanmu, Y. Gao, A magnetic biomimetic nanocatalyst for cleaving phosphoester and carboxylic ester bonds under mild conditions. *Org. Lett*. **2006**, *8*, 3215–3217.

153. N. B. Chapman, K. Clarke, R. M. Pinder, S. N. Sawhney, Highly fluorinated analogues of pharmacologically active compounds. *J. Chem. Soc. C*. **1967**, 293–296.

154. Z. Diwu, C. Beachdel, D. H. Klaubert, A facile protocol for the convenient preparation of amino-substituted α-bromo- and α, α-dibromo arylmethylketones. *Tet. Lett*. **1998**, *39*, 4987–4990.

155. M. Prokopowicz, P. Mlynarz, P. Kafarski, Synthesis of phosphonate derivatives of 2, 3-dihydroindene. *Tet. Lett*. **2009**, *50*, 7314–7317.

156. L. Pan, K. Lee, Y. Chan, Z. Ke, Y. Yeung, Applications of the DIB-BBr_3 protocol in bromination reactions. *Org. Lett*. **2023**, *25*, 53–57.

157. M. Kirihara, Y. Asai, S. Ogawa, T. Noguchi, A. Hatano, Y. Hirai, A Mild and Environmentally Benign Oxidation of Thiols to Disulfides. *Synthesis*. **2007**, *21*, 3286−3289.

158. S. E. Drewes, C. J. Hogan, P. T. Kaye, G. H. P. Roos, Medicinal plants of Southern Africa. Part 4. Synthesis of brackenin-like molecules from 1,4-dicarbonyl precursors and by oxidative coupling. *X*-Ray molecular structure of racemic-2,3-dibenzyl-1,4-diphenylbutane-1,4-dione. *J. Chem. Soc. C*. **1989**, Perkin Transactions 1, 1585–1591.

159. K. Murayama, M. Tomida, Heat-Induced Secondary Structure and Conformation Change of Bovine Serum Albumin Investigated by Fourier Transform Infrared Spectroscopy. *Biochemistry*. **2004**, *43*, 11526–11532.

140. J. O. Speare, T. S. Rush, IR spectra of cytochrome *c* denatured with deuterated guanidine hydrochloride show increase in β sheet. *Biopolymers (Biospectroscopy)*. **2003**, *72*, 193–204.

160. R. G. Reed, R. C. Feldhoff, O. L. Clute, T. Peters, Fragments of bovine serum albumin produced by limited proteolysis. Conformation and ligand binding. *Biochemistry*. **1975**, *14*, 4578–4583.

161. J. K. Myers, C. N. Pace, J. M. Scholtz, Trifluoroethanol effects on helix propensity and electrostatic interactions in the helical peptide from ribonuclease T1. *Protein Sci*. **1998**, *7* (2), 383–388.
