# Supplementary material for: Cysteine Thioaldehydes: Photolytic Generation, Reactivity, and Biological Implications
Source: Angew Chem Int Ed Engl. 2026 Aug 17;65(41):e26017. doi: 10.1002/anie.202526017 (PMC13638132; doi:10.1002/anie.202526017)
Supplement: Supplementary file 1 [file ANIE-65-e26017-s001.zip › anie74148-sup-0002-SuppMat_Section P.pdf]

P. Computational Chemistry

Table of Contents:

**P1**	*Density Functional Theory (DFT) Methods*	**2**
**P2**	*Molecular Dynamics (MD) Methods*	**2**
**P3**	*Conformational Analysis of Cysteine Derivatives*	**3**
**P4**	*Calculation of* pK_a *values*	**28**
**P5**	*Natural Bond Orbital Analysis:* $n \rightarrow \pi^*$ *interactions*	**32**
**P6**	*Cyclisation to Oxazolidine Intermediates*	**32**
**P7**	*Hydration of Cysteine Thioaldehyde*	**36**
**P8**	*Transformation of Hemi-thioacetal into Formylglycine*	**41**
**P9**	*Enolisation of Cysteine Thioaldehyde*	**47**
**P10**	*Phosphonium Adduct of Cysteine Thioaldehyde*	**55**
**P11**	*Reactivity of* C_β*-centered Radical of Cysteine Residues*	**57**
**P12**	*Molecular Dynamics Simulations*	**66**
**P13**	*References*	**75**

P1 Density Functional Theory (DFT) Methods

All DFT calculations were carried out using ORCA (Version 6.1.1).[89] Avogadro (Version 2.0.0) was used to prepare initial structures, generate Cartesian coordinates (.xyz files) for ORCA input files and to visually inspect output structures. Most structures were initially optimized by using the r2SCAN-3c method[90] with implicit water as solvent, which was applied by using the conductor-like polarizable continuum model (CPCM). All final reactant, product and transition-state structures were optimized under tight convergence thresholds (TightOPT, TightSFC) by using the wB97X-D4 functional, the def2-TZVP basis set and def2/J auxiliary basis set, again with implicit water as solvent (CPCM);[91-97] these structures were also subjected to a subsequent frequency calculation to confirm their status either as reactant/product minima or as transition-state first-order saddle points with an appropriate imaginary frequency; the imaginary frequency was checked visually by using the animation function of Avogadro to ensure that it represented the desired bond-forming/bond-breaking event. All confirmed transition-state structures were subjected to an intrinsic reaction coordinate (IRC) calculation in the forward and backward directions to confirm the connecting minima. The resulting backward and forward structures were then subjected to a final optimization and frequency calculation. Free-energy values were obtained from the frequency calculation output files to calculate thermodynamic relationships and reaction barriers, which are provided in units of kcal/mol.

The conformational landscape of some molecules was calculated as follows: An initial structure of the molecule was subjected to ORCA's global optimizer algorithm (GOAT) paired with Grimme's extended tight-binding method (GFN2-xTB) and the analytical linearized Poisson-Boltzmann (ALPB) solvent model (water);[98,99] Goodman's CONFPASS (Conformer Prioritizations and Analysis for DFT Re-optimizations) method[100] was used to select suitable conformers for further DFT analysis using the r2SCAN-3c method. Subsequently, additional redundant structures were removed from the collection through comparison of the electronic energies with visual inspection of structures, before reoptimisation at wB97X-D4/def2-TZVP/CPCM (water) with frequency calculation. Boltzmann populations were determined by using the resulting Gibbs free-energy values.

Transition-state candidate structures were generated by subjecting optimized reactant and product structures as inputs for a nudged elastic band (NEB) calculation (r2SCAN-3c/CPCM), ensuring that the inputs were as close as possible in terms of root mean square deviation (RMSD). The resulting candidate transition-state structures were then subjected to rigorous subsequent optimization and characterization, as outlined above.

Structures showing possible through-space $n \rightarrow \pi^*$ interactions were subjected to Natural Bond Order (NBO) analysis within ORCA.[129] The stabilization energy associated with the donor–acceptor interaction of interest was obtained from the table of data titled 'Second-Order Perturbation Theory Analysis of Fock Matrix' found within the output file.

P2 Molecular Dynamics (MD) Methods

The conformational landscape of cysteine-thioaldehyde-containing peptides in aqueous environments were simulated by using Gaussian-Accelerated Molecular Dynamics (GaMD) within OpenMM.[141,142] The AMBER ff19SB force-field parameters were used for standard amino acid residues.[143] Parameters for the non-canonical cysteine thioaldehyde were obtained by using the automated pipeline, DrFrankenstein, which was recently developed by Wood and co-workers.[144] Parameters for chloride

anion, which was used for neutralisation, were taken from the Li/Merz 12-6 ion parameter set for the OPC water model. The LEAP module in AmberTools was used to prepare the simulation system.[145] The peptide was solvated in a truncated octahedral solvent water box (OPC water model) that allowed ∼15 Å between the solute surface and the edge of the box.

For GaMD simulation, each system was first minimised, followed by 0.4 ns of conventional MD preparation, 2 ns of conventional MD equilibration, 0.4 ns of GaMD equilibration preparation, and 0.8 ns of GaMD equilibration, during which boost potential parameters were calculated. Production GaMD simulations of 500 ns were performed for each replicate. Boost potentials were applied to both the total potential energy and the dihedral energy, with a threshold energy standard deviation parameter σ_0 of 6.0 kcal/mol for both. The Langevin integrator was used with a 2 fs timestep and friction coefficient of 1.0 ps^{-1}. Temperature was maintained at 300 K and pressure at 1 bar using a Monte Carlo barostat with a coupling frequency of 25 steps. Non-bonded interactions were treated with PME electrostatics and a 10 Å cutoff, with hydrogen bond constraints applied. Coordinates and energies were saved every 10,000 steps (20 ps). Three independent replicates were run for each system initiated from different random seeds, giving 1.5 µs total sampling per system. The PyReweighting toolkit was used to recover unbiased thermodynamic populations.[146]

P3 Conformational Analysis of Cysteine Derivatives

P 3.1 Cysteine thioaldehyde

N-acetyl C-terminal methyl amide cysteine thioaldehyde **CT** was used as a model system for DFT calculations. Ten unique conformers were found (**CT_C1-C10**), the first five (**CT_C1-C5**) contributing to >98% of the conformational landscape.

Table P3.1: Cartesian coordinates, Gibbs energies (Hartree), relative Gibbs energies (kcal/mol) and Boltzmann populations of conformers of thioaldehyde **CT** (wB97X-D4/def2-TZVP/def2/J, CPCM: water).

Conformer 1 (**CT_C1**): Gibbs Energy: -893.10394988 Hartree<br>Relative Gibbs Energy = 0.00 kcal/mol<br>Boltzmann Population: 51.2%				Conformer 2 (**CT_C2**): Gibbs Energy: -893.10570385 Hartree<br>Relative Gibbs Energy = 0.46 kcal/mol<br>Boltzmann Population: 23.6%			
C	-5.55410445605065	2.36491458967528	0.97322002795081	C	-5.43797843127627	2.17806748859565	0.70821220311256
C	-4.23442021577181	1.72812914545217	1.46466619492232	C	-4.37269517950944	1.05073754421708	0.68265570259781
N	-5.81284830564565	2.20520109252915	-0.42746539087794	N	-6.01293851905127	2.38438795960542	-0.58585678752972
C	-6.65226274977681	1.81884100396278	1.83914701844071	C	-6.45382127974430	1.78956349882760	1.74248417623832
H	-5.45188070572173	3.42744378012291	1.22320628224698	H	-4.95276247519791	3.09487285968155	1.06323217440375
H	-6.44479170987148	1.92879273386962	2.90275911861269	H	-6.03308211301485	1.70814226920446	2.74451269316015
O	-3.75081240410945	2.08963130786445	2.53278854256863	O	-4.52083936851522	0.06572539996456	-0.02789535121658
S	-8.00458776463628	1.12281236660219	1.32746979227205	S	-8.00597578555723	1.48080947504994	1.47635431049143
H	-6.61560636932071	1.65577929716960	-0.69921638644807	H	-6.97798457195885	2.12330458788828	-0.72270816450060
C	-2.48647752539720	0.08830435682868	1.03549261997308	C	-2.29501436632775	0.23205242374259	1.65940897018410
N	-3.71807218250084	0.77670735495049	0.68779879392361	N	-3.34727121156490	1.22560141869606	1.52019123606300
H	-4.18376027129990	0.55120348107849	-0.17611871097716	H	-3.26455251585166	2.09397831423274	2.02307859293372
H	-2.59228373569235	-0.43115186720716	1.99035995981470	H	-2.71898871176169	-0.73451488677784	1.93849089450693
H	-2.26644901742742	-0.63746997136431	0.25454116222735	H	-1.74253558867392	0.11557661488416	0.72390303942157
H	-1.65773666765980	0.79584176330111	1.11216003148406	H	-1.61180300537177	0.56236312957143	2.44001944601245
C	-5.14133613985318	2.93969548564702	-1.34794950164241	C	-5.25416150093597	2.79994210634472	-1.62179971252434
O	-4.22757842409226	3.69199031399028	-1.02246917147427	O	-4.05966038452134	3.05578567141494	-1.47904161595457
C	-5.56551872602286	2.75849712869385	-2.78296470057256	C	-5.95015762982160	2.93077126914707	-2.95286943024429
H	-6.43161637451827	2.10505775712905	-2.88726580247805	H	-7.02012976961028	2.73154838922509	-2.89135559710401
H	-5.79290376866435	3.73906910332737	-3.20471465200562	H	-5.78733439946047	3.94031569921312	-3.33423689014144
H	-4.72592248596674	2.33772977637693	-3.34045522796092	H	-5.49134319227310	2.22796876727126	-3.65174988991026

Conformer 3 (**CT_C3**): Gibbs Energy: -893.10534779 Hartree
Relative Gibbs Energy = 0.68 kcal/mol
Boltzmann Population: 16.2%

C	-5.11530848582067	2.02383965214507	0.45064494885053
C	-3.95201477030602	1.02674537997783	0.61952261936893
N	-5.38083627003134	2.18176483829574	-0.94730091195609
C	-6.28299476447138	1.50174716614026	1.26011187544020
H	-4.82865082686896	2.98477775935207	0.88910744947493
H	-6.10403462058126	1.50752650674812	2.33584493012597
O	-3.72307677265303	0.19207595125730	-0.24923963432815
S	-7.66551618597907	0.95590110010321	0.66283891598390
H	-5.10449784488501	1.42480271871624	-1.55315312212146
C	-2.19494226508707	0.19993900603197	2.09022592043196
N	-3.27451616045035	1.11912260228481	1.76523189865851
H	-3.48546509170957	1.86710563356461	2.40596294841305
H	-1.83577237856538	0.43465054225482	3.09062394109745
H	-2.55328836640251	-0.83107587481662	2.06885962576750
H	-1.37182167633486	0.30075924312483	1.37922749832550
C	-6.10644569334408	3.21633752106505	-1.42228958454386
O	-6.51224971308935	4.11680445720697	-0.69047804449530
C	-6.38865278494777	3.19962750728498	-2.90394057399574
H	-7.46239986864622	3.05624957979296	-3.04573796468802
H	-6.11862635182253	4.16937919968007	-3.32475942812490
H	-5.84991910800328	2.40894950978962	-3.42628330768493

Conformer 4 (**CT_C4**): Gibbs Energy: -893.10406328 Hartree
Relative Gibbs Energy = 1.49 kcal/mol
Boltzmann Population: 4.2%

C	-4.84091942136969	2.22440213631907	0.32962150665230
C	-4.04302755670368	0.93174304630304	0.61887853760576
N	-5.38886294141419	2.11278448842828	-0.99588608955573
C	-5.92430441459146	2.39533304037229	1.37000945130865
H	-4.15419304476015	3.07203512423717	0.40096360505948
H	-6.88107220490461	1.95099942980035	1.09167329278370
O	-4.15570995644907	-0.04952437691342	-0.10829291349637
S	-5.72666524763317	3.11935968089382	2.78787320574271
H	-5.26901949241004	1.22792098608365	-1.46642397962266
C	-2.47180049733026	-0.16426237502951	2.12387041263138
N	-3.25173210323357	0.98371557543471	1.68940516529741
H	-3.22704054972456	1.82844828811646	2.23850310497536
H	-3.12391016750198	-1.01117709805175	2.34862304754315
H	-1.76287194159523	-0.46288124511411	1.34882725896516
H	-1.92378763237634	0.11554329806596	3.02176709898681
C	-6.07638696509567	3.12824276491780	-1.55692152295353
O	-6.25959126743116	4.18657318054751	-0.95622125566587
C	-6.60272949282483	2.89322600553073	-2.95032199351256
H	-6.34389088207812	1.90784950376603	-3.33776129435338
H	-7.68872156214527	3.00459222127542	-2.93391975689489
H	-6.19475265842672	3.66208632501645	-3.60928688149692

Conformer 5 (**CT_C5**): Gibbs Energy: -893.10394988 Hartree
Relative Gibbs Energy = 1.56 kcal/mol
Boltzmann Population: 3.7%

C	-5.60259370573559	2.12035783304666	0.95908992413169
C	-4.57591529999306	1.03314489097133	1.34930525655279
N	-5.87334013688382	2.11206376437497	-0.45851547917438
C	-6.83716122934948	1.94726892176580	1.79401706795996
H	-5.17203412993986	3.09395411251751	1.22506975565230
H	-6.63165672896795	1.94933741216726	2.86303910414385
O	-4.89798663432794	0.05139943341176	2.01407600801605
S	-8.33678051034408	1.78050537940565	1.25010964105669
H	-6.72749820416872	1.67067984111721	-0.76559248410952
C	-2.26557483814407	0.30172287008825	1.08726548503027
N	-3.34254152831002	1.25636797651812	0.88942575150194
H	-3.19316592762482	2.07693157630208	0.31663545928962
H	-1.36584685214631	0.69840203236223	0.61960533214625
H	-2.07717024741168	0.14805629762164	2.15205561574472
H	-2.51018706758350	-0.66231020303531	0.63429548090580
C	-5.12085027217456	2.80326779355635	-1.33840117239004
O	-4.09647650255422	3.39623936136057	-0.99205304021137
C	-5.59678390358668	2.80672767271126	-2.76757551613204
H	-6.49644304717993	2.20926045015041	-2.91394950608371
H	-5.79525840972612	3.83979201804359	-3.06053013099631
H	-4.79572482380849	2.42383056554255	-3.40235255303456

Conformer 6 (**CT_C6**): Gibbs Energy: -893.10213822 Hartree
Relative Gibbs Energy = 2.70 kcal/mol
Boltzmann Population: <1%

C	-5.42681761477982	2.59147494744726	0.99525558700979
C	-4.11348984519390	1.96955557001298	1.50046676040084
N	-5.42221373471424	2.94661392784492	-0.41290455476000
C	-6.64284443797959	1.77567571846430	1.36034310328667
H	-5.53263756719980	3.52970594639265	1.54834216935799
H	-7.52770135287253	2.02878528553865	0.77614442804051
O	-3.52343459893547	2.46306323546819	2.45858452388998
S	-6.72025210949019	0.70539795161724	2.55386146364375
H	-5.26592034026227	3.91236922824981	-0.65126286280909
C	-2.48208012619342	0.18235496810229	1.22963812631910
N	-3.68489018276385	0.89341407333069	0.83606107783782
H	-4.27729177283406	0.51649606967742	0.10869755633276
H	-2.58528655805930	-0.23679531340020	2.23423601105195
H	-2.31292645428644	-0.62644367340845	0.52036844312684
H	-1.61942329140341	0.85183219939834	1.22022817215979
C	-5.64596945661903	2.05127816759893	-1.39571537291736
O	-5.82339470359126	0.85669505454317	-1.14401049483374
C	-5.66781528307042	2.58150862939579	-2.80418616900242
H	-4.90896161524959	2.05240653976332	-3.38372670023748
H	-6.64324964159131	2.35717629807356	-3.24038218174107
H	-5.48440931290988	3.65445517588903	-2.85503908615662

Conformer 7 (**CT_C7**): Gibbs Energy: -893.1010713 Hartree
Relative Gibbs Energy = 3.37 kcal/mol
Boltzmann Population: <1%

C	-5.48315385866439	2.41041840562833	1.02801240844313
C	-4.17561762016481	1.79210186540121	1.57180791859738
N	-5.48393624214468	2.86289764877399	-0.34507546249104
C	-6.64827914023275	1.53099627195921	1.41531458164030
H	-5.60085363769149	3.30733980656353	1.64426887376848
H	-6.52552416025220	1.07026196416516	2.39643544244390
O	-3.83240818267433	2.05787717877533	2.72226704847364
S	-7.98984312602670	1.30157437589260	0.56976479855420
H	-5.65153757942421	3.84639374976330	-0.48210228453797
C	-2.27712069362072	0.32488979916311	1.19073216578525
N	-3.50447517828670	0.97376881014602	0.76349420779766
H	-3.93866470840430	0.71779533502166	-0.11624916085018
H	-1.88246800926364	-0.24802803241437	0.35297450945302
H	-1.53786987966342	1.06836862521851	1.49544704154618

Conformer 8 (**CT_C8**): Gibbs Energy: -893.10106442 Hartree
Relative Gibbs Energy = 3.37 kcal/mol
Boltzmann Population: <1%

C	-5.26433437070188	2.13983195419319	0.68390964117766
C	-4.02397020979572	1.23352223804266	0.60737325891014
N	-5.45526010922492	2.90633336113092	-0.52523179425857
C	-6.51782250998492	1.39014972033261	1.06524165133255
H	-5.11915579547575	2.86479934695190	1.49432376312556
H	-7.42584016105718	1.96513354860553	0.89056325637489
O	-3.68328486018161	0.69818196807890	-0.44142485700074
S	-6.58266188457855	-0.04645948373342	1.78448863787426
H	-4.94623146503540	3.77066405690236	-0.61309523376149
C	-2.23098862123146	0.18480801088404	1.88440282669779
N	-3.35924160674702	1.09149814591635	1.75957440359256
H	-3.71967261656025	1.53020057667407	2.59100423036156
H	-1.44526354633768	0.45407621979100	1.17600653115245
H	-1.83927386631456	0.25980422056224	2.89749532943728

H	-2.46015950816544	-0.34802624376484	2.03255971138700
C	-5.51801705552757	2.06708117890737	-1.43711292108738
O	-5.33049195002775	0.85133884035563	-1.37443119273073
C	-5.80339052770520	2.75547606192409	-2.74633008823869
H	-6.84216408570524	2.54481872537900	-3.01453894399896
H	-5.66613993463686	3.83569017172800	-2.69456402853706
H	-5.15888492171732	2.33397546141307	-3.51769462541815

H	-2.53618676943438	-0.84720428229386	1.69193493521198
C	-6.10880475760160	2.41787624765582	-1.60404906191678
O	-6.69919744440272	1.34137829016029	-1.57189640856012
C	-6.08681640916487	3.28137235570554	-2.83991721853220
H	-7.11171891021733	3.42378985243376	-3.18590232426926
H	-5.61730082011437	4.25029171913588	-2.67115940248198
H	-5.53797326583762	2.74695193287012	-3.61864216446755

Conformer 9 (**CT_C9**): Gibbs Energy: -893.10087683 Hartree
Relative Gibbs Energy = 3.49 kcal/mol
Boltzmann Population: <1%

C	-5.33756837755730	2.22304621051600	0.79349077576396
C	-3.97162247982593	1.79469261500423	1.36445429108136
N	-5.22998468231934	2.40015967637532	-0.63224382391564
C	-6.42680379261288	1.27580794332021	1.23479048822061
H	-5.55965899984068	3.19095232239207	1.24792557786293
H	-6.52033848342912	1.19982836367130	2.31929859560987
O	-3.24942159684476	2.59502459240428	1.94678240846117
S	-7.39069607933005	0.43476935281488	0.26883419934282
H	-4.70294871103650	1.72693705552960	-1.16555399402090
C	-2.35470403852514	-0.02280428275074	1.56296632234164
N	-3.64029846283423	0.51620797394329	1.14917535211832
H	-4.28311527366721	-0.08942281210200	0.66409655417026
H	-1.53744731895292	0.51257864319660	1.07467176177622
H	-2.23343828880656	0.06364552833897	2.64472060965571
H	-2.31655919932963	-1.07319671961457	1.27985270283192
C	-6.03990726117868	3.26445724863528	-1.29290163042611
O	-6.81398378276809	4.01009056132993	-0.70101812572065
C	-5.92864982101172	3.25332734633331	-2.79690181537367
H	-6.84904233808968	2.82730592201217	-3.20351542019796
H	-5.84647026146572	4.28192727381646	-3.15041011926950
H	-5.07835075057361	2.67167478483331	-3.15350471031238

Conformer 10 (**CT_C10**): Gibbs Energy: -893.09991061 Hartree
Relative Gibbs Energy = 4.09 kcal/mol
Boltzmann Population: <1%

C	-5.56500947954185	2.23523007365393	0.96398875247013
C	-4.61834473764191	1.08808814500980	1.37479377345416
N	-5.87032823421732	2.18510351931824	-0.45630475005882
C	-6.83889336201482	2.14025978253985	1.76106613261581
H	-5.06918189676671	3.18389831013074	1.17292450879317
H	-7.53922194611829	1.37483288620283	1.42453277071873
O	-4.99054374462446	0.17369523217973	2.10571686915042
S	-7.18110396664739	3.06203828063340	3.02499569255927
H	-6.65521747318758	1.63122753874957	-0.76188713111396
C	-2.38060787405755	0.16297201701119	1.08175793975846
N	-3.38957111748456	1.18451589392692	0.85940495010847
H	-3.18554514305601	1.97266597037650	0.26026216585679
H	-2.16076724106347	0.06182331538810	2.14693104874169
H	-2.71684616881877	-0.80492387751083	0.70209023414022
H	-1.47327566421684	0.45685461300307	0.55654981698609
C	-5.09460326683191	2.79405681507056	-1.37910311739681
O	-4.06520783643447	3.39435372007469	-1.06646714762657
C	-5.55827048860484	2.69681293997797	-2.80935249399729
H	-4.76843860045544	2.22718927181645	-3.39860447212857
H	-6.48149452379514	2.12774504442073	-2.91705791160159
H	-5.70852723442046	3.70857050802645	-3.19124763142980

Figure P3.1: Structures of conformers of thioaldehyde **CT** (wB97X-D4/def2-TZVP/def2/J, CPCM: water).

Conformer 1 (**CT_C1)**

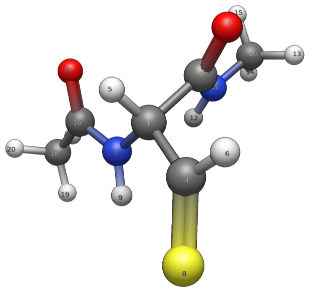

Conformer 2 (**CT_C2**)

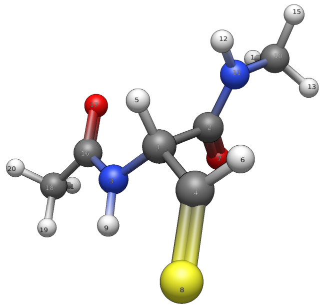

Conformer 3 (**CT_C3**)

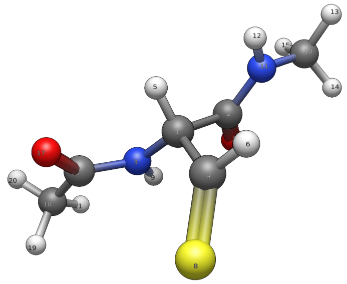

Conformer 4 (**CT_C4**)

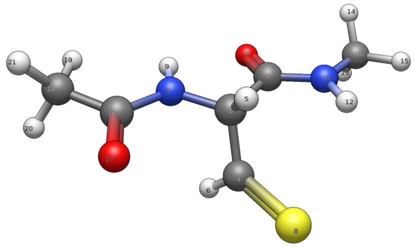

Conformer 5 (**CT_C5**)<br>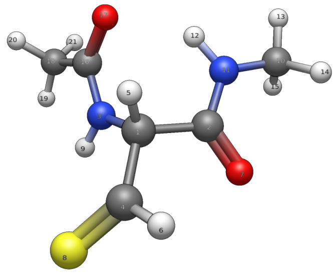	Conformer 6 (**CT_C6**)<br>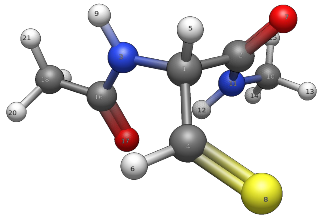
Conformer 7 (**CT_C7**)<br>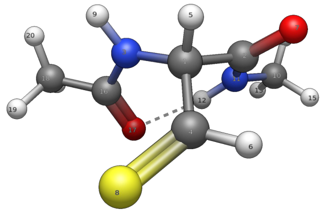	Conformer 8 (**CT_C8**)<br>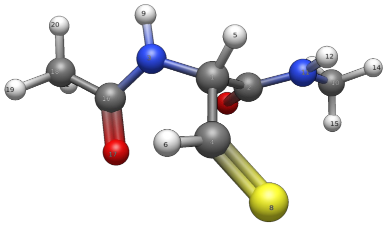
Conformer 9 (**CT_C9**)<br>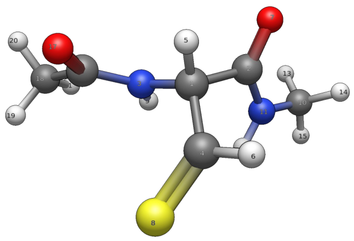	Conformer 10 (**CT_C10**)<br>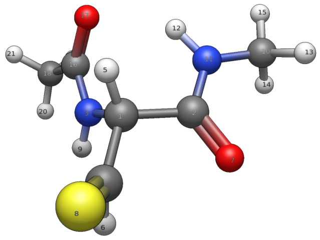

P3.2 Cysteine

The conformational landscape of N-acetyl C-terminal methyl amide cysteine **Cys** was explored. Twelve conformers were found (**Cys_C1-C12**), with Boltzmann distributions >1%.

Table P3.2: Cartesian coordinates, Gibbs energies (Hartree), relative Gibbs energies (kcal/mol) and Boltzmann populations of conformers of cysteine derivative **Cys** (wB97X-D4/def2-TZVP/def2/J, CPCM: water).

Conformer 1 (**Cys_C1**): Gibbs Energy: -894.33208227 Hartree
Relative Gibbs Energy = 0.00 kcal/mol
Boltzmann Population: 17.2%

C	-5.12514631800871	2.28661268532403	0.22997063337819
C	-3.99258418163187	1.28776161527917	0.45303942848794
N	-5.92625051394892	1.83036393245701	-0.88023878340005
C	-5.94570600677070	2.48008700800939	1.51219582208261
H	-4.68779112451696	3.25540771424639	-0.02463897135361
H	-7.28611111844677	2.51971865567293	-3.62248919728095
O	-4.00888150701553	0.16464455094854	-0.06130413409304
S	-6.70163130193786	0.96734966876869	2.15543125436442
H	-5.89276629893640	0.84688014347736	-1.10004498903234
C	-1.90459947441338	0.83960307405969	1.62743013177772
N	-3.01191160730940	1.70402545828334	1.25464377912230
H	-3.06616905393873	2.63084244058033	1.64780892494983
H	-2.26482863074769	-0.05343729432270	2.14260636999396
H	-1.33847493824990	0.53274454758584	0.74579694722664
H	-1.24727277591091	1.39339982346826	2.29502213133423
C	-6.73105070069604	2.64587344425969	-1.57774676434322
O	-6.81673562461483	3.85700278365330	-1.33172089338170
C	-7.52600725477461	2.01165789707784	-2.68619117372238
H	-7.32587037580367	0.94496287936327	-2.78870261395537
H	-8.58812881243800	2.16386159714601	-2.48261521599810
H	-6.77504638582949	3.15458046171157	1.29674487440576
H	-5.33385614383219	2.94713154186543	2.28352854273634
H	-5.57388808382719	0.36527330118453	2.56063710560082

Conformer 2 (**Cys_C2**): Gibbs Energy: -894.33196582 Hartree
Relative Gibbs Energy = 0.07 kcal/mol
Boltzmann Population: 15.2%

C	-5.59646739905677	2.51399210906261	0.90822785549241
C	-4.22711111177037	1.95179680391645	1.29648777308945
N	-6.05066782672329	2.12182701008150	-0.40638467196051
C	-6.62893760903710	2.15417131849490	1.97430631543257
H	-5.47931472176985	3.60007058583540	0.91572164472398
H	-6.88189862937027	1.36288551093406	-2.67162779192009
O	-3.74494577836438	2.24537594313949	2.39854285469817
S	-6.97415088100168	0.37886236538531	2.09720966857871
H	-6.59267332611071	1.27431441460081	-0.49104390714113
C	-2.29911129592682	0.59256066080952	0.70218514198814
N	-3.60624490044851	1.16293789413468	0.42356534283622
H	-4.05431353381192	0.95967439252557	-0.45471109226871
H	-1.56143107799054	1.37964500268532	0.87045430377106
H	-2.33624077610573	-0.04834299705858	1.58542012179712
H	-1.99399213236066	-0.00282415700970	-0.15624088099383
C	-5.72544953377457	2.79958850926570	-1.52631688584844
O	-5.00985888422023	3.80611865333162	-1.49816548139555
C	-6.28489627027962	2.26357057376270	-2.81494955025271
H	-6.90143985402174	3.03954145734388	-3.27360035810767
H	-5.45515794690259	2.04834137111328	-3.49118668428749
H	-7.58040907923635	2.62760280591269	1.72917157567987
H	-6.30434049231573	2.53261136064186	2.94113398464637
H	-5.78165517310035	0.01202634099084	2.59096393054206

Conformer 3 (**Cys_C3**): Gibbs Energy: -894.33172348 Hartree
Relative Gibbs Energy = 0.23 kcal/mol
Boltzmann Population: 11.8%

C	-5.49981566837771	2.50503734759468	0.97905834928381
C	-4.08412758108371	2.00734635217602	1.27570156333641
N	-6.02993950412941	2.07151039062788	-0.29486011221125
C	-6.43430497367058	2.13509414591862	2.12787621186892
H	-5.42637883615234	3.59496249249388	0.95485954778504
H	-7.04638458906803	2.93343670542411	-3.13876846188863
O	-3.52949276198730	2.36022199281004	2.32500981979956
S	-6.57176329164609	0.35504519378378	2.44658562101421
H	-6.58014251255818	1.22745836685841	-0.33788320859598
C	-2.15278499033343	0.70465256731174	0.57818939131653
N	-3.49850647727350	1.21568561928055	0.38139247420013
H	-4.00703140447702	0.95487372865583	-0.44723432288352
H	-2.09736208026725	0.09548248020613	1.48280217212628
H	-1.88942559398932	0.09002855867952	-0.28057774259476
H	-1.43609913631817	1.52397815025664	0.66263680090623
C	-5.78489078490685	2.73040903997174	-1.44690663315978
O	-5.07880443371676	3.74293459370315	-1.48264506905724
C	-6.41716110991805	2.16345573347003	-2.68771295926695
H	-5.62525623303281	1.91655913676732	-3.39791339548476
H	-7.01633035488345	1.27486178959106	-2.48832248236719
H	-7.42394294970985	2.55601428603537	1.95535201385554
H	-6.04289531693024	2.56694258383504	3.04785958106858
H	-7.39786764926963	0.05035667454834	1.43466405014883

Conformer 4 (**Cys_C4**): Gibbs Energy: -894.33153912 Hartree
Relative Gibbs Energy = 0.34 kcal/mol
Boltzmann Population: 9.7%

C	-5.47878364398466	2.08371490963516	0.67502732781514
C	-4.14532081910851	1.33328632026972	0.59164062052471
N	-6.33599975506704	1.72628857729813	-0.43189507761276
C	-6.13648399593943	1.71259595305926	2.00364480872811
H	-5.27909348190246	3.15911512130910	0.65790843874016
H	-7.16109346909461	2.37875246172056	-3.39628116773448
O	-4.09365290564795	0.14816338533306	0.24830794800727
S	-7.73958581832246	2.50599568039993	2.28113493746067
H	-7.25278031940540	1.35634310072003	-0.23732626722468
C	-1.74993101074627	1.42764251306593	1.02681633326329
N	-3.07243401727283	2.02663816558129	0.97293207156844
H	-3.18438866166226	2.99595191017167	1.22402866277975
H	-1.43800749622234	1.08467113318741	0.03798750089269
H	-1.04619725947862	2.17955904404688	1.37907845789777
H	-1.73618249291580	0.57835843720053	1.71304836352838
C	-5.96352531751921	1.91900937181097	-1.70664151887251
O	-4.86088325286784	2.40500372244273	-1.99078462142607
C	-6.94758339249217	1.51198415088628	-2.76755661827466
H	-6.48536631104830	0.74633451859911	-3.39433673822094
H	-7.87793001791845	1.12622156965248	-2.35064536622272
H	-5.46019204808075	1.96065724714417	2.82043190132808
H	-6.32668352687867	0.63740843322984	2.03839493229515
H	-7.29860922002373	3.77265220313567	2.25424827955918

Conformer 5 (**Cys_C5**): Gibbs Energy: -894.33138409 Hartree
Relative Gibbs Energy = 0.44 kcal/mol
Boltzmann Population: 8.2%

C	-5.39767935284302	2.21715003323382	0.68555751284690
C	-4.14376627691811	1.33680588324544	0.63739706893850
N	-6.29838697774883	1.88169853564640	-0.39534011186093
C	-6.08384713987741	2.00600324399213	2.03368414322589
H	-5.09774693525508	3.26332842175506	0.59374637805945
H	-7.83081728648825	1.16890919953357	-2.28414249735112
O	-4.20696182393711	0.13950647874231	0.33974961995136
S	-7.44449649818177	3.15394243043883	2.36058053707161
H	-7.15287856688026	1.38874983977324	-0.18853877545206
C	-1.74726851703971	1.21651763959058	1.05954632054076
N	-3.00584183777631	1.93844608544935	0.98489740193244
H	-3.02199450107346	2.92437175601147	1.19275444165932
H	-1.81128159134799	0.39892337664970	1.78050100881400
H	-1.47733039737058	0.80430352075652	0.08488656689451
H	-0.97095400447223	1.90978958155637	1.37788601646088
C	-5.92772065595285	2.01732541963201	-1.68026314536639
O	-4.83349778338470	2.50702606355364	-1.98630286539292
C	-6.90887189734406	1.55376380786792	-2.72010180520573
H	-7.14026574502438	2.39228861379114	-3.38010089431975
H	-6.43589547603210	0.77292960042033	-3.31937231919540
H	-5.36063361160019	2.18470760419751	2.83018229109277
H	-6.43364404389106	0.97672545661313	2.12723058716714
H	-8.33892731326028	2.62313533784944	1.51472572828875

Conformer 6 (**Cys_C6**): Gibbs Energy: -894.33132828 Hartree
Relative Gibbs Energy = 0.47 kcal/mol
Boltzmann Population: 7.8%

C	-5.40596030863966	1.91197198012226	0.55052110853469
C	-4.14454215307682	1.07553331275182	0.73134263330140
N	-5.82959910921046	1.77692975149253	-0.83311612891795
C	-6.50798154375640	1.43895058059198	1.49749615335912
H	-5.18894102011786	2.96394366375194	0.73486219075282
H	-6.64560762109607	3.16097904202068	-3.53677720726696
O	-4.18197229816555	-0.15068264428802	0.57039200422489
S	-6.04119305212688	1.54071391424968	3.24172525032504
H	-5.73359366202054	0.87056202830848	-1.26678260309279
C	-1.76538183874226	1.04937086262436	1.24664016761118
N	-3.02922980452823	1.73509092910732	1.03578938450498
H	-3.07617316557768	2.73280730257622	1.16720813121942
H	-1.00529078129382	1.79329483125044	1.47745682440327
H	-1.83989154505308	0.34525454925382	2.07818670308998
H	-1.46806827729107	0.50321436725395	0.34944749983591
C	-6.55156792069880	2.72303722412416	-1.46261468880248
O	-6.81752483410990	3.81029377506438	-0.93625153442296
C	-7.01151577996336	2.39036171858504	-2.85540210976672
H	-6.66479135661140	1.41288514615586	-3.19057161050084
H	-8.10326275844375	2.41442015292044	-2.87374754191253
H	-6.72859528113761	0.38699475632536	1.30795439706160
H	-7.41449364947651	2.01554670987036	1.31577355737582
H	-5.91553047206203	2.87487397598681	3.29963062798310

Conformer 7 (**Cys_C7**): Gibbs Energy: -894.33127403 Hartree
Relative Gibbs Energy = 0.51 kcal/mol
Boltzmann Population: 7.3%

C	-5.60999075527873	2.11572523560656	0.71759597147395
C	-4.44033951768242	1.13970360204477	0.86347627390299
N	-6.29402083047270	1.86948316687156	-0.54028585904233
C	-6.59721570395923	1.93688972274540	1.86432867381712
H	-5.24269027561204	3.14249121248765	0.70629485193138
H	-5.95430517125921	1.44728824768751	-3.59643597031677
O	-4.64687512883478	-0.07395967311047	0.98627726308967
S	-5.83541132026293	2.15035400445862	3.48952451244265
H	-7.00345991636778	1.15220138573319	-0.55867339197724
C	-2.02440156362478	0.84402364797569	0.93330539011323
N	-3.21780714940782	1.66638319864114	0.83094175221882
H	-3.12271977767174	2.65788015874874	0.67977196270333
H	-1.97315837607610	0.12769139918253	0.11054237832135
H	-1.15310708117737	1.49503241113755	0.89268489227284
H	-2.01468859547269	0.29533104905366	1.87711547270083
C	-5.88537161895079	2.40354727922051	-1.70719415708582
O	-4.94459416813847	3.20340318330204	-1.77022113383434
C	-6.64222305071526	1.98025445741072	-2.93606883479603
H	-7.49356346008106	1.33866238081846	-2.70798318656168
H	-6.98718922650406	2.87399816260506	-3.45924686782028
H	-6.99787275262719	0.92105741834890	1.85625657917037
H	-7.42841849592803	2.63111333473163	1.74655925011330
H	-5.56128429729465	3.45779294468266	3.37059738616266

Conformer 8 (**Cys_C8**): Gibbs Energy: -894.33116601 Hartree
Relative Gibbs Energy = 0.57 kcal/mol
Boltzmann Population: 6.5%

C	-5.61975077900755	2.23442889262205	0.71123587118406
C	-4.46419615992214	1.24405475570794	0.87185301494385
N	-6.31300712891807	1.97053077253178	-0.53820649303686
C	-6.60178671784723	2.09072777857857	1.86770097873680
H	-5.23645351785294	3.25417355052279	0.67994209941287
H	-7.53274972149737	1.41735588429216	-2.68938931575640
O	-4.68922490244185	0.04087959123212	1.05522242194807
S	-5.84485413685383	2.39784622869734	3.48066947390922
H	-7.04061923795178	1.27147787485312	-0.53647207379272
C	-2.05225950369648	0.90941845578716	0.90525980346903
N	-3.23424399504232	1.74817046453325	0.79977392136647
H	-3.12673199535304	2.72868168760668	0.59399091135859
H	-2.02557969651084	0.17025744408445	0.10167251198047
H	-1.17182535104690	1.54521813117479	0.83369222501051
H	-2.03668306524477	0.38677153667738	1.86345854107743
C	-5.89903444500073	2.47077013352614	-1.71818137333720
O	-4.94217494049674	3.24917105748707	-1.80239719324675
C	-6.66978847989933	2.03668104778406	-2.93453739179091
H	-6.99908615052460	2.92572170261460	-3.47552536846681
H	-5.99563099694624	1.47599150788368	-3.58608583503227
H	-7.06204902769280	1.10215437652203	1.85980891949327
H	-7.39255312041367	2.83450333584180	1.76081339964168
H	-5.12042516323850	1.27136161953896	3.54486415992759

Conformer 9 (**Cys_C9**): Gibbs Energy: -894.33104664 Hartree
Relative Gibbs Energy = 0.65 kcal/mol
Boltzmann Population: 5.8%

C	-5.35935948371404	2.15435234091103	0.49939274284002
C	-4.27109931692417	1.14194806619975	0.83691613691035
N	-5.89756400224121	1.79976754860801	-0.80329229524143
C	-6.45928908619998	2.13284633868277	1.56081879798425
H	-4.93457970569810	3.15511380566566	0.43208590426003
H	-8.14274205302535	2.41054264618081	-2.88417545429210
O	-4.55713867384416	-0.05254882167811	0.98908673497494
S	-5.87452179072918	2.59425236584538	3.20925173037485
H	-5.99852946570086	0.81893617422743	-1.01882782649277
C	-1.90799592165306	0.74669206389098	1.26629274563226

Conformer 10 (**Cys_C10**): Gibbs Energy: -894.33083558 Hartree
Relative Gibbs Energy = 0.78 kcal/mol
Boltzmann Population: 4.6%

C	-5.25272225357515	1.98971863039343	0.64831001800136
C	-3.99109816462931	1.13296070350657	0.70067228140462
N	-5.95938368972137	1.71395598941197	-0.58463986984053
C	-6.11039556509817	1.65670669574311	1.87175126246904
H	-4.99271654882897	3.04654469012993	0.65526636485646
H	-8.13137945146955	2.61472412276833	-2.62168643351616
O	-4.03653489573339	-0.07348361794831	0.43073232082781
S	-7.57598339276033	2.70323415990826	2.02748371002677
H	-6.17518867539446	0.74948096855086	-0.78806720566581
C	-1.61487004348050	1.04251983502376	1.23089306112191

N	-3.03012840009461	1.61113506567871	0.94041957500706
H	-2.87200168441235	2.59747207046113	0.80971288577343
H	-1.78935949698262	-0.03357109647450	0.51202681043263
H	-1.00477542198282	1.35297222371932	1.29605004723878
H	-2.05363806560309	0.27491111790803	2.24028003082238
C	-6.46837852872918	2.70675374210504	-1.61823441999789
O	-6.48656394840136	3.91407336048712	-1.35100508846844
C	-7.07677041283915	2.17280567137649	-2.88593488950913
H	-6.61856170418834	2.68395441448239	-3.73490811319903
H	-6.94573319472661	1.09614407825083	-2.99422357701742
H	-6.93082145302853	1.15081877827975	1.59641718556556
H	-7.22096213461937	2.86705296920295	1.29405713365194
H	-5.17019428826162	1.47992300608895	3.45695641184972

N	-2.87352265299238	1.75290716436980	1.07723320484534
H	-2.90836244732399	2.74159822125852	1.26877009036559
H	-0.85635735634152	1.75344086792096	1.55280216980396
H	-1.70425024292477	0.25319128234020	1.98003262099852
H	-1.30431352160121	0.59518523944744	0.28458245714676
C	-6.36888041036508	2.66425466984252	-1.44506834609277
O	-6.14067112334932	3.86753503826087	-1.27428753174177
C	-7.13075532563470	2.17766399373944	-2.64819606555233
H	-6.62882689143612	2.53845561898092	-3.54803199179868
H	-7.21141944758038	1.09127040602012	-2.68495796401135
H	-5.52968168685150	1.82597859366694	2.78084050410701
H	-6.40166315040032	0.60567287276983	1.84980054901484
H	-8.17173129630733	2.33283178409450	0.88492800202941

Conformer 11 (**Cys_C11**): Gibbs Energy: -894.3304799 Hartree
Relative Gibbs Energy = 1.00 kcal/mol
Boltzmann Population: 3.2%

C	-5.29281491186617	1.94552558685974	0.56362318400638
C	-4.00405746611639	1.13940917949110	0.69813895841246
N	-5.96140368284831	1.56696970489378	-0.66118359150883
C	-6.15801509041586	1.66731088216433	1.79653889246995
H	-5.06314568485203	3.00831018130112	0.50529929909412
H	-7.19830762356065	0.77801970183910	-2.71615489458880
O	-4.00451560403104	-0.08346111377195	0.51090529911297
S	-7.71849533623049	2.57956995589611	1.80784064399408
H	-6.15544190361592	0.58625654085192	-0.79644079782143
C	-1.63750360179538	1.16816963702866	1.27926244256457
N	-2.91590486235238	1.82158042721547	1.05220766976163
H	-2.98810740863368	2.81880641097676	1.17725599319153
H	-1.71835899023820	0.42475032877191	2.07486274877762
H	-1.28786759402972	0.67379784969581	0.37072315373091
H	-0.91259286055165	1.92513896036478	1.57241826640525
C	-6.43145145837545	2.45067162127023	-1.55915792357833
O	-6.25613536379677	3.67088455676055	-1.45584501511397
C	-7.19029617545587	1.86800263378885	-2.72084637056814
H	-8.21781340682463	2.23655759595586	-2.67839421987404
H	-6.74152331585485	2.22724692543254	-3.64878160859328
H	-5.59443809697757	1.88880942726812	2.70338950156696
H	-6.43087033631975	0.60985631530997	1.82135693697486
H	-7.19164745885704	3.80416462073513	1.95214464048354

Conformer 12 (**Cys_C12**): Gibbs Energy: -894.32949363 Hartree
Relative Gibbs Energy = 1.62 kcal/mol
Boltzmann Population: 1.1%

C	-5.48950015368820	2.15689587496772	1.09627142862211
C	-4.13833765063436	1.43891042606686	1.21064898831481
N	-5.81416013287687	2.47749991087037	-0.28110158077105
C	-6.59006895672130	1.37747717506675	1.81512380804458
H	-5.35409728322651	3.11315473767966	1.60948106230920
H	-7.33604703530895	1.45720627512214	-2.81109503545119
O	-3.94939454871090	0.55864788871988	2.05638758298360
S	-8.18699148148483	2.22773675313369	1.79718729534682
H	-6.17472745602695	3.39708244198133	-0.47659717416599
C	-1.84888943835772	1.27567443754146	0.40103649627457
N	-3.16944821330705	1.88045709962378	0.40488423089132
H	-3.41019653862273	2.53352225463045	-0.32312049873298
H	-1.23540192414044	1.79498863698912	-0.33310498444096
H	-1.37909139952861	1.36721999257747	1.38204853573902
H	-1.90181666066147	0.21687018050624	0.13541421011844
C	-5.92213984422343	1.53087590775133	-1.23447572508742
O	-5.62110737714549	0.35240457505420	-1.01596707982035
C	-6.40893979697125	1.98723468498465	-2.58182095115567
H	-6.58638361390047	3.06188507320653	-2.62358427003962
H	-5.66662056792273	1.70982802712052	-3.33278595115427
H	-6.29243276848142	1.18625261576136	2.84355435858792
H	-6.75933480481492	0.41551693337748	1.33103697017199
H	-7.83558058694322	3.25900602756695	2.57974149231511

Figure P3.2: Structures of conformers of cysteine derivative **Cys** (wB97X-D4/def2-TZVP/def2/J, CPCM: water).

Conformer 1 (**Cys_C1**)

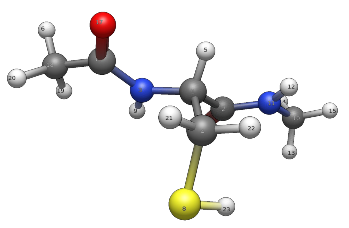

Conformer 2 (**Cys_C2**)

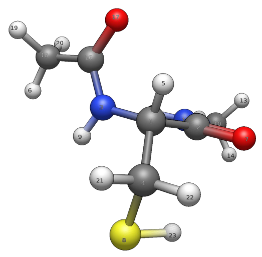

Conformer 3 (**Cys_C3**) 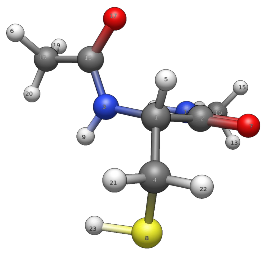	Conformer 4 (**Cys_C4**) 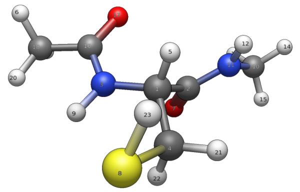
Conformer 5 (**Cys_C5**) 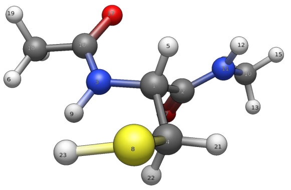	Conformer 6 (**Cys_C6**) 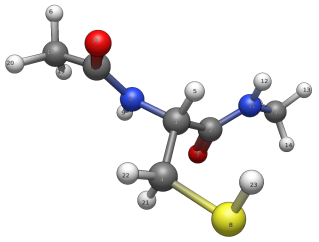
Conformer 7 (**Cys_C7**) 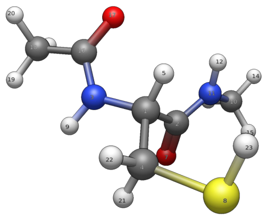	Conformer 8 (**Cys_C8**) 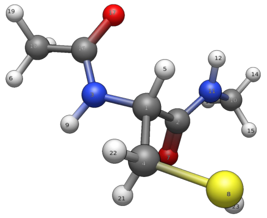

Conformer 9 (**Cys_C9**)

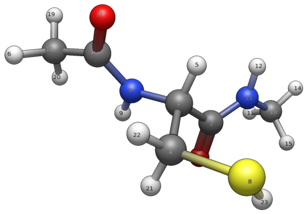

Conformer 10 (**Cys_C10**)

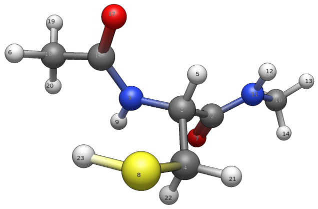

Conformer 11 (**Cys_C11**)

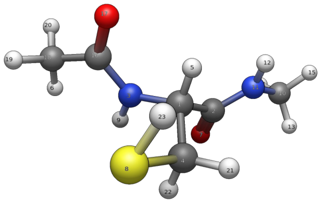

Conformer 12 (**Cys_C12**)

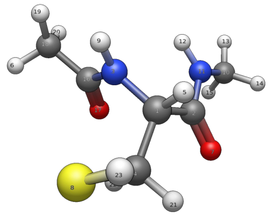

P3.3 Cysteine Thiolate

The conformational landscape of N-acetyl C-terminal methyl amide cysteine thiolate **Cys-CB** was explored. Six conformers were found (**Cys-CB_C1-C6**), with Boltzmann distributions >1%.

Table P3.3: Cartesian coordinates, Gibbs energies (Hartree), relative Gibbs energies (kcal/mol) and Boltzmann populations of conformers of cysteine thiolate derivative **Cys-CB** (wB97X-D4/def2-TZVP/def2/J, CPCM: water).

Conformer 1 (**Cys-CB_C1**): Gibbs Energy: -893.8608607 Hartree<br>Relative Gibbs Energy = 0.00 kcal/mol<br>Boltzmann Population: 55.6%				Conformer 2 (**Cys-CB_C2**): Gibbs Energy: -893.86006943 Hartree<br>Relative Gibbs Energy = 0.50 kcal/mol<br>Boltzmann Population: 24.1%			
C	-4.92665920471627	2.90216083179626	0.61604745298153	C	-5.05429205081729	2.72787548413986	0.94743445176570
C	-3.62601280106020	2.11241555441428	0.46351338338484	C	-3.81046037610167	1.86571198382937	1.13610784619174
N	-5.99934482163675	2.37598067653668	-0.20389141041286	N	-5.88307857956273	2.32695995339017	-0.16600249260009
C	-5.39664368354326	2.92237567084190	2.08373969452638	C	-5.90205446491305	2.70969460370887	2.22773694467885
H	-4.69371794236090	3.91944136267197	0.29622600175427	H	-4.68570903844029	3.74130895441700	0.76852446931509
H	-7.56896519936028	2.35097135221298	-2.92273260537968	H	-6.03159486850251	1.47288341579916	-3.17945242764040
O	-2.57543710986991	2.67395251737651	0.11585009787256	O	-2.95853822210791	2.20531591412658	1.97222009478369
S	-6.20537751832675	1.38142733611743	2.63214215435238	S	-6.74160088922792	1.11907670655980	2.49591240535300
H	-6.66958879874521	1.80595010196253	0.30562527497585	H	-6.64217437630434	1.69961850182237	0.08372841934619
C	-2.51065671391531	-0.02310041508963	0.79074308880317	C	-2.54490042527637	-0.12597520367840	0.53966344072396
N	-3.69798080945083	0.81088377831323	0.75201418249861	N	-3.67118877760024	0.77619994326617	0.38199894680125

H	-4.53117551622838	0.53094474341058	1.27671750789461
H	-2.81122084931349	-1.04402448201661	1.02279939241562
H	-2.00845124502587	-0.01857785861150	-0.17803383456718
H	-1.80152737997085	0.31899357957722	1.55035401105767
C	-5.95444611421044	2.34348592698552	-1.54031508779159
O	-5.02823439257534	2.86159996770091	-2.18460264573017
C	-7.09518120689581	1.64881689491966	-2.23358871783667
H	-6.69181787419813	0.82133148923163	-2.82159866117207
H	-7.83986430067420	1.26731786676611	-1.53457847793151
H	-6.08976520254126	3.76137766963858	2.18823515942146
H	-4.52552714268040	3.15106047894369	2.70409744978278

H	-4.43041933920198	0.51199178221377	-0.22375791423350
H	-2.62244521980069	-0.90886323785922	-0.21293248855737
H	-1.60281195974781	0.40699513930103	0.39915771374253
H	-2.54201373483213	-0.58481412532496	1.53174238184166
C	-5.62213435595422	2.62579390231272	-1.44497775070807
O	-4.64357553055524	3.31108583147976	-1.78105491349845
C	-6.58605448391647	2.08853556618937	-2.46798022519703
H	-7.38498684345283	1.49683148462666	-2.02051558888744
H	-7.01859518093405	2.92832016127012	-3.01612786149016
H	-6.62852451961148	3.52394565713380	2.14660369804026
H	-5.24044259073851	2.95629262507592	3.06073426082861

Conformer 3 (**Cys-CB_C3**): Gibbs Energy: -893.85907075 Hartree
Relative Gibbs Energy = 1.12 kcal/mol
Boltzmann Population: 8.4%

C	-4.84124335718755	2.24602259001834	0.24851155941110
C	-3.91159684076777	1.03791435491010	0.35407180428680
N	-5.90159552020257	1.89926877922896	-0.67969000844191
C	-5.39046088802253	2.67954623601240	1.62134168080048
H	-4.26980825479586	3.07958450160735	-0.16850132834411
H	-8.63790536580461	2.64750066085543	-1.99328093032794
O	-4.09852355496591	0.01207783390115	-0.32123676990336
S	-4.19446526311396	3.60010038437976	2.64144476880757
H	-5.99225078397217	0.92601988521098	-0.92973693693297
C	-2.00273252957518	0.06770799027031	1.49656864556394
N	-2.90328361148844	1.16883047326532	1.21173919731024
H	-2.95120235150313	1.98645418962088	1.83510394569220
H	-1.26810413267580	0.40470314241927	2.22700481062494
H	-2.53808272266500	-0.79369530955838	1.90555125514734
H	-1.47737708981615	-0.24865483201851	0.59325076697480
C	-6.62808179858520	2.81658888642762	-1.32881539943001
O	-6.47824020318806	4.03527893496557	-1.14715162735071
C	-7.65111315233487	2.29619505355986	-2.30253260708399
H	-7.43884307782879	2.71564619093055	-3.28825661607659
H	-7.65777594006428	1.20780639951755	-2.36474864374403
H	-5.75102911298730	1.78556062220864	2.14284409968283
H	-6.26388027585475	3.30432807586680	1.42528174393337

Conformer 4 (**Cys-CB_C4**): Gibbs Energy: -893.85892078 Hartree
Relative Gibbs Energy = 1.22 kcal/mol
Boltzmann Population: 7.1%

C	-4.94318120039502	2.49576307898127	0.58969664681572
C	-4.31879373331560	1.11861301573226	0.77069034640699
N	-6.06718328966064	2.44227340260102	-0.31737704805983
C	-5.43451658835221	2.99994289204247	1.95824715690763
H	-4.18554015078436	3.18554104757260	0.20442140599060
H	-6.93778812247107	2.69071064671137	-3.32364851988218
O	-5.00452725073531	0.09571931233544	0.89897579429464
S	-6.42364754399457	4.52044175650371	1.84515049371313
H	-6.84015588062542	3.04195158827636	-0.04140744662863
C	-2.24649363699010	-0.12223555711648	1.11695018931789
N	-2.98559359439501	1.09964803907927	0.85579030556026
H	-2.48098639190448	1.96128120450173	0.72388860803908
H	-1.18684155692304	0.12100399002485	1.17215305743133
H	-2.55682884755171	-0.57019222415061	2.06330307457879
H	-2.40080651872436	-0.85141849310701	0.31826592219303
C	-5.97608008522248	1.98015424147947	-1.56842202130202
O	-4.94255873473884	1.44605341537889	-2.00323808543132
C	-7.20232737007345	2.12233317228350	-2.42945266215285
H	-7.52535888944725	1.12813186607104	-2.74594658597264
H	-8.02159861442845	2.62032630834602	-1.91036310912263
H	-4.54876346671204	3.15142839311661	2.58056142215565
H	-6.01853805995444	2.19731394703616	2.42052446594733

Conformer 5 (**Cys-CB_C5**): Gibbs Energy: -893.85834954 Hartree
Relative Gibbs Energy = 1.58 kcal/mol
Boltzmann Population: 3.9%

C	-4.78087755589959	2.88344137266041	0.58305769171392
C	-3.56372949864462	1.97627470588934	0.65890423444577
N	-5.90355295489320	2.28540983697658	-0.10786938268957
C	-5.21286662964054	3.24802936558193	2.01624200972944
H	-4.45891513555235	3.78949948237051	0.06141424265294
H	-7.07170964354096	0.62801628718525	-2.48214961673171
O	-2.43667531401028	2.47169026756830	0.81163994875176
S	-6.77360114843564	4.17621075108146	2.07409892284521
H	-6.79710651657983	2.50139260834063	0.32954868500623
C	-2.67147903266105	-0.29077057050847	0.71856971493812
N	-3.76667927294361	0.65809986677845	0.63113853691749
H	-4.69782026728575	0.32296662549036	0.44371092171282
H	-2.12327433918651	-0.16546839399044	1.65442450806886
H	-3.08471586180227	-1.29746759704377	0.68450843161126
H	-1.97473724156440	-0.16533751851581	-0.11394257059520
C	-5.86854007891283	2.02379402290741	-1.42600743808046
O	-4.81755288137704	2.08121642900999	-2.07934328373196
C	-7.17176881907026	1.63381668855718	-2.06826693307552
H	-8.00540351831505	1.65536704871374	-1.36602389726127
H	-7.37181030579735	2.31952120636733	-2.89427178385986
H	-4.39290516125310	3.81901786936814	2.45790586603106
H	-5.30187465003362	2.32006468881143	2.59147460230069

Conformer 6 (**Cys-CB_C6**): Gibbs Energy: -893.85698368 Hartree
Relative Gibbs Energy = 2.43 kcal/mol
Boltzmann Population: 0.9%

C	-4.59420600859044	2.72154135070501	0.14676481844781
C	-3.84238275156517	1.38274942342769	0.17176908399305
N	-5.60628967755832	2.68490034653149	-0.90138588987899
C	-5.18112806690951	3.20363791468881	1.48277200082296
H	-3.85228141797806	3.45788348487141	-0.17533919861366
H	-8.54340909338299	1.95579164560598	-1.72300612491770
O	-3.65715411682190	0.73620370934422	-0.87164862767853
S	-3.94455486263618	3.69495466458893	2.72969640173531
H	-5.57304006851289	3.38764411160071	-1.62126232926681
C	-2.52587445127156	-0.18128152465214	1.47753813848331
N	-3.36827811313388	0.99248118472091	1.35211674820034
H	-3.41608723360723	1.68245561054090	2.11261022350132
H	-1.61433025612024	-0.08624721829008	0.88061501725033
H	-2.25007054198018	-0.29691971626583	2.52517358399194
H	-3.05707648532751	-1.07913223605425	1.15490975536734
C	-6.56954260022530	1.75620861750615	-0.96545653792939
O	-6.67230421700506	0.84754935888999	-0.12831188823586
C	-7.52996622241292	1.86420775371620	-2.11936370168339
H	-7.31616882620497	2.71374506445542	-2.76809208968524
H	-7.47853215216529	0.94158148353543	-2.70151521952672
H	-5.84484452361851	2.43082308821915	1.87821144223785
H	-5.81007414017171	4.06400695621385	1.24196780448474

Figure P3.3: Structures of conformers of cysteine thiolate derivative **Cys-CB** (wB97X-D4/def2-TZVP/def2/J, CPCM: water).

Conformer 1 (**Cys-CB_C1**)<br>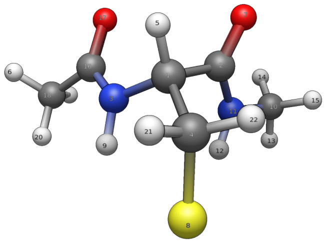	Conformer 2 (**Cys-CB_C2**)<br>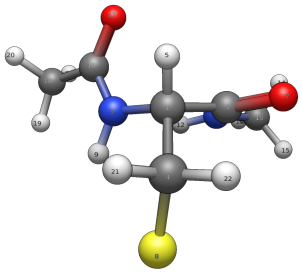
Conformer 3 (**Cys-CB_C3**)<br>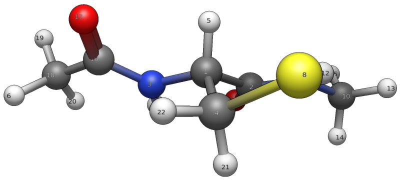	Conformer 4 (**Cys-CB_C4**)<br>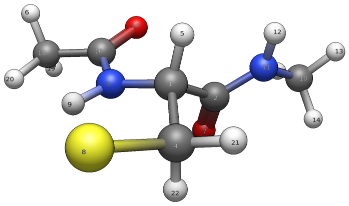
Conformer 5 (**Cys-CB_C5**)<br>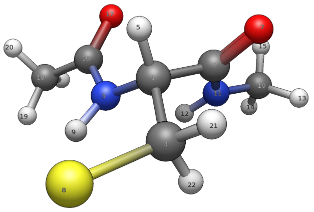	Conformer 6 (**Cys-CB_C6**)<br>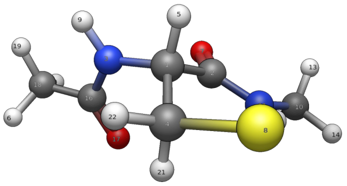

P3.4 Cysteine E-Enethiol

The conformational landscape of N-acetyl C-terminal methyl amide *E*-configured cysteine enethiol **E-ET** was explored. Five conformers were found (**E-ET_C1-C5**), with Boltzmann distributions >0.5%.

Table P3.4: Cartesian coordinates, Gibbs energies (Hartree), relative Gibbs energies (kcal/mol) and Boltzmann populations of conformers of *E*-configured cysteine enethiol derivative **E-ET** (wB97X-D4/def2-TZVP/def2/J, CPCM: water).

Conformer 1 (**E-ET_C1**): Gibbs Energy: -893.11232488 Hartree
Relative Gibbs Energy = 0.00 kcal/mol
Boltzmann Population: 57.8%

C	-5.35135890252268	1.78160754087084	1.02568352989778
C	-4.11043300691012	1.02906632484621	1.34286269840651
N	-5.70047230176663	1.97265623441333	-0.34248118256729
C	-6.17572035176213	2.22974770713306	1.97728179955615
H	-6.42600763114656	2.37452736874279	-2.74162781490268
H	-7.07185636115217	2.75638317572056	1.66991207938389
O	-3.67192107324724	1.00357519002623	2.50242614215663
S	-5.98435633321942	2.04330939852103	3.69534953514868
H	-6.52476820103085	1.51528590011847	-0.70212716992639
C	-2.29850930854291	-0.38620695354440	0.54387584269094
N	-3.52047742832429	0.37004016483634	0.33929004444092
H	-3.91332649925673	0.42791177629626	-0.58545550485911
H	-2.45308567249653	-1.19001759304047	1.26694878666737
H	-2.00067829979643	-0.82054678159643	-0.40880691640372
H	-1.49558833781812	0.25968561264345	0.90631577576716
C	-5.02658793932444	2.83004239313810	-1.14405346587114
O	-4.05858773664016	3.47373418095873	-0.73239342770761
C	-5.50953370289986	2.93726961072111	-2.56308037780748
H	-5.67269932900628	3.99065242421252	-2.79722834973730
H	-4.72236441588675	2.56294609522734	-3.22180078418611
H	-7.18040872604948	2.57838559289486	3.98848366205281

Conformer 2 (**E-ET_C2**): Gibbs Energy: -893.11172652 Hartree
Relative Gibbs Energy = 0.38 kcal/mol
Boltzmann Population: 30.7%

C	-5.53926009885219	1.72233066143420	0.99346942279525
C	-4.21886254519839	1.17477299586205	1.41476803618936
N	-5.78152410346924	1.83164789420149	-0.40915257985952
C	-6.52724946334139	2.06433645751681	1.82898444548211
H	-4.67107251691234	2.53840516041561	-3.20512889160138
H	-7.44751128238277	2.43066460878050	1.38350073514962
O	-3.81858785762099	1.24825730927450	2.58911029838612
S	-6.62952213733295	1.99357776764114	3.55717659025561
H	-6.49959833216559	1.25758504465206	-0.82512082734721
C	-2.17990267344513	0.02540559985707	0.74621038778038
N	-3.49186970234888	0.57913898962219	0.46414622695401
H	-3.84336154062520	0.55412458602172	-0.47865668538996
H	-2.24337030118725	-0.76211998479201	1.49997918228017
H	-1.78178113175842	-0.39699565672816	-0.17458009081936
H	-1.49880153667795	0.79980366827968	1.10614242809084
C	-5.18728727639050	2.78330613610324	-1.16681788012675
O	-4.37190777713358	3.57421370104458	-0.68684754089397
C	-5.55674352874982	2.80479293851065	-2.62327640435379
H	-6.36537492546841	2.11477479862992	-2.86448941663536
H	-5.84616906368512	3.82124657878895	-2.89509308158151
H	-5.36898376405364	1.58078612928371	3.76505054764530

Conformer 3 (**E-ET_C3**): Gibbs Energy: -893.1102954 Hartree
Relative Gibbs Energy = 1.27 kcal/mol
Boltzmann Population: 6.7%

C	-5.17083639049258	2.02191269143734	0.73083804505490
C	-3.77667947249268	1.47005303191883	0.66971545218127
N	-5.61583319387849	2.68026298239838	-0.43755157215589
C	-5.91330638017872	2.05930653999829	1.83739155724702
H	-6.53386969339885	2.39187099385504	-3.49169129219863
H	-6.88506537443085	2.53905027839910	1.80058437991609
O	-2.85667879023363	2.13685829094765	0.18451499944131
S	-5.44234302865222	1.45049576186729	3.41235909233790
H	-6.11636001997946	3.55030455327678	-0.33691561129422
C	-2.28452913390509	-0.36300236307811	1.25896729583272
N	-3.59594071576599	0.26064536088126	1.20487319648655
H	-4.40191635977447	-0.27163630583424	1.49136419946061
H	-2.37568320738449	-1.30561828775015	1.79577130090590
H	-1.90187305936859	-0.55888114795632	0.25447830268565
H	-1.57744484435689	0.27986639594625	1.78584548088933
C	-5.46005968935832	2.15778256614428	-1.67574077676342
O	-4.94054898419711	1.05331990641187	-1.85138500584746
C	-5.93657192566669	3.00816030164200	-2.81837401040634
H	-6.51947263098514	3.86742595451069	-2.48684221564303
H	-5.06089283057822	3.36135655262814	-3.36899457248315
H	-6.50283583372136	1.95052132685556	4.06016665675286

Conformer 4 (**E-ET_C4**): Gibbs Energy: -893.10984865 Hartree
Relative Gibbs Energy = 1.55 kcal/mol
Boltzmann Population: 4.2%

C	-5.46636460846559	1.84243815520153	0.70552866707954
C	-4.47748085248457	0.71721486428235	0.58062657637276
N	-5.89080479686749	2.41204768050993	-0.51684095640509
C	-6.06586887968192	2.20702155713081	1.83911466090505
H	-6.74443247796255	3.46450955606466	-2.65637632741159
H	-6.80145563662541	3.00545603064808	1.79662178287645
O	-4.68290878611364	-0.21282510256606	-0.20603611508521
S	-5.94224625475149	1.53681146120184	3.44802255046808
H	-6.86714231844035	2.64549403023982	-0.61672583758088
C	-2.40161515221908	-0.26919985634980	1.38851615675010
N	-3.41033167936336	0.77676168596665	1.37893360166398
H	-3.26889689377388	1.60362008755516	1.93725650460876
H	-2.85471528541430	-1.23615896129302	1.61483002091314
H	-1.89661296845070	-0.33437609903444	0.42175614659184
H	-1.66865814487490	-0.03164919709613	2.15749043234697
C	-5.04880444781095	2.70408703385349	-1.53507067399900
O	-3.83286668918815	2.52195925263264	-1.44513406715437
C	-5.68473963881922	3.24388914655640	-2.78448018163487
H	-5.15274437824625	4.14717640635736	-3.08714515364499
H	-5.56638603522496	2.49987648648822	-3.57612056834839
H	-5.14366563402102	0.49590116635046	3.17460768298770

Conformer 5 (**E-ET_C5**): Gibbs Energy: -893.10780772 Hartree
Relative Gibbs Energy = 2.83 kcal/mol
Boltzmann Population: 0.5%

C	-5.09026028298606	1.58086040459321	0.42786744203682
C	-3.94652006044427	0.60740294765539	0.41062212238720
N	-5.28998840447306	2.11466666509966	-0.86170853473243
C	-5.87498812384706	1.84813869882902	1.47652906238280
H	-7.03947814474066	3.42388866363274	-3.08496191069602
H	-6.70176535165126	2.53016598131514	1.35160498585924
O	-3.70682887550355	-0.03359808857460	-0.62080835058079
S	-5.74692539520765	1.13708429504470	3.07548664733954
H	-4.76821525123611	1.63963055727440	-1.58455526557465
C	-2.10264438776019	-0.44818906766184	1.59415254950377
N	-3.21750089100110	0.48266453907337	1.52008911891367
H	-3.36204616513212	1.12289379485239	2.28471171195458
H	-2.43646770351595	-1.45979844070656	1.35882735227975
H	-1.30895362167542	-0.16723603889383	0.89810343848916
H	-1.71069594285262	-0.42987597817492	2.60942088741399
C	-6.04375523816626	3.17602869719492	-1.22983190578127
O	-6.70979057259177	3.84086911594932	-0.43316306507464
C	-6.02120539619314	3.50548456483396	-2.69837888285897
H	-5.69927876707492	4.54230578542797	-2.81498861676323
H	-5.36325971431513	2.85366752383902	-3.27292931097392
H	-6.72817326823153	1.87300076379646	3.61328542687541

Figure P3.4: Structures of conformers of *E*-configured cysteine enethiol derivative **E-ET** (wB97X-D4/def2-TZVP/def2/J, CPCM: water).

Conformer 1 (**E-ET_C1**)

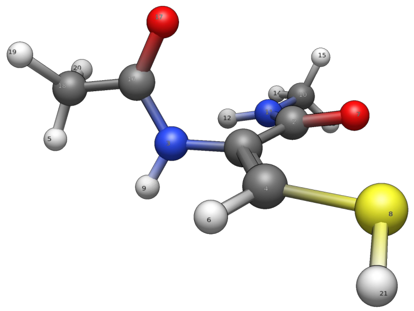

Conformer 2 (**E-ET_C2**)

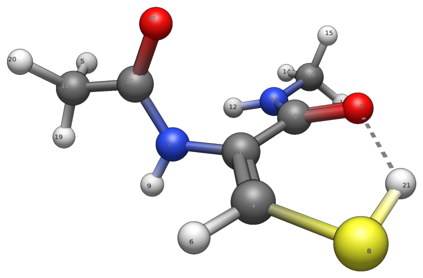

Conformer 3 (**E-ET_C3**) 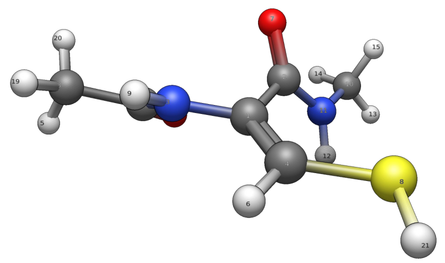	Conformer 4 (**E-ET_C4**) 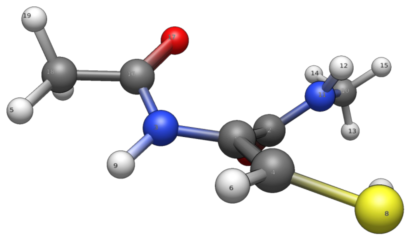
Conformer 5 (**E-ET_C5**) 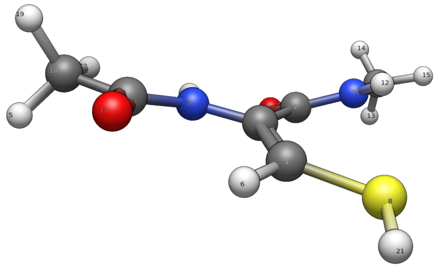	

P3.5 Cysteine E-Enethiolate

The conformational landscape of N-acetyl C-terminal methyl amide *E*-configured cysteine enethiolate **E-ETe** was explored. One main conformer was found (**E-ETe_C1**).

Table P3.5: Cartesian coordinates, Gibbs energy (Hartree), relative Gibbs energy (kcal/mol) and structure of main conformer of *E*-configured cysteine enethiolate derivative **E-ETe** (wB97X-D4/def2-TZVP/def2/J, CPCM: water).

Conformer 1 (**E-ETe_C1**): Gibbs Energy: -892.66040905 Hartree
Relative Gibbs Energy = 0.00 kcal/mol
Boltzmann Population: 100%

C	-5.36516915497433	1.39463268680759	0.45452904386449
C	-3.90938339641688	1.46786306548519	0.69343164801078
N	-5.86435344912320	2.38207872520888	-0.45652550256199
C	-6.25786061819443	0.52244359515011	0.99457733126592
H	-5.21328795348883	3.98406567804499	-3.03711812785142
H	-7.27710871461948	0.68087160664929	0.64135854564763
O	-3.20545691691857	2.35262400544742	0.15751506473503
S	-6.06892613416856	-0.75527756813014	2.11848977657019
H	-6.25768053670364	3.23438191353714	-0.08754583726603
C	-1.95225862342186	0.54923390839692	1.81448647413234
N	-3.36706463246872	0.54850403807918	1.50349218723141
H	-3.99835763433070	-0.12484438683632	1.93635661530316
H	-1.64884648495316	1.48115074607932	2.29907917719140
H	-1.74987857946567	-0.27861022504826	2.49345074383466
H	-1.34640340055374	0.41818215228670	0.91426923289603
C	-5.63325293655666	2.32966473063952	-1.77908486057812
O	-5.07984535636944	1.36209053622284	-2.31952361615421
C	-6.09071140970395	3.51546076756717	-2.58531371579794
H	-6.73793752455486	3.16543688410959	-3.39169109885323
H	-6.62219890661318	4.25345230760283	-1.98384865272012

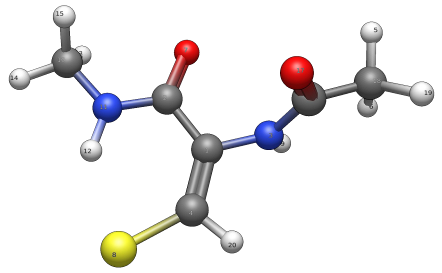

P3.6 Cysteine Z-Enethiol

The conformational landscape of N-acetyl C-terminal methyl amide *Z*-configured cysteine enethiol **Z-ET** was explored. Six conformers were found (**Z-ET_C1-C6**), with Boltzmann distributions >0.5%.

Table P3.6: Cartesian coordinates, Gibbs energies (Hartree), relative Gibbs energies (kcal/mol) and Boltzmann populations of conformers of *E*-configured cysteine enethiol derivative **Z-ET** (wB97X-D4/def2-TZVP/def2/J, CPCM: water).

Conformer 1 (**Z-ET_C1**): Gibbs Energy: -893.11585557 Hartree
Relative Gibbs Energy = 0.00 kcal/mol
Boltzmann Population: 43.0%

C	-4.56662437290449	0.86817622271955	-0.65293033435193
C	-3.49754379539766	0.92351384466770	0.38914960170073
N	-5.83115382750383	1.44493637302081	-0.36285660214211
C	-4.29280925019425	0.30771133505860	-1.83250184106610
H	-8.05847757619170	2.44472800523269	0.28864245297987
H	-3.29980111552427	-0.09586820130194	-1.99639667430093
O	-2.40731321080895	0.35400586715275	0.23371644220849
S	-5.34073289554103	0.13622828965302	-3.19639184860627
H	-6.44760988085248	0.96427179249670	0.27709632248784
C	-2.82583160573594	1.76268295828455	2.57372366384784
N	-3.78470776766860	1.62315374934510	1.49221147819646

Conformer 2 (**Z-ET_C2**): Gibbs Energy: -893.11567177 Hartree
Relative Gibbs Energy = 0.12 kcal/mol
Boltzmann Population: 35.4%

C	-4.47959418262050	1.24214180115943	-0.78751356491456
C	-3.53005769039940	1.08173544890684	0.35186094091440
N	-5.83571692172375	1.57940770259801	-0.52256241564418
C	-4.05493970176510	1.06575686379464	-2.03844445577786
H	-8.27225514590781	2.16232496355864	-0.14820151516704
H	-3.02806024503750	0.78187339901326	-2.22999756398691
O	-2.32052727438022	0.88604549754211	0.16179689554273
S	-5.10467862269148	1.27712594365813	-3.40574457260246
H	-6.54486328755114	0.86648002312146	-0.62219338081279
C	-3.23267740053592	1.04588027276647	2.76766953137935
N	-4.05758656135272	1.15426561109827	1.57779408146104

H	-4.67344535384069	2.09117756907315	1.56246529343065
H	-2.54880346195435	0.78549404054933	2.97428605313761
H	-1.92087774271108	2.26961686412055	2.23162400259414
H	-3.28502136652446	2.35263982500702	3.36478744710736
C	-6.19379205413041	2.67946871504760	-0.79701842478359
O	-5.45946129479091	3.35907247355728	-1.51358518753805
C	-7.55083512752032	3.15655473907833	-0.36199163801131
H	-7.43331979981807	4.10940901826624	0.15770898970246
H	-8.15704167544497	3.33049432168236	-1.25357628056130
H	-6.42941426804134	0.71169605518857	-2.66327486253186

H	-5.04399183439989	1.32890608936112	1.67790790382975
H	-2.73074200627158	0.07701217532639	2.80695123153388
H	-2.47567367075892	1.83293914582161	2.78975752007402
H	-3.87507555097183	1.14731965214395	3.64048408577136
C	-6.22432527787118	2.86088711464900	-0.30603003409479
O	-5.40714485371489	3.78163043415885	-0.26042649456422
C	-7.69764834829827	3.08792869987109	-0.12174626630756
H	-7.85519797496489	3.58974295255462	0.83485231566568
H	-8.04438766921888	3.75456821139210	-0.91429812910320
H	-4.18947322266395	0.97519185540397	-4.33702805969665

Conformer 3 (**Z-ET_C3**): Gibbs Energy: -893.11456658 Hartree
Relative Gibbs Energy = 0.81 kcal/mol
Boltzmann Population: 11.0%

C	-4.66392225461810	1.64655780949648	-0.64788116161215
C	-4.03405335152822	1.84714110258483	0.69675485413558
N	-5.87231036187451	2.33739634892880	-0.87673120489664
C	-4.06013143652558	0.96051866910128	-1.61849832707814
H	-9.00941914388014	2.57479727379171	-0.24697581975398
H	-3.08253324597750	0.52270115067679	-1.46434535229608
O	-4.13946963311008	2.92888644432817	1.28801786046170
S	-4.76874801733005	0.76059459642697	-3.20147815265071
H	-5.94031656920274	2.95451174459443	-1.67347873802313
C	-2.62767897887400	0.91348809349948	2.45126112476837
N	-3.35147210489517	0.81362145146677	1.19596286609144
H	-3.35810492996973	-0.06276185457939	0.70006795920913
H	-2.11938652727785	-0.03241849513131	2.62939317165855
H	-1.88555593379120	1.71319707305017	2.40817390048576
H	-3.30977408479928	1.11223746527370	3.28088853991882
C	-6.92116144566259	2.26057223283776	-0.02223036857826
O	-6.90357057232090	1.50245132313853	0.94873231213909
C	-8.08921015567589	3.15553978829395	-0.32210164892371
H	-8.11899898872766	3.93985688932871	0.43877704903228
H	-8.02032877649773	3.61773223926772	-1.30709865158299
H	-3.72847093096089	0.11254251192440	-3.74232215890493

Conformer 4 (**Z-ET_C4**): Gibbs Energy: -893.11435159 Hartree
Relative Gibbs Energy = 0.94 kcal/mol
Boltzmann Population: 8.8%

C	-4.80251956476312	1.12508236146662	-0.56546192059710
C	-4.40228458798168	1.06839404834500	0.87971254797342
N	-6.04363448507462	1.73684142447531	-0.83069225957089
C	-4.08316920547685	0.51703742716464	-1.51025912158578
H	-8.28705706008790	2.82049416834013	-1.32939443733164
H	-3.18902062483361	-0.03101673926815	-1.23606261104605
O	-5.25369766664856	0.90267440548627	1.76187186531912
S	-4.42534260434093	0.42869669706745	-3.21532215177239
H	-6.74208722071103	1.22608162201478	-1.35114622897849
C	-2.58805170649055	1.06898393334279	2.50135668444631
N	-3.09959042755253	1.18809645533752	1.14714381573945
H	-2.46000194680941	1.38449167166465	0.39444642466406
H	-2.83141401327894	0.09205390049057	2.92407569030261
H	-1.50597403507698	1.18222225627718	2.47023097674954
H	-3.00825805238166	1.84427350034582	3.14573338755179
C	-6.37227503163123	2.95163579198197	-0.32553914020872
O	-5.55454149947639	3.63066479127275	0.29653797178175
C	-7.78584711433188	3.40240363934660	-0.55577303192537
H	-8.33384387810453	3.28783105155247	0.38349527014121
H	-7.78515188599998	4.45917610313791	-0.82373585433581
H	-5.45085483224746	1.29304534825765	-3.24632982371699

Conformer 5 (**Z-ET_C5**): Gibbs Energy: -893.1122588 Hartree
Relative Gibbs Energy = 2.26 kcal/mol
Boltzmann Population: 1.0%

C	-4.69194003521823	1.74562198371594	-0.35855475890375
C	-3.99534727075560	1.74069509484490	0.96682552308203
N	-5.86035674660829	2.53607901957788	-0.36895253488862
C	-4.17657368106841	1.16596546838293	-1.44471857478475
H	-7.91041266334145	3.98366462460000	-0.06406645537575
H	-3.25607145210819	0.60160050037836	-1.37158397808488
O	-4.18029643203525	2.68110771024776	1.75284859315335
S	-4.87448958659280	1.26031520119112	-3.03887884382605
H	-5.83501646216163	3.35316613235563	0.22468374753994
C	-2.41598441470568	0.67896431022745	2.48249718550517
N	-3.18237000175156	0.71964276216508	1.24844656494000
H	-3.13271195321775	-0.06422807535750	0.61922226489627
H	-3.07482558495007	0.63738353386376	3.35275230553193
H	-1.79180219795191	-0.21256795251324	2.46749855903229
H	-1.77628337793741	1.55926949944069	2.56858593316174
C	-7.07933645058358	2.11177409543673	-0.79156255648764
O	-7.25620215880465	1.00224549684687	-1.29317861925699
C	-8.20456405093411	3.09500837188172	-0.62262411798729
H	-8.55037246834405	3.39313263957984	-1.61530077397638
H	-9.03072067125727	2.59516442554567	-0.11412039184918
H	-3.72893978287186	0.99515901578831	-3.68493101802145

Conformer 6 (**Z-ET_C6**): Gibbs Energy: -893.1121135 Hartree
Relative Gibbs Energy = 2.35 kcal/mol
Boltzmann Population: 0.8%

C	-4.54045097850654	1.75300798729807	-0.41572146577551
C	-3.96464987168113	1.69974838777831	0.96488284955842
N	-5.71764986750741	2.53285137690570	-0.51090759121910
C	-3.92978728629860	1.21107189508315	-1.47154306636725
H	-8.47451330596131	3.16171876479300	-1.76710282636085
H	-3.01953327916442	0.63932696695884	-1.33078644204076
O	-4.22221659055270	2.60916006574073	1.76652136795379
S	-4.36106413909904	1.32901425222375	-3.14676089823666
H	-5.71544093299953	3.40109206587027	0.00511930032156
C	-2.52648885203104	0.58322838557273	2.57895495093123
N	-3.17742615752346	0.66810005319813	1.28245342523245
H	-3.06890164318299	-0.09368467955626	0.63390042654346
H	-1.91196966452717	-0.31501959040857	2.59437540223369
H	-1.88871857478779	1.45279109694708	2.74901506252261
H	-3.26192303785546	0.52489605814967	3.38448935809281
C	-6.92817581938259	2.02802669786475	-0.86678103555671
O	-7.06733708766531	0.87651660592272	-1.27758143093749
C	-8.08840207122871	2.97936677517317	-0.76141308061100
H	-8.87779109944852	2.50103280615682	-0.17900597110171
H	-7.81795490882807	3.93075238481127	-0.30267327227633
H	-5.44422227496796	2.10616550161658	-3.01454700940663

Figure P3.6: Structures of conformers of *Z*-configured cysteine enethiol derivative **Z-ET** (wB97X-D4/def2-TZVP/def2/J, CPCM: water).

Conformer 1 (**Z-ET_C1**) 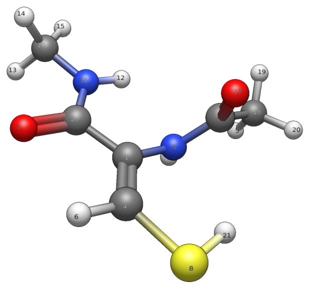	Conformer 2 (**Z-ET_C2**) 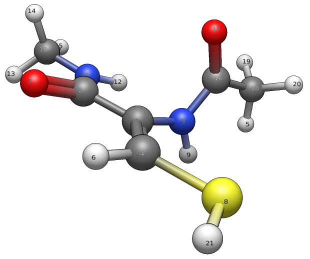
Conformer 3 (**Z-ET_C3**) 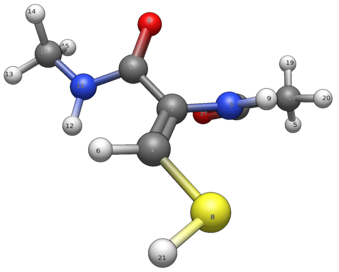	Conformer 4 (**Z-ET_C4**) 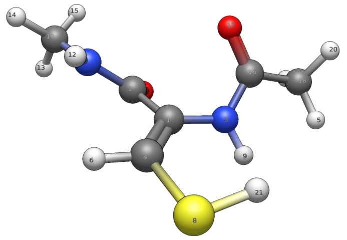
Conformer 5 (**Z-ET_C5**) 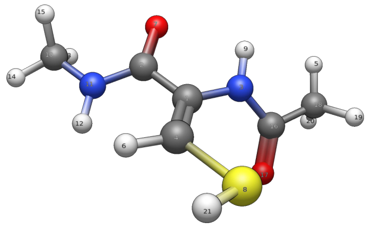	Conformer 6 (**Z-ET_C6**) 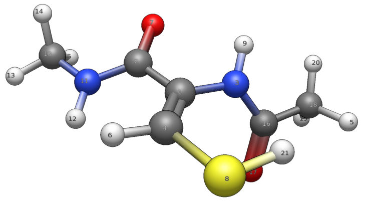

P3.7 Cysteine Z-Enethiolate

The conformational landscape of N-acetyl C-terminal methyl amide *Z*-configured cysteine enethiolate **Z-ETe** was explored. One main conformer was found (**Z-ETe_C1**).

Table P3.7: Cartesian coordinates, Gibbs energy (Hartree), relative Gibbs energy (kcal/mol) and structure of main conformer of *Z*-configured cysteine enethiolate derivative **Z-ETe** (wB97X-D4/def2-TZVP/def2/J, CPCM: water).

Conformer 1 (**Z-ETe_C1**): Gibbs Energy: -892.66067298 Hartree
Relative Gibbs Energy = 0.00 kcal/mol
Boltzmann Population: 100%

C	-5.59846558192806	0.74057003562074	0.34297691302948
C	-4.33256259838504	0.54744259739729	1.06281366444835
N	-5.82037801821858	1.97727839405032	-0.33035153808761
C	-6.58752502799278	-0.18768793751901	0.39227036187722
H	-4.92010820742807	4.21573375642975	-2.31494912060157
H	-6.35848921887580	-1.07184061929506	0.98409159752711
O	-4.01150140457556	-0.54573948113727	1.57617007429215
S	-8.12659385994307	-0.08525987639049	-0.33236761246754
H	-6.68160345289635	2.45806357393555	-0.11093845136526
C	-2.21833694553489	1.52857090906370	1.80180642900654
N	-3.51896071598202	1.61523706862272	1.16740734490349
H	-3.78560412012263	2.47198651049679	0.71337245936664
H	-1.73992549741224	2.50496202684147	1.73833631777182
H	-2.30914327724682	1.25138387894307	2.85459409377243
H	-1.58179958144121	0.79233245665759	1.30403377778014
C	-5.25357611002539	2.27589857915724	-1.51797879289856
O	-4.35098906156979	1.58949055257521	-2.01243406235940
C	-5.75379543441285	3.51929149847545	-2.20144009676251
H	-6.10516708152919	3.24956237092925	-3.19949201415128
H	-6.55859611657943	4.00672871084562	-1.65096943518164

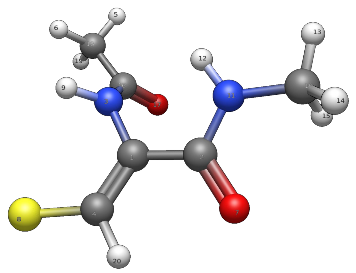

P 3.8 Cysteine Hemi-thioacetal

The conformational landscape of N-acetyl C-terminal methyl amide *R*-configured cysteine hemi-thioacetal **CTA** was explored. Eleven conformers were found (**CTA_C1-C12**), with Boltzmann distributions >3.0%.

Table P3.8: Cartesian coordinates, Gibbs energies (Hartree), relative Gibbs energies (kcal/mol) and Boltzmann populations of conformers of *R*-configured cysteine hemi-thioacetal derivative **CTA** (wB97X-D4/def2-TZVP/def2/J, CPCM: water).

Conformer 1 (**CTA_C1**): Gibbs Energy: -969.59600758 Hartree
Relative Gibbs Energy = 0.00 kcal/mol
Boltzmann Population: 26.0%

C	-5.95656236107814	1.89832505799660	0.53431698992651
C	-4.95413307299860	1.44535498596420	1.59530301153379
N	-5.43315771364065	1.92109392109542	-0.80763892733315
C	-7.23151401294618	1.03877390386166	0.60418496431081
H	-6.24483380152976	2.91689213201184	0.80231350150251
H	-7.90468680063130	1.36670074527722	-0.18918957481751
O	-5.34710596431191	1.26616482248170	2.76149518705049
S	-6.89678139483460	-0.71403741486508	0.20287288949180
H	-5.40245837187333	1.05246321693856	-1.32154123039091

Conformer 2 (**CTA_C2**): Gibbs Energy: -969.59548612 Hartree
Relative Gibbs Energy = 0.33 kcal/mol
Boltzmann Population: 15.0%

C	-5.69313912193246	2.02815109077488	0.80236092003574
C	-4.67088109964030	1.54312348807632	1.83525624340117
N	-5.29827236999566	1.79655436026696	-0.56510012281670
C	-7.04770476622046	1.38706998052026	1.09892864786131
H	-5.78071137427589	3.10474827650275	0.94989754354612
H	-7.31671424657786	1.60718497194128	2.13438118008504
O	-4.94820208117036	1.59942506040189	3.03913703885661
S	-8.38588527583547	2.11649216349167	0.11536642298280
H	-5.37499287302222	0.85891603041128	-0.93058843893940

C	-2.66934783691170	0.85438590292368	2.17434512191032
N	-3.69454392519192	1.26000684748116	1.22601338583019
H	-3.44138512759654	1.41240866883974	0.26287289912085
H	-2.58002522497996	1.58478462202336	2.98053860263987
H	-2.90652343996739	-0.11992640763046	2.60559532690235
H	-1.72112637643210	0.78912964079099	1.64474261530853
C	-4.93706147216256	3.03874718599059	-1.38526115165325
O	-4.90127656778648	4.12117426821512	-0.79502980820914
C	-4.42275949484803	2.88633716275857	-2.78998394876408
H	-3.35614648876305	3.12152114552737	-2.79246685553703
H	-4.57330670110417	1.88352179548642	-3.18990807754386
H	-4.92969712374442	3.61440819520714	-3.42600215486318
O	-7.89743244554630	1.21324375357013	1.82924457475091
H	-7.20686489832918	1.18510109476407	2.51824996759424
H	-6.03427939949153	-0.94122966741004	1.20633555963892

C	-2.44355139327522	0.67455366284385	2.27875303206493
N	-3.50016358139349	1.10560720357819	1.37892547530343
H	-3.33677054602481	1.10011552995352	0.38509275129164
H	-2.14191479162633	1.48894975627831	2.94061159889491
H	-2.77447051775701	-0.16863559153563	2.88809819434748
H	-1.58790309339573	0.36669051860096	1.68081907626602
C	-4.91588281522239	2.78625415534505	-1.40184930013367
O	-4.82771942388875	3.96075608145313	-1.03537576440498
C	-4.60168759762483	2.37265661769052	-2.81291024958678
H	-4.67125315189497	1.29516338305844	-2.96128380031399
H	-5.30125324530031	2.87746137373919	-3.48284188033968
H	-3.59400709787466	2.71259763451076	-3.05899048709762
O	-6.89749087003230	-0.00336473552979	0.89587455689596
H	-7.71310451164163	-0.44444100165755	1.16305008195739
H	-7.81933417117668	1.92931556868370	-1.08620985155773

Conformer 3 (**CTA_C3**): Gibbs Energy: -969.59507407 Hartree
Relative Gibbs Energy = 0.59 kcal/mol
Boltzmann Population: 9.7%

C	-5.27419002950205	1.86119846287082	0.48596691997069
C	-4.04809560366264	1.15861705965631	1.08293495541365
N	-5.23816615493933	1.75907974418911	-0.94995215497372
C	-6.54789927399631	1.24375667269469	1.08062747916825
H	-5.24993230875645	2.91567594066843	0.75624245222817
H	-6.55402053894681	1.42081151121734	2.16045881992736
O	-3.38615501651217	0.34692538691280	0.43079487356069
S	-8.05848600996333	2.06270416777715	0.49757075130671
H	-5.03132805646985	0.85505326381174	-1.34497132845247
C	-2.68176716422495	0.84257534047848	3.07626038955586
N	-3.76711523088571	1.47701619120232	2.34703521742679
H	-4.34924992247414	2.15036236825054	2.82035386370377
H	-1.72327477410700	1.03589004632761	2.59057690729133
H	-2.65812211082822	1.25577332422378	4.08286501923178
H	-2.83364560632506	-0.23709476124771	3.13740146095181
C	-5.32382272178619	2.82133449406332	-1.77504570060553
O	-5.48757972104132	3.97437280590272	-1.36333251586647
C	-5.22279724408108	2.51954985752596	-3.24543098758257
H	-4.45049450372902	3.15622631484669	-3.68063152919368
H	-4.99205954042809	1.47331177590432	-3.44616559000353
H	-6.17538426053048	2.77562292888842	-3.71502942720237
O	-6.51648985198773	-0.13881728761006	0.79452875071081
H	-7.25845266336447	-0.56290335746376	1.24264274984575
H	-7.81448170825732	1.92830332840889	-0.81429850771308

Conformer 4 (**CTA_C4**): Gibbs Energy: -969.59506581 Hartree
Relative Gibbs Energy = 0.59 kcal/mol
Boltzmann Population: 9.6%

C	-5.16355861334381	1.99789315583798	0.31062062865073
C	-4.10387935817453	1.11815327050878	0.97402119877766
N	-5.02421347473501	1.88322682442168	-1.11727047541766
C	-6.59243714718609	1.64060424553196	0.76044754328755
H	-4.99891104849963	3.03650424030119	0.60579834921716
H	-7.28223006931919	2.16569651760267	0.09446421614257
O	-3.49120455038163	0.26119033626628	0.32847843555793
S	-7.01546901440938	-0.11212369109215	0.53176824625071
H	-4.56023557302862	1.05960993732403	-1.46891789372990
C	-2.98195847398720	0.51244786710938	3.04578536372955
N	-3.89821731880926	1.33388696926332	2.27204693187168
H	-4.47969984335111	2.00706940606169	2.74573718929951
H	-2.97986585628547	0.87564878086202	4.07173150525083
H	-3.29305431247959	-0.53468109257799	3.03781159932921
H	-1.97012576160292	0.58144016784888	2.64252516462590
C	-5.46701186774044	2.82554071647768	-1.96653043162763
O	-6.03230216401347	3.85299834519200	-1.57104607695614
C	-5.23786904028232	2.56499327578628	-3.42958761532042
H	-4.70931235611713	1.62927520679024	-3.61189623913463
H	-6.20706600975917	2.53860800575940	-3.93232351690570
H	-4.66494321030931	3.39610876625718	-3.84567379211585
O	-6.74367636096482	2.08465707740491	2.09045755004035
H	-7.67175649583314	1.99458113123898	2.33961169546666
H	-6.07401209638664	-0.61798388057645	1.34334329210992

Conformer 5 (**CTA_C5**): Gibbs Energy: -969.59472174 Hartree
Relative Gibbs Energy = 0.81 kcal/mol
Boltzmann Population: 6.7%

C	-5.85529430451286	1.97786582527515	0.57844462715208
C	-4.81204146781061	1.60723231588312	1.63434063168328
N	-5.41083642482801	1.85370497002456	-0.78800503996291
C	-7.14275437406477	1.16582868637367	0.80231085986901
H	-6.11114176919411	3.02271653724394	0.76670887382770
H	-7.84793686270109	1.40684722547952	0.00865076965068
O	-5.14140270833720	1.63258948405297	2.83238380206395
S	-6.74009622793324	-0.61733392333349	0.66058666834504
H	-5.60859425225051	0.99748579430737	-1.28452932732806
C	-2.54457756745563	0.92493825107404	2.17529459600351
N	-3.59439649772475	1.27154722928464	1.23041369455068
H	-3.38859261636357	1.26993378976980	0.24427207354041
H	-2.33813891479928	1.76112710663435	2.84598535665425
H	-2.83449202703238	0.05774339657138	2.77129871263223
H	-1.64388492110462	0.68710449348798	1.61300194204166
C	-4.79799895298656	2.86228685012440	-1.44710032844280
O	-4.53291266207023	3.93412479396953	-0.89671705598033
C	-4.44963755926906	2.59939607885236	-2.88520124367603
H	-3.36890448128186	2.70107121236698	-3.00363413762758
H	-4.76103481588124	1.60990313283268	-3.21962819240804
H	-4.93008063322192	3.36130575703437	-3.50232882841158

Conformer 6 (**CTA_C6**): Gibbs Energy: -969.59464197 Hartree
Relative Gibbs Energy = 0.86 kcal/mol
Boltzmann Population: 6.1%

C	-5.91407246198821	2.06406252226330	0.81436506168521
C	-4.85493698169889	1.71729443379117	1.86574308893244
N	-5.39419246205344	2.45348897749449	-0.48802307216857
C	-6.97213530516593	0.94368739579412	0.79667404718514
H	-6.41339434613622	2.95316385359346	1.20365937313165
H	-7.51636129154493	0.99575032537875	1.73666685924606
O	-5.22453128841854	1.44858816946304	3.01461155346207
S	-8.15431597235344	1.31685020337475	-0.54585581198153
H	-5.39791574733418	3.44176831323512	-0.68751511070856
C	-2.51254066569577	1.45029300580518	2.45353454425947
N	-3.57462624793398	1.74970960819535	1.50714346211101
H	-3.33066428369029	1.97764313992150	0.55813621805413
H	-2.63158899214355	0.44513929169782	2.86248264477747
H	-1.56060890214256	1.51076916668545	1.92948474933871
H	-2.51315873834554	2.16712628528242	3.27710731392144
C	-4.92294205140616	1.63729805033520	-1.44979518223672
O	-4.86690269711091	0.40693300332967	-1.31889599079107
C	-4.47124399716946	2.29887141373062	-2.72002019994197
H	-5.17751453625629	2.03034544846494	-3.50961689835728
H	-4.42041473984921	3.38412809196658	-2.63801214303181
H	-3.49256462776144	1.90104921398049	-2.99196404511762

O	-7.75449317300399	1.49859005451858	2.02280408832132
H	-7.04181278033647	1.52942216892502	2.68768006744642
H	-7.99195402293582	-1.02008565165302	0.40437025875506

O	-6.42561754805984	-0.34916077465479	0.71810824118133
H	-5.79898000920605	-0.32885378938699	-0.03534984536411
H	-9.10178612363504	0.47940022955831	-0.10126598888688

Conformer 7 (**CTA_C7**): Gibbs Energy: -969.59431086 Hartree
Relative Gibbs Energy = 1.06 kcal/mol
Boltzmann Population: 4.3%

C	-5.43491212916358	1.56715260266469	0.44090815181718
C	-4.35730091115287	0.72343948556412	1.11261086362172
N	-5.06347296175794	1.73213281601905	-0.95138752664775
C	-6.80613314917671	0.89067661830411	0.58692213213272
H	-5.48339390326018	2.55897647204418	0.89389277760048
H	-6.74721685466662	-0.13964552671074	0.23064135683683
O	-4.14271054965889	-0.42981134988394	0.72306287114787
S	-7.25264649873711	0.73662922842254	2.34552538904468
H	-4.55877299437059	0.98431859670952	-1.40340944898380
C	-2.62846343421743	0.59573526024422	2.81693590381683
N	-3.66343204498330	1.30746408472271	2.08598097309403
H	-3.91752789127653	2.23944945071916	2.37302956115932
H	-1.84370900101790	0.25626193371137	2.13867632422844
H	-2.19620044138043	1.27532700083006	3.54879180409804
H	-3.04491426704113	-0.27091302918050	3.33515925825038
C	-5.42810180681108	2.80983199378544	-1.66916281490858
O	-6.10773510207683	3.72325839912455	-1.18174836579974
C	-4.96621967562680	2.85035251220016	-3.09832742296213
H	-5.84623624838398	2.87566249035066	-3.74461720640738
H	-4.40560058207668	3.77391094243755	-3.25555785719485
H	-4.34417488800228	1.99570561443940	-3.36454802264070
O	-7.78815489327711	1.53636990660303	-0.18981035698226
H	-7.60825151663876	2.48834035440236	-0.19885481482421
H	-7.00772827214514	2.01471972197628	2.67668933880287

Conformer 8 (**CTA_C8**): Gibbs Energy: -969.59407182 Hartree
Relative Gibbs Energy = 1.21 kcal/mol
Boltzmann Population: 3.3%

C	-5.66601970503935	1.68046991695723	0.41712909965496
C	-4.41877890110147	0.93502523757192	0.90010105500233
N	-5.60133603518105	1.91477871387609	-1.01148928793528
C	-6.91351637440641	0.82571770800166	0.69003188181234
H	-5.76230481087615	2.63188627022458	0.95103247899899
H	-6.79818077158383	-0.13873652322413	0.19111951281378
O	-4.02906492081297	-0.07931405112446	0.31281482058747
S	-7.09442917703565	0.41821770987534	2.44718990312034
H	-6.39092172222905	1.63499384517371	-1.57166226606068
C	-2.69235093294841	0.74666226138837	2.60124866923069
N	-3.82096185172851	1.42167155309398	1.98538538847627
H	-4.21062308588273	2.23559106674743	2.43318246364032
H	-2.97149091144193	-0.25656534379705	2.93241738004154
H	-1.86098529300513	0.66578081122746	1.89822374755312
H	-2.37178345492786	1.32945210029094	3.46298055925577
C	-4.58308241504229	2.56876382833060	-1.59326103211095
O	-3.62161467610152	2.98833712936727	-0.93780681168465
C	-4.66881986832415	2.75807536857966	-3.08247882574783
H	-5.58549585198065	2.34878283069855	-3.50743542908579
H	-4.61012345242843	3.82620749149783	-3.30121318800936
H	-3.80624386676898	2.27174034899564	-3.54302124660505
O	-8.06429699775059	1.41800233856607	0.12981844988385
H	-8.19671717847337	2.28675838400813	0.53284460516250
H	-6.90386776182938	1.66304658297318	2.91425094080530

Conformer 9 (**CTA_C9**): Gibbs Energy: -969.59404126 Hartree
Relative Gibbs Energy = 1.23 kcal/mol
Boltzmann Population: 3.2%

C	-5.64073434143639	1.83654072370320	0.67229050237242
C	-4.63633143304788	1.09164751783876	1.55417470382424
N	-5.35266950768896	1.54786593740813	-0.71680093221094
C	-7.07485653430725	1.43556356897040	1.03914119380176
H	-5.52766023667036	2.90814925208805	0.82219696671676
H	-7.22825851477919	1.66457351728050	2.10006888977894
O	-4.81336214245679	-0.09532466560533	1.87162619815944
S	-8.29731283673989	2.43680866559540	0.17331515530599
H	-5.55746989700510	0.61956879689340	-1.05491713884619
C	-2.48483135876881	1.18284723974908	2.69042655657661
N	-3.56219209740892	1.78330701639591	1.92012979647302
H	-3.47786274861698	2.74120259398663	1.61719904296789
H	-1.71970435567995	1.93867611450178	2.85600080501678
H	-2.85206632203788	0.82954977732905	3.65561435020959
H	-2.04594123248874	0.34142193974925	2.15063227292863
C	-4.94334912245206	2.48483666568857	-1.59734545365393
O	-4.71030766166505	3.65071036814444	-1.26442459746592
C	-4.78555449018225	2.02443227185608	-3.02043769827887
H	-4.96446193034684	0.95575845766796	-3.13950923895017
H	-5.49126698050387	2.58129748578488	-3.64096658078601
H	-3.77592935350501	2.26694785201254	-3.35699707929379
O	-7.30859788642719	0.06271783207867	0.78151878590255
H	-6.56043689417056	-0.40199255109568	1.20296446544340
H	-7.83185213831389	2.20823920127825	-1.06449809739219

Conformer 10 (**CTA_C10**): Gibbs Energy: -969.59402186 Hartree
Relative Gibbs Energy = 1.25 kcal/mol
Boltzmann Population: 3.2%

C	-5.53938432579180	2.08452873602862	0.54978100542481
C	-4.15588369124655	1.77135615091635	1.14262142110354
N	-5.44549643571600	2.47259701386473	-0.84460899126325
C	-6.50318785200762	0.91603262417573	0.84270255995335
H	-5.93636500572711	2.95448142945647	1.07470291953649
H	-6.76179758662222	0.95374449685418	1.89958170512043
O	-3.16941915846172	1.57001438644279	0.43249629379842
S	-8.04199643623211	1.21026196264411	-0.09875680750015
H	-5.44513533797978	3.46383768417346	-1.02683488708662
C	-2.88817355152249	1.41658506350190	3.19401815674320
N	-4.10974372619148	1.73920029981945	2.47603545971095
H	-4.94742230579041	1.93757721494028	3.00025970900998
H	-2.10644738670400	2.14878229071100	2.98110778418013
H	-3.10436359599639	1.42982079349212	4.26063705682826
H	-2.52547689447286	0.42458756140097	2.91707433162796
C	-5.15328954259253	1.66704068017984	-1.88008255293899
O	-5.08123187440213	0.43304192781283	-1.78360449517562
C	-4.91905711925925	2.34649955873544	-3.19941288410915
H	-5.19799300228707	3.39994115901506	-3.18645635702882
H	-3.85515574248326	2.26202918807447	-3.43604929425306
H	-5.48089620514545	1.82167818709855	-3.97297796382073
O	-5.93815213828793	-0.34944750267328	0.60984076919603
H	-5.56646059764130	-0.30605018445802	-0.29912176840659
H	-8.77048050433842	0.32720485749290	0.59844969814942

Conformer 11 (**CTA_C11**): Gibbs Energy: -969.59395407 Hartree
Relative Gibbs Energy = 1.29 kcal/mol
Boltzmann Population: 3.0%

C	-5.14261669760498	1.95486561669354	0.34515439201001
C	-3.99444182089720	1.19968552477805	1.01623460131322
N	-4.95033894743052	1.90576991894055	-1.08091415624867
C	-6.51625106255332	1.39091847878976	0.75028349948991

H	-5.13062566115914	2.99489610766976	0.67629182005710	
H	-7.26135163821895	1.79281172995953	0.06311880113631	
O	-3.29584308848874	0.40756131393400	0.37467914717626	
S	-6.52091660835589	-0.42596036601038	0.56132651270863	
H	-4.36588502603963	1.16712586368829	-1.44194138424414	
C	-2.83690431769177	0.71165169215989	3.09947900708165	
N	-3.82004843622120	1.43902323687808	2.31389831396222	
H	-4.46640999320237	2.05581176893026	2.78025046752158	
H	-2.88078696059705	1.07580607414411	4.12409733223123	
H	-3.04665523694356	-0.36037094036875	3.09138462485159	
H	-1.83214154712493	0.87642826934873	2.70662627378758	
C	-5.49154221386286	2.81023545695298	-1.91460581342457	
O	-6.20082353817548	3.73790983884036	-1.50527823101972	
C	-5.18634961467430	2.63564094964313	-3.37647494883020	
H	-4.54153572722378	1.77860249442000	-3.57094718946944	
H	-6.12797845897077	2.51265870758649	-3.91560783208581	
H	-4.70486497434161	3.54509138455081	-3.74180585145429	
O	-6.77625092269014	1.81717640360737	2.06926829466700	
H	-7.69681844061459	1.62056788851653	2.28053527708365	
H	-7.85562908381709	-0.54856183445314	0.54634991039888	

Figure P3.8: Structures of conformers of *R*-configured cysteine hemi-thioacetal derivative **CTA** (wB97X-D4/def2-TZVP/def2/J, CPCM: water).

Conformer 1 (**CTA_C1**)

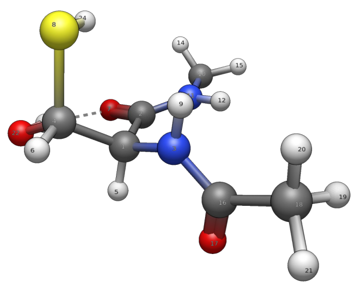

Conformer 2 (**CTA_C2**)

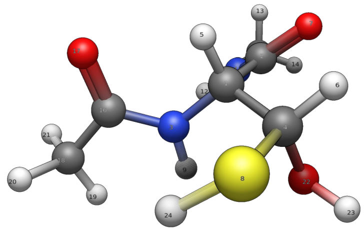

Conformer 3 (**CTA_C3**)

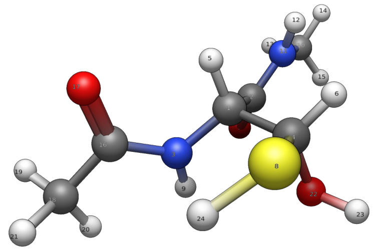

Conformer 4 (**CTA_C4**)

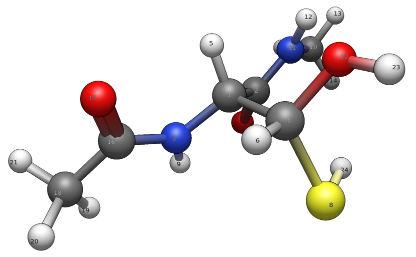

Conformer 5 (**CTA_C5**)<br>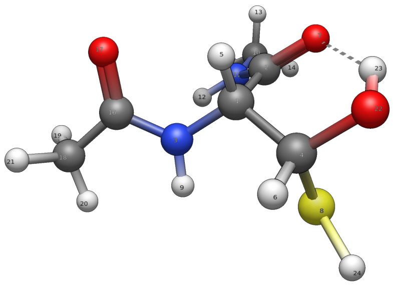	Conformer 6 (**CTA_C6**)<br>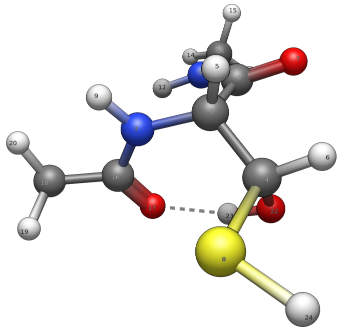
Conformer 7 (**CTA_C7**)<br>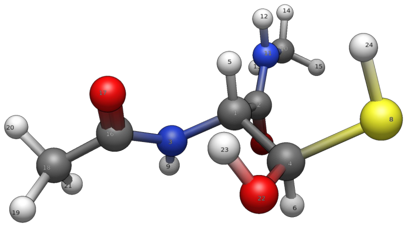	Conformer 8 (**CTA_C8**)<br>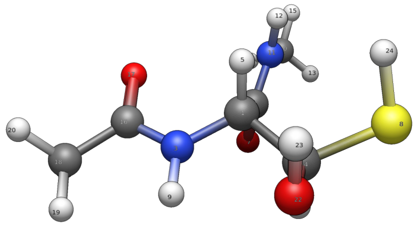
Conformer 9 (**CTA_C9**)<br>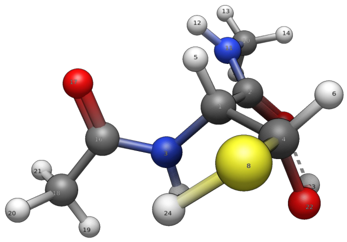	Conformer 10 (**CTA_C10**)<br>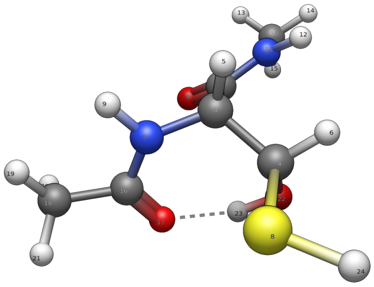

Conformer 11 (**CTA_C11**)	
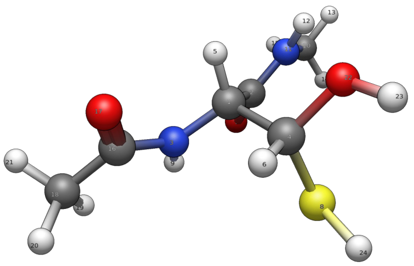	

P3.9 Cysteine Hemi-thioacetal Conjugate Base

The conformational landscape of N-acetyl C-terminal methyl amide *R*-configured cysteine hemi-thioacetal conjugate base **CTA-CB** was explored. Seven conformers were found (**CTA-CB_C1-C7**), with Boltzmann distributions >1.0%.

Table P3.9: Cartesian coordinates, Gibbs energies (Hartree), relative Gibbs energies (kcal/mol) and Boltzmann populations of conformers of *R*-configured cysteine hemi-thioacetal conjugate base derivative **CTA-CB** (wB97X-D4/def2-TZVP/def2/J, CPCM: water).

Conformer 1 (**CTA-CB_C1**): Gibbs Energy: -969.13262542 Hartree
Relative Gibbs Energy = 0.00 kcal/mol
Boltzmann Population: 71.8%

C	0.09740339092320	-0.37555894400827	-0.68199012445217
C	0.29372950331716	1.13097585555804	-0.49307578939222
N	0.88506057139299	-1.16539906558127	0.24062440591824
C	-1.38590338284844	-0.79177506797040	-0.54678417603679
H	0.42057348636339	-0.58686804711203	-1.70274243025299
H	-1.46534087198721	-1.79001309175073	-0.98567286225300
O	0.69593510090237	1.84001063428510	-1.42665886198672
S	-2.00167902723851	-0.82245442277295	1.14626489182869
H	0.38189154036993	-1.51058590750126	1.05031157428736
C	0.03801437942988	3.03817744416592	0.99182065028968
N	0.01248158248317	1.61256417201885	0.71944410674193
H	-0.54096981241125	0.99470863747895	1.31451116513859
H	-0.68284682350166	3.57899164005000	0.37178096085155
H	-0.20994461555494	3.19490350973899	2.04076162395978
H	1.03241297106453	3.44561010470333	0.80255851644512
C	2.22520286892895	-1.19902419837191	0.21214361694827
O	2.88016301884482	-0.62988676534904	-0.67308379884628
C	2.90079352799832	-1.97250309066008	1.31160614838944
H	2.19136255171051	-2.43434793812960	1.99874860249036
H	3.52837993696678	-2.74482949289611	0.86219620857453
H	3.55088091862456	-1.29067297651318	1.86436581062872
O	-2.15293735164871	0.08303651639740	-1.39307150772388
H	-2.65476051692992	0.64594982442020	-0.79013059554822

Conformer 2 (**CTA-CB_C2**): Gibbs Energy: -969.13109369 Hartree
Relative Gibbs Energy = 0.96 kcal/mol
Boltzmann Population: 14.2%

C	0.01443059281195	-0.12126938155614	-0.76223981964702
C	-0.02706802282694	1.35200660766901	-0.38099835754641
N	0.75896590271438	-0.94832891787418	0.16071918572811
C	-1.43428507419509	-0.64515454419908	-0.89182862198287
H	0.46850288096317	-0.16090730410708	-1.75578933620629
H	-1.88360186747751	-0.08820086782124	-1.71744534145691
O	-0.38975985903022	2.18463144411207	-1.22651344436213
S	-1.52349910940548	-2.41383379679275	-1.17887128035929
H	0.29149900962036	-1.81733251954978	0.40015266486896
C	0.25310014159081	3.06927221096048	1.32158875260873
N	0.29664723044618	1.69224345595416	0.86447851839769
H	0.59138295279382	0.96663670641847	1.49716527480833
H	-0.75745381817276	3.47559414256672	1.23919350225098
H	0.56059371646553	3.09532694491916	2.36559777260929
H	0.93033039574744	3.69612898863305	0.73705614486287
C	2.10303390386052	-0.91211360287263	0.22043481700897
O	2.76199420308941	-0.02906610181256	-0.34366069011927
C	2.76839878115413	-1.99163441333522	1.02962884486093
H	2.05684269413508	-2.70653037340889	1.44366624690631
H	3.48165184414682	-2.51522581136977	0.38960016287323
H	3.32765582790615	-1.52458212188245	1.84325664495821
O	-2.16526981189909	-0.28104928975415	0.30144764840420
H	-2.44418956763869	-1.12560212439724	0.67728884643337

Conformer 3 (**CTA-CB_C3**): Gibbs Energy: -969.1305371 Hartree
Relative Gibbs Energy = 1.31 kcal/mol
Boltzmann Population: 7.9%

C	0.27104072116803	-0.23558414392480	-0.34775861649881
C	0.33673709963669	1.04234600553429	0.49713518003056
N	0.98815251257909	-1.31104985239833	0.29102552553550
C	-1.17013197968873	-0.72001926859256	-0.60280120560291
H	0.73998368188817	-0.02369448806186	-1.31210374471520
H	-1.07438073691277	-1.65208341607619	-1.16892324362023
O	0.90697685476571	1.08332102150356	1.59286005633399
S	-2.09661971752275	-0.99053319156524	0.90837520703064
H	0.44480174462642	-1.86870751597573	0.93680574745704
C	-0.33101071347975	3.37622609528456	0.66568887518256
N	-0.22096197299419	2.12336791399343	-0.05919300908248
H	-0.80263203330392	1.96630501308739	-0.86928479592000
H	0.65667123836802	3.74742190342863	0.94423702407107
H	-0.81004976000819	4.10899746514304	0.01814973416641
H	-0.92864909046241	3.26103753507491	1.57436113454719
C	2.32790236245812	-1.34760011976364	0.36188722304049
O	3.04668319795741	-0.55407473365128	-0.26179053318237
C	2.92252837231415	-2.42395798462133	1.22895436741946
H	3.47471453448501	-1.94507760446407	2.04093290885610
H	2.16852021280865	-3.08957697243729	1.64948913363908
H	3.63246003491414	-3.00327853904103	0.63550635461953
O	-1.81992203848157	0.22456541703625	-1.48019343781232
H	-2.62291157821534	0.48265879058728	-1.00943174949532

Conformer 4 (**CTA-CB_C4**): Gibbs Energy: -969.12914971 Hartree
Relative Gibbs Energy = 2.18 kcal/mol
Boltzmann Population: 1.8%

C	0.00023473271362	-0.18737760186450	-0.19542999271153
C	0.14739187615788	1.09488812327640	0.61804079375540
N	0.84237031057498	-1.23309966985579	0.35456558084413
C	-1.47173655139928	-0.64496738534827	-0.15418966349199
H	0.27231330229782	0.00525212566055	-1.23632973742076
H	-1.74027210196270	-0.78678039439518	0.89770348196170
O	0.21823911986227	1.06783866259328	1.85178018983519
S	-2.57080588012261	0.51431137581090	-0.96416146235837
H	0.41588993764902	-2.12575342653982	0.54411504211812
C	0.05318333744973	3.51993895298120	0.57268004592713
N	0.18071948642974	2.23235975922962	-0.08700071587293
H	-0.15107413962321	2.16216152328700	-1.03802253481149
H	0.13346747766141	4.30462114710274	-0.17811381021968
H	-0.90907427311112	3.61381295372927	1.08546897653771
H	0.85229795735286	3.65192036859665	1.30267317088736
C	2.16769274248661	-1.10416515342634	0.49311236074976
O	2.76002652583045	-0.05714567915072	0.19170397236543
C	2.90845567137610	-2.29358940949982	1.04070473239489
H	2.25414379898800	-3.14406337786506	1.23293812132277
H	3.68197550705855	-2.58270778231098	0.32636523063781
H	3.40100280709494	-1.99831178757836	1.96974195771495
O	-1.54881581793021	-1.95854648605370	-0.74632130051418
H	-1.85772287983484	-1.79958750797908	-1.64809630375142

Conformer 5 (**CTA-CB_C5**): Gibbs Energy: -969.12905987 Hartree
Relative Gibbs Energy = 2.24 kcal/mol
Boltzmann Population: 1.6%

C	-0.21272812871772	-0.35062372002312	-0.48736553938897
C	-0.39876332049565	1.12671688240490	-0.15204879127711
N	0.83596790501933	-0.98922456893198	0.27205663942978
C	-1.52793722943301	-1.12616517223244	-0.25002552895193
H	0.04209978404139	-0.39849996619261	-1.54896637360662
H	-1.33585624355775	-2.15871737662768	-0.55183717684596
O	-1.30244609503610	1.77657556563004	-0.70819585563672
S	-2.01732795541553	-1.06569366388761	1.48520778519527
H	0.54485313944999	-1.38487083823612	1.15825983606470
C	0.32359023624035	3.08263710758041	1.09889317359572
N	0.44327047500075	1.68853584077068	0.71035241263605
H	1.12651395586069	1.10712262434520	1.16678259197538
H	1.12984983459984	3.31708552019377	1.79154129349793
H	0.40409581172729	3.73481359796332	0.22728990269227
H	-0.63406689103344	3.27091448744868	1.58983701645430
C	2.12499884273752	-1.02516434485690	-0.10334101273034
O	2.52645786696957	-0.49804374436132	-1.14972556682325
C	3.06700785122149	-1.74592356206551	0.82247817861022
H	3.85100442887819	-1.05143772870930	1.13134336662201
H	2.56447886586788	-2.14411600056300	1.70417795801308
H	3.53809545765808	-2.56290461478097	0.27176234224189
O	-2.51814960894287	-0.65854273785541	-1.17315468267411
H	-2.53510603544033	0.30653574338693	-1.07139383299361

Conformer 6 (**CTA-CB_C6**): Gibbs Energy: -969.12870922 Hartree
Relative Gibbs Energy = 2.46 kcal/mol
Boltzmann Population: 1.1%

C	0.46929444782863	-0.02292411898444	-0.12859906744045
C	0.38908325520754	1.31762540151073	0.59255096962481
N	1.30781784539939	-0.91557632102228	0.63493931210605
C	-0.92346521405833	-0.64821965557714	-0.36339579146880
H	0.93926646873644	0.13742535360701	-1.10241414464002
H	-0.73492494354591	-1.66044296831264	-0.73423401818806
O	0.90889725073129	1.50121084983907	1.70021595398981
S	-1.96162073503260	-0.69052055219982	1.09683676870902
H	1.29151915011845	-0.81341585203258	1.63712902687419
C	-0.53835765636511	3.56523173894305	0.54708604607443
N	-0.24566677763877	2.28647368458247	-0.07493621285979
H	-0.77315270579514	2.00222375309757	-0.88755560745261
H	0.38270320281768	4.06222797257411	0.85536973726280
H	-1.04777654818230	4.19433832547851	-0.18113405428978
H	-1.18067082114553	3.44430731006597	1.42412679108864
C	2.00665593614128	-1.91723252519258	0.08657894375055
O	2.03419525482213	-2.12157786071628	-1.13702615714166
C	2.77441344975915	-2.79521021726953	1.03827606984980
H	2.39154269383440	-3.81477059781235	0.95368382696092
H	3.82386163474960	-2.80426509801028	0.73732373913742
H	2.69514982448987	-2.46539341085256	2.07439786253153
O	-1.55193051002535	0.08186633638720	-1.44442891709747
H	-2.41693155604683	0.32762778229678	-1.09086296118133

Conformer 7 (**CTA-CB_C7**): Gibbs Energy: -969.12866693 Hartree
Relative Gibbs Energy = 2.48 kcal/mol
Boltzmann Population: 1.1%

C	0.30048706505113	0.04531071497681	-0.09869023587261
C	0.36860899147161	1.37293874697202	0.66500133154476
N	1.14363038987315	-0.90001633235200	0.59846991714660
C	-1.16949102183067	-0.44498706773433	-0.24712520415452
H	0.70443193920151	0.19415248339235	-1.10130383766374
H	-1.65817301796888	-0.33434979069111	0.72672764031025
O	0.99216111287943	1.46957386044121	1.73323809417685
S	-2.05733964440053	0.45120195519040	-1.53682104394093
H	1.28390122521908	-0.74872178412838	1.58527175956393
C	-0.38835585103246	3.67634731832838	0.78242900432669

N	-0.27269738139578	2.39692005512912	0.10757061393080	
H	-0.91209380918098	2.15274286534888	-0.65392271601759	
H	0.59763554666171	4.10814571076842	0.96332087256018	
H	-0.95356300753986	4.35279061605895	0.14281159282139	
H	-0.90588420191972	3.57918520585374	1.74082134082472	
C	1.87075038600533	-1.84049264129696	-0.01877702369412	
O	1.85048118362281	-2.01206829172406	-1.24661458098135	
C	2.73423152243832	-2.69898841677291	0.86734741691405	
H	2.41641662534643	-3.73837198178622	0.75878872593630	
H	3.76820714214942	-2.62610018084669	0.52408367748994	
H	2.67804980125636	-2.41301849280733	1.91789316346079	
O	-1.17935959133832	-1.85515988983594	-0.50710691993659	
H	-1.17213245766909	-1.93602533228441	-1.46948545264578	

Figure P3.9: Structures of conformers of *R*-configured cysteine hemi-thioacetal conjugate base derivative **CTA-CB** (wB97X-D4/def2-TZVP/def2/J, CPCM: water).

Conformer 1 (**CTA-CB_C1**)<br>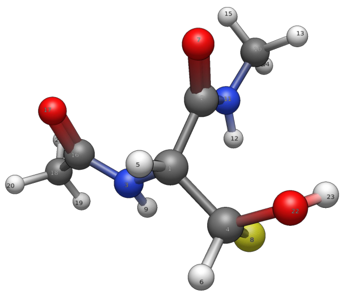	Conformer 2 (**CTA-CB_C2**)<br>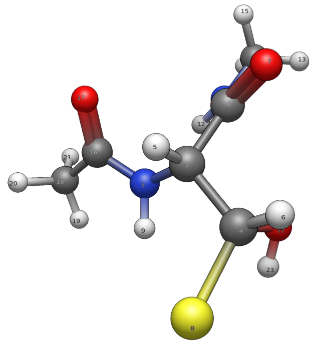
Conformer 3 (**CTA-CB_C3**)<br>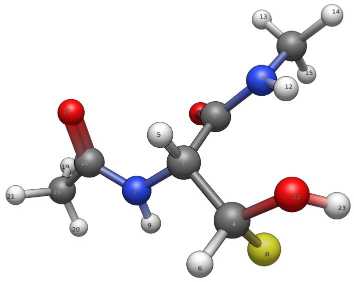	Conformer 4 (**CTA-CB_C4**)<br>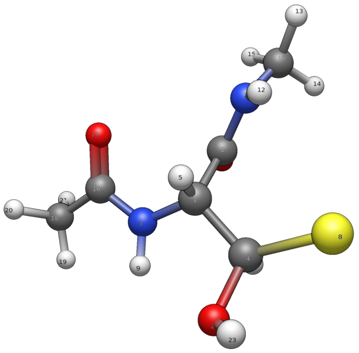

Conformer 5 (**CTA-CB_C5**)<br>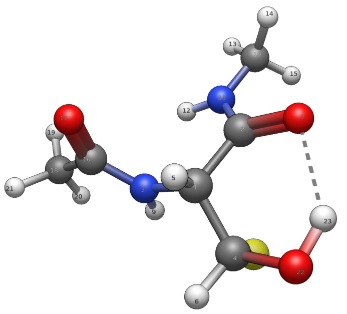	Conformer 6 (**CTA-CB_C6**)<br>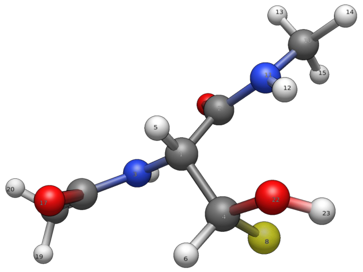
Conformer 7 (**CTA-CB_C7**)<br>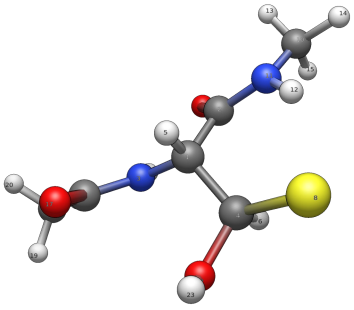	

P4 Calculation of pK_a values

We wanted to calculate the pK_a value of putative intermediate cysteine hemi-thioacetal. As we had an experimental value for a cysteine derivative, we decided to calculate the pK_a value of the former through an isodesmic equilibrium, with the cysteine thiol derivative and cysteine hemi-thioacetal conjugate base on one side, and cysteine thiolate and cysteine hemi-thioacetal derivative on the other side. We also carried out calculations for the *E*- and *Z*-enethiols, for which we had experimental values and thus would allow use to ascertain the accuracy of the computational approach. Initial calculations, using the data provided above (Section 3) gave ΔpK_a values that were unreasonably large. However, the inclusion of two water molecules, which were allowed to hydrogen-bond with the thiolate moieties, gave more reasonable values.

Table P4.1: Cartesian coordinates, Gibbs energies (Hartree) and structures of the most populated conformer of the conjugate bases of cysteine thiol (**Cys-CB**), *E*-enethiol (**E-ETe**), *Z*-enethiol (**Z-ETe**) and *R*-configured hemi-thioacetal (**CTA-CB**), each with two explicit water molecules hydrogen-bonding with the thiolate (wB97X-D4/def2-TZVP/def2/J, CPCM: water).

Cys-CB + 2H_2O: Gibbs Energy: -1046.81867057 Hartree

C	-4.86004452048801	2.93719456082317	0.75853780808092
C	-3.66927405051951	1.98834639325996	0.86355961489539
N	-6.01522703511964	2.37186165331524	0.09843912519640
C	-5.25739336194978	3.42964945576178	2.15473907227566
H	-4.50122517129766	3.78972126965880	0.17592367986580
H	-7.63136928109052	1.82336989449875	-2.61103568562629
O	-2.55235745753266	2.45776981253947	1.13129797043455
S	-5.94839071140118	2.14084409646628	3.24292053881793
H	-6.77656226602572	2.07180593971860	0.68876803797790
C	-2.81180070060092	-0.28460771776955	0.87198926309324
N	-3.87266507149486	0.68631323568994	0.67192805949822
H	-4.82272196985868	0.35239387063998	0.60239615022688
H	-3.20288526340320	-1.27393609251287	0.63984384785717
H	-1.96894400738619	-0.07464076798099	0.21134799780326
H	-2.45609325575246	-0.27564216824815	1.90587533551299
C	-6.04842510865974	2.06316318820484	-1.20694401568746
O	-5.10999868866835	2.32861386166510	-1.97042268451419
C	-7.28604448207995	1.35536296484029	-1.68801631860738
H	-7.01908193161350	0.31924093120395	-1.91252391645173
H	-8.08678739245332	1.36405520394345	-0.94726947747828
H	-5.97955431001855	4.23968586133423	2.02432450914595
H	-4.36733585337694	3.86495781510502	2.61231793498751
O	-8.92730826216633	2.26076839360326	2.16392046086862
H	-8.94890941023539	1.49174504618565	1.58197561435642
H	-8.02203648534449	2.23344054865791	2.56426378622702
O	-6.68032529722406	-0.53116529974977	1.59047730634357
H	-6.45879535243345	0.28622495340258	2.09535668277335
H	-7.35162919020434	-0.24855765185704	0.95763907822653
H	-2.65476051692992	0.64594982442020	-0.79013059554822

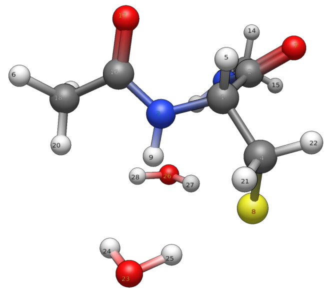

E-ETe + 2H_2O: Gibbs Energy: -1045.60956260 Hartree

C	-5.70641029125963	0.98279339960325	0.22126314259135
C	-4.36131693962934	1.06529662984586	0.84555230088334
N	-6.08651222077196	2.15351680388792	-0.50717835271847
C	-6.60558899954572	-0.02168829090043	0.31670350890412
H	-5.03103233657597	4.59655194885913	-2.05622111916546
H	-3.99086663187422	-1.61186228302899	3.99164986535502
O	-3.76650726356874	2.15371004989917	0.95306559975369
S	-6.57322147448477	-1.52417239789087	1.16889354835713
H	-6.79904468159318	2.75667600351282	-0.12629116280037
C	-2.56639845707485	-0.08932844938748	2.01494917069219
N	-3.80449753787692	-0.08154685307568	1.25967357663064
H	-4.40866965516461	-0.89705545780850	1.28737364029838
H	-2.65719769827367	0.48298601513624	2.94275387578157
H	-2.31916024005628	-1.12228155307998	2.25831756392241
H	-1.74874343313326	0.33337838567277	1.42802172672238
C	-5.42825177485228	2.56514854225923	-1.60482091660769
O	-4.51800250177072	1.89950905444382	-2.11597262888332
C	-5.85236254610152	3.88701118115377	-2.18402060387374
H	-6.02891215644381	3.76514640613298	-3.25394457073934
H	-6.74699021372917	4.28729597444463	-1.70661735423861
O	-6.67414626291353	0.64252109988457	3.69390330372345
H	-6.78873458725039	0.02995419532645	2.94528272508213
H	-6.03774159774045	0.17782699831487	4.25237428241371
O	-4.90174667330325	-1.91472185335562	4.08219513168007
H	-5.29062763185781	-1.78498864712972	3.19932369230732
H	-7.52977873275278	0.16511280027960	-0.22862672027213

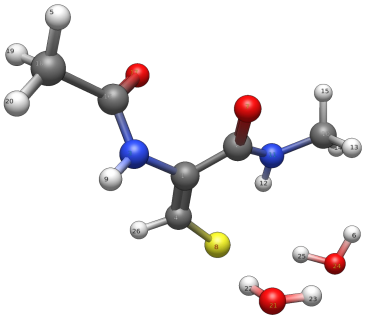

Z-ETe + 2H_2O: Gibbs Energy: -1045.61380075 Hartree

C	-5.74013428237428	0.96129187609849	-0.06917288887313
C	-4.51348969870628	0.40091109049918	0.53647474348038
N	-5.77153005198218	2.34883844059980	-0.38469871048612
C	-6.84098923953254	0.19902861359662	-0.22855625627035
H	-4.47109063139845	4.88200757248113	-1.63562239394017
H	-6.75301696964481	-0.83316790928313	0.10206586156280
O	-4.34415727125311	-0.82570410091467	0.67694423319708
S	-8.35563040273402	0.71444912863135	-0.85824607743625
H	-6.55051971944626	2.88098710007343	-0.02401677705087
C	-2.32611379396280	0.84360532640416	1.52553915091677
N	-3.58609051839737	1.27810389547060	0.95342122385975
H	-3.74274000967562	2.26053031772602	0.80397706458940
H	-1.73528721300400	1.72650017214327	1.76462735859084
H	-2.48622791216302	0.26945181886749	2.44087870956600
H	-1.76574191323235	0.22418501830036	0.82088745475583
C	-5.11614535861525	2.86964234756606	-1.44547192819033
O	-4.30924476711828	2.20977509063922	-2.10896253066502
C	-5.39647606139423	4.31415091286383	-1.75588373753695
H	-5.70630760149961	4.39216215639860	-2.79975423298412
H	-6.16697397877639	4.73947543390320	-1.11255139898989
O	-8.98642453200535	-2.36678786012419	-1.56690303992342
H	-9.36026939573321	-2.70224653925997	-0.74392751356128
H	-8.84903039245665	-1.41108937555745	-1.39031951444054
O	-9.52845815089604	-1.14798999042469	1.53824546173778
H	-9.25208766183973	-0.55845743730461	0.80656269454208
H	-8.93391877255703	-1.90394426859428	1.46686958474972

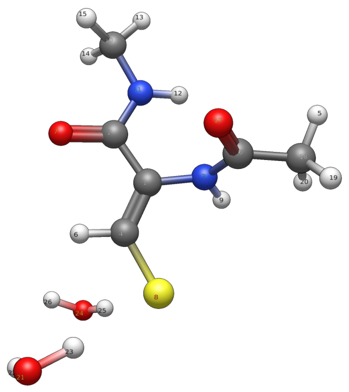

CTA-CB + 2H_2O: Gibbs Energy: -1122.08618343 Hartree

C	0.07048287105145	-0.41164846885463	-0.49660549997694
C	0.31753883706800	1.09636270811287	-0.59572608112803
N	0.93103309584381	-1.06473936346743	0.46625092462046
C	-1.40149288676224	-0.74407919149175	-0.16310828775045
H	0.28203294901373	-0.80424582924127	-1.49258127488500
H	-1.54038533978362	-1.80161502920361	-0.39873246160660
O	0.54632350923293	1.62724734471165	-1.69057344771563
S	-1.84274844182811	-0.45593878373395	1.56720933436619
H	0.51205694913425	-1.29768781078314	1.35758440366124
C	0.39121712841179	3.23077496273100	0.57210841952633
N	0.27258654623381	1.78363509364851	0.54768014059994
H	-0.13342107199323	1.30578961472970	1.34453758916535
H	-0.42247348435403	3.70896637433870	0.01974528188910
H	0.35964130836783	3.56258914580444	1.60876203785268
H	1.34006628318965	3.54174136504386	0.13233368264868
C	2.26497328720943	-1.11239756363414	0.32194208851347
O	2.82908764082007	-0.67453760303187	-0.68995011117832
C	3.04212177444044	-1.72999621737970	1.45166900171655
H	2.40057718469608	-2.08078393622518	2.26027843205825
H	3.62450592308498	-2.56630601389711	1.05948513959902
H	3.74073067778508	-0.98522800224911	1.83904444743706
O	-2.23920837390325	-0.03127611932811	-1.07740685675899
H	-2.52989143083925	0.77181822672228	-0.62705704206931
O	-5.02225076492145	-0.51975093972476	0.97419855959468
H	-5.39615615841004	0.29922884609870	1.31831043632226
H	-4.06318605784875	-0.45388496172724	1.16917358897564
O	-3.21436235465578	-3.34373038423197	1.65136054501846
H	-4.09615451874822	-3.14836274921268	1.31407964388792
H	-2.77873767973529	-2.46388240422405	1.69989765221592

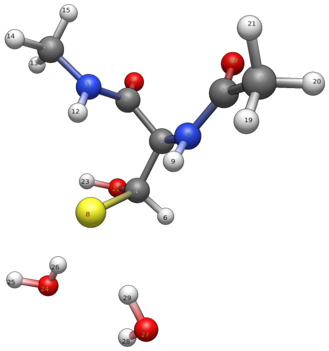

Table P4.2: Gibbs Energies (Hartree) of acids and conjugate bases used in the calculation of pK_a values

	**Compound**	**Gibbs Energy (Hartree)**
A	Cysteine (**Cys**): Boltzmann Averaged (12 conformers)	-894.331529539015
B	Cysteine thiolate (**Cys-CB**) + 2 water molecules (Conformer 1)	-1046.81867057
C	*E*-Enethiol (**E-ET**): Boltzmann Averaged (5 conformers)	-893.111878385098
D	*E*-Enethiolate (**E-ETe**) + 2 waters (one major conformer)	-1045.6095626
E	*Z*-Enethiol (**Z-ET**): Boltzmann Averaged (6 conformers)	-893.115452186077
F	*Z*-Enethiolate (**Z-ETe**) + 2 waters (one major conformer)	-1045.61380075
G	*R*-Thioacetal (**CTA**): Boltzmann Averaged (11 conformers)	-969.595171017151
H	*R*-Thioacetal Conjugate Base (**CTA-CB**) + 2 waters (Conformer 1)	-1122.08618343

Table P4.3: Experimental (*Exp*) and Calculated (*Cal*) pK_a values. T = 298.15 K;
$R = 1.987*10^{-3}$ kcal/mol.K

Cysteine (ACA tripeptide)	8.6 (*Exp*)
E-enethiol (AC*A tripeptide)	4.8 (*Exp*)
Z-enethiol (AC*A tripeptide)	4.3 (*Exp*)
E-Enethiol (**E-ET**)<br><br>Isodesmic Rxn (see Table 1.4.2) : A + D $\rightarrow$ B + C<br>ΔG = 0.0105 Hartree (6.62 kcal/mol)<br>ΔpK_a (8.6 – pK_a (**E-ET**)) = ΔG/(2.303RT) = 4.85<br><br>pK_a (**E-ET**) *Cal = 8.6 – 4.9 = 3.7*	3.7 (*Cal*)
Z-Enethiol (Z-**ET**)<br><br>Isodesmic Rxn (see Table 1.4.2) : A + F $\rightarrow$ B + E<br>ΔG = 0.0112 Hartree (7.03 kcal/mol)<br>ΔpK_a (8.6 – pK_a (**Z-ET**)) = ΔG/(2.303RT) = 5.15<br><br>pK_a (**Z-ET**) *Cal = 8.6 – 5.2 = 3.4*	3.4 (*Cal*)
R-Thioacetal (**CTA**)<br><br>Isodesmic Rxn (see Table 1.4.2) : A + H $\rightarrow$ B + G<br>ΔG = 0.0039 Hartree (2.43 kcal/mol)<br>ΔpK_a (8.6 – pK_a (**CTA**)) = ΔG/(2.303RT) = 1.78<br><br>pK_a (**CTA**) *Cal = 8.6 – 1.8 = 6.8*	6.8 (*Cal*)

P5 Natural Bond Orbital Analysis: n→π interactions*

Visual inspection of the cysteine thioaldehyde conformers depicted in Figure 1.3.1 shows possible n→π* interactions involving either the N-terminal acetyl carbonyl oxygen atom or the C-terminal amide carbonyl oxygen atom acting as a donor and the thiocarbonyl group acting as a π-acceptor (Conformers 4, 5, 6 and 8). These structures were subjected to additional Natural Bond Orbital (NBO) analysis to ascertain the stabilisation energy associated with these interactions.

Figure P5.1: Distances, angles and interaction energies of n→π* interactions in Conformers 4, 5, 6 and 8 of cysteine thioaldehyde **CT**. The donor–acceptor interaction energies were obtained from the 2nd order perturbation theory analysis table in the NBO output file.

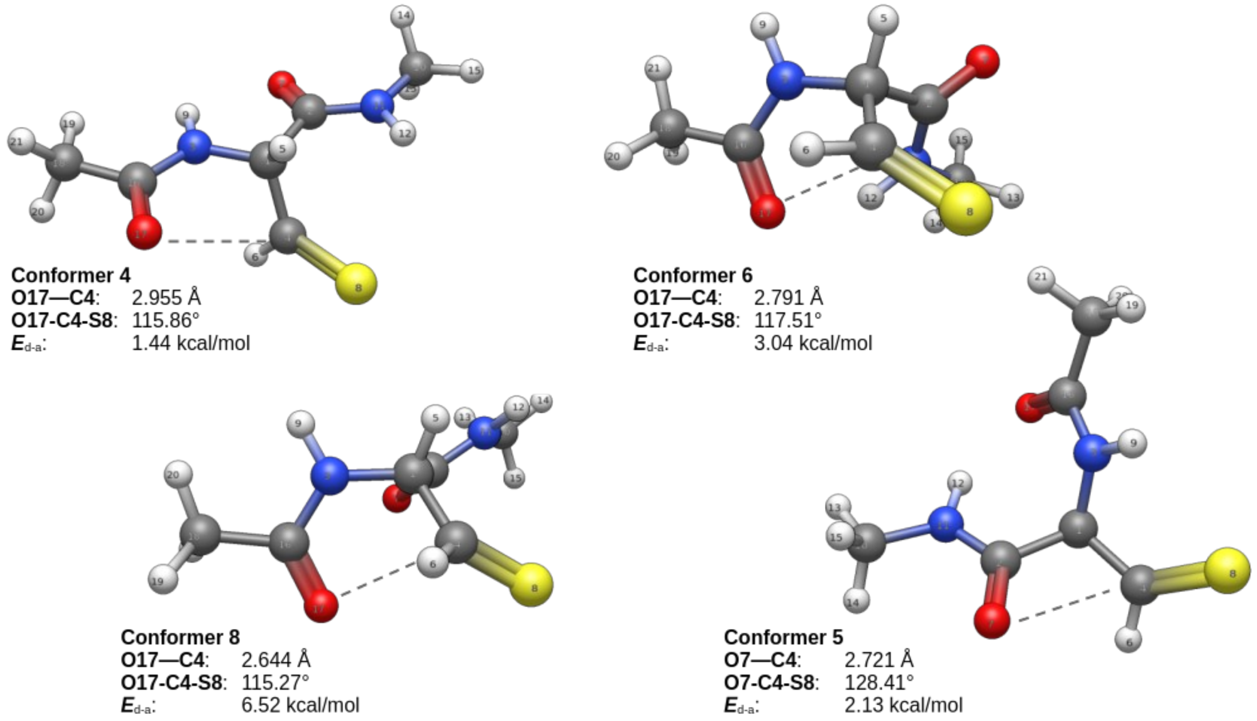

P6 Cyclisation to Oxazolidine Intermediates

The n→π* interactions involving the backbone N-terminal amide carbonyl and the thiocarbonyl group suggest a pathway to the experimentally observed oxazole products via an oxazolidine intermediate. Transition states for cyclisations leading to both diastereomers of the zwitterionic oxazolidine were found, indicating a very low barrier. Subsequent proton transfer leads to relatively stable oxazolidine species.

Figure P6.1: Structures and relative Gibbs energies of reactants, products and transition states for the conversion of thioaldehyde **CT** into *trans*-configured zwitterionic oxazolidine ***trans*-ZOD**, via transition state **CT---trans-ZOD** and then, through proton transfer, into *trans*-configured oxazolidine ***trans*-OD**.

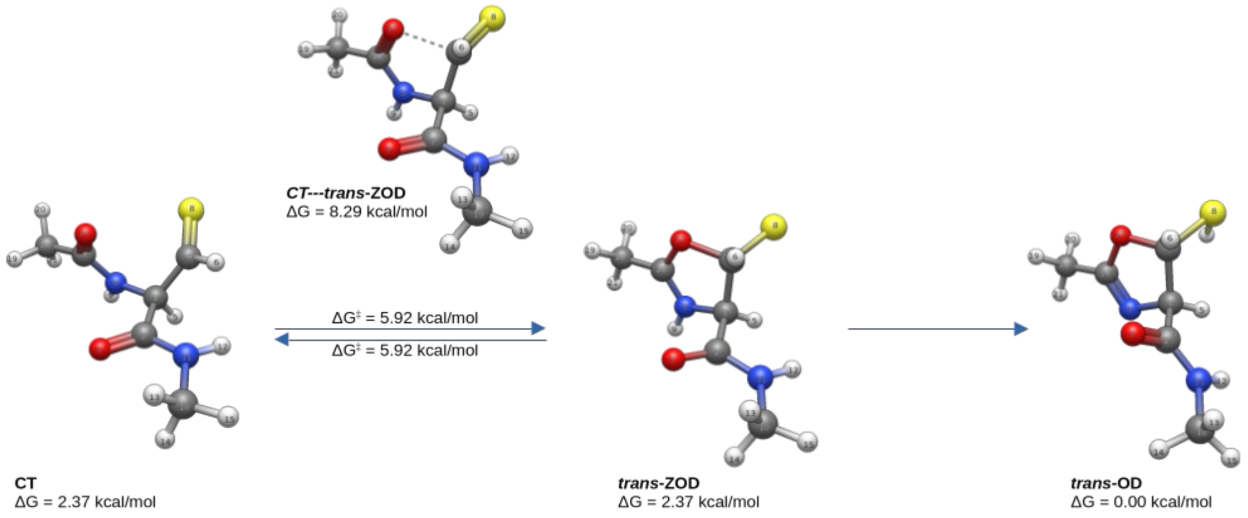

Table P6.1: Cartesian coordinates, Gibbs energies (Hartree), relative Gibbs energies (kcal/mol) and imaginary frequencies, where relevant, of reactant cysteine thioaldehyde **CT**, product zwitterionic *trans*-configured oxazolidine **trans-ZOD** and connecting transition state **CT---trans-ZOD**. Computational data for non-zwitterionic *trans*-configured oxazolidine **trans-OD** is also provided. All species calculated at the following level: (wB97X-D4/def2-TZVP/def2/J, CPCM: water).

Reactant **CT**:<br>Gibbs Energy: -893.09925902 Hartree<br>Relative Gibbs Energy = 2.37 kcal/mol<br>ΔG‡: 5.92 kcal/mol				Transition state **CT---trans-ZOD**:<br>Imaginary frequency: −262.73 cm^{-1}<br>Gibbs Energy: -893.08982047 Hartree<br>Relative Gibbs Energy = 8.29 kcal/mol			
C	0.55039239225182	-0.00353352486580	-0.74068507784383	C	0.52081835904012	0.00937800668914	-0.67799027541829
C	0.49031764168742	-0.77811997574162	0.59856483797677	C	0.50784404017867	-0.80274982488297	0.62643906540158
N	-0.75439053476959	0.20666010678110	-1.29554065165596	N	-0.78625561680839	0.02428492093867	-1.28931227381328
C	1.39014812496827	1.25630694023146	-0.67705850456657	C	0.87413753236304	1.47449491310797	-0.38399275563940
H	1.09317938459663	-0.66568774996478	-1.42783546011639	H	1.25218034273990	-0.39839420451682	-1.37545324521467
H	2.15478556605780	1.26216844503445	0.09804172967055	H	1.04174829138038	1.69829328877741	0.66583726611460
O	-0.57022842008640	-1.16485620168764	1.06910460625936	O	-0.52773407551565	-1.00043585926659	1.25254847921417
S	1.27630408184083	2.50110336624532	-1.68192327601134	S	1.63336157349638	2.40869273246308	-1.53778827746533
H	-1.04165259661358	-0.35523856666772	-2.07839213184913	H	-1.15151547182974	-0.76638001413749	-1.79662369918482
C	1.77987711579785	-1.82176440444446	2.38473174399833	C	1.90164649728348	-1.92320790796032	2.28734952890266
N	1.67819684625379	-1.02959311683149	1.16947318380848	N	1.71359597603939	-1.21753072044168	1.03024323158790
H	2.52862925416103	-0.80890345599937	0.67835562099084	H	2.51734228610308	-1.03894505684620	0.44974542900561
H	1.18822939025752	-1.36927318920052	3.18233260954526	H	1.58263543582624	-1.30395324389129	3.12873764692811
H	1.42349824488569	-2.84216488074664	2.22104717848094	H	1.32613882476784	-2.85144152103804	2.29830815542885
H	2.82509598182956	-1.85077589472571	2.68848053923241	H	2.95945600378359	-2.15681417232340	2.39501324301199
C	-1.63807365855267	1.05487559461509	-0.72087681336965	C	-1.52997405163945	1.06711247199085	-0.95570135969183
O	-1.34285697052272	1.72929145751728	0.26093736825266	O	-0.97174603375224	1.96169266108105	-0.27435718947059
C	-3.00197450482074	1.12632015740031	-1.36171792591301	C	-2.95220305868643	1.17365696749393	-1.39153008896098
H	-3.73396524979114	0.72639168109846	-0.65626742115111	H	-3.58704673455897	1.15003147283546	-0.50319717963120
H	-3.24685961296230	2.17358234579517	-1.54555331625791	H	-3.09647269801968	2.13375338003397	-1.88900931484377
H	-3.06215180928703	0.56660273771479	-2.2952219567187	H	-3.22795742219159	0.35846170989331	-2.05926638626132

Product **trans-ZOD**:
Gibbs Energy: -893.09925902 Hartree
Relative Gibbs Energy = 2.37 kcal/mol
ΔG‡: 5.92 kcal/mol

C	0.49739548988735	0.02870714990604	-0.61504238368392
C	0.52891031575015	-0.79430720883368	0.67543683560210
N	-0.81269873444189	-0.05597357475306	-1.23364323285776
C	0.59763452942410	1.54828852978637	-0.30931123494040
H	1.27549686078951	-0.27263580627882	-1.31274153965485
H	0.82036697431129	1.73057749609252	0.74059392106406
O	-0.48120242455030	-0.93921983759035	1.35751623691600
S	1.58929109426503	2.48525999783154	-1.42026251051220
H	-1.18207343132157	-0.88309317055809	-1.67965358871846
C	1.95856317275382	-1.97453923250313	2.25972884807536
N	1.73317395751508	-1.26407893799359	1.01115135584627
H	2.51020139433351	-1.12230501118346	0.38596795984458
H	1.68891659587842	-1.34734899008837	3.11216547278954
H	1.36336280997803	-2.88963156765657	2.29599437477998
H	3.01455403973162	-2.23125807343882	2.32245752805733
C	-1.51378916791184	1.02058284939033	-1.02531212572495
O	-0.86977465544547	1.95773351656249	-0.41679006962757
C	-2.91922125612808	1.22273987925563	-1.43541561627600
H	-3.51101111795978	1.45032594245811	-0.54652391466094
H	-2.96502868884322	2.08203831824783	-2.10731297877111
H	-3.30956842372977	0.33474585990426	-1.92900022231793

Product **trans-OD**:
Gibbs Energy: -893.10303272 Hartree
Relative Gibbs Energy = 0.00 kcal/mol

C	0.54735366261060	0.27414601294767	-0.42202949791621
C	0.70178196726299	-0.44515733601603	0.91893956924598
N	-0.71859667123708	-0.15088751138569	-1.02710064442976
C	0.38857267480283	1.79695827770033	-0.22725140120969
H	1.38132079252836	0.03601774061415	-1.08308402747305
H	0.57541707430965	2.11632709188305	0.79661681753280
O	-0.00238947048824	-0.14032458550550	1.87985199976052
S	1.44330396778821	2.81162353919483	-1.27246950114577
H	1.24204196665712	2.13084136395065	-2.41014615496696
C	1.82802415697056	-2.22835243681200	2.14562262207492
N	1.61392536649997	-1.42146271003331	0.95592990624162
H	2.15865051709469	-1.61589184119581	0.13212099419571
H	2.12975199945376	-1.60100294897900	2.98727066212984
H	0.91565932265369	-2.76334885099264	2.41909163234411
H	2.61678602561969	-2.94878653764431	1.93535372042739
C	-1.49352154010159	0.85571469788280	-1.00491760126637
O	-1.00438702770037	2.02177093150137	-0.51241000610504
C	-2.90351304725180	0.90767346815093	-1.47167351976165
H	-3.55079371505436	1.20911207261395	-0.64490712586028
H	-3.00102215055529	1.65195423816781	-2.26515134757299
H	-3.20902725226340	-0.06942142894324	-1.84052820404511

Figure P6.2: Structures and relative Gibbs energies of reactants, products and transition states for the conversion of thioaldehyde **CT** into *cis*-configured zwitterionic oxazolidine ***cis*-ZOD**, via transition state **CT---cis-ZOD** and then, through proton transfer, into *cis*-configured oxazolidine ***cis*-OD.**

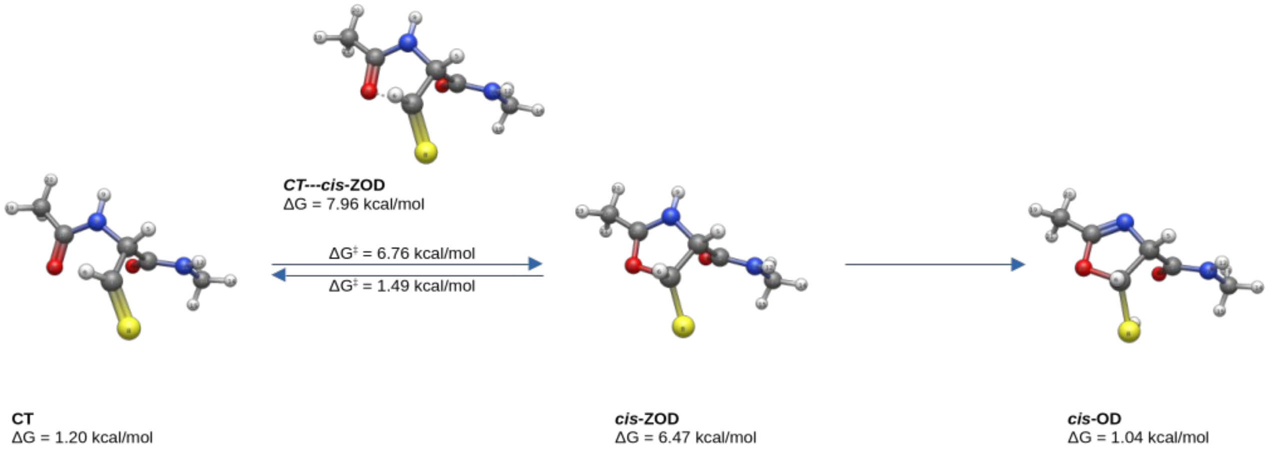

Table P6.2: Cartesian coordinates, Gibbs energies (Hartree), relative Gibbs energies (kcal/mol; relative to ***trans*-OD**) and imaginary frequencies, where relevant, of reactant cysteine thioaldehyde **CT**, product zwitterionic *cis*-configured oxazolidine **cis-ZOD** and connecting transition state **CT---cis-ZOD**. Computational data for non-zwitterionic *cis*-configured oxazolidine **cis-OD** is also provided. All species calculated at the following level: (wB97X-D4/def2-TZVP/def2/J, CPCM: water).

Reactant **CT**:
Gibbs Energy: -893.10112191 Hartree
Relative Gibbs Energy = 1.20 kcal/mol
$\Delta G^{\ddagger}$: 6.76 kcal/mol

C	-0.43645595192275	0.38660095962616	0.61593928507963
C	0.79692424617391	-0.52873445113021	0.53380973659764
N	-0.62352698257251	1.15927625214772	-0.58985186021082
C	-1.69503836349447	-0.35535411374365	0.99573828886074
H	-0.28490280933849	1.10717344675486	1.42907009273769
H	-2.59894255311142	0.22711523030188	0.82455548896960
O	1.13181139758465	-1.06218051418576	-0.51785735291791
S	-1.76994155163542	-1.79443046043515	1.70902521327307
H	-0.10676793243089	2.01922358570452	-0.67585657222190
C	2.58433490567837	-1.59593931199058	1.80334004719726
N	1.46230081823431	-0.68079093895120	1.68433356688621
H	1.10639017068824	-0.24327728199911	2.51834103342784
H	3.36888262326626	-1.33146080857944	1.09187867169755
H	2.98098472890120	-1.52498887866102	2.81481010897556
H	2.27091519543487	-2.62552906681547	1.61102156409748
C	-1.28090927903843	0.67907492200865	-1.67012944257835
O	-1.87987386324388	-0.39269274907065	-1.64097180573975
C	-1.24926526047877	1.54500799082929	-2.90408441951168
H	-2.26868400944775	1.66951656959286	-3.27190039258252
H	-0.79992897495506	2.52146306263880	-2.72408995563444
H	-0.67368568817149	1.02186284708052	-3.67131469808870

Transition state **CT---cis-ZOD**:
Imaginary frequency: −201.51 cm^{-1}
Gibbs Energy: -893.09035245 Hartree
Relative Gibbs Energy = 7.96 kcal/mol

C	-0.40289789569090	0.46565155030544	0.62439279056940
C	0.84998160231980	-0.41738609775311	0.54510850490021
N	-0.50537379433450	1.29841256025341	-0.55595670682845
C	-1.69149890819086	-0.35417011922279	0.65651293730822
H	-0.38309291053123	1.09049419300426	1.52002285326190
H	-2.55742026235038	0.27925625507686	0.83832352428493
O	1.25529811242931	-0.84211017901864	-0.53240753950101
S	-1.74547997956813	-1.93301895937581	1.21226475132745
H	0.12550813506411	2.06559434078699	-0.73060712477720
C	2.56160301490219	-1.59178165962587	1.81865209197537
N	1.44440901364444	-0.66895117947567	1.71466608864599
H	1.02487922057849	-0.32096262267909	2.56113827149039
H	3.40443805584956	-1.24184642495306	1.21937394360116
H	2.86523959168793	-1.64904079549246	2.86262778107212
H	2.27550407666865	-2.58818166843354	1.47159675091276
C	-1.26412106978439	0.77764417820202	-1.50850092889125
O	-1.94273198670863	-0.23352144731289	-1.19560437458810
C	-1.31467411627626	1.37079585533801	-2.87429082237081
H	-2.35523030988484	1.55907848212859	-3.14179367929982
H	-0.73754768647245	2.29285095923126	-2.92823621842136
H	-0.90679190335192	0.64119277901612	-3.57728289467188

Product **cis-ZOD**:
Gibbs Energy: -893.09271635 Hartree
Relative Gibbs Energy = 6.47 kcal/mol
$\Delta G^{\ddagger}$: 1.49 kcal/mol

C	-0.37679701387447	0.47512199117319	0.60839918665551
C	0.88882418630295	-0.38772717492542	0.56038698232926
N	-0.42975763113543	1.31554391844819	-0.58023970962174
C	-1.68091477852058	-0.34435444237403	0.45415415351900
H	-0.41760839622757	1.07880349263296	1.51411406099542
H	-2.50207839872799	0.23236755872096	0.88175871007575
O	1.34343057969804	-0.77276718029898	-0.51271300427780
S	-1.69197229117626	-2.02550238491628	0.94758809637799
H	0.23229698798002	2.04904693602064	-0.79007892519462
C	2.55549135887414	-1.58186633717402	1.87210699315403
N	1.43677115014404	-0.66360761156113	1.74631444570523
H	0.96938567535245	-0.35525845554381	2.58315801641793
H	3.39625122457584	-1.24047463005571	1.26581707741615
H	2.85873084713806	-1.61490554023693	2.91725399323524
H	2.27378471924291	-2.58697631932049	1.54621165192384
C	-1.24120653671937	0.82043762146219	-1.47237483317218
O	-1.94341761013025	-0.17563799507144	-1.05329809404203
C	-1.39113897031005	1.31493444057046	-2.85742407153355
H	-2.42912145816970	1.61419537105539	-3.01390561704524
H	-0.72199013332223	2.15331666246837	-3.04195900186362
H	-1.16358437679246	0.49437378744425	-3.54107670839334

Product **cis-OD**:
Gibbs Energy: -893.10137155 Hartree
Relative Gibbs Energy = 1.04 kcal/mol

C	-0.31128411277124	0.46020777877938	0.48431874740800
C	0.88194676723250	-0.49105218436815	0.56297963779107
N	-0.21091943830401	1.26842179491481	-0.72970376780565
C	-1.67223850818310	-0.27572556668983	0.33893273877642
H	-0.33268199266560	1.10022008174878	1.36749204845096
H	-2.41729530054883	0.17774083798682	0.99447063752428
O	1.24556534767315	-1.13594414164238	-0.41947463184478
S	-1.76011590841234	-2.02999103692563	0.69625862619449
H	-0.72298986681088	-2.41100670723876	-0.06235216746178
C	2.56445130242503	-1.50856404964776	2.00647915020201
N	1.46388432547504	-0.59230965952378	1.76160095447397
H	1.11996558176905	-0.02961024387176	2.52213173200839
H	3.41943385969229	-1.26388935506036	1.37233780230923
H	2.85777788059651	-1.42316694235804	3.05146455305481
H	2.26109904110936	-2.53776530996488	1.80076048844154
C	-1.17484501425995	0.93243570773661	-1.48481819936464
O	-2.05492400899783	0.00861531846477	-1.02245922568235
C	-1.45788072369102	1.44552942296765	-2.85078015532641
H	-2.44542379571070	1.91170366144796	-2.87239519087874
H	-0.69967763996149	2.17099864700748	-3.13889591053008
H	-1.46190067745594	0.61391650733707	-3.55917674184074

P7 Hydration of Cysteine Thioaldehyde

The addition of a water molecule to the thiocarbonyl group of cysteine thioaldehyde (**CT**) was investigated. A relatively low barrier (~10 kcal/mol) was calculated for a system involving participation of the C-terminal backbone carbonyl oxygen atom, leading to the zwitterionic hemi-thioacetal (**ZTA**). The barrier was lowered to ~5 kcal/mol in the presence of guanidinium ion, which appeared to stabilize the thiolate through hydrogen-bonding. The non-zwitterionic hemi-thioacetal (**TA**), obtained through proton transfer, was also calculated. Similar transformations were calculated involving participation of the N-terminal backbone carbonyl oxygen atom.

Figure P7.1: Structures and relative Gibbs energies of reactants, products and transition states for the conversion of thioaldehyde **CT** into zwitterionic hemi-thioacetal **ZTA**, via transition state **CT---ZTA** and then, through proton transfer, into non-zwitterionic hemi-thioacetal **TA**.

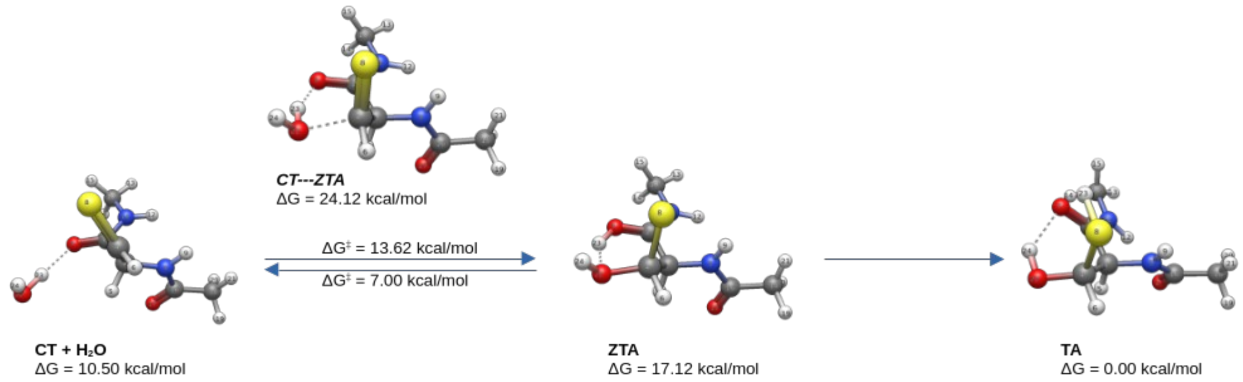

Table P7.1: Cartesian coordinates, Gibbs energies (Hartree), relative Gibbs energies (kcal/mol) and imaginary frequencies, where relevant, of reactant cysteine thioaldehyde and water **CT+H$_2$O**, product zwitterionic hemi-thioacetal **ZTA** and connecting transition state **CT---ZTA**. Computational data for non-zwitterionic hemi-thioacetal **TA** is also provided. All species calculated at the following level: (wB97X-D4/def2-TZVP/def2/J, CPCM: water).

Reactant **CT + H$_2$O**:
Gibbs Energy: -969.57497414 Hartree
Relative Gibbs Energy = 10.50 kcal/mol
ΔG‡: 13.62 kcal/mol

C	-0.71877306619173	-0.52735098341803	-0.11049529641914
C	-0.48915361578522	0.98603724752150	-0.08295219974785
N	0.43039032474995	-1.31621863352713	0.27326753790952
C	-1.90506015347933	-0.95180702220866	0.71852009230069
H	-0.96640719167842	-0.76805046715165	-1.15080298881562
H	-2.07453004619944	-2.02918365900130	0.69086310822091
O	-1.16014733214690	1.72204152635436	-0.81181590515215
S	-2.85840251501869	0.00505328443599	1.57827955022793
H	0.46570776482873	-1.74135846376983	1.18661734099609
C	0.72851641368257	2.85851945838838	0.88313330822352
N	0.43546659977765	1.44031919499137	0.75669910213163
H	0.95864489516824	0.78015303953773	1.30842051176109
H	1.50999007766003	2.98220984420060	1.63065182194734
H	1.07337269225950	3.26594052416151	-0.06969030460616

Transition state **CT---ZTA**:
Imaginary frequency: −271.22 cm^{-1}
Gibbs Energy: -969.55325319 Hartree
Relative Gibbs Energy = 24.12 kcal/mol

C	-0.59249954559128	-0.29488159925372	-0.50402331016574
C	-0.52088462463035	1.19618252196657	-0.16529773644494
N	0.43428024928364	-1.09248491961193	0.11746563782445
C	-1.94368047277541	-0.89085096832152	-0.11099134293154
H	-0.45735058706860	-0.37339591783680	-1.58485357448311
H	-2.13845801740300	-1.82922923576556	-0.62289228745629
O	-1.39919195949426	1.96316918524178	-0.60128247320353
S	-2.50834811511761	-0.69529171704573	1.46190712511216
H	0.29388600689198	-1.37138635125421	1.07706719899308
C	0.68487996092116	3.02244988291571	0.89412345868958
N	0.50494994168804	1.61967769449086	0.55278813669266
H	1.17757940656501	0.94197149654647	0.87428643260612
H	1.59845078711474	3.11734282754737	1.47775575322003
H	0.76840118246258	3.62760185399558	-0.01062480477250

H	-0.16186367145795	3.40721235694756	1.19704859301473	H	-0.16068044848429	3.38339185659612	1.48287022811801
C	1.47694859180143	-1.46333440440565	-0.57937844890473	C	1.49443376319473	-1.58877153545592	-0.57606965129685
O	1.47493555614057	-0.92960760088320	-1.68340450924660	O	1.68128465063405	-1.32461881720642	-1.75792486055093
C	2.63069514772947	-2.29670069301763	-0.08461416400238	C	2.42876530514381	-2.47862530108974	0.20457348216902
H	2.85453425486683	-3.05885213454143	-0.83272327871408	H	2.41274591799384	-3.47261226029628	-0.24752774899069
H	3.50523841929245	-1.64944932616226	0.01146740111495	H	3.44156666418479	-2.08260990772106	0.11333802213048
H	2.42763550146884	-2.77267103968580	0.87462362345280	H	2.16018238422718	-2.55647033651647	1.25791612934854
O	-3.28739179162410	0.68534550552278	-2.31561684702808	O	-2.96821020133402	0.08978199399679	-1.30789587027944
H	-2.55732805167997	1.05270972040405	-1.78100690020390	H	-2.54949862665249	0.99138072268118	-1.13434385686396
H	-3.88832655531327	0.29321022200395	-1.67360086429469	H	-3.84260362175426	0.09827883139693	-0.89036408746460

Product **ZTA**:
Gibbs Energy: -969.56442605 Hartree
Relative Gibbs Energy = 17.12 kcal/mol
$\Delta G^{\ddagger}$: 7.00 kcal/mol

C	-0.57913460266079	-0.28715707227127	-0.57365604170373
C	-0.50996873939172	1.17166117493029	-0.17678877331321
N	0.42060657849395	-1.07603498267682	0.08559582756769
C	-1.98466430997076	-0.84425089416623	-0.28358561273301
H	-0.38745089540502	-0.31639311971804	-1.65143748959088
H	-2.00154529075389	-1.87212290363354	-0.65392951882677
O	-1.45325446683218	1.97315465306876	-0.55743606341528
S	-2.40469937454116	-0.76186107822125	1.45420021403783
H	0.17826015323651	-1.41524945727952	1.00709201318080
C	0.61478159067283	3.05829235906419	0.86199564214249
N	0.48912153106642	1.65073673264122	0.48708809739283
H	1.20543883413175	0.99461227552131	0.76917288332757
H	1.53168095151289	3.16979500628996	1.43490597677880
H	0.66007529141414	3.67808616807847	-0.03384868318338
H	-0.24056457554617	3.35571399019615	1.46906719380451
C	1.50565191950443	-1.57738046613850	-0.56553236859318
O	1.77106949852285	-1.26944956941802	-1.72143187271582
C	2.35958126743346	-2.53233500585036	0.22918608388092
H	2.25191866266837	-3.52865693276994	-0.20617468339427
H	3.40366956705259	-2.23189837230648	0.13280368469441
H	2.08167525632256	-2.57025296662910	1.28243516366600
O	-2.87912973959434	-0.05917055018557	-1.11901892185303
H	-2.19584452469389	1.39762035817042	-0.92062841607829
H	-3.74196683045427	-0.08162684393456	-0.68356461616994

Product **TA**:
Gibbs Energy: -969.59170527 Hartree
Relative Gibbs Energy = 0.00 kcal/mol

C	-0.18525949388595	-0.16272586281982	-0.59468274914863
C	-0.13236360469054	1.27669550162245	-0.05232747894237
N	0.68391772454760	-1.07355290320417	0.12074700187186
C	-1.61775304845272	-0.71617933226041	-0.63112152090214
H	0.16222335522286	-0.11654214796206	-1.63108693375168
H	-1.58560838908428	-1.68987112026267	-1.12350052211798
O	-1.16009034496142	1.90111466053285	0.23498933517510
S	-2.28366074121968	-1.10114336245860	1.02699902200720
H	0.28007708556339	-1.58722251274503	0.88978867663095
C	1.30616889325962	3.15874655740204	0.51618301708887
N	1.08861862460070	1.79846148522094	0.05286021211099
H	1.86956595640929	1.25599576820621	-0.29170737524835
H	2.37868714194775	3.34323136380457	0.54598959657809
H	0.83491843081809	3.88051561561796	-0.15552550590141
H	0.89201613842587	3.29266275681422	1.51746554361326
C	1.98555956704746	-1.24802501663981	-0.18394017953092
O	2.54825187259342	-0.57122732241050	-1.04797689861852
C	2.71822184451346	-2.31019352707112	0.59442439857626
H	3.09089293957915	-3.05578572416508	-0.11098978576104
H	3.57831327623702	-1.85107225182180	1.08527172369018
H	2.08931133685570	-2.79855359348054	1.33851344992721
O	-2.45427071534905	0.10981415920702	-1.39227469756624
H	-2.14996219185688	0.12935316231328	1.53853752479936
H	-2.41616835732087	0.98530175186005	-0.97279125068004

Figure P7.2: Structures and relative Gibbs energies of reactants, products and transition states for the conversion of cysteine thioaldehyde and water in the presence of guanidinium ion **CT + H_2O + Gdn** into zwitterionic hemi-thioacetal and guanidinium ion **ZTA + Gdn** , via transition state **CT---ZTA/Gdn** and then, through proton transfer, into non-zwitterionic hemi-thioacetal **TA + Gdn**.

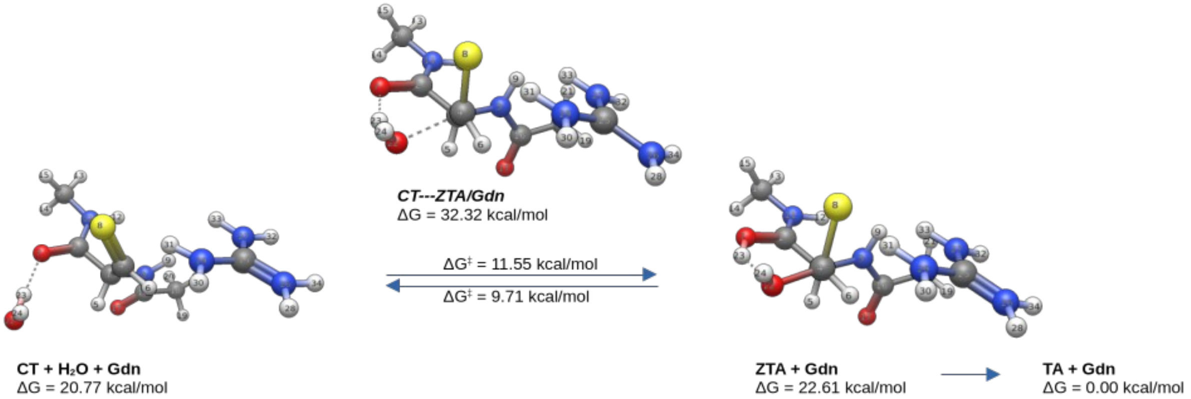

Table P7.2: Cartesian coordinates, Gibbs energies (Hartree), relative Gibbs energies (kcal/mol) and imaginary frequencies, where relevant, of reactant cysteine thioaldehyde and water in the presence of guanidinium **CT+H_2O+Gdn**, product zwitterionic hemi-thioacetal and guanidinium **ZTA+Gdn** and connecting transition state **CT---ZTA/Gdn**. All species calculated at the following level: (wB97X-D4/def2-TZVP/def2/J, CPCM: water).

Reactant **CT + H_2O + Gdn**:<br>Gibbs Energy: -1175.52915241 Hartree<br>Relative Gibbs Energy = 20.77 kcal/mol<br>$\Delta G^{\ddagger}$: 11.55 kcal/mol				Transition state **CT---ZTA/Gdn**:<br>Imaginary frequency: −292.47 cm^{-1}<br>Gibbs Energy: -1175.51074833 Hartree<br>Relative Gibbs Energy = 32.32 kcal/mol			
C	-0.27935024698376	1.04147179325518	-0.67571380921654	C	-0.38560328403924	1.25078341049335	-0.56924539271944
C	-0.27988295928246	2.42449472431180	-0.01844403340383	C	-0.39477516651222	2.64481146674891	0.06162729004802
N	1.02632466878464	0.55886296551573	-1.06501173651621	N	0.91214009592111	0.62343143534180	-0.60171145192989
C	-0.95035498612702	-0.00971544678411	0.17095062282389	C	-1.32880352192325	0.28949595013396	0.14534474615891
H	-0.88856374425766	1.14453536743346	-1.58110413410563	H	-0.70865479737571	1.37295683555661	-1.60488045060778
H	-0.95138049238828	-0.99263871689269	-0.30339534268143	H	-1.59499216214831	-0.56426865072395	-0.47195889911739
O	-1.29788255310518	3.12109758867899	-0.05885382508933	O	-1.47603397525043	3.23229064803148	0.23231151522367
S	-1.59574763096578	0.19610064297396	1.62315390147452	S	-1.25160122349665	0.10041888425329	1.80370830024565
H	1.49329159321925	-0.13592045683760	-0.50398424781117	H	1.28824244913090	0.27726200829122	0.26826022072939
C	0.95758939581402	4.08834620628853	1.25720426902803	C	0.87894873396996	4.53073950037696	0.91824221801277
N	0.84058611187543	2.80363766750528	0.58675007890392	N	0.77289603513855	3.18883468006286	0.36598533284620
H	1.63850015388176	2.18992669319113	0.56000308589102	H	1.61200230029869	2.66344137498343	0.17924822676540
H	1.96017093729616	4.16755647379844	1.67349494067938	H	1.92966014883889	4.73859233222912	1.11102322618533
H	0.79468683270755	4.90581713223214	0.55155997889690	H	0.48658060350237	5.26777914420003	0.21451318703752
H	0.22452223177555	4.16878337052836	2.06248817732819	H	0.31836152340006	4.60195184021731	1.85202115967352
C	1.63552065603911	1.03936929862359	-2.18032673718339	C	1.52713527403318	0.27062596200085	-1.76595890684511
O	1.10391541230506	1.89971610733109	-2.87289306323240	O	1.07626800185717	0.57768574741921	-2.86182918776026
C	2.99344073624806	0.46661712691600	-2.49460199610939	C	2.79096567585107	-0.53618451211509	-1.60765054234825
H	2.98969840016607	0.10878309876337	-3.52544469538135	H	2.55732420608778	-1.57843697939846	-1.84085058826108
H	3.73134766629687	1.26800367556122	-2.41683720930574	H	3.53059304177451	-0.18277618387740	-2.32611213970888
H	3.27256607788286	-0.34692295140649	-1.82512555211984	H	3.19729990111309	-0.48428048679851	-0.59738153022765
O	-3.64438740056973	2.06870111771823	-1.17653151741715	O	-2.95988114549564	1.21165015846401	-0.36100011462833
H	-2.84081459371107	2.44327290876952	-0.76700220897570	H	-2.64423895449593	2.12436074537266	-0.10184308411373
H	-3.94186213439678	1.39198611065144	-0.55951954333304	H	-3.59017342072843	0.92953097799546	0.31714086651539
C	-0.80795237636762	-4.00448591592211	1.35589456523009	C	-0.65342669123994	-3.66529286087822	0.73830909994400
N	-0.85897555808806	-5.28348014914712	0.99299078872071	N	-0.59595852424749	-4.87822615014699	0.19476302114382

N	0.37134228616442	-3.40573382240573	1.50529721366885
H	-1.74315318966340	-5.74402894433519	0.85549397553725
N	-1.93206632622517	-3.32737882972878	1.56869621066504
H	-2.82627065326061	-3.76979240250211	1.43540689699326
H	-1.91057501606494	-2.33770902260373	1.77577945437795
H	1.22705514904536	-3.91658738770354	1.36474039798028
H	0.43098400541903	-2.45734255340044	1.83545793279674
H	-0.01570389422049	-5.81595893768597	0.85855851940952

N	0.44728141478138	-2.91991332546550	0.81912233266991
H	-1.42270178645571	-5.44625129652706	0.11570344231953
N	-1.80092685425795	-3.19743140498209	1.21918605855075
H	-2.64647770957847	-3.73678683776429	1.13627011526252
H	-1.85272243641587	-2.24366454762283	1.56505462437532
H	1.31077722758272	-3.22911651054434	0.40516771816371
H	0.39809549442172	-1.99053309158110	1.20599759100820
H	0.27239952595812	-5.24348026374672	-0.15857800461174

Product **ZTA + Gdn**:
Gibbs Energy: -1175.52622072 Hartree
Relative Gibbs Energy = 1.84 kcal/mol
$\Delta G^{\ddagger}$: 22.61 kcal/mol

C	-0.38205178298258	1.22432889218648	-0.62301481089465
C	-0.38297173533615	2.58949502841945	0.02951672221724
N	0.92180385689253	0.62784413703562	-0.61381306402261
C	-1.41372528181109	0.30148647766074	0.05080134408972
H	-0.67267896345252	1.38759621990033	-1.66624291654913
H	-1.42077333080289	-0.63177708881128	-0.51890527723818
O	-1.51174106981414	3.16277480862421	0.30015910581731
S	-1.03995905860458	0.00104094428784	1.77953573947252
H	1.21541274826887	0.21467874459031	0.26068386648797
C	0.76707053165397	4.55919626618986	0.86770750935408
N	0.71550992186427	3.22122287715722	0.27972862741547
H	1.57787280590221	2.74441888234155	0.04964776467672
H	1.81284668444813	4.82605947361504	0.99602429363977
H	0.28039577565474	5.27455331838790	0.20420896191624
H	0.26328119493155	4.55592239739251	1.83441976237881
C	1.58721218066880	0.30712211700833	-1.75969093331689
O	1.19857805974902	0.66873790209479	-2.86270340259576
C	2.82392438094786	-0.53362136617142	-1.57065278051360
H	2.57214038789136	-1.56576539851933	-1.82911920026106
H	3.59829185173331	-0.18828678620836	-2.25543190724595
H	3.19253386753341	-0.50859276187588	-0.54501422256814
O	-2.69263613930911	0.95398339935654	-0.14381994286427
H	-2.23630944809175	2.48027388032257	0.15028684190728
H	-3.28470847860435	0.61880857569470	0.54290920861807
C	-0.73908257327333	-3.63671879112423	0.70898847133364
N	-0.66657630962674	-4.86644245675435	0.20244720549912
N	0.34611861078995	-2.87075195960623	0.77715658521420
H	-1.48729756055344	-5.44445424284215	0.13540443098861
N	-1.89490216730466	-3.15956961602303	1.16161190729132
H	-2.73487906708698	-3.71067571911466	1.11165127831181
H	-1.92497334068570	-2.20680279045784	1.52144081753997
H	1.22808783597316	-3.19029684273228	0.41395798622336
H	0.25300112225210	-1.92864225578149	1.14982570871655
H	0.20456593289523	-5.22653080004334	-0.14883703745619

Product **TA + Gdn** (species calculated separately):
Gibbs Energy: (-969.59170527) + (-205.97054166) Hartree
Relative Gibbs Energy = 0.00 kcal/mol

TA

C	-0.18525949388595	-0.16272586281982	-0.59468274914863
C	-0.13236360469054	1.27669550162245	-0.05232747894237
N	0.68391772454760	-1.07355290320417	0.12074700187186
C	-1.61775304845272	-0.71617933226041	-0.63112152090214
H	0.16222335522286	-0.11654214796206	-1.63108693375168
H	-1.58560838908428	-1.68987112026267	-1.12350052211798
O	-1.16009034496142	1.90111466053285	0.23498933517510
S	-2.28366074121968	-1.10114336245860	1.02699902200720
H	0.28007708556339	-1.58722251274503	0.88978867663095
C	1.30616889325962	3.15874655740204	0.51618301708887
N	1.08861862460070	1.79846148522094	0.05286021211099
H	1.86956595640929	1.25599576820621	-0.29170737524835
H	2.37868714194775	3.34323136380457	0.54598959657809
H	0.83491843081809	3.88051561561796	-0.15552550590141
H	0.89201613842587	3.29266275681422	1.51746554361326
C	1.98555956704746	-1.24802501663981	-0.18394017953092
O	2.54825187259342	-0.57122732241050	-1.04797689861852
C	2.71822184451346	-2.31019352707112	0.59442439857626
H	3.09089293957915	-3.05578572416508	-0.11098978576104
H	3.57831327623702	-1.85107225182180	1.08527172369018
H	2.08931133685570	-2.79855359348054	1.33851344992721
O	-2.45427071534905	0.10981415920702	-1.39227469756624
H	-2.14996219185688	0.12935316231328	1.53853752479936
H	-2.41616835732087	0.98530175186005	-0.97279125068004

Gdn

C	-5.83432803517862	2.61277209222037	0.10813600026502
N	-4.82052479485062	2.14787000217135	0.83345156551696
N	-5.70124181293328	2.75558222695354	-1.20787200744014
H	-4.90764029632897	2.04009247655337	1.83044087188343
N	-6.98325895383676	2.93055025938593	0.69846399639979
H	-7.09553224822764	2.83223040988631	1.69363215442021
H	-7.74963511279270	3.30810248546118	0.16668082024360
H	-4.83224824083322	2.53535049306750	-1.66549376783994
H	-6.45927482494574	3.11030382031482	-1.76707924118528
H	-3.94944663547240	1.89153111868562	0.39939365813633

Figure P7.3: Structures and relative Gibbs energies of reactants, products and transition states for the conversion of thioaldehyde **CT** into zwitterionic hemi-thioacetal **ZTA**, via transition state **CT---ZTA**.

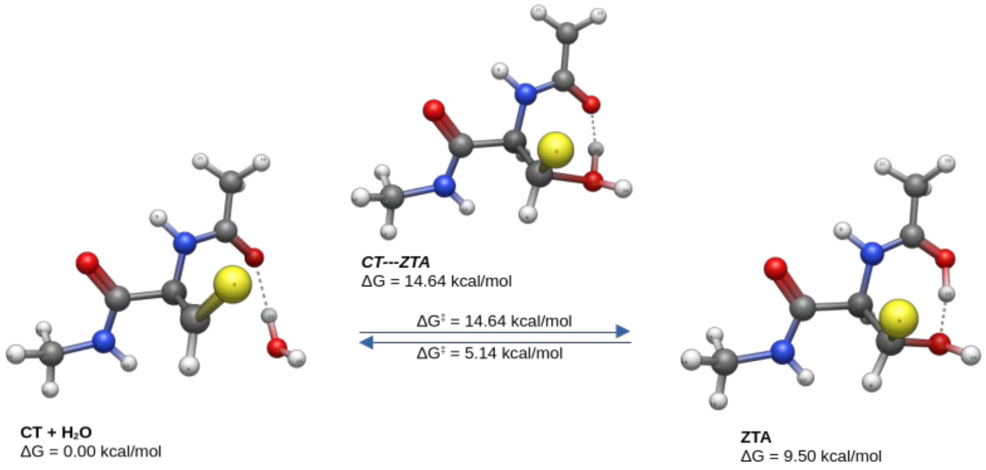

Table P7.3: Cartesian coordinates, Gibbs energies (Hartree), relative Gibbs energies (kcal/mol) and imaginary frequencies, where relevant, of reactant cysteine thioaldehyde and water **CT+H_2O**, product zwitterionic hemi-thioacetal **ZTA** and connecting transition state **CT---ZTA**. All species calculated at the following level: (wB97X-D4/def2-TZVP/def2/J, CPCM: water).

Reactant **CT + H_2O**:
Gibbs Energy: -969.57775553 Hartree
Relative Gibbs Energy = 0.00 kcal/mol
$\Delta G^{\ddagger}$: 14.64 kcal/mol

C	-0.00645707912244	-0.07084968433247	0.16977419589264
C	1.26664274253170	-0.93633398555841	0.26021833336843
N	-0.26365905291103	0.20789766975097	-1.21213024377435
C	-1.11628735844339	-0.81618305555141	0.87910796818797
H	0.15924826420355	0.85992417842045	0.72052815722588
H	-0.95258432555367	-0.91831637888583	1.95187447261123
O	1.61874385715277	-1.61793406051847	-0.69623369388740
S	-2.41596341024578	-1.44120845477285	0.18205647733228
H	0.14523700612807	-0.42402590582074	-1.88381030202591
C	3.07409751777905	-1.72658422420062	1.68891639604135
N	1.89632563332854	-0.91096618842070	1.43563851064825
H	1.57747571359320	-0.28099000229113	2.15423240872108
H	3.36959349781073	-1.58684663769726	2.72715356514403
H	2.85028617682713	-2.78106658071542	1.51667711019642
H	3.89886965026688	-1.43086337886940	1.03660443558158
C	-1.09809660313649	1.18139992816249	-1.61327016311751
O	-1.65984203807888	1.93957811636590	-0.81718119644513
C	-1.31305629151162	1.30615751423126	-3.09870018490333
H	-2.36249795326408	1.09078007391697	-3.31154525693806
H	-1.11292770781530	2.33687632070046	-3.39597358747083
H	-0.68165222722644	0.62823741816554	-3.67280271944319
O	-2.14569240581940	2.03349953126214	1.94785702935767
H	-1.98626994544705	1.97887082926046	0.98737411480629
H	-2.84987952909535	1.39903417882346	2.11693446365959

Transition state **CT---ZTA**:
Imaginary frequency: −309.4 cm^{-1}
Gibbs Energy: -969.55443033 Hartree
Relative Gibbs Energy = 14.64 kcal/mol

C	0.03610644348068	0.16433943027179	0.25656777659630
C	1.19972478836947	-0.84361204391882	0.29601866017746
N	-0.32984236067089	0.33156431098625	-1.12732828046959
C	-1.07633939765139	-0.38569224961731	1.15342236452036
H	0.38808366217718	1.11398275630437	0.66955233185069
H	-0.73617556461659	-0.49245464436263	2.17970274622632
O	1.45595937349253	-1.54550392128544	-0.67714549534100
S	-2.16800788718344	-1.52930857296661	0.56310338059039
H	0.07509553715146	-0.33897596687718	-1.76563982136155
C	2.96749597380353	-1.82663780268289	1.65471416168163
N	1.86601472370495	-0.89890166952261	1.45041250895308
H	1.63952900080921	-0.24597961227776	2.18341594932627
H	3.31152733932845	-1.72753541037517	2.68263089033208
H	2.63679928598909	-2.85272260830217	1.48310848159939
H	3.79360949151995	-1.60517753702075	0.97505442492900
C	-1.13458691616374	1.28973080689555	-1.58662915233014
O	-1.70219164857974	2.10469245577024	-0.83412566583954
C	-1.33502069292923	1.36028523999031	-3.07363359026381
H	-2.40093777704143	1.25500229485304	-3.28443711168032
H	-1.01907476017654	2.34732935065138	-3.41723679162956
H	-0.77685308256228	0.59175275552936	-3.60692400485444
O	-1.93149208683882	1.18285934129270	1.52539436543847
H	-1.91378202887041	1.65156768413046	0.62178678046086
H	-2.84564141654202	0.89939561253388	1.67821509108768

Product **ZTA**:
Gibbs Energy: -969.56261225 Hartree
Relative Gibbs Energy = 9.50 kcal/mol
$\Delta G^{\ddagger}$: 5.14 kcal/mol

C	0.07208648669183	0.26106913623108	0.32132864796448
C	1.19654987041111	-0.78394486395731	0.32198859379512
N	-0.31701246234833	0.40412384248969	-1.07638521922366
C	-1.07015931994967	-0.15797705562276	1.26339723086472
H	0.48114807039598	1.21570649936470	0.67067026792617
H	-0.61656733287085	-0.38232410581881	2.23030974668457
O	1.46905949562146	-1.41160251255780	-0.69965457666667
S	-2.05717234697162	-1.52561822519564	0.68716762196340
H	0.19207118048447	-0.21861302012805	-1.69822594020859
C	2.91882287327849	-1.90675172678435	1.62660261629548
N	1.83566060893223	-0.94681304516195	1.47944930757178
H	1.60512048570887	-0.35591555170256	2.26136434365472
H	3.26607587531544	-1.87367861290693	2.65766867565155
H	2.56894254071171	-2.91507143103390	1.39709853822957
H	3.74754019567786	-1.66123006124688	0.95870399591894
C	-1.17854906064445	1.23253921939916	-1.58761371330044
O	-1.91813513650644	2.00479804286611	-0.86479400876936
C	-1.34826408633281	1.33301248250929	-3.06154227373946
H	-2.37796334671735	1.06842692665073	-3.31025336305655
H	-1.18454125877544	2.37176081848927	-3.35577869702697
H	-0.65480879284402	0.68225919726357	-3.58992642615383
O	-1.85912724897973	1.05694237961446	1.46778325582287
H	-1.91349524700786	1.73299854531731	0.12690360365439
H	-2.74893617646562	0.75581589886490	1.70043748029144

P8 Transformation of Hemi-thioacetal into Formylglycine

The transformation of cysteine hemi-thioacetal (**TA**) into formylglycine (**FG**) was explored under neutral conditions (mediated by a water molecule) for two different cysteine hemi-thioacetal conformers (**TA^1** and **TA^2**). Both approaches gave similar barriers (ca. 29 kcal/mol), which were too high to represent feasible pathways. The transformation of the thiolate conjugate base of cysteine hemi-thioacetal (**TA-CB**) into formylglycine (**FG**) was also explored, again mediated by a water molecule. The latter pathway shows significantly lower reaction barriers and is the most likely mechanism for the transformation.

Figure P8.1: Structures and relative Gibbs energies of reactants, products and transition states for the conversion of cysteine hemi-thioacetal and water **TA^1 + H_2O** into formylglycine, water and dihydrogen sulfide **FG + H_2O + H_2S** , via transition state **TA^1/H_2O---FG**.

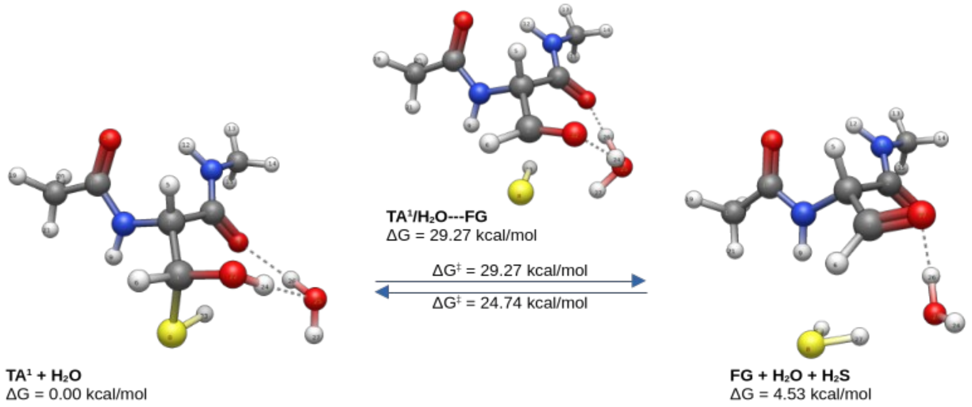

Table P8.1: Cartesian coordinates, Gibbs energies (Hartree), relative Gibbs energies (kcal/mol) and imaginary frequencies, where relevant, of reactant cysteine hemi-thioacetal and water **TA^1+H_2O**, product formylglycine, water and dihydrogen sulfide **FG+H_2O+H_2S** , and transition state **TA^1/H_2O---FG**. All species calculated at the following level: (wB97X-D4/def2-TZVP/def2/J, CPCM: water).

Reactant **TA^1+H_2O**:<br>Gibbs Energy: -1046.06285858 Hartree<br>Relative Gibbs Energy = 0.00 kcal/mol<br>ΔG‡: 29.27 kcal/mol				Transition state **TA^1/H_2O---FG**:<br>Imaginary frequency: −292.03 cm^{-1}<br>Gibbs Energy: -1046.01621764 Hartree<br>Relative Gibbs Energy = 29.27 kcal/mol			
C	-0.22492046560982	-0.47910195767224	-0.62621756908612	C	-0.26405014121587	-0.30607365176966	-0.77569938732173
C	-0.19907928283156	0.90063993718863	0.05059571291500	C	-0.23459731341509	1.09590847202882	-0.14165332996066
N	0.74046495492311	-1.36519684483638	-0.00557904620936	N	0.59374769105172	-1.22233224677066	-0.05876698081874
C	-1.60453107182973	-1.14893224044223	-0.69071471711371	C	-1.61475261726740	-0.95384728255536	-1.06259612472441
H	0.08700964519819	-0.33441120686567	-1.66304198725275	H	0.16828709080861	-0.18122223253472	-1.77662716675445
H	-1.46343868704355	-2.07964259450252	-1.24652582525342	H	-1.50586941331951	-2.02427010422096	-1.27256073209835
O	-1.07908380908482	1.30146099923335	0.82008464027292	O	-1.17784944434270	1.66146560544546	0.45613735385790
S	-2.22960471296270	-1.72954447954474	0.91849772957681	S	-2.28974063302404	-1.60902440767072	1.16993669862339
H	0.42405973406950	-1.93659922748604	0.76351715171583	H	0.14635951604693	-1.81750672469086	0.62490876353344
C	1.13769268229998	2.91488327780206	0.36946750360190	C	1.20511639636216	2.99869543021733	0.34015519944903
N	0.88300347580802	1.62133973273944	-0.24182992220881	N	0.93675523183079	1.69409029173538	-0.24660883700417
H	1.58773236435259	1.19689590342064	-0.83061270725157	H	1.68296699562560	1.18605545974284	-0.71044553031159
H	2.06995587175729	3.30703515041074	-0.03363639569937	H	2.23616080917615	3.26496683530884	0.11622670565278
H	0.32803330127652	3.61132225187598	0.14257803811582	H	0.53557328008099	3.74991069158021	-0.08221775726448
H	1.22503124706705	2.82292050622754	1.45484166011946	H	1.06400832622702	2.96854274935511	1.42231019955223
C	2.03201256292719	-1.41622685739878	-0.39296612705637	C	1.90920998648445	-1.35126560979572	-0.32708535656237
O	2.47780408166964	-0.68135243130595	-1.27687345970318	O	2.48455649128723	-0.61336731572518	-1.13121818986736
C	2.90384190003618	-2.42105513834786	0.31531861971204	C	2.63875728550525	-2.44130614002668	0.41270460189838
H	3.32017483561108	-3.10141899547200	-0.43020475634575	H	3.04443997054110	-3.14055379398555	-0.32140787390996
H	3.73272042981671	-1.88941405376790	0.78663899120012	H	3.47599464118429	-1.99564835823409	0.95282401172434
H	2.36372909968639	-2.99483483632831	1.06846763032946	H	1.99696555136593	-2.97951112681618	1.10982984716749
O	-2.51122013404452	-0.37260041735732	-1.42340057836757	O	-2.53688443461528	-0.30652124225425	-1.60370663201994
H	-2.11303351143453	-0.56643569179241	1.57102446609543	H	-1.58055034680865	-0.78437387907584	1.95505578506999
H	-2.94209957214754	0.29379330860666	-0.85197452920762	H	-3.36730130461807	0.58580092956119	-0.62519809677794
O	-3.74186407811989	1.60962045848438	0.12193867263265	O	-3.51483739551470	1.00803973434806	0.28785983210684
H	-2.89351752365692	1.68806993967990	0.59136052955025	H	-2.47514852291163	1.31881243572778	0.43041041226613
H	-4.36088442214121	1.24965919204885	0.76751033215140	H	-3.55731769652531	0.19453548107544	0.85743258449422

Product **FG+H_2O+H_2S**:
Gibbs Energy: -1046.05564591 Hartree
Relative Gibbs Energy = 4.53 kcal/mol
$\Delta G^{\ddagger}$: 24.74 kcal/mol

C	-0.07787025842515	-0.04421601765802	-1.19787872578537
C	-0.06024579960064	1.32916158339677	-0.50943758546397
N	0.42706591497122	-1.06664081000709	-0.31020475442859
C	-1.44600116208367	-0.40573545622800	-1.74485763265346
H	0.58641955886374	-0.00645840618656	-2.06799367682257
H	-1.77373339872237	-1.44634415673355	-1.56594314317783
O	-1.03812009771908	1.78548971798318	0.08699926536972
S	-2.01900270377703	-2.40360404098138	1.85341115343973
H	-0.20975484467793	-1.51344330688065	0.33909187283456
C	1.36027038968355	3.19916429100824	0.14250264934300
N	1.11623456734912	1.95166759365175	-0.56336021313810
H	1.88826118784501	1.46783091202863	-0.99806399269810
H	2.38281398622816	3.51292650130392	-0.05978812302856
H	0.67210100373087	3.97255695305642	-0.20333404601124
H	1.22847481678889	3.06872946059255	1.21937596975068
C	1.74864131412914	-1.35083721021161	-0.25282213142062
O	2.56927868536209	-0.75966893453174	-0.95371659947789
C	2.15485478099361	-2.42984745488294	0.71755686486120
H	2.71543238541099	-3.19196554083803	0.17333621972553
H	2.81967852526617	-1.99045535921529	1.46419876453380
H	1.30232800910638	-2.88992371101603	1.21722718236557
O	-2.11673373231804	0.37241390058009	-2.37300908615953
H	-1.50617186757867	-1.80376586854172	2.93569464986107
H	-3.97467705753785	0.44533331557271	-0.03023957692355
O	-3.41960764819995	0.51118613396991	0.75414696494895
H	-2.61382929093256	0.98052244799224	0.46189181692658
H	-2.66609618014092	-1.29495022584990	1.43695185585460

Figure P8.2: Structures and relative Gibbs energies of reactants, products and transition states for the conversion of cysteine hemi-thioacetal and water **TA^2 + H_2O** into formylglycine, water and dihydrogen sulfide **FG + H_2O + H_2S** , via transition state **TA^2/H_2O---FG**.

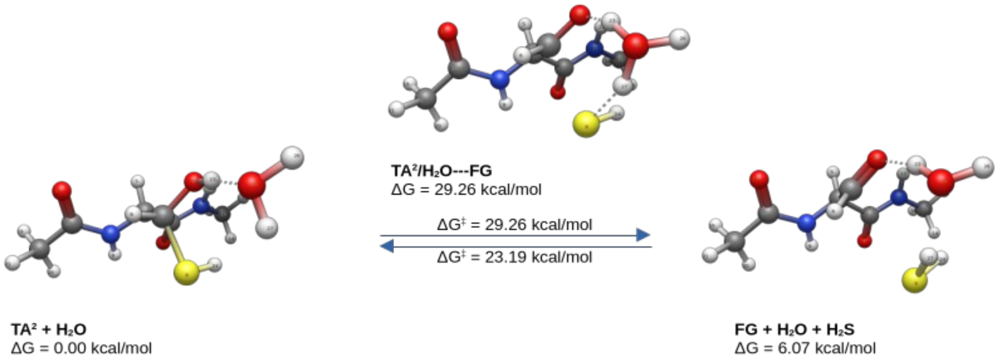

Table P8.2: Cartesian coordinates, Gibbs energies (Hartree), relative Gibbs energies (kcal/mol) and imaginary frequencies, where relevant, of reactant cysteine hemi-thioacetal and water **TA^2+H_2O**, product formylglycine, water and dihydrogen sulfide **$FG+H_2O+H_2S$** , and transition state **TA^2/H_2O---FG**. All species calculated at the following level: (wB97X-D4/def2-TZVP/def2/J, CPCM: water).

Reactant **TA^2+H_2O**:
Gibbs Energy: -1046.06710299 Hartree
Relative Gibbs Energy = 0.00 kcal/mol
$\Delta G^‡$: 29.26 kcal/mol

C	0.37526372871408	0.49956773548718	-0.26001207469173
C	1.46941218141118	-0.37956239787443	0.35642834520985
N	0.47737426071735	0.40639595520351	-1.69173808245892
C	-1.03864775678618	0.13129543357416	0.22510506058035
H	0.54453796691113	1.53538253511033	0.05025525492551
H	-1.74861029346608	0.63079196157633	-0.44096437757587
O	2.24826670797250	-1.01665378839366	-0.35133899293227
S	-1.42833352287111	-1.63753688770235	0.00943493828798
H	1.05422860595877	-0.33779264500305	-2.05292827573197
C	2.45267991212577	-1.23044523393052	2.41632444461855
N	1.50694917716241	-0.39934640419178	1.69144514555031
H	0.77809676206596	0.08383587483055	2.19495570798016
H	2.30752967958057	-1.06463665164213	3.48259537604488
H	2.29610811526126	-2.28895732685404	2.19272057867701
H	3.47804519766669	-0.96653550937549	2.14978026087415
C	0.00213411811291	1.36517930724818	-2.51200053131920
O	-0.61355578748392	2.34258955033499	-2.08650467263766
C	0.26285739934893	1.16801655742561	-3.98554089382745
H	0.83799677830567	2.02061922620076	-4.35241188244760
H	0.80406025825306	0.24621031938481	-4.19943365742635
H	-0.69453291324387	1.15709514676250	-4.51000984901262
O	-1.17431117019356	0.57706135805942	1.54597709979044
H	-2.08812516504261	0.38806353203005	1.84765193837287
H	-0.53538609737023	-2.11290768552005	0.88935050297136
O	-3.75999067705085	-0.07079165161773	2.22964347252420
H	-4.01783736012619	0.04145314781521	3.15243333192149
H	-3.83038351327818	-1.01845599666044	2.06066181720123

Transition state **TA^2/H_2O---FG**:
Imaginary frequency: −346.22 cm^{-1}
Gibbs Energy: -1046.020475 Hartree
Relative Gibbs Energy = 29.26 kcal/mol

C	0.42048819301505	0.65793477611156	-0.24827204422481
C	1.36496682648504	-0.35627139279355	0.40752240729969
N	0.41722050479453	0.45376821076629	-1.66837936438492
C	-0.98647380816892	0.64647406283734	0.32957363951631
H	0.80455810922495	1.66157107301715	-0.02380400873056
H	-1.78846295354202	0.75117334952965	-0.40511220328589
O	2.03093941625667	-1.12718009219671	-0.27778993892358
S	-1.53015408029015	-1.79287461464571	0.14082277689957
H	0.83208383813022	-0.40108608733433	-2.00544656481932
C	2.21359988578185	-1.25992872466081	2.50123283816991
N	1.41925737572468	-0.30999599122402	1.74073902780000
H	0.76619228708602	0.28861494024316	2.22340425210845
H	2.13001709056118	-1.00860891850947	3.55736773936269
H	1.85838826977424	-2.28231613540376	2.34530770744680
H	3.26213628402384	-1.20561255009400	2.20285669683787
C	0.03032806394545	1.42386491568280	-2.52209410177335
O	-0.40106790783553	2.50457918383448	-2.12039439192686
C	0.15077272572157	1.10655039029701	-3.99166875116846
H	0.81525584810679	1.84151542244626	-4.45060877620237
H	0.53582662445489	0.10365504649216	-4.17645375335898
H	-0.83388618768600	1.20892450741282	-4.45183079214105
O	-1.17051616267615	0.95376484487355	1.52832567622204
H	-2.41135758148535	0.39440511638031	1.94680997879300
H	-0.69352027165486	-2.20340973118043	1.10425490985909
O	-3.19826690071180	-0.32180354957922	2.01066958991632
H	-3.26148172678614	-0.66380623688232	2.91699154508306
H	-2.77684376225006	-1.06390181542018	1.38597590562538

Product **$FG+H_2O+H_2S$**:
Gibbs Energy: -1046.05742756 Hartree
Relative Gibbs Energy = 6.07 kcal/mol
$\Delta G^‡$: 23.19 kcal/mol

C	0.57466982876616	0.75924558567003	-0.29342773515804
C	1.39249884870803	-0.37226451332128	0.35801479927895
N	0.57451317435967	0.57003925602508	-1.71563070283915
C	-0.84341084527860	0.81813650868896	0.25290673771581
H	1.05364196819337	1.70611750524351	-0.01845888099969
H	-1.63428314100242	0.49421297610094	-0.44065068662252
O	1.94700755327474	-1.21887931151461	-0.33469006896170
S	-1.46163155016954	-2.46336293160167	0.54869989052795
H	1.06147946160439	-0.23826437730077	-2.07269224942105
C	2.20100395846605	-1.32895669379790	2.44419341146113
N	1.46670536200122	-0.32777186191567	1.68824333456202
H	0.95625207635521	0.38712702356370	2.18236805485645
H	2.11150120201825	-1.09163528809946	3.50277186832358
H	1.79367920735026	-2.32652422677571	2.26316599313650
H	3.25623027362050	-1.32568524138074	2.16327992463869
C	0.01709227542288	1.49024077829756	-2.52805097181378
O	-0.54035770209356	2.48708628708589	-2.06643427130014
C	0.10576261551376	1.22768701175313	-4.00904723043226
H	0.61429168577308	2.07078640345481	-4.48075913664150
H	0.63846348280352	0.30524957445324	-4.24035729660794
H	-0.90682353170619	1.17511555846703	-4.41378998325280
O	-1.09068032432275	1.19327377228129	1.37610531375112
H	-2.84364268540661	0.50667197037182	1.93042207320860
H	-0.70609026572289	-2.52834631696598	1.65206295108859

O	-3.62058948647283	-0.06272463182019	2.04967001833690	
H	-3.65915559134750	-0.23245324862440	2.99738594123079	
H	-2.42395444423660	-1.74405703161979	1.15281891509477	

Figure P8.3: Structures and relative Gibbs energies of reactants, products and transition states for the conversion of cysteine hemi-thioacetal conjugate base (S-centered anion) and water **TA-CB^1 + H_2O** into formylglycine, water and hydrogen sulfide anion **FG + H_2O + HS^-**, via O-centered anion intermediate **TA-CB^2 + H_2O** and respective transition state structures **TA-CB^1/H_2O---TA-CB^2** and **TA-CB^2---FG/H_2O/HS^-**.

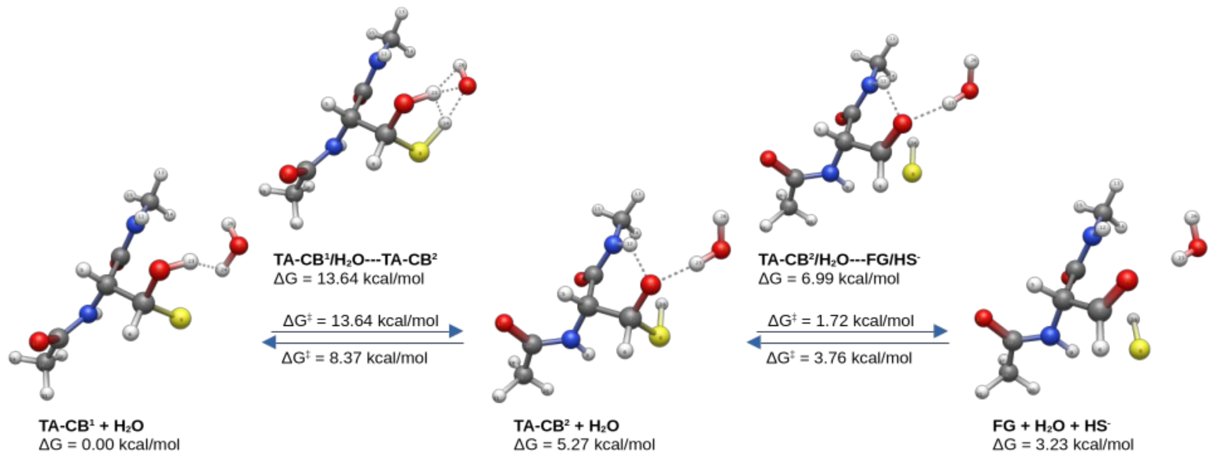

Table P8.3: Cartesian coordinates, Gibbs energies (Hartree), relative Gibbs energies (kcal/mol) and imaginary frequencies, where relevant, of reactant cysteine hemi-thioacetal conjugate base (S-centered anion) and water **TA-CB^1 + H_2O**, intermediate O-centered anion intermediate **TA-CB^2 + H_2O**, product formylglycine, water and hydrogen sulfide anion **FG + H_2O + HS^-**, and respective transition state structures **TA-CB^1/H_2O---TA-CB^2** and **TA-CB^2---FG/H_2O/HS^-**. All species calculated at the following level: (wB97X-D4/def2-TZVP/def2/J, CPCM: water).

Reactant **TA-CB^1+H_2O**:<br>Gibbs Energy: -1045.60564524 Hartree<br>Relative Gibbs Energy = 0.00 kcal/mol<br>ΔG‡: 13.64 kcal/mol				Transition state **TA-CB^1/H_2O---TA-CB^2**:<br>Imaginary frequency: −319.38 cm^{-1}<br>Gibbs Energy: -1045.58391172 Hartree<br>Relative Gibbs Energy = 13.64 kcal/mol			
C	0.13864079450918	0.59095691203499	-0.31674226607470	C	0.14622012946702	0.58406227694959	-0.25754330629710
C	0.95143989819525	-0.46577801108660	0.42806802021948	C	0.97005047634011	-0.47011394184204	0.47893546394017
N	0.34338292317335	0.40552745764709	-1.73164727137873	N	0.31234672792165	0.37922933036243	-1.67506347979505
C	-1.36502103007432	0.54190672864968	0.04617527249500	C	-1.35023274321840	0.55829668746446	0.14135164999903
H	0.50840469513792	1.58202949581747	-0.03732434863505	H	0.52308798810038	1.57642837617669	0.00746104993734
H	-1.85765006952211	1.22122644245010	-0.65424045921044	H	-1.85234500657201	1.23488062815455	-0.55664029763273
O	1.54835389351830	-1.36677399367526	-0.16250372000553	O	1.56608394423826	-1.36601988781343	-0.12070219852347
S	-2.08555378136531	-1.10771144643737	-0.12306631026428	S	-2.07708938527347	-1.11086121048137	-0.16115784360577
H	0.64416673508865	-0.50991804080620	-2.02611636882934	H	0.65843785781184	-0.52391889560625	-1.95973803959485
C	1.60345582804257	-1.30721112874717	2.62360360931974	C	1.63179617795696	-1.31302071041454	2.67074459501492
N	0.97409746077764	-0.32658135804272	1.75898596713451	N	0.99451511364821	-0.33457534179633	1.80881276822721
H	0.38901300450947	0.38855036232648	2.16174115834209	H	0.38306734152870	0.36088093916966	2.21015829604643
H	1.52325019833599	-0.96088938708947	3.65316039895646	H	1.55201518656138	-0.96779806772863	3.70065891678117
H	1.11587757782299	-2.28269193538954	2.53577604227137	H	1.15026730866147	-2.29143824913459	2.58322663243151
H	2.65877267566876	-1.42333238091353	2.36898040842248	H	2.68701276270752	-1.42123562662275	2.41273800555669
C	0.23017068225131	1.40071789263488	-2.62755261593299	C	0.16688902501156	1.36442588472767	-2.58043070243281
O	-0.07951982403429	2.55082274872647	-2.30756293388201	O	-0.16383191863921	2.50894856178027	-2.26529347782931

C	0.50583527213225	1.03141929687929	-4.06632805763366
H	1.32262556469201	1.65513731739489	-4.43520612841982
H	0.76706572572412	-0.01969556282293	-4.19136879089356
H	-0.38292334083784	1.25639773478505	-4.65934318951169
O	-1.52339543675709	1.11504539313536	1.35139837419226
H	-1.87667279733142	0.43352187700375	1.94350925381021
H	-2.23224026066134	-1.45329369344397	2.04506384378317
O	-2.29724024023395	-1.21882623071775	3.00069946423216
H	-1.48480112753835	-1.55237256762446	3.39775489907423

C	0.43018825309929	0.98620520977262	-4.01881942892463
H	1.22707545475823	1.62450779002759	-4.40582636756555
H	0.71365859592450	-0.05998902240420	-4.13633441566685
H	-0.47200447271817	1.18440511644915	-4.60101644037619
O	-1.53839481764754	1.00165836901106	1.44680848191355
H	-1.81094268876370	0.18537143247554	2.08400742962787
H	-2.18468419476874	-1.36136781119038	1.22966126791597
O	-2.21704111684687	-0.96544172561451	2.77382128039988
H	-1.44614599928896	-1.36352011187225	3.19018016045253

Intermediate **TA-CB² + H_2O**:
Gibbs Energy: -1045.59724855 Hartree
Relative Gibbs Energy = 5.27 kcal/mol
$\Delta G^{\ddagger}$: 8.37 kcal/mol (back to **TA-CB¹+H_2O**)
$\Delta G^{\ddagger}$: 1.72 kcal/mol (forward to **FG + H_2O + HS^-**)

C	-0.13673286702194	0.66782009389819	-0.32983456445093
C	0.91750294856864	-0.24210625712353	0.31619899246930
N	-0.12654764703722	0.54502252129024	-1.76362788223853
C	-1.56627123255395	0.44536587756356	0.24120742800830
H	0.12717245247433	1.69869527240893	-0.08241883817791
H	-2.24529018107873	1.01137876178492	-0.42321813232445
O	1.75538331271522	-0.85514171374457	-0.34620410523210
S	-2.10197300640426	-1.32788280179507	-0.21622099527214
H	-0.60222112423565	-0.25285936804121	-2.15589744422864
C	1.68551297507357	-1.16608950525815	2.43976342315554
N	0.84619993847732	-0.28543643002538	1.65167637472304
H	-0.00111688098567	0.13108215785494	2.04242816773997
H	1.46323896141531	-1.00446783950934	3.49422190477761
H	1.50493903395288	-2.21788392088949	2.19615397672276
H	2.74171784272196	-0.95019293472521	2.26484227985884
C	0.67264996294596	1.28079571246619	-2.56033085465852
O	1.38647102925337	2.18960736007639	-2.13392017878612
C	0.63702396474513	0.93427868621835	-4.03045816478376
H	1.62636705961257	0.57602384301583	-4.32389217678233
H	-0.10566029165742	0.17210242627276	-4.26821873951799
H	0.42519847420999	1.84089835748161	-4.59996578249169
O	-1.68093161270860	0.73275703128151	1.53597517848698
H	-2.07462881965602	-0.42215392810331	2.66555094421677
H	-1.25400419685648	-1.96489032755490	0.60292485300422
O	-2.24176518523422	-1.08495152681797	3.39014817825084
H	-1.79615877549916	-0.72331141425652	4.16259019686280

Transition state **TA-CB²---FG/H_2O/HS^-** :
Imaginary frequency: −141.8 cm^{-1}
Gibbs Energy: -1045.59450559 Hartree
Relative Gibbs Energy = 6.99 kcal/mol

C	-0.09306066147888	0.76800366095819	-0.29585575768069
C	0.90126147516239	-0.20850396033879	0.35090213649156
N	-0.13852738246043	0.61135543175377	-1.72105163117592
C	-1.50794449597324	0.72615116935427	0.29463646193844
H	0.27388568022556	1.77928389491159	-0.08637859072021
H	-2.26575986735351	1.06707442277473	-0.42324199783475
O	1.69036633826587	-0.86305185342894	-0.32616568332363
S	-2.06050510696962	-1.52347860326594	-0.36266120432807
H	-0.64607820807584	-0.19653945449493	-2.05403363273531
C	1.68404604268848	-1.17475914063538	2.44838402521552
N	0.86369387477762	-0.25022326918100	1.68815343994281
H	0.08780576672103	0.22253635645175	2.13331689960324
H	1.50302564729042	-1.00419031916904	3.50897726175030
H	1.44039793200703	-2.21386427553742	2.20751123399020
H	2.74272669170964	-1.01051765537041	2.23781313299513
C	0.67592168968594	1.28630439108799	-2.55748885381288
O	1.43336904718515	2.17830783712287	-2.17505608622138
C	0.59709177401134	0.88892827382446	-4.01220504438263
H	1.56146803014618	0.46824869060714	-4.30628571457692
H	-0.18786685177599	0.15820117369072	-4.20849298744270
H	0.42324496826723	1.78369040265596	-4.61236398153713
O	-1.68788914984138	0.86949928916132	1.51925095087708
H	-2.10268854401779	-0.49927323758533	2.71033182916222
H	-1.17973130836990	-2.09565345948310	0.46961490495081
O	-2.21144330828429	-1.17435768754078	3.40315116800962
H	-1.83634507354297	-0.77135507832371	4.19332272084526

Product **FG + H_2O + HS^-**:
Gibbs Energy: -1045.60050156 Hartree
Relative Gibbs Energy = 3.23 kcal/mol
$\Delta G^{\ddagger}$: 3.76 kcal/mol

C	-0.07672987236624	0.91077744638688	-0.22083807771213
C	0.81589081860406	-0.14819032676248	0.45565856390338
N	-0.18920565036008	0.69071634552843	-1.63217724811956
C	-1.46314627398584	1.08391483918373	0.38135645882582
H	0.41883047407209	1.87824951649734	-0.06765573506120
H	-2.23911194575655	1.34189618764788	-0.35434541385549
O	1.46831546339533	-0.94007668867145	-0.21563281779511
S	-2.27683592349909	-1.65144350320727	-0.79374420976886
H	-0.80402650680977	-0.07629066584148	-1.89441135744831
C	1.63823557735080	-1.05907237679582	2.56064657135565
N	0.86659216729029	-0.09849148475371	1.79148241793671
H	0.22535075603759	0.51471114294367	2.26928995480899
H	1.53939014065530	-0.81104239227708	3.61652224690908
H	1.27517779617466	-2.07724353460062	2.39689025696565
H	2.69271342589641	-1.01632611650128	2.28094428686126
C	0.70088634375387	1.19855748599310	-2.50942798738985
O	1.57788698482907	1.99620343224281	-2.17872952700959
C	0.54702642406699	0.73653092893989	-3.93800642527725
H	1.44407074768843	0.17961307288533	-4.21795929338474
H	-0.32892950657199	0.10377271282378	-4.08234651760705
H	0.47836006583163	1.61200142129552	-4.58604036291327
O	-1.69591269380609	1.07706409745720	1.57134143278810

H	-1.99007564477021	-0.61908057231000	2.70706495600828	
H	-1.20636622577616	-2.25986208089416	-0.26296193354125	
O	-2.00470973201757	-1.41231746353529	3.25871057024903	
H	-1.34882334580017	-1.23939755738946	3.94306515032795	

P9 Enolisation of Cysteine Thioaldehyde

The mediation of the conversion of cysteine thioaldehyde **CT** into either the *E*- or *Z*-configured enethiol tautomers (***E*-ET** or ***Z*-ET**, respectively) was explored by using one or two water molecules, or acetate.

Figure P9.1: Structures and relative Gibbs energies of reactants, products and transition state for the conversion of cysteine thioaldehyde **CT+H_2O** into O-protonated *Z*-enethiolate (**OP-*Z*-ETe**) via transition-state structure **CT/H_2O---OP-*Z*-ETe**, as mediated by one explicit water molecule. O-to-S proton transfer gives the Z-enethiol (**Z-ET** + **H_2O**).

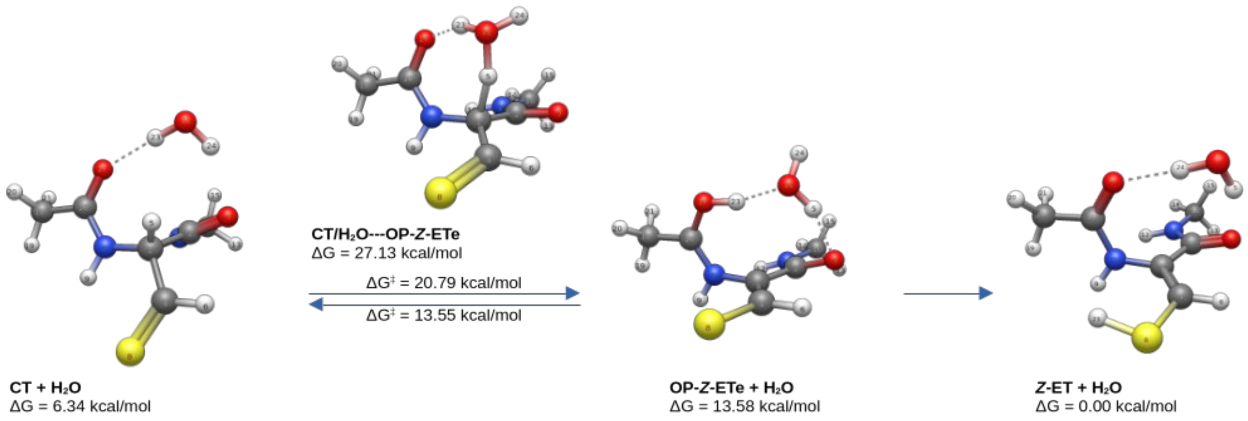

Table P9.1: Cartesian coordinates, Gibbs energies (Hartree), relative Gibbs energies (kcal/mol) and imaginary frequencies, where relevant, of reactant cysteine thioaldehyde and water, **CT+H_2O**, intermediate O-protonated enethiolate and water (**OP-*Z*-ETe + H_2O**), and transition state **CT/H_2O---OP-*Z*-ETe**. Data is also provided for product enethiol and water (***Z*-ET+H_2O**). All species calculated at the following level: (wB97X-D4/def2-TZVP/def2/J, CPCM: water).

Reactant **CT + H_2O**:<br>Gibbs Energy: -969.57918584 Hartree<br>Relative Gibbs Energy = 6.34 kcal/mol<br>$\Delta G^{\ddagger}$: 20.79 kcal/mol				Transition state **CT---OP-Z-ETe**:<br>Imaginary frequency: −908.3 cm^{-1}<br>Gibbs Energy: -969.54606013 Hartree<br>Relative Gibbs Energy = 27.13 kcal/mol			
C	-0.99432396495763	0.15619424137103	0.73005965825271	C	-0.80000136708088	-0.00145564260353	0.78690236868788
C	0.38450807740652	-0.32864857073050	1.23076463149183	C	0.43064515692520	-0.69253658538091	1.30107082749239
N	-1.19299227897914	0.01538310162200	-0.68316590173995	N	-1.03610207687731	-0.02036323745113	-0.62870241227341
C	-2.03093708741232	-0.56852503632951	1.54123875763061	C	-1.93528302289136	-0.03448681471048	1.62192665141396
H	-1.04560542543234	1.21066476319544	1.02403399108629	H	-0.43874218802353	1.36938507885639	1.16455681141686
H	-1.88046401094553	-0.45693975788896	2.61446619548239	H	-1.70040184754845	-0.23770332823286	2.66548619026913
O	0.84569547277598	0.13078996558542	2.27478191258679	O	0.72891794972822	-0.63861454562273	2.49714435807875
S	-3.24423963869501	-1.44154012147131	0.96025951413499	S	-3.50561043997582	0.23997848005910	1.18024985479973
H	-1.92021970327890	-0.61201160147587	-0.99744710491385	H	-1.77598274214475	-0.62752266392552	-0.95455139090520
C	2.27625246350800	-1.81354610403129	0.85929345978706	C	2.42063890709853	-2.00102136434542	0.76823343415812

N	0.98054063466369	-1.26225175679118	0.49639393706819
H	0.52863462944847	-1.57873843786545	-0.34663542136452
H	2.22520798448465	-2.30587649381325	1.83259290412246
H	2.56273572697344	-2.54101381962471	0.10208864847224
H	3.02886618868361	-1.02361101775655	0.90393348141805
C	-0.54776895100910	0.80822537530617	-1.56568952336872
O	0.28807523539045	1.63573052801659	-1.19999288688291
C	-0.88741297073784	0.61264398970327	-3.01879634984118
H	-1.68036452453139	-0.11935202389243	-3.16997183047874
H	-1.18982458756643	1.57428518113313	-3.43703120153125
H	0.01364896694957	0.28516830614021	-3.54169591095444
O	1.27269368925715	2.87750884696042	1.19877232741372
H	0.94498776310638	2.50408470964507	0.36366988487635
H	1.23082396493237	2.12979943817622	1.81062291818869

N	1.19341905069702	-1.32141175308616	0.39383022752836
H	0.86047431219478	-1.40431262271146	-0.55154397954327
H	2.21980887720081	-2.84864106206749	1.42880883636334
H	2.90332418358819	-2.36260753085375	-0.13858187266596
H	3.09541234959095	-1.31331831696027	1.28184442384560
C	-0.46289958426187	0.78165531699010	-1.54103534100683
O	0.37155118470565	1.65879815245793	-1.25552118982343
C	-0.84989558661935	0.54951101554577	-2.97534063464505
H	-1.65775558920180	-0.17357259145389	-3.08227188439513
H	-1.14317376430695	1.50341281350521	-3.41599387449635
H	0.03083441122862	0.18687609352168	-3.51054460809701
O	-0.01550905146256	2.47348362632469	1.11148243588638
H	0.31176152344748	2.36317166418043	0.15631798190534
H	0.75456935398924	2.55129581796429	1.69623278600581

Intermediate **OP-Z-ETe + H_2O**:
Gibbs Energy: -969.56765525 Hartree
Relative Gibbs Energy = 13.58 kcal/mol
$\Delta G^{\ddagger}$: 13.55 kcal/mol

C	-0.93966505037920	-0.35119979553875	0.62439142327062
C	0.33658158808046	-0.77221730959599	1.22447141920272
N	-1.05412303598217	-0.35587924194862	-0.80368193290120
C	-1.97663100707933	0.10473710808558	1.37408557913845
H	0.53127104746234	1.44790906579111	1.98179412298753
H	-1.79459912245456	0.07955108698423	2.44668628341319
O	0.63631894812980	-0.44684289866609	2.38867204160466
S	-3.46678012325366	0.69922805397502	0.81974399066171
H	-1.50211547210769	-1.13445316423628	-1.27139793703050
C	2.48447453587561	-1.90206397641673	0.93704659597319
N	1.18087574352356	-1.47906667244928	0.45639341428911
H	0.89201893427172	-1.76401814186199	-0.46413080390921
H	2.39077495540751	-2.61604547624578	1.75917763660956
H	3.01571673641009	-2.37566072425042	0.11272254781235
H	3.06009023279415	-1.04286269076291	1.28724200626465
C	-0.77721534949522	0.69007657394368	-1.53595398441092
O	-0.27440505350238	1.77050830147366	-1.04732247313119
C	-1.02669072700130	0.68668600595435	-3.00052675838241
H	-1.44839935341587	-0.26043070662480	-3.33074358475291
H	-1.71348220265458	1.50317851524688	-3.23285030315623
H	-0.08350883350489	0.87847586285691	-3.51563254989027
O	0.39967024355131	2.23720434927820	1.42279652691089
H	-0.07723080522257	1.80107517939636	-0.05020400918003
H	1.23853551417646	2.71368698987105	1.43467465527976

Product **Z-ET+H_2O**
Gibbs Energy: -969.58929543 Hartree
Relative Gibbs Energy = 0.00 kcal/mol

C	-0.87071421115131	-0.16020929868557	0.71495882379215
C	0.42184574863142	-0.69380688316042	1.24793119879881
N	-1.06568537197414	-0.13454305866774	-0.68972696808147
C	-1.79395249090997	0.27040560294629	1.57594218586288
H	0.99406582848057	1.61898299959748	2.17777361713661
H	-1.58464280844382	0.19041151472081	2.63672463880980
O	0.78575563486756	-0.45790441035786	2.40629411730324
S	-3.33916915520698	0.96462348309086	1.22336252875836
H	-1.74548727780618	-0.75376483683713	-1.10624167203551
C	2.44055659476676	-1.97629339975899	0.77660744900323
N	1.15317645216207	-1.42201175877235	0.39779550780352
H	0.78728727556210	-1.61583859139307	-0.51962332807069
H	2.33085379489287	-2.71444443818755	1.57488708119239
H	2.87837639708155	-2.45646868480307	-0.09699287208750
H	3.10767562314050	-1.18471681714723	1.12408820696629
C	-0.42168448927857	0.76492114790407	-1.47629680353644
O	0.36193024385014	1.58759145257887	-1.00640710930061
C	-0.70063037784594	0.68222060115606	-2.95261908235825
H	-1.50372752300152	-0.01529537206346	-3.18965077562831
H	-0.95520963657687	1.67843418112133	-3.31686736565146
H	0.21462894905720	0.36110196236731	-3.45535233161228
O	1.05992805644463	2.43789910102687	1.66691022906508
H	-3.23830236877060	0.97941822632574	-0.11297245656735
H	0.78501438242855	2.17177799339875	0.77298554263750

Figure P9.2: Structures and relative Gibbs energies of reactants, products and transition state for the conversion of cysteine thioaldehyde **CT+H_2O** into O-protonated *E*-enethiolate (**OP-*E*-ETe**) via transition-state structure **CT/H_2O---OP-*E*-ETe**, as mediated by one explicit water molecule. O-to-S proton transfer gives the *E*-enethiol (***E*-ET** + **H_2O**).

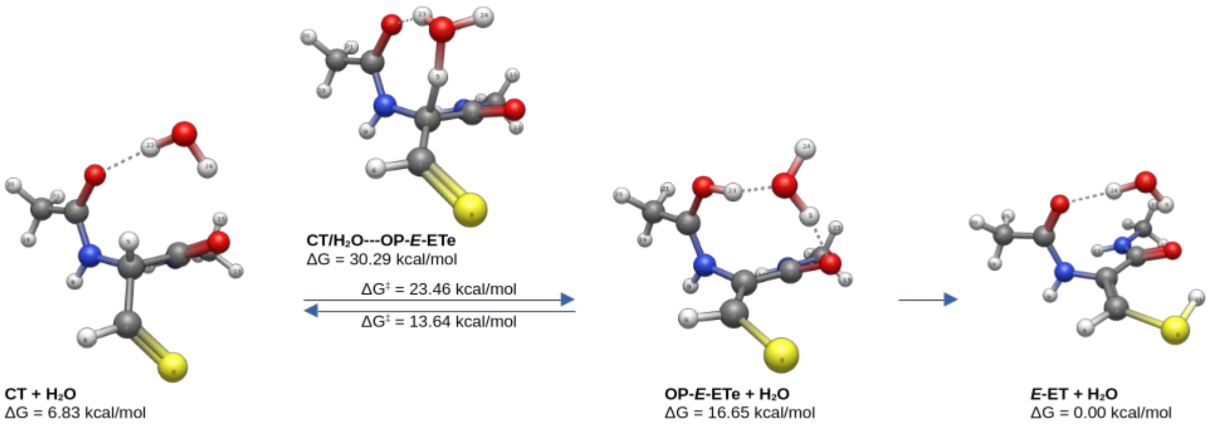

Table P9.2: Cartesian coordinates, Gibbs energies (Hartree), relative Gibbs energies (kcal/mol) and imaginary frequencies, where relevant, of reactant cysteine thioaldehyde and water, **CT+H_2O**, intermediate O-protonated enethiolate and water (**OP-*E*-ETe + H_2O**), and transition state **CT/H_2O---OP-*E*-ETe**. Data is also provided for product enethiol and water (***E*-ET+H_2O**). All species calculated at the following level: (wB97X-D4/def2-TZVP/def2/J, CPCM: water).

Reactant **CT + H_2O**:<br>Gibbs Energy: -969.57491721 Hartree<br>Relative Gibbs Energy = 6.83 kcal/mol<br>$\Delta G^{\ddagger}$: 23.46 kcal/mol				Transition state **CT---OP-*E*-ETe**:<br>Imaginary frequency: −926.43 cm^{-1}<br>Gibbs Energy: -969.53753844 Hartree<br>Relative Gibbs Energy = 30.29 kcal/mol			
C	-0.92021034245257	0.12272462142891	0.77316171048885	C	-0.77520770575413	-0.01378177842303	0.83282927130613
C	0.49167993811067	-0.31618183711718	1.17636734962836	C	0.54899098311768	-0.60850598067024	1.21684872830952
N	-1.20680224849415	-0.01644713258624	-0.63882836183473	N	-1.08750979504733	-0.02052509271459	-0.57829438962334
C	-1.99413456855329	-0.58248347650049	1.56492866898164	C	-1.89561782769571	-0.06711277969705	1.68645974301922
H	-0.99364976018224	1.18302598605016	1.04045683456455	H	-0.48512592374161	1.38627342384525	1.21966615668265
H	-3.00173496871927	-0.29157555224145	1.26378901497510	H	-2.81522365373489	0.21270282667913	1.16782819826891
O	1.05686891006876	0.23281434253915	2.12392001906792	O	1.08804257808201	-0.33467221543638	2.29021963181320
S	-1.76476111093386	-1.65068531928188	2.73483335208002	S	-2.02178964841226	-0.42024773705904	3.30054220780989
H	-1.79867969730244	-0.76763418889815	-0.95749927164850	H	-1.72024862003947	-0.72741924924965	-0.92338227645768
C	2.35374033790945	-1.83053335638081	0.76830826956532	C	2.43974202417629	-1.99646916810965	0.53626408558691
N	1.03344572429820	-1.30299005539493	0.46713069030720	N	1.12939123701394	-1.41434167821729	0.31032831719065
H	0.51146285611364	-1.69877921569961	-0.29770391239319	H	0.63596494000374	-1.64397295204991	-0.53521699079206
H	2.37649367397617	-2.26392371968381	1.77070896694120	H	2.42774845949528	-2.68112849782789	1.38818034230110
H	2.59023629300276	-2.60180772518236	0.03749027301303	H	2.73023871764782	-2.54623361549090	-0.35790857205686
H	3.10340287236868	-1.03860711790224	0.71226174316142	H	3.17806830899131	-1.21526972348876	0.73093395315424
C	-0.67273032292064	0.83313072039317	-1.54585886024697	C	-0.54564160586155	0.80437732911866	-1.49044683943642
O	0.07405127953329	1.75011458955862	-1.20676629299915	O	0.24099876437880	1.72200495273720	-1.19893286353958
C	-1.03510805586927	0.59355911932316	-2.98706057762644	C	-0.91726371003969	0.55987178665717	-2.92654390235663
H	-1.68724302175791	-0.26922457757551	-3.12050232029584	H	-1.64834969396066	-0.23933839384522	-3.04363926474659
H	-1.53003360609727	1.48776519083286	-3.37100602999386	H	-1.31177491445220	1.48683754569875	-3.34604229220404
H	-0.11414366926743	0.44695739560192	-3.55438375412215	H	-0.00812624451820	0.30192645196378	-3.47419758990524
O	1.13395266206542	3.00099995956332	1.17363090656307	O	-0.09175996922086	2.48494555463795	1.19461775929944
H	0.77981285632085	2.66459314557087	0.33476238914160	H	0.21816549745363	2.40670524036790	0.23107689255233
H	1.19242823270846	2.20353831574324	1.72053316963813	H	0.68628780211811	2.56337375057382	1.76880969382425

Intermediate **OP-*E*-ETe + H_2O**:<br>Gibbs Energy: -969.55925392 Hartree<br>Relative Gibbs Energy = 16.65 kcal/mol<br>$\Delta G^{\ddagger}$: 13.64 kcal/mol				Product ***E*-ET+H_2O**<br>Gibbs Energy: -969.5858021 Hartree<br>Relative Gibbs Energy = 0.00 kcal/mol			
C	-0.91424981117754	-0.32728740414696	0.69783188335979	C	-0.95937020226574	-0.13041087062503	0.59659121470499
C	0.44068362247566	-0.71105699124731	1.15100723511518	C	0.36792025438345	-0.58202414962416	1.10641632296560
N	-1.11300224506867	-0.28271027956471	-0.73103905644388	N	-1.16203532173250	-0.18903770498899	-0.81490494019864
C	-1.98383570319042	-0.02036690501274	1.48008069402478	C	-1.96543622610033	0.30429743461554	1.36440673023591
H	0.51260553938530	1.49829233731968	1.94376473053353	H	0.85379025479814	1.82915568808658	1.93181746282686
H	-2.87042411487112	0.23853314347033	0.89856087470020	H	-2.88182870124514	0.59694842244917	0.86033419883474
O	0.94029848885053	-0.25154001739131	2.19090578956029	O	0.77187380578437	-0.28381917265145	2.24098869913865
S	-2.17624340115895	-0.00949103022593	3.16907071060541	S	-2.09395403926557	0.46716054209559	3.08377317392744
H	-1.53810259022505	-1.06912273093598	-1.20643730890155	H	-1.82211547337291	-0.85238836996903	-1.19048166146055
C	2.49677953926072	-1.94136938033535	0.67421410885296	C	2.42218607487144	-1.80267635520150	0.63058147370071
N	1.13415021833526	-1.54813588790936	0.36129336414972	N	1.10236638248364	-1.31845749500640	0.26745808481704
H	0.68753691606536	-1.96273477314744	-0.43928065921936	H	0.72420910270748	-1.55428612645907	-0.63487511908823
H	2.53722598485338	-2.53857964380592	1.58880083401935	H	2.36833367206355	-2.47518697642636	1.48985230666641
H	2.88194672513458	-2.53267167013989	-0.15520149470143	H	2.83528697465954	-2.34104153546459	-0.22071695551647
H	3.12583181377894	-1.05917137868761	0.80844205016245	H	3.08008156982253	-0.96805111611526	0.88229702504915
C	-0.79982643717168	0.74864724771862	-1.47221150938837	C	-0.57601946537814	0.70321876612786	-1.64856808799113
O	-0.27981766687949	1.82376909144214	-0.99577107105502	O	0.17516967545801	1.58392544792165	-1.23004035937183
C	-1.02958634810345	0.72604195663974	-2.94139165508456	C	-0.87030158265160	0.54390787267579	-3.11635315252932
H	-1.51560358633545	-0.19518920253862	-3.25620063706646	H	-1.61941360147679	-0.22227036165580	-3.31499293103649
H	-1.64334362577228	1.58713744864442	-3.21134182995607	H	-1.20993136407155	1.50316797198891	-3.51000777508291
H	-0.06359827064307	0.82684787738413	-3.44090487330207	H	0.05946582584686	0.27952514028645	-3.62508601583054
O	0.29771587936512	2.32106573847391	1.45105079840011	O	0.84093221704462	2.61563512387934	1.37017680796871
H	-0.10163779606305	1.89138353822148	0.01412080518564	H	-0.83287662770173	0.11280655161196	3.37386904547248
H	1.09950052593015	2.85750807679122	1.46544883137036	H	0.57774793153838	2.27287679674880	0.49845096179742

Figure P9.3: Structures and relative Gibbs energies of reactants, products and transition state for the conversion of cysteine thioaldehyde **CT+2H_2O** into O-protonated *E*-enethiolate (**OP-*E*-ETe**) via transition-state structure **CT/2H_2O---OP-*E*-ETe**, as mediated by two explicit water molecules. O-to-S proton transfer gives the *E*-enethiol (***E*-ET** + **2H_2O**).

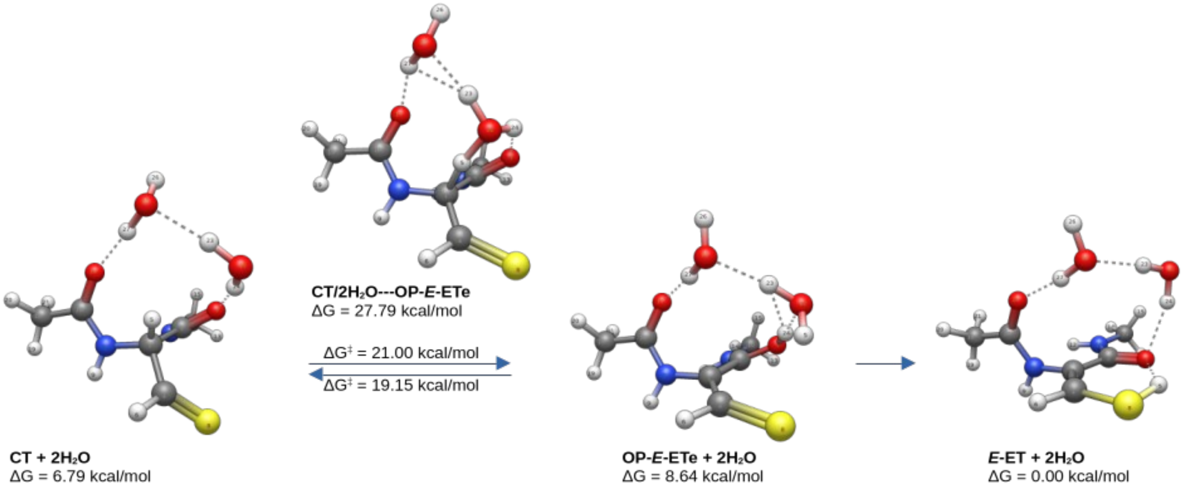

Table P9.3: Cartesian coordinates, Gibbs energies (Hartree), relative Gibbs energies (kcal/mol) and imaginary frequencies, where relevant, of reactant cysteine thioaldehyde and two water molecules, **CT+2H_2O**, intermediate O-protonated enethiolate and two water molecules (**OP-*E*-ETe + 2H_2O**), and transition state **CT/2H_2O---OP-*E*-ETe**. Data is also provided for product enethiol and two water molecules (***E*-ET+2H_2O**). All species calculated at the following level: (wB97X-D4/def2-TZVP/def2/J, CPCM: water).

Reactant **CT + 2H_2O**:
Gibbs Energy: -1046.04797432 Hartree
Relative Gibbs Energy = 6.79 kcal/mol
$\Delta G^{\ddagger}$: 21.00 kcal/mol

C	-0.85552771692382	-0.02860230309758	0.62487775925689
C	0.48559593187529	-0.65586710572006	1.01739668216367
N	-1.29916494388193	-0.34326932403761	-0.71591594694206
C	-1.96194473848142	-0.36853479698540	1.59384453120013
H	-0.71406121668935	1.05465679627358	0.69297945618600
H	-2.91149659703534	0.09807212119542	1.32800440615390
O	1.17851846505657	-0.12513390907425	1.88830204778714
S	-1.84219373914737	-1.30897646224179	2.88352473845602
H	-1.98791445486692	-1.06669972189464	-0.85406301059290
C	2.06493863602034	-2.48099755283821	0.70463671493101
N	0.83080843985580	-1.77868145269615	0.39356313512679
H	0.22139402730314	-2.15260001141740	-0.31532988891642
H	2.07487227703509	-2.79513013568023	1.75057765393971
H	2.13123921348399	-3.35885374346763	0.06446585023547
H	2.92827186032120	-1.83716195715084	0.52405641439615
C	-0.77139542748151	0.27725327950589	-1.79520210714421
O	0.09896375169509	1.13971512184199	-1.68209859750421
C	-1.28979153173448	-0.15641081053387	-3.13965847424446
H	-2.12070071421480	-0.85755815273128	-3.06465434966668
H	-1.60389842654691	0.73021970178042	-3.69262651088059
H	-0.46921008722374	-0.62437765191326	-3.68797647439095
O	0.60330940051828	2.57854203663938	2.55417898189603
H	0.75143966988206	2.93449627384052	1.66036020401762
H	0.79036222503638	1.62921393012242	2.45129453696753
O	0.99811643924869	3.35858054349721	-0.17471488001946
H	1.93614610445906	3.44331724873051	-0.37821308382454
H	0.69931262808527	2.57736149553188	-0.67367580614515

Transition state **CT---OP-*E*-ETe**:
Imaginary frequency: −1077.81 cm^{-1}
Gibbs Energy: -1046.01450459 Hartree
Relative Gibbs Energy = 27.79 kcal/mol

C	-0.86845579097132	-0.22538468630779	0.79266945938776
C	0.47235923806549	-0.84263045554386	1.12079992437964
N	-1.27130974491758	-0.30765310212549	-0.58661926233342
C	-1.92052735170485	-0.30992781531807	1.72958642775705
H	-0.55113039638233	1.12857383333911	1.19711617459376
H	-2.89156379182479	-0.05100456054182	1.30153329307618
O	1.22484222023258	-0.34197115904690	1.96608834191623
S	-1.86150437024832	-0.66834346625492	3.34439461375306
H	-2.07758603325896	-0.86281862016203	-0.82931223850357
C	2.08600083843907	-2.60097530348781	0.60623077674626
N	0.80131131582962	-1.94392072196746	0.43839441768921
H	0.14583974158869	-2.31713292735570	-0.22828175871961
H	2.18972888303160	-3.00753984718908	1.61498245778852
H	2.15293141024720	-3.41400704877983	-0.11503009223709
H	2.90164787578839	-1.89649252860080	0.42910099174610
C	-0.57679437028645	0.27749191542820	-1.57957185110742
O	0.45185907474169	0.92422614701965	-1.35498526522238
C	-1.09712390091979	0.08109962638718	-2.97863301278432
H	-2.04654718721596	-0.45344022737542	-3.00552121139767
H	-1.21365261040460	1.06084897741126	-3.44522673063105
H	-0.35121008462736	-0.47703349226287	-3.54882894056495
O	-0.00545288897923	2.07821683487502	1.71978523197470
H	0.35737931119953	2.66652536074036	0.98823946334297
H	0.74387274769844	1.57473091424929	2.09986309555200
O	0.76670530047968	3.45756433287076	-0.36372435463945
H	1.66793320704034	3.80084273779257	-0.37157943558088
H	0.77044735735923	2.67015528220646	-0.94147051598162

Intermediate **OP-*E*-ETe + 2H_2O**:
Gibbs Energy: -1046.04501992 Hartree
Relative Gibbs Energy = 8.84 kcal/mol
$\Delta G^{\ddagger}$: 19.15 kcal/mol

C	-0.94023369717526	-0.25063649167700	0.55812426935414
C	0.34796421373494	-0.81002206968529	0.93430418238887
N	-1.31443214006888	-0.47273978008444	-0.80349463334349
C	-1.86261601585878	0.28728954794275	1.40666848186038
H	-0.14378311142237	2.02472923311876	2.90433884772745
H	-2.77281152738610	0.59033574990539	0.88788738112475
O	1.07587588317923	-0.36030840232328	1.90475519203567
S	-1.87610502584840	0.47862928245927	3.09857002561553
H	-2.10946131312026	-1.06127253647861	-1.00017831781156
C	2.15297948829591	-2.40420971528524	0.52368375252148
N	0.83680306849265	-1.83222530962326	0.26954455425595
H	0.25719347514744	-2.24614081218368	-0.44433685266194
H	2.17934560622853	-2.89503900389360	1.49812411009059
H	2.35580074332624	-3.13501146894783	-0.25618748692287
H	2.91112960391666	-1.62088775560282	0.49713780618866
C	-0.67012981731344	0.10434330550956	-1.84084129054338
O	0.28857334544180	0.86251037055737	-1.67357482325253
C	-1.15698068778734	-0.25452828409028	-3.21881583328658
H	-2.07652862379371	-0.83934511430647	-3.20239173312037

Product ***E*-ET+2H_2O**
Gibbs Energy: -1046.05879046 Hartree
Relative Gibbs Energy = 0.00 kcal/mol

C	-1.13898117088145	-0.27926885418334	0.46376052743139
C	0.10613856609973	-0.79302000610466	1.09541455436902
N	-1.36859577515976	-0.62334358584615	-0.90487542794062
C	-2.05289894118746	0.48731271352462	1.07494121096167
H	-0.97386843978715	0.55909839333966	3.10872845665149
H	-2.90825492991363	0.79652811846455	0.48152999728546
O	0.39007108156068	-0.56204742083114	2.28339538097743
S	-2.12642910032588	1.10507259852100	2.68693866728123
H	-1.89599397823890	-1.45849285589931	-1.11093360116846
C	2.16368359073261	-2.06598198165428	0.81376894018222
N	0.91550721841834	-1.51551690164438	0.31454776378030
H	0.64057988456559	-1.70938577310528	-0.63363558316414
H	1.98066544585748	-2.75480984697256	1.64164135268973
H	2.64987785824827	-2.60210963829976	0.00057653628810
H	2.82196719179494	-1.26780046209631	1.16331099125164
C	-0.96383587891091	0.15708001308703	-1.93343894976694
O	-0.33803172353585	1.20277956581731	-1.75900319710556
C	-1.30786050055861	-0.32933624826077	-3.31629762347851
H	-1.89070500729773	-1.25005052665935	-3.30744307039757

H	-1.31351209248999	0.66535344643164	-3.78402965181070	H	-1.86890767099870	0.45437662771190	-3.82841935405171
H	-0.37431880135242	-0.82979253222393	-3.71900719643609	H	-0.37800317231383	-0.49249169228465	-3.86488844539607
O	0.75366498875534	2.06796281913502	2.52670480678086	O	2.24317974372789	1.67411802918214	2.16520293115432
H	0.65758852589397	2.52365481272385	1.65635141684181	H	1.66824107204160	2.12721492230151	1.52089022471496
H	0.86268924922925	0.61000194296658	2.18233783943897	H	1.75734623474862	0.86136279574873	2.36764928564694
O	0.43247319780324	3.05360912366302	0.02290663734418	O	0.46054476471026	2.87824554117267	0.31523245314852
H	1.13402218947750	3.60421631618556	-0.34194760062167	H	0.85299922918479	3.59482013304134	-0.19529753856975
H	0.39881979750434	2.25694986895399	-0.54456786659071	H	0.15027149131906	2.23291065832947	-0.35037878467506

Figure P9.4: Structures and relative Gibbs energies of reactants, products and transition state for the conversion of cysteine thioaldehyde and acetate **CT+AcO**$^-$ into *Z*-enethiolate and acetic acid (***Z*-ETe + AcOH**) via transition-state structure **CT/AcO**$^-$**---*Z*-ETe**.

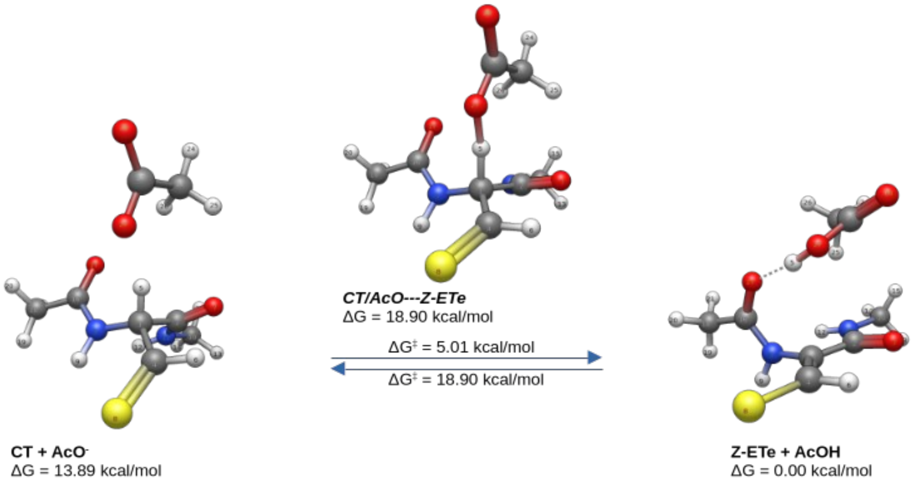

Table P9.4: Cartesian coordinates, Gibbs energies (Hartree), relative Gibbs energies (kcal/mol) and imaginary frequencies, where relevant, of reactant cysteine thioaldehyde and acetate, **CT+AcO**$^-$, product Z-enethiolate and acetic acid (**Z-ETe + AcOH**), and transition state **CT/AcO**$^-$**---*Z*-ETe**. All species calculated at the following level: (wB97X-D4/def2-TZVP/def2/J, CPCM: water).

Reactant **CT + AcO**$^-$:<br>Gibbs Energy: -1121.85743682 Hartree<br>Relative Gibbs Energy = 13.89 kcal/mol<br>$\Delta G^{‡}$: 5.01 kcal/mol				Transition state **CT/AcO**$^-$**---*Z*-ETe**:<br>Imaginary frequency: −1336.23 cm^{-1}<br>Gibbs Energy: -1121.84944404 Hartree<br>Relative Gibbs Energy = 18.90 kcal/mol			
C	-0.78958920981817	-0.28221039216065	0.58369682622227	C	-0.98139688111807	-0.27929934307909	0.61001607815277
C	0.49655074182320	-0.96819455945378	1.08242774577596	C	0.29420144017719	-0.81242839339065	1.22073563628684
N	-1.22666962319319	-0.68920797622038	-0.72212650312812	N	-1.30258302621153	-0.69755188087931	-0.72666536244454
C	-1.83691364260148	-0.47660956520857	1.63433151592353	C	-2.08966393497872	-0.33605783179662	1.52407913300277
H	-0.57436837004291	0.79954435288181	0.59134376719893	H	-0.82627824820146	1.01803130406597	0.63997122745211
H	-1.48449834206387	-0.22034564259482	2.63250427144366	H	-1.79254143338716	-0.24641078534892	2.56717919380421
O	1.09730027227966	-0.50286946673794	2.04746908136026	O	0.61517858365897	-0.50512461866149	2.37159457962655
S	-3.34051549645319	-0.99281980291344	1.39928827865590	S	-3.67698157069948	-0.45667347370411	1.13957375001540
H	-2.10540533330179	-1.18216316177285	-0.79015257503200	H	-2.13740255184972	-1.25772795275749	-0.82544989331368
C	2.06268321602646	-2.81252351964289	0.80530777544016	C	2.32351082454154	-2.12892214799117	0.90900380297642
N	0.86985776694802	-2.07163144570350	0.43336490578619	N	1.05326350779187	-1.59994771269076	0.44766750706875
H	0.31258996800287	-2.38846314914504	-0.34288844317008	H	0.71994055235378	-1.85050500898150	-0.46699091833237
H	1.98741112195956	-3.17614479162851	1.83258435954903	H	2.18765160121135	-2.78047049815006	1.77585882447497
H	2.16460802050748	-3.66120871730513	0.13108932497498	H	2.77131237667088	-2.70292180788175	0.09898509677373

H	2.95003969750906	-2.18072881732709	0.72313240572158	H	3.00022322575463	-1.31795591101027	1.18819603917381
C	-0.61277288191852	-0.24605199788652	-1.84515422152330	C	-0.74945808378472	-0.19447495088811	-1.85450093500436
O	0.40879144698021	0.43335406401766	-1.79823863735816	O	0.21152126247160	0.56992841855817	-1.84185837154913
C	-1.24459535831288	-0.65561023575189	-3.15215271080910	C	-1.36936181919109	-0.66467727834007	-3.15005112728513
H	-2.13697694308259	-1.26707164025065	-3.01776381148397	H	-2.21978039806619	-1.33020005830059	-3.00005928995629
H	-1.50323499574531	0.24748442422638	-3.70857215697749	H	-1.69117198868045	0.21256544841361	-3.71484607187225
H	-0.50732114058571	-1.21104943250671	-3.73503899976026	H	-0.60538079057439	-1.17981914189510	-3.73579419452634
C	1.31074239295708	3.10864485885143	0.90548673764884	C	1.48575062225559	2.34366625704158	0.72195587406951
C	-0.06089538381804	3.67638235136381	0.52579780723050	C	0.15775376753947	3.07406211548587	0.63438463620188
H	2.08827824864656	3.87249566469122	0.86806187317881	H	2.31464243224094	3.03837427996941	0.59046025897423
H	1.26549635679494	2.68584508222408	1.91251860142406	H	1.56543224555777	1.85885852711299	1.69742432977644
H	1.56988803657663	2.29342610411026	0.22483334645127	H	1.52206916727685	1.56253750834864	-0.03892943289196
O	-1.02980891995406	2.86810160955769	0.55483662748921	O	-0.91013576315873	2.35383021386593	0.66526527833146
O	-0.13281399276809	4.89052048364874	0.21494230251898	O	0.12968488039934	4.30931472288487	0.55279435101418

Product **Z-ETe + AcOH**:
Gibbs Energy: -1121.87956883 Hartree
Relative Gibbs Energy = 0.00 kcal/mol
$\Delta G^{\ddagger}$: 18.90 kcal/mol

C	-1.27482993339295	-0.81307494889600	0.68865669783996
C	0.02092967712978	-0.94343771509114	1.38507898674165
N	-1.32599860910086	-1.13129943049138	-0.70229994757741
C	-2.38118998576696	-0.39640376326067	1.34615605890975
H	-0.65520773079203	1.79525122089695	-0.02306662818739
H	-2.22314768197824	-0.19053563725959	2.40257230951331
O	0.18430798648043	-0.61876455756429	2.56997743934927
S	-3.94989359839951	-0.15834248162602	0.70943528369762
H	-1.67819582623154	-2.03608982288445	-0.97787710865132
C	2.36805746446825	-1.61644914198796	1.21133634054783
N	1.04409601501234	-1.43400976104985	0.65145066043882
H	0.87145193265401	-1.71079718749810	-0.29959064156694
H	2.35383112222086	-2.33008708074475	2.03959473901314
H	3.02405258212701	-1.99533758121318	0.42845721347030
H	2.76921066329628	-0.66952441254264	1.58206694064108
C	-1.19505428590970	-0.20551529278460	-1.66589722157245
O	-0.87791494923633	0.96897364784911	-1.43167873072917
C	-1.43850639310285	-0.66042863315671	-3.07979265943316
H	-1.64762532989973	-1.72783669872778	-3.14727402851622
H	-2.28576912684847	-0.09889035472162	-3.47918936061954
H	-0.56056865410937	-0.41949044188117	-3.68170865493568
C	1.74683885972031	2.11050495723557	0.10267439640372
C	0.71234099236997	2.68402280099366	1.03646263777459
H	2.74369464210720	2.33550575486251	0.47599568184178
H	1.61452084815267	1.03211264117927	0.00495942735318
H	1.61579892463789	2.54959198810898	-0.88942232366613
O	-0.55720105895136	2.40970107541334	0.75723353845568
O	0.98411379669350	3.37975617430887	1.99475945683745

Figure P9.5: Structures and relative Gibbs energies of reactants, products and transition state for the conversion of cysteine thioaldehyde and acetate **CT+AcO**$^{-}$ into *E*-enethiolate and acetic acid (***E*-ETe + AcOH**) via transition-state structure **CT/AcO**$^{-}$**---*E*-ETe**.

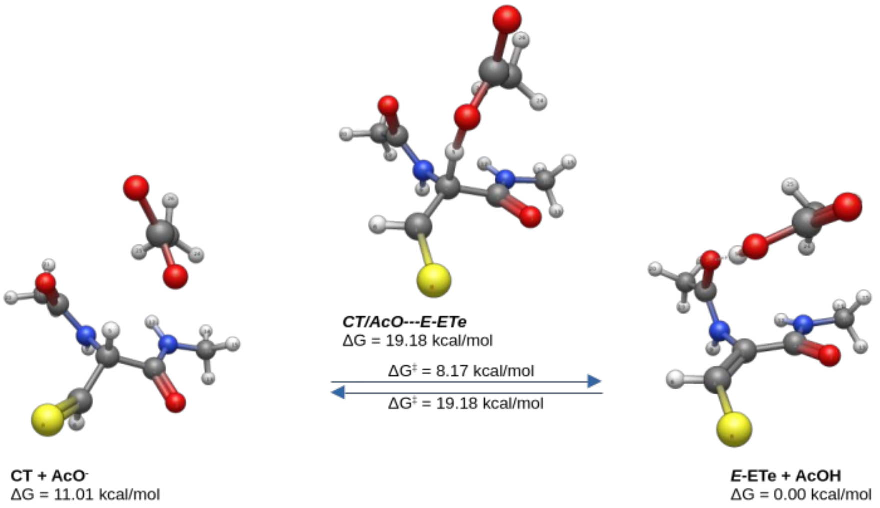

Table P9.5: Cartesian coordinates, Gibbs energies (Hartree), relative Gibbs energies (kcal/mol) and imaginary frequencies, where relevant, of reactant cysteine thioaldehyde and acetate, **CT+AcO**$^{-}$, product *E*-enethiolate and acetic acid (***E*-ETe + AcOH**), and transition state **CT/AcO**$^{-}$**---*E*-ETe**. All species calculated at the following level: (wB97X-D4/def2-TZVP/def2/J, CPCM: water).

Reactant **CT + AcO**$^{-}$:<br>Gibbs Energy: -1121.85217696 Hartree<br>Relative Gibbs Energy = 11.01 kcal/mol<br>$\Delta G^{\ddagger}$: 8.17 kcal/mol				Transition state **CT/AcO**$^{-}$**---*E*-ETe**:<br>Imaginary frequency: −1452.67 cm^{-1}<br>Gibbs Energy: -1121.83916204 Hartree<br>Relative Gibbs Energy = 19.18 kcal/mol			
C	-1.14705255423375	-0.38497532796022	0.68866321299159	C	-1.03374085309598	-0.27913539696080	0.65144320983890
C	-0.03390373481310	-1.04989733059805	1.49991697059231	C	0.15270887536449	-0.81569227536300	1.41258129828850
N	-1.10250929529766	-0.71607282206897	-0.71899876419753	N	-1.08033211493365	-0.73316982834854	-0.72515901862894
C	-2.48963145326478	-0.76314630766251	1.26602237035904	C	-2.34608646350318	-0.29873537098989	1.27011434373426
H	-0.98870893169466	0.68998864474158	0.79328000424832	H	-0.77014175650539	1.01051549627596	0.76388598240923
H	-2.73831048234865	-1.82247996590056	1.18181102772486	H	-3.13568182516501	-0.38186840688937	0.52036680152744
O	-0.20367552019508	-1.27880281852022	2.69595162789352	O	0.28901558439872	-0.65999545963525	2.62492568992399
S	-3.52984403578470	0.27366381642833	1.90911175167407	S	-2.81879019592424	-0.11888087953244	2.82317869792259
H	-1.01134294171580	-1.68481344295515	-0.98464845140868	H	-1.17951676674384	-1.72572878872447	-0.88090263021305
C	2.27785683217430	-1.81778434592627	1.53133848885728	C	2.35012463884456	-1.86145967028238	1.25928997514607
N	1.10115487816646	-1.30993786092692	0.84744320298236	N	1.08888400739536	-1.44809636737106	0.67442546295217
H	1.17541205454921	-1.04312980767260	-0.12048946840451	H	0.99478985546182	-1.45465612713448	-0.32643640607688
H	2.05016475633879	-2.75860791722107	2.03591858039738	H	2.18434357983792	-2.56486388576006	2.07766328229614
H	3.05798045476707	-1.98890566287183	0.79136919310705	H	2.94328919971201	-2.35027042973241	0.48735545917877
H	2.63745455426433	-1.10069804666180	2.27390743102875	H	2.90706640710832	-1.00396471329473	1.64942674802488
C	-1.44181013366668	0.18632175935404	-1.67898430196997	C	-1.26770535498570	0.06658738592875	-1.80320578055341
O	-1.72371399511778	1.34847833348219	-1.41370546379974	O	-1.32385613433411	1.28962343554314	-1.73204140006294
C	-1.43143582887122	-0.33231641999372	-3.09534003252514	C	-1.40103631481667	-0.65025321780624	-3.12769076576154
H	-1.11490929301850	-1.37310002721030	-3.16372680930941	H	-1.27101852100374	-1.72972727790903	-3.04678249746959
H	-2.43714315920243	-0.23056898565782	-3.50826312230713	H	-2.39032088620709	-0.43628366884596	-3.53786000574992
H	-0.75907086106869	0.29253671998152	-3.68607843773320	H	-0.65689339451638	-0.24543708704179	-3.81599706724856
C	1.84300511260475	2.05406838953034	-0.23557052096006	C	1.57655377444169	1.97337021037096	0.10330507997544
C	0.84805724249127	2.86404493175797	0.60283163983754	C	0.51601896781211	2.84520380072874	0.75511681114223
H	2.47560792105525	1.42807724311267	0.39610067029390	H	2.02378564211407	1.32805455457377	0.86415640290486
H	1.27571997806462	1.39411101446986	-0.89980282291141	H	1.13117456922544	1.33374969980725	-0.65947565037159
H	2.46044476875060	2.70655370817642	-0.85529776643176	H	2.35731679644781	2.59082858386651	-0.33923136586454

O	0.44374460507221	2.34202119066295	1.67574000837275	O	-0.56393608663443	2.26486262424093	1.14387702310019
O	0.48192374282472	3.97659935566261	0.14568723990969	O	0.72398477020508	4.05542306028588	0.91367031963531

Product ***E*-ETe + AcOH**:
Gibbs Energy: -1121.86972133 Hartree
Relative Gibbs Energy = 0.00 kcal/mol
ΔG‡: 19.18 kcal/mol

C	-1.31870553831690	-0.76749829385231	0.75070722297509
C	0.01915285096481	-0.84857899147317	1.37285324963135
N	-1.39040073710351	-1.12863524868497	-0.64158767404849
C	-2.48727306542748	-0.41002141072743	1.34668658358986
H	-0.67188554725263	1.82505031868691	-0.08378664690565
H	-3.32593684739076	-0.43874797909502	0.64885220013480
O	0.27969669222179	-0.43890460774938	2.50893003682427
S	-2.90020313412956	0.05350827903967	2.93928497491305
H	-1.65841758586677	-2.06872228915594	-0.89184180272576
C	2.34027858013596	-1.57840861010516	1.11174261257894
N	0.99402409087390	-1.40172885525934	0.60674162976742
H	0.75839816428102	-1.78832941932036	-0.29003927971362
H	2.36017159204486	-2.25194566350384	1.97361089887989
H	2.95352771364622	-2.00171466345026	0.31682303900946
H	2.76983394718274	-0.62143008324457	1.41824420322193
C	-1.17693175920352	-0.25912230012411	-1.64160126630633
O	-0.87032318010394	0.92617232992280	-1.44937214750312
C	-1.31244192951586	-0.79029918216516	-3.04378244158403
H	-1.63521807230649	-1.83083479672233	-3.06987958684316
H	-2.03017803478453	-0.16989797352631	-3.58329108043649
H	-0.34511871119693	-0.69797010690108	-3.54224244218208
C	1.72882967506149	2.13585643098685	0.09365257667626
C	0.67248671448125	2.73963458636311	0.98272886353197
H	1.56733072985582	1.06370929407701	-0.02295277195064
H	1.65799212870194	2.58995894222452	-0.89818369764780
H	2.71446702959305	2.32653157006303	0.51311456316271
O	-0.59102398833214	2.47096135434454	0.67195883399852
O	0.92240354075149	3.46017935054151	1.92844189050088

P10 Phosphonium Adduct of Cysteine Thioaldehyde

Figure P10.1: Structures and relative Gibbs energies of reactants, products and transition state for the conversion of cysteine thioaldehyde **CT** and trimethylphosphine (**PMe3**) into phosphonium adduct **PhosThiolate** via transition-state structure **CT/PMe3---PhosThiolate**.

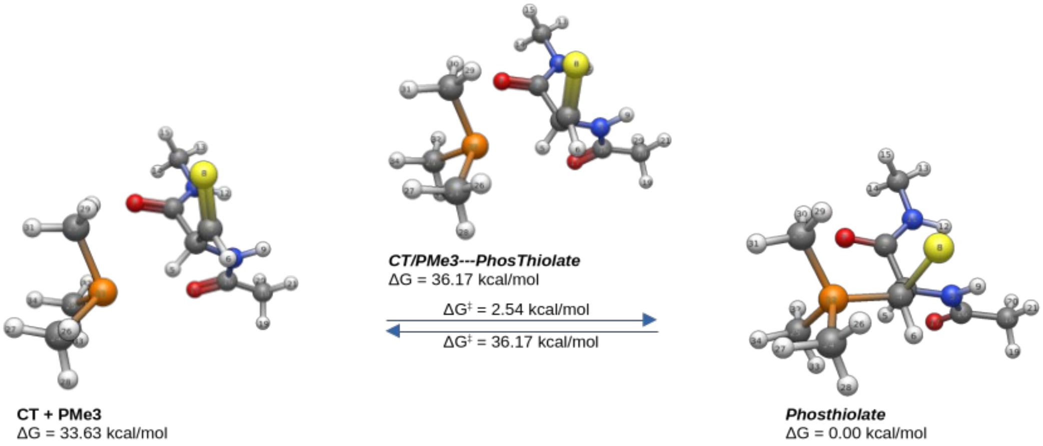

Table P10.1: Cartesian coordinates, Gibbs energies (Hartree), relative Gibbs energies (kcal/mol) and imaginary frequency where relevant of reactant molecules, cysteine thioaldehyde **CT** and trimethylphosphine (**PMe3)**, product phosphonium adduct **PhosThiolate** and transition-state structure **CT/PMe3---PhosThiolate**. All species calculated at the following level: (wB97X-D4 /def2-TZVP/def2/J, CPCM: water).

Reactant **CT + PMe3**:
Gibbs Energy: -1354.25994195 Hartree
Relative Gibbs Energy = 33.63 kcal/mol
$\Delta G^{\ddagger}$: 2.54 kcal/mol

C	0.27550958534589	-0.42404857458564	0.89020194916773
C	0.80061121291770	0.99152609746006	1.14668016172753
N	1.02332925738125	-1.47446378491746	1.54709538363286
C	-1.18165699421510	-0.58026259112524	1.24727478215538
H	0.34995954820793	-0.57499979179421	-0.19129454227231
H	-1.57226783575414	-1.56823307663323	1.00442212383128
O	0.59559502964659	1.87645502843792	0.31921218268370
S	-2.11398552526080	0.52065262143512	1.94394944851619
H	0.65375159849898	-1.90849208487351	2.37814643453001
C	1.97663376330696	2.49213248069915	2.66273914001838
N	1.45590830902041	1.18771274396876	2.29302514086958
H	1.59629220989795	0.40880026938434	2.91498581400750
H	2.46295046108396	2.40687251599317	3.63307195642242
H	2.70439770292144	2.84051719467155	1.92622321469749
H	1.16928843946428	3.22518449518760	2.72797370636183
C	2.23368706389522	-1.86128787875087	1.07156564130516
O	2.72710198933865	-1.33315593863967	0.08033651105947
C	2.93028897764550	-2.95860221745821	1.83458774229073
H	3.15795346887848	-3.77141315053312	1.14256641894818
H	3.87454987861643	-2.56615032377972	2.21765655846276
H	2.33425832283559	-3.34081615667986	2.66319463807404
P	-1.97808557912361	-0.12237092225527	-2.13127880367720
C	-2.60253244066771	1.50625966530233	-1.55962723606298
C	-3.16810818678530	-0.46518308997184	-3.48901978417468
C	-0.52026569860256	0.41901059506640	-3.10985296119919
H	-4.15679060373550	-0.66235260029947	-3.06721185253405
H	-3.23718996065276	0.37867925256027	-4.18181515292370
H	-2.84967957423456	-1.35395089532774	-4.03936395825823
H	-3.55723511908739	1.37269424368177	-1.04461042045454
H	-1.89059097372936	1.93901118191128	-0.85362100256906
H	-2.74089625060269	2.19394053374710	-2.39914401829142
H	0.24986194568222	0.80025218818435	-2.43508613640167
H	-0.10716261928260	-0.43467561592638	-3.65282606926438
H	-0.78829092687530	1.20204097222560	-3.82538735572612

Transition state **CT/PMe3---PhosThiolate**:
Imaginary frequency: −92.32 cm^{-1}
Gibbs Energy: -1354.2558935 Hartree
Relative Gibbs Energy = 36.17 kcal/mol

C	0.40513296277806	-0.57742822720346	0.61043555181066
C	0.79831443622308	0.88185507056984	0.86589334098355
N	1.01336488895232	-1.53450980989107	1.51479454070979
C	-1.09340985355471	-0.79193979374040	0.68414702811102
H	0.74383141782730	-0.81033748816412	-0.39846410462724
H	-1.42045808633041	-1.71216184322696	0.20912144336304
O	0.54161674924786	1.73872995611123	0.02271961555575
S	-2.07996740172539	0.03605160151617	1.66914518320761
H	0.51202461539783	-1.81411383007728	2.34355281459705
C	1.83823940398357	2.49482185309262	2.36495667656950
N	1.42495234929803	1.14763535426899	2.01482349243486
H	1.60290152799839	0.39569257728242	2.65916928513096
H	2.33219440311554	2.46308492798236	3.33478593556262
H	2.53360562081568	2.89057286513321	1.62121243138634
H	0.97481901062645	3.16199064275766	2.42303924067186
C	2.23285500967482	-2.07475401335513	1.26309890626562
O	2.89001195415001	-1.75665370958677	0.27731214290735
C	2.73423946314229	-3.07637258354365	2.27314020543333
H	2.97004850841931	-4.00548884872535	1.75098044502378
H	3.65744367577731	-2.69166394680225	2.71148509312249
H	2.01343145507275	-3.27669196288218	3.06585340295275
P	-1.65764753082163	0.21239739865592	-1.93690757381629
C	-2.56467447441689	1.70072776825535	-1.38900155727325
C	-2.94254287017297	-0.61978554737931	-2.94960512718185
C	-0.57263723130787	0.90345571067595	-3.24318242112914
H	-3.73816614567432	-0.98586055151745	-2.29569401563443
H	-3.37422399122484	0.06028973351351	-3.68985480905220
H	-2.50310918485987	-1.47695959632973	-3.46560890847124
H	-3.36416463502005	1.40791619944091	-0.70389414767792
H	-1.87967954648122	2.36208267999957	-0.85570274638831
H	-3.00146303150505	2.23266809109333	-2.23859383424108
H	0.18640248672510	1.53692762710308	-2.78037256730157
H	-0.06920772035266	0.08627490527516	-3.76559598287172
H	-1.14407823617782	1.49154678979780	-3.96718898023372

Product **PhosThiolate**:
Gibbs Energy: -1354.31353371 Hartree
Relative Gibbs Energy = 0.00 kcal/mol
$\Delta G^{\ddagger}$: 36.17 kcal/mol

C	0.55719047291400	-0.41751345648570	0.13779371623384
C	0.80603046306024	1.01570499595495	0.60069527890619
N	0.95688808646724	-1.42203078093871	1.09517836545753
C	-0.92300822778750	-0.65954098740552	-0.19914462420377
H	1.16915630961960	-0.54851702341212	-0.75835994963455
H	-0.95892987527995	-1.67817500006318	-0.60345879164864
O	0.58528188823172	1.94148829660739	-0.18352881764377
S	-2.03059371018691	-0.52592527229449	1.22939514269143
H	0.23874188743288	-1.75098784711230	1.72619799242841
C	1.54520919541944	2.52336964145359	2.36410787160706
N	1.27793097568531	1.19848998802706	1.83304113218958
H	1.39482094931560	0.39536834262151	2.42791612001934
H	1.97205502435271	2.41597177404522	3.35994032021838
H	2.25376079240063	3.05491364510382	1.72569267770591
H	0.62578424667818	3.11079380467195	2.42870408428104
C	2.20209538053415	-1.94376352071768	1.13469960326245

O	3.09848371686147	-1.55733023234969	0.38645930415663
C	2.43171698536969	-3.02824688296573	2.15851817493405
H	2.74037816738441	-3.93655333484399	1.63684001587770
H	3.24986678986300	-2.72144958414465	2.81293793653201
H	1.54569166531035	-3.23852428508315	2.75762926350357
P	-1.60624011616162	0.29620109543068	-1.57911818610019
C	-2.31317082240672	1.88713721556874	-1.16256591041304
C	-2.94565825636763	-0.71006078621759	-2.22318417706668
C	-0.41400617866985	0.49326732265309	-2.90671325002568
H	-3.66649195149447	-0.89840109463667	-1.42733194472701
H	-3.42966245174358	-0.17594274831922	-3.04259851979836
H	-2.54194850539617	-1.65572316707783	-2.58897242117359
H	-3.11984298764750	1.73859700441220	-0.44635778609126
H	-1.55178997239565	2.54213308735599	-0.74795789524372
H	-2.71315163040401	2.30900363066425	-2.08790135971296
H	0.37824069590627	1.16874441704327	-2.58809436232297
H	0.00432271088588	-0.48440630017050	-3.15481614246256
H	-0.92634219551811	0.90062465617223	-3.78041251631788

P11 Reactivity of C_β-centered Radical of Cysteine Residues

*The C_β-centered radical of C-methylated cysteine derivative **MeCYS-Rad** was subjected to a GOAT conformational search with subsequent reoptimisation by using the r2SCAN-3c method. All stable conformers placed the C_α–H bond orthogonal to the singly-occupied sp_2-type orbital. A selection of racemisation pathways were explored, including an amide-to-imidate rearrangement via an oxazolidine intermediate, C_α-to-C_β acyl transfer, C_α-to-C_β-hydrogen atom transfer and fragmentation/recombination (Figure 1.12.1).*

*P11.1 Conformers of C_β-centered radical of C-methylated cysteine derivative **MeCYS-Rad***

Table P11.1: Cartesian coordinates, Gibbs energies (Hartree), relative Gibbs energies (kcal/mol) and Boltzmann populations of conformers of *C_β-centered radical of C-methylated cysteine derivative* ***MeCYS-CbRad*** (*r2SCAN-3c*, CPCM: water).

Conformer 1 (**MeCys_CbRad_C1**): Gibbs Energy: -932.43879262 Hartree<br>Relative Gibbs Energy = 0.00 kcal/mol<br>Boltzmann Population: 50.8%				Conformer 2 (**MeCys_CbRad_C2**): Gibbs Energy: -932.43834734 Hartree<br>Relative Gibbs Energy = 0.28 kcal/mol<br>Boltzmann Population: 31.7%			
C	-0.33439541442174	0.13276698064064	-0.10173930634110	C	-0.29555841246853	0.02020637183554	-0.67577531667510
C	0.87734206223521	-0.82066097894443	-0.01815437820765	C	0.76499846265241	-0.98314999749567	-0.16397580404467
N	-1.55134589959329	-0.54813749308939	0.32926662863920	N	-1.60200181840841	-0.29322937467768	-0.10778580106389
C	-0.05201776326477	1.29903843225391	0.78243329104047	C	0.12478364337248	1.41252185378286	-0.34327196315500
H	-0.45239654792691	0.45838285380559	-1.14529675192267	H	-0.34502782704372	-0.06059938247900	-1.76951518569188
H	-2.93576204955218	-3.33838914850133	0.64724274449150	H	-3.42483973183504	-2.56999467483749	1.04290060530333
O	1.00929836507769	-1.62345829033286	0.91856192206152	O	0.74302597169388	-1.42285507074667	0.99436143722559
S	0.26404870545469	2.84433082735526	0.07525623861175	S	0.26633167571357	1.92204726624518	1.31154505230814
H	-2.03139395663225	-0.19941291539930	1.14687059949649	H	-2.05504827482673	0.39471148935895	0.47688726114352
C	3.05491756168256	-1.39395777364008	-0.97065946209206	C	2.86861908539374	-2.11659918024650	-0.68273902904143
N	1.79613514648323	-0.65796974320686	-0.97804856339887	N	1.73504836274481	-1.27210467877961	-1.03995194548491
H	1.61809340345438	0.00255472305307	-1.72118805732192	H	1.68025887614661	-0.89123167377077	-1.97360346625864
H	2.87916868563786	-2.47277362997881	-1.03728869139696	H	3.41617845341121	-1.69687076913713	0.16748799363696
H	3.64473193674495	-1.07876755282909	-1.83259432537737	H	2.53882638787883	-3.12730095222643	-0.41968492771222
H	3.62307613672440	-1.18949765320262	-0.05711045502944	H	3.53913422717488	-2.17674981274302	-1.54110508554607
C	-1.96927765213524	-1.68431606440253	-0.26298243857006	C	-2.18072766423514	-1.49487997203615	-0.29700776665216
O	-1.36578261218952	-2.16509616575315	-1.23907482296916	O	-1.63802063257210	-2.37341145655222	-0.99160373165680
C	-3.19854397351270	-2.33140055836902	0.30770521531370	C	-3.51129183098528	-1.71594608556884	0.36359351375877
H	-3.62937792923436	-1.77067572166691	1.13991082097848	H	-3.86253646185236	-0.84539165825978	0.92198182911308
H	-3.94381579797017	-2.43416867238131	-0.48685794042921	H	-4.24666851148894	-1.97691404478367	-0.40363973227140
H	0.12739533748488	1.14776770709860	1.84366986478978	H	0.58737703338916	2.02400038836364	-1.11128842345704

C	0.77525601101732	3.79608758931971	1.54767283304405	C	0.65660996151471	3.69071734501284	1.05996439920590
H	-0.02948722970911	3.80680016287348	2.28690578588914	H	0.79886621724266	4.13104651447591	2.04985323865101
H	1.68371837186691	3.36805872379343	1.97847309686851	H	1.57809188136599	3.79208416934774	0.48098743318798
H	0.97541624627816	4.81706074290398	1.21388474983188	H	-0.17030213887868	4.19171371941795	0.55139256227694

Conformer 3 (**MeCys_CbRad_C3**): Gibbs Energy: -932.4374368 Hartree<br>Relative Gibbs Energy = 0.85 kcal/mol<br>Boltzmann Population: 12.1%				Conformer 4 (**MeCys_CbRad_C4**): Gibbs Energy: -932.43667687 Hartree<br>Relative Gibbs Energy = 1.32 kcal/mol<br>Boltzmann Population: 5.4%			
C	-0.08717586117654	-0.36821927256774	-0.22252626548535	C	0.02651609399769	-0.24860603742931	-0.01528675752115
C	1.26690111065841	-0.92252063117825	0.24063497382300	C	1.37662112472847	-0.97392483309718	0.05019676591770
N	-1.11445164579581	-1.31442531210815	0.19961009055245	N	-1.01983344076849	-1.23854612396487	0.21022284298003
C	-0.26611960404474	0.98342022174389	0.39883165450760	C	0.01537045872973	0.81611989463263	1.04287169352162
H	-0.10090472820605	-0.29114576505997	-1.31732794924698	H	-0.10956443833762	0.18970282062073	-1.00944825789892
H	-2.88893035700029	-2.91474069939418	1.06520866458877	H	-3.99327723633969	-1.65021570053542	1.09633139245368
O	1.37116815304252	-1.53576706283172	1.31675014025215	O	1.56079683026981	-1.91603032697135	0.83972545217925
S	-0.03441698803722	2.40870957105607	-0.55660858239153	S	0.42549847052514	2.48047915974439	0.79668058543616
H	-0.87725923508704	-1.94118975891213	0.95815078087383	H	-0.75460489478271	-2.08997045496243	0.68885282597529
C	3.67582084854091	-0.98122257838818	-0.16794933077056	C	3.70764100872329	-0.96677111706930	-0.69039979233137
N	2.30826546363024	-0.64764905552920	-0.55173311521094	N	2.33291504537235	-0.48169842077792	-0.74451527550095
H	2.14867810705839	-0.15000199234863	-1.41575477481922	H	2.10498486419040	0.27799298713527	-1.36999689929507
H	3.79759321136798	-2.06297773246461	-0.05279728383211	H	4.12955309393070	-0.83498241828117	0.31151652325846
H	4.34874112706298	-0.63136135756635	-0.95186565831558	H	3.75992030708799	-2.02783974280389	-0.95466602095111
H	3.94620345032203	-0.49691012309584	0.77631427092552	H	4.30259566021098	-0.39550930657008	-1.40412694969675
C	-2.37685283640701	-1.26716162514447	-0.26778008053949	C	-2.31988905417722	-0.96564617760061	-0.01476162487827
O	-2.72971109438968	-0.45406598059096	-1.14088202969758	O	-2.69191658372455	0.11476161378137	-0.50688694582916
C	-3.34042830493383	-2.26603182374534	0.31106403614975	C	-3.30151176921683	-2.04551926030592	0.34552364300420
H	-4.17997067161194	-1.72270877695029	0.75608141757472	H	-3.88819133302768	-2.29799394920409	-0.54305825839630
H	-3.73884362693975	-2.87985689498710	-0.50286804434950	H	-2.82529745950257	-2.94852548344043	0.73376871836181
H	-0.33725281400422	1.07230444621093	1.47983290695388	H	0.05199214891150	0.49849423580647	2.08282868093689
C	-0.01490885076015	3.69157687761924	0.74466105793429	C	0.04290996757009	2.77501675463755	-0.96773653121496
H	0.83252404688133	3.53538669937211	1.41652767593310	H	-0.98217050978179	2.46960857971009	-1.19333241052265
H	0.09016317751106	4.65582186259292	0.24144043471638	H	0.13955929261279	3.85359081096095	-1.11739783085393
H	-0.95367345888158	3.67502839786796	1.30398547407338	H	0.75265814309821	2.26315167558455	-1.62290742923451

Figure P11.1: Structures of conformers of *C*$_{\beta}$*-centered radical of C-methylated cysteine derivative* ***MeCYS-CbRad*** (*r2SCAN-3c*, CPCM: water).

Conformer 1 (**MeCys_CbRad_C1)**

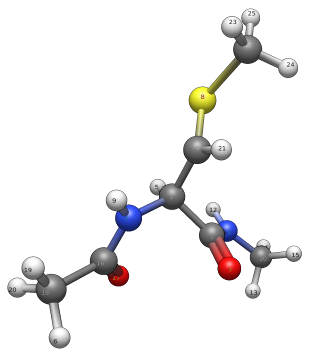

Conformer 2 (**MeCys_CbRad_C2**)

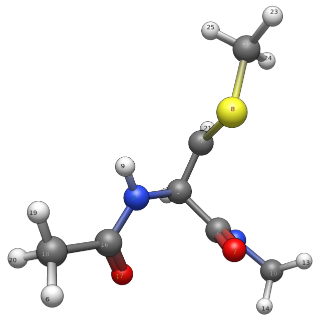

Conformer 3 (**MeCys_CbRad_C3**)	Conformer 4 (**MeCys_CbRad_C4**)
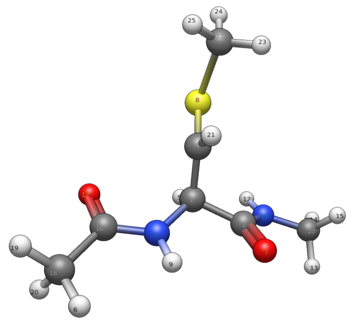	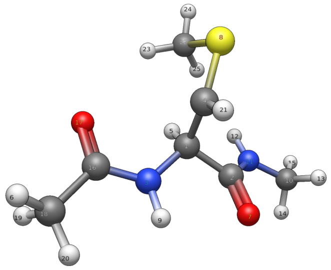

P11.2 Amide-to-Imidate Rearrangement of ***MeCYS-Rad***

Figure P11.2: Structures and relative Gibbs energies of reactants, products and transition states for the conversion of *C_β-centered radical* ***MeCYS-CbRad*** *into imidate derivative* ***Imid*** *via oxazolidine intermediate* ***Oxaz*** and respective transition-state structures ***MeCYS-CbRad---Oxaz*** and **Oxaz---Imid**.

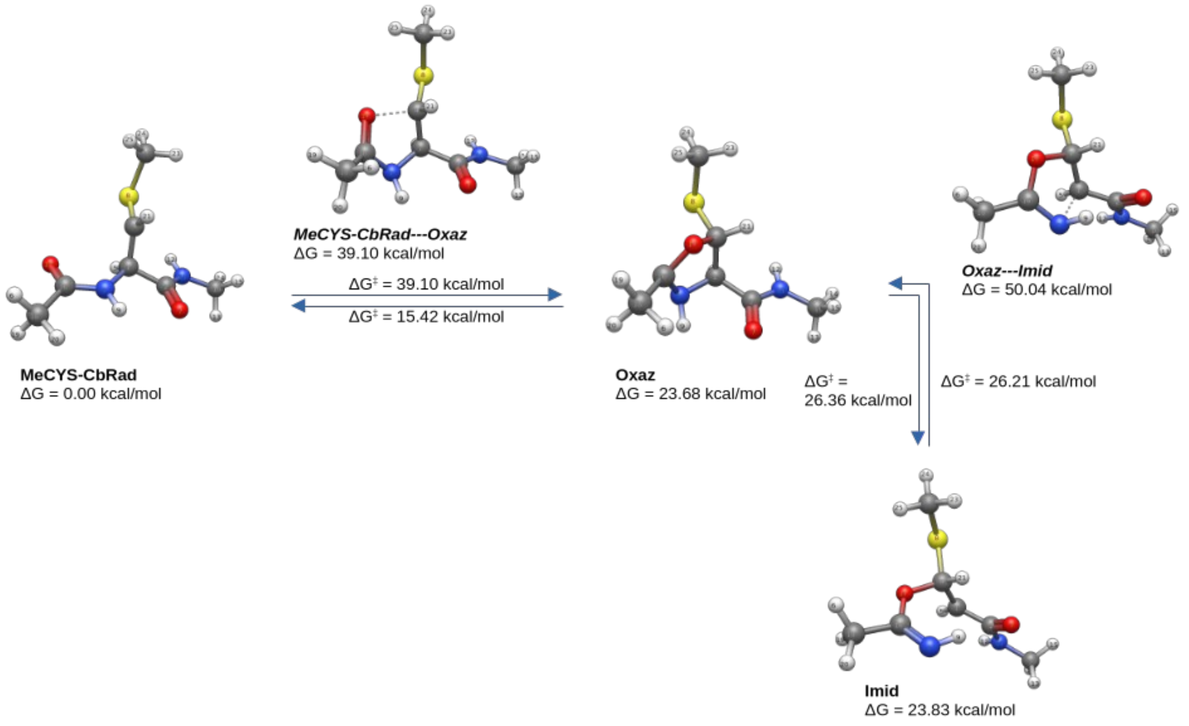

Table P11.2: Cartesian coordinates, Gibbs energies (Hartree), relative Gibbs energies (kcal/mol) and imaginary frequencies, where relevant, of *C_{β}-centered radical* ***MeCYS-CbRad***, *oxazolidine intermediate* ***Oxaz***, *imidate derivative* ***Imid*** and respective transition-state structures ***MeCYS-CbRad---Oxaz*** and **Oxaz---Imid**. All species calculated at the following level: (wB97X-D4/def2-TZVP/def2/J, CPCM: water).

Reactant **MeCYS-CbRad**:
Gibbs Energy: -933.00623559 Hartree
Relative Gibbs Energy = 0.00 kcal/mol
$\Delta G^{\ddagger}$: 39.10 kcal/mol

C	-0.12012066725124	-0.27820147120778	-0.37332554272338
C	1.25028839562543	-0.86515283475310	0.00429135200055
N	-1.12882573690406	-1.23226351604012	0.03809964194673
C	-0.28662029407940	1.06079035200723	0.28016982491135
H	-0.16135617624284	-0.16297705427345	-1.46051400015248
H	-4.16356471435712	-1.77351270715036	0.55851087792368
O	1.35600223542286	-1.76503657830082	0.83596484765960
S	-0.15883166789745	2.47280863817310	-0.66456883723856
H	-0.84510188009224	-1.92509729608525	0.71417339065925
C	3.66609986971105	-0.66632620377369	-0.28329641339364
N	2.29730699302782	-0.29899996503209	-0.60488648883702
H	2.13523029593890	0.44966633771608	-1.25880565764183
H	3.83611966529048	-1.72735726790439	-0.47797486595953
H	4.33535901591827	-0.07772645056652	-0.90867326329067
H	3.88699109300306	-0.46445858189663	0.76777282535047
C	-2.38004663715739	-1.21804245058338	-0.45690178410899
O	-2.74405660141906	-0.39710147458267	-1.29977926690504
C	-3.31792786650454	-2.27270876765242	0.08072772410588
H	-3.70192320631874	-2.85769735741060	-0.75724759112607
H	-2.83829790521637	-2.93789623648419	0.79898647752059
H	-0.33737876054569	1.13407850598843	1.35993974893287
C	-0.14364052079005	3.75483442227665	0.59691026324335
H	0.73086852284903	3.64338616127229	1.23769188240486
H	-0.09750220752870	4.71155532792389	0.07664908273020
H	-1.05677164442053	3.70693241549810	1.19046885382867

Transition state **MeCYS-CbRad---Oxaz**:
Imaginary frequency: −1044.23 cm^{-1}
Gibbs Energy: -932.94392435 Hartree
Relative Gibbs Energy = 39.1 kcal/mol

C	0.03916434762428	-0.38344571573121	-0.90029041610117
C	1.27460932924303	-0.92717193239739	-0.18408233607766
N	-1.01689338948057	-1.41580803675257	-0.94594393081938
C	-0.51634922799160	0.79323171296194	-0.13438351142075
H	0.30688577580575	-0.07210925339730	-1.91411945991256
H	-2.57097838054005	-1.59747254878788	1.53080851770107
O	1.18017622122031	-1.75733410020013	0.71865284221895
S	-0.09944314251056	2.36607568270058	-0.74006646019797
H	-0.73775251515742	-2.28909986666989	-0.51506798218842
C	3.69906046338466	-0.77155695750316	0.04638125302062
N	2.44207507217614	-0.41468854038909	-0.58946177617534
H	2.45259064572665	0.24896852401966	-1.34669770627926
H	3.89240207733975	-1.84212857514882	-0.05242538728318
H	4.49967346678911	-0.21756799067403	-0.44102447950226
H	3.68126224776329	-0.51687906536308	1.10853490043187
C	-2.21053741618538	-0.89685077250335	-0.45977871964833
O	-2.23911445281260	0.39445357320599	-0.52177076714754
C	-3.02074476437577	-1.67641552908992	0.52984177274187
H	-4.03980906895044	-1.29082715826999	0.57903796059800
H	-3.05579042722155	-2.73314580282529	0.25645961944975
H	-0.50783312484487	0.70086767285191	0.94911927031957
C	-0.76846039748136	3.40798095206074	0.56800601443938
H	-0.30175612063707	3.16433179984508	1.52278899549170
H	-0.53283994566762	4.43885022715099	0.30226061038338
H	-1.84959727321611	3.28774170090621	0.63322117595768

Oxazolidine Intermediate **Oxaz**:
Gibbs Energy: -932.96850543 Hartree
Relative Gibbs Energy = 23.68 kcal/mol
$\Delta G^{\ddagger}$: 15.42 kcal/mol (back to **MeCYS-CbRad**)
$\Delta G^{\ddagger}$: 26.36 kcal/mol (forward to **Imid**)

C	0.14383384758060	-0.25633293890404	-0.95224552304550
C	1.26877519832401	-1.05411853121463	-0.28208796263928
N	-1.06756456454083	-1.04574803577727	-1.10238980855060
C	-0.32440083136472	0.89025414825450	-0.03180385691919
H	0.50607135517300	0.13155632639090	-1.90788515340633
H	-2.25793851680602	-2.28094911798763	1.21771628133164
O	1.04758737249138	-2.10345454362444	0.31707334314817
S	-0.82656001999235	2.30570508952796	-1.03992585135977
H	-0.87727113407576	-2.04230044040137	-1.06276828039481
C	3.65485723950507	-1.10133577065888	0.23918274758562
N	2.48557940799223	-0.50756862932259	-0.38703620077541
H	2.59715472976720	0.34013771940872	-0.91953564288220
H	3.83549993506744	-2.10528906701370	-0.15180026901964
H	4.51735716200270	-0.47323846717342	0.02233431072529
H	3.52228244786041	-1.16702368016021	1.32149389929739
C	-1.99573973525605	-0.62506145845052	-0.12272855565608
O	-1.39428941802457	0.32400422419644	0.70007174203515
C	-2.85649474758351	-1.61916955699253	0.57319740405815
H	-3.59931576637633	-1.11468776170947	1.19362204628379
H	-3.37947971022184	-2.23750015557826	-0.15909905035196
H	0.43013606786271	1.21731062649082	0.68311436781871
C	-1.28892151318190	3.44546763228118	0.27599762985102
H	-0.44037446706285	3.62954992470355	0.93680266975138
H	-1.58149703063012	4.38114825339052	-0.20162724337478

Transition state **Oxaz---Imid**:
Imaginary frequency: −861.9 cm^{-1}
Gibbs Energy: -932.92649082 Hartree
Relative Gibbs Energy = 50.04 kcal/mol

C	0.23505933386393	-0.23530321073443	-0.36997747163527
C	1.62160898172318	-0.55090238288843	0.02805302664712
N	-0.80277418454172	-1.64636939819364	0.47087120368699
C	-0.48421255002973	0.83535647064199	0.39584615228552
H	-0.00347419663469	-0.30939996567893	-1.42752058430149
H	-4.05564574049571	-1.00795506437599	0.22177014850228
O	1.97512065989575	-0.50322878154883	1.21369475310007
S	-0.53786068241386	2.36240515574251	-0.53654944078781
H	-0.26300865006547	-1.43321791382859	1.30917333741129
C	3.81842766599282	-1.34512316942339	-0.69535875322567
N	2.44872081515546	-0.94350359164514	-0.95690762848241
H	2.10942389190632	-0.95123235805739	-1.90421466163526
H	3.84965537010660	-2.19668577575351	-0.01126625504656
H	4.27960000608872	-1.62999896106122	-1.63985841594029
H	4.38867553531163	-0.52448457674426	-0.25291965883755
C	-1.93608817675805	-0.95843006416110	0.42553360773579
O	-1.85479936051320	0.37301174353297	0.65499582403505
C	-3.16987698884652	-1.41150797443604	-0.27419536972396
H	-3.17045600674322	-1.03836164077652	-1.30634877545475
H	-3.22452813532802	-2.49903390650122	-0.28484862492773
H	-0.00524636291004	1.01132542384136	1.35940905702118
C	-1.20690466703548	3.47211569266172	0.71682209146826
H	-0.56297953968259	3.48366870372698	1.59709984792743
H	-1.22913554030386	4.46860787737489	0.27425414923871

H	-2.13158690769755	3.05514826297494	0.84594387306940	H	-2.21930147774223	3.17824766828621	0.99244244093903

Product **Imid**:
Gibbs Energy: -932.9682557 Hartree
Relative Gibbs Energy = 23.83 kcal/mol
$\Delta G^{\ddagger}$: 26.21 kcal/mol

C	0.64859918032188	0.22518335733695	-0.85201195077792
C	1.80832504512153	-0.40821303912005	-0.23012618826875
N	-1.16967731118296	-1.59768875188605	1.08073312164188
C	-0.39149353378843	0.87988286236509	-0.02022518804446
H	0.53270901736232	0.22716333435637	-1.93058613155978
H	-4.05576946831866	-0.56674179633258	0.41008098584725
O	1.90990986572816	-0.52295792443468	1.00120518883202
S	-0.47832863124442	2.61255345241957	-0.49202704150728
H	-0.24408694055934	-1.19048147378361	1.20067591620581
C	3.94703816240062	-1.53140920006066	-0.60288558211235
N	2.74622565992473	-0.86967638235191	-1.07755474553108
H	2.61554256765952	-0.74355300913689	-2.06789581443775
H	3.69358229428700	-2.42305309853498	-0.02431942530252
H	4.54396268127151	-1.82185355020461	-1.46584625385925
H	4.53412037941438	-0.86253894730245	0.03140547414707
C	-1.95101873680539	-0.90495416505857	0.36336666902849
O	-1.68627655550532	0.29138406249361	-0.23335394147471
C	-3.35552909030375	-1.32493137293734	0.05181171419143
H	-3.48254058631502	-1.41219894200198	-1.02980032961765
H	-3.57215996772113	-2.28013961199662	0.52578772032279
H	-0.13457207897358	0.82483206255458	1.03881913436728
C	-1.46071492845099	3.25110722969347	0.87774397917106
H	-0.95013864085325	3.07956399879350	1.82591821902325
H	-1.56600268918708	4.32396707760280	0.71251334502244
H	-2.44775647988288	2.78977728418188	0.88848994461264

*P11.3 1,2-Acyl Migration of **MeCYS-Rad***

Figure P11.3: Structures and relative Gibbs energies of reactants, products and transition state for the conversion of C_β-centered radical **MeCYS-CbRad** into C_α-centered radical β-amino amide **β-AA** via transition-state structure **MeCYS-CbRad---β-AA**.

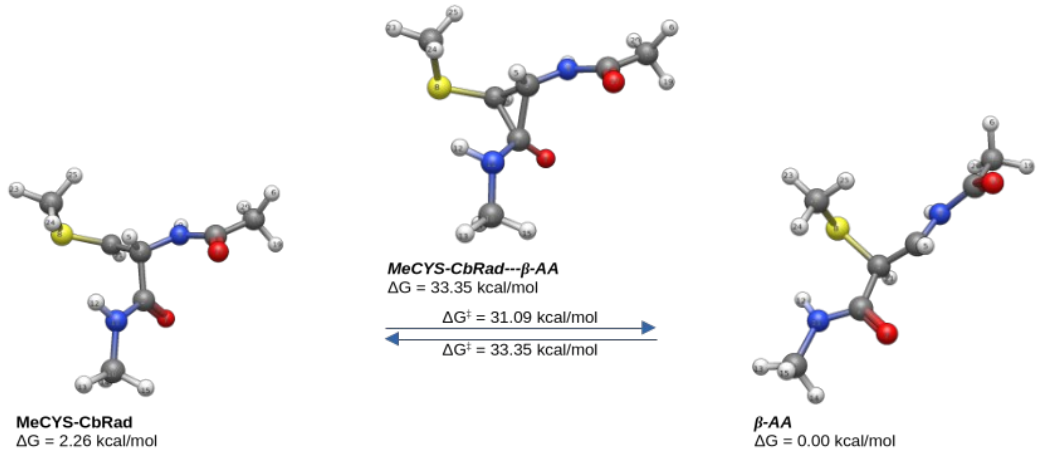

Table P11.3: Cartesian coordinates, Gibbs energies (Hartree), relative Gibbs energies (kcal/mol) and imaginary frequencies, where relevant, of *C_β-centered radical **MeCYS-CbRad***, C_α-centered radical β-amino amide **β-AA** and transition-state structure **MeCYS-CbRad---β-AA**. All species calculated at the following level: (wB97X-D4/def2-TZVP/def2/J, CPCM: water).

Reactant **MeCYS-CbRad**:
Gibbs Energy: -933.00349933 Hartree
Relative Gibbs Energy = 2.26 kcal/mol
$\Delta G^{\ddagger}$: 31.09 kcal/mol

C	-0.20775130429745	0.22658649322072	0.38173251978826
C	1.00762127057462	-0.68245217297087	0.10335424751133
N	-1.39166168763848	-0.57326926641697	0.62139944227585
C	0.10363480619190	1.05801175086463	1.58722189408932
H	-0.37972255267434	0.85558863319940	-0.49337479227377
H	-3.90263425253785	-2.00927513412009	-0.67751162249741
O	1.15287411408877	-1.75718005130516	0.67869143751451
S	0.74370334871254	2.63541704591355	1.54904191858831
H	-1.78173447165202	-0.59812222479573	1.54889824398558
C	3.15913209007508	-0.85924042104919	-1.03856602680841
N	1.91182756162449	-0.17484590146016	-0.74447077017935
H	1.71326843403788	0.69011108465425	-1.21859787709350
H	3.74130035585483	-0.23737418094493	-1.71698037397903
H	3.73279747002075	-1.02448693680294	-0.12381242203227
H	2.97248798449641	-1.82653038983735	-1.51163585259812
C	-1.90315900138811	-1.36435879341808	-0.34183100244401
O	-1.41430786601272	-1.40137308256632	-1.47134610484596
C	-3.10202298751293	-2.19687404451469	0.04006800317453
H	-2.82581641551138	-3.25133717237647	-0.03017363008770
H	-3.45763085497282	-1.98465617400783	1.04859293550179
H	0.26376774474335	0.54097445644016	2.52825999570392
C	0.06800385440051	3.36648622173970	0.04798851291879
H	0.31025919989769	4.42832349943310	0.10014390991895
H	0.52245731463028	2.94280656211313	-0.84700517483222
H	-1.01462379601385	3.24212603262972	0.02199173595845

Transition state **MeCYS-CbRad---β-AA**:
Imaginary frequency: −513.21 cm^{-1}
Gibbs Energy: -932.95395759 Hartree
Relative Gibbs Energy = 33.35 kcal/mol

C	-0.40997605024708	0.25448460030790	0.17018656499877
C	1.09799422048165	-0.45144735065057	0.26339385611036
N	-1.50781040490628	-0.54962184571246	0.50817874788873
C	0.45543104659747	0.76447145676101	1.21632282798021
H	-0.49574293408737	0.79666151210823	-0.76263690792061
H	-4.04455289261120	-2.17711219926109	-0.60845322657027
O	1.16543689351450	-1.59447454410366	0.76530791474806
S	1.14353450035861	2.38607326364547	1.21273797150724
H	-1.79233841437048	-0.60874434688242	1.47327612639409
C	3.19212565312617	-0.77672929825683	-0.93008724057082
N	1.96204398620238	-0.02195719713415	-0.74029543340959
H	2.08513040087821	0.98028876337947	-0.78318086768187
H	3.76837914294094	-0.29772306986145	-1.72209419174079
H	3.80321976933209	-0.81938246425206	-0.02026094891643
H	2.96080660820807	-1.79818554704938	-1.23474750315537
C	-2.08557262942850	-1.37544934915419	-0.40879398374876
O	-1.69962496092449	-1.40801075481852	-1.57099980930180
C	-3.21411719437934	-2.23753399178698	0.09606884064195
H	-2.86708948211973	-3.27343195482523	0.12307881857485
H	-3.55234073905824	-1.94913126911066	1.09146759602286
H	0.31457411765239	0.33812742611509	2.20368008523484
C	0.08017526878079	3.29838290703098	0.07570945846099
H	0.37055707351247	4.34434886198931	0.18354127996829
H	0.23608681555302	2.99238767628452	-0.95809496441922
H	-0.96632979500602	3.18370871523765	0.35669498890431

Product **β-AA**:
Gibbs Energy: -933.00710012 Hartree
Relative Gibbs Energy = 0.00 kcal/mol
$\Delta G^{\ddagger}$: 33.35 kcal/mol

C	-0.59739218313456	-0.72771898390091	-0.24619457252843
C	1.86023288974680	-0.38505808030769	-0.30821159771351
N	-1.78700111186014	-0.93189878405974	0.41395426377416
C	0.57646188391226	-0.27040709246728	0.52889730457500
H	-0.62404065614703	-0.72384482813680	-1.32311121536606
H	-4.93119154896735	-0.69725263121921	0.42065923258815
O	1.94037516293084	-1.20985127989656	-1.21685307612325
S	0.32075252106139	1.37764080797222	1.29355970001145
H	-1.79310099586144	-0.86981079132391	1.42173894752466
C	4.16210256317845	0.35051006375815	-0.62714025132916
N	2.87510845518076	0.41050192522077	0.04136969975190
H	2.72198154263524	1.08578504410115	0.77391013700765
H	4.82362377251337	1.08209271215400	-0.16568219543281
H	4.60381565034463	-0.64395758474653	-0.52933919073612
H	4.05914809873250	0.58143951190957	-1.69017379297264
C	-2.96868828415603	-1.21364786789878	-0.21144700125326
O	-3.04641408917246	-1.29363504046067	-1.43396129305493
C	-4.15904304926973	-1.42114236590811	0.68976355500051
H	-4.56040866309571	-2.42125219920374	0.51234516122162
H	-3.91506788362112	-1.31302102409914	1.74679783800277
H	0.72919520235328	-0.91430425689890	1.40567456227340
C	-0.02397683919417	2.36362512372301	-0.17264107091751
H	-0.19631122315345	3.38432951228845	0.17059138926906

H	0.82584053921151	2.35536841940024	-0.85664736047802	
H	-0.91807211183125	2.00045385775725	-0.67993831856660	

*P11.4 1,2-Hydrogen Atom Transfer of **MeCYS-Rad***

Figure P11.4: Structures and relative Gibbs energies of reactants, products and transition state for the conversion of C_β-centered radical **MeCYS-CbRad** into C_α-centered radical **MeCYS-CaRad** via transition-state structure **MeCYS-CbRad---MeCYS-CaRad**.

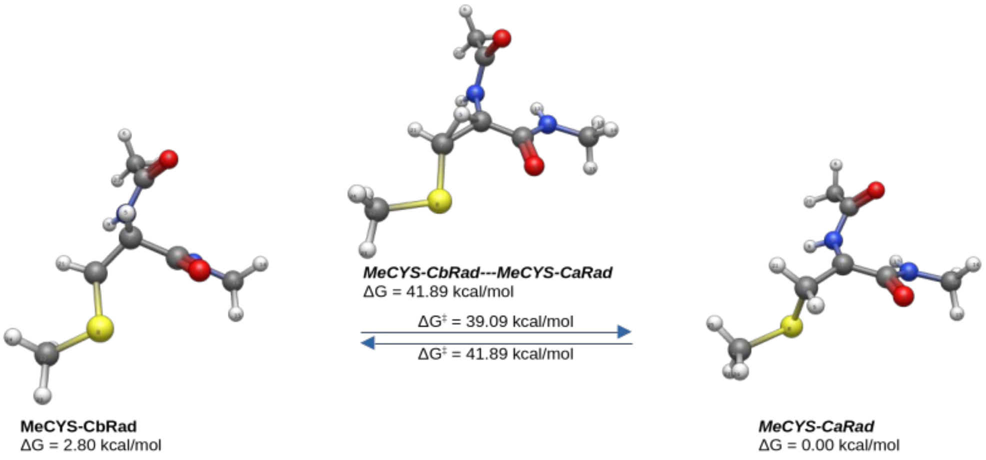

Table P11.4: Cartesian coordinates, Gibbs energies (Hartree), relative Gibbs energies (kcal/mol) and imaginary frequencies, where relevant, of *C_β-centered radical **MeCYS-CbRad***, C_α-centered radical **MeCYS-CaRad** and transition-state structure **MeCYS-CbRad---MeCYS-CaRad**. All species calculated at the following level: (wB97X-D4/def2-TZVP/def2/J, CPCM: water).

Reactant **MeCYS-CbRad**:<br>Gibbs Energy: -933.00489815 Hartree<br>Relative Gibbs Energy = 2.80 kcal/mol<br>$\Delta G^‡$: 39.09 kcal/mol				Transition state **MeCYS-CbRad---MeCYS-CaRad**:<br>Imaginary frequency: −1878.46 cm^{-1}<br>Gibbs Energy: -932.94260819 Hartree<br>Relative Gibbs Energy = 41.89 kcal/mol			
C	-0.33603883181993	0.49938774541187	-0.64525735605896	C	0.00194986852360	0.29662894258311	-0.04574250519087
C	1.11636402239240	0.04427532393657	-0.84958430275366	C	1.38539903056595	-0.06413736890135	-0.39306058216526
N	-1.21657894506325	-0.56973351463633	-0.21135784750742	N	-0.97046170082257	-0.70447251726627	0.17201972583655
C	-0.41469066025141	1.62467117657704	0.34334590688144	C	-0.34469127736625	1.65422380206426	0.44653736749127
H	-0.66485275182808	0.82957368762602	-1.63874519694461	H	-0.35547632969977	1.26630969835318	-0.82032040041923
H	-3.40735401415153	-2.64286266271279	-1.14910768633987	H	-3.41885804201615	-2.38543574154241	-1.11985763480562
O	1.84242989936309	0.65368658628161	-1.63378655119092	O	2.21694020256780	0.79605432412042	-0.71336902225687
S	0.86167777903228	2.74404007712347	0.52373040753597	S	0.80734597283904	2.78969021107598	1.09539952528537
H	-1.59638709661025	-0.53859954204217	0.72105742370955	H	-1.35524325809417	-0.79948987471036	1.10113656443595
C	2.89204082060601	-1.49001176140541	-0.19009223256985	C	3.00818018949152	-1.86428067876333	-0.70291478550391
N	1.53166627071555	-0.98817760068906	-0.11193024615018	N	1.68610886619242	-1.37783723328811	-0.35828957220627
H	0.87172536258966	-1.44607879663554	0.49480767703229	H	0.99876342804662	-2.02778887943863	-0.01739713036220
H	2.98747904498477	-2.33510472900848	0.48986207522353	H	3.00240720332920	-2.95127708665261	-0.63356873038115
H	3.12556310059090	-1.81926222281768	-1.20537899069268	H	3.27377416475508	-1.57480368283299	-1.72245192087533
H	3.60717511583320	-0.71588597904964	0.09762252422739	H	3.76752088084673	-1.46829167523966	-0.02304880882557
C	-1.57802623339595	-1.56641860864602	-1.05034246820829	C	-1.54717877782103	-1.41324547451368	-0.84222604389180
O	-1.13885158223627	-1.63127904347059	-2.19662367887974	O	-1.21841510704404	-1.25853776748569	-2.01119408096742
C	-2.53318596424435	-2.59419970594162	-0.49712267209249	C	-2.58403046285410	-2.42286930454790	-0.41965943535867
H	-2.04208963493905	-3.56932621047135	-0.51814593455955	H	-2.13591241585145	-3.41829902195232	-0.47643194110212
H	-2.85057603675819	-2.37143362105814	0.52169956798249	H	-2.94226295984428	-2.25579766591117	0.59645691820142

H	-1.39334959913731	1.89962605639835	0.72146363415139	H	-1.37848771217323	1.76053016706511	0.75257620325742
C	0.17629235713962	3.81315619830427	1.79948208142976	C	-0.31977170372524	4.14528377963667	1.47111260494729
H	0.91027009792922	4.59867819588153	1.97985001490076	H	0.28318662191770	4.93290616012186	1.92443243518454
H	-0.75702921418349	4.25931073751109	1.45542696507958	H	-0.78004777432354	4.52449974867107	0.55846497138751
H	0.01037555306751	3.24915203241665	2.71716830328853	H	-1.08789616133983	3.82754866905482	2.17686850318492

Product **MeCYS-CaRad**:
Gibbs Energy: -933.00936801 Hartree
Relative Gibbs Energy = 0.00 kcal/mol
$\Delta G^{\ddagger}$: 41.89 kcal/mol

C	-0.00490589410634	0.36805012832845	-0.33939547571618
C	1.32917298052392	-0.07437712358373	-0.77655026417841
N	-0.99229091406299	-0.52908923031638	0.04219841559156
C	-0.23508314584837	1.80414813342199	-0.04951008323994
H	0.46746019650881	2.41038678659022	-0.61933144375988
H	-3.14469692054471	-2.84406163296327	-0.86009676746031
O	2.07689861748805	0.69651559651394	-1.39420471698931
S	0.01807631532422	2.11676698465718	1.73269132967230
H	-1.59463055344329	-0.24133962646142	0.80223108061860
C	2.98961190038007	-1.86310770986985	-0.84983096020388
N	1.71078945664995	-1.31890791760585	-0.43585758838471
H	1.09122544295576	-1.89613413176720	0.10548066452482
H	3.06087785055869	-2.88714459099511	-0.48593157772886
H	3.07707273680682	-1.86449741731177	-1.93939794689887
H	3.81728589667979	-1.28002962291896	-0.43801204509974
C	-1.42247761212976	-1.60371747512226	-0.70862826996318
O	-0.92436336461063	-1.87964783601715	-1.78702445470091
C	-2.51766480445166	-2.42207902870765	-0.07570245448581
H	-2.05395749810103	-3.24365595472180	0.47773316355694
H	-3.12488627122080	-1.83904904994469	0.61783701938931
H	-1.25978313098360	2.08880909409860	-0.29927829785032
C	-0.51719601627468	3.83481938266970	1.80221753891548
H	-0.41720480380093	4.16072489089667	2.83830308041355
H	0.11274042622710	4.45939757435893	1.16717818559338
H	-1.56043425627376	3.92425901515101	1.49578490126078

*P11.5 Fragmentation of **MeCYS-Rad***

Figure P11.5: Structures and relative Gibbs energies of reactants, products and transition state for the fragmentation of C_{β}-centered radical **MeCYS-CbRad** into acetamidyl radical and enesulfide **AR/ES** via transition-state structure **MeCYS-CbRad---AR/ES**.

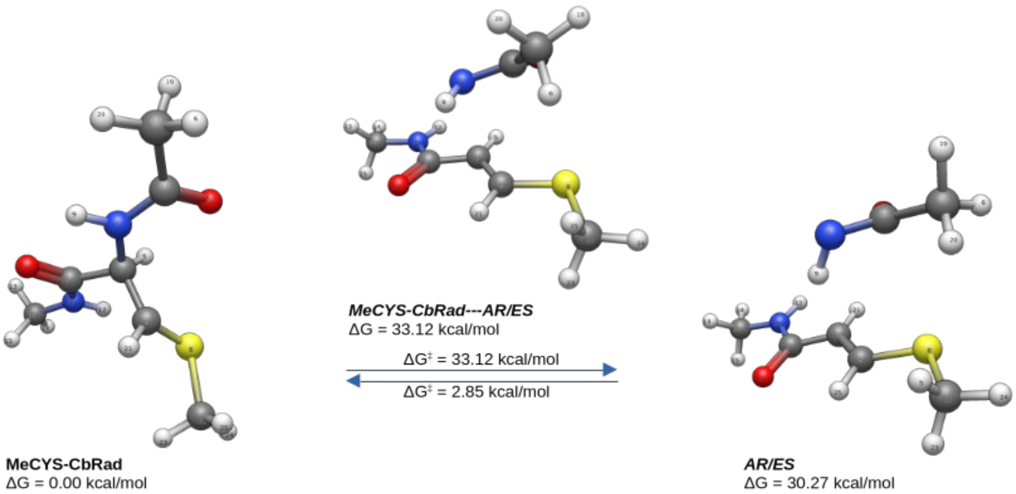

Table P11.5: Cartesian coordinates, Gibbs energies (Hartree), relative Gibbs energies (kcal/mol) and imaginary frequencies, where relevant, of C_β*-centered radical* ***MeCYS-CbRad***, acetamidyl radical and enesulfide **AR/ES** and transition-state structure **MeCYS-CbRad---AR/ES**. All species calculated at the following level: (wB97X-D4/def2-TZVP/def2/J, CPCM: water).

Reactant **MeCYS-CbRad**:
Gibbs Energy: -933.00620583 Hartree
Relative Gibbs Energy = 0.00 kcal/mol
$\Delta G^{\ddagger}$: 33.12 kcal/mol

C	-0.13143273650431	-0.30995610606002	-0.36389804753790
C	1.24420168931336	-0.89118709903984	0.00369261201950
N	-1.13347766280417	-1.26385727247552	0.06402640180958
C	-0.29494818197240	1.03309485923540	0.28200810859105
H	-0.18392965486031	-0.20218775715112	-1.45139193681598
H	-4.16266400354157	-1.80702507760092	0.61916529542989
O	1.36005058764545	-1.78948013626064	0.83569319244632
S	-0.18270763665280	2.43865447699798	-0.67425450235988
H	-0.84120314623493	-1.95097211566804	0.74232023098650
C	3.65682252924824	-0.68507129518492	-0.30416226097698
N	2.28433353717901	-0.32228005641867	-0.61476027251583
H	2.11451822205062	0.42566427280633	-1.26755793192590
H	3.82786705281451	-1.74620575094175	-0.49733472142411
H	4.31898667089611	-0.09651977981058	-0.93709684212328
H	3.88653489033876	-0.47968816673549	0.74435021506883
C	-2.39015103069517	-1.25502368181746	-0.41715348583965
O	-2.76487438117986	-0.44029825762144	-1.26132279404350
C	-3.32026551884002	-2.30786051367873	0.13740283492164
H	-3.71056966666428	-2.90025344199800	-0.69241553211613
H	-2.83231798118914	-2.96629030283350	0.85630016738407
H	-0.33316965940000	1.11395622547814	1.36175842291326
C	-0.15473201083653	3.72975031999852	0.57769239864761
H	0.72823218447752	3.62534029271231	1.20796572014622
H	-0.11802854487606	4.68283980130302	0.05006663878002
H	-1.06002164288525	3.68363168614833	1.18329398789625

Transition state **MeCYS-CbRad---AR/ES**:
Imaginary frequency: −115.53 cm^{-1}
Gibbs Energy: -932.95341879 Hartree
Relative Gibbs Energy = 33.12 kcal/mol

C	0.81493608988795	0.47917941136954	-0.64163660800062
C	1.94620408596179	-0.26163596307074	-0.01890233816138
N	-0.70169796340782	-1.48829221405257	-0.13209233578150
C	0.08162024077256	1.32652142079036	0.10003723557404
H	0.65740208699221	0.37896007183599	-1.71013103862997
H	-3.01848100182781	-0.08834453874376	0.81362888128999
O	2.15620434088581	-0.25911056453395	1.19709545312686
S	-1.20201527659301	2.28871978920371	-0.52668492522780
H	-0.67559541888248	-1.26237674405825	0.86427613068184
C	3.85110740445021	-1.73685683407705	-0.43805636061930
N	2.72449416670235	-0.93690941825031	-0.88186786293979
H	2.49664327948232	-0.92005849664830	-1.86199287241019
H	3.52299633770446	-2.53258533274276	0.23587614675692
H	4.32274618112500	-2.18283318164131	-1.31277519417387
H	4.58417587589863	-1.11924517106079	0.08657868862447
C	-1.90858642575988	-1.09451773123833	-0.68562646311578
O	-1.99043966058426	-0.85322101472861	-1.88137020674820
C	-3.09618229715345	-1.01969963608442	0.24312837529303
H	-4.02145728551963	-1.01809797163652	-0.33185950141773
H	-3.09366380534761	-1.84875180128740	0.95427604181627
H	0.28634954053458	1.41639170015647	1.16321070786008
C	-1.82650456479600	3.06353827560949	0.97650510899003
H	-1.05022136672468	3.66622376435467	1.44700797679251
H	-2.64937869320237	3.70920506628587	0.66849112565340
H	-2.19835627069888	2.30729306124903	1.66726691656668

Product **AR/ES**:
Gibbs Energy: -932.9579653 Hartree
Relative Gibbs Energy = 30.27 kcal/mol
$\Delta G^{\ddagger}$: 2.85 kcal/mol

C	0.73941417934184	0.14038123743141	-0.64650021739920
C	2.01857275338043	-0.39462009602049	-0.10996839514627
N	-1.87534034328374	-1.94900885561088	0.65483732107097
C	-0.03056137296582	0.92040308700775	0.11402013262607
H	0.45728936039922	-0.11988390408263	-1.66228056753264
H	-4.34975189663324	-0.56633206636784	-1.12989247846677
O	2.41786366715131	-0.16383491741224	1.03525528526006
S	-1.53034074587480	1.61242131162896	-0.41525099547612
H	-0.98302128517256	-1.44307574860393	0.67687846849836
C	3.98369047654564	-1.76536875650838	-0.60571543060729
N	2.71575543015261	-1.16067622030393	-0.96876969990656
H	2.34705099985386	-1.32108735531847	-1.89124580744910
H	3.86393676786425	-2.44324934704863	0.24311232970516
H	4.35461479541353	-2.32845741873801	-1.46093355834100
H	4.71677124396868	-1.00097467921424	-0.33647273542281
C	-2.60251740272906	-1.59414125847605	-0.47734661263631
O	-2.14360110921742	-1.84542411304424	-1.57860969204625
C	-3.97799116502065	-1.05131718412119	-0.22823066584001
H	-4.63257307768365	-1.88771822822629	0.03484646230621
H	-3.97063905733711	-0.35160598362869	0.60934285160577
H	0.27859919457826	1.15385360360657	1.12916627238353
C	-2.16973212860240	2.27543483335081	1.13399125309114
H	-1.47160453087297	2.99620343039194	1.55882813095297
H	-3.10495896215458	2.77969612990280	0.88938274466432
H	-2.36813238880154	1.47075729000596	1.84154161620577

P12 Molecular Dynamics Simulations

The following peptides were simulated according to the procedure outlined above (see **P2**).

WT: Ac-Ala-Leu-Cth-Thr-Pro-Ser-**Arg**-Gly-Ser-Leu-Phe-Thr-Gly-Arg-NH2

R7A: Ac-Ala-Leu-Cth-Thr-Pro-Ser-**Ala**-Gly-Ser-Leu-Phe-Thr-Gly-Arg-NH2

Cth = cysteine thioaldehyde

Figure P12.1: Reweighted conformational distributions showing that peptides WT and R7A have similar conformational distributions, with the latter showing slightly more compact structures. The α-helix, β-sheet and random coil contributions are determined by using the Define Secondary Structure of Proteins (DSSP) algorithm, which shows that the peptides are intrinsically disordered.

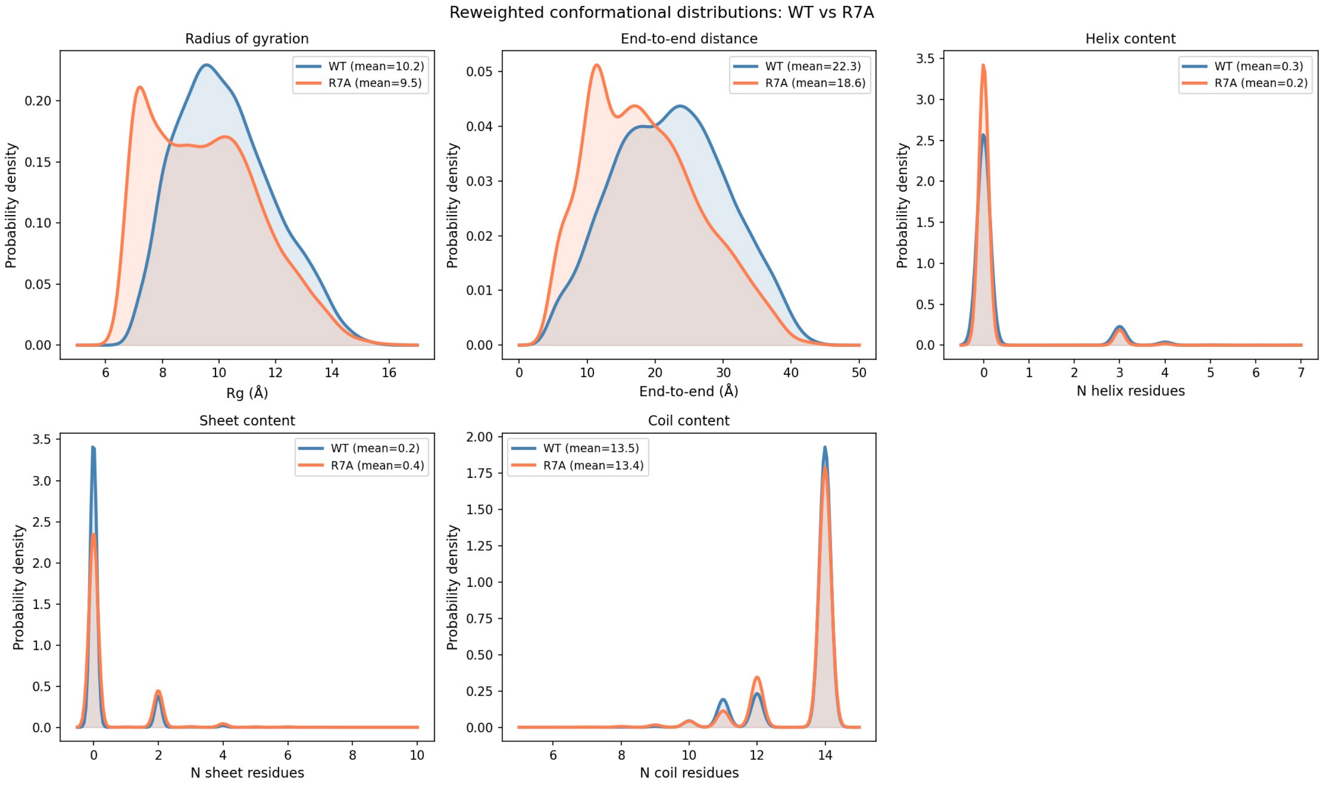

Figure P12.2: Reweighted structures showing the free-energy landscape associated with the distance between the leucine carbonyl oxygen atom (LEU2-O) and the thiocarbonyl carbon atom (CTH-CB). There are two minimum, one where the leucine carbonyl is pointing away (global minimum) and another approx 0.5 kcal/mol less favored where it is in proximity for an n → π* interaction (towards oxazole) or to assist in the addition of water (hydration towards formylglycine). The WT peptide exhibits a slightly higher population of conformers that place the leucine carbonyl oxygen in close proximity.

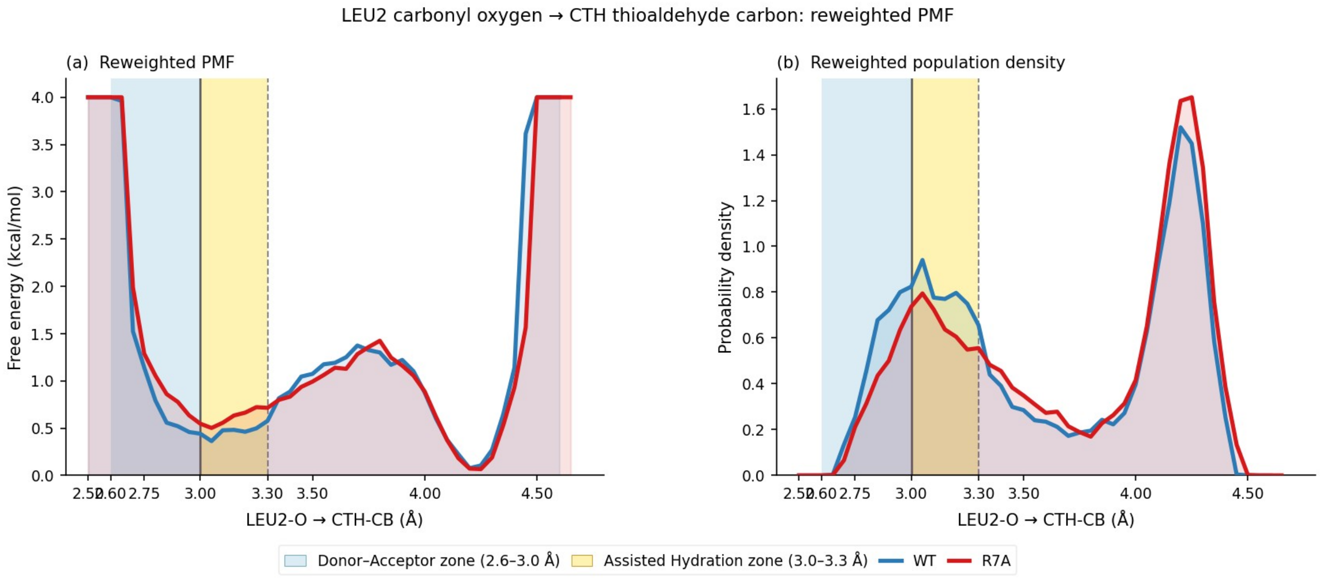

Figure P12.3: Reweighted structures showing the free-energy landscape associated with the distance between the cysteine thioaldehyde carbonyl oxygen atom (CTH-O) and the thiocarbonyl carbon atom (CTH-CB) – the intraresidue interaction. The peptides show very similar profiles.

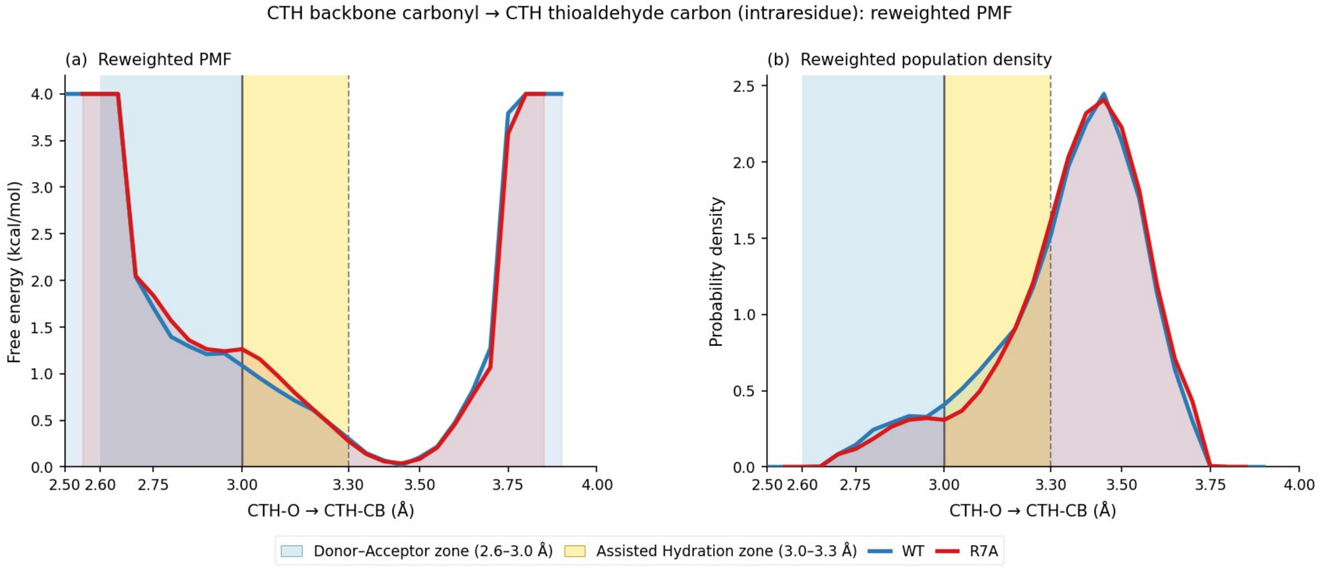

Figure P12.4: Reweighted structures showing the free-energy landscape of the dihedral angles associated with enolisation of the cysteine thioaldehyde residue. The residue adopts conformations that facilitate enolisation with transfer of the proton to the carbonyl oxygen atom rather than to the thiocarbonyl sulfur atom.

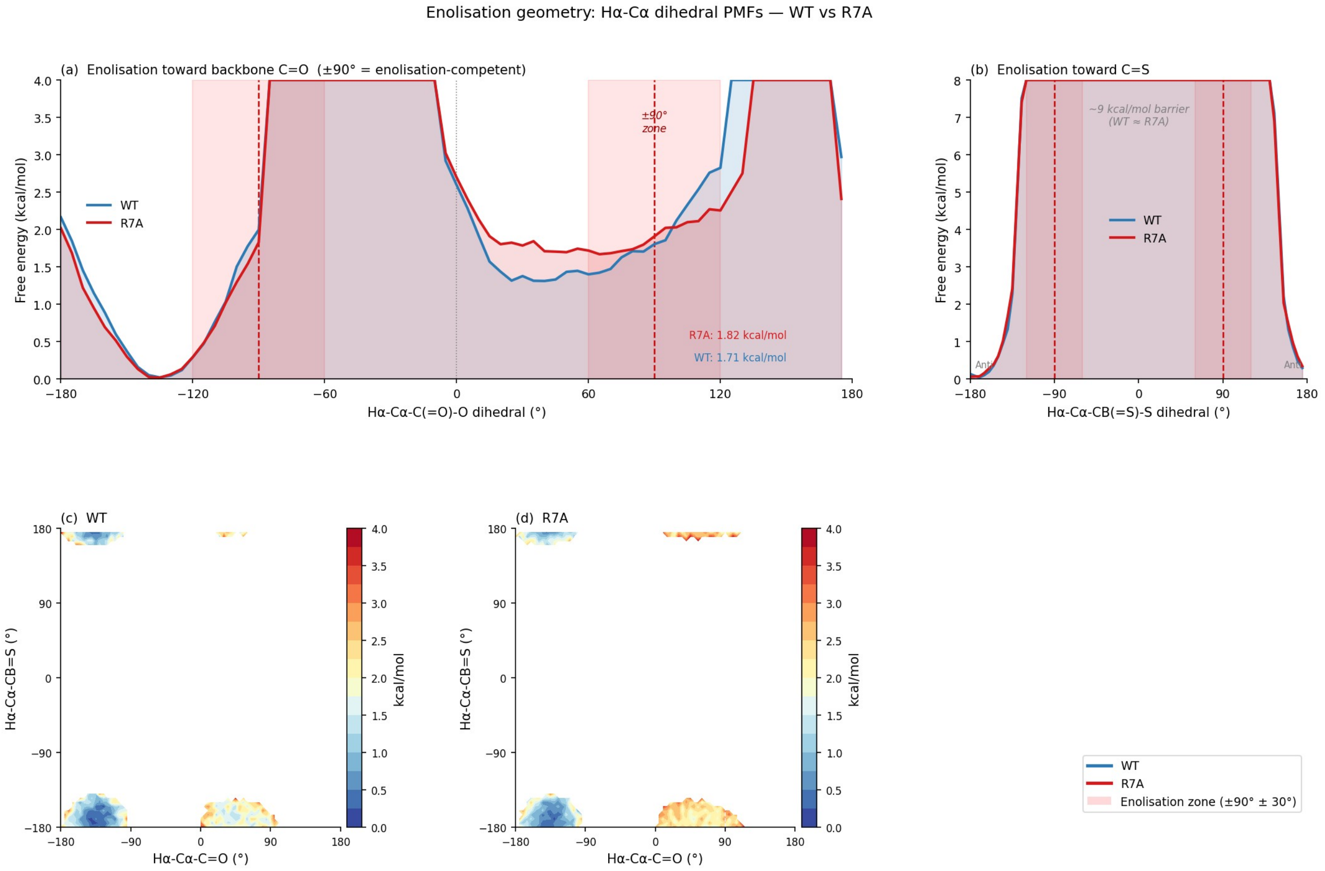

Figure P.12.5 Reweighted Ramachandran Plots

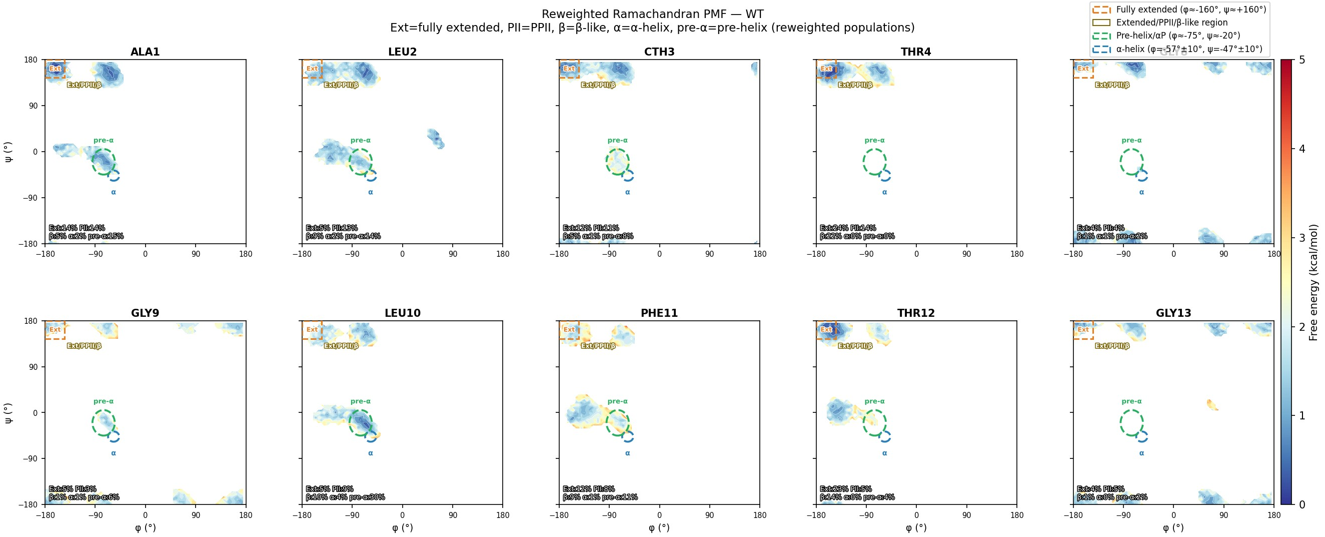

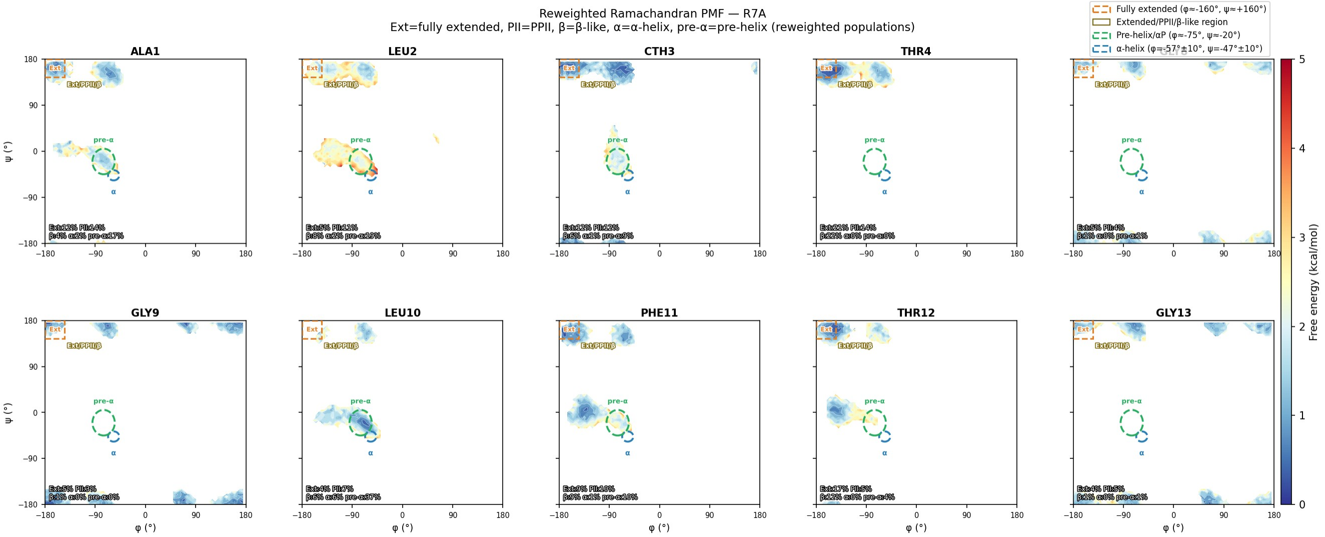

Table P.12.1 % Secondary structure of select residues in WT and R7A

System: WT

Residue	Fully ext	PPII	Beta	Alpha	Pre-hel	G-turn	Other
ALA1	13.5%	14.5%	4.9%	1.6%	15.1%	0.2%	50.2%
LEU2	5.2%	12.6%	8.9%	1.7%	14.0%	0.1%	57.5%
CTH3	11.9%	11.4%	5.4%	1.4%	8.3%	0.3%	61.3%
THR4	23.9%	14.5%	21.9%	0.0%	0.0%	0.0%	39.7%
GLY8	4.5%	4.2%	1.0%	0.7%	1.5%	0.0%	88.1%
GLY9	4.9%	3.2%	0.8%	0.7%	5.8%	0.0%	84.5%
LEU10	5.0%	9.1%	9.6%	4.1%	30.5%	0.2%	41.5%
PHE11	11.9%	8.3%	9.3%	1.0%	10.7%	0.1%	58.8%
THR12	22.6%	5.0%	13.6%	0.1%	3.6%	0.0%	55.0%
GLY13	3.9%	4.9%	0.7%	0.1%	1.5%	0.0%	88.9%

System: R7A

Residue	Fully ext	PPII	Beta	Alpha	Pre-hel	G-turn	Other
ALA1	12.0%	13.8%	4.2%	1.9%	17.1%	0.2%	50.7%
LEU2	5.1%	10.7%	8.2%	2.4%	19.3%	0.2%	54.2%
CTH3	12.5%	11.5%	5.9%	1.4%	8.9%	0.6%	59.2%
THR4	21.0%	13.8%	21.5%	0.0%	0.5%	0.0%	43.1%
GLY8	5.3%	3.6%	0.8%	0.1%	1.1%	0.0%	89.1%
GLY9	5.4%	2.8%	0.7%	0.1%	0.4%	0.0%	90.7%
LEU10	4.0%	7.0%	6.1%	6.5%	36.9%	0.2%	39.2%
PHE11	9.4%	9.6%	9.1%	0.8%	9.5%	0.0%	61.5%
THR12	17.2%	4.7%	11.9%	0.1%	4.4%	0.1%	61.5%
GLY13	4.0%	5.3%	0.7%	0.1%	1.3%	0.0%	88.5%

Region definitions (tight criteria):

```
  Fully extended: phi=-160+/-15, psi=+160+/-15
  PPII:           phi=-75+/-15,  psi=+150+/-15
  Beta-like:      phi=-120+/-30, psi=+130+/-30
  Alpha-helix:    phi=-57+/-10,  psi=-47+/-10
  Pre-helix:      phi=-75+/-20,  psi=-20+/-25
  G-turn (C7eq):  phi=-72+/-13,  psi=+80+/-30
```

Figure P.12.6 Ramachandran dihedral landscape for the cysteine thioaldehyde residue

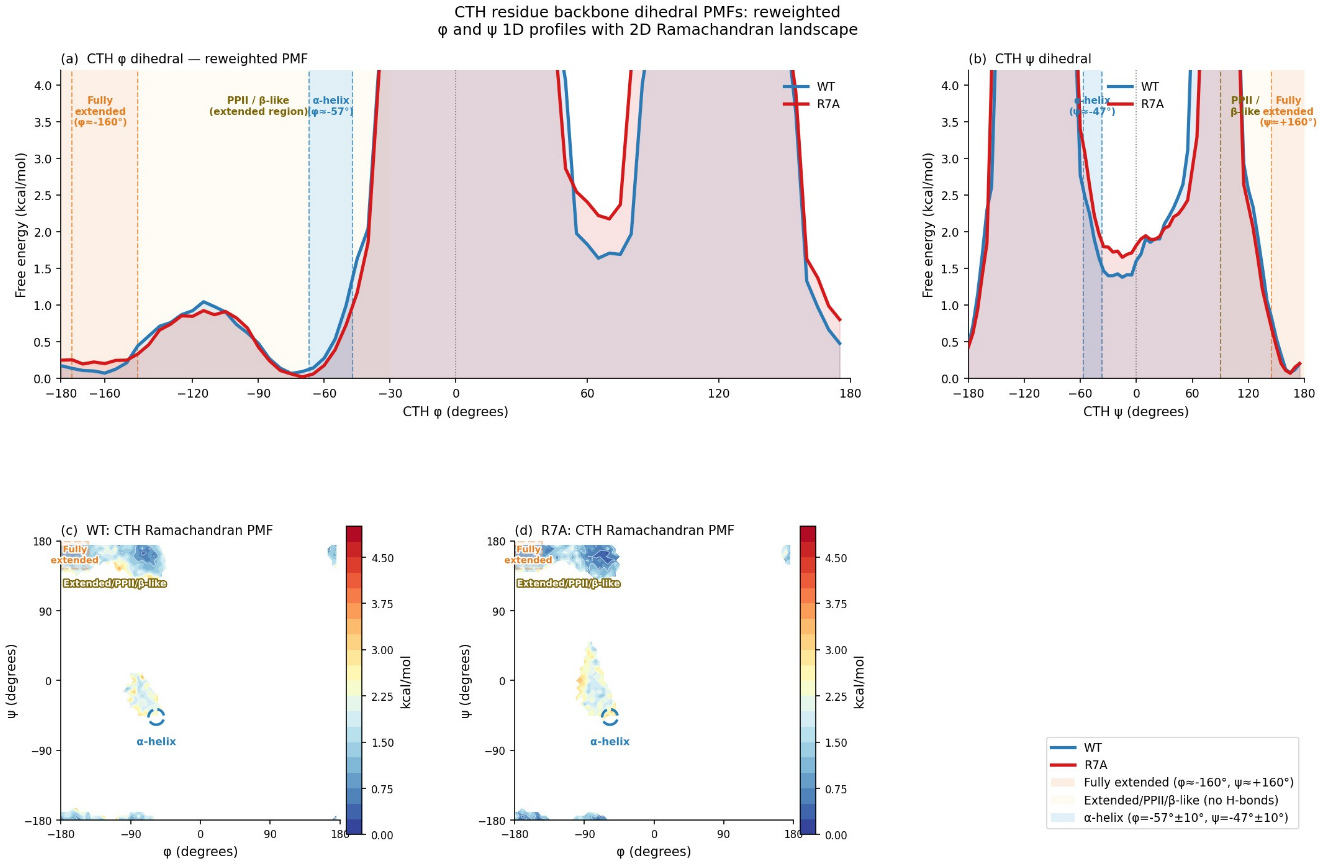

Figure P.12.7 Top 3 conformers for WT peptide

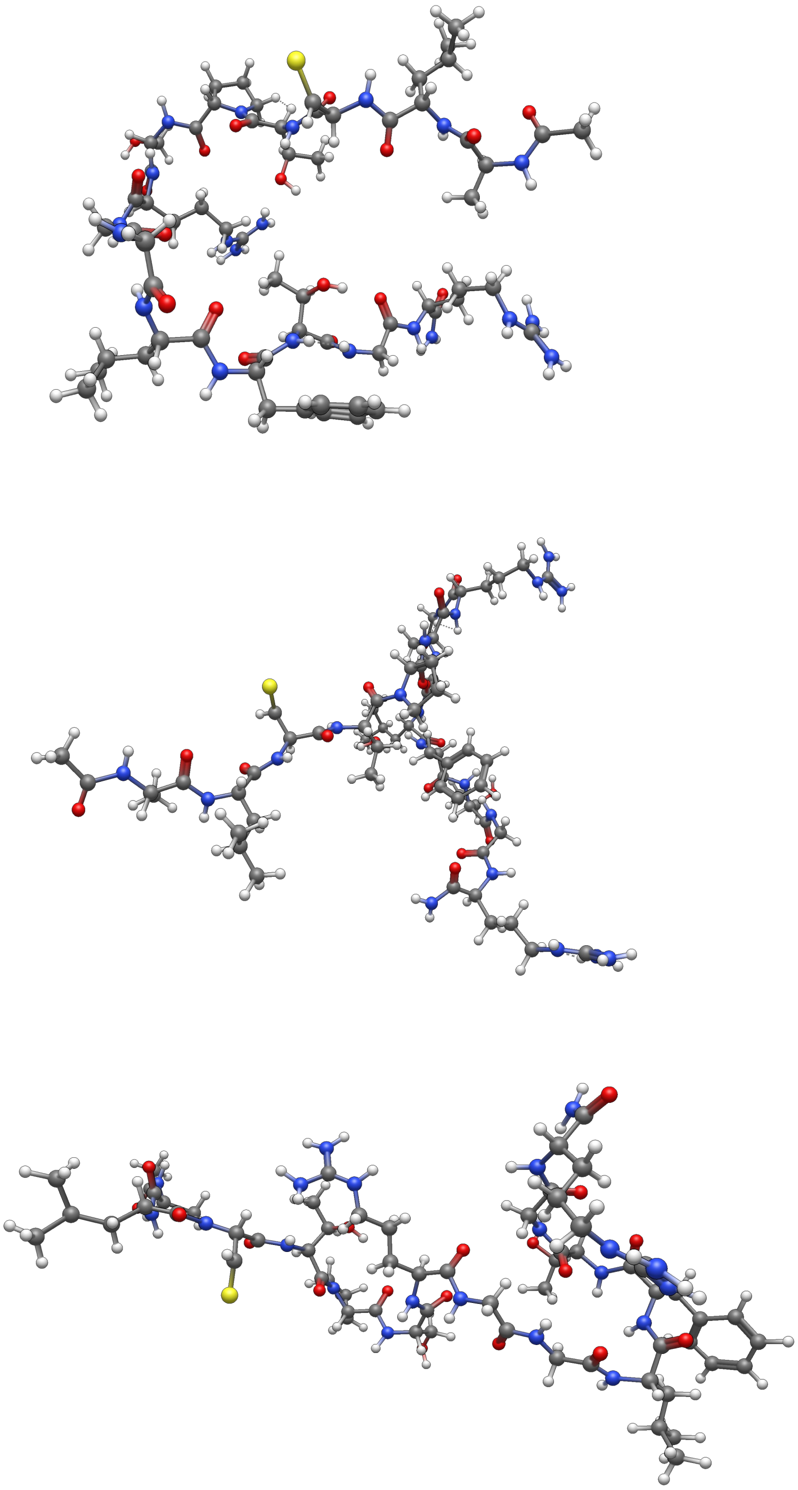

Figure P.13.8 Top 3 conformers for R7A peptide.

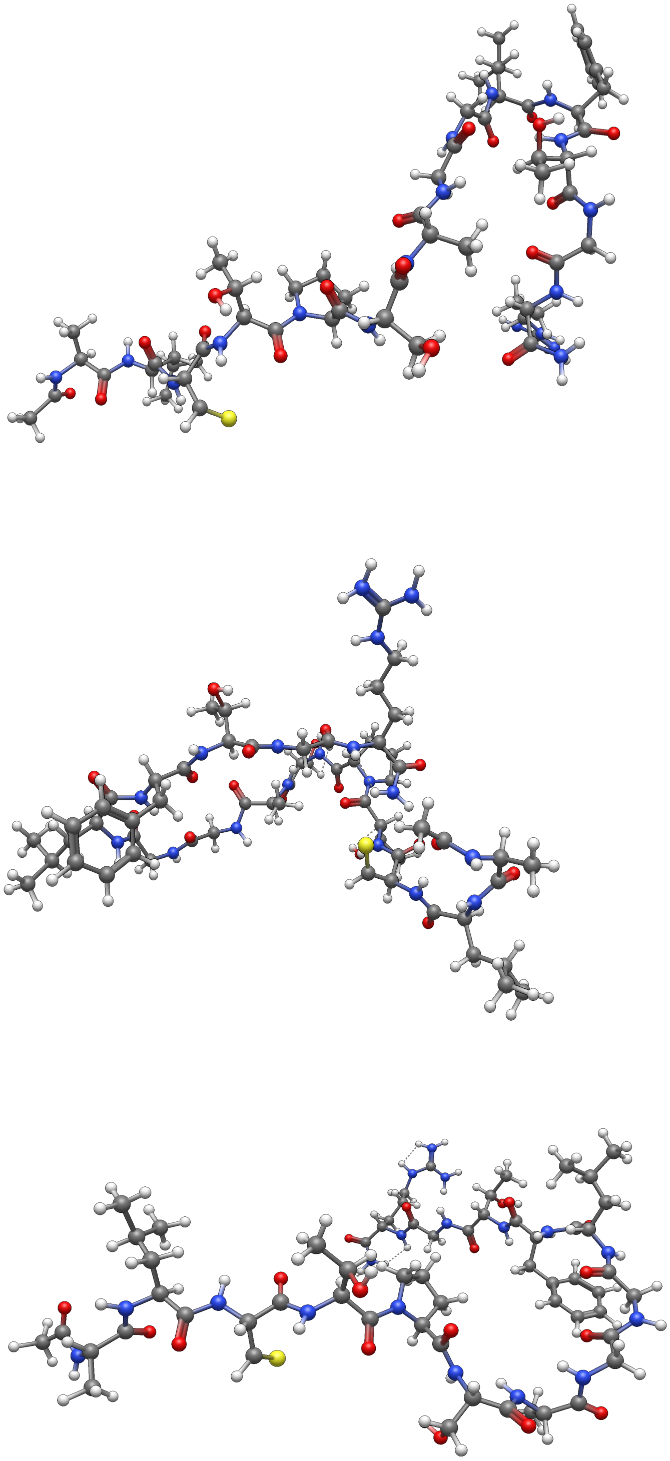

P13 References

(89) F. Neese, “Software update: the ORCA program system, version 6.0.,” *WIRES Comput. Molec. Sci.* 15 (2025): e70019; DOI: 10.1002/wcms.70019

(90) S. Grimme, A. Hansen, S. Ehlert, and J. Mewes, “r2SCAN-3c: A 'Swiss army knife' composite electronic-structure method,” *J. Chem. Phys*. 154 (2021): 064103; DOI: 10.1063/5.0040021

(91) F. Neese, “An improvement of the resolution of the identity approximation for the formation of the Coulomb matrix,” *J. Comp. Chem*. 24 (2003): 1740-1747; DOI: 10.1002/jcc.10318

(92) F. Neese, F. Wennmohs, A. Hansen, and U. Becker, “Efficient, approximate and parallel Hartree-Fock and hybrid DFT calculations. A 'chain-of-spheres' algorithm for the Hartree-Fock exchange,” *Chem. Phys*. 356 (2009): 98-109; DOI: 10.1016/j.chemphys.2008.10.036

(93) E. Caldeweyher, C. Bannwarth, and S. Grimme, “Extension of the D3 dispersion coefficient model,” *J. Chem. Phys*. 147 (2017): 034112; DOI: 10.1063/1.4993215

(94) E. Caldeweyher, S. Ehlert, A. Hansen, H. Neugebauer, S. Spicher, C. Bannwarth, and S. Grimme, “A generally applicable atomic-charge dependent London dispersion correction,” *J. Chem. Phys*. 150 (2019): 154122; DOI: 10.1063/1.5090222

(95) M. Garcia-Rates, and F. Neese, “Effect of the Solute Cavity on the Solvation Energy and its Derivatives within the Framework of the Gaussian Charge Scheme,” *J. Comput. Chem*. 41 (2020): 922-939; DOI: 10.1002/jcc.26139

(96) B. Helmich-Paris, B. de Souza, F. Neese, and R. Izsák, “An improved chain of spheres for exchange algorithm,” *J. Chem. Phys*. 155 (2021): 104109; DOI: 10.1063/5.0058766

(97) F. Neese, “The SHARK Integral Generation and Digestion System,” *J. Comp. Chem*. 44 (2022) : 381; DOI: 10.1002/jcc.26942

(98) C. Bannwarth, S. Ehlert, and S. Grimme, “GFN2-xTB—An Accurate and Broadly Parametrized Self-Consistent Tight-Binding Quantum Chemical Method with Multipole Electrostatics and Density-Dependent Dispersion Contributions,” *J. Chem. Theory Comput*. 15, (2019): 1652-1671; DOI: 10.1021/acs.jctc.8b01176

(99) B. de Souza, “GOAT: A Global Optimization Algorithm for Molecules and Atomic Clusters,” *Angewandte Chemie* 64 (2025): e202500393; DOI: 10.1002/anie.202500393

(100) C. -C. Lam, J. M. Goodman, “CONFPASS: Fast DFT Re-Optimisations of Structures from Conformational Searches,” *J. Chem. Inf. Mode*l. 63 (2023): 4364-4375; DOI: 10.1021/acs.jcim.3c00649.

(129) E. D. Glendening, J. K. Badenhoop, A. E. Reed, J. E. Carpenter, J. A. Bohmann,C. M. Morales, P. Karafiloglou, C. R. Landis, F. Weinhold, “NBO 7.0,” Theoretical Chemistry Institute, University of Wisconsin, Madison, WI (2018)

(141) M. M. Copeland, H. N. Do, L.Votapka, K. Joshi, J. Wang, R. E. Amaro, Y. Miao, “Gaussian Accelerated Molecular Dynamics in OpenMM,” *J. Phys. Chem. B* 126 (2022): 5810-5820; DOI: 10.1021/acs.jpcb.2c03765

(142) P. Eastman, R. Galvelis, R. P. Peláez, C. R. A. Abreu, S. E. Farr, E. Gallicchio, A. Gorenko, M. M. Henry, F. Hu, J. Huang, A. Krämer, J. Michel, J. A. Mitchell, V. S. Pande, J. PGLM Rodrigues, J. Rodriguez-Guerra, A. C. Simmonett, S. Singh, J. Swails, P. Turner, Y. Wang, I. Zhang, J. D. Chodera, G. De Fabritiis, and T. E. Markland. “OpenMM 8: Molecular Dynamics Simulation with Machine Learning Potentials,” *J. Phys. Chem. B* 128 (2023): 109-116.

(143) C. Tian, K. Kasavajhala, K. A. A. Belfon, L. Raguette, H. Huang, A. N. Migues, J. Bickel, Y. Wang, J. Pincay, Q. Wu, and C. Simmerling, “ff19SB: Amino-Acid-Specific Protein Backbone Parameters Trained against Quantum Mechanics Energy Surfaces in Solution,” *J. Chem. Theory. Comput*. 16 (2020): 528-552; DOI: 10.1021/acs.jctc.9b00591.

(144) E. Shrimpton-Phoenix, E. Notari, and C. W. Wood, “drFrankenstein: An Automated Pipeline for the Parameterisation of Non-Canonical Amino Acids,” bioRxiv (2026); DOI: https://doi.org/10.64898/2026.03.16.712088.

(145) D. A. Case, H. M. Aktulga, K. Belfon, D. S. Cerutti, G. A. Cisneros, V. W. D. Cruz eiro, N. Forouzesh, T. J. Giese, A. W. Götz, H. Gohlke, S. Izadi, K. Kasavajhala, M. C. Kaymak, E. King, T. Kurtzman, T. -S. Lee, P. Li, J. Liu, T. Luchko, R. Luo, M. Manathunga, M. R. Machado, H. M. Nguyen, K. A. O’Hearn, A. V. Onufriev, F. Pan, S. Pantano, R. Qi, A. Rahnamoun, A. Risheh, S. Schott-Verdugo, A. Shajan, J. Swails, J. Wang, H. Wei, X. Wu, Y. Wu, S. Zhang, S. Zhao, Q. Zhu, T. E. Cheatham III, D. R. Roe, A. Roitberg, C. Simmerling, D. M. York, M. C. Nagan, and K. M. Merz Jr., “AmberTools,”. *J. Chem. Inf. Model.* 63 (2023): 6183-6191;

(146) Y. Miao, W. Sinko, L. Pierce, D. Bucher, R. C. Walker, J. A. McCammon, “Improved reweighting of accelerated molecular dynamics simulations for free energy calculation,” *J. Chem. Theory Comput*. 10 (2014): 2677−2689.
